# Stereoselective Epimerization of 1,3-Diols Using a Chiral Hydrogen Atom Abstraction Catalyst

**DOI:** 10.1021/jacs.6c10166

**Published:** 2026-07-30

**Authors:** Miran Lemmerer, Darcy C. Emmet, Antti S. K. Lahdenperä, Daniel J. Davies, Amit Dahiya, Kristaps Ermanis, Robert J. Phipps

**Affiliations:** † Yusuf Hamied Department of Chemistry, Lensfield Road, Cambridge CB2 1EW, U.K.; ‡ School of Chemistry, 6123 University of Nottingham, University Park, Nottingham NG7 2RD, U.K.

## Abstract

The 1,3-diol unit is an extremely important functional group motif, and significant effort has gone into development of methods to access it with definition of relative and absolute stereochemistry. Conventionally, stereochemical complexity is built up in a stepwise manner alongside functional group interconversion. It is interesting to consider an unconventional approach whereby the diol may be synthesized nonselectively and the stereoisomers “descrambled” by a chiral catalyst in a subsequent step. We report studies toward this goal using a cinchona alkaloid-derived hydrogen atom abstraction catalyst. This catalyst permits selective hydrogen atom abstraction from a given stereoisomer out of, in some cases three or four, depending on diol substitution. The relative rates of abstraction from different stereoisomers determine the ultimate product outcome after hydrogen atom delivery from an achiral thiol. Symmetrical 1,3-diols participate in a kinetic resolution process whereby one enantiomer is converted to the *meso* diastereomer with extremely high selectivity, a rare example of a kinetic resolution involving conversion of one enantiomer to an achiral diastereomer. Nonsymmetrical 1,3-diols exhibit different outcomes depending on substitution, and their behavior is systematically explored. Notably, sterically differentiated substrates can allow high ee of both *syn* and *anti* diastereomers to be achieved from racemic starting material. On the basis of mechanistic studies, a unified rationalization of the behavior of 1,3-diols of various types with our catalyst is presented, and we additionally evaluate the analogous chiral 1,2-diols, uncovering promising outcomes with nonsymmetrical diols.

## Graphical abstract

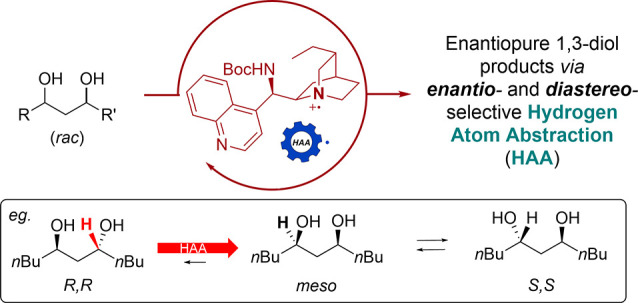

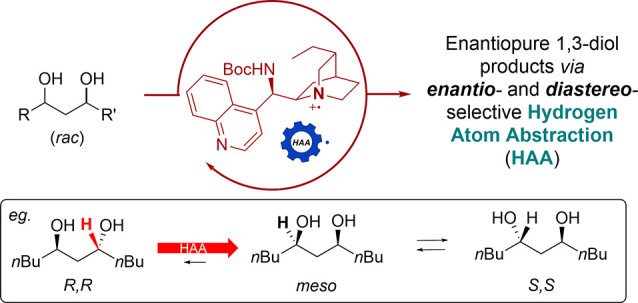

## Introduction

The 1,3-diol is a highly privileged structural motif due to its ubiquity in polyketide natural products,^1^ and nature uses polyketide synthases to produce sequences of 1,3-diols exhibiting immense stereochemical complexity.^2^ Accordingly, synthetic chemists have devoted much attention to control of relative and absolute stereochemistry in the synthesis of the 1,3-diol unit.^3^ The most widely used methods for setting relative stereochemistry revolve around diastereoselective reduction of β-hydroxyketones, which can generally be tuned to obtain either *syn* or *anti* diol (Figure 1A, lower).^4^ The β-hydroxycarbonyl compounds may be obtained in enantioenriched form by a variety of asymmetric aldol methods, as well as asymmetric reduction (Figure 1A, upper).^5^ These strategies follow the conventional synthetic logic of a stepwise building up of stereochemical complexity as part of a series of sequential functional group interconversions.

**Figure 1:**
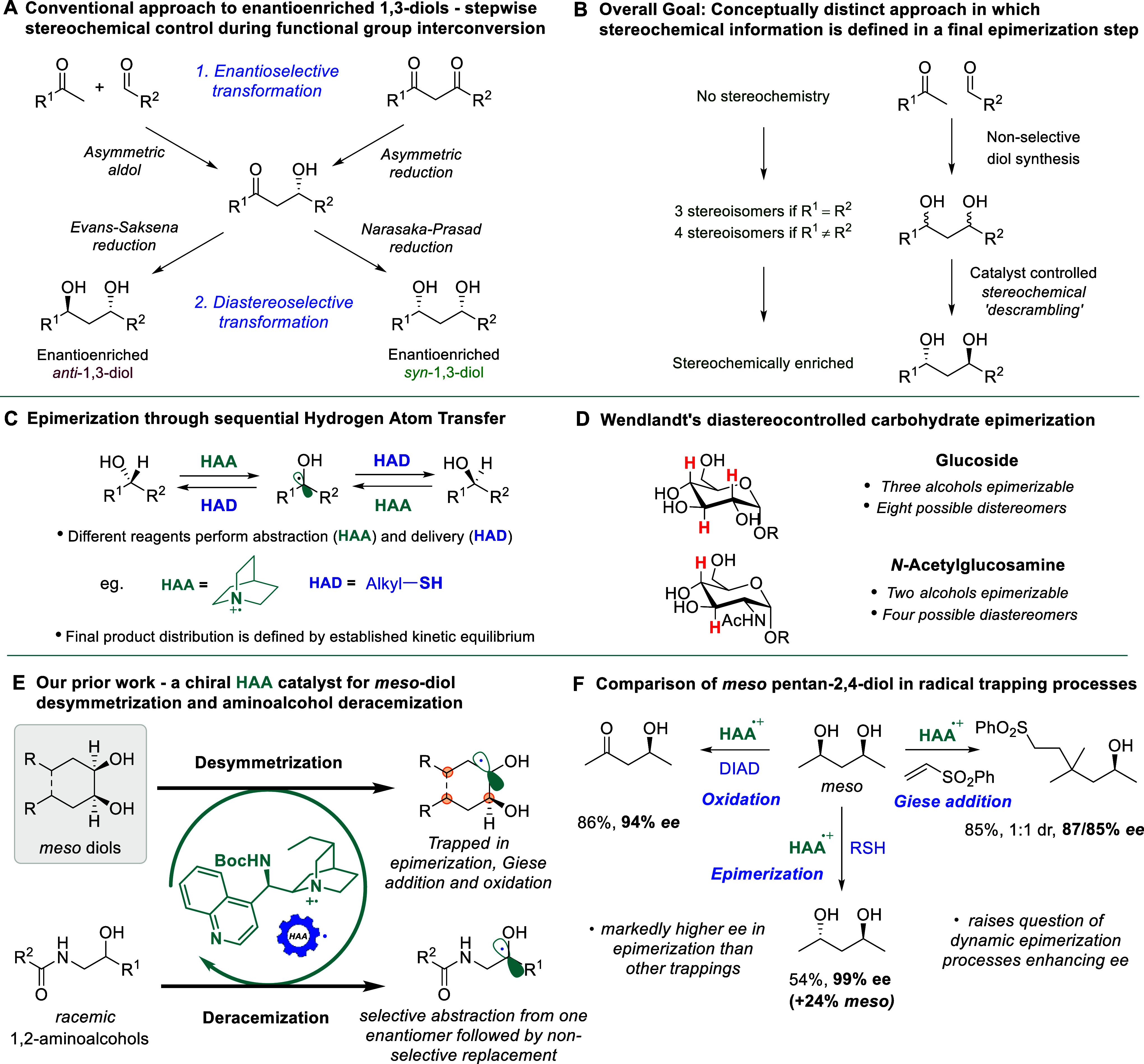
Established approaches to 1,3-diol synthesis and the background to this work.

At the outset of this work, we questioned whether a conceptually distinct approach toward enantiomerically enriched 1,3-diols may be viable. Specifically, whether an appropriate chiral catalyst may be able to selectively epimerize alcohol stereocenters in a *meso*- or racemic 1,3-diol such that only one or two out of up to four possible stereoisomers are selectively obtained. If successful this could allow a straightforward, nonselective 1,3-diol synthesis phase, followed by a key step in which the stereochemical complexity, potentially both relative and absolute, is refined under catalyst control in what could be regarded as a stereochemical “descrambling” step (Figure 1B).

The success of such an approach would require methodology for the *epimerization* of secondary alcohols, and here radical processes provide unique opportunities. Hydrogen atom transfer (HAT) adjacent to alcohols is often thermodynamically favorable due to the stability of the resulting radicals. Polarity matching of electrophilic radical abstractors with the hydridic hydrogen atom has resulted in much development of new methods with various abstractors.^6^ Following hydrogen atom abstraction (HAA), the stereochemistry at the alcohol is ablated but can be reformed, in either direction, if an appropriate reagent or catalyst for hydrogen atom delivery (HAD) is present (Figure 1C). With HAA and HAD proceeding via distinct pathways, the possibility exists to influence stereochemistry in each step separately, or in combination.^7^ In this vein, Wendlandt and co-workers have carried out innovative studies on diastereoselective epimerization of alcohol-containing molecules using achiral HAT catalysts, typically with quinuclidine or decatungstate as HAA catalysts and thiols as HAD catalysts.^8,9^ Their detailed work on carbohydrate epimerization delves into the stereochemical complexity of these processes and permits defined outcomes in a number of cases. For example, a glucoside possesses three epimerizable alcohols, equating to eight possible diastereomers and 48 distinct rate constants for HAA and HAD (Figure 1D, upper).^8c^ A protected glucosamine, possessing two epimerizable alcohols, is somewhat less complex but still equates to four diastereomers and 16 HAA/HAD rate constants (Figure 1D, lower).^8e^

We have recently developed a chiral quinuclidine-containing HAA catalyst based on a cinchona alkaloid scaffold. Using this in combination with an achiral thiol, we demonstrated the enantioselective epimerization of *meso*-1,2-diols to form the chiral stereoisomers with high *ee*.^10^ The selective abstraction of enantiotopic hydrogen atoms in the *meso*-diols was deduced to be the overriding contributor to the overall enantioselectivity outcome, as demonstrated in trapping experiments with Giese acceptors and a subsequent oxidation version of the process (Figure 1E, upper).^11^ We have subsequently shown that the same catalyst can deracemize the secondary alcohols in *N*-acetyl-1,2-aminoalcohols, the catalyst abstracting a hydrogen atom selectively from one enantiomer of the starting material before nonselective replacement by a hydrogen atom originating from an achiral thiol. Over sufficient cycles, this results in accumulation of the unreactive enantiomer (Figure 1E, lower).^12^ Our present working hypothesis is that functionality on the carbon atom adjacent to the secondary alcohol undergoing epimerization (either alcohol or protected amine) engages in hydrogen-bonding interactions with the Cinchona alkaloid-derived chiral HAA catalyst, resulting in a highly ordered transition state for abstraction.^12^ Deracemization studies from Bach and co-workers have also demonstrated this principle using a chiral HAA catalyst based on a bifunctional benzophenone.^13^

While the majority of our prior work on diol epimerization focused on *meso*-1,2-diols, both cyclic and acyclic, we also examined several examples of acyclic, *meso*-1,3-diols.^10^ For the simplest case, pentan-2,4-diol, epimerization starting from the *meso* isomer produced the chiral diastereomer in a remarkable 99% *ee*, albeit with significant amounts of the *meso*-diastereomer present at the end of the reaction (Figure 1F, *epimerization*). Trapping the radical irreversibly in oxidation or Giese addition still gave high *ee* outcomes but noticeably not at the same remarkable level (Figure 1F, *oxidation* and *Giese addition*). These observations, combined with the importance of the 1,3-diol motif in synthesis, prompted us to examine the possible dynamic behavior of 1,3-diols in more detail under our epimerization conditions. The potential complexity involved can be appreciated by first considering a substrate containing a single epimerizable stereocenter, as in our aminoalcohol deracemization, in which a chiral HAA catalyst selectivity abstracts from one enantiomer.^12^ In this situation, there are only two competing abstraction rate constants (*k*_1_ and *k*_4_); these define the final ee outcome if the thiol HAD catalyst is achiral (Figure 2A). The next increase in complexity lies in the addition of a second alcohol, to form a symmetrical diol (Figure 2B). There are now two diastereomers: one chiral and one *meso*, leading to a total of three possible stereoisomers. In this scenario, assuming that HAD rate constants from the achiral thiol are similar, four competing HAA rate constants will determine the final product outcome (*k*_1_, *k*_4_, *k*_6_, and *k*_7_). Progressing to a nonsymmetrical 1,3-diol increases complexity further; now both diastereomers are chiral and therefore a chiral HAA catalyst will exhibit eight distinct abstraction rate constants, assembled along a circular reaction network, which would all be expected to contribute to the final kinetic equilibrium, if it is reached before potential catalyst decomposition (Figure 2C). Catalytic deracemization where a single stereocenter is involved (as in Figure 2A) already poses a great challenge for asymmetric catalysis, albeit one in which significant progress is now being made.^14^ Exercising control in dynamic processes involving chiral molecules of greater complexity (as in Figure 2B,C) arguably represents the frontier in dynamic stereocontrol and a stimulating challenge for stereoselective catalysis.

**Figure 2:**
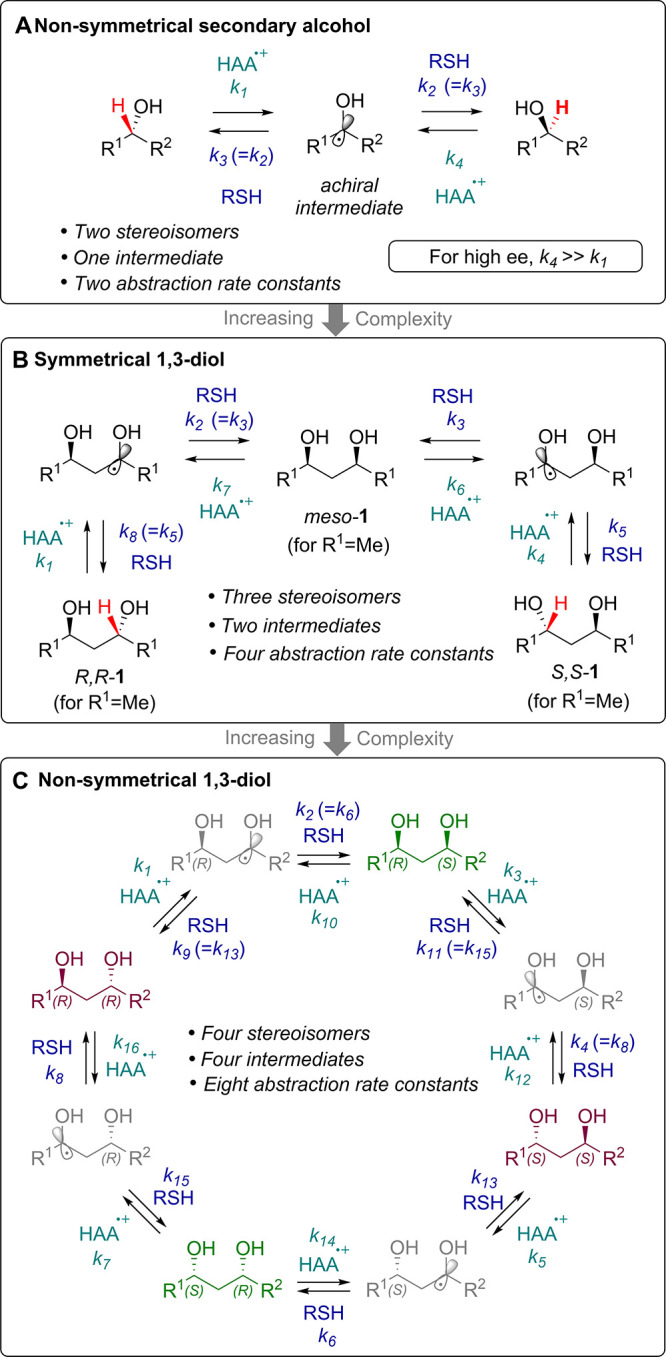
General kinetic schemes outlining use of a chiral HAA catalyst with achiral thiol for three different classes of alcohol substrate, of increasing complexity.

With these challenges in mind, in this work, we systematically evaluate the application of our chiral HAA catalyst to racemic 1,3-diols. We first explore symmetrical substrates (as in Figure 2B) and then proceed to nonsymmetrical substrates (as in Figure 2C), steadily increasing complexity. We reveal that our chiral HAA catalyst exhibits a remarkable propensity to abstract very rapidly and selectively from one enantiomer of an *anti*-1,3-diol. In a symmetrical system, this permits a highly effective kinetic resolution through selective epimerization to the achiral *meso* diastereomer, with *ee* values often >99%. In the more complex, nonsymmetrical 1,3-diols, for certain substitution patterns, high ee of both diastereomers can be obtained and for the *anti*-diastereomer this is often 99%, underlining the outstanding selectivity of our catalyst for asymmetric HAA in these systems. Steric properties of substituents around the site of abstraction play an important role in the abstraction rate, and for certain substrate types, the outcomes occupy a region between kinetic resolution and deracemization; an easy classification is not always straightforward. With sufficient results in hand, we are able to advance a unified rationalization of the behavior of 1,3-diols of various types with our catalyst, providing a tentative framework for understanding and predicting its application. In addition, we evaluated the translation to 1,2-diols and uncovered different trends with some promising synthetic outcomes.

## Results and Discussion

We first performed a detailed study of the behavior of pentane-2,4-diol (**1a**) under our system of 5 mol % *epi* **-NHBoc-DHCN** as HAA catalyst and 1 equiv of 1-dodecanethiol as the HAD reagent (Scheme 1A). As reported in our previous study, *meso*-**1a** undergoes epimerization to the chiral diastereomer (*S*,*S*)-**1a** with a moderate yield of 54% but a remarkable 99% *ee*. Significant amounts of *meso* diol remained in the reaction mixture after 16h (approximate 2:1 ratio of (*S*,*S*)-**1a**:*meso*-**1a**). We next subjected the racemate of the *anti*-diastereomer (*rac*-**1a**) to the reaction conditions and found that a virtually identical reaction outcome was achieved after the same time, suggesting that within the reaction time scale, a common kinetic equilibrium is reached. This could be visualized by following a reaction time course starting from *rac*-**1a** (Scheme 1B). Independent reactions were set up for each time point as obtaining reliable data by taking aliquots from a single reaction was found to be challenging using our present setup. This revealed that over the first 2 h (*R*,*R*)-**1a** is rapidly and selectively converted to *meso*-**1a**. The 2 h time point features an almost equal mix of *meso*-**1a** and (*S*,*S*)-**1a**, the *ee* of the chiral diastereoisomer being 96% at that point. From here forward, the yield of the chiral diastereomer slowly increases beyond 50%, with a concomitant decrease in yield of the *meso*-diastereomer, at a relatively slow rate. During this time, *ee* of the chiral diastereomer is refined from 96% to 99%. After 8 h the same product distribution is obtained as when the reaction is left for 24 h. Remarkably, the equilibrium mixture contains essentially no (*R*,*R*)-**1a**. We examined an analogous time course starting from (*R*,*R*)-**1a** (Scheme 1C). The anticipated rapid conversion of (*R*,*R*)-**1a** to *meso*-**1a** meant that a ∼1:1 ratio of the two was reached after only 1 h. After 2 h, the reaction mixture is predominantly *meso*-**1a** and from here on the concentration of (*S*,*S*)-**1a** starts to build as it gradually evolves from *meso*-**1a**. By 16 h, the presumed kinetic equilibrium is almost reached.

**Scheme 1:**
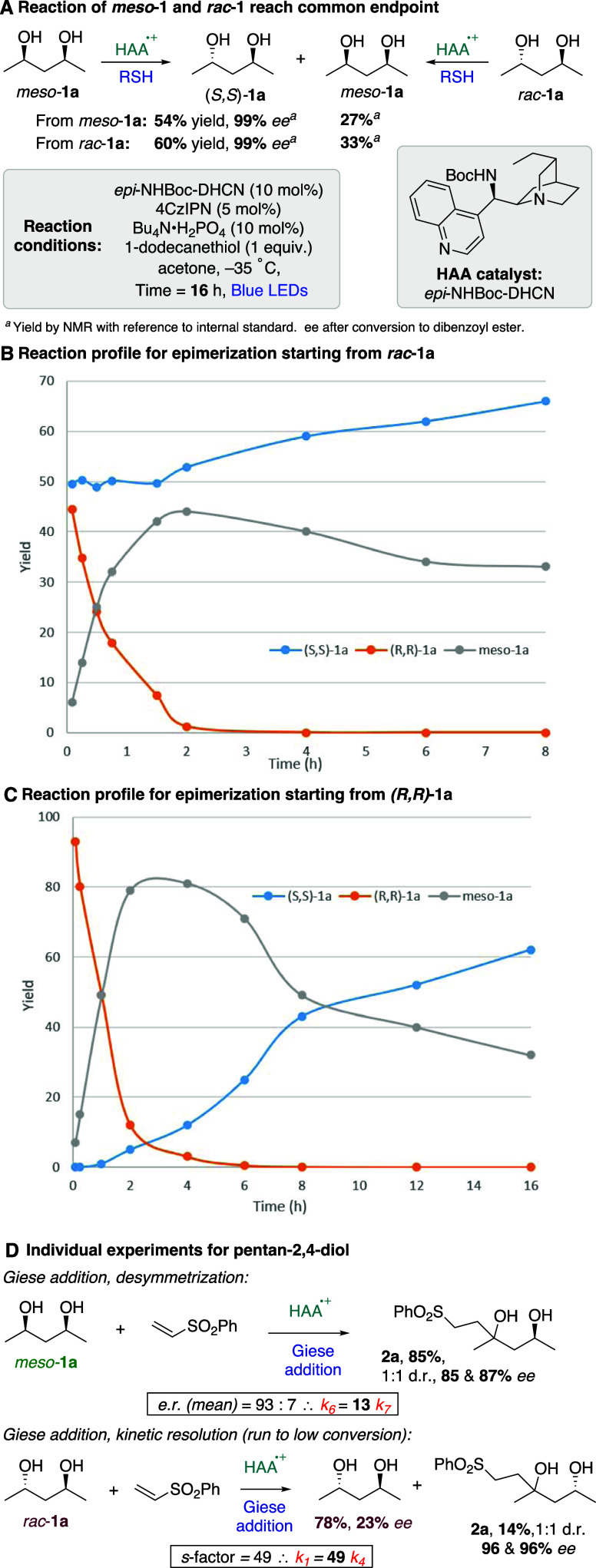
Investigations into the Dynamic Behavior of Pentan-2,4-diol 1a under Epimerization Conditions

We sought a more quantitative view of the relative abstraction rate constants involved for diol **1a**, mapped onto the kinetic scheme for a symmetrical 1,3-diol (Figure 2B). Quenching of the radical intermediate by the achiral thiol (*k*_2_ and *k*_5_) will necessarily contribute to the kinetic picture, but we base our analysis on the assumption that there is low or negligible diastereoselectivity (i.e., *k*_2_ and *k*_5_ are very similar) and therefore the HAA steps are the major contributors to the overall stereochemical outcome. We established in our previous work that reaction of *meso*-**1a** in the presence of a Giese acceptor (phenyl vinyl sulfone) gave a mean ee of 86%, taking into account both diastereomers (Scheme 1D, upper). If this is assumed to constitute an irreversible trapping of the radical intermediate, the product ee reflects that *k*_6_ = 13*k*_7_. We next carried out the Giese trapping but in the kinetic resolution mode (from *rac*-**1a**), running to low conversion and giving product ee of 96%, equating to *k*_1_ = 49*k*_4_ (Scheme 1D, lower)_._ As the established kinetic equilibrium only contains (*S*,*S*)-**1a**:*meso*-**1a** in a 2:1 ratio, one can approximate that *k*_6_ ≈ 2*k*_4_ and propose a relative ordering of *k*_1_ ≫ 2*k*_4_ ≈ *k*_6_≫ *k*_7_, based on 0.04*k*_1_ = 2*k*_4_ ≈ *k*_6_ = 13*k*_7_. Inserting rate constants corresponding to this relationship into a simple web-based kinetics simulator that we have produced specifically tailored to this methodology provides a time course simulation that matches extremely well those in Schemes 1B,C (see Supporting Information for full details and instructions on how to access). This simulator offers users the ability to input different rate constants to see how the reaction composition will evolve. Based on this analysis, the behavior of *rac*-**1a** can be divided into two phases—the first can be regarded as a kinetic resolution phase in which the catalyst reacts rapidly with (*R*,*R*)-**1a** to convert it into *meso*-**1a**, resulting in a highly effective kinetic resolution outcome after 2 h. This constitutes a rare type of kinetic resolution in which the “product” is an achiral stereoisomer of the starting material. Then evolves a second, slower phase wherein the *meso*-isomer, reactive but at a lower rate than (*R*,*R*)-**1a**, gradually reaches a kinetic equilibrium with (*S*,*S*)-**1a**. A modest increase in yield of (*S*,*S*)-**1a** results and is limited to around 60% by the fact that *k*_4_ and *k*_6_ are not dramatically different. Crucially, the extremely large difference between *k*_1_ and *k*_7_ (around 2 orders of magnitude) combine to make the *ee* of the chiral diastereomer extremely high.

Armed with this understanding of the simplest compound of the class, we next explored how it translated to other symmetrical 1,3-diols, in all cases starting from the chiral racemic diastereomer and extending the chains of the R groups beyond methyl (Scheme 2). It became quickly apparent that once the substituent is elongated beyond methyl, the yield of the *anti*-diastereomer after 48 h reaction time does not exceed 50%, or thereabouts (**1a**-**1d**, yields by ^1^H NMR with reference to internal standard are shown in square brackets). In most cases, the remaining *anti*-diastereomer could be separated from the formed *syn* by column chromatography, although in several cases, the isolated yield was reduced by the challenging separation. Notably, the *ee* of the isolated *anti* diastereomer was extremely high across the board, and in many cases 99%, with the minor enantiomer often not detected by chiral SFC. This was found to be consistent for a variety of chains, including linear alkyl chains (**1a**-**1d**) and those involving branching at various points (**1e**, **1f**). Cyclobutyl (**1g**) and cyclohexyl (**1h**-**1j**) groups could be included on the chain termini, but if a bulky cyclohexyl group was incorporated close to the diol unit, the *ee* was reduced (**1h**). In the case of **1j**, the NMR yields suggest that a suboptimal conversion reduces the *ee* of the isolated *anti*-**1j**. Phenyl rings could be incorporated at the ends of the chains, still with a high *ee* (**1k** and **1l**).

**Scheme 2:**
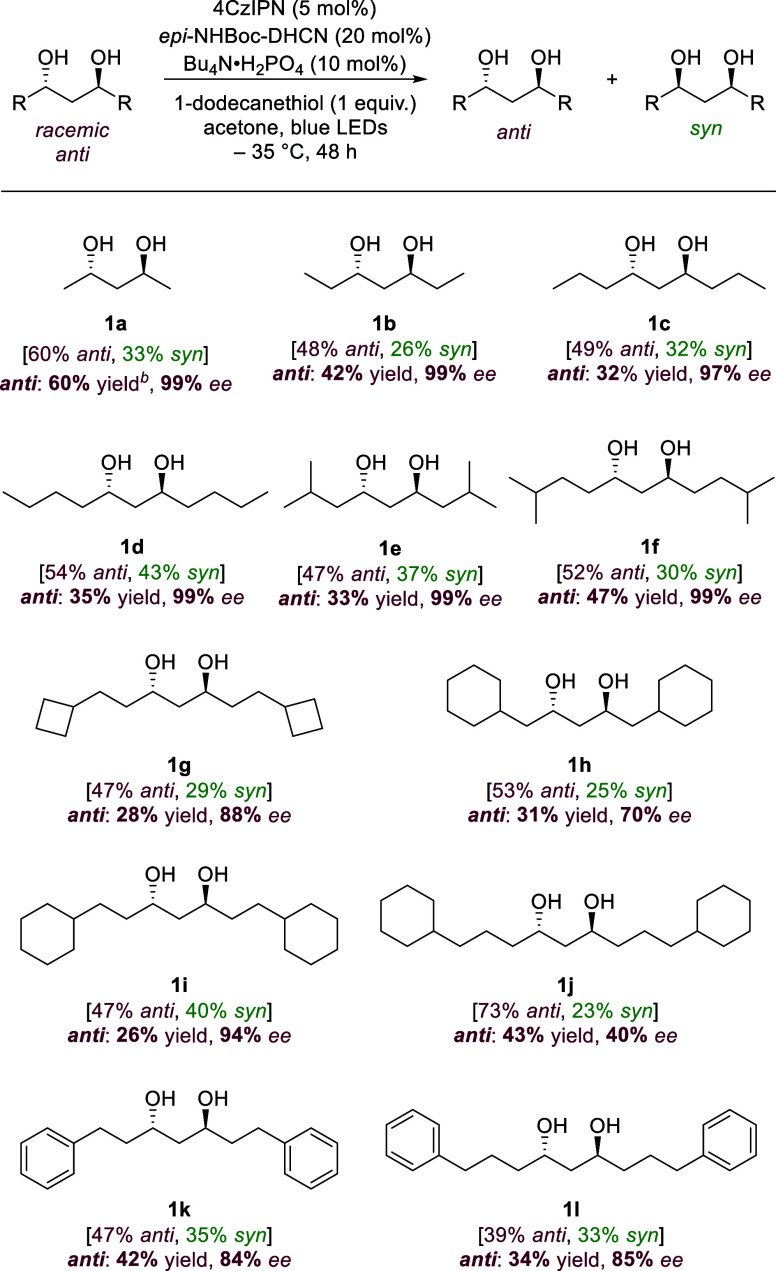
Scope of Symmetrical 1,3-Diols^a^ ^b^

a The indicated yield is the isolated yield after column chromatography. The value in parentheses is the yield by NMR with reference to an internal standard. *ee* in examples without chromophore were determined after conversion to dibenzoyl ester.
b Yield determined by NMR due to the volatility of the product.

The results paint a picture for these substrates where the kinetic resolution phase is occurring readily, i.e., the (*R*,*R*)-isomer converts rapidly to the *meso*, resulting in a mixture of *meso* and unreacted (*S*,*S*). However, the slow evolution to a point where the *anti* becomes the major diastereomer, as seen in the detailed study of **1a**, does not seem to occur to a significant extent. To probe this further, we carried out a time course study on the *n*-butyl-substituted diol **1d** (Scheme 3A). This showed a clear kinetic resolution phase which is largely complete after around 4–5 h, significantly slower than methyl-substituted **1a**, for which this phase was complete after only 2 h (Scheme 1B). For **1d**, after the KR phase is complete there is little change apart from a refinement of the *ee* of *anti*-**1d** to 99%. A key difference with these longer chain substrates is that attempted epimerization of *meso*-**1d** gave very low yield of only 11% of chiral **1d** after 48 h, suggesting that the abstraction rate is far lower from the *meso* diastereomer when the substate has longer chains, potentially due to increased steric hindrance at the site of abstraction (Scheme 3B). To gain further insight we conducted experiments starting from deuterated substrates *anti*-**1d** and *meso*-**1d** (Scheme 3C). Here, all reagents (thiol, acetone, phosphate) are nondeuterated, as are the hydroxyl groups of the diols. Relative erosion of deuteration adjacent to a given alcohol can be broadly equated with the extent of abstraction occurring there. When racemic *anti*-**1d**, fully deuterated at both alcohols, was employed, *anti*-**1d** with 91% *ee* was recovered in 48% yield. Importantly, this material retained 96% of the deuterium, indicating minimal abstraction by the catalyst, in line with our hypothesis that the (*S*,*S*) enantiomer is essentially untouched. The formed *meso*-**1d** had lost approximately half of its deuterium, consistent with one enantiomer selectively converted to the *syn*—the abstracted deuterium is replaced by a hydrogen from the thiol. When deuterated *meso*-**1d** was used, very low conversion to *anti*-**1d** was observed, as expected (only 2% yield by NMR). This was obtained in very high *ee* (99%) and with substantial deuterium loss (63% D).^15^

**Scheme 3:**
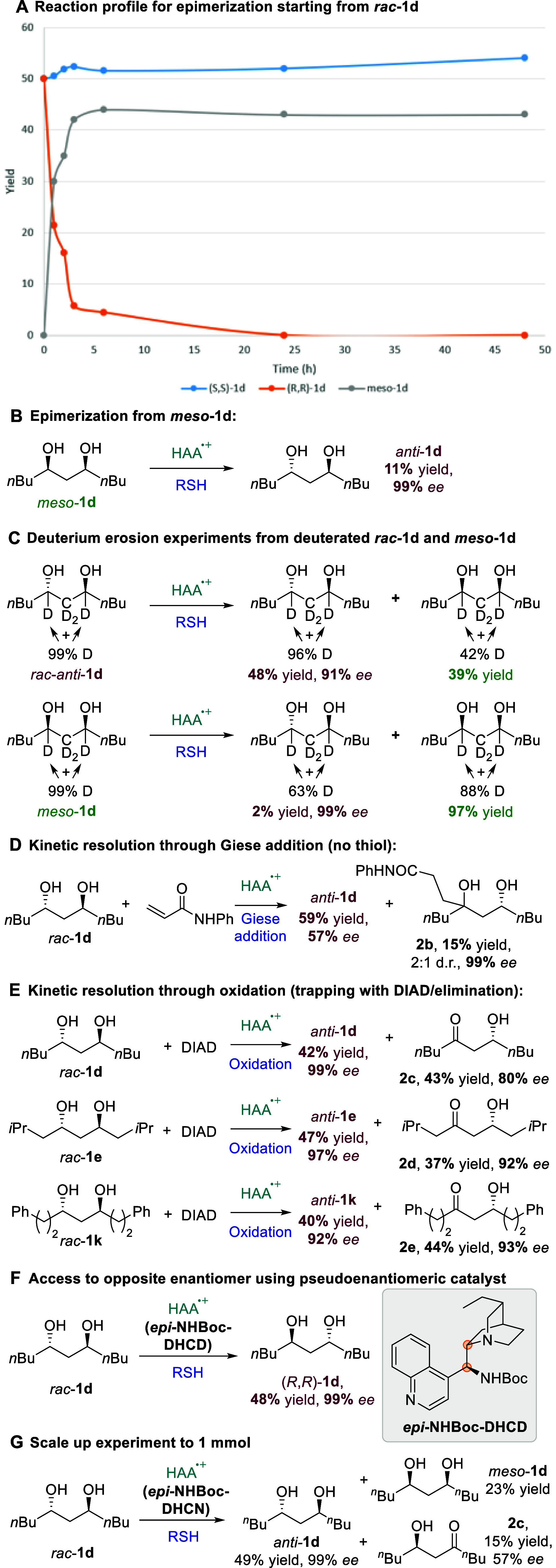
Reaction Time Course and Ancillary Experiments for Symmetrical 1,3-Diol 1d

With these *anti*-1,3-diol substrates appearing to undergo kinetic resolution through epimerization, we sought to expand the trapping of the intermediate radical beyond hydrogen atom delivery; trapping with other agents should deliver chiral, enantioenriched products in addition to enantioenriched starting materials. We first evaluated Giese addition^16^ and found that, surprisingly, conversion was modest, although the diastereomeric products could be obtained in very high *ee*, indicating very high discrimination of the catalyst between the starting material enantiomers (Scheme 3D). We then evaluated trapping via oxidation using diisopropyl azodicarboxylate (DIAD), according to our previous report to form α-hydroxyketones.^11^ In this case, conversion was around 50%, as should be optimal in a KR. For *rac*-**1d**, the recovered starting material exhibited 99% *ee* and the oxidized product exhibited 80% *ee* (Scheme 3E). The *ee* of the latter was lower than anticipated but it is possible that undesired abstraction from the product could impact stereochemical integrity. Two slightly different substrates (**1e** and **1k**) bearing different chain termini gave high *ee* for the oxidized product in highly effective KR outcomes. We demonstrate also that we can use the catalyst pseudoenantiomer, derived from cinchonidine, to obtain (*R*,*R*)-**1d**, without any detriment to yield or *ee* (Scheme 3F). Finally, we scaled up the reaction to 1 mmol starting material (Scheme 3G). This was successful, and *anti*-**1d** was obtained as single enantiomer and 49% isolated yield. Interestingly, a significant amount of oxidized product was obtained, presumably derived from the radical intermediate undergoing oxidation in competition with radical trapping under the scaled-up reaction conditions. The *ee* of the oxidized product was moderate, in line with the lower-than-expected *ee* for this compound under deliberate oxidation.

We next examined nonsymmetrical 1,3-diols, featuring different substituents on either side of the diol and translating to a more complex kinetic situation (see Figure 2C), evaluating each racemic diastereomer of **3a** in turn under the standard reaction conditions. A*nti*-**3a** was converted to a mixture of *anti* and *syn* after 48 h reaction (Scheme 4A, upper). We were very pleased to find that, in analogy with the symmetrical diols, the *anti*-diastereomer that remained was obtained in 99% *ee*. The corresponding *syn*-diol, in this scenario also chiral, formed in 37% yield and with a considerable *ee* of 65%. Submitting racemic *syn*-**3a** to the same conditions gave *anti*-**3a** in similarly high *ee* but in a markedly lower yield and the *syn*-**3a**, recovered in high yield, was minimally enantioenriched (Scheme 4A, lower). This likely reflects low reactivity of the *syn*-configured isomers with the catalyst. Absolute configuration for these isomers was confirmed by independent syntheses through Noyori asymmetric hydrogenation (see the Supporting Information for details). We conducted a time course study starting from racemic *anti*-**3a** (Scheme 4B). This showed that the (*R*,*R*) enantiomer is rapidly depleted over the first 3 h to form *syn*-**3a** with 62% *ee* and the remaining *anti*-**3a** at 82% *ee*. Over a longer period of time (3–10 h), the *ee* of *anti*-**3a** evolves to 99% while the *ee* of *syn*-**3a** changes little. Beyond 10 h, further change is relatively minimal. This overall behavior is analogous to that observed in the time course of *rac*-**1a** (Scheme 1B). In that case, (*R*,*R*)-**1a** converts to the *meso*-diastereomer. In this case, (*R*,*R*)-**3a** converts to both enantiomers of *syn*-**3a** with a moderate selectivity of ∼4:1. To provide indication as to whether a final, stable stereoisomeric distribution in the epimerization of **3a** is obtained due to catalyst decomposition or a final kinetic equilibrium being reached, we isolated **3a** as a stereoisomer mixture after a 24 h reaction and resubjected the mixture to the reaction conditions for a further 24 h. Only a minor change to the stereoisomeric composition was observed, suggestive of a kinetic equilibrium being reached (see the Supporting Information for details). We next carried out deuterium erosion experiments to gauge the extent of abstraction occurring at each position. First examined was *anti*-**3a**, deuterated adjacent to both alcohols and at the intermediate methylene (Scheme 4C, upper). A modest but significant degree of deuterium erosion (78% D) was observed in the high *ee* *anti* diastereomer indicating that this compound is not derived solely from unreactive (*S*,*S*)-*anti*-**3a**; some likely originates from epimerization of other stereoisomers. While it was not possible to determine individual deuteration at the two positions of *anti*-**3a**,^17^ *syn*-**3a** was illustrative in that it showed almost complete deuterium loss (5% D) at the alcohol adjacent to the methyl group.^18^ The loss was markedly less at the more hindered ‘inner’ alcohol (48% D). We tentatively attribute this regioselectivity to the difference in steric hindrance of the benzyl versus methyl substituents on either side of the diol (see later discussion). We also examined monodeuterated *anti* **-3a** (Scheme 4C, lower). Any deuterium transposition in the formed *anti*-**3a** could not be determined due to the two peaks being indistinguishable, although the extent of deuterium loss was consistent with the prior experiment. In the formed *syn*-**3a**, the deuterium partially transposed into the more hindered position, most probably shuttled via some level of deuterated thiol generated during the reaction, illustrating the complex dynamic behavior of this substrate under the epimerization conditions.

**Scheme 4:**
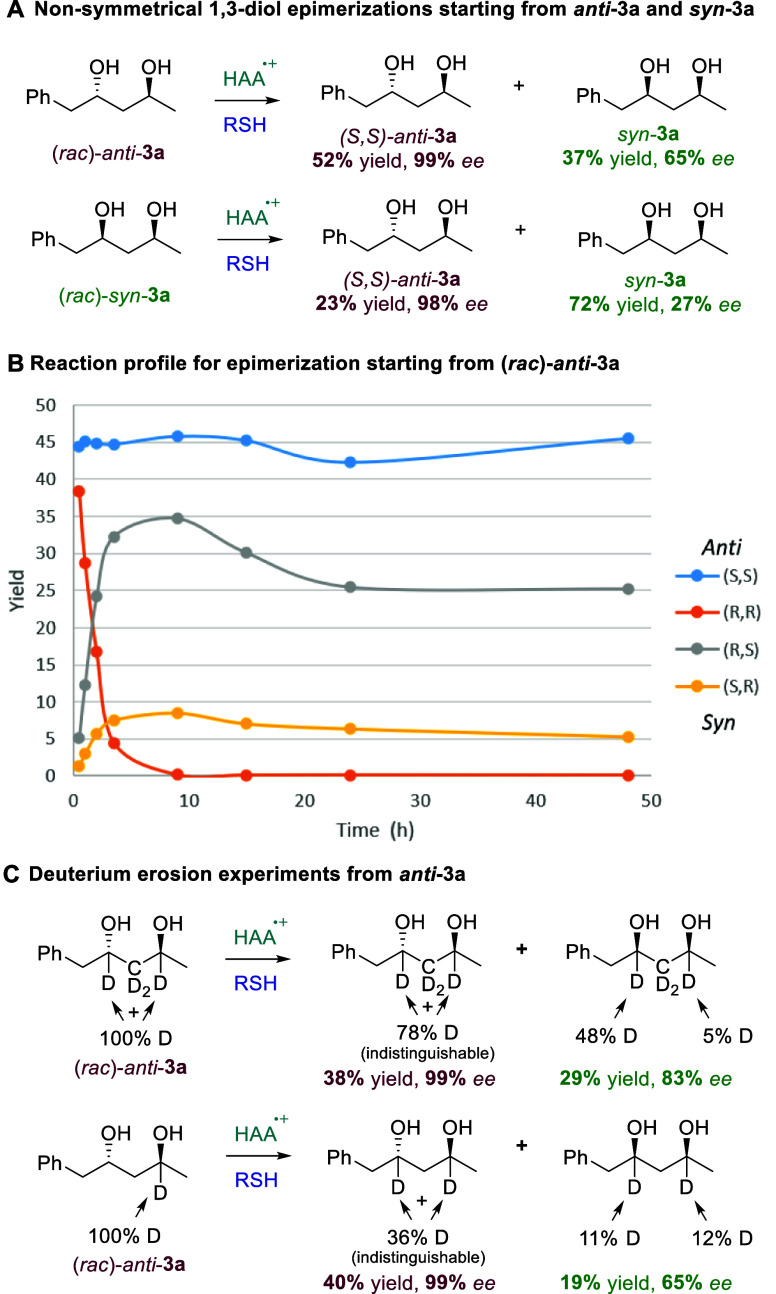
Investigation of Nonsymmetrical 1,3-Diol 3a

We proceeded to evaluate the epimerization on a selection of nonsymmetrical 1,3-diols, starting from the racemic *anti*-diastereomer bearing a variety of functional groups. In all cases, the recovered *anti*-diastereomer was obtained in very high *ee*, in many cases 99% (Scheme 5). The formed *syn*-diastereomer exhibited varying levels of *ee* dependent on the substrate. For variants of **3a** featuring a *para*-methoxybenzyl (**3b**) and naphthyl (**3c**), good results were obtained, with a surprising switch in the major diastereomer for **3b**, for which the cause is not clear. We extended the chain connecting the diol to the aromatic ring in **3d**. Again, outstanding *ee* in the major *anti*-diastereomer but lower in the *syn*, a trend which is broadly consistent for substituents at various positions of the benzene ring, including ester (**3e**), trifluoromethyl (**3f**), nitrile (**3g**), and trifluoroacetamide (**3h**). The *ee* of the *syn* diastereomer in these cases varied in the range 32–79%, while that of the *anti* was >97% in all but one. It is notable that in all cases so far, both diastereomers could be separated and isolated using standard column chromatography. Diols containing larger substituents than methyl on both sides could also be epimerized (**3i** and **3j**). Notably, **3j**, containing a Boc-protected piperidine, gave high *ee* values for both diastereomers. It was interesting that larger linear substituents, as in **3k** and **3l**, gave negligible *ee* for the *syn* diastereomer, although the *anti* diastereomer was still high. This might be reflective of the local symmetry close to the alcohols, leading to negligible regioselectivity during the HAA (*k*_*1*_ vs *k*_*16*_, Figure 2C). Finally, a sulfone could be included relatively close to the diol unit and gave surprisingly high *ee* values for each diastereomer (**3m**).

**Scheme 5:**
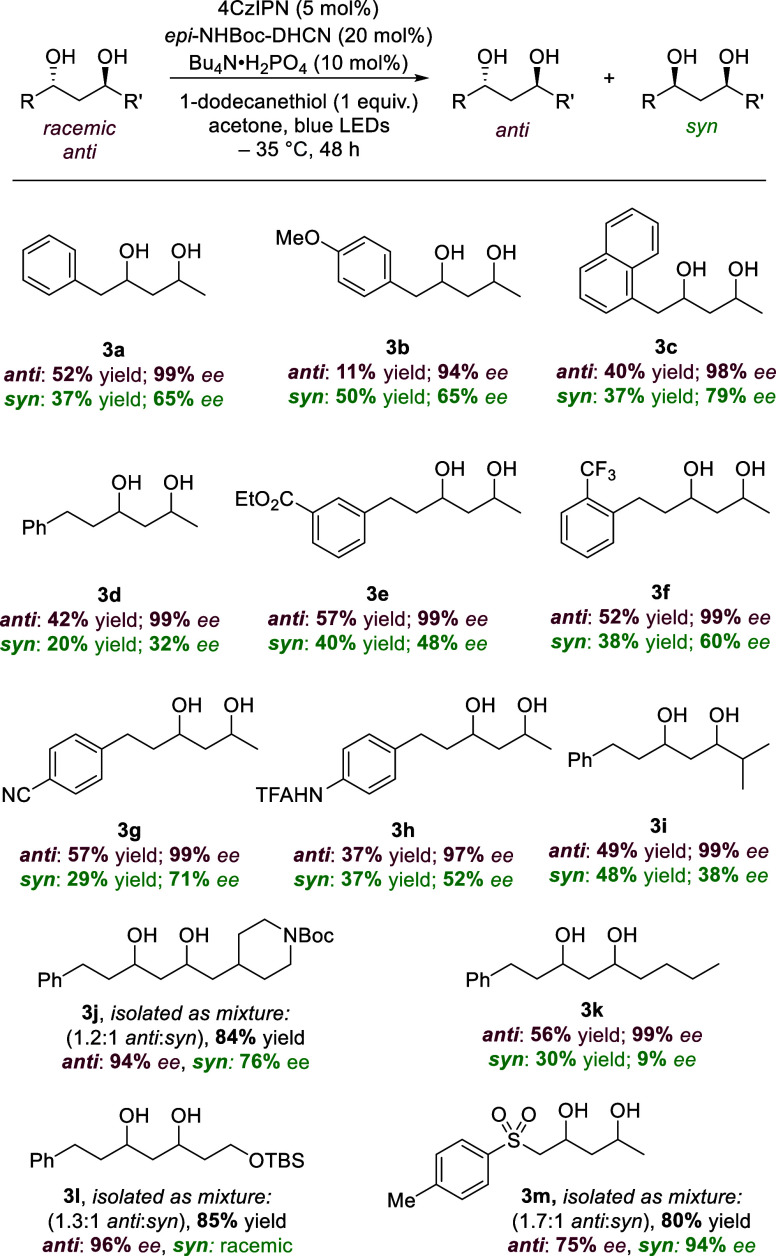
Scope of Nonsymmetrical 1,3-Diols^a^

a The indicated yield is the isolated yield after column chromatography.

Having systematically assessed various 1,3-diol substrates, we advance a tentative rationalization of their behavior. It should be emphasized that this is a qualitative analysis based on the experimental results in hand. The potential complexity of the underlying processes and the fact that some abstraction/delivery processes are effectively “unseen” without specific analysis necessitate caution in any interpretation. The first key observation is that one enantiomer (*R*,*R*) of *anti*-diol appears to be consistently far more reactive with the chiral HAA catalyst than the other *anti*-enantiomer (*S*,*S*) and the *syn*-diol. The second key observation is that reactivity with the HAA catalyst appears to decrease quickly as the length of the substituent adjacent to the alcohol is increased. It should be noted though that for the highly reactive *anti*-enantiomer, long chains do not shut down reactivity. These observations can be appreciated in a series of Scenarios (Scheme 6). In Scenario 1, both substituents are small and identical. The reactive *anti* enantiomer is very rapidly depleted to the *syn*, here achiral. Because the substituents are small, both the *syn* diastereomer and the less reactive *anti* enantiomer are still able to react and over time can establish a kinetic equilibrium which features both, as in substrate **1a** (Scheme 1B). Moving to Scenario 2, one small substituent is replaced by a larger one to give a nonsymmetrical diol, as in substrate **3a**. The unreactive *anti* enantiomer persists. The reactive *anti* enantiomer, now no longer C2-symmetric, presents the HAA catalyst with a choice of hydrogen atoms to abstract. The less hindered red hydrogen is more accessible than the green and is preferentially abstracted. This leads to enantioenrichment of the formed *syn* diastereomer, albeit at a moderate level. Although less reactive, the *syn* can be further abstracted from, most likely at the less hindered hydrogen atom, the extent of this depends on the exact substrate, giving *ee* of the *syn* which varies from one to another. Through these pathways, some *syn* can feasibly be converted to the unreactive *anti*-enantiomer (blue, *S*,*R* to *S*,*S*), meaning that for some substrates, >50% yield can be obtained with 99% *ee* (e.g., **3a**, **3e**-**3g**). Scenario 3 presents two identical, large substituents, as in substrate **1d**. Here, after conversion of the reactive *anti*-diol to the *syn*, there is little further development as the reactivity of the other two stereoisomers is very low, most likely due to lower steric accessibility, in contrast with Scenario 1. This constitutes a highly effective kinetic resolution process in which one enantiomer is selectively epimerized to a separable, achiral diastereomer (Scheme 3A). Scenario 4 considers two large substituents that are different, but only minimally, as in substrate **3k** or **3l**. Because the two substituents appear very similar to the catalyst, HAA from the reactive *anti* is essentially nonselective (red vs green), leading to a nearly racemic mixture of *syn* isomers. It should be noted that not all substrates fall neatly into this rationalization. For example, **3i** and **3j** feature bulk in the form of branching on one side and linear bulk on the other and yet give modest to high ee in the *syn*.

**Scheme 6:**
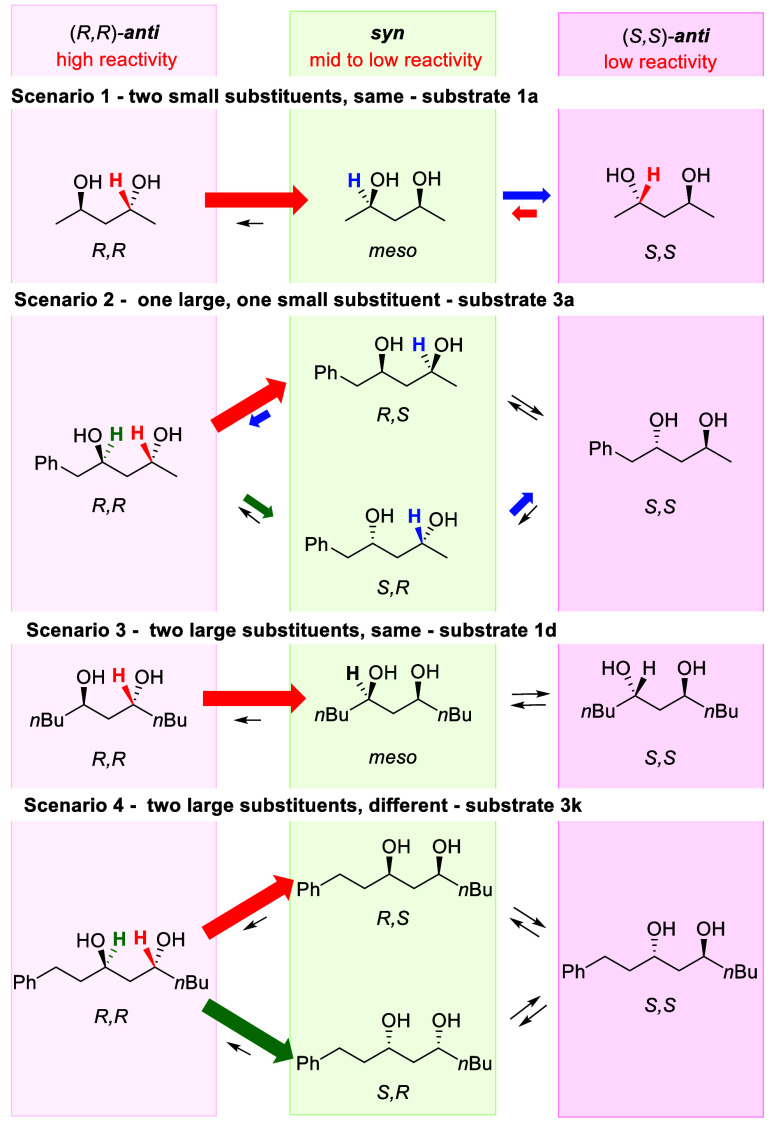
General Trends in 1,3-Diol Epimerization

Finally, we determined whether these trends translated to acyclic 1,2-diols. In our prior work, we had explored epimerization of a range of symmetrical 1,2-diols, starting in all cases from the *meso*-diol.^10^ There is also the possibility for dynamic stereochemical processes to occur for these substrates, and therefore, we systematically evaluated acyclic 1,2-diols, this time starting from the racemic *syn* diastereomer, with gradually increasing chain length (Scheme 7). For the shortest, butane-2,3-diol (**4a**), analysis of the crude reaction showed 60% yield of the *syn*-diastereomer and 7% of the *meso*, although it is feasible in this case that yields could be compromised by volatility. Following derivatization, *syn* was found to have 97% *ee*. A very similar outcome was obtained going from methyl to ethyl on either side of the diol, in this case with even less *meso* being formed, despite the *meso* certainly constituting an intermediate in the deracemization (**4b**). Interestingly, the next incremental increase in chain length from ethyl to propyl prompted a significant drop in ee to 60%, still with very little *meso*-diol (**4c**). This trend was reinforced by moving to *n*-butyl, which reduced *ee* again to 44% (**4d**). Longer chains terminated with esters delivered a similar outcome at 52% *ee*, although in this case, significant amounts of the *meso* diastereomer formed (**4e**). Termination with phenyl rings gave poor outcomes (**4f**, **4g**). The smallest two substrates aside, these outcomes contrast sharply with the analogous process commencing from the *meso* diol, as in our previously published work, where high *ee* could be obtained for the majority of substrates, regardless of chain length.^10^ Combined, these trends indicate that for the 1,2-diols, the *meso*-diastereomer (referred to in 1,2-diol form as the *anti* diastereomer) is much more reactive than the chiral diastereomer (here the *syn*), consistent with what we previously concluded.^10^ The deracemization process for this substrate class is limited to only the shortest chain lengths on either side of the diol subunit. It appears that as the chain length increases, the reactivity of any isomer but the *meso* decreases and useful outcomes cannot be achieved.

**Scheme 7:**
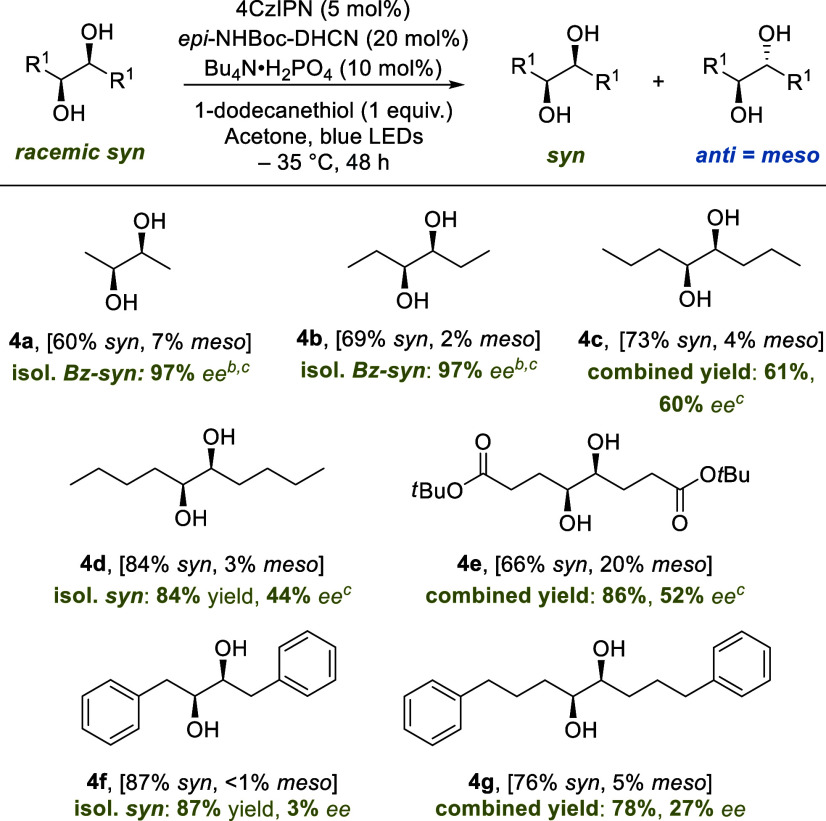
Evaluation of Symmetrical 1,2-Diols^a^–[Fn s7fn3]

a Yields in parentheses determined by NMR with reference to the internal standard.
b Volatility necessitated isolation as a dibenzoyl ester.
c
*ee* determined after conversion to dibenzoyl ester

To complete the survey, we evaluated nonsymmetrical 1,2-diols, returning to the more complex scenario of four potential stereoisomers (Scheme 8). We elected to start from the racemic *anti*-diastereomers on the basis that these should likely be more reactive than the *syn* diastereomers (see above). Submission of the racemic *anti*-diastereomer of **4h** to the epimerization conditions resulted in a 2:1 ratio of *syn*/*anti*, which was inseparable on silica. Encouragingly, analysis of this mixture showed that each diastereomer was highly enantioenriched (89% and 87% *ee*). Moving the phenyl ring closer to the diol in **4i** gave a similarly high *ee* outcome, this time with a diastereomer ratio favoring the *syn* isomer in a 5:1 ratio. Both substrates possessed a methyl group on one side of the diol and a larger substituent on the other. Increasing the size of the small substituent to propyl in **4j** gave a drastic decrease in the ee of the *anti*-isomer, while that of the *syn*-isomer remained very high, mirroring what had been observed in the related 1,3-diol substrate (**3k**). We explored several more diols possessing a methyl group on one side; a bulky diphenylmethine unit on the other intriguingly favored the *anti*-diastereomer, although its *ee* was reduced (**4k**). Longer chains with functionality (**4l,m**) gave generally good results with the *syn* being modestly favored in both cases and displaying 92% and 93% ee.

**Scheme 8:**
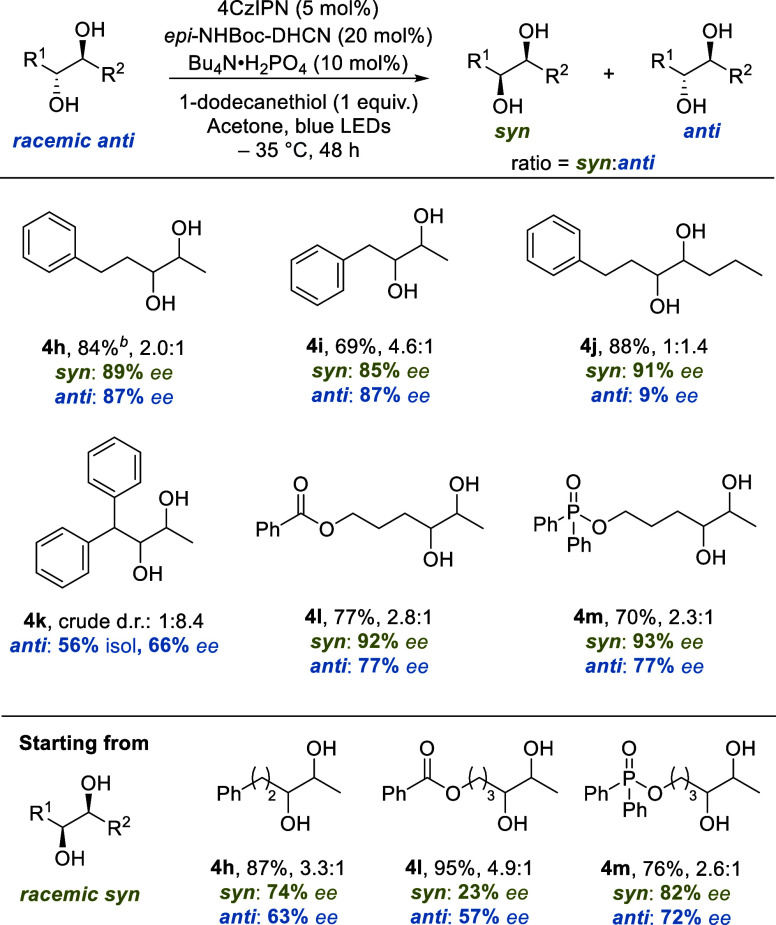
Evaluation of Nonsymmetrical 1,2-Diols^a^ ^b^

a Indicated yields are isolated after column chromatography, except where indicated.
b Yield by NMR with reference to internal standard, partially enriched samples were isolated—see the Supporting Information for details.

We also evaluated three substrates starting from the racemic *syn*-diastereomer. Phosphonate **4m** gave a very similar outcome in terms of *ee* and dr, suggesting that a kinetic equilibrium was probably reached in this case. In contrast, *syn*-**4l** gave quite different metrics, indicating a slower reaction starting from *syn* in this particular case. In summary, it seems that very good *ee* outcomes in both diastereoisomers can be achieved for the 1,2-diols as long as methyl is featured as one substituent. Otherwise, the best that can be achieved is a kinetic resolution-type outcome (as in **4j**).

For both the 1,3-diol and 1,2-diol scopes demonstrated, we commenced from a single racemic diol diastereomer. In line with the goal of developing stereochemical descrambling processes, we demonstrate for two representative substrates, **3c** and **4h**, that a mixture of racemic diastereomers can be started from (Scheme 9). These examples transform a mixture of four stereoisomers into one that predominantly comprises two. Although much development is still required, we believe that this is an indicator of how catalyst-controlled epimerization has the potential to be enabling in synthesis. With further catalyst development and understanding, higher diastereoselectivity and the ability to target different stereoisomers can be anticipated.

**Scheme 9:**
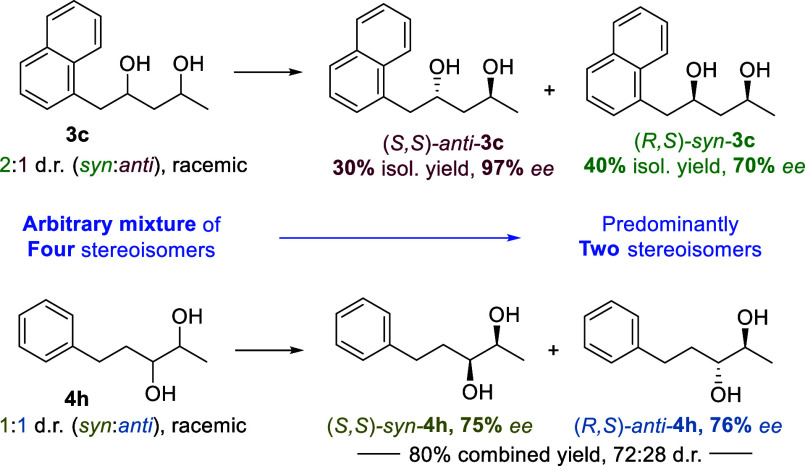
Commencing from a Racemic Mixture of Diastereomers

## Conclusion

We have systematically explored the epimerization of 1,3-diols using a combination of our chiral HAA catalyst *epi*-NHBoc-DHCN and an achiral thiol. From this study, a consistent picture has emerged whereby the (*R*,*R*) enantiomer of the *anti*-diol has far greater reactivity with the catalyst than either the (*S*,*S*) or the *syn*-configured isomers. The length of the substituents on either side of the diol unit impacts the reactivity with the HAA catalyst, contributing different extents of dynamic behavior for different substrate classes, which have been comprehensively explored. In symmetrical 1,3-diols, our chiral HAA catalyst performs a highly effective kinetic resolution in which one enantiomer of the racemate is converted to the *meso* diastereomer with extremely high enantioselectivity. This constitutes a rare example of a kinetic resolution process whereby one enantiomer is converted to an achiral diastereomer. For nonsymmetrical 1,3-diols, where both diastereoisomers are chiral, our catalyst is able to give remarkably high enantioselectivities for the *anti*-diastereomer. For the *syn*-diastereomer, in many cases moderate to high *ee* can also be achieved if sufficient steric differentiation exists between the groups on either side of the 1,3-diol unit. We have also carried out analogous investigations of chiral, racemic 1,2-diols. We had already established in previous work that a wide range of symmetrical *anti*-1,2-diols, *meso* compounds, had high reactivity with our catalyst and underwent desymmetrizing epimerization with high *ee*.^10^ Here, we found that the *syn* 1,2-diols (chiral) appear to have much lower reactivity unless the substituents are very small, such as methyl or ethyl. This means that deracemization can only be achieved for the very smallest of such diols. For nonsymmetrical 1,2-diols, the situation is more complex but high *ee* of both *syn* and *anti* diastereomers can be achieved with sufficient steric differentiation, clearly showing the potential for further development and exploration, potentially in combination with further catalyst design modifications or with a chiral hydrogen atom donor. This work constitutes a foundational study toward complex dynamic processes, based on HAT, which have the potential to “descramble” mixtures of stereoisomers in a fundamentally different approach to stereocontrol in synthesis.

## Supplementary Material





## Notes

The Supporting Information is available free of charge at https://pubs.acs.org/doi/10.1021/jacs.6c10166. The kinetic simulator html can be downloaded from the following link https://repository.nottingham.ac.uk/entities/product/3adc90df-b092-4f37-b0d9-4377ea3f36e2.

Kinetic simulator html (ZIP)
Additional reaction optimization, procedures, and characterization data (PDF)
