# Supplementary material for: Stereoselective Epimerization of 1,3-Diols Using a Chiral Hydrogen Atom Abstraction Catalyst
Source: J Am Chem Soc. 2026 Jul 30;148(31):33641–51. doi: 10.1021/jacs.6c10166 (PMC13474961; doi:10.1021/jacs.6c10166)

Stereoselective Epimerization of 1,3-Diols using a Chiral Hydrogen Atom Abstraction Catalyst

Miran Lemmerer, [#a] Darcy C. Emmet, [#a] Antti S. K. Lahdenperä,[a] Daniel J. Davies,[a] Amit Dahiya,[a] Kristaps Ermanis[b] and Robert J. Phipps*[a]

[a] Yusuf Hamied Department of Chemistry, Lensfield Road, Cambridge, CB2 1EW, United Kingdom.

[b] School of Chemistry, University of Nottingham, University Park, Nottingham NG7 2RD United Kingdom

* rjp71@cam.ac.uk.

Supporting Information: Experimental

Contents

General remarks 2

Synthesis of Substrates 3

- General procedures 3
- Synthesis and Characterization of Racemic 1,3-Diols 5
- Synthesis and Characterization of Racemic 1,2-Diols 41

Stereoselective epimerization of diols 54

- General Procedures 54
- Scheme 1B and 1C - Reaction profiles for epimerization starting from rac-1 and (*R*,*R*)-1 56
- Scheme 1D – individual experiments from pentan-2,4-diol 59
- Scheme 2 Scope of symmetrical 1,3-diols........ 63
- Scheme 3A - Reaction Profile Starting from *rac*-(**1d**) 74
- Scheme 3B - Reaction starting from *syn*-1,3-diol (**1d**) 75
- Scheme 3C – Deuterium Studies on Symmetrical 1,3-diol (1d) 76
- Scheme 3D – Giese addition 79
- Scheme 3E - Oxidation 81
- Scheme 3F – Access to Opposite Enantiomer........ 87
- Scheme 3G - 1 mmol scale up of symmetrical 1,3-diol (**1d**)........ 88
- Scheme 4A – Non-symmetrical 1,3-diol 3a 91
- Scheme 4B – Reaction Time Course Study for Epimerization of *rac-anti*-**3a** 93
- Scheme 4C – Deuterium Experiments Using non-Symmetrical Diol 95
- Scheme 5 – Scope of non-symmetrical 1,3-diols 99
- Scheme 7. Evaluation of Symmetrical 1,2-diols 111
- Scheme 8 – Evaluation of non-symmetrical 1,2-diols 121
- Scheme 9 - Commencing from a racemic mixture of diastereomers. 126
- Catalyst degradation study 127
- Deuterium incorporation studies........ 128
- Absolute stereochemistry assignments 130
- Noyori Hydrogenation to Confirm Absolute Stereochemistry of 3a with Authentic Sample........ 133
- Sharpless dihydroxylation to Confirm Absolute Stereochemistry of **4l** with Authentic Sample 138

References 140

General remarks

Reagents: All reagents were used as supplied from commercial sources without further purification. Acetone used in the deracemization reaction was HPLC grade (≥ 99.8%) and degassed in 100 mL or 250 mL batches by purging with nitrogen for 2 – 3 hours followed by storing under a nitrogen atmosphere.

Reaction setup: Deracemization reactions were carried out in 4 mL, 15 × 45mm crimp-top vials. Reactions were performed in a custom-made cooling setup (Picnic Photo reactor) under a nitrogen atmosphere at –35 °C as detailed in previous work.[1, 2] The synthesis of 4CzIPN was conducted as described in the literature.[3] The syntheses of HAA catalysts epi-NHBocdihydrocinchonine (epi-NHBoc-DHCN) and epi-NHBoc-dihydrocinchonidine (epi-NHBocDHCD) were conducted as previously reported.[1]

NMR spectra: NMR spectra were recorded on a 700 MHz Bruker TXO spectrometer, DRX600 spectrometer, 500 MHz Bruker DCH Cryoprobe, 400 MHz Bruker QNP Cryoprobe or 400 MHz Bruker Avance NEO prodigy N2 Cryoprobe. Chemical shifts are reported in parts per million (ppm) and the spectra are calibrated to the resonance resulting from incomplete deuteration of the solvent (^{1}H- NMR: $CDCl_3$: 7.26 ppm, s; ^{13}C-NMR: $CDCl_3$: 77.16 ppm, t). Data are reported as follows: chemical shift δ/ppm, integration, multiplicity (s = singlet, d = doublet, t = triplet, q = quartet, br. = broad, m = multiplet or combinations thereof; ^{13}C signals are singlets unless otherwise stated), coupling constants *J* in Hz.

High Resolution Mass Spectrometry (HRMS): Recorded on a Waters Xevo G2-S bench top QTOF using an electrospray ionization (ESI). Measured values are reported to four decimal places and are within ±5 ppm of the calculated value. The calculated values are based on the most abundant isotope.

Chromatography: Analytical and preparatory thin layer chromatography was performed using precoated Merck glass backed silica gel plates (Silicagel 60 F254). Visualisation was by ultraviolet fluorescence (λ = 254 or 365 nm) and/or staining with potassium permanganate. Flash column chromatography was performed using silica gel 60 (0.040-0.063 µm).

Optical rotations: Measured in spectrophotometric grade $CHCl_3$ or MeOH or HPLC grade EtOH on an Anton Paar Polarimeter using a sodium lamp (λ = 589 nm, D-line). $[\alpha]_D$ values are reported at a given temperature (°C) with concentration in g/100mL.

Chiral HPLC analysis: Performed on a Waters ARC-HPLC system with DAICEL CHIRALPAK SC column (4.6 × 250 mm, 3 µm) in a mixed solvent system of *n*hexane and *i*PrOH.

Chiral SFC analysis: Performed on a Waters ACQUITY UPC2 system with DAICEL CHIRALPAK IA, IC, IE, IJ or IK columns (4.6 x 250 mm, 3 µm) in a mixed solvent system of supercritical CO_2 and MeOH. A system backpressure of 138 bar was used in all cases.

Synthesis of Substrates

General procedures

General Procedure 1: Symmetrical 1,3-diol synthesis via epoxide opening

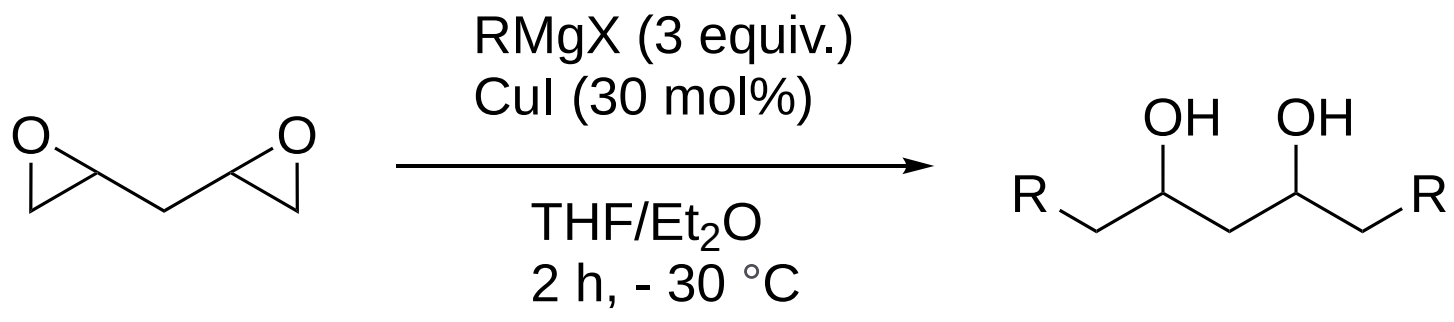

Preparation based on a literature procedure.[4] Grignard reagent (3 equiv.) was added to a solution of CuI (30 mol%) in dry THF/Et_2O (1 M) at - 30 °C and stirred for 5–10 minutes. Then a solution of di(oxiran-2-yl)methane (1 equiv.) in dry THF/Et_2O (0.9 M) was added and the reaction mixture was stirred at - 30 °C for 2 h then quenched with NH_4Cl. The reaction mixture was then extracted with Et_2O, and the organic layer dried over $MgSO_4$ then concentrated *in vacuo*. The crude product was purified *via* column chromatography to give a pure mixture of symmetrical *syn*- and *anti*-1,3-diols.

General Procedure 2: Aldol reaction

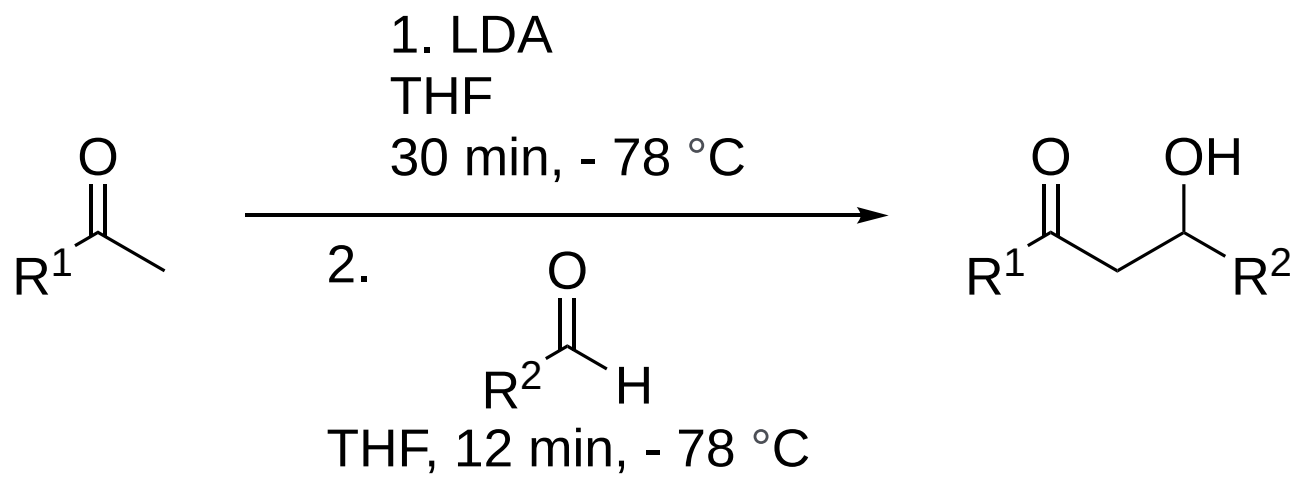

Preparation based on a literature procedure.[5] *N*-butyllithium (1.1 equiv.) was added slowly dropwise to a solution of diisopropylamine (1.1 equiv.) in dry THF (0.9 M) at – 78 °C. The reaction mixture was then allowed to warm to 0 °C before being cooled back down to – 78 °C. Ketone (1 equiv.) in dry THF (1 M) was then added slowly dropwise to the reaction mixture and stirred for 30 minutes. Then aldehyde in dry THF (1 M) was added slowly dropwise to the reaction mixture which was then stirred for 12 minutes before being quenched with NH_4Cl and then stirred at room temperature for 20 minutes. Et_2O was then added, and the reaction mixture stirred for 5 minutes. The layers were then separated, where the organic layer was washed with NH_4Cl and brine and the aqueous layer was back extracted with Et_2O. The organic layers were combined then dried over $MgSO_4$ and the solvent removed *in vacuo*. The crude product was purified *via* silica gel chromatography to give the hydroxyketone.

General Procedure 3: Evans-Saksena reduction

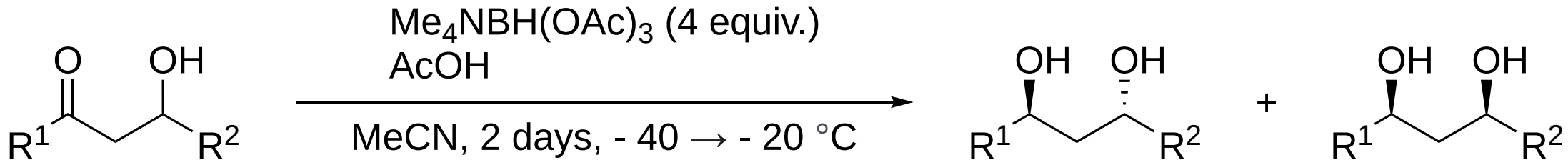

This procedure was adapted from the literature.[6] To a flask containing MeCN:AcOH (1:1, 0.1 M with respect to the hydroxy ketone) was added $Me_4NBH(OAc)_3$ (4.0 equiv.) and stirred at room temperature for 30 min. Then it was cooled to – 40 °C leading to the mixture freezing. The hydroxy ketone (dissolved in MeCN (0.5 M)) was added and the flask kept at – 40 °C for 1 h. Then it was placed in a freezer at – 25 °C and left for 3 days. Then, sodium potassium tartrate (10 mL/mmol) was added and filtered

through a pad of celite. The mixture was extracted with DCM (3 x 10 mL/mmol) and the combined organic fractions dried over $MgSO_4$. The solvent was removed under reduced pressure and MeOH was added (10 mL). The solvent was removed under reduced pressure again and the addition of MeOH was repeated two more times. The crude material was purified by flash column chromatography on silica gel yielding (*anti*)-diol as the major and (*syn*)-diol as the minor product.

General Procedure 4: Dihydroxylation of symmetrical alkenes

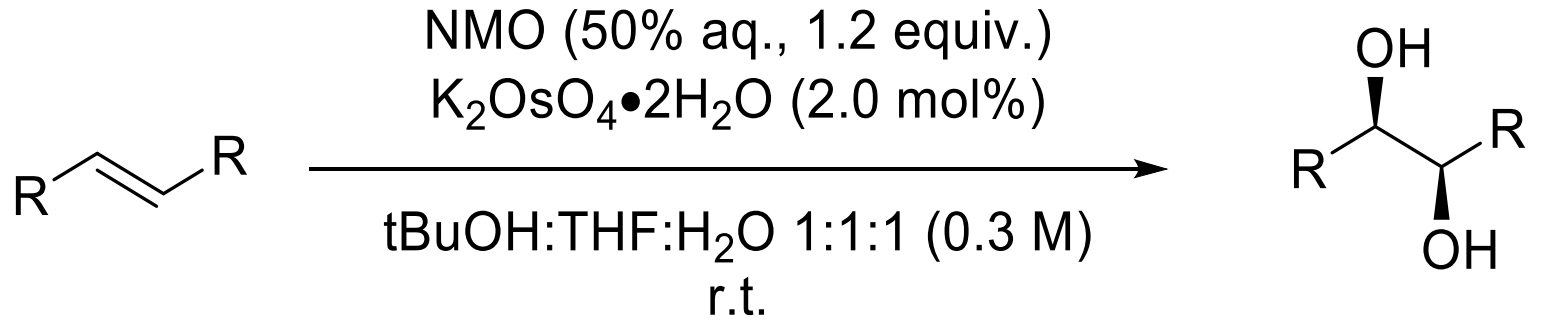

A solution of *N*-methylmorpholine-*N*-oxide (NMO) (50% w/w in water, 1.2 eq.) was added to a stirred solution of alkene substrate (1.0 eq.) and $K_2OsO_4{\cdot}2H_2O$ (2.0 mol%) in *tert*-butanol/THF/H_2O (1:1:1 v/v, 0.3 M). The solution was stirred at r.t. for the specified time, quenched with Na_2SO_3 and extracted with ethyl acetate. The combined organic extracts were dried over $MgSO_4$, filtered, and the solvent removed *in vacuo* to afford crude mixture. The crude mixture was purified by silica gel column chromatography to afford *syn* diol product.

General Procedure 5: Dihydroxylation of unsymmetric alkenes

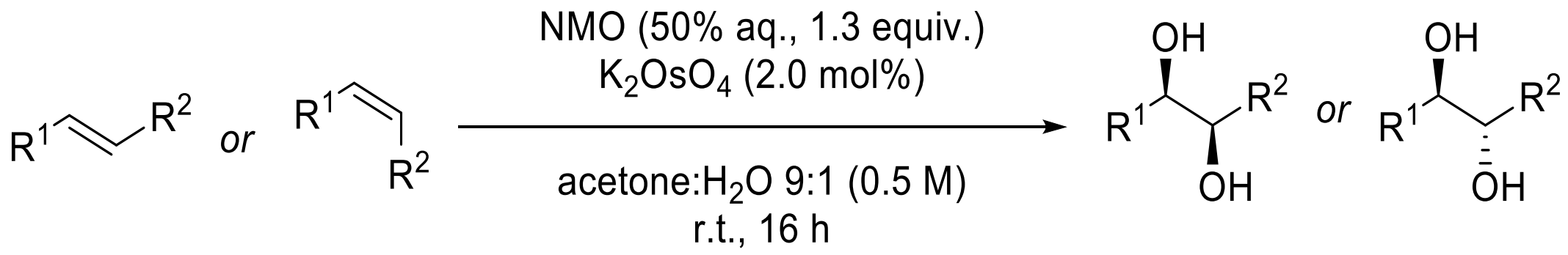

In a vial, the alkene (1.0 equiv.) was dissolved in acetone:water (9:1, 0.5 M) and potassium osmate (0.02 equiv) was added. The vial was sealed with a septum and 4-methylmorpholine N-oxide (50% in water, 1.3 equiv.) was added. The mixture was stirred vigorously for 16 h. Then $Na_2S_2O_3$ (sat. aq., 1 mL/mmol alkene) was added and stirred for 30 min. The mixture was extracted with EtOAc (3 x 5 mL/mmol alkene) and the combined organic fractions dried over $MgSO_4$. The solvent was removed under reduced pressure and the crude material purified by flash column chromatography on silica gel.

General procedure 6: Acylation of 4-hexene-1ol

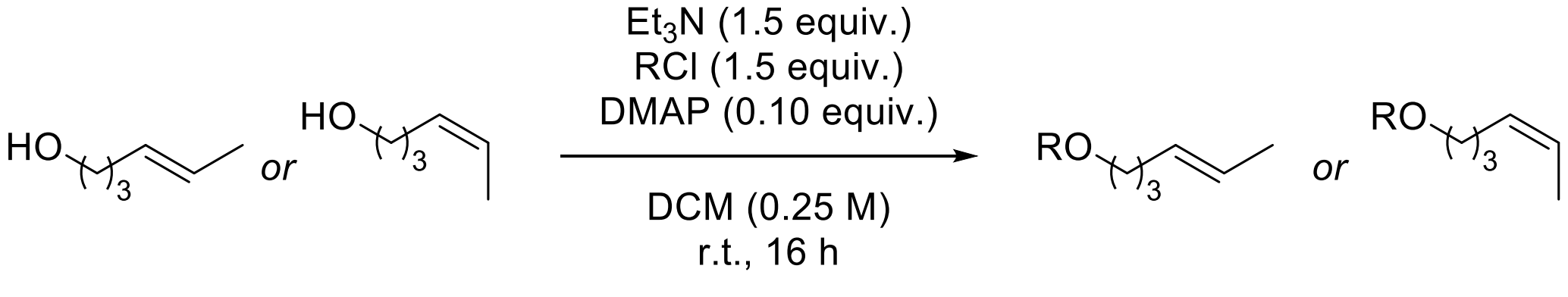

To a heat gun-dried flask was added 4-hexen-1-ol (500 mg, 5 mmol, 1.0 equiv.), dry DCM (20 mL), triethylamine (1.1 mL, 7.5 mmol, 1.5 equiv.), 4-dimethylaminopyridine (61.1 mg, 0.50 mmol, 0.10 equiv.) and acyl chloride (1.5 equiv.). The mixture was stirred for 16 h upon which water (20 mL) was added and the layers separated. The aqueous layer was extracted with DCM (20 mL) and the combined organic fractions dried over $MgSO_4$. The solvent was removed under reduced pressure and the crude material purified by flash column chromatography on silica gel.

Synthesis and Characterization of Racemic 1,3-Diols

Di(oxiran-2-yl)methane

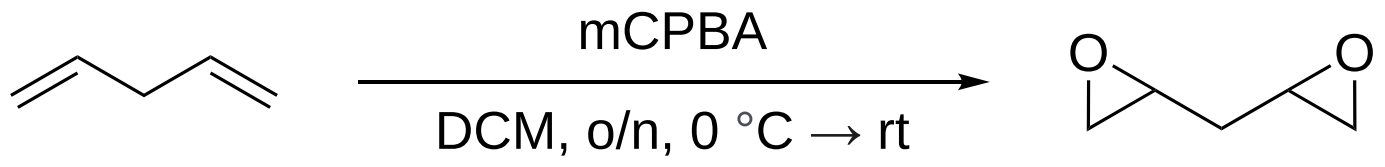

Preparation based on a literature procedure.[4] mCPBA (77% by weight) was added in five portions (8.22 g, 36.7 mmol) to a solution of 1,4-pentadiene (7.6 ml, 73.4 mmol) in DCM (163 ml) at 0 °C. The reaction mixture was then stirred at room temperature overnight before being quenched with saturated $NaHCO_3$ solution (109 ml). The organic layer was then washed with 1 M KOH (7 × 54 ml), dried over $MgSO_4$ and concentrated *in vacuo* to give di(oxiran-2-yl)methane (5.14 g, 51.30 mmol, 70%) as a 1:1.1 mixture of diastereomers and a colourless liquid.

^{1}H NMR (700 MHz, $CDCl_3$) δ 3.11 – 3.07 (m, 2H), 3.05 – 3.01 (m, 2H), 2.81 (dd, *J* = 4.9, 4.0 Hz, 2H), 2.79 (t, *J* = 4.4 Hz , 2H), 2.59 (dd, *J* = 4.9, 2.7 Hz, 2H), 2.53 (dd, *J* = 5.0, 2.7 Hz, 2H), 2.00 (dt, *J* = 14.9, 4.3 Hz, 1H), 1.77 – 1.72 (m, 3H) ppm.

^{13}C NMR (176 MHz, $CDCl_3$) δ 49.5, 48.9, 46.9, 46.3, 36.2, 34.8 ppm.

(*anti*)-2,4-pentane diol (rac-1a)

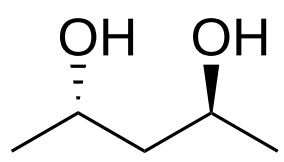

rac-**1a**

Prepared according to literature procedure.[1]

heptane-3,5-diol (1b)

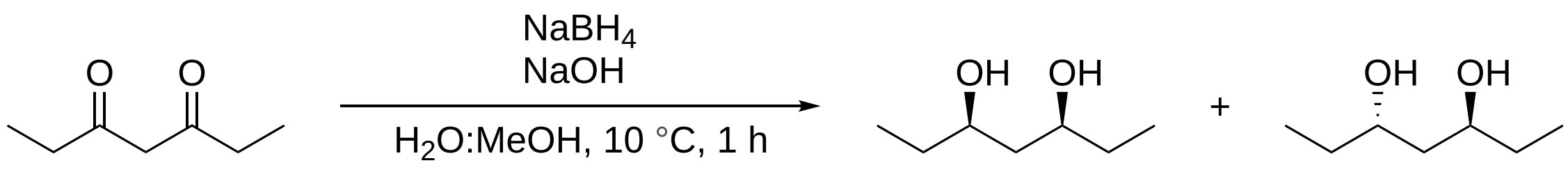

Preparation based on a literature procedure.[7] Sodium borohydride (290 mg, 7.7 mmol) and sodium hydroxide (46 mg, 1.2 mmol) were dissolved in water (3.5 ml) and cooled to 10 °C. 3,5-heptanedione (0.95 ml, 7.0 mmol) in methanol (3.5 ml) was added dropwise to the reaction mixture which was then stirred for 1 hour. After this the reaction mixture was concentrated and the aqueous layer extracted with Et_2O (3 × 4 ml) then dried over $MgSO_4$. The crude product was purified *via* silica gel chromatography (5 – 10% Acetone in Pet. Ether) to give (*syn*)-heptane-3,5-diol (264 mg, 1.99 mmol, 28%) as a colourless oil and (*anti*)-heptane-3,5-diol (385 mg, 2.91 mmol, 42%) as a colourless oil.

(*syn*)-heptane-3,5-diol (*meso*-1b)

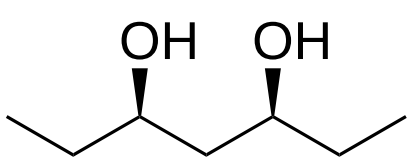

meso-**1b**

^{1}H NMR (700 MHz, $CDCl_3$) δ 3.80 – 3.75 (m, 2H), 3.17 (br s, 2H), 1.61 (dt, *J* = 14.5, 2.1 Hz, 1H), 1.52 – 1.45 (m, 4H), 1.45 – 1.39 (m, 1H), 0.92 (t, *J* = 7.5 Hz, 6H) ppm.

^{13}C NMR (176 MHz, $CDCl_3$) δ 74.6, 41.9, 31.1, 9.8 ppm.

Data is in good agreement with the literature.[8]

(*anti*)-heptane-3,5-diol (*meso*-1b)

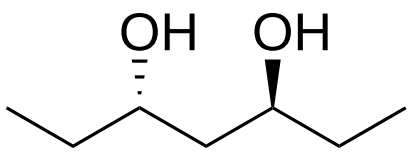

rac-**1b**

^{1}H NMR (700 MHz, $CDCl_3$) δ 3.85 (p, *J* = 6.0 Hz, 2H), 2.53 (br s, 2H), 1.60 (dd, *J* = 6.0, 5.3 Hz, 2H), 1.58 – 1.46 (m, 4H), 0.94 (t, *J* = 7.5 Hz, 6H) ppm.

^{13}C NMR (176 MHz, $CDCl_3$) δ 70.9, 41.5, 30.4, 10.2 ppm.

Data is in good agreement with the literature.[8]

nonane-4,6-diol (1c)

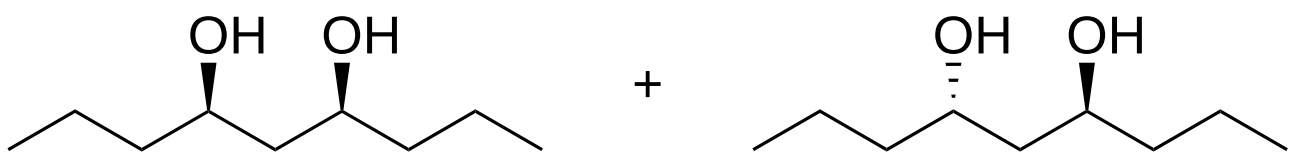

meso-**1c** *rac*-**1c**

Prepared according to **General Procedure 1** using ethylmagnesium chloride solution (7.5 ml, 15 mmol, 2.0 M in THF), CuI (299 mg, 1.5 mmol, 30 mol%), di(oxiran-2-yl)methane (500 mg, 5.0 mmol) and THF. The crude product was purified *via* column chromatography (5% Acetone in Pet. Ether) to give (*syn*)-nonane-4,6-diol (190 mg, 1.18 mmol, 24%) as a colourless oil, (*anti*)-nonane-4,6-diol (101 mg, 0.63 mmol, 13%) as a white solid and mixed fractions (87 mg, 0.55 mmol, 11%).

(*syn*)-nonane-4,6-diol (*meso*-1c)

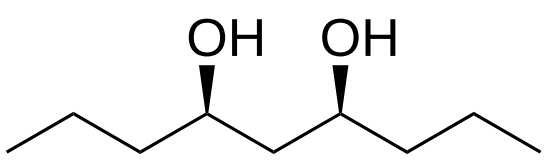

meso-**1c**

^{1}H NMR (700 MHz, $CDCl_3$) δ 3.89 – 3.84 (m, 2H), 2.90 (br s, 2H), 1.59 (dd, *J* = 14.5, 1.5 Hz, 1H), 1.51 – 1.38 (m, 7H), 1.38 – 1.31 (m, 2H), 0.93 (t, *J* = 7.0 Hz, 6H) ppm.

^{13}C NMR (176 MHz, $CDCl_3$) δ 73.1, 43.1, 40.6, 18.7, 14.2 ppm.

HRMS [M+H]$^+$ m/z calc'd for $[C_9H_{21}O_2]^+$ expect 161.1542; found 161.1537

(*anti*)-nonane-4,6-diol (*rac*-1c)

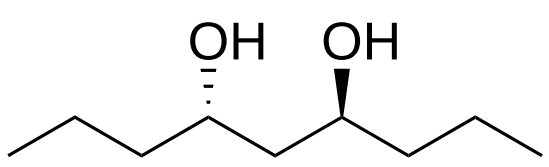

rac-**1c**

^{1}**H NMR** (700 MHz, $CDCl_3$) δ 3.99 – 3.93 (m, 2H), 2.27 (d, *J* = 4.1 Hz, 2H), 1.61 (dd, *J* = 5.9, 5.3 Hz, 2H), 1.56 – 1.50 (m, 2H), 1.49 – 1.40 (m, 4H), 1.39 – 1.31 (m, 2H), 0.94 (t, *J* = 7.2 Hz, 6H) ppm.

^{13}**C NMR** (176 MHz, $CDCl_3$) δ 69.4, 42.5, 39.8, 19.1, 14.2 ppm.

HRMS [M+Na]$^{+}$ m/z calc'd for $[C_9H_{20}O_2Na]^+$ expect 183.1361; found 183.1357

undecane-5,7-diol (1d)

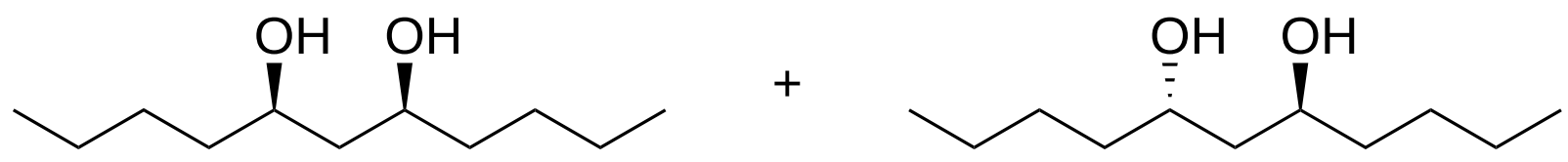

meso-**1d** *rac*-**1d**

Prepared according to **General Procedure 3** using 7-hydroxyundecan-5-one (r*ac*-2c) (689 mg, 3.70 mmol, 1 equiv.), $Me_4NBH(OAc)_3$ (4.2 g, 15.6 mmol, 4 equiv.) and glacial acetic acid (19.5 ml) stirring overnight. The crude product was purified *via* column chromatography (5% Acetone in Pet. Ether) to give (*syn*)-undecane-5,7-diol (31 mg, 0.16 mmol, 4%) as a colourless oil, (*anti*)-undecane-5,7-diol (229 mg, 1.22 mmol, 33%) as a white solid and mixed fractions (169 mg, 0.90 mmol, 24%).

(*syn*)-undecane-5,7-diol (*meso*-1d)

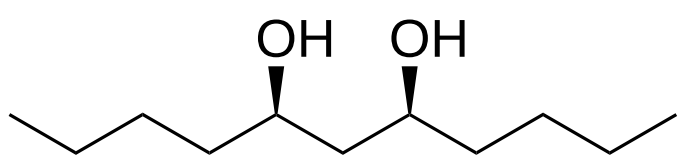

meso-**1d**

^{1}**H NMR** (700 MHz, $CDCl_3$) δ 3.85 – 3.81 (m, 2H), 3.14 (s, 2H), 1.59 (dt, *J* = 14.5, 2.2 Hz, 1H), 1.51 – 1.40 (m, 5H), 1.40 – 1.26 (m, 8H), 0.89 (t, *J* = 7.1 Hz, 6H) ppm.

^{13}**C NMR** (176 MHz, $CDCl_3$) δ 73.3, 42.9, 38.1, 27.7, 22.8, 14.2 ppm.

The data is in good agreement with the literature.[9]

(*anti*)-undecane-5,7-diol (*rac*-1d)

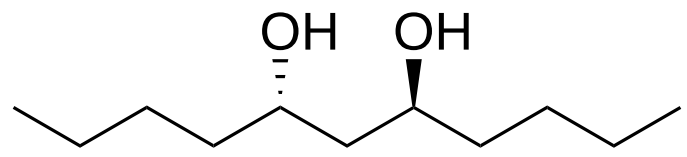

rac-**1d**

^{1}**H NMR** (700 MHz, $CDCl_3$) δ 3.94 (p, *J* = 5.9 Hz, 2H), 1.99 (s, 2H), 1.61 (t, *J* = 5.6 Hz, 2H), 1.57 – 1.51 (m, 2H), 1.50 – 1.43 (m, 2H), 1.43 – 1.26 (m, 8H), 0.91 (t, *J* = 7.1 Hz, 6H) ppm.

^{13}**C NMR** (176 MHz, $CDCl_3$) δ 69.7, 42.4, 37.4, 28.1, 22.9, 14.2 ppm.

The data is in good agreement with the literature.[9]

2,8-dimethylnonane-4,6-diol (1e)

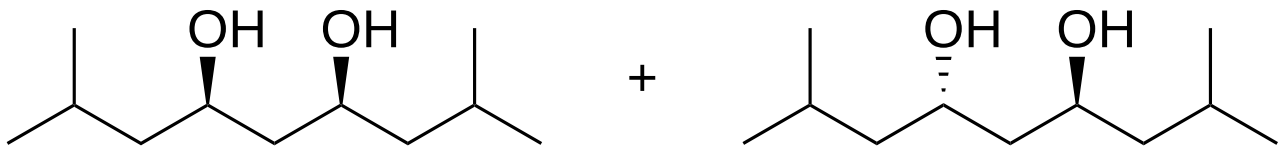

meso-**1e** *rac*-**1e**

Prepared according to General Procedure 1 using Isopropylmagnesium bromide solution (7.5 ml, 15 mmol, 2.0 M in THF), CuI (299 mg, 1.5 mmol, 30 mol%), di(oxiran-2-yl)methane (500 mg, 5.0 mmol) and THF. The crude product was purified via silica gel column chromatography (5% Acetone in Pet. Ether) to give (syn)-2,8-dimethylnonane-4,6-diol (176 mg, 0.93 mmol, 19%) as a colourless oil and (anti)-2,8-dimethylnonane-4,6-diol (215 mg, 1.14 mmol, 23%) as a white solid.

(*syn*)-2,8-dimethylnonane-4,6-diol (*meso*-1e)

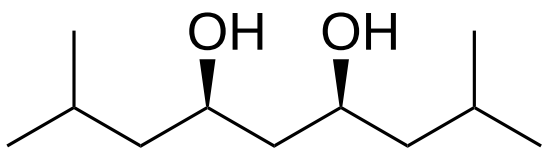

meso-**1e**

^{1}H NMR (700 MHz, $CDCl_3$) δ 3.97 – 3.92 (m, 2H), 2.93 (br s, 2H), 1.79 – 1.71 (m, 2H), 1.55 (dt, *J* = 14.6, 2.2 Hz, 1H), 1.46 – 1.41 (m, 3H), 1.21 (ddd, *J* = 13.6, 8.7, 4.8 Hz, 2H), 0.92 (app dd, *J* = 6.6, 2.8 Hz, 12H) ppm.

^{13}C NMR (176 MHz, $CDCl_3$) δ 71.4, 47.6, 44.2, 24.4, 23.5, 22.3 ppm.

The data is in good agreement with the literature.[6, 10]

(*anti*)-2,8-dimethylnonane-4,6-diol (*rac*-1e)

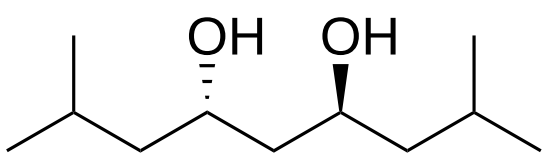

rac-**1e**

^{1}H NMR (700 MHz, $CDCl_3$) δ 4.07 – 4.02 (m, 2H), 2.19 (d, J = 2.7 Hz, 2H), 1.78 – 1.70 (m, 2H), 1.58 (dd, J = 5.0, 0.7 Hz, 2H), 1.49 (ddd, J = 13.9, 8.5, 5.7 Hz, 2H), 1.25 (ddd, J = 13.5, 8.5, 4.9 Hz, 2H), 0.93 (app dd, J = 6.6, 3.7 Hz, 12H) ppm.

^{13}C NMR (176 MHz, $CDCl_3$) δ 67.6, 46.8, 43.4, 24.8, 23.4, 22.4 ppm.

The data is in good agreement with the literature.[6, 10]

2,10-dimethylundecane-5,7-diol (1f)

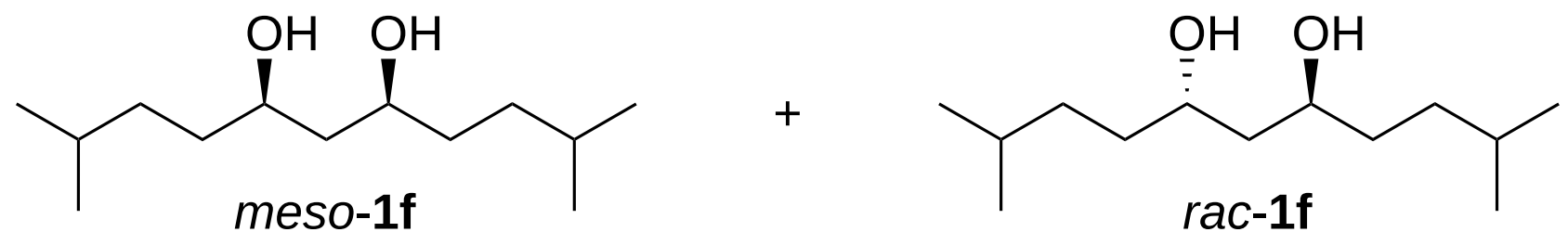

Prepared according to **General Procedure 1** using Isobutylmagnesium chloride solution (7.5 ml, 15 mmol, 2.0 M in THF), CuI (299 mg, 1.5 mmol, 30 mol%), di(oxiran-2-yl)methane (500 mg, 5.0 mmol) and THF. The crude product was purified *via* silica gel column chromatography (5 – 10% Acetone in Pet. Ether) to give (*syn*)-2,10-dimethylundecane-5,7-diol (238 mg, 1.10 mmol, 22%) as a colourless oil, (*anti*)-2,10-dimethylundecane-5,7-diol (179 mg, 0.83 mmol, 17%) as a white solid and mixed fractions (246 mg, 1.14 mmol, 23%).

(*syn*)-2,10-dimethylundecane-5,7-diol (*meso*-1f)

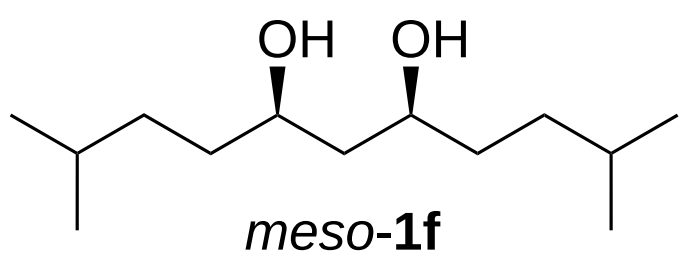

^{1}H NMR (700 MHz, $CDCl_3$) δ 3.85 – 3.79 (m, 2H), 2.91 (br s, 2H), 1.62 (dt, *J* = 14.5, 2.1 Hz, 1H), 1.54 (sep, *J* = 6.6 Hz, 2H), 1.51 – 1.42 (m, 5H), 1.33 – 1.26 (m, 2H), 1.22 – 1.15 (m, 2H), 0.89 (dd, *J* = 6.7, 3.3 Hz, 12H) ppm.

^{13}C NMR (176 MHz, $CDCl_3$) δ 73.7, 43.0, 36.3, 34.6, 28.2, 22.8, 22.7 ppm.

HRMS [M+Na]$^+$ m/z calc'd for $[C_{13}H_{28}O_2Na]^+$ expect 239.1987; found 239.1987

(*anti*)-2,10-dimethylundecane-5,7-diol (*rac*-1f)

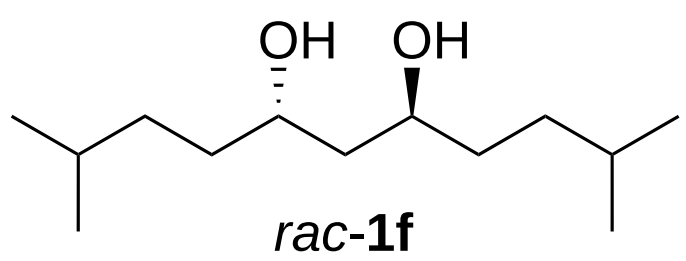

^{1}H NMR (700 MHz, $CDCl_3$) δ 3.94 – 3.88 (m, 2H), 2.28 (d, *J* = 3.8 Hz, 2H), 1.62 (t, *J* = 5.6 Hz, 2H), 1.59 – 1.50 (m, 4H), 1.46 (dddd, *J* = 13.4, 11.2, 5.5, 5.5 Hz, 2H), 1.31 (dddd, *J* = 13.1, 11.4, 6.6, 4.9 Hz, 2H), 1.18 (dddd, *J* = 12.9, 11.6, 6.6, 5.2 Hz, 2H), 0.90 (dd, *J* = 6.6, 2.5 Hz, 12H) ppm.

^{13}C NMR (176 MHz, $CDCl_3$) δ 70.0, 42.4, 35.5, 35.1, 28.2, 22.8, 22.7 ppm.

HRMS [M+Na]$^+$ m/z calc'd for $[C_{13}H_{28}O_2Na]^+$ expect 239.1987; found 239.1995

1,7-dicyclobutylheptane-3,5-diol (1g)

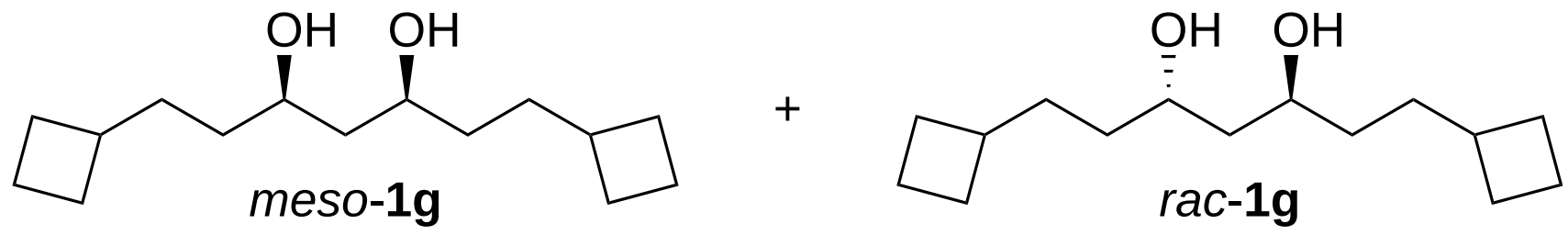

meso-**1g** + *rac*-**1g**

Grignard solution was prepared based upon a literature procedure.[11] 1,2-dibromoethane (17 μl, 0.2 mmol, 2 mol%) was added to magnesium turnings (547 mg, 22.5 mmol) in THF (3 ml) followed by dropwise addition of (Bromomethyl)cyclobutene (0.3 ml, 2.65 mmol). Then (Bromomethyl)cyclobutene (1.4 ml, 12.35 mmol) in THF (10 ml) was added dropwise and the resulting solution heated at reflux for 30 minutes.

Prepared according to **General Procedure 1** using cooled Grignard solution, CuI (299 mg, 1.5 mmol, 30 mol%), di(oxiran-2-yl)methane (500 mg, 5.0 mmol) and THF. The crude product was purified *via* column chromatography (5% Acetone in Pet. Ether) twice to give (*syn*)-1,7-dicyclobutylheptane-3,5-diol (289 mg, 1.20 mmol, 24%) as a white solid, (*anti*)-1,7-dicyclobutylheptane-3,5-diol (95 mg, 0.39 mmol, 8%) as a white solid and mixed fractions (52 mg, 0.22 mmol, 4%).

(*syn*)-1,7-dicyclobutylheptane-3,5-diol (*meso*-1g)

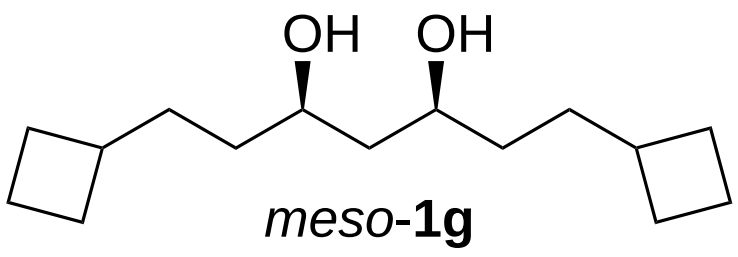

meso-**1g**

^{1}H NMR (700 MHz, $CDCl_3$) δ 3.84 – 3.80 (m, 2H), 2.35 (br s, 2H), 2.24 (sep, *J* = 7.6 Hz, 2H), 2.08 – 2.00 (m, 4H), 1.88 – 1.76 (m, 4H), 1.62 – 1.55 (m, 5H), 1.51 – 1.30 (m, 9H) ppm.

^{13}C NMR (176 MHz, $CDCl_3$) δ 73.4, 43.0, 36.1, 35.9, 32.7, 28.4, 28.4, 18.5 ppm.

HRMS [M+Na]$^+$ m/z calc'd for $[C_{15}H_{28}O_2Na]^+$ expect 263.1987; found 263.1999

(*anti*)-1,7-dicyclobutylheptane-3,5-diol (*rac*-1g)

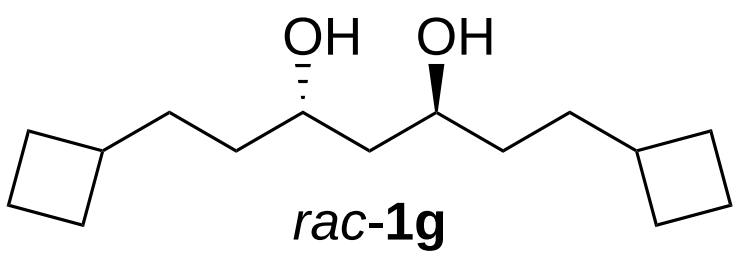

rac-**1g**

^{1}H NMR (700 MHz, $CDCl_3$) δ 3.93 – 3.88 (m, 2H), 2.25 (sep, *J* = 7.7 Hz, 2H), 2.08 – 2.00 (m, 4H), 1.96 (br s, 2H), 1.89 – 1.76 (m, 4H), 1.62 – 1.55 (m, 6H), 1.53 – 1.32 (m, 8H) ppm.

^{13}C NMR (176 MHz, $CDCl_3$) δ 69.6, 42.4, 36.1, 35.2, 33.2, 28.4, 28.4, 18.5 ppm.

HRMS [M+Na]$^+$ m/z calc'd for $[C_{15}H_{28}O_2Na]^+$ expect 263.1987; found 263.1985

1,5-dicyclohexylpentane-2,4-diol (1h)

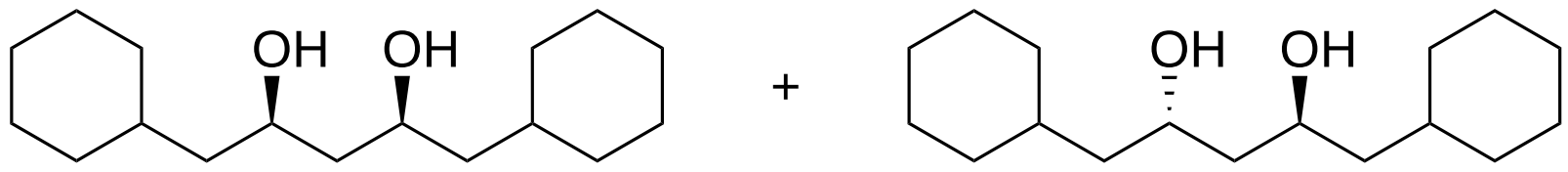

meso-**1h** *rac*-**1h**

Prepared according to **General Procedure 1** using cyclohexylmagnesium chloride solution (7.5 ml, 15 mmol, 2.0 M in Et_2O), CuI (299 mg, 1.5 mmol, 30 mol%), di(oxiran-2-yl)methane (500 mg, 5.0 mmol) and Et_2O. The crude product was purified *via* column chromatography (5% Acetone in Pet. Ether) to give (*syn*)-1,5-dicyclohexylpentane-2,4-diol (309 mg, 1.15 mmol, 23%) as a white solid, (*anti*)-1,5-dicyclohexylpentane-2,4-diol (107 mg, 0.40 mmol, 8%) as a white solid and mixed fractions (94 mg, 0.35 mmol, 7%).

(*syn*)-1,5-dicyclohexylpentane-2,4-diol (*meso*-1h)

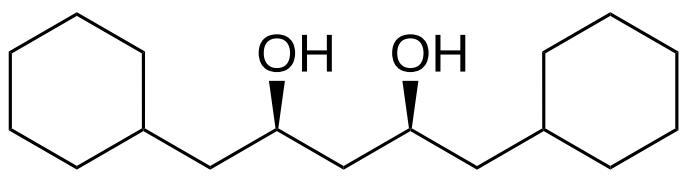

meso-**1h**

^{1}H NMR (700 MHz, $CDCl_3$) δ 4.00 – 3.95 (m, 2H), 2.83 (s, 2H), 1.80 (br d, *J* = 11.2 Hz, 2H), 1.72 – 1.62 (m, 8H), 1.54 (dt, *J* = 14.6, 2.1 Hz, 1H), 1.47 – 1.38 (m, 5H), 1.29 – 1.21 (m, 6H), 1.15 (qt, *J* = 12.5, 3.3 Hz, 2H), 0.98 – 0.90 (m, 2H), 0.90 – 0.83 (m, 2H) ppm.

^{13}C NMR (176 MHz, $CDCl_3$) δ 70.6, 46.3, 44.4, 34.3, 33.9, 33.1, 26.7, 26.5, 26.3 ppm.

HRMS [M+Na]$^+$ m/z calc'd for $[C_{17}H_{32}O_2Na]^+$ expect 291.2300; found 291.2298

(*anti*)-1,5-dicyclohexylpentane-2,4-diol (*rac*-1h)

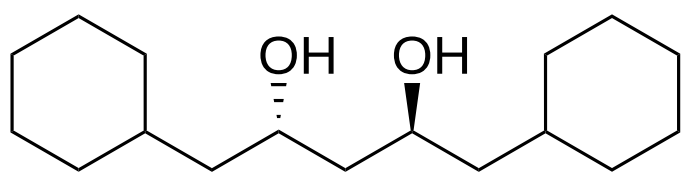

rac-**1h**

^{1}H NMR (700 MHz, $CDCl_3$) δ 4.10 – 4.05 (m, 2H), 2.19 (br s, 2H), 1.79 (br d, *J* = 12.8 Hz, 2H), 1.72 – 1.62 (m, 8H), 1.57 (t, *J* = 5.6 Hz, 2H), 1.50 – 1.38 (m, 4H), 1.29 – 1.20 (m, 6H), 1.15 (qt, *J* = 12.5, 3.3 Hz, 2H), 0.99 – 0.91 (m, 2H), 0.88 (qd, *J* = 12.1, 3.5 Hz, 2H) ppm.

^{13}C NMR (176 MHz, $CDCl_3$) δ 67.0, 45.5, 43.6, 34.3, 34.2, 33.2, 26.7, 26.5, 26.4 ppm.

HRMS [M+Na]$^+$ m/z calc'd for $[C_{17}H_{32}O_2Na]^+$ expect 291.2300; found 291.2306

1,7-dicyclohexylheptane-3,5-diol (1i)

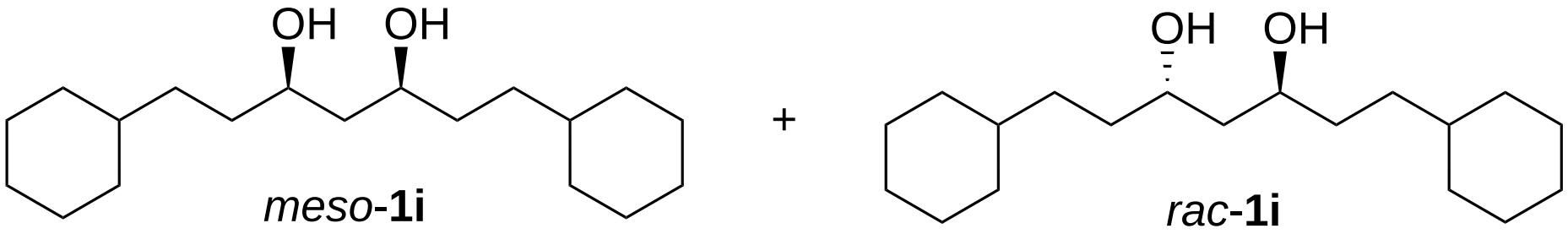

meso-**1i** *rac*-**1i**

Grignard solution was prepared based upon a literature procedure.[12] Magnesium chips (935 mg, 38.4 mmol) and bromomethylcyclohexane (0.3 ml, 2.1 mmol) in THF (5.6 ml) was heated to 60 °C. Following this a solution of bromomethylcyclohexane (5.1 ml, 36.3 mmol) in THF (38.8 ml) was added dropwise and the reaction mixture was maintained at 60 °C for 4 h before being cooled.

Prepared according to **General Procedure 1** using the cooled Grignard reagent, CuI (767mg, 3.8 mmol, 30 mol%), di(oxiran-2-yl)methane (1.29 g, 12.8 mmol) and THF. The crude product was purified *via* silica gel column chromatography (5% Acetone in Pet. Ether) to give (*syn*)-1,7-dicyclohexylheptane-3,5-diol (26.6 mg, 0.90 mmol, 7%) as a white solid, (*anti*)-1,7-dicyclohexylheptane-3,5-diol as a white solid (657 mg, 2.19 mmol, 17%) and mixed fractions (1.89 g, 6.36 mmol, 50%).

(*syn*)-1,7-dicyclohexylheptane-3,5-diol (*meso*-1i)

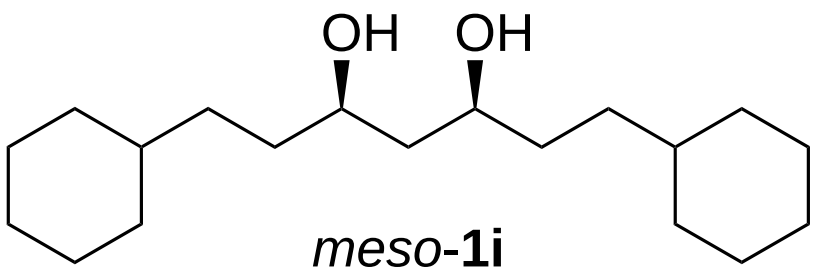

meso-**1i**

^{1}H NMR (700 MHz, $CDCl_3$) δ 3.82 – 3.77 (m, 2H), 3.24 (s, 2H), 1.72 – 1.65 (m, 8H), 1.63 (br d, *J* = 12.4 Hz, 2H), 1.59 (dt, *J* = 14.5, 2.0 Hz , 1H), 1.50 – 1.39 (m, 5H), 1.31 – 1.24 (m, 2H), 1.24 – 1.09 (m, 10H), 0.91 – 0.83 (m, 4H) ppm.

^{13}C NMR (176 MHz, $CDCl_3$) δ 73.7, 42.9, 37.8, 35.7, 33.6, 33.5, 33.2, 26.8, 26.5, 26.5 ppm.

HRMS $[M+Na]^+$ m/z calc'd for $[C_{19}H_{36}O_2Na]^+$ expect 319.2613; found 319.2608.

(*anti*)-1,7-dicyclohexylheptane-3,5-diol (*rac*-1i)

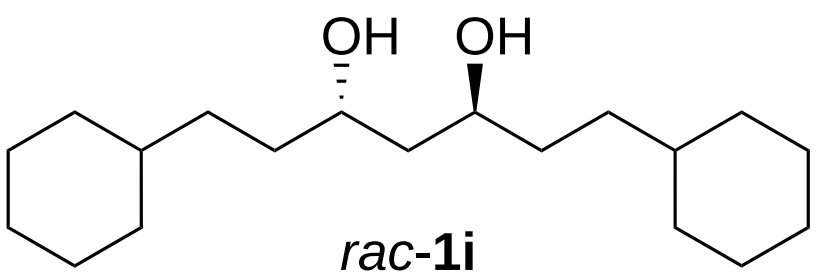

rac-**1i**

^{1}H NMR (700 MHz, $CDCl_3$) δ 3.89 (p, *J* = 5.9 Hz, 2H) 2.27 (br s, 2H), 1.69 (br t, *J* = 13.0 Hz, 8H), 1.64 (br d, *J* = 13.6 Hz, 2H), 1.60 (t, *J* = 5.5 Hz , 2H, 1.56 – 1.49 (m, 2H), 1.49 – 1.43 (m, 2H), 1.34 – 1.27 (m, 2H), 1.25 – 1.10 (m, 10H), 0.92 – 0.84 (m, 4H) ppm.

^{13}C NMR (176 MHz, $CDCl_3$) δ 70.0, 42.3, 37.9, 34.9, 33.6, 33.6, 33.5, 26.8, 26.5 ppm.

HRMS $[M+Na]^+$ m/z calc'd for $[C_{19}H_{36}O_2Na]^+$ expect 319.2613; found 319.2603.

1,9-dicyclohexylnonane-4,6-diol (1j)

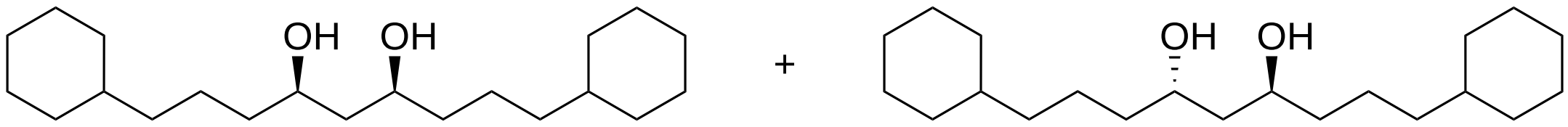

meso-**1j** *rac*-**1j**

Grignard solution was prepared based upon a literature procedure.[12] Magnesium chips (365 mg, 15.0 mmol) and 1-bromo-2-cyclohexylethane (0.1 ml, 0.8 mmol) in THF (2.3 ml) was heated to 60 °C. Following this a solution of 1-bromo-2-cyclohexylethane (2.8 ml, 14.2 mmol) in THF (15.2 ml) was added dropwise and the reaction mixture was maintained at 60 °C for 4 h before being cooled.

Prepared according to **General Procedure 1** using the cooled Grignard reagent, CuI (299 mg, 1.5 mmol, 30 mol%), di(oxiran-2-yl)methane (500 mg, 5.0 mmol) and THF. The crude product was purified *via* silica gel column chromatography (10% Acetone in Pet. Ether) to give (*syn*)- 1,9-dicyclohexylnonane-4,6-diol (162 mg, 0.50 mmol, 10%) as a white solid, (*anti*)- 1,9-dicyclohexylnonane-4,6-diol (119 mg, 0.37 mmol, 7%) as a white solid and mixed fractions (564 mg, 1.74 mmol, 35%).

(*syn*)-1,9-dicyclohexylnonane-4,6-diol (*meso*-1j)

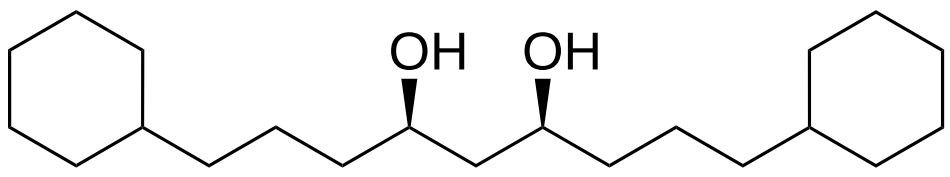

meso-**1j**

^{1}H NMR (700 MHz, $CDCl_3$) δ 3.87 – 3.82 (m, 2H), 2.67 (s, 2H), 1.72 – 1.65 (m, 8H), 1.63 (br d, *J* = 12.3 Hz, 2H), 1.60 (dt, *J* = 14.5, 2.0 Hz, 1H), 1.49 – 1.37 (m, 7H), 1.35 – 1.28 (m, 2H), 1.24 – 1.09 (m, 12H), 0.90 – 0.82 (m, 4H) ppm.

^{13}C NMR (176 MHz, $CDCl_3$) δ 73.4, 43.1, 38.7, 37.8, 37.6, 33.5, 33.5, 26.9, 26.6, 22.7 ppm.

HRMS [M+Na]$^+$ m/z calc'd for $[C_{21}H_{40}O_2Na]^+$ expect 347.2927; found 347.2914

(*anti*)-1,9-dicyclohexylnonane-4,6-diol (*rac*-1j)

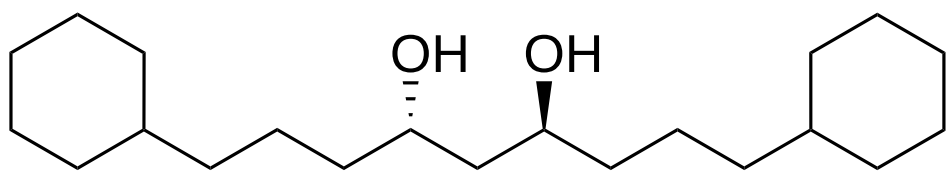

rac-**1j**

^{1}H NMR (700 MHz, $CDCl_3$) δ 3.96 – 3.91 (m, 2H), 2.15 (s, 2H), 1.72 – 1.65 (m, 8H), 1.63 (br d, *J* = 12.5 Hz, 2H), 1.60 (t, *J* = 5.6 Hz, 1H), 1.54 – 1.47 (m, 2H), 1.46 – 1.38 (m, 4H), 1.34 – 1.26 (m, 2H), 1.25 – 1.09 (m, 12H), 0.90 – 0.82 (m, 4H) ppm.

^{13}C NMR (176 MHz, $CDCl_3$) δ 69.6, 42.4, 37.9, 37.7, 37.6, 33.5, 26.9, 26.6, 23.2 ppm.

HRMS [M+Na]$^+$ m/z calc'd for $[C_{21}H_{40}O_2Na]^+$ expect 347.2927; found 347.2923

1,7-diphenylheptane-3,5-diol (1k)

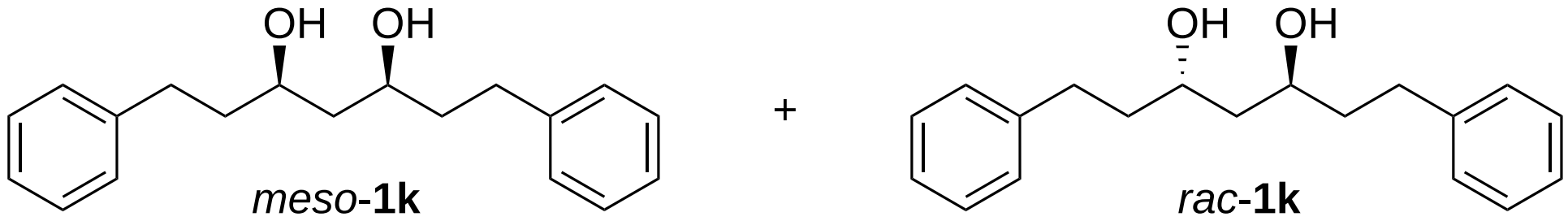

meso-**1k** + *rac*-**1k**

Prepared according to **General Procedure 1** using Benzylmagnesium chloride solution (10.7 ml, 15 mmol, 1.4 M in THF), CuI (299 mg, 1.5 mmol, 30 mol%), di(oxiran-2-yl)methane (500 mg, 5.0 mmol) and THF. The crude product was purified *via* silica gel column chromatography (10 – 15% Acetone in Pet. Ether) to give (*syn*)-1,7-diphenylheptane-3,5-diol (325 mg, 1.14 mmol, 23%) as a white solid, (*anti*)-1,7-diphenylheptane-3,5-diol (319 mg, 1.14 mmol, 23%) as a white solid and mixed fractions (605 mg, 2.13 mmol, 43%).

(*syn*)-1,7-diphenylheptane-3,5-diol (*meso*-1k)

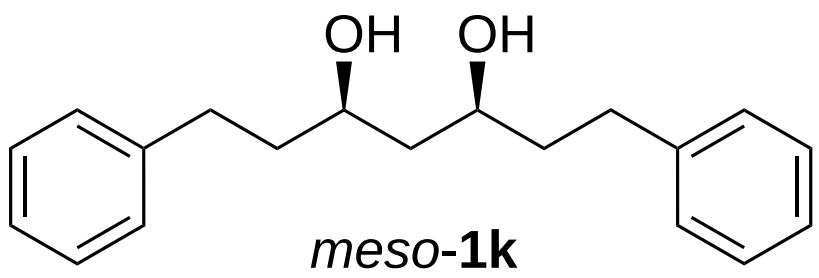

meso-**1k**

^{1}H NMR (700 MHz, $CDCl_3$) δ 7.29 (app t, *J* = 7.7 Hz, 4H), 7.22 – 7.18 (m, 6H), 3.91 – 3.86 (m, 2H), 2.95 (br s, 2H), 2.76 (ddd, *J* = 13.8, 9.6, 5.8 Hz, 2H), 2.68 (ddd, *J* = 13.8, 9.4, 6.7 Hz, 2H), 1.85 – 1.74 (m, 4H), 1.64 (dt, *J* = 14.5, 2.4 Hz, 1H), 1.58 (dt, *J* = 14.5, 9.9 Hz, 1H) ppm.

^{13}C NMR (176 MHz, $CDCl_3$) δ 142.0, 128.6, 128.5, 126.1, 72.5, 43.2, 39.9, 31.8 ppm.

HRMS [M+Na]$^+$ m/z calc'd for $[C_{19}H_{24}O_2Na]^+$ expect 307.1674; found 307.1674

(*anti*)- 1,7-diphenylheptane-3,5-diol (*rac*-1k)

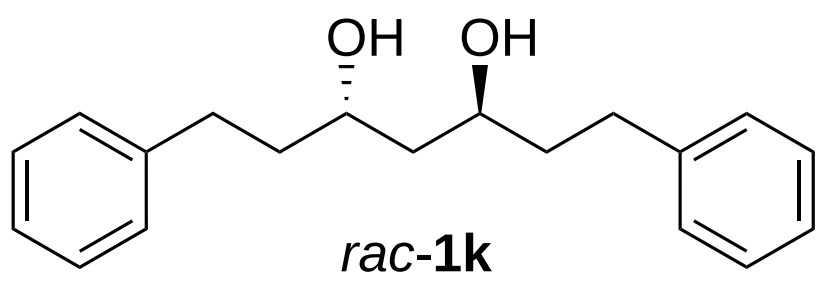

rac-**1k**

^{1}H NMR (700 MHz, $CDCl_3$) δ 7.29 (app t, *J* = 7.6 Hz, 4H), 7.22 – 7.18 (m, 6H), 4.02 – 3.97 (m, 2H), 2.79 (ddd, *J* = 13.9, 9.8, 5.6 Hz, 2H), 2.67 (ddd, *J* = 13.8, 9.6, 6.4 Hz, 2H), 2.34 (br s, 2H), 1.87 (dddd, *J* = 13.8, 9.7, 8.2, 5.6 Hz, 2H), 1.77 (dddd, *J* = 13.8, 9.9, 6.4, 4.4 Hz, 2H), 1.68 (t, *J* = 5.6 Hz, 2H) ppm.

^{13}C NMR (176 MHz, $CDCl_3$) δ 142.0, 128.6, 128.5, 126.0, 69.1, 42.7, 39.2, 32.3 ppm.

The data is in good agreement with the literature.[13]

1,9-diphenylnonane-4,6-diol (**1l**)

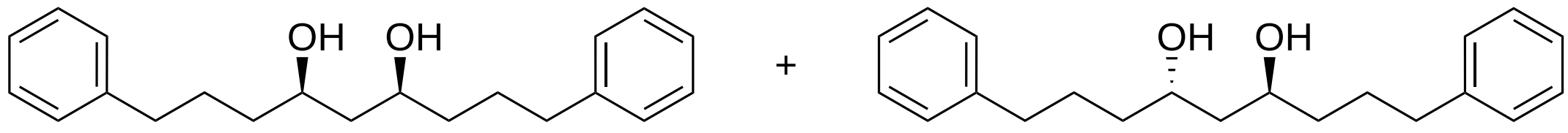

meso-**1l** *rac*-**1l**

Prepared according to literature procedure.[14] A solution of 1-phenyl-2-bromoethane (2.1 ml, 15.0 mmol) in Et_2O (2 ml) was made and then 0.75 ml of this was added dropwise to a suspension of magnesium (437 mg, 18 mmol) in Et_2O (10 ml) in a microwave vial at room temperature. The mixture was then heated to 50 °C for 10 minutes before being cooled back to room temperature. The rest of the 1-phenyl-2-bromoethane solution was then added dropwise to the reaction mixture which was heated at 50 °C for 2 hours.

Prepared according to **General Procedure 1** using the cooled Grignard reagent, CuI (120 mg, 0.6 mmol, 30 mol%), di(oxiran-2-yl)methane (214 mg, 2.1 mmol) and Et_2O. The crude product was purified *via* column chromatography (10 – 15% Acetone in Pet. Ether) twice to give (*syn*)-1,9-diphenylnonane-4,6-diol (50 mg, 0.16 mmol, 5%) as a white solid, (*anti*)-1,9-diphenylnonane-4,6-diol (91 mg, 0.29 mmol, 14%) as a white solid and mixed fractions (291 mg, 0.93 mmol, 44%).

(*syn*)-1,9-diphenylnonane-4,6-diol (*meso*-1l)

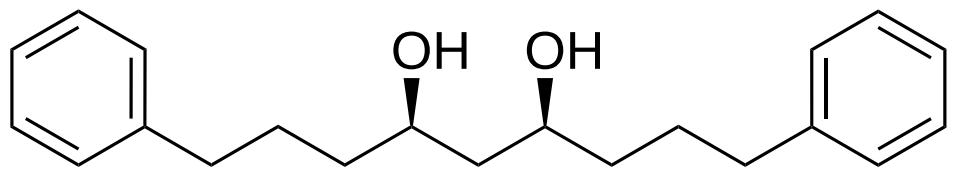

meso-**1l**

^{1}H NMR (700 MHz, $CDCl_3$) δ 7.29 – 7.25 (m, 4H), 7.20 – 7.16 (m, 6H), 3.89 – 3.83 (m, 2H), 2.63 (t, *J* = 7.5 Hz, 4H), 1.79 – 1.71 (m, 2H), 1.69 – 1.61 (m, 2H), 1.57 (d, *J* = 14.5 Hz, 1H), 1.55 – 1.42 (m, 5H) ppm.

^{13}C NMR (176 MHz, $CDCl_3$) δ 142.4, 128.5, 128.5, 125.9, 73.2, 43.0, 37.9, 35.9, 27.3 ppm.

HRMS [M+Na]$^+$ m/z calc'd for $[C_{21}H_{28}O_2Na]^+$ expect 335.1987; found 335.1989

(*anti*)-1,9-diphenylnonane-4,6-diol (*rac*-1l)

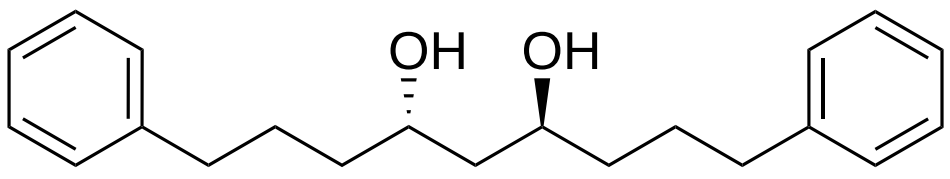

rac-**1l**

^{1}H NMR (700 MHz, $CDCl_3$) δ 7.28 (app t, *J* = 7.5 Hz, 4H), 7.19 (app t, *J* = 8.1 Hz, 6H), 3.96 – 3.90 (m, 2H), 2.64 (t, *J* = 7.6 Hz, 6H), 1.81 – 1.73 (m, 2H), 1.68 – 1.60 (m, 2H), 1.60 – 1.53 (m, 4H), 1.51 – 1.44 (m, 2H) ppm.

^{13}C NMR (176 MHz, $CDCl_3$) δ 142.4, 128.5, 128.4, 125.9, 69.3, 42.4, 37.1, 35.9, 27.7 ppm.

HRMS [M+Na]$^+$ m/z calc'd for $[C_{21}H_{28}O_2Na]^+$ expect 335.1987; found 335.1983

4-Hydroxy-5-phenylpentan-2-one (*int₁*-3a)

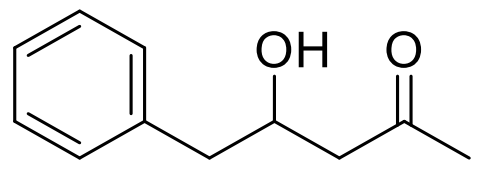

This procedure was adapted from literature.[15] In a vial, a mixture of KOH (5 M in MeOH, 2 mL, 561 mg, 10 mmol, 1.25 equiv.) and acetone (2 mL) was cooled to 0 °C and a solution of phenylacetaldehyde (961 mg, 8.0 mmol, 1.0 equiv.) in 1 mL acetone was added dropwise. The mixture was stirred vigorously at 0 °C for 2 h upon which NH_4Cl (sat. aq., 1 mL) was added, the mixture transferred to a separatory funnel and water (20 mL) added. The mixture was extracted with DCM (3 x 20 mL). The combined organic fractions were dried over $MgSO_4$ and the solvent removed under reduced pressure. The crude material was purified by flash column chromatography silica gel (15 – 20% acetone in hexane) to give the desired product (520 mg, 37%) as a colourless oil.

^{1}H NMR (500 MHz, $CDCl_3$) δ 7.34 – 7.29 (m, 2H), 7.26 – 7.19 (m, 3H), 4.33 – 4.26 (m, 1H), 2.92 (br. s, 1 H), 2.85 (dd, *J* = 13.5, 7.1 Hz, 1H), 2.73, (dd, *J* = 13.5, 6.3 Hz, 1H), 2.65 – 2.53 (m, 2H), 2.15 (s, 3H) ppm.

^{13}C NMR (126 MHz, $CDCl_3$) δ 209.6, 138.0, 129.6, 128.7, 126.8, 68.8, 49.1, 43.0, 30.9 ppm.

The spectral data are in agreement with the literature.[15]

1-Phenylpentane-2,4-diol (3a)

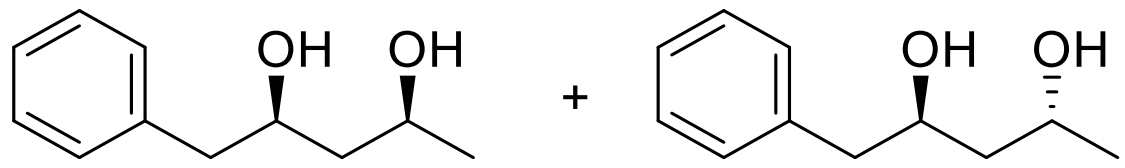

The compound was synthesised according to **General Procedure 3** using 4-hydroxy-5-phenylpentan-2-one (356 mg, 2.0 mmol), separated with 15 – 20% acetone in hexane, giving (*syn*)-1-Phenylpentane-2,4-diol (17.1 mg, 5%) as a colourless oil and (*anti*)-1-Phenylpentane-2,4-diol (143.6 mg, 40%) as a colourless solid.

(*anti*)-1-Phenylpentane-2,4-diol (*rac-anti*-3a)

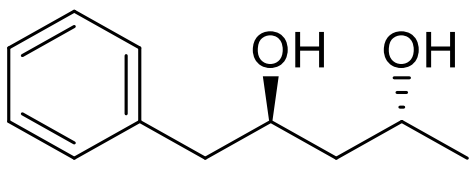

^{1}H NMR (500 MHz, $CDCl_3$) δ 7.34 – 7.29 (m, 2H), 7.26 – 7.20 (m, 3H), 4.23 – 4.14 (m, 2H), 2.84 – 2.74 (m, 2H), 2.12 (br. s, 2H), 1.70 – 1.64 (m, 2H), 1.23 (d, *J* = 6.3 Hz, 3H) ppm.

^{13}C NMR (126 MHz, $CDCl_3$) δ 138.4, 129.5, 128.8, 126.7, 70.3, 65.5, 44.2, 43.7, 23.7 ppm.

HRMS $[M+H]^+$ m/z calc'd for $[C_{11}H_{16}O_2Na]^+$ expect 203.1048; found 203.1057.

(*syn*)-1-Phenylpentane-2,4-diol (*rac-syn*-3a)

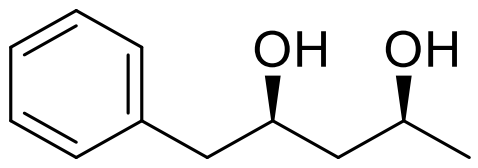

^{1}H NMR (700 MHz, $CDCl_3$) δ 7.34 – 7.30 (m, 2H), 7.26 – 7.23 (m, 1H), 7.21, (d *J* = 7.6 Hz, 2H), 4.10 – 4.06 (m, 1H), 4.06 – 4.01 (m, 1H), 2.80 – 2.73 (m, 2H), 2.51 (br. s, 2H), 1.67 – 1.63 (m, 1H), 1.59 – 1.53 (m, 1H), 1.19 (d, *J* = 6.2 Hz, 3H) ppm.

^{13}C NMR (176 MHz, $CDCl_3$) δ 138.0, 129.6, 128.8, 126.8, 74.0, 69.1, 44.8, 44.2, 24.2 ppm.

HRMS $[M+H]^+$ m/z calc'd for $[C_{11}H_{16}O_2Na]^+$ expect 203.1048; found 203.1059.

4-Hydroxy-5-(4-methoxyphenyl)pentan-2-one (*int₁*-3b)

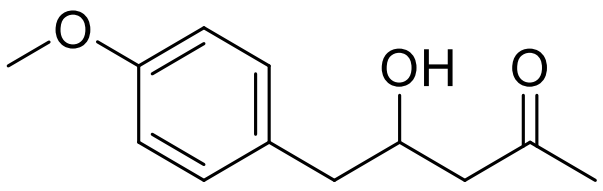

This procedure was adapted from literature.[16] 1,1,1-Triacetoxy-1,1-dihydro-1,2-benziodoxol-3(1H)-one (5.39 g, 12.7 mmol, 1.1 equiv.) was suspended in DCM (45 mL) and 4-Methoxyphenethyl alcohol (1.77 g, 11.6 mmol, 1.0 equiv.) was added as a solution in DCM (35 mL). The mixture was stirred at room temperature for 3 h upon which it was diluted with $NaHCO_3$ (sat. aq., 20 mL) and Et_2O (50 mL) and further stirred for 10 min. Then $Na_2S_2O_3$ (sat. aq., 5 mL) was added and the layers separated. The mixture was extracted with Et_2O (2 x 50 mL) and the combined organic layers dried over $MgSO_4$. The solvent was removed under reduced pressure and the crude aldehyde used directly without further purification.

This procedure was adapted from literature.[15] In a vial, a mixture of KOH (5 M in MeOH, 2.4 mL, 673 mg, 12 mmol) and acetone (2.4 mL) was cooled to 0 °C and a solution of the crude aldehyde in 1 mL acetone was added dropwise. The mixture was stirred vigorously at 0 °C for 2 h upon which NH_4Cl (sat. aq., 1.5 mL) was added, the mixture transferred to a separatory funnel and water (20 mL) added. The mixture was extracted with DCM (3 x 20 mL). The combined organic fractions were dried over $MgSO_4$ and the solvent removed under reduced pressure. The crude material was purified by flash column chromatography silica gel (30% EtOAc in hexane) to give the desired product (776 mg, 32% over two steps) as an orange oil.

^{1}H NMR (500 MHz, $CDCl_3$) δ 7.13 (d, *J* = 8.6 Hz, 2H), 6.85 (d, *J* = 8.6 Hz, 2H), 4.89 – 4.20 (m, 1H), 3.79 (s, 3H), 2.89 (br. s, 1 H), 2.78 (dd, *J* = 13.7, 7.1 Hz, 1H), 2.67, (dd, *J* = 13.7, 6.3 Hz, 1H), 2.63 – 2.50 (m, 2H), 2.15 (s, 3H) ppm.

^{13}C NMR (126 MHz, $CDCl_3$) δ 209.7, 158.5, 130.5, 129.9, 114.1, 68.9, 55.4, 49.1, 42.0, 30.9 ppm.

HRMS $[M+H]^+$ m/z calc'd for $[C_{12}H_{17}O_3]^+$ expect 209.1178; found 209.1170.

1-(4-Methoxyphenyl)pentane-2,4-diol (3b)

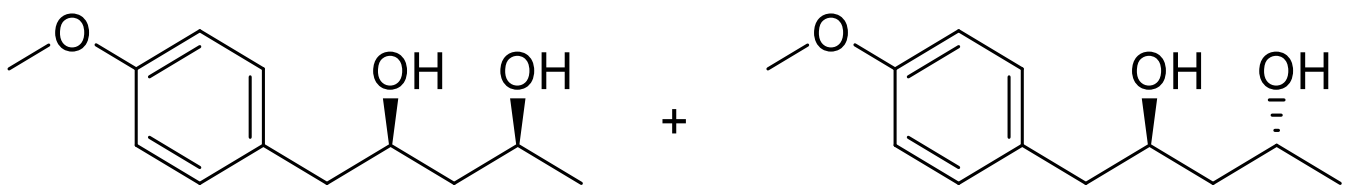

The compound was synthesised according to **General Procedure 3** reduction using 4-hydroxy-5-(4-methoxyphenyl)pentan-2-one (416 mg, 2.0 mmol), separated with 20% acetone in hexane, giving (*syn*)-1-(4-Methoxyphenyl)pentane-2,4-diol (19.9 mg, 5%) as a colourless oil and (*anti*)-1-(4-Methoxyphenyl)pentane-2,4-diol (295.8 mg, 70%) as a colourless solid.

(*anti*)-1-(4-Methoxyphenyl)pentane-2,4-diol (*rac-anti*-3b)

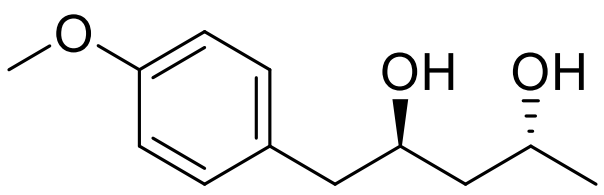

^{1}H NMR (500 MHz, $CDCl_3$) δ 7.15 – 7.11 (m, 2H), 6.88 – 6.84 (m, 2H), 4.22 – 4.10 (m, 2H), 3.79 (s, 3H), 2.78 – 2.68 (m, 2H), 2.19 (br. s, 2H), 1.69 – 1.62 (m, 2H), 1.23 (d, *J* = 6.3 Hz, 3H) ppm.

^{13}C NMR (126 MHz, $CDCl_3$) δ 158.5, 130.5, 130.3, 114.2, 70.4, 65.6, 55.4, 43.6, 43.2, 23.7 ppm.

HRMS [M+H]$^+$ m/z calc'd for $[C_{12}H_{19}O_3]^+$ expect 211.1334; found 211.1337.

(*syn*)-1-(4-Methoxyphenyl)pentane-2,4-diol (*rac-syn*-3b)

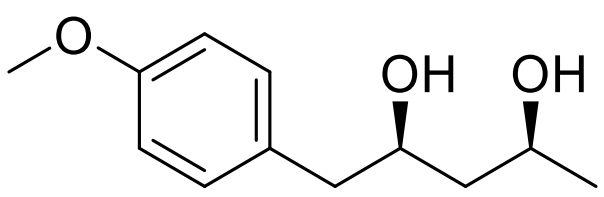

^{1}H NMR (500 MHz, $CDCl_3$) δ 7.14 – 7.10 (m, 2H), 6.87 – 6.84 (m, 2H), 4.06 – 3.98 (m, 2H), 3.79 (s, 3H), 2.76 – 2.65 (m, 2H), 2.72 (br. s, 2H), 1.66 – 1.61 (m, 1H), 1.57 – 1.49 (m, 1H), 1.18 (d, *J* = 6.2 Hz, 3H) ppm.

^{13}C NMR (126 MHz, $CDCl_3$) δ 158.5, 130.5, 129.9, 114.2, 74.1, 69.0, 55.4, 44.2, 43.9, 24.2 ppm.

HRMS [M+H]$^+$ m/z calc'd for $[C_{12}H_{19}O_3]^+$ expect 211.1334; found 211.1339.

4-Hydroxy-5-(naphthalen-1-yl)pentan-2-one (*int$_1$*-3c)

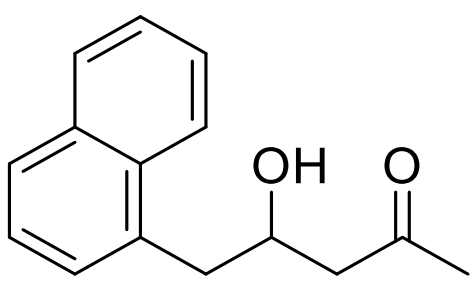

This procedure was adapted from literature.[16] 1,1,1-Triacetoxy-1,1-dihydro-1,2-benziodoxol-3(1H)-one (5.39 g, 12.7 mmol, 1.1 equiv.) was suspended in DCM (45 mL) and 2-(Naphthalen-1-yl)ethan-1-ol (2.0 g, 11.6 mmol, 1.0 equiv.) was added as a solution in DCM (35 mL). The mixture was stirred at room temperature for 3 h upon which it was diluted with $NaHCO_3$ (sat. aq., 20 mL) and Et_2O (50 mL) and further stirred for 10 min. Then $Na_2S_2O_3$ (sat. aq., 5 mL) was added and the layers separated. The

mixture was extracted with Et_2O (2 x 50 mL) and the combined organic layers dried over $MgSO_4$. The solvent was removed under reduced pressure and the crude aldehyde used directly without further purification.

This procedure was adapted from literature.[15] In a vial, a mixture of KOH (5 M in MeOH, 2.4 mL, 673 mg, 12 mmol) and acetone (2.4 mL) was cooled to 0 °C and a solution of the crude aldehyde in 1 mL acetone was added dropwise. The mixture was stirred vigorously at 0 °C for 2 h upon which NH_4Cl (sat. aq., 1 mL) was added, the mixture transferred to a separatory funnel and water (20 mL) added. The mixture was extracted with DCM (3 x 20 mL). The combined organic fractions were dried over $MgSO_4$ and the solvent removed under reduced pressure. The crude material was purified by flash column chromatography silica gel (20 – 40% EtOAc in hexane) to give the desired product (861 mg, 33% over two steps) as an orange oil.

^{1}H NMR (500 MHz, $CDCl_3$) δ 8.10 (d, *J* = 8.3 Hz, 1H), 7.87 (d, *J* = 8.1 Hz, 1H), 7.77 (d, *J* = 8.1 Hz, 1H), 7.56 – 7.47 (m, 2H) 7.42, (dd, *J* = 8.1, 7.0 Hz, 1H) 7.36, (d, *J* = 7.0 Hz, 1H), 4.51 – 4.43 (m, 1H), 3.32 (dd, *J* = 13.8, 7.0 Hz, 1H), 3.22, (dd, *J* = 13.8, 6.7 Hz, 1H), 2.96 (br. s, 1H), 2.67 – 2.62 (m, 2H), 2.13 (s, 3H) ppm.

^{13}C NMR (126 MHz, $CDCl_3$) δ 209.6, 134.12, 134.10, 132.3, 129.0, 127.8, 127.7, 126.3, 125.9, 125.6, 124.0, 68.1, 49.4, 40.1, 30.9 ppm.

HRMS [M+H]$^+$ m/z calc'd for $[C_{15}H_{17}O_2]^+$ expect 229.1229; found 229.1231.

1-(Naphthalen-1-yl)pentane-2,4-diol (3c)

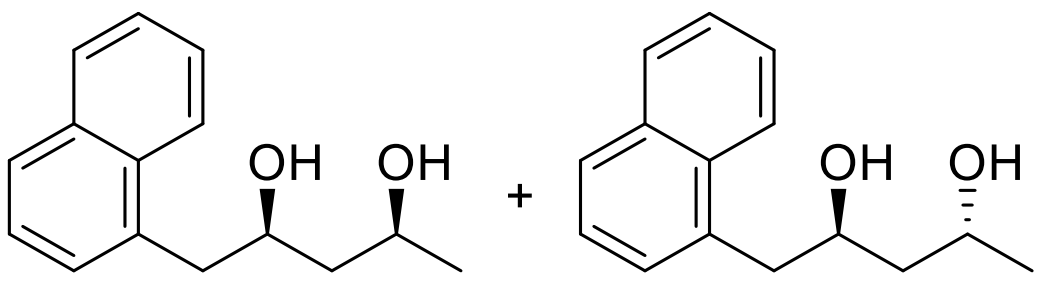

The compound was synthesised according to **General Procedure 3** using 4-hydroxy-5-(naphthalen-1-yl)pentan-2-one (457 mg, 2.0 mmol), separated with 15 – 20% acetone in hexane, giving (*syn*)-1-(Naphthalen-1-yl)pentane-2,4-diol (14.6 mg, 3%) as a colourless oil and (*anti*)-1-(Naphthalen-1-yl)pentane-2,4-diol (189.6 mg, 41%) as a colourless solid.

(*anti*)-1-(Naphthalen-1-yl)pentane-2,4-diol (*rac-anti*-3c)

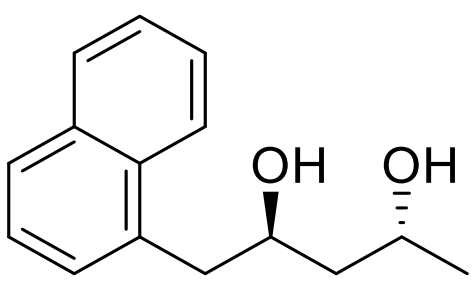

^{1}H NMR (500 MHz, $CDCl_3$) δ 8.05 (d, *J* = 8.3 Hz, 1H), 7.89 – 7.85 (m, 1H), 7.77 (d, *J* = 8.1 Hz, 1H), 7.55 – 7.47 (m, 2H), 7.44 – 7.40 (m, 1H), 7.37 (d, *J* = 7.0 Hz, 1H), 4.37 – 4.31 (m, 1H), 4.28 – 4.20 (m, 1H), 3.33 (dd, *J* = 13.9, 4.6 Hz, 1H), 3.20 (dd, *J* = 13.9, 8.6 Hz, 1H), 2.13 (br. s, 2H), 1.84 – 1.72 (m, 2H), 1.24 (d, *J* = 6.3 Hz, 3H) ppm.

^{13}C NMR (126 MHz, $CDCl_3$) δ 134.5, 134.2, 132.3, 127.8, 127.7, 126.3, 125.9, 125.6, 123.9, 69.6, 65.6, 44.2, 41.2, 23.7 ppm.

HRMS [M+H]$^+$ m/z calc'd for $[C_{15}H_{18}O_2Na]^+$ expect 253.1204; found 253.1210.

(*syn*)-1-(Naphthalen-1-yl)pentane-2,4-diol (*rac-syn*-3c)

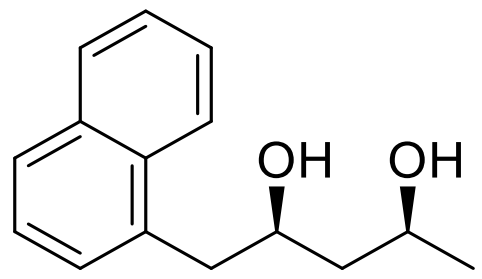

^{1}H NMR (500 MHz, $CDCl_3$) δ 8.05 (d, *J* = 8.3 Hz, 1H), 7.89 – 7.85 (m, 1H), 7.77 (d, *J* = 8.1 Hz, 1H), 7.56 – 7.47 (m, 2H), 7.45 – 7.40 (m, 1H), 7.35 (d, *J* = 6.9 Hz, 1H), 4.28 – 4.22 (m, 1H), 4.05 – 3.97 (m, 1H), 3.29 – 3.17 (m, 2H), 2.60 (br. s, 2H), 1.76 – 1.63 (m, 2H), 1.19 (d, *J* = 6.2 Hz, 3H) ppm.

^{13}C NMR (126 MHz, $CDCl_3$) δ 134.2, 134.1, 132.3, 129.0, 127.9, 127.7, 126.3, 125.9, 125.6, 123.9, 73.2, 69.0, 44.6, 41.9, 24.2 ppm.

HRMS [M+H]$^+$ m/z calc'd for $[C_{15}H_{18}O_2Na]^+$ expect 253.1204; found 253.1207.

6-Phenylhexane-2,4-diol (3d)

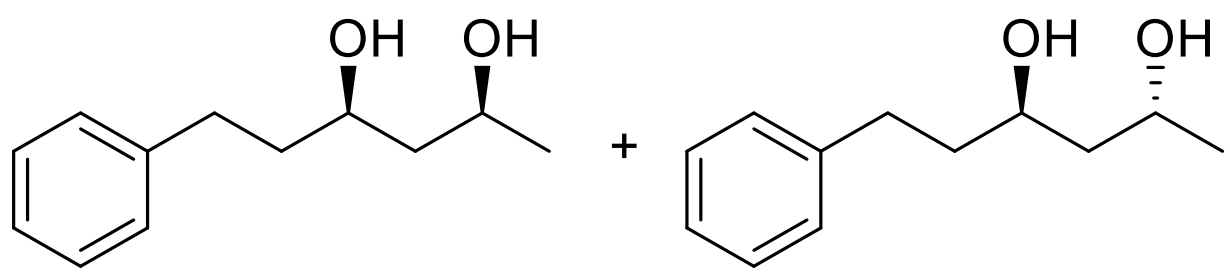

To a vial was added 6-Phenyl-2,4-hexanedione (380 mg, 2 mmol, 1.0 equiv.) (prepared by a reported procedure)[17] and MeOH (10 mL). The mixture was cooled to 0 °C and $NaBH_4$ (37.8 mg, 4.0 mmol, 2.0 equiv.) was added upon which the vial was lightly covered (hydrogen evolution) and the mixture stirred at room temperature for 3 h. Then, NaOH (1 M, aq., 1 mL) was added and the mixture extracted with Et2O (3 x 20 mL). The combined organic fractions were dried over $MgSO_4$ and the solvent removed under reduced pressure. The crude material was purified by flash column chromatography on silica gel (10 – 20% acetone in hexane) to give (*syn*)-6-Phenylhexane-2,4-diol (162.8 mg, 42%) as a colourless oil and (*anti*)-6-Phenylhexane-2,4-diol (102.5 mg, 26%) as a colourless solid.

(*anti*)-6-Phenylhexane-2,4-diol (*rac-anti*-3d)

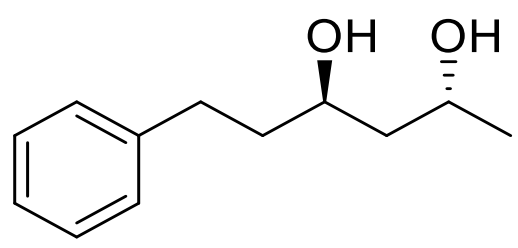

^{1}H NMR (500 MHz, $CDCl_3$) δ 7.31 – 7.26 (m, 2H), 7.22 – 7.17 (m, 3H), 4.23 – 4.14 (m, 1H), 4.02 – 3.94 (m, 1H), 2.83 – 2.76 (m, 1H), 2.72 – 2.63 (m, 1H), 2.47 (br. s, 1H), 2.27 (br. s, 1H), 1.91 – 1.83 (m, 1H), 1.81 – 1.74 (m, 1H), 1.69 – 1.60 (m, 2H), 1.24 (d, *J* = 6.3 Hz, 3H) ppm.

^{13}C NMR (126 MHz, $CDCl_3$) δ 142.1, 128.59, 128.54, 126.0, 69.0, 65.7, 44.2, 39.2, 32.3, 23.8 ppm.

The spectral data are in agreement with the one in literature.[18]

(*syn*)-6-Phenylhexane-2,4-diol (*rac*-*syn*-3d)

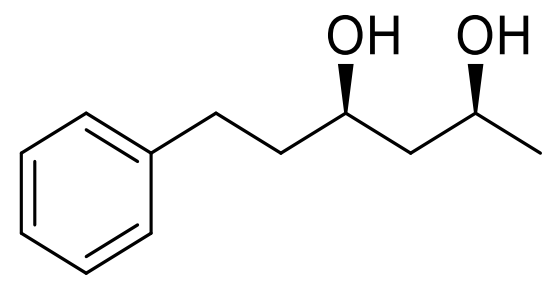

^{1}H NMR (500 MHz, $CDCl_3$) δ 7.31 – 7.26 (m, 2H), 7.22 – 7.17 (m, 3H), 4.08 – 4.00 (m, 1H), 3.92 – 3.85 (m, 1H), 3.62 (br. s, 1H), 3.50 (br. s, 1H), 2.80 – 2.73 (m, 1H), 2.72 – 2.65 (m, 1H), 1.85 – 1.71 (m, 2H), 1.62 – 1.50 (m, 2H), 1.21 (d, *J* = 6.2 Hz, 3H) ppm.

^{13}C NMR (126 MHz, $CDCl_3$) δ 142.1, 128.51, 128.50, 125.9, 72.3, 69.2, 44.7, 39.8, 31.8, 24.3 ppm.

The spectral data are in agreement with the literature.[19]

Ethyl 3-(3-hydroxy-5-oxohexyl)benzoate (*int$_1$*-3e)

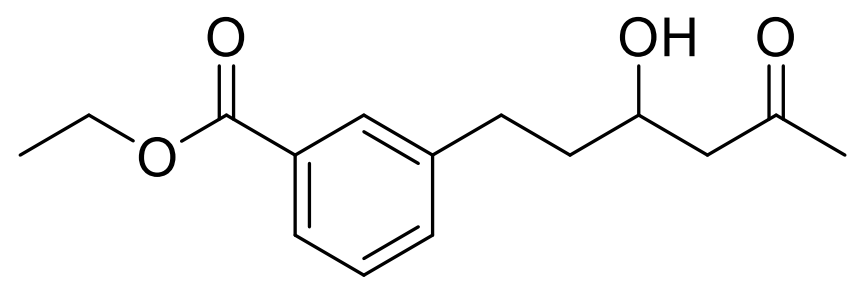

This procedure was adapted from literature.[20] In a heatgun-dried flask, 2-(Trimethylsilyloxy)propene (0.75 mL, 4.5 mmol, 1.5 equiv.) was dissolved in dry DCM (10 mL) and ethyl 3-(3-oxopropyl)benzoate (619 mg, 3.0 mmol, 1.0 equiv.)[21] was added. The mixture was cooled to –78 °C and $TiCl_4$ (1 M in DCM, 4.5 mL, 4.5 mmol, 1.5 equiv.) was added dropwise. The mixture was stirred for 3 h at –78 °C upon which it was quenched with cold water (10 mL) and $NaHCO_3$ (sat. aq.) was added. The layers were separated and the aqueous layer extracted with DCM (2 x 10 mL). The combined organic fractions were dried over $MgSO_4$ and the solvent removed under reduced pressure. The crude material was purified by flash column chromatography silica gel (20% acetone in hexane) to give the desired product (371 mg, 47%) as a pale-yellow oil.

^{1}H NMR (500 MHz, $CDCl_3$) δ 7.92 – 7.83 (m, 2H), 7.42 – 7.31 (m, 2H), 7.37, (q, *J* = 7.1 Hz, 2H), 4.07 – 4.00 (m, 1H), 3.11 (br. s, 1H), 2.91 – 2.81 (m, 1H), 2.78 – 2.69 (m, 1H), 2.67 – 2.53 (m, 2H), 2.17 (s, 3H), 1.87 – 1.78 (m, 1H), 1.74 – 1.66 (m, 1H), 1.39, (t, *J* = 7.1 Hz, 3H) ppm.

^{13}C NMR (126 MHz, $CDCl_3$) δ 210.0, 166.9, 142.3, 133.2, 130.8, 129.6, 128.6, 127.3, 66.7, 61.1, 50.0, 37.9, 31.7, 30.9, 14.5 ppm.

HRMS $[M+H]^+$ m/z calc'd for $[C_{15}H_{21}O_4]^+$ expect 265.1440; found 265.1451.

Ethyl 3-(3,5-dihydroxyhexyl)benzoate (3e)

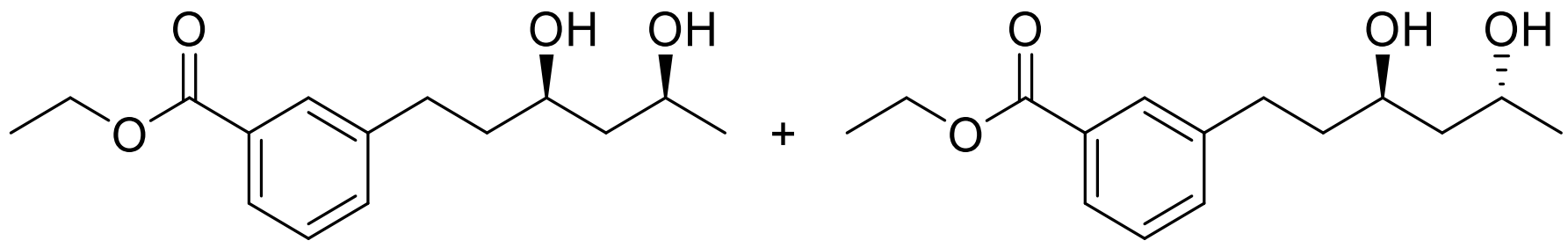

The compound was synthesised according to **General Procedure 3** using ethyl 3-(3-hydroxy-5-oxohexyl)benzoate (264 mg, 1.0 mmol), separated with 20% acetone in hexane, giving ethyl 3-((*syn*)-3,5-dihydroxyhexyl)benzoate (14.7 mg, 6%) as a colourless oil and 3-((*anti*)-3,5-dihydroxyhexyl)benzoate (154.3 mg, 58%) as a colourless solid.

Ethyl 3-((*anti*)-3,5-dihydroxyhexyl)benzoate (*rac-anti*-3e)

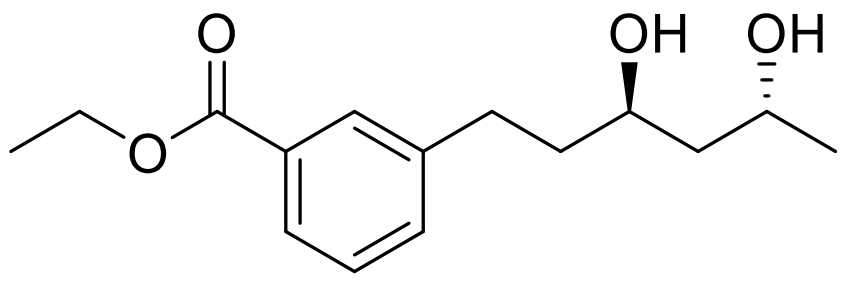

^{1}H NMR (500 MHz, $CDCl_3$) δ 7.90 – 7.84 (m, 2H), 7.41 – 7.37 (m, 1H), 7.35 (app. t, *J* = 7.6 Hz, 1H), 4.37 (q, *J* = 7.2 Hz, 2H), 4.23 – 4.16 (m, 1H), 4.01 – 3.93 (m, 1H), 2.90 – 2.82 (m, 1H), 2.77 – 2.69 (m, 1H), 2.11 (br. s, 2H), 1.92 – 1.83 (m, 1H), 1.82 – 1.74 (m, 1H), 1.70 – 1.59 (m, 2H), 1.40 (t, *J* = 7.2 Hz, 3H), 1.25 (d, *J* = 6.3 Hz, 3H) ppm.

^{13}C NMR (126 MHz, $CDCl_3$) δ 166.9, 142.5, 133.1, 130.8, 129.6, 128.6, 127.3, 68.8, 65.8, 61.1, 44.2, 39.1, 32.1, 23.8, 14.5 ppm.

HRMS [M+H]$^+$ m/z calc'd for $[C_{15}H_{22}O_4Na]^+$ expect 289.1416; found 289.1401.

Ethyl 3-((*syn*)-3,5-dihydroxyhexyl)benzoate (*rac-syn*-3e)

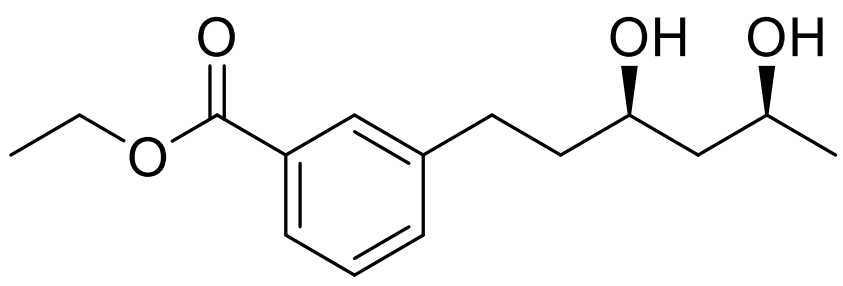

^{1}H NMR (500 MHz, $CDCl_3$) δ 7.90 – 7.85 (m, 2H), 7.41 – 7.37 (m, 1H), 7.35 (app. t, *J* = 7.6 Hz, 1H), 4.37 (q, *J* = 7.2 Hz, 2H), 4.10 – 4.00 (m, 1H), 3.92 – 3.85 (m, 1H), 2.86 – 2.79 (m, 1H), 2.77 – 2.70 (m, 1H), 2.52 (br. s, 2H), 1.83 – 1.73 (m, 2H), 1.64 – 1.51 (m, 2H), 1.39 (t, *J* = 7.2 Hz, 3H), 1.22 (d, *J* = 6.2 Hz, 3H) ppm.

^{13}C NMR (126 MHz, $CDCl_3$) δ 166.9, 142.4, 133.2, 130.8, 129.6, 128.6, 127.3, 72.2, 69.4, 61.1, 44.9, 39.8, 31.6, 24.6, 14.5 ppm.

HRMS [M+H]$^+$ m/z calc'd for $[C_{15}H_{23}O_4]^+$ expect 267.1596; found 267.1583.

4-Hydroxy-6-(2-(trifluoromethyl)phenyl)hexan-2-one (*int_1*-3f)

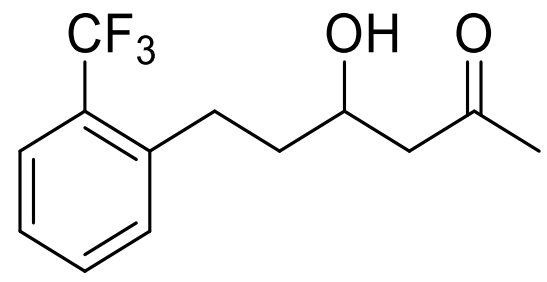

This procedure was adapted from literature.[20] In a heatgun-dried flask, 2-(Trimethylsilyloxy)propene (0.46 mL, 2.8 mmol, 1.4 equiv.) was dissolved in dry DCM (10 mL) and 3-(2-(trifluoromethyl)phenyl)propanal (404 mg, 2.0 mmol, 1.0 equiv.)[22] was added. The mixture was cooled to –78 °C and $TiCl_4$ (1 M in DCM, 3.0 mL, 3.0 mmol, 1.5 equiv.) was added dropwise. The mixture was stirred for 5 h at –78 °C upon which it was quenched with cold water (10 mL) and $NaHCO_3$ (sat. aq.) was added. The layers were separated and the aqueous layer extracted with DCM (2 x 10 mL). The combined organic fractions were dried over $MgSO_4$ and the solvent removed under reduced pressure. The crude material was purified by flash column chromatography silica gel (10% acetone in hexane) to give the desired product (321 mg, 62%) as a pale-yellow oil.

^{1}H NMR (700 MHz, $CDCl_3$) δ 7.61 (d, *J* = 7.8 Hz, 1H), 7.46 (app. t, *J* = 7.5 Hz, 1H), 7.36 (d, *J* = 7.7 Hz, 1H), 7.29 (app. t, *J* = 7.6 Hz, 1H), 4.14 – 4.08 (m, 1H), 3.13 (br. s, 1H), 3.02 – 2.95 (m, 1H), 2.87 – 2.80 (m, 1H), 2.66 (dd, , *J* = 17.8, 2.6 Hz, 1H), 2.59 (dd, *J* = 17.8, 9.1 Hz, 1H), 2.18 (s, 3H), 1.83 – 1.76 (m, 1H), 1.73 – 1.67 (m, 1H) ppm.

^{13}C NMR (176 MHz, $CDCl_3$) δ 210.0, 140.9, 132.0, 131.4, 128.6 (q, *J* = 29.8 Hz), 126.2, 126.1 (q, *J* = 5.1 Hz), 124.8 (q, *J* = 273.7 Hz), 67.1, 49.8, 38.3, 30.9, 28.8 ppm.

^{19}F NMR (376 MHz, $CDCl_3$) δ –59.6 ppm.

HRMS [M+H]$^+$ m/z calc'd for $[C_{13}H_{16}O_2F_3]^+$ expect 261.1102; found 261.1112.

6-(2-(Trifluoromethyl)phenyl)hexane-2,4-diol (3f)

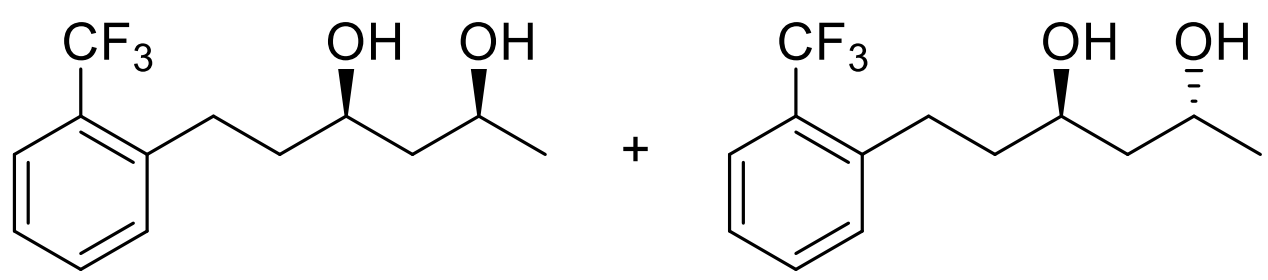

The compound was synthesised according to **General Procedure 3** using 4-hydroxy-6-(2-(trifluoromethyl)phenyl)hexan-2-one (260 mg, 1.0 mmol), separated with 10 – 20% acetone in hexane, giving (*syn*)-6-(2-(Trifluoromethyl)phenyl)hexane-2,4-diol (17.6 mg, 7%) as a colourless oil and (*anti*)-6-(2-(Trifluoromethyl)phenyl)hexane-2,4-diol (165.2 mg, 63%) as a colourless solid.

(*anti*)-6-(2-(Trifluoromethyl)phenyl)hexane-2,4-diol (*rac-anti*-3f)

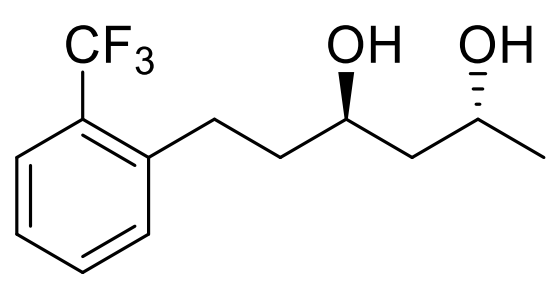

^{1}H NMR (700 MHz, $CDCl_3$) δ 7.62 (d, *J* = 7.9 Hz, 1H), 7.47 (app. t, *J* = 7.6 Hz, 1H), 7.36 (d, *J* = 7.7 Hz, 1H), 7.29 (app. t, *J* = 7.6 Hz, 1H), 4.24 – 4.18 (m, 1H), 4.06 – 4.01 (m, 1H), 3.01 – 2.94 (m, 1H), 2.86 – 2.79 (m, 1H), 1.96 (br. s, 2H), 1.87 – 1.81 (m, 1H), 1.81 – 1.75 (m, 1H), 1.71 – 1.62 (m, 2H), 1.26 (d, *J* = 6.3 Hz, 3H) ppm.

^{13}C NMR (176 MHz, $CDCl_3$) δ 141.1, 131.9, 131.3, 128.5 (q, *J* = 29.7 Hz), 126.12 (q, *J* = 5.8 Hz), 126.10, 124.8 (q, *J* = 273.8 Hz), 69.1, 65.8, 44.1, 39.5, 29.2, 23.8 ppm.

^{19}F NMR (376 MHz, $CDCl_3$) δ –59.6 ppm.

HRMS [M+H]$^+$ m/z calc'd for $[C_{13}H_{17}O_2F_3Na]^+$ expect 285.1078; found 285.1091.

(*syn*)-6-(2-(Trifluoromethyl)phenyl)hexane-2,4-diol (*rac-syn*-3f)

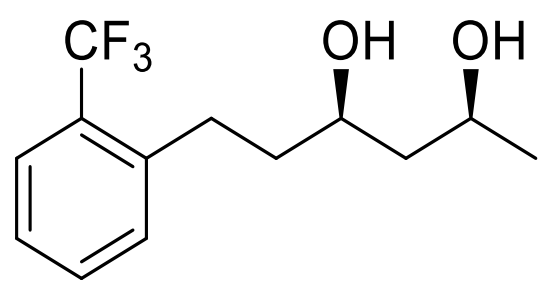

^{1}H NMR (700 MHz, $CDCl_3$) δ 7.61 (d, *J* = 7.9 Hz, 1H), 7.46 (app. t, *J* = 7.5 Hz, 1H), 7.36 (d, *J* = 7.7 Hz, 1H), 7.28 (app. t, *J* = 7.7 Hz, 1H), 4.11 – 4.06 (m, 1H), 3.97 – 3.92 (m, 1H), 2.98 – 2.91 (m, 1H), 2.87 – 2.80 (m, 1H), 2.47 (br. s, 2H), 1.82 – 1.73 (m, 2H), 1.64 – 1.53 (m, 2H), 1.24 (d, *J* = 6.2 Hz, 3H) ppm.

^{13}C NMR (176 MHz, $CDCl_3$) δ 141.1, 131.9, 131.3, 128.5 (q, *J* = 29.7 Hz), 126.12 (q, *J* = 5.7 Hz), 126.10, 124.8 (q, *J* = 273.8 Hz), 72.5, 69.5, 44.7, 40.1, 28.6, 24.6 ppm.

^{19}F NMR (376 MHz, $CDCl_3$) δ –59.6 ppm.

HRMS [M+H]$^+$ m/z calc'd for $[C_{13}H_{17}O_2F_3Na]^+$ expect 285.1078; found 285.1091.

4-(3-Hydroxy-5-oxohexyl)benzonitrile (*int$_1$*-3g)

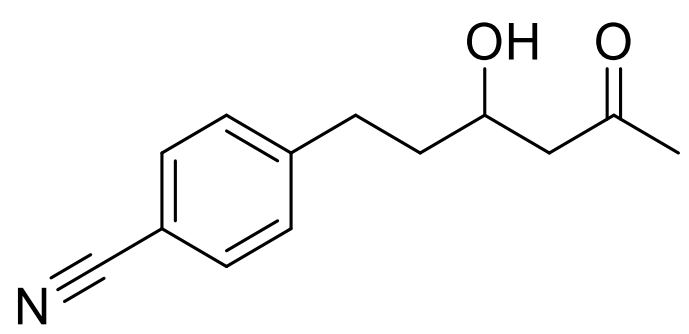

This procedure was adapted from literature.[20] In a heatgun-dried flask, 2-(Trimethylsilyloxy)propene (1.5 mL, 9.0 mmol, 1.5 equiv.) was dissolved in dry DCM (30 mL) and 4-(3-oxopropyl)benzonitrile (955 mg, 6.0 mmol, 1.0 equiv.)[23] was added. The mixture was cooled to –78 °C and $TiCl_4$ (1 M in DCM, 9.0 mL, 9.0 mmol, 1.5 equiv.) was added dropwise. The mixture was stirred for 5 h at –78 °C upon which it was quenched with cold water (10 mL) and $NaHCO_3$ (sat. aq.) was added. The layers were separated and the aqueous layer extracted with DCM (2 x 10 mL). The combined organic fractions were dried over $MgSO_4$ and the solvent removed under reduced pressure. The crude material was purified by flash column chromatography silica gel (20% acetone in hexane) to give the desired product (217 mg, 14%) as an orange solid.

^{1}H NMR (700 MHz, $CDCl_3$) δ 7.57 (d, *J* = 8.2 Hz, 2H), 7.30 (d, *J* = 8.2 Hz, 2H), 4.04 – 3.99 (m, 1H), 3.15 (br. s, 1H), 2.92 – 2.86 (m, 1H), 2.79 – 2.73 (m, 1H), 2.63 (dd, , *J* = 18.0, 2.9 Hz, 1H), 2.57 (dd, , *J* = 18.0, 8.9 Hz, 1H), 2.17 (s, 3H), 1.83 – 1.77 (m, 1H), 1.69 – 1.63 (m, 1H) ppm.

^{13}C NMR (176 MHz, $CDCl_3$) δ 209.9, 147.8, 132.4, 129.4, 119.2, 109.9, 66.5, 50.0, 37.4, 32.0, 30.9 ppm.

HRMS [M+H]$^+$ m/z calc'd for $[C_{13}H_{16}NO_2]^+$ expect 218.1181; found 218.1181.

4-((*anti*)-3,5-Dihydroxyhexyl)benzonitrile (*rac-anti*-3g)

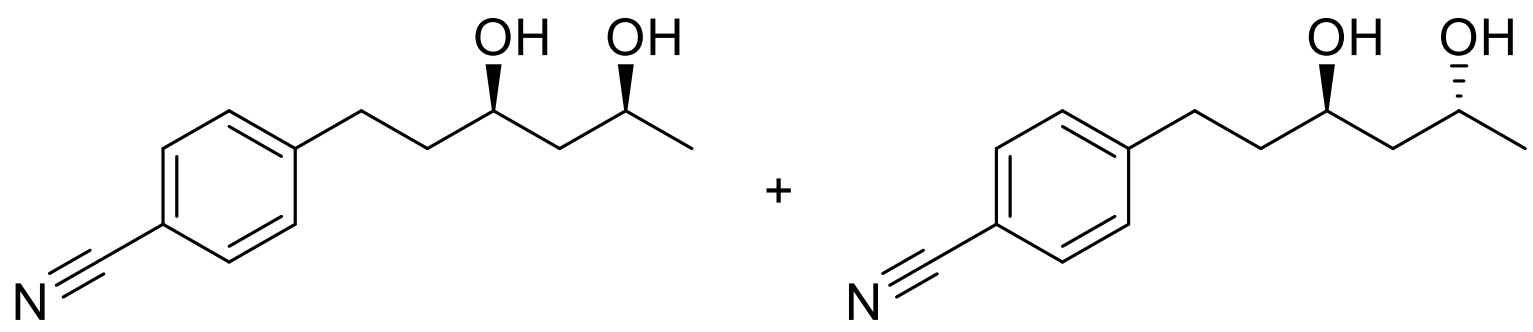

The compound was synthesised according to **General Procedure 3** using 4-(3-hydroxy-5-oxohexyl)benzonitrile (217 mg, 1.0 mmol), separated with 30% acetone in hexane, giving 4-((*syn*)-3,5-Dihydroxyhexyl)benzonitrile (17.6 mg, 8%) as a colourless solid and 4-((*anti*)-3,5-Dihydroxyhexyl)benzonitrile (110.7 mg, 50%) as a colourless solid.

4-((*anti*)-3,5-Dihydroxyhexyl)benzonitrile (*rac-anti*-3g)

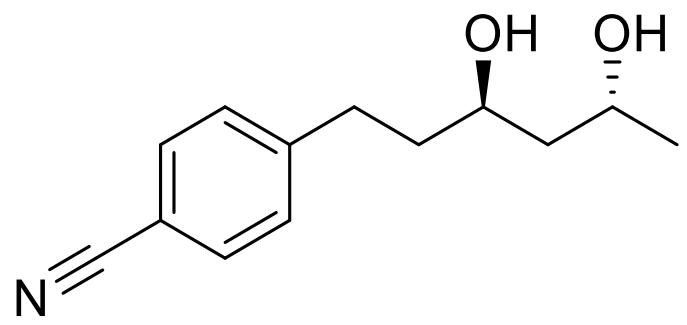

^{1}H NMR (700 MHz, $CDCl_3$) δ 7.57 (d, *J* = 8.3 Hz, 2H), 7.31 (d, *J* = 8.3 Hz, 2H), 4.22 – 4.17 (m, 1H), 3.98 – 3.93 (m, 1H), 2.91 – 2.85 (m, 1H), 2.77 – 2.71 (m, 1H), 1.99 (br. s, 2H), 1.88 – 1.82 (m, 1H), 1.78 – 1.72 (m, 1H), 1.69 – 1.59 (m, 2H), 1.25 (d, *J* = 6.3 Hz, 3H) ppm.

^{13}C NMR (176 MHz, $CDCl_3$) δ 148.0, 132.4, 129.4, 119.2, 109.9, 68.5, 65.9, 44.2, 38.7, 32.5, 23.9 ppm.

HRMS [M+H]$^+$ m/z calc'd for $[C_{13}H_{18}NO_2]^+$ expect 220.1338; found 220.1336.

4-((*syn*)-3,5-Dihydroxyhexyl)benzonitrile (*rac-syn*-3g)

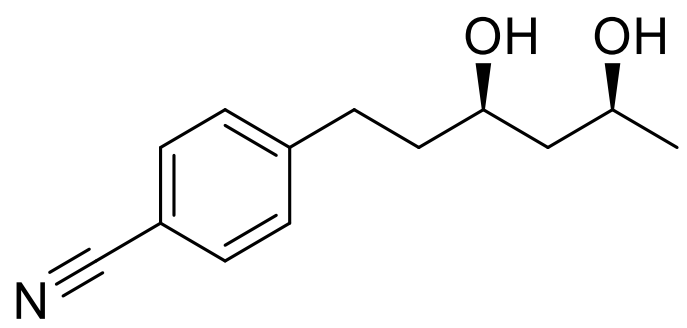

^{1}H NMR (700 MHz, $CDCl_3$) δ 7.57 (d, *J* = 8.2 Hz, 2H), 7.31 (d, *J* = 8.2 Hz, 2H), 4.09 – 4.04 (m, 1H), 3.88 – 3.84 (m, 1H), 2.87 – 2.82 (m, 1H), 2.78 – 2.73 (m, 1H), 2.42 (br. s, 2H), 1.82 – 1.70 (m, 2H), 1.60 – 1.52 (m, 2H), 1.23 (d, *J* = 6.2 Hz, 3H) ppm.

^{13}C NMR (176 MHz, $CDCl_3$) δ 148.0, 132.4, 129.4, 119.2, 109.9, 71.7, 69.6, 44.8, 39.2, 31.9, 24.8 ppm.

HRMS [M+H]$^+$ m/z calc'd for $[C_{13}H_{17}NO_2Na]^+$ expect 242.1157; found 242.1162.

2,2,2-trifluoro-*N*-(4-(3-oxopropyl)phenyl)acetamide (*int₁*-3h)

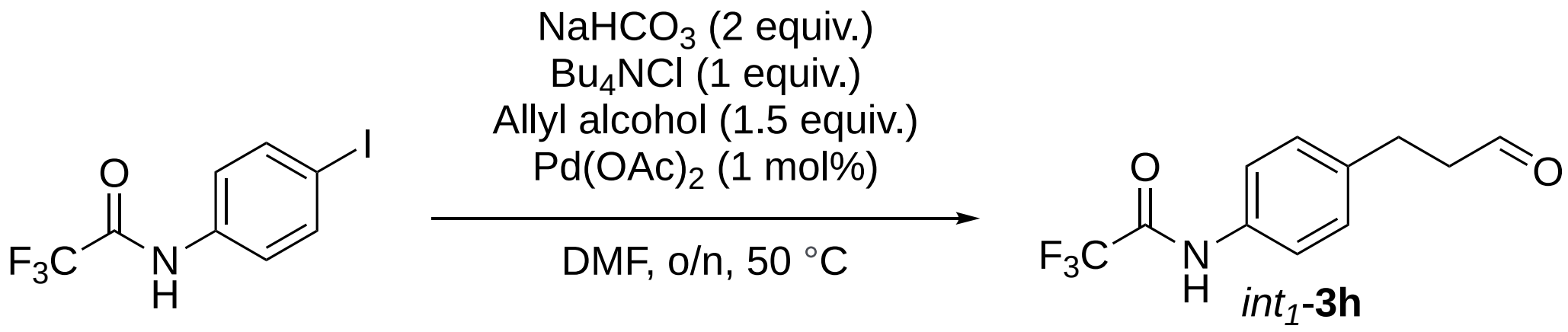

Preparation based on a literature procedure.[24] $NaHCO_3$ (1.68 g, 20 mmol, 2 equiv.), NBu_4Cl (2.78 g, 10 mmol, 1 equiv.) and $Pd(OAc)_2$ (22.5 mg, 0.1 mmol, 1 mol%) were added to a dried flask. A solution of 2,2,2-trifluoro-*N*-(4-iodophenyl)acetamide (3.15 g, 10 mmol, 1 equiv.) in DMF (5 ml) and allyl alcohol (1.0 ml, 15 mmol, 1.5 equiv.) were added successively. The reaction mixture was then stirred for overnight at 50 °C. The mixture was then diluted with Et_2O and filtered through celite washing with Et_2O. The filtrate was washed with water and then dried over $MgSO_4$. The solvent was removed *in vacuo* and the crude product purified *via* silica gel chromatography (15% EtOAc in Hexane) to give 2,2,2-trifluoro-*N*-(4-(3-oxopropyl)phenyl)acetamide as a pale yellow solid (677 mg, 2.76 mmol, 28%).

^{1}H NMR (700 MHz, $CDCl_3$) δ 9.81 (t, *J* = 1.3 Hz, 1H), 8.02 (br s, 1H), 7.49 (d, *J* = 8.5 Hz, 2H), 7.22 (d, *J* = 8.5 Hz, 2H), 2.96 (t, *J* = 7.5 Hz, 2H), 2.79 (td, *J* = 7.4, 1.1 Hz, 2H) ppm.

^{13}C NMR (176 MHz, $CDCl_3$) δ 201.4, 154.9 (q, *J* = 37.2 Hz), 138.8, 133.5, 129.4, 120.9, 115.9 (q, *J* = 288.6 Hz), 45.2, 27.6 ppm.

HRMS [M+Na]$^+$ m/z calc'd for $[C_{11}H_{10}F_3NO_2]^+$ expect 268.0561 found 268.0563

2,2,2-trifluoro-N-(4-iodophenyl)acetamide was prepared by Sumei Kinzelbach.

2,2,2-trifluoro-*N*-(4-(3-hydroxy-5-oxohexyl)phenyl)acetamide (*int₂*-3h)

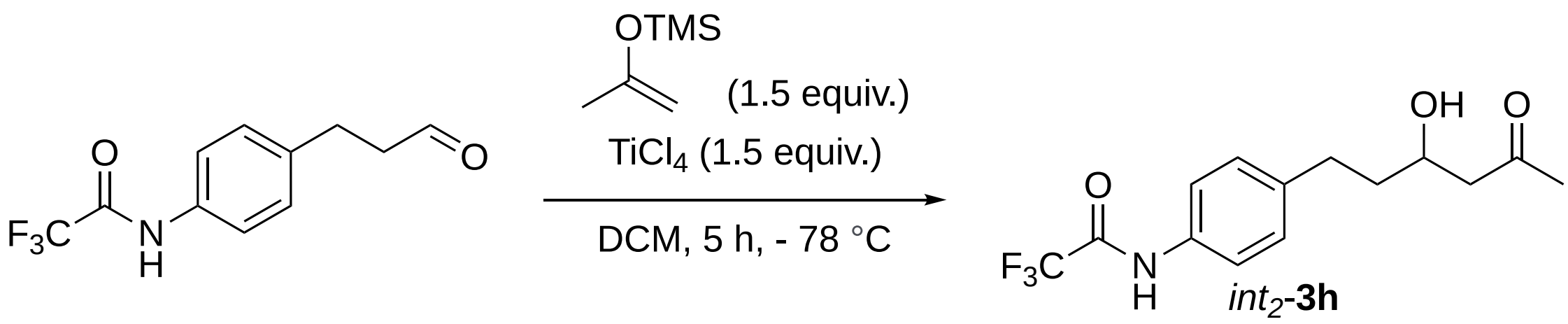

Preparation based on a literature procedure.[25] A solution of 2,2,2-trifluoro-*N*-(4-(3-oxopropyl)phenyl)acetamide (613 mg, 2.5 mmol, 1 equiv.) in DCM (1.3 ml) was added slowly to (Isopropenyloxy)trimethylsilane (0.6 ml, 3.75 mmol, 1.5 equiv.) in DCM (8 ml) at – 78 °C and stirred for 15 minutes. A solution of $TiCl_4$ (0.4 ml, 3.75 mmol, 1.5 equiv.) in DCM (2 ml) was added slowly to the reaction mixture. After stirring for 5 hours at – 78 °C the reaction mixture was quenched by the addition of cold water (4 ml) followed by saturated $NaHCO_3$ (2 ml). The layers were separated, and the aqueous layer washed with DCM. The organic layers were combined and dried over $MgSO_4$. The solvent was removed *in vacuo* and the crude product purified *via* silica gel chromatography (15 – 20% Acetone in Pet. Ether) to give 2,2,2-trifluoro-*N*-(4-(3-hydroxy-5-oxohexyl)phenyl)acetamide as a pale yellow solid (198 mg, 0.65 mmol, 26%).

^{1}H NMR (700 MHz, $CDCl_3$) δ 7.85 (br s, 1H), 7.47 (d, *J* = 8.5 Hz, 2H), 7.23 (d, *J* = 8.5 Hz, 2H), 4.05 – 4.00 (m, 1H), 3.09 (br s, 1H), 2.82 (ddd, *J* = 14.2, 9.4, 5.0 Hz, 1H), 2.69 (ddd, *J* = 13.4, 8.8, 7.6 Hz, 1H), 2.63 (dd, *J* = 17.9, 2.8 Hz, 1H), 2.57 (dd, *J* = 17.9, 9.0 Hz, 1H), 2.17 (s, 3H), 1.80 (dddd, *J* = 13.9, 9.2, 9.2, 4.9 Hz, 1H), 1.66 (dddd, *J* = 13.6, 9.9, 7.2, 3.8 Hz, 1H) ppm.

^{13}C NMR (176 MHz, $CDCl_3$) δ 210.0, 154.8 (q, *J* = 37.1 Hz), 140.4, 133.1, 129.5, 120.8, 115.9 (q, *J* = 288.8 Hz), 66.7, 50.0, 37.9, 31.3, 30.9 ppm.

HRMS [M+H]$^+$ m/z calc'd for $[C_{14}H_{17}F_3NO_3]^+$ expect 304.1161 found 304.1155

_N_-(4-(3,5-dihydroxyhexyl)phenyl)-2,2,2-trifluoroacetamide (3h)

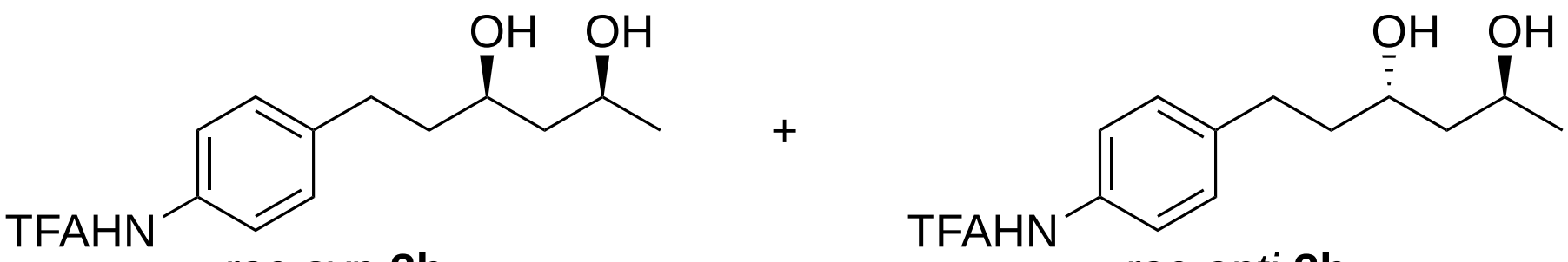

rac-syn-**3h** *rac-anti*-**3h**

Prepared according to **General Procedure 3** using hydroxy ketone *int₂*-**3h** (230 mg, 0.75 mmol), $Me_4NBH(OAc)_3$ (790 mg, 3.0 mmol, 4 equiv.) and glacial acetic acid (4.4 ml) stirring overnight. The crude product was purified *via* column chromatography (15 – 20% Acetone in Pet. Ether) to give (*syn*)-*N*-(4-(3,5-dihydroxyhexyl)phenyl)-2,2,2-trifluoroacetamide (16 mg, 0.05 mmol, 6%) as a white solid and (*anti*)-*N*-(4-(3,5-dihydroxyhexyl)phenyl)-2,2,2-trifluoroacetamide (116 mg, 0.38 mmol, 51%) as a white solid.

(_syn_)-_N_-(4-(3,5-dihydroxyhexyl)phenyl)-2,2,2-trifluoroacetamide (_rac-syn_-3h)

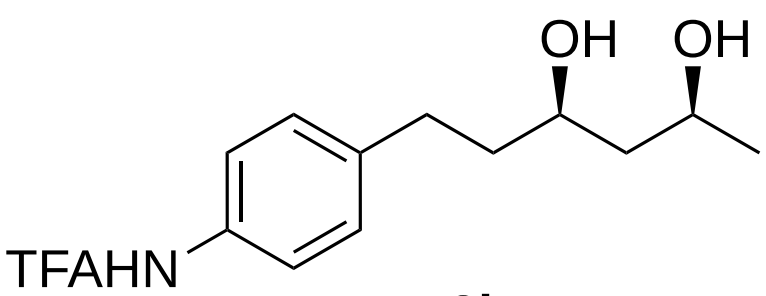

rac-syn-**3h**

^{1}H NMR (700 MHz, $(CD_3)_2CO$) δ 10.16 (br s, 1H), 7.63 (d, *J* = 8.5 Hz, 2H), 7.27 (d, *J* = 8.5 Hz, 2H), 4.21 (d, *J* = 3.4 Hz, 1H), 4.11 (d, *J* = 3.4 Hz, 1H), 4.01 – 3.94 (m, 1H), 3.83 – 3.77 (m, 1H), 2.78 (ddd, *J* = 13.8, 9.3, 6.0 Hz, 1H), 2.67 (ddd, *J* = 13.7, 9.5, 7.0 Hz, 1H), 1.76 – 1.67 (m, 2H), 1.57 (dt, *J* = 14.0, 3.3 Hz, 1H), 1.51 (dt, *J* = 14.0, 9.3 Hz, 1H), 1.13 (d, *J* = 6.2 Hz, 3H) ppm.

^{13}C NMR (176 MHz, $(CD_3)_2CO$) δ 155.5 (q, *J* = 36.9 Hz), 141.3, 135.1, 129.8, 121.7, 117.0 (q, *J* = 287.9 Hz), 71.3, 68.3, 46.2, 40.7, 31.8, 24.5 ppm.

HRMS [M+H]$^+$ m/z calc'd for $[C_{14}H_{19}F_3NO_3]^+$ expect 306.1317; found 306.1304

(_anti_)-_N_-(4-(3,5-dihydroxyhexyl)phenyl)-2,2,2-trifluoroacetamide (_rac-anti_-3h)

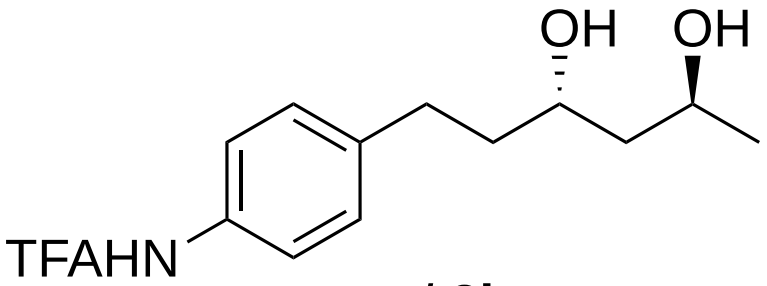

rac-anti-**3h**

^{1}H NMR (700 MHz, $(CD_3)_2CO$) δ 10.15 (br s, 1H), 7.62 (d, *J* = 8.5 Hz, 2H), 7.27 (d, *J* = 8.5 Hz, 2H), 4.09 – 4.03 (m, 1H), 3.89 – 3.84 (m, 1H), 3.77 (d, *J* = 5.3 Hz, 1H), 3.67 (d, *J* = 4.6 Hz, 1H), 2.79 (ddd, *J* = 13.7, 9.2, 6.1 Hz, 1H), 2.68 (ddd, *J* = 13.7, 9.0, 7.4 Hz, 1H), 1.78 – 1.69 (m, 2H), 1.53 (t, *J* = 5.8 Hz, 2H), 1.14 (d, *J* = 6.3 Hz, 3H) ppm.

^{13}C NMR (176 MHz, $(CD_3)_2CO$) δ 155.5 (q, *J* = 37.0 Hz), 141.4, 135.0, 129.8, 121.7, 117.0 (q, *J* = 288.0 Hz), 68.2, 65.0, 46.7, 40.6, 32.2, 24.5 ppm.

HRMS [M+H]$^+$ m/z calc'd for $[C_{14}H_{19}F_3NO_3]^+$ expect 306.1317; found 306.1311

5-Hydroxy-2-methyl-7-phenylheptan-3-one (*int₁*-3i)

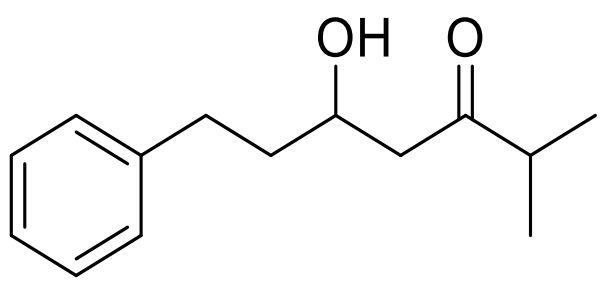

To a heat gun-dried flask was added NaHMDS (1 M in THF, 22 mL, 22 mmol, 1.1 equiv.) and dry THF (30 mL). The mixture was cooled to –78 °C, 3-methyl-2-butanone (2.2 mL, 20 mmol, 1.0 equiv.) was added and stirred at –78 °C for 30 min. Then, 3-phenylpropanal (4.0 mL, 30 mmol, 1.5 equiv.) was added and stirred at –78 °C for 90 min. The reaction was quenched with NH_4Cl (sat. aq., 20 mL) and the mixture extracted with EtOAc (3 x 50 mL) The combined organic fractions were dried over $MgSO_4$ and the solvent removed under reduced pressure. The crude material was purified by flash column chromatography on silica gel (10% EtOAc in hexane) to give the desired product (1.72 g, 37%) as a colourless oil.

^{1}H NMR (400 MHz, $CDCl_3$) δ 7.31 – 7.26 (m, 2H), 7.23 – 7.16 (m, 3H), 4.08 – 4.00 (m, 1H), 3.23 (br. s, 1H), 2.87 – 2.78 (m, 1H), 2.74 – 2.52, (m, 4H), 1.89 – 1.78 (m, 1H), 1.74 – 1.64 (m, 1H), 1.10 (d, *J* = 6.9 Hz, 3H), 1.09 (d, *J* = 6.9 Hz, 3H) ppm.

^{13}C NMR (176 MHz, $CDCl_3$) δ 216.3, 142.1, 128.62, 128.55, 126.0, 67.1, 46.7, 41.6, 38.2, 31.9, 18.2, 18.1 ppm.

The spectral data are in agreement with the one in literature.[26]

6-Methyl-1-phenylheptane-3,5-diol (3i)

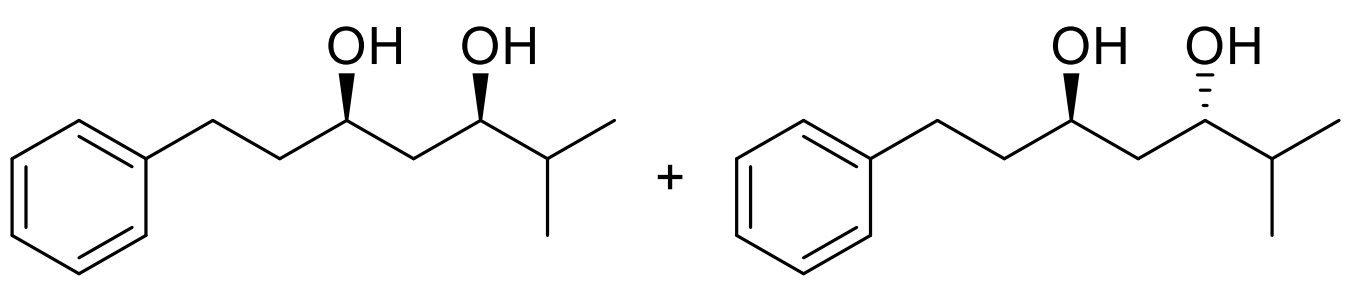

The compound was synthesised according to **General Procedure 3** using 5-hydroxy-2-methyl-7-phenylheptan-3-one (441 mg, 2.0 mmol), separated with 5 – 10% acetone in hexane, giving (*syn*)-6-Methyl-1-phenylheptane-3,5-diol (16.9 mg, 4%) as a colourless oil and (*anti*)-6-Methyl-1-phenylheptane-3,5-diol (225.4 mg, 51%) as a colourless solid.

(*anti*)-6-Methyl-1-phenylheptane-3,5-diol (*rac-anti*-3i)

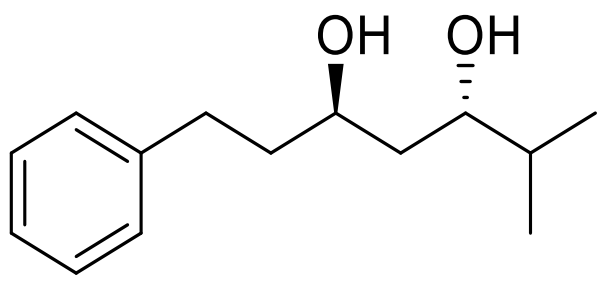

^{1}H NMR (500 MHz, $CDCl_3$) δ 7.31 – 7.27 (m, 2H), 7.23 – 7.16 (m, 3H), 4.00 – 3.94 (m, 1H), 3.72 – 3.66 (m, 1H), 2.85 – 2.76 (m, 1H), 2.72 – 2.64 (m, 1H), 2.02 (br. s, 2H), 1.93 – 1.84 (m, 1H), 1.83 – 1.75 (m, 1H), 1.72 – 1.64 (m, 2H), 1.60 (ddd, *J* = 14.5, 7.8, 2.8 Hz, 1H), 0.94 (d, *J* = 6.8 Hz, 3H), 0.90 (d, *J* = 6.8 Hz, 3H) ppm.

^{13}C NMR (126 MHz, $CDCl_3$) δ 142.2, 128.6, 128.5, 126.0, 74.2, 69.3, 39.6, 39.2, 34.0, 32.5, 18.7, 18.0 ppm.

HRMS [M+H]$^+$ m/z calc'd for $[C_{14}H_{22}O_2Na]^+$ expect 245.1517; found 245.1503.

(*syn*)-6-Methyl-1-phenylheptane-3,5-diol (*rac-syn*-3i)

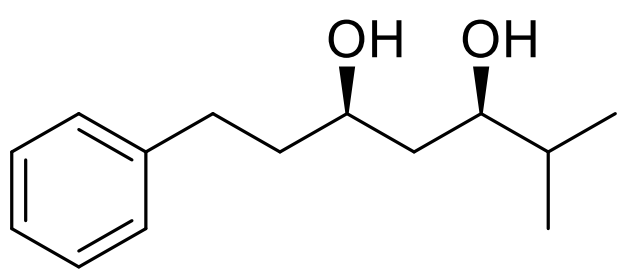

^{1}H NMR (700 MHz, $CDCl_3$) δ 7.30 – 7.27 (m, 2H), 7.22 – 7.18 (m, 3H), 3.90 – 3.85 (m, 1H), 3.66 – 3.62 (m, 1H), 2.81 – 3.76 (m, 1H), 3.73 – 2.67 (m, 1H), 2.51 (br. s, 2H), 1.86 – 1.75 (m, 2H), 1.69 – 1.63 (m, 1H), 1.61 (dt, *J* = 14.5, 2.0 Hz, 1H), 1.53 – 1.47 (m, 1H), 0.93 – 0.91 (m, 6H) ppm.

^{13}C NMR (176 MHz, $CDCl_3$) δ 142.2, 128.58, 128.56, 126.0, 78.3, 72.6, 39.9, 39.6, 34.5, 31.9, 18.4, 17.5 ppm.

HRMS [M+H]$^+$ m/z calc'd for $[C_{14}H_{22}O_2Na]^+$ expect 245.1517; found 245.1507.

***tert*-butyl 4-(2-hydroxy-4-oxo-6-phenylhexyl)piperidine-1-carboxylate (*int$_1$*-3j)**

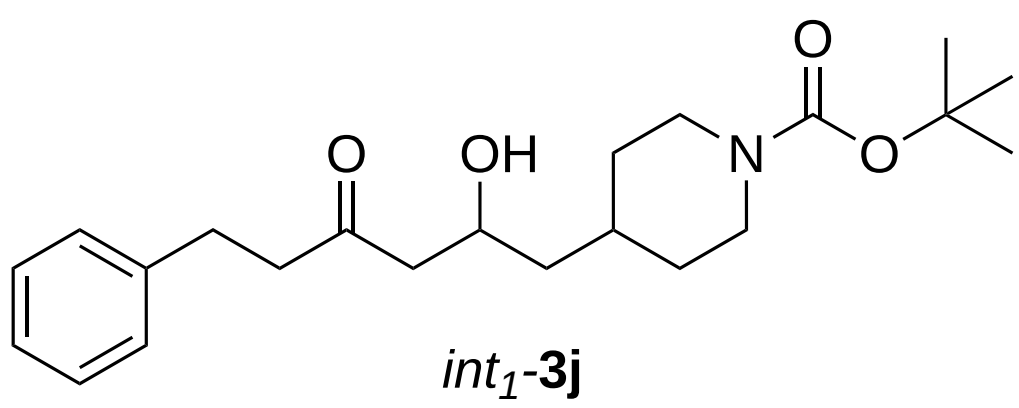

int$_1$-**3j**

Prepared according to **General Procedure 2** using diisopropylamine (1.15 ml, 8.1 mmol), *n*-butyllithium (5.2 ml, 8.25 mmol, 1.6 M in THF), 4-phenylbutan-2-one (1.15 ml, 7.5 mmol) and *tert*-butyl 4-(2-oxoethyl)piperidine-1-carboxylate (1.7 g, 7.5 mmol). The crude product was purified *via* column chromatography (15 – 20% Acetone in Pet. Ether) to give the title compound as a colourless oil (2.22 g, 5.9 mmol, 79%).

^{1}H NMR (700 MHz, $CDCl_3$) δ 7.29 – 7.25 (m, 2H), 7.19 (t, *J* = 7.0 Hz, 1H), 7.18 – 7.13 (m, 2H), 4.16 – 4.11 (m, 1H), 4.05 (br t, *J* = 12.8 Hz, 2H), 2.90 (t, *J* = 7.5 Hz, 2H), 2.75 (t, *J* = 7.7 Hz, 2H), 2.68 (br t, *J* = 12.6 Hz, 2H), 2.52 (dd, *J* = 17.6, 2.6 Hz, 1H), 2.48 (dd, *J* = 17.6, 8.6 Hz, 1H), 1.74 (br d, *J* = 13.0 Hz, 1H), 1.69 –

1.63 (m, 1H), 1.59 (br d, *J* = 12.95 Hz, 1H), 1.49 – 1.45 (m, 1H), 1.44 (s, 9H), 1.16 – 1.08 (m, 2H), 1.02 (qd, *J* = 12.2, 4.4 Hz, 1H) ppm.

^{13}C NMR (176 MHz, $CDCl_3$) δ 211.3, 155.0, 140.7, 128.7, 128.4, 126.4, 79.3, 64.8, 49.9, 45.1, 44.1, 44.0, 43.2, 33.0, 32.3, 31.6, 29.6, 28.6 ppm.

HRMS [M+H-Boc]$^+$ m/z calc'd for $[C_{17}H_{26}NO_2]^+$ expect 276.1964; found 276.1975

tert-butyl 4-(2,4-dihydroxy-6-phenylhexyl)piperidine-1-carboxylate (3j)

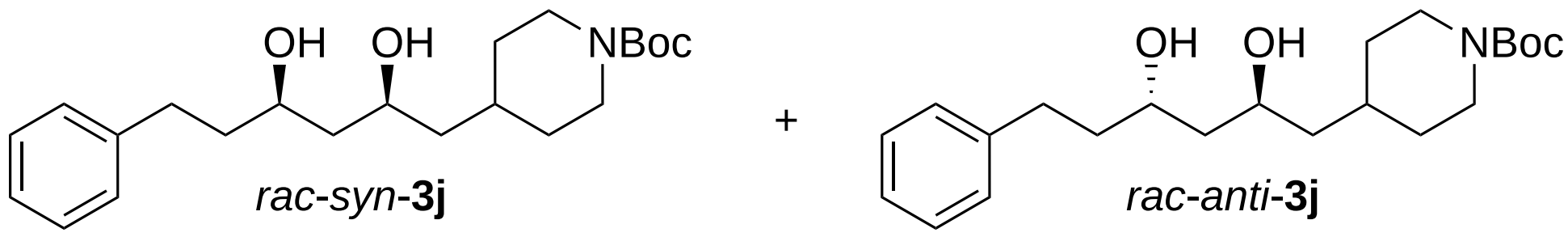

rac-syn-**3j** *rac-anti*-**3j**

Prepared according to **General Procedure 3** using hydroxy ketone *int$_1$*-**3j** (1.89 g, 5.0 mmol), $Me_4NBH(OAc)_3$ (3.95 g, 15.0 mmol, 3 equiv.) and glacial acetic acid (24 ml) stirring overnight. The crude product was purified *via* column chromatography (20% Acetone in Pet. Ether) to give (*syn*)-*tert*-butyl 4-(2,4-dihydroxy-6-phenylhexyl)piperidine-1-carboxylate (46 mg, 0.12 mmol, 2%) as a colourless oil, (*anti*)-*tert*-butyl 4-(2,4-dihydroxy-6-phenylhexyl)piperidine-1-carboxylate (516 mg, 1.37 mmol, 27%) as a white solid and mixed fractions (211 mg, 0.56 mmol, 11%).

(*syn*)-*tert*-butyl 4-(2,4-dihydroxy-6-phenylhexyl)piperidine-1-carboxylate (*rac-syn*-3j)

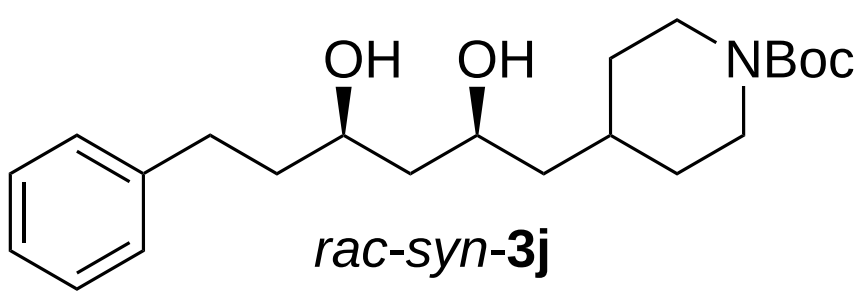

rac-syn-**3j**

^{1}H NMR (700 MHz, $CDCl_3$) δ 7.30 – 7.26 (m, 2H), 7.21 – 7.17 (m, 3H), 4.06 (br s, 2H), 3.97 – 3.92 (m, 1H), 3.91 – 3.87 (m, 1H), 3.08 (br s, 2H), 2.76 (ddd, *J* = 13.9, 9.5, 5.7 Hz, 1H), 2.72 – 2.64 (m, 3H), 1.85 – 1.72 (m, 3H), 1.66 – 1.45 (m, 5H), 1.45 (s, 9H), 1.27 (ddd, *J* = 13.9, 8.3, 4.2 Hz, 1H), 1.14 (qd, *J* = 12.8, 4.2 Hz, 1H), 1.06 (qd, *J* = 12.2, 4.4 Hz, 1H) ppm.

^{13}C NMR (176 MHz, $CDCl_3$) δ 155.0, 141.9, 128.6, 128.5, 126.0, 79.4, 72.6, 70.1, 45.1, 43.7, 40.0, 33.0, 32.2, 31.8, 28.6 ppm.

HRMS [M+H]$^+$ m/z calc'd for $[C_{22}H_{36}NO_4]^+$ expect 378.2644; found 378.2636

(*anti*)-*tert*-butyl 4-(2,4-dihydroxy-6-phenylhexyl)piperidine-1-carboxylate (*rac-anti*-3j)

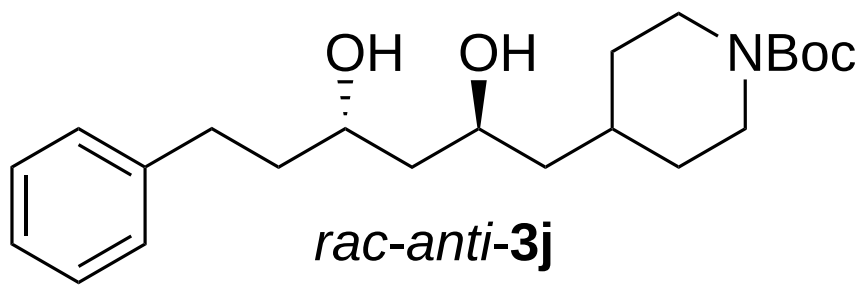

rac-anti-**3j**

^{1}H NMR (700 MHz, $CDCl_3$) δ 7.30 – 7.27 (m, 2H), 7.21 – 7.17 (m, 3H), 4.10 – 4.03 (m, 3H), 3.99 (p, *J* = 4.0 Hz, 1H), 2.79 (ddd, *J* = 13.8, 9.8, 5.7 Hz, 1H), 2.72 – 2.64 (m, 3H), 2.30 (br s, 2H), 1.87 (dddd, *J* = 13.8,

9.6, 8.2, 5.7 Hz, 1H), 1.81 – 1.71 (m, 2H), 1.66 – 1.58 (m, 4H), 1.52 (ddd, *J* = 14.0, 9.1, 5.1 Hz, 1H), 1.45 (s, 9H), 1.25 (ddd, *J* = 13.6, 8.8, 4.4 Hz, 1H), 1.12 (qd, *J* = 12.3, 4.2 Hz, 1H), 1.05 (qd, *J* = 12.2, 4.3 Hz, 1H) ppm.

^{13}C NMR (176 MHz, CDCl$_3$) δ 155.1, 142.0, 128.6, 128.5, 126.1, 79.4, 69.1, 66.5, 44.4, 43.3, 39.3, 33.0, 32.7, 32.4, 31.9, 28.6 ppm.

HRMS [M+H]$^+$ m/z calc'd for $[C_{22}H_{36}NO_4]^+$ expect 378.2644; found 378.2643

3-Hydroxy-1-phenylnonan-5-one (*int$_1$*-3k)

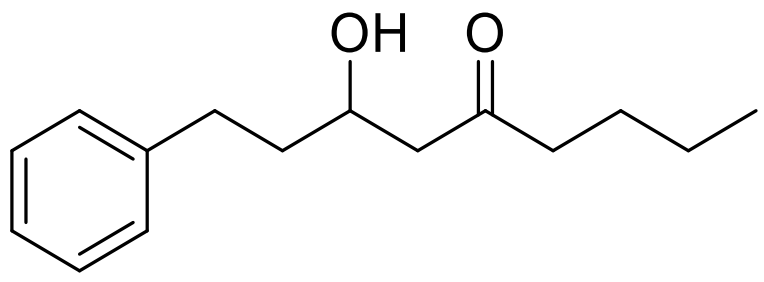

This procedure was adapted from literature.[26] To a heat gun-dried flask was added diisopropylamine (3.1 mL, 22 mmol, 1.1 equiv.) and dry THF (60 mL). The solution was cooled to –78 °C and *n*BuLi (1.6 M in hexanes, 13.8 mL, 22 mmol, 1.1 equiv.) was added and the mixture stirred at –30 °C for 30 min. Then, it was again cooled to –78 °C and hexan-2-one (2.5 mL, 20 mmol, 1.0 equiv.) was added. The mixture was stirred at –78 °C for 30 min upon which 3-phenylpropanal (4.0 mL, 30 mmol, 1.5 equiv.) was added and stirred at –78 °C for 80 min. The reaction was quenched with NH_4Cl (sat. aq., 20 mL) and the mixture extracted with EtOAc (3 x 50 mL) The combined organic fractions were dried over $MgSO_4$ and the solvent removed under reduced pressure. The crude material was purified by flash column chromatography on silica gel (10% EtOAc in hexane) to give the desired product (1.48 g, 32%) as a colourless oil.

^{1}H NMR (500 MHz, CDCl$_3$) δ 7.32 – 7.26 (m, 2H), 7.22 – 7.16 (m, 3H), 4.08 – 4.01 (m, 1H), 3.07 (br. s, 1H), 2.86 – 2.76 (m, 1H), 2.73 – 2.64 (m, 1H), 2.63 – 2.48 (m, 2H), 2.41 (t, *J* = 7.4 Hz, 2H), 1.87 – 1.77 (m, 1H), 1.73 – 1.64 (m, 1H), 1.60 – 1.51 (m, 2H), 1.35 – 1.26 (m, 2H), 0.90 (t, *J* = 7.3 Hz, 3H) ppm.

^{13}C NMR (126 MHz, CDCl$_3$) δ 212.7, 142.0, 128.61, 128.55, 126.0, 67.0, 49.0, 43.5, 38.2, 31.9, 25.8, 22.4, 14.0 ppm.

The spectral data are in agreement with the one in literature.[26]

(*anti*)-1-Phenylnonane-3,5-diol (*rac-anti*-3k)

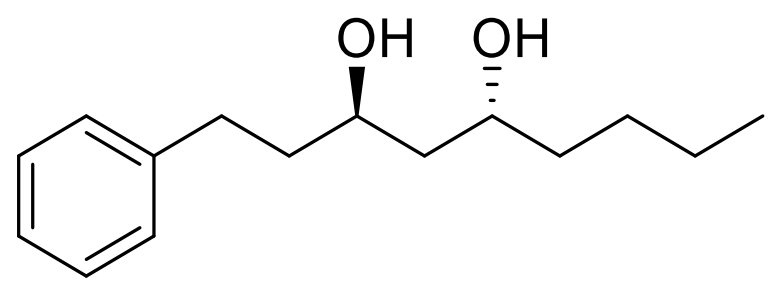

The compound was synthesised according to **General Procedure 3** using 3-hydroxy-1-phenylnonan-5-one (234 mg, 1.0 mmol), separated with 5 – 10% acetone in hexane, giving (*anti*)-1-Phenylnonane-3,5-diol (108.0 mg, 45%) as a colourless solid.

^{1}H NMR (500 MHz, CDCl$_3$) δ 7.31 – 7.26 (m, 2H), 7.22 – 7.17 (m, 3H), 4.03 – 3.91 (m, 2H), 2.84 – 2.75 (m, 1H), 2.72 – 2.63 (m, 1H), 1.92 – 1.73 (m, 4H), 1.70 – 1.60 (m, 2H), 1.57 – 1.23 (m, 6H), 0.91 (t, *J* = 6.9 Hz, 3H) ppm.

^{13}C NMR (126 MHz, $CDCl_3$) δ 142.1, 128.6, 128.5, 126.0, 69.7, 69.1, 42.6, 39.3, 37.4, 32.4, 28.1, 22.8, 14.2 ppm.

HRMS [M+H]$^+$ m/z calc'd for $[C_{15}H_{25}O_2]^+$ expect 237.1855; found 237.1851.

(*syn*)-1-Phenylnonane-3,5-diol (*rac*-*syn*-3k)

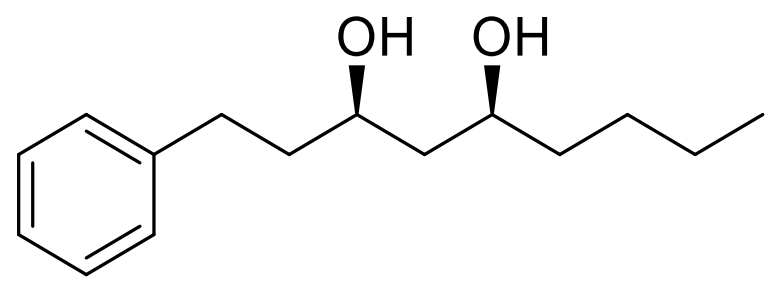

This procedure was adapted from literature.[27] In a heat gun-dried flask under nitrogen 3-hydroxy-1-phenylnonan-5-one (234 mg, 1.0 mmol, 1.0 equiv.) was dissolved in dry THF (7 mL) and dry MeOH (2 mL) and the mixture cooled to –78 °C. Methoxydiethylborane (1 M in THF, 1.1 mL, 1.1 mmol, 1.1 equiv.) was added dropwise and the mixture stirred for 15 min. Then, $NaBH_4$ (41.6 mg, 1.1 mmol, 1.1 equiv.) was added and the mixture stirred for 4 h upon which acetic acid (1 mL) was added. The mixture was then diluted with EtOAc (20 mL), washed with $NaHCO_3$ (sat. aq., 10 mL) and the layers separated. The organic layer was dried over $MgSO_4$ and the solvent evaporated under reduced pressure. MeOH was added (10 mL). The solvent was removed under reduced pressure again and the addition of MeOH was repeated two more times. The crude material was purified by flash column chromatography silica gel (5% acetone in hexane) to give the desired product (85.5 mg, 36%) as colourless oil containing 12% (*anti*)-1-Phenylnonane-3,5-diol.

^{1}H NMR (500 MHz, $CDCl_3$) δ 7.31 – 7.27 (m, 2H), 7.22 – 7.17 (m, 3H), 3.92 – 3.81 (m, 2H), 2.81 – 2.74 (m, 1H), 2.74 – 2.65 (m, 1H), 2.01 (br. s, 2H), 1.86 – 1.72 (m, 2H,), 1.62 (dt, *J* = 14.5, 2.3 Hz, 1H), 1.56 – 1.25 (m, 7H), 0.91 (t, *J* = 7.0 Hz, 3H) ppm.

^{13}C NMR (126 MHz, $CDCl_3$) δ 142.1, 128.6 (2C signals), 126.0, 73.4, 72.5, 43.1, 39.9, 38.2, 31.9, 27.6, 22.8, 14.2 ppm.

HRMS [M+H]$^+$ m/z calc'd for $[C_{15}H_{25}O_2]^+$ expect 237.1855; found 237.1855.

5-hydroxy-1-phenyl-7-((2,3,3-trimethylbutan-2-yl)oxy)heptan-3-one (*int$_1$*-3l)

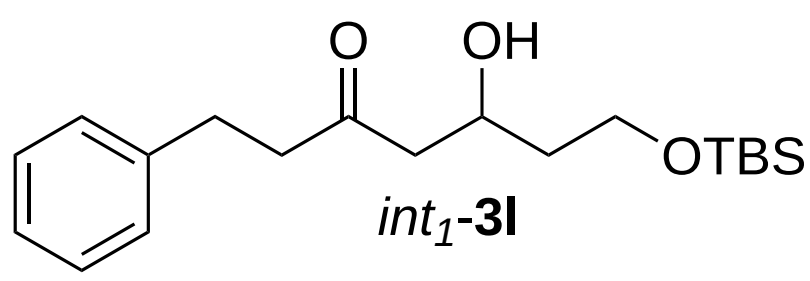

int$_1$-**3l**

Prepared according to **General Procedure 2** using diisopropylamine (1.15 ml, 8.1 mmol), *n*-butyllithium (5.2 ml, 8.25 mmol, 1.6 M in THF), 4-phenylbutan-2-one (1.15 ml, 7.5 mmol) and 3-((2,3,3-trimethylbutan-2-yl)oxy)propanal (1.6 ml, 7.5 mmol). The crude product was purified *via* column chromatography (5% Acetone in Pet. Ether) to give the title compound as a pale orange oil (993 mg, 2.95 mmol, 39%).

^{1}H NMR (700 MHz, $CDCl_3$) δ 7.27 (app t, *J* = 7.6 Hz, 2H), 7.18 (app t, *J* = 8.4 Hz, 3H), 4.26 (sep, *J* = 4.1 Hz, 1H), 3.83 (ddd, *J* = 10.5, 5.9, 4.6 Hz, 1H), 3.78 (ddd, *J* = 10.3, 7.5, 4.5 Hz, 1H), 2.90 (t, *J* = 7.6 Hz, 2H),

2.79 – 2.76 (m, 2H), 2.62 (dd, *J* = 16.6, 8.2 Hz, 1H), 2.53 (dd, *J* = 16.7, 4.1 Hz, 1H), 1.70 – 1.60 (m, 2H), 0.90 (s, 9H), 0.07 (s, 6H) ppm.

^{13}C NMR (176 MHz, $CDCl_3$) δ 210.0, 140.9, 128.5, 128.3, 126.2, 67.2, 61.5, 49.9, 45.2, 38.4, 29.5, 25.9, 18.2, -5.4, -5.4 ppm.

Data is in good agreement with the literature.[28]

1-phenyl-7-((2,3,3-trimethylbutan-2-yl)oxy)heptane-3,5-diol (3l)

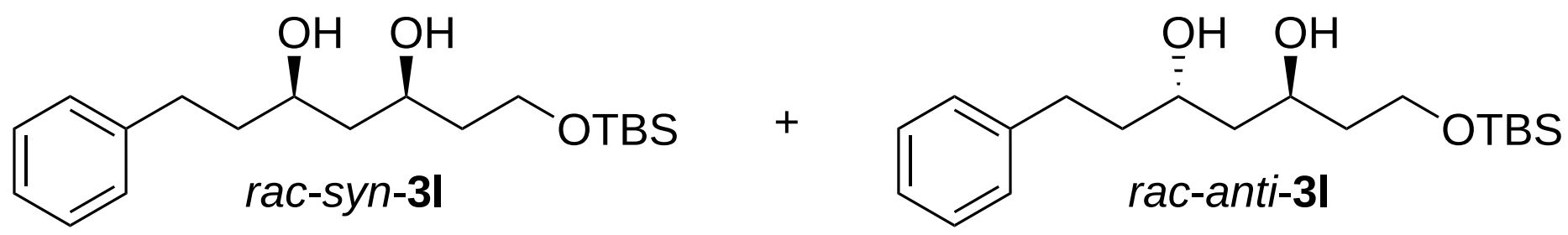

Prepared according to **General Procedure 3** using hydroxy ketone *int$_1$*-**3l** (841 mg, 2.5 mmol), $Me_4NBH(OAc)_3$ (2.63 g, 10.0 mmol, 4 equiv.) and glacial acetic acid (13 ml) stirring overnight. The crude product was purified *via* column chromatography (5% Acetone in Pet. Ether) to give (*syn*)-1-phenyl-7-((2,3,3-trimethylbutan-2-yl)oxy)heptane-3,5-diol (22 mg, 0.07 mmol, 3%) as a colourless oil, (*anti*)-1-phenyl-7-((2,3,3-trimethylbutan-2-yl)oxy)heptane-3,5-diol (193 mg, 0.57 mmol, 23%) as a white solid and mixed fractions (108 mg, 0.32 mmol, 13%).

(*syn*)-1-phenyl-7-((2,3,3-trimethylbutan-2-yl)oxy)heptane-3,5-diol (*rac-syn*-3l)

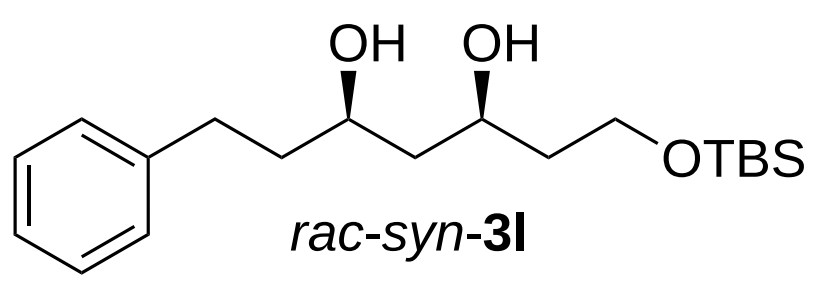

^{1}H NMR (700 MHz, $CDCl_3$) δ 7.28 (app t, *J* = 7.6 Hz, 2H), 7.22 (br d, *J* = 6.9 Hz, 2H), 7.18 (app t, *J* = 7.2 Hz, 1H), 4.13 – 4.08 (m, 1H), 3.94 – 3.89 (m, 2H), 3.83 (ddd, *J* = 10.0, 9.2, 3.6 Hz, 1H), 2.79 (ddd, *J* = 13.8, 10.1, 5.4 Hz, 1H), 2.70 (ddd, *J* = 14.3, 9.5, 6.0 Hz, 1H), 1.82 (dddd, *J* = 13.5, 10.0, 7.9, 5.6 Hz, 1H), 1.75 – 1.69 (m, 2H), 1.66 – 1.60 (m, 2H), 1.54 (dt, *J* = 14.1, 2.3 Hz, 1H), 0.90 (s, 9H), 0.09 (s, 6H) ppm.

^{13}C NMR (176 MHz, $CDCl_3$) δ 142.5, 128.6, 128.5, 125.8, 73.8, 71.7, 62.8, 43.4, 39.7, 38.8, 31.9, 26.0, 18.2, -5.4, -5.4 ppm.

HRMS $[M+H]^+$ m/z calc'd for $[C_{19}H_{35}O_3Si]^+$ expect 339.2355; found 339.2350

(*anti*)-1-phenyl-7-((2,3,3-trimethylbutan-2-yl)oxy)heptane-3,5-diol (*rac-anti*-3l)

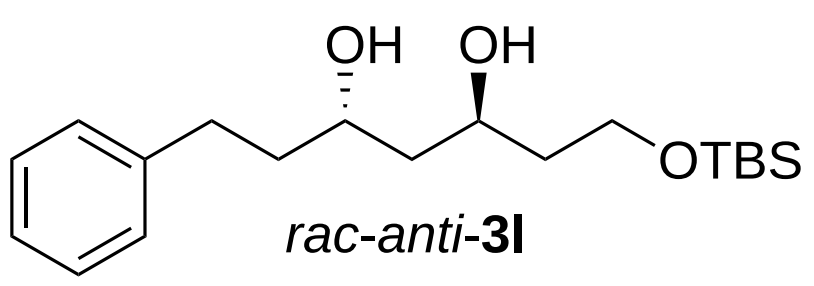

^{1}H NMR (700 MHz, $CDCl_3$) δ 7.28 (app t, *J* = 7.6 Hz, 2H), 7.21 (d, *J* = 7.0 Hz, 2H), 7.18 (app t, *J* = 7.3 Hz, 1H), 4.24 – 4.19 (m, 1H), 3.98 (sep, *J* = 4.0 Hz, 1H), 3.92 (dt, *J* = 9.4, 4.7 Hz, 1H), 3.84 (td, *J* = 10.1, 3.2 Hz, 1H), 2.82 (ddd, *J* = 13.8, 10.2, 6.2 Hz, 1H), 2.67 (ddd, *J* = 13.8, 10.2, 6.2 Hz, 1H), 1.90 – 1.80 (m, 2H),

1.75 (dddd, J = 13.6, 10.4, 6.2, 4.3 Hz, 1H), 1.69 – 1.62 (m, 2H), 1.60 – 1.54 (m, 1H), 0.90 (s, 9H), 0.09 (d, J = 1.3 Hz, 6H) ppm.

^{13}C NMR (176 MHz, $CDCl_3$) δ 142.5, 128.6, 128.5, 125.9, 70.8, 68.7, 63.4, 42.9, 39.4, 38.0, 32.4, 26.0, 18.2, -5.4, -5.4 ppm.

HRMS [M+H]$^+$ m/z calc'd for $[C_{19}H_{35}O_3Si]^+$ expect 339.2355; found 339.2354

4-hydroxy-1-tosylpentan-2-one (*int₁*-3m)

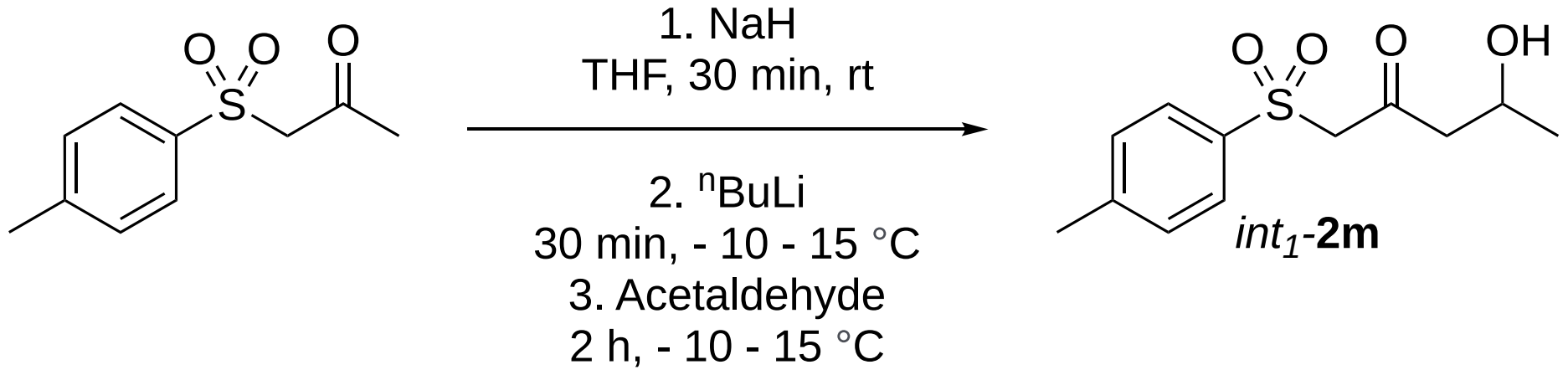

Preparation based upon a literature procedure.[29] 1-tosylpropan-2-one (1.06 g, 5.0 mmol, 1 equiv.) in THF (1.5 ml) was added slowly to a suspension of NaH (144 mg, 6.0 mmol, 1.2 equiv.) in THF (1.5 ml). The resulting mixture was stirred for 30 minutes at room temperature before being cooled to – 10 – – 15 °C. At this temperature ^{n}BuLi (3.75 ml, 6.0 mmol, 1.6 M in THF, 1.2 equiv.) was added slowly then the mixture stirred for 30 minutes. After this acetaldehyde (0.34 ml, 6.0 mmol, 1.2 equiv.) was added and the mixture stirred for 2 hours at low temperature before being quenched with saturated NH_4Cl (18 ml). The layers were separated, and the aqueous layer washed with EtOAc (3 × 20 ml). The organic layers were combined and dried over $MgSO_4$. The solvent was removed *in vacuo* and the crude product purified *via* silica gel chromatography (15 – 20% Acetone in Hexane) to give 4-hydroxy-1-tosylpentan-2-one (205 mg, 0.85 mmol, 17%) as a colourless oil.

^{1}H NMR (700 MHz, $CDCl_3$) δ 7.74 (d, J = 8.3 Hz, 2H), 7.35 (d, J = 8.1 Hz, 2H), 4.24 – 4.13 (m, 3H), 2.85 (dd, J = 17.6, 3.2 Hz, 1H), 2.79 (dd, J = 17.6, 8.5 Hz, 1H), 2.70 – 2.65 (m, 1H), 2.43 (s, 3H), 1.19 (d, J = 6.3 Hz, 3H) ppm.

^{13}C NMR (176 MHz, $CDCl_3$) δ 199.0, 145.7, 135.7, 130.1, 128.3, 67.6, 63.8, 52.5, 22.6, 21.8 ppm.

The data is in good agreement with the literature.[30]

(*anti*)-(2*R*,4*S*)-1-tosylpentane-2,4-diol (*rac-anti*-3m)

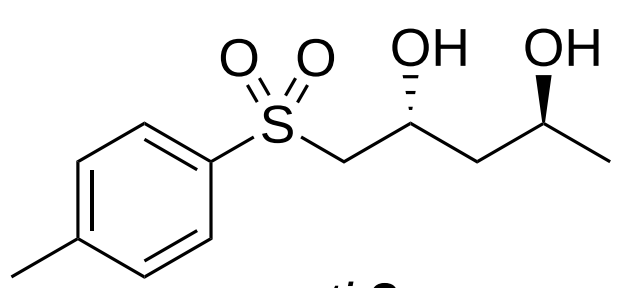

rac-anti-**3m**

Prepared according to **General Procedure 3** using hydroxy ketone 4-hydroxy-1-tosylpentan-2-one (205 mg, 0.9 mmol), $Me_4NBH(OAc)_3$ (950 mg, 3.6 mmol, 4 equiv.) and glacial acetic acid (4.4 ml) stirring overnight. The crude product was purified *via* column chromatography (20 – 25% Acetone in Pet. Ether) to give (*anti*)-(2*R*,4*S*)-1-tosylpentane-2,4-diol (149 mg, 0.58 mmol, 64%) as a white solid.

^{1}H NMR (700 MHz, $CDCl_3$) δ 7.80 (d, *J* = 7.4 Hz, 2H), 7.37 (d, *J* = 7.8 Hz, 2H), 4.48 – 4.42 (m, 1H), 4.12 – 4.06 (m, 1H), 3.31 (dd, *J* = 14.4, 9.2 Hz, 1H), 3.22 (br d, *J* = 14.3 Hz, 1H), 2.46 (s, 3H), 1.71 – 1.65 (m, 1H), 1.57 (ddd, *J* = 13.9, 9.4, 4.0 Hz, 1H), 1.20 (d, *J* = 6.3 Hz, 3H) ppm.

^{13}C NMR (176 MHz, $CDCl_3$) δ 145.4, 136.4, 130.2, 128.1, 64.8, 64.2, 62.3, 43.9, 23.8, 21.8 ppm.

HRMS [M+H]$^+$ m/z calc'd for $[C_{12}H_{19}O_4S]^+$ expect 259.1004 found 259.1008

(*syn*)-(2*R*,4*S*)-1-tosylpentane-2,4-diol and (*anti*)-(2*R*,4*S*)-1-tosylpentane-2,4-diol (*rac-syn*-3m)

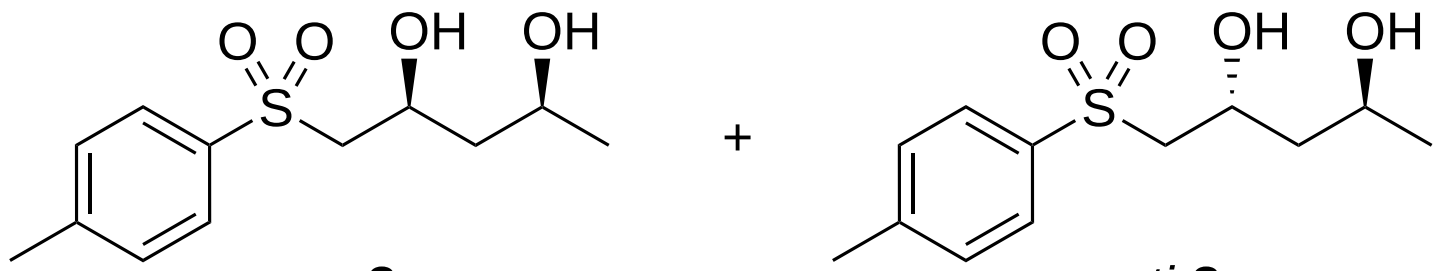

rac-syn-**3m** + *rac-anti*-**3m**

Preparation based on a literature procedure.[31] Methoxydiethylborane (0.5 ml, 0.5 mmol, 1 M in THF, 1.1 equiv.) was added to a solution of 4-hydroxy-1-tosylpentan-2-one (113 mg, 0.44 mmol, 1 equiv.) in THF:MeOH (4:1, 5 ml) at – 70 °C. After being stirred for 35 minutes $NaBH_4$ (19 mg, 0.52 mmol, 1.2 equiv.) was added and the reaction mixture stirred for 6 hours at – 70 °C. The reaction mixture was quenched with saturated NH_4Cl and the layers separated then the aqueous phase was washed with EtOAc. The combined organic layers were washed with saturated $NaHCO_3$ and brine then dried over $MgSO_4$ before the solvent was removed *in vacuo*. The crude product was purified *via* silica gel chromatography (20 – 25% EtOAc in Pet. Ether) to give (*syn*)-(2*R*,4*S*)-1-tosylpentane-2,4-diol and (*anti*)-(2*R*,4*S*)-1-tosylpentane-2,4-diol as a 7:1 mixture (21 mg, 0.08 mmol, 18%) and as a colourless oil.

(*syn*)-(2*R*,4*S*)-1-tosylpentane-2,4-diol

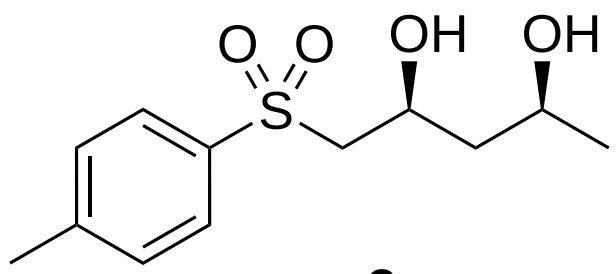

rac-syn-**3m**

^{1}H NMR (700 MHz, $CDCl_3$) δ 7.81 (d, *J* = 7.7 Hz, 2H), 7.38 (d, *J* = 7.9 Hz, 2H), 4.41 (t, *J* = 9.4 Hz, 1H), 4.09 – 4.04 (m, 1H), 3.29 (dd, *J* = 14.4, 9.0 Hz, 1H), 3.15 (d, *J* = 14.4 Hz, 1H), 2.46 (s, 3H), 1.68 – 1.62 (m, 1H), 1.60 – 1.54 (m, 1H), 1.18 (d, *J* = 6.2 Hz, 3H) ppm.

^{13}C NMR (176 MHz, $CDCl_3$) δ 145.4, 136.4, 130.3, 128.1, 67.9, 66.9, 62.6, 44.1, 23.9, 21.8 ppm.

HRMS [M+H]$^+$ m/z calc'd for $[C_{12}H_{19}O_4S]^+$ expect 259.1004 found 259.1014

7-hydroxyundecan-5-one (*rac*-2c)

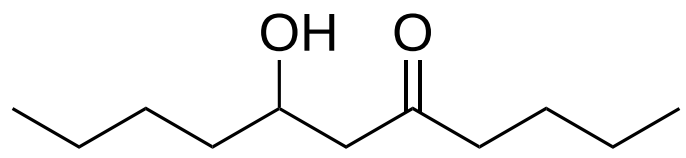

rac-**2c**

Prepared according to **General Procedure 2** using diisopropylamine (2.3 ml, 16.2 mmol), *n*-butyllithium (10.3 ml, 16.5 mmol, 1.6 M in THF), 2-hexanone (1.9 ml, 15.0 mmol) and pentanal (1.6 ml, 15.0 mmol). The crude product was purified *via* column chromatography (0 – 5% Acetone in Pet. Ether) to give the title compound as a colourless oil (763 mg, 4.10 mmol, 27%).

^{1}H NMR (700 MHz, $CDCl_3$) δ 4.04 – 3.98 (m, 1H), 3.06 (br s, 1H), 2.58 (dd, *J* = 17.5, 2.6 Hz, 1H), 2.48 (dd, *J* = 17.5, 9.2 Hz, 1H), 2.41 (t, *J* = 7.5 Hz, 2H), 1.55 (app p, *J* = 7.5 Hz, 2H), 1.52 – 1.46 (m, 1H), 1.42 – 1.35 (m, 2H), 1.35 – 1.26 (m, 5H), 0.91 – 0.87 (m, 6H) ppm.

^{13}C NMR (176 MHz, $CDCl_3$) δ 212.8, 67.8, 49.1, 43.5, 36.3, 27.8, 25.8, 22.7, 22.4, 14.1, 13.9 ppm.

The data is in good agreement with the literature.[6]

5-hydroxy-1,7-diphenylheptan-3-one (*rac*-2d)

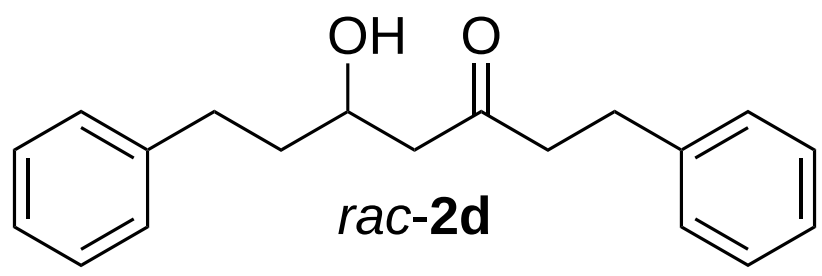

rac-**2d**

Prepared according to **General Procedure 2** using diisopropylamine (0.76 ml, 5.4 mmol), *n*-butyllithium (2.0 ml, 5.5 mmol, 1.6 M in THF), 4-phenylbutan-2-one (0.75 ml, 5.0 mmol) and 3-phenylpropanal (0.66 ml, 5.0 mmol). The crude product was purified *via* column chromatography (0 – 5% Acetone in Pet. Ether) to give the title compound as a white solid (427 mg, 1.51 mmol, 30%).

^{1}H NMR (500 MHz, $CDCl_3$) δ 7.29 (app t, *J* = 7.6 Hz, 4H), 7.22 – 7.15 (m, 6H), 4.05 (sep, *J* = 4.1 Hz, 1H), 2.90 (t, *J* = 7.5 Hz, 2H), 2.80 (ddd, *J* = 14.1, 9.4, 5.1 Hz, 1H), 2.75 (t, *J* = 7.6 Hz, 2H), 2.67 (ddd, *J* = 13.8, 9.5, 6.9 Hz, 1H), 2.60 – 2.50 (m, 2H), 1.81 (dddd, *J* = 13.8, 9.2, 9.0, 4.9 Hz, 1H), 1.67 (dddd, *J* = 13.8 9.7, 6.9, 4.1 Hz, 1H) ppm.

^{13}C NMR (126 MHz, $CDCl_3$) δ 211.2, 141.9, 140.8, 128.7, 128.6, 128.5, 128.4, 126.4, 126.0, 67.0, 49.4, 45.1, 38.1, 31.9, 29.6 ppm.

Data is in good agreement with the literature.[32]

6-hydroxy-2,8-dimethylnonan-4-one (*rac*-2e)

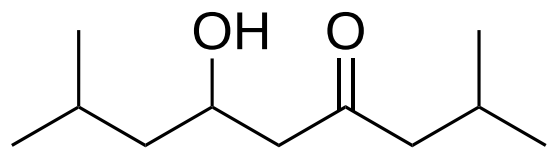

rac-**2e**

Prepared according to **General Procedure 2** using diisopropylamine (0.76 ml, 5.4 mmol), *n*-butyllithium (2.0 ml, 5.5 mmol, 1.6 M in THF), 4-methyl-2-pentanone (0.62 ml, 5.0 mmol) and 3-methylbutanal (0.54 ml, 5.0 mmol). The crude product was purified *via* column chromatography (0 – 5% Acetone in Pet. Ether) to give the title compound as a colourless oil (110 mg, 0.59 mmol, 12%) with 14% (*E*)-2,8-dimethylnon-5-en-4-one impurity.

^{1}H NMR (700 MHz, $CDCl_3$) δ 4.12 – 4.07 (m, 1H), 2.53 (dd, *J* = 17.6, 2.8 Hz, 1H), 2.45 (dd, *J* = 17.6, 9.1 Hz, 1H), 2.28 (d, *J* = 6.9 Hz, 2H), 2.12 (sep, *J* = 6.8 Hz, 1H), 1.82 – 1.72 (m, 1H), 1.44 (ddd, *J* = 13.9, 8.8, 5.3 Hz, 1H), 1.11 (ddd, *J* = 13.6, 8.7, 4.5 Hz, 1H), 0.90 – 0.88 (m, 12H) ppm.

^{13}C NMR (176 MHz, $CDCl_3$) δ 212.5, 65.8, 52.7, 50.1, 45.6, 24.7, 24.5, 23.4, 22.7, 22.6, 22.1 ppm.

The data is in good agreement with the literature.[6]

undecane-5,7-dione (*int$_1$*-1d)

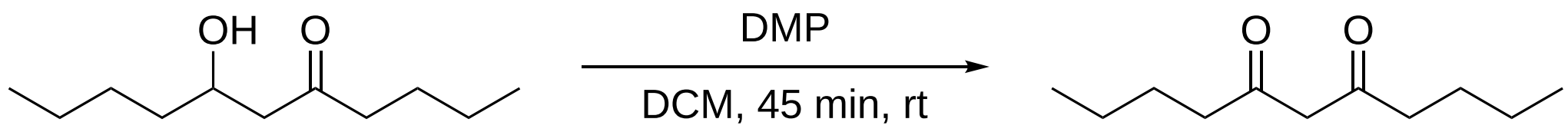

int$_1$-**1d**-d4

Preparation based on a literature procedure.[5] 7-hydroxyundecan-5-one (652 mg, 3.5 mmol, 1 equiv.) was dissolved in DCM (7 ml) in a flask wrapped in foil to shield from light. Dess-Martin periodinane (1.78 g, 4.2 mmol, 1.2 equiv.) in DCM (14 ml) was added to the reaction mixture which was stirred for 30 minutes while shielded. The reaction mixture was then diluted with ether (33 ml) and stirred for a further 15 minutes. An aqueous solution of $Na_2S_2O_3$ (2.79 g) in saturated bicarbonate (13 ml) was added and the reaction mixture stirred for a further 15 minutes. The flask was then uncovered, and the phases separated. The organic layer was washed with saturated sodium bicarbonate (26 ml), brine (26 ml) and dried over $MgSO_4$. The solvent was removed *in vacuo* and the crude product purified *via* silica gel chromatography (50 – 90% DCM in Hexane) to give undecane-5,7-dione and its enol tautomer as an orange oil (375 mg, 2.03 mmol, 58%).

^{1}H NMR (700 MHz, $CDCl_3$) δ 5.48 (s, 1H), 3.55 (s, 0.44H), 2.50 (t, *J* = 7.4 Hz, 0.91H), 2.27 (t, *J* = 5.4 Hz, 4H), 1.62 – 1.53 (m, 5.65H), 1.39 – 1.27 (m, 5.31H), 0.92 (t, *J* = 7.4 Hz, 6H), 0.90 (t, *J* = 7.4 Hz, 1.84H) ppm.

^{13}C NMR (176 MHz, $CDCl_3$) δ 204.6, 194.7, 99.2, 57.3, 43.7, 38.3, 28.0, 25.6, 22.5, 22.3, 14.0 ppm.

The data is in good agreement with the literature.[5]

undecane-5,6,6,7-d_4-5,7-diol (1d)

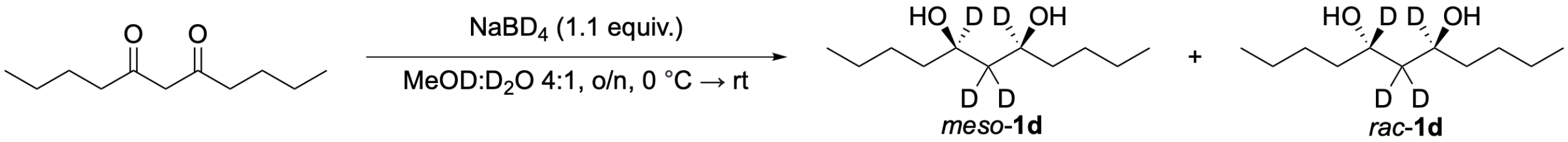

Preparation based on a literature procedure.[33] undecane-5,7-dione (276mg, 1.5 mmol, 1 equiv.) was dissolved in $CD_3OD:D_2O$ (4:1, 5.4 ml) and cooled to 0 °C. $NaBD_4$ (69 mg, 1.65 mmol, 1.1 equiv.) was then added slowly at 0 °C after which the reaction mixture was stirred overnight at room temperature. The reaction mixture was quenched by the addition of 2M HCl and stirred for 30 minutes. 2M NaOH was then added until the pH was greater than 10. The two layers were then separated, and the aqueous layer washed with DCM. The organic layers were combined and dried over $MgSO_4$. The solvent was removed *in vacuo* and the crude product purified *via* silica gel chromatography (5% Acetone in Pet. Ether) to give (*syn*)-undecane-5,6,6,7-d_4-5,7-diol (111 mg, 0.59 mmol, 39%, 99% (α to OH) and 93% (β to OH) D incorporation) as a colourless oil, (*anti*)-undecane-5,6,6,7-d_4-5,7-diol (109 mg, 0.57 mmol, 38%, 99% (α to OH) and 86% (β to OH) D incorporation) as a white solid and mixed fractions (37 mg, 0.19 mmol, 13%). Deuterium incorporation was determined from the ^{1}H NMR of the isolated material, with the assumption that no deuteration would occur at both terminal CH_3 groups.

(*syn*)-undecane-5,6,6,7-d_4-5,7-diol (*meso*-1d)

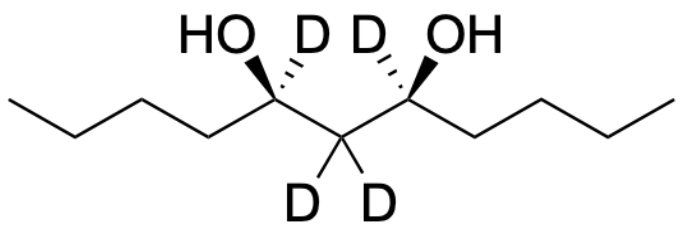

meso-**1d**

^{1}H NMR (700 MHz, $CDCl_3$) δ 3.86 – 3.83 (m, 0.02H), 2.39 (br s, 2H), 1.51 – 1.41 (m, 4H), 1.41 – 1.27 (m, 8H), 0.91 (t, *J* = 7.1 Hz, 6H) ppm.

^{13}C NMR (176 MHz, $CDCl_3$) δ 72.8 (t, *J* = 21.5 Hz), 42.6 – 41.9 (m), 38.0, 27.6, 22.9, 14.2 ppm.

^{2}H NMR (700 MHz, $CDCl_3$) δ 3.85 (s, 2D), 1.58 (s, 0.89D), 1.44 (s, 0.98D) ppm.

HRMS [M+Na]$^+$ m/z calc'd for $[C_{11}H_{20}D_4O_2Na]^+$ expect 215.1920 found 215.1926

(*anti*)-undecane-5,6,6,7-d_4-5,7-diol (*rac*-1d)

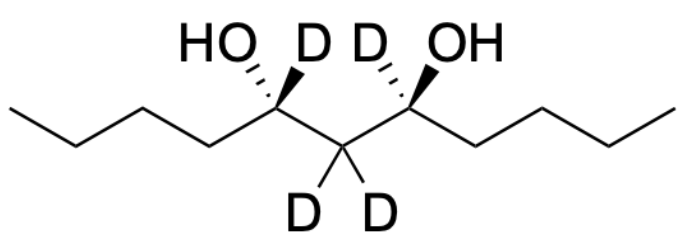

rac-**1d**

^{1}H NMR (700 MHz, $CDCl_3$) δ 3.95 – 3.92 (m, 0.01H), 1.68 (br s, 2H), 1.56 – 1.50 (m, 2H), 1.48 – 1.43 (m, 2H), 1.43 – 1.26 (m, 8H), 0.91 (t, *J* = 7.1 Hz, 6H) ppm.

^{13}C NMR (176 MHz, $CDCl_3$) δ 69.1 (t, *J* = 21.4 Hz), 37.2, 28.1, 22.9, 14.2 ppm.

^{2}H NMR (700 MHz, $CDCl_3$) δ 3.94 (s, 2D), 1.59 (s, 1.71D) ppm.

HRMS [M+Na]$^+$ m/z calc'd for $[C_{11}H_{20}D_4O_2Na]^+$ expect 215.1920 found 215.1919

(*anti*)-1-Phenylpentane-2,4-diol (*rac*-*anti*-3a)

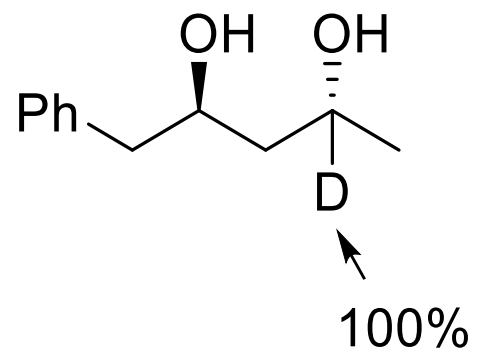

To a vial was added 4-hydroxy-5-phenylpentan-2-one (356 mg, 2.0 mmol, 1.0 equiv.) and MeOH-d4 (7.1 mL). The mixture was cooled to 0 °C and $NaBD_4$ (92.1 mg, 2.2 mmol, 1.1 equiv.) was added upon which point the vial was lightly covered (hydrogen evolution) and the mixture stirred at room temperature for 3 h. Then, water (10 mL) was added and the mixture extracted with DCM (2 x 20 mL). The combined organic fractions were washed with brine and dried over $MgSO_4$ and the solvent removed under reduced pressure. MeOH was added (10 mL). The solvent was removed under reduced pressure again and the addition of MeOH was repeated two more times. The crude material was purified by flash column chromatography on silica gel (15% acetone in hexane) to give the product (61.9 mg, 34%, 100% D incorporation) as a colourless solid.

^{1}H NMR (700 MHz, $CDCl_3$) δ 7.34 – 7.31 (m, 2H), 7.26 – 7.21 (m, 3H), 4.21 – 4.16 (m, 1H), 2.84 – 2.76 (m, 2H), 2.22 (br. s, 2H), 1.67 (d, J = 6.0 Hz, 2H), 1.23 (s, 3H) ppm.

^{13}C NMR (176 MHz, $CDCl_3$) δ 138.4, 129.5, 128.8, 126.8, 70.3, 65.2 (t, J = 22.0 Hz), 44.2, 43.6, 23.6 ppm.

^{2}H NMR (107 MHz, $CDCl_3$) δ 4.20 (s) ppm.

HRMS [M+H]$^+$ m/z calc'd for $[C_{11}H_{15}DO_2Na]^+$ expect 204.1111; found 204.1118.

(*anti*)-1-Phenylpentane-2,4-diol (*rac*-*anti*-3a)

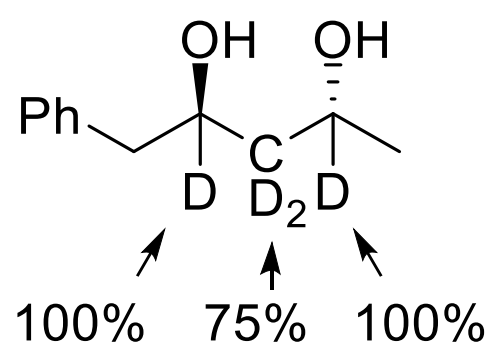

To a vial was added 1-phenylpentane-2,4-dione (250 mg, 1.4 mmol, 1.0 equiv.) and MeOH-d4 (7.1 mL). The mixture was cooled to 0 °C and $NaBD_4$ (130.6 mg, 3.1 mmol, 2.2 equiv.) was added upon which the vial was lightly covered (hydrogen evolution) and the mixture stirred at room temperature for 3 h. Then, water (10 mL) was added and the mixture extracted with DCM (2 x 20 mL). The combined organic fractions were washed with brine and dried over $MgSO_4$ and the solvent removed under reduced pressure. MeOH was added (10 mL). The solvent was removed under reduced pressure again and the addition of MeOH was repeated two more times. The crude material was purified by flash column chromatography on silica gel (15% acetone in hexane) to give the product (85.4 mg, 33%, 100% (α to OH) and 75% (β to OH) D incorporation) as a colourless solid.

^{1}H NMR (700 MHz, $CDCl_3$) δ 7.34 – 7.30 (m, 2H), 7.26 – 7.21 (m, 3H), 2.83 – 2.75 (m, 2H), 2.11 (br. s, 2H), 1.67 – 1.63 (m, 0.5H), 1.23 (s, 3H) ppm.

^{13}C NMR (176 MHz, $CDCl_3$) δ 138.4, 129.5, 128.8, 126.8, 70.1 – 69.6 (m), 65.3 – 64.9 (m), 44.1 – 44.0 (m), 43.5 – 43.0 (m), 23.5 ppm.

^{2}**H NMR** (107 MHz, $CDCl_3$) δ 4.19 (s, 2D), 1.65 (s, 1.5D) ppm.

HRMS [M+H]$^+$ m/z calc'd for $[C_{11}H_{12}D_4O_2Na]^+$ expect 207.1299; found 207.1304.

(*anti*)-6-Phenylhexane-2,4-diol (*rac-anti*-3d)

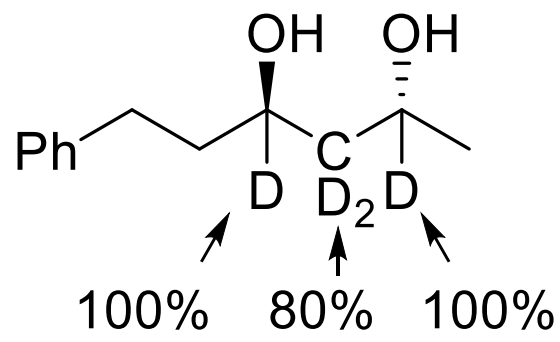

To a vial was added 6-Phenyl-2,4-hexanedione (380 mg, 2 mmol, 1.0 equiv.) (prepared by a reported procedure)[17] and MeOH-d4 (10 mL). The mixture was cooled to 0 °C and $NaBD_4$ (184 mg, 4.4 mmol, 2.2 equiv.) was added upon which the vial was lightly covered (hydrogen evolution) and the mixture stirred at room temperature for 3 h. Then, water (10 mL) was added and the mixture extracted with DCM (2 x 20 mL). The combined organic fractions were washed with brine and dried over $MgSO_4$ and the solvent removed under reduced pressure. MeOH was added (10 mL). The solvent was removed under reduced pressure again and the addition of MeOH was repeated two more times. The crude material was purified by flash column chromatography on silica gel (12% acetone in hexane) to give the product (182.1 mg, 46%, 100% (α to OH) and 80% (β to OH) D incorporation) as a colourless solid.

^{1}**H NMR** (700 MHz, $CDCl_3$) δ 7.31 – 7.27 (m, 2H), 7.22 – 7.17 (m, 3H), 2.83 – 2.76 (m, 1H), 2.72 – 2.64 (m, 1H), 2.47 (br. s, 1H), 2.27 (br. s, 1H), 1.91 – 1.82 (m, 1H), 1.81 – 1.74 (m, 1H), 1.69 – 1.60 (m, 0.4H), 1.23 (s, 3H) ppm.

^{13}**C NMR** (176 MHz, $CDCl_3$) δ 142.1, 128.59, 128.54, 126.0, 68.7 – 68.3 (m), 65.5 – 65.1 (m), 44.1 – 43.4 (m), 39.1 – 39.0 (m) 32.3, 23.7 – 23.6 (m) ppm.

^{2}**H NMR** (107 MHz, $CDCl_3$) δ 4.18 (s, 1D), 3.98 (s, 1D), 1.62 (s, 1.6D) ppm.

HRMS [M+H]$^+$ m/z calc'd for $[C_{12}H_{14}D_4O_2Na]^+$ expect 221.1456; found 221.1462.

Synthesis and Characterization of Racemic 1,2-Diols

***syn*-butane-2,3-diol (4a-rac)**

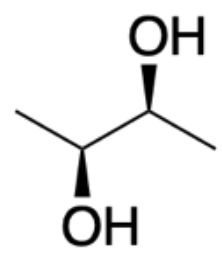

4a-rac

A solution of commercially available (2*S*,3*S*)-butane-2,3-diol (100 mg) in acetone (5 mL) and a solution of commercially available (2*R*,3*R*)-butane-2,3-diol (100 mg) in acetone (5 mL) were prepared. The solutions were combined in a separate vial and concentrated *in vacuo* to afford racemic **4a-rac**. Racemate formation was confirmed by equal signal integration in the chiral SFC analysis of **4a-rac-Bz**.

^{1}H NMR (700 MHz, $CDCl_3$) δ 3.56 – 3.48 (m, 2H), 2.10 (br. s, 2H), 1.18 (d, *J* = 6.0 Hz, 6H) ppm.

^{13}C NMR (176 MHz, $CDCl_3$) δ 72.7, 19.5 ppm.

The data is in good agreement with the literature.[34]

***syn*-hexane-3,4-diol (4b-rac)**

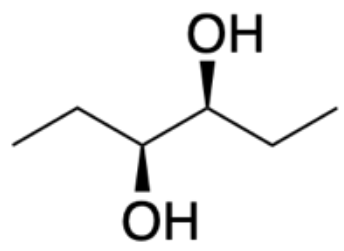

4b-rac

4b-rac was synthesised according to literature procedure.[35]

***syn*-octane-4,5-diol (4c-rac)**

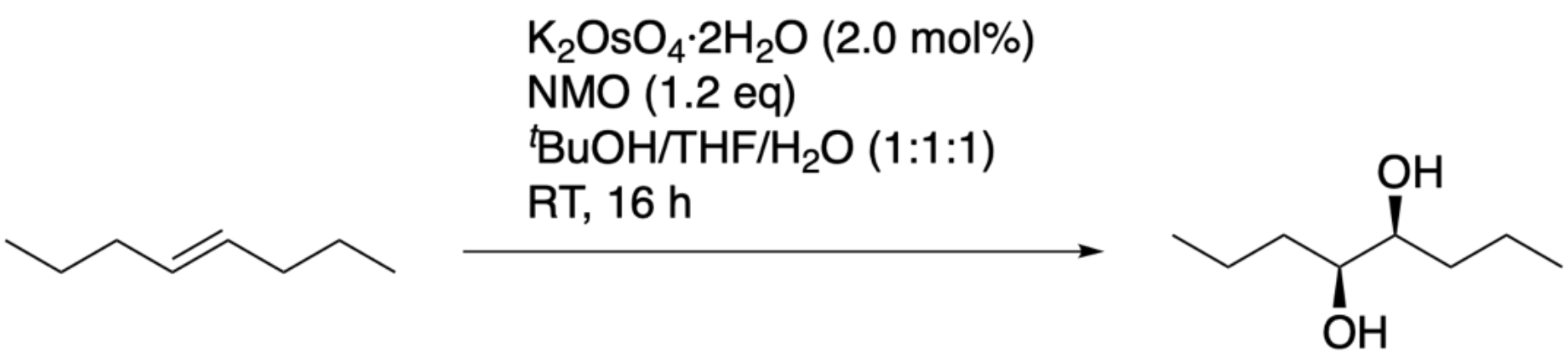

4c-rac

4c-rac was prepared according to **General Procedure 4** using *trans*-4-octene (0.79 mL, 5.03 mmol), $K_2OsO_4.2H_2O$ (36.8 mg, 0.10 mmol) and NMO (1.20 mL, 6.00 mmol) stirred for 16 h. Purification by silica gel column chromatography (elution with 15% acetone in petroleum ether) afforded **4c-rac** as a pale yellow oil (626 mg, 4.28 mmol, 85% yield).

^{1}H NMR (500 MHz, $CDCl_3$) δ 3.45 – 3.37 (m, 2H), 2.12 (d, *J* = 4.4 Hz, 2H), 1.56 – 1.33 (m, 8H), 0.94 (t, *J* = 7.0 Hz, 6H) ppm.

^{13}C NMR (126 MHz, $CDCl_3$) δ 74.4, 35.9, 19.0, 14.2 ppm.

The data is in good agreement with the literature.[36]

syn-decane-5,6-diol (4d-rac)

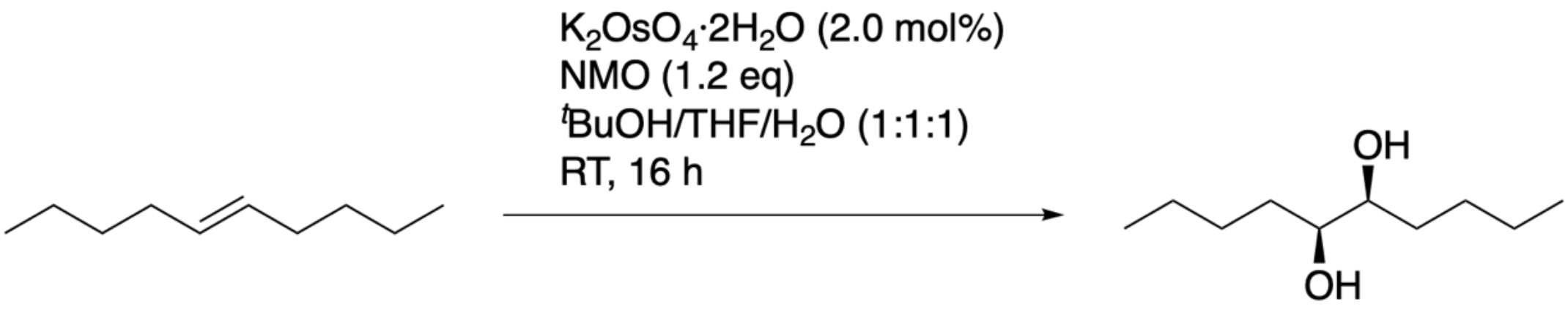

4d-rac

4d-rac was prepared according to **General Procedure 4** using *trans*-5-decene (0.95 mL, 5.01 mmol), $K_2OsO_4.2H_2O$ (36.8 mg, 0.10 mmol) and NMO (1.20 mL, 6.00 mmol) stirred for 16 h. Purification by silica gel column chromatography (elution with 10% acetone in petroleum ether) afforded **4d-rac** as a white solid (711 mg, 4.08 mmol, 81% yield).

^{1}H NMR (700 MHz, $CDCl_3$) δ 3.44 – 3.37 (m, 2H), 2.00 (br. s, 2H), 1.56 – 1.49 (m, 2H), 1.49 – 1.40 (m, 4H), 1.40 – 1.29 (m, 6H), 0.92 (t, J = 7.1 Hz, 6H) ppm.

^{13}C NMR (176 MHz, $CDCl_3$) δ 74.7, 33.5, 28.0, 22.9, 14.2 ppm.

The data is in good agreement with the literature.[37]

Di-*tert*-butyl *syn*-4,5-dihydroxyoctanedioate (4e-rac)

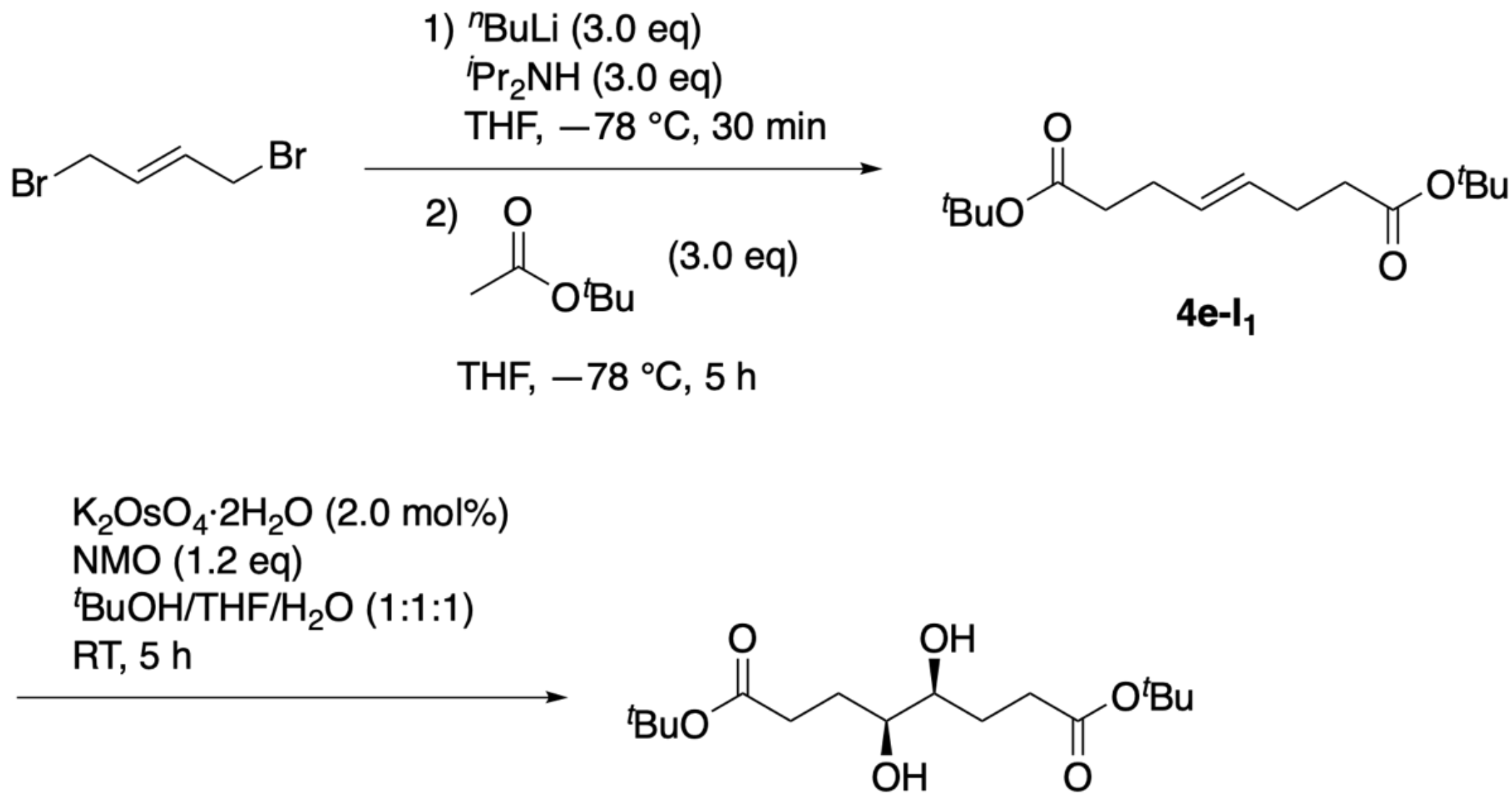

4e-rac

Crude **4e-I₁** was prepared according to literature procedure.[38] To a stirred solution of diisopropylamine (3.14 mL, 22.5 mmol) in dry THF (32 mL) in an oven-dried two-necked RBF under nitrogen atmosphere at –78 °C was added *n*-butyllithium (1.6 M in hexanes, 14.0 mL, 22.4 mmol) dropwise. The mixture was stirred for 15 min and *tert*-butyl acetate (3.01 mL, 22.5 mmol) was added dropwise. The solution was stirred at –78 °C for 30 min. In a separate oven-dried flask under nitrogen atmosphere, a solution of

(E)-1,4-dibromobutene (1.60 g, 7.48 mmol) in dry THF (17 mL) was prepared and added dropwise to the reaction mixture. The mixture was stirred for a further 5 h at –78 °C, quenched with saturated aqueous ammonium chloride solution and extracted with ethyl acetate. The combined organic extracts were dried over $MgSO_4$, filtered, and the solvent removed *in vacuo* to afford crude **4e-I$_1$** (2.28 g). Crude **4e-I$_1$** (1.10 g) was brought forward without additional purification to **General Procedure 4** using $K_2OsO_4.2H_2O$ (28.4 mg, 0.08 mmol) and NMO (0.93 mL, 4.49 mmol) stirred for 5 h. Purification by silica gel column chromatography (elution with 20% acetone in petroleum ether followed by a second purification eluting with 25% ethyl acetate in petroleum ether) afforded **4e-rac** as a colourless oil that slowly forms an off-white waxy solid (473 mg, 1.49 mmol, 42% yield over two steps accounting for only 1.10 g out of 2.28 g of crude **4e-I$_1$** being brought forward).

^{1}H NMR (500 MHz, $CDCl_3$) δ 3.45 – 3.38 (m, 2H), 2.87 (br. d, J = 5.1 Hz, 2H), 2.42 (t, J = 7.0 Hz, 4H), 1.86 – 1.70 (m, 4H), 1.44 (s, 18H) ppm.

^{13}C NMR (126 MHz, $CDCl_3$) δ 174.0, 80.9, 74.0, 32.1, 28.6, 28.2 ppm.

The data is in good agreement with the literature.[38]

***syn*-1,4-diphenylbutane-2,3-diol (4f-rac)**

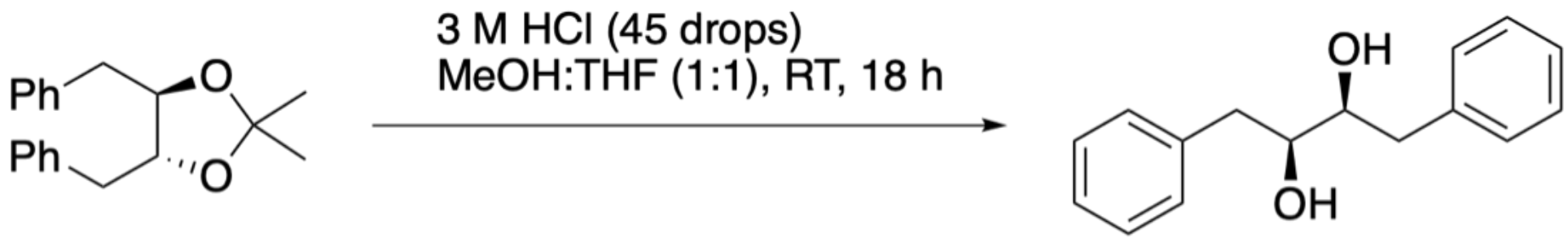

S-4f

4f-rac

To a solution of **S-4f** (280 mg, 0.99 mmol; prepared according to procedure previously reported by the group[1]) in MeOH (6 mL) and THF (6 mL) was added 3 M HCl (45 drops by pipette) and the reaction mixture stirred at RT for 18 h. The mixture was concentrated and redissolved in DCM, dried over $MgSO_4$, filtered and concentrated *in vacuo*. Purification by silica gel column chromatography (elution with 20% acetone in petroleum ether) afforded **4f-rac** as a white solid (208 mg, 0.86 mmol, 87% yield).

^{1}H NMR (700 MHz, $CDCl_3$) δ 7.31 (t, J = 7.3 Hz, 4H), 7.26 – 7.21 (m, 6H), 3.79 – 3.74 (m, 2H), 2.93 (dd, J = 13.7, 4.4 Hz, 2H), 2.85 (dd, J = 13.7, 8.3 Hz, 2H), 2.01 (d, J = 3.9 Hz, 2H) ppm.

^{13}C NMR (176 MHz, $CDCl_3$) δ 138.2, 129.5, 128.8, 126.7, 74.2, 40.5 ppm.

The data is in good agreement with the literature.[39]

syn-1,8-diphenyloctane-4,5-diol (4g-rac)

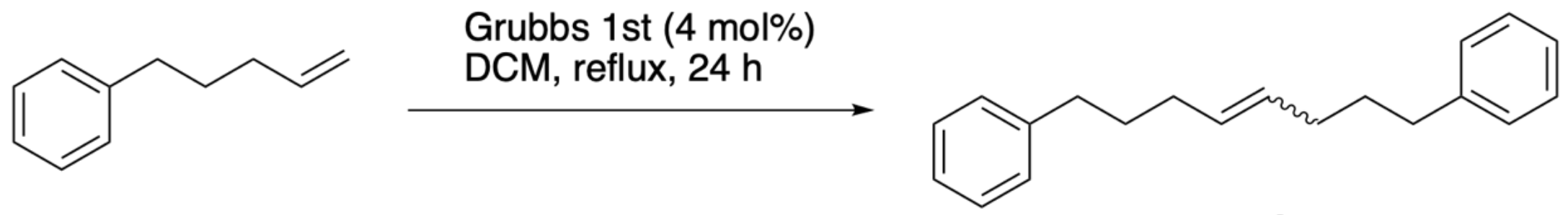

S-4g **4g-I$_1$**

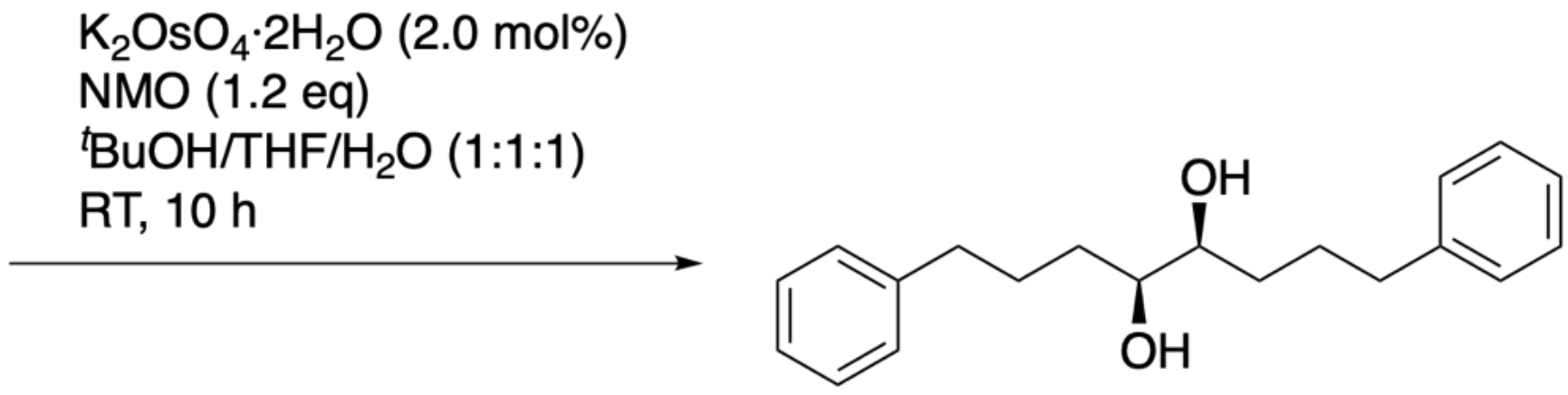

4g-rac

A solution of **S-4g** (782 mg, 5.35 mmol; prepared according to literature procedure[40]) in dry DCM (20 mL) in an oven-dried two-necked RBF was degassed and kept under nitrogen pressure. Grubbs 1st generation catalyst (176 mg, 0.21 mmol) was added and the reaction mixture stirred at 40 °C for 24 h. The mixture was cooled to RT and concentrated *in vacuo* to afford crude mixture. Partial purification by silica gel column chromatography (elution with 0 to 5% acetone in hexane) afforded **4g-I$_1$** containing a small amount of impurity as a colourless oil (534 mg, *approx.* 2.02 mmol, *approx.* 38% yield). Isolated **4g-I$_1$** contained a mixture of *E* and *Z* alkenes with discernible signals in the ^{13}C NMR spectrum, indicating that the *E* isomer is the major isomer. This is consistent with the predicted selectivity of the Grubbs metathesis reaction. Some signals from the *Z* isomer in the ^{13}C NMR spectrum are not observed due to the low concentration of this isomer.

Combined signals for both isomers are denoted (*E + Z*) and discernable signals are denoted (*E*) or (*Z*) for each respective isomer in the ^{13}C NMR spectrum. No discernable signals were observed in ^{1}H NMR.

^{1}H NMR (400 MHz, $CDCl_3$) δ 7.31 – 7.26 (m, 4H) (overlaps with $CHCl_3$ signal), 7.21 – 7.15 (m, 6H), 5.49 – 5.40 (m, 2H), 2.62 (t, *J* = 7.7 Hz, 4H), 2.10 – 1.99 (m, 4H), 1.74 – 1.63 (m, 4H) ppm.

^{13}C NMR (101 MHz, $CDCl_3$) δ 142.8 (*E*), 142.7 (*Z*), 130.5 (*E*), 130.0 (*Z*), 128.6 (2C: *E*, *Z*), 128.4 (*E*+*Z*), 125.8 (*E*), 35.5 (*E*+*Z*), 32.3 (*E*), 31.5 (*E*), 27.0 (*Z*) ppm.

The data is in good agreement with the literature for the individual *E* and *Z* isomers.[41]

4g-rac was prepared according to **General Procedure 4** using **4g-I$_1$** (520 mg, *approx.* 1.97 mmol), $K_2OsO_4.2H_2O$ (14.5 mg, 0.04 mmol) and NMO (0.47 mL, 2.27 mmol) stirred for 10 h. Purification by silica gel column chromatography (elution with 10% acetone in petroleum ether) afforded **4g-rac** as an off-white waxy solid together with a small amount of inseparable impurity (287 mg, 0.96 mmol, *approx.* 49% yield). 18mg of the *meso* diol and 184 mg of a mixture of diols (2:1 *syn*:*meso*) were additionally isolated. Our group has previously reported the synthesis of the *meso* diol.[1,2] By deduction, the other isomer isolated was the *syn* isomer. The ^{1}HNMR and ^{13}CNMR signals of **4g-rac** are also consistent with discernible signals of (*S*,*S*)-diphenyloctane-4,5-diol, previously reported by the group as a mixture

containing the *meso* diol.[1] The relative yields are also consistent with the Grubbs metathesis step, and two signals of equal integration are observed in the chiral SFC analysis of **4g-rac**.

^{1}H NMR (700 MHz, $CDCl_3$) δ 7.28 (t, *J* = 7.6 Hz, 4H), 7.21 – 7.16 (m, 6H), 3.47 – 3.39 (m, 2H), 2.70 – 2.58 (m, 4H), 1.97 (br. s, 2H), 1.85 – 1.77 (m, 2H), 1.73 – 1.65 (m, 2H), 1.56 – 1.50 (m, 2H), 1.50 – 1.43 (m, 2H) ppm.

^{13}C NMR (176 MHz, $CDCl_3$) δ 142.3, 128.5 (2C), 125.9, 74.4, 35.9, 33.3, 27.5 ppm.

HRMS $[M+Na]^+$ m/z calc'd for $[C_{20}H_{26}O_2Na]^+$ expect 321.1830; found 321.1832.

(*Z*)- + (*E*)-Pent-3-en-1-ylbenzene (*int$_1$*-4h)

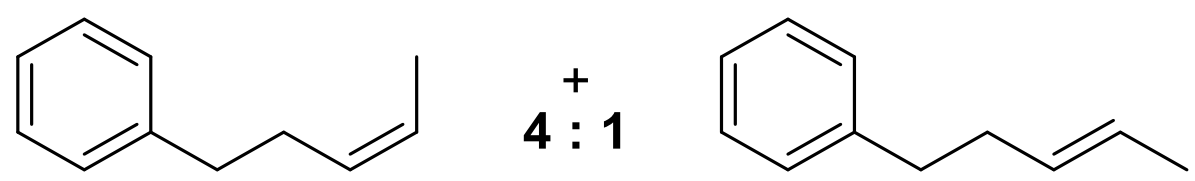

This procedure was adapted from literature.[42] To a heat gun-dried flask was added Ethyl triphenylphosphonium bromide (7.43 g, 20 mmol, 1.0 equiv.) and dry THF (80 mL). The mixture was cooled to 0 °C and nBuLi (1.6 M in hexanes, 12.5 mL, 20 mmol, 1.0 equiv.) was added slowly. The mixture was stirred at 0 °C for 30 min and then cooled to –78 °C. 3-Phenylpropionaldehyde (2.7 mL, 20 mmol, 1.0 equiv.) was added and the mixture stirred at room temperature for 5 days. Water (20 mL) and hexane (100 mL) was added and the phases separated. The organic fraction was dried over $MgSO_4$ and the solvent removed under reduced pressure. The crude material was purified by flash column chromatography on silica gel (hexane) to give the product (1.476 g, 51%, *Z*:*E* = 4:1) as a colourless oil.

Discernible peaks are denoted *Z* and *E* respectively.

^{1}H NMR (400 MHz, $CDCl_3$) δ 7.31 – 7.26 (m, 2H), 7.23 – 7.15 (m, 3H), 5.53 – 5.38 (m, 2H), 2.67 (t, *J* = 7.4 Hz, 2H), 2.40 – 2.33 (m, 1.6H^Z), 2.33 – 2.26 (m, 0.4H^E), 1.66 – 1.63 (m, 0.6H^E), 1.58 – 1.52 (m, 2.4H^E) ppm.

The spectral data are in agreement with the one in literature. (*Z*[43], *E*[44])

5-Phenylpentane-2,3-diol (4h)

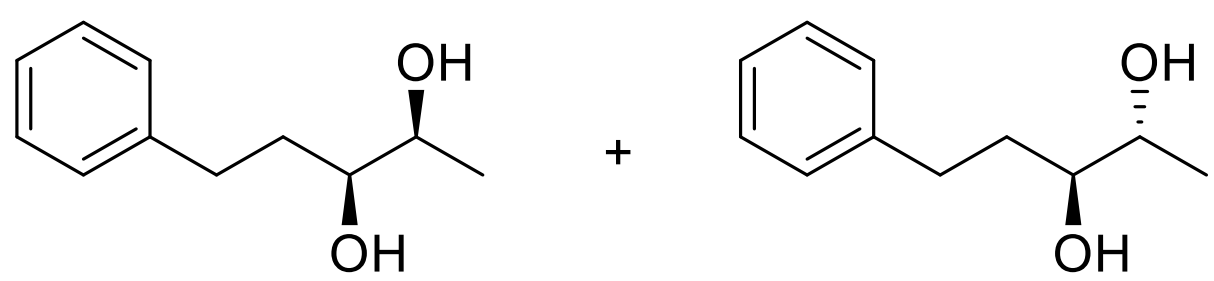

The compound was synthesised according to **General Procedure 5** using (Z)- + (E)-Pent-3-en-1-ylbenzene (1.46 g, 10 mmol), separated with 20% acetone in hexane, giving (*syn*)-5-Phenylpentane-2,3-diol (112.7 mg, 6%) as a colourless oil and (*anti*)-5-Phenylpentane-2,3-diol (482.4 mg, 27%) as a colourless solid.

(*anti*)-5-Phenylpentane-2,3-diol (*rac-anti*-4h)

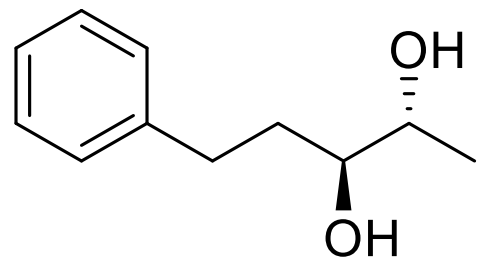

^{1}H NMR (500 MHz, $CDCl_3$) δ 7.32 – 7.26 (m, 2H), 7.24 – 7.17 (m, 3H), 3.83 – 3.77 (m, 1H), 3.67 – 3.59 (m, 1H), 2.93 – 2.82 (m, 1H), 2.72 – 2.63 (m, 1H), 1.80 (br. s, 2H), 1.76 – 1.70 (m, 2H), 1.15 (d, *J* = 6.5 Hz, 3H) ppm.

^{13}C NMR (126 MHz, $CDCl_3$) δ 142.0, 128.60, 128.60, 126.1, 74.3, 70.6, 33.5, 32.4, 17.0 ppm.

HRMS [M+H]$^+$ m/z calc'd for $[C_{11}H_{16}O_2Na]^+$ expect 203.1048; found 203.1056.

(*syn*)-5-Phenylpentane-2,3-diol (*rac-syn*-4h)

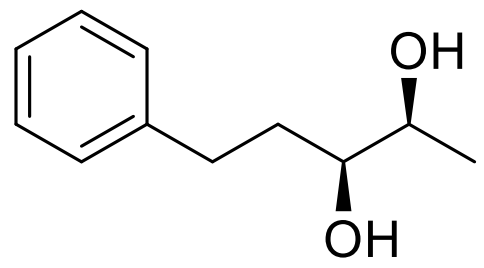

^{1}H NMR (500 MHz, $CDCl_3$) δ 7.32 – 7.27 (m, 2H), 7.23 – 7.17 (m, 3H), 3.67 – 3.60 (m, 1H), 3.40 – 3.34 (m, 1H), 2.91 – 2.81 (m, 1H), 1.76 – 1.66 (m, 1H), 1.94 – 1.70 (m, 4H), 1.19 (d, *J* = 6.3 Hz, 3H) ppm.

^{13}C NMR (126 MHz, $CDCl_3$) δ 142.0, 128.61, 128.58, 126.1, 75.6, 71.1, 35.3, 32.1, 19.7 ppm.

HRMS [M+H]$^+$ m/z calc'd for $[C_{11}H_{16}O_2Na]^+$ expect 203.1048; found 203.1057.

(*Z*)- + (*E*)-But-2-en-1-ylbenzene (*int$_1$*-4i)

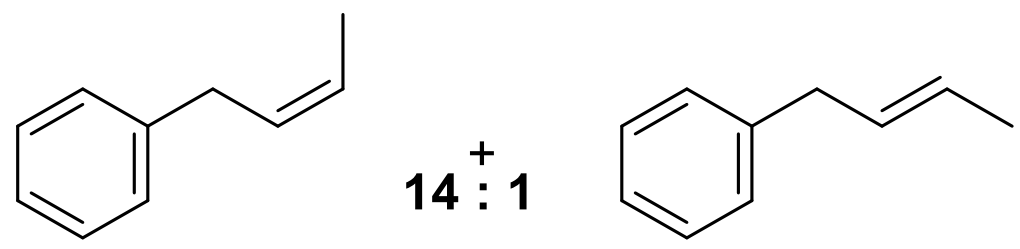

This procedure was adapted from literature.[42] To a heat gun-dried flask was added Ethyl triphenylphosphonium bromide (7.43 g, 20 mmol, 1.0 equiv.) and dry THF (80 mL). The mixture was cooled to 0 °C and nBuLi (1.6 M in hexanes, 12.5 mL, 20 mmol, 1.0 equiv.) was added slowly. The mixture was stirred at 0 °C for 30 min and then cooled to –78 °C. Phenylacetaldehyde (3.60 g, 30 mmol, 1.5 equiv.) was added and the mixture stirred at room temperature for 5 days. Water (20 mL) and hexane (100 mL) was added and the phases separated. The organic fraction was dried over $MgSO_4$ and the solvent removed under reduced pressure. The crude material was purified by flash column chromatography on silica gel (hexane) to give the product (256.9 mg, 10%, *Z*:*E* = 14:1) as a colourless oil.

Discernible peaks are denoted *Z* and *E* respectively.

^{1}H NMR (400 MHz, $CDCl_3$) δ 7.31 – 7.26 (m, 2H), 7.22 – 7.15 (m, 3H), 5.66 – 5.52 (m, 2H), 3.45 – 3.37 (m, 1.86H^Z), 3.35 – 3.30 (m, 0.14H^E), 1.77 – 1.67 (m, 3H) ppm.

The spectral data are in agreement with the one in literature.[45]

(*anti*)-1-Phenylbutane-2,3-diol (*rac-anti*-4i)

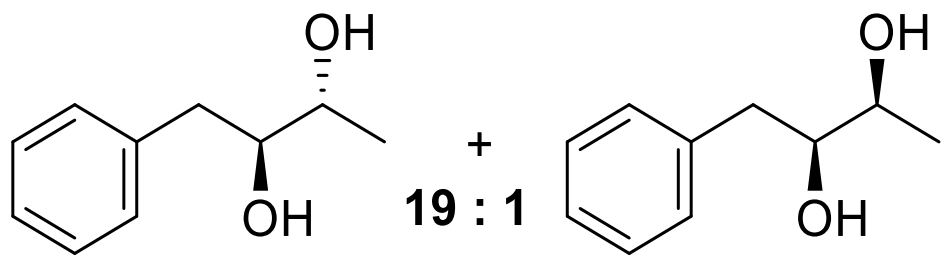

The compound was synthesised according to **General Procedure 5** using (Z)- + (E)-But-2-en-1-ylbenzene (198.3 mg, 1.5 mmol), separated with 25% acetone in hexane, giving (*anti*)-1-Phenylbutane-2,3-diol (216.6 mg, 87%) containing 5% (*syn*)-1-Phenylbutane-2,3-diol as a colourless solid.

Discernible peaks are denoted *a* (*anti*) and *s* (*syn*) respectively.

^{1}H NMR (700 MHz, $CDCl_3$) δ 7.35 – 7.31 (m, 2H), 7.26 – 7.23 (m, 3H), 3.91 – 3.86 (m, 0.95H^a), 3.85 – 3.81 (m, 0.95H^a), 3.72 – 3.67 (m, 0.05H^s), 3.63 – 3.58 (m, 0.05H^s), 2.22 (br. s, 0.05H^s), 1.99 (br. s, 0.05H^s), 1.96 (br. s, 0.95H^a), 1.86 (br. s, 0.95H^a), 1.26 (d, *J* = 6.4 Hz, 3H) ppm.

^{13}C NMR (176 MHz, $CDCl_3$) δ 138.3 (*a*), 138.1 (*s*), 129.6 (*s*),129.5 (*a*), 128.9, 126.8, 76.8 (*s*), 76.0 (*a*), 70.2 (*s*), 70.0 (*a*), 40.2 (*s*), 38.2 (*a*), 19.7 (*s*), 17.4 (*a*) ppm.

The spectral data are in agreement with the one in literature.[46]

(*Z*)- + (*E*)-Hept-3-en-1-ylbenzene (*int$_1$*-4j)

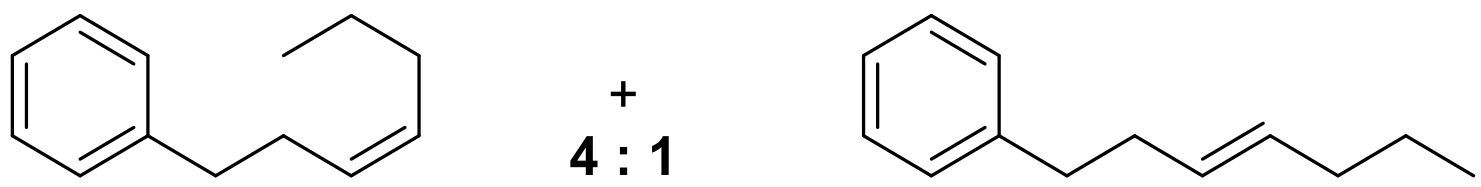

This procedure was adapted from literature.[42] To a heat gun-dried flask was added butyltriphenylphosphonium bromide (7.99 g, 20 mmol, 1.0 equiv.) and dry THF (80 mL). The mixture was cooled to 0 °C and nBuLi (1.6 M in hexanes, 12.5 mL, 20 mmol, 1.0 equiv.) was added slowly. The mixture was stirred at 0 °C for 30 min and then cooled to –78 °C. 3-Phenylpropionaldehyde (2.7 mL, 20 mmol, 1.0 equiv.) was added and the mixture stirred at room temperature for 5 days. Water (20 mL) and hexane (100 mL) was added and the phases separated. The organic fraction was dried over $MgSO_4$ and the solvent removed under reduced pressure. The crude material was purified by flash column chromatography on silica gel (hexane) to give the product (1.779 g, 51%, *Z*:*E* = 4:1) as a colourless oil.

Discernible peaks are denoted *Z* and *E* respectively.

^{1}H NMR (500 MHz, $CDCl_3$) δ 7.30 – 7.26 (m, 2H), 7.22 – 7.15 (m, 3H), 5.49 – 5.35 (m, 2H), 2.69 – 2.63 (m, 2H), 2.40 – 2.33 (m, 1.6H^Z), 2.33 – 2.28 (m, 0.4H^E), 2.01 – 1.92 (m, 2H), 1.39 – 1.27 (m, 2H), 0.87 (t, *J* = 7.3 Hz, 3H) ppm.

^{13}C NMR (126 MHz, $CDCl_3$) δ 142.3, 131.1 (*E*), 130.6 (*Z*), 129.6 (*E*), 129.0 (*Z*), 128.62 (*E*), 128.60 (*Z*), 128.39 (*Z*), 128.36 (*E*), 125.9 (*Z*), 125.8 (*E*), 36.3 (*E*), 36.2 (*Z*), 34.8 (*E*), 34.6 (*E*), 29.4 (*Z*), 29.3 (*Z*), 22.9 (*Z*), 22.8 (*E*), 13.9 (*Z*), 13.8 (*E*) ppm.

The spectral data are in agreement with the one in literature. (*Z*[47], *E*[48])

1-Phenylheptane-3,4-diol (4j)

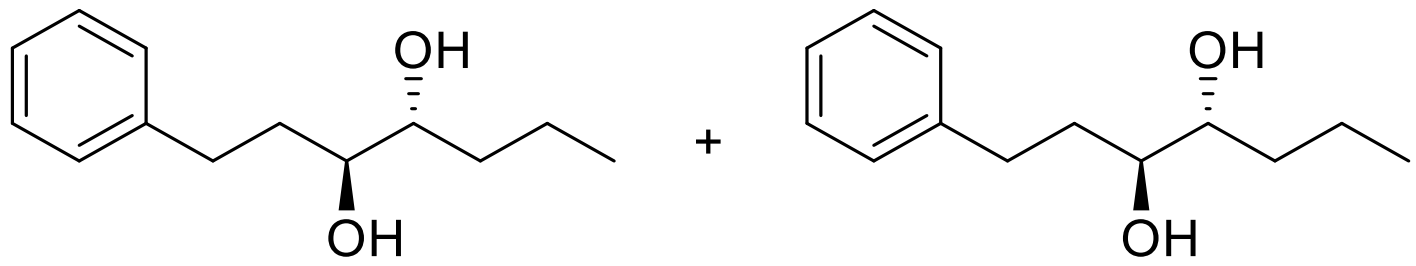

The compound was synthesised according to **General Procedure 5** using (*Z*)- + (*E*)-Hept-3-en-1-ylbenzene (1.57 g, 9.0 mmol), separated with 10% acetone in hexane, giving (*syn*)-1-Phenylheptane-3,4-diol (221.1 mg, 12%) as an off-white solid and (*anti*)-1-Phenylheptane-3,4-diol (871.6 mg, 46%) as a colourless solid.

(*anti*)-1-Phenylheptane-3,4-diol (*rac-anti*-4j)

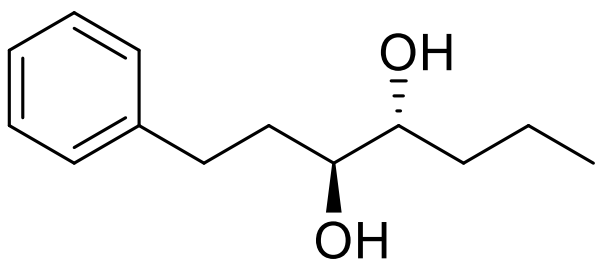

^{1}H NMR (500 MHz, $CDCl_3$) δ 7.31 – 7.27 (m, 2H), 7.24 – 7.17 (m, 3H), 3.65 – 3.59 (m, 2H), 2.92 – 2.85 (m, 1H), 2.71 – 2.63 (m, 1H), 1.83 – 1.62 (m, 4H), 1.57 – 1.27 (m, 4H), 0.93 (t, *J* = 7.2 Hz, 3H) ppm.

^{13}C NMR (126 MHz, $CDCl_3$) δ 142.1, 128.6, 126.1, 74.6, 74.1, 33.6, 33.0, 23.4, 19.3, 14.2 ppm.

HRMS [M+H]$^+$ m/z calc'd for $[C_{13}H_{20}O_2Na]^+$ expect 231.1361; found 231.1369.

(*syn*)-1-Phenylheptane-3,4-diol (*rac-syn*-4j)

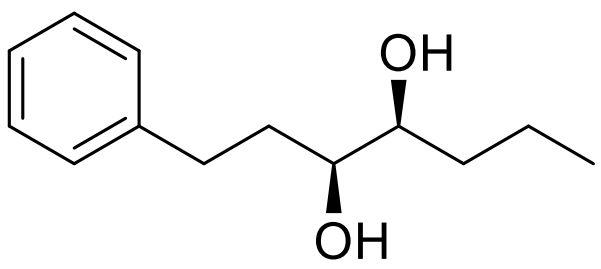

^{1}H NMR (500 MHz, $CDCl_3$) δ 7.32 – 7.27 (m, 2H), 7.24 – 7.16 (m, 3H), 3.50 – 3.39 (m, 2H), 2.91 – 2.80 (m, 1H), 2.76 – 2.66 (m, 1H), 1.97 – 1.63 (m, 4H), 1.54 – 1.31 (m, 4H), 0.93 (t, *J* = 6.9 Hz, 3H) ppm.

^{13}C NMR (126 MHz, $CDCl_3$) δ 142.1, 128.59, 128.58, 126.1, 74.5, 74.0, 35.9, 35.5, 32.1, 18.9, 14.2 ppm.

HRMS [M+H]$^+$ m/z calc'd for $[C_{13}H_{20}O_2Na]^+$ expect 231.1361; found 231.1367.

(*Z*)- + (*E*)-But-2-ene-1,1-diyldibenzene (*int₁*-4k)

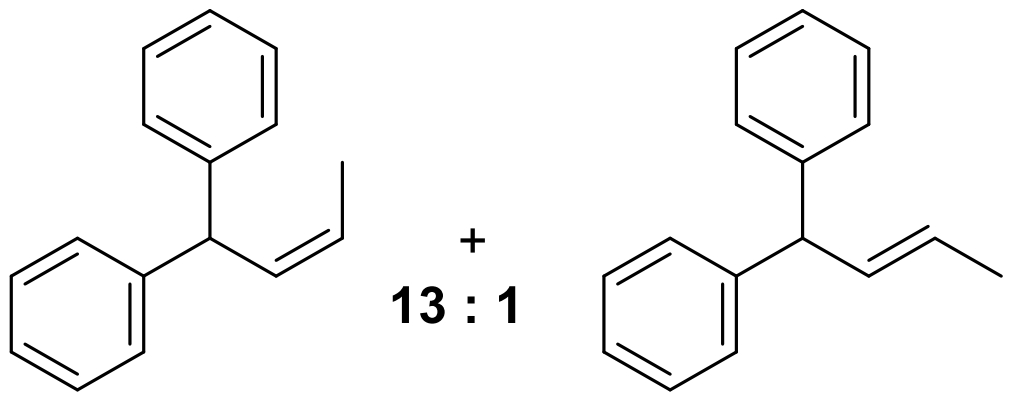

This procedure was adapted from literature.[42] To a heat gun-dried flask was added Ethyl triphenylphosphonium bromide (2.23 g, 6.0 mmol, 1.2 equiv.) and dry THF (80 mL). The mixture was cooled to 0 °C and nBuLi (1.6 M in hexanes, 8.8 mL, 5.5 mmol, 1.1 equiv.) was added slowly. The mixture was stirred at 0 °C for 30 min and then cooled to –78 °C. α-Phenylbenzene acetaldehyde (981 mg, 5.0 mmol, 1.0 equiv.) was added and the mixture stirred at room temperature for 5 days. Water (20 mL) and hexane (100 mL) was added, and the phases separated. The organic fraction was dried over $MgSO_4$ and the solvent removed under reduced pressure. The crude material was purified by flash column chromatography on silica gel (hexane) to give the product (535.6 mg, 51%, *Z*:*E* = 13:1) as a colourless oil.

Discernible peaks are denoted *Z* and *E* respectively.

^{1}H NMR (500 MHz, $CDCl_3$) δ 7.32 – 7.27 (m, 4H), 7.23 – 7.17 (m, 6H), 5.96 – 5.86 (m, 2H), 3.45 – 3.37 (m, 1.86H^Z), 3.35 – 3.30 (m, 0.14H^E), 1.77 – 1.67 (m, 3H) ppm.

^{13}C NMR (126 MHz, $CDCl_3$) δ 144.8, 133.7 (*E*), 133.1 (*Z*), 128.64 (*E*), 128.57 (*Z*), 128.5 (*E*), 128.4 (*Z*), 127.1 (*E*), 126.30 (*E*), 126.27 (*Z*), 124.8 (*Z*), 54.2 (*E*), 48.3 (*Z*), 18.2 (*E*), 13.2 (*Z*) ppm.

The spectral data are in agreement with the one in literature.[49]

1,1-Diphenylbutane-2,3-diol (4k)

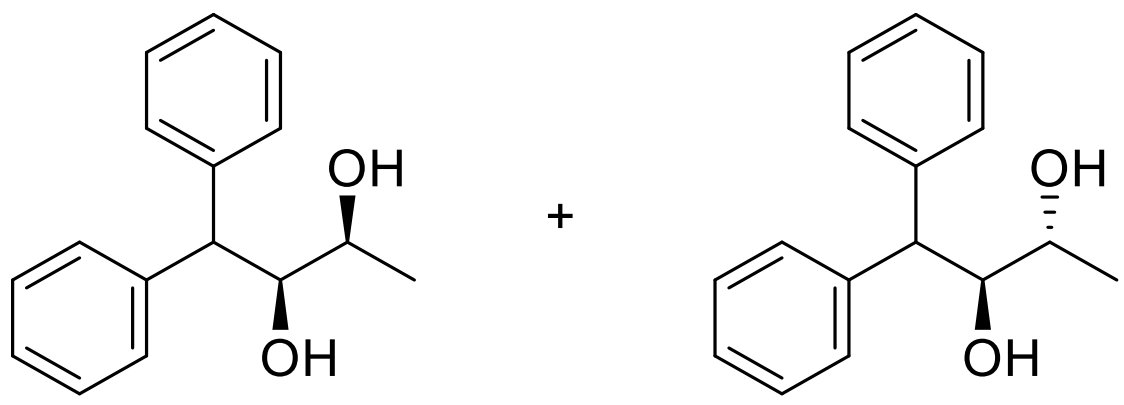

The compound was synthesised according to **General Procedure 5** using (*Z*)- + (*E*)-But-2-ene-1,1-diyldibenzene (312.5 g, 1.5 mmol), separated with 20% acetone in hexane, giving (*syn*)-1,1-Diphenylbutane-2,3-diol (8.4 mg, 2%) as a colourless oil and (*anti*)-1,1-Diphenylbutane-2,3-diol (209.6 mg, 58%) as a colourless solid.

(*anti*)-1,1-Diphenylbutane-2,3-diol (*rac-anti*-4k)

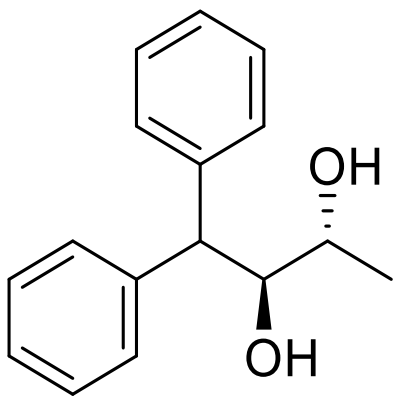

^{1}**H NMR** (700 MHz, $CDCl_3$) δ 7.42 – 7.39 (m, 2H), 7.34 – 7.31 (m, 4H), 7.31 – 7.27 (m, 2H), 7.24 – 7.18 (m, 2H), 4.48 (dd, *J* = 9.5, 3.5 Hz, 1H), 3.96 (d, *J* = 9.5 Hz, 1H), 3.73 – 3.67 (m, 1H), 1.89 (br. s, 1H), 1.88 (br. s, 1H), 1.21 (d, *J* = 6.4 Hz, 3H) ppm.

^{13}**C NMR** (176 MHz, $CDCl_3$) δ 141.5, 141.4, 129.1, 129.0, 128.7, 128.3, 127.1, 127.0, 76.7, 68.4, 54.9, 16.5 ppm.

HRMS [M+H]$^+$ m/z calc'd for $[C_{16}H_{18}O_2Na]^+$ expect 265.1204; found 265.1217.

(*syn*)-1,1-Diphenylbutane-2,3-diol (*rac*-*syn*-4k)

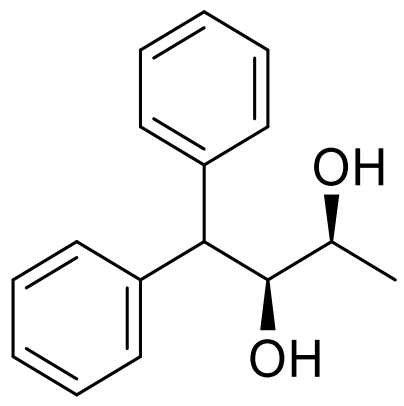

^{1}**H NMR** (700 MHz, $CDCl_3$) δ 7.41 (d, *J* = 7.4 Hz, 2H), 7.35 – 7.27 (m, 6H), 7.25 – 7.22 (m, 1H), 7.21 – 7.18 (m, 1H), 4.21 (d, *J* = 8.2 Hz, 1H), 4.15 – 4.11 (m, 1H), 3.68 – 3.62 (m, 1H), 2.00 – 1.91 (m, 2H), 1.24 (d, *J* = 6.4 Hz, 3H) ppm.

^{13}**C NMR** (176 MHz, $CDCl_3$) δ 142.0, 141.1, 129.07, 129.05, 128.9, 128.5, 127.1, 126.8, 77.1, 67.3, 54.9, 20.8 ppm.

HRMS [M+H]$^+$ m/z calc'd for $[C_{16}H_{18}O_2Na]^+$ expect 265.1204; found 265.1216.

(*Z*)-Hex-4-en-1-yl benzoate ((*Z*)-*int$_1$*-4l)

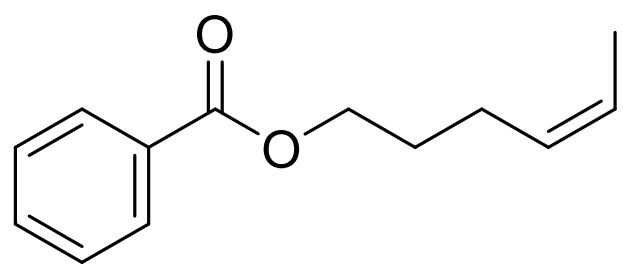

The compound was synthesised according to **General Procedure 6** using (*Z*)-4-hexen-1-ol (500 mg, 5 mmol, 1.0 equiv.) and benzoyl chloride (0.87 mL, 7.5 mmol, 1.5 equiv.), separated with 5% EtOAc in hexane, giving the product (998.8 mg, 98%) as a colourless oil.

^{1}**H NMR** (500 MHz, $CDCl_3$) δ 8.08 – 8.02 (m, 2H), 7.58 – 7.53 (m, 1H), 7.47 – 7.42 (m, 2H), 5.55 –5.48 (m, 1H), 5.46 – 5.38 (m, 1H), 4.33 (t, *J* = 6.6 Hz, 2H), 2.22 (app. q, *J* = 7.4 Hz, 2H), 1.88 – 1.81 (m, 2H) 1.63 – 1.60 (m, 3H) ppm.

^{13}**C NMR** (126 MHz, $CDCl_3$) δ 166.8, 133.0, 130.6, 129.7, 129.2, 128.5, 125.2, 64.6, 28.7, 23.4, 12.9 ppm.

The spectral data are in agreement with the one in literature.[50]

(*anti*)-4,5-Dihydroxyhexyl benzoate (*rac-anti*-4l)

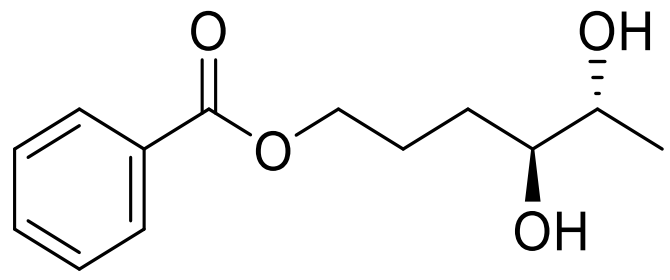

The compound was synthesised according to **General Procedure 5** using (*Z*)-Hex-4-en-1-yl benzoate (510.7 mg, 2.5 mmol), separated with 30% acetone in hexane, giving (*anti*)-4,5-Dihydroxyhexyl benzoate (549.2 mg, 92%) as a colourless solid.

^{1}H NMR (500 MHz, $CDCl_3$) δ 8.06 – 8.02 (m, 2H), 7.58 – 7.53 (m, 1H), 7.47 – 7.42 (m, 2H), 4.43 – 4.33 (m, 2H), 3.86 – 3.80 (m, 1H), 3.69 (dt, *J* = 9.3, 3.6 Hz, 1H), 2.08 – 1.98 (m, 1H), 1.97 – 1.68 (m, 3H), 1.65 – 1.50 (m, 2H), 1.18 (d, *J* = 6.4 Hz, 3H) ppm.

^{13}C NMR (126 MHz, $CDCl_3$) δ 166.8, 133.1, 130.5, 129.7, 128.5, 74.7, 70.6, 65.0, 28.3, 25.6, 17.1 ppm.

HRMS [M+H]$^{+}$ m/z calc'd for $[C_{13}H_{19}O_4]^+$ expect 239.1283; found 239.1283.

(*E*)-Hex-4-en-1-yl benzoate ((*E*)-*int$_1$*-4l)

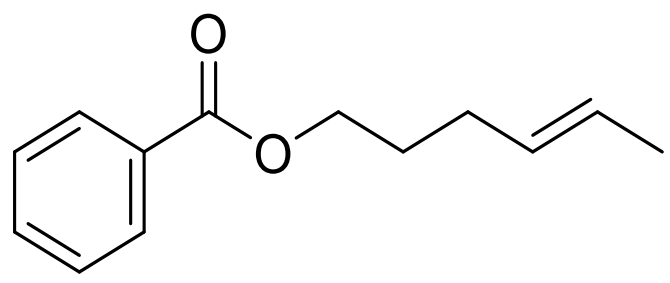

The compound was synthesised according to **General Procedure 6** using (*E*)-4-hexen-1-ol (500 mg, 5 mmol, 1.0 equiv.) and benzoyl chloride (0.87 mL, 7.5 mmol, 1.5 equiv.), separated with 5% EtOAc in hexane, giving the product (943.9 mg, 92%) as a colourless oil.

^{1}H NMR (500 MHz, $CDCl_3$) δ 8.07 – 8.02 (m, 2H), 7.58 – 7.53 (m, 1H), 7.47 – 7.41 (m, 2H), 5.54 –5.40 (m, 2H), 4.32 (t, *J* = 6.6 Hz, 2H), 2.18 – 2.11 (m, 2H), 1.87 – 1.79 (m, 2H) 1.68 – 1.63 (m, 3H) ppm.

^{13}C NMR (126 MHz, $CDCl_3$) δ 166.8, 133.0, 130.6, 130.1, 129.7, 128.5, 126.1, 64.6, 29.1, 28.7, 18.1 ppm.

The spectral data are in agreement with the one in literature.[51]

(*syn*)-4,5-Dihydroxyhexyl benzoate (*rac-syn*-4l)

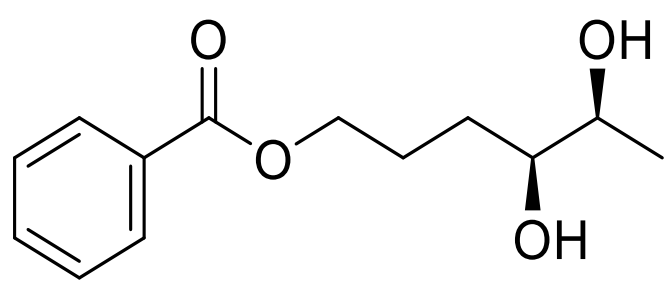

The compound was synthesised according to **General Procedure 5** using (*E*)-Hex-4-en-1-yl benzoate (510.7 mg, 2.5 mmol), separated with 30% acetone in hexane, giving (*syn*)-4,5-Dihydroxyhexyl benzoate (528.1 mg, 89%) as a colourless oil.

^{1}H NMR (500 MHz, $CDCl_3$) δ 8.07 – 8.00 (m, 2H), 7.59 – 7.52 (m, 1H), 7.48 – 7.40 (m, 2H), 4.37 (t, *J* = 6.6 Hz, 2H), 3.67 – 3.59 (m, 1H), 3.47 – 3.38 (m, 1H), 2.35 (br. d, *J* = 4.7 Hz, 1H), 2.09 (br. d, *J* = 4.0 Hz, 1H),

2.06 – 1.96 (m, 1H), 1.94 – 1.83 (m, 1H), 1.72 – 1.63 (m, 1H), 1.60 – 1.50 (m, 1H), 1.21 (d, *J* = 6.3 Hz, 3H) ppm.

^{13}C NMR (126 MHz, $CDCl_3$) δ 166.8, 133.1, 130.4, 129.7, 128.5, 75.8, 71.0, 65.0, 30.0, 25.2, 19.8 ppm.

HRMS [M+H]$^+$ m/z calc'd for $[C_{13}H_{19}O_4]^+$ expect 239.1283; found 239.1283.

(*Z*)-Hex-4-en-1-yl diphenylphosphinate ((*Z*)-*int$_1$*-4m)

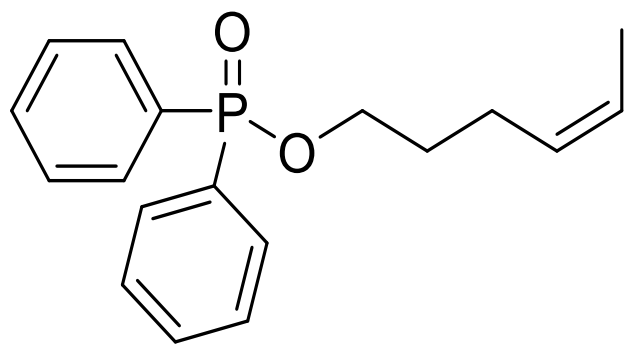

The compound was synthesised according to **General Procedure 6** using (*Z*)-4-hexen-1-ol (500 mg, 5 mmol, 1.0 equiv.) and diphenylphosphinic chloride (1.43 mL, 7.5 mmol, 1.5 equiv.), separated with 50% EtOAc in hexane, giving the product (1.397 g, 93%) as a colourless oil.

^{1}H NMR (500 MHz, $CDCl_3$) δ 7.85 – 7.78 (m, 4H), 7.54 – 7.49 (m, 2H), 7.48 – 7.42 (m, 4H), 5.51 –5.43 (m, 1H), 5.38 – 5.31 (m, 1H), 4.04 (app. q, *J* = 6.7 Hz, 2H), 2.17 (app. q, *J* = 7.2 Hz, 2H), 1.82 – 1.75 (m, 2H) 1.60 – 1.56 (m, 3H) ppm.

^{13}C NMR (126 MHz, $CDCl_3$) δ 132.2 (d, *J* = 2.8 Hz), 131.9 (d, *J* = 137.0 Hz), 131.8 (d, *J* = 10.1 Hz), 129.2, 128.7 (d, *J* = 13.1 Hz), 125.1, 64.6 (d, *J* = 6.0 Hz), 30.6 (d, *J* = 6.6 Hz), 23.1, 12.9 ppm.

^{31}P NMR (252 MHz, $CDCl_3$) δ 31.2 ppm.

HRMS [M+H]$^+$ m/z calc'd for $[C_{18}H_{21}O_2NaP]^+$ expect 323.1177; found 323.1158.

(*anti*)-4,5-Dihydroxyhexyl diphenylphosphinate (*rac-anti*-4m)

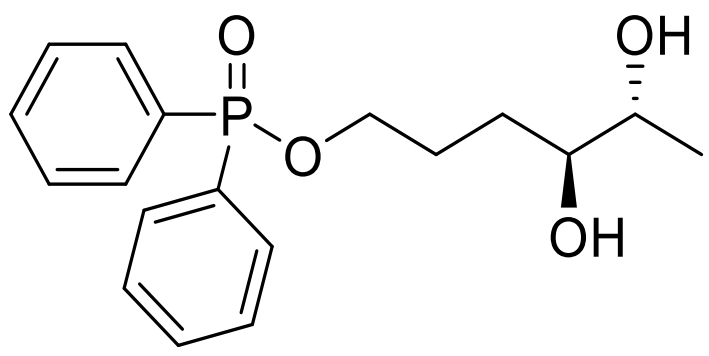

The compound was synthesised according to **General Procedure 5** using (*Z*)-Hex-4-en-1-yl diphenylphosphinate (750.8 mg, 2.5 mmol), separated with 50 – 80% acetone in hexane, giving (anti)-4,5-Dihydroxyhexyl diphenylphosphinate (743.8 mg, 89%) as a light brown oil.

^{1}H NMR (500 MHz, $CDCl_3$) δ 7.83 – 7.74 (m, 4H), 7.55 – 7.48 (m, 2H), 7.48 – 7.40 (m, 4H), 4.13 – 4.03 (m, 2H), 3.79 – 3.72 (m, 1H), 3.63 – 3.56 (m, 1H), 3.10 (br. s, 1H), 2.94 (br. s, 1H), 2.00 – 1.89 (m, 1H), 1.89 – 1.77 (m, 1H), 1.63 – 1.49 (m, 2H), 1.14 – 1.10 (m, 3H) ppm.

^{13}C NMR (126 MHz, $CDCl_3$) δ 132.4 (d, *J* = 2.4 Hz), 131.76 (d, *J* = 10.1 Hz), 131.72 (d, *J* = 10.1 Hz), 131.6 (d, *J* = 137.1 Hz), 128.76 (d, *J* = 13.2 Hz), 128.74 (d, *J* = 13.2 Hz), 74.7, 70.7, 65.5 (d, *J* = 6.2 Hz), 27.6, 27.5 (d, *J* = 5.7 Hz), 17.2 ppm.

^{31}P NMR (252 MHz, $CDCl_3$) δ 32.2 ppm.

HRMS [M+H]$^+$ m/z calc'd for $[C_{18}H_{23}O_4NaP]^+$ expect 357.1232; found 357.1236.

(*E*)-Hex-4-en-1-yl diphenylphosphinate ((*E*)-*int₁*-4m)

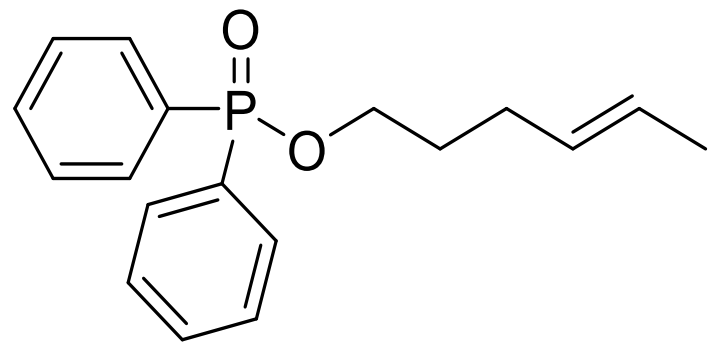

The compound was synthesised according to **General Procedure 6** using (*E*)-4-hexen-1-ol (500 mg, 5 mmol, 1.0 equiv.) and diphenylphosphinic chloride (1.43 mL, 7.5 mmol, 1.5 equiv.), separated with 50% EtOAc in hexane, giving the product (1.413 g, 94%) as a colourless oil.

^{1}H NMR (500 MHz, $CDCl_3$) δ 7.84 – 7.78 (m, 4H), 7.54 – 7.49 (m, 2H), 7.48 – 7.42 (m, 4H), 5.47 –5.33 (m, 2H), 4.02 (app. q, *J* = 6.6 Hz, 2H), 2.12 – 2.07 (m, 2H), 1.82 – 1.74 (m, 2H) 1.63 – 1.60 (m, 3H) ppm.

^{13}C NMR (126 MHz, $CDCl_3$) δ 132.2 (d, *J* = 2.8 Hz), 131.9 (d, *J* = 137.0 Hz), 131.8 (d, *J* = 10.1 Hz), 130.0, 128.7 (d, *J* = 13.1 Hz), 126.1, 64.5 (d, *J* = 6.0 Hz), 30.5 (d, *J* = 6.7 Hz), 28.7, 18.0 ppm.

^{31}P NMR (252 MHz, $CDCl_3$) δ 31.1 ppm.

HRMS [M+H]$^+$ m/z calc'd for $[C_{18}H_{21}O_2NaP]^+$ expect 323.1177; found 323.1172.

(*syn*)-4,5-Dihydroxyhexyl diphenylphosphinate (*rac*-*syn*-4m)

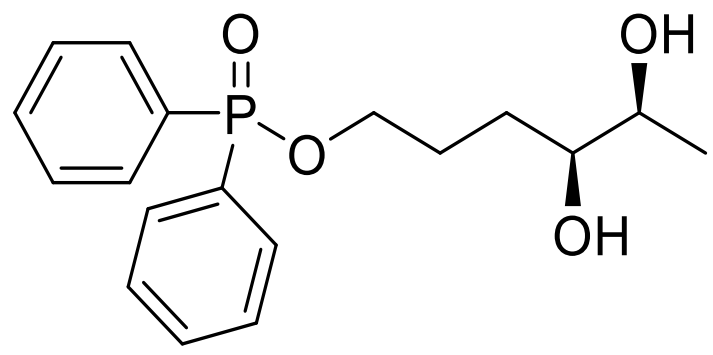

The compound was synthesised according to **General Procedure 5** using (*E*)-Hex-4-en-1-yl diphenylphosphinate (750.8 mg, 2.5 mmol), separated with 50 – 80% acetone in hexane, giving (syn)-4,5-Dihydroxyhexyl diphenylphosphinate (775.4 mg, 93%) as a light brown oil.

^{1}H NMR (500 MHz, $CDCl_3$) δ 7.80 – 7.75 (m, 4H), 7.55 – 7.49 (m, 2H), 7.48 – 7.41 (m, 4H), 4.15 – 4.02 (m, 2H), 3.62 – 3.55 (m, 1H), 3.38 – 3.32 (m, 1H), 2.78 (br. s, 2H), 1.98 – 1.81 (m, 2H), 1.69 – 1.60 (m, 1H), 1.60 – 1.49 (m, 1H), 1.16 (d, *J* = 6.3 Hz, 3H) ppm.

^{13}C NMR (126 MHz, $CDCl_3$) δ 132.4 (d, *J* = 2.5 Hz), 131.76 (d, *J* = 10.1 Hz), 131.73 (d, *J* = 10.1 Hz), 131.3 (d, *J* = 137.9 Hz), 131.76 (d, *J* = 10.1 Hz), 131.75 (d, *J* = 10.1 Hz), 75.7, 70.9, 65.6 (d, *J* = 6.2 Hz), 29.3, 27.2 (d, *J* = 5.6 Hz), 19.5 ppm.

^{31}P NMR (252 MHz, $CDCl_3$) δ 32.3 ppm.

HRMS [M+H]$^+$ m/z calc'd for $[C_{18}H_{23}O_4NaP]^+$ expect 357.1232; found 357.1236.

Stereoselective epimerization of diols

General Procedures

General Procedure 7: Epimerization

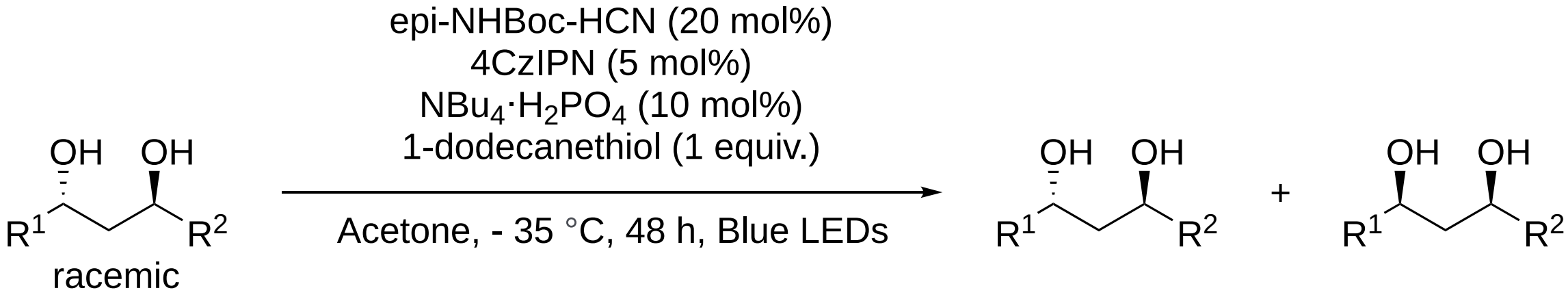

Diol substrate (0.10 mmol, 1 eq.), 4CzIPN (3.9 mg, 0.0050 mmol, 5 mol%), *epi*-NHBoc-DHCN (7.9 mg, 0.020 mmol, 20 mol%) and tetrabutylammonium phosphate monobasic (3.4 mg, 0.010 mmol, 10 mol%) were added to a 4.0 mL crimp-top vial unless otherwise specified. The vial was sealed with a crimp-top cap unless otherwise specified and evacuated and backfilled with nitrogen 10 times. Freshly prepared 0.1 M stock solution of 1-dodecanethiol in degassed acetone (1.0 mL) followed by additional degassed acetone (3.0 mL) was added via syringe to the sealed vial (reaction molarity of 0.025 M unless otherwise specified). The top of the vial was further sealed with Teflon tape then electrical tape and placed in the custom made photoreactor with a positive flow of nitrogen through the apparatus. The reaction vial was cooled to –35 °C and the Kessil® A160WE Tuna blue, LED lamp (100% intensity, 100% ocean blue color) was switched on. After stirring for 48 h (unless otherwise specified) the crude mixture was concentrated in vacuo, analysed by ^{1}H-NMR using internal standard (CH_2Br_2) and purified by silica gel column chromatography.

General Conditions 8: Benzoylation

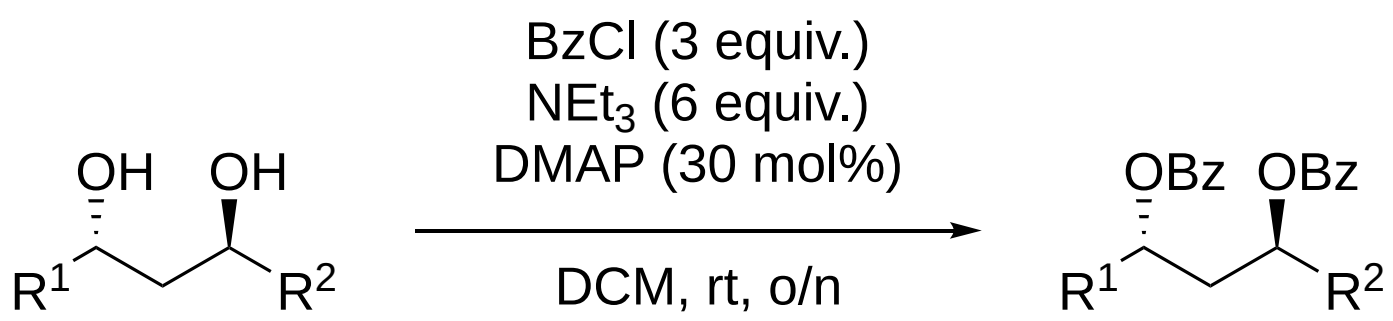

Derivatisation *via* benzoylation was carried out for products which did not contain a chromophore. To purified diol product (1 eq.), DMAP (30 mol%) and triethylamine (6 eq.) in DCM was added benzoyl chloride (3 eq.) at room temperature. The reaction mixture was stirred at room temperature overnight, concentrated under nitrogen flow and purified *via* preparative thin layer chromatography to afford di-benzoylated product.

Racemic samples for chiral SFC or chiral HPLC analysis were obtained using this procedure with racemic diol as substrate. The crude mixture was purified by preparative thin layer chromatography for analysis.

General Procedure 9: TBDPS Protection

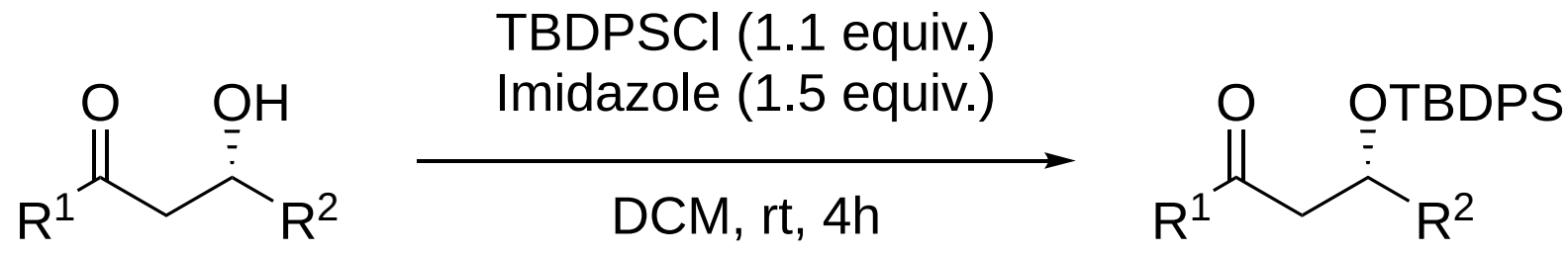

TBDPSCl (1.1 equiv.) and imidazole (1.5 equiv.) were added to a solution of hydroxyketone in DCM (0.05 M) which was then stirred for 4 hours at room temperature. The reaction mixture was then concentrated under a stream of air and purified *via* preparative thin layer chromatography to give the TBDPS protected product.

Scheme 1B and 1C - Reaction profiles for epimerization starting from rac-1 and (*R*,*R*)-1

Reaction profile starting from *rac*-1a

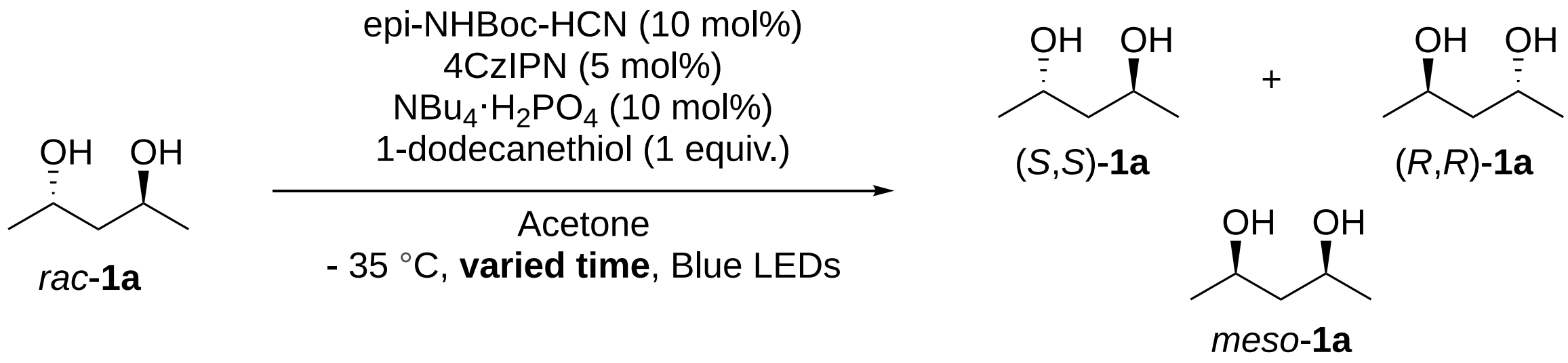

I ndividual reactions were prepared according to **General Procedure 7** using diol substrate *rac*-**1a** (10.4 mg, 0.1 mmol), epi-NHBoc-HCN (4.0 mg, 0.010 mmol, 10 mol%) and stirred for the specified time. The crude mixture was concentrated *in vacuo* then analysed by ^{1}H NMR using internal standard (CH_2Br_2) and ~3 drops of CD_3OD (for sharpening the NMR signals). The crude material in NMR solvent was transferred back to the 4 mL vial and concentrated *in vacuo*. The material was subjected to benzoylation conditions using DCM (2 mL), NEt_3 (0.3 mL, 2.16 mmol) and BzCl (0.15 mL, 1.3 mmol) and a hint of DMAP. The mixture was stirred at ambient temperature overnight and was partially purified by preparative TLC (20% acetone in hexane) which afforded the material for chiral SFC analysis for determination of the *ee* of **1a.** The yield of each stereoisomer was determined by using the NMR yield of the diastereomer and the enantiomeric ratio.

time (h)	(*S*,*S*)-1a (%)	(*R*,*R*)-1a (%)	*meso*-1a (%)
0	50	50	0
0.083	50	45	6
0.25	49	35	14
0.5	50	24	25
0.75	50	18	32
1.5	50	7	42
2	53	1	44
4	59	0	40
6	62	0	34
8	66	0	33

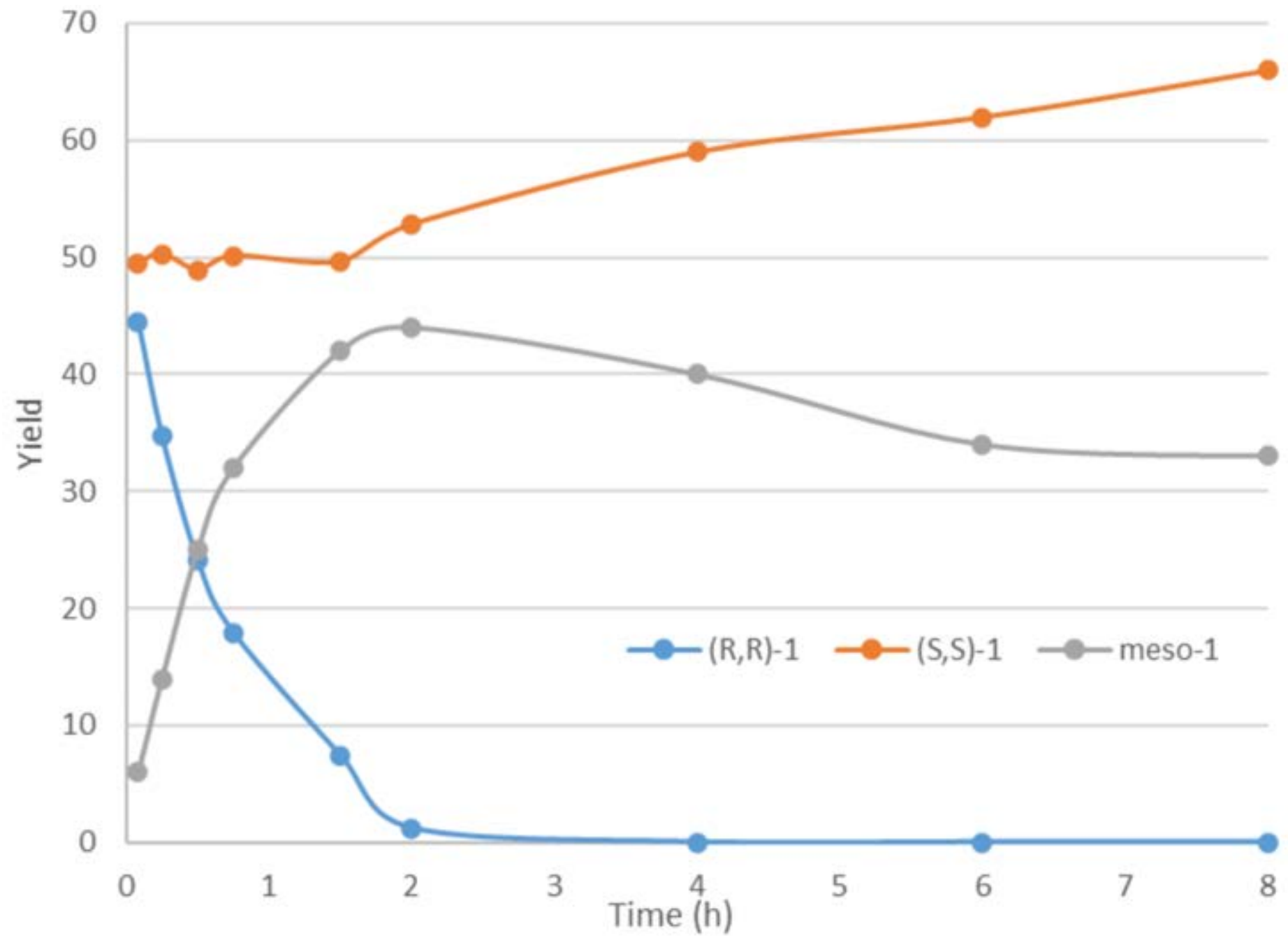

Reaction profile starting from (*R*,*R*)-1a

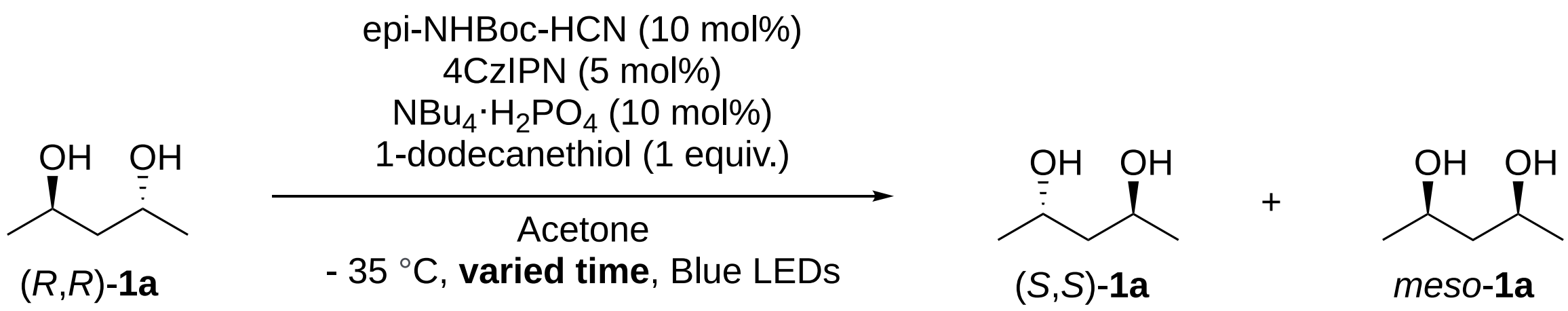

Individual reactions were prepared according to **General Procedure 7** using diol substrate (*R*,*R)*-**1a** (10.4 mg, 0.1 mmol), epi-NHBoc-HCN (4.0 mg, 0.010 mmol, 10 mol%) and stirred for the specified time. The crude mixture was concentrated *in vacuo* then analysed by ^{1}H NMR using internal standard (CH_2Br_2) and ~3 drops of CD_3OD (for sharpening the NMR signals). The crude material in NMR solvent was transferred back to the 4 mL vial and concentrated *in vacuo*. The material was subjected to benzoylation conditions using DCM (2 mL), NEt_3 (0.3 mL, 2.16 mmol) and BzCl (0.15 mL, 1.3 mmol) and a hint of DMAP. The mixture was stirred at ambient temperature overnight and was partially purified by preparative TLC (20% acetone in hexane) which afforded the material for chiral SFC analysis for determination of the *ee* of **1a.** The yield of each stereoisomer was determined by using the NMR yield of the diastereomer and the enantiomeric ratio.

time (h)	(*S*,*S*)-1a (%)	(*R*,*R*)-1a (%)	*meso*-1a (%)
0	0	100	0
0.083	0	93	7
0.25	0	80	15
1	1	49	49
2	5	12	79
4	12	2	81
6	25	1	71
8	43	0	49
12	52	0	40
16	62	0	32

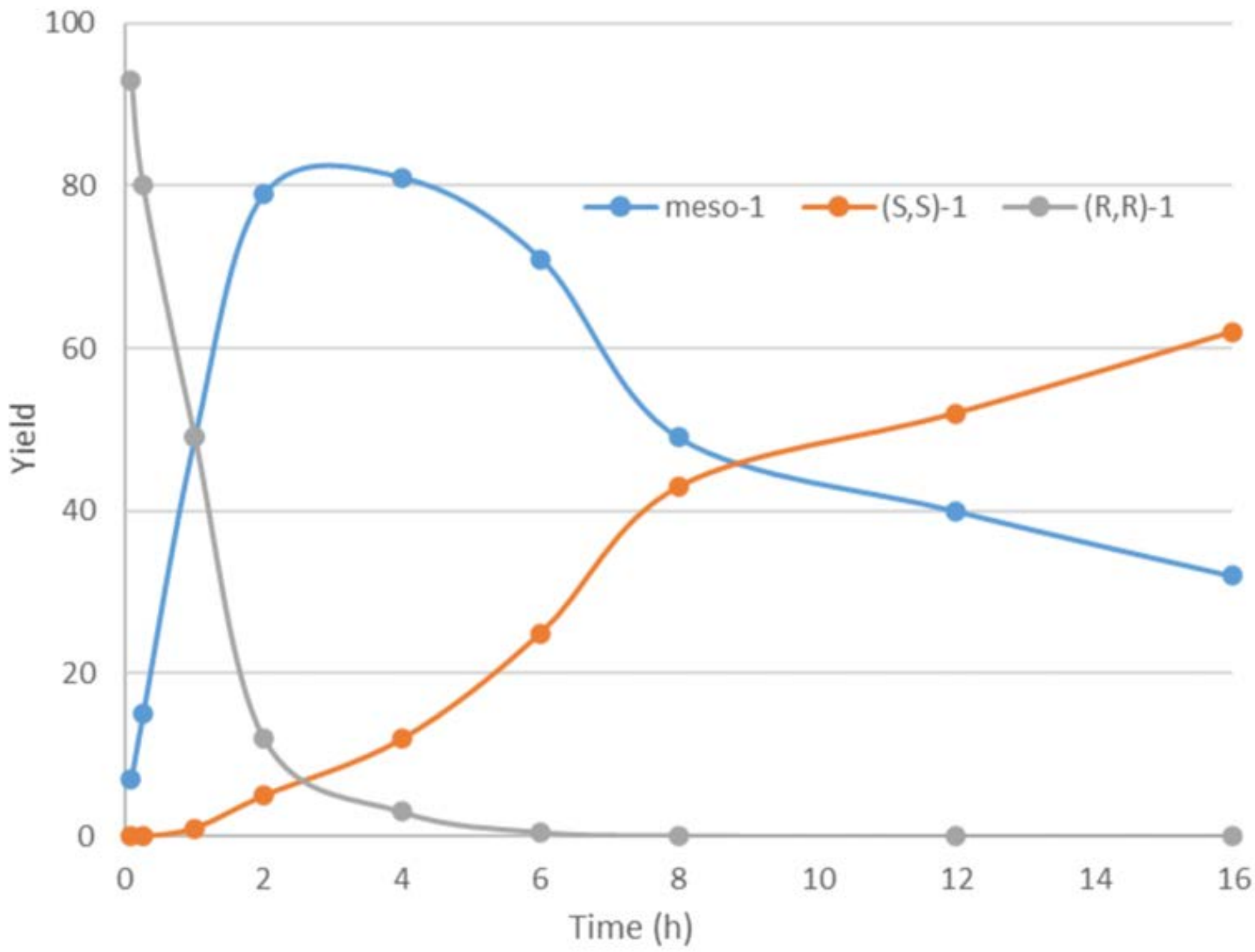

100
80
60
40
20
0
Yield
0
2
4
6
8
10
12
14
16
Time (h)
meso-1
(S,S)-1
(R,R)-1

Scheme 1D – individual experiments from pentan-2,4-diol

Desymmetrisation of *syn*-pentane-2,4-diol

This result was taken from our previous study.[1]

The ee of the diastereomers was 85% and 87% so we take the mean being 86% and this corresponds to a ratio of 93:7 which approximates to 13:1. Therefore $k_6 = 13k_7$.

(2*S*,4*S*)-pentane-2,4-diol (1a) + (2*R*)-4-methyl-6-(phenylsulfonyl)hexane-2,4-diol (2a) at low conversion

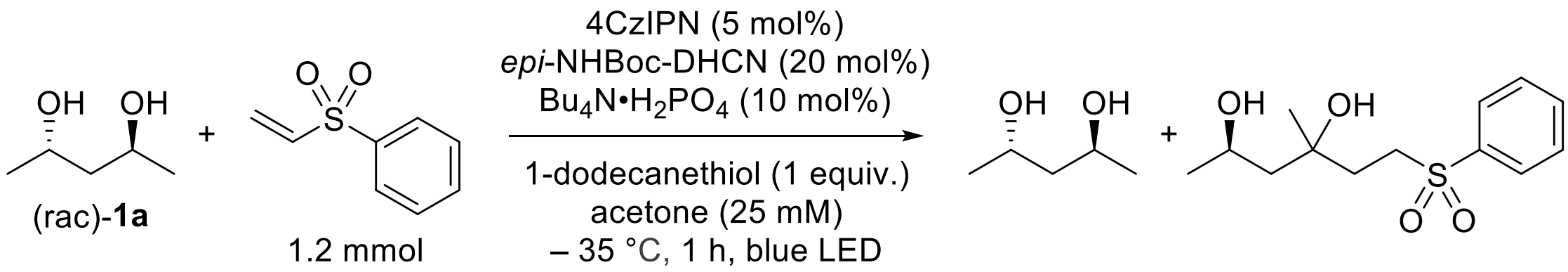

(*anti*)-Pentane-2,4-diol (10.4 mg, 0.10 mmol, 1 eq.), 4CzIPN (3.9 mg, 0.0050 mmol, 5 mol%), *epi*-NHBoc-DHCN (7.9 mg, 0.020 mmol, 20 mol%), (vinylsulfonyl)benzene (20.2 mg, 0.12 mmol, 1.2 equiv.) and tetrabutylammonium phosphate monobasic (3.4 mg, 0.010 mmol, 10 mol%) were added to a 4.0 mL crimp-top vial. The vial was sealed with a crimp-top cap and evacuated and backfilled with nitrogen 7 times. Degassed acetone (4.0 mL) was added via syringe to the sealed vial (reaction molarity of 0.025 M). The top of the vial was further sealed with Teflon tape then electrical tape and placed in the custom made photoreactor with a positive flow of nitrogen through the apparatus. The reaction vial was cooled to –35 °C and the Kessil® A160WE Tuna blue, LED lamp (100% intensity, 100% ocean blue color) was switched on. After stirring for 1 h the crude mixture was concentrated in vacuo, analysed by ^{1}H-NMR using internal standard (CH_2Br_2) and purified by silica gel column chromatography using 0 – 2% MeOH in DCM. This delivered (*anti*)-Pentane-2,4-diol (1a) as a colourless solid (8.1 mg, 78%, 23% *ee*) and impure 4-methyl-6-(phenylsulfonyl)hexane-2,4-diol (2a) which was further purified by preparative TLC using 40% acetone in hexane to yield the two diastereomers as a colourless solid (3.7 mg, 14%, d.r.: 1:1, 96 & 96% *ee*).

From this outcome because the conversion is very low, we approximate an S-factor of 49 to reflect the observed ratio of product enantiomers at 49:1. Therefore $k_1 = 49k_4$

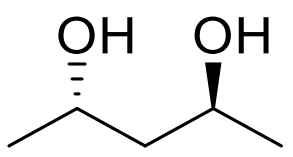

^{1}H NMR (500 MHz, $CDCl_3$) δ 4.22 (m, 2H), 2.23 (s, 2H), 1.61 (dd, *J* = 5.9, 5.3 Hz, 2H), 1.24 (d, *J* = 6.3 Hz, 6H) ppm.

^{13}C NMR (150 MHz, $CDCl_3$) δ 65.7, 45.7, 23.6 ppm.

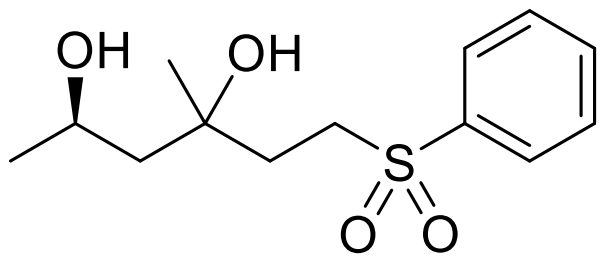

d.r. = 1:1

^{1}H NMR (700 MHz, $CDCl_3$) δ 7.92 (app. t, *J* = 7.5 Hz, 2H), 7.69 – 7.64 (m, 1H), 7.61 – 7.55 (m, 2H), 4.27 – 4.16 (m, 1H), 3.36 (td, *J* = 12.8, 4.0 Hz, 0.5H), 3.14 (td, *J* = 12.8, 4.6 Hz, 0.5H), 1.90 – 1.80 (m, 2H), 1.70 (dd, *J* = 14.7, 11.0 Hz, 0.5H), 1.59 (dd, *J* = 14.4, 10.9 Hz, 0.5H), 1.55 (d, *J* = 14.7 Hz, 0.5H), 1.42 (d, *J* = 14.4 Hz, 0.5H), 1.27 (s, 1.5H), 1.23 – 1.19 (m, 3H), 1.12 (s, 1.5H) ppm.

^{13}C NMR (176 MHz, $CDCl_3$) δ 139.6, 139.3, 133.9, 133.8, 129.47, 129.45, 128.2, 128.1, 71.7, 71.6, 65.9, 65.8, 52.4, 51.8, 48.9, 47.6, 36.1, 32.4, 28.2, 26.3, 25.2, 24.9 ppm.

Chiral SFC Analysis diastereomer 1: CHIRALPAK IK (CO_2:MeOH, 85:15, 2.5 ml min^{-1}, 40 °C, 214 nm) t_R = 4.9 (minor), 5.2 (major) min., 96% *ee*.

Chiral SFC Analysis diastereomer 2: CHIRALPAK IK (CO_2:MeOH, 85:15, 2.5 ml min^{-1}, 40 °C, 214 nm) t_R = 5.7 (major), 6.8 (minor) min., 96% *ee*.

The data is in good agreement with the one reported in literature.[1]

(*S*,*S*)-pentane-2,4-diyl dibenzoate (1a-Bz)

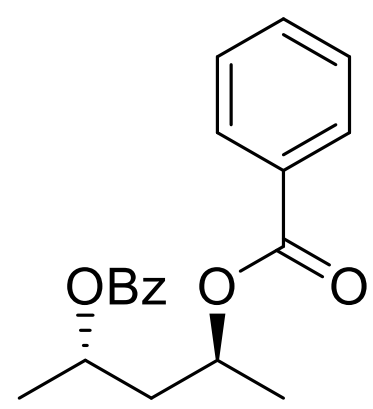

Prepared according to **General Procedure 8** using **1a** (8.1 mg, 0.078 mmol), DMAP (3.7 mg, 0.03 mmol), triethylamine (0.04 ml, 0.2 mmol), benzoyl chloride (0.035 ml, 0.2 mmol) in DCM (1 ml) Purified *via* preparative thin layer chromatography (20% Acetone in Hexane) to give (S,S)-pentane-2,4-diyl dibenzoate as a colourless oil (8.9 mg, 28%, 23% *ee*).

^{1}H NMR (500 MHz, $CDCl_3$) δ 8.00 – 7.95 (m, 4H), 7.54 – 7.85 (m, 2H), 7.37 (app. t, *J* = 8.0 Hz, 4H), 5.35 – 5.28 (m, 2H), 2.10 (app. t, *J* = 6.4 Hz, 2H), 1.41 (d, *J* = 6.3 Hz, 6H) ppm.

^{13}C NMR (150 MHz, $CDCl_3$) δ 166.1, 132.9, 130.7, 129.7, 128.4, 68.5, 42.4, 20.7 ppm.

Chiral SFC Analysis CHIRALPAK IG (CO_2:MeOH, 95:05, 2.5 ml min^{-1}, 40 °C, 225 nm) t_R = 6.1 (minor), 9.6 (major) min, 23% *ee*.

*The end point of the kinetic equilibrium for **1a** only contains (S,S)-**1a** and meso-**1a** in a 2:1 ratio. Based on this one can make the approximation that* $k_6 \approx 2k_4$.

$k_1 = 49k_4$ $\qquad$ $k_6 \approx 2k_4$ $\qquad$ $k_6 = 13k_7$

From the above an approximate relative ordering can be made of: $k_1 >> 2k_4 \approx k_6 >> k_7$

This is based on the following coefficients: $0.04k_1 = 2k_4 \approx k_6 = 13k_7$

Specific numbers for the relative the coefficients can be deduced based on the above:

$k_1 = 318$ $k_4 = 6.5$, $k_6 = 13$, $k_7 = 1$

These estimates can be inputted into a kinetic simulator web program that we have devised to allow rate constants to be readily evaluated against obtained reaction profiles. The simulator provides an interactive browser-based interface for modelling epimerisation kinetics in 1,3-diol systems, allowing the user to enter or adjust individual H-atom abstraction rate constants and starting concentrations and immediately visualise the resulting time-course profiles for each stereoisomer. The kinetic model is formulated as a system of coupled ordinary differential equations describing interconversion between stereoisomeric states, which are solved numerically in the browser using a fixed-step explicit Euler integration scheme. For symmetrical 1,3-diols, the model treats the three interconverting stereoisomeric states using four rate constants, while an extended model for non-symmetrical 1,3-diols allows simulation of four stereoisomeric states using eight HAA rate constants. In both cases, the subsequent RSH-mediated steps are assumed to be fast relative to HAA and therefore not rate-limiting . The simulator for symmetrical 1,3-diols can be accessed at:
https://repository.nottingham.ac.uk/entities/product/3adc90df-b092-4f37-b0d9-4377ea3f36e2
Inputting these relative rate constants gives a reaction time course profile which matches very well in shape with that observed in our studies (see below).

Symmetrical 1,3 diol epimerization - time course simulator

Three stereoisomers with four HAA abstraction rate constants • RSH steps assumed fast and not rate-limiting

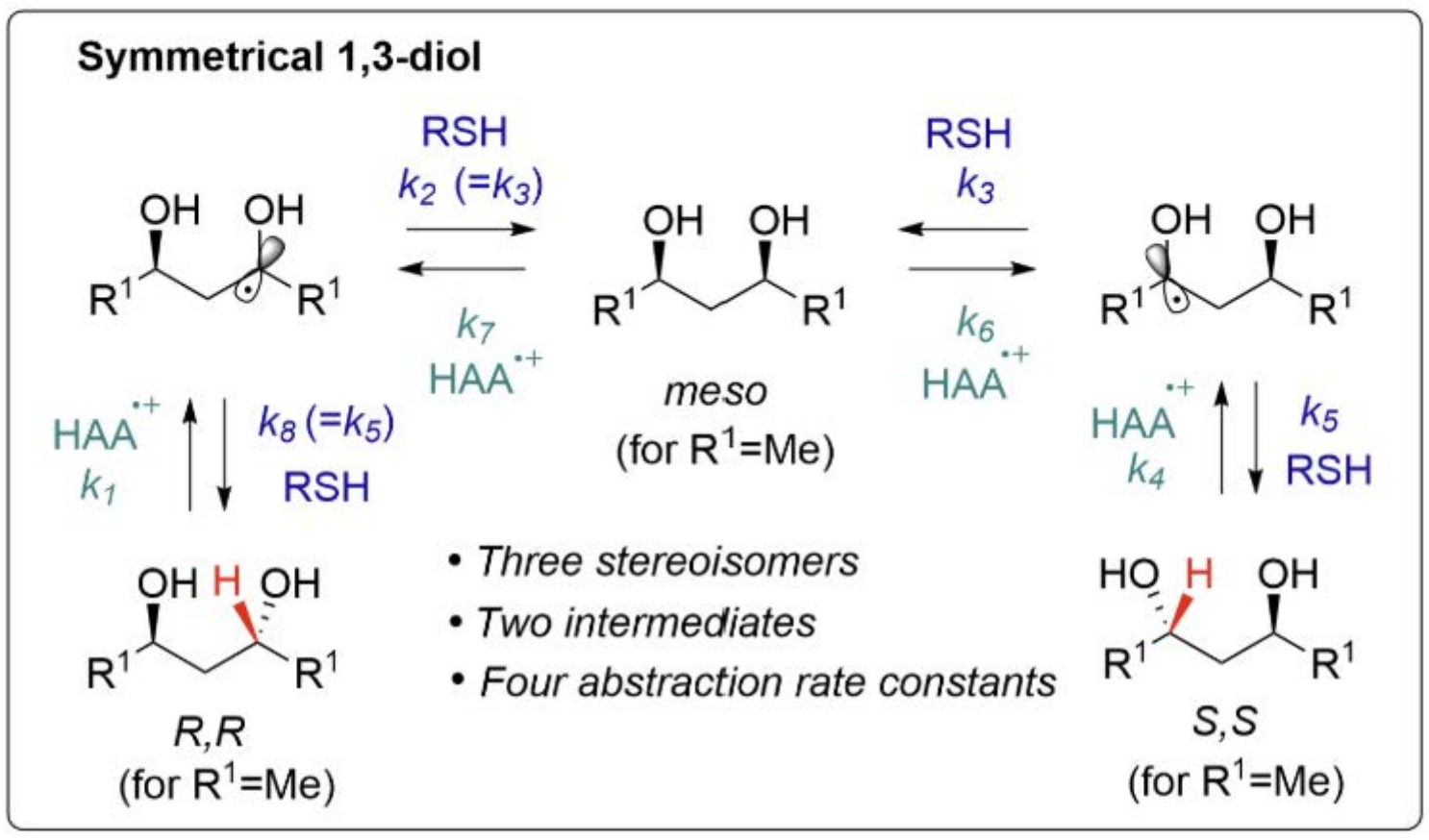

Model

◉ 4 rate constants ○ 8 HAA abstraction rate constants

k_1 (RR → meso) (3.180 s^{-1})	k_7 (meso → RR) (0.010 s^{-1})	k_6 (meso → SS) (0.130 s^{-1})	k_4 (SS → meso) (0.065 s^{-1})
3.180	0.010	0.130	0.065

$[RR]_0$ (M)	$[meso]_0$ (M)	$[SS]_0$ (M)
0.50	0.00	0.50

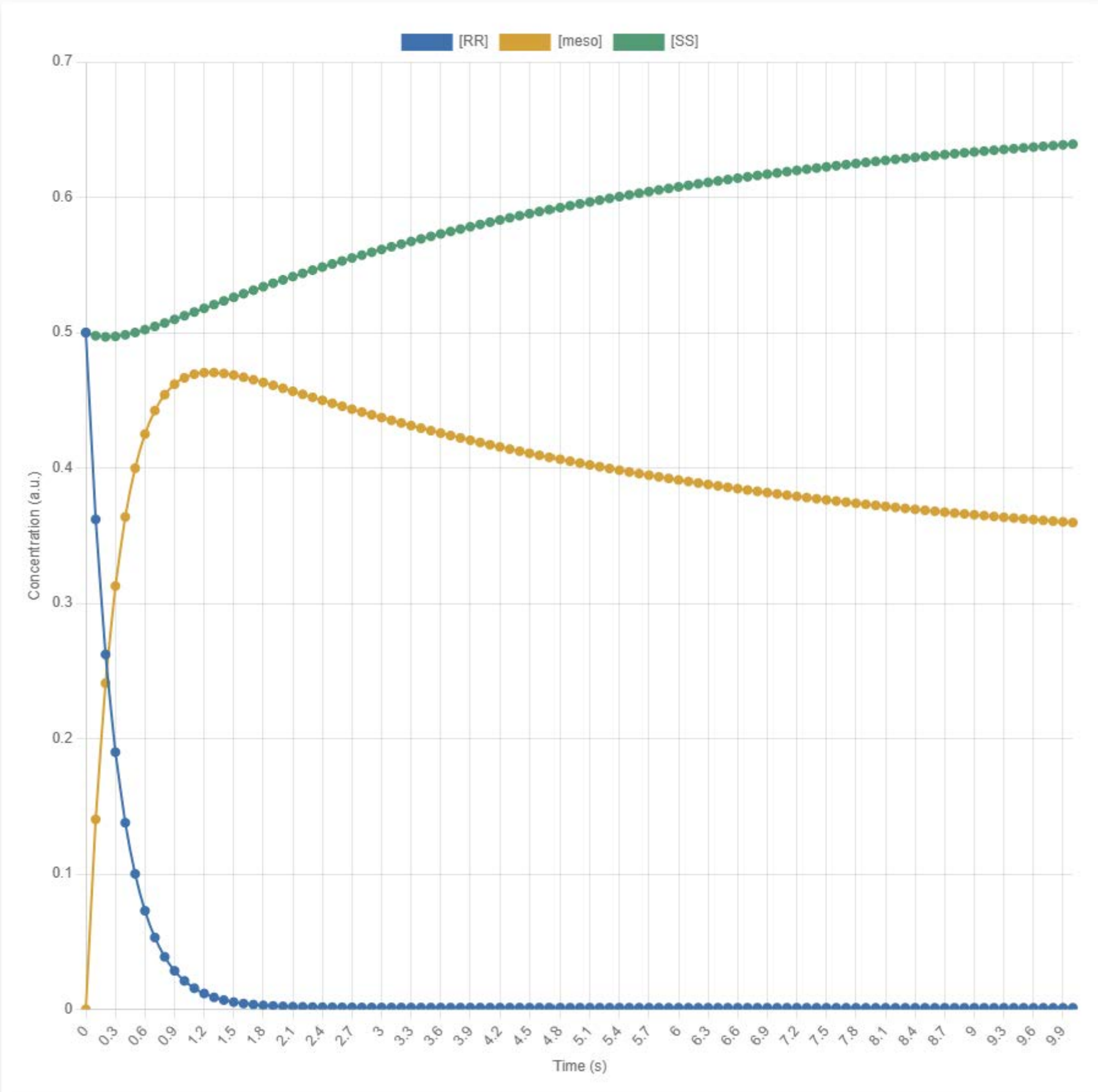

Scheme 2 Scope of symmetrical 1,3-diols

(2S,4S)-pentane-2,4-diol (1a)

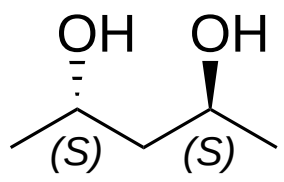

1a

Prepared according to **General Procedure 7** using diol substrate *rac*-**1a** (10.4 mg, 0.1 mmol). The product was purified by silica gel chromatography (elution with 10% Acetone in Hexane) to give (2S,4S)-pentane-2,4-diol as a colourless oil in reduced yield due to the high volatility of the product (2.6 mg, 0.025 mmol, 25%, 99% *ee*).

^{1}H NMR (700 MHz, $CDCl_3$) δ 4.17 (sext, *J* = 6.0 Hz, 2H), 1.93 (br s, 2H), 1.61 (t, *J* = 5.6 Hz, 2H), 1.24 (d, *J* = 6.4 Hz, 6H) ppm.

^{13}C NMR (176 MHz, $CDCl_3$) δ 65.7, 45.7, 23.7 ppm.

$[\alpha]_D^{25.0}$ = + 20.3 (*c* 0.11, $CHCl_3$)

(2*S*,4*S*)-pentane-2,4-diyl dibenzoate (1a-Bz)

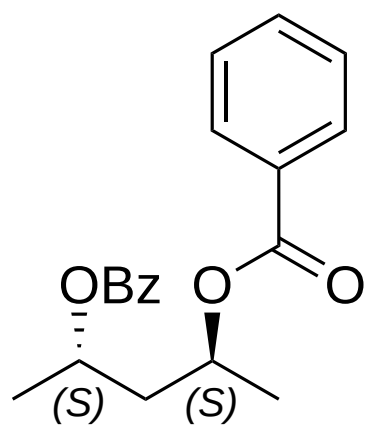

1a-Bz

Prepared according to **General Procedure 8** using **1a** (2.6 mg, 0.025 mmol), DMAP (0.7 mg, 0.0057 mmol), triethylamine (0.02 ml, 0.14 mmol), benzoyl chloride (0.01 ml, 0.06 mmol) in DCM (0.12 ml) Purified *via* preparative thin layer chromatography (15% Acetone in Hexane) to give (2*S*,4*S*)-pentane-2,4-diyl dibenzoate as a colourless oil (3.2 mg, 0.010 mmol, 41%).

^{1}H NMR (700 MHz, $CDCl_3$) δ 7.98 (d, *J* = 8.0 Hz, 4H), 7.51 (app t, *J* = 7.8 Hz, 2H), 7.38 (app t, *J* = 7.4 Hz, 4H), 5.31 (sext, *J* = 6.3 Hz, 2H), 2.10 (t, *J* = 6.3 Hz, 2H), 1.41 (d, *J* = 6.2 Hz, 6H).

^{13}C NMR (176 MHz, $CDCl_3$) δ 166.1, 132.9, 130.7, 129.7, 128.4, 68.5, 42.4, 20.7.

HRMS $[M+H]^+$ m/z calc'd for $[C_{21}H_{25}O_4]^+$ expect 335.1254 found 335.1270

Chiral SFC Analysis CHIRALPAK IG (CO_2:MeOH, 95:05, 2.5 ml min^{-1}, 40 °C, 226 nm) t_R = 10.3 (major) minutes, 99% *ee*.

(3S,5S)-heptane-3,5-diol (1b)

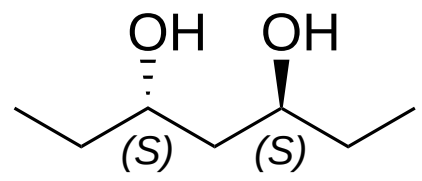

1b

Prepared according to **General Procedure 7** using diol substrate *rac*-**1b** (13.2 mg, 0.1 mmol). Crude NMR yields were obtained [48% anti, 26% syn], then the crude product was purified by silica gel chromatography (elution with 5 – 10% Acetone in Pet. Ether) to give (3S,5S)-heptane-3,5-diol as a white solid (5.6 mg, 0.042 mmol, 42%, 99% *ee*).

^{1}H NMR (700 MHz, $CDCl_3$) δ 3.87 (p, *J* = 6.0 Hz, 2H), 2.10 (br s, 2H), 1.62 (t, *J* = 5.6 Hz, 2H), 1.59 – 1.48 (m, 4H), 0.95 (t, *J* = 7.5 Hz, 6H) ppm.

^{13}C NMR (176 MHz, $CDCl_3$) δ 71.0, 41.5, 30.4, 10.2 ppm.

$[\alpha]_D^{25.0}$ = + 9.7 (*c* 0.37, $CHCl_3$)

*Literature $[\alpha]_D$ values for S,S-**1b**: + 20.6 (c = 1.12; T = 25* °C, $CHCl_3$)[52] *and + 25 (c= 1; T = 25, $CHCl_3$)*[53]

*Absolute configuration of **1b** therefore assigned as S,S.*

(3S,5S)-heptane-3,5-diyl dibenzoate (1b-Bz)

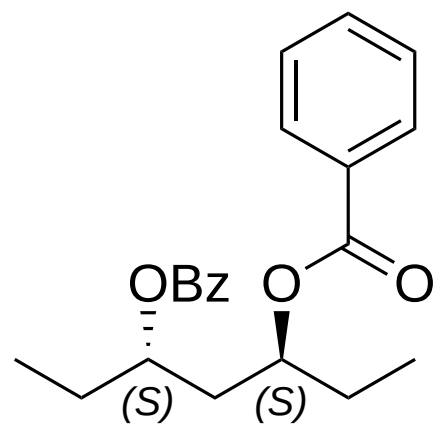

1b-Bz

Prepared according to **General Procedure 8** using **1b** (2.6 mg, 0.02 mmol), DMAP (0.7 mg, 0.0057 mmol), triethylamine (0.02 ml, 0.14 mmol), benzoyl chloride (0.01 ml, 0.06 mmol) in DCM (0.08 ml) Purified *via* preparative thin layer chromatography (15% Acetone in Hexane) to give (3S,5S)-heptane-3,5-diyl dibenzoate as a colourless oil (2.1 mg, 0.006 mmol, 31%).

^{1}H NMR (700 MHz, $CDCl_3$) δ 7.96 (app dd, *J* = 8.3, 1.3 Hz, 4H), 7.49 (app t, *J* = 7.4 Hz, 2H), 7.35 (app t, *J* = 7.8 Hz, 4H), 5.21 (p, *J* = 6.1 Hz, 2H), 2.09 (t, *J* = 6.1 Hz, 2H), 1.81 – 1.73 (m, 4H), 0.95 (t, *J* = 7.5 Hz, 6H).

^{13}C NMR (176 MHz, $CDCl_3$) δ 166.2, 132.8, 130.7, 129.7, 128.3, 72.8, 37.4, 27.6, 9.6.

HRMS $[M+H]^+$ m/z calc'd for $[C_{21}H_{25}O_4]^+$ expect 341.1753 found 341.1740

Chiral SFC Analysis CHIRALPAK IG (CO_2:MeOH, 97:03, 2.5 ml min^{-1}, 40 °C, 225 nm) t_R = 7.8 (major) minutes, 99% *ee*.

(4*S*,6*S*)-nonane-4,6-diol (1c)

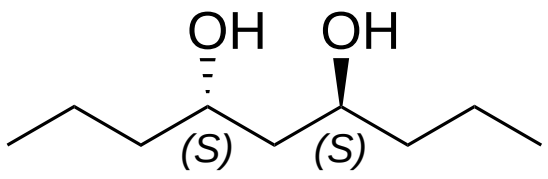

1c

Prepared according to **General Procedure 7** using diol substrate *rac*-**1c** (16.0 mg, 0.1 mmol). The reaction was carried out a concentration of 0.0125 M, using 8 ml of acetone in a 6 dram vial sealed with a B19 suba seal. Crude NMR yields were obtained [49% anti, 32% syn], then the crude product was purified by silica gel chromatography (elution with 5% Acetone in Pet. Ether) to give (4*S*,6*S*)-nonane-4,6-diol as a white solid (5.1 mg, 0.032 mmol, 32%, 97% *ee*).

^{1}**H NMR** (500 MHz, $CDCl_3$) δ 3.99 – 3.92 (m, 2H), 2.35 (br s, 2H), 1.60 (dd, *J* = 6.1, 5.2 Hz, 2H), 1.56 – 1.49 (m, 2H), 1.49 – 1.39 (m, 4H), 1.39 – 1.29 (m, 2H), 0.94 (t, *J* = 7.1 Hz, 6H) ppm.

^{13}**C NMR** (176 MHz, $CDCl_3$) δ 69.3, 42.5, 39.8, 19.1, 14.2 ppm.

$[\alpha]_D^{25.0}$ = + 10.5 (*c* 0.14, MeOH)

(4S,6S)-nonane-4,6-diyl dibenzoate (1c-Bz)

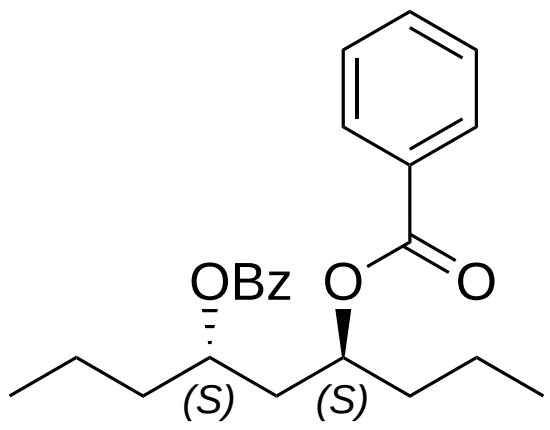

1c-Bz

Prepared according to **General Procedure 8** using **1c** (3.2 mg, 0.02 mmol), DMAP (0.7 mg, 0.0057 mmol), triethylamine (0.02 ml, 0.14 mmol), benzoyl chloride (0.01 ml, 0.06 mmol) in DCM (0.08 ml) Purified *via* preparative thin layer chromatography (15% Acetone in Hexane) to give (4S,6S)-nonane-4,6-diyl dibenzoate as a colourless oil (5.8 mg, 0.016 mmol, 97%).

^{1}**H NMR** (700 MHz, $CDCl_3$) δ 7.94 (d, *J* = 8.2 Hz, 4H), 7.48 (app td, *J* = 7.4, 1.0 Hz, 2H), 7.34 (app t, *J* = 7.5 Hz, 4H), 5.28 (p, *J* = 6.1 Hz, 2H), 2.09 (t, *J* = 6.1 Hz, 2H), 1.79 – 1.73 (m, 2H), 1.69 – 1.63 (m, 2H), 1.46 – 1.35 (m, 4H), 0.92 (t, *J* = 7.3 Hz, 6H) ppm.

^{13}**C NMR** (176 MHz, $CDCl_3$) δ 166.2, 132.8, 130.7, 129.7, 128.3, 71.6, 38.4, 36.9, 18.6, 14.1 ppm.

HRMS $[M+H]^+$ m/z calc'd for $[C_{23}H_{29}O_4]^+$ expect 369.2066 found 369.2063

Chiral SFC Analysis CHIRALPAK IA (CO_2:MeOH, 99:01, 2.5 ml min^{-1}, 40 °C, 223 nm) t_R = 9.2 (major), 10.4 (minor) minutes, 97% *ee*.

(5S,7S)-undecane-5,7-diol (1d)

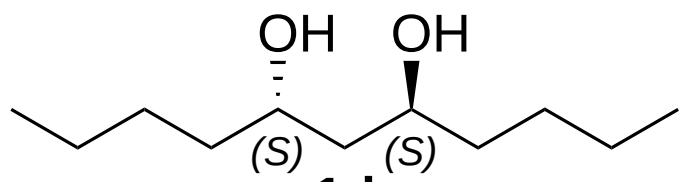

1d

Prepared according to **General Procedure 7** using diol substrate *rac*-**1d** (18.8 mg, 0.1 mmol). Crude NMR yields were obtained [54% anti, 43% syn], then the crude product was purified by silica gel chromatography (elution with 5% Acetone in Pet. Ether) to give (5S,7S)-undecane-5,7-diol as a white solid (6.5 mg, 0.035 mmol, 35%, 99% *ee*).

^{1}H NMR (700 MHz, $CDCl_3$) δ 3.97 – 3.90 (m, 2H), 2.33 (br s, 2H), 1.61 (t, *J* = 5.6 Hz, 2H), 1.57 – 1.50 (m, 2H), 1.50 – 1.43 (m, 2H), 1.43 – 1.24 (m, 8H), 0.91 (t, *J* = 7.2 Hz, 6H) ppm.

^{13}C NMR (176 MHz, $CDCl_3$) δ 69.6, 42.4, 37.3, 28.1, 22.8, 14.2 ppm.

$[\alpha]_D^{25.0}$ = + 25.4 (*c* 0.19, $CHCl_3$)

*Literature $[\alpha]_D$ values for R,R-**1d**: - 6.0 (c = 1; T = 23* °C, CH_2Cl_2)[54]

*Absolute configuration of **1d** therefore assigned as S,S.*

(5S,7S)-undecane-5,7-diyl dibenzoate (1d-Bz)

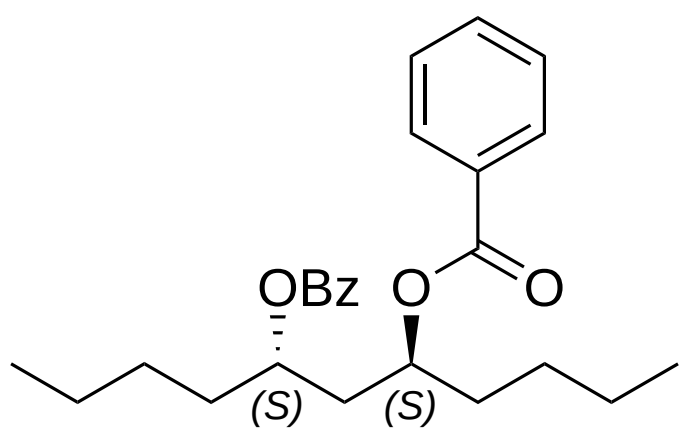

1d-Bz

Prepared according to **General Procedure 8** using **1d** (3.8 mg, 0.02 mmol, DMAP (0.7 mg, 0.0057 mmol), triethylamine (0.02 ml, 0.14 mmol), benzoyl chloride (0.01 ml, 0.06 mmol) in DCM (0.08 ml) Purified *via* preparative thin layer chromatography (15% Acetone in Hexane) to give (5*S*,7*S*)-undecane-5,7-diyl dibenzoate as a colourless oil (6.7 mg, 0.017 mmol, 84%).

^{1}H NMR (700 MHz, $CDCl_3$) δ 7.95 (app dd, *J* = 8.3, 1.3 Hz, 4H), 7.48 (app t, *J* = 7.4 Hz, 2H), 7.34 (app t, *J* = 7.8 Hz, 4H), 5.26 (p, *J* = 6.1 Hz, 2H), 2.09 (t, *J* = 6.1 Hz, 2H), 1.81 – 1.73 (m, 2H), 1.73 – 1.64 (m, 2H), 1.41 – 1.24 (m, 8H), 0.87 (t, *J* = 7.1 Hz, 6H) ppm.

^{13}C NMR (176 MHz, $CDCl_3$) δ 166.2, 132.8, 130.7, 129.7, 128.3, 71.8, 38.4, 34.4, 27.5, 22.7, 14.1 ppm.

HRMS $[M+H]^+$ m/z calc'd for $[C_{25}H_{33}O_4]^+$ expect 397.2379 found 397.2371

Chiral SFC Analysis CHIRALPAK IE (CO_2:MeOH, 97:03, 2.5 ml min^{-1}, 40 °C, 225 nm) t_R = 11.6 (major) minutes, 99% *ee*.

(4S,6S)-2,8-dimethylnonane-4,6-diol (1e)

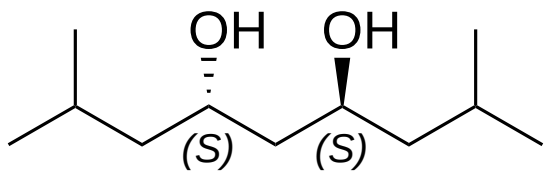

1e

Prepared according to **General Procedure 7** using diol substrate *rac*-**1e** (18.8 mg, 0.1 mmol). Crude NMR yields were obtained [47% anti, 37% syn], then the crude product was purified by silica gel chromatography (elution with 5% Acetone in Pet. Ether) to give (4S,6S)-2,8-dimethylnonane-4,6-diol as a white solid (6.2 mg, 0.033 mmol, 33%, 99% *ee*).

^{1}H NMR (700 MHz, $CDCl_3$) δ 4.07 – 4.02 (m, 2H), 1.88 (br s, 2H), 1.78 – 1.71 (m, 2H), 1.58 (t, *J* = 5.6 Hz, 2H), 1.49 (ddd, *J* = 13.9, 8.4, 5.7 Hz, 2H), 1.25 (ddd, *J* = 13.5, 8.5, 4.9 Hz, 2H), 0.93 (app dd, *J* = 6.6, 3.7 Hz, 12H) ppm.

^{13}C NMR (176 MHz, $CDCl_3$) δ 67.6, 46.8, 43.4, 24.8, 23.4, 22.4 ppm.

$[\alpha]_D^{25.0}$ = + 19.3 (*c* 0.15, $CHCl_3$)

(4*S*,6*S*)-2,8-dimethylnonane-4,6-diyl dibenzoate (1e-Bz)

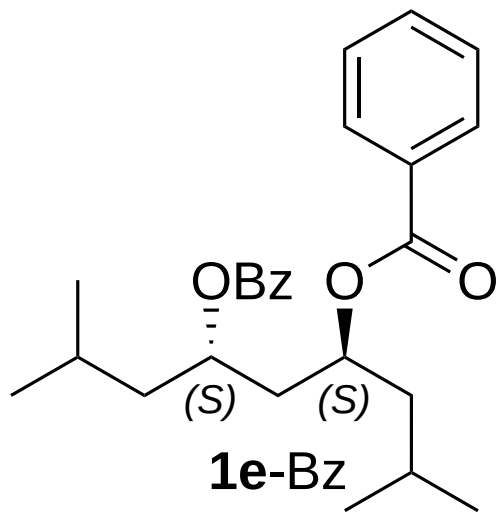

1e-Bz

Prepared according to **General Procedure 8** using **1e** (3.8 mg, 0.02 mmol), DMAP (0.7 mg, 0.0057 mmol), triethylamine (0.02 ml, 0.14 mmol), benzoyl chloride (0.01 ml, 0.06 mmol) in DCM (0.08 ml) Purified *via* preparative thin layer chromatography (15% Acetone in Hexane) to give (4*S*,6*S*)-2,8-dimethylnonane-4,6-diyl dibenzoate as a colourless oil (3.5 mg, 0.009 mmol, 44%).

^{1}H NMR (700 MHz, $CDCl_3$) δ 7.93 (app d, *J* = 8.1 Hz, 4H), 7.47 (t, *J* = 7.7 Hz, 2H), 7.32 (t, *J* = 7.8 Hz, 4H), 5.37 – 5.32 (m, 2H), 2.08 (t, *J* = 6.0 Hz, 2H), 1.77 (ddd, *J* = 14.0, 8.0, 6.0 Hz, 2H), 1.68 (sep, *J* = 6.7 Hz, 2H), 1.48 (ddd, *J* = 13.8, 8.0, 5.1 Hz, 2H), 0.93 (dd, *J* = 6.5, 3.7 Hz, 12H) ppm.

^{13}C NMR (176 MHz, $CDCl_3$) δ 166.2, 132.8, 130.6, 129.6, 128.3, 70.5, 43.8, 39.3, 24.9, 23.1, 22.6 ppm.

HRMS $[M+H]^+$ m/z calc'd for $[C_{25}H_{33}O_4]^+$ expect 397.2379 found 397.2373

Chiral SFC Analysis CHIRALPAK IG (CO_2:MeOH, 97:03, 2.5 ml min^{-1}, 40 °C, 226 nm) t_R = 5.1 (major) minutes, 99% *ee*.

(5S,7S)-2,10-dimethylundecane-5,7-diol (1f)

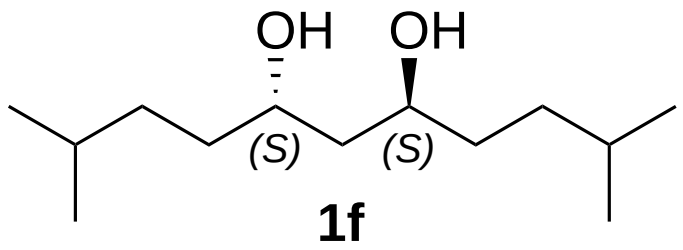

1f

Prepared according to **General Procedure 7** using diol substrate *rac*-**1f** (21.6 mg, 0.1 mmol). Crude NMR yields were obtained [52% anti, 30% syn], then the crude product was purified by silica gel chromatography (elution with 5% Acetone in Pet. Ether) to give (5S,7S)-2,10-dimethylundecane-5,7-diol as a white solid (10.1 mg, 0.047 mmol, 47%, 99% *ee*).

^{1}H NMR (700 MHz, $CDCl_3$) δ 3.91 (p, *J* = 6.0 Hz, 2H), 2.29 (br s, 2H), 1.62 (dd, *J* = 5.9, 5.3 Hz, 2H), 1.58 – 1.50 (m, 4H), 1.46 (dddd, *J* = 13.4, 11.2, 5.5, 5.5 Hz, 2H), 1.31 (dddd, *J* = 13.0, 11.5, 6.6, 4.9 Hz, 2H), 1.18 (dddd, *J* = 12.9, 11.6, 6.5, 5.1 Hz, 2H), 0.89 (dd, *J* = 6.6, 2.5 Hz, 12H) ppm.

^{13}C NMR (176 MHz, $CDCl_3$) δ 70.0, 42.4, 35.5, 35.1, 28.2, 22.8, 22.7 ppm.

$[\alpha]_D^{25.0}$ = + 7.1 (*c* 0.38, $CHCl_3$)

(5*S*,7*S*)-2,10-dimethylundecane-5,7-diyl dibenzoate (1f-Bz)

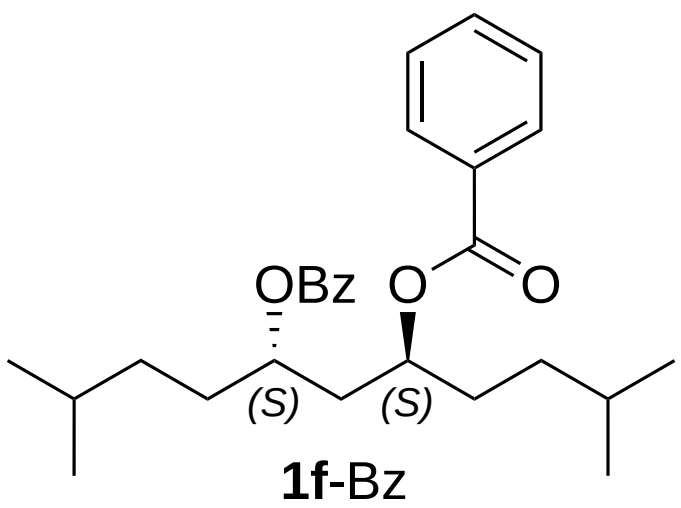

1f-Bz

Prepared according to **General Procedure 8** using **1f** (4.3 mg, 0.02 mmol), DMAP (0.7 mg, 0.0057 mmol), triethylamine (0.02 ml, 0.14 mmol), benzoyl chloride (0.01 ml, 0.06 mmol) in DCM (0.08 ml) Purified *via* preparative thin layer chromatography (15% Acetone in Hexane) to give (5*S*,7*S*)-2,10-dimethylundecane-5,7-diyl dibenzoate as a colourless oil (5.2 mg, 0.012 mmol, 61%).

^{1}H NMR (700 MHz, $CDCl_3$) δ 7.95 (app dd, *J* = 8.3, 1.3 Hz, 4H), 7.49 (tt, *J* = 7.4, 1.3 Hz, 2H), 7.35 (app t, *J* = 7.8 Hz, 4H), 5.24 (p, *J* = 6.1 Hz, 2H), 2.09 (t, *J* = 6.1 Hz, 2H), 1.79 – 1.72 (m, 2H), 1.72 – 1.66 (m, 2H), 1.53 (sep, *J* = 6.6 Hz, 2H), 1.31 – 1.21 (m, 4H), 0.86 (d, *J* = 6.7 Hz, 12H) ppm.

^{13}C NMR (176 MHz, $CDCl_3$) δ 166.1, 132.8, 130.7, 129.7, 128.3, 72.1, 38.3, 34.3, 32.6, 28.1, 22.6, 22.6 ppm.

HRMS [M+H]$^+$ m/z calc'd for $[C_{27}H_{37}O_4]^+$ expect 425.2692 found 425.2689

Chiral SFC Analysis CHIRALPAK IE (CO_2:MeOH, 97:03, 2.5 ml min^{-1}, 40 °C, 225 nm) t_R = 8.2 (major) minutes, 99% *ee*.

(3S,5S)-1,7-dicyclobutylheptane-3,5-diol (1g)

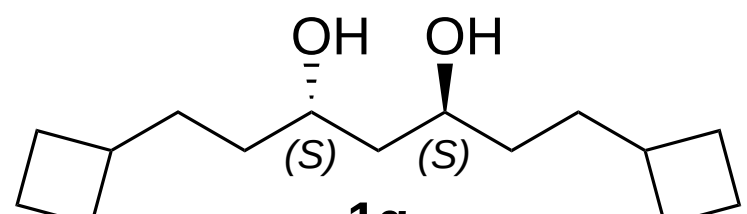

1g

Prepared according to **General Procedure 7** using diol substrate *rac*-**1g** (24.0 mg, 0.1 mmol). The reaction was carried out a concentration of 0.0125 M, using 8 ml of acetone in a 6 dram vial sealed with a B19 suba seal. Crude NMR yields were obtained [47% anti, 29% syn], then the crude product was purified by silica gel chromatography (elution with 5% Acetone in Pet. Ether) to give (3S,5S)-1,7-dicyclobutylheptane-3,5-diol as a white solid (6.6 mg, 0.028 mmol, 28%, 88% *ee*).

^{1}H NMR (700 MHz, $CDCl_3$) δ 3.93 – 3.87 (m, 2H), 2.25 (sep, *J* = 7.6 Hz, 2H), 2.13 (br s, 2H) 2.07 – 2.00 (m, 4H), 1.88 – 1.76 (m, 4H), 1.62 – 1.54 (m, 5H), 1.53 – 1.32 (m, 8H) ppm.

^{13}C NMR (176 MHz, $CDCl_3$) δ 69.6, 42.4, 36.1, 35.1, 33.2, 28.4, 28.4, 18.5 ppm.

$[\alpha]_D^{25.0}$ = + 4.4 (*c* 0.50, $CHCl_3$)

(3*S*,5*S*)-1,7-dicyclobutylheptane-3,5-diyl dibenzoate (1g-Bz)

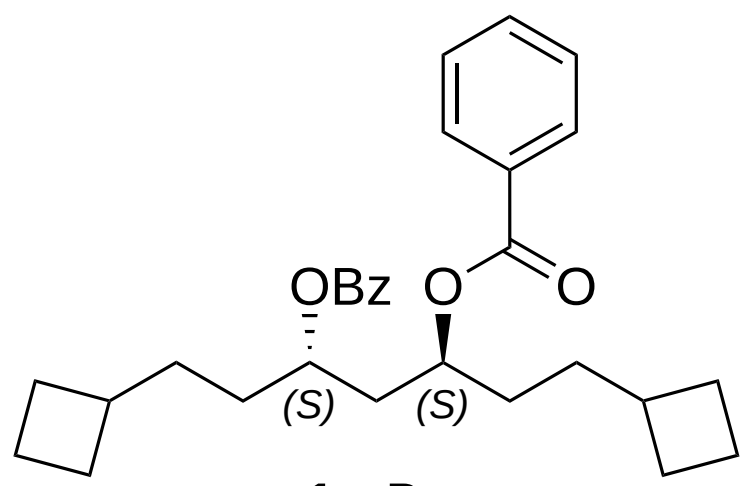

1g-Bz

Prepared according to **General Procedure 8** using **1g** (4.8 mg, 0.02 mmol), DMAP (0.7 mg, 0.0057 mmol), triethylamine (0.02 ml, 0.14 mmol), benzoyl chloride (0.01 ml, 0.06 mmol) in DCM (0.08 ml) Purified *via* preparative thin layer chromatography (15% Acetone in Hexane) to give (3*S*,5*S*)-1,7-dicyclobutylheptane-3,5-diyl dibenzoate as a colourless oil (0.7 mg, 0.002 mmol, 8%).

^{1}H NMR (700 MHz, $CDCl_3$) δ 7.95 (d, *J* = 7.1 Hz, 4H), 7.49 (t, *J* = 7.4 Hz, 2H), 7.35 (t, *J* = 7.8 Hz, 4H), 5.22 (p, *J* = 6.1 Hz, 2H), 2.23 (sep, *J* = 7.8 Hz, 2H), 2.07 (t, *J* = 6.1 Hz, 2H), 2.03 – 1.96 (m, 4H), 1.85 – 1.72 (m, 4H), 1.69 – 1.50 (m, 8H), 1.50 – 1.40 (m, 4H) ppm.

^{13}C NMR (176 MHz, $CDCl_3$) δ 166.2, 132.8, 130.7, 129.7, 128.3, 71.8, 38.4, 35.9, 32.5, 32.3, 28.3, 28.3, 18.5 ppm.

HRMS $[M+H]^+$ m/z calc'd for $[C_{29}H_{37}O_4]^+$ expect 449.2692 found 449.2692

Chiral SFC Analysis CHIRALPAK IJ (CO_2:MeOH, 97:03, 2.5 ml min^{-1}, 40 °C, 226 nm) t_R = 4.0 (major), 4.4 (minor) minutes, 88% *ee*.

(2S,4S)-1,5-dicyclohexylpentane-2,4-diol (1h)

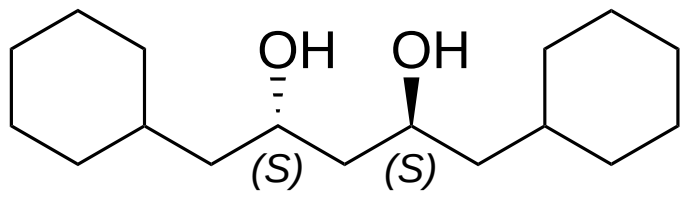

1h

Prepared according to **General Procedure 7** using diol substrate *rac*-**1h** (26.8 mg, 0.1 mmol). The reaction was carried out a concentration of 0.0125 M, using 8 ml of acetone in a 6 dram vial sealed with a B19 suba seal. Crude NMR yields were obtained [53% anti, 25% syn], then the crude product was purified by silica gel chromatography (elution with 5% Acetone in Pet. Ether) to give (2S,4S)-1,5-dicyclohexylpentane-2,4-diol as a white solid (8.4 mg, 0.031 mmol, 31%, 70% *ee*).

^{1}H NMR (700 MHz, $CDCl_3$) δ 4.10 – 4.04 (m, 2H), 2.29 (br s, 2H), 1.79 (br d, *J* = 12.8 Hz, 2H), 1.72 – 1.62 (m, 8H), 1.56 (t, *J* = 5.6 Hz, 2H), 1.49 – 1.37 (m, 4H), 1.29 – 1.20 (m, 6H), 1.19 – 1.11 (m, 2H), 0.99 – 0.91 (m, 2H), 0.87 (qd, *J* = 12.1, 3.4 Hz, 2H) ppm.

^{13}C NMR (176 MHz, $CDCl_3$) δ 67.0, 45.5, 43.5, 34.3, 34.2, 33.2, 26.7, 26.5, 26.4 ppm.

$[\alpha]_D^{25.0}$ = + 8.4 (*c* 0.25, $CHCl_3$)

(2S,4S)-1,5-dicyclohexylpentane-2,4-diyl dibenzoate (1h-Bz)

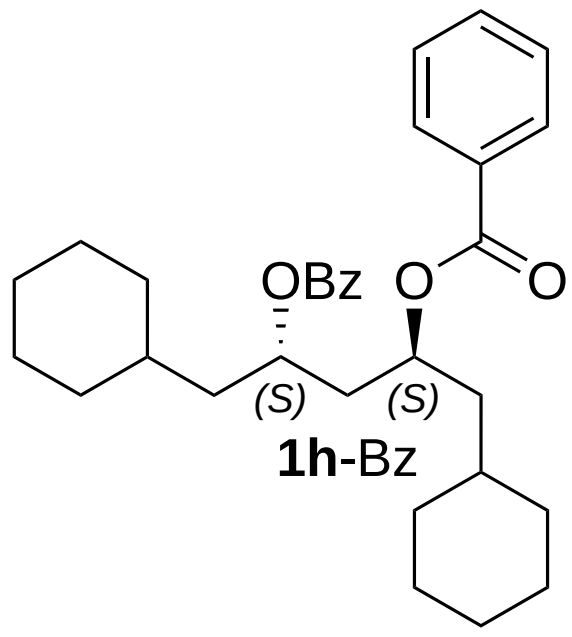

1h-Bz

Prepared according to **General Procedure 8** using **1h** (5.4 mg, 0.02 mmol), DMAP (0.7 mg, 0.0057 mmol), triethylamine (0.02 ml, 0.14 mmol), benzoyl chloride (0.01 ml, 0.06 mmol) in DCM (0.08 ml) Purified *via* preparative thin layer chromatography (15% Acetone in Hexane) to give (2S,4S)-1,5-dicyclohexylpentane-2,4-diyl dibenzoate as a colourless oil (7.4 mg, 0.016 mmol, 80%).

^{1}H NMR (700 MHz, $CDCl_3$) δ 7.93 (app dd, *J* = 8.3, 1.3 Hz, 4H), 7.47 (app t, *J* = 7.4 Hz, 2H), 7.33 (app t, *J* = 7.8 Hz, 4H), 5.39 – 5.31 (m, 2H), 2.07 (t, *J* = 6.0 Hz, 2H), 1.81 (br d, *J* = 12.6 Hz, 2H), 1.77 – 1.57 (m, 10H), 1.50 (ddd, *J* = 14.0, 7.7, 5.3 Hz, 2H), 1.40 – 1.30 (m, 2H), 1.27 – 1.06 (m, 6H), 1.02 – 0.93 (m, 2H), 0.93 – 0.83 (m, 2H) ppm.

^{13}C NMR (176 MHz, $CDCl_3$) δ 166.1, 132.7, 130.7, 129.7, 128.3, 70.0, 42.4, 39.4, 34.3, 33.7, 33.3, 26.6, 26.3, 26.3 ppm.

HRMS $[M+Na]^+$ m/z calc'd for $[C_{31}H_{40}O_4Na]^+$ expect 499.2824 found 499.2801

Chiral SFC Analysis CHIRALPAK IE (CO_2:MeOH, 90:10, 2.5 ml min^{-1}, 40 °C, 226 nm) t_R = 6.7 (major), 7.3 (minor) minutes, 70% *ee*.

(3*S*,5*S*)-1,7-dicyclohexylheptane-3,5-diol (1i)

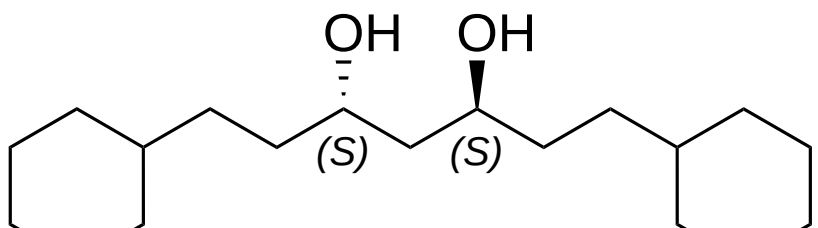

1i

Prepared according to **General Procedure 7** using diol substrate *rac*-**1i** (29.6 mg, 0.1 mmol). The reaction was carried out a concentration of 0.0125 M, using 8 ml of acetone in a 6 dram vial sealed with a B19 suba seal. Crude NMR yields were obtained [47% anti, 40% syn], then the crude product was purified by silica gel chromatography (elution with 5% Acetone in Pet. Ether) to give (3*S*,5*S*)-1,7-dicyclohexylheptane-3,5-diol as a white solid (7.8 mg, 0.026 mmol, 26%, 94% *ee*).

^{1}H NMR (700 MHz, $CDCl_3$) δ 3.90 (p, 2H), 1.70 (br t, *J* = 13.0 Hz, 8H), 1.64 (br d, *J* = 12.9 Hz, 2H), 1.61 (t, *J* = 5.5 Hz , 2H), 1.57 – 1.50 (m, 2H), 1.50 – 1.44 (m, 2H), 1.34 – 1.27 (m, 2H), 1.27 – 1.10 (m, 10H), 0.93 – 0.84 (m, 4H) ppm.

^{13}C NMR (176 MHz, $CDCl_3$) δ 70.0, 42.4, 37.9, 35.0, 33.6, 33.6, 33.5, 26.8, 26.5 ppm.

$[\alpha]_D^{25.0}$ = + 4.5 (*c* 0.20, $CHCl_3$)

(3*S*,5*S*)-1,7-dicyclohexylheptane-3,5-diyl dibenzoate (1i-Bz)

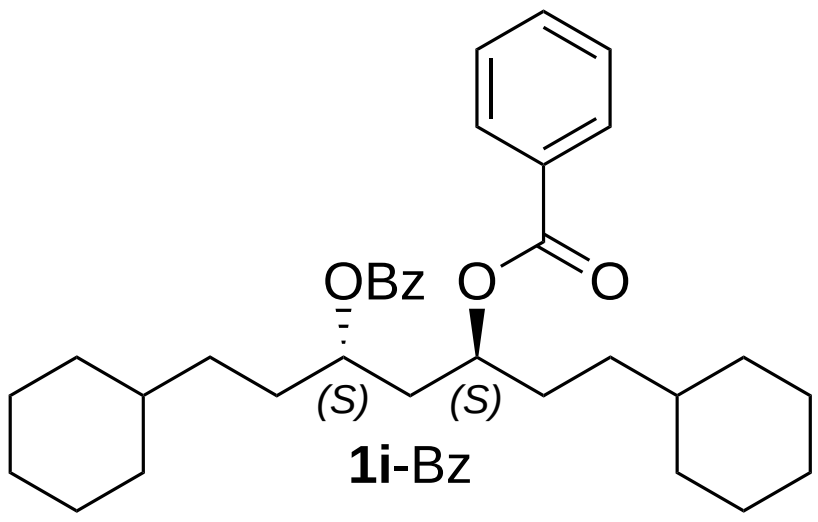

1i-Bz

Prepared according to **General Procedure 8** using **1i** (5.9 mg, 0.02 mmol), DMAP (0.7 mg, 0.0057 mmol), triethylamine (0.02 ml, 0.14 mmol), benzoyl chloride (0.01 ml, 0.06 mmol) in DCM (0.08 ml) Purified *via* preparative thin layer chromatography (15% Acetone in Hexane) to give (3*S*,5*S*)-1,7-dicyclohexylheptane-3,5-diyl dibenzoate as a colourless oil (5.4 mg, 0.011 mmol, 53%).

^{1}H NMR (700 MHz, $CDCl_3$) δ 7.95 (app d, *J* = 7.9 Hz, 4H), 7.48 (t, *J* = 7.4 Hz, 2H), 7.34 (t, *J* = 7.8 Hz, 4H), 5.22 (p, *J* = 6.1 Hz, 2H), 2.08 (t, *J* = 6.0 Hz, 2H), 1.80 – 1.73 (m, 2H), 1.73 – 1.63 (m, 10H), 1.61 (br d, *J* = 13.2 Hz, 2H), 1.31 – 1.06 (m, 12H), 0.89 – 0.80 (m, 2H) ppm.

^{13}C NMR (176 MHz, $CDCl_3$) δ 166.2, 132.8, 130.7, 129.7, 128.3, 72.2, 38.2, 37.7, 33.4, 33.4, 32.9, 32.0, 26.8, 26.5 ppm.

HRMS $[M+Na]^+$ m/z calc'd for $[C_{33}H_{44}O_4Na]^+$ expect 527.3132 found 527.3138

Chiral SFC Analysis CHIRALPAK IG (CO_2:MeOH, 90:10, 2.5 ml min^{-1}, 40 °C, 226 nm) t_R = 8.7 (minor), 10.7 (major) minutes, 94% *ee*.

(4S,6S)-1,9-dicyclohexylnonane-4,6-diol (1j)

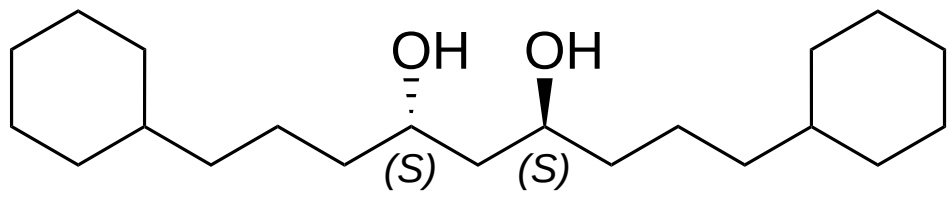

1j

Prepared according to **General Procedure 7** using diol substrate *rac*-**1j** (32.5 mg, 0.1 mmol). The reaction was carried out a concentration of 0.0125 M, using 8 ml of acetone in a 6 dram vial sealed with a B19 suba seal. Crude NMR yields were obtained [73% anti, 23% syn], then the crude product was purified by silica gel chromatography (elution with 5% Acetone in Pet. Ether) to give (4S,6S)-1,9-dicyclohexylnonane-4,6-diol as a white solid (14 mg, 0.043 mmol, 43%, 40% *ee*).

^{1}H NMR (700 MHz, $CDCl_3$) δ 3.96 – 3.91 (m, 2H), 2.18 (s, 2H), 1.72 – 1.66 (m, 8H), 1.64 (br d, *J* = 12.4 Hz, 2H), 1.60 (t, *J* = 5.6 Hz, 1H), 1.53 – 1.49 (m, 2H), 1.46 – 1.38 (m, 4H), 1.35 – 1.27 (m, 2H), 1.25 – 1.10 (m, 12H), 0.92 – 0.82 (m, 4H) ppm.

^{13}C NMR (176 MHz, $CDCl_3$) δ 69.7, 42.4, 38.0, 37.8, 37.6, 33.5, 26.9, 26.6, 23.2 ppm.

$[\alpha]_D^{25.0}$ = - 3.2 (*c* 0.16, $CHCl_3$)

(4*S*,6*S*)-1,9-dicyclohexylnonane-4,6-diyl dibenzoate Number (1j-Bz)

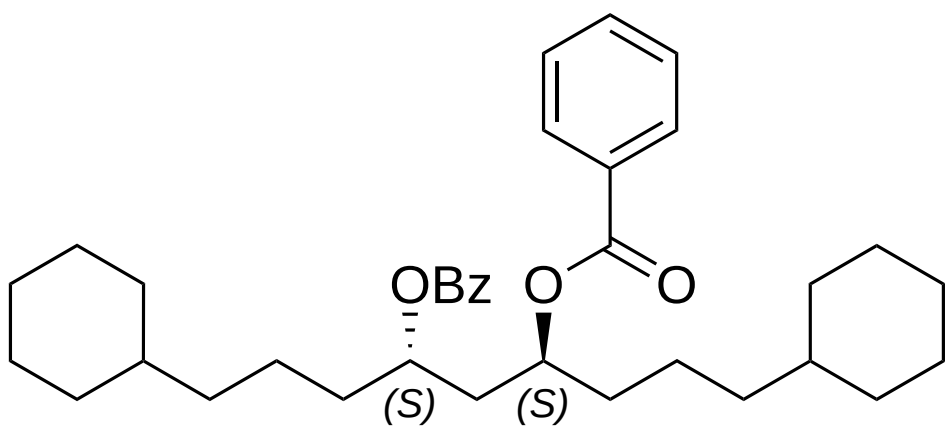

1j-Bz

Prepared according to **General Procedure 8** using **1j** (6.5 mg, 0.02 mmol), DMAP (0.7 mg, 0.0057 mmol), triethylamine (0.02 ml, 0.14 mmol), benzoyl chloride (0.01 ml, 0.06 mmol) in DCM (0.08 ml) Purified *via* preparative thin layer chromatography (15% Acetone in Hexane) to (4*S*,6*S*)-1,9-dicyclohexylnonane-4,6-diyl dibenzoate as a colourless oil (7.2 mg, 0.014 mmol, 68%).

^{1}H NMR (700 MHz, $CDCl_3$) δ 7.94 (app dd, *J* = 8.3, 1.3 Hz, 4H), 7.48 (tt, *J* = 7.5, 1.4 Hz, 2H), 7.34 (app t, *J* = 7.8 Hz, 4H), 5.25 (p, *J* = 6.1 Hz, 2H), 2.08 (t, *J* = 6.1 Hz, 2H), 1.77 – 1.70 (m, 2H), 1.68 – 1.58 (m, 12H), 1.42 – 1.31 (m, 4H), 1.22 – 1.07 (m, 12H), 0.87 – 0.78 (m, 4H) ppm.

^{13}C NMR (176 MHz, $CDCl_3$) δ 166.2, 132.8, 130.7, 129.7, 128.3, 71.9, 38.3, 37.6, 37.4, 35.0, 33.5, 33.4, 26.8, 26.5, 22.6 ppm.

HRMS $[M+H]^+$ m/z calc'd for $[C_{35}H_{49}O_4]^+$ expect 533.3625 found 533.3623

Chiral SFC Analysis CHIRALPAK IK (CO_2:MeOH, 95:05, 2.5 ml min^{-1}, 40 °C, 225 nm) t_R = 6.5 (minor), 7.0 (major) minutes, 40% *ee*.

(3S,5S)-1,7-diphenylheptane-3,5-diol (1k)

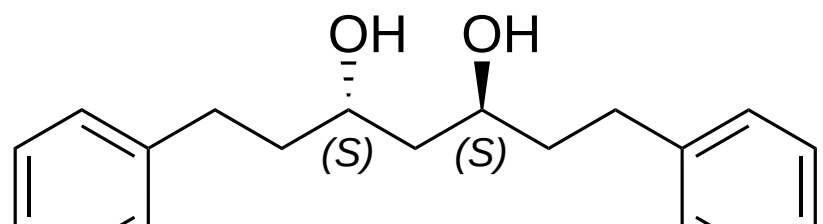

1k

Prepared according to **General Procedure 7** using diol substrate *rac*-**1k** (28.4 mg, 0.1 mmol). The reaction was carried out a concentration of 0.0125 M, using 8 ml of acetone in a 6 dram vial sealed with a B19 suba seal. Crude NMR yields were obtained [47% anti, 35% syn], then the crude product was purified by silica gel chromatography (elution with 5 – 15% Acetone in Pet. Ether) to give (3S,5S)-1,7-diphenylheptane-3,5-diol as a white solid (12 mg, 0.042 mmol, 42%, 84% *ee*).

^{1}H NMR (700 MHz, $CDCl_3$) δ 7.29 (app t, *J* = 7.6 Hz, 4H), 7.22 – 7.17 (m, 6H), 4.02 – 3.97 (m, 2H), 2.78 (ddd, *J* = 13.8, 9.8, 5.5 Hz, 2H), 2.67 (ddd, *J* = 13.8, 9.6, 6.4 Hz, 2H), 2.30 (br s, 2H), 1.87 (dddd, *J* = 13.8, 9.6, 8.3, 5.6 Hz, 2H), 1.77 (dddd, *J* = 13.8, 10.0, 6.2, 4.3 Hz, 2H), 1.68 (t, *J* = 5.6 Hz, 2H) ppm.

^{13}C NMR (176 MHz, $CDCl_3$) δ 142.0, 128.6, 128.5, 126.1, 69.1, 42.7, 39.2, 32.3 ppm.

$[\alpha]_D^{25.0}$ = - 5.2 (*c* 0.85, $CHCl_3$)

*Literature $[\alpha]_D$ values for S,S-**1k**:* – 3.6 (c = 1; T = 25 °C, $CHCl_3$) [55] and – 7.0 (c = 1.0; T = 20 °C, $CHCl_3$)[56]

*Absolute configuration of **1k** therefore assigned as S,S.*

Chiral SFC Analysis CHIRALPAK IA (CO_2:MeOH, 85:15, 2.5 ml min^{-1}, 40 °C, 204 nm) t_R = 5.0 (major), 5.6 (minor) minutes, 84% *ee*.

(4S,6S)-1,9-diphenylnonane-4,6-diol (1l)

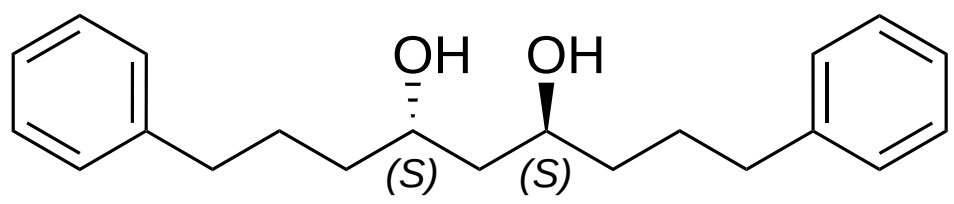

1l

Prepared according to **General Procedure 7** using diol substrate *rac*-**1l** (31.2 mg, 0.1 mmol). The reaction was carried out a concentration of 0.0125 M, using 8 ml of acetone in a 6 dram vial sealed with a B19 suba seal. Crude NMR yields were obtained [39% anti, 33% syn], then the crude product was purified by silica gel chromatography (elution with 5 – 15% Acetone in Pet. Ether) to give (4S,6S)-1,9-diphenylnonane-4,6-diol as a white solid (10.5 mg, 0.034 mmol, 34%, 85% *ee*).

^{1}H NMR (700 MHz, $CDCl_3$) δ 7.28 (app t, *J* = 7.6 Hz, 4H), 7.21 – 7.16 (m, 6H), 3.96 – 3.91 (m, 2H), 2.64 (t, *J* = 7.6 Hz, 4H), 2.07 (br s, 2H), 1.81 – 1.73 (m, 2H), 1.68 – 1.60 (m, 2H), 1.60 – 1.53 (m, 4H), 1.51 – 1.45 (m, 2H) ppm.

^{13}C NMR (176 MHz, $CDCl_3$) δ 142.3, 128.5, 128.5, 125.9, 69.4, 42.5, 37.1, 35.9, 27.7 ppm.

$[\alpha]_D^{25.0}$ = + 2.9 (*c* 0.75, $CHCl_3$)

Chiral HPLC Analysis CHIRALPAK IG (Hexane:iPrOH, 90:10, 1.25 mL min-1, 40 °C) t_R = 9.6 (major), 11.7 (minor) minutes, 85% *ee*.

Scheme 3A - Reaction Profile Starting from *rac*-(**1d**)

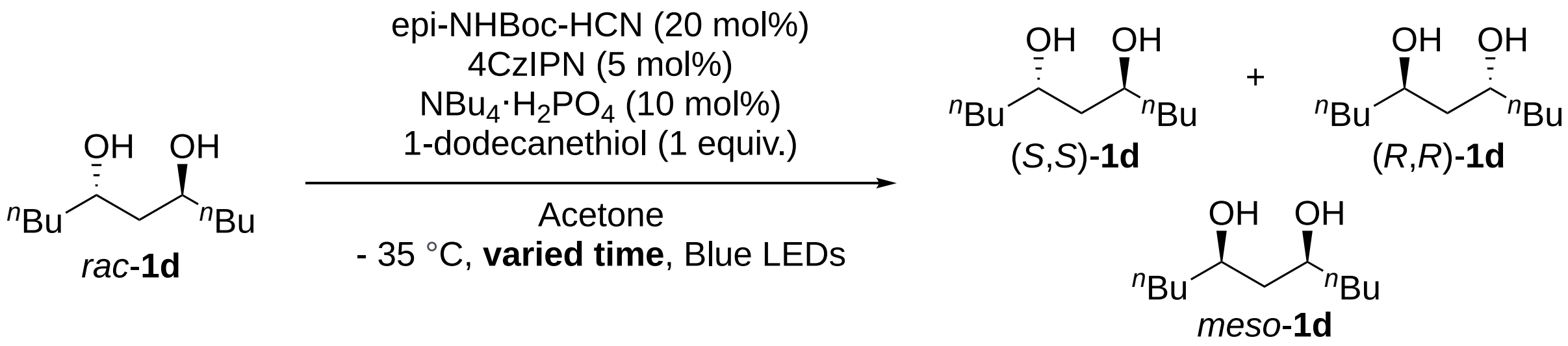

Individual reactions were prepared according to **General Procedure 7** using diol substrate *rac*-**1d** (18.8 mg, 0.1 mmol) and stirred for the specified time. The crude mixture was concentrated *in vacuo* then analysed by ^{1}H NMR using internal standard (CH_2Br_2). The crude reaction mixture was purified by silica gel chromatography (5% Acetone in Pet. Ether) to obtain the pure *anti* diol which was then derivatised according to **General Procedure 8**. The benzoylated product was analysed by chiral SFC to determine the *ee*. The yield of each stereoisomer was determined by using the NMR yield of the diastereomer and the enantiomeric ratio.

time (h)	(*S*,*S*)-1d (%)	(*R*,*R*)-1d (%)	*meso*-1d (%)
0	50	50	0
1	51	21	30
2	52	16	35
3	52	6	42
6	52	4	44
24	52	0	43
48	54	0	43

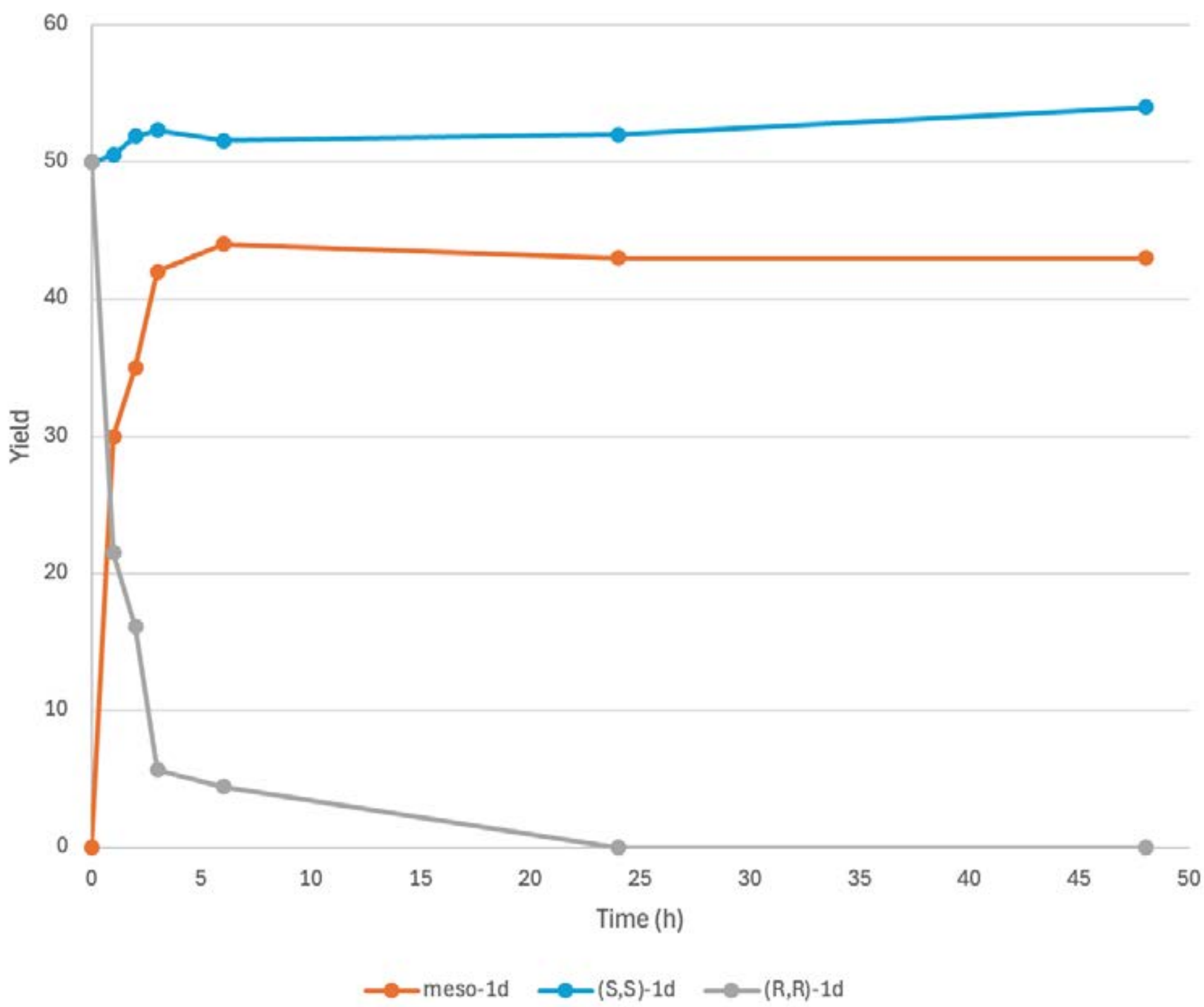

Scheme 3B - Reaction starting from *syn*-1,3-diol (**1d**)

(5S,7S)-undecane-5,7-diol (1d)

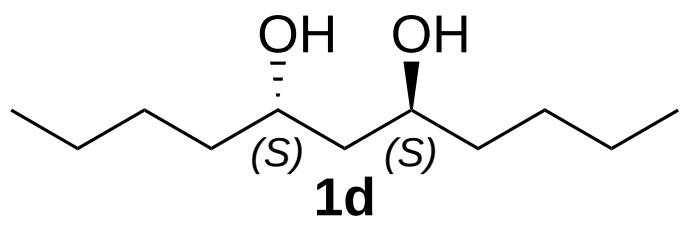

1d

Prepared according to **General Procedure 7** using diol substrate *meso*-**1d** (18.8 mg, 0.1 mmol). Crude NMR yields were obtained [11% anti, 93% syn], then the crude product was purified by silica gel chromatography (elution with 5% Acetone in Pet. Ether) to give (5S,7S)-undecane-5,7-diol as a white solid (2 mg, 0.010 mmol, 10%, 99% *ee*).

^{1}H NMR (700 MHz, $CDCl_3$) δ 3.98 – 3.92 (m, 2H), 1.61 (t, *J* = 5.6 Hz, 2H), 1.58 – 1.51 (m, 2H), 1.50 – 1.44 (m, 2H), 1.43 – 1.27 (m, 8H), 0.91 (t, *J* = 7.1 Hz, 6H) ppm.

^{13}C NMR (176 MHz, $CDCl_3$) δ 69.7, 42.4, 37.4, 28.1, 22.9, 14.2 ppm.

$[\alpha]_D^{25.0}$ = + 5.1 (*c* 0.08, $CHCl_3$)

(5*S*,7*S*)-undecane-5,7-diyl dibenzoate (1d-Bz)

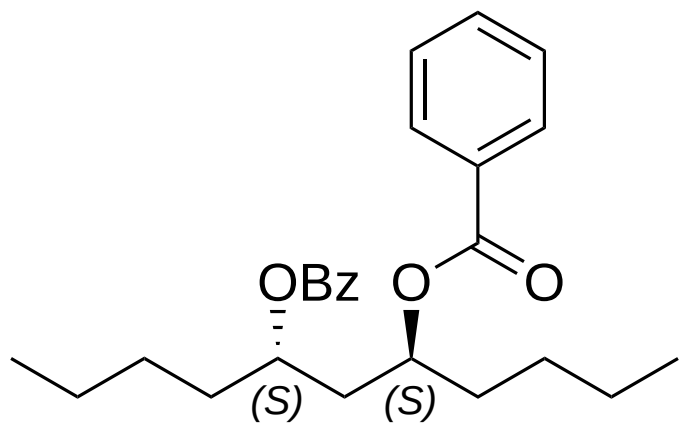

1d-Bz

Prepared according to **General Procedure 8** using (*S*,*S*)-**1d** (1.9 mg, 0.01 mmol), DMAP (0.3 mg, 0.003 mmol), triethylamine (0.01 ml, 0.07 mmol), benzoyl chloride (0.005 ml, 0.03 mmol) in DCM (0.04 ml) Purified *via* preparative thin layer chromatography (15% Acetone in Hexane) to give (4*R*,6*R*)-2,8-dimethylnonane-4,6-diyl dibenzoate as a colourless oil (3.4 mg, 0.0086 mmol, 86%).

^{1}H NMR (700 MHz, $CDCl_3$) δ 7.95 (d, *J* = 7.8 Hz, 4H), 7.48 (t, *J* = 7.1 Hz, 2H), 7.34 (t, *J* = 7.4 Hz, 4H), 5.26 (p, *J* = 6.3 Hz, 2H), 2.09 (t, *J* = 6.1Hz, 2H), 1.80 – 1.73 (m, 2H), 1.72 – 1.65 (m, 2H), 1.40 – 1.27 (m, 8H), 0.87 (t, *J* = 6.8 Hz, 6H) ppm.

^{13}C NMR (176 MHz, $CDCl_3$) δ 166.2, 132.8, 130.7, 129.7, 128.3, 71.8, 38.4, 34.4, 27.5, 22.7, 14.1 ppm.

Chiral SFC Analysis CHIRALPAK IE (CO_2:MeOH, 97:03, 2.5 ml min^{-1}, 40 °C, 237 nm) t_R = 11.1 (major) minutes, 99% *ee*.

Scheme 3C – Deuterium Studies on Symmetrical 1,3-diol (1d)

(*syn*)-undecane-5,6,6,7-d_4-5,7-diol from deuterated *rac*-1d

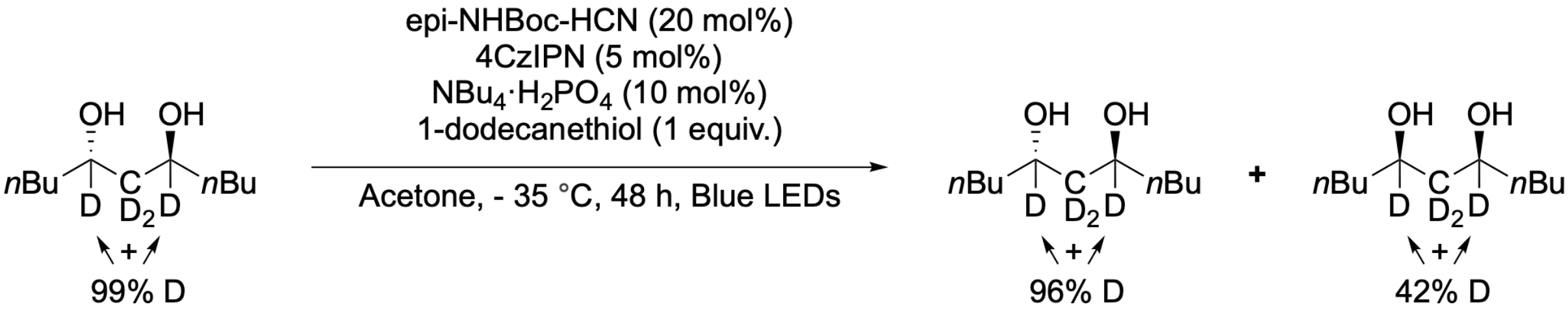

rac-**1d** **1d** *meso*-**1d**

Prepared according to **General Procedure 7** using deuterated diol substrate *rac*-**1d** (19.0 mg, 0.1 mmol). Purification by silica gel chromatography (elution with 5% Acetone in Pet. Ether) to give (*syn*)-undecane-5,6,6,7-d_4-5,7-diol (8 mg, 0.039 mmol, 39%, 42% (α to OH) D incorporation) as a colourless oil and (5*S*,7*S*)-undecane-5,6,6,7-d_4-5,7-diol (9 mg, 0.048 mmol, 48%, 91% *ee*, 96% (α to OH) D incorporation) as a white solid.

(*syn*)-undecane-5,6,6,7-d_4-5,7-diol (*meso*-1d)

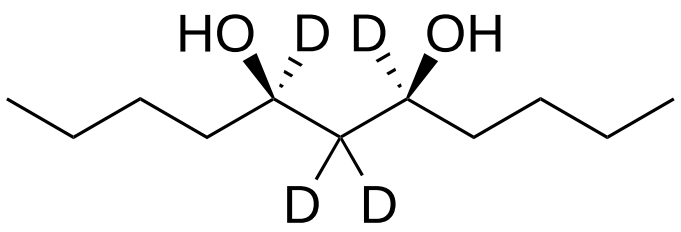

meso-**1d**

^{1}H NMR (700 MHz, $CDCl_3$) δ 3.87 – 3.82 (m, 1.1H), 2.58 (s, 2H), 1.52 – 1.41 (m, 4H), 1.41 – 1.27 (m, 8H), 0.91 (t, *J* = 7.0 Hz, 6H) ppm.

^{13}C NMR (176 MHz, $CDCl_3$) δ 73.3, 73.2, 72.8 (t, *J* = 21.2 Hz), 43.0 – 41.9 (m), 38.1, 38.1, 38.0, 38.0, 27.7, 27.6, 22.8, 14.2 ppm.

^{2}H NMR (107 MHz, $CDCl_3$) δ 3.85 (s, 2D), 1.58 (s, 0.89D), 1.44 (s, 0.98D) ppm.

(5*S*,7*S*)-undecane-5,6,6,7-d_4-5,7-diol (1d)

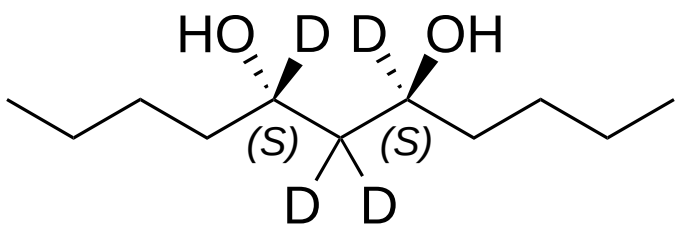

1d

^{1}H NMR (700 MHz, $CDCl_3$) δ 3.95 – 3.92 (m, 0.09H), 2.01 (br s, 2H), 1.56 – 1.51 (m, 2H), 1.49 – 1.43 (m, 2H), 1.43 – 1.27 (m, 8H), 0.91 (t, *J* = 7.1 Hz, 6H) ppm.

^{13}C NMR (176 MHz, $CDCl_3$) δ 69 .6, 69.1 (t, *J* = 21.6 Hz), 42.3 – 41.3 (m), 37.2, 28.1, 22.9, 14.2 ppm.

^{2}H NMR (107 MHz, $CDCl_3$) δ 3.93 (s, 2D), 1.58 (s, 2.04D) ppm.

$[\alpha]_D^{25.0}$ = + 3.2 (*c* 0.22, $CHCl_3$)

(5*S*,7*S*)-undecane-5,7-diyl-5,6,6,7-d_4 dibenzoate (1d-Bz)

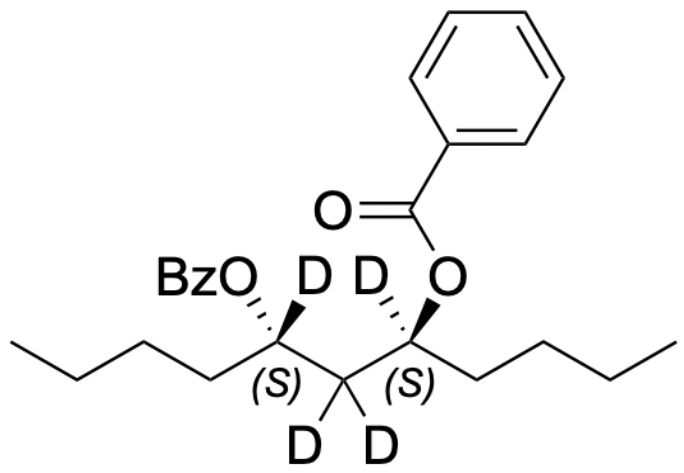

1d-Bz

Prepared according to **General Procedure 8** using deuterated **1d** (3.8 mg, 0.02 mmol), DMAP (0.7 mg, 0.0057 mmol), triethylamine (0.02 ml, 0.14 mmol), benzoyl chloride (0.01 ml, 0.06 mmol) in DCM (0.08 ml) Purified *via* preparative thin layer chromatography (15% Acetone in Hexane) to give (4*R*,6*R*)-2,8-dimethylnonane-4,6-diyl dibenzoate as a colourless oil (4.8 mg, 0.012 mmol, 60%). Deuterium incorporation was determined from the ^{1}H NMR of the isolated material, with the assumption that minimal deuteration would occur at both terminal CH_3 groups.

^{1}H NMR (700 MHz, $CDCl_3$) δ 7.95 (d, *J* = 7.8 Hz, 4H), 7.48 (t, *J* = 7.1 Hz, 2H), 7.34 (t, *J* = 7.2 Hz, 4H), 5.26 – 5.23 (m, 0.10H), 1.79 – 1.72 (m, 2H), 1.71 – 1.65 (m, 2H), 1.41 – 1.27 (m, 8H), 0.87 (t, *J* = 6.7 Hz, 6H) ppm.

^{13}C NMR (176 MHz, $CDCl_3$) δ 166.2, 132.8, 130.7, 129.7, 128.3, 71.8, 71.4 (t, *J* = 21.9 Hz), 38.2 – 37.3 (m), 34.3, 27.4, 22.7, 14.1 ppm.

^{2}H NMR (107 MHz, $CDCl_3$) δ 5.26 (s, 2D), 2.05 (s, 1.99D) ppm.

HRMS [M+H]$^+$ m/z calc'd for $[C_{25}H_{29}D_4O_4]^+$ expect 401.2624 found 401.2621

Chiral SFC Analysis CHIRALPAK IE (CO_2:MeOH, 97:03, 2.5 ml min^{-1}, 40 °C, 231 nm) t_R = 10.8 (major) 12.0 (minor) minutes, 91% *ee*.

(5*S*,7*S*)-undecane-5,6,6,7-d_4-5,7-diol (1d) from deuterated *meso*-1d

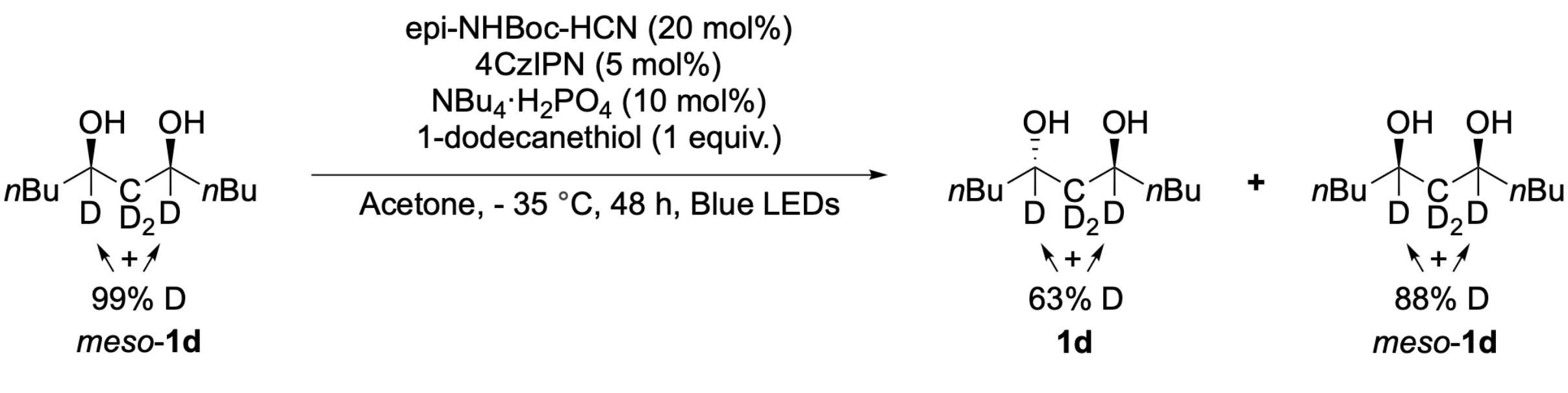

Prepared according to **General Procedure 7** using the deuterated diol substrate *meso*-**1d** (19.0 mg, 0.1 mmol). Purification by silica gel chromatography (elution with 5% Acetone in Pet. Ether) to give (*syn*)-undecane-5,6,6,7-d_4-5,7-diol (19 mg, 0.097 mmol, 97%, 88% (α to OH) D incorporation) as a colourless oil and (5*S*,7*S*)-undecane-5,6,6,7-d_4-5,7-diol (0.4 mg, 0.002 mmol, 2%, 99% *ee*, 63% (α to OH) D incorporation) as a white solid. Deuterium incorporation was determined from the ^{1}H NMR of the isolated material, with the assumption that minimal deuteration would occur at both terminal CH_3 groups.

(*syn*)-undecane-5,6,6,7-d_4-5,7-diol (*meso*-1d)

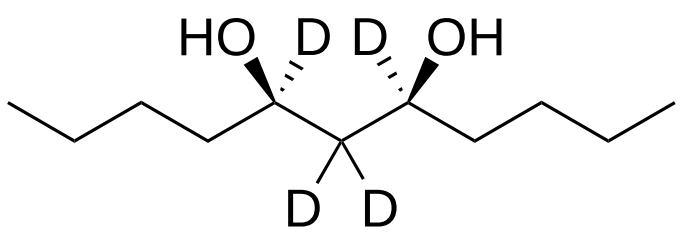

meso-**1d**

^{1}H NMR (700 MHz, $CDCl_3$) δ 3.85 – 3.82 (m, 0.24H), 3.02 (br s, 2H), 1.50 – 1.40 (m, 4H), 1.40 – 1.26 (m, 8H), 0.90 (t, *J* = 7.1 Hz, 6H) ppm.

^{13}C NMR (176 MHz, $CDCl_3$) δ 73.2, 72.8 (t, *J* = 21.4 Hz), 42.6 – 41.7 (m), 38.0, 37.9, 27.6, 22.8, 14.2 ppm.

^{2}H NMR (107 MHz, $CDCl_3$) δ 3.84 (s, 2D), 1.58 (s, 1.03D), 1.43 (s, 1.06D) ppm.

(5*S*,7*S*)-undecane-5,6,6,7-d_4-5,7-diol (1d)

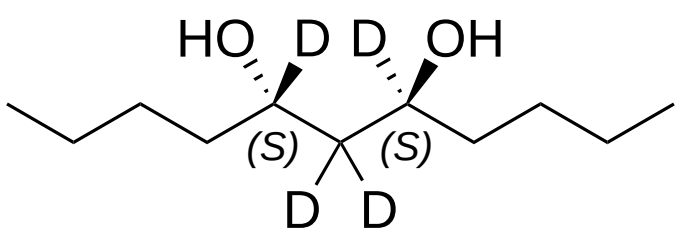

1d

^{1}H NMR (700 MHz, $CDCl_3$) δ 3.95 – 3.91 (m, 0.75H), 1.68 (br s, 2H), 1.56 – 1.50 (m, 2H), 1.49 – 1.43 (m, 2H), 1.43 – 1.27 (m, 8H), 0.91 (t, J = 7.1 Hz, 6H) ppm.

^{13}C NMR Carried forward to benzoylation without carbon obtained

^{2}H NMR (107 MHz, $CDCl_3$) δ 3.94 (s, 2D), 1.59 (s, 5.43D) ppm.

(5*S*,7*S*)-undecane-5,7-diyl-5,6,6,7-d_4 dibenzoate (1d-Bz)

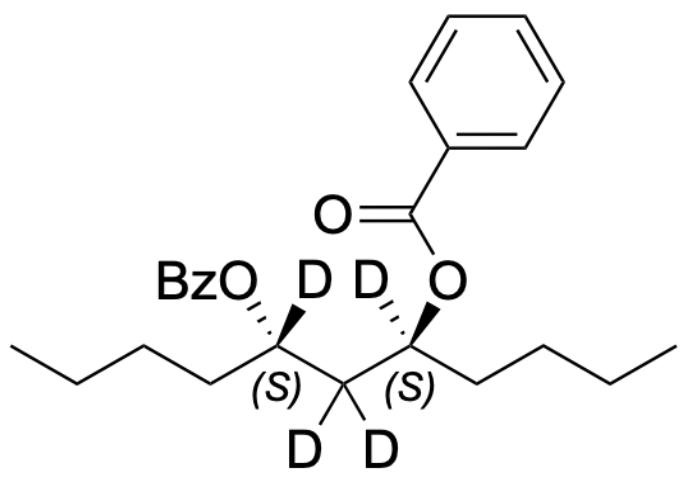

1d-Bz

Prepared according to **General Procedure 8** using deuterated **1d** (0.4 mg, 0.002 mmol), DMAP (0.1 mg, 0.0005 mmol), triethylamine (2 μl, 0.01 mmol), benzoyl chloride (1 μl, 0.006 mmol) in DCM (0.01 ml) Purified *via* preparative thin layer chromatography (15% Acetone in Hexane) to give (4*R*,6*R*)-2,8-dimethylnonane-4,6-diyl dibenzoate as a colourless oil (0.5 mg, 0.001 mmol, 63%).

^{1}H NMR (700 MHz, $CDCl_3$) δ 7.95 (d, *J* = 8.2 Hz, 4H), 7.48 (t, *J* = 7.4 Hz, 2H), 7.34 (t, *J* = 7.8 Hz, 4H), 5.26 – 5.23 (m, 1H) ppm.

^{13}C NMR (176 MHz, $CDCl_3$) δ 166.2, 132.8, 130.7, 129.7, 128.3, 34.3, 27.4, 22.7, 14.1 ppm.

Chiral SFC Analysis CHIRALPAK IE (CO_2:MeOH, 97:03, 2.5 ml min^{-1}, 40 °C, 231 nm) t_R = 11.3 (major) minutes, 99% *ee*.

Scheme 3D – Giese addition

Giese reaction addition (Scheme 3D, upper) starting from *rac*-1d:

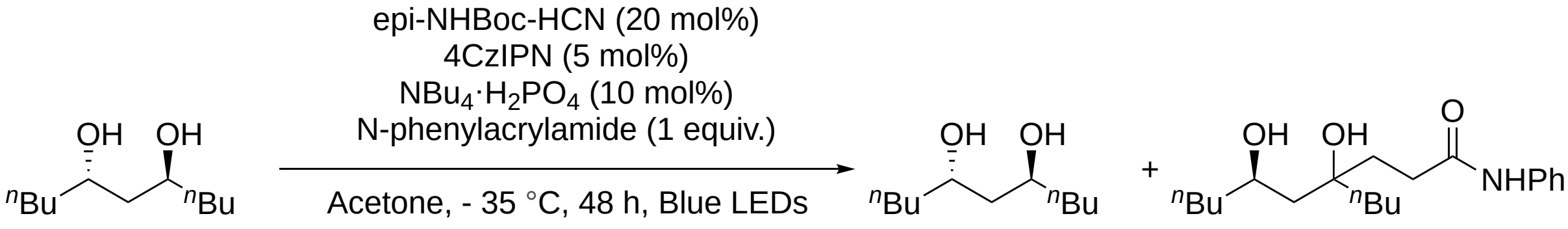

rac-**1d** **1d** **2b**

Rac-**1d** (18.8 mg, 0.10 mmol, 1 eq.), 4CzIPN (3.9 mg, 0.0050 mmol, 5 mol%), *epi*-NHBoc-DHCN (7.9 mg, 0.020 mmol, 20 mol%), tetrabutylammonium phosphate monobasic (3.4 mg, 0.010 mmol, 10 mol%) and *N*-phenylacrylamide (14.7 mg, 0.1 mmol, 1 eq.) were added to a 4.0 mL crimp-top vial. The vial was sealed with a crimp-top cap and evacuated and backfilled with nitrogen 10 times. Degassed acetone (4.0 mL) was added via syringe to the sealed vial (reaction molarity of 0.025 M). The top of the vial was further sealed with Teflon tape then electrical tape and placed in the custom made photoreactor with a positive flow of nitrogen through the apparatus. The reaction vial was cooled to –35 °C and the Kessil® A160WE Tuna blue, LED lamp (100% intensity, 100% ocean blue color) was switched on. After stirring for 48 h the crude mixture was concentrated in vacuo then analysed by ^{1}H-NMR using internal standard (CH_2Br_2) to give NMR yield [65% anti, 31% Giese product]. The crude product was purified by silica gel column chromatography (elution with 5 – 10% Acetone in Pet. Ether) to give (5*S*,7*S*)-undecane-5,7-diol (11.2 mg, 0.059 mmol, 59%, 57% *ee*) with *N*-phenylacrylamide impurity (6.5 mg, 0.044 mmol, 44%) as a white solid and (6*R*)-4-butyl-4,6-dihydroxy-N-phenyldecanamide (2:1 d.r., 4.9 mg, 0.015 mmol, 15%, 99 & 99% *ee*) as a mixture of diastereomers and a yellow oil.

(5*S*,7*S*)-undecane-5,7-diol (1d) with *N*-phenylacrylamide impurity (*N*-PhAcr)

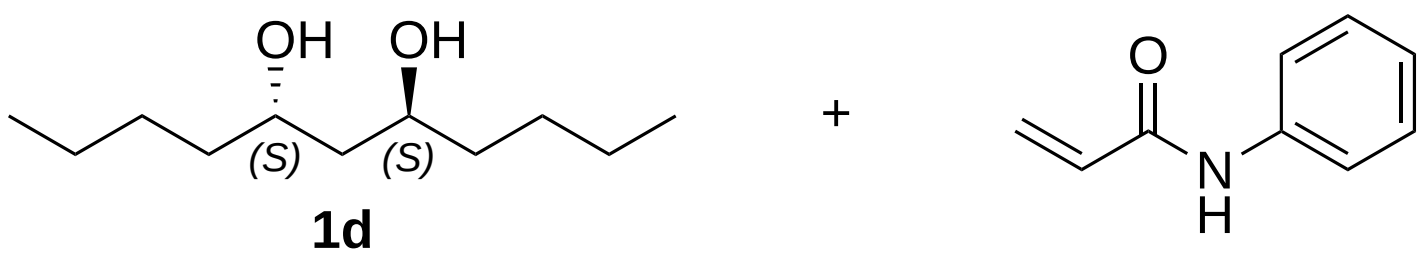

1d

N-phenylacrylamide

^{1}H NMR (700 MHz, $CDCl_3$) δ 7.58 (br d, *J* = 7.4 Hz, 2H ***N*-PhAcr**), 7.32 (t, *J* = 7.8 Hz, 2H ***N*-PhAcr**), 7.12 (t, *J* = 7.1 Hz, 1H ***N*-PhAcr**), 6.43 (d, *J* = 16.8 Hz, 1H ***N*-PhAcr**), 6.27 (dd, *J* = 16.9, 10.3 Hz, 1H ***N*-PhAcr**), 5.75 (d, *J* = 10.2 Hz, 1H ***N*-PhAcr**), 3.96 – 3.91 (m, 2H), 2.50 (br s, 2H), 1.60 (t, *J* = 5.6 Hz, 2H), 1.57 – 1.50 (m, 2H), 1.48 – 1.42 (m, 2H), 1.42 – 1.24 (m, 8H), 0.90 (t, *J* = 7.1 Hz, 6H) ppm.

^{13}C NMR (176 MHz, $CDCl_3$) δ 163.7 (***N*-PhAcr**), 137.9 (***N*-PhAcr**), 131.3 (***N*-PhAcr**), 129.1 (***N*-PhAcr**), 127.9 (***N*-PhAcr**), 124.7 (***N*-PhAcr**), 120.1 (***N*-PhAcr**), 69.6, 42.4, 37.3, 28.1, 22.8, 14.2 ppm.

$[\alpha]_D^{25.0}$ = + 5.8 (*c* 0.66, $CHCl_3$)

(5*S*,7*S*)-undecane-5,7-diyl dibenzoate (1d-Bz)

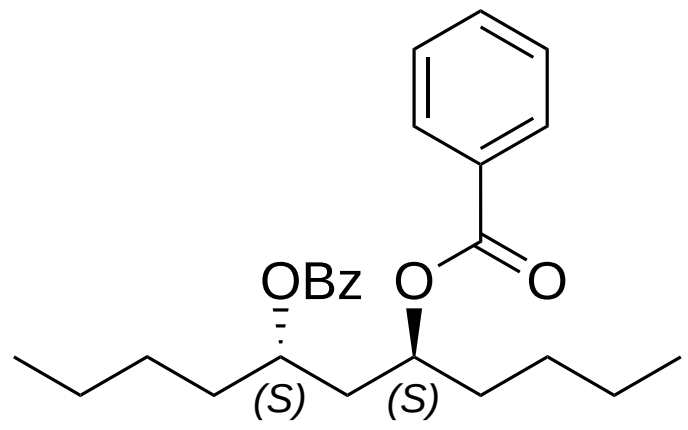

1d-Bz

Prepared according to **General Procedure 8** using **1d** (3.8 mg, 0.02 mmol), DMAP (0.7 mg, 0.0057 mmol), triethylamine (0.02 ml, 0.14 mmol), benzoyl chloride (0.01 ml, 0.06 mmol) in DCM (0.08 ml). Purified *via* preparative thin layer chromatography (15% Acetone in Hexane) to give (5*S*,7*S*)-undecane-5,7-diyl dibenzoate as a colourless oil (4.0 mg, 0.010 mmol, 50%).

^{1}H NMR (700 MHz, $CDCl_3$) δ 7.95 (d, *J* = 8.0 Hz, 4H), 7.48 (t, *J* = 7.4 Hz, 2H), 7.34 (t, *J* = 7.4 Hz, 4H), 5.26 (p, *J* = 6.1 Hz, 2H), 2.09 (t, *J* = 6.0 Hz, 2H), 1.80 – 1.72 (m, 2H), 1.72 – 1.65 (m, 2H), 1.40 – 1.28 (m, 8H), 0.87 (t, *J* = 6.7 Hz, 6H) ppm.

^{13}C NMR (176 MHz, $CDCl_3$) δ 166.2, 132.8, 130.7, 129.7, 128.3, 71.8, 38.4, 34.4, 27.5, 22.7, 14.1 ppm.

Chiral SFC Analysis CHIRALPAK IE (CO_2:MeOH, 97:03, 2.5 ml min^{-1}, 40 °C, 225 nm) t_R = 11.4 (major), 12.5 (minor) minutes, 57% *ee*.

(6*R*)-4-butyl-4,6-dihydroxy-*N*-phenyldecanamide (2b)

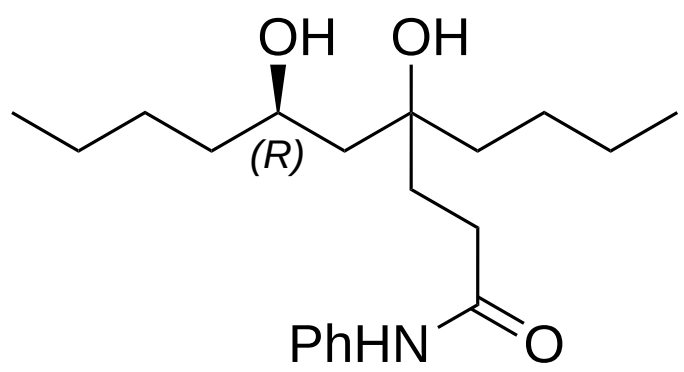

2b

2b-major diastereomer (DR-1) and 2b-minor diastereomer (DR-2)

^{1}H NMR (700 MHz, $CDCl_3$) δ 7.99 (s, 1H, DR-2), 7.66 (s, 1H DR-1), 7.52 – 7.48 (m, 2H DR-1 & 2H DR-2), 7.33 – 7.29 (m, 2H DR-1 & 2H DR-2), 7.12 – 7.08 (m, 1H DR-1 & 1H DR-2), 4.28 (s, 1H DR-2), 4.20 (s, 1H DR-1), 4.07 – 3.98 (m, 1H DR-1 & 1H DR-2), 3.19 – 3.08 (m, 1H DR-1 & 1H DR-2), 2.62 – 2.57 (m, 1H DR-1), 2.57 – 2.48 (m, 2H DR-2), 2.48 – 2.42 (m, 1H DR-1), 2.18 – 2.13 (m, 1H DR-1), 1.97 – 1.92 (m, 1H DR-2), 1.88 – 1.78 (m, 1H DR-1 & 1H DR-2), 1.72 – 1.63 (m, 1H DR-1 & 1H DR-2), 1.63 – 1.58 (m, 2H DR-2), 1.58 – 1.52 (m, 1H DR-1 & 1H DR-2), 1.52 – 1.46 (m, 3H DR-1 & 1H DR-2), 1.44 – 1.22 (m, 9H DR-1 & 8H DR-2), 1.20 – 1.12 (m, 1H DR-2), 0.94 – 0.89 (m, 6H DR-1 & 6H DR-2) ppm.

^{13}C NMR (176 MHz, $CDCl_3$) δ 172.6 (DR-2), 172.4 (DR-1), 138.2 (DR-2), 138.0 (DR-1), 129.1 (DR-1), 129.1 (DR-2), 124.5 (DR-1), 124.3 (DR-2), 120.0 (DR-1), 120.0 (DR-2), 74.8 (DR-2), 74.4 (DR-1), 69.5 (DR-2), 69.5 (DR-1), 44.9 (DR-1), 43.4 (DR-2), 41.0 (DR-1), 38.8 (DR-2), 38.5 (DR-1), 38.4 (DR-2), 35.3 (DR-2), 33.4 (DR-1), 32.5 (DR-1), 32.0 (DR-2), 27.7 (DR-1), 26.8 (DR-2), 26.1 (DR-1 & DR-2), 23.4 (DR-1), 23.4 (DR-2), 22.9 (DR-1 & DR-2), 14.2 (DR-2), 14.2 (DR-1) ppm.

HRMS $[M+H]^+$ m/z calc'd for $[C_{20}H_{34}NO_3]^+$ expect 336.2539 found 336.2537

$[\alpha]_D^{25.0}$ = - 3.4 (*c* 0.35, $CHCl_3$)

Chiral SFC Analysis CHIRALPAK IC (CO_2:MeOH, 90:10, 2.5 ml min^{-1}, 40 °C, 240 nm) Diastereomer 1: t_R = 9.8 (major) minutes, 99% *ee*. Diastereomer 2: t_R = 10.8 (major) minutes, 99% *ee*.

Scheme 3E - Oxidation

Oxidation reaction with DIAD starting from *rac*-1d:

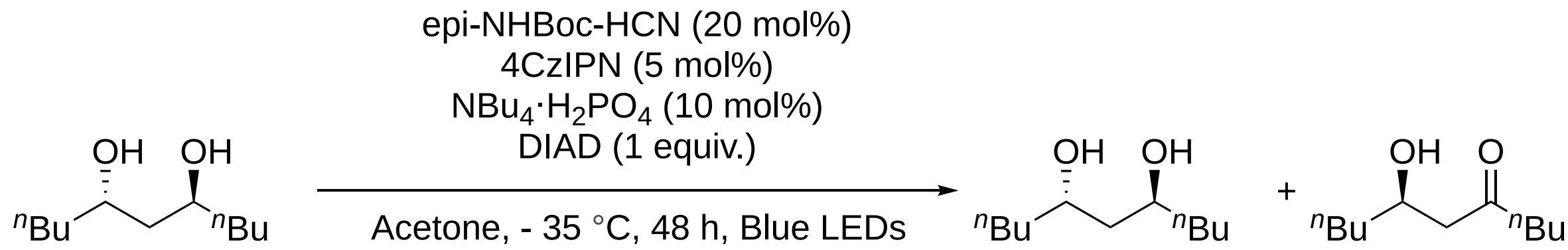

rac-**1d** **1d** **2c**

rac-**1d** (18.8 mg, 0.10 mmol, 1 eq.), 4CzIPN (3.9 mg, 0.0050 mmol, 5 mol%), *epi*-NHBoc-DHCN (7.9 mg, 0.020 mmol, 20 mol%) and tetrabutylammonium phosphate monobasic (3.4 mg, 0.010 mmol, 10 mol%) were added to a 4.0 mL crimp-top vial. The vial was sealed with a crimp-top cap and evacuated and backfilled with nitrogen 10 times. Freshly prepared 0.2 M stock solution of DIAD in degassed acetone (0.5 ml) followed by additional degassed acetone (3.5 ml) was added *via* syringe to the sealed vial (reaction molarity of 0.025 M). The top of the vial was further sealed with Teflon tape then electrical tape and placed in the custom made photoreactor with a positive flow of nitrogen through the apparatus. The reaction vial was cooled to –35 °C and the Kessil® A160WE Tuna blue, LED lamp (100% intensity, 100% ocean blue color) was switched on. After stirring for 48 h the crude mixture was concentrated in vacuo then analysed by ^1H-NMR using internal standard (CH_2Br_2) to give NMR yield [42% anti, 47% oxidation product]. The crude product was purified by silica gel column chromatography (elution with 5% Acetone in Pet. Ether) to give (5*S*,7*S*)-undecane-5,7-diol (8.0 mg, 0.042 mmol, 42%, 99% *ee*) as a white solid and (*R*)-7-hydroxyundecan-5-one (8.1 mg, 0.043 mmol, 43%, 80% *ee*) as a colourless oil.

(5*S*,7*S*)-undecane-5,7-diol (1d)

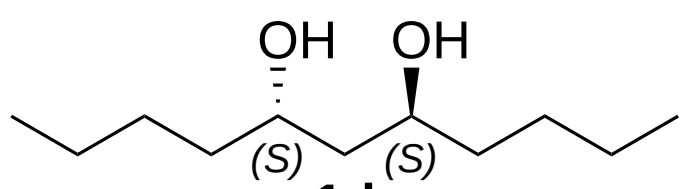

1d

^{1}**H NMR** (700 MHz, $CDCl_3$) δ 3.97 – 3.91 (m, 2H), 2.39 (br s, 2H), 1.60 (t, *J* = 5.6 Hz, 2H), 1.57 – 1.50 (m, 2H), 1.49 – 1.43 (m, 2H), 1.43 – 1.26 (m, 8H), 0.91 (t, *J* = 7.2 Hz, 6H) ppm.

^{13}**C NMR** (176 MHz, $CDCl_3$) δ 69.6, 42.4, 37.3, 28.1, 22.9, 14.2 ppm.

$[\alpha]_D^{25.0}$ = + 4.8 (*c* 0.17, $CHCl_3$)

(5*S*,7*S*)-undecane-5,7-diyl dibenzoate (1d-Bz)

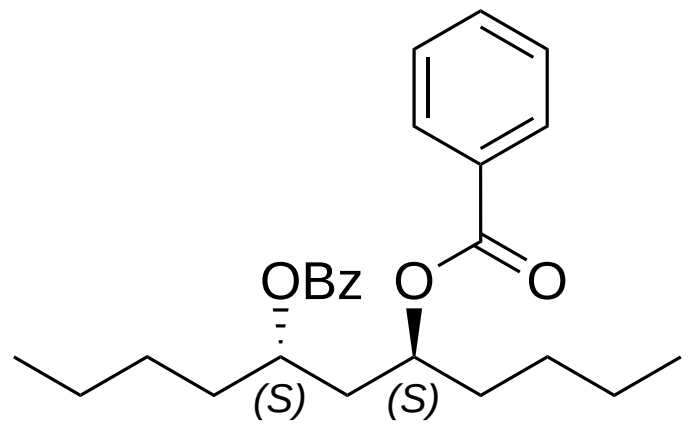

1d-Bz

Prepared according to **General Procedure 8** using **1d** (3.8 mg, 0.02 mmol), DMAP (0.7 mg, 0.0057 mmol), triethylamine (0.02 ml, 0.14 mmol), benzoyl chloride (0.01 ml, 0.06 mmol) in DCM (0.08 ml). Purified *via* preparative thin layer chromatography (15% Acetone in Hexane) to give (5*S*,7*S*)-undecane-5,7-diyl dibenzoate as a colourless oil (5.7 mg, 0.014 mmol, 72%).

^{1}H NMR (700 MHz, $CDCl_3$) δ 7.95 (d, *J* = 8.0 Hz, 4H), 7.48 (td, *J* = 7.4, 0.9 Hz, 2H), 7.34 (t, *J* = 7.3 Hz, 4H), 5.26 (p, *J* = 6.0 Hz, 2H), 2.09 (t, *J* = 5.8 Hz, 2H), 1.80 – 1.72 (m, 2H), 1.72 – 1.65 (m, 2H), 1.42 – 1.27 (m, 8H), 0.87 (t, *J* = 6.7 Hz, 6H) ppm.

^{13}C NMR (176 MHz, $CDCl_3$) δ 166.2, 132.8, 130.7, 129.7, 128.3, 71.8, 38.4, 34.4, 27.5, 22.7, 14.1 ppm.

Chiral SFC Analysis CHIRALPAK IE (CO_2:MeOH, 97:03, 2.5 ml min^{-1}, 40 °C, 225 nm) t_R = 11.1 (major) minutes, 99% *ee*.

(*R*)-7-hydroxyundecan-5-one (2c)

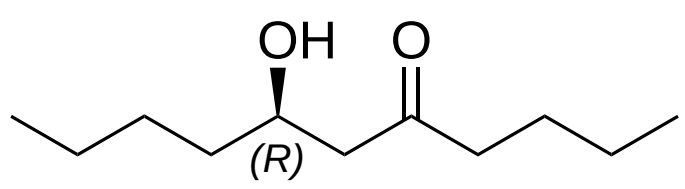

2c

^{1}H NMR (700 MHz, $CDCl_3$) δ 4.04 – 4.00 (m, 1H), 2.59 (dd, *J* = 17.5, 2.6 Hz, 1H), 2.49 (dd, *J* = 17.5, 9.3 Hz, 1H), 2.42 (t, *J* = 7.5 Hz, 2H), 1.56 (app p, *J* = 7.5 Hz, 2H), 1.53 – 1.47 (m, 1H), 1.44 – 1.37 (m, 2H), 1.37 – 1.26 (m, 5H), 0.93 – 0.88 (m, 6H) ppm.

^{13}C NMR (176 MHz, $CDCl_3$) δ 212.8, 67.8, 49.1, 43.5, 36.3, 27.8, 25.9, 22.8, 22.4, 14.2, 14.0 ppm.

$[\alpha]_D^{25.0}$ = - 21.3 (*c* 0.58, $CHCl_3$)

*Literature $[\alpha]_D$ values for R-**2c**: - 34.1 (c = 1; T = 20 ℃, $CHCl_3$)[6] and – 42.0 (c = 1; T = 23 ℃, CH_2Cl_2)[54]*

*Absolute configuration of **2c** therefore assigned as R.*

(*R*)-7-((*tert*-butyldiphenylsilyl)oxy)undecan-5-one (2c-TBDPS)

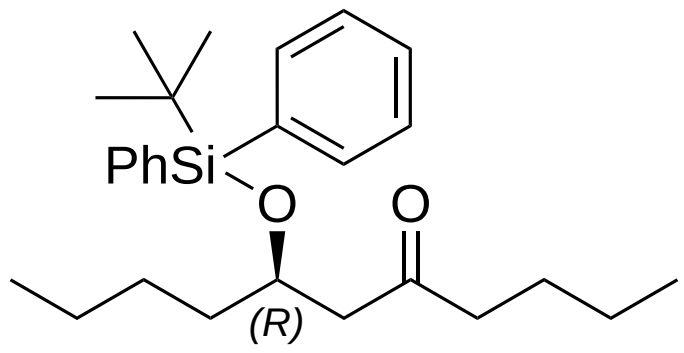

2c-TBDPS

Prepared according to **General Procedure 9** using **2c** (7.1 mg, 0.038 mmol), TBDPSCl (0.01 ml, 0.04 mmol), imidazole (3.8 mg, 0.06 mmol) in DCM (0.8 ml). Purified *via* preparative thin layer chromatography (5% Acetone in Hexane) to give (*R*)-7-((*tert*-butyldiphenylsilyl)oxy)undecan-5-one (1.8 mg, 0.004 mmol, 11%) as a colourless oil.

^{1}H NMR (700 MHz, $CDCl_3$) δ 7.69 – 7.65 (m, 4H), 7.43 – 7.40 (m, 2H), 7.39 – 7.35 (m, 4H), 4.22 (p, *J* = 5.9 Hz, 1H), 2.55 (dd, *J* = 15.6, 6.1 Hz, 1H), 2.45 (dd, *J* = 15.2, 6.1 Hz, 1H), 2.29 – 2.20 (m, 2H), 1.45 – 1.38 (m, 4H), 1.24 – 1.09 (m, 6H), 1.02 (s, 9H), 0.85 (t, *J* = 7.4 Hz, 3H), 0.76 (t, *J* = 7.2 Hz, 3H) ppm.

^{13}C NMR (176 MHz, $CDCl_3$) δ 209.9, 136.0, 134.4, 134.2, 129.7, 129.7, 127.7, 127.6, 70.2, 50.0, 43.8, 37.0, 27.1, 25.7, 22.6, 22.4, 19.5, 14.1, 14.0 ppm.

HRMS [M+Na]$^+$ m/z calc'd for $[C_{27}H_{40}O_2SiNa]^+$ expect 447.2695 found 447.2699

Chiral SFC Analysis CHIRALPAK IA (CO_2:MeOH, 99:01, 2.5 ml min^{-1}, 40 °C, 220 nm) t_R = 5.3 (major), 6.0 (minor) minutes, 80% *ee*.

Oxidation reaction with DIAD starting from *rac*-1e:

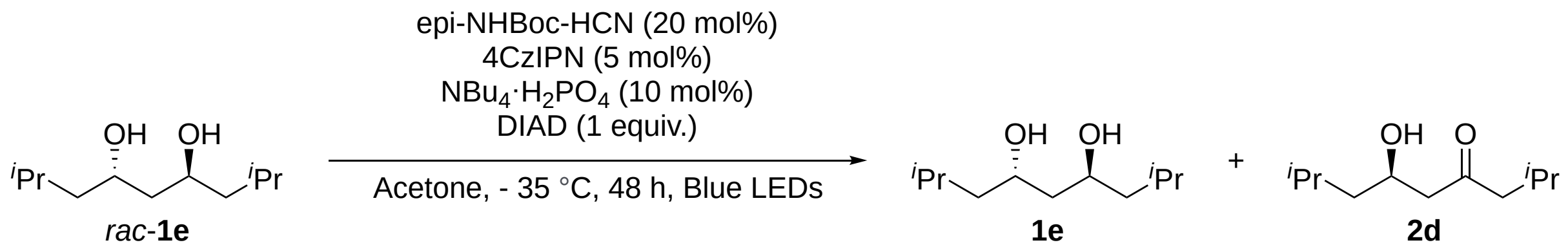

rac-**1e** (18.8 mg, 0.10 mmol, 1 eq.), 4CzIPN (3.9 mg, 0.0050 mmol, 5 mol%), *epi*-NHBoc-DHCN (7.9 mg, 0.020 mmol, 20 mol%) and tetrabutylammonium phosphate monobasic (3.4 mg, 0.010 mmol, 10 mol%) were added to a 4.0 mL crimp-top vial. The vial was sealed with a crimp-top cap and evacuated and backfilled with nitrogen 10 times. Freshly prepared 0.2 M stock solution of DIAD in degassed acetone (0.5 ml) followed by additional degassed acetone (3.5 ml) was added *via* syringe to the sealed vial (reaction molarity of 0.025 M). The top of the vial was further sealed with Teflon tape then electrical tape and placed in the custom made photoreactor with a positive flow of nitrogen through the apparatus. The reaction vial was cooled to –35 °C and the Kessil® A160WE Tuna blue, LED lamp (100% intensity, 100% ocean blue color) was switched on. After stirring for 48 h the crude mixture was concentrated in vacuo then analysed by ^{1}H-NMR using internal standard (CH_2Br_2) to give NMR yield [41% anti, 41% oxidation product]. The crude product was purified by silica gel column chromatography (elution with 5% Acetone in Pet. Ether) to give (4*S*,6*S*)-2,8-dimethylnonane-4,6-diol (8.8 mg, 0.047 mmol, 47%, 97% *ee*) as a white solid and (*R*)-6-hydroxy-2,8-dimethylnonan-4-one (6.8 mg, 0.037 mmol, 37%, 92% *ee*) as a colourless oil.

(4*S*,6*S*)-2,8-dimethylnonane-4,6-diol (1e)

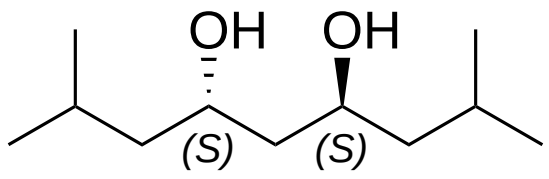

1e

^{1}H NMR (700 MHz, $CDCl_3$) δ 4.07 – 4.01 (m, 2H), 2.30 (d, *J* = 3.6 Hz, 2H), 1.78 – 1.70 (m, 2H), 1.57 (t, *J* = 5.6 Hz, 2H), 1.49 (ddd, *J* = 13.2, 7.7, 6.3 Hz, 2H), 1.24 (ddd, *J* = 13.6, 8.5, 4.9 Hz, 2H), 0.92 (app dd, *J* = 6.6, 3.6 Hz, 12H) ppm.

^{13}C NMR (176 MHz, $CDCl_3$) δ 67.6, 46.8, 43.4, 24.8, 23.4, 22.4 ppm.

$[\alpha]_D^{25.0}$ = + 0.76 (*c* 0.26, $CHCl_3$)

(4*S*,6*S*)-2,8-dimethylnonane-4,6-diyl dibenzoate (1e-Bz)

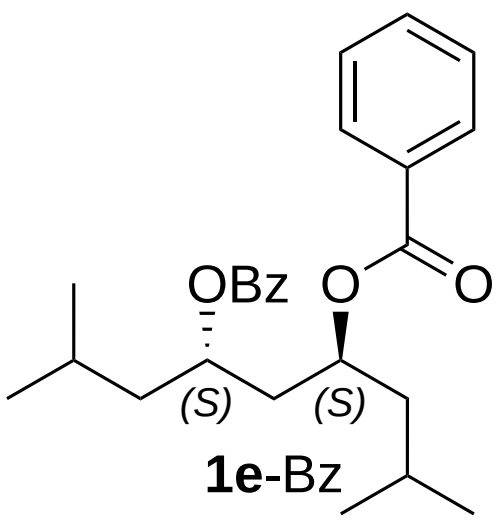

1e-Bz

Prepared according to **General Procedure 8** using **1e** (3.8 mg, 0.02 mmol), DMAP (0.7 mg, 0.0057 mmol), triethylamine (0.02 ml, 0.14 mmol), benzoyl chloride (0.01 ml, 0.06 mmol) in DCM (0.08 ml) Purified *via* preparative thin layer chromatography (15% Acetone in Hexane) to give (4*S*,6*S*)-2,8-dimethylnonane-4,6-diyl dibenzoate as a colourless oil (6.4 mg, 0.016 mmol, 81%).

^{1}H NMR (700 MHz, $CDCl_3$) δ 7.93 (dd, *J* = 8.3, 1.3 Hz, 4H), 7.47 (app t, *J* = 7.4 Hz, 2H), 7.32 (t, *J* = 7.9 Hz, 4H), 5.37 – 5.32 (m, 2H), 2.08 (t, *J* = 6.0 Hz, 2H), 1.77 (ddd, *J* = 14.0, 8.1, 5.9 Hz, 2H), 1.68 (sep, *J* = 6.7 Hz, 2H), 1.48 (ddd, *J* = 13.8, 8.0, 5.1 Hz, 2H), 0.93 (dd, *J* = 6.6, 3.7 Hz, 12H) ppm.

^{13}C NMR (176 MHz, $CDCl_3$) δ 166.2, 132.8, 130.6, 129.6, 128.3, 70.5, 43.8, 39.3, 24.9, 23.1, 22.6 ppm.

Chiral SFC Analysis CHIRALPAK IG (CO_2:MeOH, 97:03, 2.5 ml min^{-1}, 40 °C, 225 nm) t_R = 4.7 (minor), 5.2 (major) minutes, 97% *ee*.

(*R*)-6-hydroxy-2,8-dimethylnonan-4-one (2d)

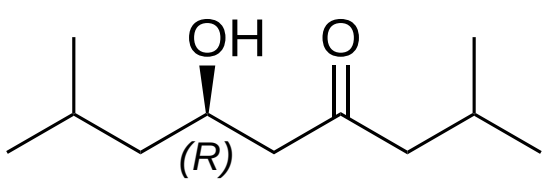

2d

^{1}H NMR (700 MHz, $CDCl_3$) δ 4.15 – 4.09 (m, 1H), 3.02 (d, *J* = 3.4 Hz, 1H), 2.55 (dd, *J* = 17.7, 2.7 Hz, 1H), 2.47 (dd, *J* = 17.7, 9.1 Hz, 1H), 2.30 (d, *J* = 6.8 Hz, 2H), 2.14 (sep, *J* = 6.8 Hz, 1H), 1.83 – 1.75 (m, 1H), 1.46 (ddd, *J* = 13.9, 8.8, 5.3 Hz, 1H), 1.12 (ddd, *J* = 13.5, 8.8, 4.6 Hz, 1H), 0.94 – 0.90 (m, 12H) ppm.

^{13}C NMR (176 MHz, $CDCl_3$) δ 212.5, 65.8, 52.8, 50.1, 45.6, 24.7, 24.5, 23.5, 22.7, 22.7, 22.2 ppm.

$[\alpha]_D^{25.0}$ = - 20.5 (*c* 0.41, $CHCl_3$)

*Literature $[\alpha]_D$ values for R-**2d**: - 26.9 (c = 1.0; T = 20 ℃, $CHCl_3$)*[6]

*Absolute configuration of **2d** therefore assigned as R.*

(*R*)-6-((*tert*-butyldiphenylsilyl)oxy)-2,8-dimethylnonan-4-one (2d-TBDPS)

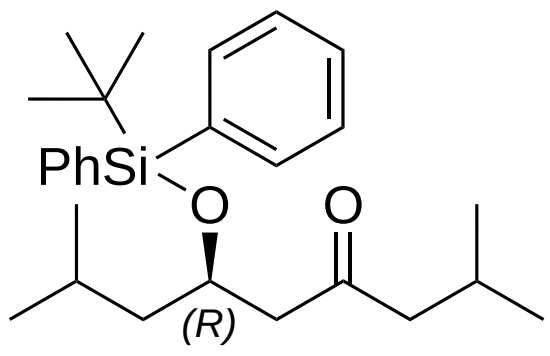

2d-TBDPS

Prepared according to **General Procedure 9** using **2d** (6.1 mg, 0.033 mmol), TBDPSCl (0.01 ml, 0.04 mmol), imidazole (3.3 mg, 0.05 mmol) in DCM (0.7 ml). Purified *via* preparative thin layer chromatography (5% Acetone in Hexane) to give (*R*)-6-((*tert*-butyldiphenylsilyl)oxy)-2,8-dimethylnonan-4-one as a colourless oil (0.8 mg, 0.0019 mmol, 6%).

^{1}H NMR (700 MHz, $CDCl_3$) δ 7.69 (app td, *J* = 8.4, 1.4 Hz, 4H), 7.44 – 7.40 (m, 2H), 7.40 – 7.36 (m, 4H), 4.23 (p, *J* = 6.2 Hz, 1H), 2.52 (dd, *J* = 15.2, 5.7 Hz, 1H), 2.43 (dd, *J* = 15.2, 6.4 Hz, 1H), 2.12 (dd, *J* = 16.2, 7.1 Hz, 1H), 2.07 (dd, *J* = 16.2, 6.5 Hz, 1H), 1.98 (sep, *J* = 6.8 Hz, 1H), 1.60 – 1.54 (m, 1H), 1.34 (ddd, *J* = 13.5, 6.7, 6.7 Hz, 1H), 1.24 (ddd, *J* = 13.9, 7.1, 6.5 Hz, 1H), 1.02 (s, 9H), 0.81 (app t, *J* = 4.7 Hz, 6H), 0.68 (d, *J* = 6.6 Hz, 3H), 0.63 (d, *J* = 6.6 Hz, 3H) ppm.

^{13}C NMR (176 MHz, $CDCl_3$) δ 209.3, 136.1, 136.1, 134.3, 134.2, 129.8, 129.7, 127.7, 127.7, 68.7, 52.9, 51.2, 47.0, 27.1, 24.6, 24.3, 22.7, 22.7, 19.5 ppm.

HRMS $[M+Na]^+$ m/z calc'd for $[C_{27}H_{40}O_2SiNa]^+$ expect 447.2695 found 447.2699

Chiral SFC Analysis CHIRALPAK IJ (CO_2:MeOH, 999:001, 2.5 ml min^{-1}, 40 °C, 214 nm) t_R = 2.0 (major), 2.2 (minor) minutes, 92% *ee*.

Oxidation reaction with DIAD starting from *rac*-1k:

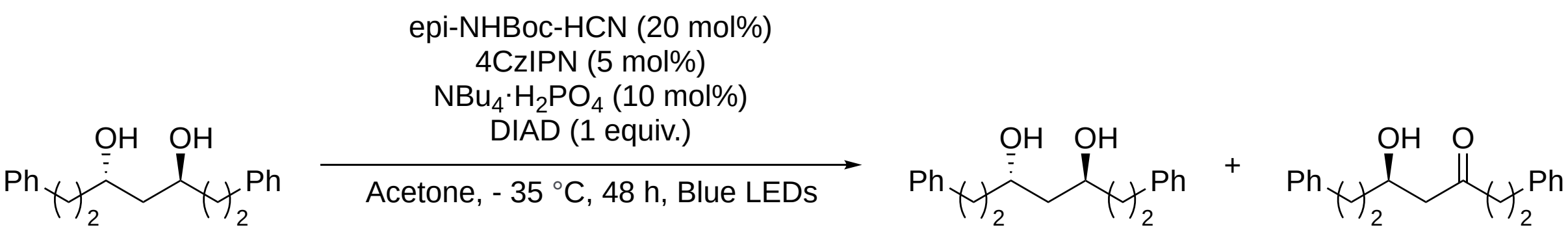

rac-**1k** (28.4 mg, 0.10 mmol, 1 eq.), 4CzIPN (3.9 mg, 0.0050 mmol, 5 mol%), *epi*-NHBoc-DHCN (7.9 mg, 0.020 mmol, 20 mol%) and tetrabutylammonium phosphate monobasic (3.4 mg, 0.010 mmol, 10 mol%) were added to a 6 dram vial. The vial was sealed with a B19 suba seal and evacuated and backfilled with nitrogen 10 times. Freshly prepared 0.2 M stock solution of DIAD in degassed acetone (0.5 ml) followed by additional degassed acetone (7.5 ml) was added *via* syringe to the sealed vial (reaction molarity of 0.0125 M). The top of the vial was further sealed with Teflon tape then electrical tape and placed in the custom made photoreactor with a positive flow of nitrogen through the apparatus. The

reaction vial was cooled to –35 °C and the Kessil® A160WE Tuna blue, LED lamp (100% intensity, 100% ocean blue color) was switched on. After stirring for 48 h the crude mixture was concentrated in vacuo then analysed by ^{1}H-NMR using internal standard (CH_2Br_2) to give NMR yield [40% anti, 44% oxidation product]. The crude product was purified by silica gel column chromatography (elution with 5 – 10% Acetone in Pet. Ether) to give (3S,5S)-1,7-diphenylheptane-3,5-diol (11.2 mg, 0.39 mmol, 39%, 92% *ee*) with Diisopropyl 1,2-hydrazinedicarboxylate impurity (15.1 mg, 0.074 mmol, 74%) as a white solid and (*R*)-5-hydroxy-1,7-diphenylheptan-3-one (12.5 mg, 0.44 mmol, 44%, 93% *ee*) as a white solid.

(3S,5S)-1,7-diphenylheptane-3,5-diol (1k) and Diisopropyl 1,2-hydrazinedicarboxylate

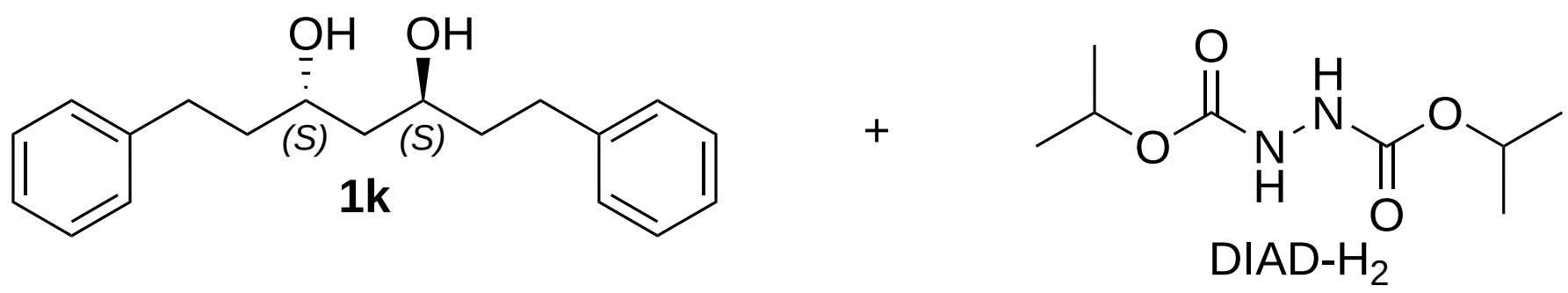

^{1}H NMR (700 MHz, $CDCl_3$) δ 7.28 (app t, *J* = 7.6 Hz, 4H), 7.20 – 7.16 (m, 6H), 4.97 (sep, *J* = 6.2 Hz, 2H DIAD-H_2), 4.02 – 3.95 (m, 2H,), 2.78 (ddd, *J* = 14.2, 9.5, 5.2 Hz, 2H), 2.66 (ddd, *J* = 13.8, 9.8, 6.4 Hz, 2H), 2.30 (br s, 2H), 1.86 (dddd, *J* = 13.8, 9.0, 9.0, 5.3 Hz, 2H), 1.76 (dddd, *J* = 13.8, 10.0, 6.1, 4.1 Hz, 2H), 1.67 (t, *J* = 5.6 Hz, 2H), 1.26 (sep, *J* = 6.4 Hz, 6H DIAD-H_2). ppm.

^{13}C NMR (176 MHz, $CDCl_3$) δ 156.5 (DIAD-H_2), 142.1, 128.6, 128.5, 126.0, 70.2 (DIAD-H_2), 69.0, 42.7, 39.2, 32.3, 22.1 (DIAD-H_2), 22.0 (DIAD-H_2) ppm.

$[\alpha]_D^{25.0}$ = + 24.0 (*c* 0.07, $CHCl_3$)

Chiral SFC Analysis CHIRALPAK IA (CO_2:MeOH, 85:15, 2.5 ml min^{-1}, 40 °C, 204 nm) t_R = 4.9 (major), 5.5 (minor) minutes, 92% *ee*.

(*R*)-5-hydroxy-1,7-diphenylheptan-3-one (2e)

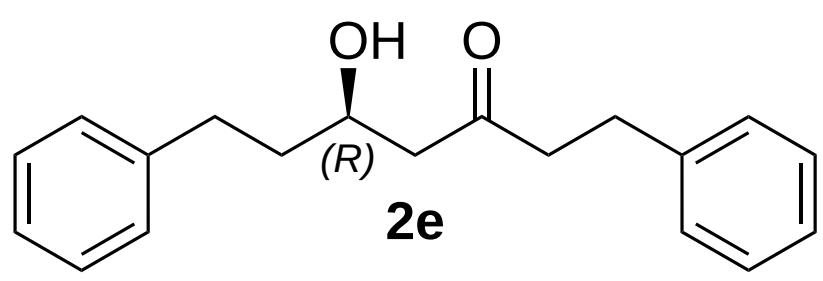

^{1}H NMR (700 MHz, $CDCl_3$) δ 7.28 (app t, *J* = 7.6 Hz, 4H), 7.22 – 7.15 (m, 6H), 4.08 – 4.02 (m, 1H), 3.05 (d, *J* = 3.1 Hz, 1H), 2.90 (t, *J* = 7.6 Hz, 2H), 2.80 (ddd, *J* = 14.1, 9.4, 5.0 Hz, 1H), 2.75 (t, *J* = 7.6 Hz, 2H), 2.67 (ddd, *J* = 13.8, 9.4, 6.9 Hz, 1H), 2.59 – 2.50 (m, 2H), 1.81 (dddd, *J* = 13.9, 9.1, 9.1, 4.9 Hz, 1H), 1.67 (dddd, *J* = 13.6, 10.0, 6.8, 3.8 Hz, 1H) ppm.

^{13}C NMR (176 MHz, $CDCl_3$) δ 211.2, 141.9, 140.8, 128.7, 128.6, 128.6, 128.6, 128.4, 126.4, 126.0, 67.0, 49.4, 45.2, 38.2, 31.9, 29.6 ppm.

$[\alpha]_D^{25.0}$ = -14.1 (*c* 0.85, $CHCl_3$)

Chiral SFC Analysis CHIRALPAK IJ (CO_2:MeOH, 85:15, 2.5 ml min^{-1}, 40 °C, 206 nm) t_R = 6.5 (minor), 7.0 (major) minutes, 93% *ee*.

Scheme 3F – Access to Opposite Enantiomer

(5*R*,7*R*)-undecane-5,7-diol (*R*,*R*-1d)

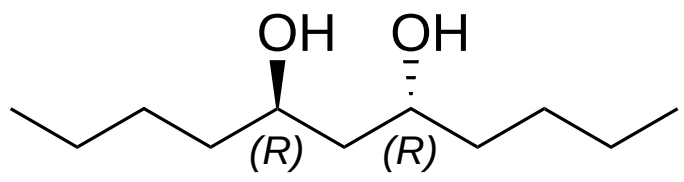

(*R*,*R*)-**1d**

Prepared according to **General Procedure 7** using diol substrate *rac*-**1d** (18.8 mg, 0.1 mmol), *epi*-NHBoc-DHCD (synthesized as previously reported)[1] instead and a concentration of 0.025 M. Purification by silica gel chromatography (elution with 5% acetone in Pet. Ether) to give the title compound as a white solid (9.0 mg, 0.048 mmol, 48%, 99% *ee*).

^{1}H NMR (700 MHz, $CDCl_3$) δ 3.96 – 3.91 (m, 2H, H_5), 2.16 (s, 2H, H_7), 1.61 (dd, *J* = 6.0, 5.3 Hz, 2H, H_6), 1.57 – 1.51 (m, 2H, H_4), 1.49 – 1.43 (m, 2H, $H_{4'}$), 1.43 – 1.26 (m, 8H, $H_2 + H_3$), 0.91 (t, *J* = 7.1 Hz, 6H, H_1).

^{13}C NMR (176 MHz, $CDCl_3$) δ 69.6, 42.4, 37.3, 28.1, 22.8, 14.2.

$[\alpha]_D^{25.0}$ = - 12.3 (*c* 0.18, $CHCl_3$)

*Literature $[\alpha]_D$ values for R,R-**1d**: - 6.0 (c = 1; T = 23* °C, CH_2Cl_2)[54]

*Absolute configuration of (R,R)-**1d** therefore assigned as R,R.*

(5*R*,7*R*)-undecane-5,7-diyl dibenzoate ((*R*,*R*)-1d-Bz)

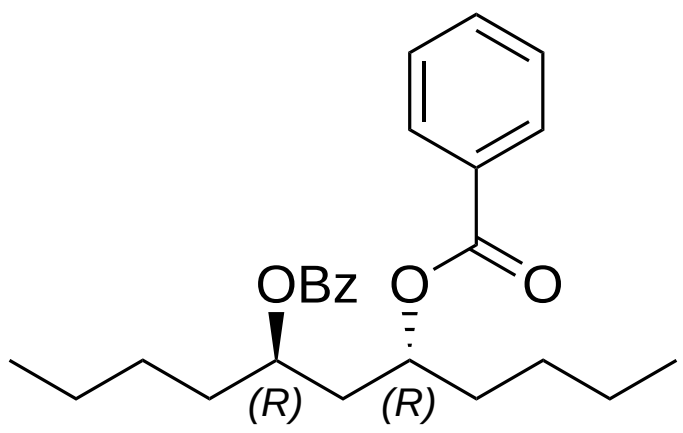

(*R*,*R*)-**1d**-Bz

Prepared according to **General Procedure 8** using (*R*,*R*)-**1d** (3.8 mg, 0.02 mmol), DMAP (0.7 mg, 0.0057 mmol), triethylamine (0.02 ml, 0.14 mmol), benzoyl chloride (0.01 ml, 0.06 mmol) in DCM (0.08 ml) Purified *via* preparative thin layer chromatography (15% Acetone in Hexane) to give (5*R*,7*R*)-undecane-5,7-diyl dibenzoate as a colourless oil (5.3 mg, 0.013 mmol, 67%).

^{1}H NMR (700 MHz, $CDCl_3$) δ 7.95 (d, *J* = 7.1 Hz, 4H), 7.48 (t, *J* = 7.4 Hz, 2H), 7.34 (t, *J* = 7.8 Hz, 4H), 5.26 (p, *J* = 6.1 Hz, 2H), 2.09 (t, *J* = 6.1Hz, 2H), 1.80 – 1.73 (m, 2H), 1.72 – 1.65 (m, 2H), 1.41 – 1.27 (m, 8H), 0.87 (t, *J* = 7.1 Hz, 6H) ppm.

^{13}C NMR (176 MHz, $CDCl_3$) δ 166.2, 132.8, 130.7, 129.7, 128.3, 71.8, 38.4, 34.4, 27.5, 22.7, 14.1 ppm.

Chiral SFC Analysis CHIRALPAK IE (CO_2:MeOH, 97:03, 2.5 ml min^{-1}, 40 °C, 225 nm) t_R = 12.2 (major) minutes, 99% *ee*.

Scheme 3G - 1 mmol scale up of symmetrical 1,3-diol (**1d**)

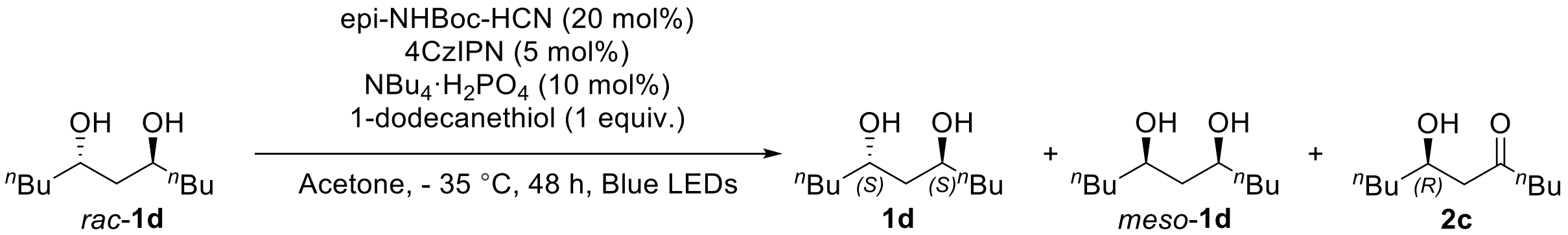

Diol substrate *rac*-**1d** (188 mg, 1 mmol, 1 eq.), *epi*-NHBoc-HCN (79 mg, 0.20 mmol, 20 mol%), 4CzIPN (39 mg, 0.050 mmol, 5 mol%) and tetrabutylammonium phosphate monobasic (34 mg, 0.10 mmol, 10 mol%) were added to a 50 ml conical flask. The flask was sealed with a suba seal and evacuated and backfilled with nitrogen 3 times. Freshly prepared 0.2 M stock solution of 1-dodecanethiol in degassed acetone (5 ml) followed by additional degassed acetone (35 ml) was added *via* syringe to the sealed flask (reaction molarity 0.025 M). The top of the flask was further sealed with Teflon tape then electrical tape and placed in the custom made photoreactor with a positive flow of nitrogen through the apparatus. The reaction vial was cooled to –35 °C and the Kessil® A160WE Tuna blue, LED lamp (100% intensity, 100% blue colour) was switched on. After stirring for 48 h the crude mixture was concentrated *in vacuo*, analysed by 1H NMR using internal standard (CH_2Br_2) to give NMR yield [58% anti, 15% syn, 27% oxidation product]. The crude product was purified by silica gel chromatography (elution with 5% Acetone in Pet. Ether) to isolate (*syn*)-undecane-5,7-diol (43 mg, 0.23 mmol, 23%) as a colourless oil, (5*S*,7*S*)-undecane-5,7-diol (92 mg, 0.49 mmol, 49%, 99% *ee*) as a white solid and (*R*)-7-hydroxyundecan-5-one (28 mg, 0.15 mmol, 15%, 57% *ee*) as a colourless oil.

(*syn*)-undecane-5,7-diol (*meso*-1d)

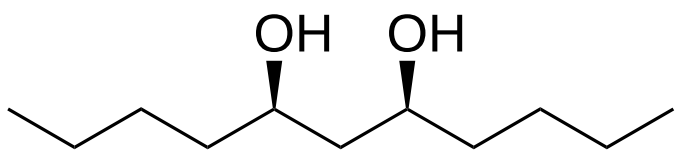

meso-**1d**

^{1}H NMR (700 MHz, $CDCl_3$) δ 3.85 – 3.79 (m, 2H), 3.32 (s, 2H), 1.58 (dt, *J* = 14.5, 2.0 Hz, 1H), 1.50 – 1.39 (m, 5H), 1.39 – 1.24 (m, 8H), 0.89 (t, *J* = 7.0 Hz, 6H) ppm.

^{13}C NMR (176 MHz, $CDCl_3$) δ 73.3, 42.9, 38.0, 27.7, 22.8, 14.2 ppm.

(5*S*,7*S*)-undecane-5,7-diol (1d)

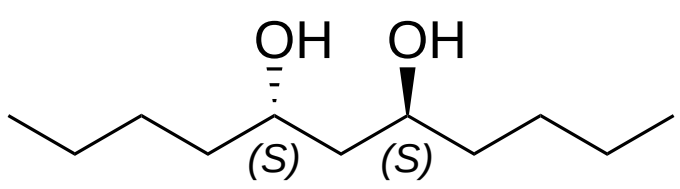

1d

^{1}H NMR (700 MHz, $CDCl_3$) δ 3.96 – 3.91 (m, 2H), 2.22 (s, 2H), 1.60 (t, *J* = 5.6 Hz, 2H), 1.57 – 1.50 (m, 2H), 1.49 – 1.25 (m, 8H), 0.91 (t, *J* = 7.1 Hz, 6H) ppm.

^{13}C NMR (176 MHz, $CDCl_3$) δ 69.6, 42.4, 37.3, 28.1, 22.8, 14.2 ppm.

$[\alpha]_D^{25.0}$ = + 9.44 (*c* 0.49, $CHCl_3$)

(5*S*,7*S*)-undecane-5,7-diyl dibenzoate (1d-Bz)

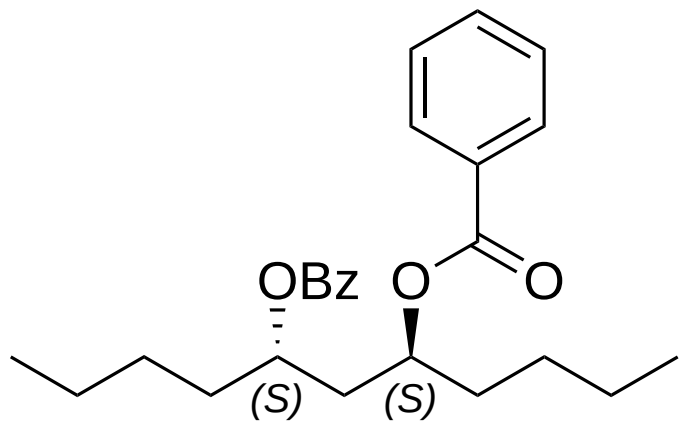

1d-Bz

Prepared according to **General Procedure 8** using (*R*,*R*)-**1d** (7.6 mg, 0.05 mmol), DMAP (1.7 mg, 0.014 mmol), triethylamine (0.04 ml, 0.30 mmol), benzoyl chloride (0.02 ml, 0.15 mmol) in DCM (0.2 ml) Purified *via* preparative thin layer chromatography (15% Acetone in Hexane) to give (5*S*,7*S*)-undecane-5,7-diyl dibenzoate as a colourless oil (13 mg, 0.03 mmol, 67%).

^{1}H NMR (700 MHz, $CDCl_3$) δ 7.95 (dd, *J* = 8.3, 1.3 Hz, 4H), 7.48 (app t, *J* = 7.4 Hz, 2H), 7.34 (t, *J* = 7.8 Hz, 4H), 5.26 (p, *J* = 6.1 Hz, 2H), 2.09 (t, *J* = 6.2Hz, 2H), 1.80 – 1.73 (m, 2H), 1.72 – 1.66 (m, 2H), 1.41 – 1.28 (m, 8H), 0.88 (t, *J* = 7.1 Hz, 6H) ppm.

^{13}C NMR (176 MHz, $CDCl_3$) δ 166.2, 132.8, 130.7, 129.7, 128.3, 71.8, 38.4, 34.4, 27.5, 22.7, 14.1 ppm.

Chiral SFC Analysis CHIRALPAK IE (CO_2:MeOH, 97:03, 2.5 ml min^{-1}, 40 °C, 222 nm) t_R = 10.9 (major) minutes, 99% *ee*.

(*R*)-7-hydroxyundecan-5-one (2c)

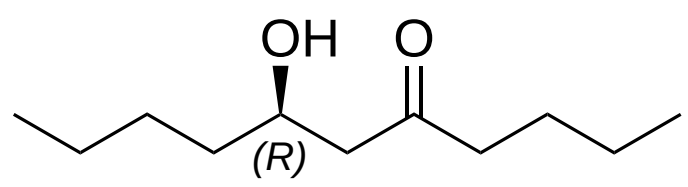

2c

^{1}H NMR (700 MHz, $CDCl_3$) δ 4.04 – 4.00 (m, 1H), 2.59 (dd, *J* = 17.5, 2.6 Hz, 1H), 2.48 (dd, *J* = 17.5, 9.2 Hz, 1H), 2.41 (t, *J* = 7.5 Hz, 2H), 1.55 (app p, *J* = 7.5 Hz, 2H), 1.51 – 1.41 (m, 1H), 1.43 – 1.35 (m, 2H), 1.35 – 1.27 (m, 5H), 0.92 – 0.87 (m, 6H) ppm.

^{13}C NMR (176 MHz, $CDCl_3$) δ 212.8, 67.8, 49.1, 43.5, 36.3, 27.8, 25.8, 22.8, 22.4, 14.1, 13.9 ppm.

$[\alpha]_D^{25.0}$ = - 2.07 (*c* 1.01, $CHCl_3$)

(*R*)-7-((*tert*-butyldiphenylsilyl)oxy)undecan-5-one (2c-TBDPS)

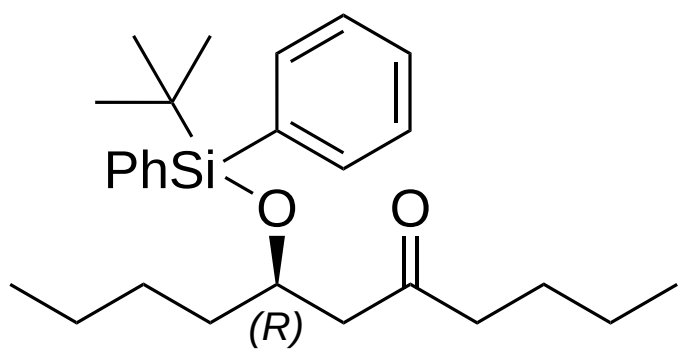

2c-TBDPS

Prepared according to **General Procedure 9** using **2c** (9.4 mg, 0.05 mmol), TBDPSCl (0.02 ml, 0.06 mmol), imidazole (5.0 mg, 0.075 mmol) in DCM (1.1 ml). Purified *via* preparative thin layer chromatography (5% Acetone in Hexane) to give (*R*)-7-((*tert*-butyldiphenylsilyl)oxy)undecan-5-one as a colourless oil (0.7 mg, 0.0016 mmol, 3%).

^{1}H NMR (700 MHz, $CDCl_3$) δ 7.69 – 7.65 (m, 4H), 7.43 – 7.40 (m, 2H), 7.39 – 7.35 (m, 4H), 4.22 (p, *J* = 5.9 Hz, 1H), 2.55 (dd, *J* = 15.3, 6.1 Hz, 1H), 2.45 (dd, *J* = 15.2, 6.2 Hz, 1H), 2.29 – 2.19 (m, 2H), 1.45 – 1.38 (m, 4H), 1.25 – 1.08 (m, 6H), 1.02 (s, 9H), 0.85 (t, *J* = 7.4 Hz, 3H), 0.76 (t, *J* = 7.2 Hz, 3H) ppm.

^{13}C NMR (176 MHz, $CDCl_3$) δ 209.9, 136.0, 134.4, 134.2, 129.8, 129.7, 127.7, 127.6, 70.2, 50.0, 43.8, 37.0, 27.1, 25.7, 22.6, 22.4, 19.5, 14.1, 14.0 ppm.

Chiral SFC Analysis CHIRALPAK IA (CO_2:MeOH, 99:01, 2.5 ml min^{-1}, 40 °C, 216 nm) t_R = 5.5 (minor), 6.3 (major) minutes, 57% *ee*.

Scheme 4A – Non-symmetrical 1,3-diol 3a

enriched-1-Phenylpentane-2,4-diol (3a) from (*anti*)-1-phenylpentane-2,4-diol (rac-anti-3a)

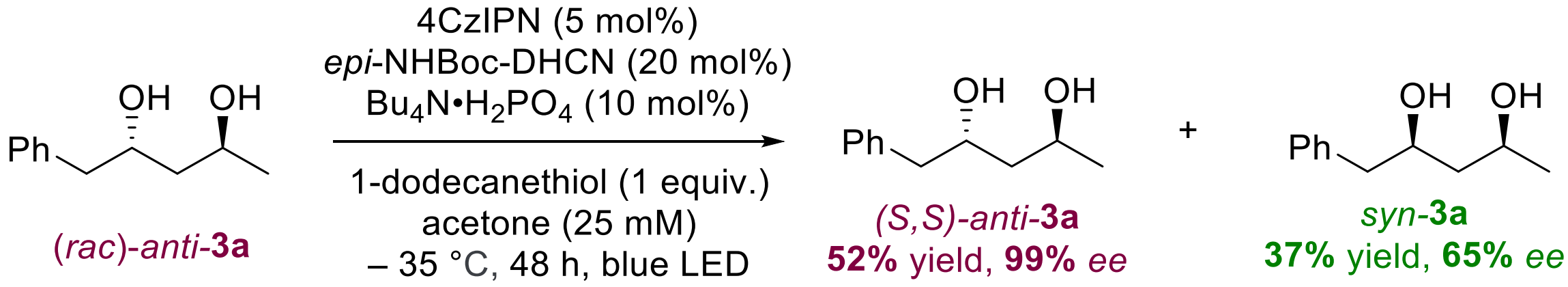

The compound was synthesised according to **General Procedure 7** using (*anti*)-1-phenylpentane-2,4-diol (18.0 mg, 0.10 mmol), separated with DCM then 10% acetone in hexane, giving (2*R*,4*S*)-1-phenylpentane-2,4-diol (6.7 mg, 37%, 65% *ee*) as a colourless oil and (*S*,*S*)-1-phenylpentane-2,4-diol (9.3 mg, 52%, 99% *ee*) as a colourless solid.

(*S*,*S*)-1-Phenylpentane-2,4-diol (*S*,*S*-3a)

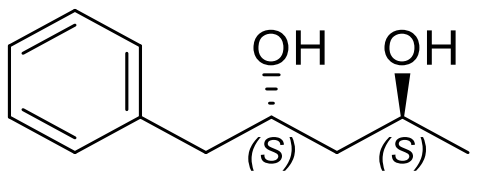

^{1}H NMR (500 MHz, $CDCl_3$) δ 7.34 – 7.29 (m, 2H), 7.26 – 7.20 (m, 3H), 4.23 – 4.14 (m, 2H), 2.84 – 2.75 (m, 2H), 2.34 (br. s, 1H), 2.24 (br. s, 1H), 1.71 – 1.64 (m, 2H), 1.24 (d, *J* = 6.3 Hz, 3H) ppm.

^{13}C NMR (126 MHz, $CDCl_3$) δ 138.4, 129.5, 128.8, 126.7, 70.3, 65.6, 44.2, 43.7, 23.7 ppm.

$[\alpha]_D^{25}$ = -1.9 (*c* 0.37, $CHCl_3$).

Chiral SFC Analysis: CHIRALPAK IC (CO_2:MeOH, 96:4, 2.5 mL min^{-1}, 40 °C, 205 nm) t_R = 12.0 (major), 12.4 (minor) min, 99% *ee*.

(2*R*,4*S*)-1-Phenylpentane-2,4-diol (2*R*,4*S*-3a)

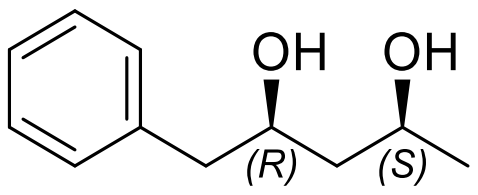

^{1}H NMR (500 MHz, $CDCl_3$) δ 7.34 – 7.29 (m, 2H), 7.26 – 7.19 (m, 3H), 4.13 – 3.99 (m, 2H), 2.81 – 2.72 (m, 2H), 2.14 (br. s, 2H), 1.66 (dt, *J* = 14.5, 2.4 Hz, 1H), 1.61 – 1.52 (m, 1H), 1.20 (d, *J* = 6.2 Hz, 3H) ppm.

^{13}C NMR (126 MHz, $CDCl_3$) δ 138.0, 129.6,128.8, 126.8, 74.0, 69.2, 44.8, 44.2, 24.2 ppm.

$[\alpha]_D^{25}$ = +4.6 (*c* 0.26, $CHCl_3$).

Chiral SFC Analysis: CHIRALPAK IK (CO_2:MeOH, 93:7, 2.5 mL min^{-1}, 40 °C, 205 nm) t_R = 6.6 (minor), 8.0 (major) min, 65% *ee*.

Enriched-1-Phenylpentane-2,4-diol (3a) from (*syn*)-1-phenylpentane-2,4-diol (rac-syn-3a)

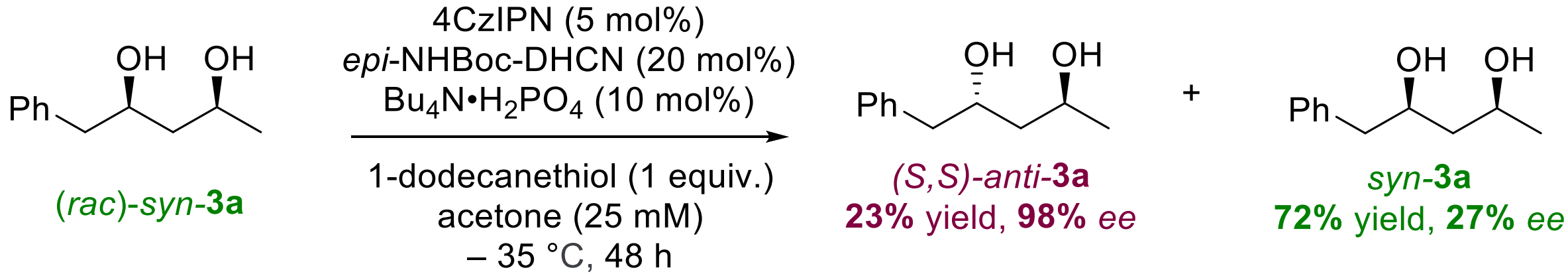

The compound was synthesised according to **General Procedure 7** using (*syn*)-1-phenylpentane-2,4-diol (18.0 mg, 0.10 mmol), separated with DCM then 10% acetone in hexane, giving (2*R*,4*S*)-1-phenylpentane-2,4-diol (13.0 mg, 72%, 27% *ee*) as a colourless oil and (*S*,*S*)-1-phenylpentane-2,4-diol (4.1 mg, 23%, 98% *ee*) as a colourless solid.

Scheme 4B – Reaction Time Course Study for Epimerization of *rac-anti*-3a

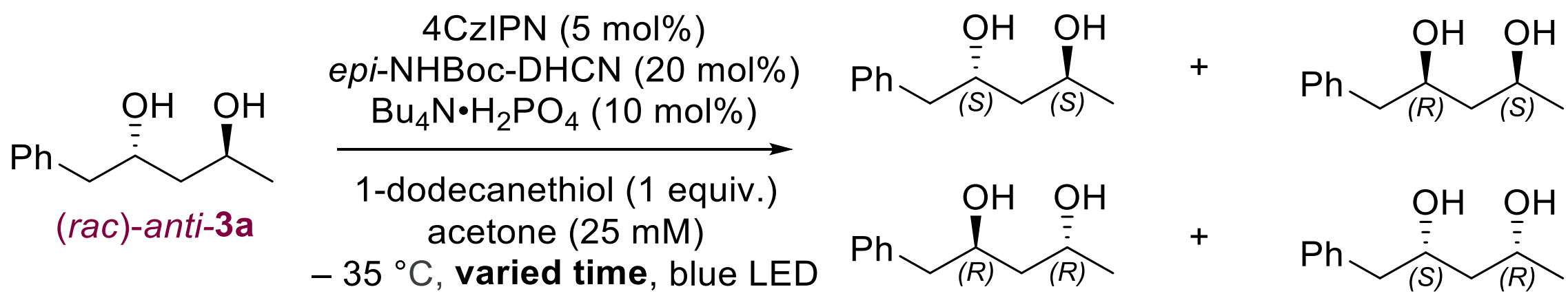

In a Schlenk flask a stock solution in degassed acetone (25 mM) was prepared according to the ratios in **General Procedure 7** so that 4 mL solution contains (*anti*)-1-phenylpentane-2,4-diol (18.0 mg, 0.10 mmol), 4CzIPN (3.9 mg, 0.0050 mmol, 5 mol%), *epi*-NHBoc-DHCN (7.9 mg, 0.020 mmol, 20 mol%), tetrabutylammonium phosphate monobasic (3.4 mg, 0.010 mmol, 10 mol%) and 1-dodecanethiol (20.2 mg, 0.10 mmol, 1 equiv.). 4 mL was transferred to a crimp top vial containing a stir bar which was purged with nitrogen before for each time point. The top of the vial was further sealed with Teflon tape then electrical tape and placed in the custom made photoreactor with a positive flow of nitrogen through the apparatus. The reaction vial was cooled to –35 °C and the Kessil® A160WE Tuna blue, LED lamp (100% intensity, 100% ocean blue color) was switched on. After stirring for the specified time, the crude mixture was concentrated in mixture was concentrated in vacuo, analysed by 1H NMR using internal standard (CH_2Br_2). The mixture was partially purified by preparative TLC using DCM followed by a second run with 30% acetone in hexane. This afforded the diastereomeric mixture which was analysed by chiral SFC to determine the *ee* of each diastereomer. The combination of NMR yield and ee allows for the yield determination of each individual stereoisomer.

time (h)	(*S*,*S*)-3a (%)	(*R*,*R*)-3a (%)	(2*R*,4*S*)-3a (%)	(2*S*,4*R*)-3a (%)
0.5	44.4	38.4	5.1	1.3
1	45.1	28.6	12.3	2.9
2	44.9	16.7	24.2	5.7
3.5	44.8	4.4	32.3	7.5
9	45.9	0.1	34.7	8.5
15	45.3	0.1	30.1	7.0
24	42.3	0.1	25.4	6.4
48	45.6	0.1	25.2	5.3

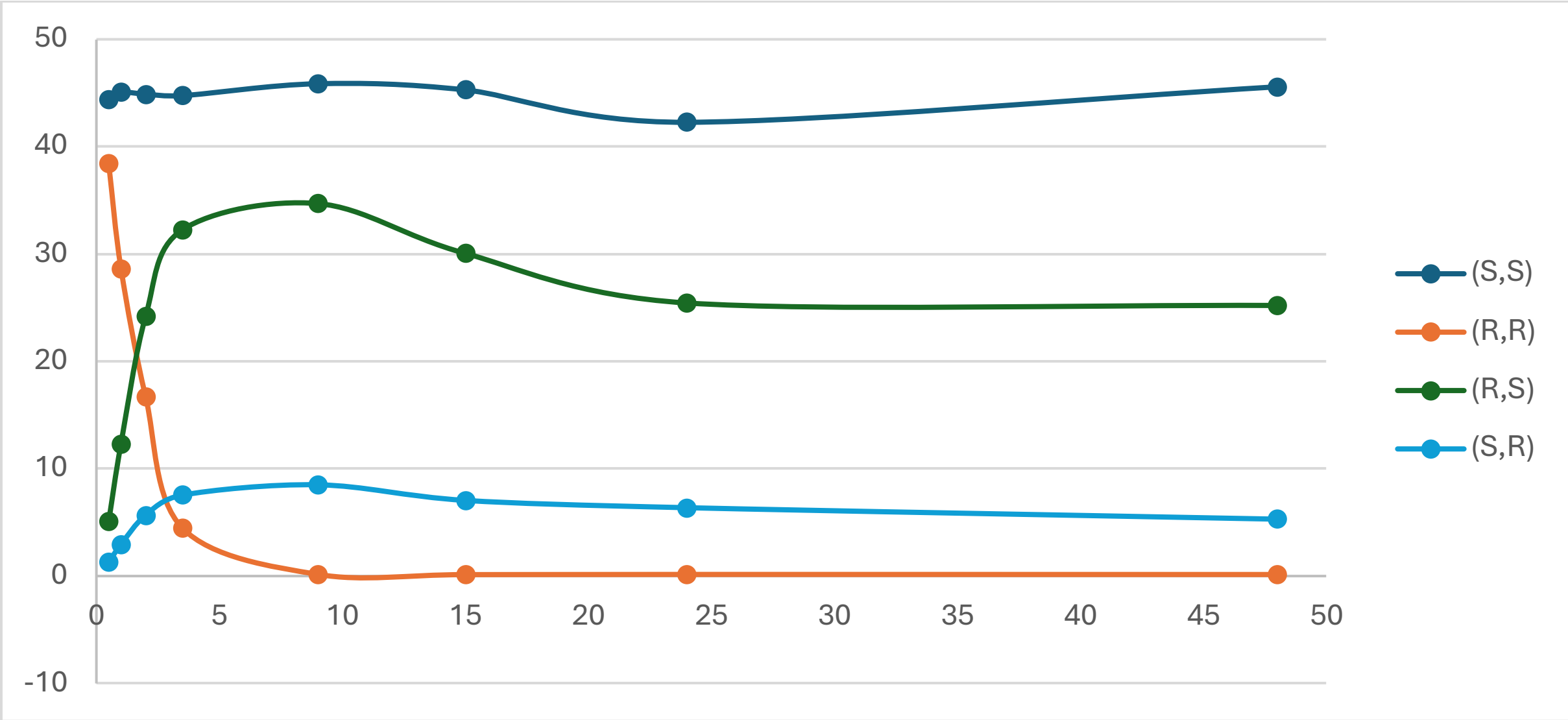
50
40
30
20
10
0
-10
0
5
10
15
20
25
30
35
40
45
50
(S,S)
(R,R)
(R,S)
(S,R)

Scheme 4C – Deuterium Experiments Using non-Symmetrical Diol

From diol 3a

Enriched-1-Phenylpentane-2,4-diol (3a) from rac-(*anti*)-1-phenylpentane-2,4-diol-d1 (*rac-anti*-3a)

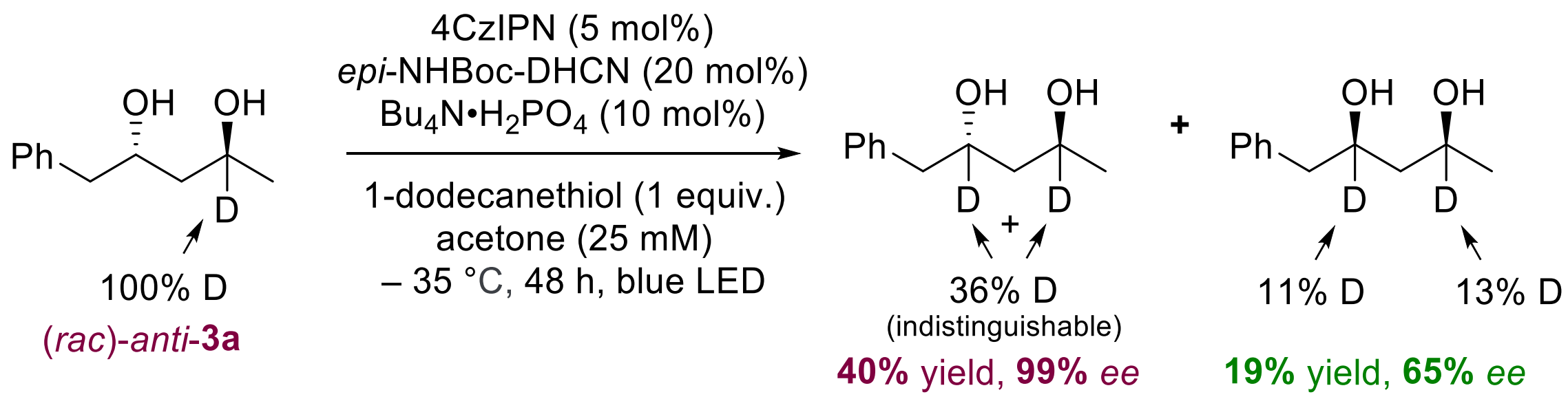

The compound was synthesised according **General Procedure 7** using (*anti*)-1-phenylpentane-2,4-diol mono deuterated (18.1 mg, 0.10 mmol), separated with DCM then 10% acetone in hexane, giving (2*R*,4*S*)-1-phenylpentane-2,4-diol (3.4 mg, 19%, 65% *ee*) as a colourless oil and (*S*,*S*)-1-phenylpentane-2,4-diol (7.3 mg, 40%, 99% *ee*) as a colourless solid.

(*S*,*S*)-1-Phenylpentane-2,4-diol (*S*,*S*-3a)

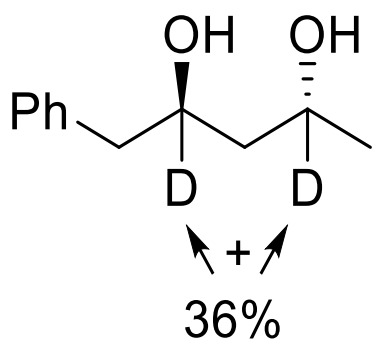

^{1}H NMR (700 MHz, $CDCl_3$) δ 7.34 – 7.31 (m, 2H), 7.26 – 7.20 (m, 3H), 4.23 – 4.15 (m, 1.28H), 2.84 – 2.75 (m, 2H), 2.11 (br. s, 2H), 1.70 – 1.64 (m, 2H), 1.25 – 1.22 (m, 3H) ppm.

^{13}C NMR (176 MHz, $CDCl_3$) δ 138.4, 129.5, 128.8, 126.8, 70.5 – 70.1 (m), 65.4 – 65.0 (m), 44.2, 43.8 – 44.6 (m), 23.7 – 23.5 (m) ppm.

^{2}H NMR (107 MHz, $CDCl_3$) δ 4.20 (s) ppm.

$[\alpha]_D^{25}$ = -6.2 (*c* 0.29, $CHCl_3$).

Chiral SFC Analysis: CHIRALPAK IC (CO_2:MeOH, 96:4, 2.5 mL min^{-1}, 40 °C, 205 nm) t_R = 12.0 (major), 12.4 (minor) min, 99% *ee*.

(2*R*,4*S*)-1-Phenylpentane-2,4-diol (2*R*,4*S*-3a)

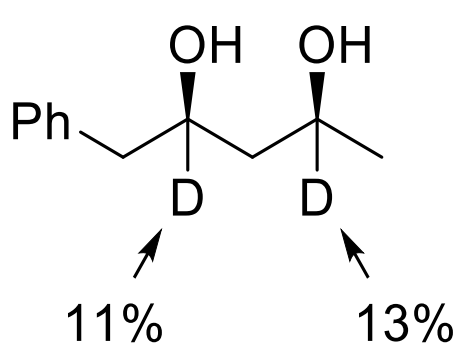

^{1}H NMR (700 MHz, $CDCl_3$) δ 7.34 – 7.30 (m, 2H), 7.26 – 7.23 (m, 1H), 7.21, (d, *J* = 7.4 Hz, 2H), 4.11 – 4.06 (m, 0.89H), 4.06 – 4.01 (m, 0.87H), 2.82 – 2.72 (m, 2H), 2.13 (br. s, 2H), 1.66 (dt, *J* = 14.5, 2.1 Hz 1H) , 1.60 – 1.53 (m, 1H), 1.59 – 1.53 (m, 1H), 1.20 (d, *J* = 6.2 Hz, 3H) ppm.

^{13}C NMR (176 MHz, $CDCl_3$) δ 138.0, 129.6, 128.8, 126.8, 74.0, 69.1, 44.8, 44.2, 24.2 ppm.

^{2}H NMR (107 MHz, $CDCl_3$) signals not detected.

$[\alpha]_D^{25}$ = +3.6 (*c* 0.14, $CHCl_3$).

Chiral SFC Analysis: CHIRALPAK IK (CO_2:MeOH, 93:7, 2.5 mL min^{-1}, 40 °C, 205 nm) t_R = 6.6 (minor), 8.0 (major) min, 65% *ee*.

Enriched-1-Phenylpentane-2,4-diol (3a) from *rac*-(*anti*)-1-phenylpentane-2,4-diol (rac-anti-3a)

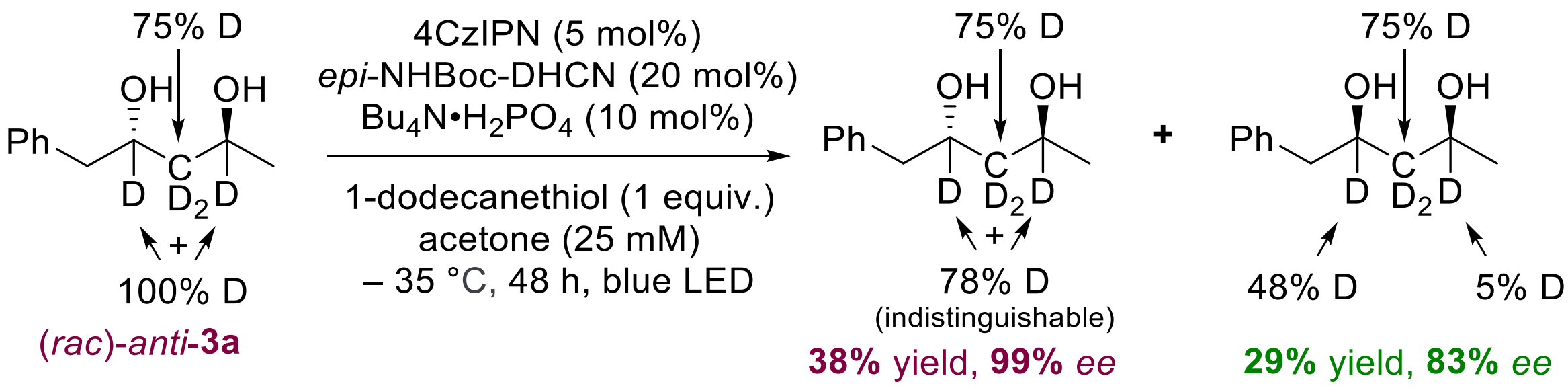

The compound was synthesised according to **General Procedure 7** using (*anti*)-1-phenylpentane-2,4-diol quadruple deuterated (18.4 mg, 0.10 mmol), separated with DCM then 10% acetone in hexane, giving (2*R*,4*S*)-1-phenylpentane-2,4-diol (5.4 mg, 29%, 84% *ee*) as a colourless oil and (*S*,*S*)-1-phenylpentane-2,4-diol (7.0 mg, 38%, 99% *ee*) as a colourless solid.

(*S*,*S*)-1-Phenylpentane-2,4-diol (*S*,*S*-3a)

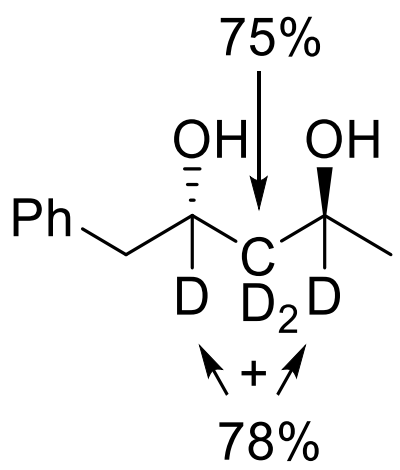

^{1}H NMR (700 MHz, $CDCl_3$) δ 7.34 – 7.30 (m, 2H), 7.26 – 7.20 (m, 3H), 4.22 – 4.15 (m, 0.44H), 2.83 – 2.75 (m, 2H), 2.02 (br. s, 2H), 1.69 – 1.62 (m, 0.5H), 1.24 – 1.21 (m, 3H) ppm.

^{13}C NMR (176 MHz, $CDCl_3$) δ 138.4, 129.5, 128.8, 126.7, 70.4 – 69.6 (m), 65.6 – 64.9 (m), 44.2 – 43.9 (m), 43.6 – 42.9 (m), 23.7 – 23.4 (m) ppm.

^{2}H NMR (107 MHz, $CDCl_3$) δ 4.16 (s, 1.56D), 1.62 (s, 1.5D) ppm.

$[\alpha]_D^{25}$ = -2.1 (*c* 0.28, $CHCl_3$).

Chiral SFC Analysis: CHIRALPAK IC (CO_2:MeOH, 96:4, 2.5 mL min^{-1}, 40 °C, 205 nm) t_R = 12.0 (major), 12.4 (minor) min, 99% *ee*.

(2*R*,4*S*)-1-Phenylpentane-2,4-diol (2*R*,4*S*-3a)

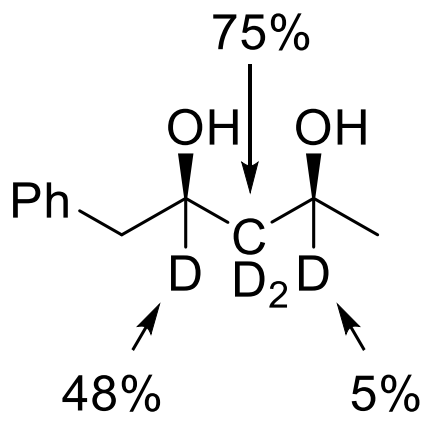

^{1}H NMR (700 MHz, $CDCl_3$) δ 7.34 – 7.30 (m, 2H), 7.26 – 7.23 (m, 1H), 7.21, (d, *J* = 7.6 Hz, 2H), 4.10 – 4.06 (m, 0.52H), 4.06 – 4.00 (m, 0.95H), 2.81 – 2.72 (m, 2H), 2.37 (br. s, 2H), 1.68 – 1.52 (m, 0.51H), 1.59 – 1.53 (m, 1H), 1.19 (d, *J* = 6.2 Hz, 3H) ppm.

^{13}C NMR (176 MHz, $CDCl_3$) δ 138.0, 129.6, 128.8, 126.8, 74.1 – 73.8 (m), 69.1 – 68.8 (m), 44.9 – 44.6 (m), 44.3 – 44.2 (m), 24.2 ppm.

^{2}H NMR (107 MHz, $CDCl_3$) δ 4.08 (s, 0.48D), 1.69 – 1.45 (s, 1.5D) ppm.

$[\alpha]_D^{25}$ = +6.4 (*c* 0.22, $CHCl_3$).

Chiral SFC Analysis: CHIRALPAK IK (CO_2:MeOH, 93:7, 2.5 mL min^{-1}, 40 °C, 205 nm) t_R = 6.6 (minor), 8.0 (major) min, 84% *ee*.

From diol 3d

(*S*,*S*)-6-Phenylhexane-2,4-diol (*S*,*S*-3d)

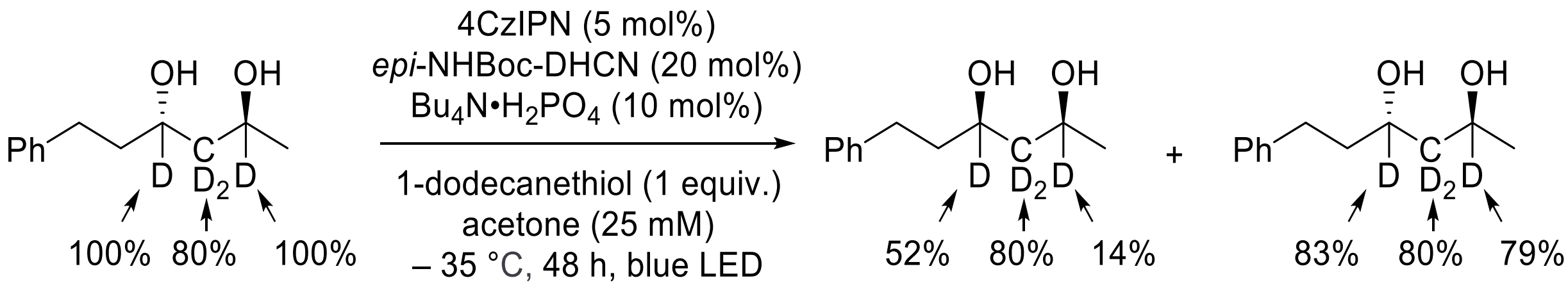

The compound was synthesised according to **General Procedure 7** using (*anti*)-6-phenylhexane-2,4-diol quadruple deuterated (19.7 mg, 0.10 mmol), separated with DCM then 10% acetone in hexane, giving (2*S*,4*R*)-6-phenylhexane-2,4-diol (5.5 mg, 28%, 47% *ee*) as a colourless oil and (*S*,*S*)-6-phenylhexane-2,4-diol (7.9 mg, 41%, 99% *ee*) as a colourless solid.

(*S*,*S*)-6-Phenylhexane-2,4-diol (*S*,*S*-3d)

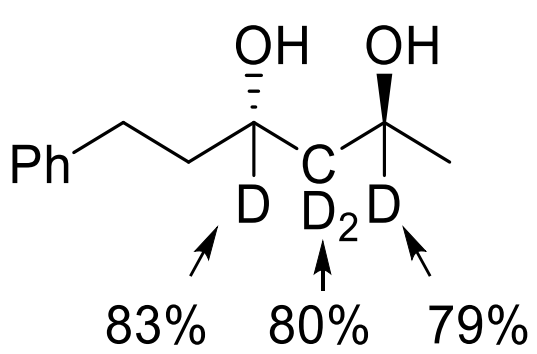

^{1}H NMR (700 MHz, $CDCl_3$) δ 7.31 – 7.27 (m, 2H), 7.22 – 7.17 (m, 3H), 4.18 (br. s, 0.21H), 3.98 (br. s, 0.17H), 2.83 – 2.76 (m, 1H), 2.71 – 2.65 (m, 1H), 2.45 – 2.36 (m, 1H), 2.25 – 2.15 (m, 1H), 1.90 – 1.82 (m, 1H), 1.81 – 1.74 (m, 1H), 1.69 – 1.60 (m, 0.4H), 1.25 – 1.22 (m, 3H) ppm.

^{13}C NMR (176 MHz, $CDCl_3$) δ 142.1, 128.59, 128.54, 126.0, 69.0 – 68.3 (m), 65.8 – 65.0 (m), 44.3 – 43.1 (m), 39.2 – 38.9 (m) 32.4 – 32.3 (m), 23.8 – 23.6 (m) ppm.

^{2}H NMR (107 MHz, $CDCl_3$) δ 4.18 (s, 0.79D), 3.97 (s, 0.83D), 1.62 (s, 1.6D) ppm.

$[\alpha]_D^{25}$ = +2.5 (*c* 0.32, $CHCl_3$).

Chiral SFC Analysis: CHIRALPAK IK (CO_2:MeOH, 95:5, 2.5 mL min^{-1}, 40 °C, 205 nm) t_R = 10.7 (major), 11.1 (minor) min, 99% *ee*.

(2*S*,4*R*)-6-Phenylhexane-2,4-diol (2*S*,4*R*-3d)

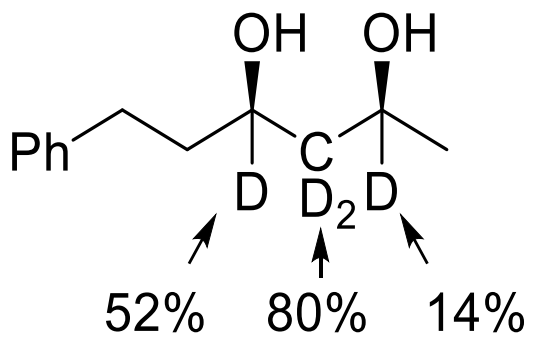

^{1}H NMR (700 MHz, $CDCl_3$) δ 7.31 – 7.27 (m, 2H), 7.22 – 7.17 (m, 3H), 4.08 – 4.01 (m, 0.86H), 3.89 (br. s, 0.48H), 2.99 – 2.86 (m, 1H), 2.81 – 2.63 (m, 3H), 1.85 – 1.72 (m, 2H), 1.60 – 1.51 (m, 0.4H), 1.21 (d, *J* = 6.4 Hz, 3H) ppm.

^{13}C NMR (176 MHz, $CDCl_3$) δ 142.1, 128.59, 128.55, 126.0, 72.6 – 71.6 (m), 69.5 – 69.1 (m), 44.9 – 43.9 (m), 39.9 – 39.7 (m), 31.9 – 31.8 (m), 24.6 – 24.3 (m) ppm.

^{2}H NMR (107 MHz, $CDCl_3$) δ 4.05 (s, 0.14D), 3.89 (s, 0.52D), 1.68 – 1.45 (s, 1.6D) ppm.

$[\alpha]_D^{25}$ = +5.9 (*c* 0.22, $CHCl_3$).

Chiral SFC Analysis: CHIRALPAK IC (CO_2:MeOH, 95:5, 2.5 mL min^{-1}, 40 °C, 205 nm) t_R = 11.6 (minor), 14.0 (major) min, 47% *ee*.

Scheme 5 – Scope of non-symmetrical 1,3-diols

Enriched-1-(4-Methoxyphenyl)pentane-2,4-diol (3b)

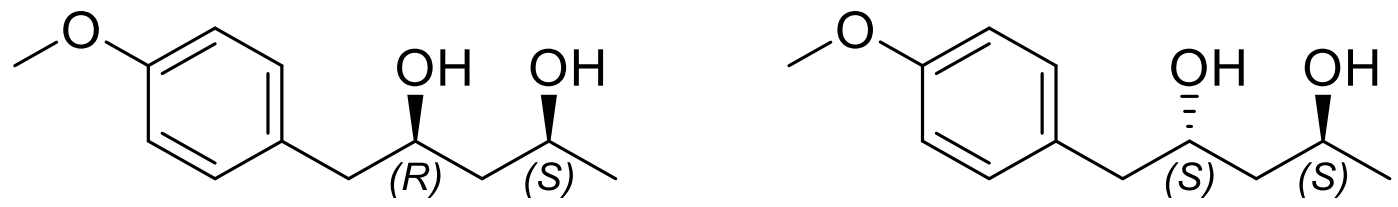

The compound was synthesised according to **General Procedure 7** using (*anti*)-1-(4-methoxyphenyl)pentane-2,4-diol (21.0 mg, 0.10 mmol), separated with DCM then 10 – 15% acetone in hexane, giving (2*R*,4*S*)-1-(4-methoxyphenyl)pentane-2,4-diol (10.6 mg, 50%, 65% *ee*) as a colourless oil and (*S*,*S*)-1-(4-methoxyphenyl)pentane-2,4-diol (2.1 mg, 11%, 94% *ee*) as a colourless solid.

(*S*,*S*)-1-(4-Methoxyphenyl)pentane-2,4-diol (*S*,*S*-3b)

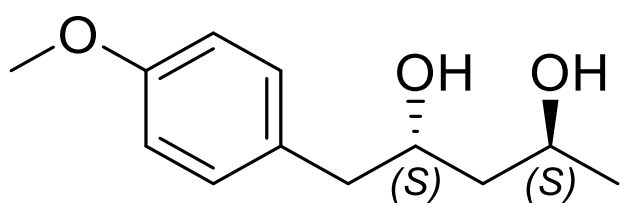

^{1}H NMR (700 MHz, $CDCl_3$) δ 7.14 (d, *J* = 8.1 Hz, 2H), 6.86 (d, *J* = 8.1 Hz, 2H), 4.22 – 4.17 (m, 1H), 4.15 – 4.11 (m, 1H), 3.80 (s, 3H), 2.78 – 2.69 (m, 2H), 1.79 (br. s, 2H), 1.68 – 1.64 (m, 2H), 1.24 (d, *J* = 6.3 Hz, 3H) ppm.

^{13}C NMR (156 MHz, $CDCl_3$) δ 158.5, 130.5, 130.3, 114.2, 70.4, 65.6, 55.4, 43.6, 43.2, 23.7 ppm.

$[\alpha]_D^{25.0}$ = -1.0 (*c* 0.10, $CHCl_3$). (The low specific rotation results in this measurement to lie within the error range of the instrument)

Chiral SFC Analysis: CHIRALPAK IC (CO_2:MeOH, 94:6, 2.5 mL min^{-1}, 40 °C, 223 nm) t_R = 11.6 (minor), 12.2 (major) min, 94% *ee*.

(2*R*,4*S*)-1-(4-Methoxyphenyl)pentane-2,4-diol (2*R*,4*S*-3b)

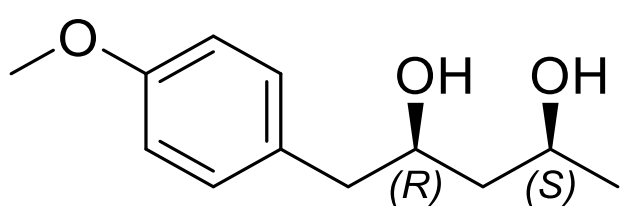

^{1}H NMR (500 MHz, $CDCl_3$) δ 7.15 – 7.10 (m, 2H), 6.89 – 6.84 (m, 2H), 4.07 – 3.99 (m, 2H), 3.80 (s, 3H), 2.76 – 2.65 (m, 2H), 2.30 (br. s, 2H), 1.65 (dt, *J* = 14.5, 2.4 Hz, 1H), 1.58 – 1.50 (m, 1H), 1.19 (d, *J* = 6.2 Hz, 3H) ppm.

^{13}C NMR (126 MHz, $CDCl_3$) δ 158.5, 130.5, 129.9, 114.2, 74.1, 69.0, 55.4, 44.2, 43.9, 24.2 ppm.

$[\alpha]_D^{25}$ = +2.1 (*c* 0.42, $CHCl_3$).

Chiral SFC Analysis: CHIRALPAK IK (CO_2:MeOH, 93:7, 2.5 mL min^{-1}, 40 °C, 223 nm) t_R = 10.8 (minor), 11.5 (major) min, 65% *ee*.

enriched-1-(Naphthalen-1-yl)pentane-2,4-diol (3c)

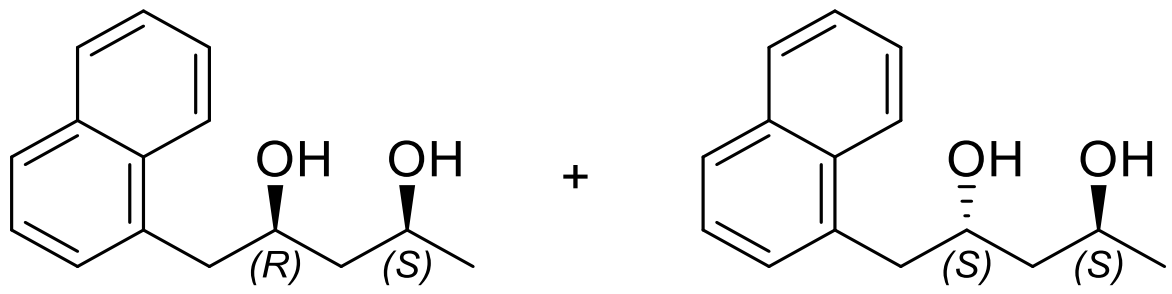

The compound was synthesised according to **General Procedure 7** using (*anti*)-1-(naphthalen-1-yl)pentane-2,4-diol (23.0 mg, 0.10 mmol), separated with DCM then 10% acetone in hexane, giving (2*R*,4*S*)-1-(naphthalen-1-yl)pentane-2,4-diol (8.6 mg, 37%, 79% *ee*) as a colourless oil and (*S*,*S*)-1-(naphthalen-1-yl)pentane-2,4-diol (9.1 mg, 40%, 98% *ee*) as a colourless solid.

(*S*,*S*)-1-(Naphthalen-1-yl)pentane-2,4-diol (*S*,*S*-3c)

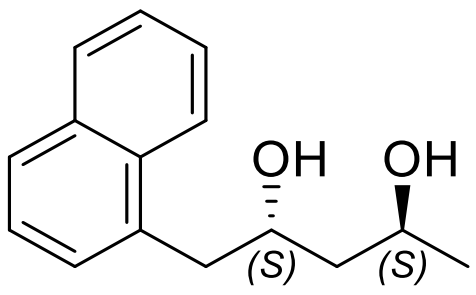

^{1}H NMR (500 MHz, $CDCl_3$) δ 8.05 (d, *J* = 8.3 Hz, 1H), 7.89 – 7.85 (m, 1H), 7.77 (d, *J* = 8.1 Hz, 1H), 7.55 – 7.47 (m, 2H), 7.45 – 7.40 (m, 1H), 7.37 (d, *J* = 7.0 Hz, 1H), 4.38 – 4.31 (m, 1H), 4.29 – 4.20 (m, 1H), 3.33 (dd, *J* = 13.9, 4.6 Hz, 1H), 3.20 (dd, *J* = 13.9, 8.6 Hz, 1H), 2.04 (br. s, 2H), 1.84 – 1.72 (m, 2H), 1.24 (d, *J* = 6.3 Hz, 3H) ppm.

^{13}C NMR (126 MHz, $CDCl_3$) δ 134.5, 134.2, 132.3, 127.8, 127.7, 126.3, 125.9, 125.6, 123.9, 69.6, 65.6, 44.2, 41.2, 23.7 ppm.

$[\alpha]_D^{25}$ = -19.4 (*c* 0.36, $CHCl_3$).

Chiral SFC Analysis: CHIRALPAK IK (CO_2:MeOH, 91:9, 2.5 mL min^{-1}, 40 °C, 280 nm) t_R = 9.9 (major), 11.2 (minor) min, 98% *ee*.

(2*R*,4*S*)-1-(Naphthalen-1-yl)pentane-2,4-diol (2*R*,4*S*-3c)

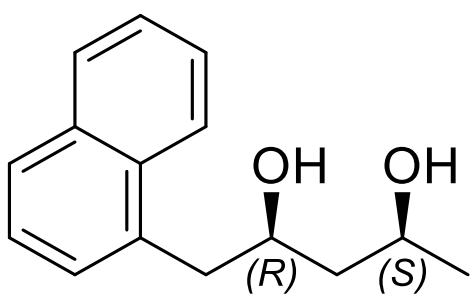

^{1}H NMR (500 MHz, $CDCl_3$) δ 8.05 (d, *J* = 8.3 Hz, 1H), 7.89 – 7.85 (m, 1H), 7.77 (d, *J* = 8.1 Hz, 1H), 7.56 – 7.47 (m, 2H), 7.45 – 7.40 (m, 1H), 7.36 (d, *J* = 6.9 Hz, 1H), 4.30 – 4.22 (m, 1H), 4.05 – 3.97 (m, 1H), 3.29 – 3.17 (m, 2H), 2.34 (br. s, 2H), 1.77 – 1.63 (m, 2H), 1.19 (d, *J* = 6.2 Hz, 3H) ppm.

^{13}C NMR (126 MHz, $CDCl_3$) δ 134.2, 134.1, 132.3, 129.0, 127.9, 127.7, 126.3, 125.9, 125.6, 123.9, 73.2, 69.0, 44.6, 41.9, 24.2 ppm.

$[\alpha]_D^{25}$ = +9.1 (*c* 0.34, $CHCl_3$).

Chiral SFC Analysis: CHIRALPAK IK (CO_2:MeOH, 91:9, 2.5 mL min^{-1}, 40 °C, 280 nm) t_R = 11.5 (major), 12.8 (minor) min, 79% *ee*.

enriched-6-Phenylhexane-2,4-diol (3d)

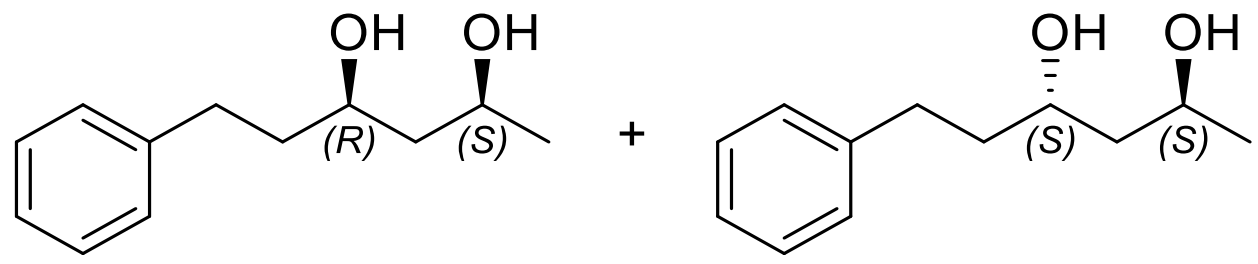

The compound was synthesised according to **General Procedure 7** using (*anti*)-6-phenylhexane-2,4-diol (19.4 mg, 0.10 mmol), separated with DCM then 10% acetone in hexane, giving (2*S*,4*R*)-6-phenylhexane-2,4-diol (3.9 mg, 20%, 32% *ee*) as a colourless oil and (*S*,*S*)-6-phenylhexane-2,4-diol (8.2 mg, 42%, 99% *ee*) as a colourless solid.

(*S*,*S*)-6-Phenylhexane-2,4-diol (*S*,*S*-3d)

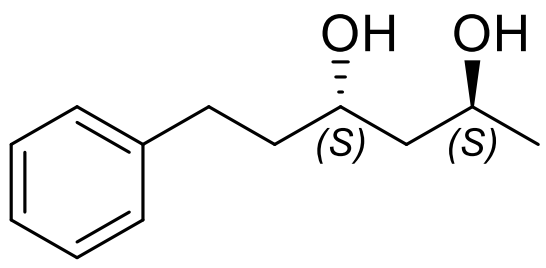

^{1}H NMR (700 MHz, $CDCl_3$) δ 7.31 – 7.26 (m, 2H), 7.22 – 7.17 (m, 3H), 4.22 – 4.15 (m, 1H), 4.02 – 3.95 (m, 1H), 2.83 – 2.76 (m, 1H), 2.72 – 2.64 (m, 1H), 2.47 (br. s, 1H), 2.20 (br. s, 1H), 1.91 – 1.84 (m, 1H), 1.82 – 1.74 (m, 1H), 1.69 – 1.61 (m, 2H), 1.24 (d, *J* = 6.3 Hz, 3H) ppm.

^{13}C NMR (156 MHz, $CDCl_3$) δ 142.1, 128.59, 128.54, 126.0, 69.0, 65.8, 44.2, 39.2, 32.3, 23.8 ppm.

$[\alpha]_D^{25}$ = +2.4 (*c* 0.33, $CHCl_3$). (lit.: ((*R*,*R*)-isomer: 88% ee, **$[\alpha]_D^{rt}$** = +2.7 (*c* 0.6, $CHCl_3$))[18] We tentatively attribute the disagreement with the specific rotation in the literature to the small magnitude and therefore large chance of measuring the opposite sign due to experimental error. Comparing the elution sequence by HPLC on the same column under the same conditions as in the reference above suggested that this is indeed the *S*,*S*-enantiomer. (See later section "Absolute Configuration")

Chiral SFC Analysis: CHIRALPAK IK (CO_2 :MeOH, 95:5, 2.5 mL min^{-1}, 40 °C, 205 nm) t_R = 10.7 (major), 11.1 (minor) min, 99% *ee*.

(2*S*,4*R*)-6-Phenylhexane-2,4-diol (2*S*,4*R*-3d)

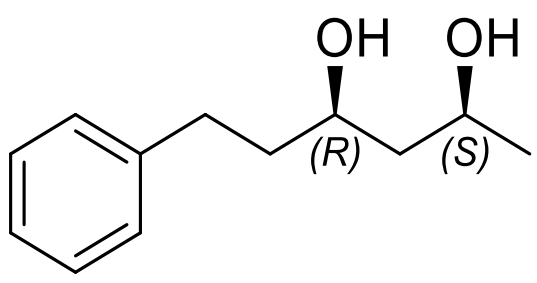

^{1}H NMR (500 MHz, $CDCl_3$) δ 7.31 – 7.27 (m, 2H), 7.22 – 7.17 (m, 3H), 4.09 – 4.02 (m, 1H), 3.93 – 3.86 (m, 1H), 2.89 (br. s, 1H), 2.80 – 2.60 (m, 2H), 2.72 – 2.65 (m, 1H), 1.85 – 1.72 (m, 2H), 1.63 – 1.52 (m, 2H), 1.22 (d, *J* = 6.2 Hz, 3H) ppm.

^{13}C NMR (126 MHz, $CDCl_3$) δ 142.1, 128.60, 128.56, 126.4, 72.5, 69.4, 44.9, 39.9, 31.9, 24.6 ppm.

$[\alpha]_D^{25}$ = +5.6 (*c* 0.16, $CHCl_3$). (lit.: (99% *ee*, **$[\alpha]_D^{20}$** = +22.4 (*c* 1.0, $CHCl_3$)),[19] (88% ee, **$[\alpha]_D^{rt}$** = +11.7 (*c* 0.6, $CHCl_3$)))[18]

Chiral SFC Analysis: CHIRALPAK IC (CO_2 :MeOH, 95:5, 2.5 mL min^{-1}, 40 °C, 205 nm) t_R = 11.6 (minor), 14.0 (major) min, 32% *ee*.

enriched-Ethyl 3-(3,5-dihydroxyhexyl)benzoate (3e)

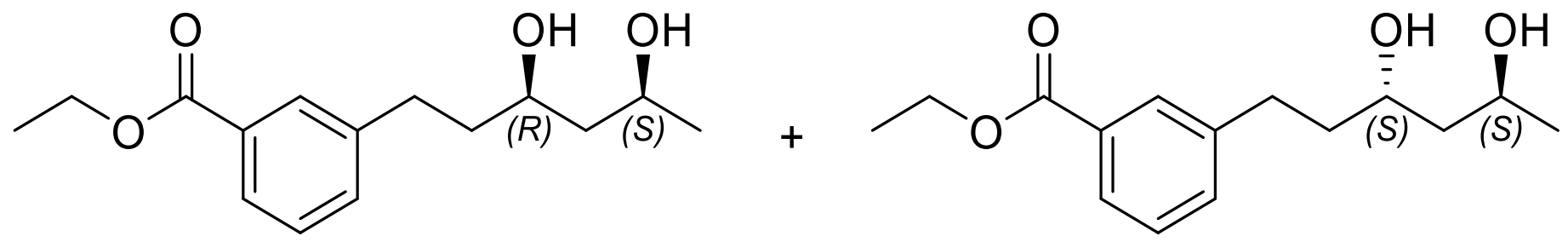

The compound was synthesised according to **General Procedure 7** using ethyl 3-((*anti*)-3,5-dihydroxyhexyl)benzoate (26.6 mg, 0.10 mmol), separated with DCM then 10 – 15% acetone in hexane, giving ethyl 3-((3*R*,5*S*)-3,5-dihydroxyhexyl)benzoate (10.6 mg, 40%, 48% *ee*) as a colourless oil and ethyl 3-((*S*,*S*)-3,5-dihydroxyhexyl)benzoate (15.1 mg, 57%, 99% *ee*) as a colourless solid.

Ethyl 3-((*S*,*S*)-3,5-dihydroxyhexyl)benzoate (*S*,*S*-3e)

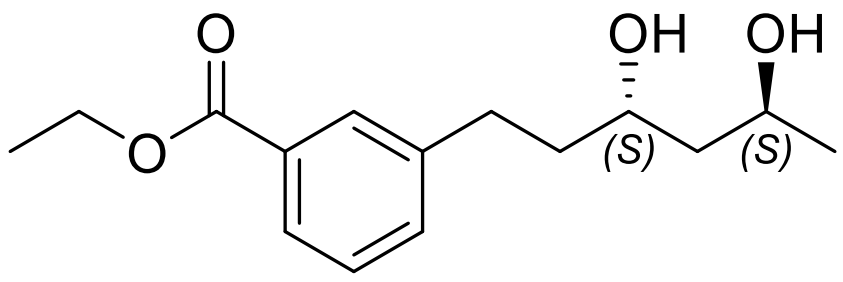

^{1}H NMR (500 MHz, $CDCl_3$) δ 7.91 – 7.84 (m, 2H), 7.42 – 7.38 (m, 1H), 7.35 (app. t, *J* = 7.6 Hz, 1H), 4.37 (q, *J* = 7.2 Hz, 2H), 4.23 – 4.15 (m, 1H), 3.97 (br. s, 1H), 2.90 – 2.81 (m, 1H), 2.77 – 2.68 (m, 1H), 2.58 (br. s, 1H), 2.19 (br. s, 1H), 1.92 – 1.83 (m, 1H), 1.82 – 1.74 (m, 1H), 1.70 – 1.59 (m, 2H), 1.39 (t, *J* = 7.2 Hz, 3H), 1.25 (d, *J* = 6.3 Hz, 3H) ppm.

^{13}C NMR (126 MHz, $CDCl_3$) δ 167.0, 142.5, 133.2, 130.7, 129.6, 128.6, 127.3, 68.7, 65.8, 61.1, 44.2, 39.1, 32.1, 23.8, 14.5 ppm.

$[\alpha]_D^{25}$ = -0.17 (*c* 0.60, $CHCl_3$). (The low specific rotation results in this measurement to lie within the error range of the instrument)

Chiral SFC Analysis: CHIRALPAK IK (CO_2 :MeOH, 93:7, 2.5 mL min^{-1}, 40 °C, 230 nm) t_R = 11.5 (major), 12.4 (minor) min, 99% *ee*.

Ethyl 3-((3*R*,5*S*)-3,5-dihydroxyhexyl)benzoate (3*R*,5*S*-3e)

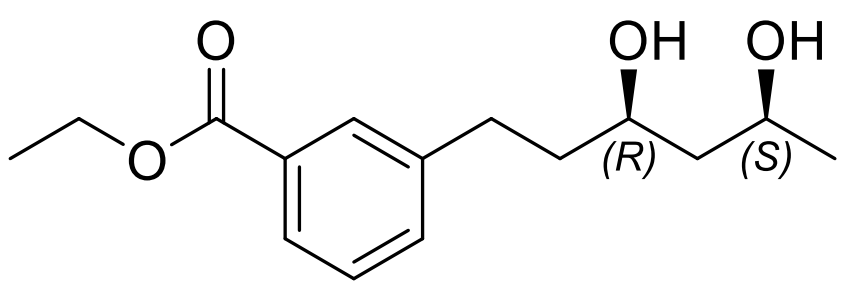

^{1}H NMR (500 MHz, $CDCl_3$) δ 7.91 – 7.85 (m, 2H), 7.41 – 7.38 (m, 1H), 7.35 (app. t, *J* = 7.6 Hz, 1H), 4.37 (q, *J* = 7.2 Hz, 2H), 4.10 – 4.01 (m, 1H), 3.92 – 3.85 (m, 1H), 3.09 (br. s, 1H), 2.87 – 2.78 (m, 1H), 2.78 – 2.69 (m, 1H), 2.55 (br. s, 1H), 1.87 – 1.73 (m, 2H), 1.63 – 1.53 (m, 2H), 1.40 (t, *J* = 7.2 Hz, 3H), 1.22 (d, *J* = 6.2 Hz, 3H) ppm.

^{13}C NMR (126 MHz, $CDCl_3$) δ 166.9, 142.4, 133.2, 130.8, 129.6, 128.6, 127.3, 72.2, 69.5, 61.1, 44.9, 39.8, 31.6, 24.6, 14.5 ppm.

$[\alpha]_D^{25}$ = +6.9 (*c* 0.42, $CHCl_3$).

Chiral SFC Analysis: CHIRALPAK IK (CO_2 :MeOH, 90:10, 2.5 mL min^{-1}, 40 °C, 230 nm) t_R = 7.56 (minor), 11.1 (major) min, 48% *ee*.

enriched-6-(2-(Trifluoromethyl)phenyl)hexane-2,4-diol (3f)

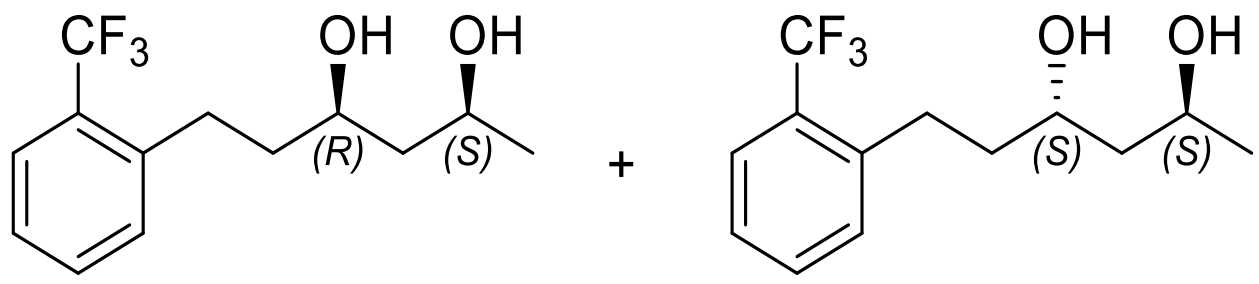

The compound was synthesised according to **General Procedure 7** using (*anti*)-6-(2-(trifluoromethyl)phenyl)hexane-2,4-diol (26.2 mg, 0.10 mmol), separated with DCM then 10% acetone in hexane, giving (2*S*,4*R*)-6-(2-(trifluoromethyl)phenyl)hexane-2,4-diol (9.9 mg, 38%, 60% *ee*) as a colourless oil and (*S*,*S*)-6-(2-(trifluoromethyl)phenyl)hexane-2,4-diol (13.7 mg, 52%, 99% *ee*) as a colourless solid.

(*S*,*S*)-6-(2-(Trifluoromethyl)phenyl)hexane-2,4-diol (*S*,*S*-3f)

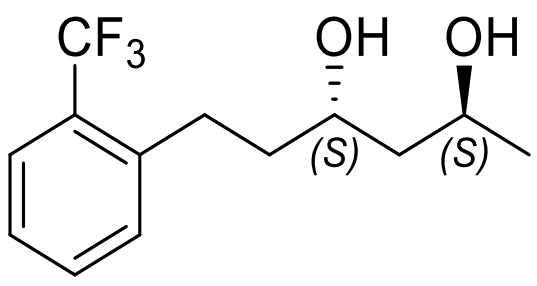

^{1}H NMR (700 MHz, $CDCl_3$) δ 7.62 (d, *J* = 7.9 Hz, 1H), 7.47 (app. t, *J* = 7.6 Hz, 1H), 7.36 (d, *J* = 7.7 Hz, 1H), 7.29 (app. t, *J* = 7.6 Hz, 1H), 4.24 – 4.17 (m, 1H), 4.07 – 4.00 (m, 1H), 3.01 – 2.94 (m, 1H), 2.86 – 2.79 (m, 1H), 2.50 (br. s, 1H), 2.09 (br. s, 1H), 1.88 – 1.81 (m, 1H), 1.81 – 1.74 (m, 1H), 1.70 – 1.62 (m, 2H), 1.26 (d, *J* = 6.3 Hz, 3H) ppm.

^{13}C NMR (176 MHz, $CDCl_3$) δ 141.1, 131.9, 131.3, 128.6 (q, *J* = 30.0 Hz), 126.12 (q, *J* = 5.8 Hz), 126.10, 124.8 (q, *J* = 274.2 Hz), 69.1, 65.8, 44.1, 39.5, 29.2, 23.8 ppm.

^{19}F NMR (376 MHz, $CDCl_3$) δ –59.6 ppm.

$[\alpha]_D^{25}$ = +3.5 (*c* 0.55, $CHCl_3$).

Chiral SFC Analysis: CHIRALPAK IK (CO_2:MeOH, 97:3, 2.5 mL min^{-1}, 40 °C, 207 nm) t_R = 8.5 (major), 9.0 (minor) min, 99% *ee*.

(2*S*,4*R*)-6-(2-(Trifluoromethyl)phenyl)hexane-2,4-diol (2*S*,4*R*-3f)

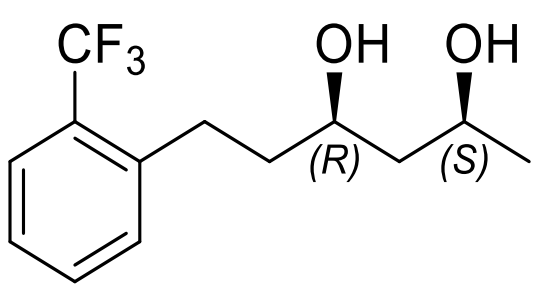

^{1}H NMR (700 MHz, $CDCl_3$) δ 7.61 (d, *J* = 7.9 Hz, 1H), 7.47 (app. t, *J* = 7.5 Hz, 1H), 7.36 (d, *J* = 7.7 Hz, 1H), 7.29 (app. t, *J* = 7.7 Hz, 1H), 4.12 – 4.06 (m, 1H), 3.98 – 3.92 (m, 1H), 3.09 (br. s, 1H), 2.98 – 2.92 (m, 1H), 2.87 – 2.80 (m, 1H), 2.55 (br. s, 1H), 1.82 – 1.73 (m, 2H), 1.64 – 1.54 (m, 2H), 1.24 (d, *J* = 6.2 Hz, 3H) ppm.

^{13}C NMR (176 MHz, $CDCl_3$) δ 141.1, 131.9, 131.3, 128.5 (q, *J* = 29.5 Hz), 126.12 (q, *J* = 5.7 Hz), 126.10, 124.8 (q, *J* = 273.8 Hz), 72.5, 69.5, 44.7, 40.1, 28.6, 24.6 ppm.

^{19}F NMR (376 MHz, $CDCl_3$) δ –59.6 ppm.

$[\alpha]_D^{25}$ = +7.8 (*c* 0.40, $CHCl_3$).

Chiral SFC Analysis: CHIRALPAK IK (CO_2:MeOH, 93:7, 2.5 mL min^{-1}, 40 °C, 207 nm) t_R = 3.8 (minor), 4.2 (major) min, 60% *ee*.

Enriched-4-(3,5-Dihydroxyhexyl)benzonitrile (3g)

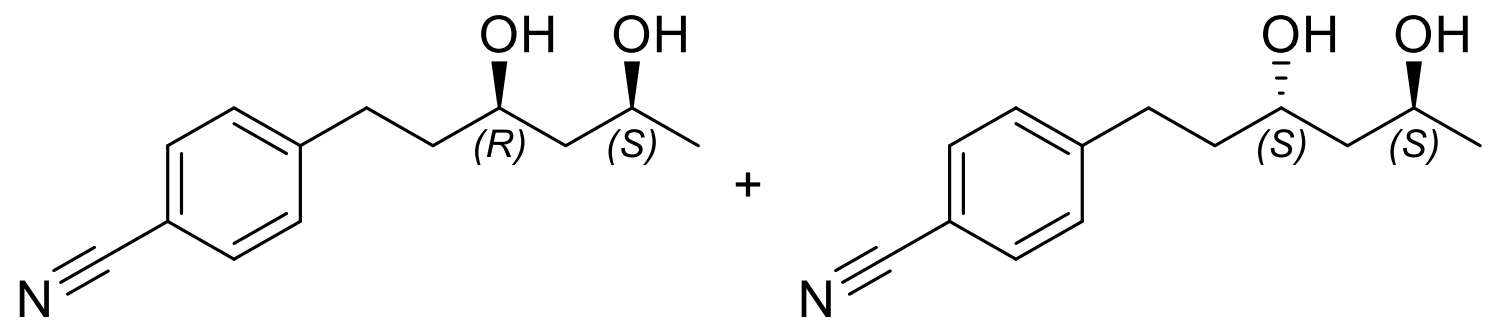

The compound was synthesised according to **General Procedure 7** using 4-((*anti*)-3,5-dihydroxyhexyl)benzonitrile (21.9 mg, 0.10 mmol), separated with DCM then 10% acetone in hexane, giving 4-((3*R*,5*S*)-3,5-dihydroxyhexyl)benzonitrile (6.4 mg, 29%, 71% *ee*) as a colourless oil and 4-((*S*,*S*)-3,5-dihydroxyhexyl)benzonitrile (12.6 mg, 57%, 99% *ee*) as a colourless solid.

4-((*S*,*S*)-3,5-Dihydroxyhexyl)benzonitrile (*S*,*S*-3g)

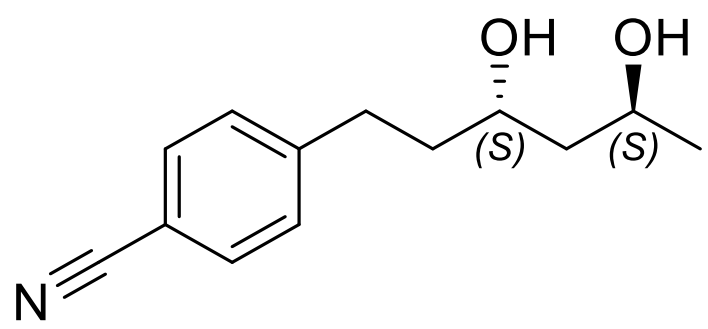

^{1}H NMR (700 MHz, $CDCl_3$) δ 7.57 (d, *J* = 8.3 Hz, 2H), 7.31 (d, *J* = 8.3 Hz, 2H), 4.22 – 4.16 (m, 1H), 3.98 – 3.92 (m, 1H), 2.92 – 2.84 (m, 1H), 2.77 – 2.71 (m, 1H), 2.68 (br. s, 1H), 2.06 (br. s, 1H), 1.88 – 1.81 (m, 1H), 1.78 – 1.71 (m, 1H), 1.68 – 1.59 (m, 2H), 1.25 (d, *J* = 6.3 Hz, 3H) ppm.

^{13}C NMR (176 MHz, $CDCl_3$) δ 148.0, 132.4, 129.4, 119.2, 109.9, 68.4, 65.8, 44.2, 38.6, 32.5, 23.8 ppm.

$[\alpha]_D^{25}$ = -2.6 (*c* 0.50, $CHCl_3$).

Chiral SFC Analysis: CHIRALPAK IA (CO_2:MeOH, 92:8, 2.5 mL min^{-1}, 40 °C, 230 nm) t_R = 10.3 (major), 11.5 (minor) min, 99% *ee*.

4-((3*R*,5*S*)-3,5-Dihydroxyhexyl)benzonitrile (3*R*,5*S*-3g)

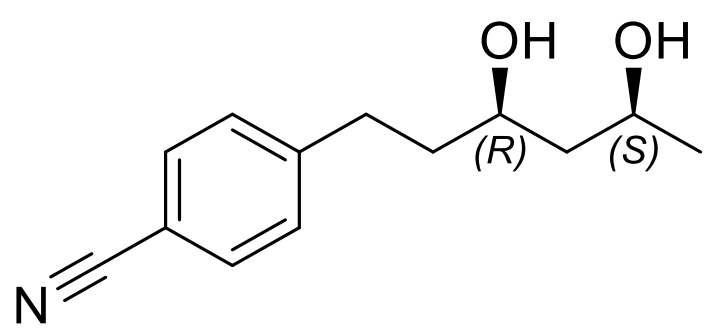

^{1}H NMR (700 MHz, $CDCl_3$) δ 7.57 (d, *J* = 8.2 Hz, 2H), 7.31 (d, *J* = 8.2 Hz, 2H), 4.10 – 4.03 (m, 1H), 3.90 – 3.82 (m, 1H), 3.34 (br. s, 1H), 2.87 – 2.81 (m, 1H), 2.79 – 2.72 (m, 1H), 2.31 (br. s, 1H), 1.82 – 1.70 (m, 2H), 1.60 – 1.53 (m, 2H), 1.23 (d, *J* = 6.2 Hz, 3H) ppm.

^{13}C NMR (176 MHz, $CDCl_3$) δ 148.0, 132.4, 129.4, 119.2, 109.9, 71.7, 69.6, 44.9, 39.2, 31.9, 24.8 ppm.

$[\alpha]_D^{25}$ = +14.2 (*c* 0.26, $CHCl_3$).

Chiral SFC Analysis: CHIRALPAK IK (CO_2:MeOH, 90:10, 2.5 mL min^{-1}, 40 °C, 230 nm) t_R = 10.1 (minor), 11.5 (major) min, 71% *ee*.

(*S*,*S*)-*N*-(4-(3,5-dihydroxyhexyl)phenyl)-2,2,2-trifluoroacetamide (*anti*-3h) and (*R*,*S*)-*N*-(4-(3,5-dihydroxyhexyl)phenyl)-2,2,2-trifluoroacetamide (*syn*-3h)

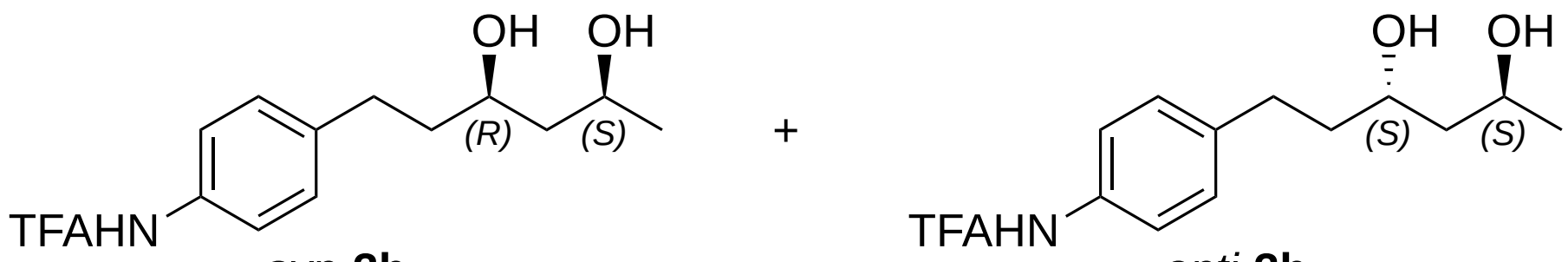

syn-**3h** *anti*-**3h**

Prepared according to **General Procedure 7** using diol substrate *rac-anti*-**3h** (30.5 mg, 0.1 mmol) and a concentration of 0.025 M. Purification by silica gel chromatography (elution with 15 – 20% Acetone in Pet. Ether) to give (*R*,*S*)-*N*-(4-(3,5-dihydroxyhexyl)phenyl)-2,2,2-trifluoroacetamide (11 mg, 0.037 mmol, 37%, 52% *ee*) as a white solid and (*S*,*S*)-*N*-(4-(3,5-dihydroxyhexyl)phenyl)-2,2,2-trifluoroacetamide (11 mg, 0.037 mmol, 37%, 97% *ee*) as a white solid.

(*R*,*S*)-*N*-(4-(3,5-dihydroxyhexyl)phenyl)-2,2,2-trifluoroacetamide (*syn*-3h)

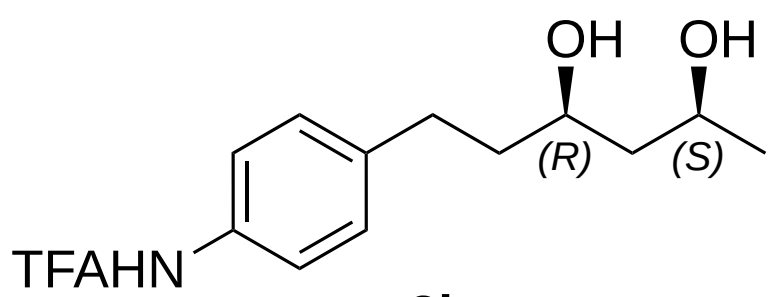

syn-**3h**

^{1}H NMR (700 MHz, $(CD_3)_2CO$) δ 10.16 (br s, 1H), 7.63 (d, *J* = 8.4 Hz, 2H), 7.26 (d, *J* = 8.4 Hz, 2H), 4.21 (d, *J* = 3.3 Hz, 1H), 4.12 (d, *J* = 3.2 Hz, 1H), 4.01 – 3.94 (m, 1H), 3.83 – 3.77 (m, 1H), 2.78 (ddd, *J* = 14.2, 9.0, 5.7 Hz, 1H), 2.67 (ddd, *J* = 13.8, 9.4, 7.1 Hz, 1H), 1.76 – 1.67 (m, 2H), 1.57 (dt, *J* = 14.0, 3.2 Hz, 1H), 1.51 (dt, *J* = 14.0, 9.2 Hz, 1H), 1.13 (d, *J* = 6.1 Hz, 3H) ppm.

^{13}C NMR (176 MHz, $(CD_3)_2CO$) δ 155.5 (q, *J* = 36.9 Hz), 141.3, 135.1, 129.8, 121.7, 117.0 (q, *J* = 288.1 Hz), 71.3, 68.3, 46.2, 40.7, 31.8, 24.6 ppm.

$[\alpha]_D^{25.0}$ = + 2.8 (*c* 0.32, $CHCl_3$)

Chiral HPLC Analysis CHIRALPAK SC (Hexane:iPrOH, 90:10, 1.25 ml min^{-1}, 40 °C) t_R = 16.0 (minor), 19.1 (major) minutes, 52% *ee*.

(*S*,*S*)-*N*-(4-(3,5-dihydroxyhexyl)phenyl)-2,2,2-trifluoroacetamide (*anti*-3h)

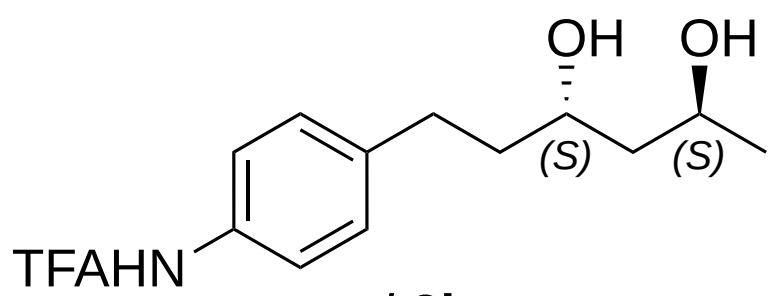

anti-**3h**

^{1}H NMR (700 MHz, $(CD_3)_2CO$) δ 10.16 (br s, 1H), 7.62 (d, *J* = 8.3 Hz, 2H), 7.27 (d, *J* = 8.3 Hz, 2H), 4.09 – 4.03 (m, 1H), 3.89 – 3.84 (m, 1H), 3.77 (d, *J* = 5.0 Hz, 1H), 3.67 (d, *J* = 4.0 Hz, 1H), 2.79 (ddd, *J* = 14.2, 8.8, 5.8 Hz, 1H), 2.68 (ddd, *J* = 13.9, 9.1, 7.3 Hz, 1H), 1.78 – 1.69 (m, 2H), 1.53 (t, *J* = 5.8 Hz, 2H), 1.14 (d, *J* = 6.2 Hz, 3H) ppm.

^{13}C NMR (176 MHz, $(CD_3)_2CO$) δ 155.5 (q, *J* = 37.0 Hz), 141.4, 135.0, 129.8, 121.7, 117.0 (q, *J* = 288.0 Hz), 68.2, 65.0, 46.7, 40.6, 32.2, 24.5 ppm.

$[\alpha]_D^{25.0}$ = + 6.6 (*c* 0.47, $CHCl_3$)

Chiral HPLC Analysis CHIRALPAK SC (Hexane:iPrOH, 90:10, 1.25 ml min^{-1}, 40 °C) t_R = 12.9 (minor), 14.1 (major) minutes, 97% *ee*.

enriched-6-Methyl-1-phenylheptane-3,5-diol (3i)

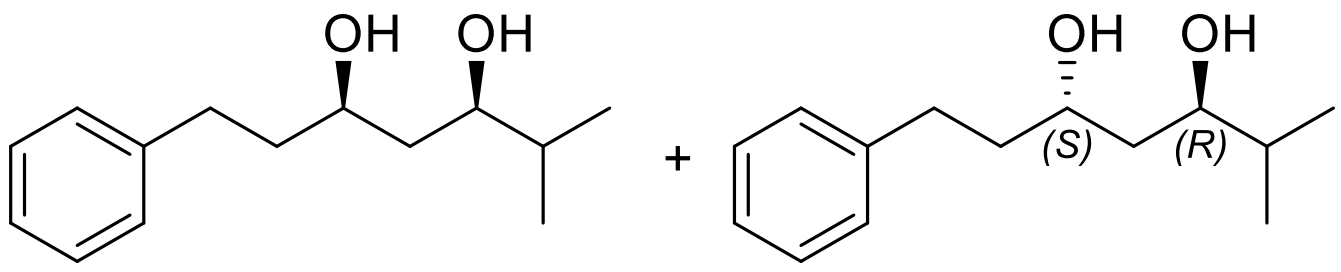

The compound was synthesised according to **General Procedure 7** using (*anti*)-6-methyl-1-phenylheptane-3,5-diol (22.2 mg, 0.10 mmol), separated with DCM then 5% acetone in hexane, giving enriched (*syn*)-6-Methyl-1-phenylheptane-3,5-diol (10.6 mg, 48%, 38% *ee*) as a colourless oil and (3*S*,5*R*)-6-methyl-1-phenylheptane-3,5-diol (10.8 mg, 49%, 99% *ee*) as a colourless solid.

(3*S*,5*R*)-6-Methyl-1-phenylheptane-3,5-diol (*S*,*R*-3i)

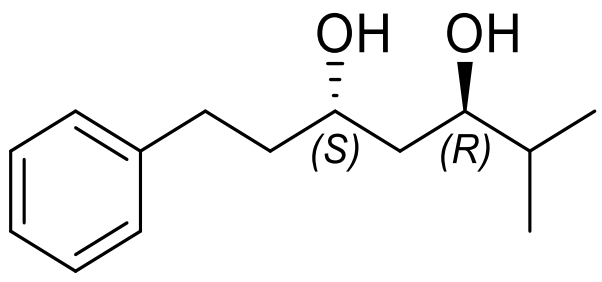

^{1}H NMR (500 MHz, $CDCl_3$) δ 7.31 – 7.27 (m, 2H), 7.23 – 7.16 (m, 3H), 4.01 – 3.93 (m, 1H), 3.73 – 3.65 (m, 1H), 2.85 – 2.76 (m, 1H), 2.72 – 2.63 (m, 1H), 2.05 – 1.75 (m, 4H), 1.72 – 1.63 (m, 2H), 1.60 (ddd, *J* = 14.5, 7.8, 2.8 Hz, 1H), 0.94 (d, *J* = 6.8 Hz, 3H), 0.90 (d, *J* = 6.8 Hz, 3H) ppm.

^{13}C NMR (126 MHz, $CDCl_3$) δ 142.2, 128.6, 128.5, 126.0, 74.2, 69.3, 39.6, 39.2, 34.0, 32.5, 18.7, 18.0 ppm.

$[\alpha]_D^{25}$ = +8.4 (*c* 0.43, $CHCl_3$).

Chiral SFC Analysis: CHIRALPAK IC (CO_2:MeOH, 95:5, 2.5 mL min^{-1}, 40 °C, 205 nm) t_R = 8.7 (minor), 9.2 (major) min, 99% *ee*.

Enriched (*syn*)-6-Methyl-1-phenylheptane-3,5-diol (*enriched-syn*-3i)

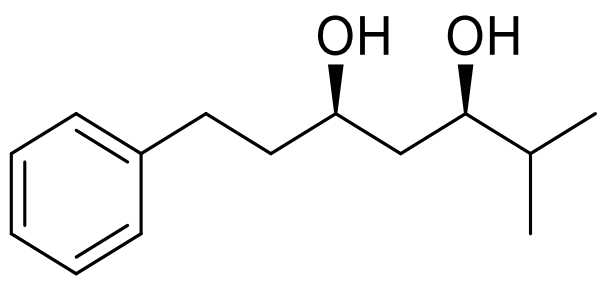

^{1}H NMR (500 MHz, $CDCl_3$) δ 7.31 – 7.27 (m, 2H), 7.23 – 7.16 (m, 3H), 3.91 – 3.83 (m, 1H), 3.67 – 3.60 (m, 1H), 2.83 – 3.74 (m, 1H), 3.74 – 2.65 (m, 1H), 2.28 (br. s, 2H), 1.87 – 1.73 (m, 2H), 1.70 – 1.63 (m, 1H), 1.61 (dt, *J* = 14.5, 2.0 Hz, 1H), 1.54 – 1.45 (m, 1H), 0.93 – 0.89 (m, 6H) ppm.

^{13}C NMR (126 MHz, $CDCl_3$) δ 142.2, 128.59, 128.56, 126.0, 78.3, 72.6, 39.9, 39.6, 34.5, 31.9, 18.4, 17.5 ppm.

$[\alpha]_D^{25}$ = -6.9 (*c* 0.42, $CHCl_3$).

Chiral SFC Analysis: CHIRALPAK IK (CO_2:MeOH, 93:7, 2.5 mL min^{-1}, 40 °C, 205 nm) t_R = 6.9 (major), 8.6 (minor) min, 38% *ee*.

***tert*-butyl 4-((2*S*,4*R*)-2,4-dihydroxy-6-phenylhexyl)piperidine-1-carboxylate (*syn*-3j) and *tert*-butyl 4-((2*S*,4*S*)-2,4-dihydroxy-6-phenylhexyl)piperidine-1-carboxylate (*anti*-3j)**

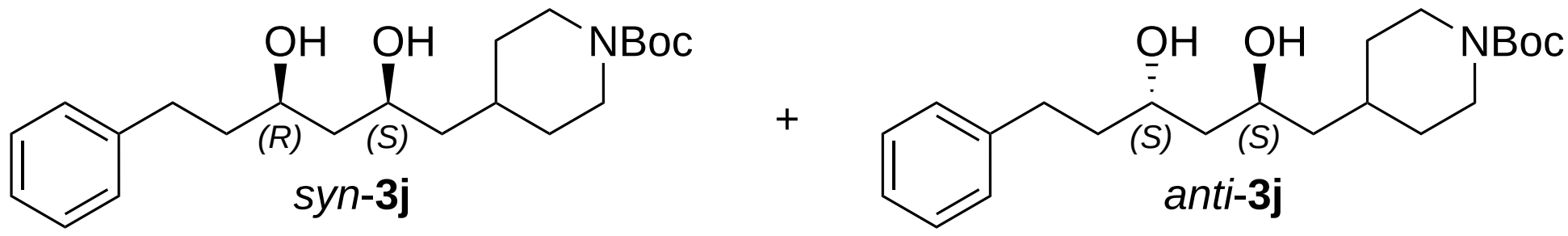

Prepared according to **General Procedure 7** using diol substrate *rac-anti*-**3j** (37.8 mg, 0.1 mmol) and a concentration of 0.025 M. Purification by silica gel chromatography (elution with 15 – 25% Acetone in Pet. Ether) to give the title compounds as a white solid in a mixture of diastereomers (1:1.2 d.r., 31.8 mg, 0.084 mmol, 84%, Syn: 76% *ee*, Anti: 94% *ee*).

^{1}H NMR (700 MHz, $CDCl_3$) δ 7.30 – 7.26 (m, 2H *syn*-**3j** & 2H *anti*-**3j**), 7.21 – 7.17 (m, 3H *syn*-**3j** & 3H *anti*-**3j**), 4.13 – 4.00 (m, 2H *syn*-**3j** & 3H *anti*-**3j**), 4.00 – 3.92 (m, 1H *syn*-**3j** & 1H *anti*-**3j**), 3.92 – 3.86 (m, 1H *syn*-**3j**), 2.82 – 2.61 (m, 4H *syn*-**3j** & 4H *anti*-**3j**), 1.90 – 1.69 (m, 3H *syn*-**3j** & 3H *anti*-**3j**), 1.66 – 1.46 (m, 5H *syn*-**3j** & 5H *anti*-**3j**), 1.45 (s, 9H *syn*-**3j** & 9H *anti*-**3j**), 1.29 – 1.23 (m, 1H *syn*-**3j** & 1H *anti*-**3j**), 1.17 – 1.09 (m, 1H *syn*-**3j** & 1H *anti*-**3j**), 1.09 – 1.01 (m, 1H *syn*-**3j** & 1H *anti*-**3j**).

^{13}C NMR (176 MHz, $CDCl_3$) δ 155.1 (*syn*-**3j** & *anti*-**3j**), 142.0 (*anti*-**3j**), 141.9 (*syn*-**3j**), 128.6 (*syn*-**3j** & *anti*-**3j**), 128.5 (*syn*-**3j**), 128.5 (*anti*-**3j**), 126.0 (*syn*-**3j** & *anti*-**3j**), 79.5 (*syn*-**3j** & *anti*-**3j**), 72.6 (*syn*-**3j**), 70.1 (*syn*-**3j**), 69.0 (*anti*-**3j**), 66.4 (*anti*-**3j**), 45.1 (*syn*-**3j**), 44.3 (*anti*-**3j**), 43.7 (*syn*-**3j**), 43.3 (*anti*-**3j**), 40.0 (*syn*-**3j**), 39.3 (*anti*-**3j**), 33.0 (*syn*-**3j** & *anti*-**3j**), 32.6 (*anti*-**3j**), 32.3 (*anti*-**3j**), 32.2 (*syn*-**3j**), 31.9 (*anti*-**3j**), 31.8 (*syn*-**3j**), 28.6 (*syn*-**3j** & *anti*-**3j**).

Chiral SFC Analysis Syn: CHIRALPAK IC (CO_2:MeOH, 80:20, 2.5 ml min^{-1}, 40 °C, 209 nm) t_R = 5.3 (minor), 7.3 (major) minutes, 76% *ee*.

Anti: CHIRALPAK IC (CO_2:MeOH, 80:20, 2.5 ml min^{-1}, 40 °C, 211 nm) t_R = 4.1 (major), 4.6 (minor) minutes, 94% *ee*.

(*S*,*S*)-1-Phenylnonane-3,5-diol (3k)

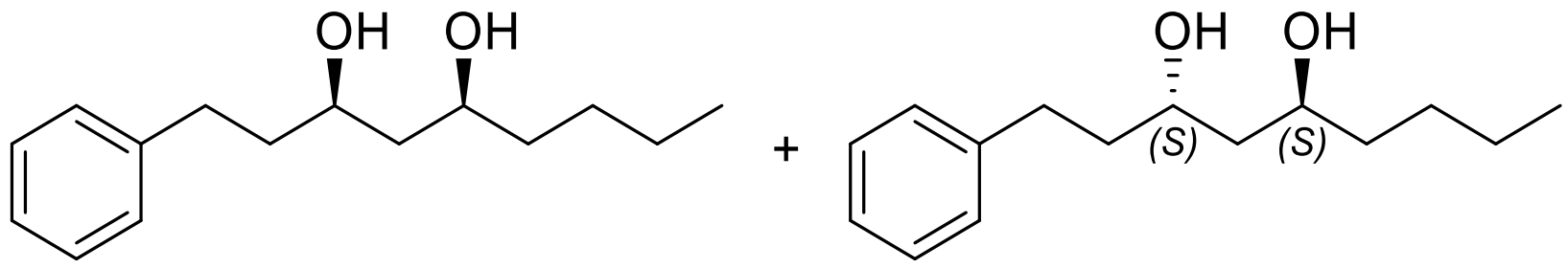

The compound was synthesised according to **General Procedure 7** using (*anti*)-1-phenylnonane-3,5-diol (23.6 mg, 0.10 mmol), separated with DCM then 5% acetone in hexane, giving enriched (*syn*)-1-phenylnonane-3,5-diol (7.1 mg, 30%, 9% *ee*) as a colourless oil and (*S*,*S*)-1-phenylnonane-3,5-diol (13.3 mg, 56%, 99% *ee*) as a colourless solid.

(*S*,*S*)-1-Phenylnonane-3,5-diol (*S*,*S*-3k)

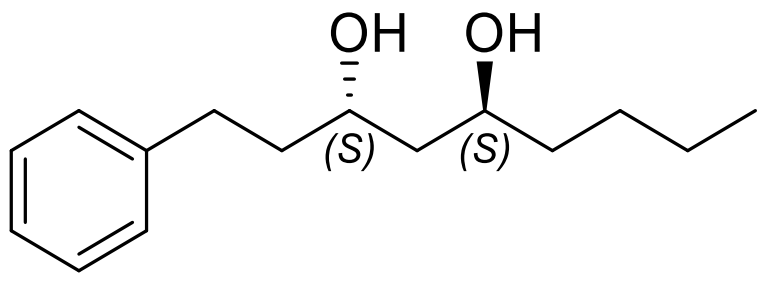

^{1}H NMR (500 MHz, $CDCl_3$) δ 7.31 – 7.26 (m, 2H), 7.23 – 7.15 (m, 3H), 4.03 – 3.91 (m, 2H), 2.84 – 2.75 (m, 1H), 2.72 – 2.62 (m, 1H), 2.32 (br. s, 2H), 1.92 – 1.73 (m, 4H), 1.70 – 1.60 (m, 2H), 1.57 – 1.23 (m, 6H), 0.91 (t, *J* = 6.9 Hz, 3H) ppm.

^{13}C NMR (126 MHz, $CDCl_3$) δ 142.1, 128.6, 128.5, 126.0, 69.7, 69.1, 42.6, 39.3, 37.4, 32.4, 28.1, 22.8, 14.2 ppm.

$[\alpha]_D^{25}$ = -4.3 (*c* 0.53, $CHCl_3$).

Chiral SFC Analysis: CHIRALPAK IC (CO_2:MeOH, 95:5, 2.5 mL min^{-1}, 40 °C, 205 nm) t_R = 10.7 (major), 11.3 (minor) min, 99% *ee*.

Enriched (*syn*)-1-Phenylnonane-3,5-diol (*enriched-syn*-3k)

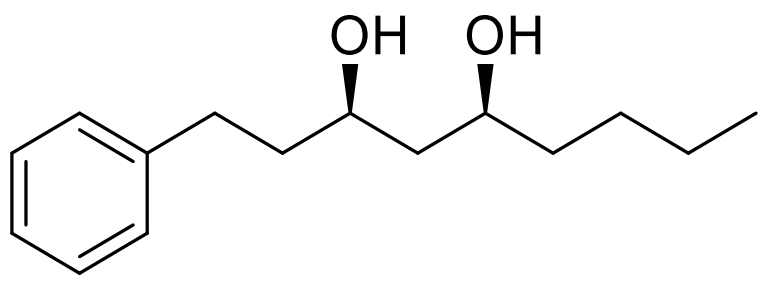

^{1}H NMR (500 MHz, $CDCl_3$) δ 7.31 – 7.27 (m, 2H), 7.23 – 7.16 (m, 3H), 3.93 – 3.80 (m, 2H), 2.82 – 2.73 (m, 1H), 2.73 – 2.65 (m, 1H), 2.11 (br. s, 2H), 1.86 – 1.72 (m, 2H,), 1.62 (dt, *J* = 14.5, 2.3 Hz, 1H), 1.56 – 1.41 (m, 3H), 1.40 – 1.25 (m, 4H), 0.91 (t, *J* = 7.0 Hz, 3H) ppm.

^{13}C NMR (126 MHz, $CDCl_3$) δ 142.1, 128.6, 126.0, 73.4, 72.5, 43.2, 39.9, 38.2, 31.9, 27.6, 22.8, 14.2 ppm.

$[\alpha]_D^{25}$ = -2.5 (*c* 0.28, $CHCl_3$).

Chiral SFC Analysis: CHIRALPAK IK (CO_2:MeOH, 93:7, 2.5 mL min^{-1}, 40 °C, 223 nm) t_R = 8.7 (minor), 10.5 (major) min, 9% *ee*.

(3*R*,5*S*)-1-phenyl-7-((2,3,3-trimethylbutan-2-yl)oxy)heptane-3,5-diol (*syn*-3l) and (3*S*,5*S*)-1-phenyl-7-((2,3,3-trimethylbutan-2-yl)oxy)heptane-3,5-diol (*anti*-3l)

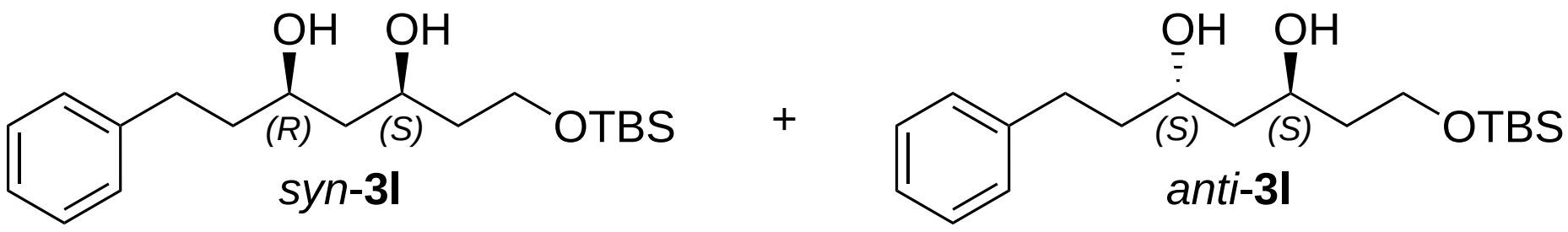

Prepared according to **General Procedure 7** using diol substrate *rac-anti*-**3l** (33.9 mg, 0.1 mmol) and a concentration of 0.025 M. Purification by silica gel chromatography (elution with 15 – 25% Acetone in Pet. Ether) to give the title compounds as a white solid in a mixture of diastereomers (1:1.2 d.r., 28.8 mg, 0.085 mmol, 85%, Syn: 4% *ee*, Anti: 96% *ee*).

^{1}H NMR (700 MHz, $CDCl_3$) δ 7.28 (app t, *J* = 7.6 Hz, 2H *syn*-**3l** & 2H *anti*-**3l**), 7.23 – 7.20 (m, 2H *syn*-**3l** & 2H *anti*-**3l**), 7.18 (app t, *J* = 7.3 Hz, 1H *syn*-**3l** & 1H *anti*-**3l**), 4.24 – 4.19 (m, 1H *anti*-**3l**), 4.13 – 4.08 (m, 1H *syn*-**3l**), 3.98 (sep, *J* = 4.0 Hz, 1H *anti*-**3l**), 3.94 – 3.88 (m, 2H *syn*-**3l** & 1H *anti*-**3l**), 3.86 – 3.80 (m, 1H *syn*-**3l** & 1H *anti*-**3l**), 2.85 – 2.76 (m, 1H *syn*-**3l** & 1H *anti*-**3l**), 2.72 – 2.64 (m, 1H *syn*-**3l** & 1H *anti*-**3l**), 1.90 – 1.79 (m, 1H *syn*-**3l** & 2H *anti*-**3l**), 1.78 – 1.60 (m, 4H *syn*-**3l** & 3H *anti*-**3l**), 1.60 – 1.56 (m, 1H *anti*-**3l**), 1.54 (dt, *J* = 14.1, 2.2Hz, 1H *syn*-**3l**), 0.90 (s, 9H *syn*-**3l** & 9H *anti*-**3l**), 0.09 (s, 6H *syn*-**3l** & 6H *anti*-**3l**) ppm.

^{13}C NMR (176 MHz, $CDCl_3$) δ 142.5 (*syn*-**3l**), 142.5 (*anti*-**3l**), 128.6 (*syn*-**3l**), 128.6 (*anti*-**3l**), 128.5 (*anti*-**3l**), 128.5 (*syn*-**3l**), 125.9 (*anti*-**3l**), 125.8 (*syn*-**3l**), 73.8 (*syn*-**3l**), 71.7 (*syn*-**3l**), 70.7 (*anti*-**3l**), 68.6 (*anti*-**3l**), 63.4 (*anti*-**3l**), 62.8 (*syn*-**3l**), 43.4 (*syn*-**3l**), 42.9 (*anti*-**3l**), 39.7 (*syn*-**3l**), 39.4 (*anti*-**3l**), 38.8 (*syn*-**3l**), 38.0 (*anti*-**3l**), 32.4 (*anti*-**3l**), 31.9 (*syn*-**3l**), 26.0 (*syn*-**3l** & *anti*-**3l**), 18.2 (*syn*-**3l** & *anti*-**3l**), -5.4 (*syn*-**3l** & *anti*-**3l**), -5.5 (*syn*-**3l** & *anti*-**3l**) ppm.

Chiral SFC Analysis Syn: CHIRALPAK IC (CO_2:MeOH, 97:03, 2.5 ml min^{-1}, 40 °C, 210 nm) t_R = 12.8 (minor), 13.9 (major) minutes, 4% *ee*.

Anti: CHIRALPAK IC (CO_2:MeOH, 97:03, 2.5 ml min^{-1}, 40 °C, 210 nm) t_R = 11.1 (minor), 11.5 (major) minutes, 96% *ee*.

(2*R*,4*S*)-1-tosylpentane-2,4-diol (*syn*-3m) and (2*S*,4*S*)-1-tosylpentane-2,4-diol (*anti*-3m)

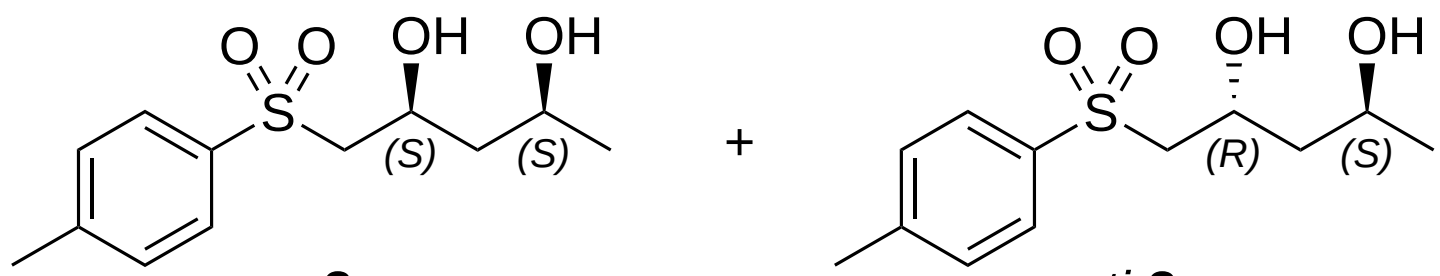

syn-**3m** *anti*-**3m**

Prepared according to **General Procedure 7** using diol substrate *rac*-*anti*-**3m** (25.8 mg, 0.1 mmol) and a concentration of 0.025 M. Purification by silica gel chromatography (elution with 5 – 20% Acetone in Pet. Ether) to give the title compounds as a white solid in a mixture of diastereomers (1:1.7 d.r., 20.7 mg, 0.085 mmol, 85%, Syn: 94% *ee*, Anti: 75% *ee*).

^{1}H NMR (700 MHz, $CDCl_3$) δ 7.80 (d, *J* = 8.3 Hz, 2H *syn*-**3m** & 2H *anti*-**3m**), 7.39 – 7.35 (m, 2H *syn*-**3m** & 2H *anti*-**3m**), 4.47 – 4.43 (m, 1H *anti*-**3m**), 4.42 – 4.38 (m, 1H *syn*-**3m**), 4.11 – 4.03 (m, 1H *syn*-**3m** & 1H *anti*-**3m**), 3.33 – 3.27 (m, 1H *syn*-**3m** & 1H *anti*-**3m**), 3.21 (dd, *J* = 14.3, 2.3 Hz, 1H *anti*-**3m**), 3.15 (dd, *J* = 14.3, 2.5 Hz, 1H *syn*-**3m**), 2.45 (s, 3H *syn*-**3m** & 3H *anti*-**3m**), 1.70 – 1.61 (m, 1H *syn*-**3m** & 1H *anti*-**3m**), 1.60 – 1.55 (m, 1H *syn*-**3m** & 1H *anti*-**3m**), 1.19 (d, *J* = 6.3 Hz, 3H *anti*-**3m**), 1.17 (d, *J* = 6.2 Hz, 3H *syn*-**3m**) ppm.

^{13}C NMR (176 MHz, $CDCl_3$) δ 145.3 (*syn*-**3m** & *anti*-**3m**), 136.4 (*syn*-**3m** & *anti*-**3m**), 130.2 (*syn*-**3m** & *anti*-**3m**), 128.1 (*syn*-**3m** & *anti*-**3m**), 67.8 (*syn*-**3m**), 66.9 (*syn*-**3m**), 64.8 (*anti*-**3m**), 64.2 (*anti*-**3m**), 62.6 (*syn*-**3m**), 62.3 (*anti*-**3m**), 44.1 (*syn*-**3m**), 43.9 (*anti*-**3m**), 23.9 (*syn*-**3m**), 23.8 (*anti*-**3m**), 21.8 (*syn*-**3m** & *anti*-**3m**) ppm.

Chiral SFC Analysis Syn: CHIRALPAK IA (CO_2:MeOH, 90:10, 2.5 ml min^{-1}, 40 °C, 228 nm) t_R = 7.2 (major), 7.5 (minor) minutes, 94% *ee*.

Anti: CHIRALPAK IA (CO_2:MeOH, 90:10, 2.5 ml min^{-1}, 40 °C, 228 nm) t_R = 8.1 (major), 11.7 (minor) minutes, 75% *ee*.

Scheme 7. Evaluation of Symmetrical 1,2-diols

(2*S*,3*S*)-butane-2,3-diol (4a)

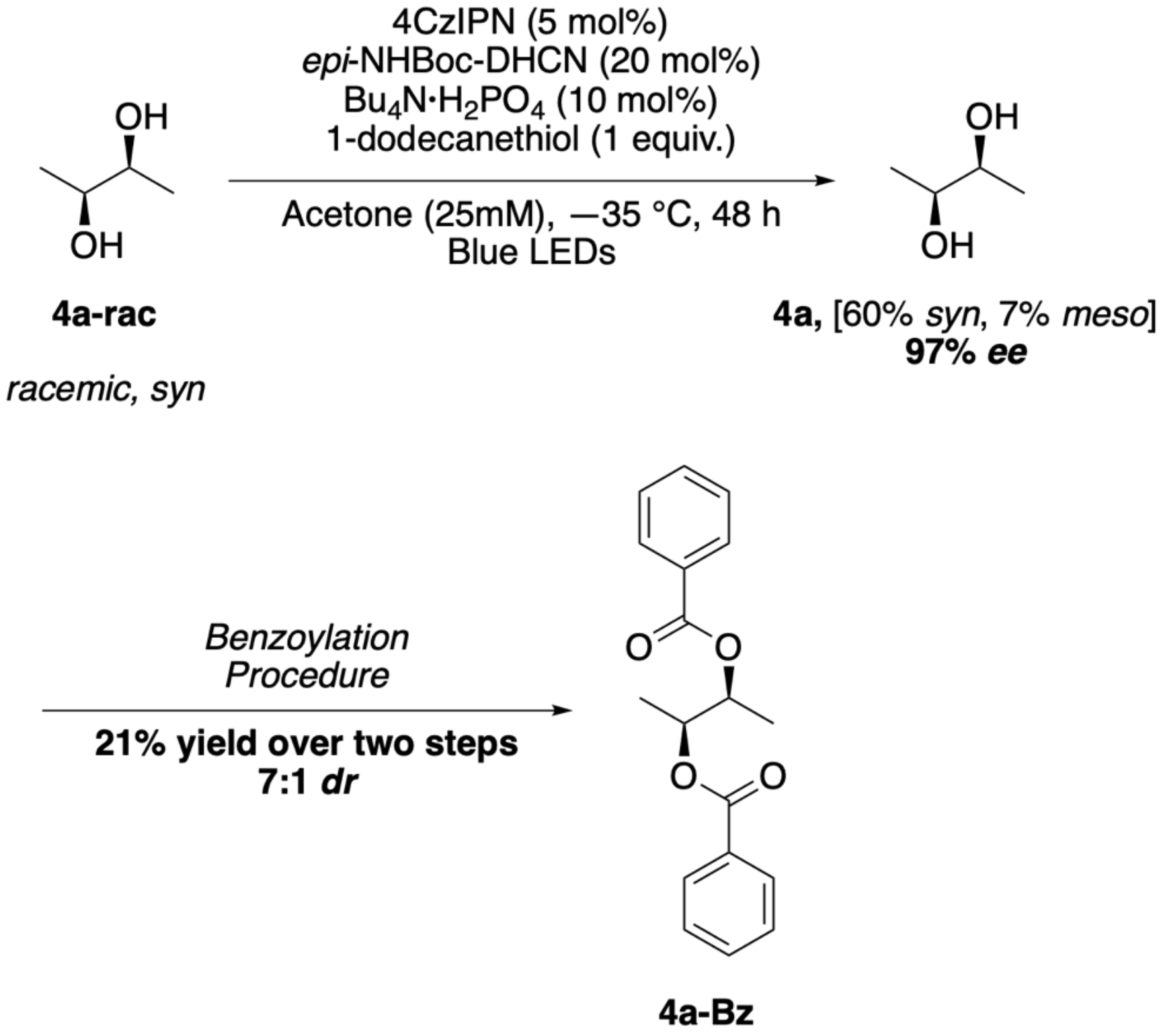

4a was prepared according to **General Procedure 7** using symmetrical *syn*-1,2-diol substrate **4a-rac** (9.0 mg, 0.10 mmol). On reaction completion, the mixture was concentrated *in vacuo* gently. Isolation at this point was avoided due to the volatility of **4a**. The crude material was brought forward without purification to **General Procedure 8** using DMAP (4.0 mg, 0.033 mmol), triethylamine (0.08 mL, 0.57 mmol) and benzoyl chloride (0.04 mL, 0.34 mmol) in DCM (1.0 mL). Purification by silica gel column chromatography (elution with 5% acetone in petroleum ether followed by a second purification eluting with 50 to 100% DCM in pentane) afforded **4a-Bz** (6.2 mg, 0.021 mmol, 21% yield over two steps, 97% ee) as a mixture of diastereomers (**4a-Bz**:*meso* = 7:1 *dr*).

Combined signals for both diastereomers (**4a-Bz** + *meso*) are denoted (‘). Discernable signals for the minor *meso* diastereomer are denoted (*).

^{1}H NMR (700 MHz, $CDCl_3$) δ 8.05 – 8.02’ (m, 4H), 7.56* (t, *J* = 7.6 Hz, 0.25H), 7.54 (t, *J* = 7.4 Hz, 1.75H), 7.43* (t, *J* = 7.8 Hz, 0.5H), 7.42 (t, *J* = 7.8 Hz, 3.5H), 5.40 – 5.34’ (m, 2H), 1.46 – 1.44* (m, 0.75H), 1.44 – 1.40 (m, 5.25H) ppm.

^{13}C NMR (176 MHz, $CDCl_3$) δ 166.1, 166.0*, 133.1’, 130.5*, 130.3, 129.79*, 129.77, 128.5’, 72.42, 72.38*, 16.5, 15.9* ppm.

HRMS $[M+Na]^+$ m/z calc’d for $[C_{18}H_{18}O_4Na]^+$ expect 321.1103; found 321.1110.

$[\alpha]_D^{25.0}$ = –8.0 (*c* 0.25, $CHCl_3$).

Chiral SFC Analysis: CHIRALPAK IE (CO_2:MeOH, 98:02, 2.5 mL min^{-1}, 40 °C, 225 nm) t_R = 9.3 (minor), 10.7 (major) minutes, 97% ee.

The data is in good agreement with the literature for the individual diastereomers of **4a-Bz**. [57,58]

NMR Yield The crude was analysed by ^{1}H NMR using CH_2Br_2 as an internal standard which showed 60% NMR yield of the *syn* diasteromer **4a** and 7% NMR yield of the *meso* diastereomer. The NMR yield of the *meso* diol was determined using discernible signals by comparison to the literature ^{1}H NMR data.[59]

Absolute Configuration (2*S*,3*S*)-butane-2,3-diyl dibenzoate and (2*R*,3*R*)-butane-2,3-diyl dibenzoate (enantiomerically pure dibenzoylated diols) were analogously synthesized according to **General Procedure 8** using commercial, enantiomerically pure diols. Analytical samples were obtained by purification by preparative thin layer chromatography (elution with 5% acetone in petroleum ether). Subjecting these to chiral SFC analysis shows the major enantiomer to have (*S*,*S*) absolute stereochemistry by signal comparison.

(3*S*,4*S*)-hexane-3,4-diol (4b)

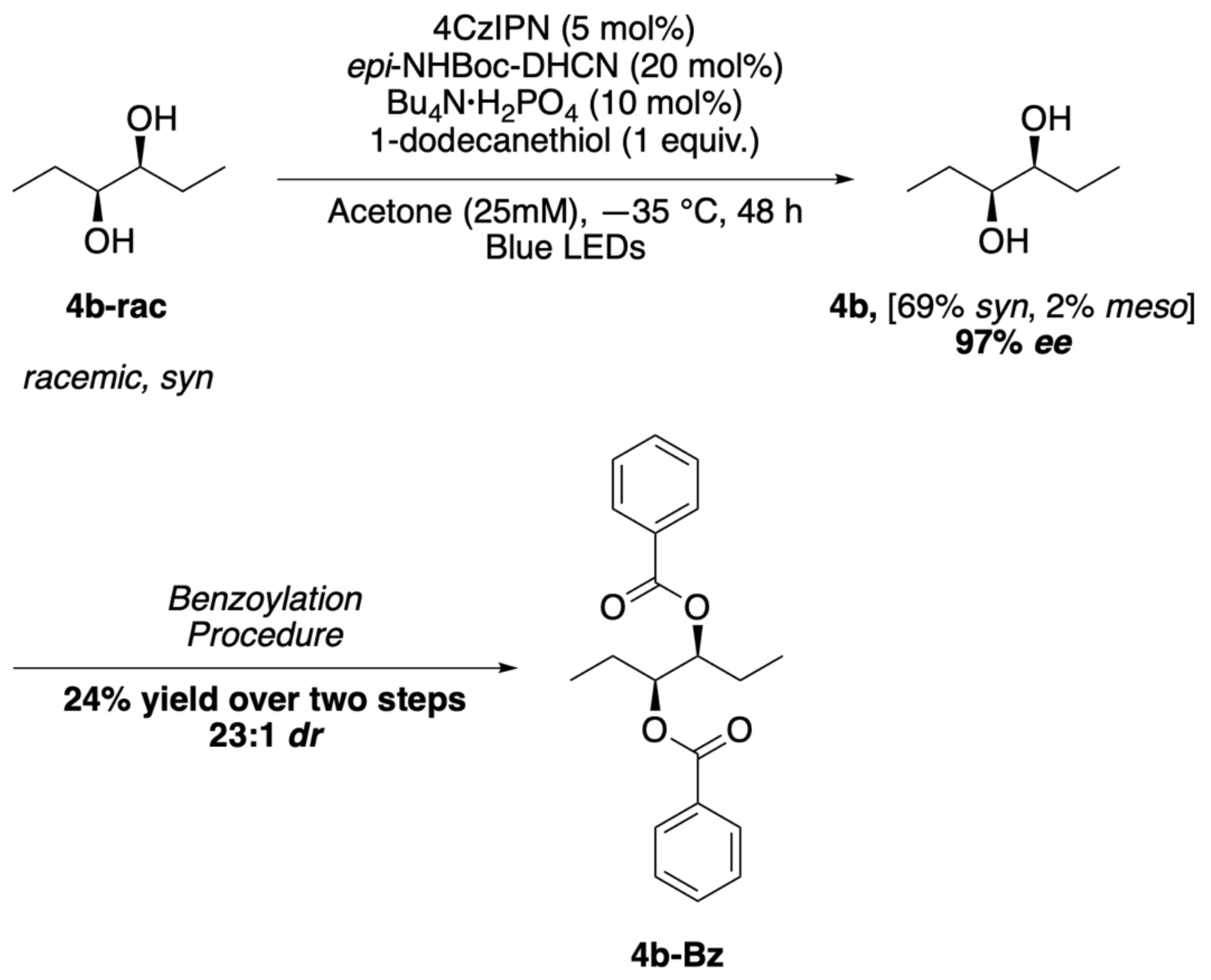

4b was prepared according to **General Procedure 7** using symmetrical *syn*-1,2-diol substrate **4b-rac** (11.8 mg, 0.10 mmol). On reaction completion, the mixture was concentrated *in vacuo* gently. Isolation at this point was avoided due to the volatility of **4b**. The crude material was brought forward without

purification to **General Procedure 8** using DMAP (4.0 mg, 0.033 mmol), triethylamine (0.08 mL, 0.57 mmol) and benzoyl chloride (0.04 mL, 0.34 mmol) in DCM (1.0 mL). Purification by silica gel column chromatography (elution with 4% acetone in petroleum ether followed by a second purification eluting with 50 to 100% DCM in pentane followed by a third purification eluting with 70% $CHCl_3$ in pentane) afforded **4b-Bz** (7.7 mg, 0.024 mmol, 24% yield over two steps, 97% ee) as a mixture of diastereomers (**4b-Bz**:*meso* = 23:1 *dr*).

Combined signals for both diastereomers (**4b-Bz** + *meso*) are denoted (‘). Discernable signals for the minor *meso* diastereomer are denoted (*).

^{1}H NMR (500 MHz, $CDCl_3$) δ 8.06 (d, *J* = 8.1 Hz, 3.83H), 8.03* (d, *J* = 8.2 Hz, 0.17H), 7.55’ (t, *J* = 7.4 Hz, 2H), 7.43’ (t, *J* = 7.7Hz, 4H), 5.35’ (app. quint, *J* = 4.7 Hz, 2H), 1.88 – 1.73’ (m, 4H), 0.99’ (t, *J* = 7.5 Hz, 6H) ppm.

^{13}C NMR (126 MHz, $CDCl_3$) δ 166.4, 166.2*, 133.1’, 130.3’, 129.8’, 128.5’, 76.0*, 75.6, 24.3, 23.3*, 10.1*, 9.9 ppm.

HRMS $[M+Na]^+$ m/z calc’d for $[C_{20}H_{22}O_4Na]^+$ expect 349.1416; found 349.1402.

$[\alpha]_D^{25.0}$ = –44.8 (*c* 0.28, $CHCl_3$).

Chiral SFC Analysis: CHIRALPAK IK (CO_2:MeOH, 99.5:0.5, 1.25 mL min^{-1}, 40 °C, 225 nm) t_R = 22.6 (minor), 23.9 (major), 97% ee.

The data is in good agreement with the literature previously reported by the group for *syn* **4b-Bz**.[1]

<u>*NMR Yield*</u> The crude was analysed by ^{1}H NMR using CH_2Br_2 as an internal standard which showed 69% NMR yield of the *syn* diasteromer **4b** and 2% NMR yield of the *meso* diastereomer. The NMR yield of the *meso* diol was determined using discernible signals by comparison to the literature ^{1}H NMR data.[60]

(4S,5S)-octane-4,5-diol (4c)

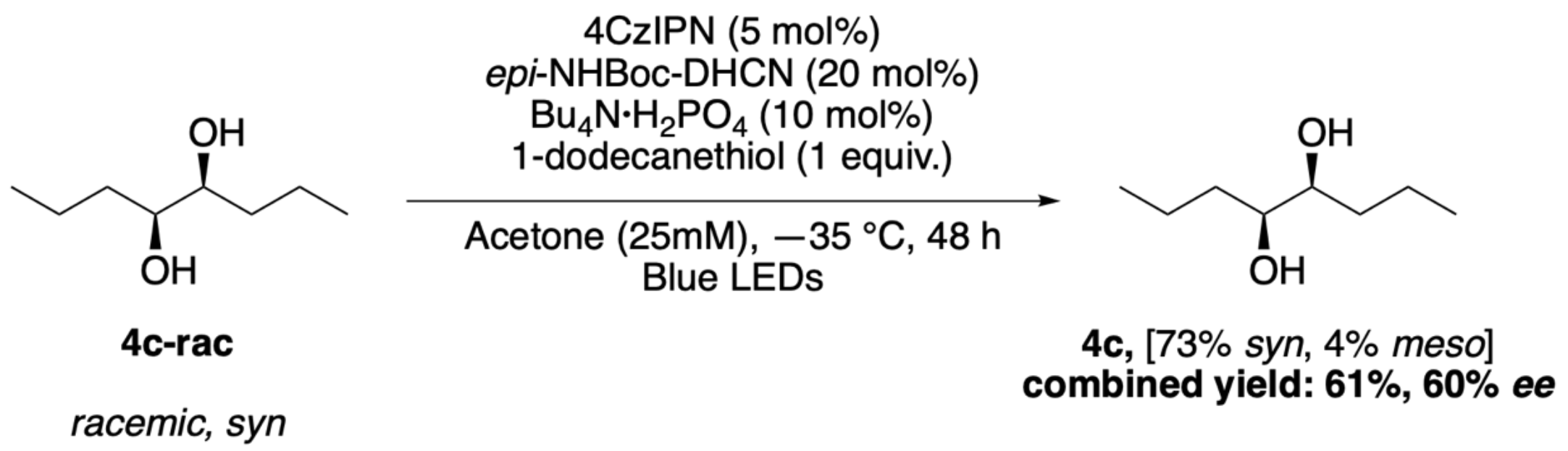

4c was prepared according to **General Procedure 7** using symmetrical *syn*-1,2-diol substrate **4c-rac** (14.6 mg, 0.10 mmol). Purification by silica gel column chromatography (first eluting with DCM to remove the photocatalyst then further eluting with 50% diethyl ether in pentane) afforded **4c** as a colorless oil (8.9 mg, 0.061 mmol, 61% yield) and as a mixture of diastereomers (**4c**:*meso* = 24:1 *dr*). After purification by silica gel column chromatography, the product was transferred exclusively with diethyl ether and concentrated carefully under *vacuo* due to its volatility.

Combined signals for both diastereomers (**4c** + *meso*) are denoted (‘). Discernable signals for the minor *meso* diastereomer are denoted (*).

^{1}H NMR (700 MHz, $CDCl_3$) δ 3.65 – 3.59* (m, 0.08H), 3.45 – 3.37 (m, 1.92H), 2.06 (br. s, 1.92H), 1.89* (br. s, 0.08H), 1.55 – 1.36’ (m, 8H), 0.95* (t, *J* = 7.3 Hz, 0.24H), 0.94 (t, *J* = 7.1 Hz, 5.76H) ppm.

^{13}C NMR (176 MHz, $CDCl_3$) δ 74.6*, 74.4, 35.9, 33.5*, 19.3*, 19.0, 14.2’ ppm.

HRMS $[M+Na]^+$ m/z calc’d for $[C_8H_{18}O_2Na]^+$ expect 169.1204; found 169.1208.

The *meso* diol assignments are in good agreement with the literature data.[61] A small amount of solvent is seen in the NMR spectra. Further concentration to remove these was avoided due to product volatility.

NMR Yield The crude was analysed by ^{1}H NMR using CH_2Br_2 as an internal standard which showed 73% NMR yield of the *syn* diasteromer **4c** and 4% NMR yield of the *meso* diastereomer.

(4*S*,5*S*)-octane-4,5-diyl dibenzoate (4c-Bz)

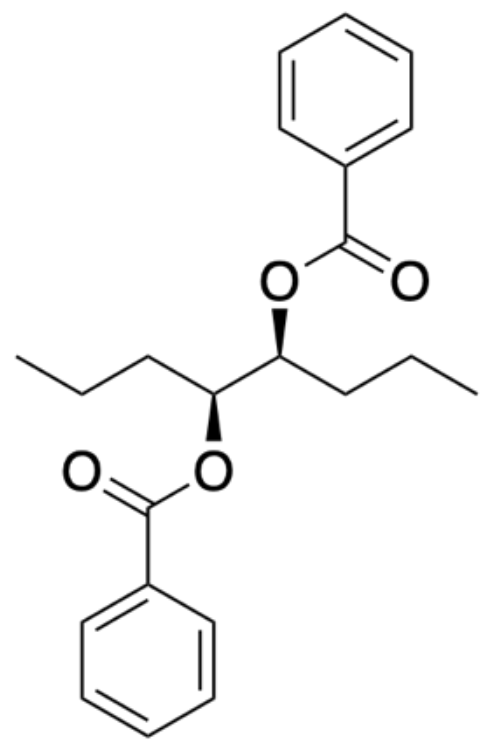

4c-Bz

4c-Bz was prepared according to **General Procedure 8** using **4c** (4.4 mg, 0.030 mmol), DMAP (0.9 mg, 0.0074 mmol), triethylamine (0.02 mL, 0.14 mmol) and benzoyl chloride (0.01 mL, 0.086 mmol) in DCM (0.4 mL). Purification by silica gel column chromatography (elution with 3% acetone in petroleum ether followed by a second purification eluting with 5% diethyl ether in pentane) afforded **4c-Bz** (5.0 mg, 0.014 mmol, 47% yield, 60% ee) as a mixture of diastereomers (**4c-Bz**:*meso* = 30:1 *dr*).

Combined signals for both diastereomers (**4c-Bz** + *meso*) are denoted (‘) and discernable signals for the minor *meso* diastereomer are denoted (*) for the ^{1}H NMR spectrum. Due to the high *dr*, the *meso* diastereomer only shows weak signals in the ^{13}C NMR spectrum. Therefore clear, discernable signals for the minor *meso* diastereomer are denoted (*) but it is unclear whether the remaining signals are combined with those for the *syn* diastereomer or are undetected.

^{1}H NMR (700 MHz, CD_3Cl) δ 8.05 (d, *J* = 8.3 Hz, 3.87H), 8.01* (d, *J* = 8.3 Hz, 0.13H), 7.54' (t, *J* = 7.4 Hz, 2H), 7.43' (t, *J* = 7.8 Hz, 4H), 5.44 – 5.39' (m, 2H), 1.79 – 1.72' (m, 2H), 1.72 – 1.65' (m, 2H), 1.42' (app. sext, *J* = 7.5 Hz, 4H), 0.95* (t, *J* = 7.3 Hz, 0.19H), 0.92 (t, *J* = 7.4 Hz, 5.81H) ppm.

^{13}C NMR (176 MHz, CD_3Cl) δ 166.3, 133.1, 130.3, 129.8, 128.5, 74.9*, 74.6, 33.3, 32.1*, 19.0*, 18.7, 14.1 ppm.

HRMS $[M+Na]^+$ m/z calc'd for $[C_{22}H_{26}O_4Na]^+$ expect 377.1729; found 377.1732.

Chiral SFC Analysis: CHIRALPAK IJ (CO_2:MeOH, 99.8:0.2, 1.25 mL min^{-1}, 40 °C, 225 nm) t_R = 7.9 (major), 9.4 (minor) minutes, 60% ee.

<u>*Assigning dr*</u> The individual diastereomers of **4c-Bz** are assigned by comparison to the *dr* of substrate **4c**. The major signals in the chiral SFC analysis of **4c-Bz** also confirm *syn* **4c-Bz** as the major diastereomer by comparison to the racemic *syn* sample.

(5*S*,6*S*)-decane-5,6-diol (4d)

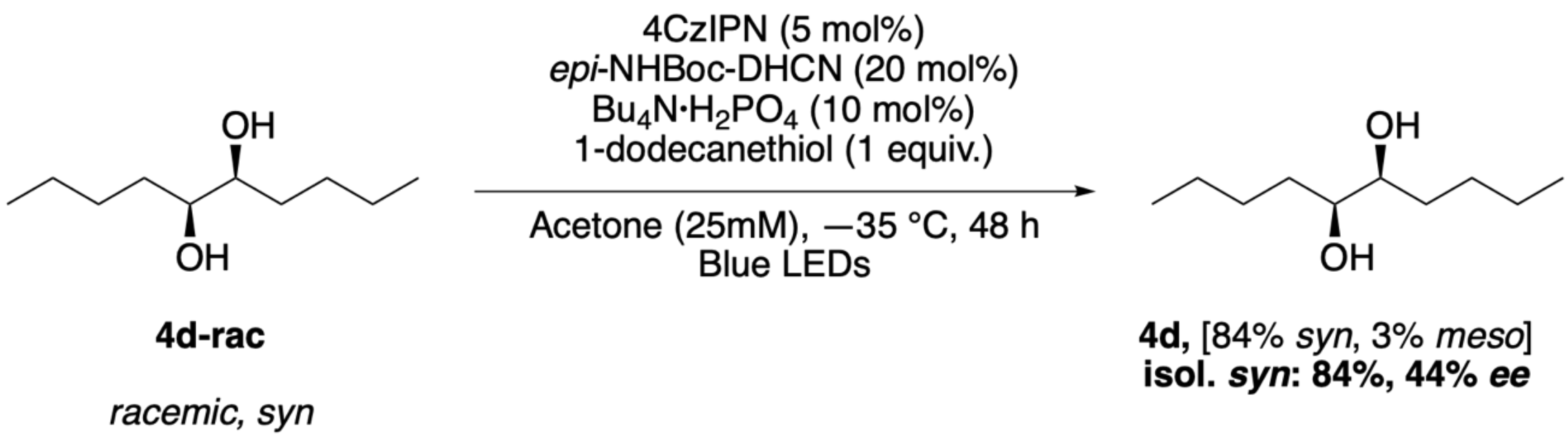

4d was prepared according to **General Procedure 7** using symmetrical *syn*-1,2-diol substrate **4d-rac** (17.4 mg, 0.10 mmol). Purification by silica gel column chromatography (first eluting with DCM to remove the photocatalyst then further eluting with 10% acetone in petroleum ether) afforded **4d** as a white solid (14.7mg, 0.084 mmol, 84% yield) and as a single diastereomer.

^{1}H NMR (700 MHz, $CDCl_3$) δ 3.44 – 3.36 (m, 2H), 2.08 (br. s, 2H), 1.55 – 1.48 (m, 2H), 1.48 – 1.40 (m, 4H), 1.40 – 1.29 (m, 6H), 0.91 (t, *J* = 7.1 Hz, 6H) ppm.

^{13}C NMR (176 MHz, $CDCl_3$) δ 74.7, 33.5, 28.0, 22.9, 14.2 ppm.

<u>*NMR Yield*</u> The crude was analysed by ^{1}H NMR using CH_2Br_2 as an internal standard which showed 84% NMR yield of the *syn* diastereomer **4d** and 3% NMR yield of the *meso* diastereomer. The NMR yield of the *meso* diol was determined using discernible signals by comparison to the literature ^{1}H NMR data.[62]

(5*S*,6*S*)-decane-5,6-diyl dibenzoate (4d-Bz)

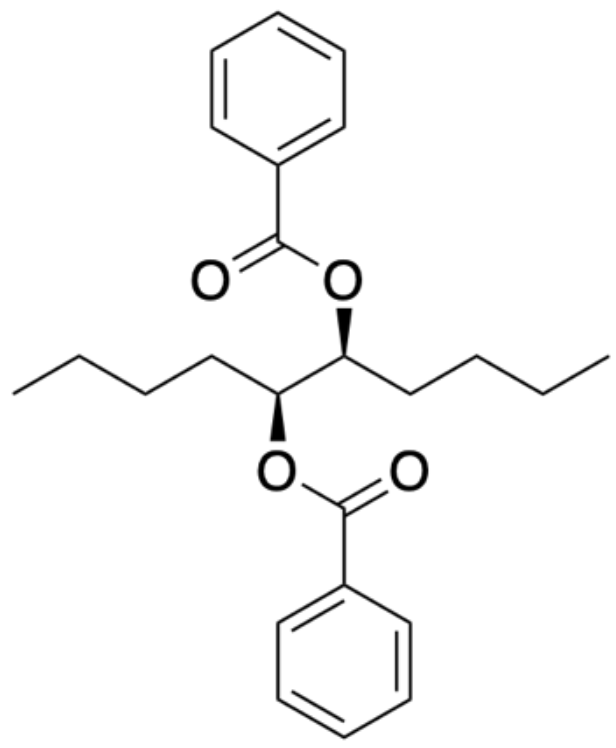

4d-Bz

4d-Bz was prepared according to **General Procedure 8** using **4d** (7.3 mg, 0.042 mmol), DMAP (1.0 mg, 0.0082 mmol), triethylamine (0.02 mL, 0.14 mmol) and benzoyl chloride (0.01 mL, 0.086 mmol) in DCM (0.4 mL). Purification by silica gel column chromatography (elution with 0 to 4% acetone in petroleum ether followed by a second purification eluting with 5% diethyl ether in petroleum ether) afforded **4d-Bz** (7.7 mg, 0.020 mmol, 48% yield, 44% ee).

^{1}H NMR (700 MHz, CD_3Cl) δ 8.05 (d, *J* = 8.4 Hz, 4H), 7.54 (t, *J* = 7.4 Hz, 2H), 7.43 (t, *J* = 7.8 Hz, 4H), 5.39 (app. quint, *J* = 4.7 Hz, 2H), 1.79 – 1.69 (m, 4H), 1.41 – 1.26 (m, 8H), 0.86 (t, *J* = 7.2 Hz, 6H) ppm.

^{13}C NMR (176 MHz, CD_3Cl) δ 166.3, 133.1, 130.3, 129.8, 128.5, 74.8, 30.9, 27.5, 22.7, 14.1 ppm.

Chiral SFC Analysis: CHIRALPAK IG (CO_2:MeOH, 97:03, 1.25 mL min^{-1}, 40 °C, 225 nm) t_R = 9.0 (minor), 10.8 (major) minutes, 44% ee.

The data is in good agreement with the data previously reported by the group.[1]

di-*tert*-butyl (4*S*, 5*S*)-4,5-dihydroxyoctanedioate (4e)

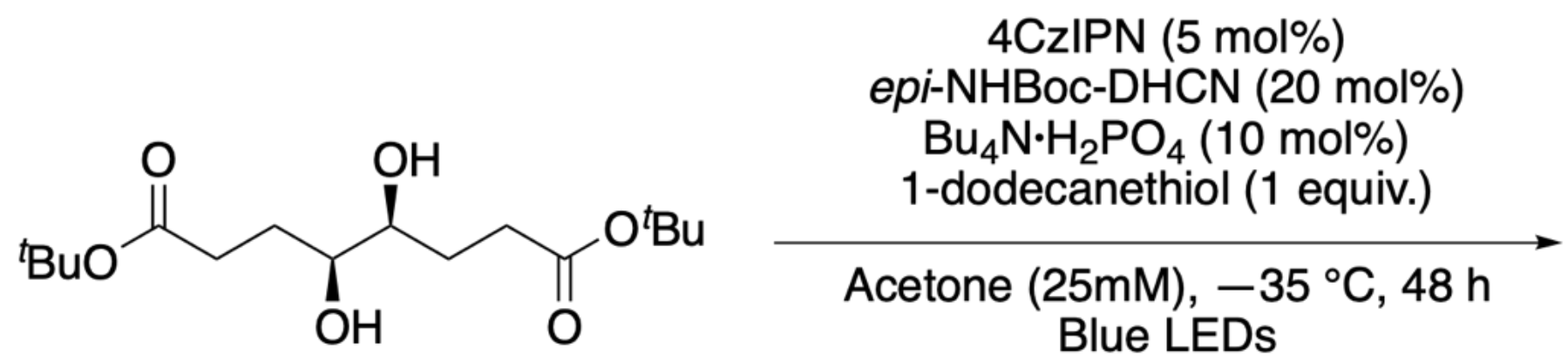

4e-rac

racemic, syn

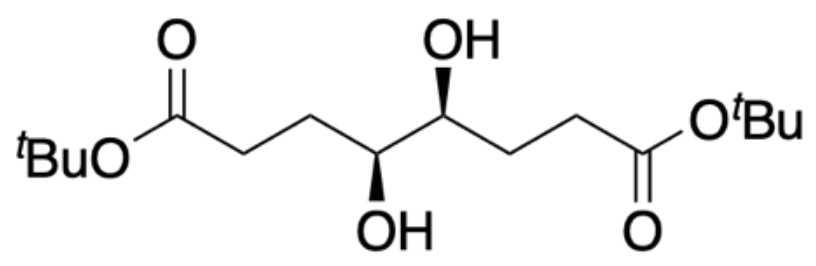

4e, [66% *syn*, 20% *meso*]
combined yield: 86%, 52% *ee*

4e was prepared according to **General Procedure 7** using symmetrical *syn*-1,2-diol substrate **4e-rac** (31.8 mg, 0.10 mmol). Purification by silica gel column chromatography (first eluting with DCM to remove the photocatalyst then further eluting with 15% acetone in petroleum ether) afforded **4e** as a colourless oil that slowly forms an off-white waxy solid (27.3 mg, 0.086 mmol, 86% yield) and as a mixture of diastereomers (**4e**:*meso* = 7:2 *dr*).

Combined signals for both diastereomers (**4e** + *meso*) are denoted (‘). Discernable signals for the minor *meso* diastereomer are denoted (*).

^{1}H NMR (500 MHz, $CDCl_3$) δ 3.58 – 3.52* (m, 0.44H), 3.45 – 3.38 (m, 1.56H), 2.92 (d, *J* = 4.9 Hz, 1.56H), 2.88* (d, *J* = 4.5 Hz, 0.44H), 2.48 – 2.36’ (m, 4H), 1.86 – 1.69’ (m, 4H) (overlaps with H_2O peak), 1.44’ (s, 18H) ppm.

^{13}C NMR (126 MHz, $CDCl_3$) δ 174.2*, 174.0, 80.92*, 80.87, 74.2*, 74.0, 32.3*, 32.1, 28.6, 28.2’, 26.8* ppm.

HRMS $[M+Na]^+$ m/z calc’d for $[C_{16}H_{30}O_6Na]^+$ expect 341.1940; found 341.1940.

The *meso* diol assignments are in good agreement with the literature.[38] The data is also in good agreement with a mixture of the diastereomers previously reported by the group.[1]

<u>*NMR Yield*</u> The crude was analysed by ^{1}H NMR using CH_2Br_2 as an internal standard which showed 66% NMR yield of the *syn* diastereomer **4e** and 20% NMR yield of the *meso* diastereomer.

di-*tert*-butyl (4*S*,5*S*)-4,5-bis(benzoyloxy)octanedioate (4e-Bz)

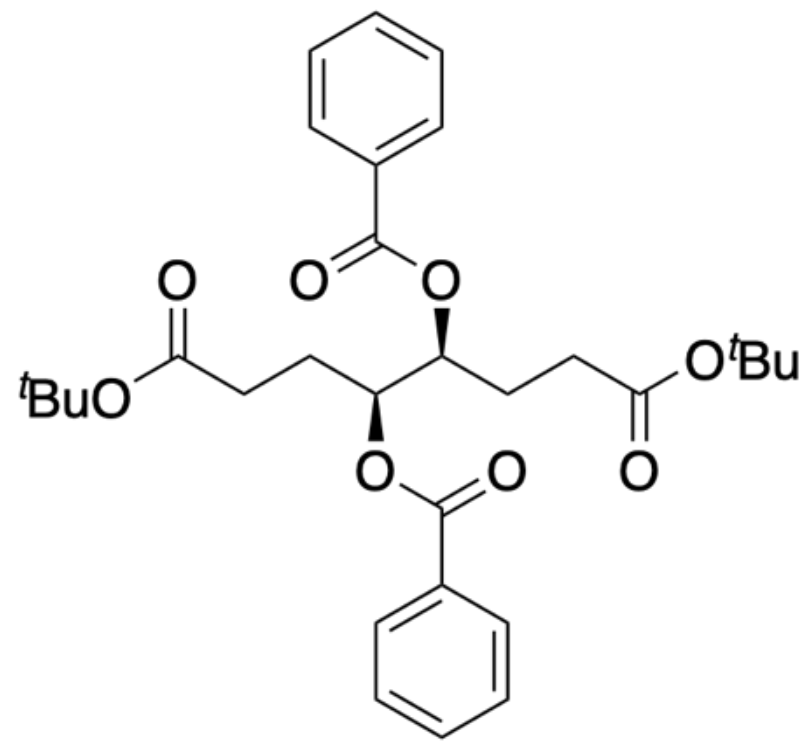

4e-Bz

4e-Bz was prepared according to **General Procedure 8** using **4e** (13.6 mg, 0.043 mmol), DMAP (1.0 mg, 0.0082 mmol), triethylamine (0.02 mL, 0.14 mmol) and benzoyl chloride (0.01 mL, 0.086 mmol) in DCM (0.4 mL). Purification by silica gel column chromatography (elution with 0.5% MeOH in DCM) afforded **4e-Bz** (20.7 mg, 0.039 mmol, 92% yield, 52% ee) as a mixture of diastereomers (**4e-Bz**:*meso* = 7:2 *dr*).

Combined signals for both diastereomers (**4e** + *meso*) are denoted (‘). Discernable signals for the minor *meso* diastereomer are denoted (*).

^{1}H NMR (500 MHz, CD_3Cl) δ 8.01 (dd, *J* = 8.3, 1.3 Hz, 3.5H), 8.00 – 7.98* (m, 0.5H), 7.58 – 7.50’ (m, 2H), 7.41’ (app. q, *J* = 8.0 Hz, 4H), 5.48 – 5.38’ (m, 2H), 2.43 – 2.28’ (m, 4H), 2.16 – 1.97’ (m, 4H), 1.41* (s, 4H), 1.40 (s, 14H) ppm.

^{13}C NMR (126 MHz, CD_3Cl) δ 172.1*, 172.0, 166.1, 166.0*, 133.3’, 130.0*, 129.9’, 129.8, 128.54*, 128.51, 80.78*, 80.77, 74.0*, 73.6, 31.7*, 31.4, 28.2’, 26.3, 25.2* ppm.

HRMS $[M+Na]^+$ m/z calc’d for $[C_{30}H_{38}O_8Na]^+$ expect 549.2464; found 549.2463.

Chiral SFC Analysis: CHIRALPAK IK (CO_2:MeOH, 98:02, 1.25 mL min^{-1}, 40 °C, 225 nm) t_R = 22.3 (minor), 23.9 (major) minutes, 52% ee.

The data is in good agreement with the literature previously reported by the group for the mixture of diastereomers.[1]

(2*S*,3*S*)-1,4-diphenylbutane-2,3-diol (4f)

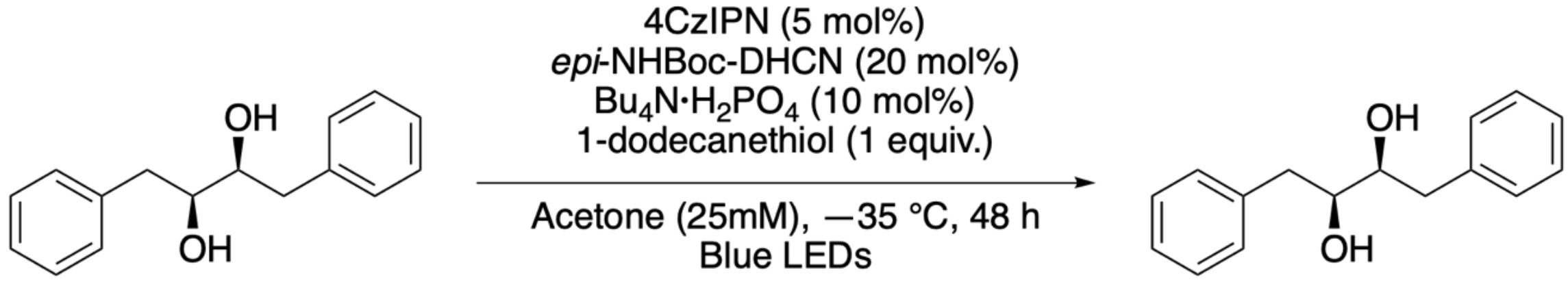

4f-rac

racemic, syn

4f, [87% *syn*, <1% *meso*]
isol. ***syn*****: 87%, 3%** ***ee***

4f was prepared according to **General Procedure 7** using symmetrical *syn*-1,2-diol substrate **4f-rac** (24.2 mg, 0.10 mmol). Purification by silica gel column chromatography (first eluting with DCM to remove the photocatalyst then further eluting with 15% acetone in petroleum ether) afforded **4f** as a white solid (21.0 mg, 0.087 mmol, 87% yield, racemic) and as a single diastereomer.

^{1}H NMR (700 MHz, $CDCl_3$) δ 7.31 (t, *J* = 7.4 Hz, 4H), 7.26 – 7.21 (m, 6H), 3.79 – 3.73 (m, 2H), 2.92 (dd, *J* = 13.7, 4.4 Hz, 2H), 2.85 (dd, *J* = 13.7, 8.3 Hz, 2H), 2.07 (d, *J* = 4.1 Hz, 2H) ppm.

^{13}C NMR (176 MHz, $CDCl_3$) δ 138.2, 129.5, 128.8, 126.7, 74.1, 40.5 ppm.

Chiral HPLC Analysis: CHIRALPAK IH (Hexane:^{i}PrOH, 85:15, 1.25 mL min^{-1}, 40 °C, 209 nm) t_R = 9.7 (major), 13.8 (minor) minutes, 3% ee.

NMR Yield The crude was analysed by ^{1}H NMR using CH_2Br_2 as an internal standard which showed 87% NMR yield of the *syn* diasteromer **4f** and trace NMR yield of the *meso* diastereomer. The NMR yield of the *meso* diol was determined using discernible signals by comparison to the literature ^{1}H NMR data.[63]

(4*S*,5*S*)-1,8-diphenyloctane-4,5-diol (4g)

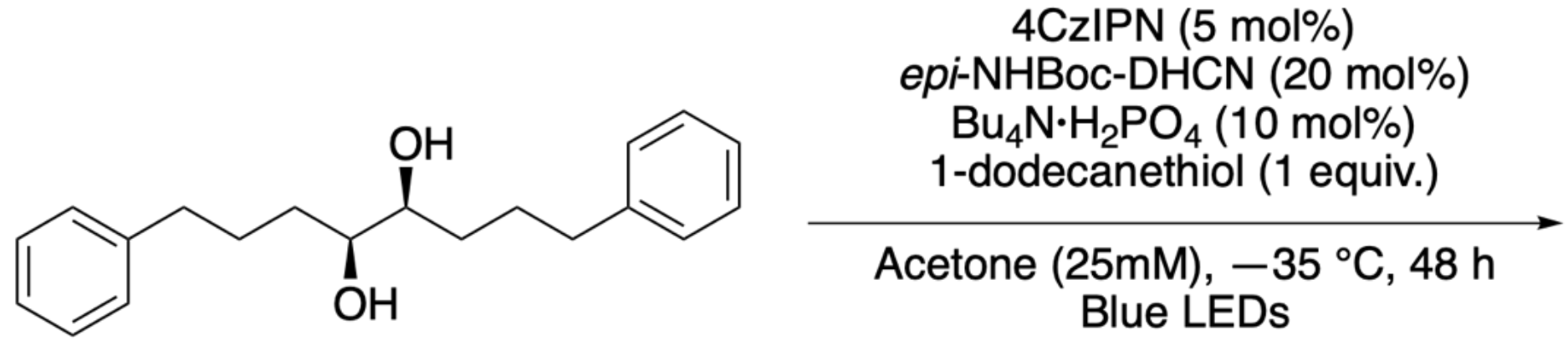

4g-rac

racemic, syn

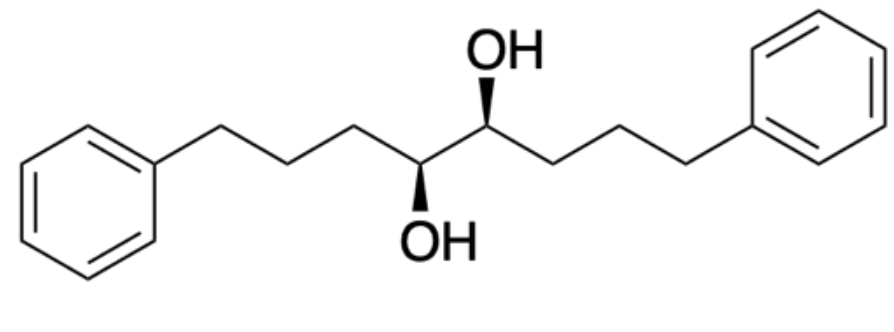

4g, [76% *syn*, 5% *meso*]
combined yield: 78%, 27% ***ee***

4g was prepared according to **General Procedure 7** using symmetrical *syn*-1,2-diol substrate **4g-rac** (29.8 mg, 0.10 mmol). Purification by silica gel column chromatography (first eluting with DCM to remove the photocatalyst then further eluting with 12% acetone in petroleum ether) afforded **4g** as a white solid together with a small amount of inseparable impurity (23.3 mg, 0.078 mmol, 78% yield, 27% ee) and as a mixture of diastereomers (**4g**:*meso* = 17:1 *dr*).

Combined signals for both diastereomers (**4g** + *meso*) are denoted (‘). Discernable signals for the minor *meso* diastereomer are denoted (*).

^{1}H NMR (700 MHz, $CDCl_3$) δ 7.28’ (t, *J* = 7.6 Hz, 4H), 7.22 – 7.16’ (m, 6H), 3.64 – 3.58* (m, 0.1H), 3.47 – 3.38 (m, 1.9H), 2.72 – 2.57’ (m, 4H), 2.13* (br. d, *J* = 4.3 Hz, 0.1H), 2.04 (br. d, *J* = 2.2 Hz, 1.9H), 1.91 – 1.87* (m, 0.1H), 1.86 – 1.76’ (m, 2H), 1.73 – 1.65 (m, 1.9H), 1.65 – 1.59* (m, 0.1H) (overlaps with H_2O signal), 1.56 – 1.50 (m, 1.9H), 1.50 – 1.43’ (m, 2H).

^{13}C NMR (176 MHz, $CDCl_3$) δ 142.3’, 128.5’ (2C), 125.9’, 74.5*, 74.4, 35.9, 33.3, 30.9*, 27.9*, 27.5 ppm.

HRMS $[M+Na]^+$ m/z calc’d for $[C_{20}H_{26}O_2Na]^+$ expect 321.1830; found 321.1832.

Chiral SFC Analysis: CHIRALPAK IG (CO_2:MeOH, 75:25, 2.5 mL min^{-1}, 40 °C, 208 nm) t_R = 6.8 (major), 12.2 (minor) minutes, 27% ee.

The *meso* diol assignments are in good agreement with the literature previously reported by the group.[1,2]

<u>*NMR Yield*</u> The crude was analysed by ^{1}H NMR using CH_2Br_2 as an internal standard which showed 76% NMR yield of the *syn* diasteromer **4g** and 5% NMR yield of the *meso* diastereomer.

<u>*Absolute Configuration*</u> Chiral SFC analysis of a mixture of **4g** and the *meso* diastereomer has previously been reported by the group using these SFC conditions.[1] The signal at t_R = 6.8 minutes corresponds to the (*S*,*S*) enantiomer and the signal at t_R = 12.2 minutes corresponds to the (*R*,*R*) enantiomer. Deracemization is therefore determined to form an excess of the (S,S)-enantiomer.

Scheme 8 – Evaluation of non-symmetrical 1,2-diols

Enriched 5-Phenylpentane-2,3-diol (4h) starting from *syn*

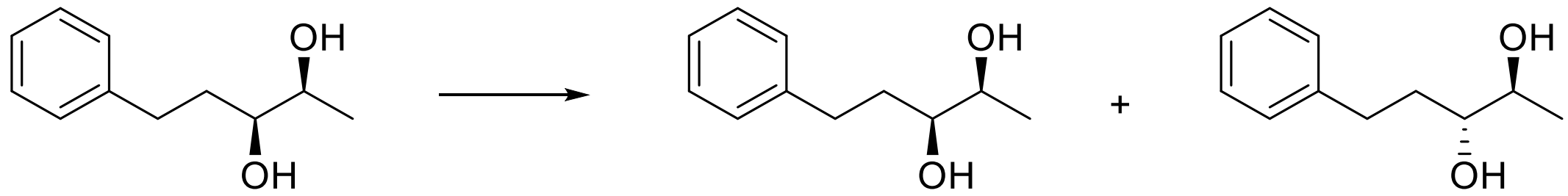

The compound was synthesised according to **General Procedure 7** using (*syn*)-5-phenylpentane-2,3-diol (18.0 mg, 0.10 mmol), separated with DCM then 5% acetone in hexane, giving 5-phenylpentane-2,3-diol (15.6 mg, 87%, mixture of *syn*:*anti* = 77:23) as a colourless solid.

Discernible peaks are denoted *a* (*anti*) and *s* (*syn*) respectively.

^{1}H NMR (500 MHz, $CDCl_3$) δ 7.31 – 7.27 (m, 2H), 7.23 – 7.17 (m, 3H), 3.83 – 3.77 (m, $0.23H^a$), 3.67 – 3.59 (m, 1H), 3.40 – 3.33 (m, $0.77H^s$), 1.90 (br. s, 2H), 1.86 – 1.70 (m, 2H), 1.89 (d, *J* = 6.3 Hz, $2.31H^s$), 1.15 (d, J = 6.4 Hz, $0.69H^a$) ppm.

Chiral SFC Analysis: *syn*: CHIRALPAK IA (CO_2:MeOH, 96:4, 2.5 mL min^{-1}, 40 °C, 204 nm) t_R = 9.3 (major), 9.72 (minor) min, 74% *ee*.

Chiral SFC Analysis: *anti* : CHIRALPAK IJ (CO_2:MeOH, 95:5, 2.5 mL min^{-1}, 40 °C, 204 nm) t_R = 5.1 (major), 5.3 (minor) min, 63% *ee*.

Enriched 5-Phenylpentane-2,3-diol (4h) starting from *anti*

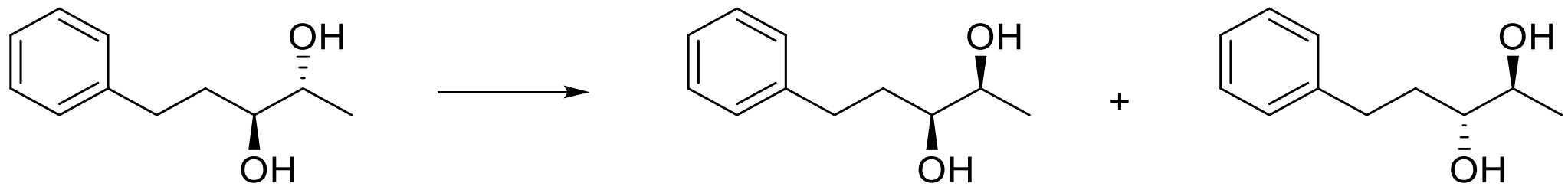

The compound was synthesised according to **General Procedure 7** using (*anti*)-5-phenylpentane-2,3-diol (18.0 mg, 0.10 mmol), separated with DCM then 10% acetone in hexane, giving enriched (*syn*)-5-phenylpentane-2,3-diol (7.1 mg, 39%) as a colourless oil, enriched (*anti*)-5-phenylpentane-2,3-diol (1.6 mg, 9%) as a colourless solid and 5-phenylpentane-2,3-diol (8.5 mg, 47%, mixture of *syn*:*anti* = 36:64).

Enriched (*syn*)-5-Phenylpentane-2,3-diol (*syn*-4h)

^{1}H NMR (700 MHz, $CDCl_3$) δ 7.31 – 7.27 (m, 2H), 7.23 – 7.17 (m, 3H), 3.66 – 3.60 (m, 1H), 3.39 – 3.34 (m, 1H), 2.90 – 2.83 (m, 1H), 2.74 – 2.67 (m, 1H), 1.92 (br. s, 2H), 1.84 – 1.78 (m, 1H), 1.78 – 1.70 (m, 1H), 1.19 (d, *J* = 6.3 Hz, 3H) ppm.

^{13}C NMR (176 MHz, $CDCl_3$) δ 142.0, 128.61, 128.57, 126.1, 75.6, 71.1, 35.3, 32.1, 19.7 ppm.

$[\alpha]_D^{25}$ = -21.4 (*c* 0.28, EtOH).

Chiral SFC Analysis: CHIRALPAK IC (CO_2:MeOH, 96:4, 2.5 mL min^{-1}, 40 °C, 205 nm) t_R = 10.5 (minor), 11.3 (major) min, 89% *ee*.

Enriched (*anti*)-5-Phenylpentane-2,3-diol (*anti*-4h)

^{1}H NMR (700 MHz, $CDCl_3$) δ 7.31 – 7.27 (m, 2H), 7.23 – 7.17 (m, 3H), 3.82 – 3.77 (m, 1H), 3.66 – 3.60 (m, 1H), 2.91 – 2.84 (m, 1H), 2.76 – 2.64 (m, 1H), 1.76 – 1.71 (m, 2H), 1.60 (br. s, 2H), 1.16 (d, *J* = 6.4 Hz, 3H) ppm.

^{13}C NMR (176 MHz, $CDCl_3$) δ 142.0, 128.60, 126.1, 74.3, 70.6, 33.5, 32.4, 17.0 ppm.

$[\alpha]_D^{25}$ = 0.0 (*c* 0.06, EtOH). (No optical rotation was observed)

Chiral SFC Analysis: CHIRALPAK IK (CO_2:MeOH, 93:7, 2.5 mL min^{-1}, 40 °C, 210 nm) t_R = 5.8 (minor), 6.1 (major) min, 87% *ee*.

Enriched 1-Phenylbutane-2,3-diol (4i) starting from *anti*

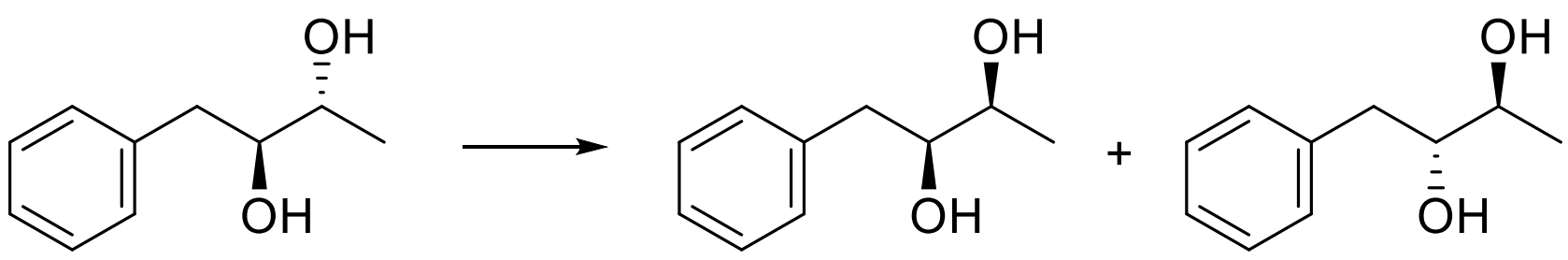

The compound was synthesised according to **General Procedure 7** using (*anti*)-1-phenylbutane-2,3-diol (16.6 mg, 0.10 mmol), separated with DCM then 15% acetone in hexane, giving 1-phenylbutane-2,3-diol (11.5 mg, 69%, mixture of *syn*:*anti* = 82:18) as a colourless oil.

Discernible peaks are denoted *a* (*anti*) and *s* (*syn*) respectively.

^{1}H NMR (700 MHz, $CDCl_3$) δ 7.35 – 7.31 (m, 2H), 7.26 – 7.23 (m, 3H), 3.90 – 3.86 (m, 0.18H^a), 3.85 – 3.81 (m, 0.18H^a), 3.72 – 3.67 (m, 0.82H^s), 3.63 – 3.58 (m, 0.82H^s), 1.99 (br. s, 2H), 1.28 – 1.25 (m, 3H) ppm.

Chiral SFC Analysis: *syn*: CHIRALPAK IE (CO_2:MeOH, 95:5, 2.5 mL min^{-1}, 40 °C, 225 nm) t_R = 8.9 (minor), 10.4 (major) min, 85% *ee*.

Chiral SFC Analysis: *anti* : CHIRALPAK IE (CO_2:MeOH, 95:5, 2.5 mL min^{-1}, 40 °C, 225 nm) t_R = 9.7 (major), 12.5 (minor) min, 87% *ee*.

Enriched 1-Phenylheptane-3,4-diol (4j) starting from *anti*

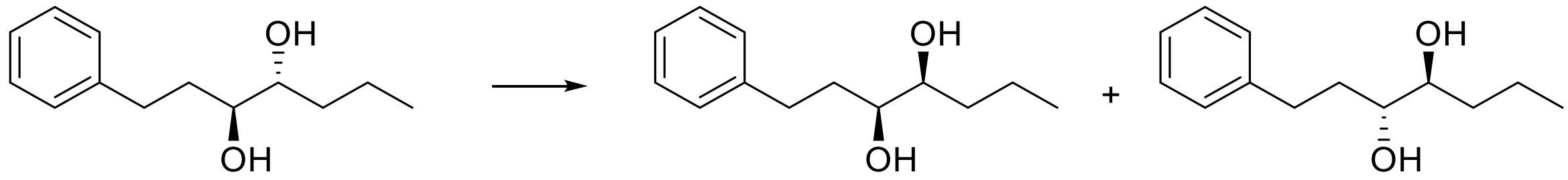

The compound was synthesised according to **General Procedure 7** using (*anti*)-1-phenylheptane-3,4-diol (20.8 mg, 0.10 mmol), separated with DCM then 20% acetone in hexane, giving 1-phenylheptane-3,4-diol (18.4 mg, 88%, mixture of *syn*:*anti* = 42:58) as a colourless solid.

Discernible peaks are denoted *a* (*anti*) and *s* (*syn*) respectively.

^{1}H NMR (400 MHz, $CDCl_3$) δ 7.32 – 7.26 (m, 2H), 7.24 – 7.16 (m, 3H), 3.65 – 3.59 (m, 1.15H^a), 3.50 – 3.39 (m, 0.85H^s), 2.93 – 2.80 (m, 1H), 2.76 – 2.63 (m, 1H), 2.16 – 2.10 (m, 0.4H^s), 2.01 – 1.89 (m, 1H), 1.89 – 1.72 (m, 2.6H), 1.56 – 1.30 (m, 4H), 0.93 (t, *J* = 7.2 Hz, 3H) ppm.

Chiral SFC Analysis: *syn*: CHIRALPAK IA (CO_2:MeOH, 96:4, 2.5 mL min^{-1}, 40 °C, 204 nm) t_R = 9.5 (major), 10.0 (minor) min, 91% *ee*.

Chiral SFC Analysis: *anti* : CHIRALPAK IJ (CO_2:MeOH, 97:3, 2.5 mL min^{-1}, 40 °C, 204 nm) t_R = 8.6 (major), 9.2 (minor) min, 9% *ee*.

Enriched 1,1-Diphenylbutane-2,3-diol (4k) starting from *anti*

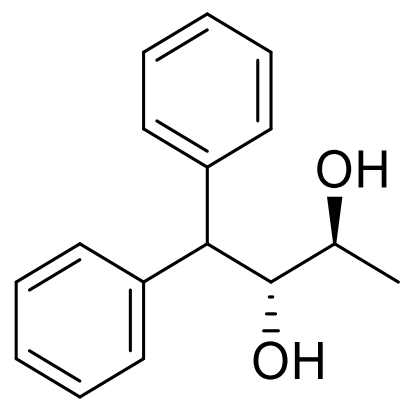

The compound was synthesised according to **General Procedure 7** using (*anti*)-1-phenylbutane-2,3-diol (24.2 mg, 0.10 mmol), separated with DCM then 15% acetone in hexane, giving Enriched (*anti*)-1,1-Diphenylbutane-2,3-diol (13.5 mg, 56%) as a colourless solid.

Enriched (*anti*)-1,1-Diphenylbutane-2,3-diol (*anti*-4k)

^{1}H NMR (700 MHz, $CDCl_3$) δ 7.42 – 7.38 (m, 2H), 7.35 – 7.27 (m, 6H), 7.24 – 7.17 (m, 2H), 4.48 (dt, *J* = 9.5, 3.5 Hz, 1H), 3.96 (d, *J* = 9.5 Hz, 1H), 3.75 – 3.64 (m, 1H), 1.93 – 1.85 (m, 2H), 1.20 (d, *J* = 6.4 Hz, 3H) ppm.

^{13}C NMR (176 MHz, $CDCl_3$) δ 141.5, 141.4, 129.05, 128.99, 128.7, 128.3, 127.1, 127.0, 76.7, 68.4, 54.9, 16.5 ppm.

$[\alpha]_D^{25}$ = 1.3 (*c* 0.54, $CHCl_3$).

Chiral SFC Analysis: CHIRALPAK IJ (CO_2:MeOH, 90:10, 2.5 mL min^{-1}, 40 °C, 217 nm) t_R = 12.6 (major), 14.1 (minor) min, 66% *ee*.

Enriched 4,5-Dihydroxyhexyl benzoate (4l) starting from *syn*

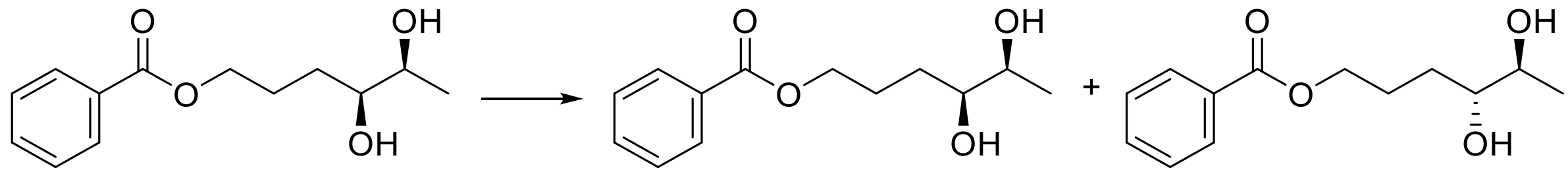

The compound was synthesised according to **General Procedure 7** using (*syn*)-4,5-dihydroxyhexyl benzoate (23.8 mg, 0.10 mmol), separated with DCM then 15% acetone in hexane, giving 4,5-dihydroxyhexyl benzoate (22.7 mg, 95%, mixture of *syn*:*anti* = 83:17) as a colourless solid.

Discernible peaks are denoted *a* (*anti*) and *s* (*syn*) respectively.

^{1}H NMR (500 MHz, $CDCl_3$) δ 8.06 – 8.01 (m, 2H), 7.58 – 7.53 (m, 1H), 7.47 – 7.41 (m, 2H), 4.37 (t, *J* = 6.6 Hz, 2H),), 3.86 – 3.79 (m, 0.17H^a), 3.71 – 3.58 (m, 1H), 3.46 – 3.37 (m, 0.83H^s), 2.39 (br. d, *J* = 4.9 Hz, 1H), 2.14 (br. d, *J* = 4.5 Hz, 1H), 2.06 – 1.96 (m, 1H), 1.94 – 1.81 (m, 1H), 1.71 – 1.63 (m, 1H), 1.60 – 1.50 (m, 1H), 1.21 (d, *J* = 6.3 Hz, 2.49H^s), 1.18 (d, *J* = 6.4 Hz, 0.51H^a) ppm.

Chiral SFC Analysis: *syn*: CHIRALPAK IA (CO_2:MeOH, 94:6, 2.5 mL min^{-1}, 40 °C, 225 nm) t_R = 10.8 (minor), 11.3 (major) min, 23% *ee*.

Chiral SFC Analysis: *anti* : CHIRALPAK IA (CO_2:MeOH, 94:6, 2.5 mL min^{-1}, 40 °C, 225 nm) t_R = 12.5 (minor), 13.0 (major) min, 57% *ee*.

Enriched 4,5-Dihydroxyhexyl benzoate (4l) starting from *anti*

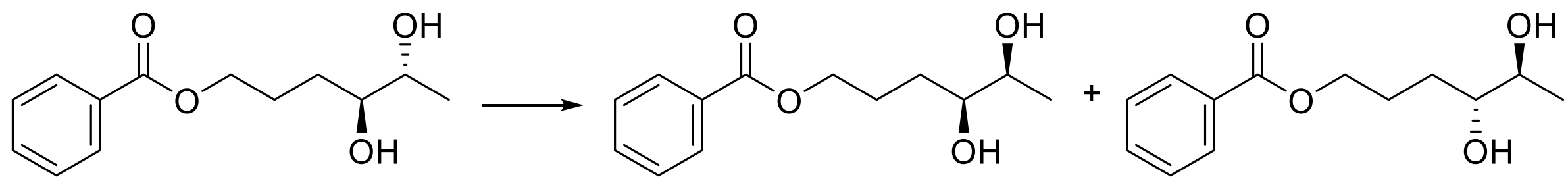

The compound was synthesised according to **General Procedure 7** using (*anti*)-4,5-dihydroxyhexyl benzoate (23.8 mg, 0.10 mmol), separated with DCM then 15% acetone in hexane, giving 4,5-dihydroxyhexyl benzoate (18.4 mg, 77%, mixture of *syn*:*anti* = 74:26) as a colourless solid.

Discernible peaks are denoted *a* (*anti*) and *s* (*syn*) respectively.

^{1}H NMR (500 MHz, $CDCl_3$) δ 8.06 – 8.00 (m, 2H), 7.59 – 7.53 (m, 1H), 7.47 – 7.41 (m, 2H), 4.37 (t, *J* = 6.6 Hz, 2H),), 3.86 – 3.79 (m, 0.26H^a), 3.71 – 3.58 (m, 1H), 3.46 – 3.37 (m, 0.74H^s), 2.12 – 1.80 (m, 4H), 1.72 – 1.50 (m, 2H), 1.21 (d, *J* = 6.3 Hz, 2.22H^s), 1.17 (d, *J* = 6.4 Hz, 0.78H^a) ppm.

Chiral SFC Analysis: *syn*: CHIRALPAK IA (CO_2:MeOH, 94:6, 2.5 mL min^{-1}, 40 °C, 225 nm) t_R = 10.8 (minor), 11.3 (major) min, 92% *ee*.

Chiral SFC Analysis: *anti* : CHIRALPAK IA (CO_2:MeOH, 94:6, 2.5 mL min^{-1}, 40 °C, 225 nm) t_R = 12.5 (minor), 13.0 (major) min, 77% *ee*.

Enriched 4,5-Dihydroxyhexyl diphenylphosphinate (4m) starting from *syn*

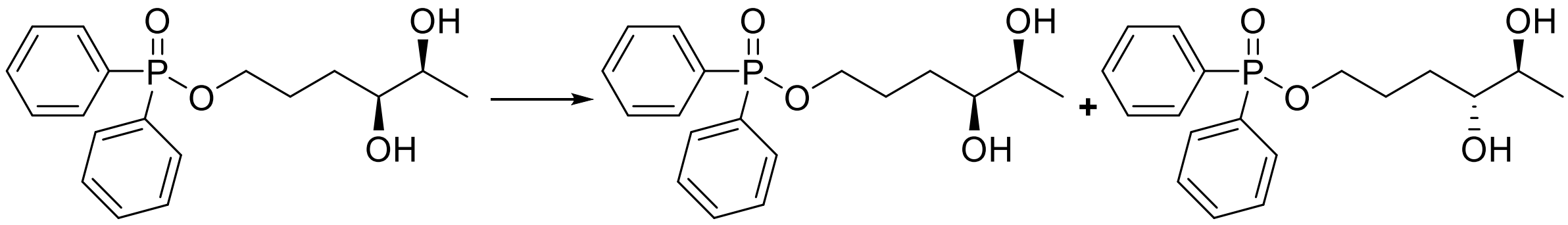

The compound was synthesised according to **General Procedure 7** using (*syn*)-4,5-dihydroxyhexyl diphenylphosphinate (33.4 mg, 0.10 mmol), separated with DCM then 50 – 80% acetone in hexane, giving 4,5-dihydroxyhexyl diphenylphosphinate (25.4 mg, 76%, mixture of *syn*:*anti* = 72:28) as a colourless oil.

Discernible peaks are denoted *a* (*anti*) and *s* (*syn*) respectively.

^{1}H NMR (500 MHz, $CDCl_3$) δ 7.80 – 7.75 (m, 4H), 7.55 – 7.49 (m, 2H), 7.48 – 7.41 (m, 4H), 4.16 – 4.02 (m, 2H), 3.79 – 3.72 (m, 0.28H^a), 3.62 – 3.55 (m, 1H), 3.38 – 3.32 (m, 0.72H^s), 2.83 (br. s, 2H), 2.00 – 1.78 (m, 2H), 1.69 – 1.50 (m, 2H), 1.16 (d, *J* = 6.3 Hz, 2.14H^s), 1.16 (d, *J* = 6.5 Hz, 0.84H^a), ppm.

Chiral HPLC Analysis: *syn*: CHIRALPAK SC (hexane:*i*PrOH, 85:15, 1.25 mL min^{-1}, 40 °C, 224 nm) t_R = 23.1 (major), 25.9 (minor) min, 82% *ee*.

Chiral HPLC Analysis: *anti* : CHIRALPAK SC (hexane:*i*PrOH, 85:15, 1.25 mL min^{-1}, 40 °C, 224 nm) t_R = 21.7 (minor), 24.6 (major) min, 72% *ee*.

Enriched 4,5-Dihydroxyhexyl diphenylphosphinate (4m) starting from *anti*

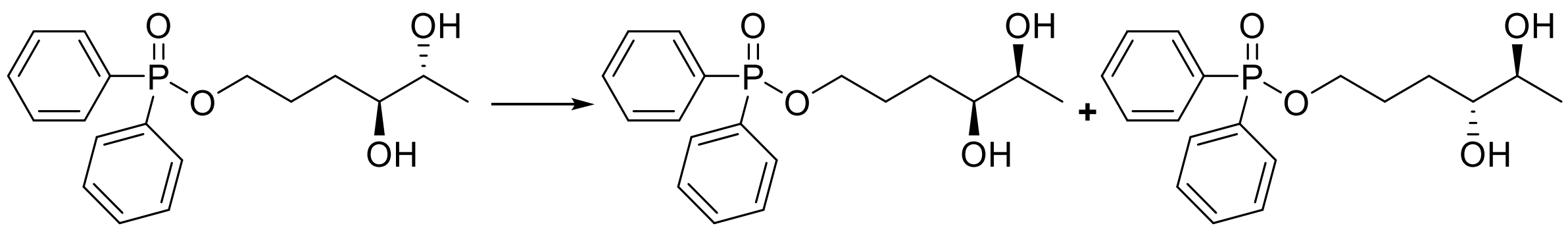

The compound was synthesised according to **General Procedure 7** using (*anti*)-4,5-dihydroxyhexyl diphenylphosphinate (33.4 mg, 0.10 mmol), separated with DCM then 50 – 80% acetone in hexane, giving 4,5-dihydroxyhexyl diphenylphosphinate (23.5 mg, 70%, mixture of *syn*:*anti* = 70:30) as a colourless oil.

Discernible peaks are denoted *a* (*anti*) and *s* (*syn*) respectively.

^{1}H NMR (500 MHz, $CDCl_3$) δ 7.83 – 7.75 (m, 4H), 7.55 – 7.49 (m, 2H), 7.48 – 7.41 (m, 4H), 4.16 – 4.02 (m, 2H), 3.79 – 3.72 (m, 0.3H^a), 3.62 – 3.55 (m, 1H), 3.38 – 3.32 (m, 0.7H^s), 3.27 – 2.49 (br. m, 2H), 2.00 – 1.78 (m, 2H), 1.69 – 1.50 (m, 2H), 1.16 (d, *J* = 6.3 Hz, 2.1H^s), 1.16 (d, *J* = 6.5 Hz, 0.9H^a), ppm.

Chiral HPLC Analysis: *syn*: CHIRALPAK SC (hexane:*i*PrOH, 85:15, 1.25 mL min^{-1}, 40 °C, 224 nm) t_R = 23.1 (major), 25.9 (minor) min, 93% *ee*.

Chiral HPLC Analysis: *anti* : CHIRALPAK SC (hexane:*i*PrOH, 85:15, 1.25 mL min^{-1}, 40 °C, 224 nm) t_R = 21.7 (minor), 24.6 (major) min, 77% *ee*.

Scheme 9 - Commencing from a racemic mixture of diastereomers.

Enriched-1-(naphthalen-1-yl)pentane-2,4-diol (3c) from a mixture of diastereomers

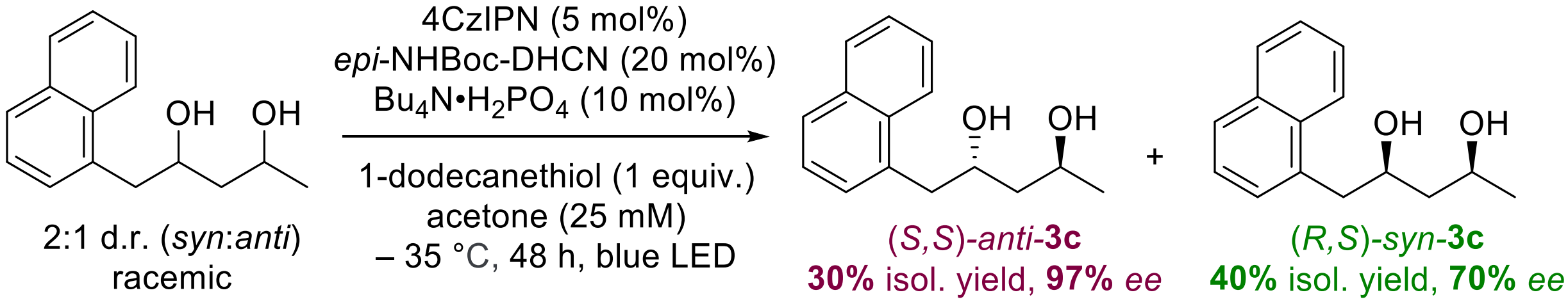

The compound was synthesised according to **General Procedure 7** using 1-(naphthalen-1-yl)pentane-2,4-diol (*syn*:*anti* = 2:1) (23.0 mg, 0.10 mmol), separated with DCM then 10% acetone in hexane, giving (2*R*,4*S*)-1-(naphthalen-1-yl)pentane-2,4-diol (9.1 mg, 40%, 70% *ee*) as a colourless oil and (*S*,*S*)-1-(naphthalen-1-yl)pentane-2,4-diol (6.8 mg, 30%, 97% *ee*) as a colourless solid. The characterization is described above.

Enriched 5-Phenylpentane-2,3-diol (4h) starting from a mixture of diastereomers

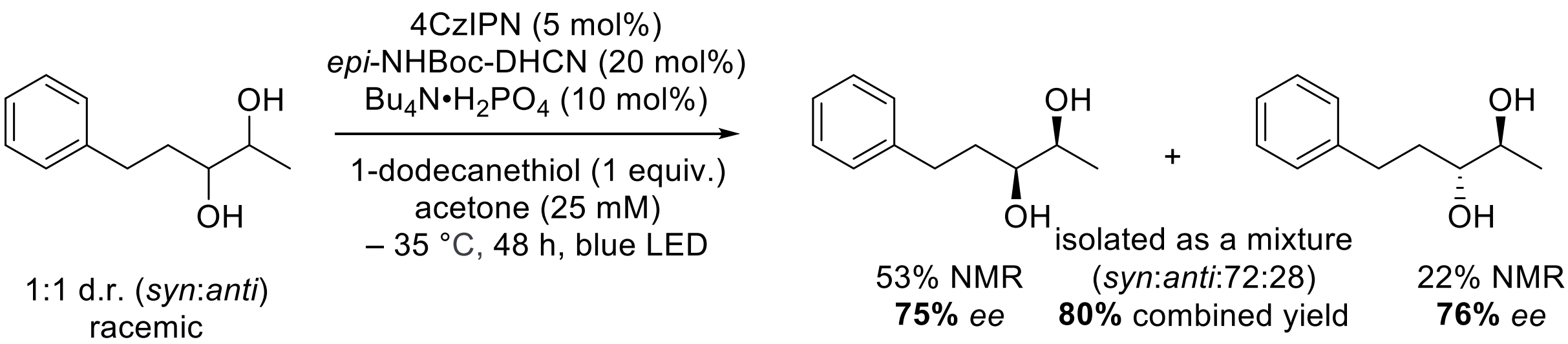

The compound was synthesised according to **General Procedure 7** using (*syn*)-5-phenylpentane-2,3-diol (*syn*:*anti* = 1:1) (18.0 mg, 0.10 mmol), separated with DCM then 12% acetone in hexane, giving 5-phenylpentane-2,3-diol (14.4 mg, 80%, mixture of *syn*:*anti* = 72:28; *syn* 75% *ee*; *anti* 76% *ee*) as a colourless oil. The characterization is described above.

Catalyst degradation study

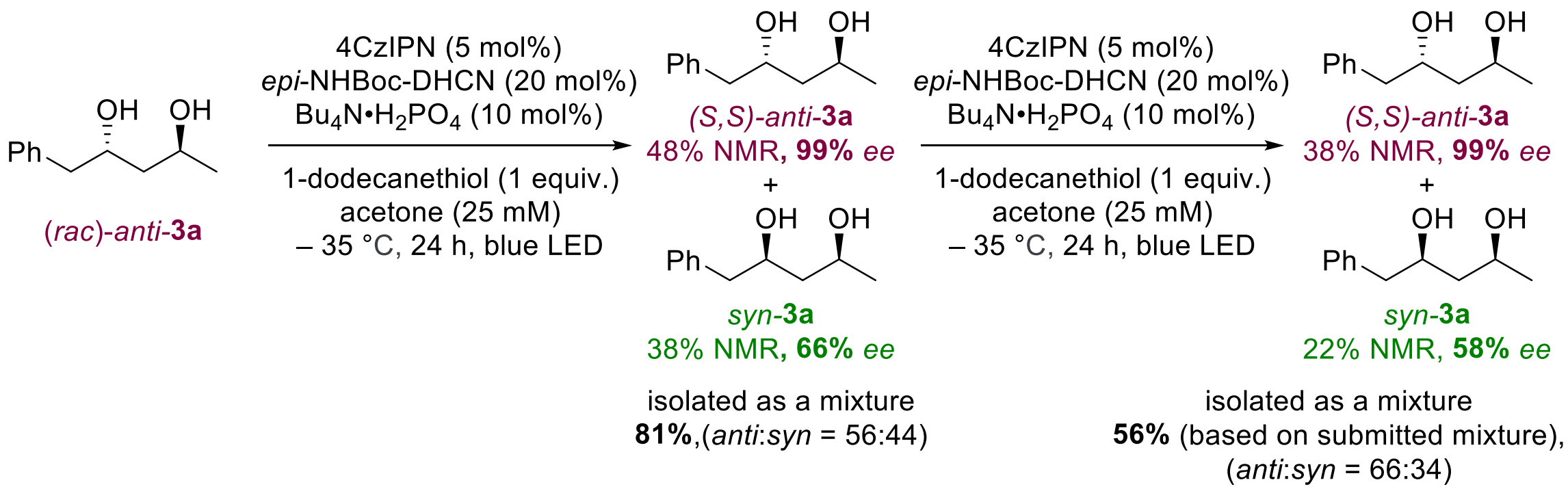

First reaction: The reaction was performed according to **General Procedure 7** for 24 h using (*anti*)-1-phenylpentane-2,4-diol (18.0 mg, 0.10 mmol), separated with DCM then 15% acetone in hexane, giving 1-phenylpentane-2,4-diol as a mixture of diastereomers (14.6 mg, 81%, mixture of *anti*:*syn* = 56:44; *anti* 99% *ee*; *syn* 66% *ee*) as a colourless oil.

This mixture was dissolved in 1 mL acetone and 50 µL solution were set aside for ee analysis. The remaining 950 µL were transferred to a reaction vial and the reaction was repeated as above using a catalyst/reagent loading as if 0.1 mmol diol would have been used.

Second reaction: The reaction was performed according to **General Procedure 7** for 24 h using the reaction mixture from above (13.9 mg, 0.077 mmol), separated with DCM then 12% acetone in hexane, giving 1-phenylpentane-2,4-diol as a mixture of diastereomers (7.7 mg, 56%, mixture of *anti*:*syn* = 66:34; *anti* 99% *ee*; *syn* 56% *ee*) as a colourless oil.

Interpretation: The minor difference in stereoisomer distribution between the two individual 24 h reactions above and the 48 h reaction described in the previous section indicates that catalyst degradation throughout the reaction plays a minor role in the overall outcome and a close-to-true kinetic equilibrium is probably reached after 24 h. In the second reaction (ie fresh catalyst on the presumed equilibrium mixture of isomers) it seems plausible that some decomposition pathway is occurring, which potentially involves the syn isomer since the amount of this was relatively reduced in the reisolated material.

Deuterium incorporation studies

Reacting (2*S*,4*S*)-pentane-2,4-diol with D_2O:

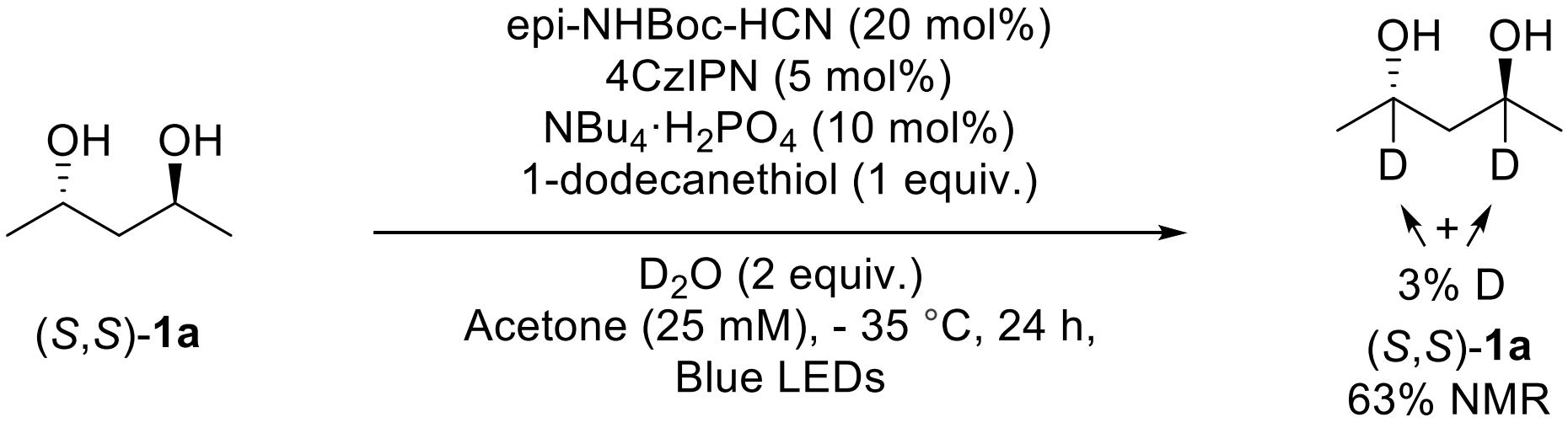

Prepared according to **General Procedure 7** using diol substrate (*S*,*S*)-**1a** (10.4 mg, 0.1 mmol) adding D_2O (3.5 µl, 0.2 mmol) and reacting for 24 hours. Purification by silica gel chromatography (elution with 10% Acetone in Hexane) to give (2*S*,4*S*)-pentane-2,4-diol (5.2 mg, 0.050 mmol, 50%, 3% (α to OH) D incorporation) as a colourless solid. (*syn*)-Pentane-2,4-diol was observed by crude NMR but could not be isolated.

Interpretation: (*S*,*S*)-**1a** is processed slowly to *meso*-**1a** towards the kinetic equilibrium. During this process, (*S*,*S*)-**1a** is partially reformed leading to traces of deuterium incorporation.

Reacting (2*R*,4*R*)-pentane-2,4-diol with D_2O:

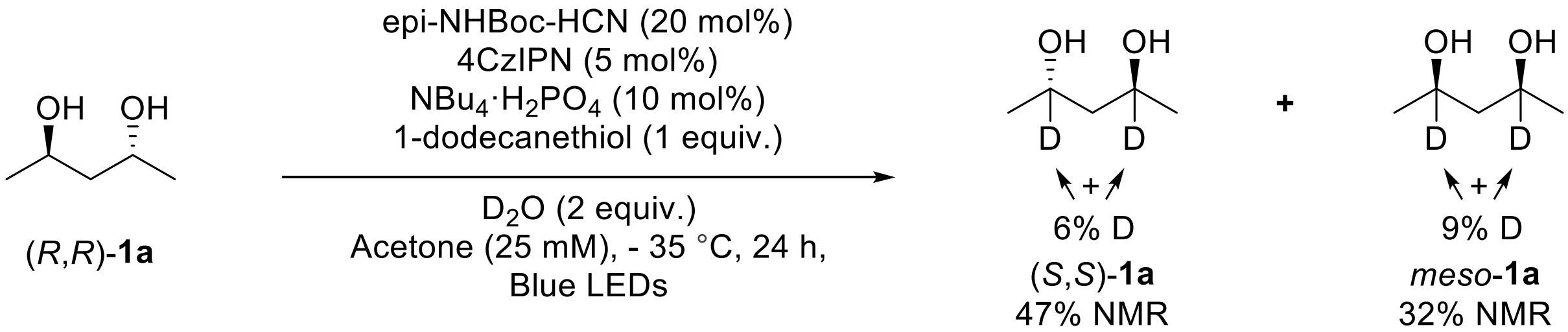

Prepared according to **General Procedure 7** using diol substrate (*R*,*R*)-**1a** (10.4 mg, 0.1 mmol) adding D_2O (3.5 µl, 0.2 mmol) and reacting for 24 hours. Purification by silica gel chromatography (elution with 10% Acetone in Hexane) to give (*syn*)-pentane-2,4-diol (0.9 mg, 0.009 mmol, 9%, 9% (α to OH) D incorporation) as a colourless oil and (2*S*,4*S*)-pentane-2,4-diol (4.7 mg, 0.045 mmol, 45%, 6% (α to OH) D incorporation) as a colourless oil.

Interpretation: (*R*,*R*)-**1a** is processed quickly to *meso*-**1a** which then forms the (*S*,*S*)-**1a**. This can react further to reform the *meso*-**1a** in a kinetic equilibrium with potential deuterium incorporation in every hydrogen donation step. This gives a benchmark for what D incorporation might be expected on a reactive system with 2 eq. added D2O in the reaction mixture.

Reacting (5*S*,7*S*)-undecane-5,7-diol ((*S*,*S*)-1d) with D_2O:

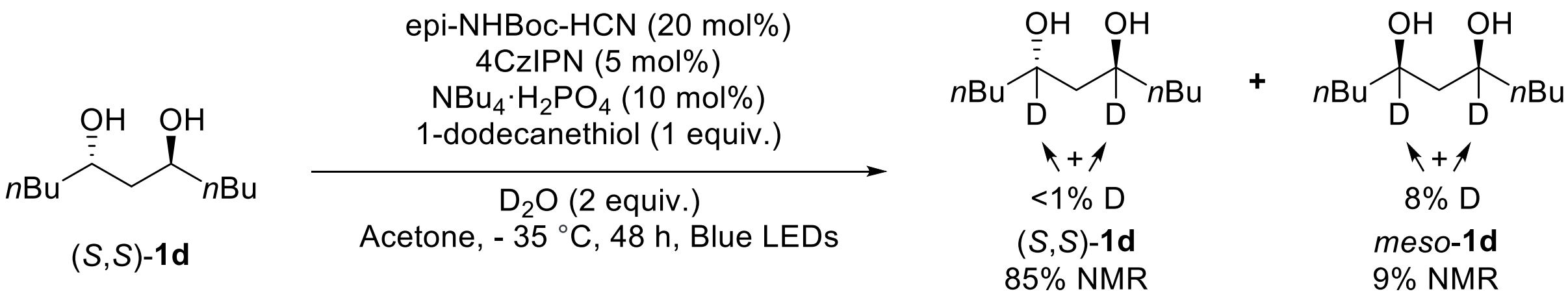

Prepared according to **General Procedure 7** using diol substrate (*S*,*S*)-**1d** (18.8 mg, 0.1 mmol) adding D_2O (3.5 μl, 0.2 mmol). Purification by silica gel chromatography (elution with 5% acetone in Pet. Ether) to give (*syn*)-undecane-5,7-diol (2 mg, 0.010 mmol, 10%, 8% (α to OH) D incorporation) as a colourless oil and (5*S*,7*S*)-undecane- 5,7-diol (15 mg, 0.081 mmol, 81%, <1% (α to OH) D incorporation) as a white solid.

Interpretation: The (*S*,*S*)-enantiomer is processed to form the *meso*-diol with some deuterium incorporation. The remaining (*S*,*S*)-enantiomer has very little deuterium incorporation due to the low conversion - only a small amount of meso is formed (9% yield). The recovered (S,S) had incorporated only a trace amount of D (0-1%) while the formed meso contained 8% D. This outcome is consistent with a very slow abstraction (ie low conversion) and reformation at the same time the meso is also formed. Because the conversion is low the amount of D incorporated into the S,S is much lower than for the meso.

Absolute stereochemistry assignments

Five of the 1,3-diols synthesized using our standard conditions (*epi*-NHBoc-DHCN) are known compounds, with recorded $[\alpha]_D$ values and HPLC chromatograms. Comparison of the optical rotation sign and chromatography elution sequence for all previously reported *anti*-1,3 diols consistently indicate that the major enantiomer formed by our enantioselective epimerisation with *epi*-NHBoc-DHCN is *S*,*S* configured and the major enantiomer formed in the enantioselective oxidation with *epi*-NHBoc-DHCN is *R* configured. This gives confidence that our assignment by analogy of the S,S-configuration for all novel *anti*-1,3-diols is likely to be reliable. The absolute configuration of all novel non-symmetric *syn*-1,3-diols bearing one methyl group is likely also correct based on the comparisons shown below. For novel non-symmetric *syn*-1,3-diols bearing substituents other than methyl (**3i**, **3j**, **3k**) the analogy is not as clear and the assignment tentative.

Some of the compounds have very low magnitudes of $[\alpha]_D$. In two cases, comparison of reported $[\alpha]_D$ with the literature produced a conflicting assignment based on what was expected based on the sign of the $[\alpha]_D$ value alone, although the magnitude was low. In these two cases careful analysis of order of elution by HPLC or SFC on identical columns suggested that these very small $[\alpha]_D$ values could not be relied on and indeed the expected stereochemical assignment was obtained. One case was (*S,S*)-**3d** (details below) where analysis of the order of retention on the same column and same solvent system as reported in the literate confirmed the assignment of stereochemistry. The second case was (R,R)-anti-**3a** obtained via Noyori asymmetric hydrogenation (details below). Again, the $[\alpha]_D$ had very low magnitude but opposite sign to that expected. Comparison of SFC elution order confirmed the expected assignment and again suggested the small $[\alpha]_D$ gave a misleading outcome.

Authentic samples of enantioenriched compounds **3a** and **4l** were independently synthesised using Noyori asymmetric hydrogenation and Sharpless asymmetric dihydroxylation respectively. Based on the established selectivity models for these reactions we further reinforce the stereochemical assignments made. Details are given in the table below and the following section.

**Structure**	**This study** $[\alpha]_D$ or HPLC (from *epi*-NHBoc-DHCN)	**Literature** $[\alpha]_D$ or HPLC or **authentic sample**
1b 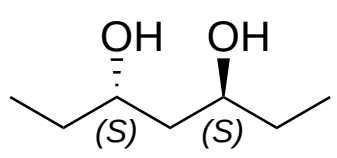	*S*,*S*-enantiomer proposed<br>**+ 9.7** (c = 0.37, T = 25 °C, $CHCl_3$)	*S*,*S*-enantiomer reported<br>**+ 20.6** (c = 1.12; T = 25 °C, $CHCl_3$)[52]<br>**+ 25** (c= 1; T = 25, $CHCl_3$)[64]
1d 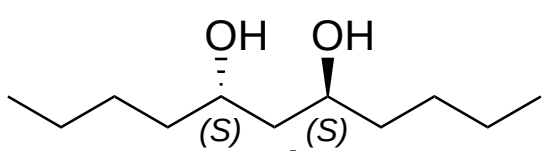	*S*,*S*-enantiomer proposed<br>**+ 25.4** (c = 0.19, T = 25 °C, $CHCl_3$)	*R*,*R*-enantiomer reported<br>**- 6.0** (c = 1; T = 23 °C, CH_2Cl_2)[54]
1k 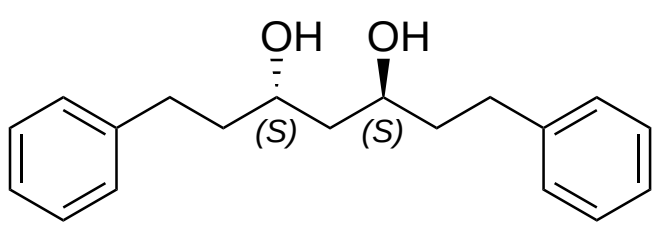	*S*,*S*-enantiomer proposed<br>**- 5.2** (c = 0.85, T = 25 °C, $CHCl_3$)	*S*,*S*-enantiomer reported<br>**– 3.6** (c = 1; T = 25 °C, $CHCl_3$)[65]<br>**– 7.0** (c = 1.0; T = 20 °C, $CHCl_3$)[66]
2c 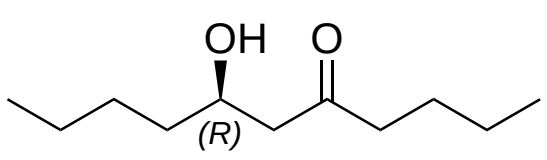	*R*-enantiomer proposed<br>**- 21.3** (c = 0.58, T = 25 °C, $CHCl_3$)	*R*-enantiomer reported<br>**- 34.1** (c = 1; T = 20 °C, $CHCl_3$)[6]<br>**– 42.0** (c = 1; T = 23 °C, CH_2Cl_2)[54]

2e<br>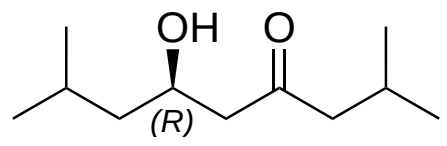 	*R*-enantiomer proposed<br>**- 20.5** (c = 0.37, T = 25 °C, $CHCl_3$)	*R*-enantiomer reported<br>**- 26.9** (c = 1.0; T = 20 °C, $CHCl_3$)[6]
3a<br>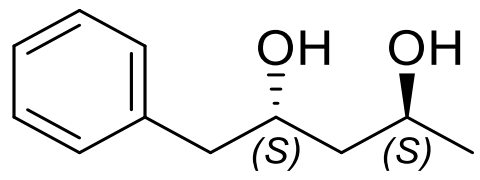 	*S*,*S*-enantiomer proposed<br>**- 1.9** (c = 0.37, T = 25 °C, $CHCl_3$)<br><br>Chiral SFC Analysis: CHIRALPAK IK (CO_2:MeOH, 96:04, 2.5 ml min^{-1}, 40 °C, 205 nm) **t_R = 11.1 (major) minutes**, 99% *ee*.<br><br>We attribute the disagreement with the specific rotation of the Noyori hydrogenation sequence product to its small magnitude leading to measurement errors.<br><br>Comparing the elution sequence on the same HPLC column leads however to the expected *S*,*S*-enantiomer deduction.	*R*,*R*-enantiomer from Noyori hydrogenation with Ru-(R)-BINAPCl$_2$<br>**- 2.4** (c = 1.00, T = 25 °C, $CHCl_3$)<br><br>Chiral SFC Analysis: CHIRALPAK IK (CO_2:MeOH, 96:04, 2.5 ml min^{-1}, 40 °C, 205 nm) **t_R = 11.0 (minor), 12.1 (major) minutes**, 91% *ee*.
3a<br>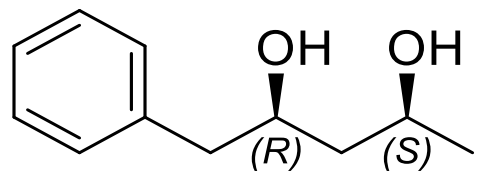 	*2R*,*4S*-enantiomer proposed<br>**+ 4.6** (c = 0.26, T = 25 °C, $CHCl_3$)<br><br>Chiral SFC Analysis: CHIRALPAK IK (CO_2:MeOH, 93:7, 2.5 mL min^{-1}, 40 °C, 205 nm) **t_R = 6.6 (minor), 8.0 (major) min**, 65% *ee*.	*R*,*S*-enantiomer from Noyori hydrogenation with Ru-(R)-BINAPCl$_2$<br>**+ 16.2** (c = 0.76, T = 25 °C, $CHCl_3$)<br><br>Chiral SFC Analysis: CHIRALPAK IK (CO_2:MeOH, 93:07, 2.5 ml min^{-1}, 40 °C, 207 nm) **t_R = 6.6 (minor), 7.8 (major) minutes**, 92% *ee*.
S,*S*-3d<br>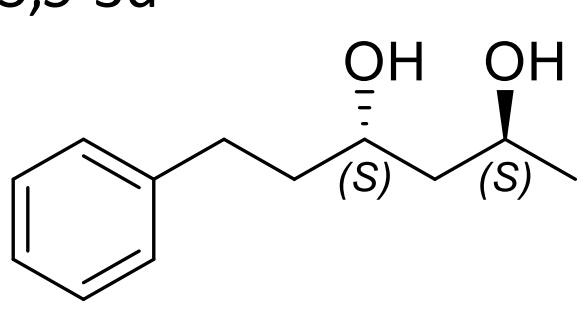 	*R*,*R*-enantiomer proposed from $[\alpha]_D$<br>**+ 2.4** (*c* = 0.33, T = 25 °C, $CHCl_3$)<br><br>*S*,*S*-enantiomer deduced from HPLC data:<br>CHIRALPAK AD-H (hexane: *i*PrOH, 90:10, 0.75 mL min^{-1}, 40 °C, 204 nm) **t_R = 9.9 (major), 10.3 (minor) min**, 99% *ee*.	*R*,*R*-enantiomer reported<br>**+ 2.7** (*c* = 0.6, $CHCl_3$)[18]<br><br>*R*,*R*-enantiomer reported HPLC data:<br>CHIRALPAK AD-H (hexane: *i*PrOH, 90:10, 0.75 mL min^{-1}, 214, 254 nm) **t_R = 10.0 (minor), 10.6 (major) min**, 88% *ee*.[18]

	We attribute the disagreement with the specific rotation in the literature to its small magnitude leading to measurement errors.<br><br>Comparing the elution sequence on the same HPLC column leads however to the expected *S*,*S*-enantiomer deduction.	
2*S*,4*R*-3d<br>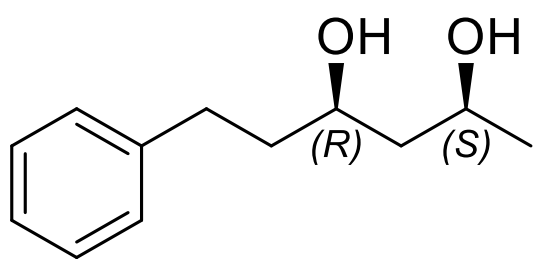	*2S*,4*R*-enantiomer proposed<br>**+ 5.6** (*c* = 0.16, T = 25 °C, $CHCl_3$)	2*S*,4*R*-enantiomer reported<br>**+ 22.4** (*c* = 1.0, $CHCl_3$)[19]<br>**+ 11.7** (*c* = 0.6, $CHCl_3$)[18]
S,*S*-4l<br>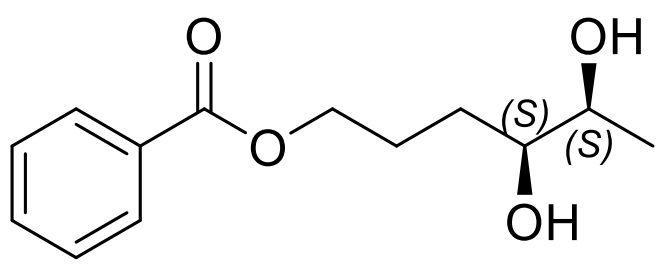	*S*,*S*-enantiomer proposed<br><br>Chiral SFC Analysis: CHIRALPAK IA (CO_2:MeOH, 94:6, 2.5 mL min^{-1}, 40 °C, 225 nm) **t$_R$ = 10.8 (minor), 11.3 (major) min**, 92% *ee*.	*S*,*S*-enantiomer from Sharpless dihydroxylation with AD-mix-α<br>Chiral SFC Analysis: CHIRALPAK IA (CO_2:MeOH, 94:6, 2.5 mL min^{-1}, 40 °C, 225 nm) **t$_R$ = 10.9 (minor), 11.3 (major) min**, 85% *ee*.
4*S*,5*R*-4l<br>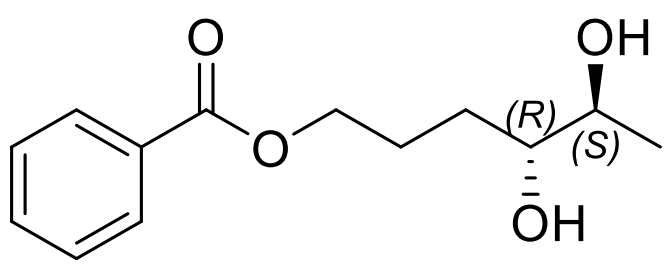	4*S*,5*R*-enantiomer proposed<br><br>Chiral SFC Analysis: CHIRALPAK IA (CO_2:MeOH, 94:6, 2.5 mL min^{-1}, 40 °C, 225 nm) **t$_R$ = 12.5 (minor), 13.0 (major) min**, 77% *ee*.	4*R*,5*S*-enantiomer from Sharpless dihydroxylation with AD-mix-α<br>Chiral SFC Analysis: CHIRALPAK IA (CO_2:MeOH, 94:6, 2.5 mL min^{-1}, 40 °C, 225 nm) **t$_R$ = 12.6 (major), 13.2 (minor) min**, 23% *ee*.

Additionally, the use of the pseudoenantiomeric catalysts (*epi*-NHBoc-DHCD) gives the opposite product—data of which corroborated by both chiral SFC analysis as well as sign comparison for optical rotation data.

Noyori Hydrogenation to Confirm Absolute Stereochemistry of 3a with Authentic Sample

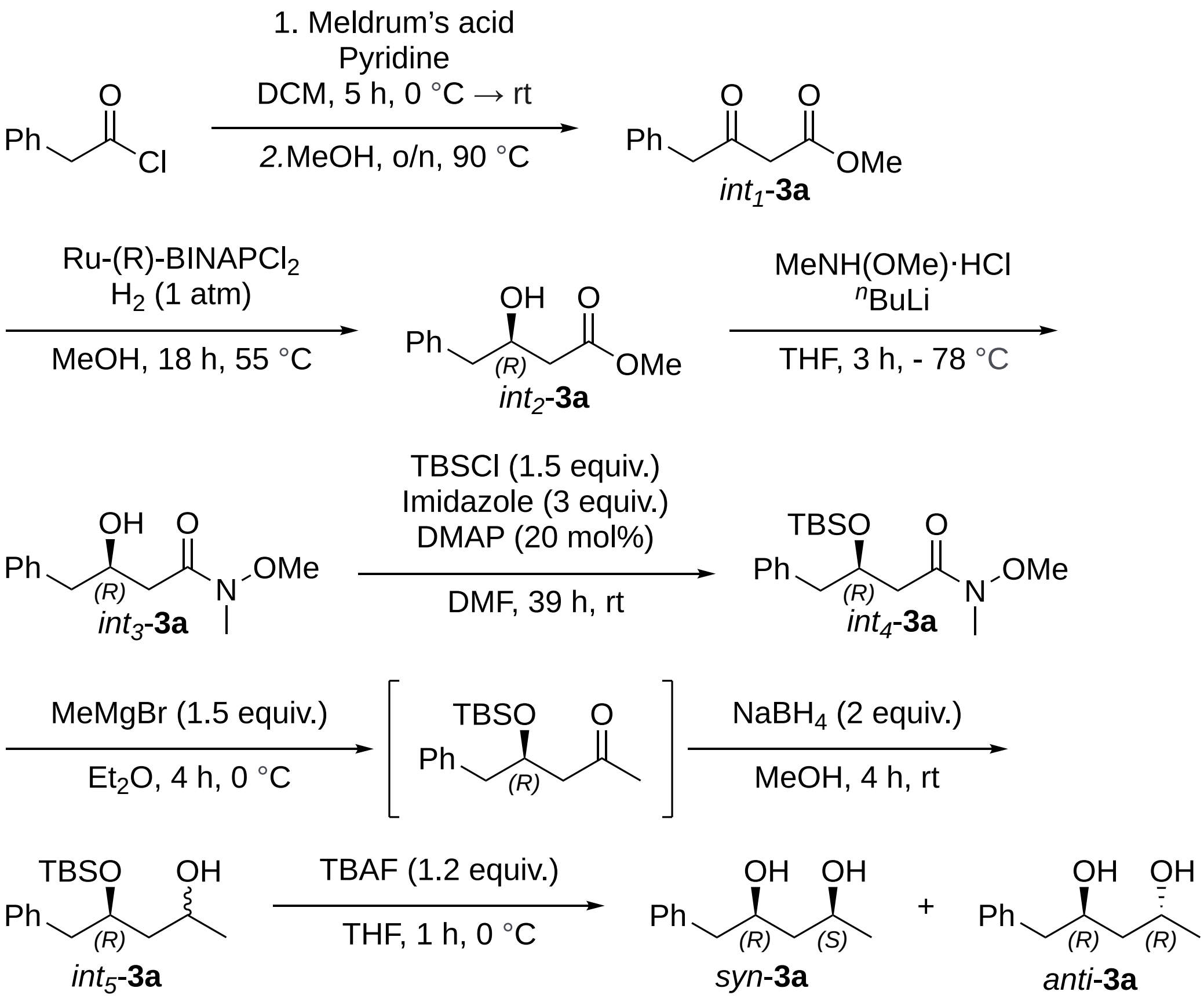

methyl 3-oxo-4-phenylbutanoate (*int₁*-3a)

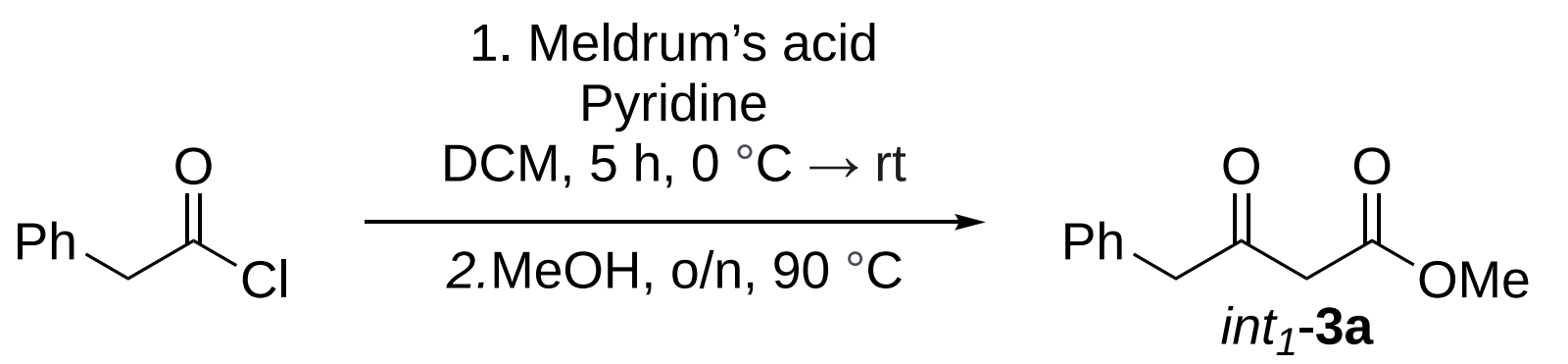

Preparation based on a literature procedure.[67] Pyridine (2 ml, 25 mmol, 2.5 equiv.) was added to a solution of Meldrum's acid (1.44 g, 10 mmol, 1 equiv.) in anhydrous DCM (29 ml). Phenyl acetyl chloride (1.3 ml, 10 mmol, 1 equiv.) in anhydrous DCM (1.5 ml) was added dropwise to the solution over an hour at 0 °C. After this the reaction mixture was stirred for 1 hour at 0 °C then for 1 hour at room temperature. The reaction mixture was washed with 1M HCl (2 × 30 ml) then the organic layer dried over $MgSO_4$ and the solvent removed *in vacuo*. The crude product was purified *via* silica gel chromatography (5 – 10% EtOAc in Hexane) to give methyl 3-oxo-4-phenylbutanoate (1.11 g, 5.8 mmol, 58%) as a pale orange oil.

^{1}H NMR (700 MHz, $CDCl_3$) δ 12.04 (s, 0.1H), 7.36 – 7.32 (m, 2H), 7.32 – 7.27 (m, 1H), 7.21 (d, *J* = 7.5 Hz, 2H), 4.94 (s, 0.1H), 3.82 (s, 1.8H), 3.71 (s, 2.6H), 3.70 (s, 0.4H), 3.51 (s, 0.2H), 3.46 (s, 1.7H) ppm.

^{13}C NMR (176 MHz, $CDCl_3$) δ 200.5, 167.6, 133.3, 129.4, 128.7, 127.5, 127.2, 90.0, 52.5, 50.2, 48.1, 41.5 ppm.

The data is in good agreement with the literature.[68]

methyl (*R*)-3-hydroxy-4-phenylbutanoate (*int₂*-3a)

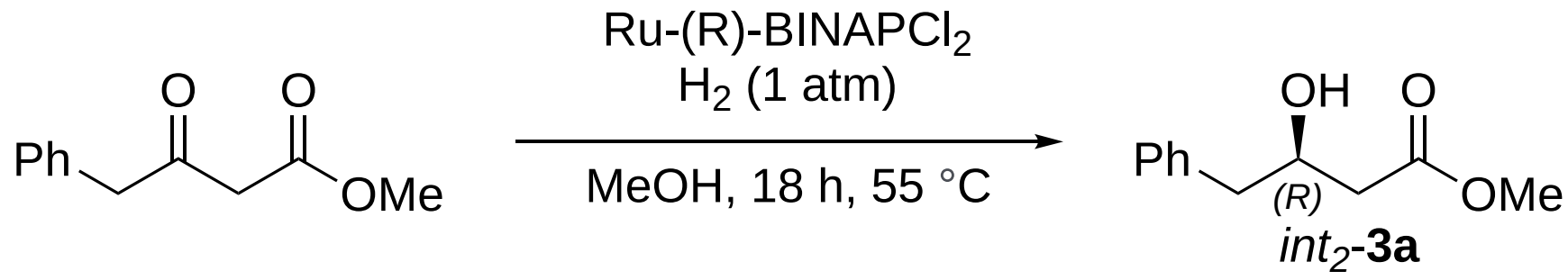

Preparation based on a literature procedure.[69] Ru-(R)-BINAPCl$_2$ (206 mg, 0.26 mmol, 2 mol%) and methyl 3-oxo-4-phenylbutanoate (2.51 g, 13 mmol) in degassed MeOH (26 ml) was purged with H_2 (1 atm). The reaction mixture was then heated for 18 hours at 55 °C before being cooled to room temperature and concentrated. The crude product was purified *via* silica gel chromatography (20% EtOAc in Hexane) to give methyl (*R*)-3-hydroxy-4-phenylbutanoate (902 mg, 4.69 mmol, 36%, 90% *ee*) as a colourless oil.

^{1}H NMR (700 MHz, $CDCl_3$) δ 7.31 (app t, *J* = 7.5 Hz, 2H), 7.26 – 7.21 (m, 3H), 4.29 – 4.25 (m, 1H), 3.70 (s, 3H), 2.87 (dd, *J* = 13.7, 7.2 Hz, 1H), 2.78 (dd, *J* = 13.6, 6.1 Hz, 1H), 2.53 (dd, *J* = 16.4, 3.4 Hz, 1H), 2.46 (dd, *J* = 16.4, 8.9 Hz, 1H) ppm.

^{13}C NMR (176 MHz, $CDCl_3$) δ 173.2, 137.8, 129.6, 128.7, 126.8, 69.2, 51.9, 43.1, 40.5 ppm.

$[\alpha]_D^{25.0}$ = - 9.0 (*c* 1.69, $CHCl_3$)

*Literature $[\alpha]_D$ values for int₂-**3a**: - 10.0 (c = 1; T = 25 ℃, $CHCl_3$)*[70]

*Absolute configuration of int₂-**3a** therefore assigned as R.*

Chiral SFC Analysis CHIRALPAK IC (CO_2:MeOH, 95:05, 2.5 ml min^{-1}, 40 °C, 210 nm) t_R = 4.3 (minor), 4.9 (major) minutes, 90% *ee*.

The data is in good agreement with the literature.[71]

(*R*)-3-hydroxy-*N*-methoxy-*N*-methyl-4-phenylbutanamide (*int₃*-3a)

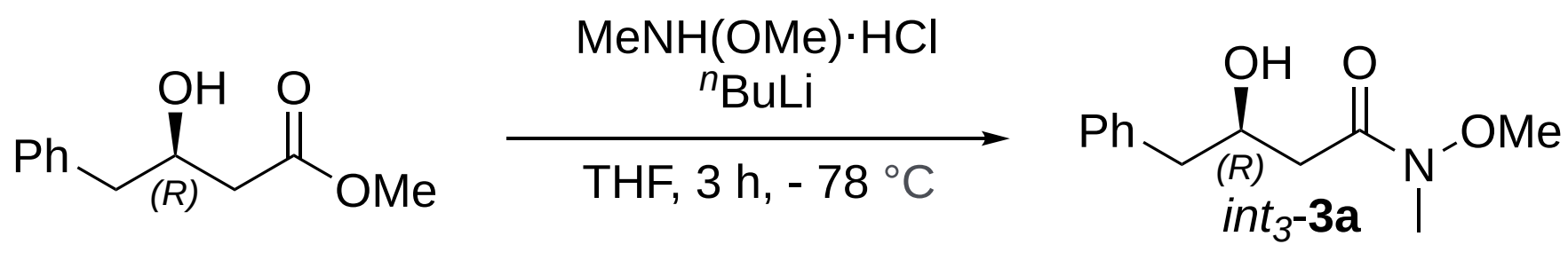

Preparation based on a literature procedure.[72] *N*,*O*-dimethylhydroxylamine hydrochloride (1.75 g, 18 mmol, 4 equiv.) in THF (65 ml) was cooled to – 78 °C and ^{n}BuLi (14.4 ml, 36 mmol, 2.5M in hexane, 8 equiv.) was added dropwise. The dry ice bath was removed and the mixture stirred for 15 minutes before being cooled back to – 78 °C where methyl (*R*)-3-hydroxy-4-phenylbutanoate (848 mg, 4.4 mmol, 1 equiv.) in THF (35 ml) was added dropwise. The reaction mixture was stirred for 3 hours at – 78 °C before being quenched with saturated NH_4Cl (30 ml). The aqueous layer was washed with EtOAc (2 × 25 ml) then the organic layers combined and dried over $MgSO_4$. The solvent was removed *in vacuo* and the crude product purified *via* silica gel chromatography to give (*R*)-3-hydroxy-*N*-methoxy-*N*-methyl-4-phenylbutanamide (883 mg, 4.0 mmol, 90%) as a colourless oil.

^{1}**H NMR** (700 MHz, $CDCl_3$) δ 7.30 (app t, *J* = 7.5 Hz, 2H), 7.25 – 7.21 (m, 3H), 4.31 – 4.26 (m, 1H), 3.61 (s, 3H), 3.31 (br s, 1H), 3.17 (s, 3H), 2.93 (dd, *J* = 13.6, 7.0 Hz, 1H), 2.77 (dd, *J* = 13.6, 6.5 Hz, 1H), 2.66 (d, *J* = 16.7 Hz, 1H), 2.49 (dd, *J* = 16.6, 9.2 Hz, 1H) ppm.

^{13}**C NMR** (176 MHz, $CDCl_3$) δ 173.7, 138.3, 129.5, 128.6, 126.6, 69.3, 61.3, 43.1, 37.5, 32.0 ppm.

The data is in good agreement with the literature.[73]

(R)-3-((tert-butyldimethylsilyl)oxy)-N-methoxy-N-methyl-4-phenylbutanamide (*int*$_4$-3a)

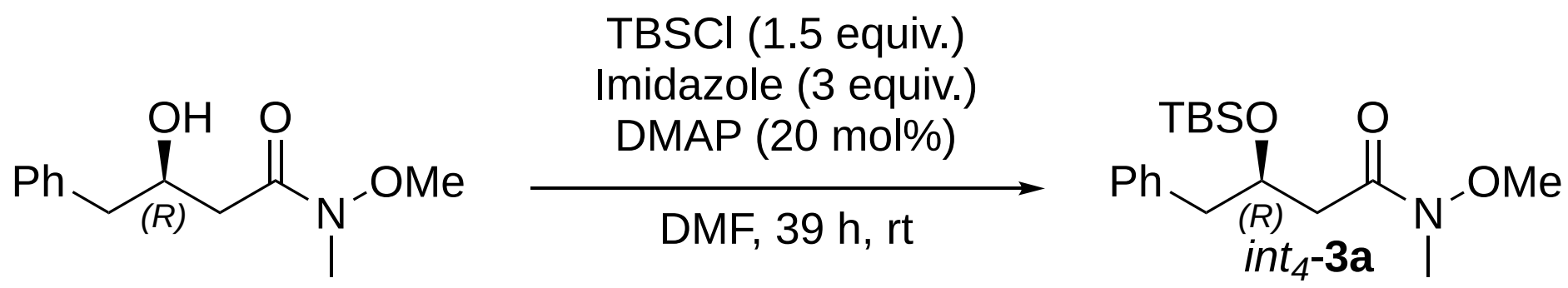

Preparation based on a literature procedure.[74] Weinreb amide int$_3$-**3a** (447 mg, 2.0 mmol, 1 equiv.) was dissolved in DMF (3 ml) then TBSCl (452 mg, 3.0 mmol, 1.5 equiv.), imidazole (408 mg, 6.0 mmol, 3 equiv.) and DMAP (24.4 mg, 0.2 mmol, 20 mol%) were added to the solution. The reaction mixture was stirred at room temperature for 39 hours then diluted with water (3 ml) and stirred for 1 hour. The product was extracted with DCM (4 × 5 ml) and the combined organics dried over $MgSO_4$. The solvent was removed *in vacuo* and the crude product purified via silica gel chromatography (5 – 10% EtOAc in Hexane) to give (R)-3-((tert-butyldimethylsilyl)oxy)-N-methoxy-N-methyl-4-phenylbutanamide (630 mg, 1.87 mmol, 93%) as a pale yellow oil.

^{1}**H NMR** (700 MHz, $CDCl_3$) δ 7.26 (app t, J = 7.4 Hz, 2H), 7.22 – 7.17 (m, 3H), 4.43 (p, J = 6.3 Hz, 1H), 3.62 (s, 3H), 3.15 (s, 3H), 3.17 (s, 3H), 2.84 (dd, J = 13.4, 5.8 Hz, 1H), 2.79 (dd, J = 13.4, 6.6 Hz, 1H), 2.71 – 2.63 (m, 1H), 2.43 (dd, J = 14.8, 5.0 Hz, 1H), 0.84 (s, 9H), - 0.04 (s, 3H), - 0.15 (s, 3H) ppm.

^{13}**C NMR** (176 MHz, $CDCl_3$) δ 172.5, 138.7, 130.0, 128.3, 126.3, 70.8, 61.4, 44.5, 39.4, 32.1, 26.0, 18.1, -4.8, -5.0 ppm.

HRMS [M+H]$^+$ m/z calc'd for $[C_{18}H_{32}NO_3Si]^+$ expect 295.2093 found 295.2088

(4*R*)-4-((*tert*-butyldimethylsilyl)oxy)-5-phenylpentan-2-ol (*int*$_5$-3a)

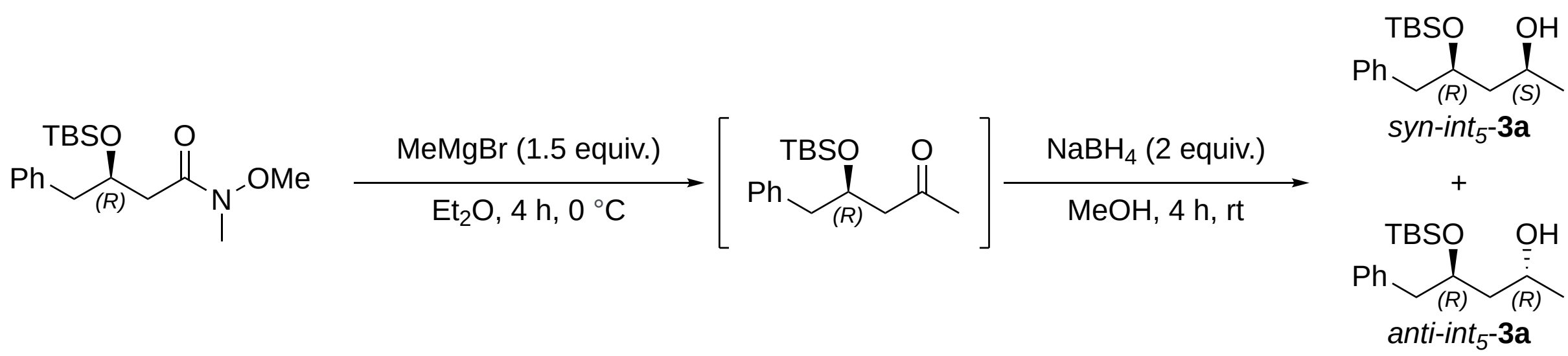

Preparation based on literature procedures.[74, 75] Weinreb amide *int*$_5$-**3a** (548 mg, 1.6 mmol, 1 equiv.) was dissolved in Et_2O (4.5 ml) at 0 °C and then methyl magnesium bromide (0.8 ml, 2.4 mmol, 3.0 M in Et_2O, 1.5 equiv.) was added slowly. The reaction mixture was then stirred at 0 °C for 4 hours before being quenched by addition of NH_4Cl (5 ml). The aqueous layer was extracted with Et_2O (2 × 10 ml) then the organic layers combined then dried over $MgSO_4$ and the solvent was removed *in vacuo*. The

crude product was carried forward and dissolved in MeOH (5 ml) at 0 °C then $NaBH_4$ (114 mg, 3.0 mmol, 2 equiv.) was added. The reaction mixture was stirred for 4 hours at 0 °C and then quenched by addition of NH_4Cl (5 ml). The aqueous layer was extracted with EtOAc (3 × 10 ml) then the organic layers combined then dried over $MgSO_4$. The solvent was removed *in vacuo* and the crude product was purified via silica gel chromatography (5% Acetone in Hexane) to give (4*R*)-4-((*tert*-butyldimethylsilyl)oxy)-5-phenylpentan-2-ol in a mixture of diastereomers (1.8:1 d.r. syn:anti, 416 mg, 1.41 mmol, 87%) as a pale yellow oil.

syn-Int$_5$-3a-major diastereomer (DR-1) and _syn-Int_$_5$-3a-minor diastereomer (DR-2)

^{1}H NMR (700 MHz, $CDCl_3$) δ 7.28 (app t, J = 7.4 Hz, 2H DR-1 & 2H DR-2), 7.23 – 7.19 (m, 1H DR-1 & 1H DR-2), 7.17 – 7.14 (m, 2H DR-1 & 2H DR-2), 4.30 – 4.24 (m, 1H DR-2), 4.21 – 4.17 (m, 1H DR-2), 4.13 – 4.07 (m, 1H DR-1), 3.93 – 3.87 (m, 1H DR-1), 2.96 – 2.88 (m, 1H DR-1 & 1H DR-2), 2.83 (dd, J = 13.6, 7.0 Hz, 1H DR-2), 2.70 (dd, J = 13.2, 8.2 Hz, 1H DR-1), 1.62 (ddd, *J* = 14.1, 10.2, 3.8 Hz, 1H DR-2), 1.54 – 1.49 (m, 2H DR-1 & 1H DR-2), 1.15 (d, J = 6.2 Hz, 3H DR-2), 1.10 (d, J = 7.1 Hz, 3H DR-1), 0.92 (s, 9H DR-1), 0.88 (s, 9H DR-2), 0.14 (s, 3H DR-1), 0.07 (s, 3H DR-1), 0.05 (s, 3H DR-2), - 0.14 (s, 3H DR-2) ppm.

^{13}C NMR (176 MHz, $CDCl_3$) δ 138.8 (DR-2), 138.3 (DR-1), 129.7 (DR-1 & DR-2), 128.5 (DR-1 & DR-2), 126.5 (DR-1), 126.5 (DR-2), 74.6 (DR-1), 73.5 (DR-2), 67.2 (DR-1), 64.6 (DR-2), 45.3 (DR-1), 44.6 (DR-1), 43.0 (DR-2), 26.0 (DR-1 & DR-2), 24.0 (DR-2), 23.7 (DR-1), 18.1 (DR-1 & DR-2), - 4.03 (DR-1), - 4.6 (DR-1), - 4.8 (DR-2), - 5.0 (DR-2) ppm.

HRMS [M+H]$^+$ m/z calc'd for $[C_{17}H_{31}O_2Si]^+$ expect 338.2151 found 338.2157

(2*R*,4*S*)-1-phenylpentane-2,4-diol (*syn*-3a) and (2*R*,4*R*)-1-phenylpentane-2,4-diol (*anti*-3a)

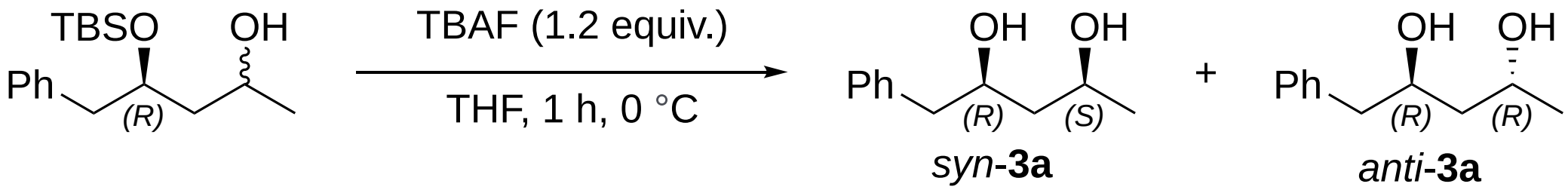

Preparation based on a literature procedure.[76] TBAF (1.6 ml, 1.6 mmol, 1.2 equiv.) was added to a solution of *int*$_5$-**3a** (398 mg, 1.4 mmol, 1 equiv.) in THF (3 ml) at 0 °C. The reaction mixture was stirred for 1 hour at 0 °C then diluted with EtOAc before being washed successively with sat. $NaHCO_3$, water and brine. The organic layer was dried over $MgSO_4$ and the solvent removed *in vacuo*. The crude product was purified via silica gel chromatography (10% Acetone in Hexane) to give (2*R*,4*S*)-1-phenylpentane-2,4-diol (88 mg, 0.49 mmol, 36%, 92% *ee*) as a white solid and (2*R*,4*R*)-1-phenylpentane-2,4-diol (40 mg, 0.22 mmol, 16%, 91 *ee*) as a white solid with mixed fractions (24 mg, 0.14 mmol, 10%).

(2*R*,4*S*)-1-phenylpentane-2,4-diol ((*S*,*S*)-*syn*-3a)

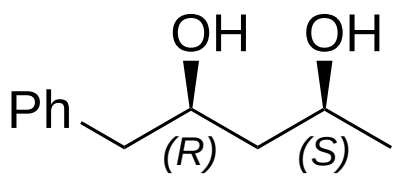

syn-**3a**

^{1}H NMR (700 MHz, $CDCl_3$) δ 7.31 (app t, *J* = 7.5 Hz, 2H), 7.24 (app t, *J* = 7.4 Hz, 1H), 7.21, (d *J* = 7.6 Hz, 2H), 4.09 – 4.04 (m, 1H), 4.04 – 3.99 (m, 1H), 3.08 (s, 2H), 2.76 (d, *J* = 6.5 Hz, 2H), 1.64 (br d, *J* = 14.5 Hz, 1H), 1.55 (ddd, *J* = 14.5, 10.0, 10.0 Hz, 1H), 1.18 (d, *J* = 6.2 Hz, 3H) ppm.

^{13}C NMR (176 MHz, $CDCl_3$) δ 138.0, 129.6, 128.7, 126.7, 74.0, 69.0, 44.8, 44.1, 24.1 ppm.

$[\alpha]_D^{25.0}$ = +16.2 (*c* 0.76, $CHCl_3$)

Chiral SFC Analysis CHIRALPAK IK (CO_2:MeOH, 93:07, 2.5 ml min^{-1}, 40 °C, 207 nm) t_R = 6.6 (minor), 7.8 (major) minutes, 92% *ee*.

(2*R*,4*R*)-1-phenylpentane-2,4-diol ((*R*,*R*)-*anti*-3a)

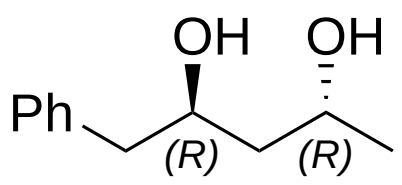

anti-**3a**

^{1}H NMR (500 MHz, $CDCl_3$) δ 7.31 (app t, *J* = 7.5 Hz, 2H), 7.25 – 7.20 (m, 3H), 4.20 – 4.14 (m, 2H), 2.82 – 2.75 (m, 2H), 2.45 (s, 2H), 1.65 (app t, *J* = 5.7 Hz, 2H), 1.22 (d, *J* = 6.3 Hz, 3H) ppm.

^{13}C NMR (126 MHz, $CDCl_3$) δ 138.4, 129.5, 128.7, 126.7, 70.2, 65.5, 44.1, 43.7, 23.6 ppm.

$[\alpha]_D^{25.0}$ = - 2.41 (*c* 1.00, $CHCl_3$)

Chiral SFC Analysis CHIRALPAK IK (CO_2:MeOH, 96:04, 2.5 ml min^{-1}, 40 °C, 205 nm) t_R = 11.0 (minor), 12.1 (major) minutes, 91% *ee*.

Sharpless dihydroxylation to Confirm Absolute Stereochemistry of **4l** with Authentic Sample

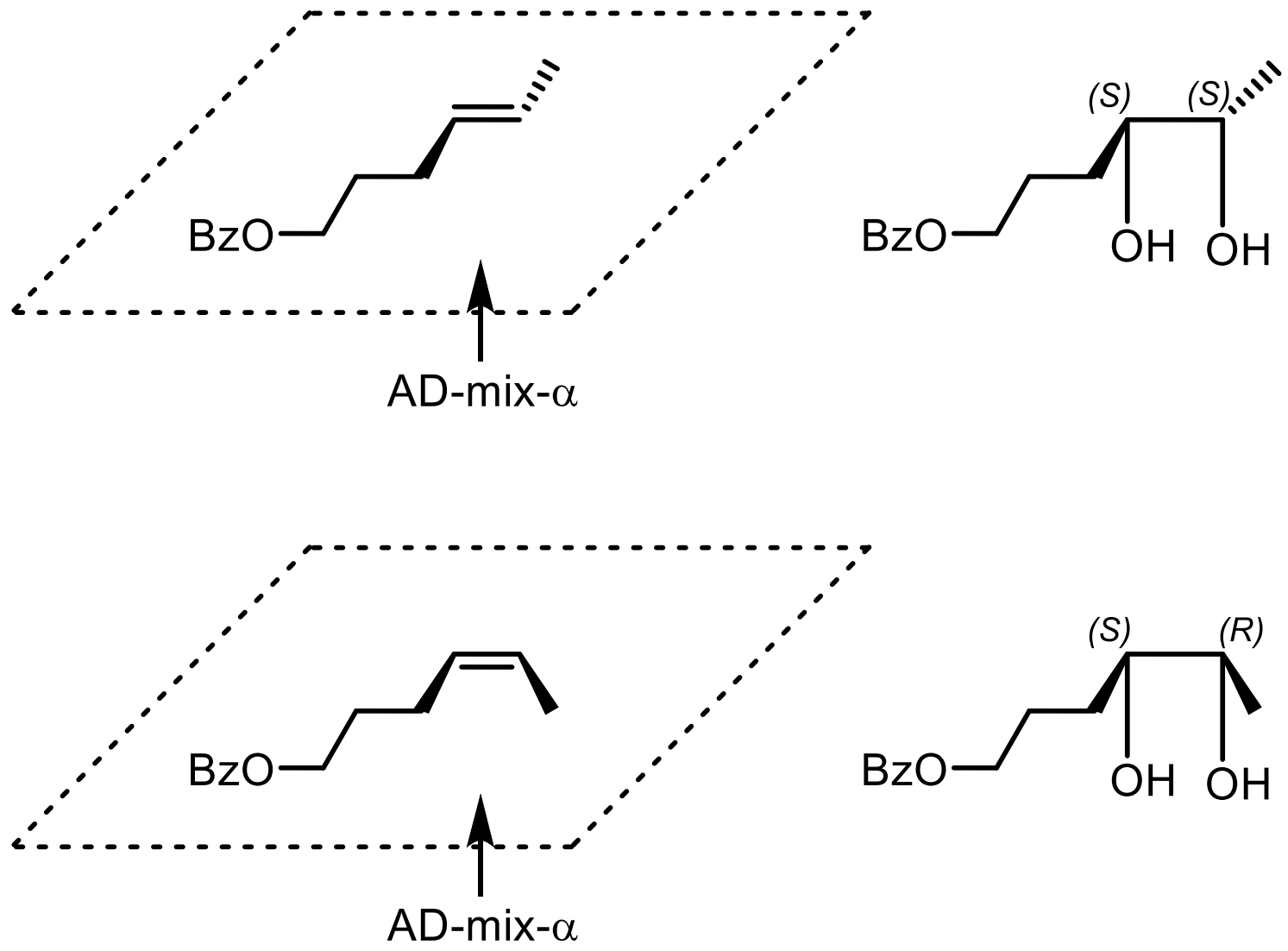

(*S*,*S*)-4,5-Dihydroxyhexyl benzoate (*S*,*S*-4l)

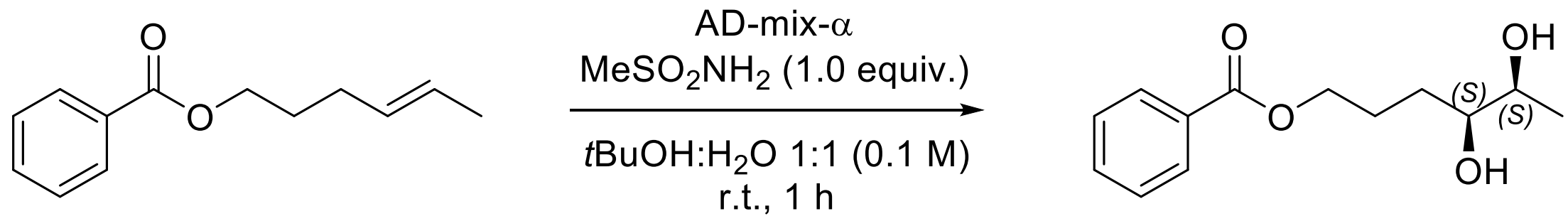

This procedure was adapted from literature.[77] To a vial was added AD-mix-α (280 mg), methanesulfonamide (19.0 mg, 0.2 mmol, 1.0 equiv.), *t*BuOH (1 mL) and water (1 mL). The mixture was stirred until clear and (*E*)-hex-4-en-1-yl benzoate (40.9 mg, 0.2 mmol, 1.0 equiv.) was added. The mixture was stirred for 1 h upon which it was cooled to 0 °C and Na_2SO_3 (3 g) was added. Then, it was warmed to r.t. and stirred for 1 h. The mixture was extracted with EtOAc (3 x 10 mL) and the combined organic layers washed with brine and dried over $MgSO_4$. The solvent was evaporated under reduced pressure and the crude product purified by flash column chromatography silica gel (20% acetone in hexane) to give the desired product (41.3 mg, 87%, 85% *ee*) as a colourless oil.

^{1}H NMR (500 MHz, $CDCl_3$) δ 8.07 – 8.00 (m, 2H), 7.59 – 7.52 (m, 1H), 7.48 – 7.40 (m, 2H), 4.37 (t, *J* = 6.6 Hz, 2H), 3.67 – 3.59 (m, 1H), 3.47 – 3.37 (m, 1H), 2.43 (br. d, *J* = 4.6 Hz, 1H), 2.19 (br. d, *J* = 3.9 Hz, 1H), 2.06 – 1.96 (m, 1H), 1.94 – 1.83 (m, 1H), 1.72 – 1.63 (m, 1H), 1.60 – 1.50 (m, 1H), 1.21 (d, *J* = 6.3 Hz, 3H) ppm.

^{13}C NMR (126 MHz, $CDCl_3$) δ 166.9, 133.1, 130.4, 129.7, 128.5, 75.8, 71.0, 65.0, 30.0, 25.2, 19.8 ppm.

Chiral SFC Analysis: CHIRALPAK IA (CO_2:MeOH, 94:6, 2.5 mL min^{-1}, 40 °C, 225 nm) t_R = 10.9 (minor), 11.3 (major) min, 85% *ee*.

$[\alpha]_D^{25}$ = -10.4 (*c* 0.92, $CHCl_3$).

(4*R*,5*S*)-4,5-Dihydroxyhexyl benzoate (4*R*,5*S*-4l)

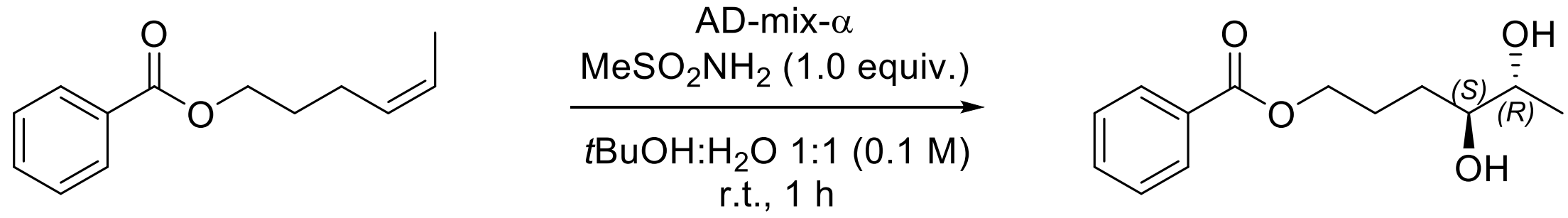

This procedure was adapted from literature.[77] To a vial was added AD-mix-α (280 mg), methanesulfonamide (19.0 mg, 0.2 mmol, 1.0 equiv.), *t*BuOH (1 mL) and water (1 mL). The mixture was stirred until clear and (*Z*)-hex-4-en-1-yl benzoate (40.9 mg, 0.2 mmol, 1.0 equiv.) was added. The mixture was stirred for 1 h upon which it was cooled to 0 °C and Na_2SO_3 (3 g) was added. Then, it was warmed to r.t. and stirred for 1 h. The mixture was extracted with EtOAc (3 x 10 mL) and the combined organic layers washed with brine and dried over $MgSO_4$. The solvent was evaporated under reduced pressure and the crude product purified by flash column chromatography silica gel (20% acetone in hexane) to give the desired product (43.2 mg, 91%, 23% *ee*) as a colourless solid.

^{1}H NMR (500 MHz, $CDCl_3$) δ 8.06 – 8.01 (m, 2H), 7.58 – 7.53 (m, 1H), 7.47 – 7.40 (m, 2H), 4.43 – 4.33 (m, 2H), 3.86 – 3.80 (m, 1H), 3.72 – 3.65 (m, 1H), 2.10 – 1.98 (m, 2H), 1.90 – 1.77 (m, 2H), 1.65 – 1.50 (m, 3H), 1.17 (d, *J* = 6.4 Hz, 3H) ppm.

^{13}C NMR (126 MHz, $CDCl_3$) δ 166.8, 133.1, 130.5, 129.7, 128.5, 74.7, 70.6, 65.0, 28.3, 25.6, 17.1 ppm.

Chiral SFC Analysis: CHIRALPAK IA (CO_2:MeOH, 94:6, 2.5 mL min^{-1}, 40 °C, 225 nm) t_R = 12.6 (major), 13.2 (minor) min, 23% *ee*.

$[\alpha]_D^{25}$ = -8.4 (*c* 0.49, $CHCl_3$).

References

(1) Lahdenperä, A. S. K.; Dhankhar, J.; Davies, D. J.; Lam, N. Y. S.; Bacos, P. D.; de la Vega-Hernandez, K.; Phipps, R. J. A chiral hydrogen atom abstraction catalyst for the enantioselective epimerization of meso-diols. *Science* **2024**, *386*, 42–49.

(2) Lam, N. Y. S.; Dhankhar, J.; Lahdenpera, A. S. K.; Phipps, R. J. Catalytic Enantioselective Hydrogen Atom Abstraction Enables the Asymmetric Oxidation of Meso Diols. *J. Am. Chem. Soc.* **2024**, *146*, 33302–33308.

(3) Luo, J.; Zhang, J. Donor–Acceptor Fluorophores for Visible-Light-Promoted Organic Synthesis: Photoredox/Ni Dual Catalytic C(sp3)–C(sp2) Cross-Coupling. *ACS Catalysis* **2016**, *6*, 873–877.

(4) Baylon, C.; Heck, M. P.; Mioskowski, C. Bis Ring Closing Olefin Metathesis for the Synthesis of Unsaturated Polycyclic Ethers. O-Membered Ring Cyclization in Favor of C-Membered Ring Cyclization. *J. Org. Chem.* **1999**, *64*, 3354–3360.

(5) Grieco, L. M.; Halliday, G. A.; Junk, C. P.; Lustig, S. R.; Marshall, W. J.; Petrov, V. A. Reactions of 1,1,2,2-tetrafluoroethyl-N,N-dimethylamine with linear and cyclic 1,3-diketones. *Journal of Fluorine Chemistry* **2011**, *132*, 1198–1206.

(6) Zörb, A.; Brückner, R. Conversion of Conjugated Enones into Enantiomerically Pure -Hydroxy Ketones or 1,3-Diols – Samarium(II) Bromide Reductions of Protected ,-Dihydroxy Ketones. *Eur. J. Org. Chem.* **2010**, *2010*, 4785–4801.

(7) Yang, G. K.; Bergman, R. G. Stereochemistry of metallacycle formation in the double alkylation of bis(triphenylphosphine)nitrogen(1+) bis(.eta.5-cyclopentadienyl)-di-.mu.-carbonyldicobaltate with .alpha.,.gamma.-diiodoalkanes. *J. Am. Chem. Soc.* **1983**, *105*, 6045–6052.

(8) Poole, W. G.; Peron, F.; Fox, S. J.; Wells, N.; Skylaris, C.-K.; Essex, J. W.; Kuprov, I.; Linclau, B. Conformational Analysis of 1,3-Difluorinated Alkanes. *J. Org. Chem.* **2024**, *89*, 8789–8803.

(9) Zhang, Y.-Q.; Poppel, C.; Panfilova, A.; Bohle, F.; Grimme, S.; Gansäuer, A. SN2 Reactions at Tertiary Carbon Centers in Epoxides. *Angew. Chem. Int. Ed.* **2017**, *56*, 9719–9722.

(10) Guijarro, A.; Yus, M. Polychlorinated materials as a source of polyanionic synthons. *Tetrahedron* **1996**, *52*, 1797–1810.

(11) Berthel, S. J.; Chen, L.; Corbett, W. L.; Feng, L.; Haynes, N.-E.; Kester, R. F.; So, S.-S.; Tilley, J. W. Azaindole glucokinase activators. WO2011073117A1, 2010.

(12) Hong, H.; Zheng, C.; Guo, L. SYNTHESIS METHOD FOR L-CYCLIC ALKYL AMINO ACID AND PHARMACEUTICAL COMPOSITION HAVING SAID ACID. WO/2013/167088, 2013.

(13) Fang, Z.; Wills, M. Asymmetric Transfer Hydrogenation of Functionalized Acetylenic Ketones. *J. Org. Chem.* **2013**, *78*, 8594–8605.

(14) Bermejo Góme, A.; González, M. A. C.; Lübcke, M.; Johansson, M. J.; Schou, M.; Szabó, K. J. Synthesis of trifluoromethyl moieties by late-stage copper (I) mediated nucleophilic fluorination. *Journal of Fluorine Chemistry* **2017**, *194*, 51–57.

(15) Kohls, H.; Anderson, M.; Dickerhoff, J.; Weisz, K.; Córdova, A.; Berglund, P.; Brundiek, H.; Bornscheuer, U. T.; Höhne, M. Selective Access to All Four Diastereomers of a 1,3-Amino Alcohol by Combination of a Keto Reductase- and an Amine Transaminase-Catalysed Reaction. *Adv. Synth. Catal.* **2015**, *357*, 1808–1814.

(16) Harris, T.; Gomes Gdos, P.; Clark, R. J.; Alabugin, I. V. Domino Fragmentations in Traceless Directing Groups of Radical Cascades: Evidence for the Formation of Alkoxy Radicals via C-O Scission. *J. Org. Chem.* **2016**, *81*, 6007–6017.

(17) Casey, B. M.; Sadasivam, D. V.; Flowers Ii, R. A. Mechanistic studies on the CAN-mediated intramolecular cyclization of delta-aryl-beta-dicarbonyl compounds. *Beilstein J. Org. Chem.* **2013**, *9*, 1472–1479.
(18) Carlone, A.; Bartoli, G.; Bosco, M.; Pesciaioli, F.; Ricci, P.; Sambri, L.; Melchiorre, P. Organocatalytic Asymmetric β-Hydroxylation of α,β-Unsaturated Ketones. *Eur. J. Org. Chem.* **2007**, *2007*, 5492–5495.
(19) Li, J.; Preinfalk, A.; Maulide, N. Diastereo- and Enantioselective Access to Stereotriads through a Flexible Coupling of Substituted Aldehydes and Alkenes. *Angew. Chem. Int. Ed.* **2019**, *58*, 5887–5890.
(20) Ermanis, K.; Hsiao, Y. T.; Kaya, U.; Jeuken, A.; Clarke, P. A. The stereodivergent formation of 2,6-cis and 2,6-trans-tetrahydropyrans: experimental and computational investigation of the mechanism of a thioester oxy-Michael cyclization. *Chem. Sci.* **2017**, *8*, 482–490.
(21) Gesinski, M. R.; Tadpetch, K.; Rychnovsky, S. D. Symmetric macrocycles by a Prins dimerization and macrocyclization strategy. *Org. Lett.* **2009**, *11*, 5342–5345.
(22) Sanford, A. B.; Thane, T. A.; McGinnis, T. M.; Chen, P. P.; Hong, X.; Jarvo, E. R. Nickel-Catalyzed Alkyl-Alkyl Cross-Electrophile Coupling Reaction of 1,3-Dimesylates for the Synthesis of Alkylcyclopropanes. *J. Am. Chem. Soc.* **2020**, *142*, 5017–5023.
(23) Lunic, D.; Sanosa, N.; Funes-Ardoiz, I.; Teskey, C. J. Mild and Chemoselective Carboxylic Acid Reduction Promoted by Borane Catalysis. *Angew. Chem. Int. Ed.* **2022**, *61*, e202207647.
(24) Schaubach, S.; Gebauer, K.; Ungeheuer, F.; Hoffmeister, L.; Ilg, M. K.; Wirtz, C.; Fürstner, A. A Two-Component Alkyne Metathesis Catalyst System with an Improved Substrate Scope and Functional Group Tolerance: Development and Applications to Natural Product Synthesis. *Chem. Eur. J.* **2016**, *22*, 8494–8507.
(25) Ermanis, K.; Hsiao, Y.-T.; Kaya, U.; Jeuken, A.; Clarke, P. A. The stereodivergent formation of 2,6-cis and 2,6-trans-tetrahydropyrans: experimental and computational investigation of the mechanism of a thioester oxy-Michael cyclization. *Chem. Sci.* **2017**, *8*, 482–490, 10.1039/C6SC03478K.
(26) Zhang, L. L.; Gao, Y. Z.; Cai, S. H.; Yu, H.; Shen, S. J.; Ping, Q.; Yang, Z. P. Ni-catalyzed enantioconvergent deoxygenative reductive cross-coupling of unactivated alkyl alcohols and aryl bromides. *Nat. Commun.* **2024**, *15*, 2733.
(27) Chen, K.-M.; Hardtmann, G. E.; Prasad, K.; Repič, O.; Shapiro, M. J. 1,3-diastereoselective reduction of β-hydroxyketones utilizing alkoxydialkylboranes. *Tetrahedron Lett.* **1987**, *28*, 155–158.
(28) Zong, Y.; Zou, X.; Tao, H.; Huang, Q.; Chen, G.-Q.; Zhang, X. Highly Efficient and Enantioselective Iridium-Catalyzed Asymmetric Reductive Cycloetherification. *J. Am. Chem. Soc.* **2024**, *146*, 34107–34117.
(29) Wu, Y.; Du, C.; Hu, C.; Li, Y.; Xie, Z. Biomimetic Synthesis of Hyperolactones. *J. Org. Chem.* **2011**, *76*, 4075–4081.
(30) Barroso, S.; Blay, G.; Al-Midfa, L.; Muñoz, M. C.; Pedro, J. R. Copper(II)−Bis(oxazoline) Catalyzed Asymmetric Diels−Alder Reaction with α'-Arylsulfonyl Enones as Dienophiles. *J. Org. Chem.* **2008**, *73*, 6389–6392.
(31) Zhao, H.; Yang, A.; Zhang, N.; Li, S.; Yuan, T.; Ding, N.; Zhang, S.; Bao, S.; Wang, C.; Zhang, Y.; et al. Insecticidal Endostemonines A–J Produced by Endophytic Streptomyces from Stemona sessilifolia. *J. Agric. Food. Chem.* **2020**, *68*, 1588–1595.
(32) Xu, X.; Peng, L.; Chang, X.; Guo, C. Ni/Chiral Sodium Carboxylate Dual Catalyzed Asymmetric O-Propargylation. *J. Am. Chem. Soc.* **2021**, *143*, 21048–21055.

(33) Dunsmore, C. J.; Carr, R.; Fleming, T.; Turner, N. J. A Chemo-Enzymatic Route to Enantiomerically Pure Cyclic Tertiary Amines. *J. Am. Chem. Soc.* **2006**, *128*, 2224–2225.
(34) Delaney, C. P.; Zahrt, A. F.; Kassel, V. M.; Denmark, S. E. Effects of Ring Size and Steric Encumbrance on Boron-to-Palladium Transmetalation from Arylboronic Esters. *J. Org. Chem.* **2024**, *89*, 16170–16184.
(35) Matsumoto, K.; Sato, Y.; Shimojo, M.; Hatanaka, M. Highly enantioselective preparation of C2-symmetrical diols: microbial hydrolysis of cyclic carbonates. *Tetrahedron Asymmetry* **2000**, *11*, 1965–1973.
(36) Drosos, N.; Cheng, G. J.; Ozkal, E.; Cacherat, B.; Thiel, W.; Morandi, B. Catalytic Reductive Pinacol-Type Rearrangement of Unactivated 1,2-Diols through a Concerted, Stereoinvertive Mechanism. *Angew. Chem. Int. Ed.* **2017**, *56*, 13377–13381.
(37) Balagam, B.; Mitra, R.; Richardson, D. E. Osmium-catalyzed asymmetric dihydroxylation by carbon dioxide-activated hydrogen peroxide and N-methylmorpholine. *Tetrahedron Lett.* **2008**, *49*, 1071–1075.
(38) Ariawan, A. D.; Mansour, F.; Richardson, N.; Bhadbhade, M.; Ho, J.; Hunter, L. The Effect of Vicinal Difluorination on the Conformation and Potency of Histone Deacetylase Inhibitors. *Molecules* **2021**, *26*.
(39) Pelz, N. F.; Morken, J. P. Modular asymmetric synthesis of 1,2-diols by single-pot allene diboration/hydroboration/cross-coupling. *Org. Lett.* **2006**, *8*, 4557–4559.
(40) Meng, Q. Y.; Schirmer, T. E.; Katou, K.; Konig, B. Controllable Isomerization of Alkenes by Dual Visible-Light-Cobalt Catalysis. *Angew. Chem. Int. Ed.* **2019**, *58*, 5723–5728.
(41) Molander, G. A.; Argintaru, O. A. Stereospecific Ni-catalyzed cross-coupling of potassium alkenyltrifluoroborates with alkyl halides. *Org. Lett.* **2014**, *16*, 1904–1907.
(42) Ding, Y.; Wu, J.; Zhang, T.; Liu, H.; Huang, H. Site-Selective Carbonylative Cyclization with Two Allylic C-H Bonds Enabled by Radical Differentiation. *J. Am. Chem. Soc.* **2024**, *146*, 19635–19642.
(43) Liu, X.; Liu, B.; Liu, Q. Migratory Hydrogenation of Terminal Alkynes by Base/Cobalt Relay Catalysis. *Angew. Chem. Int. Ed.* **2020**, *59*, 6750–6755.
(44) Wang, Y.; Qin, C.; Jia, X.; Leng, X.; Huang, Z. An Agostic Iridium Pincer Complex as a Highly Efficient and Selective Catalyst for Monoisomerization of 1-Alkenes to trans-2-Alkenes. *Angew. Chem. Int. Ed.* **2017**, *56*, 1614–1618.
(45) Saito, K.; Kondo, K.; Akiyama, T. B(C6F5)3-Catalyzed Hydrodesulfurization Using Hydrosilanes--Metal-Free Reduction of Sulfides. *Org. Lett.* **2015**, *17*, 3366–3369.
(46) Awano, K.-i.; Yanai, T.; Watanabe, I.; Takagi, Y.; Kitahara, T.; Mori, K. Synthesis of All Four Possible Stereoisomers of 1-Phenyl-2,3-butanediol and Both Enantiomers of 3-Hydroxy-4-phenyl-2-butanone to Determine the Absolute Configuration of the Natural Constituents. *Biosci. Biotechnol. Biochem.* **2014**, *59*, 1251–1254.
(47) Du, Y. M.; Lin, J. N.; Li, Y. L.; Yu, Q.; Shu, W. Nickel-Catalyzed Adaptive Migration-Enabled Asymmetric Cross-Hydrocarbonylation of Unactivated Alkenes. *J. Am. Chem. Soc.* **2025**, *147*, 18944–18952.
(48) Pospíšil, J. Simple protocol for enhanced (E)-selectivity in Julia–Kocienski reaction. *Tetrahedron Lett.* **2011**, *52*, 2348–2352.
(49) Caporusso, A. M.; Lardicci, L. Synthesis of chiral alk-1-ynes containing an α-phenylethyl group. *J. Chem. Soc., Perkin Trans. 1* **1983**, 949–953.
(50) Buslov, I.; Becouse, J.; Mazza, S.; Montandon-Clerc, M.; Hu, X. Chemoselective Alkene Hydrosilylation Catalyzed by Nickel Pincer Complexes. *Angew. Chem. Int. Ed.* **2015**, *54*, 14523–14526.

(51) Sastraruji, T.; Pyne, S. G.; Ung, A. T. Oxidation of acyclic alkenes and allyl and benzyl ethers with DIB/t-BuOOH/Mg(OAc)2. *Tetrahedron* **2012**, *68*, 598–602.
(52) Rychnovsky, S. D.; Griesgraber, G.; Zeller, S.; Skalitzky, D. J. Optically pure 1,3-diols from (2R,4R)- and (2S,4S)-1,2:4,5-diepoxypentane. *J. Org. Chem.* **1991**, *56*, 5161–5169.
(53) Marinetti, A.; Genêt, J.-P.; Jus, S.; Blanc, D.; Ratovelomanana-Vidal, V. Chiral 1,2-Bis(phosphetano)benzenes: Preparation and Use in the Ru-Catalyzed Hydrogenations of Carbonyl Derivatives. *Chem. Eur. J.* **1999**, *5*, 1160–1165.
(54) Touchette, M. H.; Bommineni, G. R.; Delle Bovi, R. J.; Gadbery, J. E.; Nicora, C. D.; Shukla, A. K.; Kyle, J. E.; Metz, T. O.; Martin, D. W.; Sampson, N. S.; et al. Diacyltransferase Activity and Chain Length Specificity of Mycobacterium tuberculosis PapA5 in the Synthesis of Alkyl β-Diol Lipids. *Biochemistry* **2015**, *54*, 5457–5468.
(55) Narasimhulu, M.; Srikanth Reddy, T.; Chinni Mahesh, K.; Sai Krishna, A.; Venkateswara Rao, J.; Venkateswarlu, Y. Synthesis of yashabushidiol and its analogues and their cytotoxic activity against cancer cell lines. *Bioorg. Med. Chem. Lett.* **2009**, *19*, 3125–3127.
(56) Gadakh, S. K.; Sudalai, A. Co-catalyzed two-stereocentered hydrolytic kinetic resolution: application to the synthesis of yashabushidiols A and B and the lactone unit of compactin and mevinolin. *Tetrahedron: Asymmetry* **2015**, *26*, 118–123.
(57) Fleming, I.; Henning, R.; Parker, D. C.; Plaut, H. E.; Sanderson, P. E. J. The phenyldimethylsilyl group as a masked hydroxy group. *J. Chem. Soc., Perkin Trans. 1.* **1995**.
(58) Kundig, E. P.; Enriquez Garcia, A.; Lomberget, T.; Perez Garcia, P.; Romanens, P. Truncated Cinchona alkaloids as catalysts in enantioselective monobenzoylation of meso-1,2-diols. *Chem. Commun.* **2008**, 3519–3521.
(59) Zubar, V.; Lebedev, Y.; Azofra, L. M.; Cavallo, L.; El-Sepelgy, O.; Rueping, M. Hydrogenation of CO(2) -Derived Carbonates and Polycarbonates to Methanol and Diols by Metal-Ligand Cooperative Manganese Catalysis. *Angew. Chem. Int. Ed.* **2018**, *57*, 13439–13443.
(60) Oswood, C. J.; MacMillan, D. W. C. Selective Isomerization via Transient Thermodynamic Control: Dynamic Epimerization of trans to cis Diols. *J. Am. Chem. Soc.* **2022**, *144*, 93–98.
(61) Matt, C.; Orthaber, A.; Streuff, J. Catalytic Asymmetric beta-Oxygen Elimination. *Angew. Chem. Int. Ed.* **2022**, *61*, e202114044.
(62) Mukaiyama, T.; Yoshimura, N.; Igarashi, K.; Kagayama, A. Efficient diastereoselective pinacol coupling reaction of aliphatic and aromatic aldehydes by using newly utilized low valent titanium bromide and iodide species. *Tetrahedron* **2001**, *57*, 2499–2506.
(63) Chen, K.; Zhu, H.; Liu, S.; Bai, J.; Guo, Y.; Ding, K.; Peng, Q.; Wang, X. Switch in Selectivities by Dinuclear Nickel Catalysis: 1,4-Hydroarylation of 1,3-Dienes to Z-Olefins. *J. Am. Chem. Soc.* **2023**, *145*, 24877–24888.
(64) Marinetti, A.; Genêt, J.-P.; Jus, S.; Blanc, D.; Ratovelomanana-Vidal, V. Chiral 1,2-Bis(phosphetano)benzenes: Preparation and Use in the Ru-Catalyzed Hydrogenations of Carbonyl Derivatives. *Chemistry - A European Journal* **1999**, *5*, 1160–1165.
(65) Narasimhulu, M.; Srikanth Reddy, T.; Chinni Mahesh, K.; Sai Krishna, A.; Venkateswara Rao, J.; Venkateswarlu, Y. Synthesis of yashabushidiol and its analogues and their cytotoxic activity against cancer cell lines. *Bioorg Med Chem Lett* **2009**, *19*, 3125–3127.

(66) Gadakh, S. K.; Sudalai, A. Co-catalyzed two-stereocentered hydrolytic kinetic resolution: application to the synthesis of yashabushidiols A and B and the lactone unit of compactin and mevinolin. *Tetrahedron Asymmetry* **2015**, *26*, 118–123.
(67) Kalník, M.; Gabko, P.; Kóňa, J.; Šesták, S.; Moncoľ, J.; Bella, M. (5S)-5-Benzylswainsonines as potent and selective inhibitors of Golgi α-mannosidase II: synthesis, enzyme evaluation and molecular modelling. *Bioorg. Chem.* **2024**, *150*, 107578.
(68) Matsubara, T.; Takahashi, K.; Ishihara, J.; Hatakeyama, S. Total Synthesis of (−)-Ophiodilactone A and (−)-Ophiodilactone B. *Angew. Chem. Int. Ed.* **2014**, *53*, 757–760.
(69) Wu, C.; Miller, P. A.; Miller, M. J. Syntheses and studies of amamistatin B analogs reveals that anticancer activity is relatively independent of stereochemistry, ester or amide linkage and select replacement of one of the metal chelating groups. *Bioorg. Med. Chem. Lett.* **2011**, *21*, 2611–2615.
(70) Bao, D.-H.; Wu, H.-L.; Liu, C.-L.; Xie, J.-H.; Zhou, Q.-L. Development of Chiral Spiro P-N-S Ligands for Iridium-Catalyzed Asymmetric Hydrogenation of β-Alkyl-β-Ketoesters. *Angewandte Chemie* **2015**, *127*, 8915–8918.
(71) Wang, Y.; Lin, C.; Zhang, Z.; Shen, L.; Zou, B. Directed Nickel-Catalyzed Selective Arylhydroxylation of Unactivated Alkenes under Air. *Org. Lett.* **2023**, *25*, 2172–2177.
(72) Davis, F. A.; Rao, A.; Carroll, P. J. Asymmetric Synthesis of Functionalized trans-2,6-Disubstituted Piperidines with N-Sulfinyl δ-Amino β-Ketoesters. Synthesis of (−)-Lasubine I. *Org. Lett.* **2003**, *5*, 3855–3857.
(73) Wang, D.-M.; She, L.-Q.; Yuan, H.; Wu, Y.; Tang, Y.; Wang, P. Ligand-Enabled NiII-Catalyzed Hydroxylarylation of Alkenes with Molecular Oxygen. *Angew. Chem. Int. Ed.* **2023**, *62*, e202304573.
(74) Denmark, S. E.; Fujimori, S. The Effects of a Remote Stereogenic Center in the Lewis Base Catalyzed Aldol Additions of Chiral Trichlorosilyl Enolates. *Org. Lett.* **2002**, *4*, 3477–3480.
(75) Wenglowsky, S. M.; Miduturu, C. V.; Neil Bifulco, J.; Kim, J. L. COMPOUNDS AND COMPOSITIONS USEFUL FOR TREATING DISORDERS RELATED TO NTRK. WO2017087778A1, 2017.
(76) Kumaraswamy, G.; Murthy, A. N.; Sadaiah, K. Organocatalytic enantioselective synthesis of 1,3,5-polyols by means of iterative asymmetric epoxidation: their application to the total synthesis of polyrhacitide A. *Tetrahedron* **2012**, *68*, 3179–3186.
(77) Yang, X.; Tsui, G. C. Radical Pentafluoroethylation of Unactivated Alkenes Using CuCF(2)CF(3). *Org. Lett.* **2020**, *22*, 4562–4567.

Di(oxiran-2-yl)methane

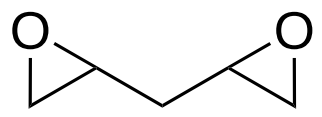

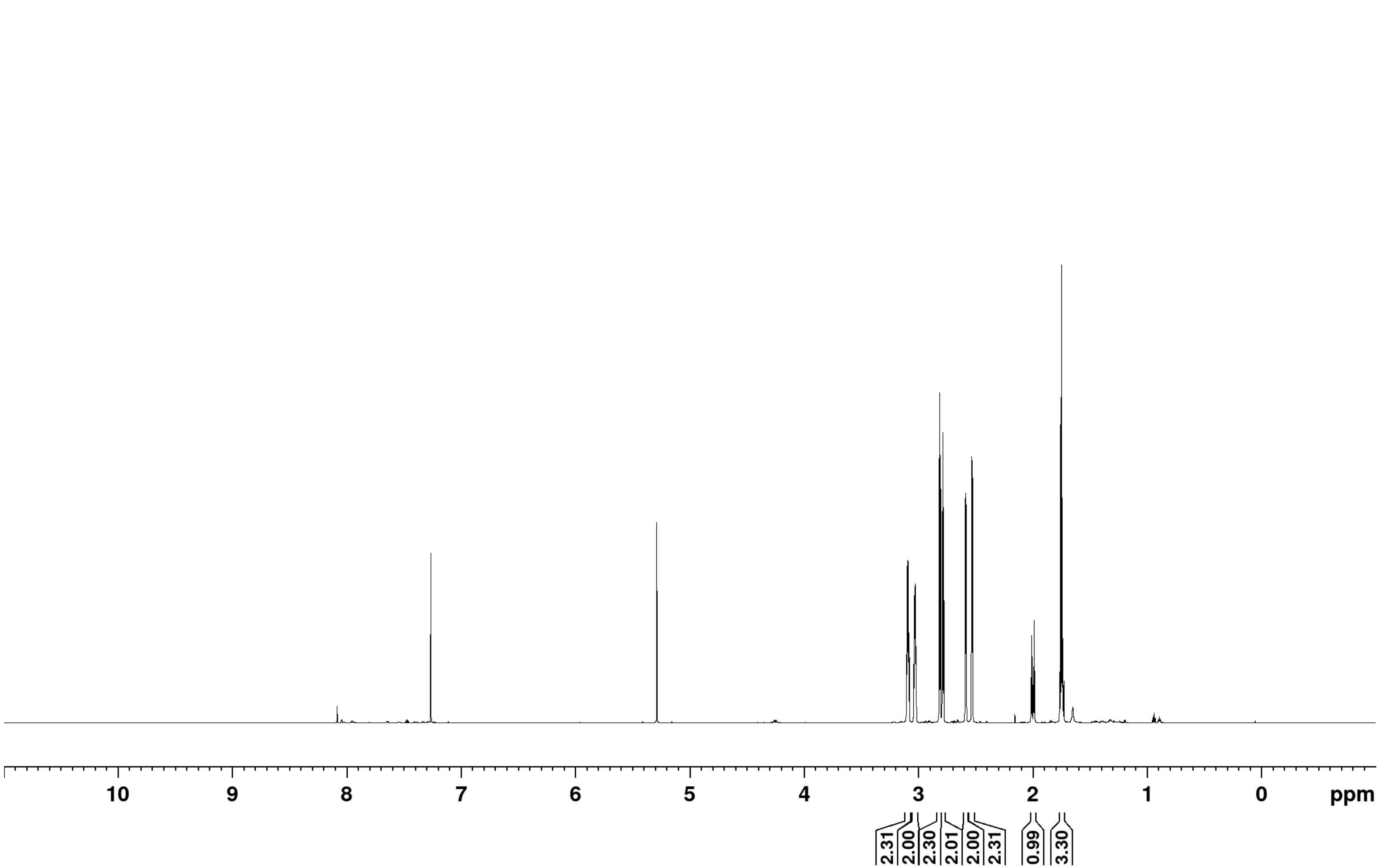

Di(oxiran-2-yl)methane

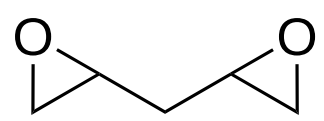

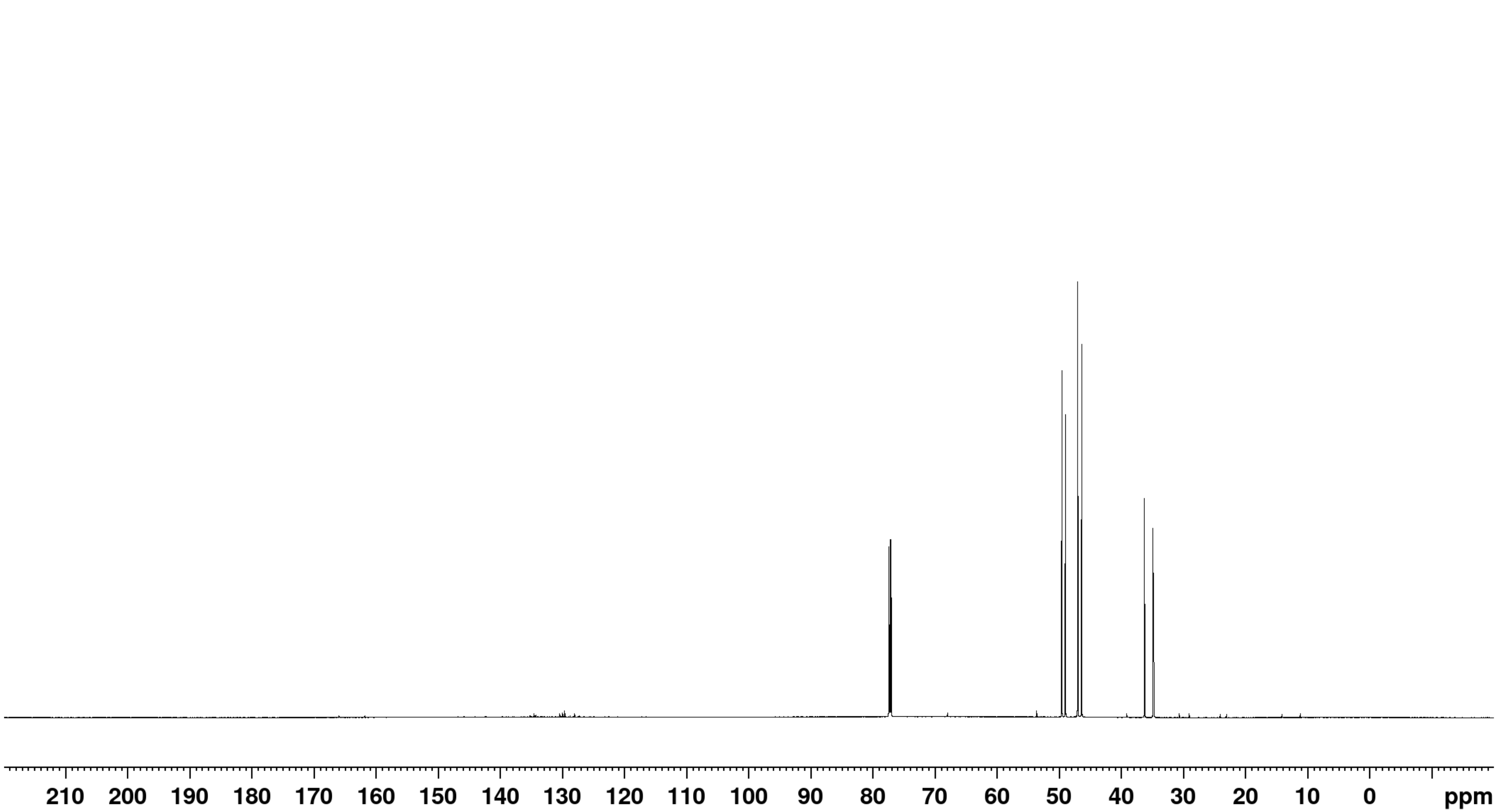

(*syn*)-heptane-3,5-diol (*meso*-1b)

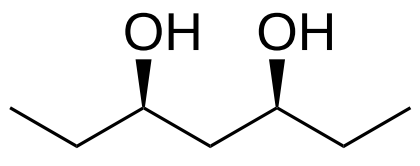

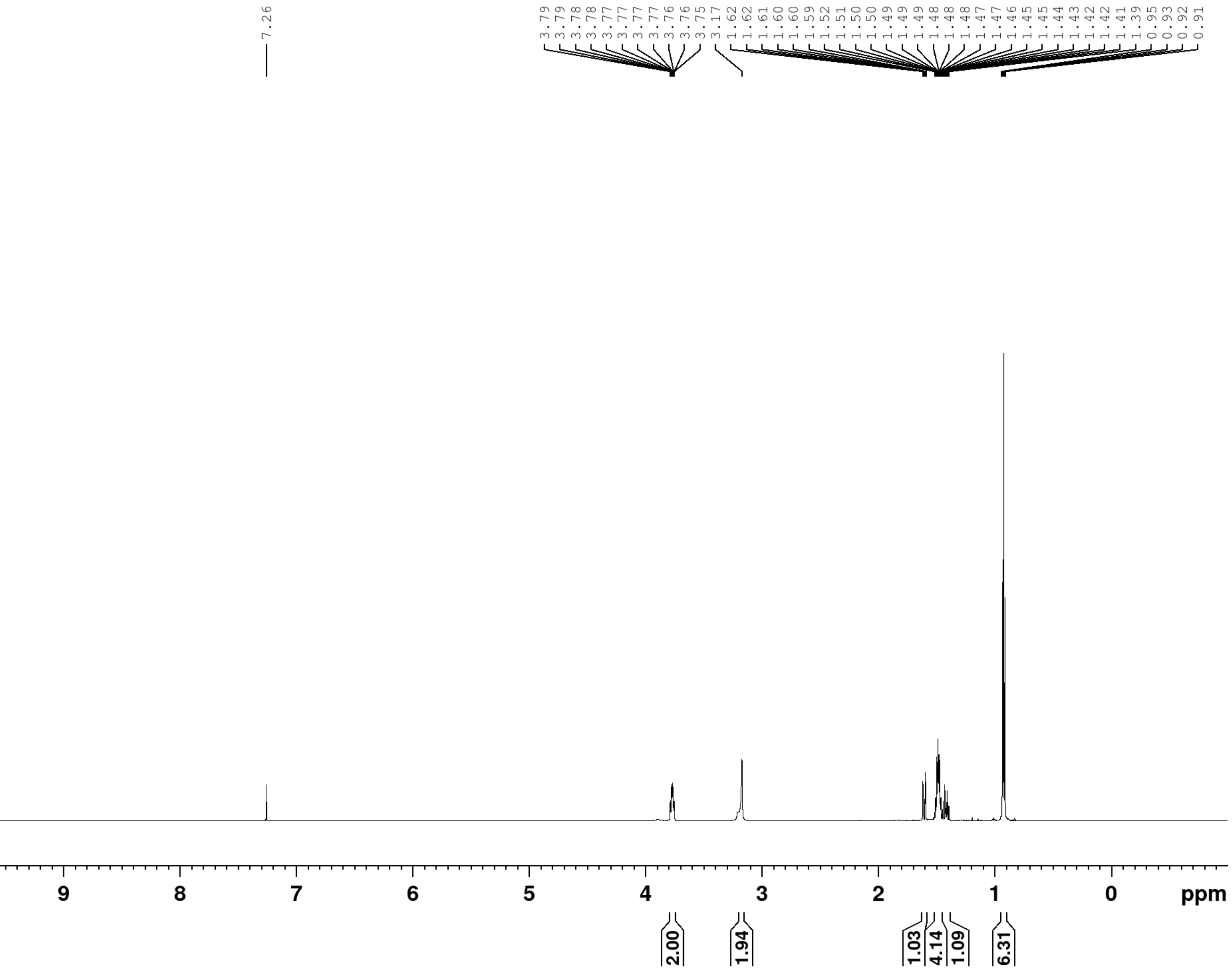

(*syn*)-heptane-3,5-diol (*meso*-1b)

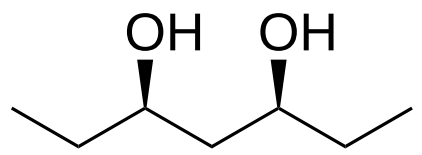

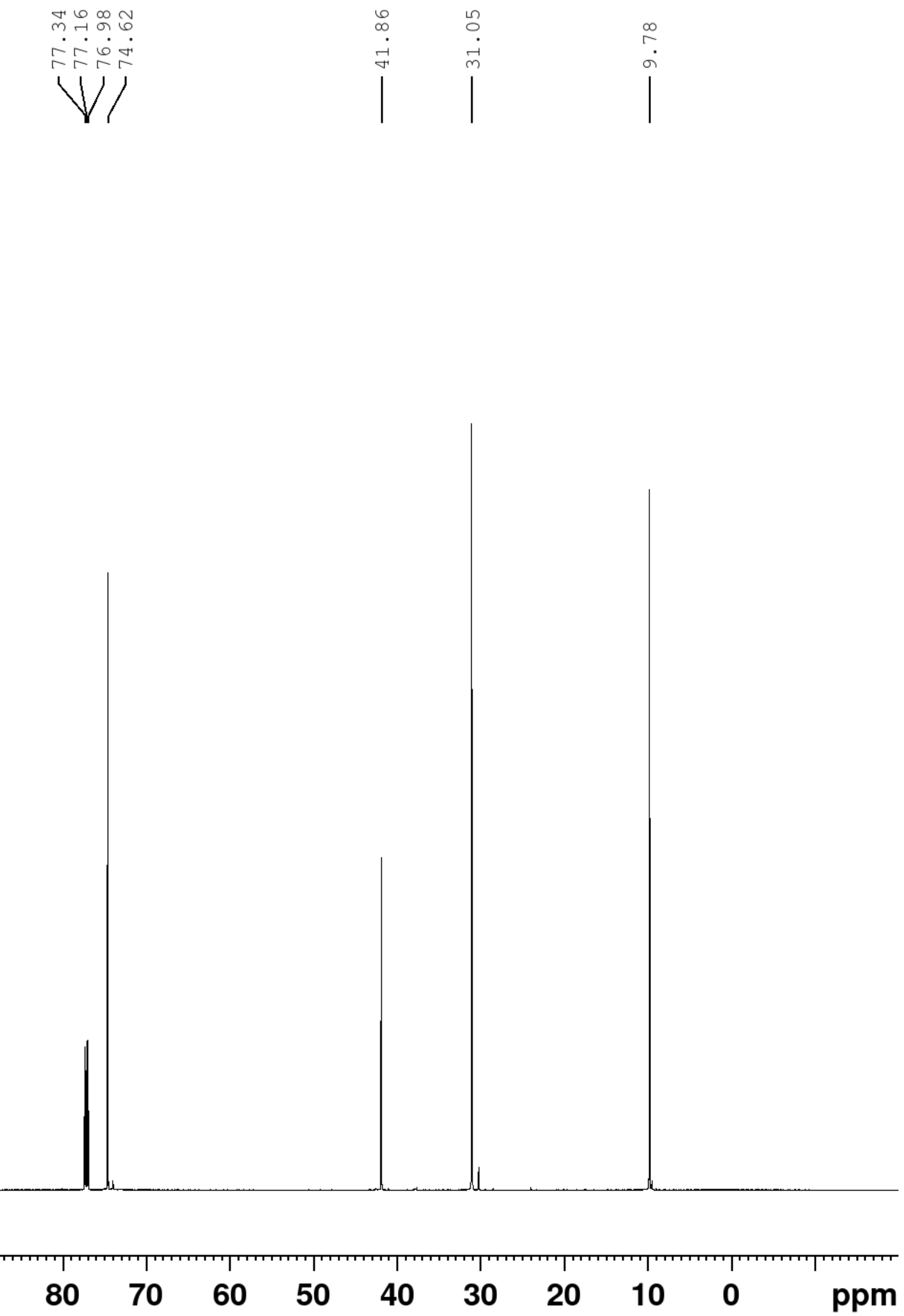

(*anti*)-heptane-3,5-diol (*rac*-1b)

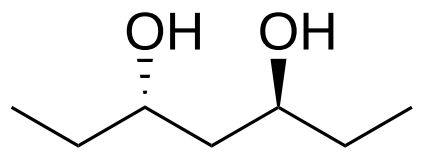

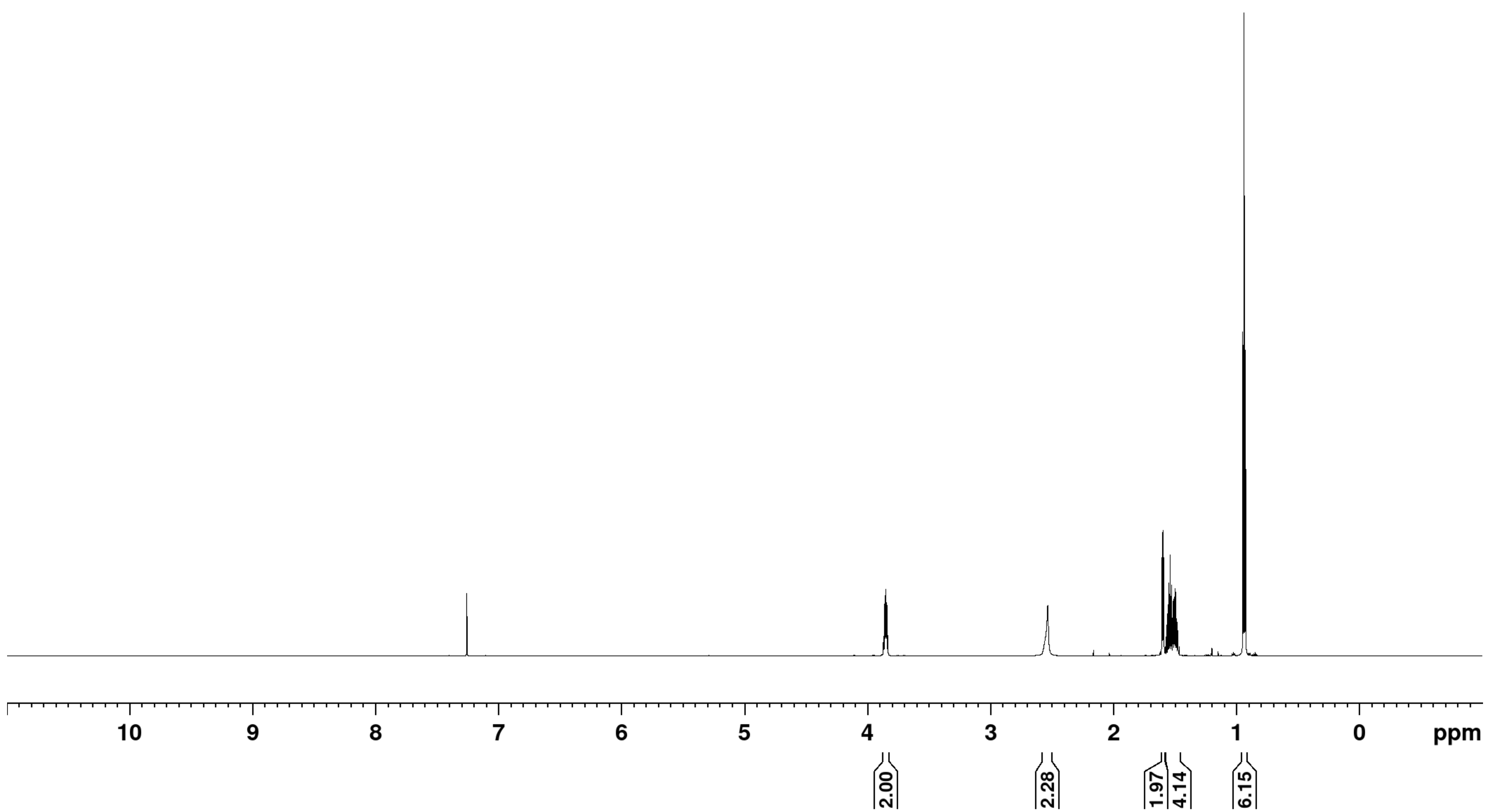

(*anti*)-heptane-3,5-diol (*rac*-1b)

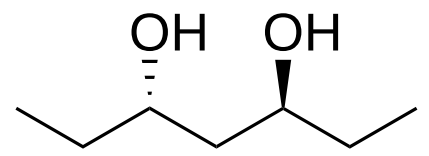

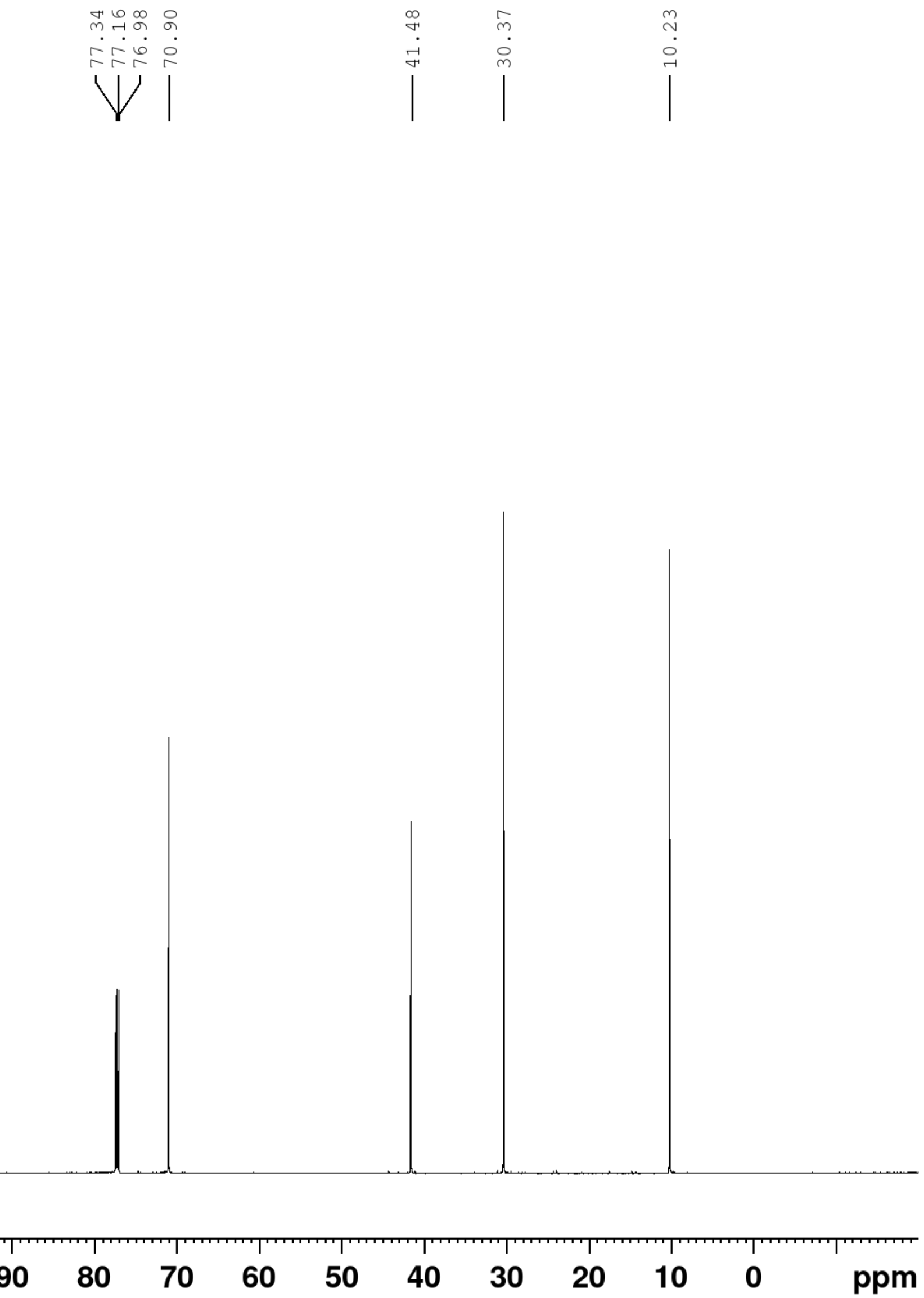

(*syn*)-nonane-4,6-diol (*meso*-1c)

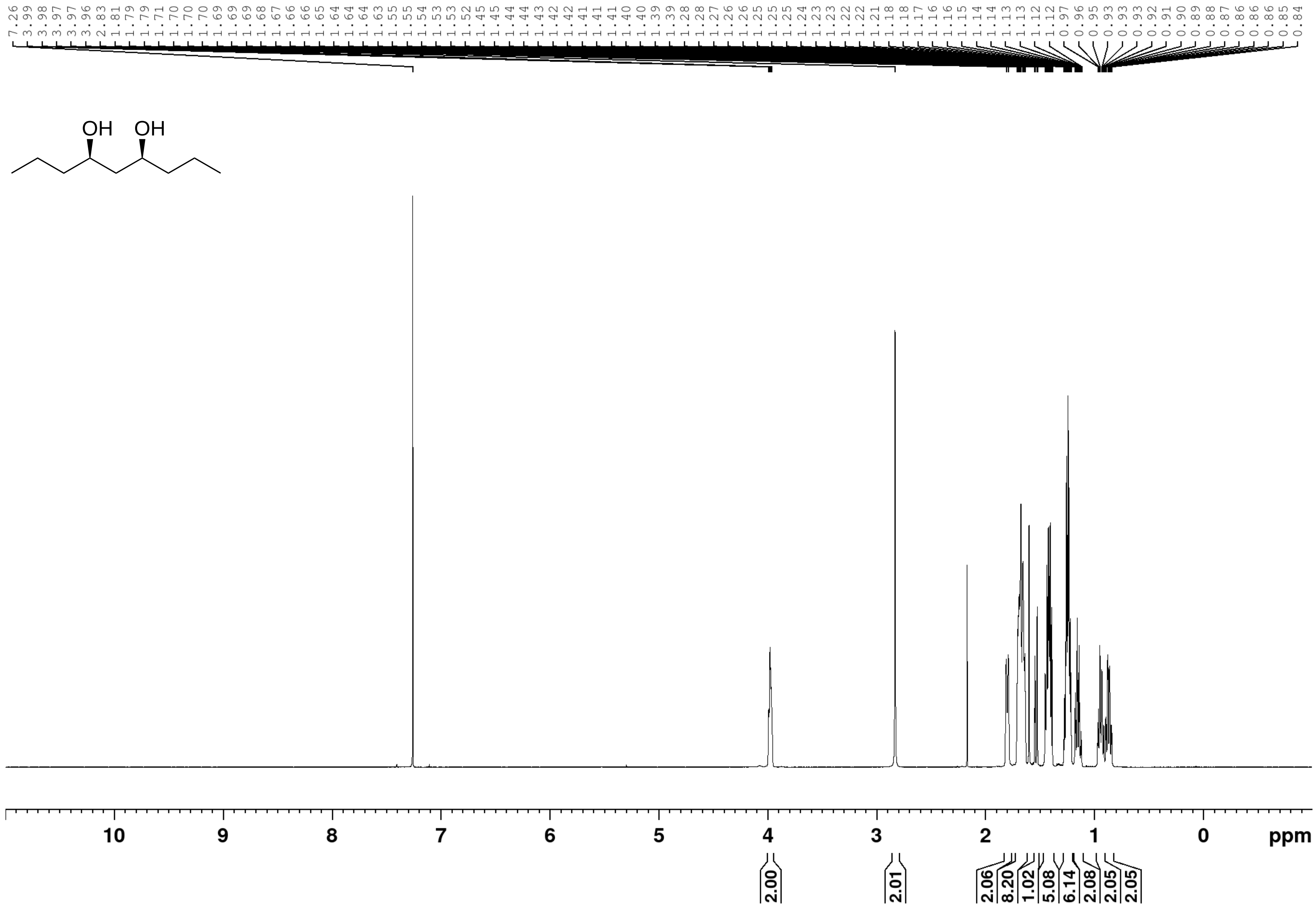

(*syn*)-nonane-4,6-diol (*meso*-1c)

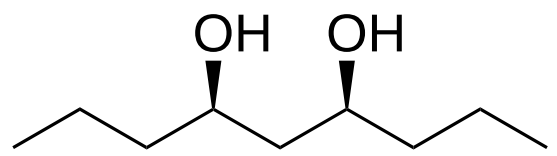

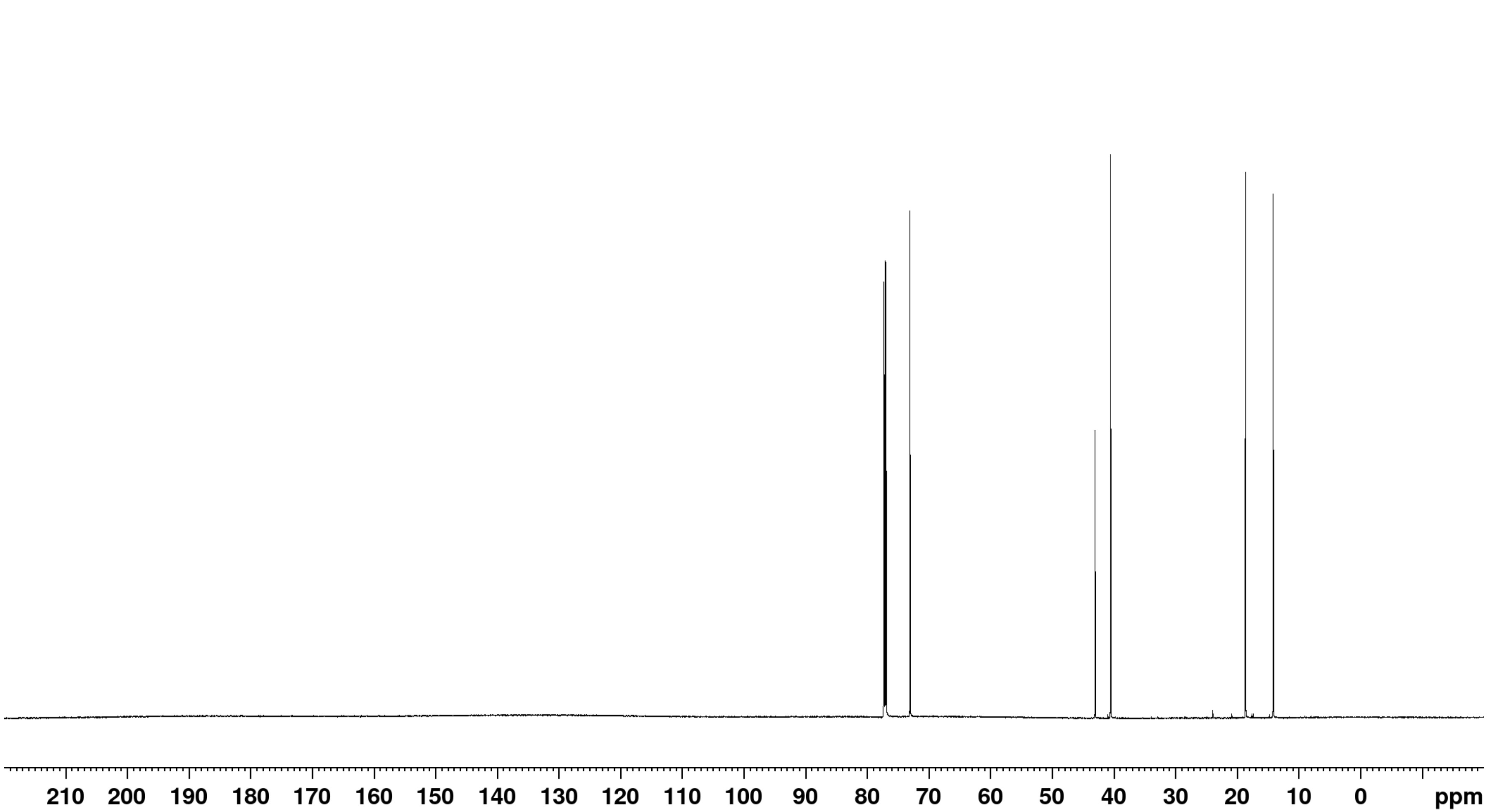

(*anti*)-nonane-4,6-diol (*rac*-1c)

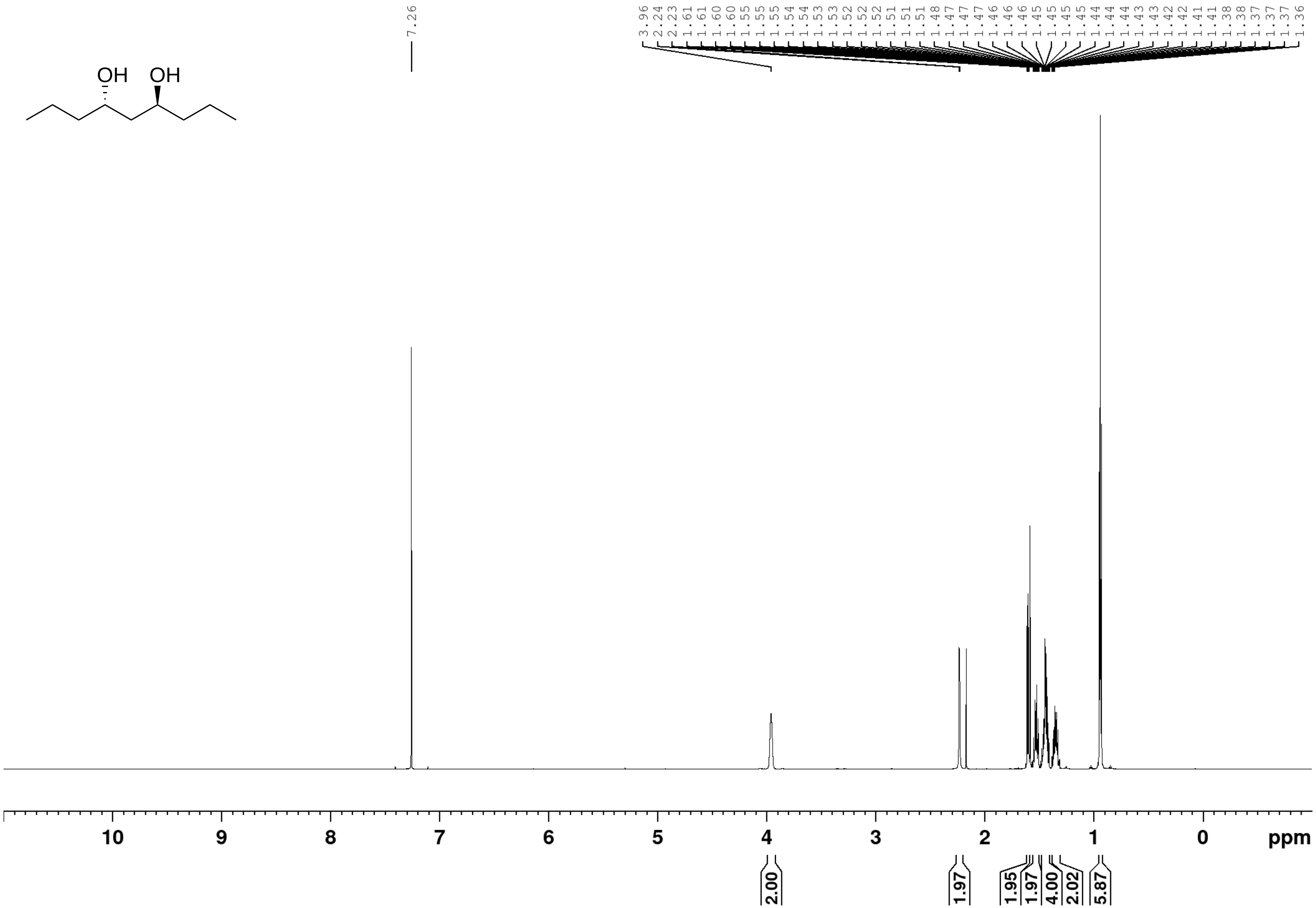

(*anti*)-nonane-4,6-diol (*rac*-1c)

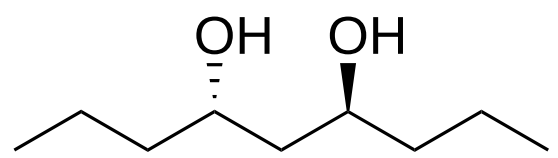

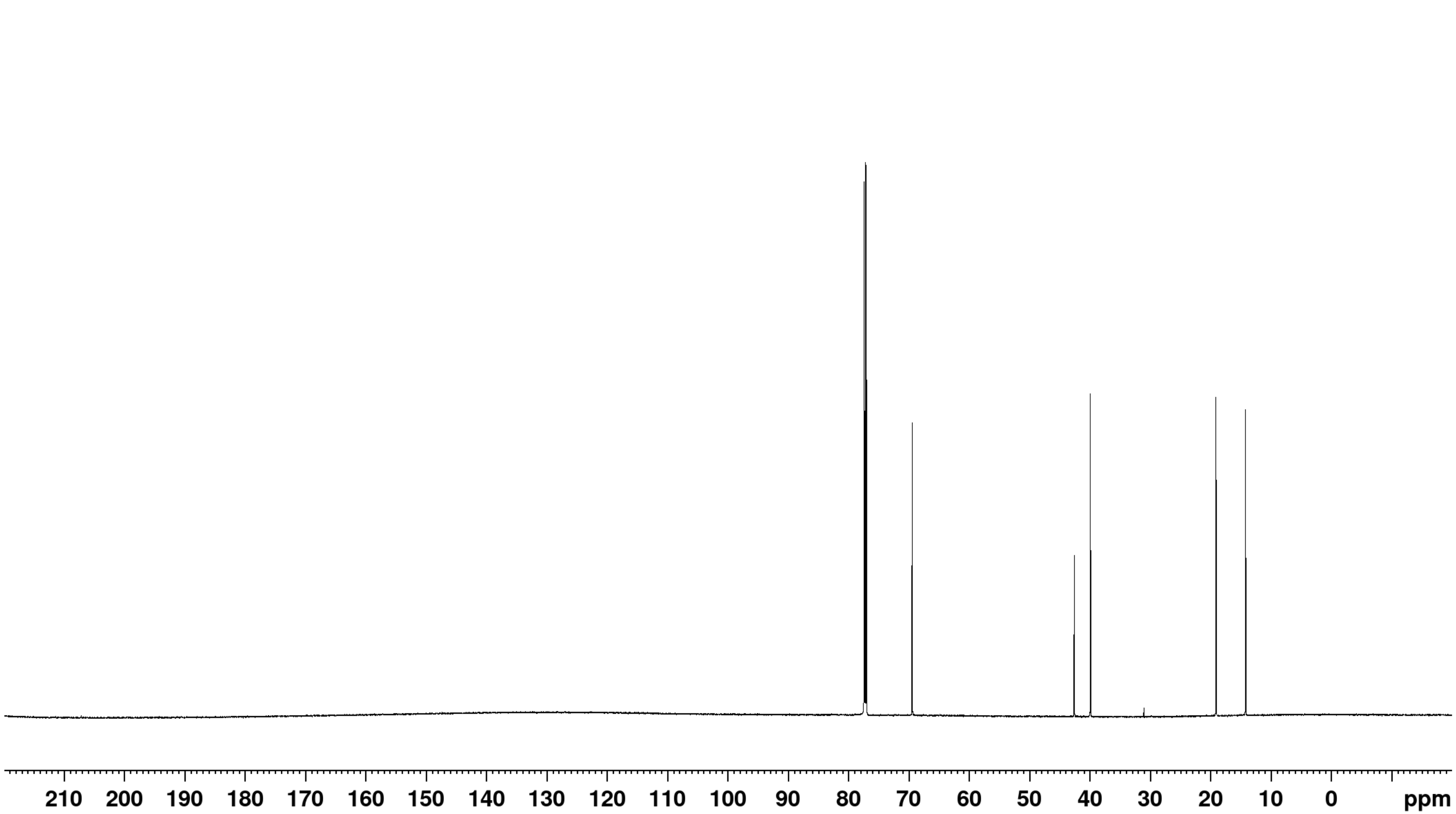

(*syn*)-undecane-5,7-diol (*meso*-1d)

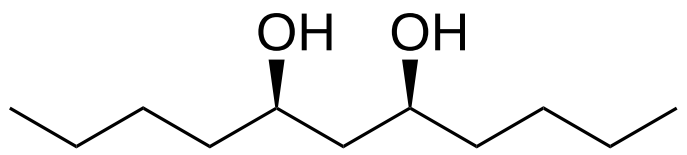

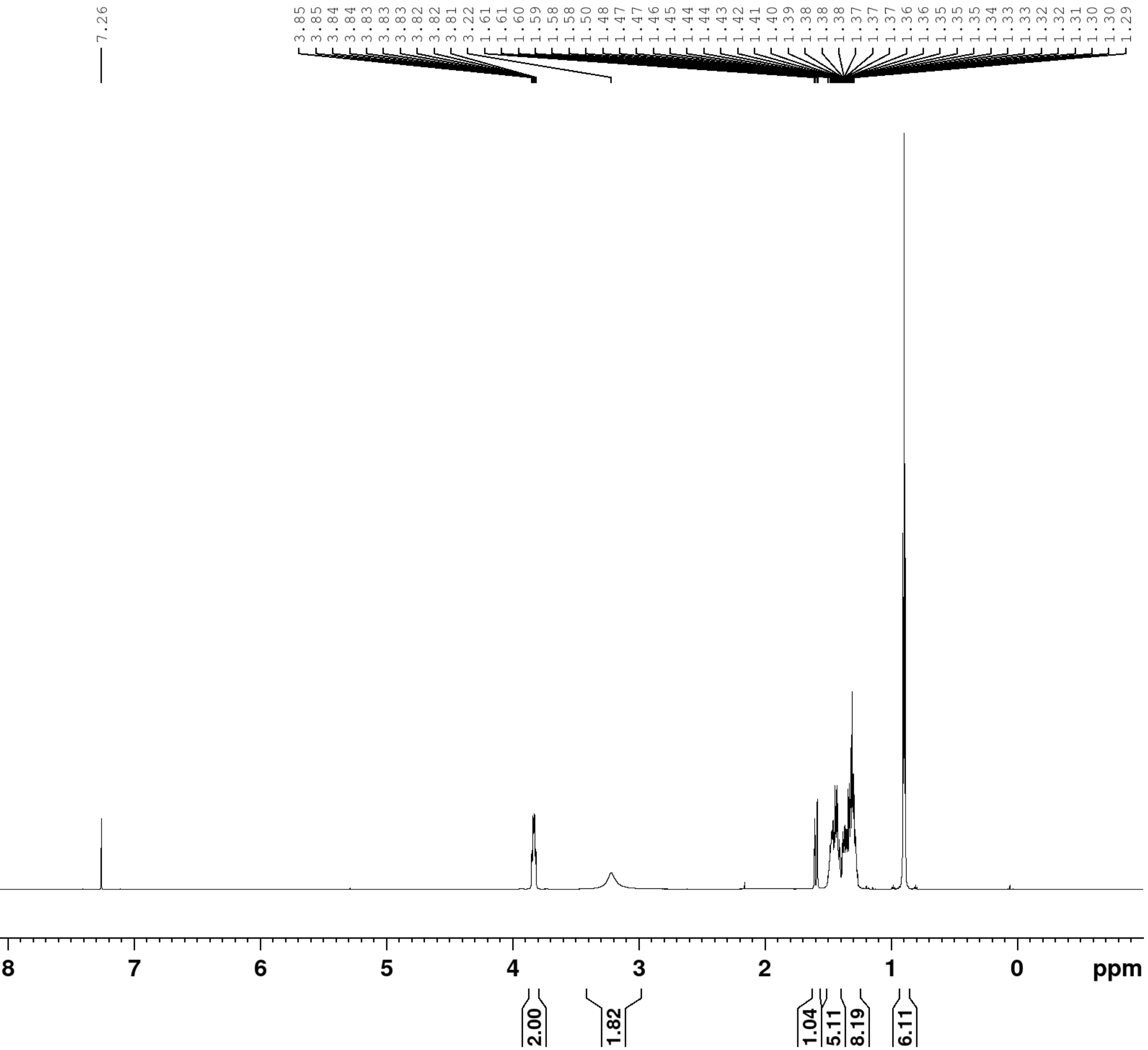

(*syn*)-undecane-5,7-diol (*meso*-1d)

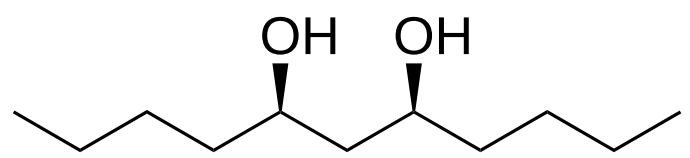

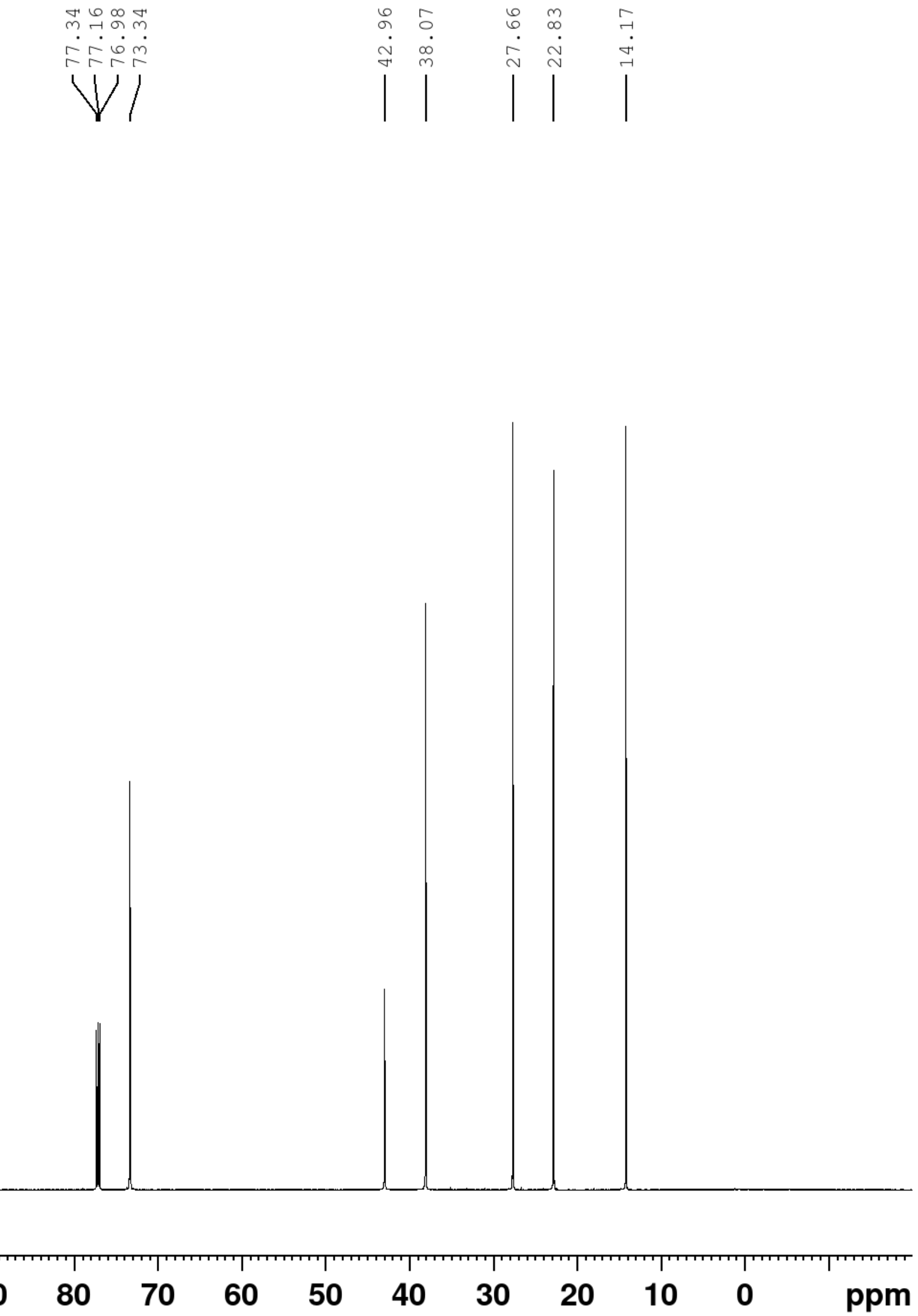

(*anti*)-undecane-5,7-diol (*rac*-1d)

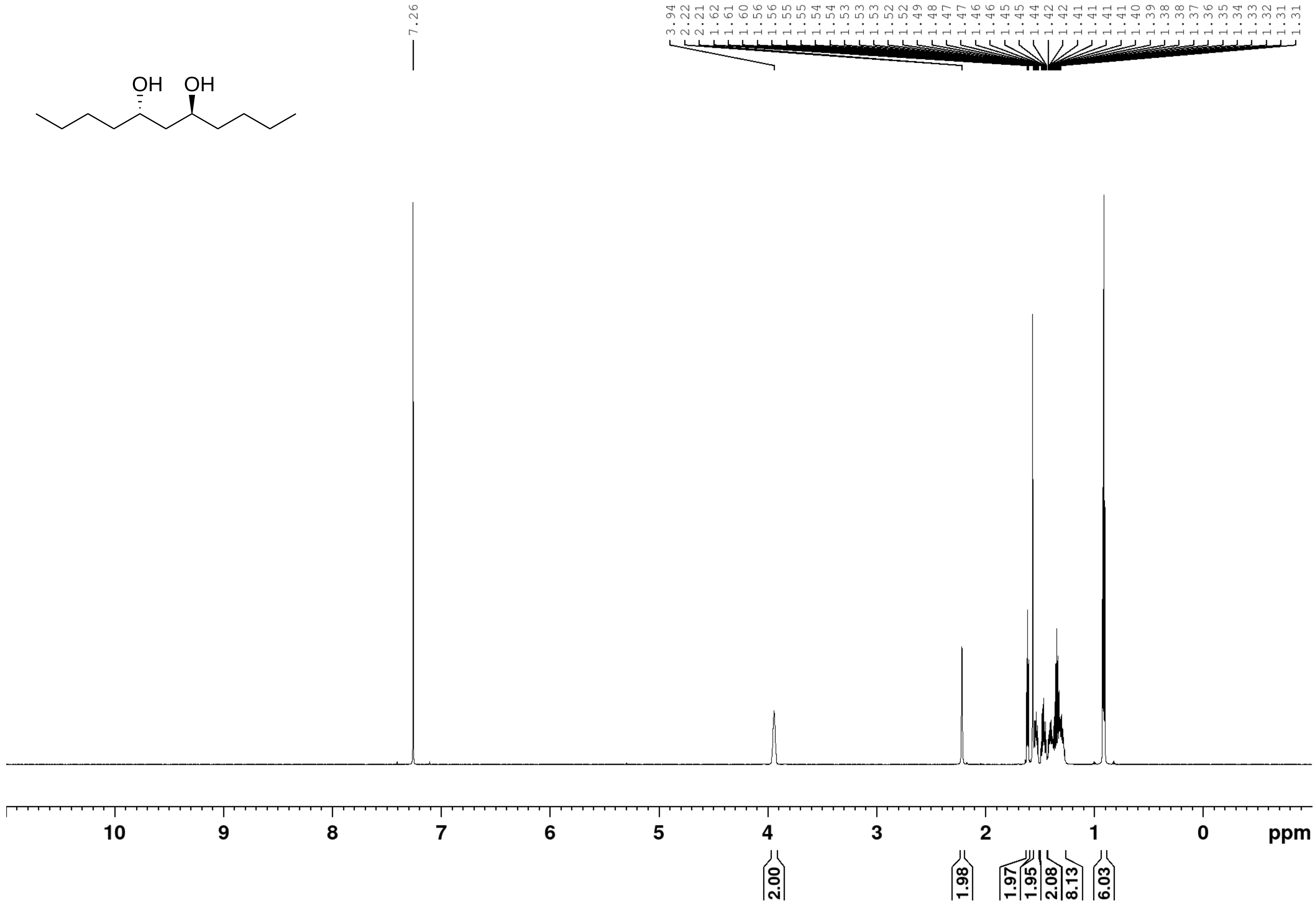

(*anti*)-undecane-5,7-diol (*rac*-1d)

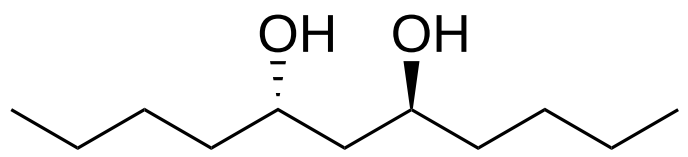

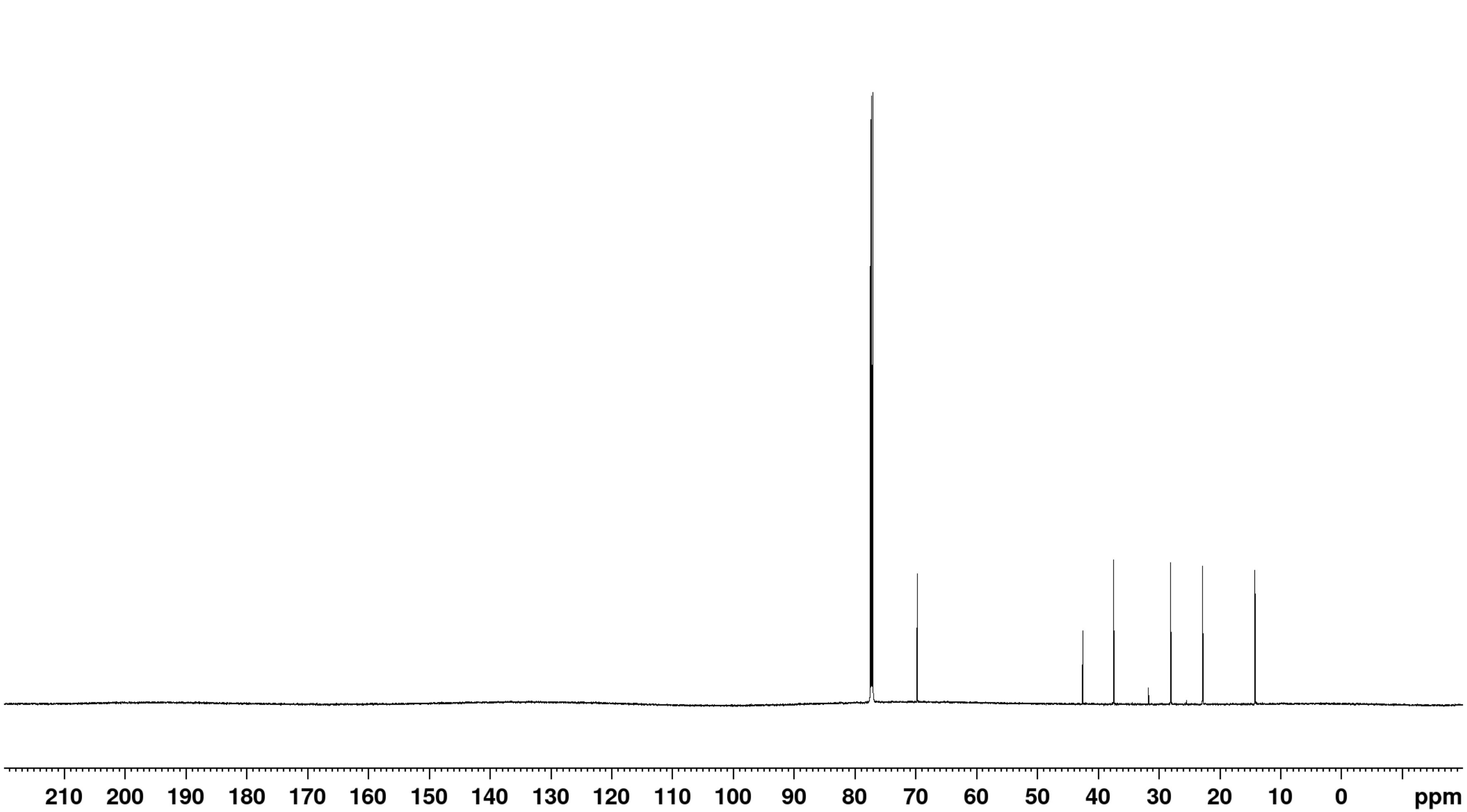

(*syn*)-2,8-dimethylnonane-4,6-diol (*meso*-1e)

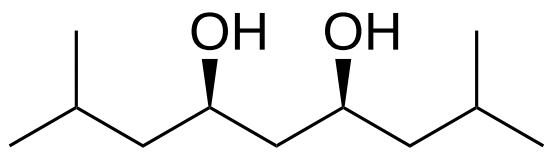

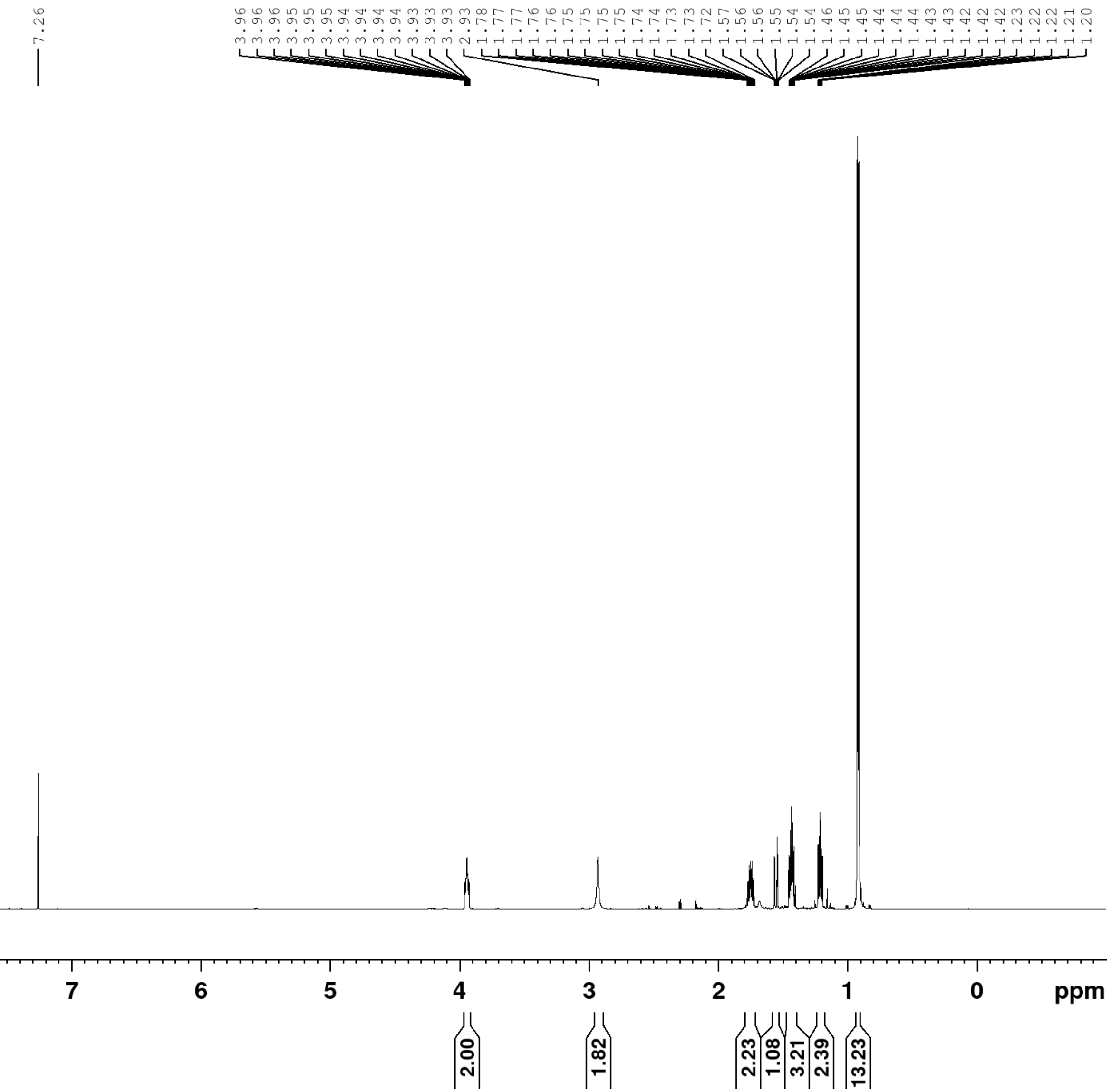

(*syn*)-2,8-dimethylnonane-4,6-diol (*meso*-1e)

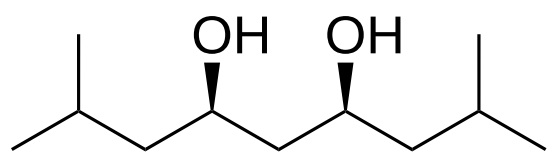

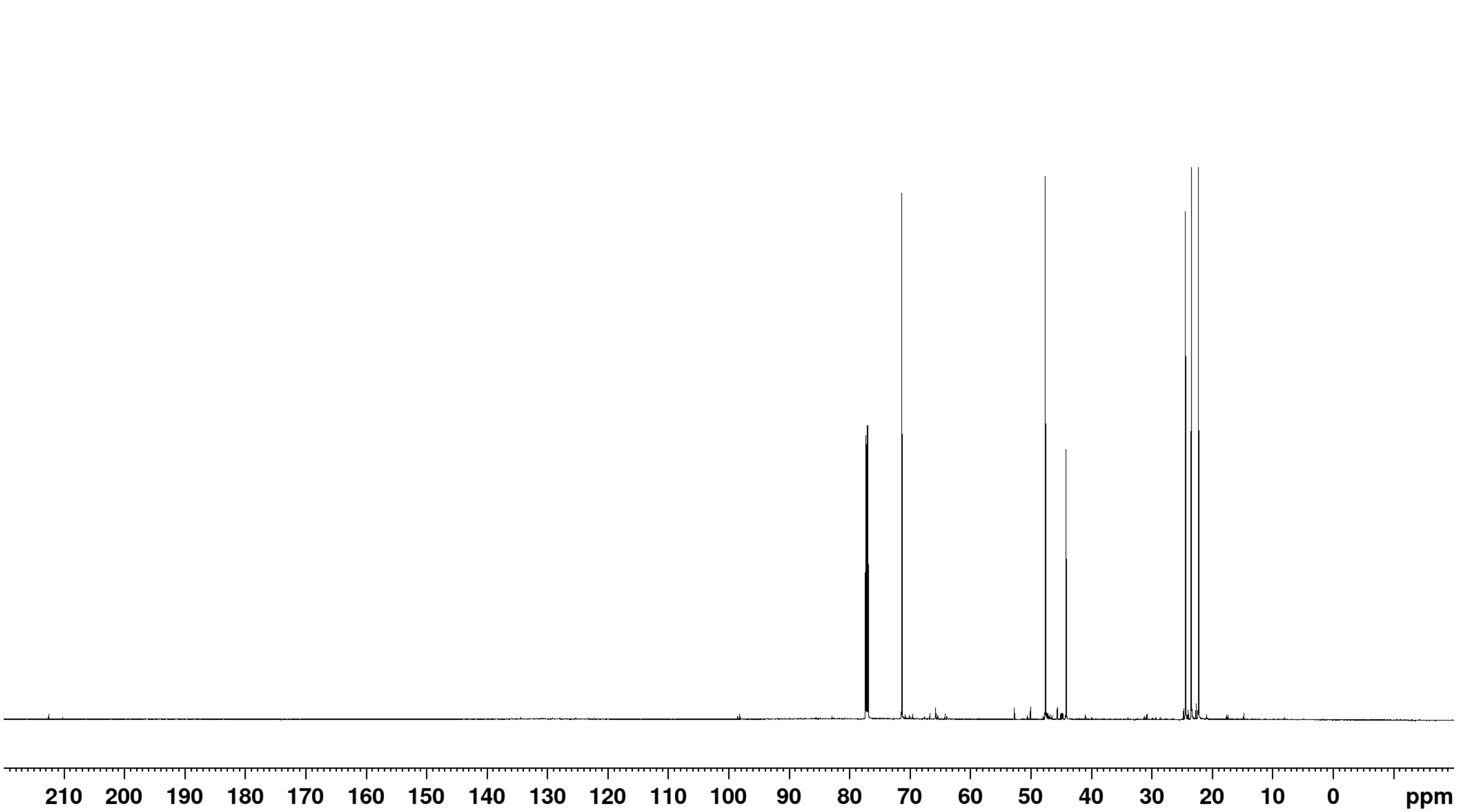

(*anti*)-2,8-dimethylnonane-4,6-diol (*rac*-1e)

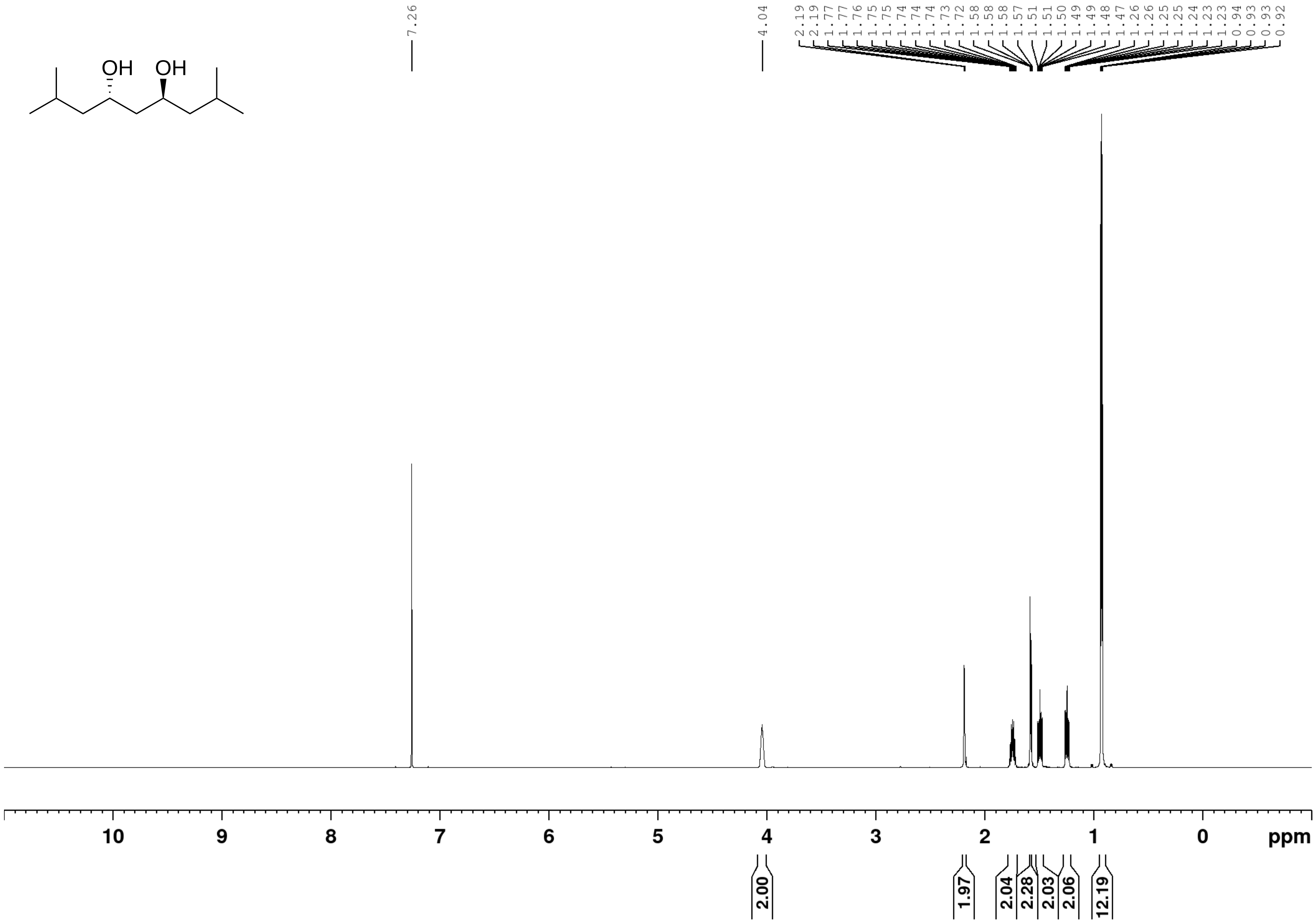

(*anti*)-2,8-dimethylnonane-4,6-diol (*rac*-1e)

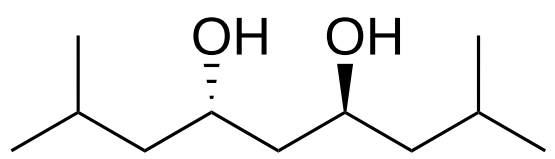

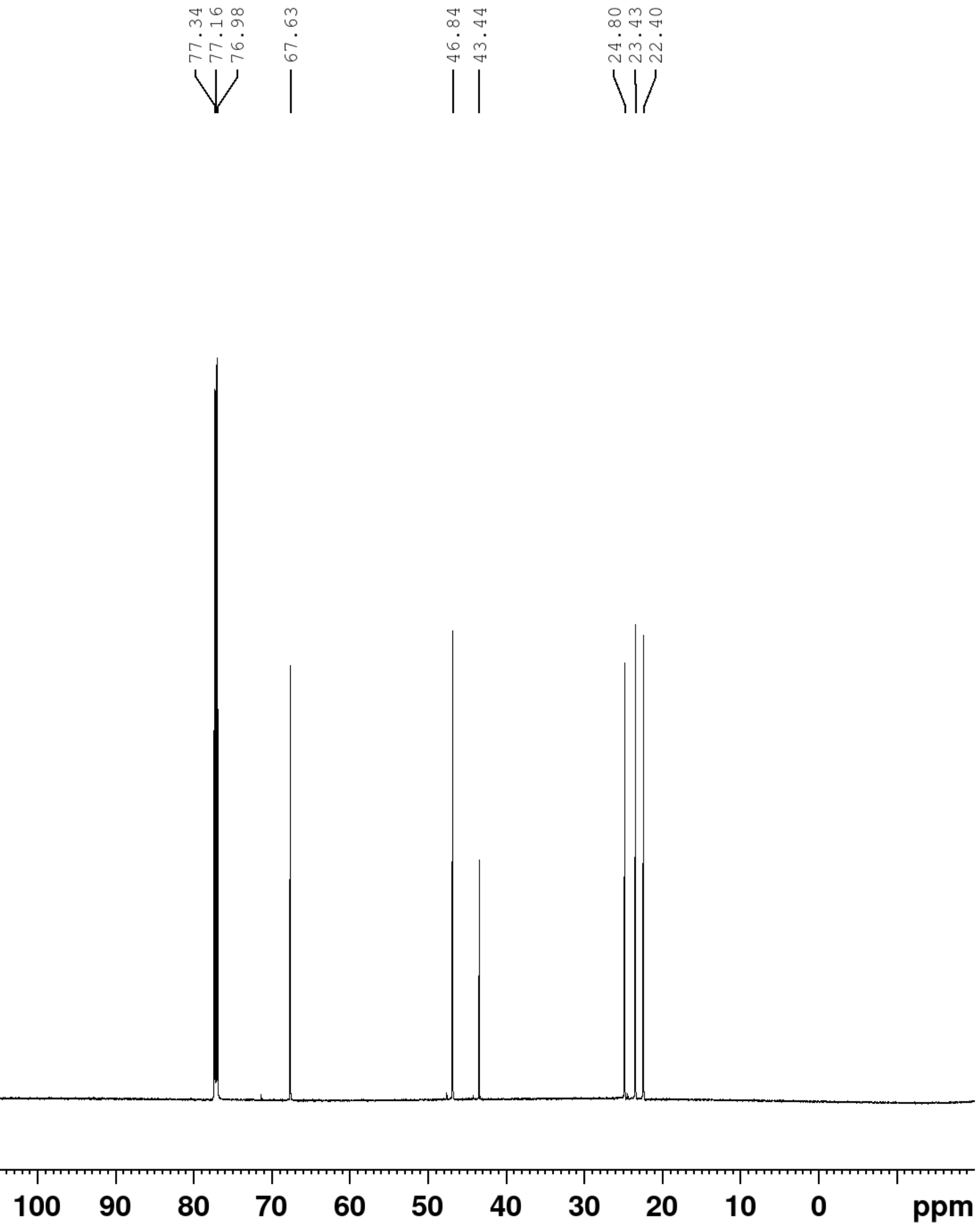

(*syn*)-2,10-dimethylundecane-5,7-diol (*meso*-1f)

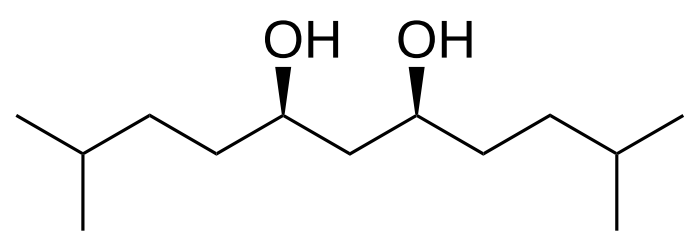

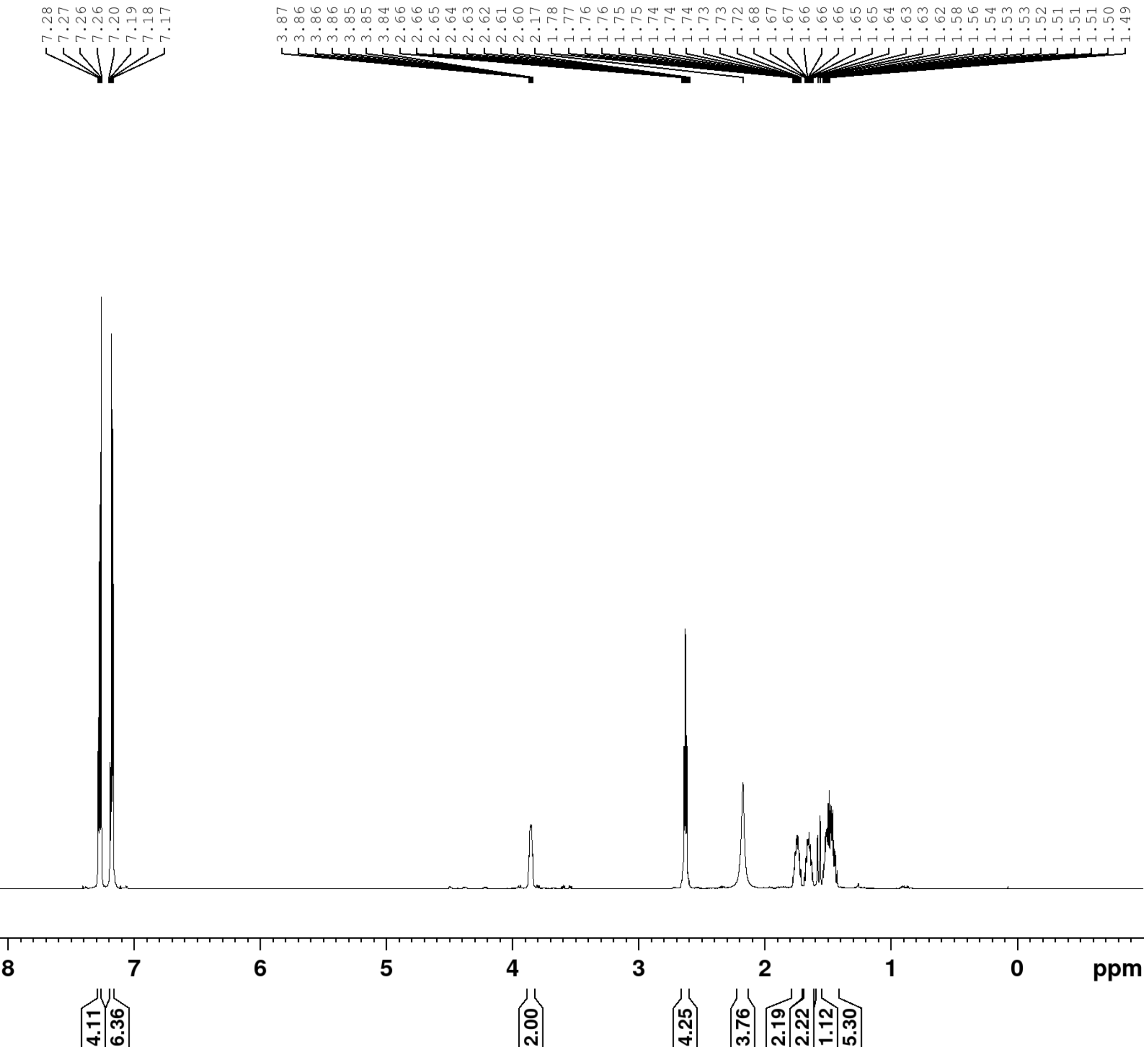

(*syn*)-2,10-dimethylundecane-5,7-diol (*meso*-1f)

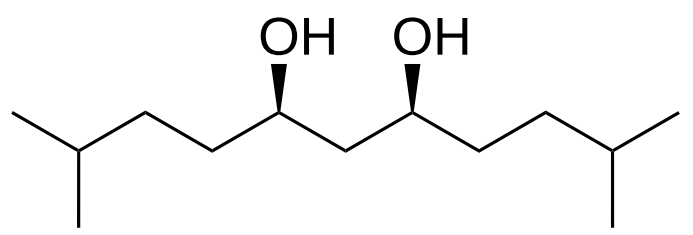

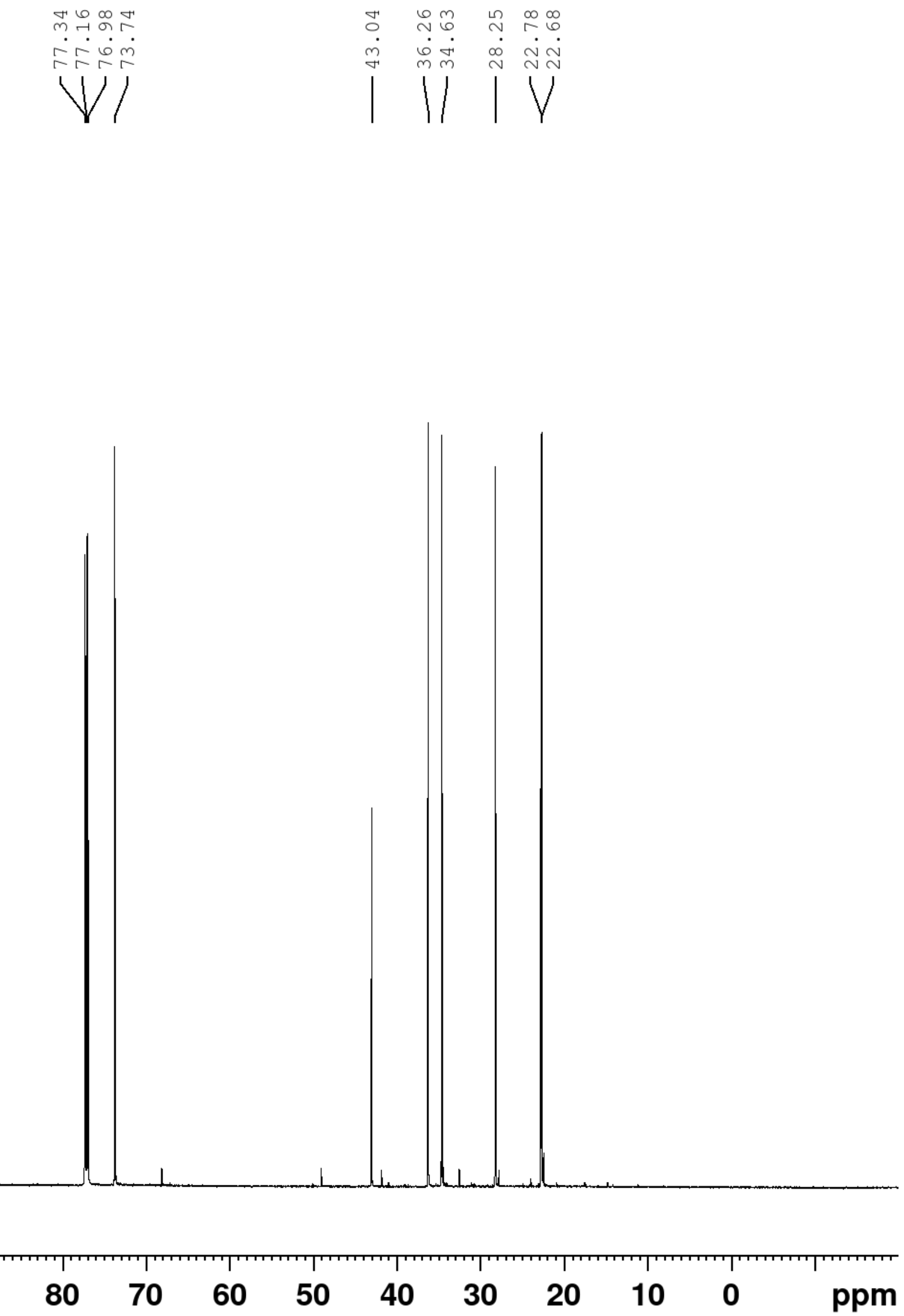

(*anti*)-2,10-dimethylundecane-5,7-diol (*rac*-1f)

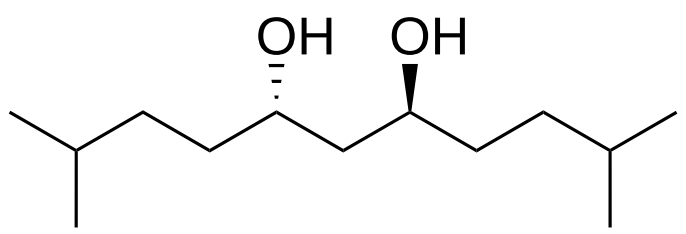

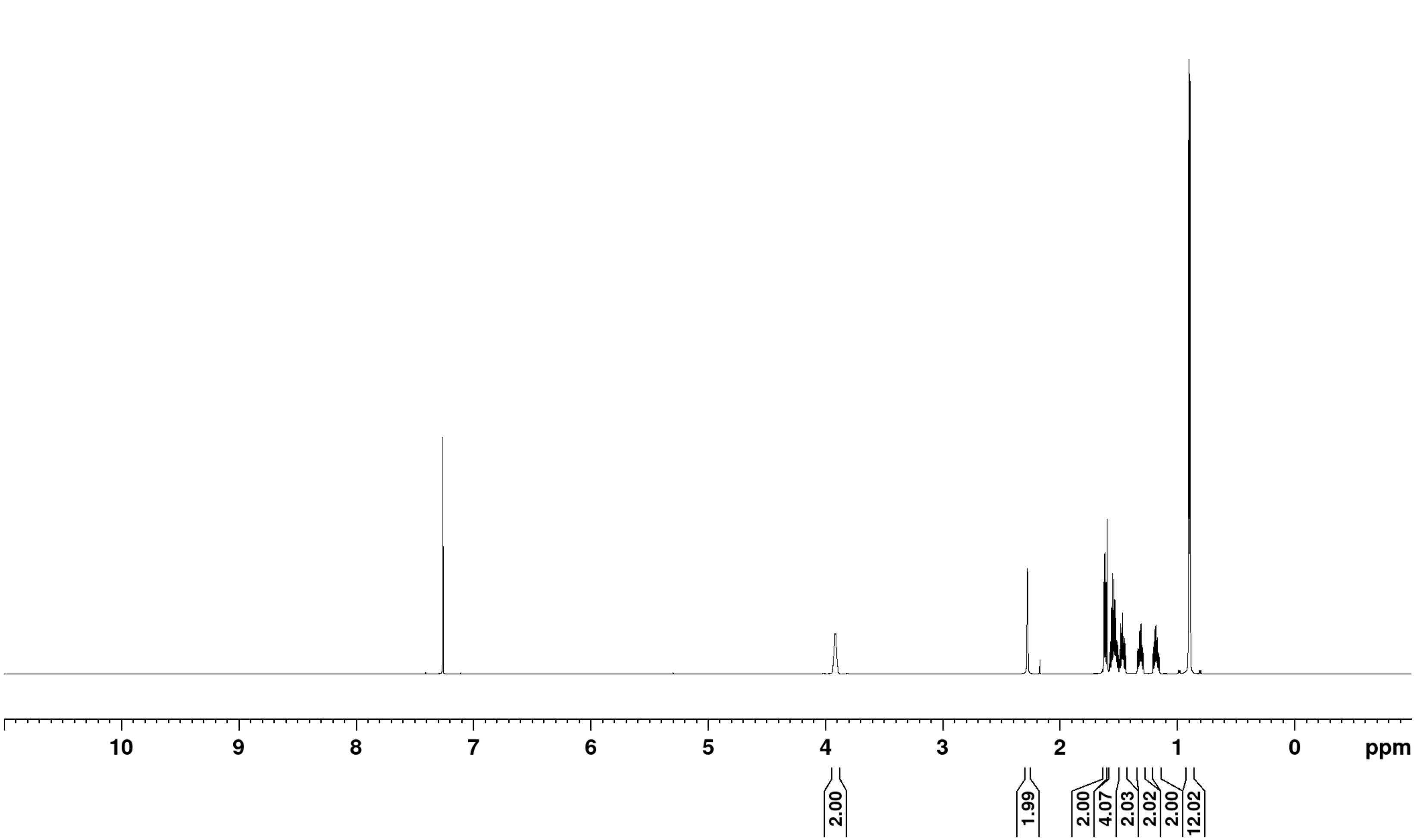

(*anti*)-2,10-dimethylundecane-5,7-diol (*rac*-1f)

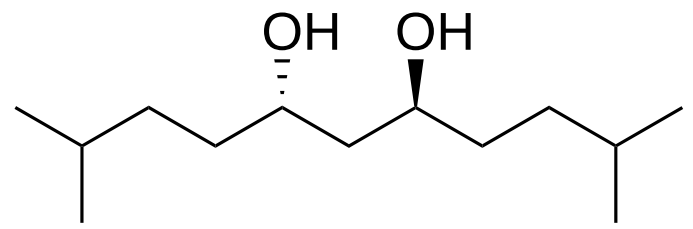

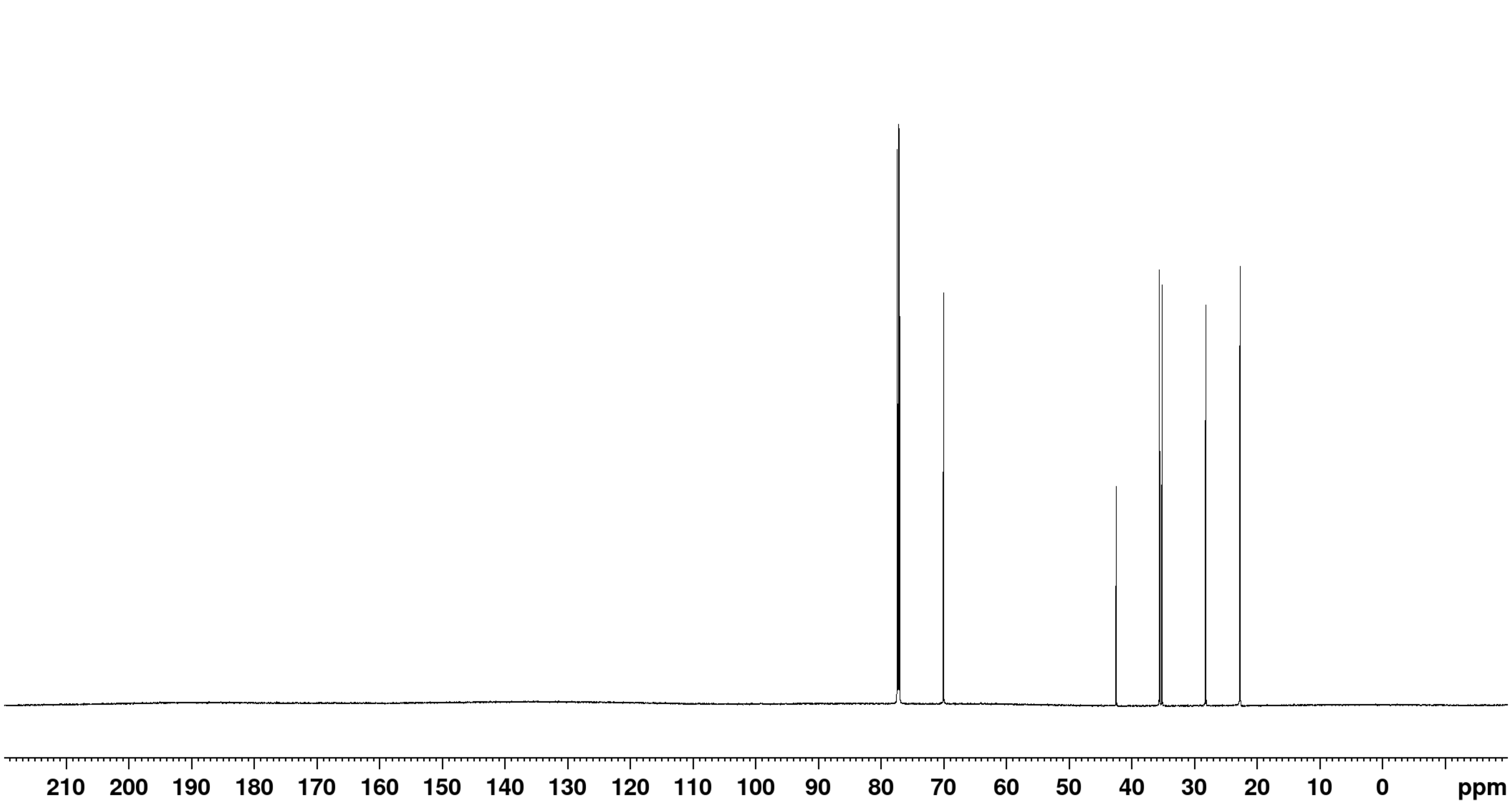

(*syn*)-1,7-dicyclobutylheptane-3,5-diol (*meso*-1g)

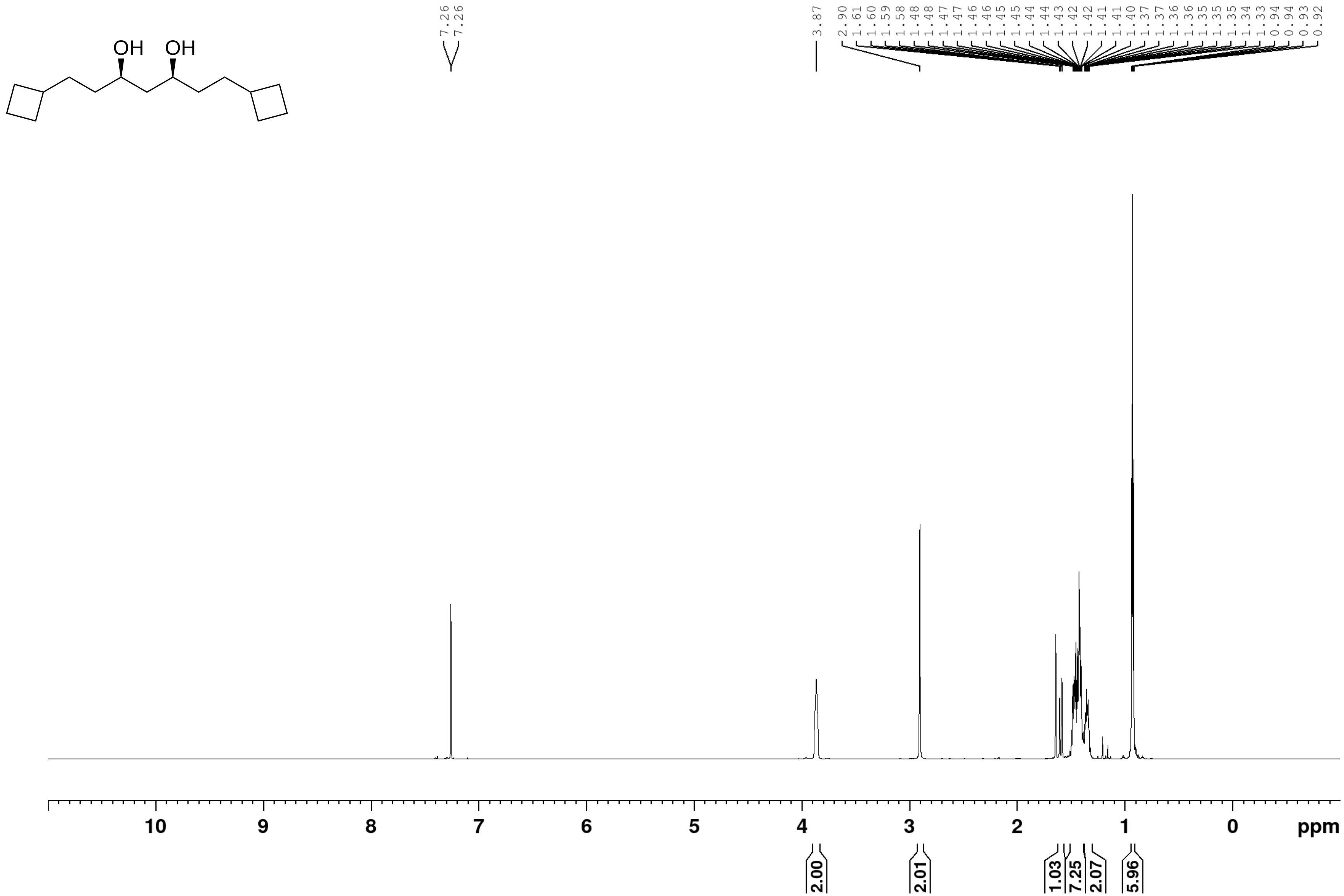

(*syn*)-1,7-dicyclobutylheptane-3,5-diol (*meso*-1g)

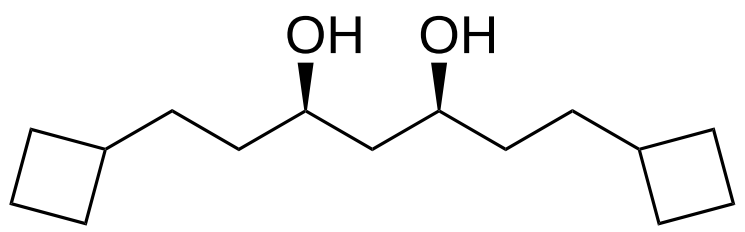

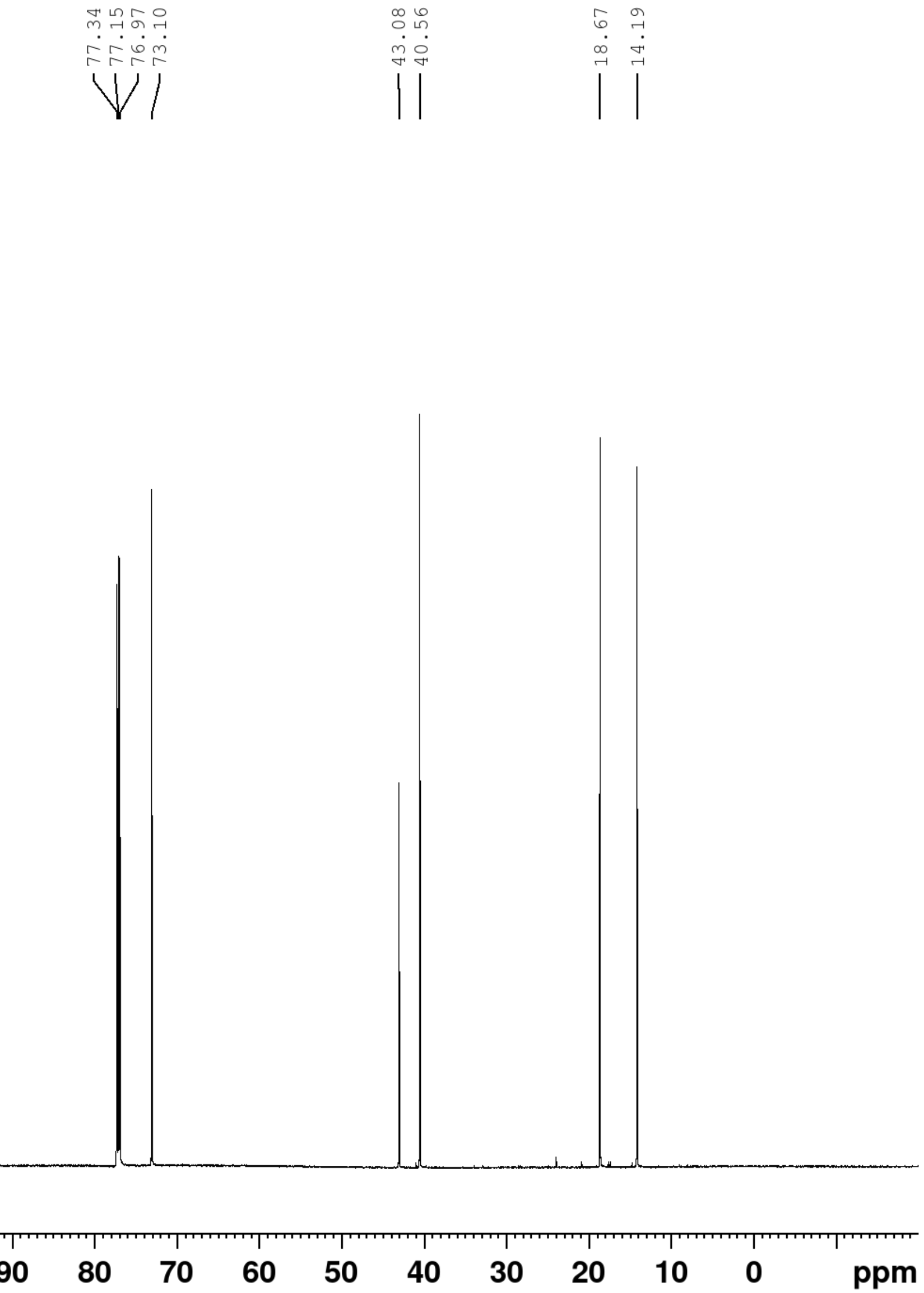

(*anti*)-1,7-dicyclobutylheptane-3,5-diol (*rac*-1g)

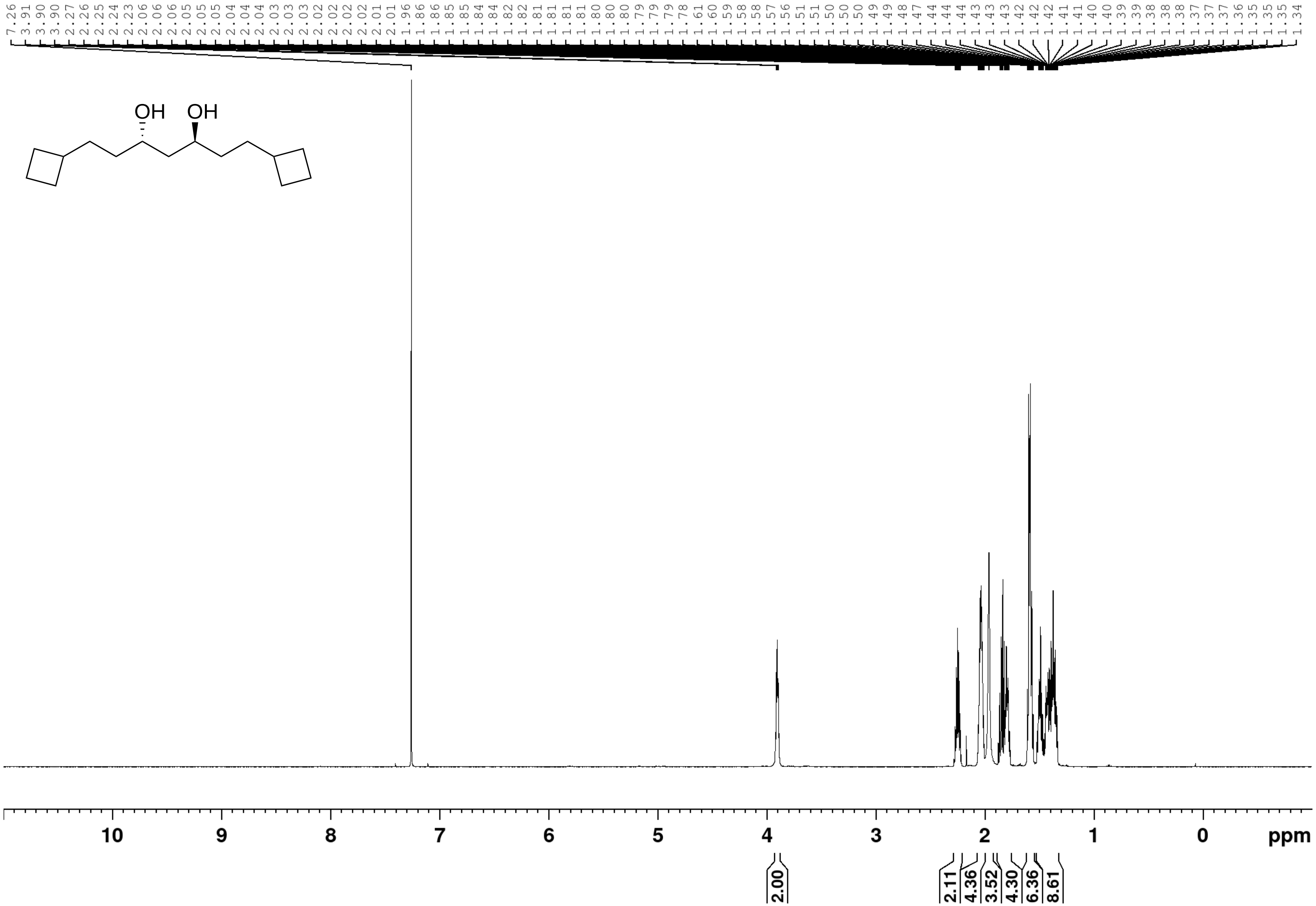

(*anti*)-1,7-dicyclobutylheptane-3,5-diol (*rac*-1g)

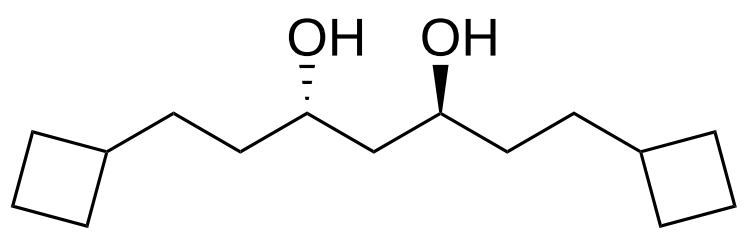

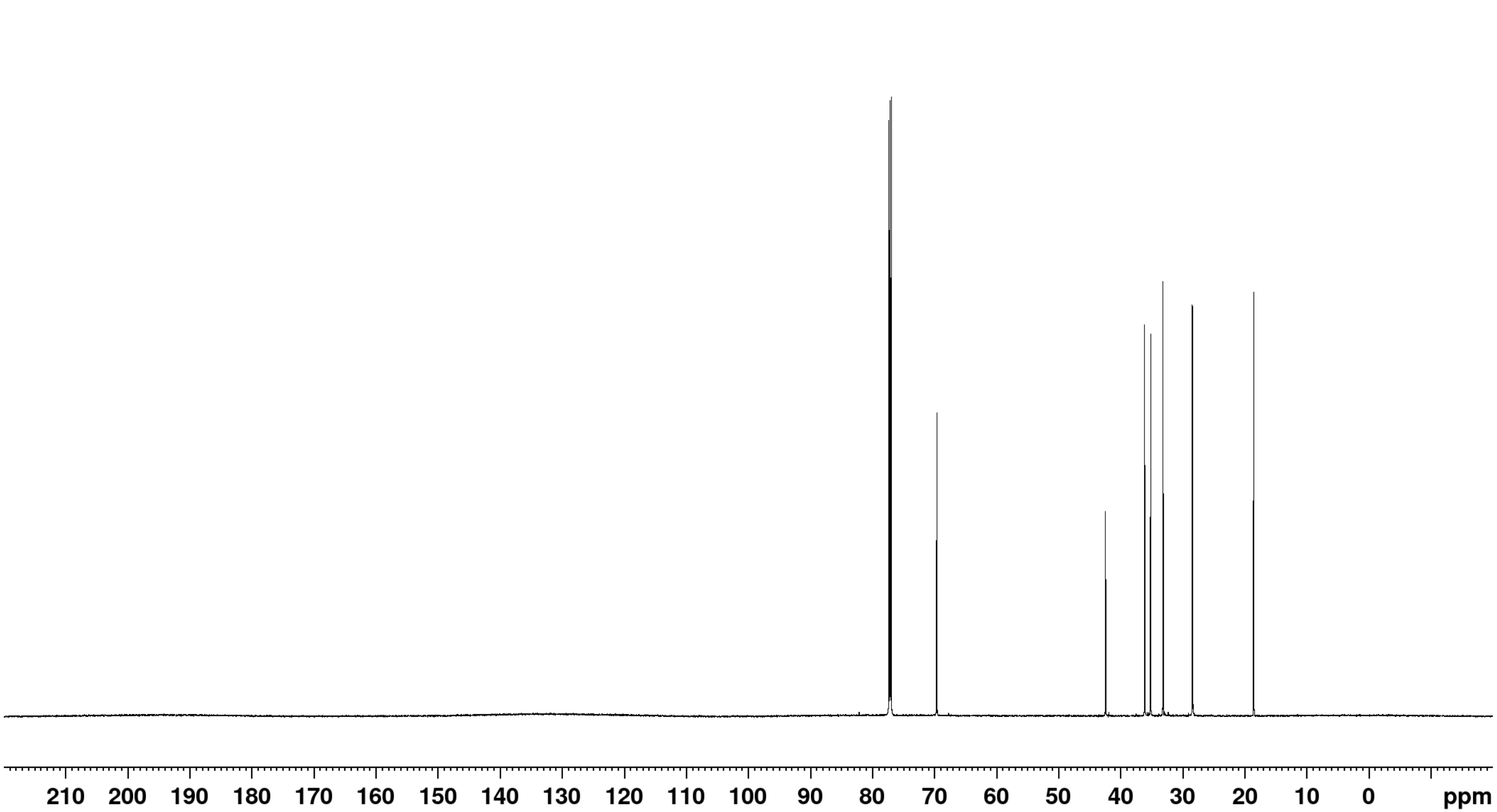

(*syn*)-1,5-dicyclohexylpentane-2,4-diol (*meso*-1h)

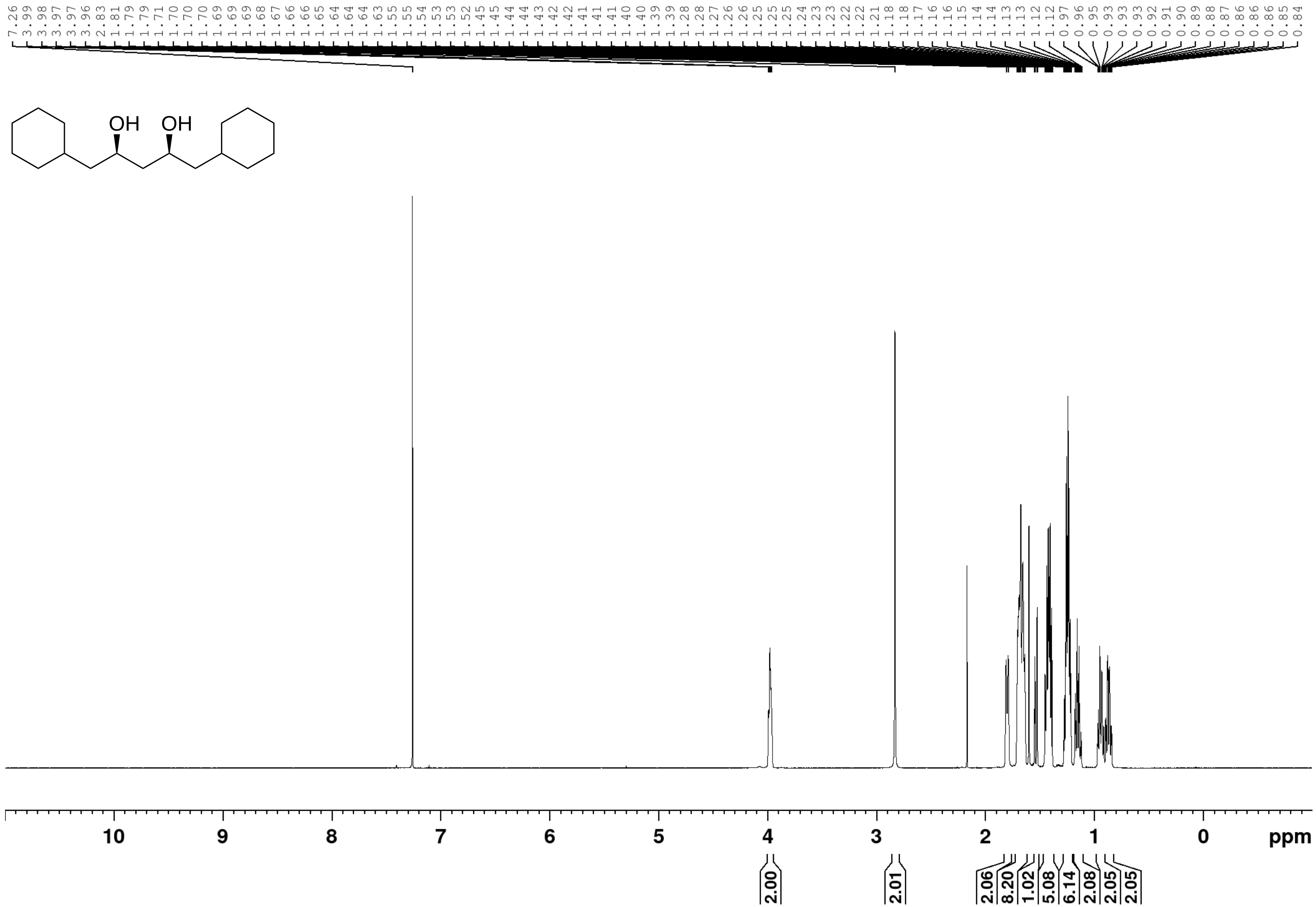

(*syn*)-1,5-dicyclohexylpentane-2,4-diol (*meso*-1h)

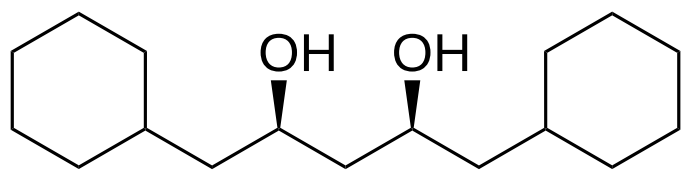

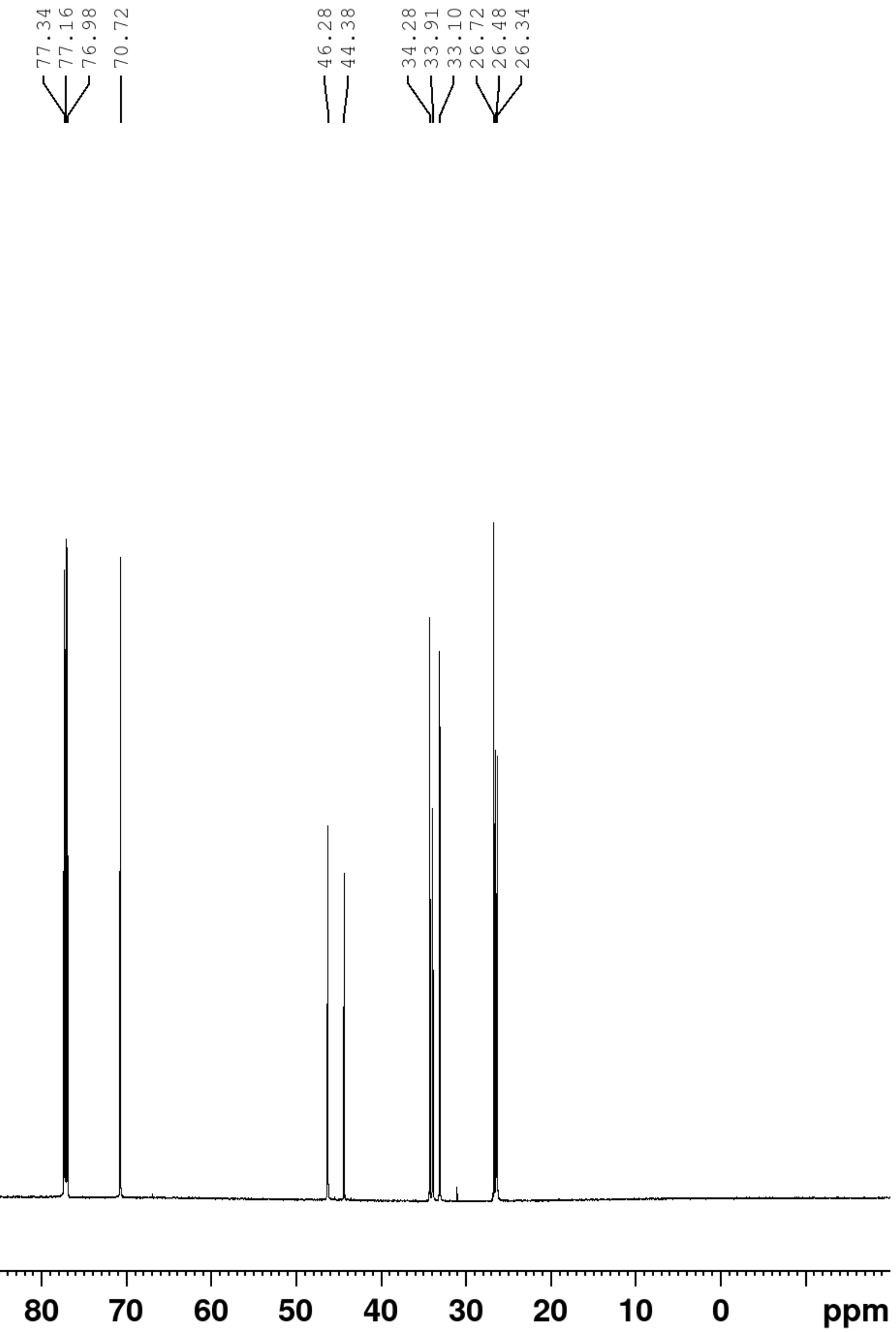

(*anti*)-1,5-dicyclohexylpentane-2,4-diol (*rac*-1h)

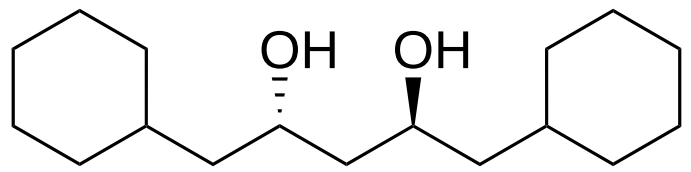

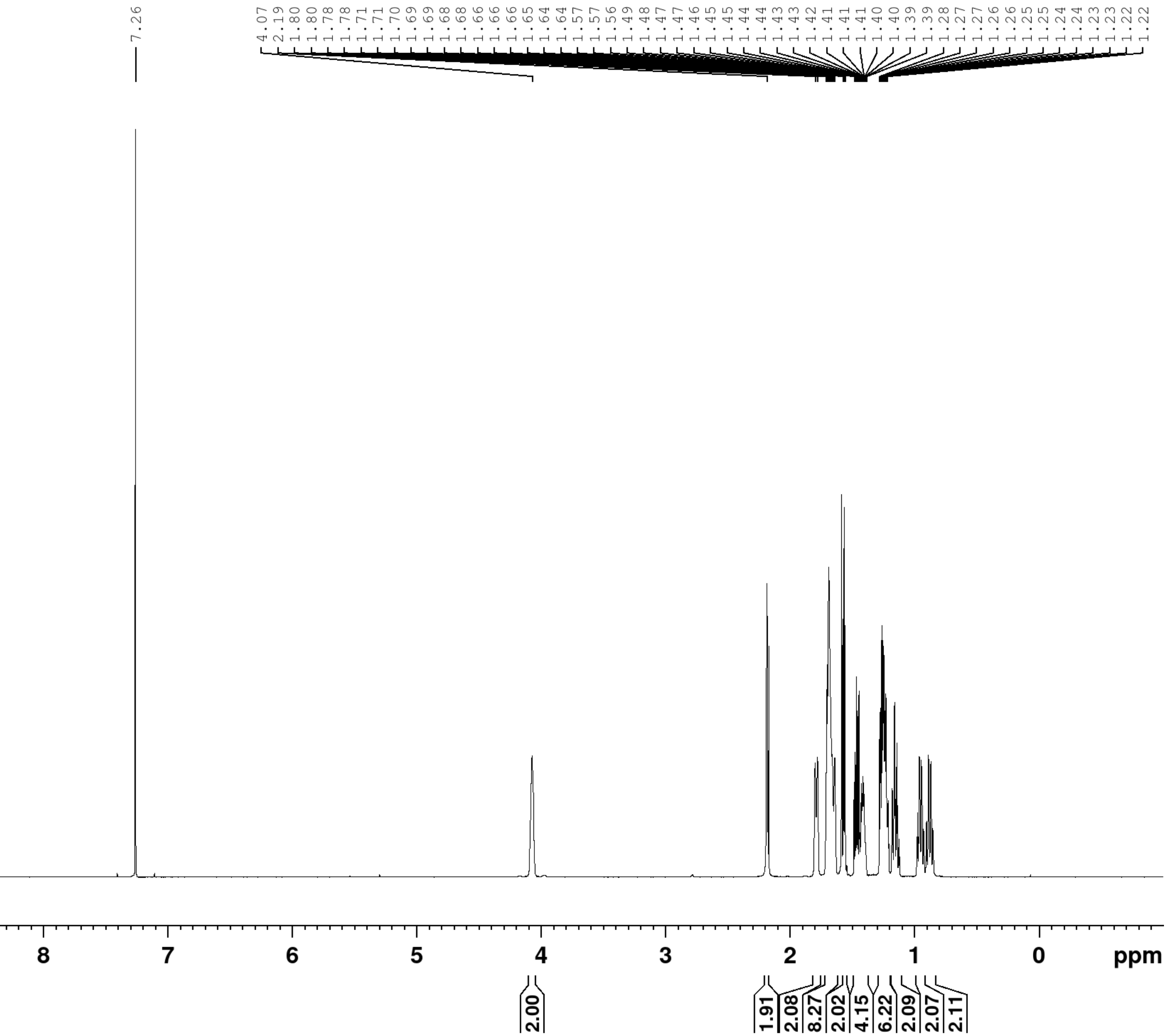

(*anti*)-1,5-dicyclohexylpentane-2,4-diol (*rac*-1h)

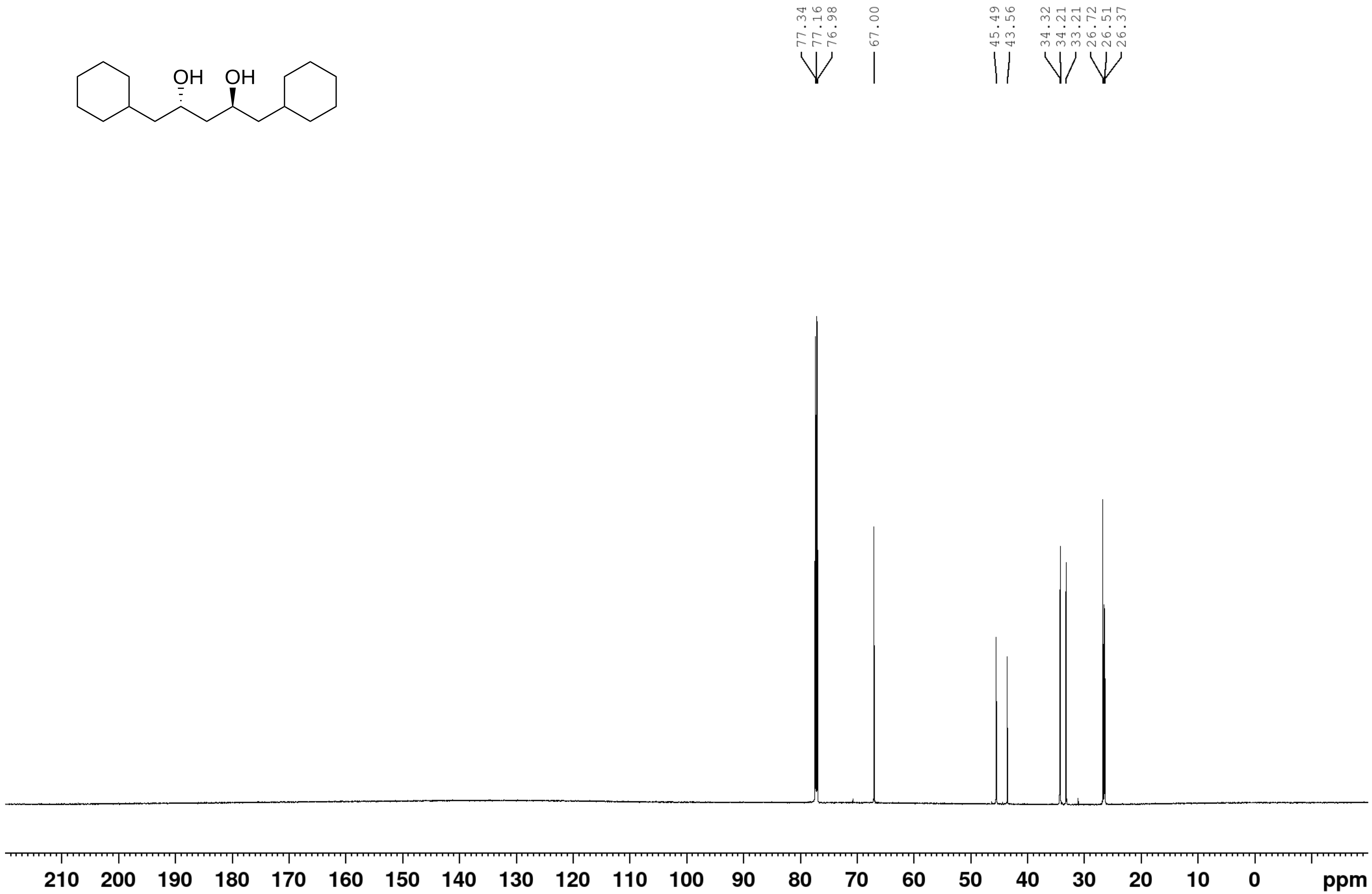

(*syn*)-1,7-dicyclohexylheptane-3,5-diol (*meso*-1i)

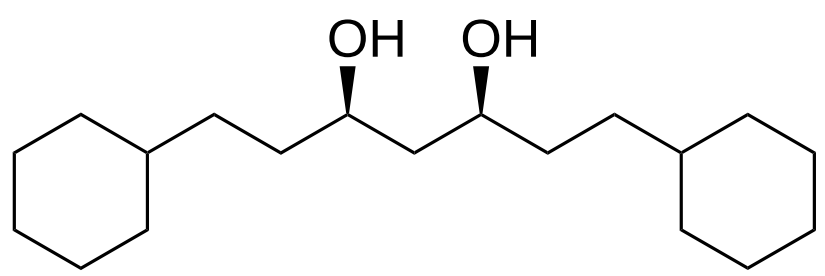

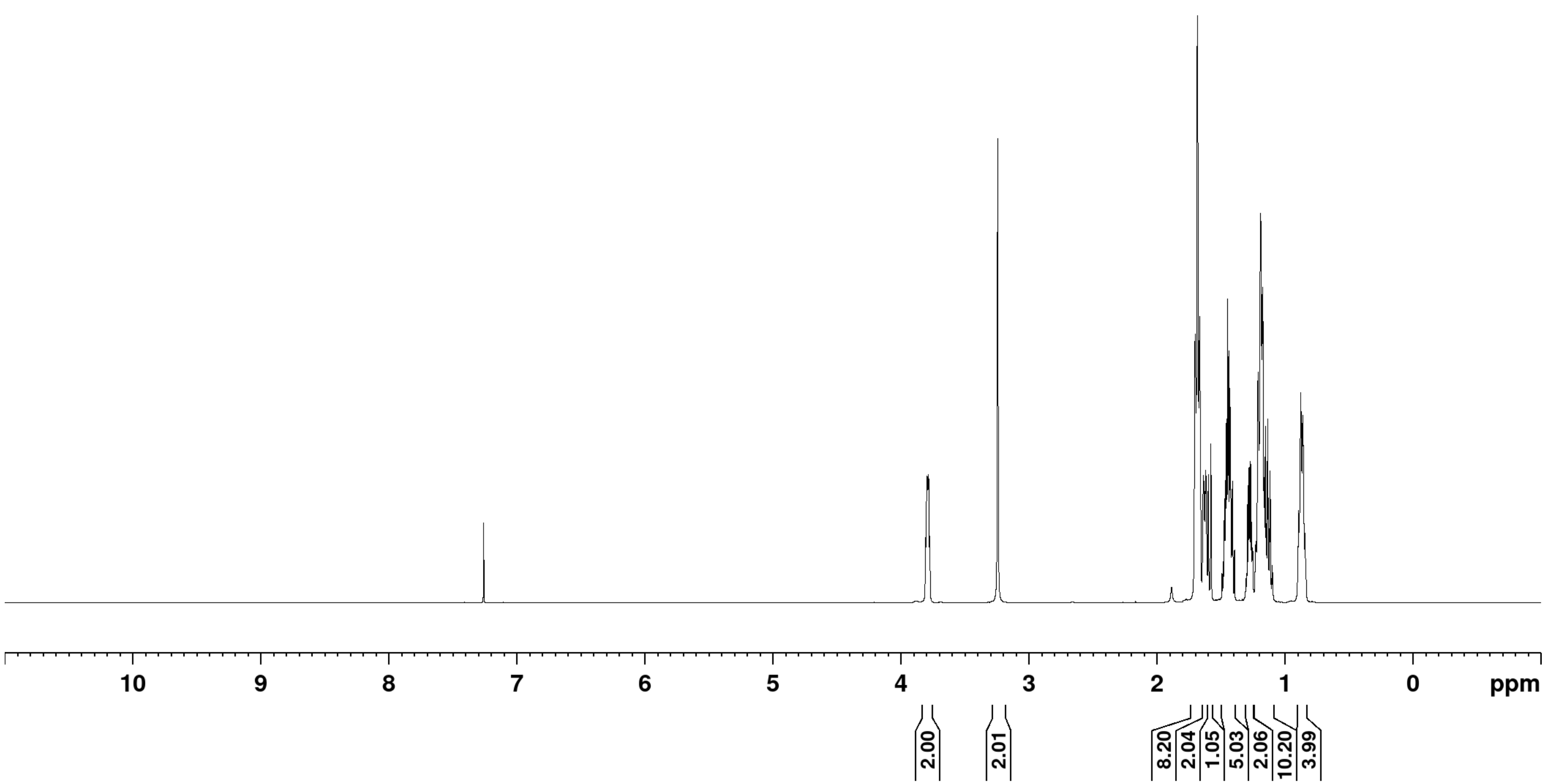

(*syn*)-1,7-dicyclohexylheptane-3,5-diol (*meso*-1i)

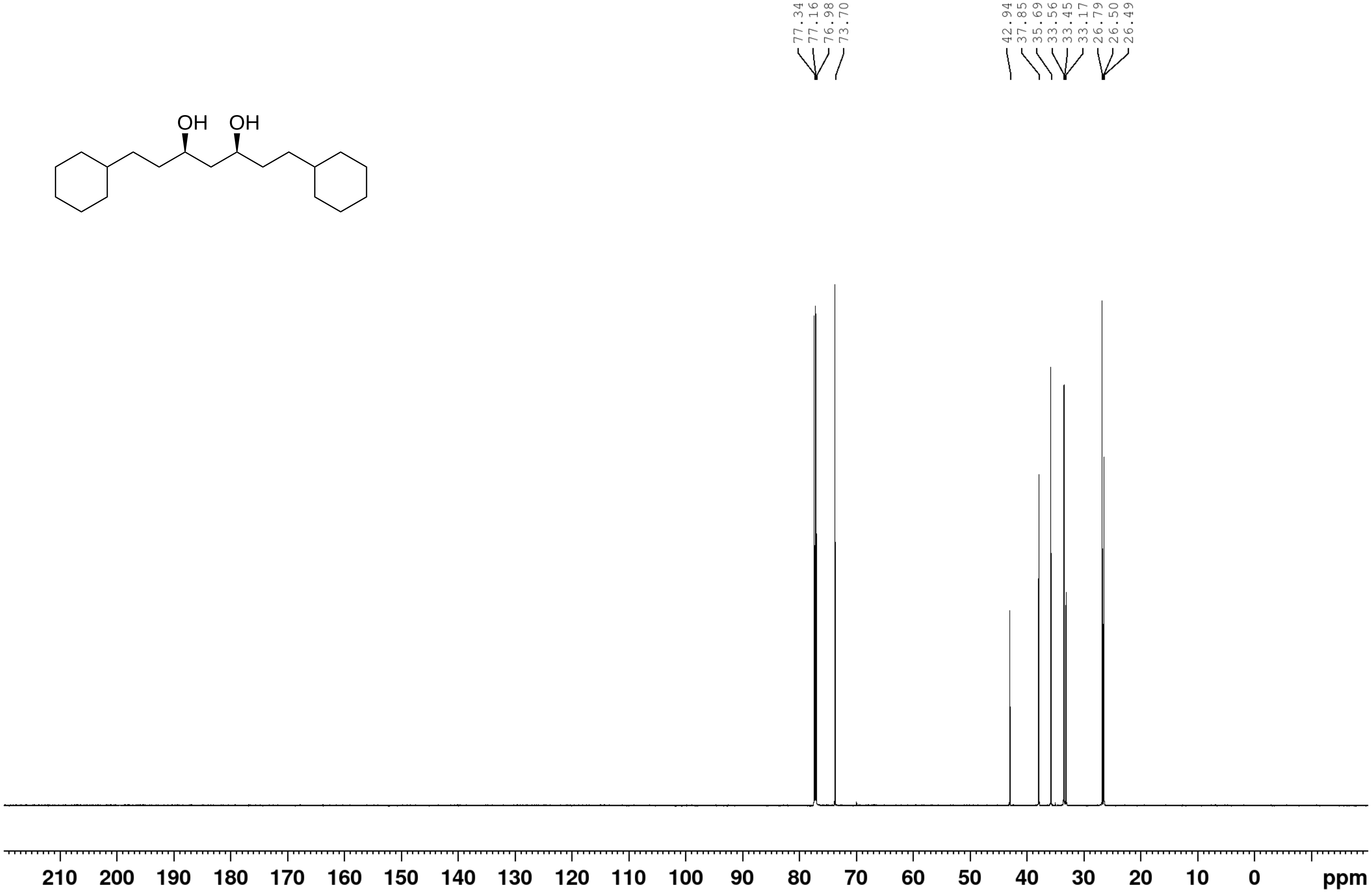

(*anti*)-1,7-dicyclohexylheptane-3,5-diol (*rac*-1i)

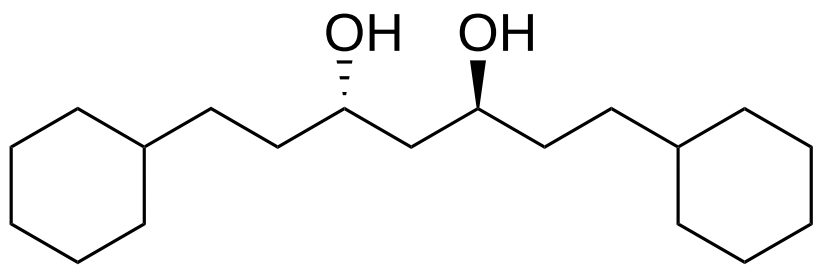

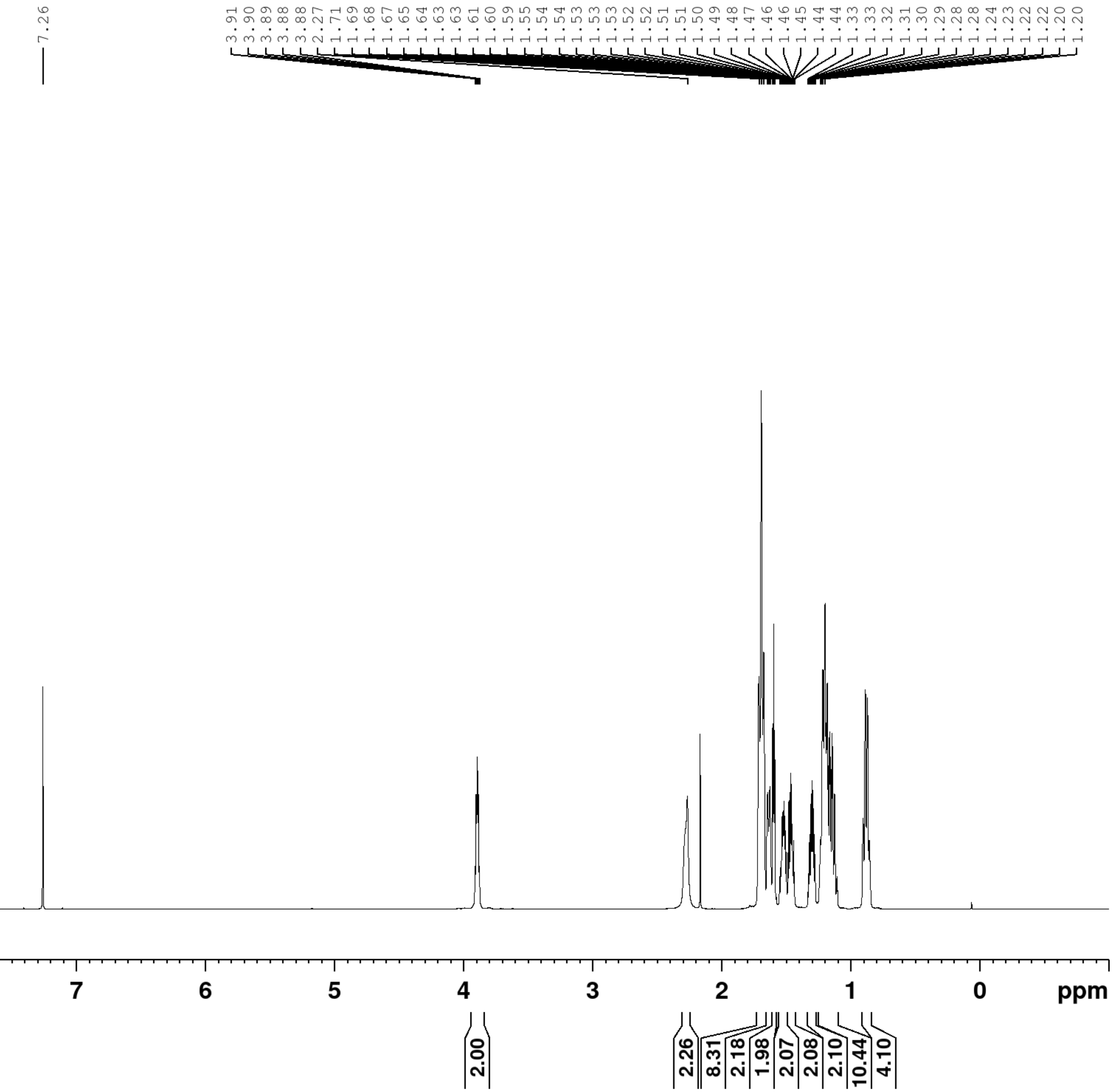

(*anti*)-1,7-dicyclohexylheptane-3,5-diol (*rac*-1i)

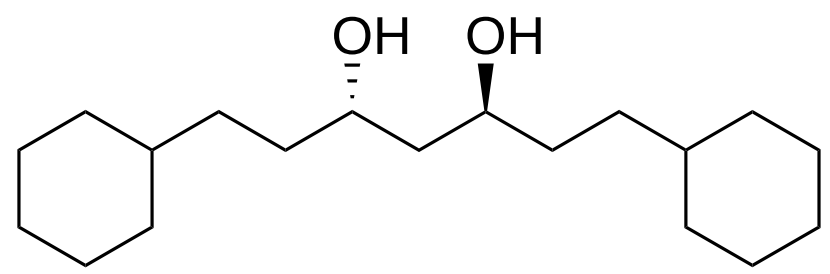

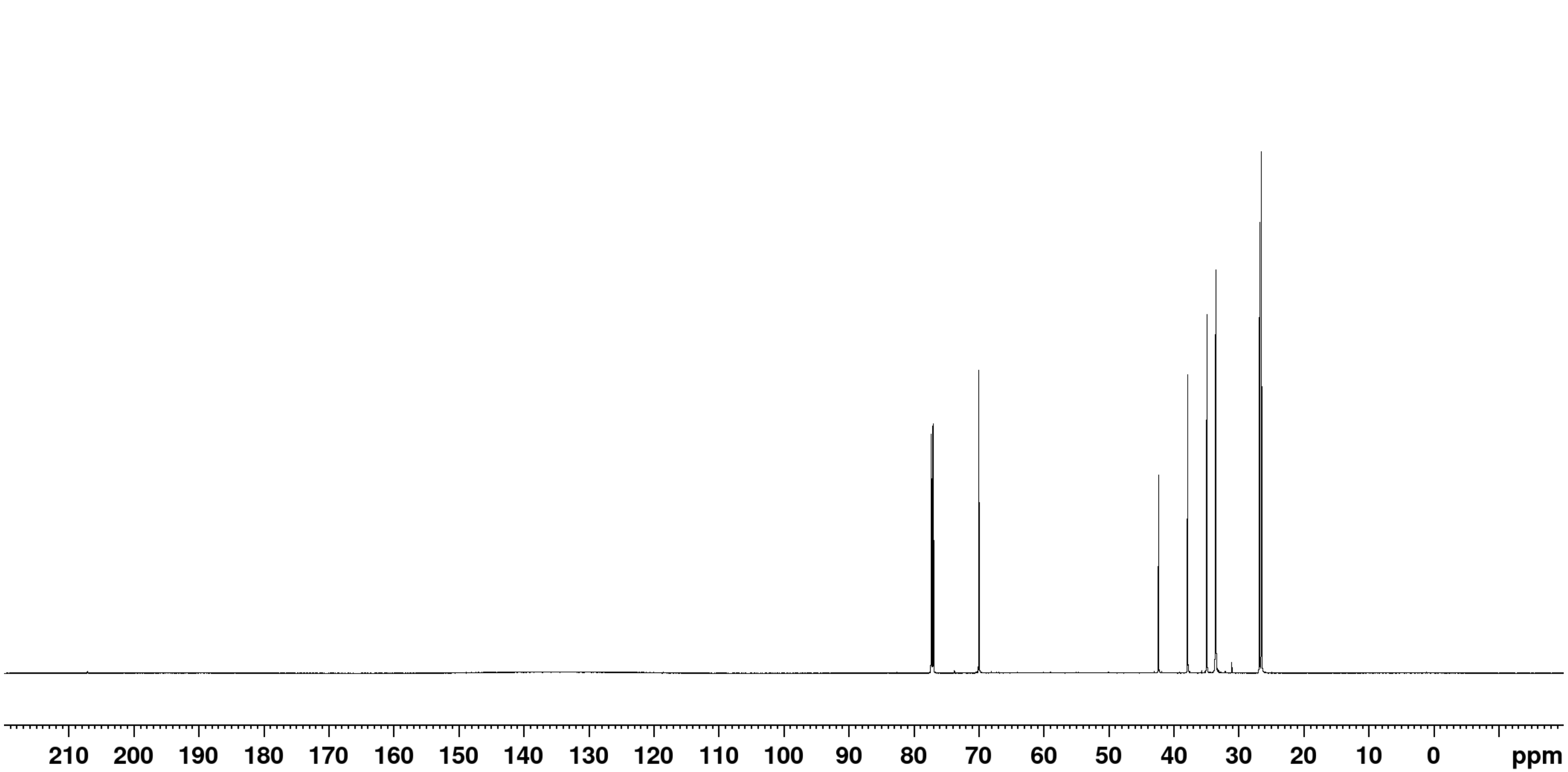

(*syn*)-1,9-dicyclohexylnonane-4,6-diol (*meso*-1j)

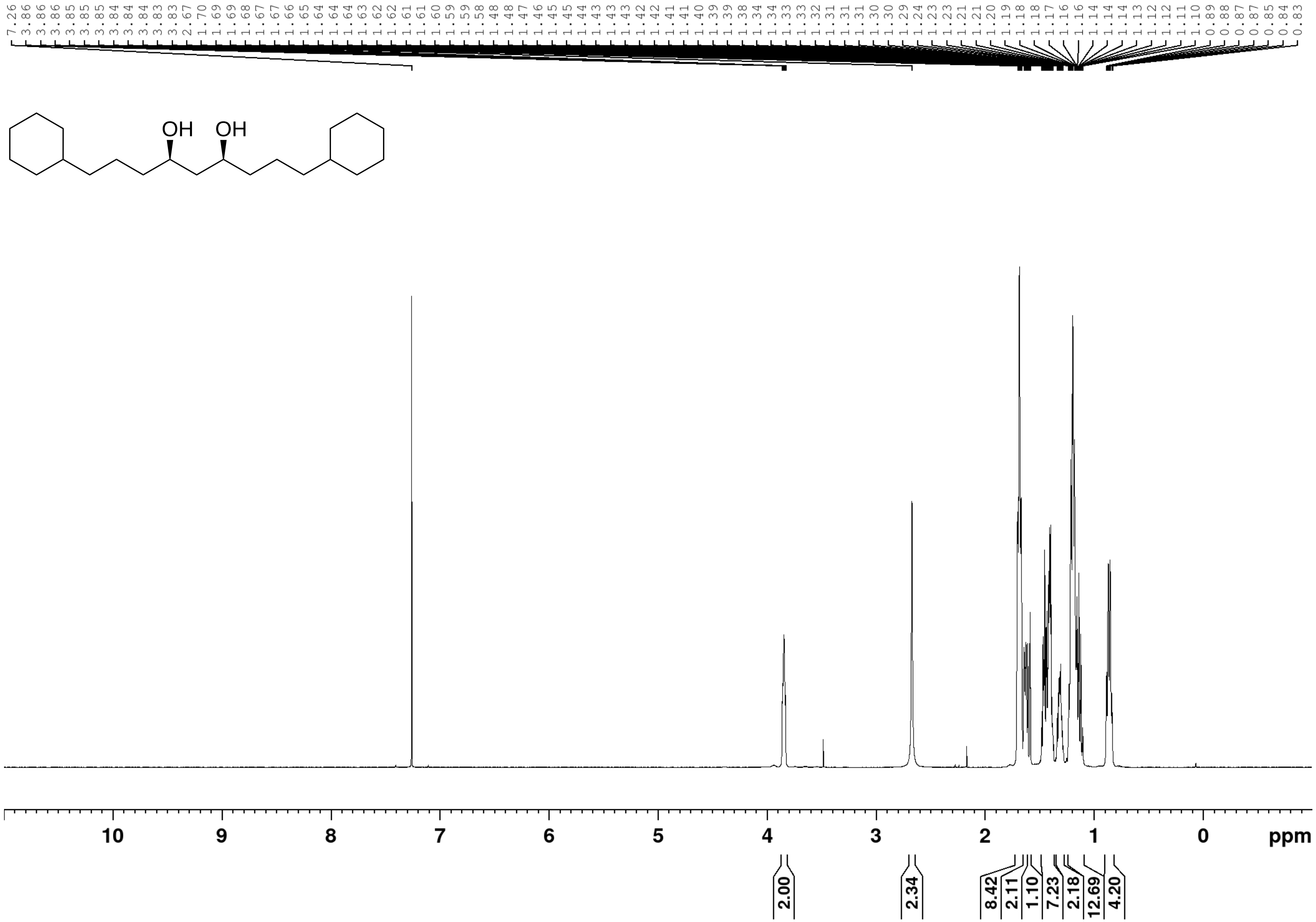

(*syn*)-1,9-dicyclohexylnonane-4,6-diol (*meso*-1j)

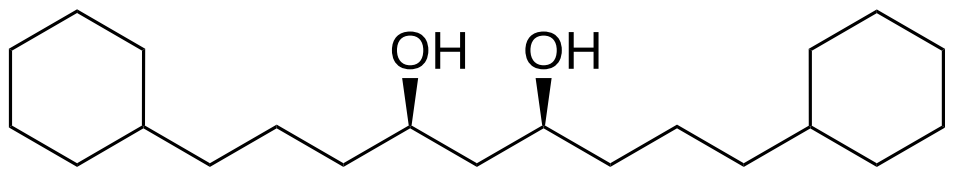

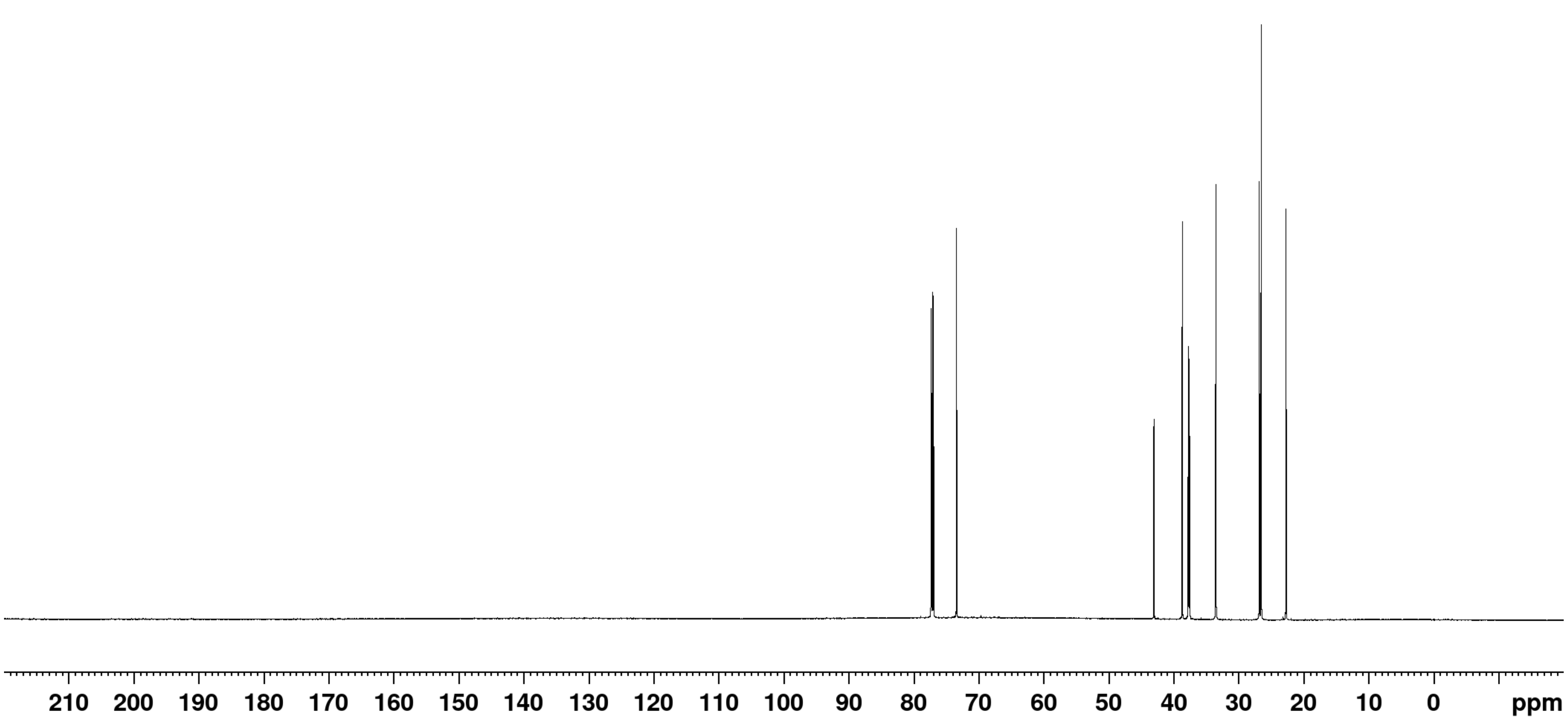

(*anti*)-1,9-dicyclohexylnonane-4,6-diol (*rac*-1j)

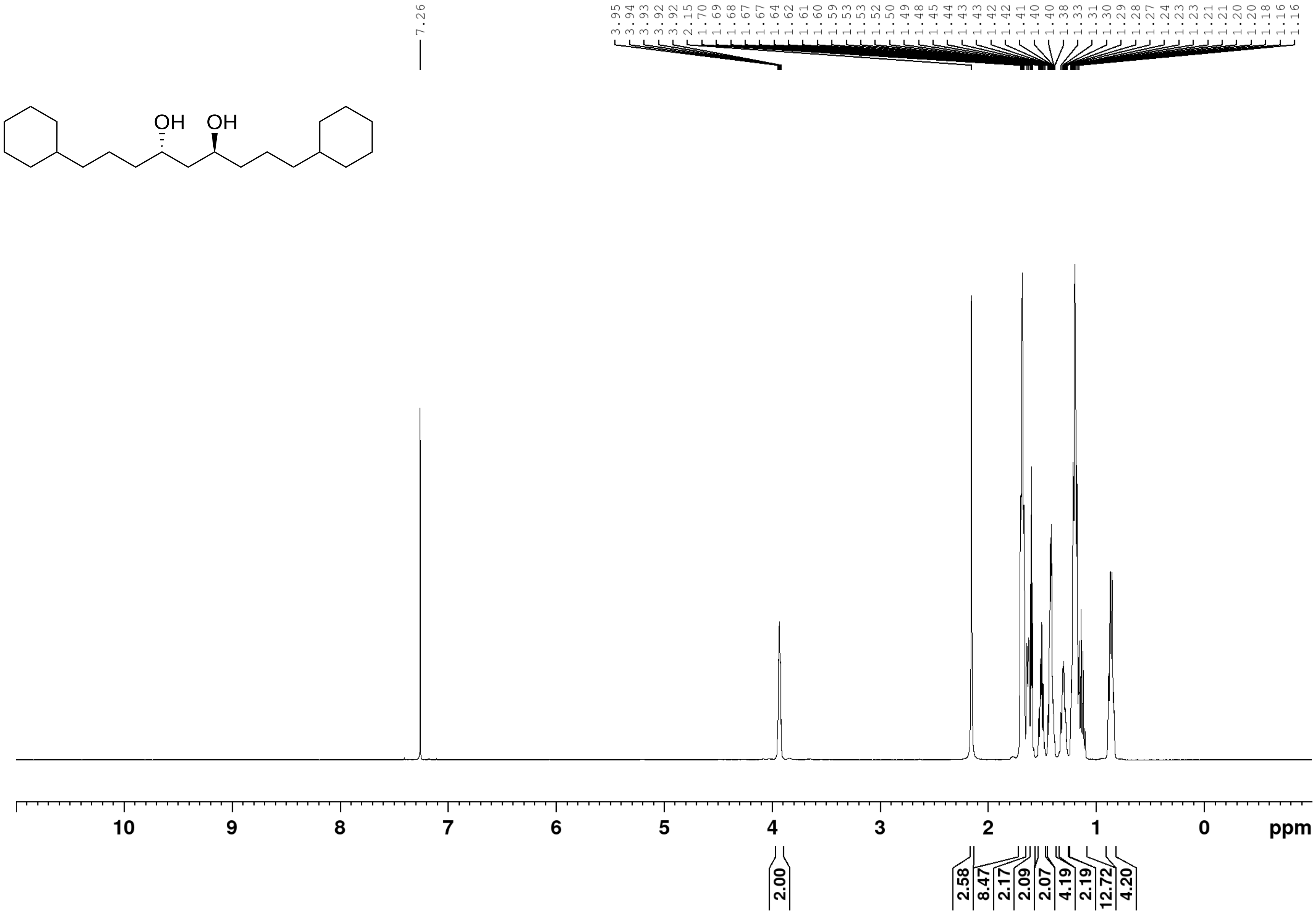

(*anti*)-1,9-dicyclohexylnonane-4,6-diol (*rac*-1j)

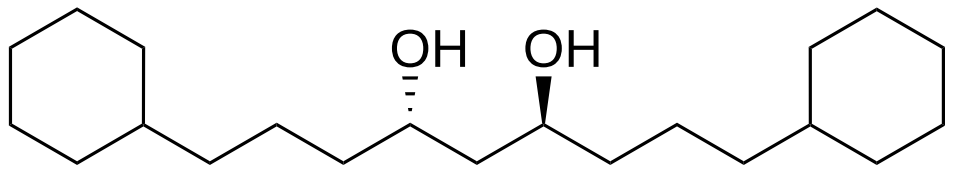

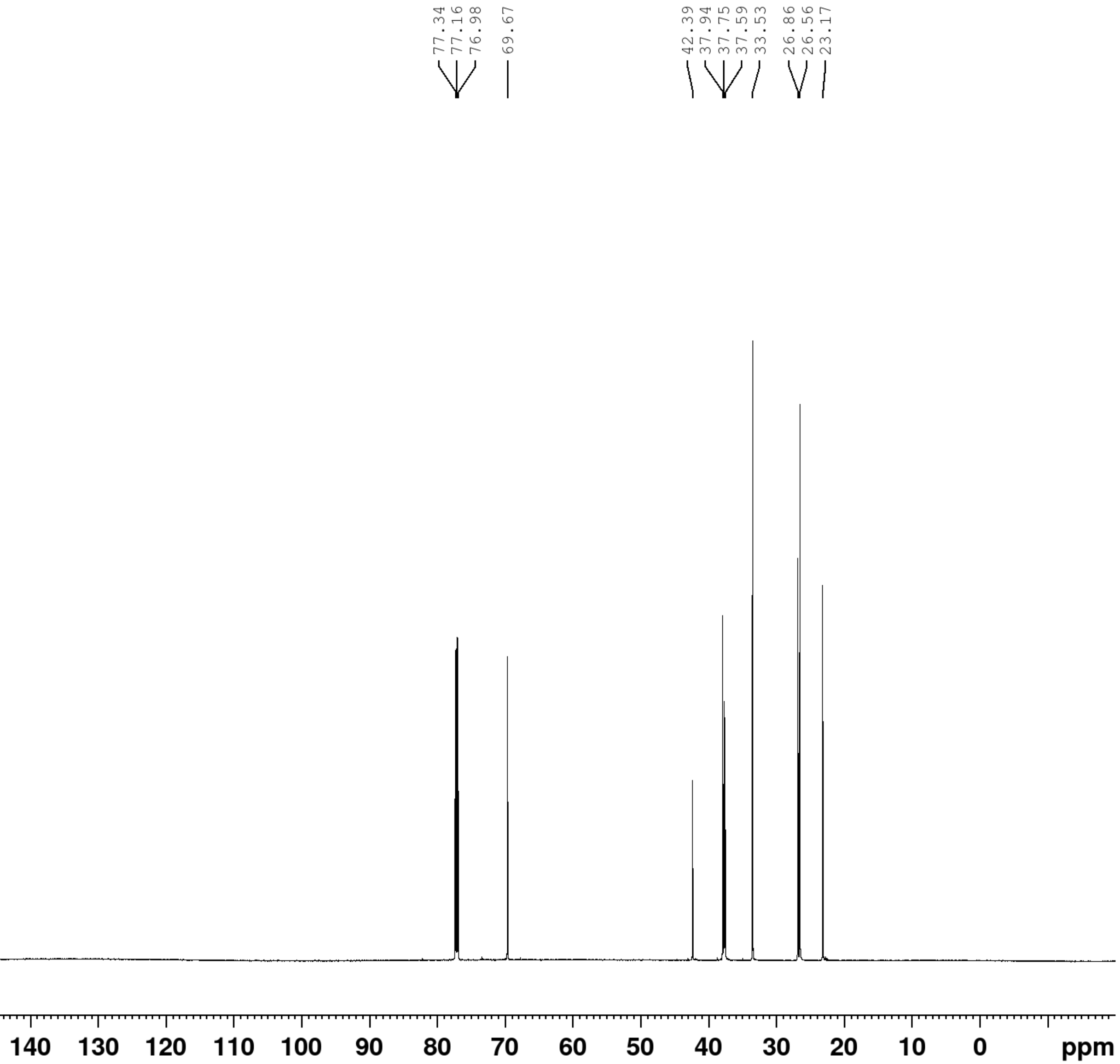

(*syn*)-1,7-diphenylheptane-3,5-diol (*meso*-1k)

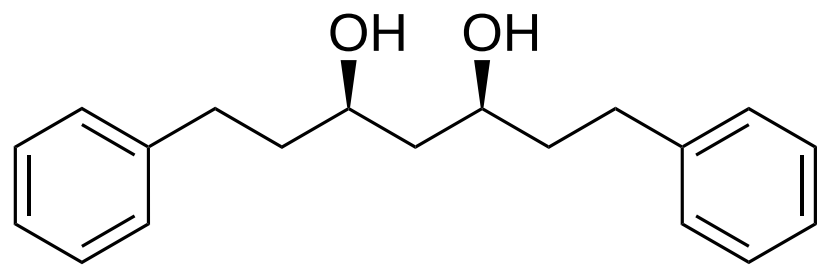

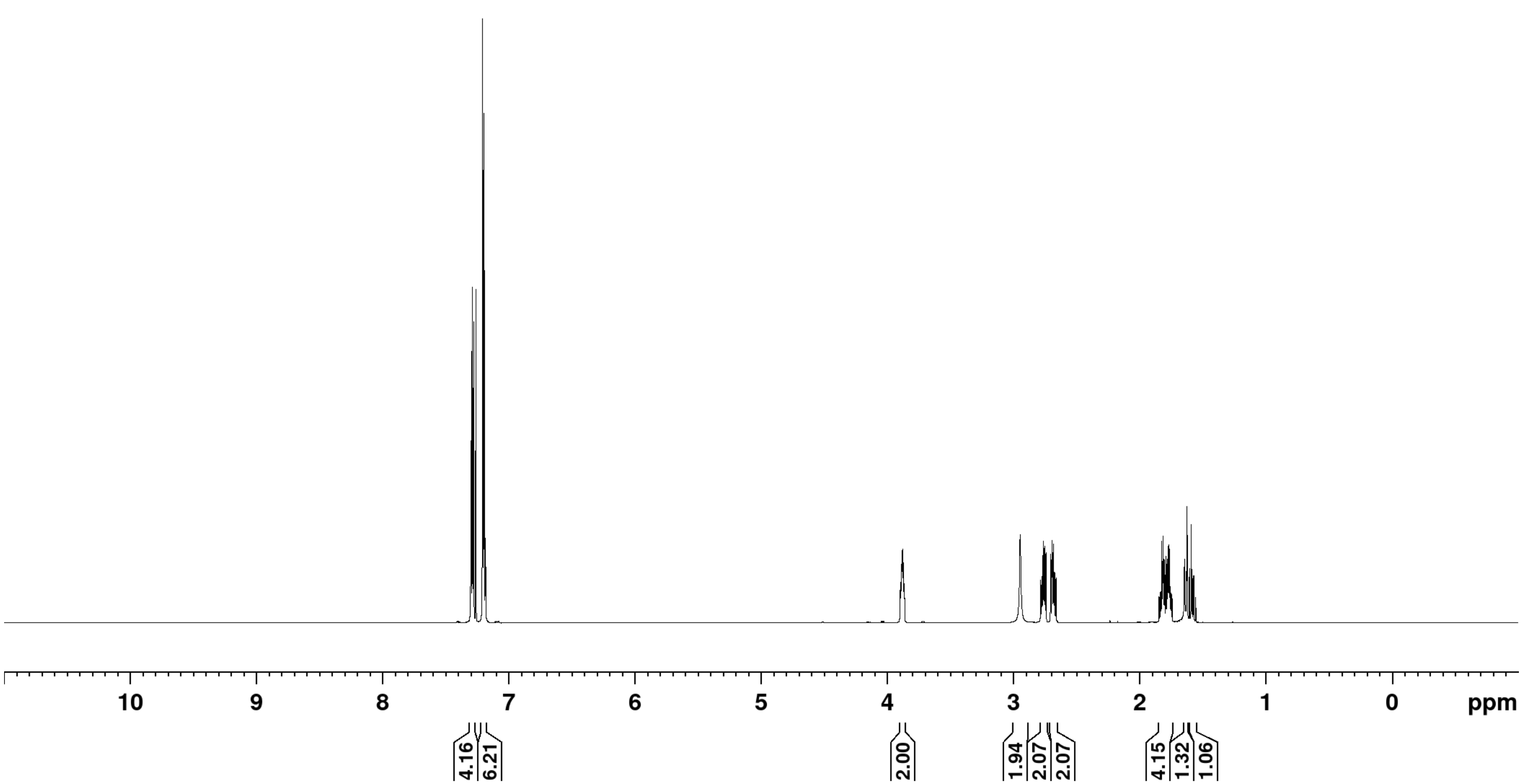

(*syn*)-1,7-diphenylheptane-3,5-diol (*meso*-1k)

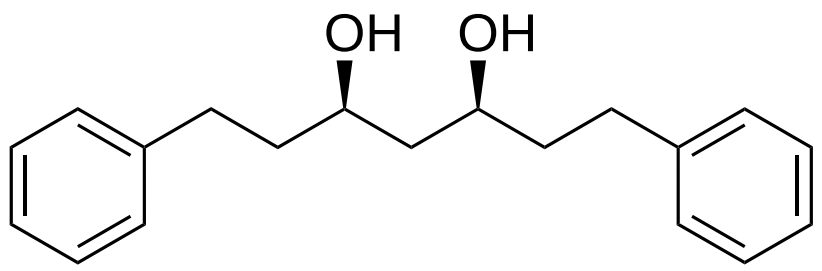

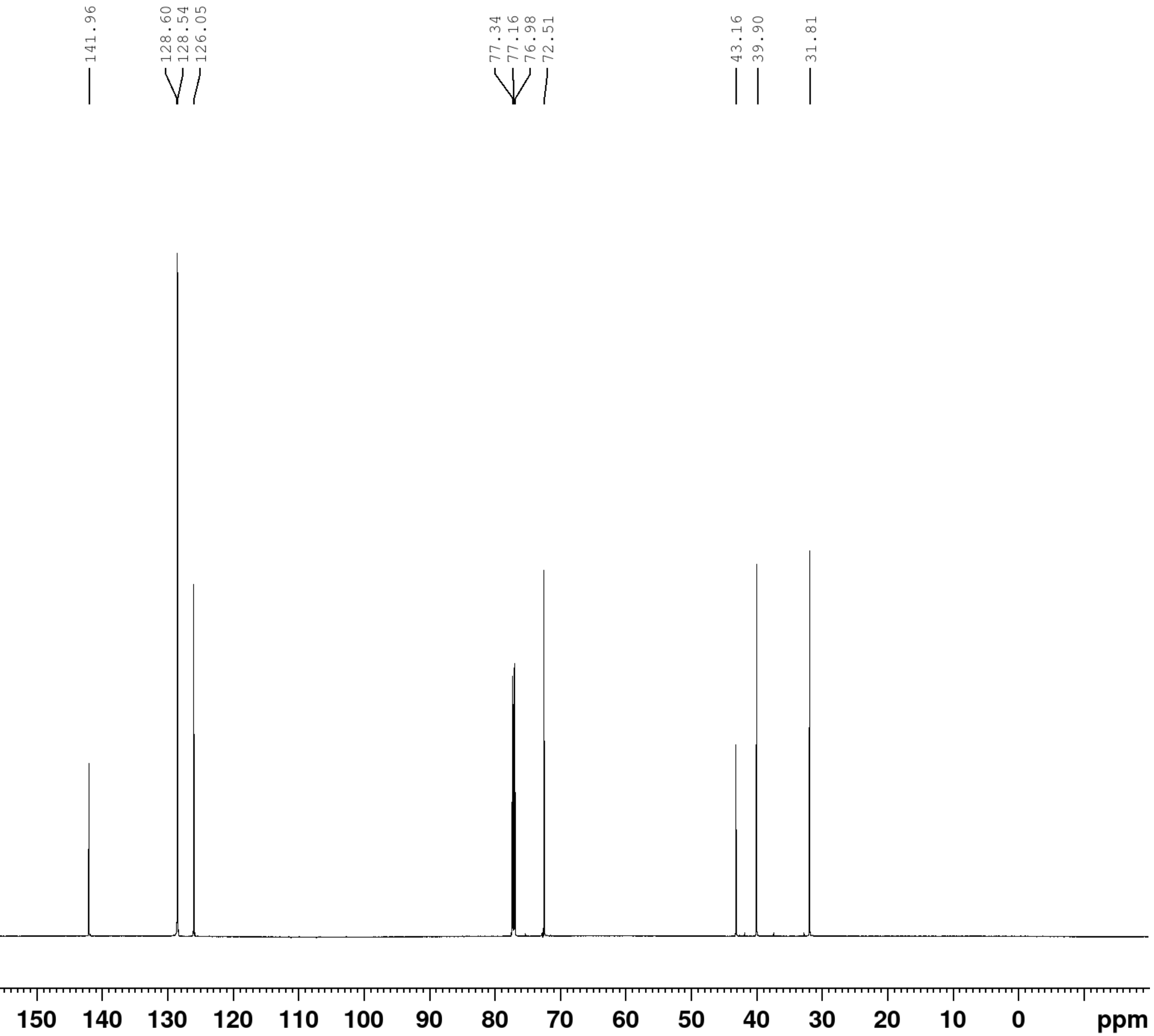

(*anti*)- 1,7-diphenylheptane-3,5-diol (*rac*-1k)

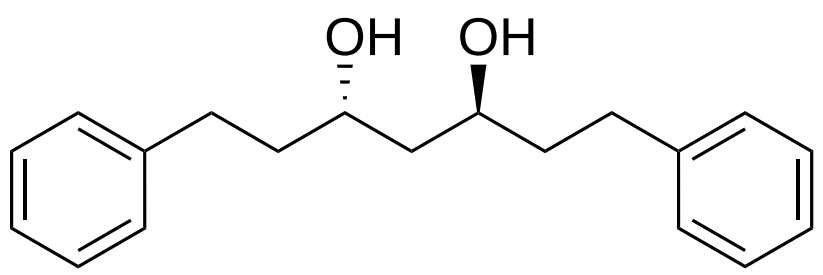

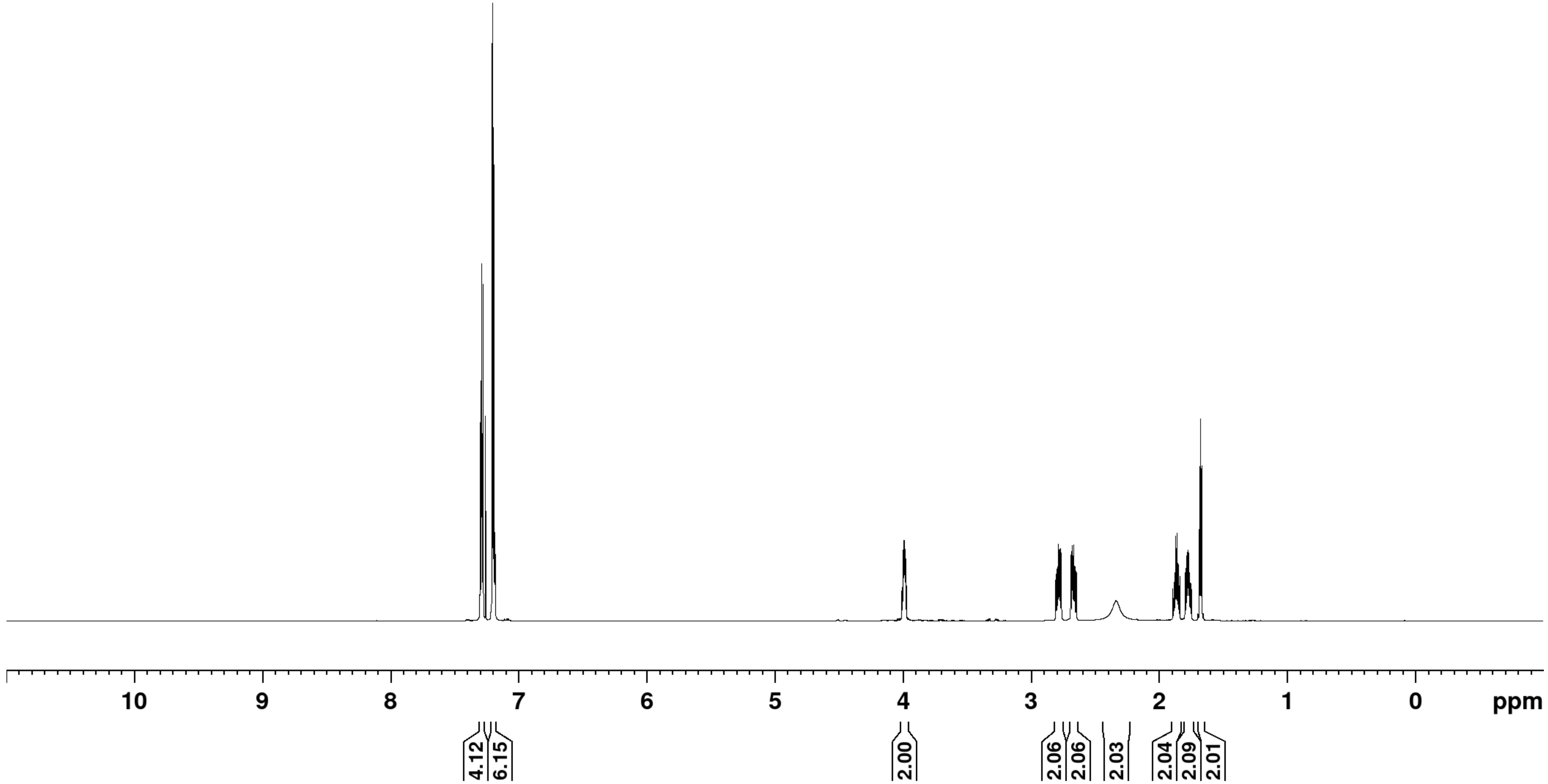

(*anti*)- 1,7-diphenylheptane-3,5-diol (*rac*-1k)

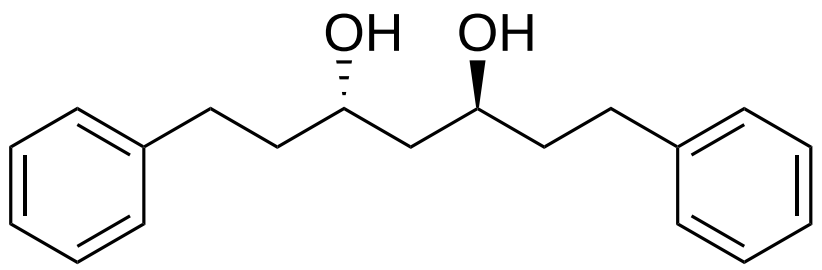

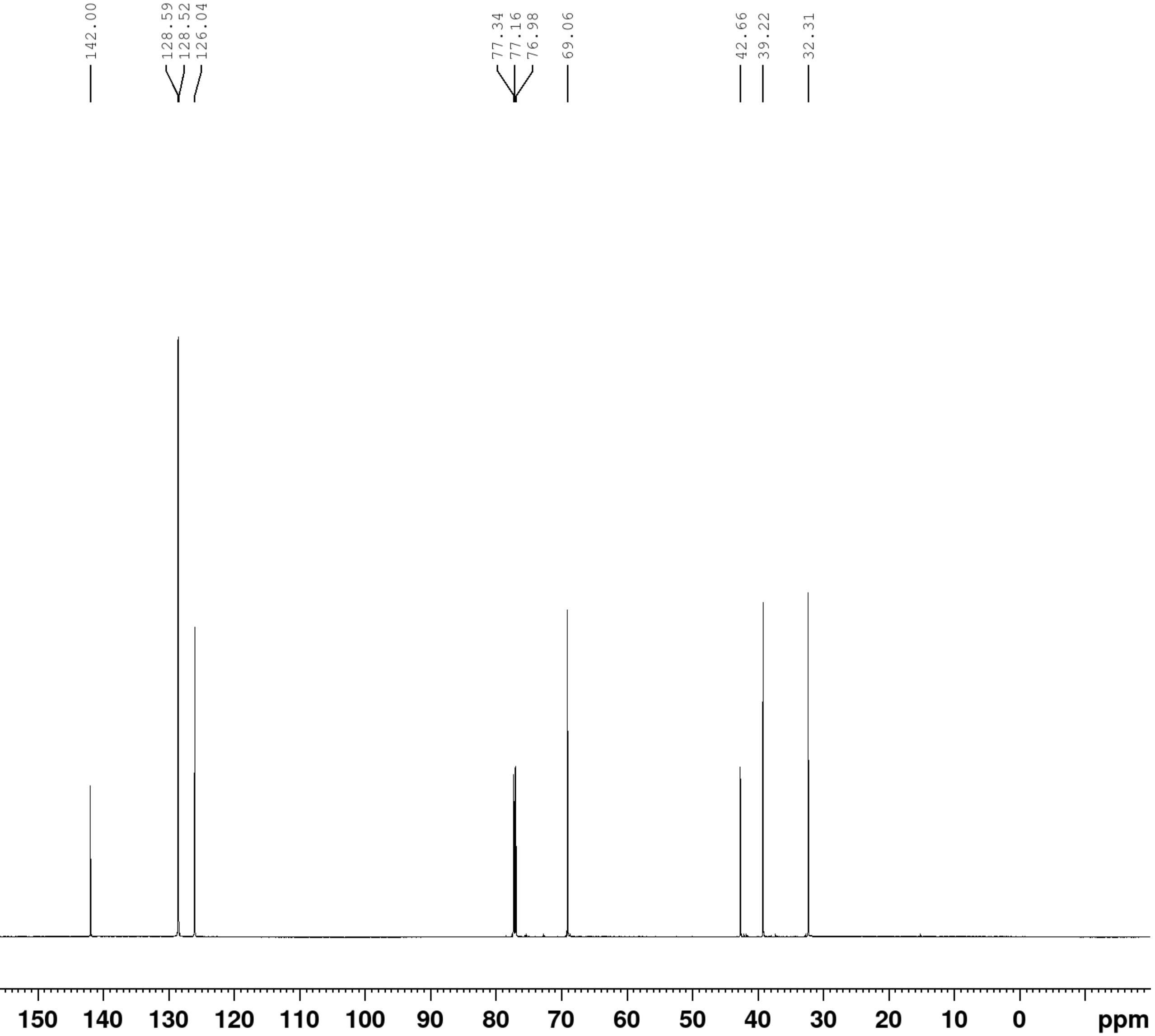

(*syn*)-1,9-diphenylnonane-4,6-diol (*meso*-1l)

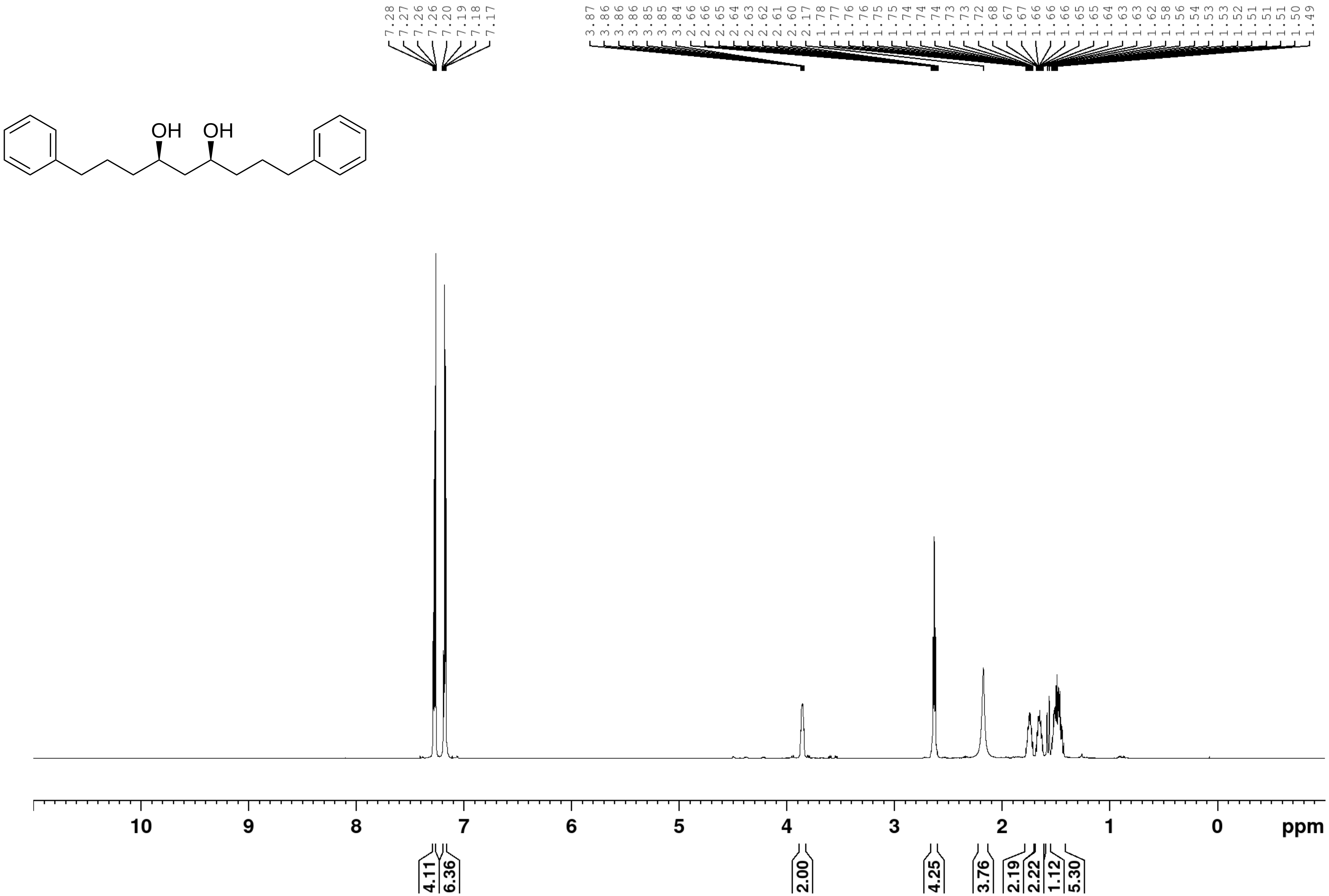

(*syn*)-1,9-diphenylnonane-4,6-diol (*meso*-1l)

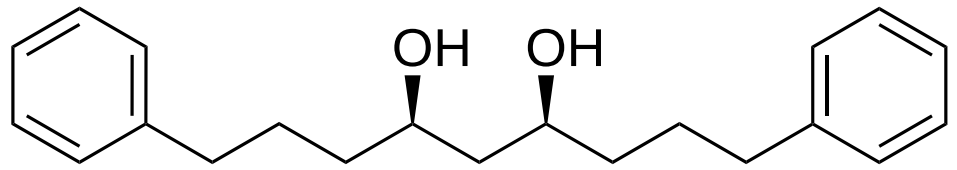

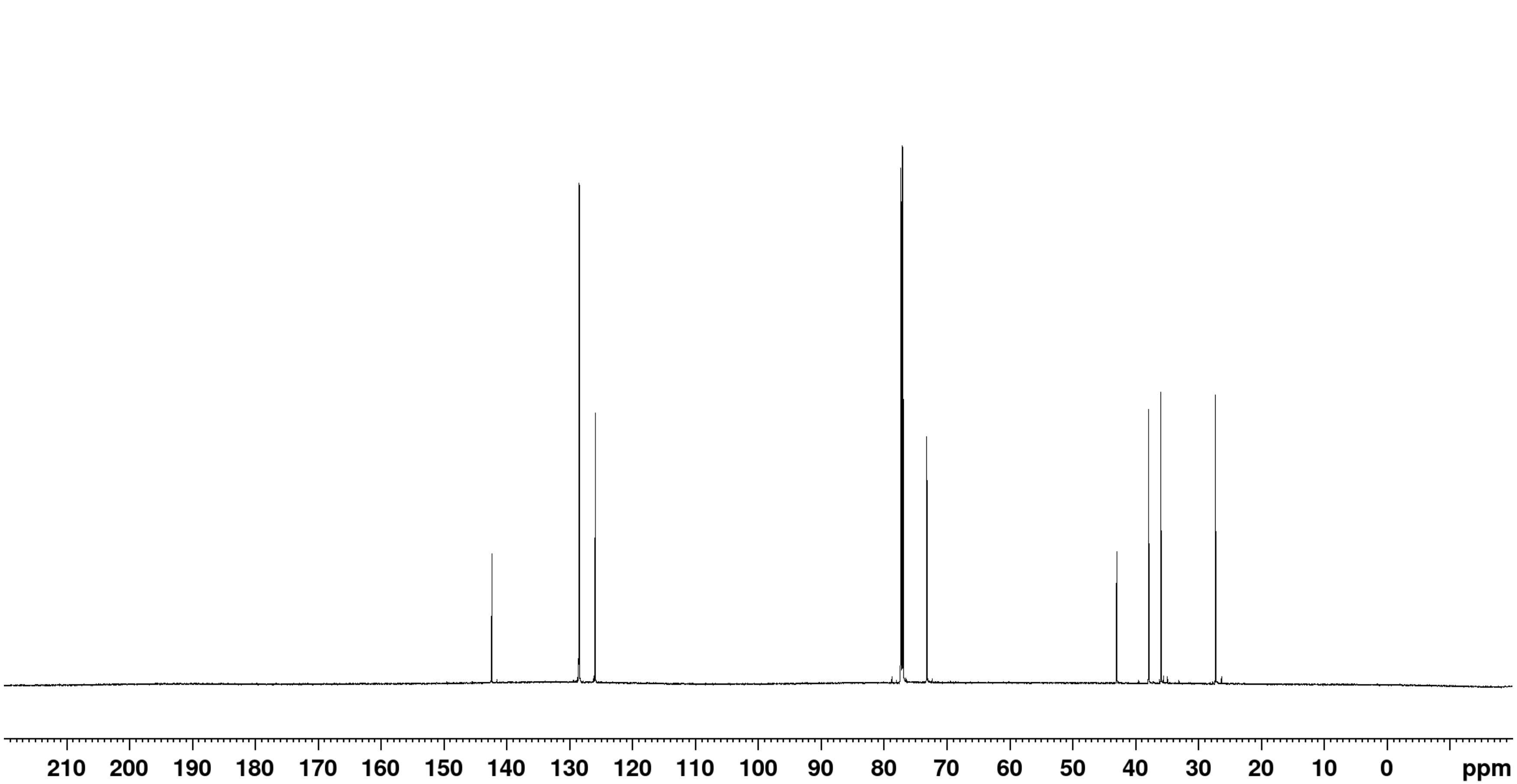

(*anti*)-1,9-diphenylnonane-4,6-diol (*rac*-1l)

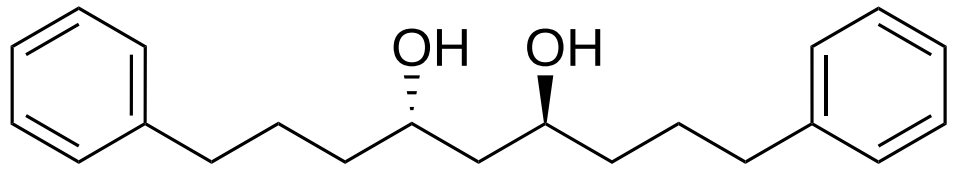

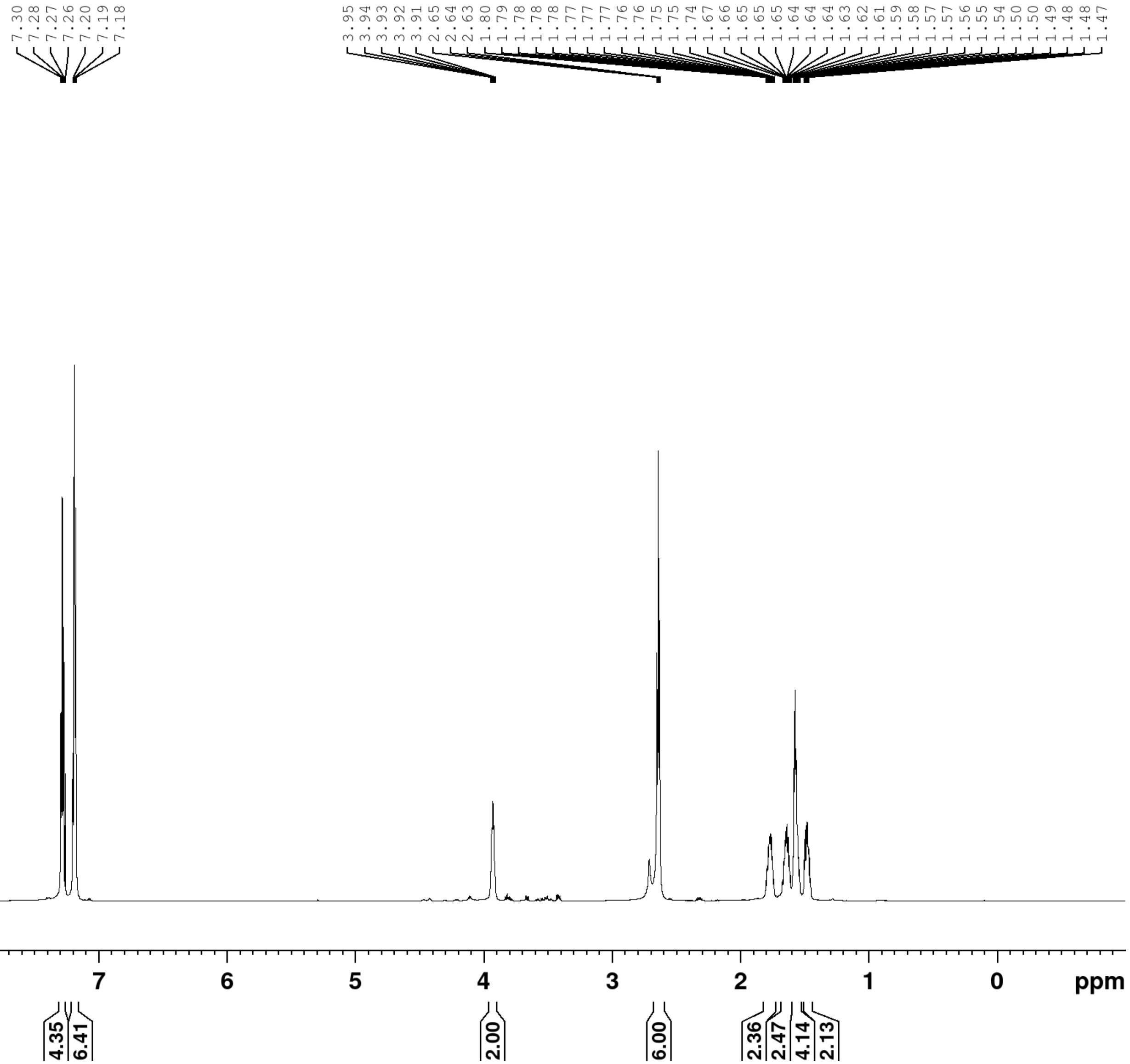

(*anti*)-1,9-diphenylnonane-4,6-diol (*rac*-1l)

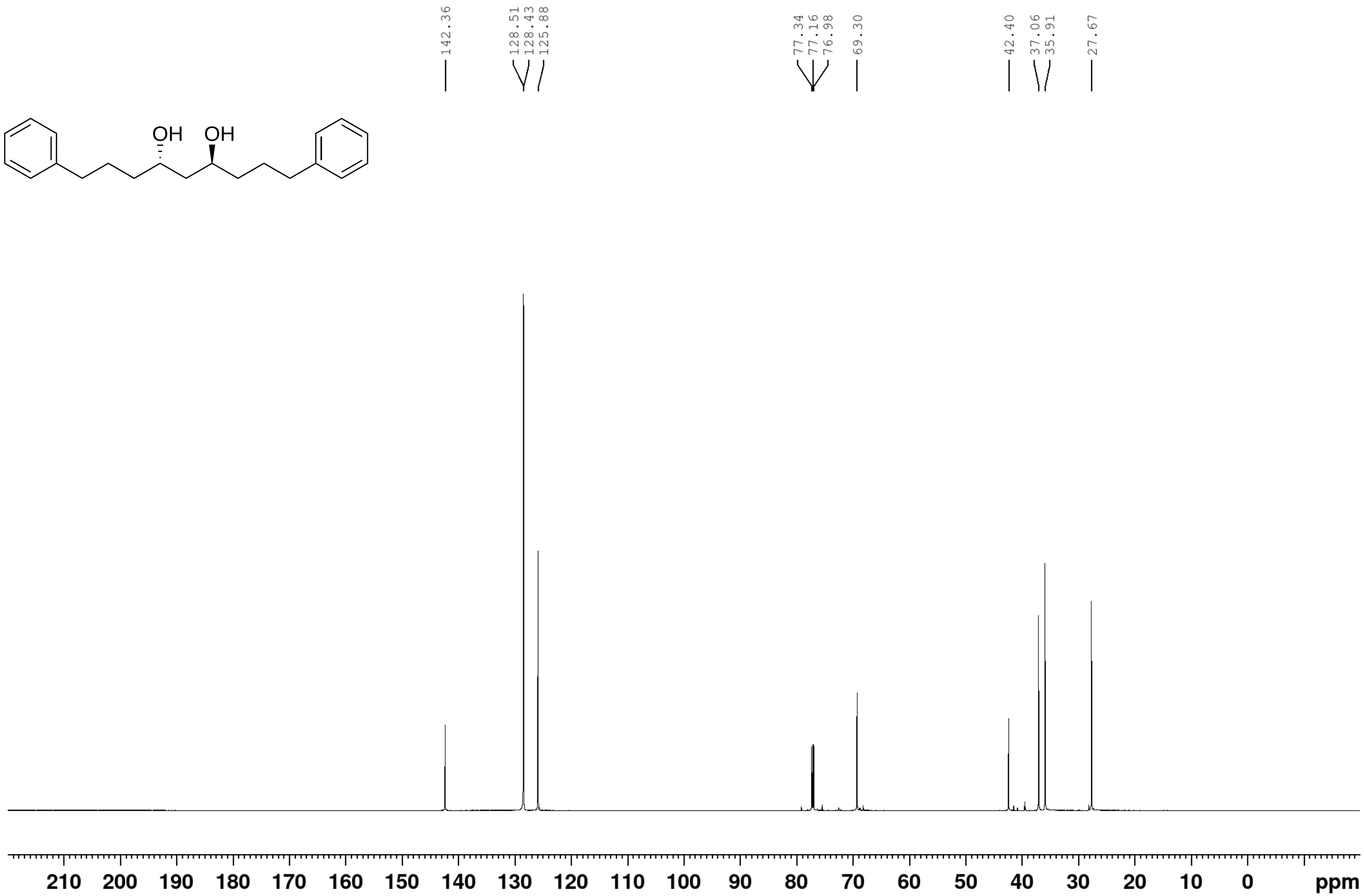

4-Hydroxy-5-phenylpentan-2-one (*int₁*-3a)

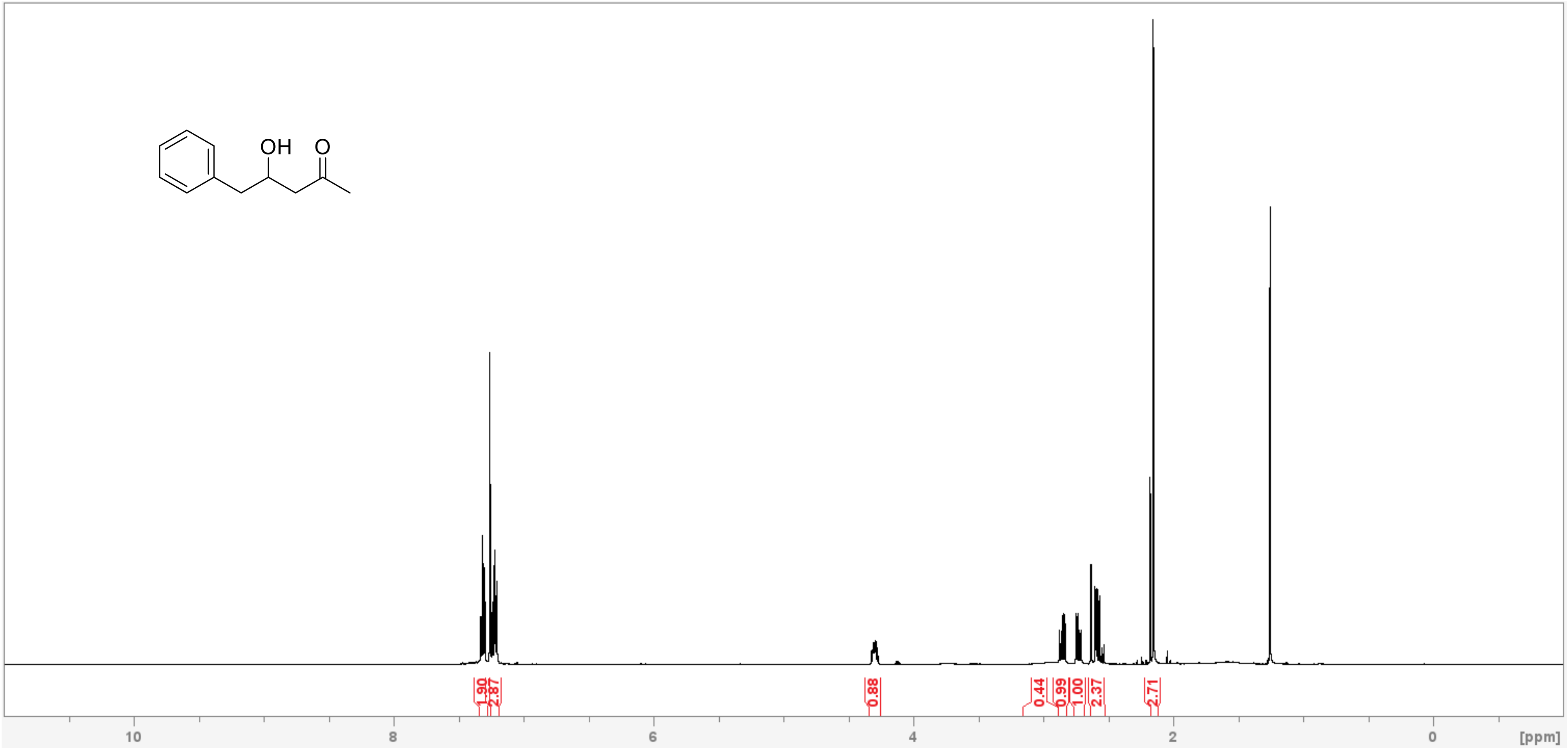

4-Hydroxy-5-phenylpentan-2-one (*int₁*-3a)

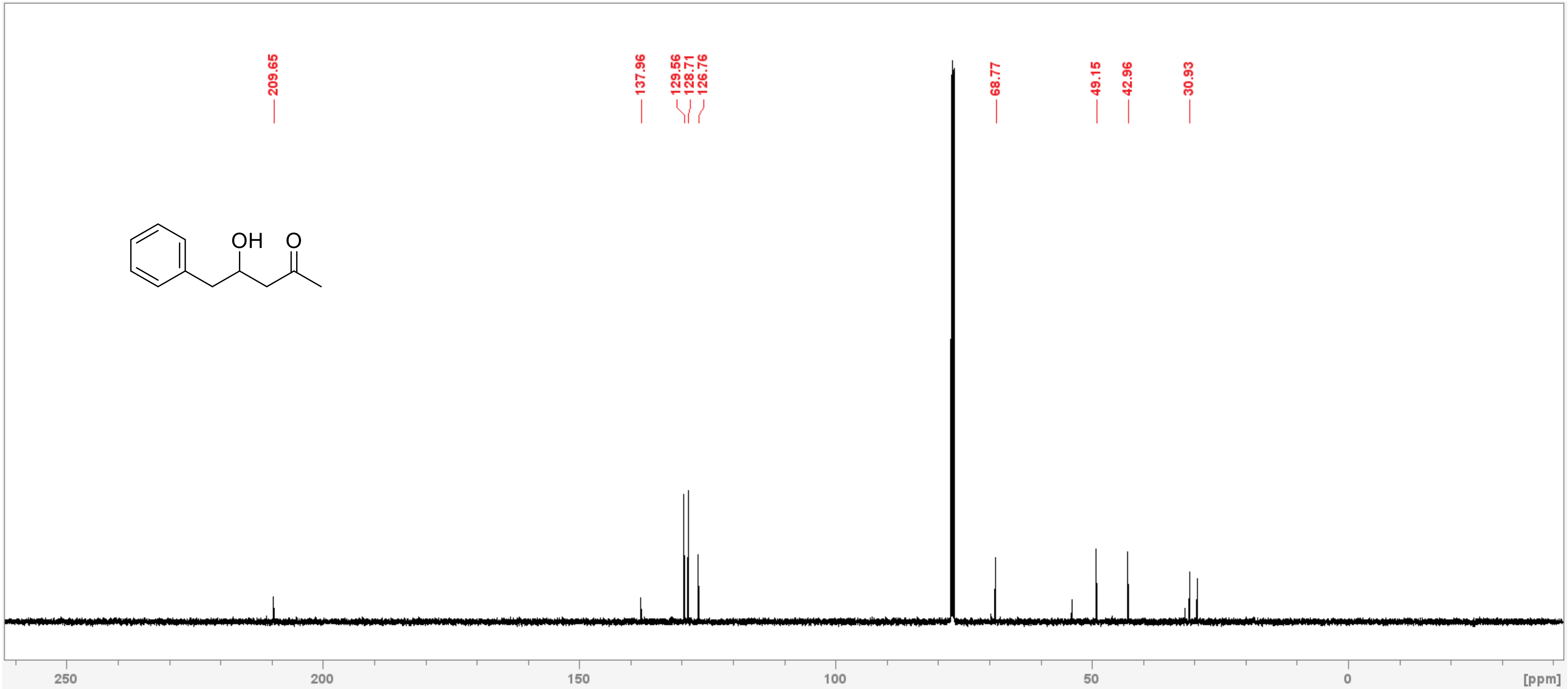

(*anti*)-1-Phenylpentane-2,4-diol (*rac*-*anti*-3a)

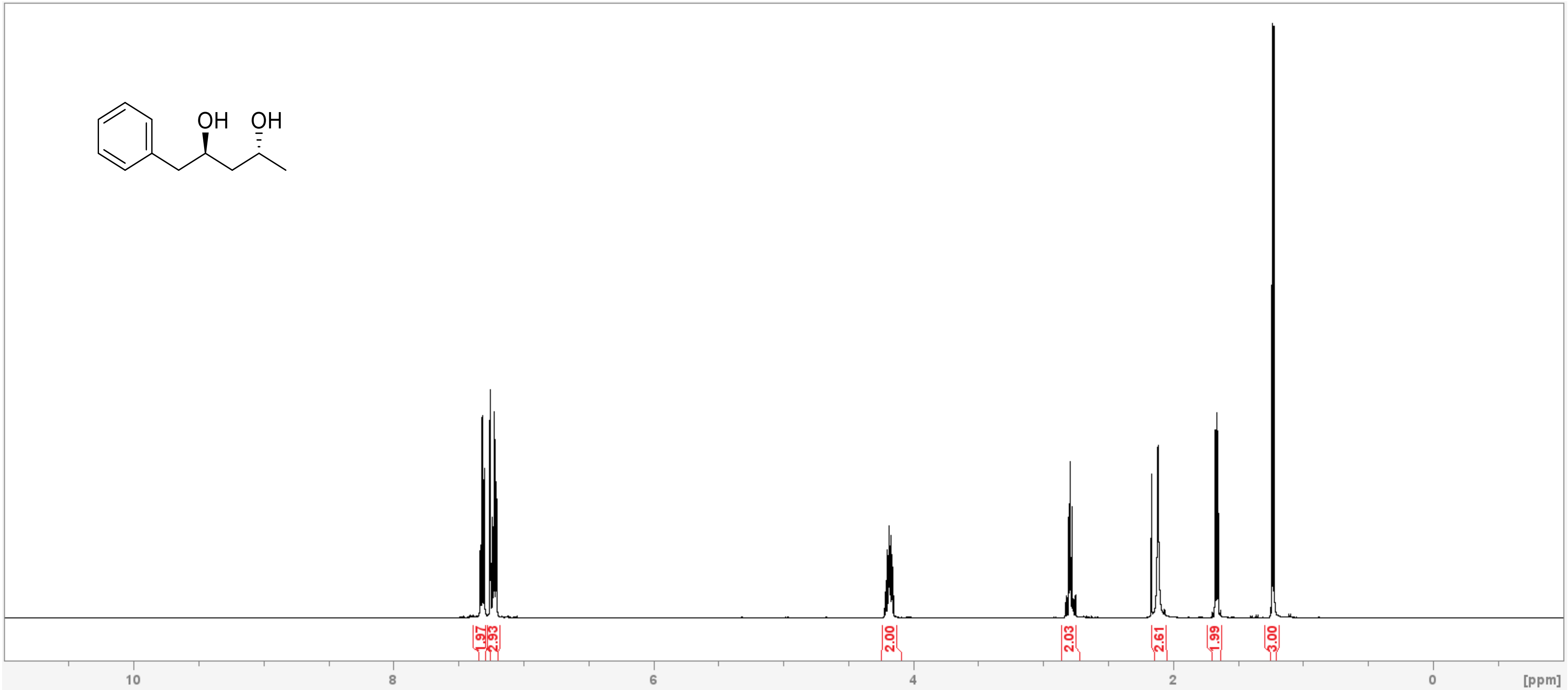

(*anti*)-1-Phenylpentane-2,4-diol (*rac*-*anti*-3a)

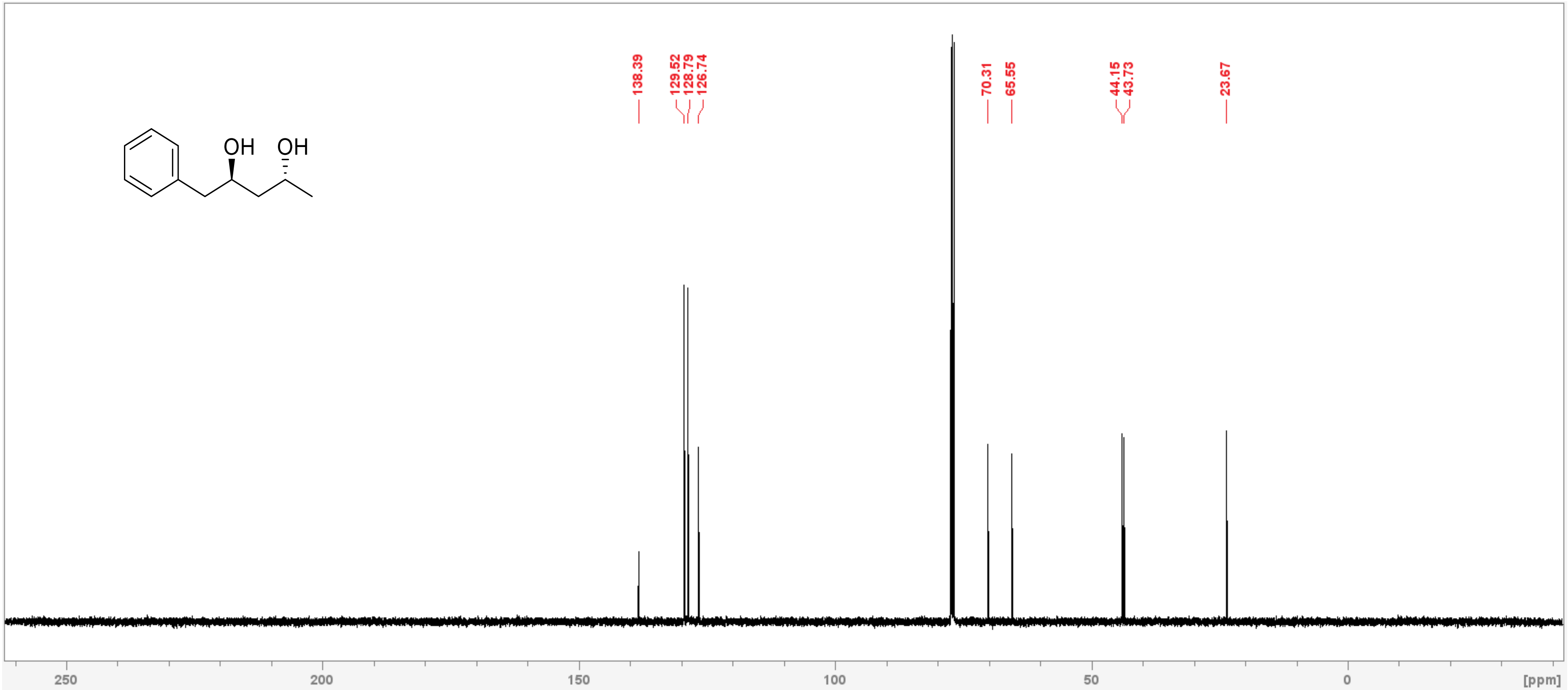

(*syn*)-1-Phenylpentane-2,4-diol (*rac*-*syn*-3a)

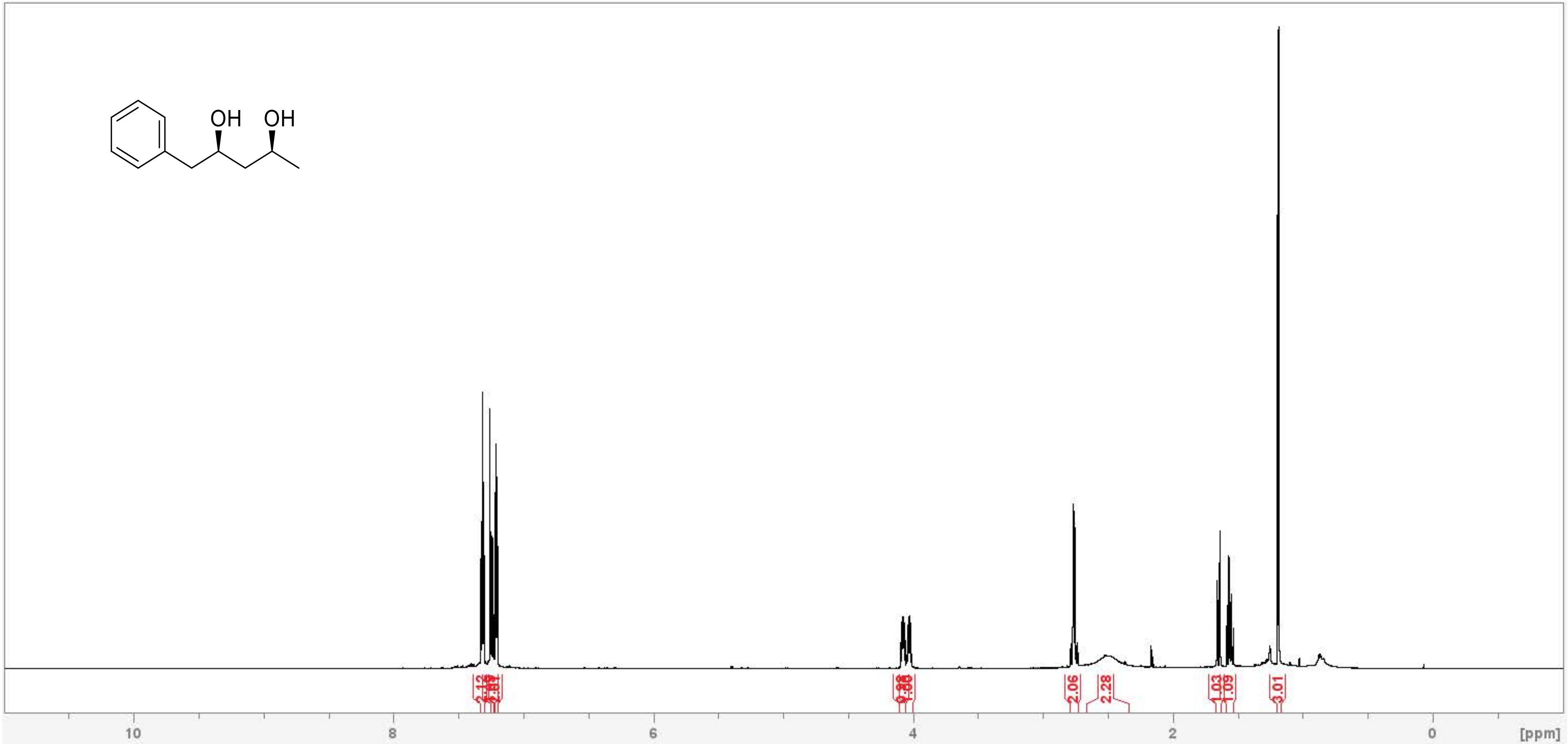

(*syn*)-1-Phenylpentane-2,4-diol (*rac*-*syn*-3a)

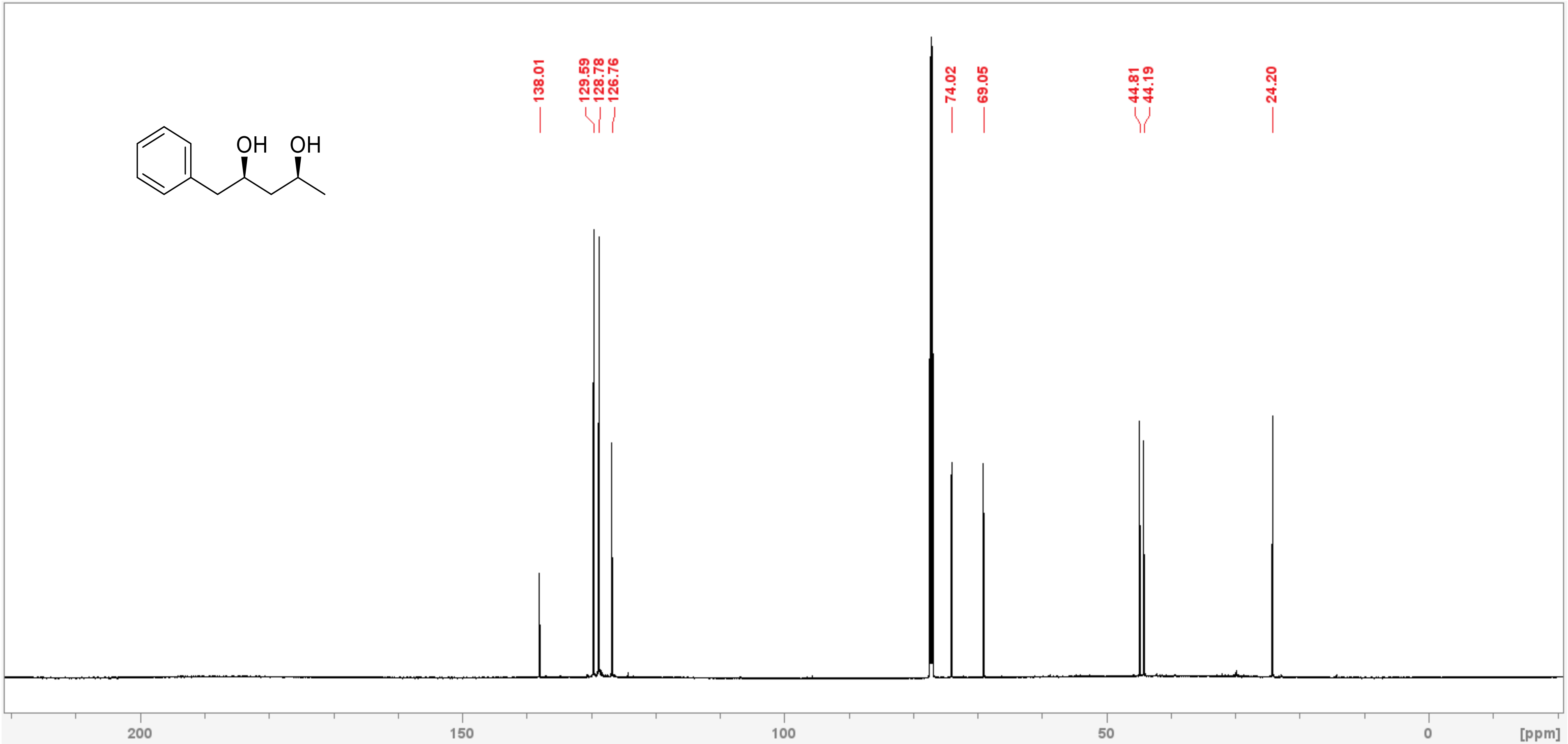

4-Hydroxy-5-(4-methoxyphenyl)pentan-2-one (*int₁*-3b)

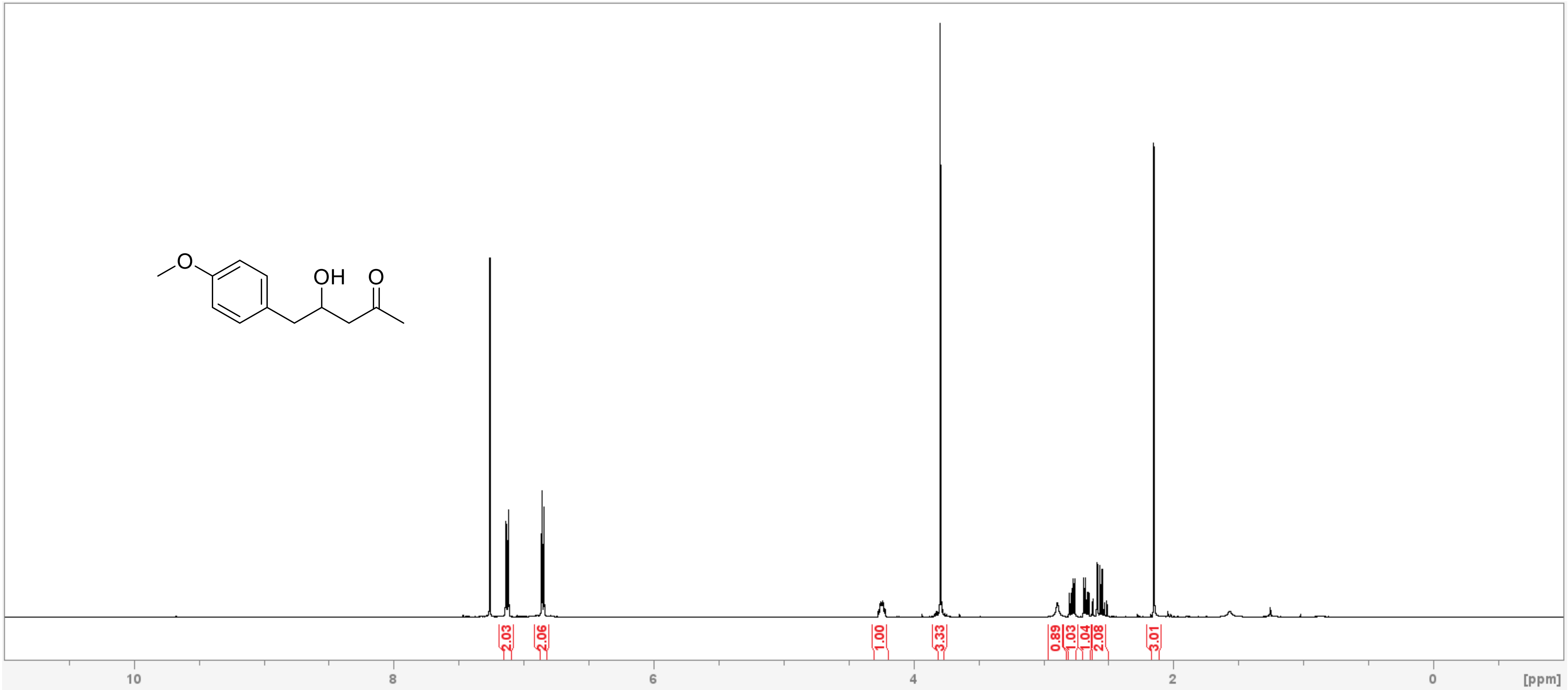

4-Hydroxy-5-(4-methoxyphenyl)pentan-2-one (*int₁*-3b)

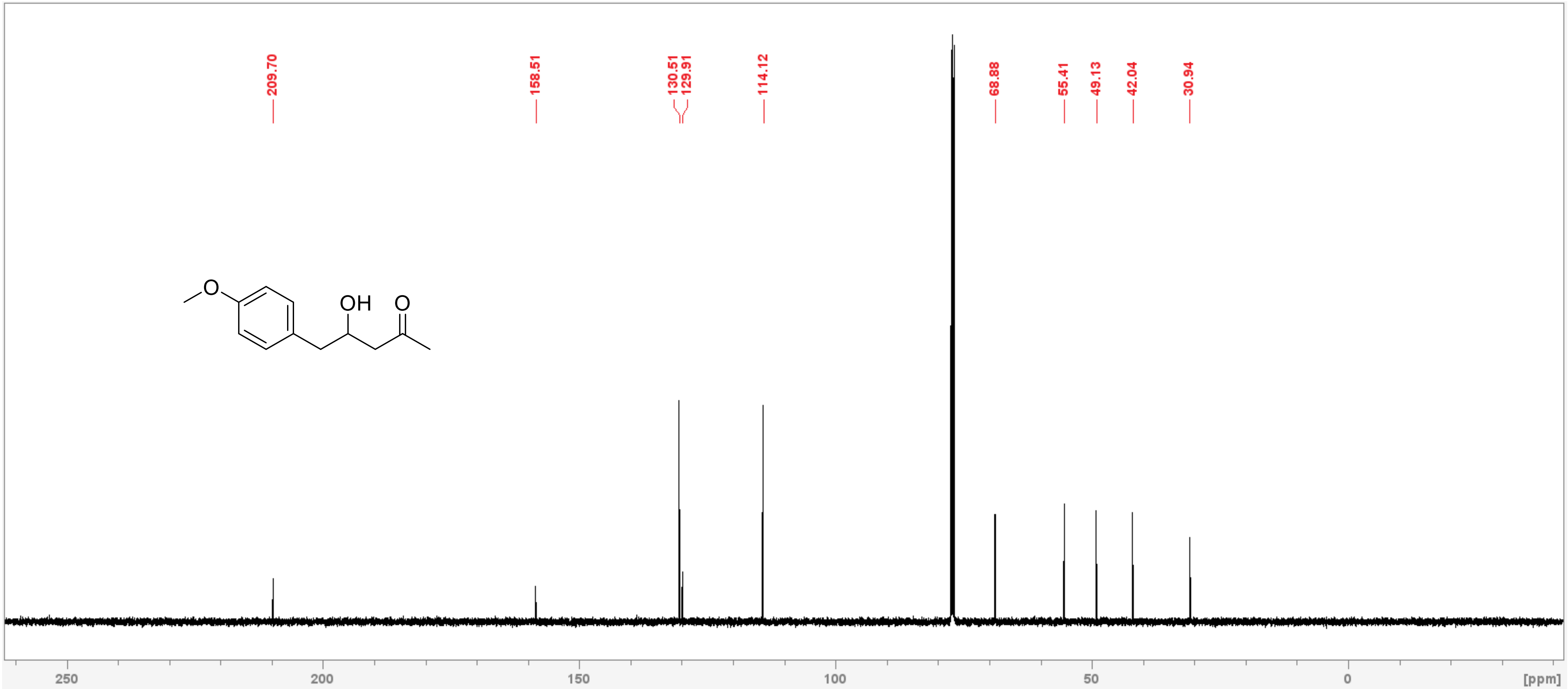

(*anti*)-1-(4-Methoxyphenyl)pentane-2,4-diol (*rac-anti*-3b)

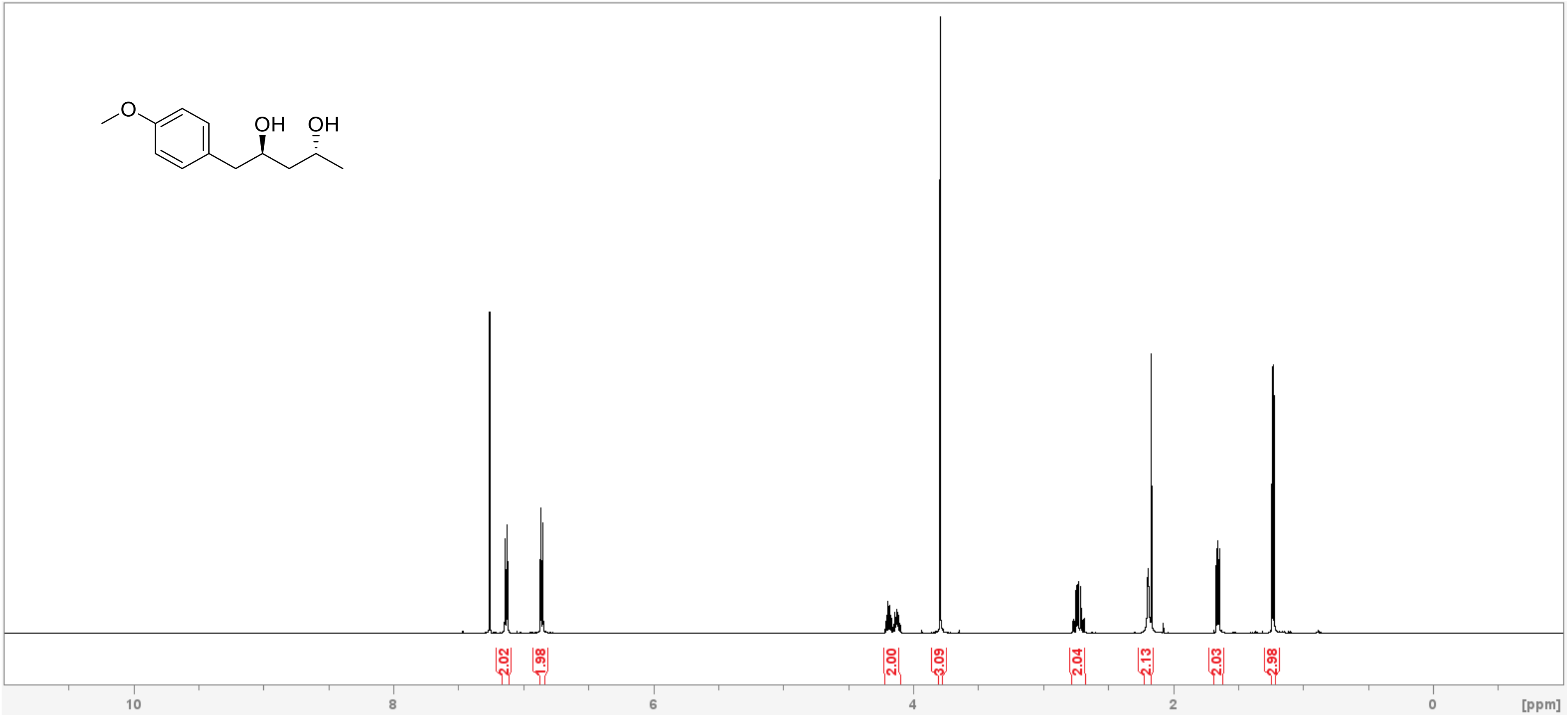

(*anti*)-1-(4-Methoxyphenyl)pentane-2,4-diol (*rac-anti*-3b)

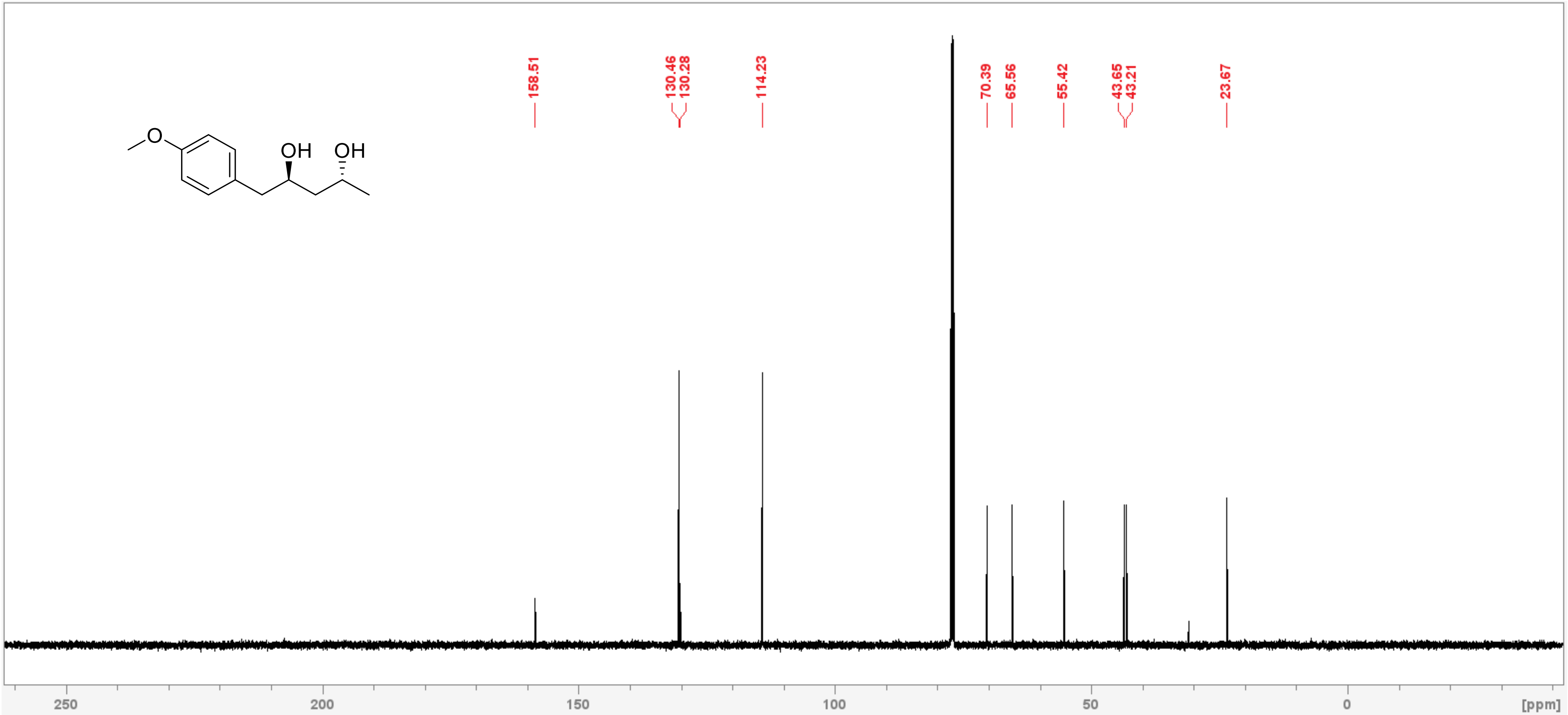

(*syn*)-1-(4-Methoxyphenyl)pentane-2,4-diol (*rac*-*syn*-3b)

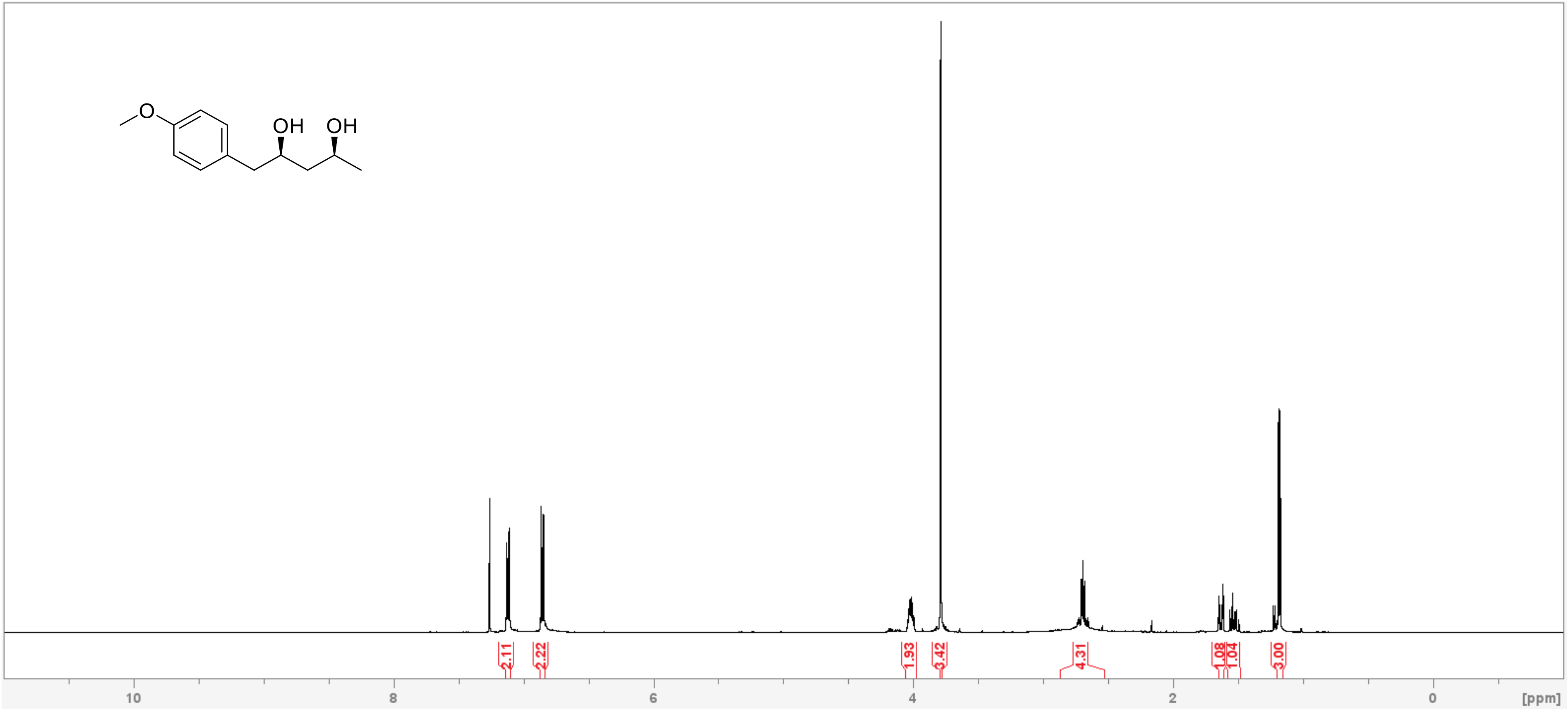

(*syn*)-1-(4-Methoxyphenyl)pentane-2,4-diol (*rac*-*syn*-3b)

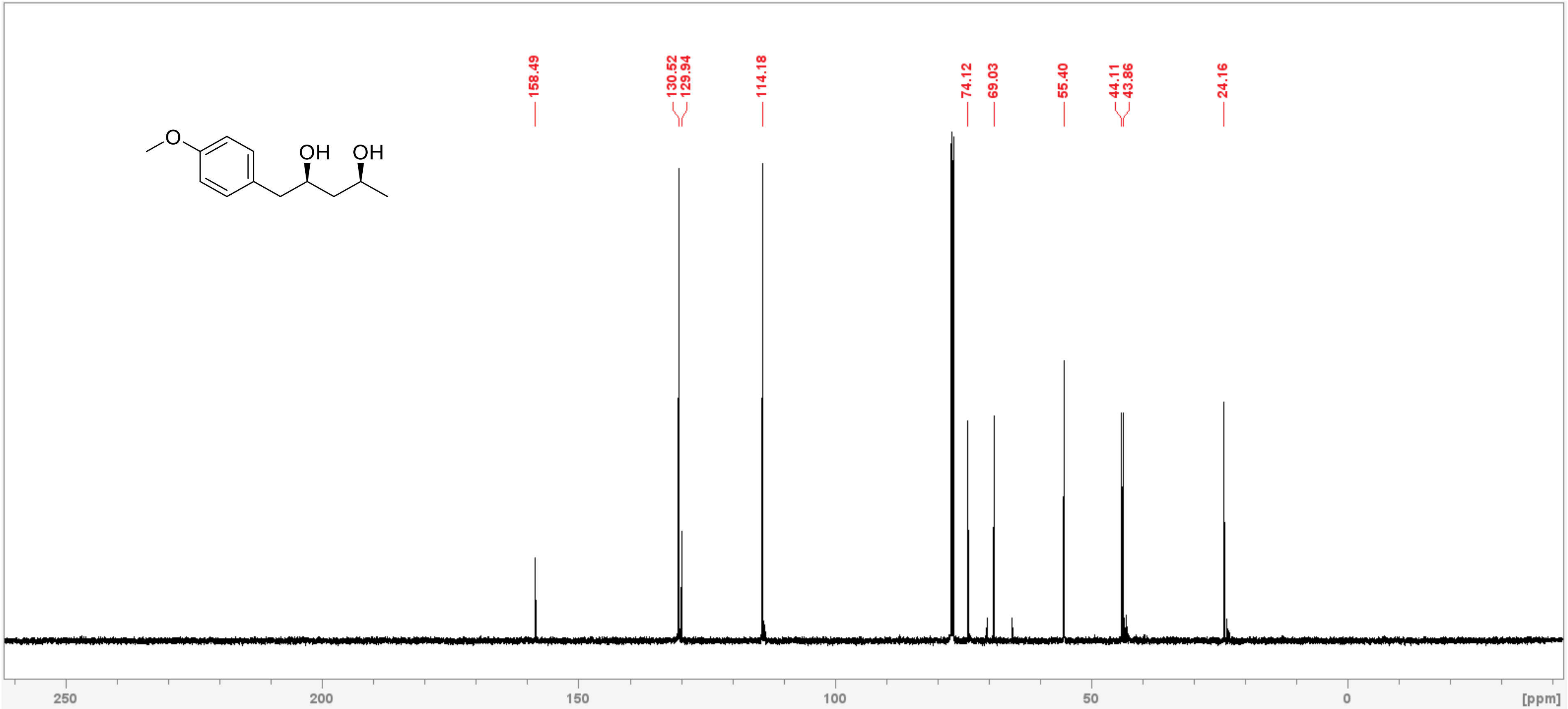

4-Hydroxy-5-(naphthalen-1-yl)pentan-2-one (*int₁*-3c)

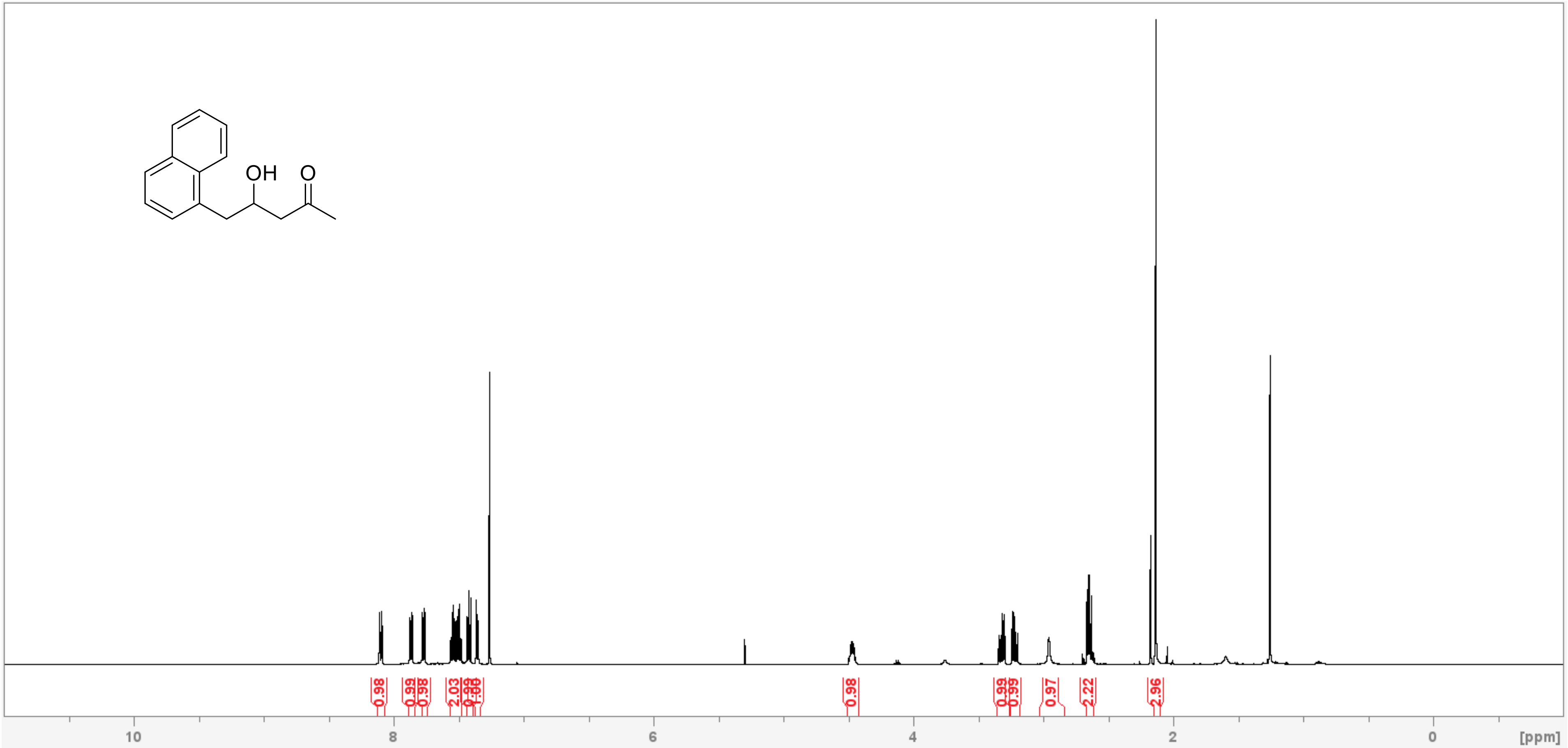

4-Hydroxy-5-(naphthalen-1-yl)pentan-2-one (*int₁*-3c)

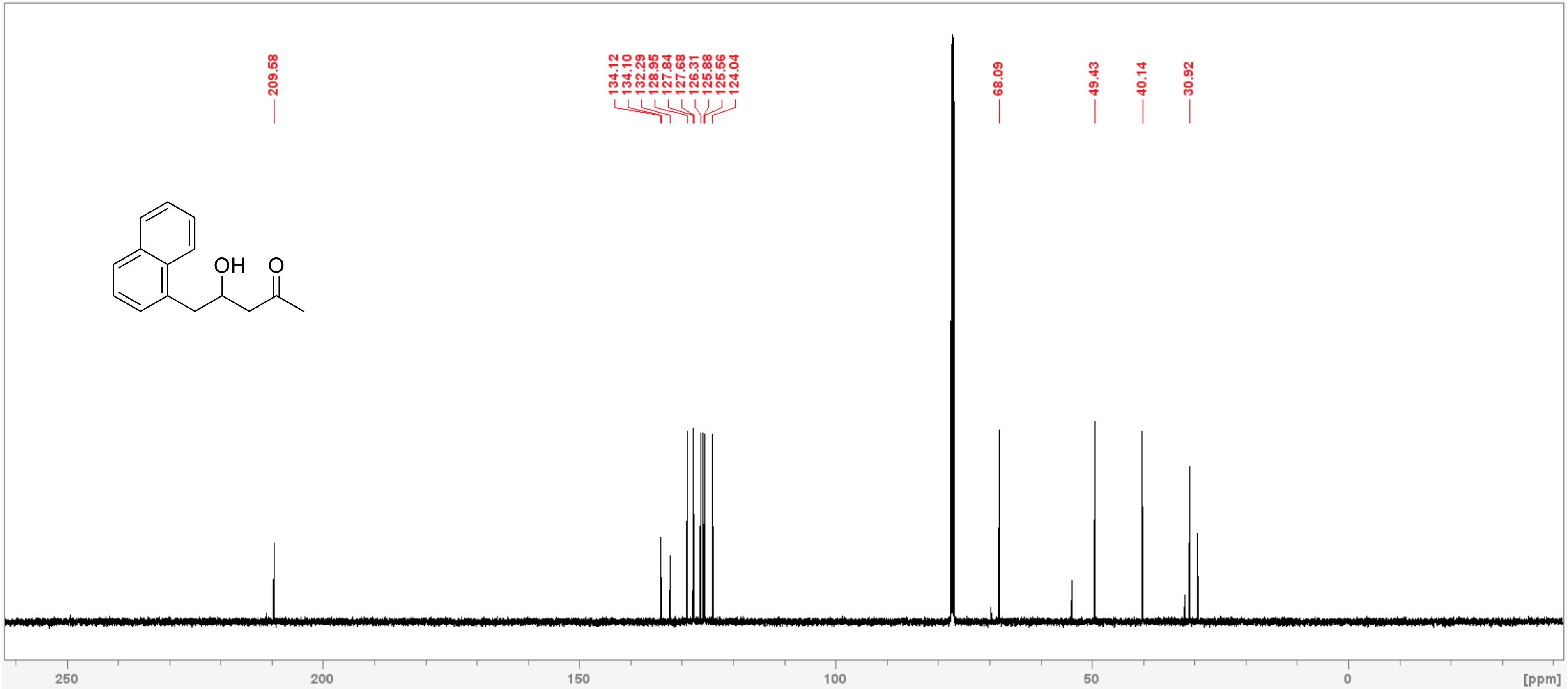

(*anti*)-1-(Naphthalen-1-yl)pentane-2,4-diol (*rac*-*anti*-3c)

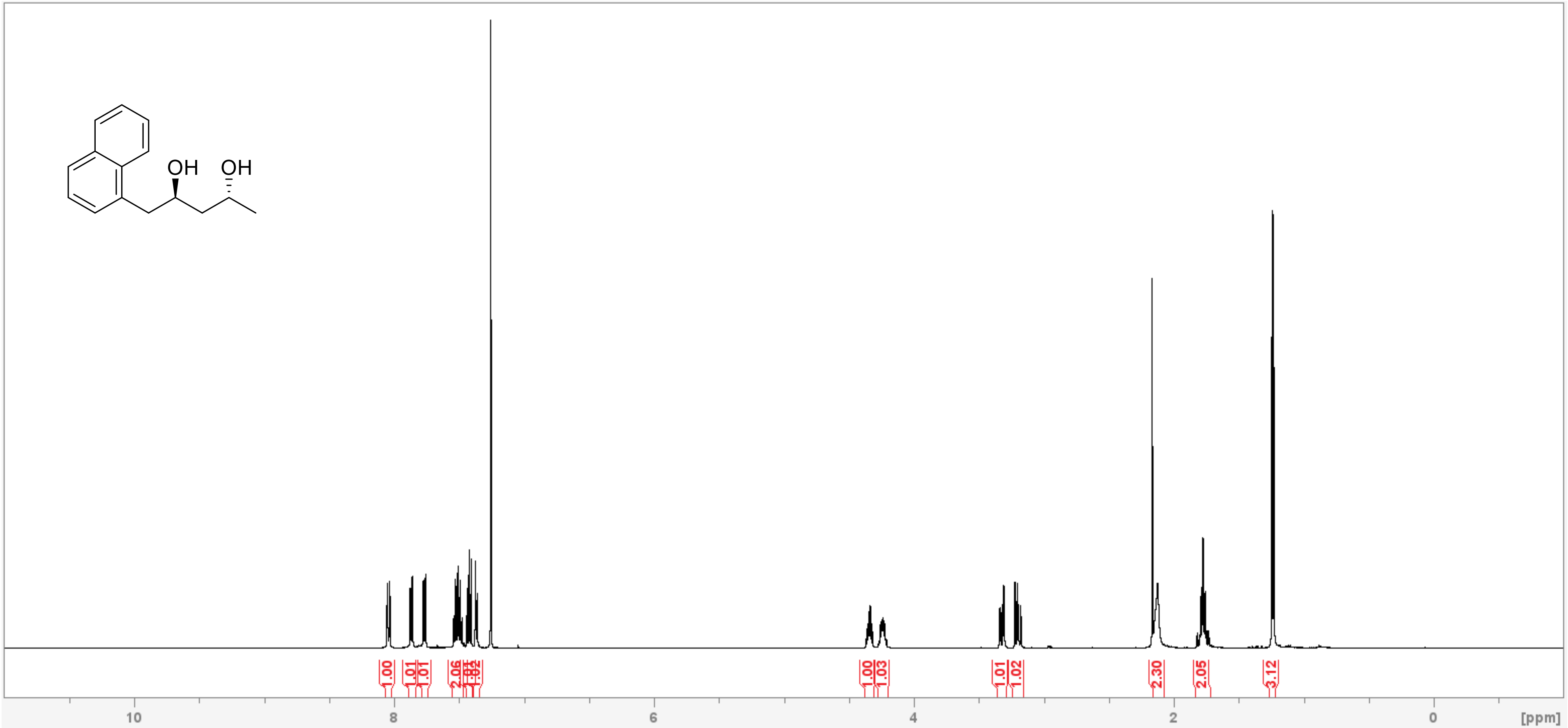

(*anti*)-1-(Naphthalen-1-yl)pentane-2,4-diol (*rac*-*anti*-3c)

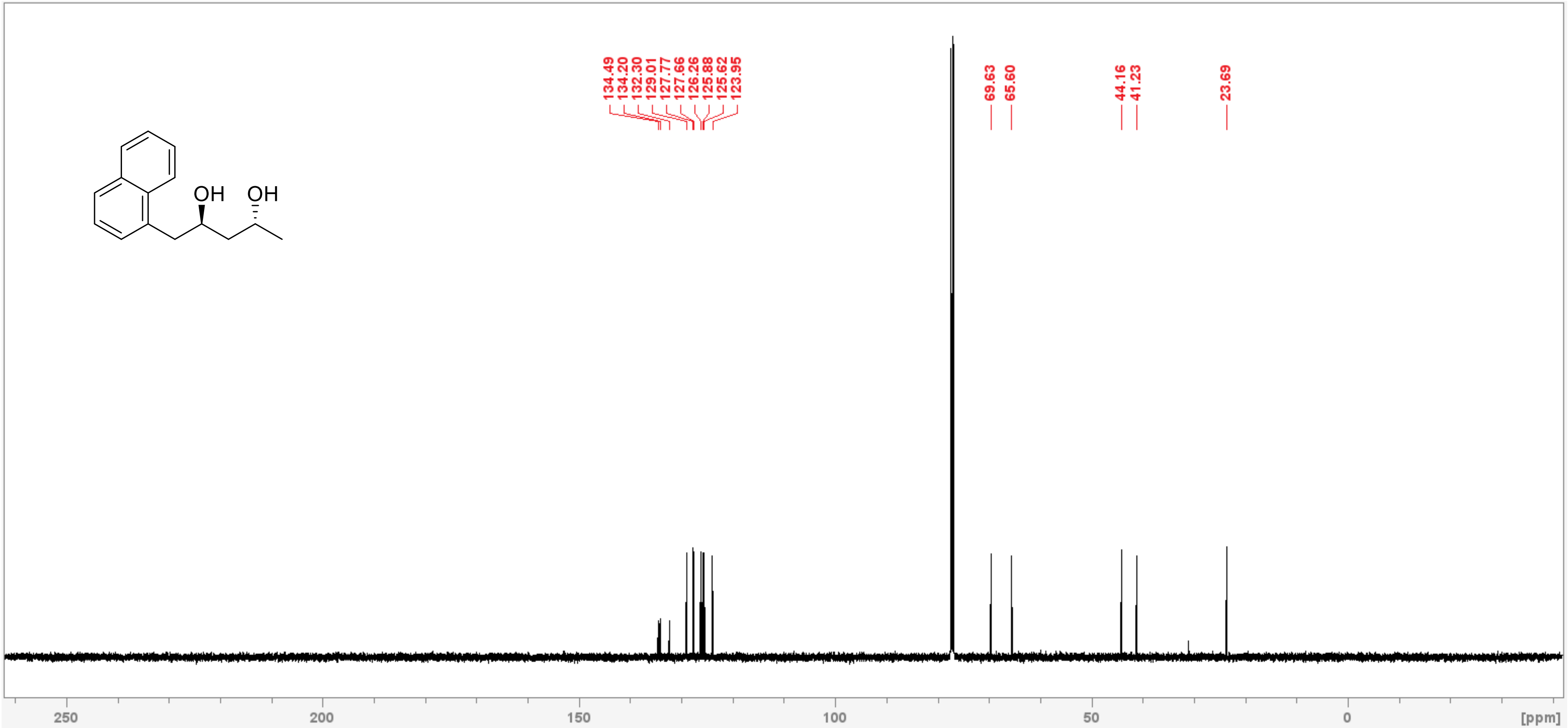

(*syn*)-1-(Naphthalen-1-yl)pentane-2,4-diol (*rac*-*syn*-3c)

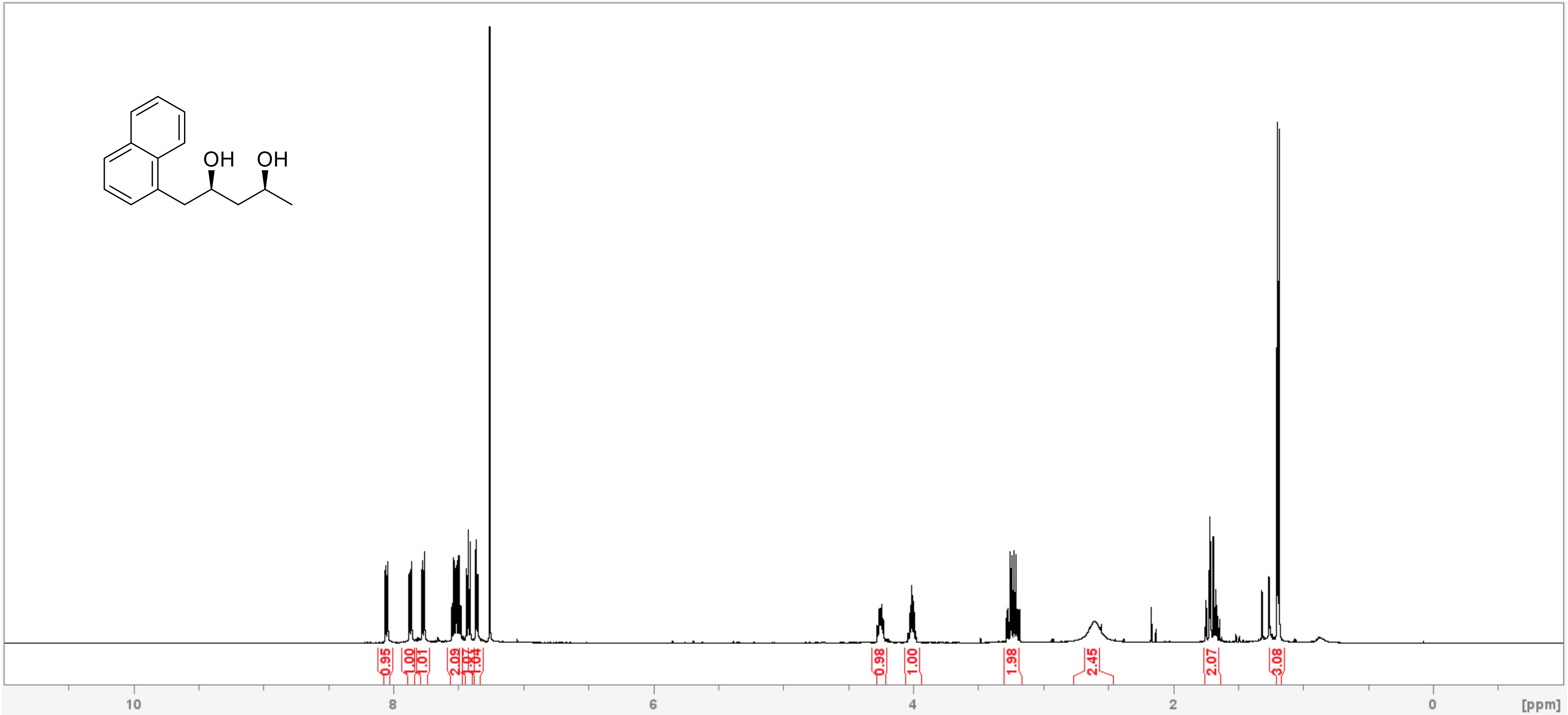

(*syn*)-1-(Naphthalen-1-yl)pentane-2,4-diol (*rac*-*syn*-3c)

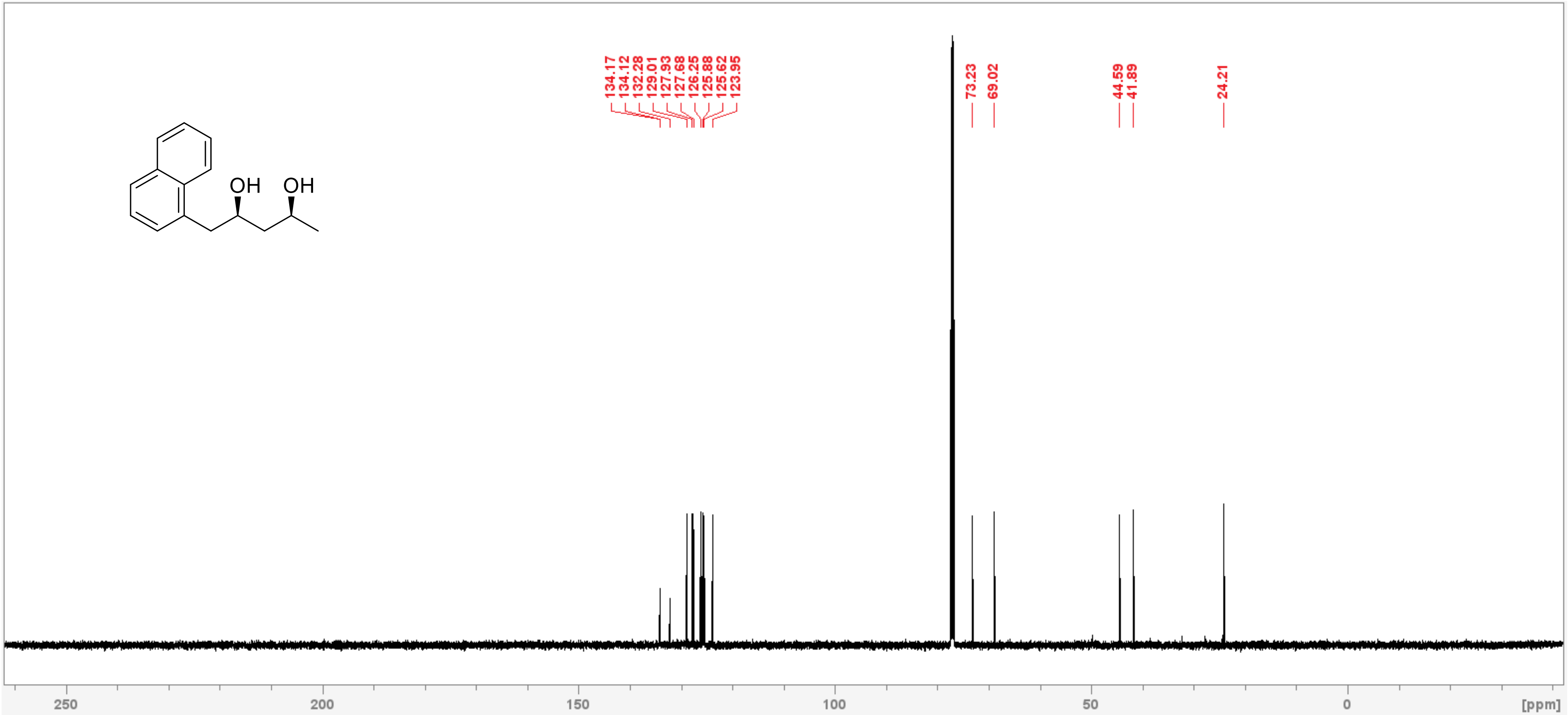

(*anti*)-6-Phenylhexane-2,4-diol (*rac-anti*-3d)

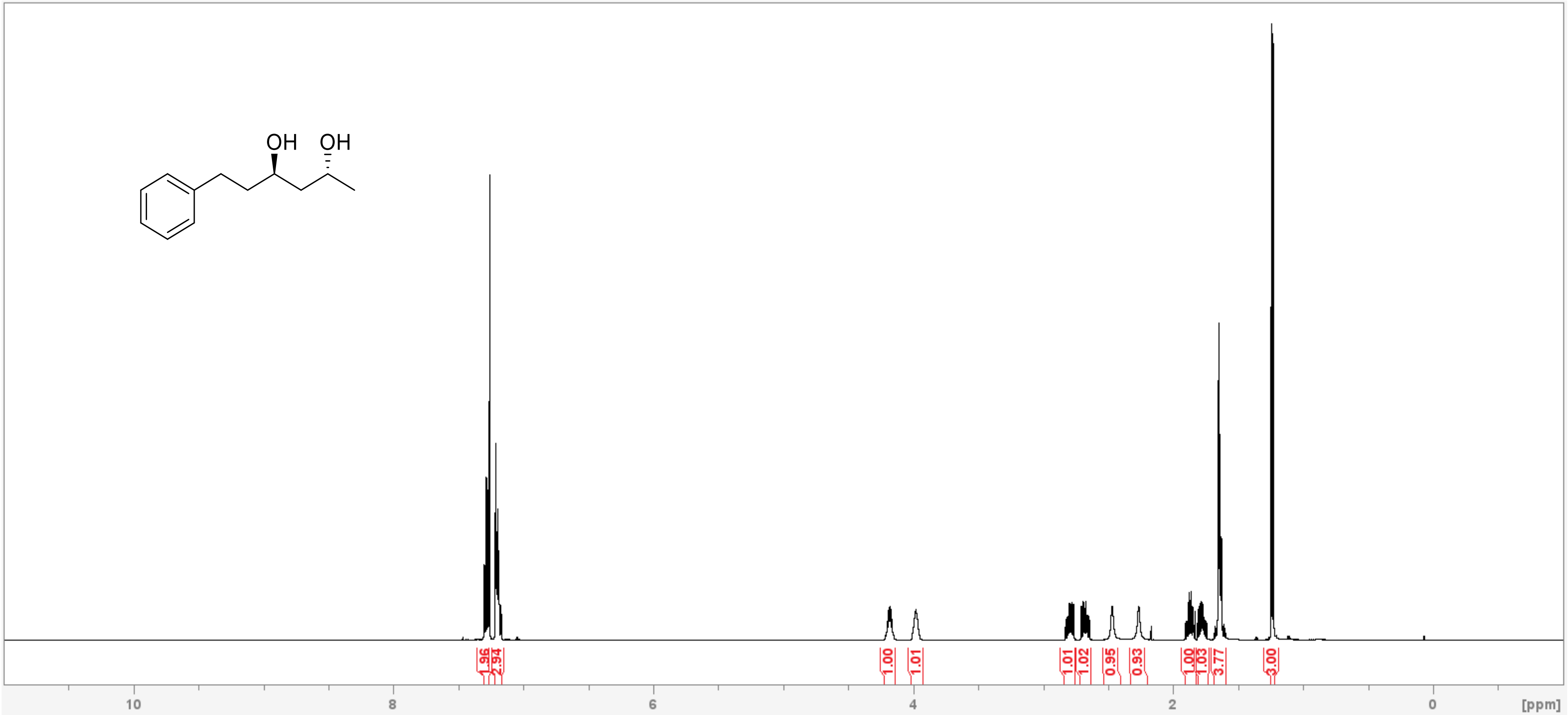

(*anti*)-6-Phenylhexane-2,4-diol (*rac*-*anti*-3d)

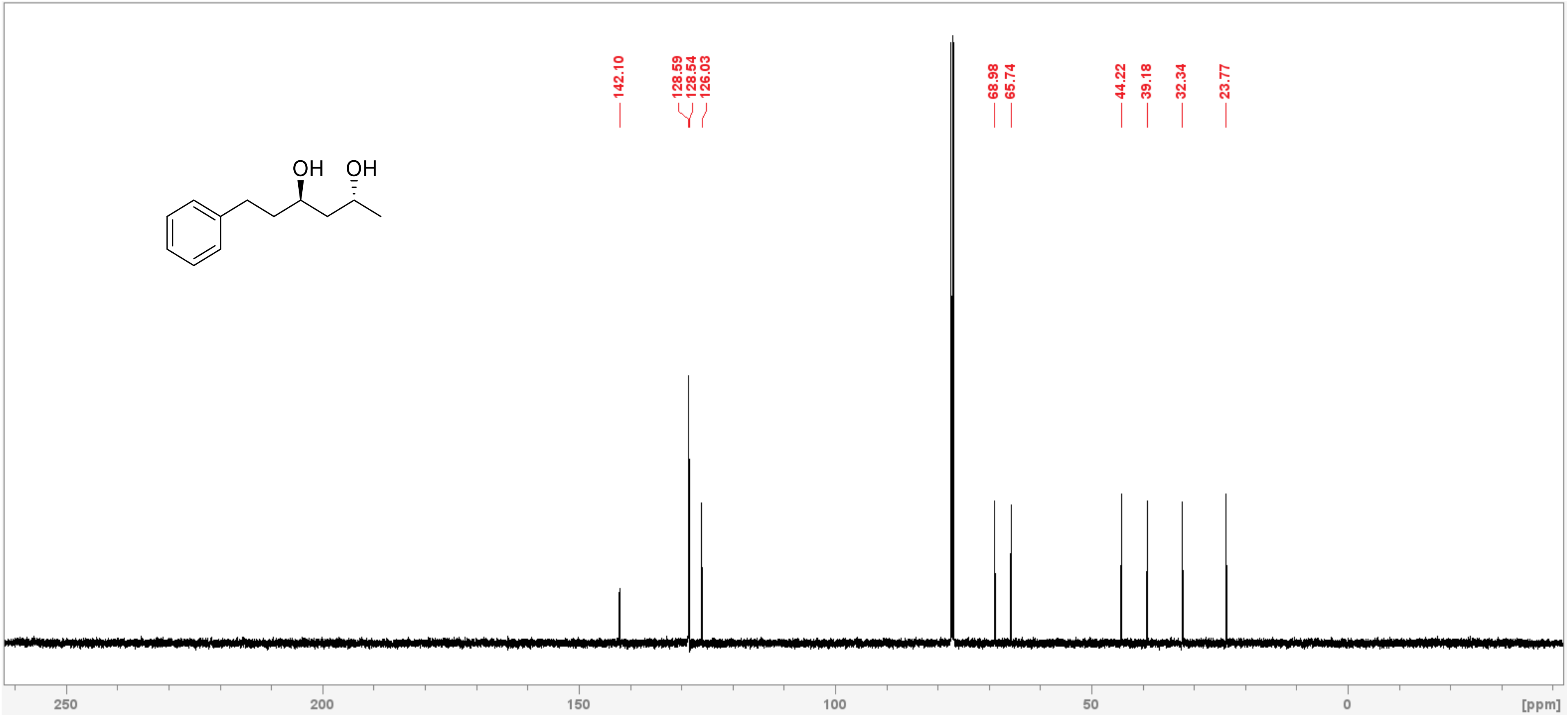

(*syn*)-6-Phenylhexane-2,4-diol (*rac*-*syn*-3d)

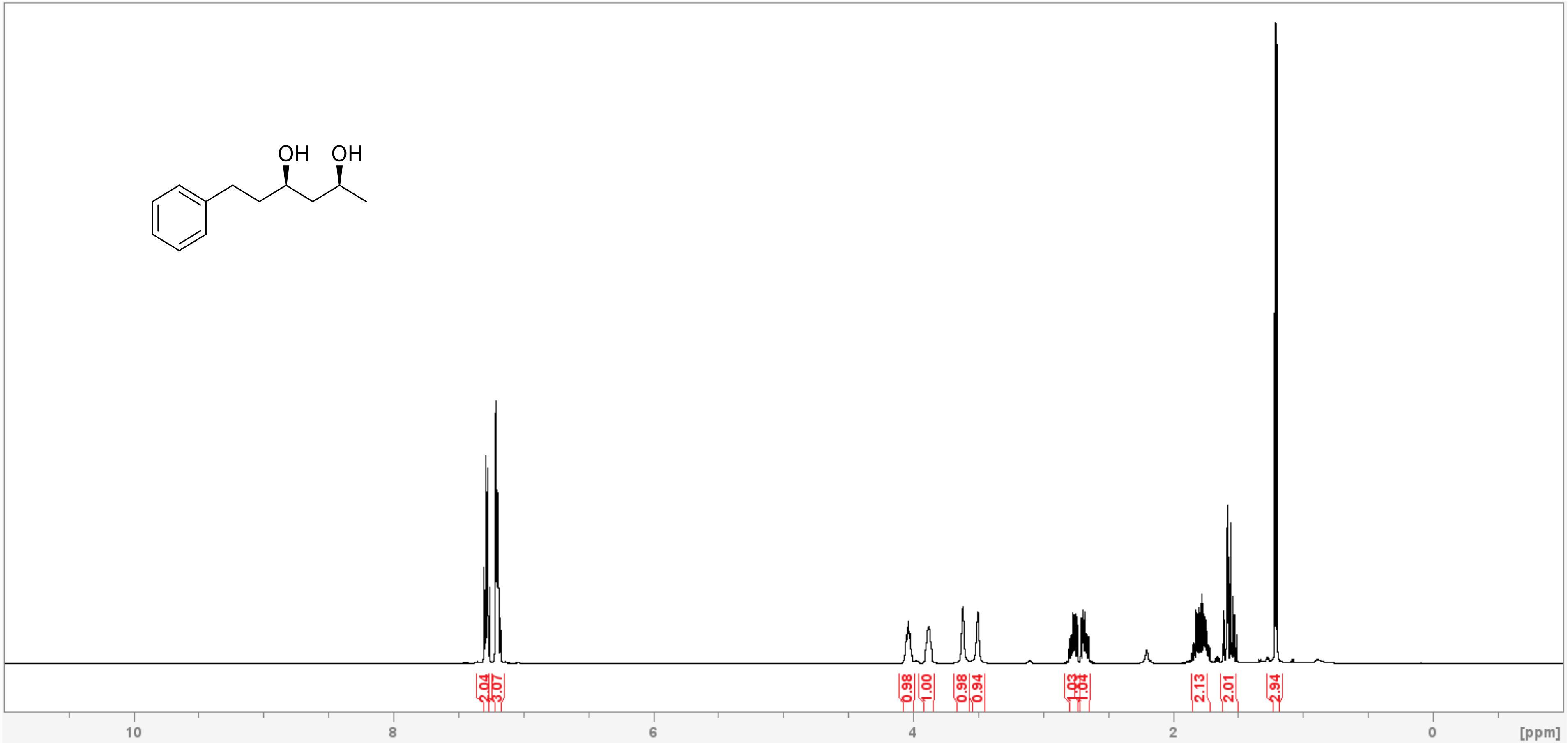

(*syn*)-6-Phenylhexane-2,4-diol (*rac*-*syn*-3d)

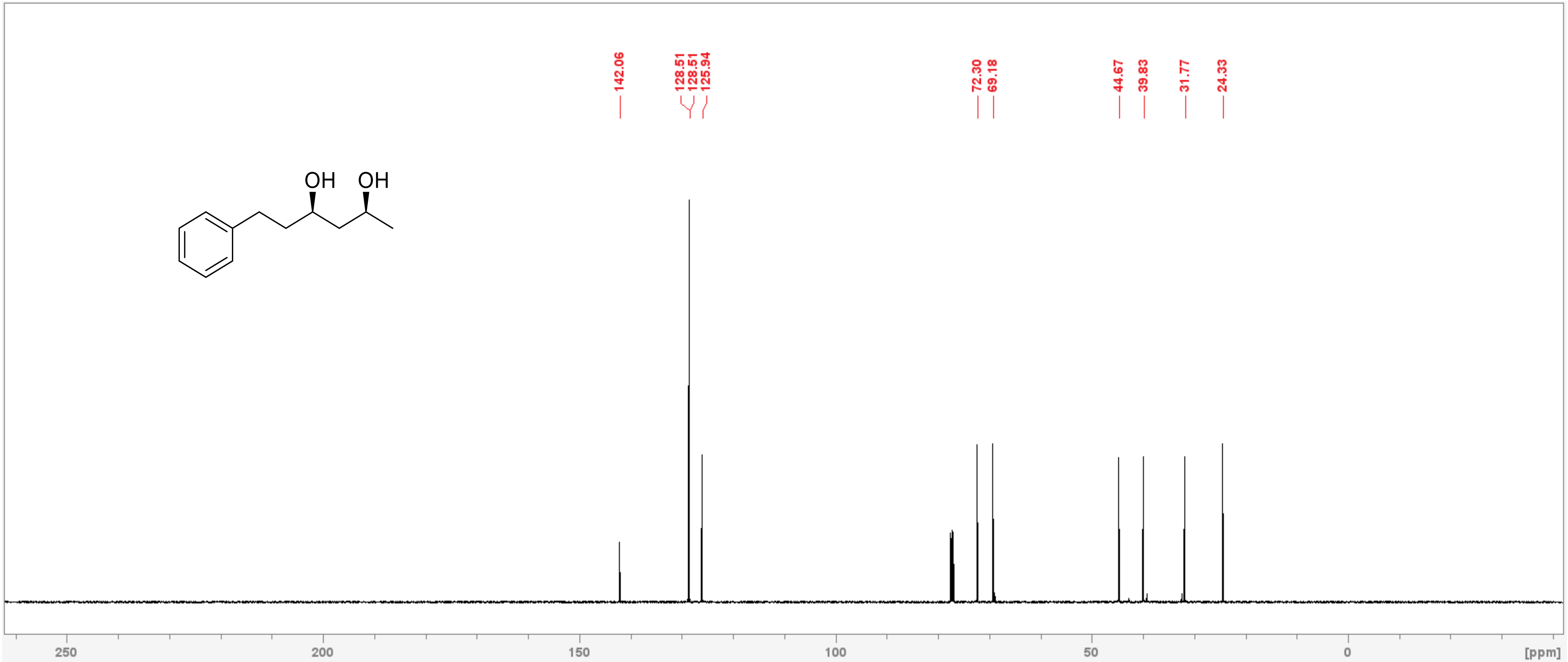

Ethyl 3-(3-hydroxy-5-oxohexyl)benzoate (*int₁*-3e)

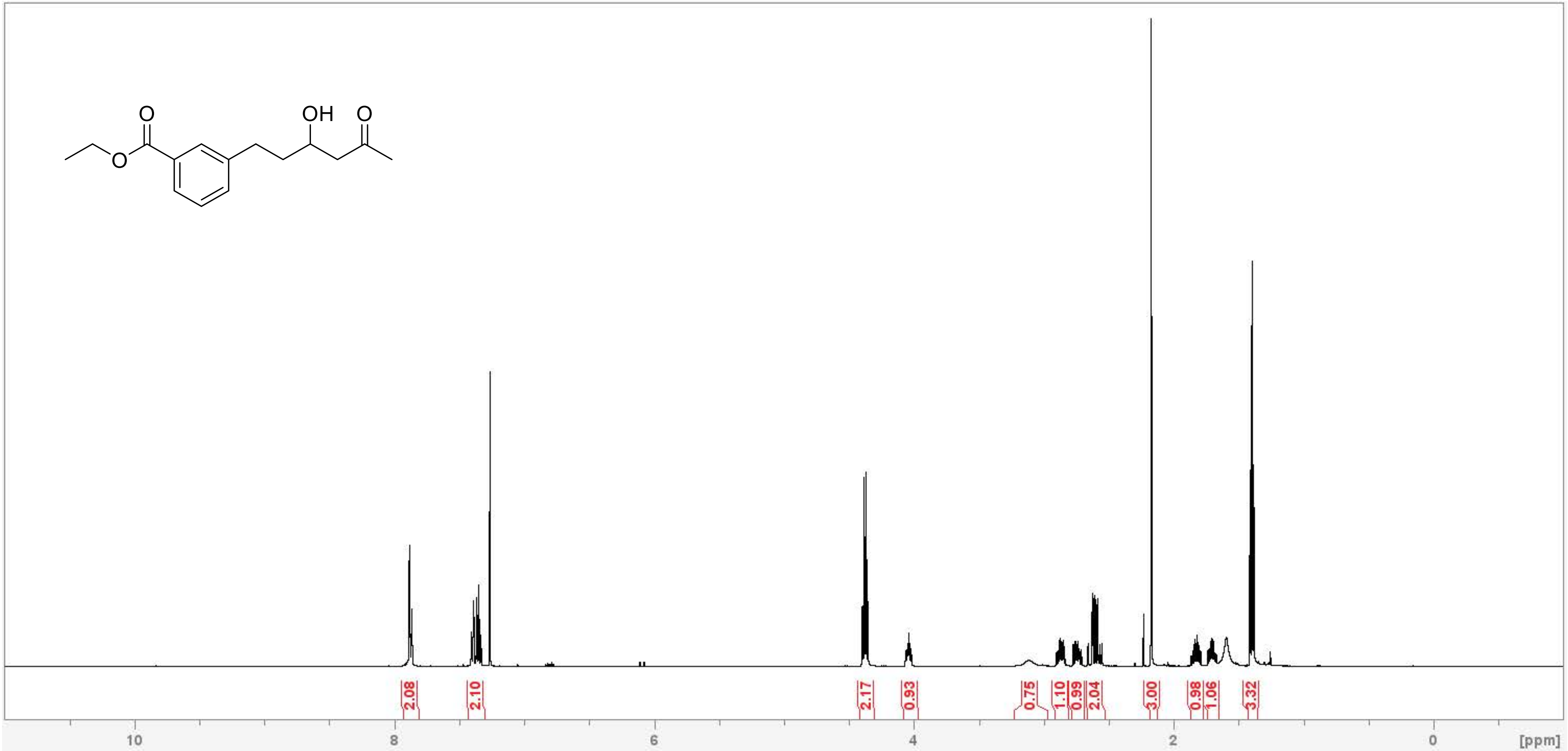

Ethyl 3-(3-hydroxy-5-oxohexyl)benzoate (*int₁*-3e)

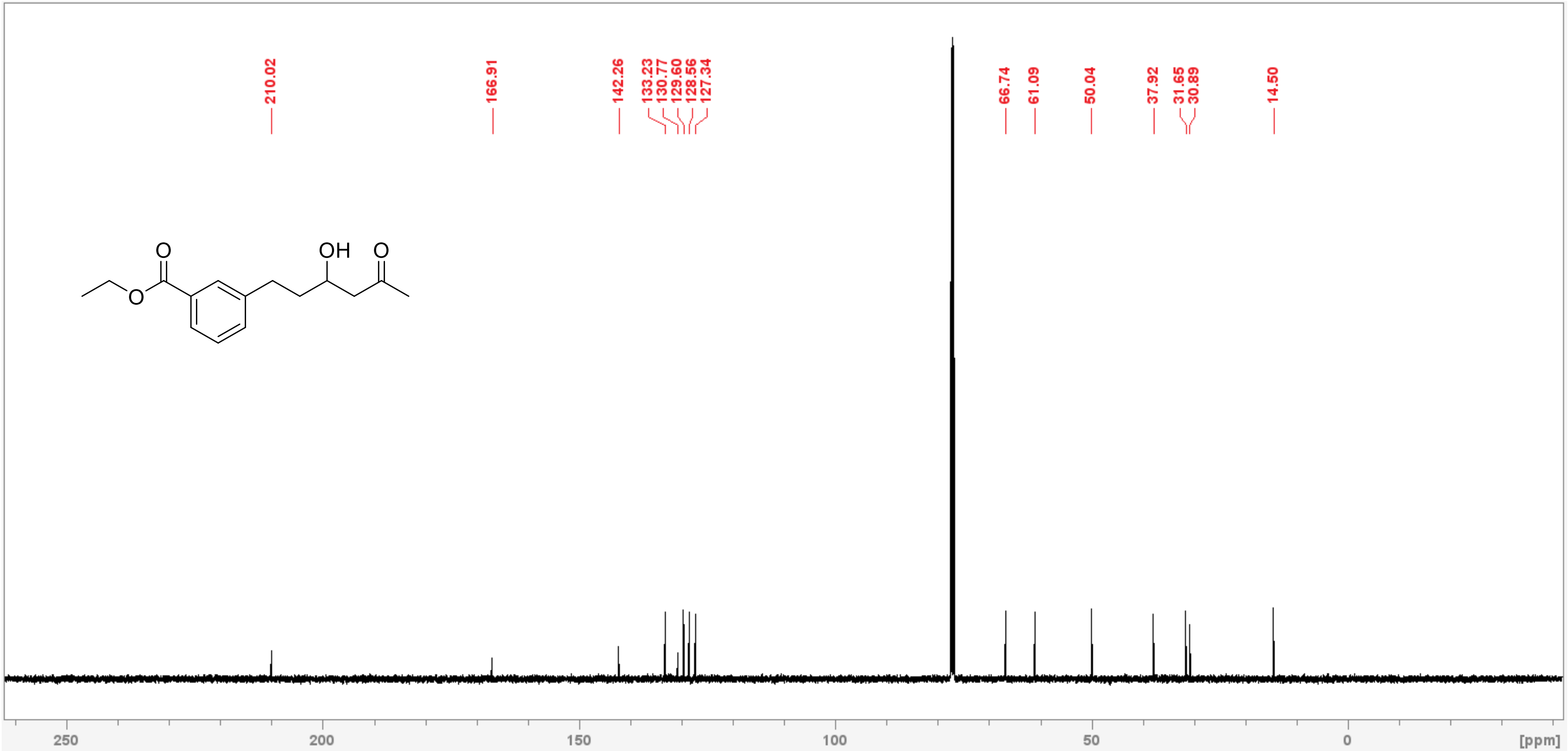

Ethyl 3-((*anti*)-3,5-dihydroxyhexyl)benzoate (*rac-anti*-3e)

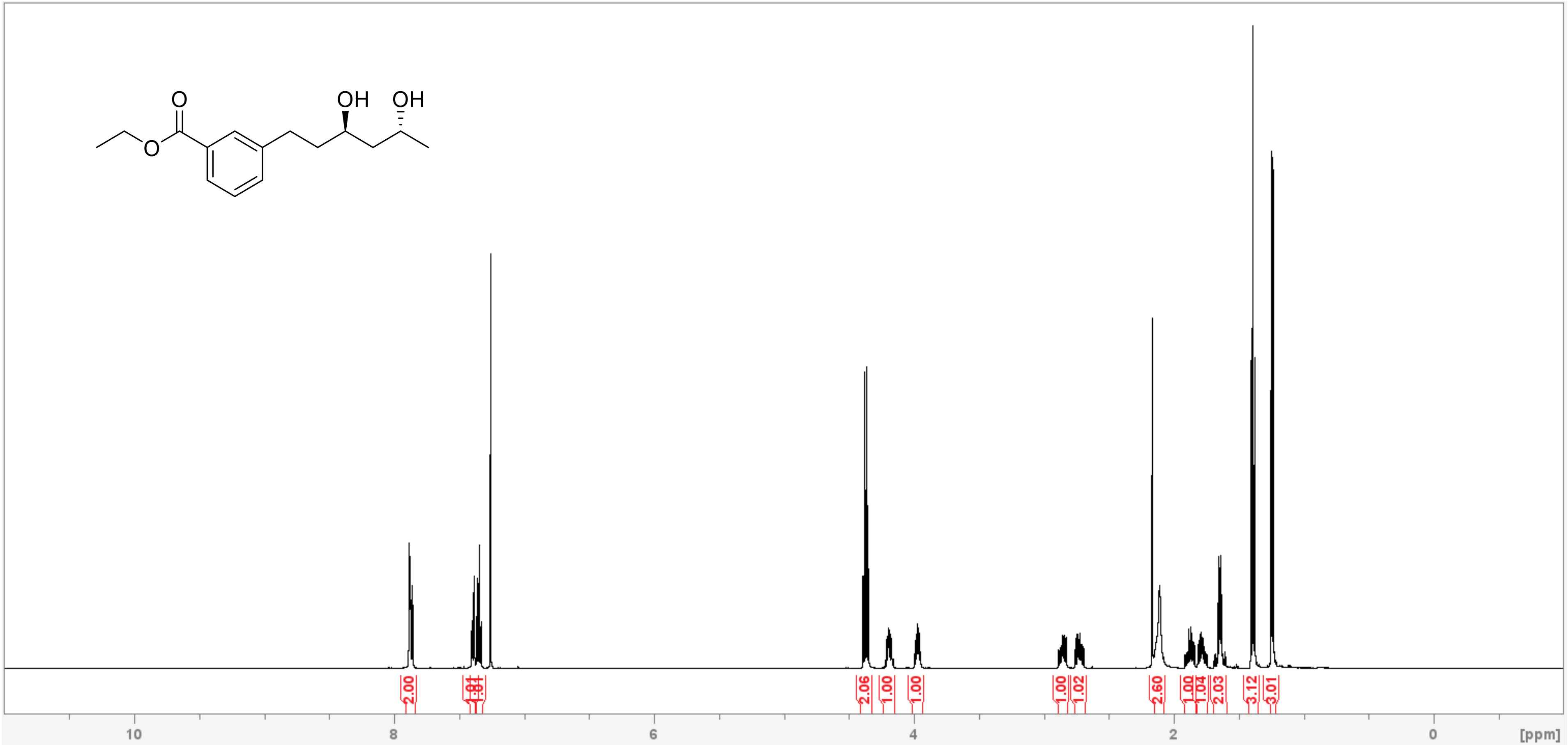

Ethyl 3-((*anti*)-3,5-dihydroxyhexyl)benzoate (*rac*-*anti*-3e)

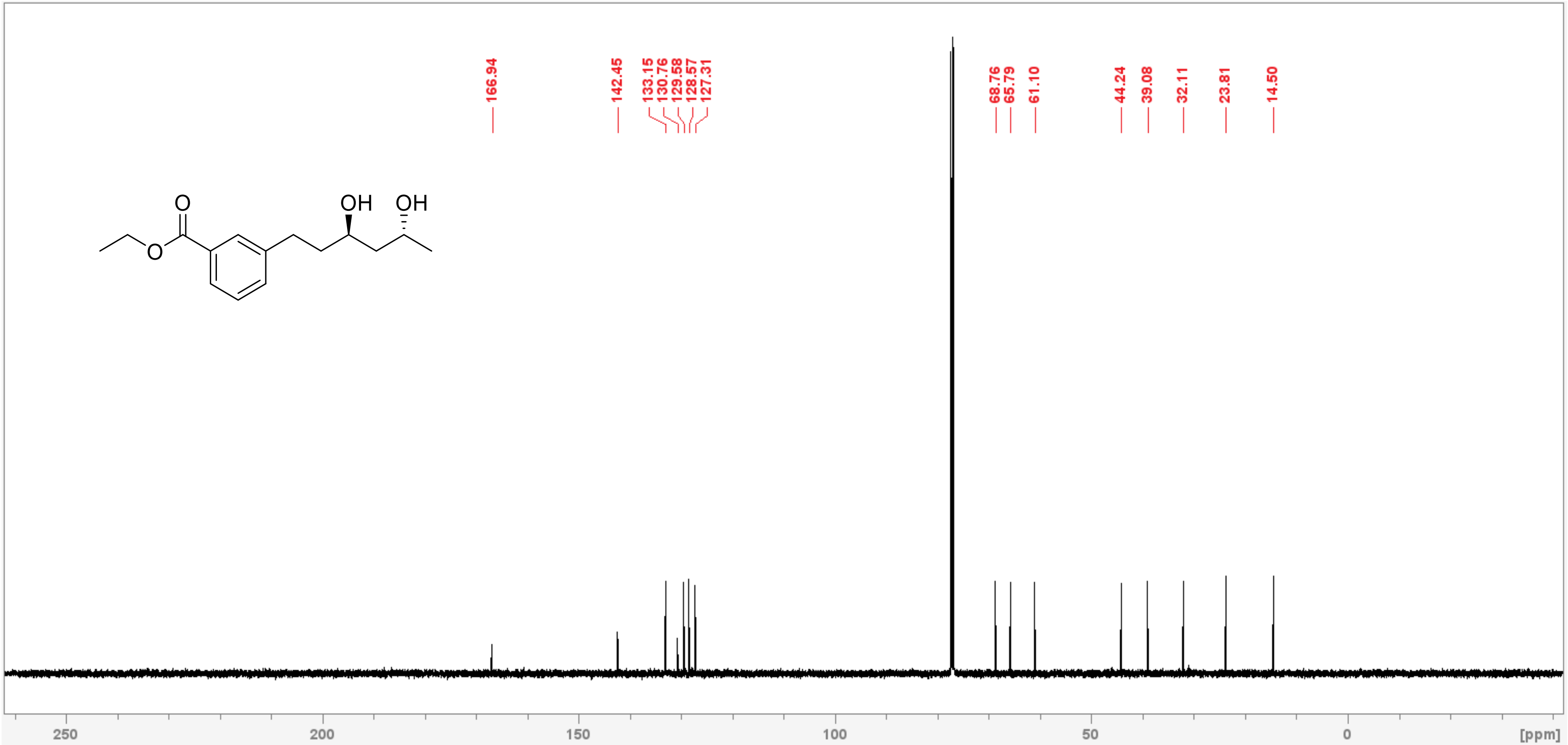

Ethyl 3-((*syn*)-3,5-dihydroxyhexyl)benzoate (*rac*-*syn*-3e)

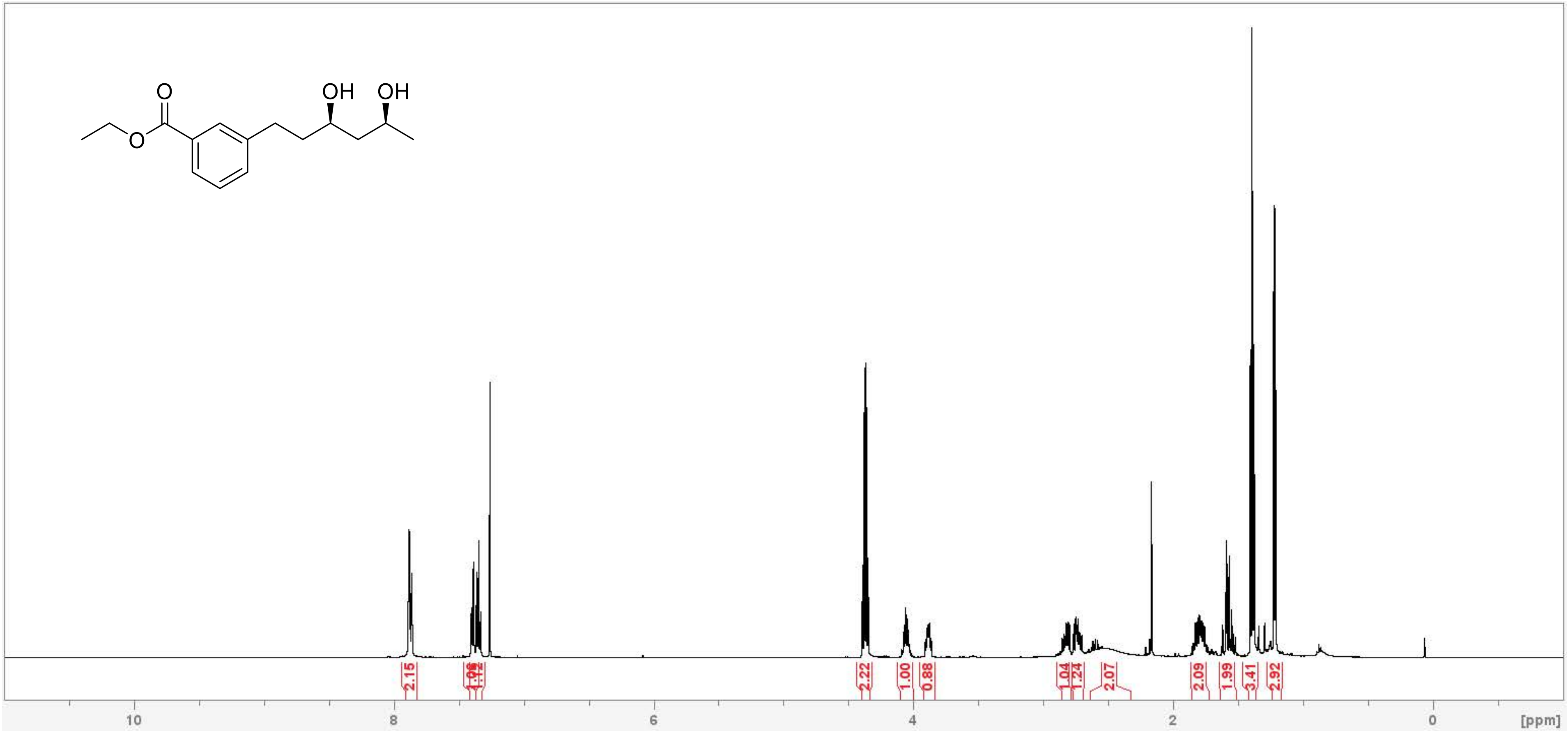

Ethyl 3-((*syn*)-3,5-dihydroxyhexyl)benzoate (*rac*-*syn*-3e)

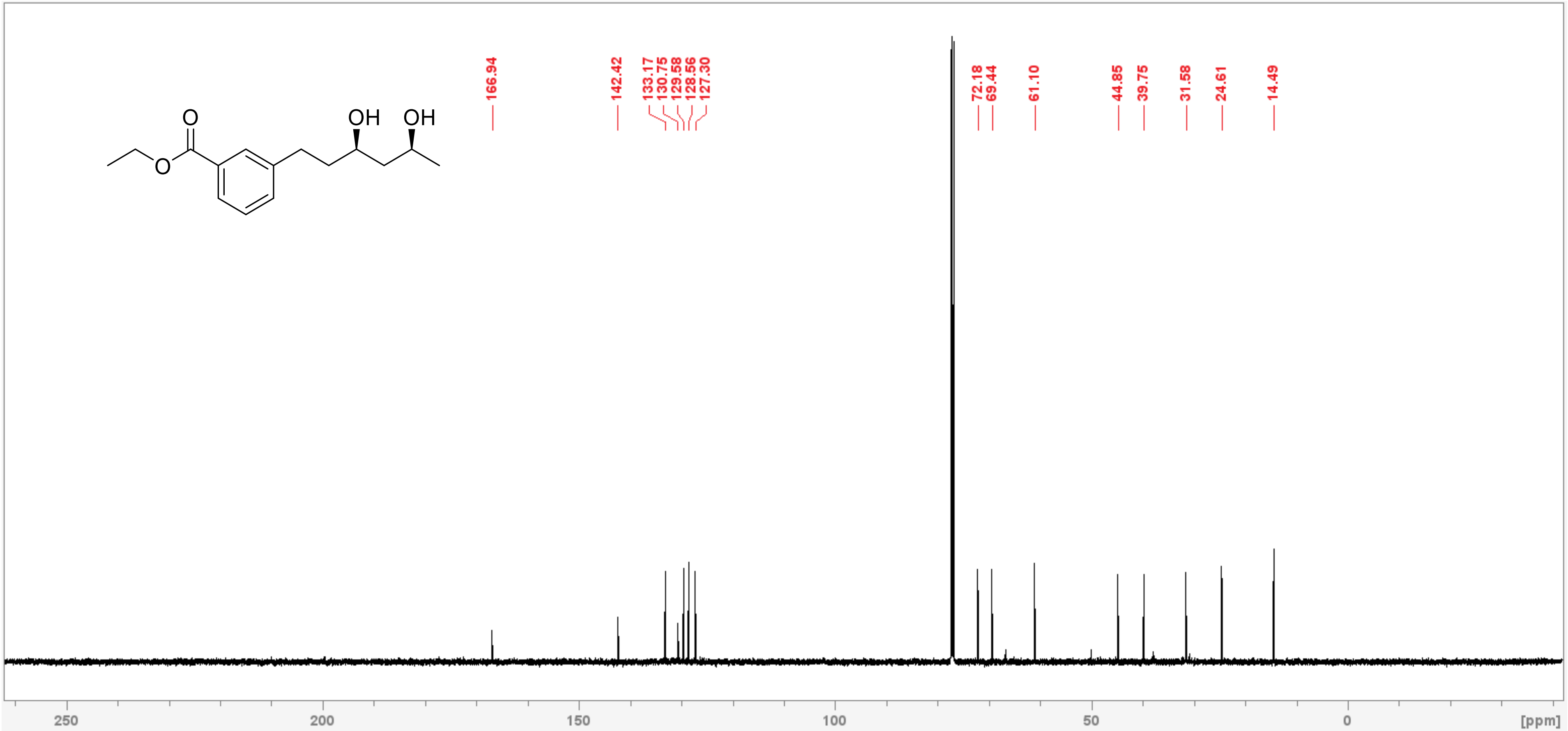

4-Hydroxy-6-(2-(trifluoromethyl)phenyl)hexan-2-one (*int₁*-3f)

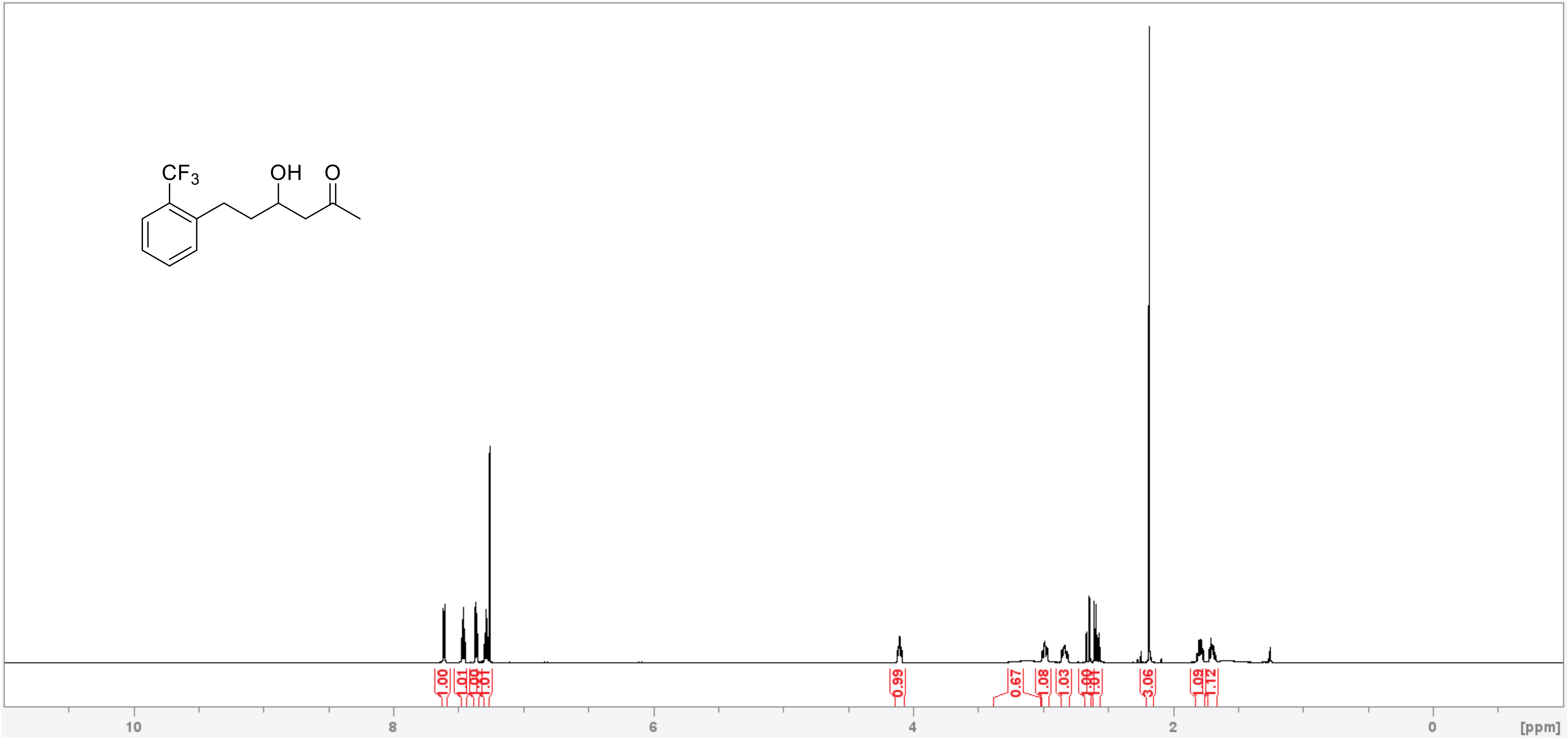

4-Hydroxy-6-(2-(trifluoromethyl)phenyl)hexan-2-one (*int₁*-3f)

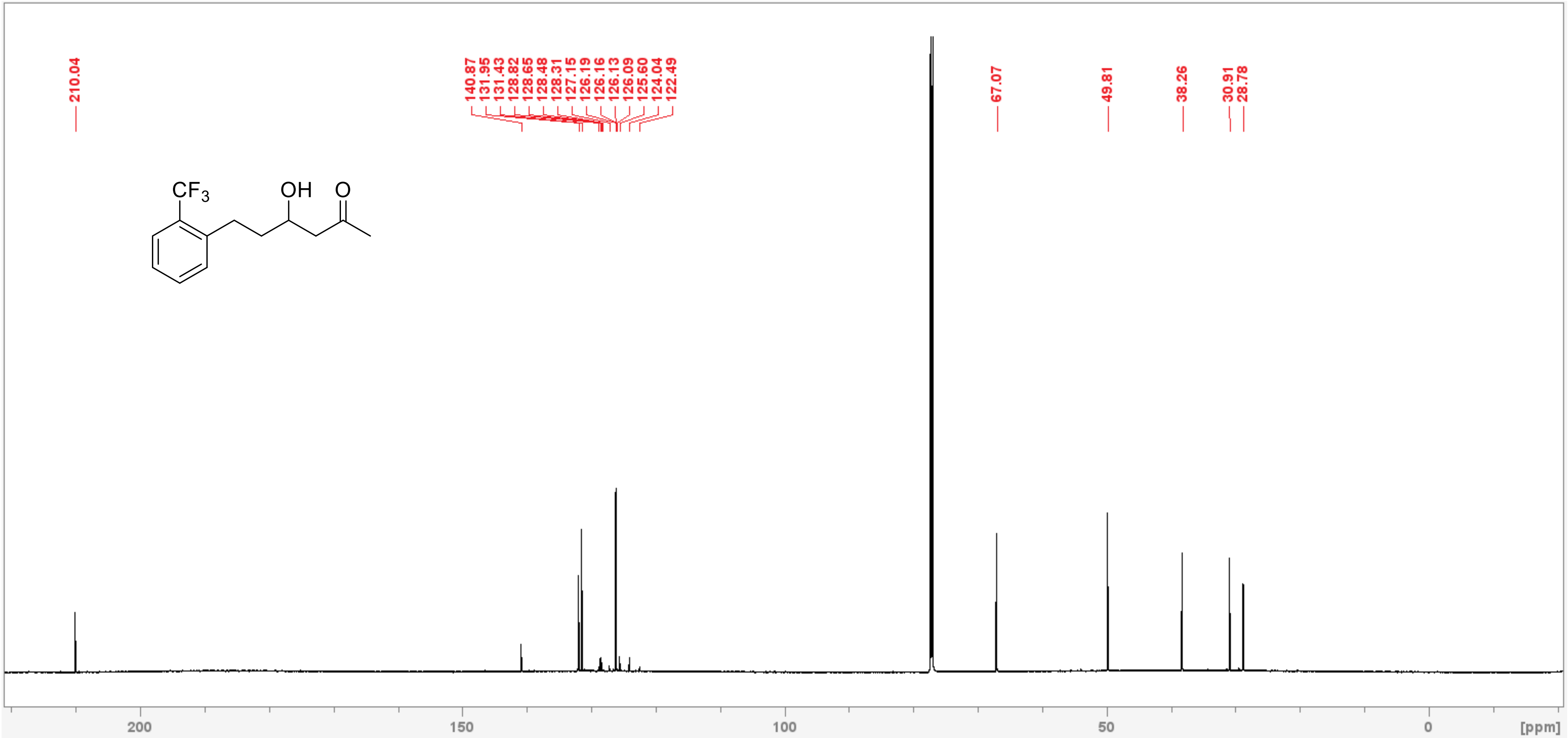

4-Hydroxy-6-(2-(trifluoromethyl)phenyl)hexan-2-one (*int₁*-3f)

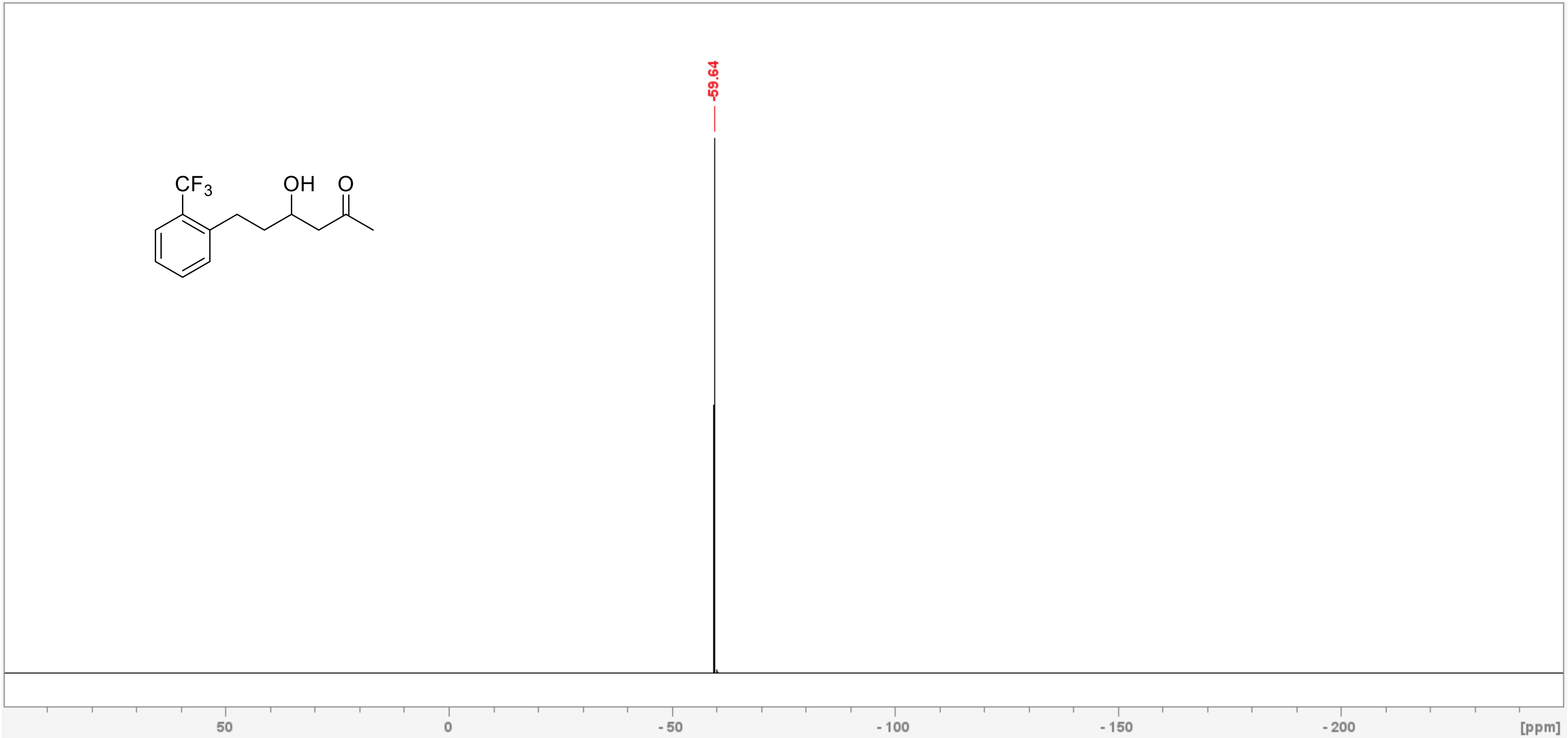

(*anti*)-6-(2-(Trifluoromethyl)phenyl)hexane-2,4-diol (*rac-anti*-3f)

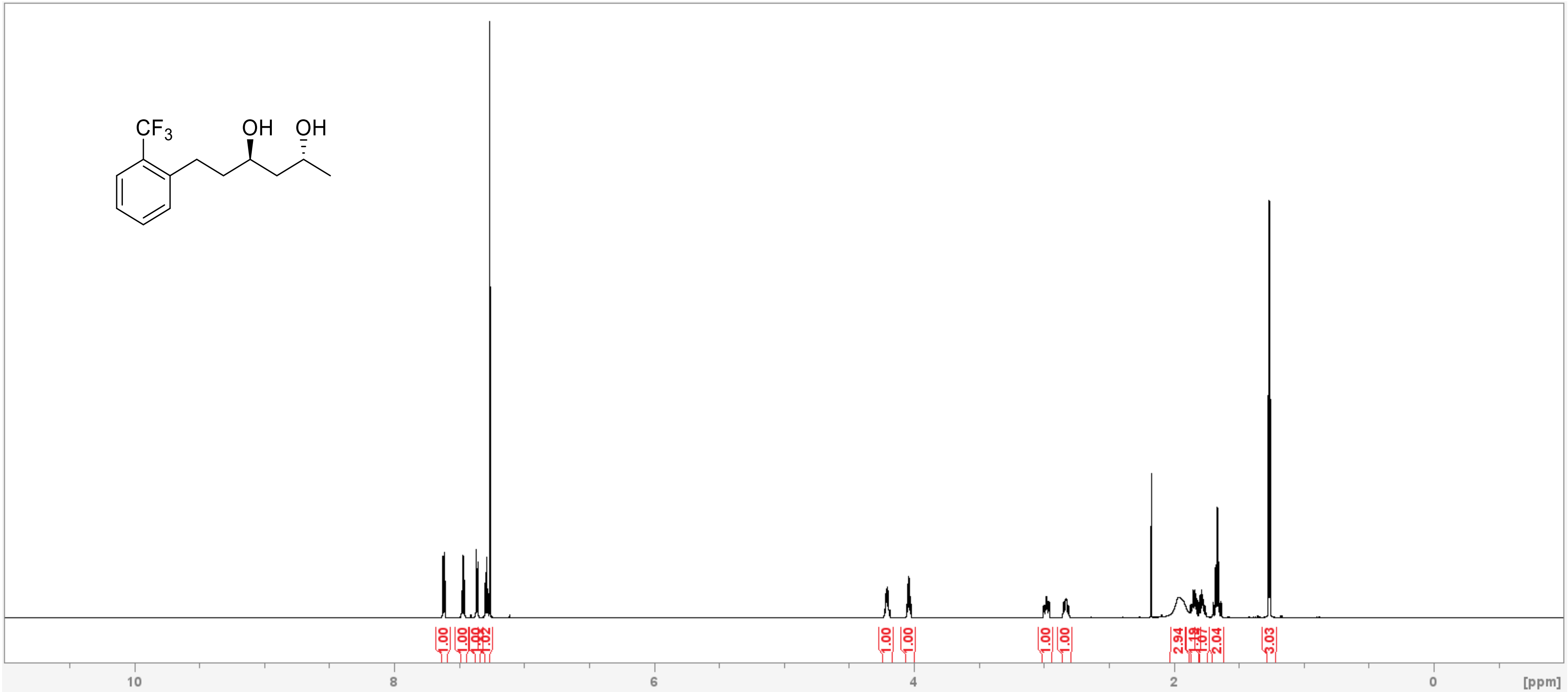

(*anti*)-6-(2-(Trifluoromethyl)phenyl)hexane-2,4-diol (*rac-anti*-3f)

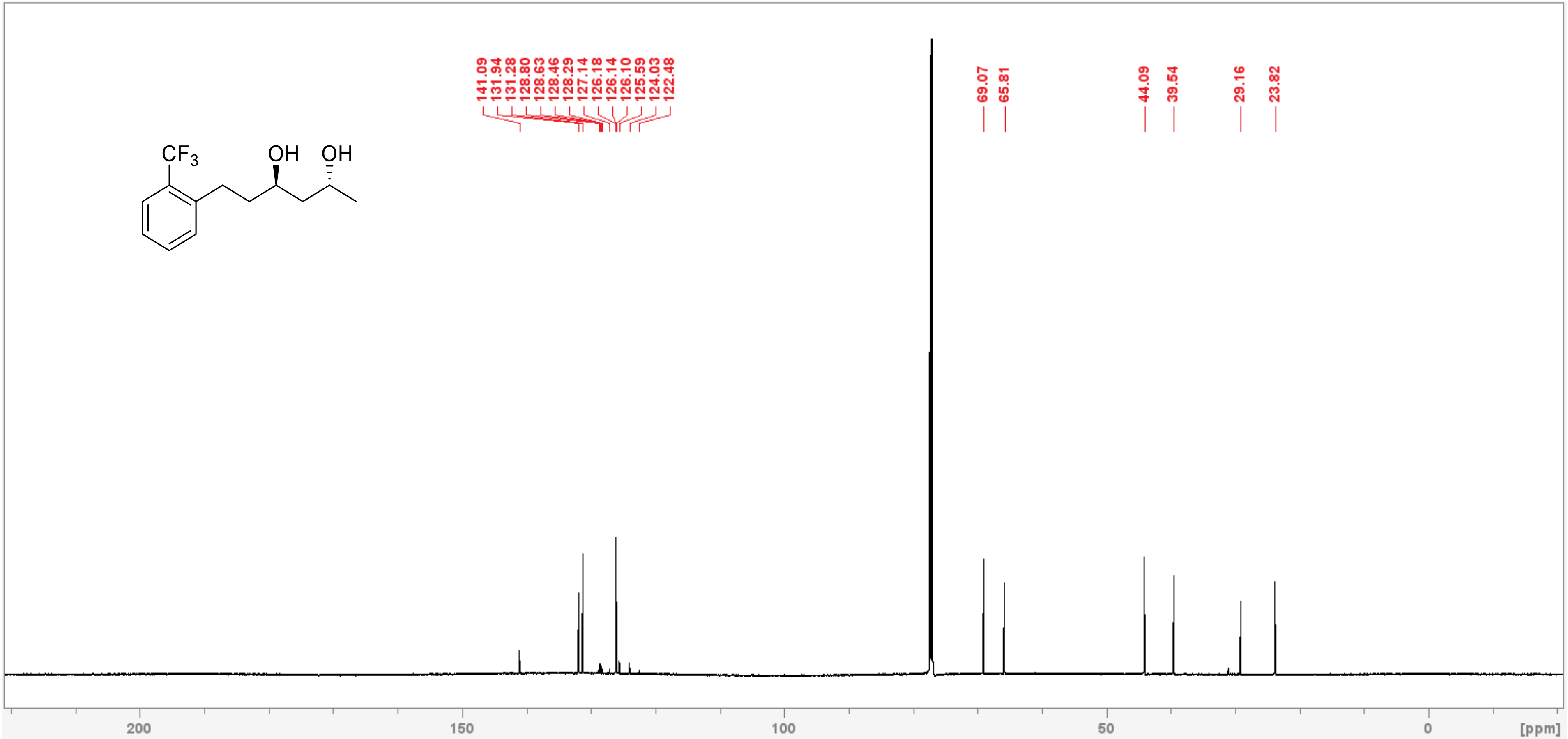

(*anti*)-6-(2-(Trifluoromethyl)phenyl)hexane-2,4-diol (*rac*-*anti*-3f)

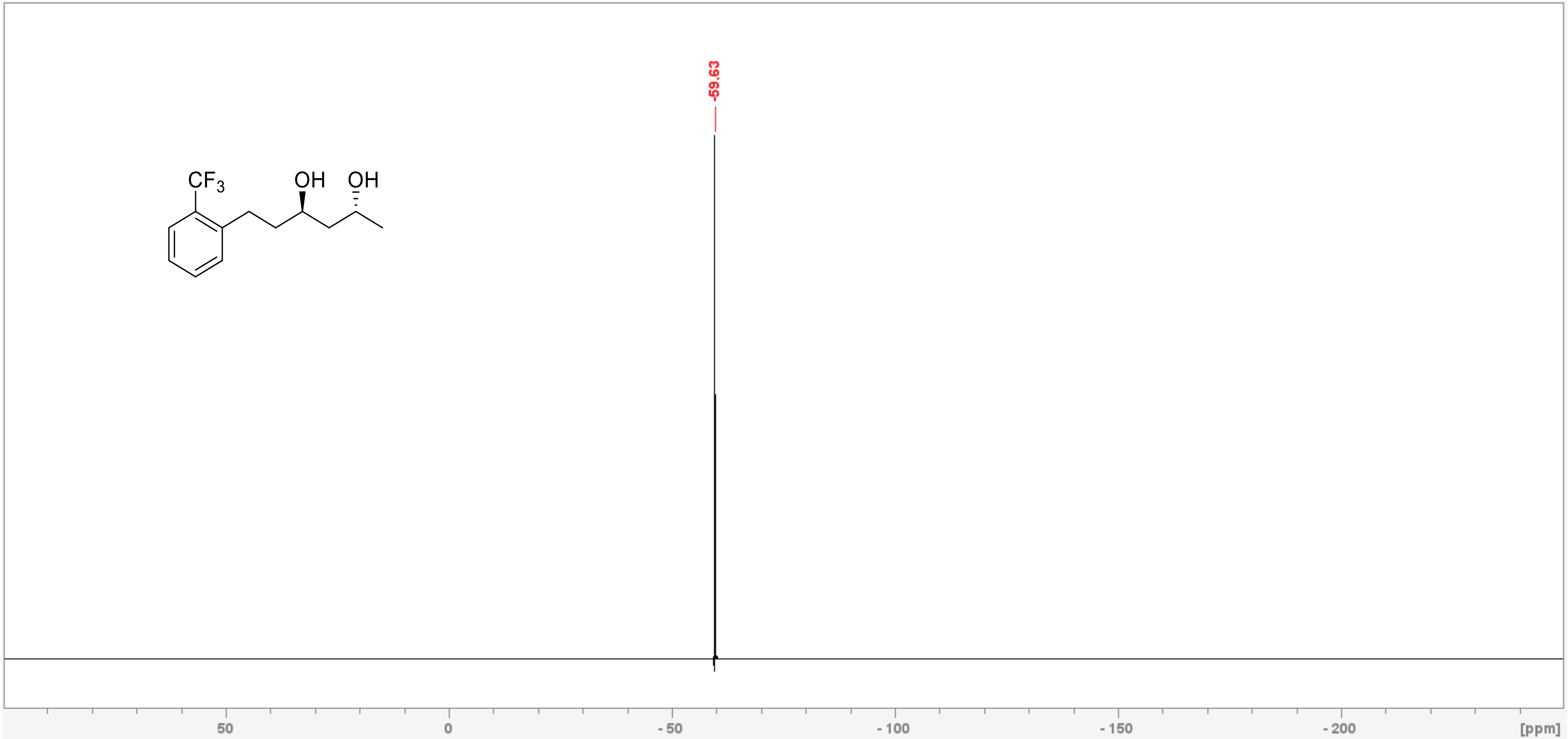

(*syn*)-6-(2-(Trifluoromethyl)phenyl)hexane-2,4-diol (*rac*-*syn*-3f)

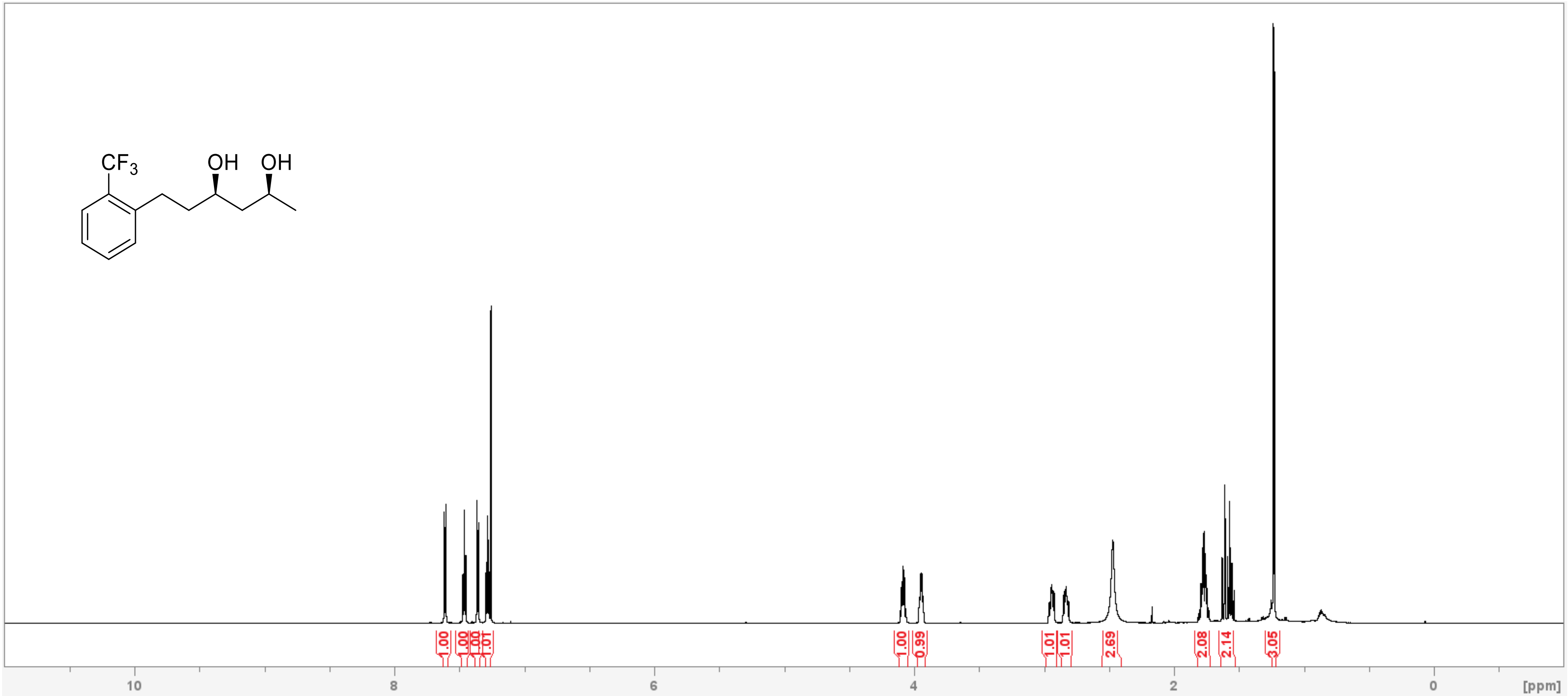

(*syn*)-6-(2-(Trifluoromethyl)phenyl)hexane-2,4-diol (*rac*-*syn*-3f)

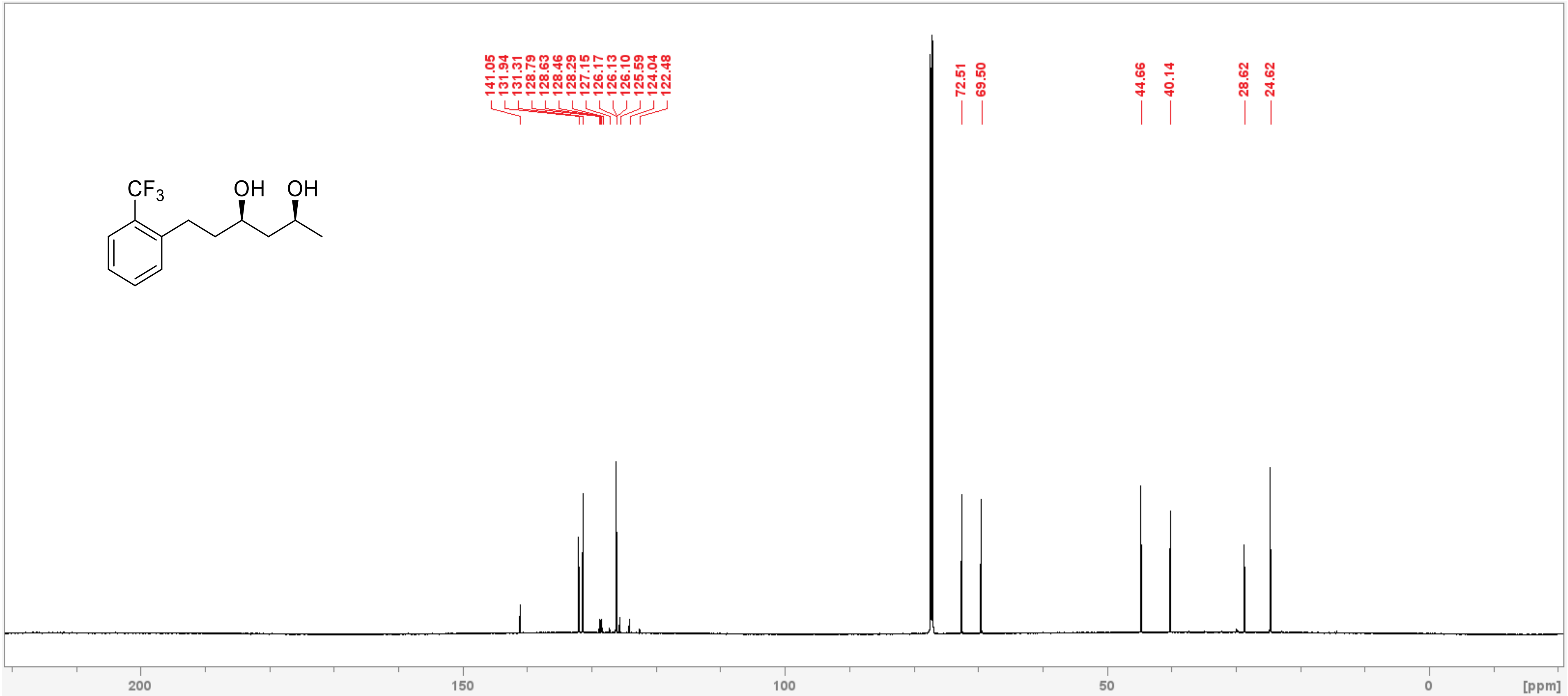

(*syn*)-6-(2-(Trifluoromethyl)phenyl)hexane-2,4-diol (*rac*-*syn*-3f)

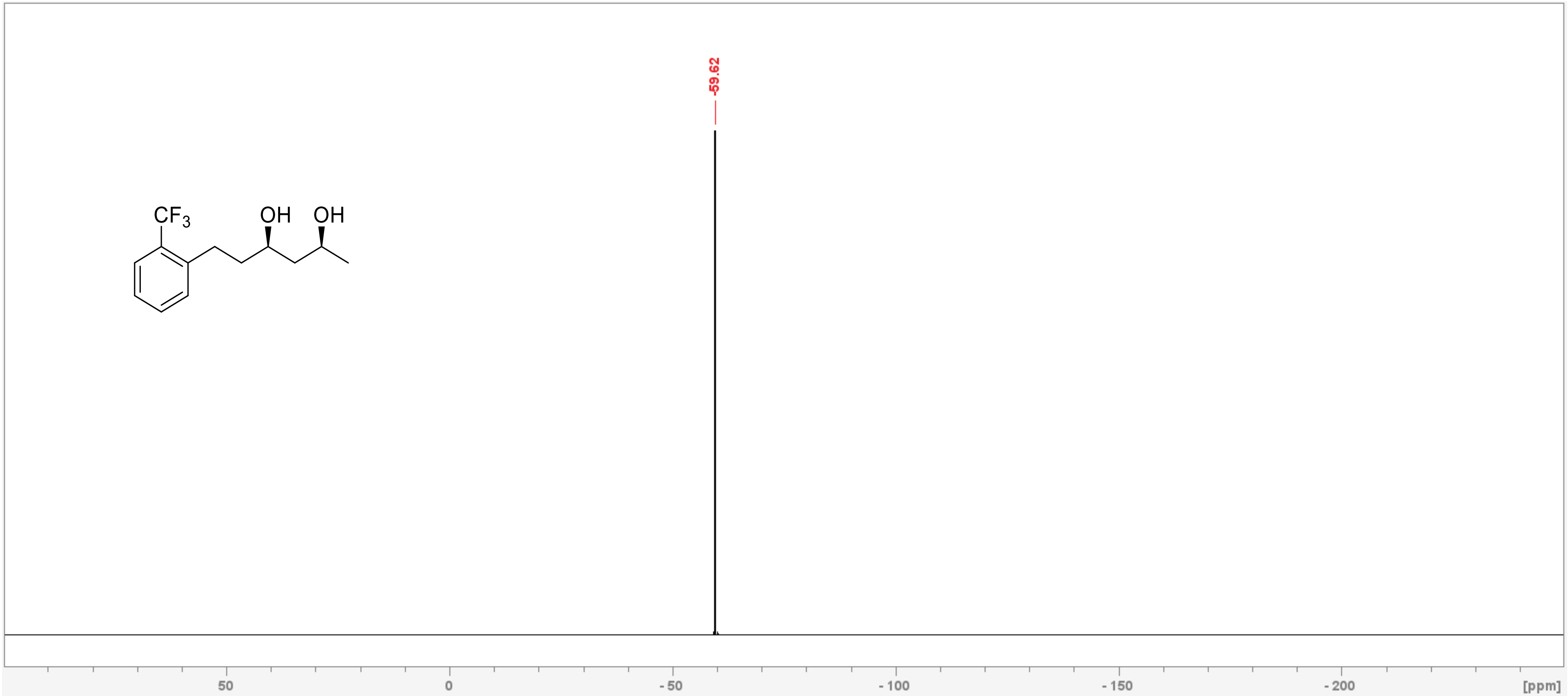

4-(3-Hydroxy-5-oxohexyl)benzonitrile (*int*$_1$-3g)

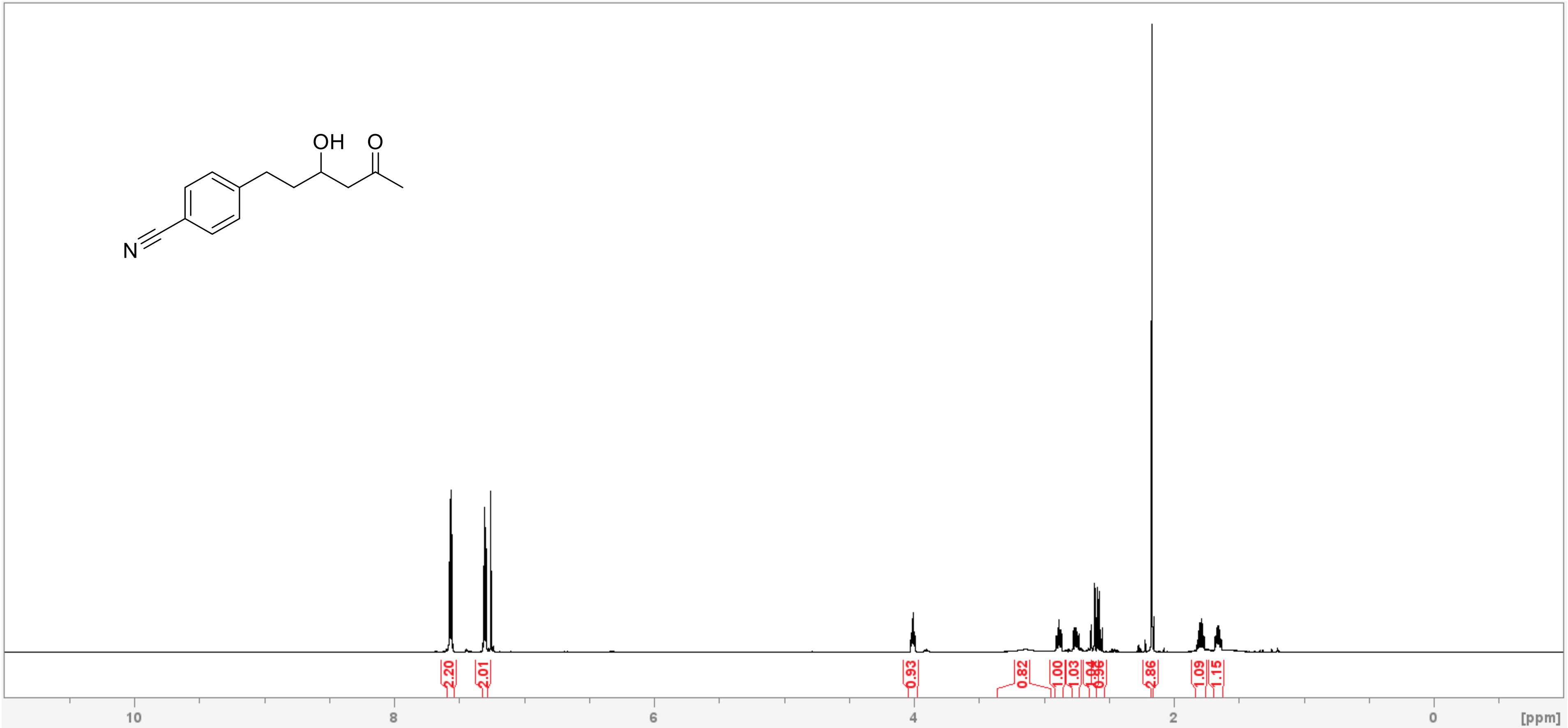

4-(3-Hydroxy-5-oxohexyl)benzonitrile (*int₁*-3g)

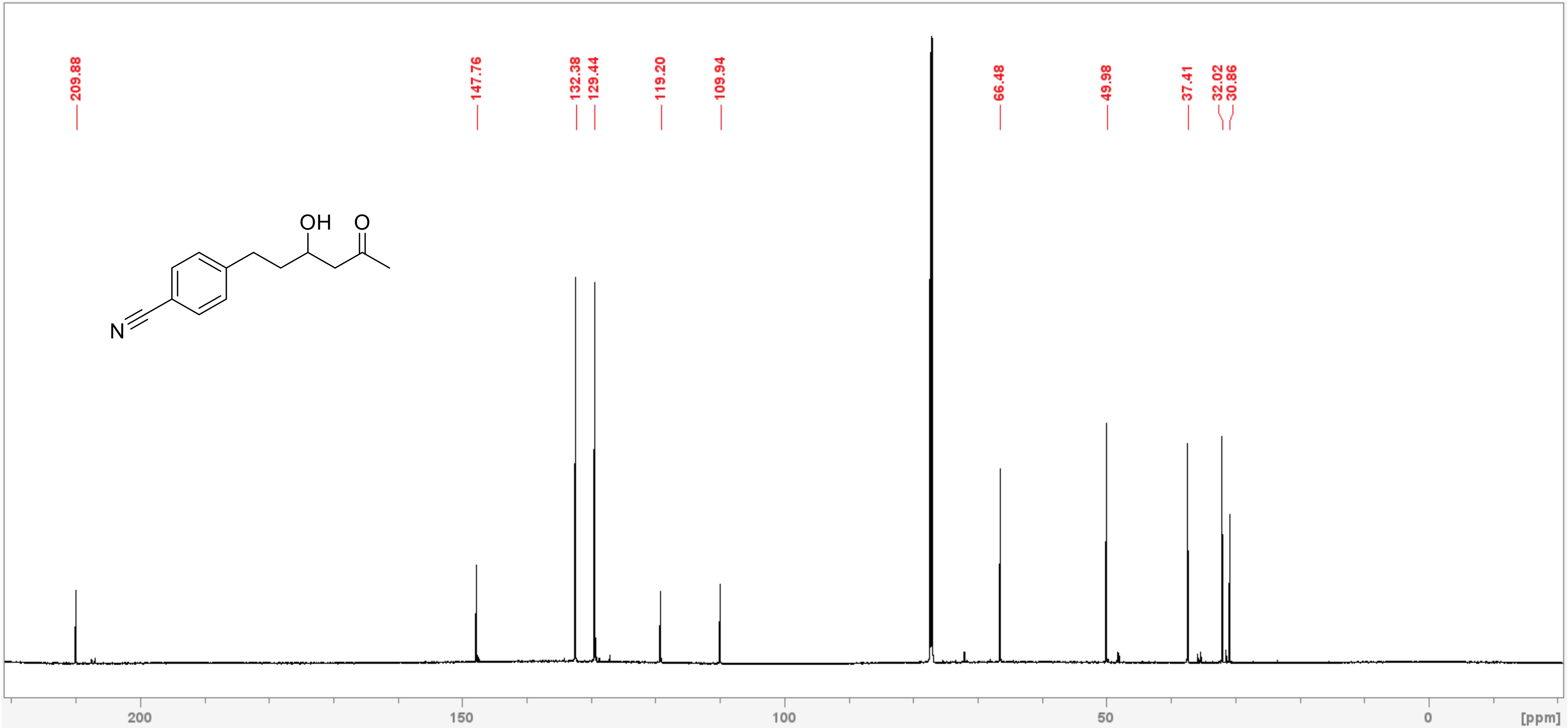

4-((*anti*)-3,5-Dihydroxyhexyl)benzonitrile (*rac-anti*-3g)

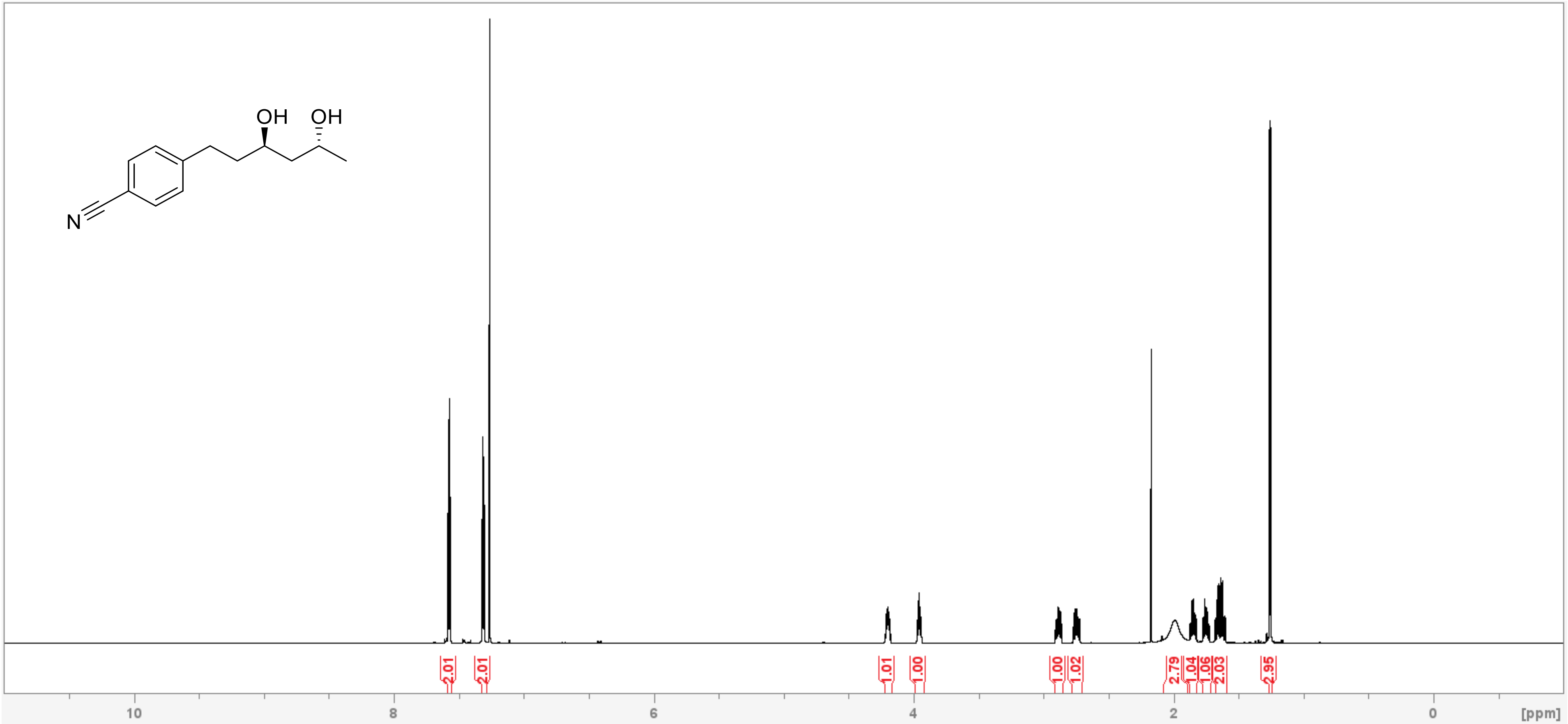

4-((*anti*)-3,5-Dihydroxyhexyl)benzonitrile (*rac*-*anti*-3g)

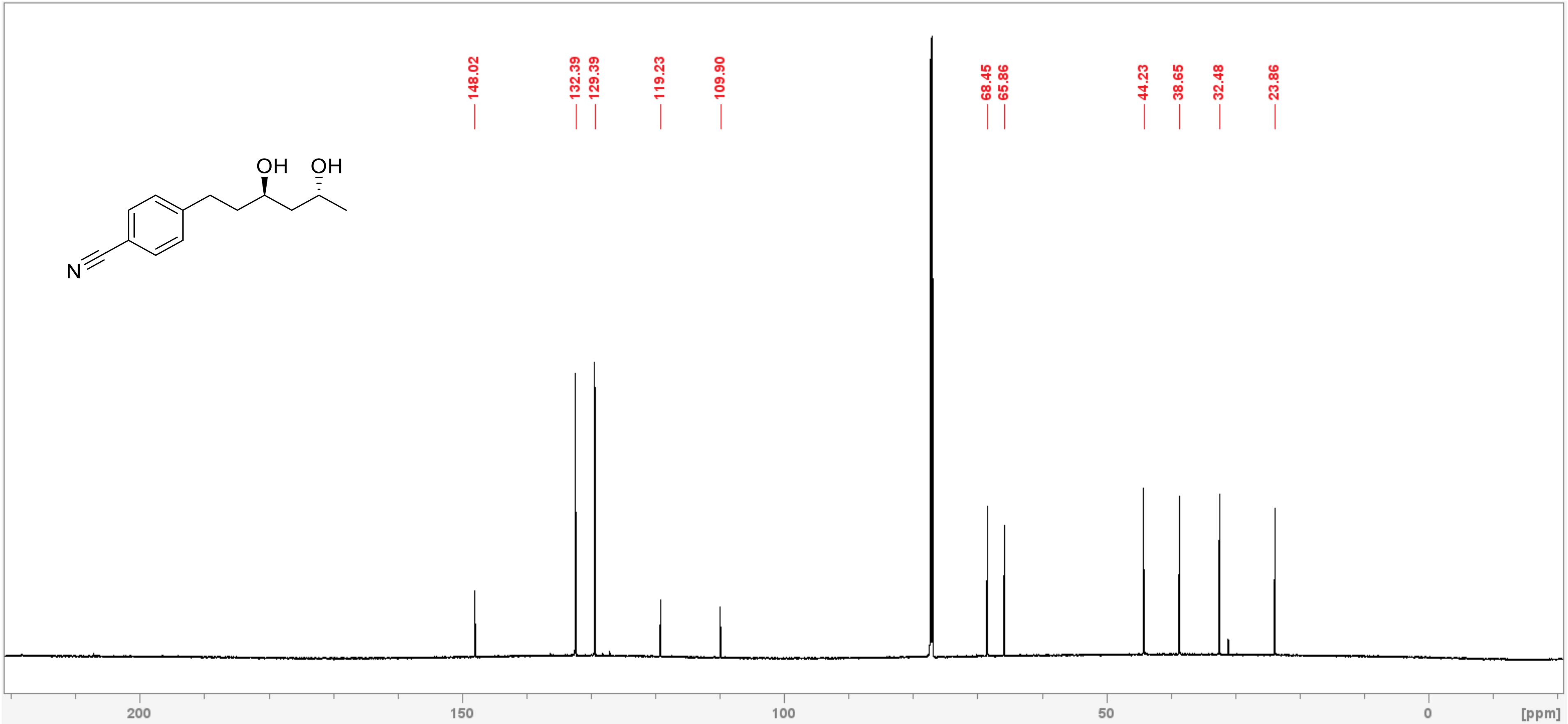

4-((*syn*)-3,5-Dihydroxyhexyl)benzonitrile (*rac*-*syn*-3g)

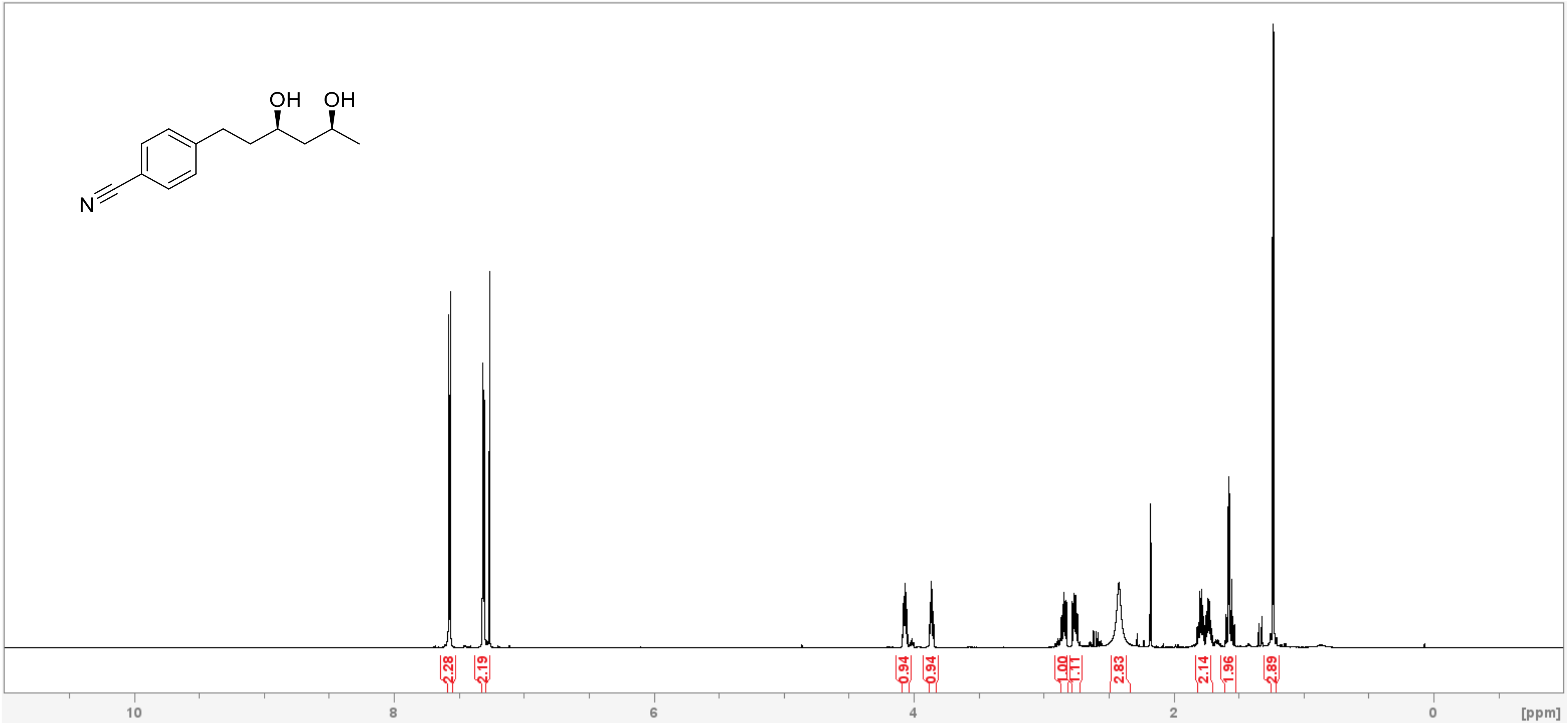

4-((*syn*)-3,5-Dihydroxyhexyl)benzonitrile (*rac*-*syn*-3g)

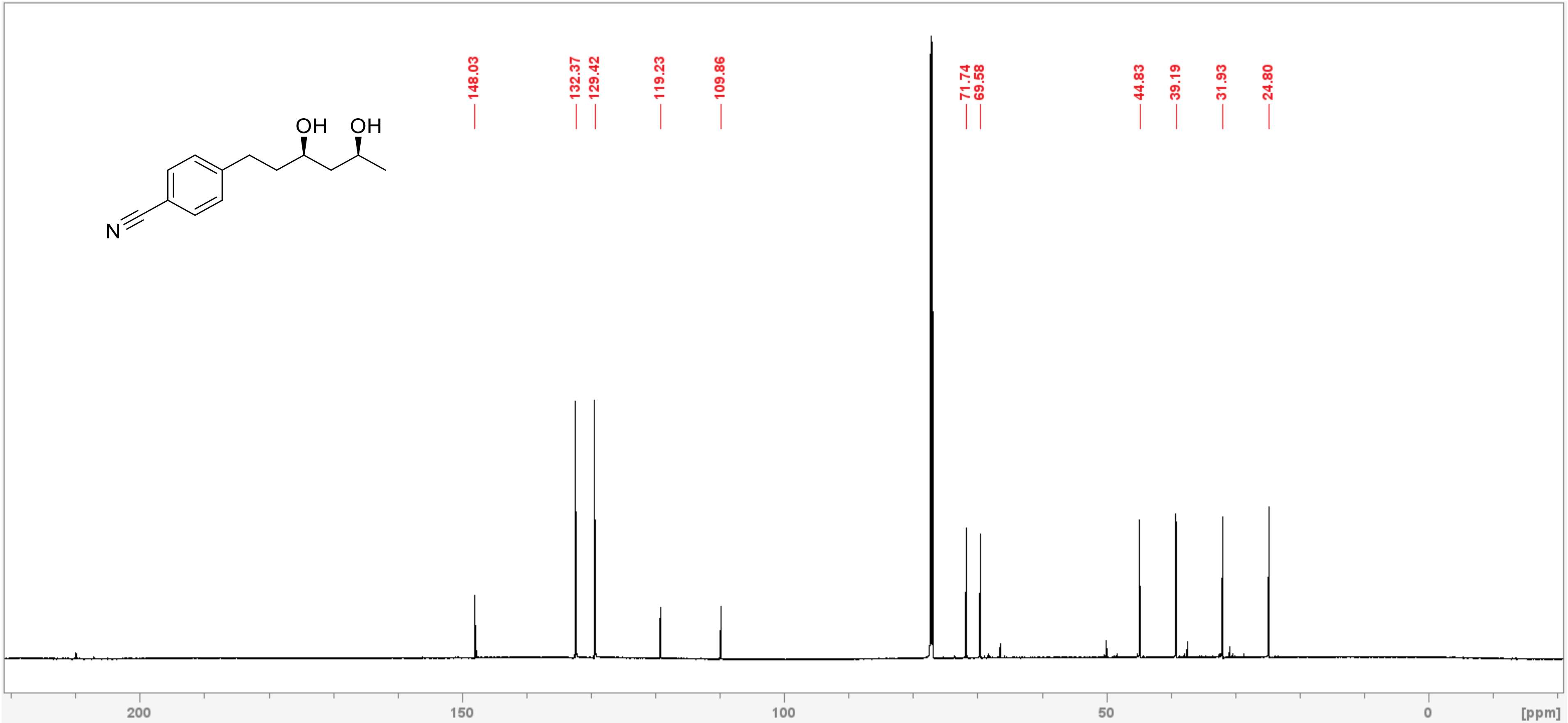

2,2,2-trifluoro-*N*-(4-(3-oxopropyl)phenyl)acetamide (*int₁*-3h)

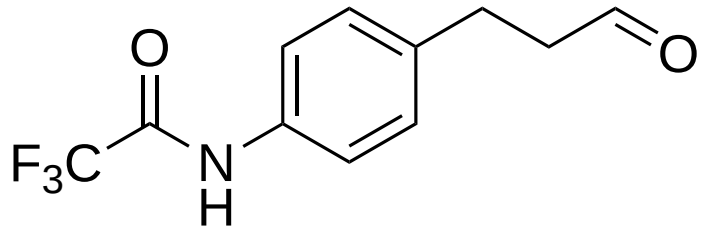

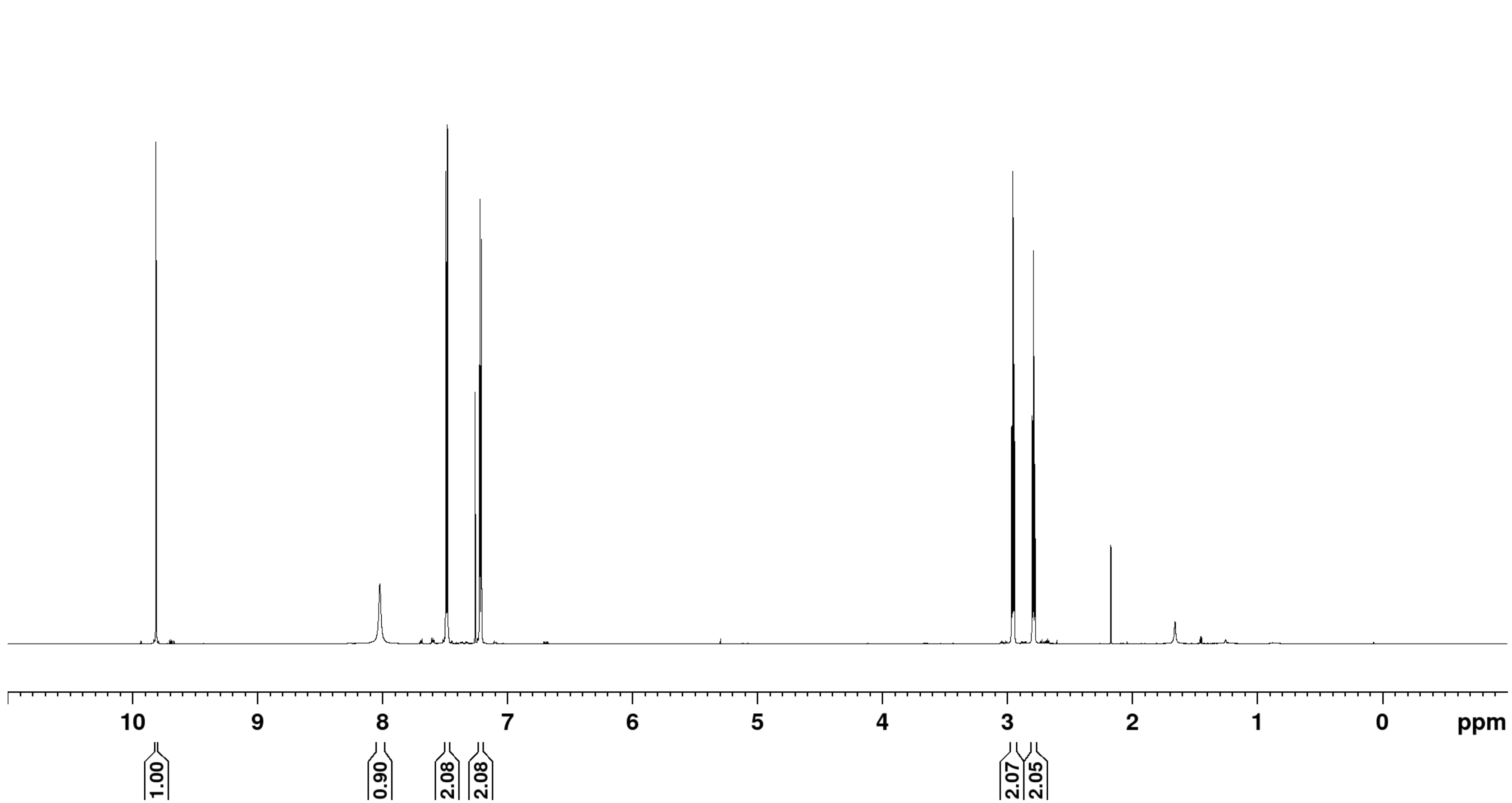

2,2,2-trifluoro-*N*-(4-(3-oxopropyl)phenyl)acetamide (*int$_1$*-3h)

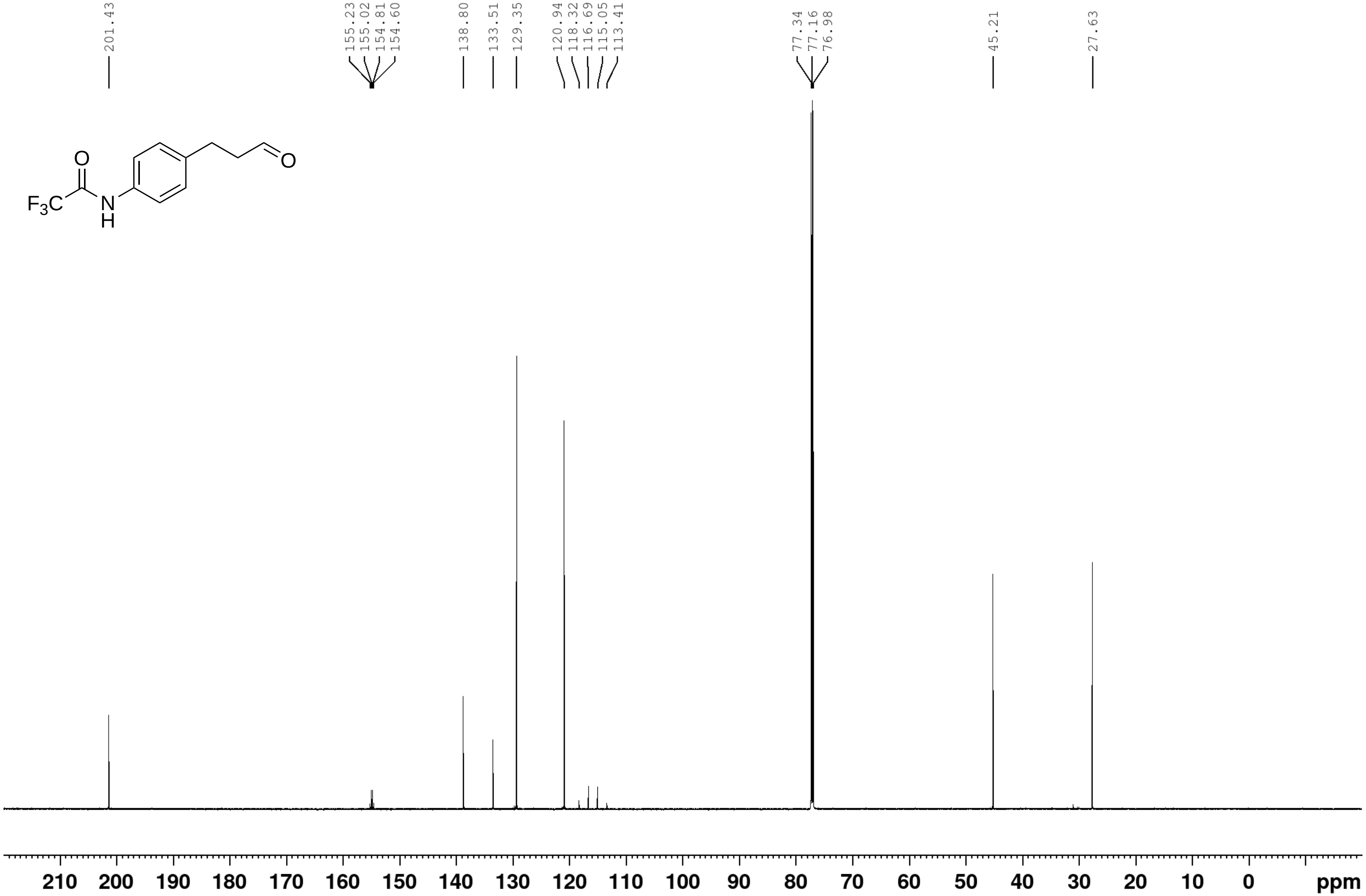

2,2,2-trifluoro-*N*-(4-(3-hydroxy-5-oxohexyl)phenyl)acetamide (*int₂*-3h)

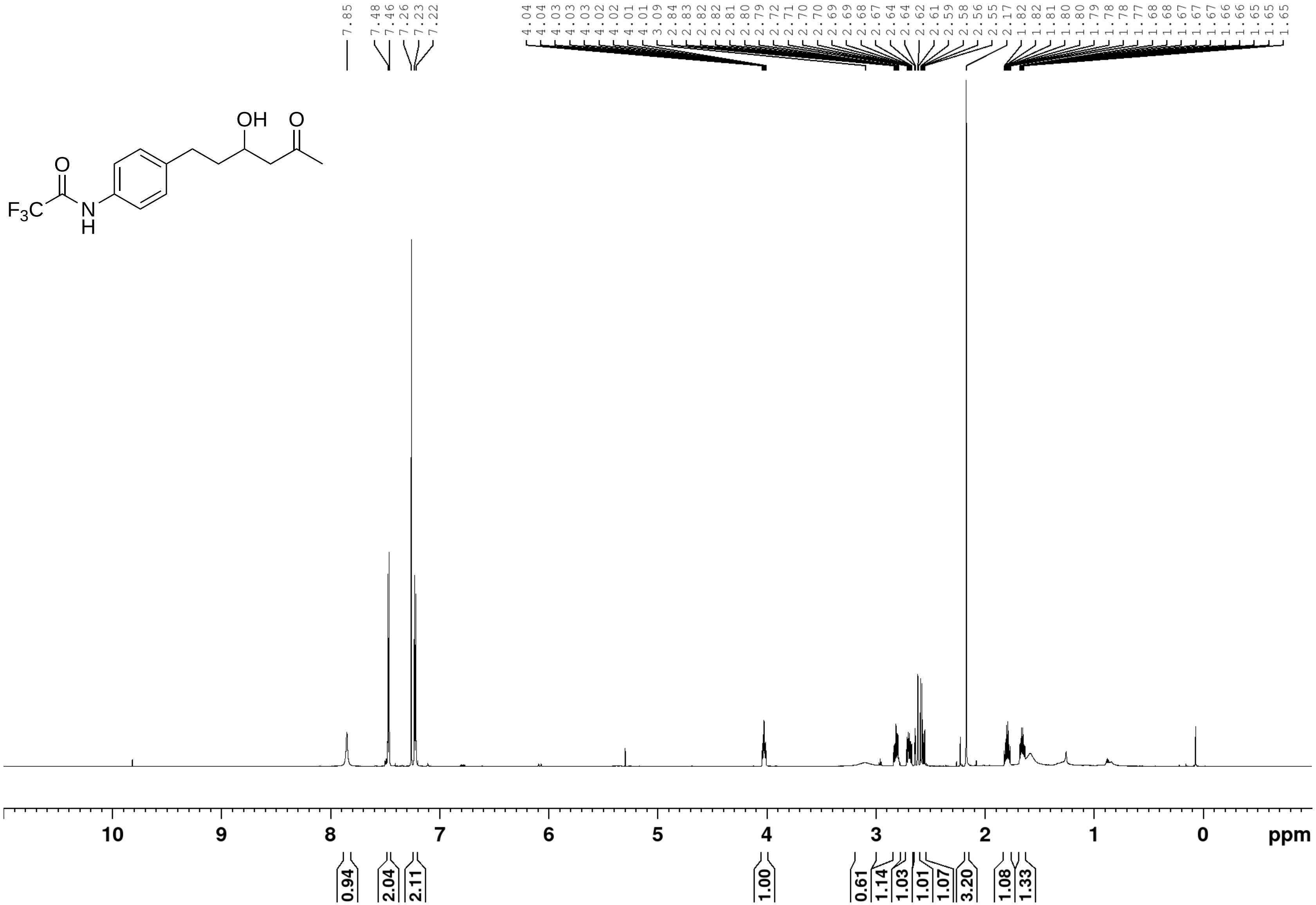

2,2,2-trifluoro-*N*-(4-(3-hydroxy-5-oxohexyl)phenyl)acetamide (*int*$_2$-3h)

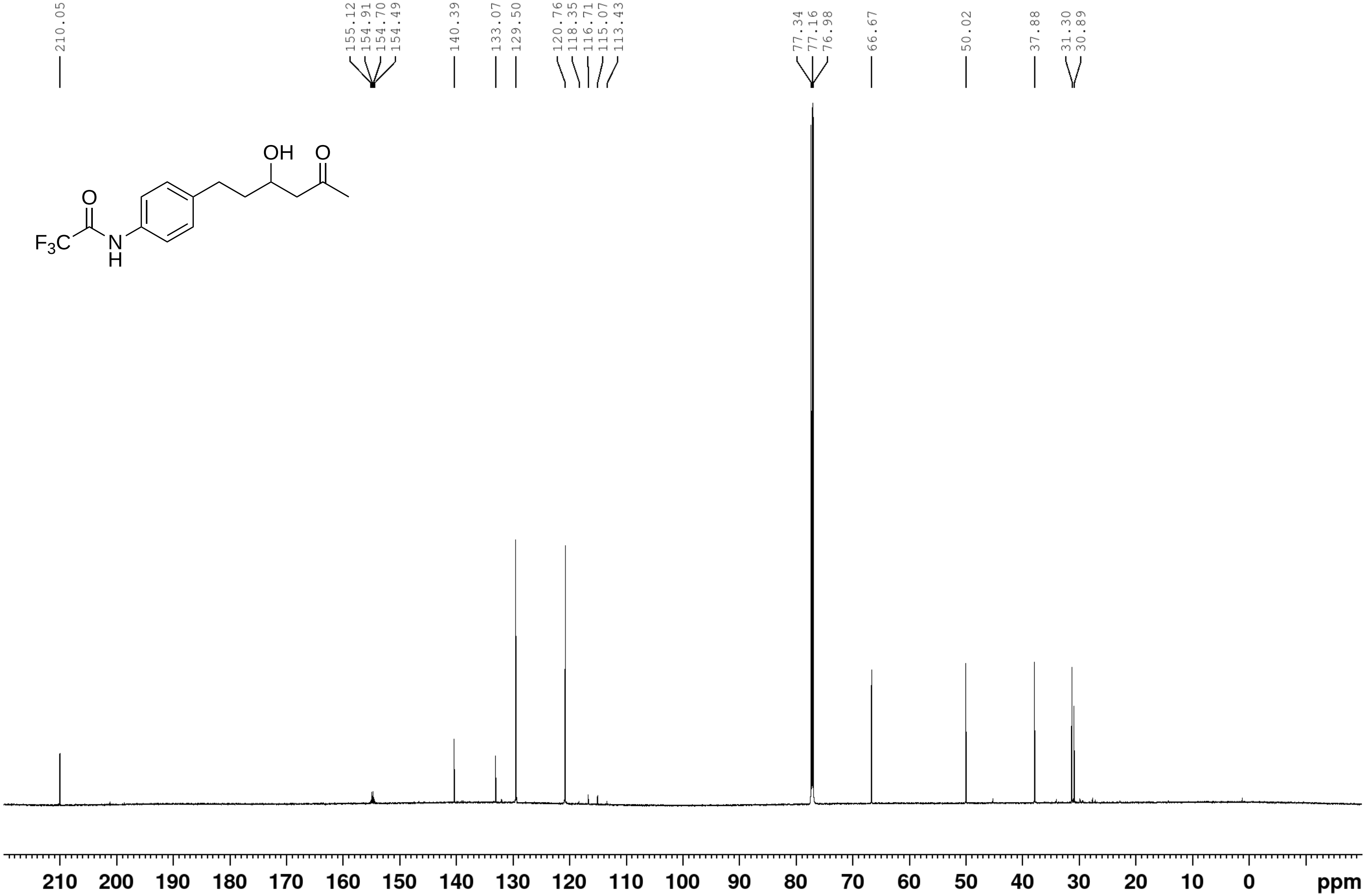

(*syn*)-*N*-(4-(3,5-dihydroxyhexyl)phenyl)-2,2,2-trifluoroacetamide (*rac*-*syn*-3h)

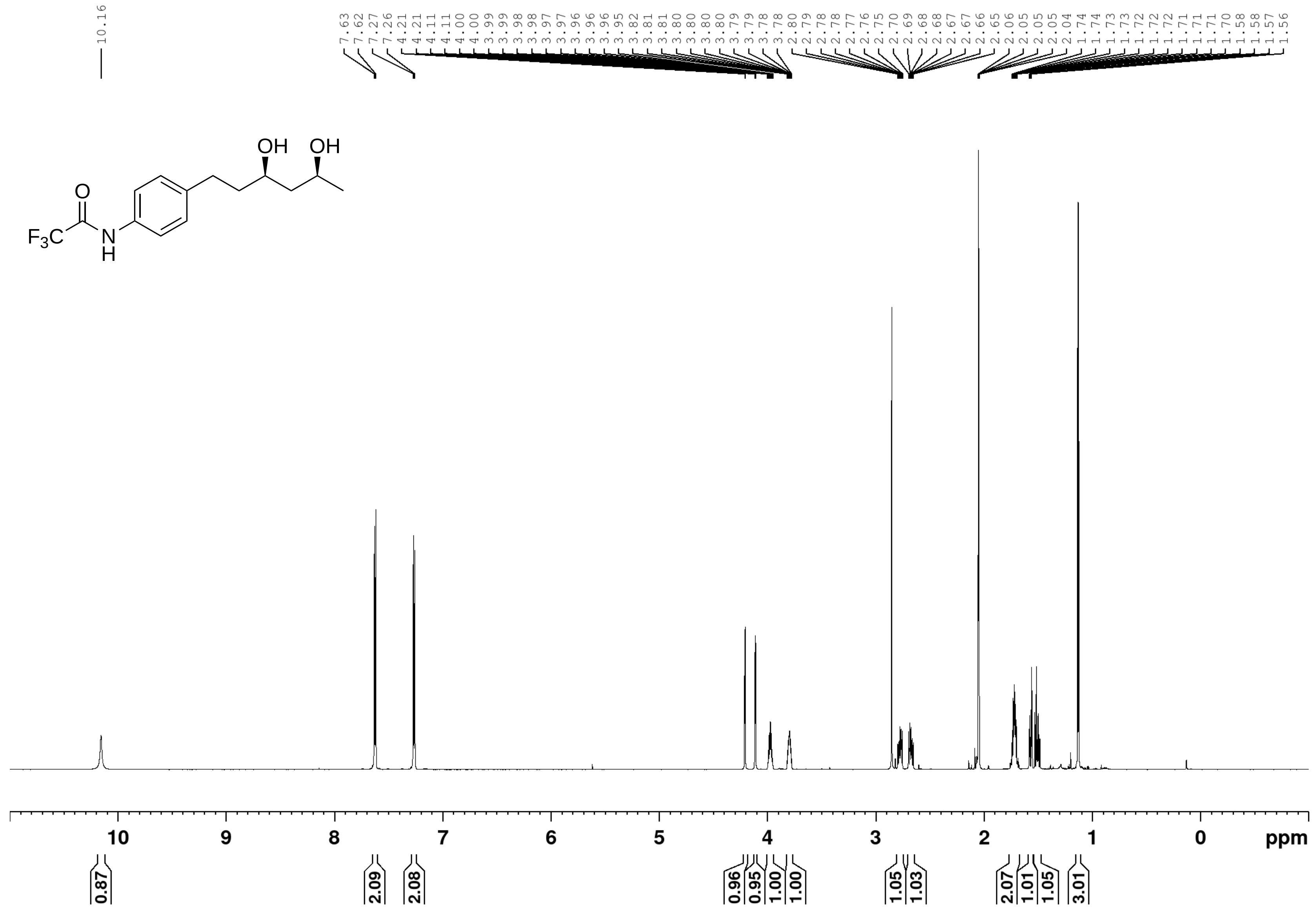

(*syn*)-*N*-(4-(3,5-dihydroxyhexyl)phenyl)-2,2,2-trifluoroacetamide (*rac*-*syn*-3h)

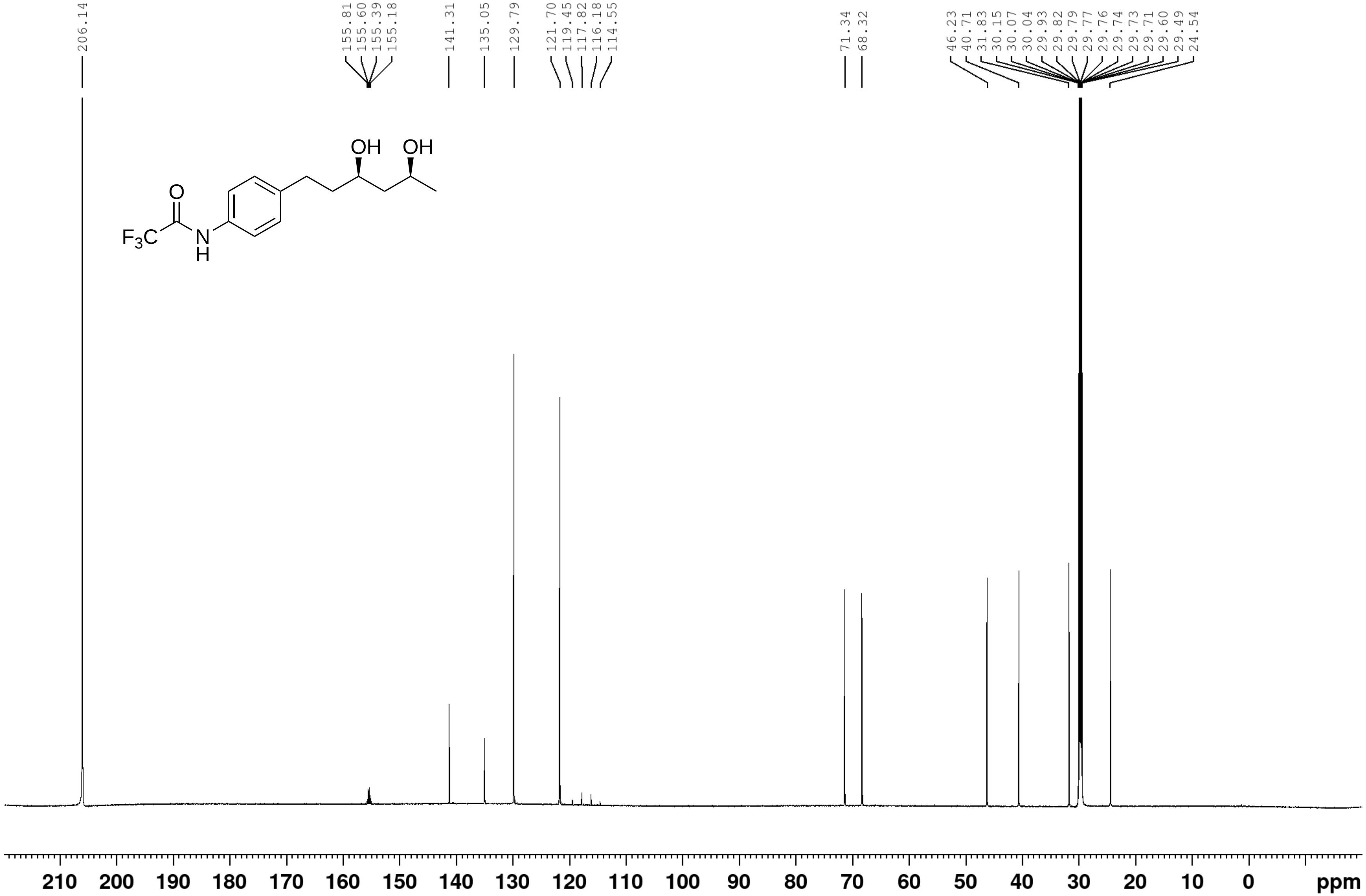

(*anti*)-*N*-(4-(3,5-dihydroxyhexyl)phenyl)-2,2,2-trifluoroacetamide (*rac-anti*-3h)

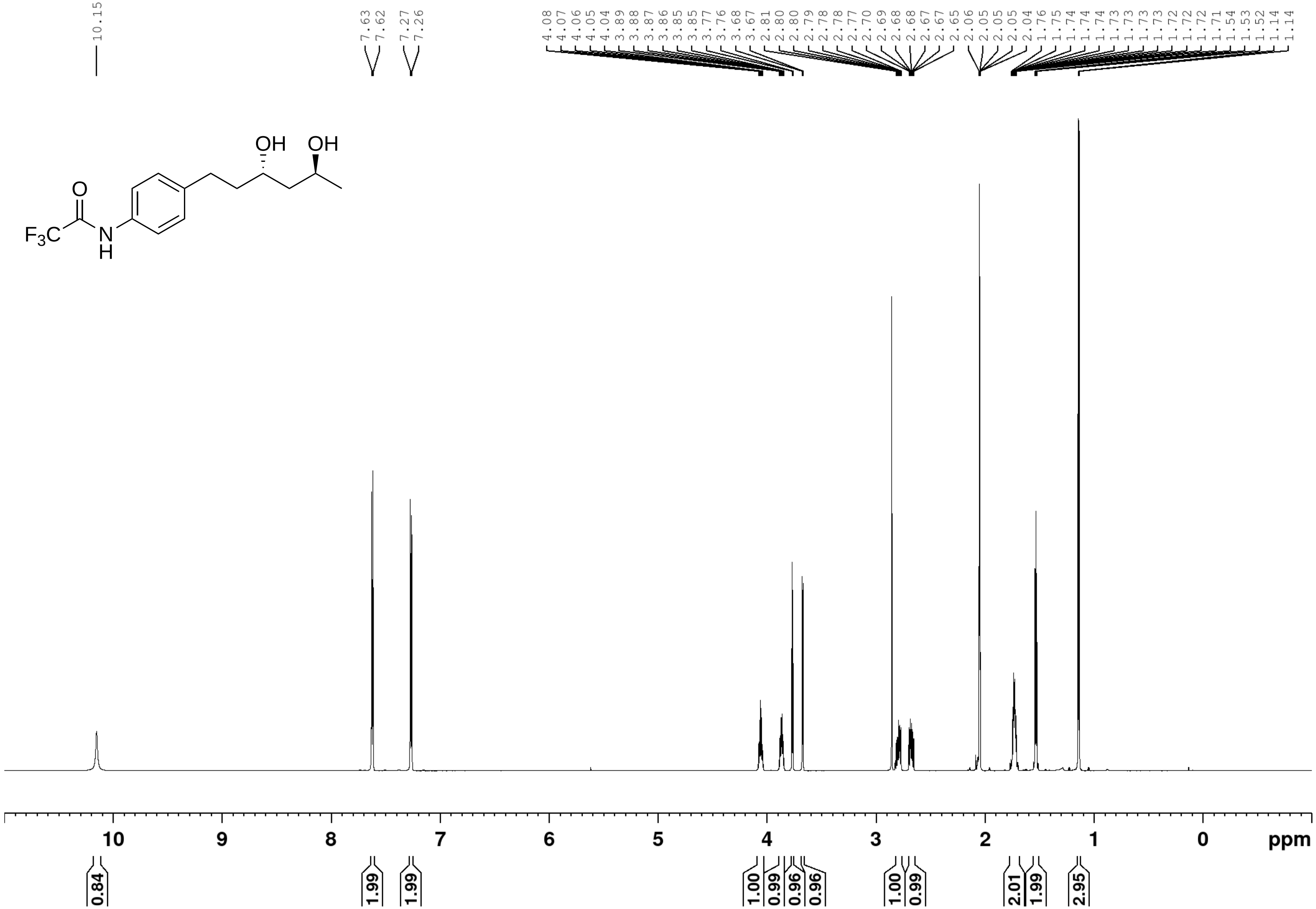

(*anti*)-*N*-(4-(3,5-dihydroxyhexyl)phenyl)-2,2,2-trifluoroacetamide (*rac-anti*-3h)

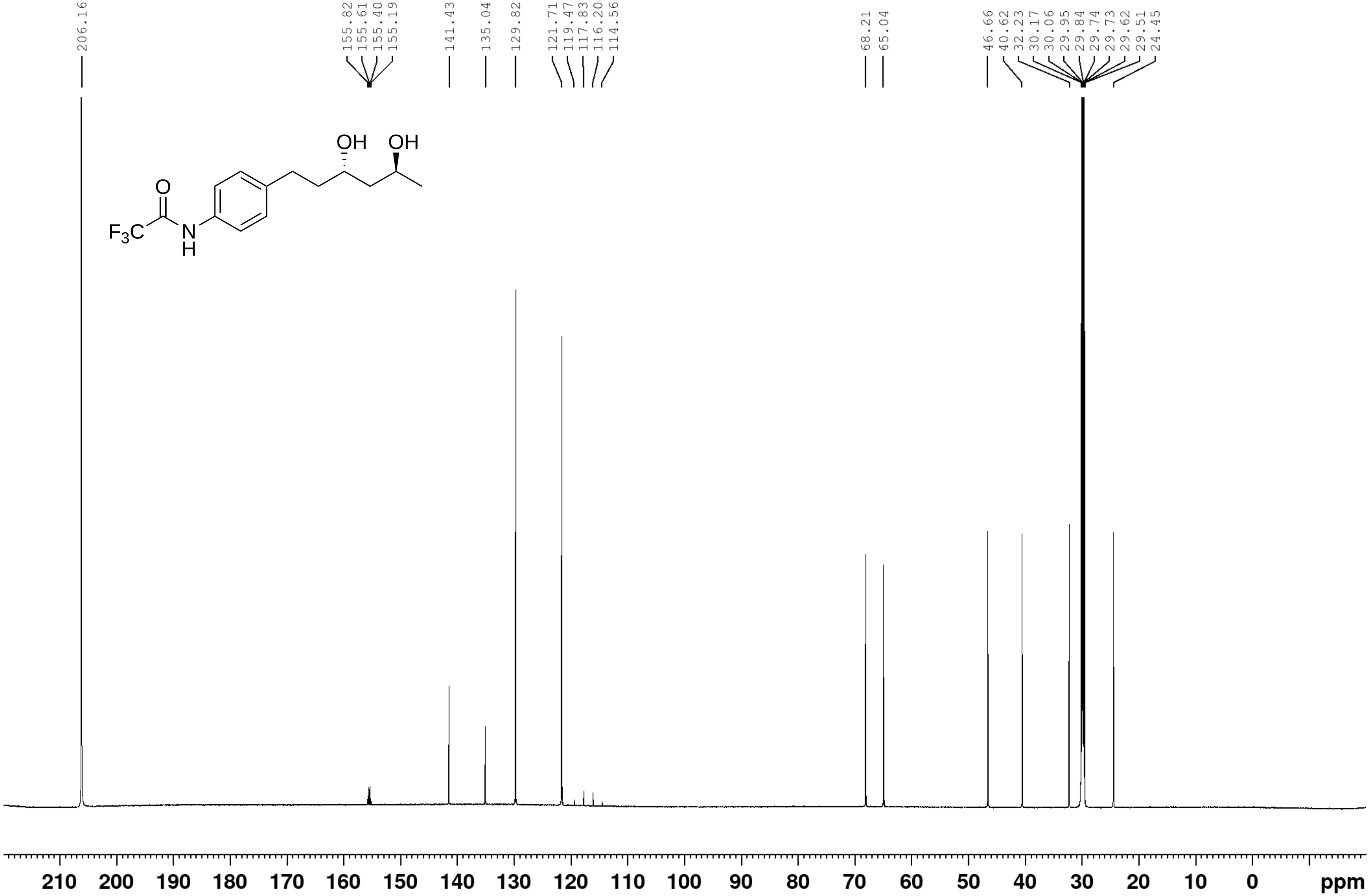

5-Hydroxy-2-methyl-7-phenylheptan-3-one (*int₁*-3i)

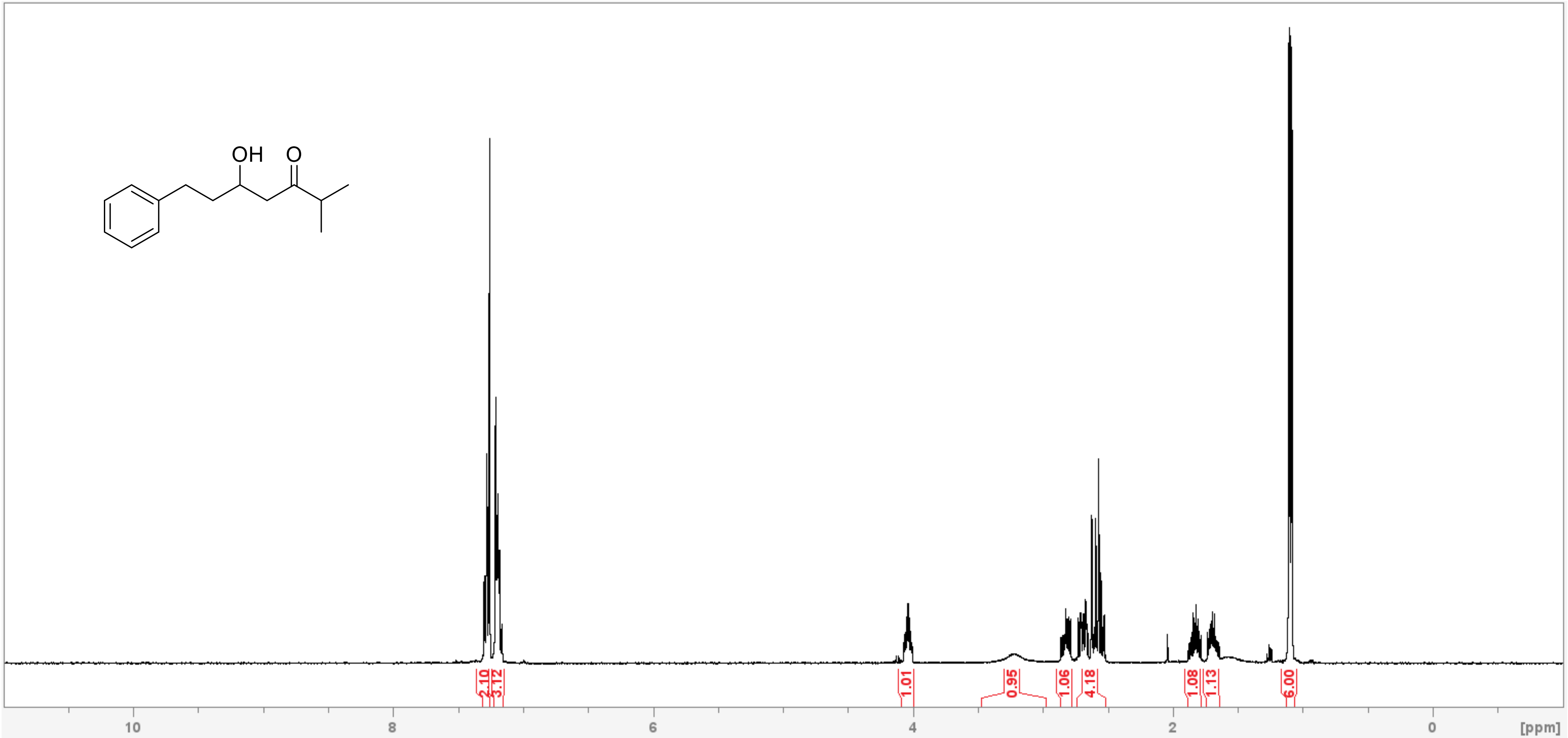

5-Hydroxy-2-methyl-7-phenylheptan-3-one (*int₁*-3i)

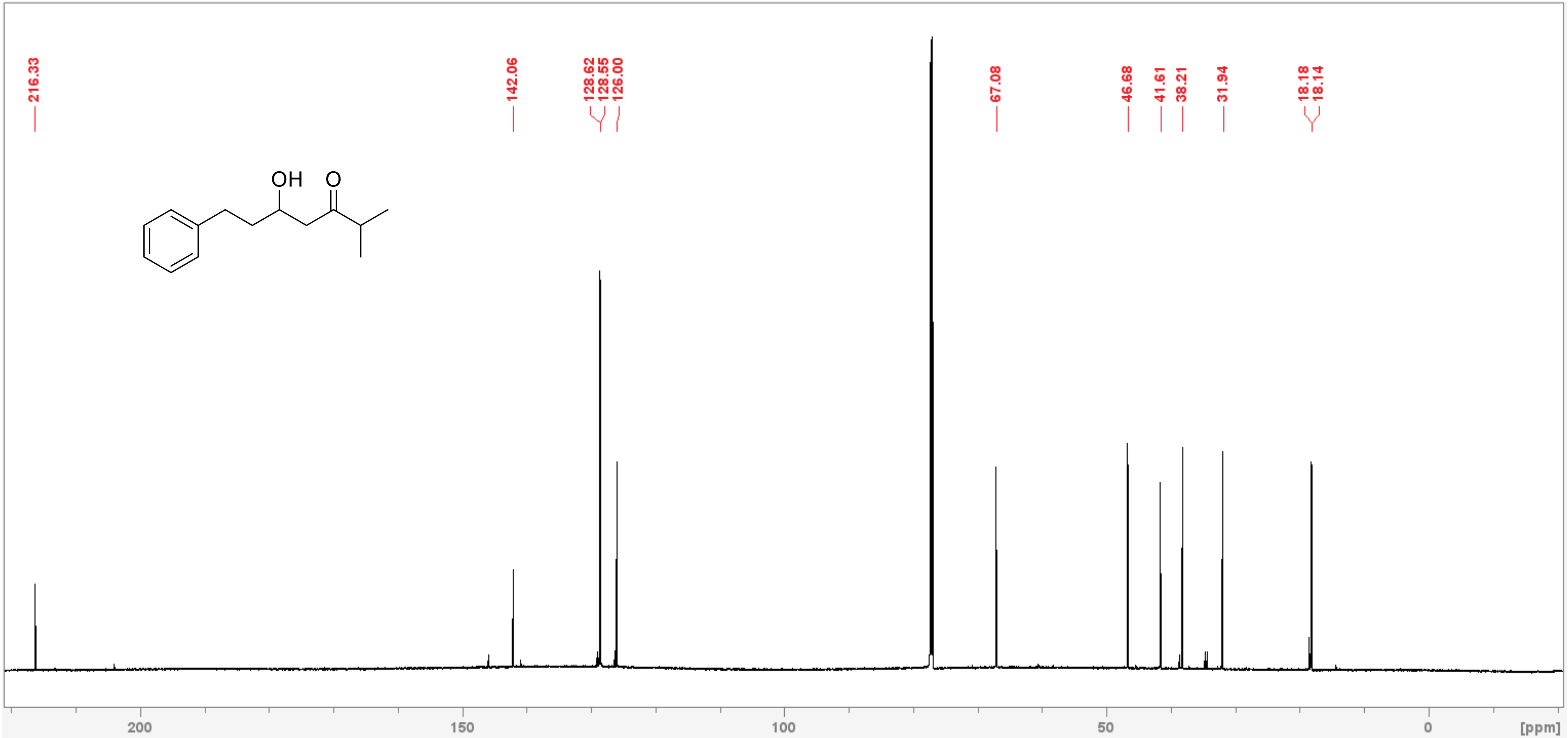

(*anti*)-6-Methyl-1-phenylheptane-3,5-diol (*rac*-*anti*-3i)

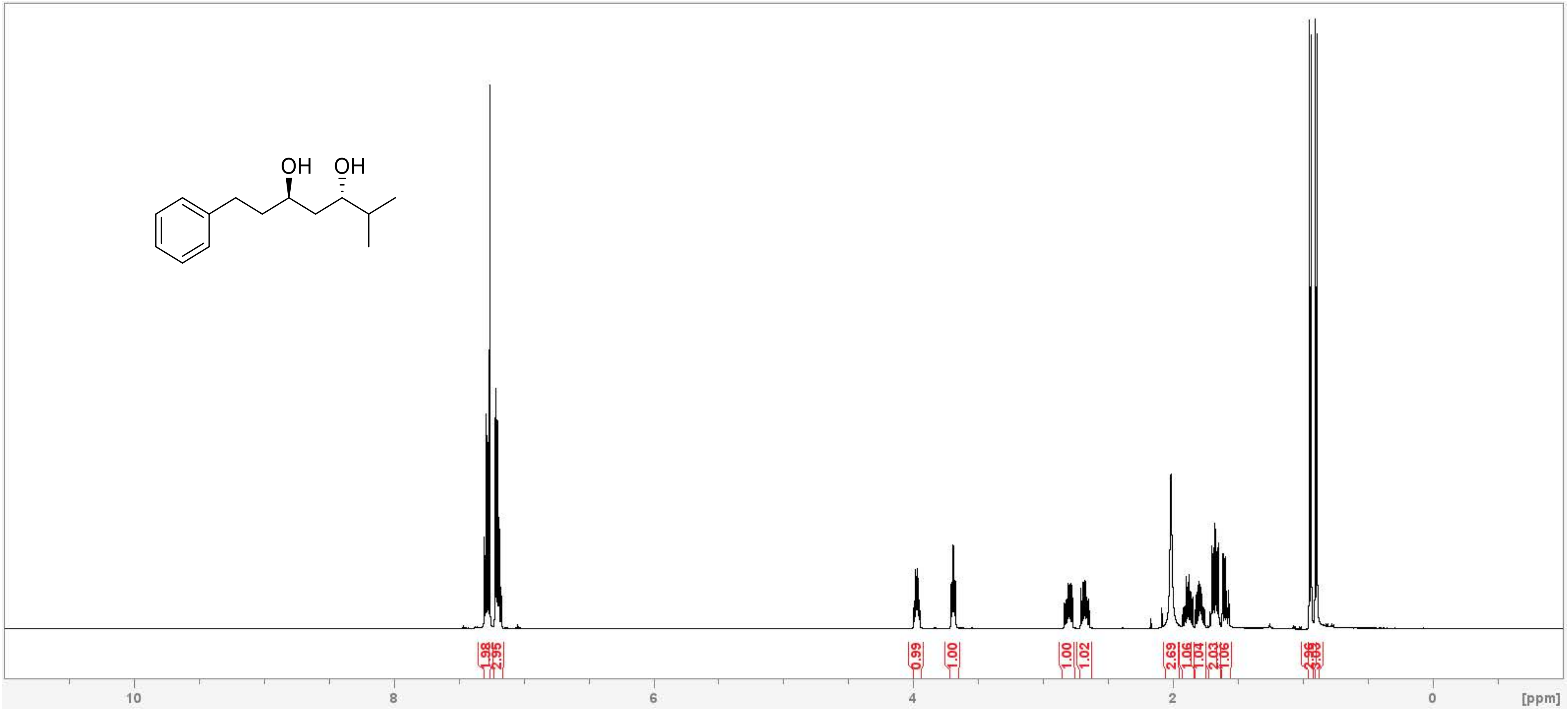

(*anti*)-6-Methyl-1-phenylheptane-3,5-diol (*rac*-*anti*-3i)

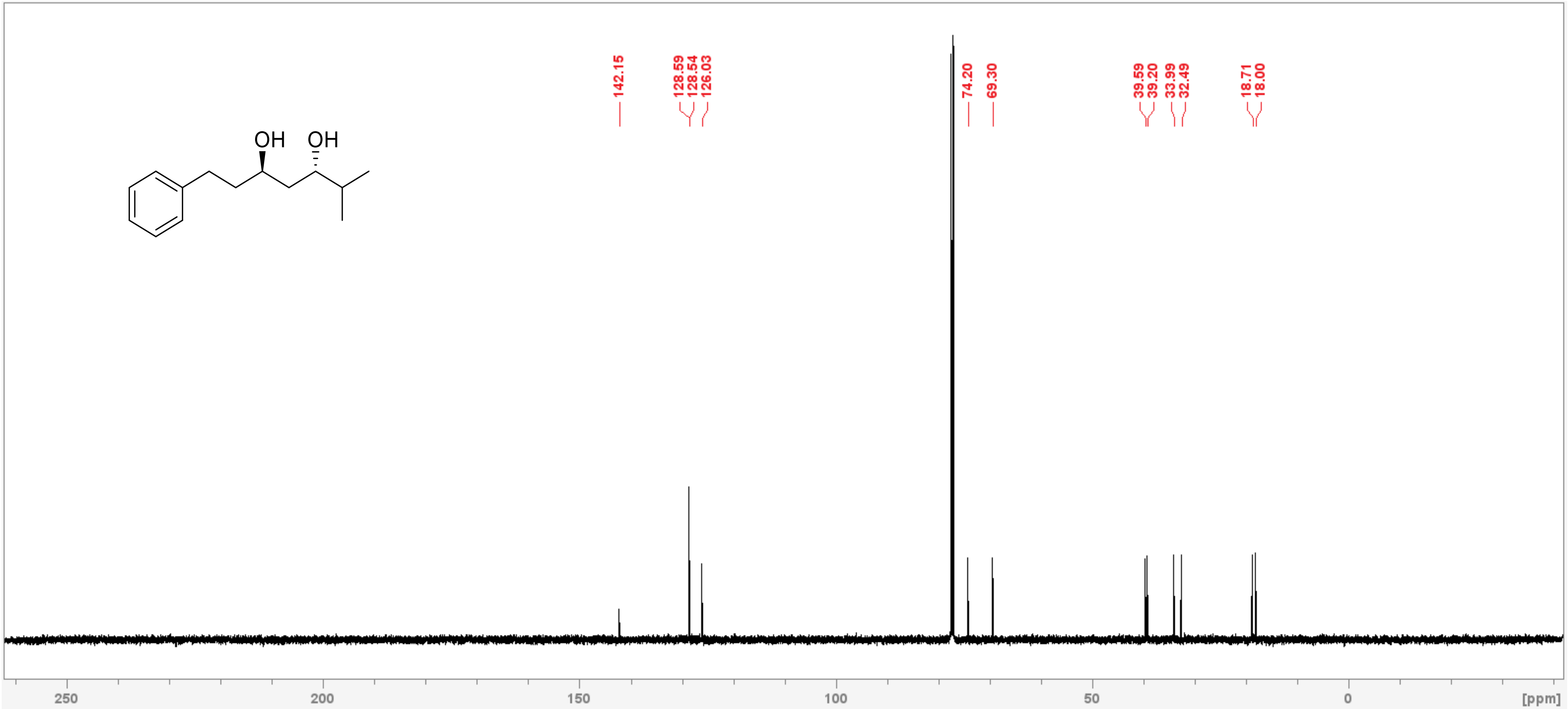

(*syn*)-6-Methyl-1-phenylheptane-3,5-diol (*rac*-*syn*-3i)

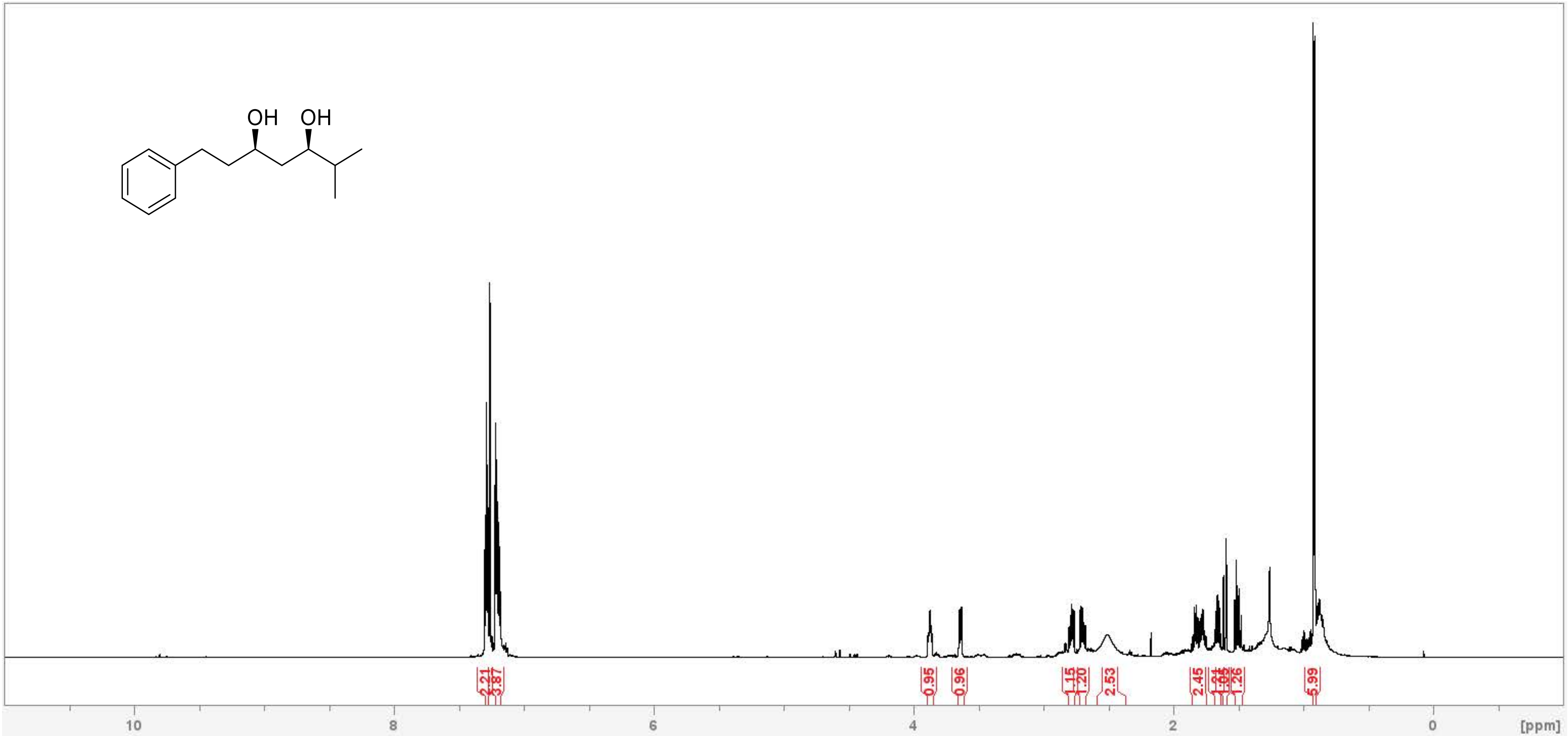

(*syn*)-6-Methyl-1-phenylheptane-3,5-diol (*rac-syn*-3i)

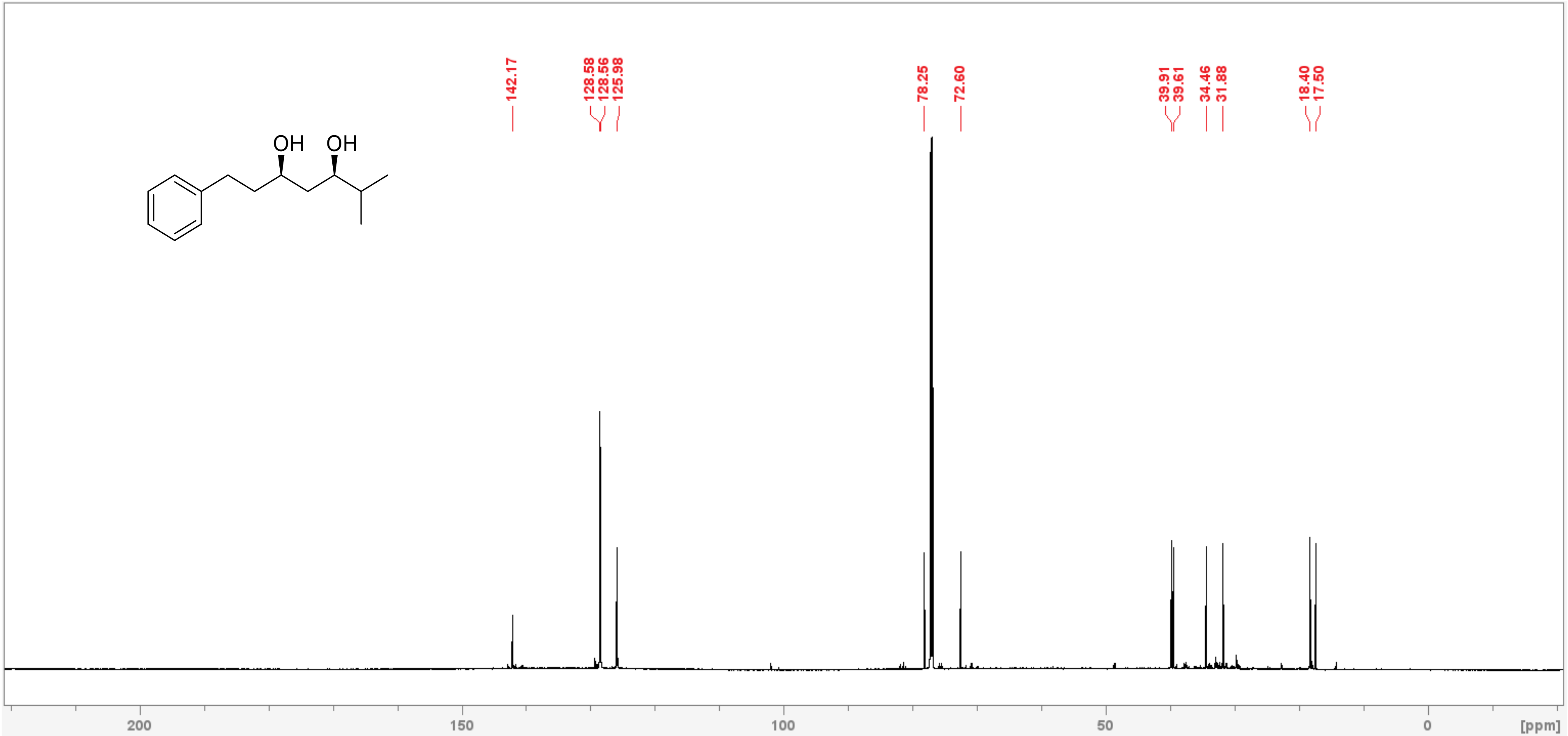

tert-butyl 4-(2-hydroxy-4-oxo-6-phenylhexyl)piperidine-1-carboxylate (_int_$_1$-3j)

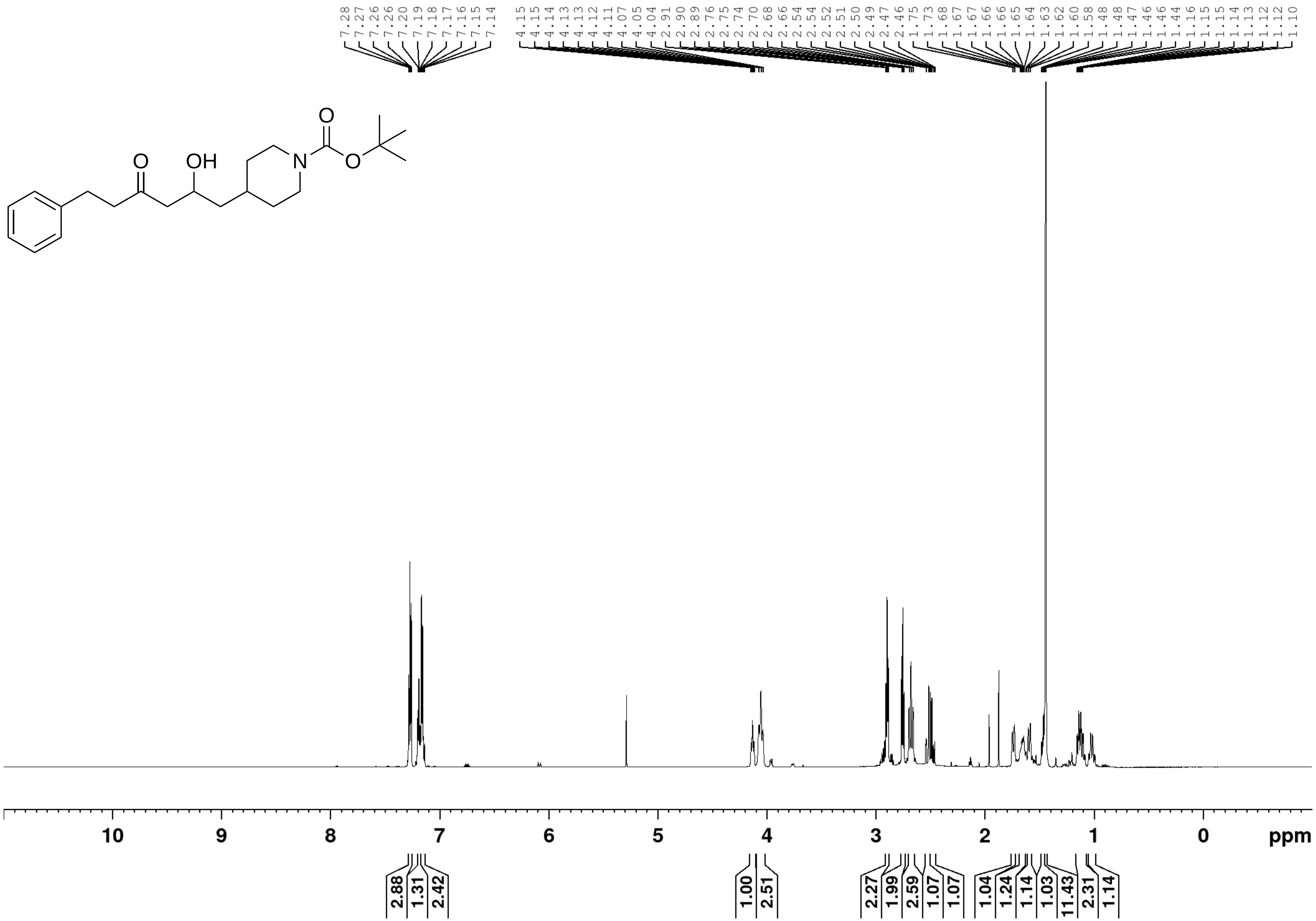

tert-butyl 4-(2-hydroxy-4-oxo-6-phenylhexyl)piperidine-1-carboxylate (_int₁_-3j)

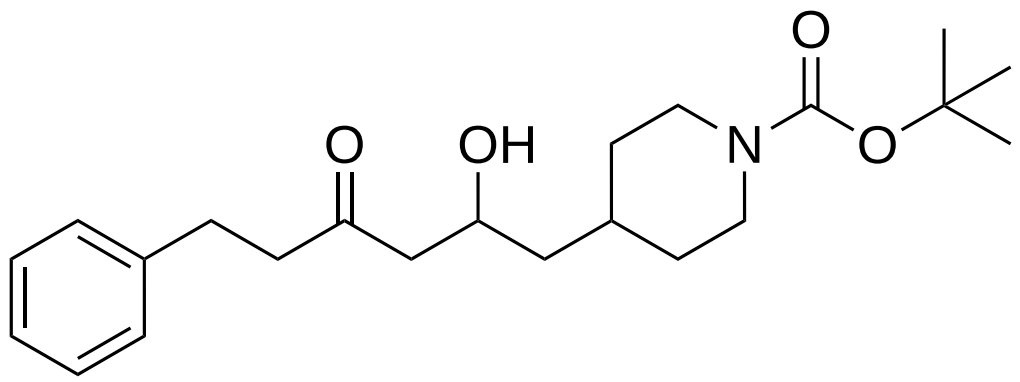

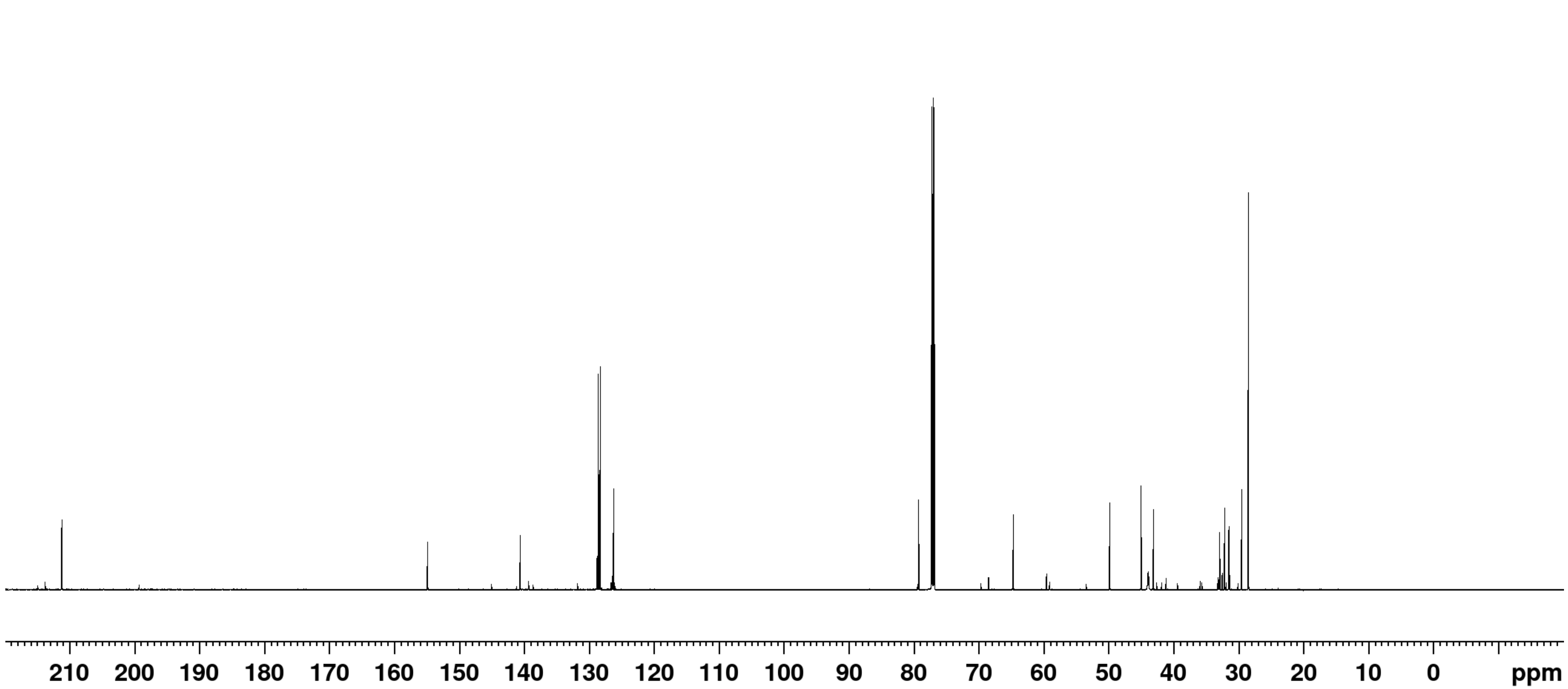

(*syn*)-*tert*-butyl 4-(2,4-dihydroxy-6-phenylhexyl)piperidine-1-carboxylate (*rac*-*syn*-3j)

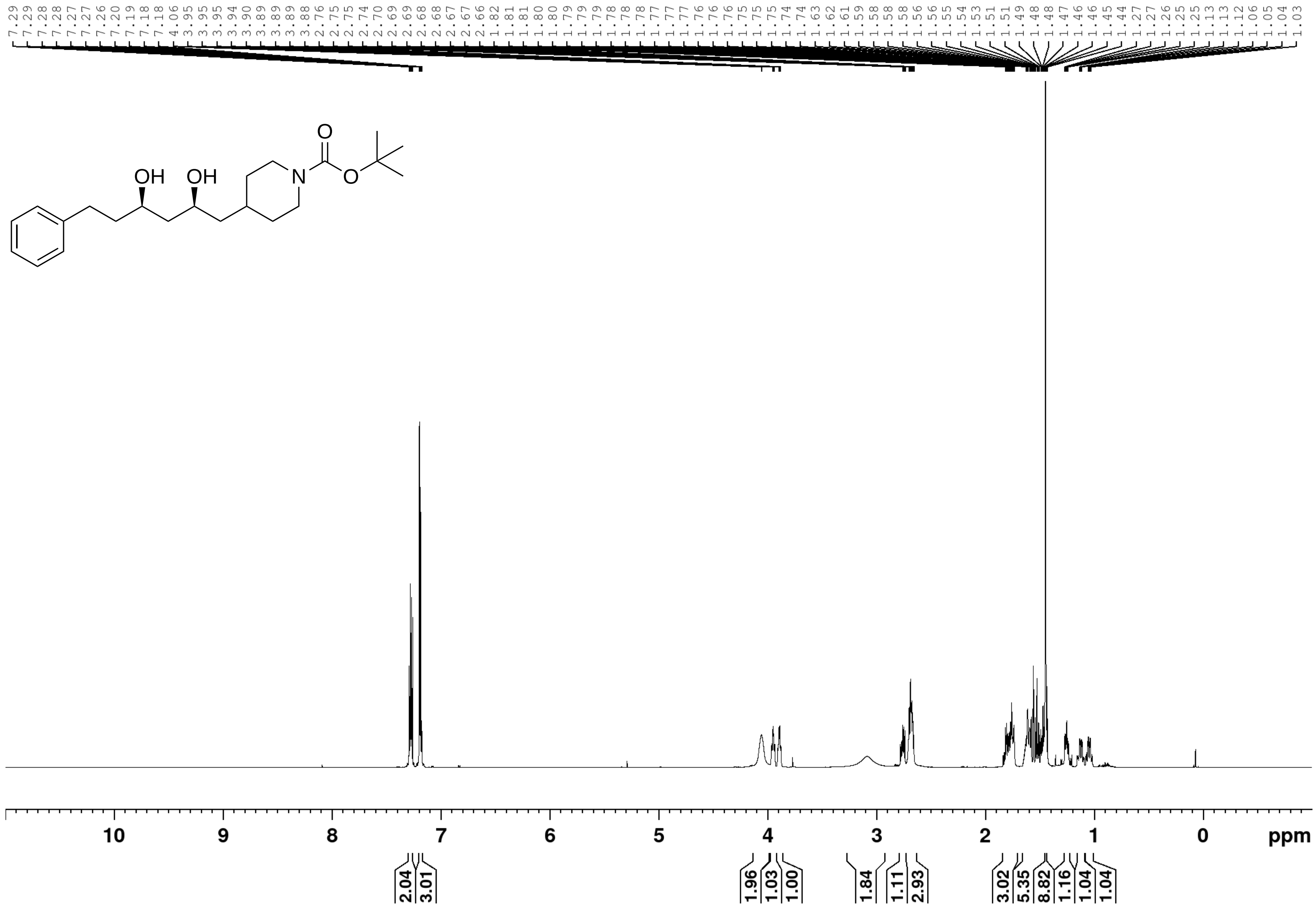

(*syn*)-*tert*-butyl 4-(2,4-dihydroxy-6-phenylhexyl)piperidine-1-carboxylate (*rac*-*syn*-3j)

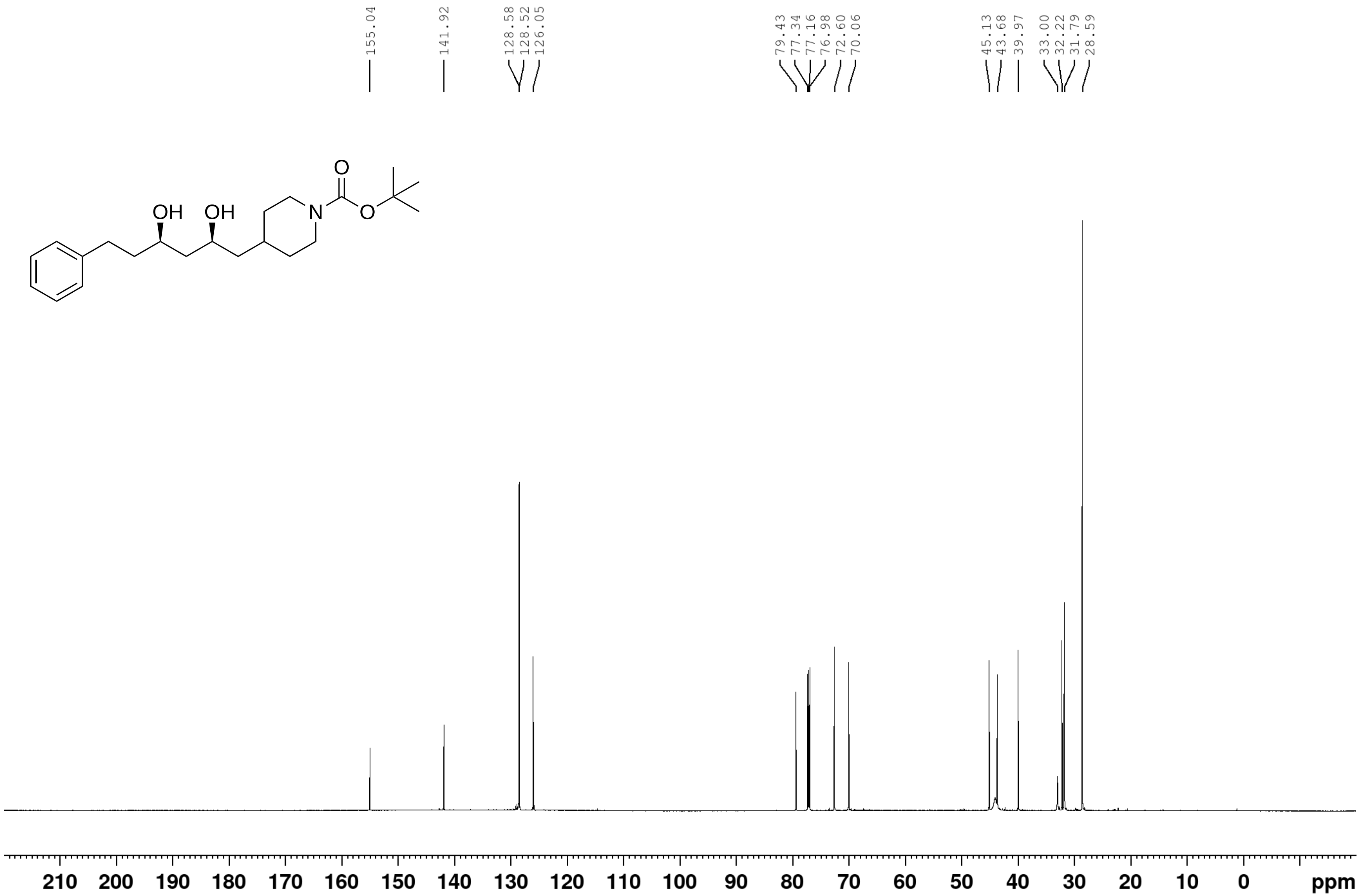

(*anti*)-*tert*-butyl 4-(2,4-dihydroxy-6-phenylhexyl)piperidine-1-carboxylate (*rac-anti*-3j)

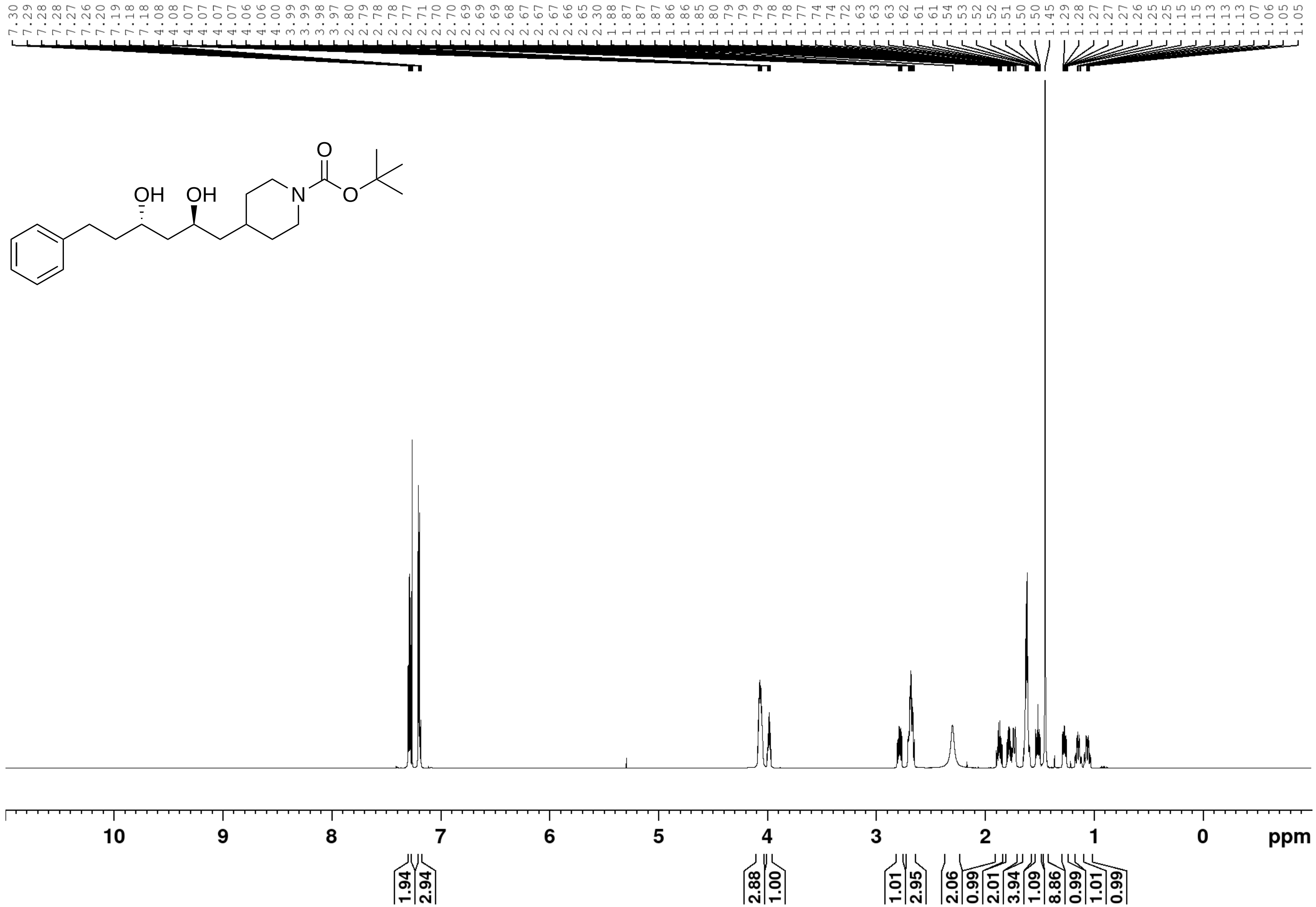

(*anti*)-*tert*-butyl 4-(2,4-dihydroxy-6-phenylhexyl)piperidine-1-carboxylate (*rac*-*anti*-3j)

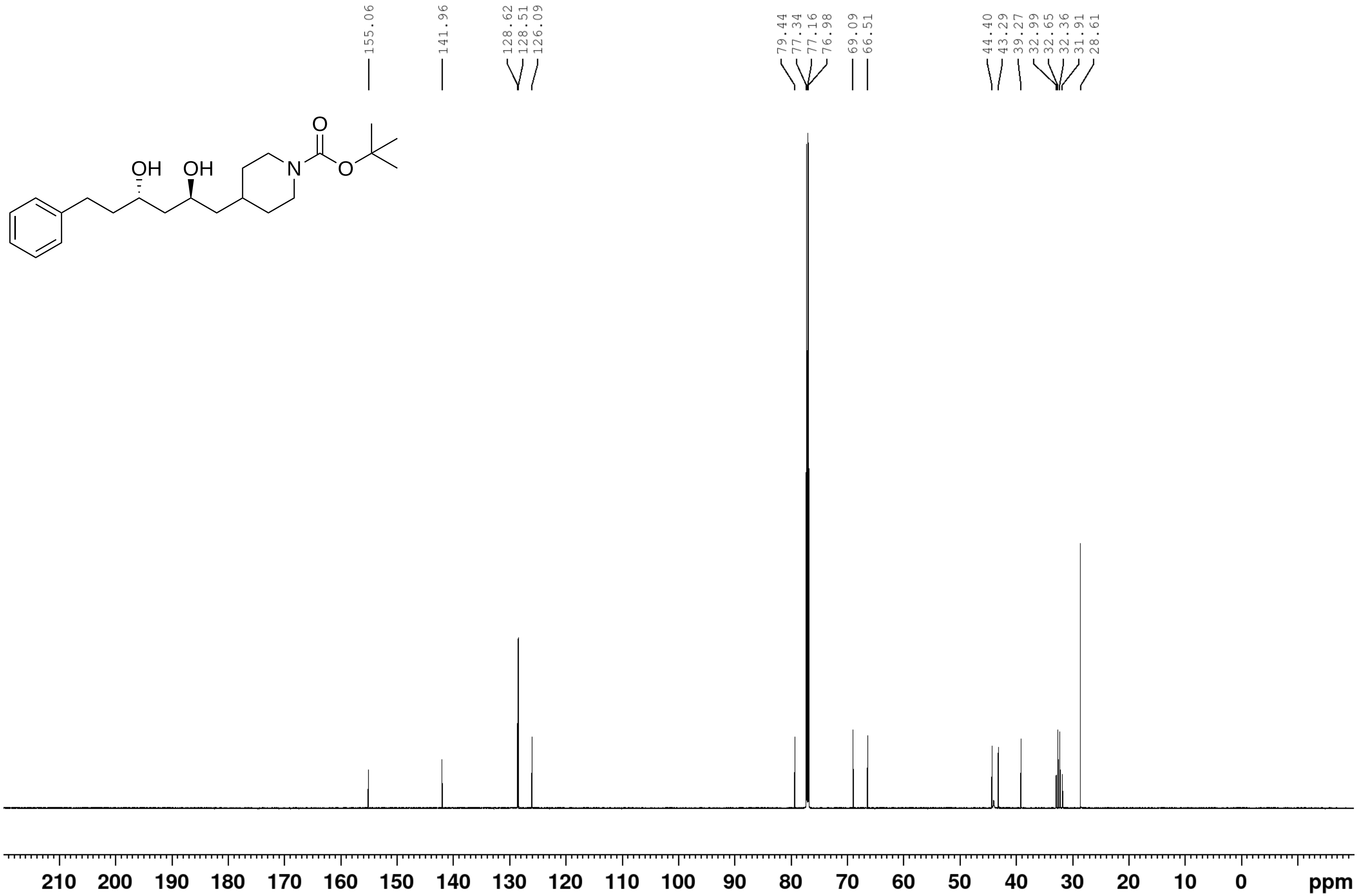

3-Hydroxy-1-phenylnonan-5-one (*int*$_1$-3k)

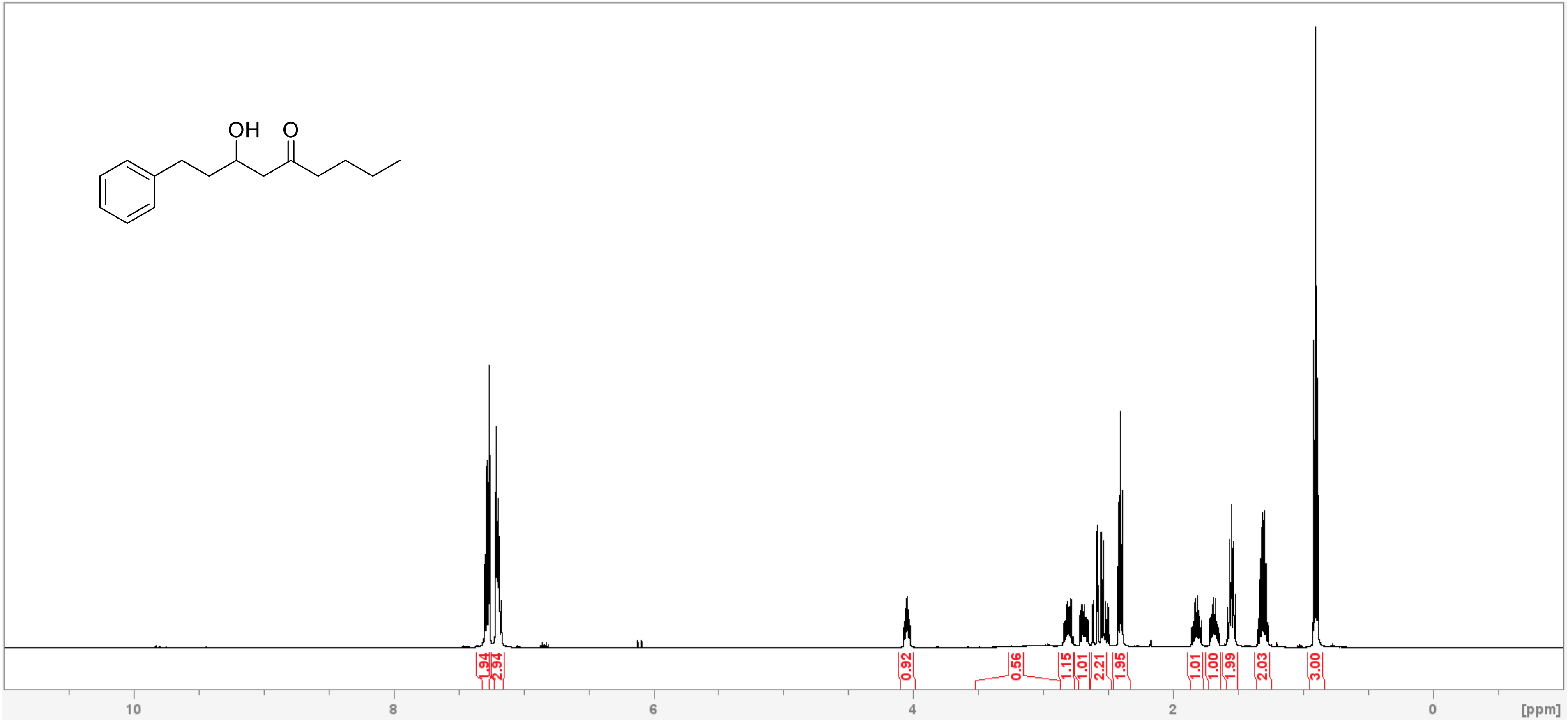

3-Hydroxy-1-phenylnonan-5-one (*int₁*-3k)

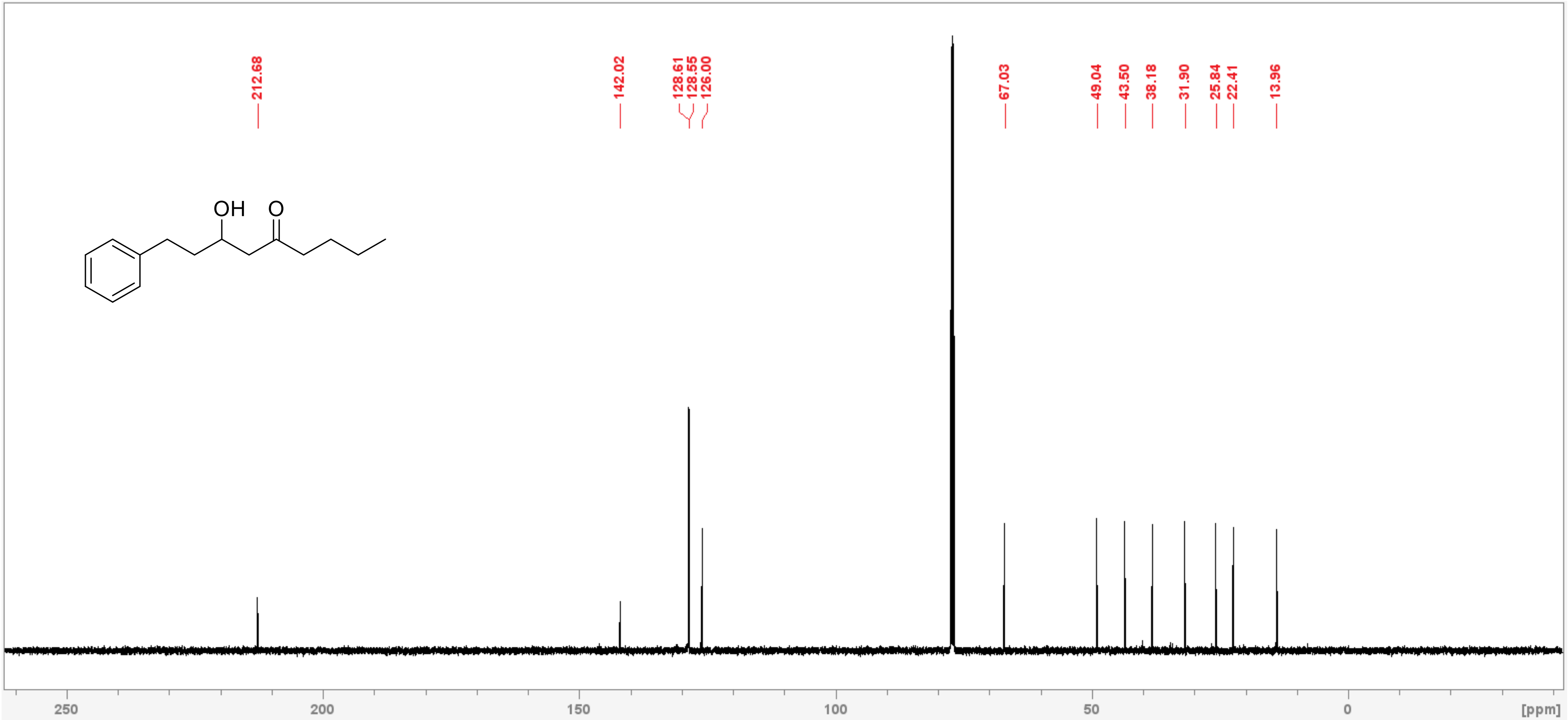

(*anti*)-1-Phenylnonane-3,5-diol (*rac*-*anti*-3k)

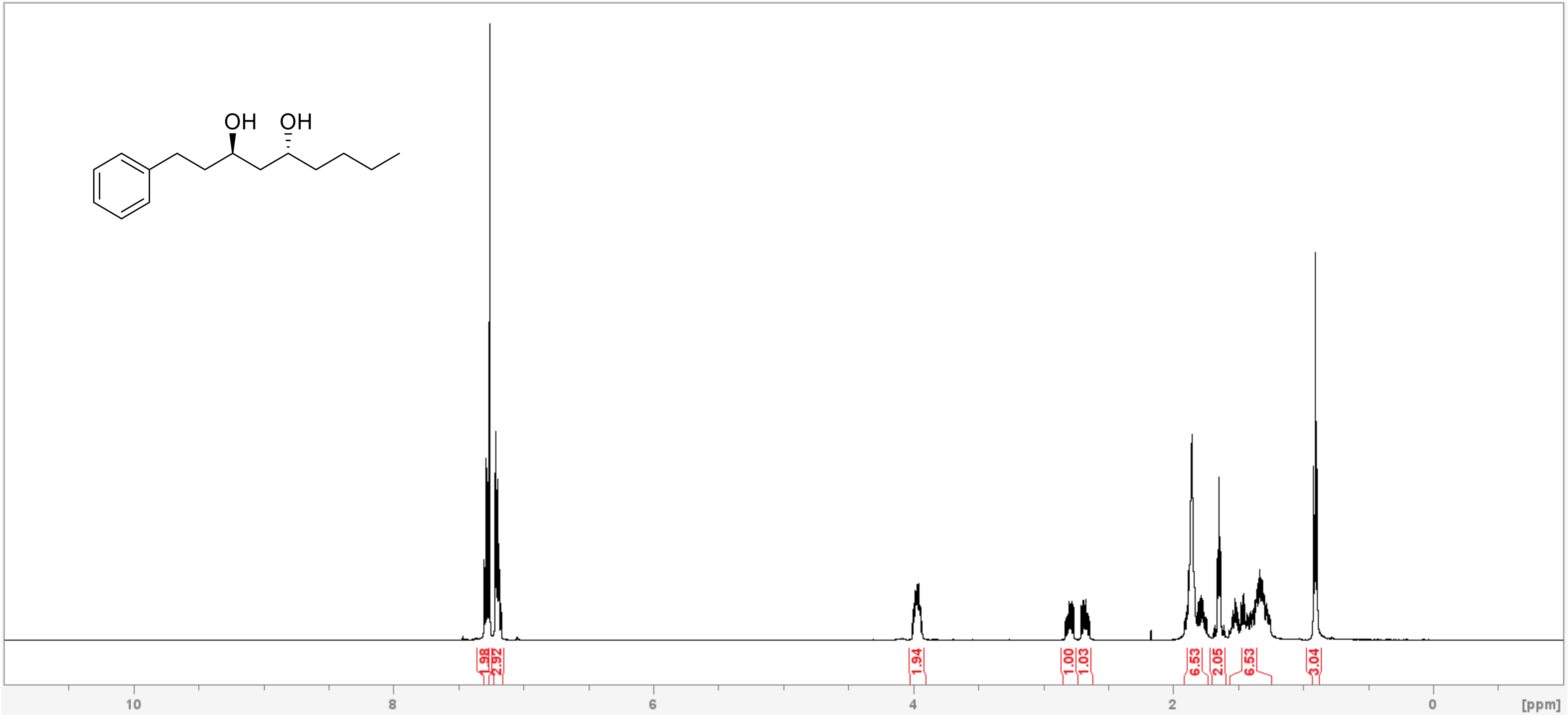

(*anti*)-1-Phenylnonane-3,5-diol (*rac*-*anti*-3k)

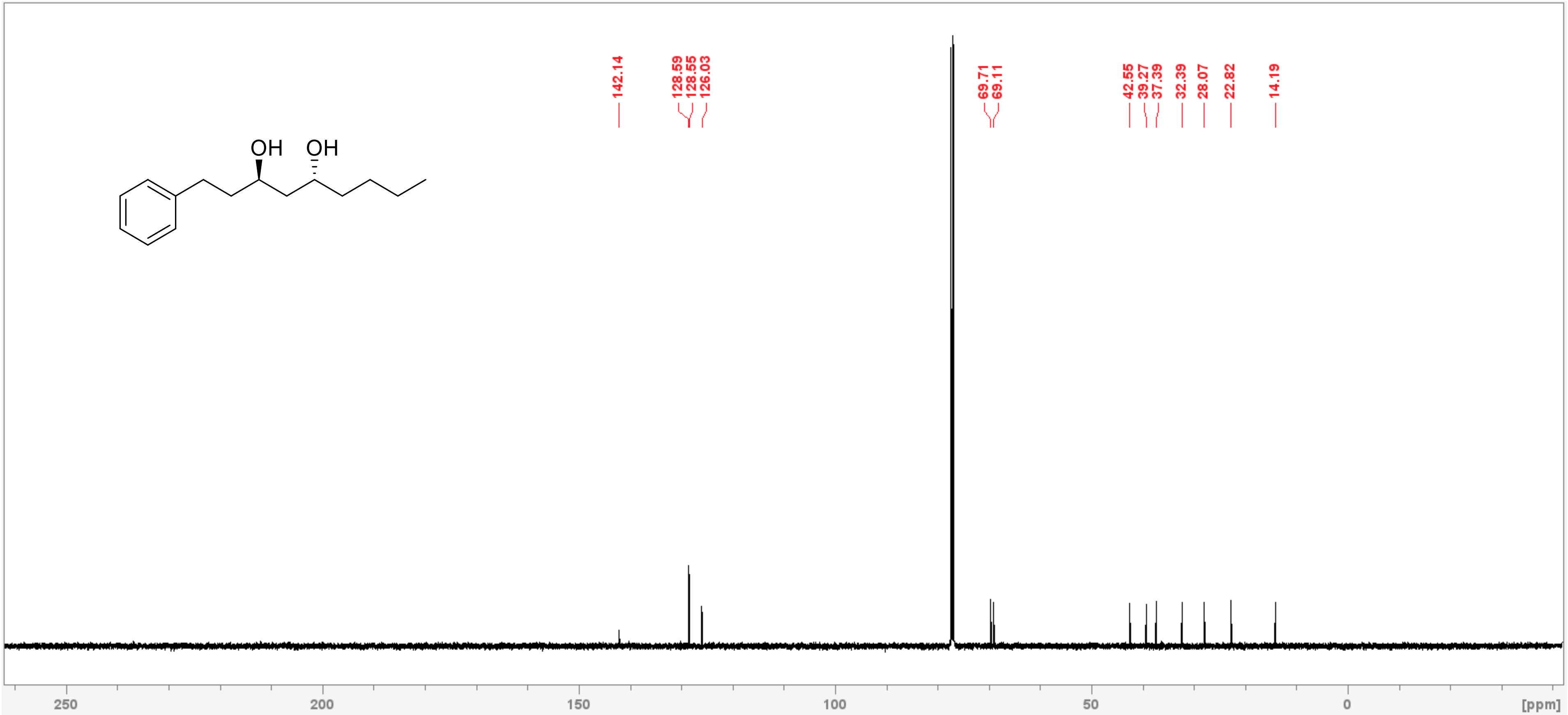

(*syn*)-1-Phenylnonane-3,5-diol (*rac*-*syn*-3k)

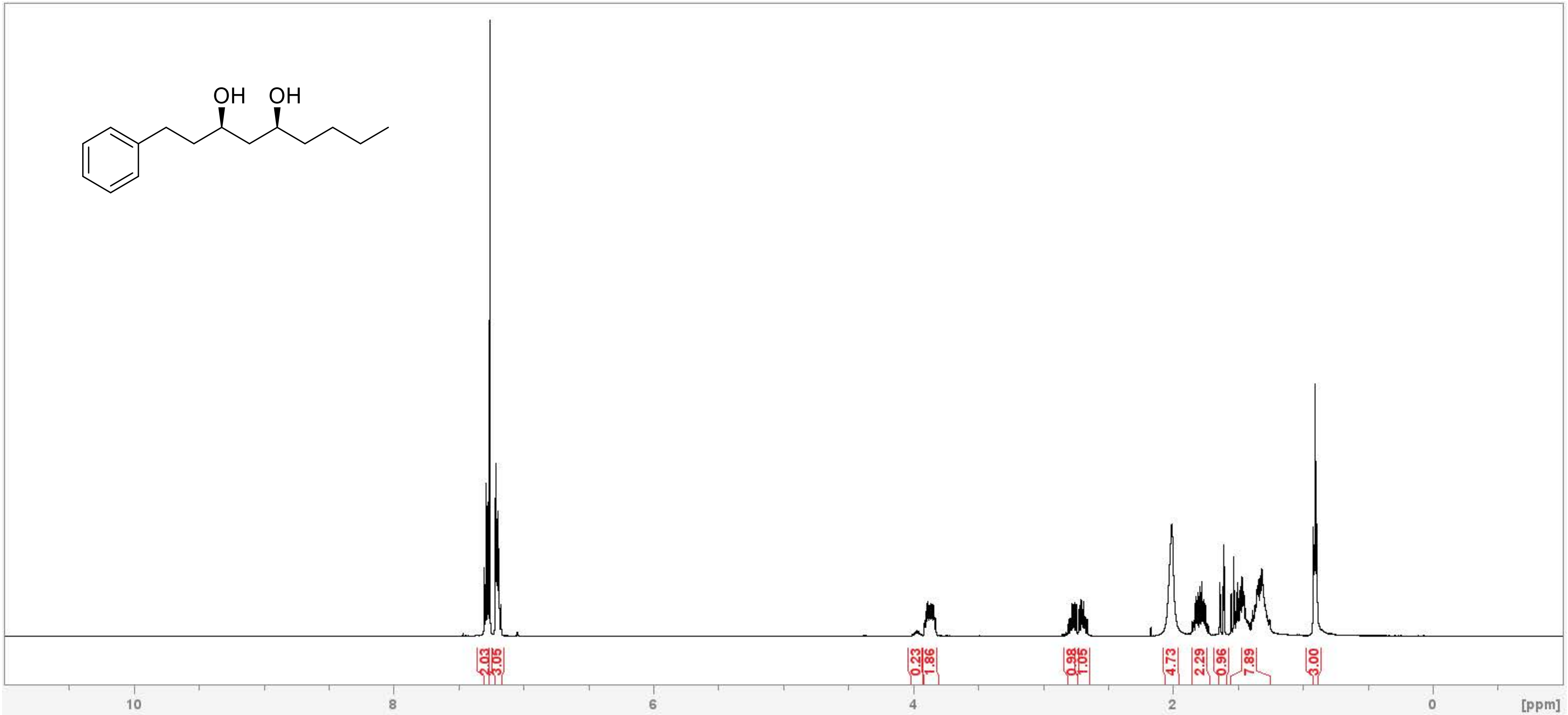

(*syn*)-1-Phenylnonane-3,5-diol (*rac*-*syn*-3k)

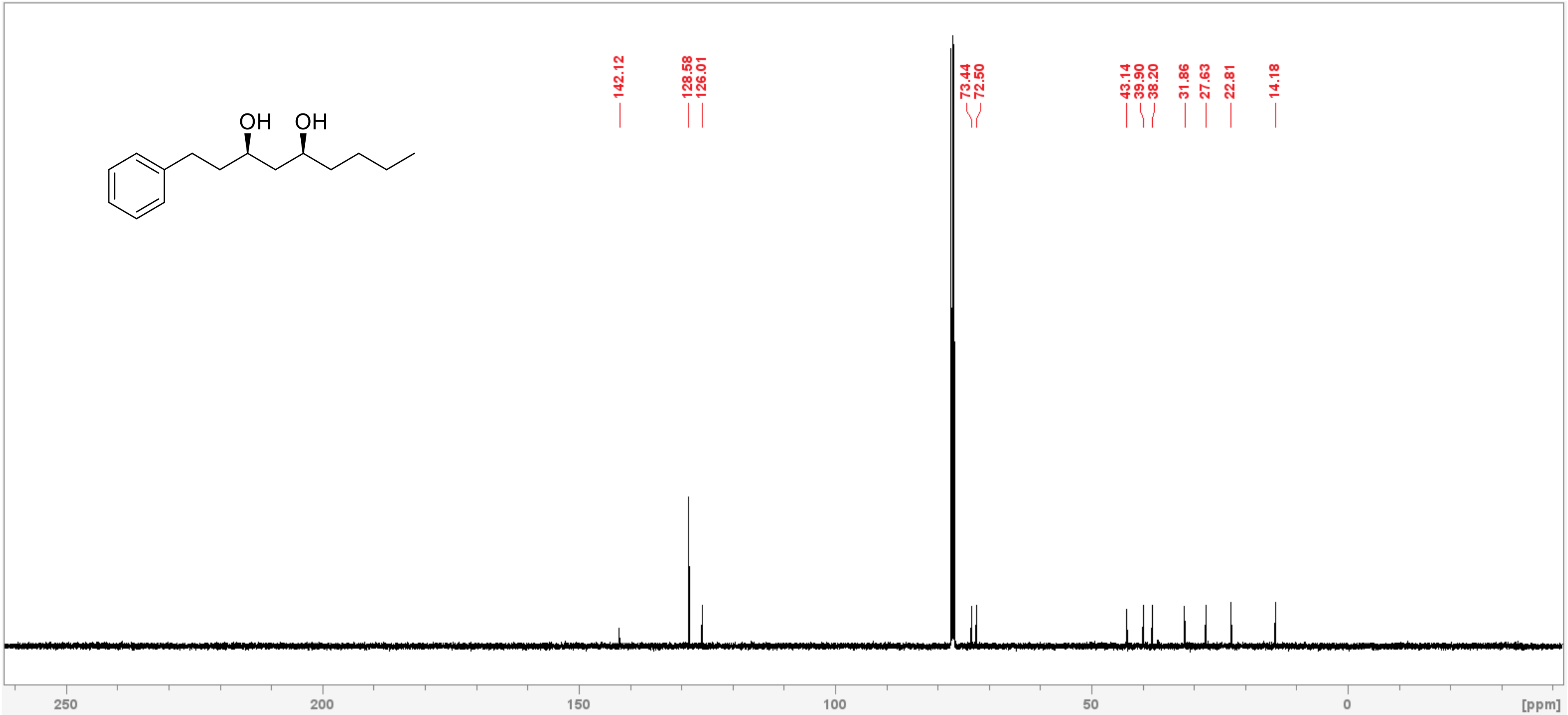

5-hydroxy-1-phenyl-7-((2,3,3-trimethylbutan-2-yl)oxy)heptan-3-one (*int₁*-3l)

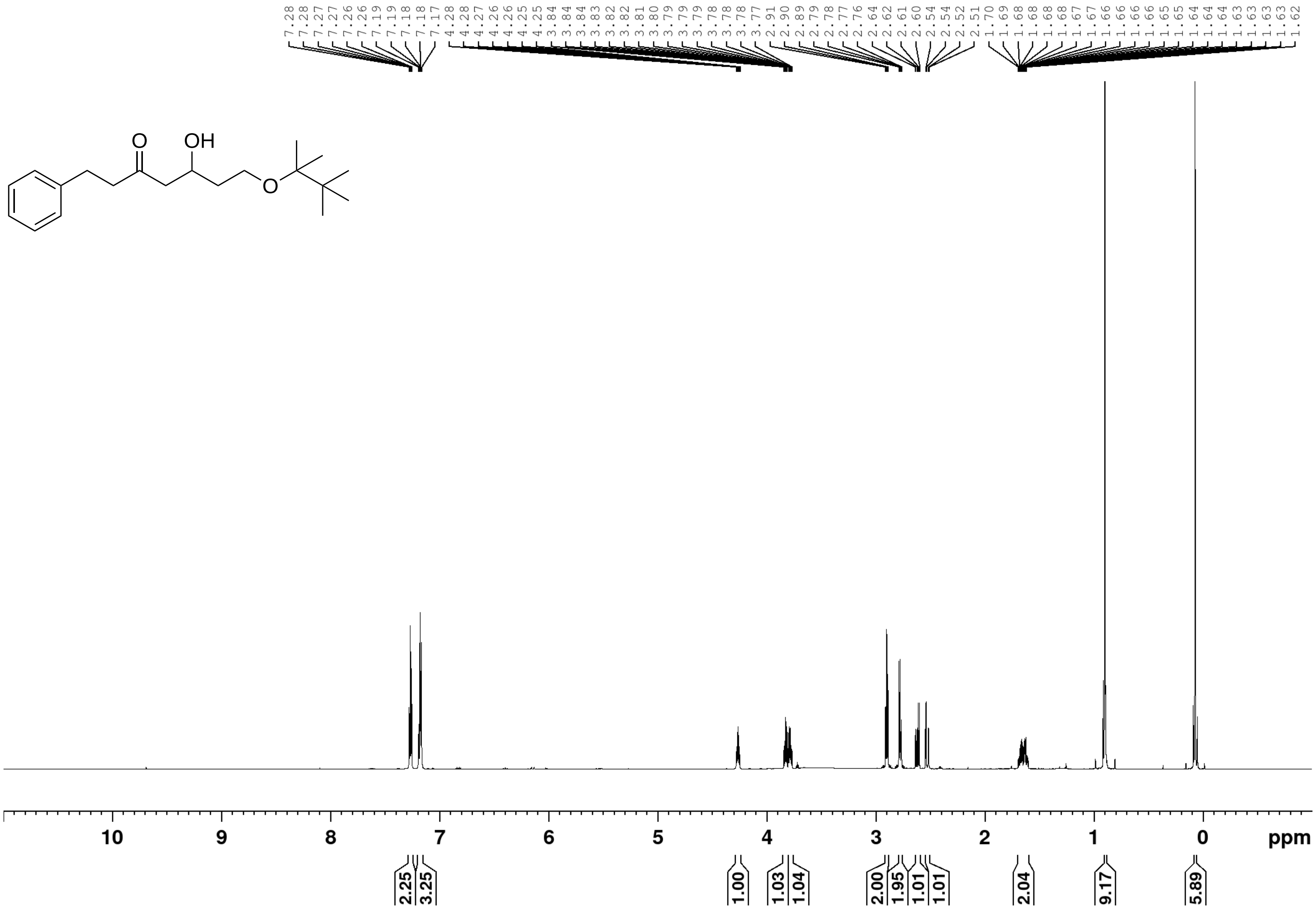

5-hydroxy-1-phenyl-7-((2,3,3-trimethylbutan-2-yl)oxy)heptan-3-one (*int₁*-3l)

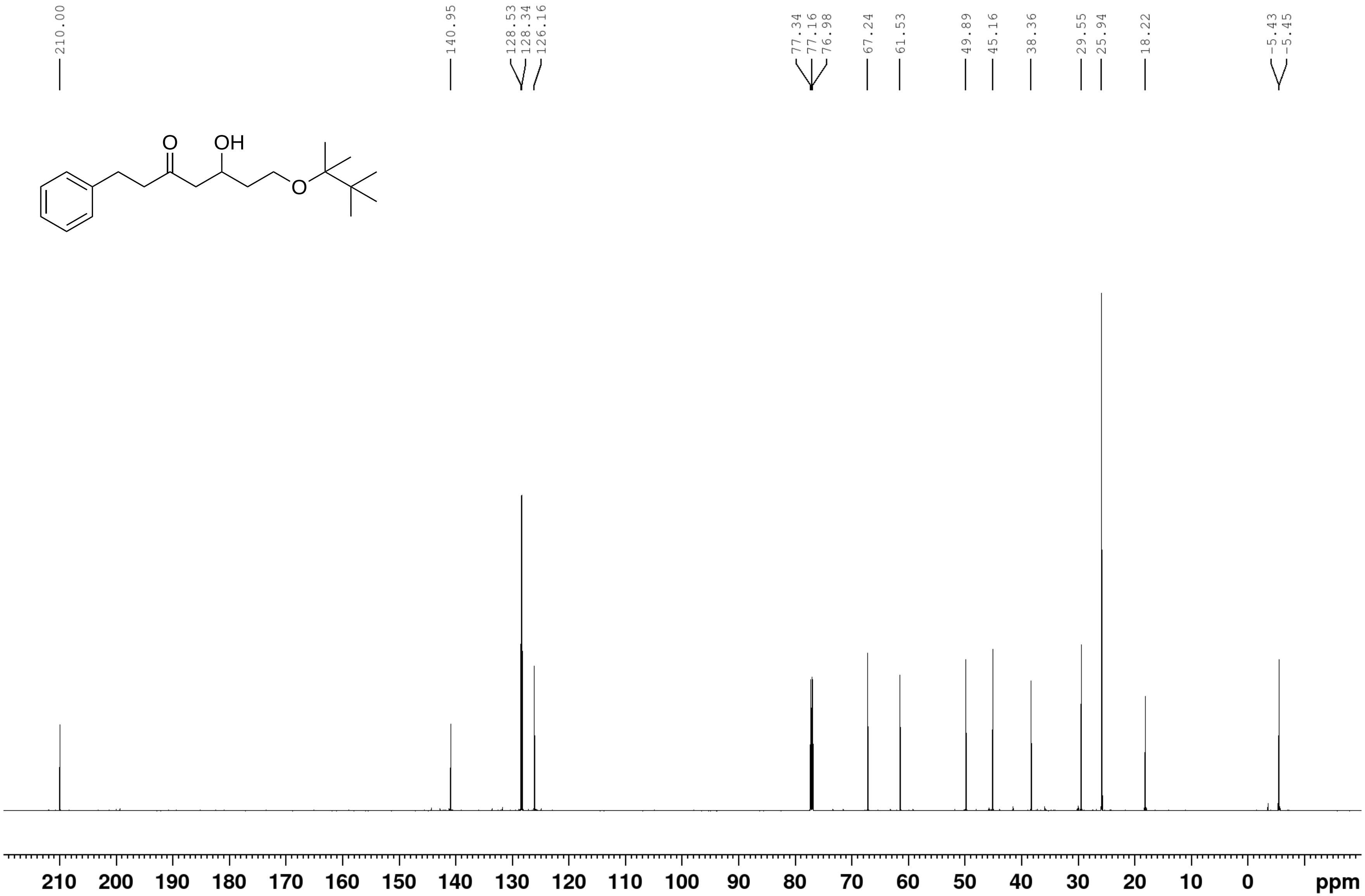

(*syn*)-1-phenyl-7-((2,3,3-trimethylbutan-2-yl)oxy)heptane-3,5-diol (*rac-syn*-3l)

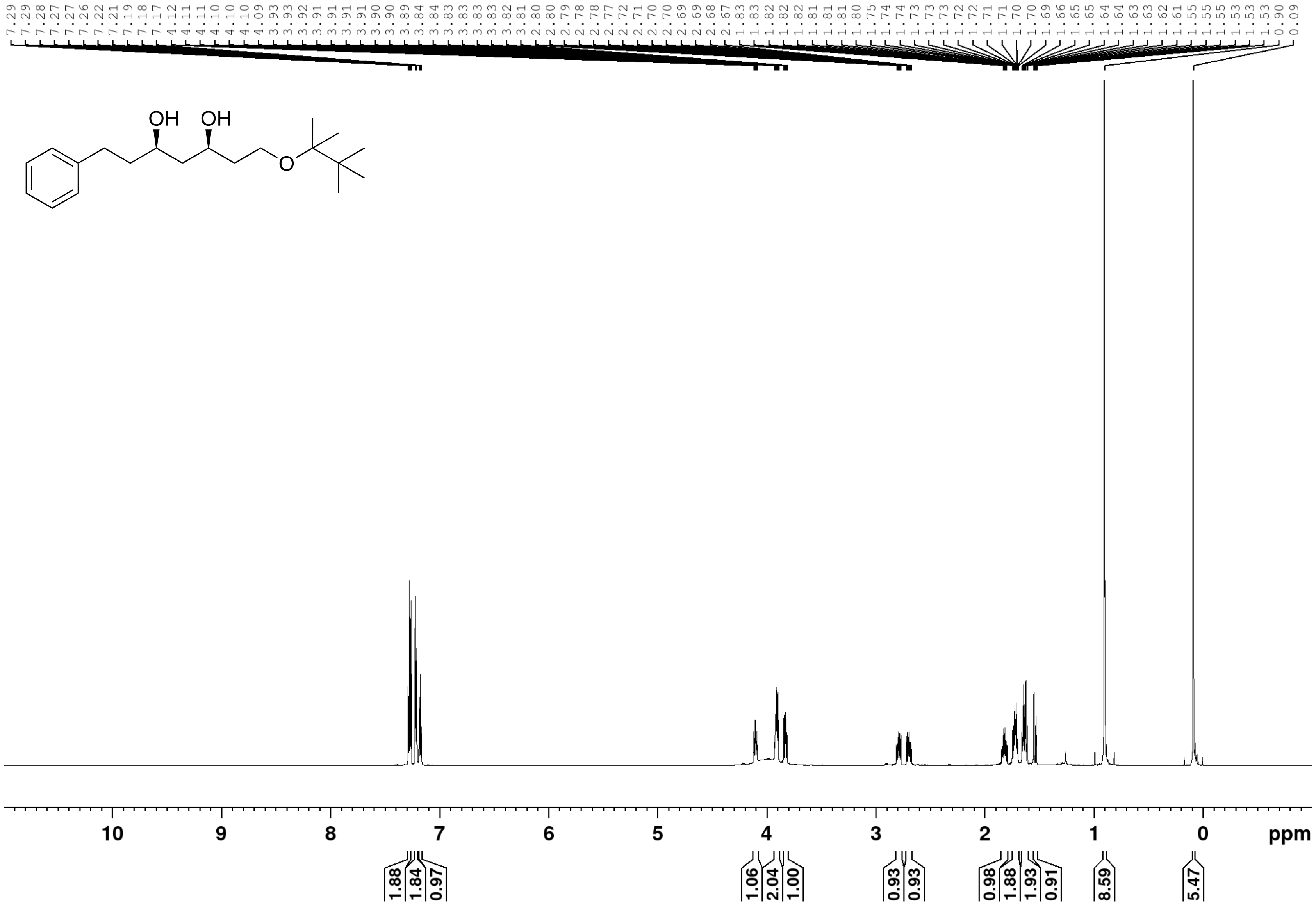

(*syn*)-1-phenyl-7-((2,3,3-trimethylbutan-2-yl)oxy)heptane-3,5-diol (*rac*-*syn*-3l)

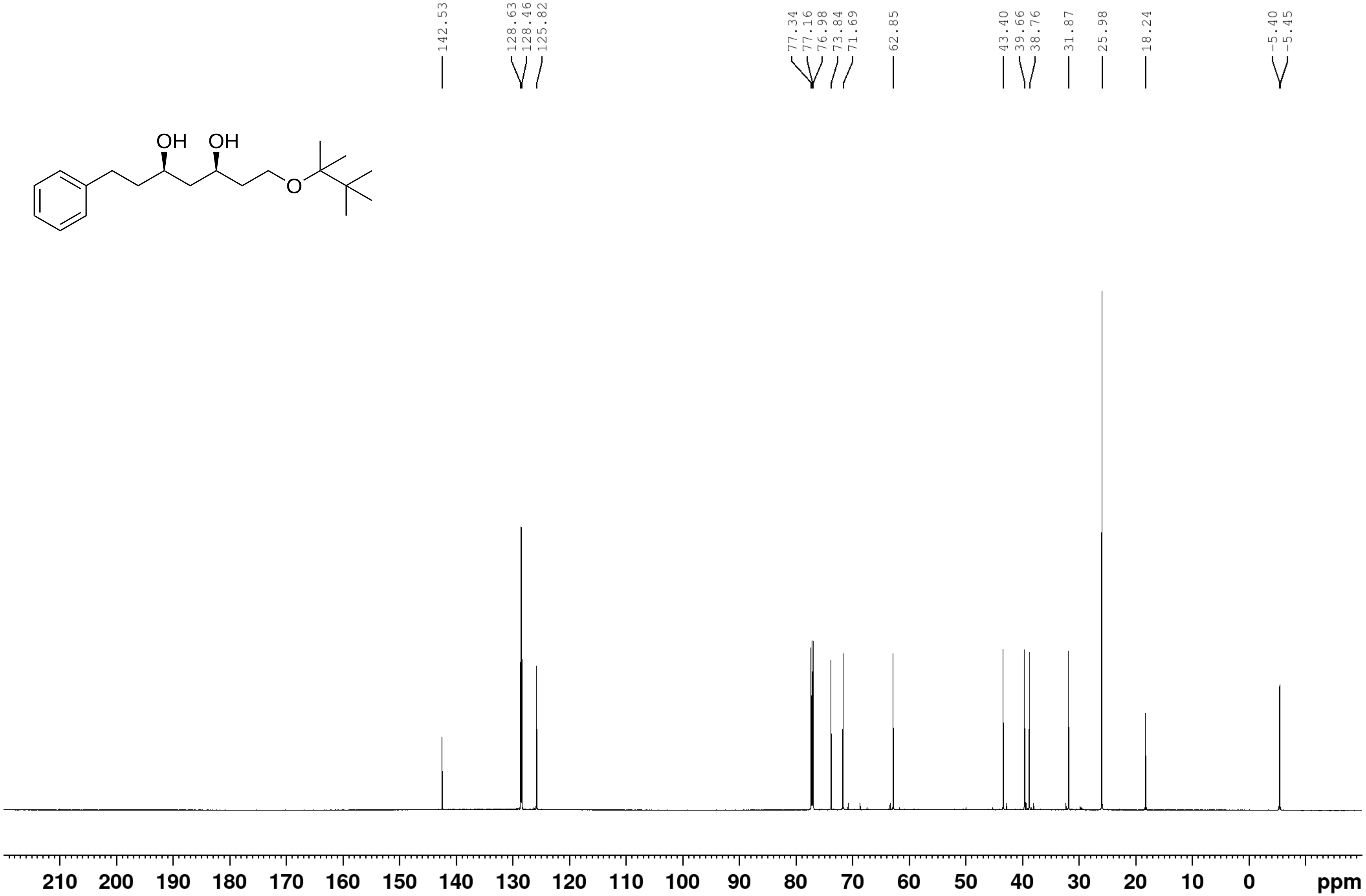

(*anti*)-1-phenyl-7-((2,3,3-trimethylbutan-2-yl)oxy)heptane-3,5-diol (*rac-anti*-3l)

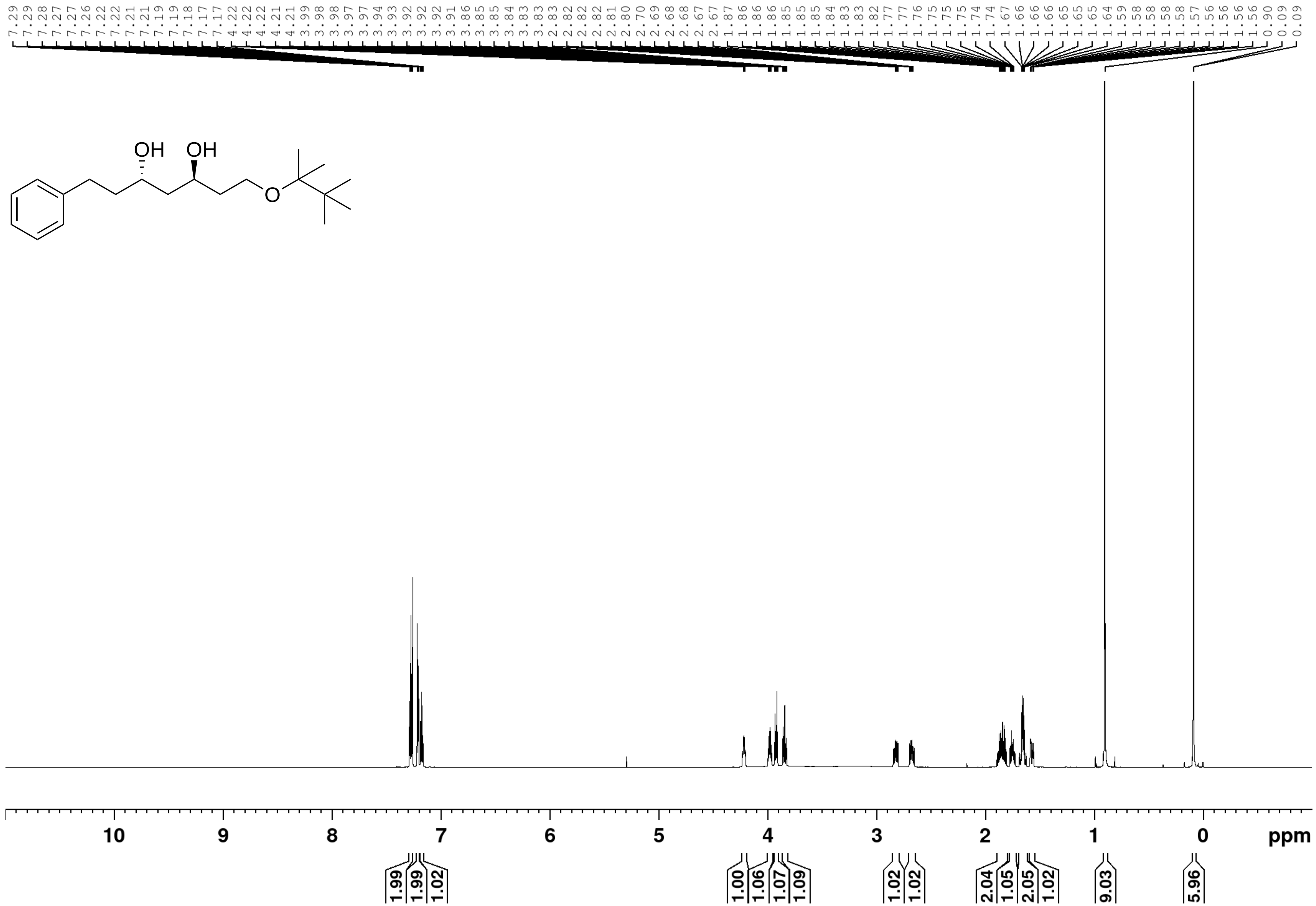

(*anti*)-1-phenyl-7-((2,3,3-trimethylbutan-2-yl)oxy)heptane-3,5-diol (*rac-anti*-3l)

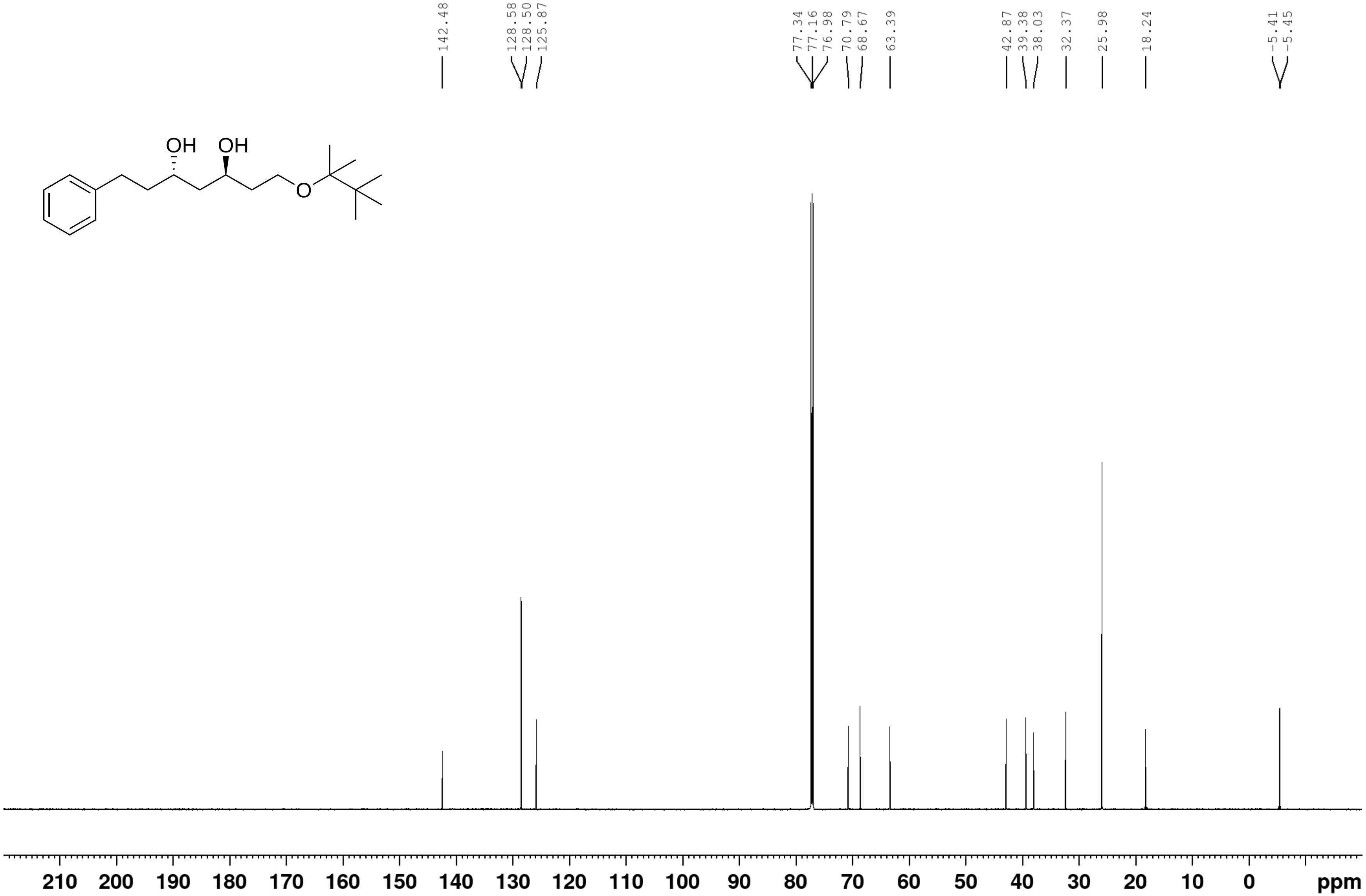

4-hydroxy-1-tosylpentan-2-one (*int₁*-3m)

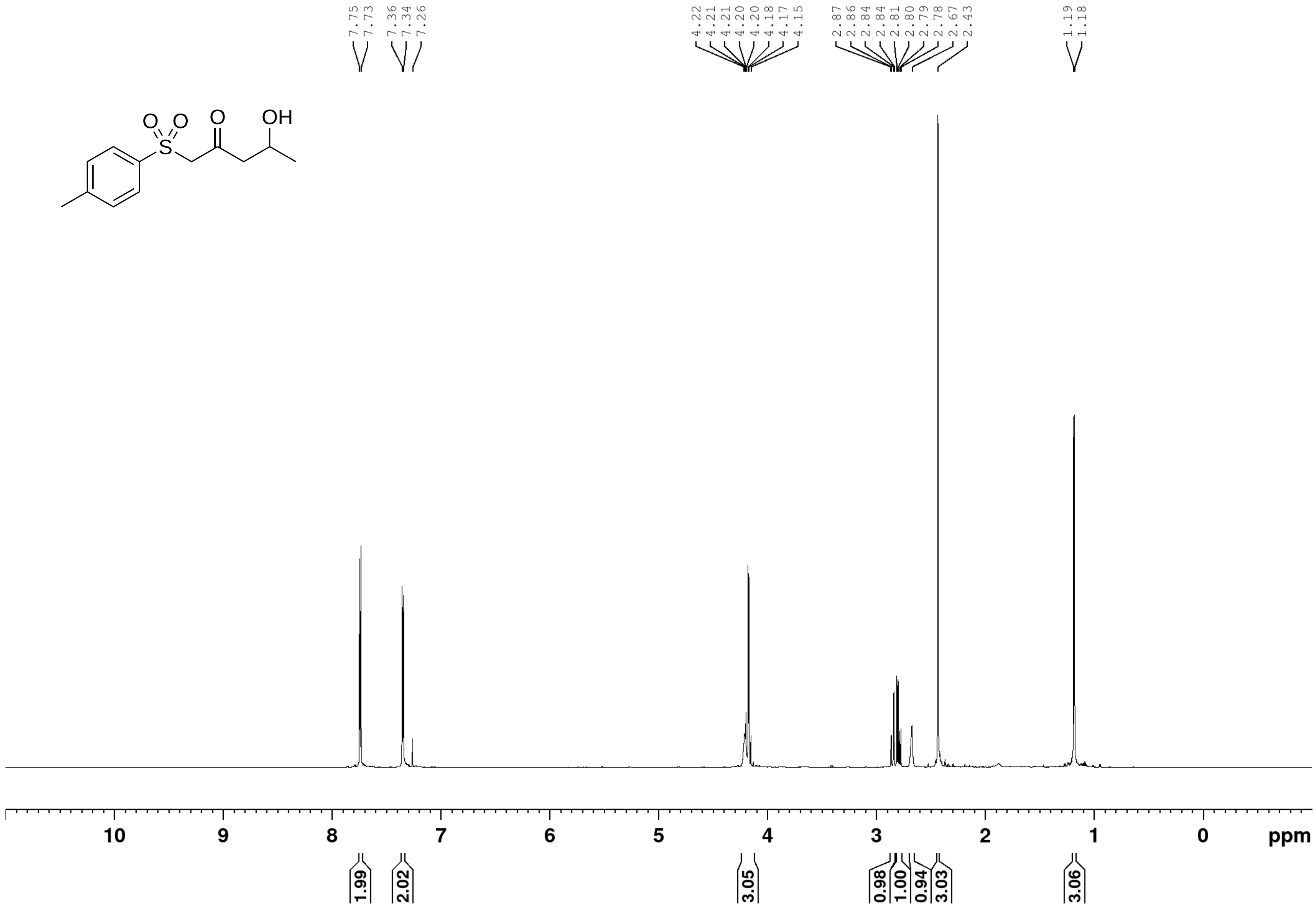

4-hydroxy-1-tosylpentan-2-one (*int₁*-3m)

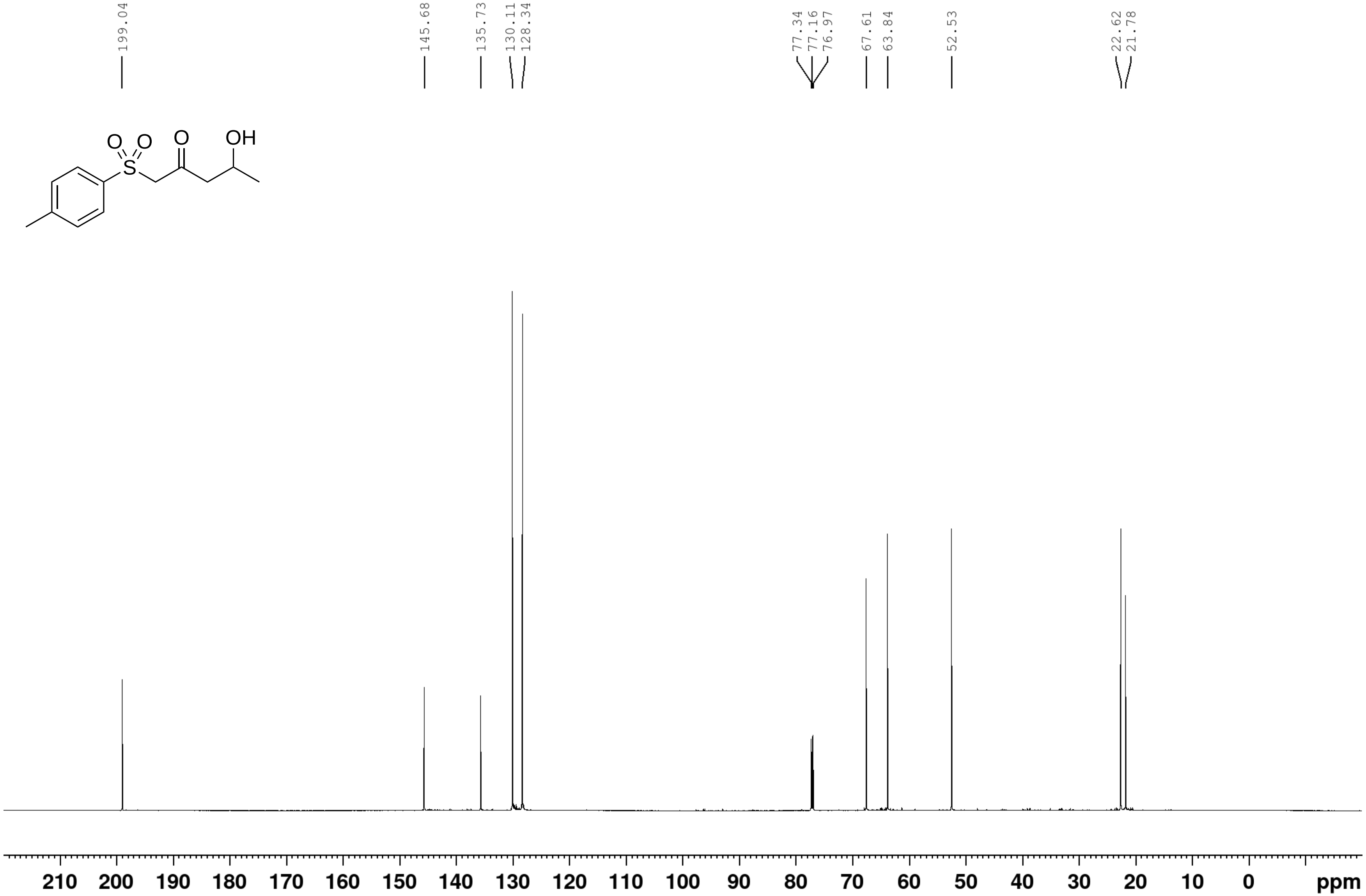

(*anti*)-(2*R*,4*S*)-1-tosylpentane-2,4-diol (*rac-anti*-3m)

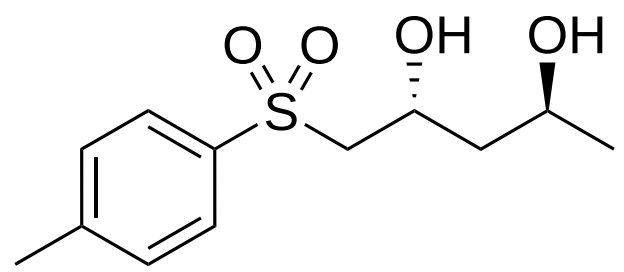

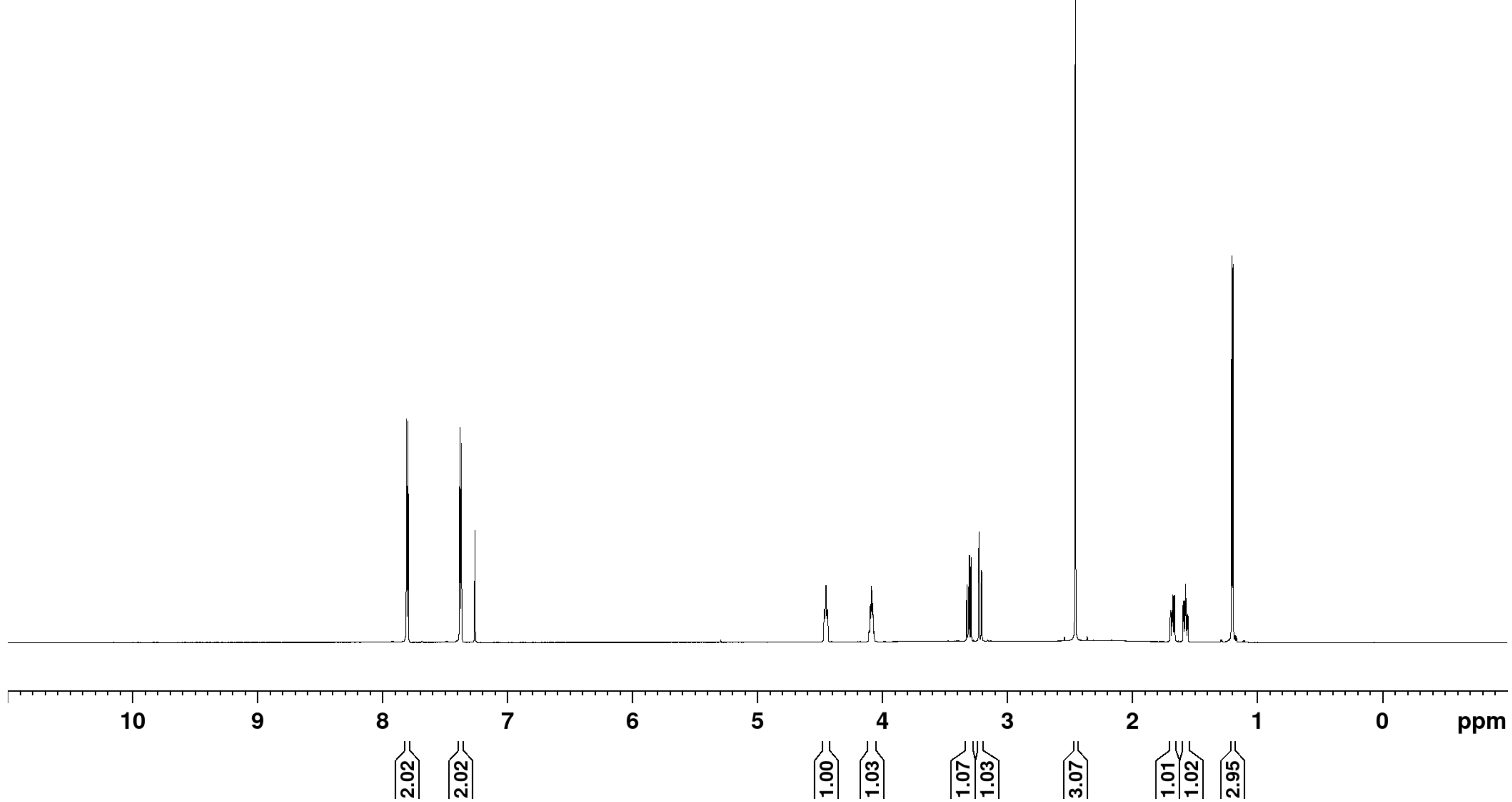

(*anti*)-(2*R*,4*S*)-1-tosylpentane-2,4-diol (*rac-anti*-3m)

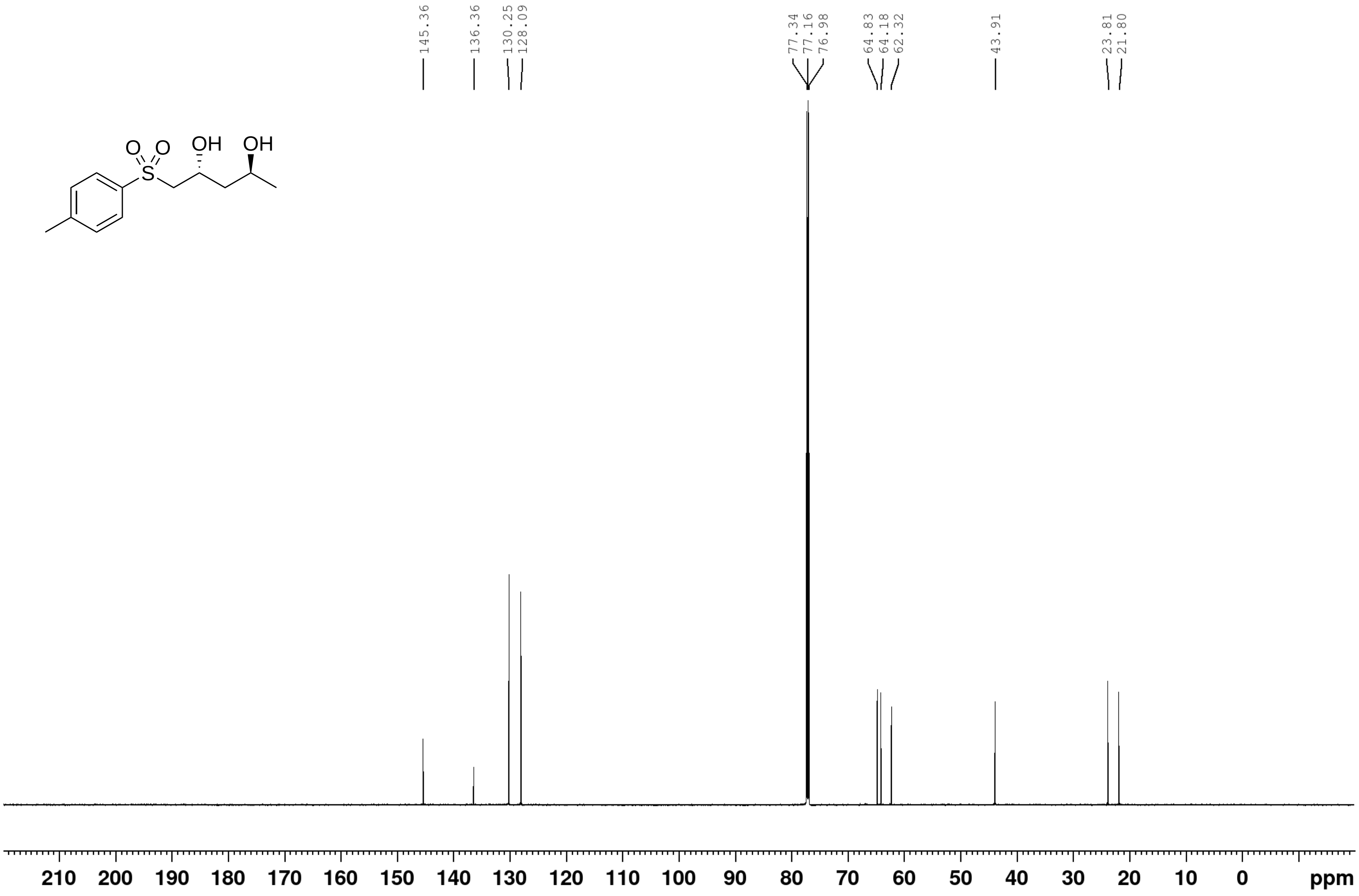

(*syn*)-(2*R*,4*S*)-1-tosylpentane-2,4-diol and (*anti*)-(2*R*,4*S*)-1-tosylpentane-2,4-diol (*rac*-*syn*-3m)

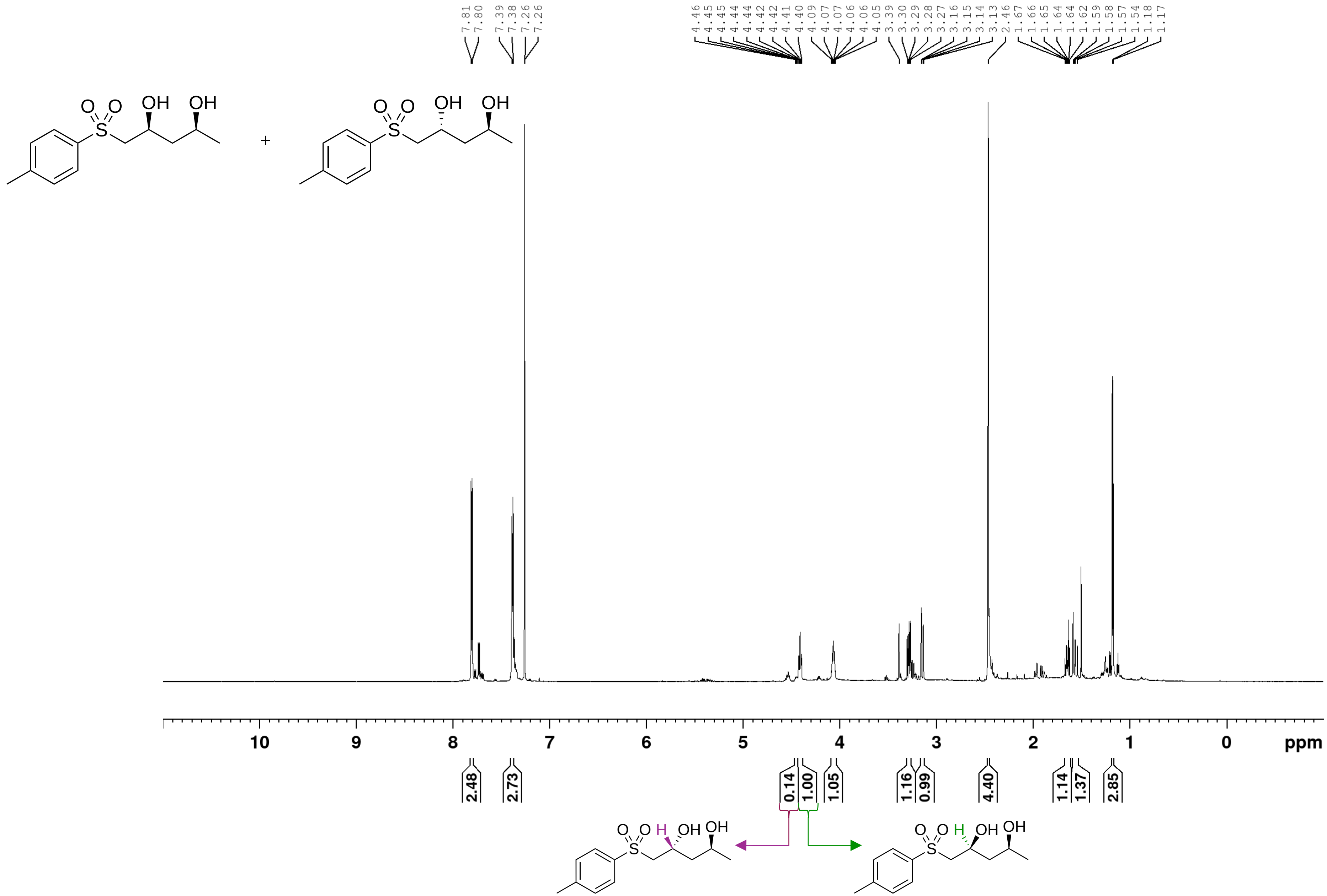

(*syn*)-(2*R*,4*S*)-1-tosylpentane-2,4-diol and (*anti*)-(2*R*,4*S*)-1-tosylpentane-2,4-diol (*rac*-*syn*-3m)

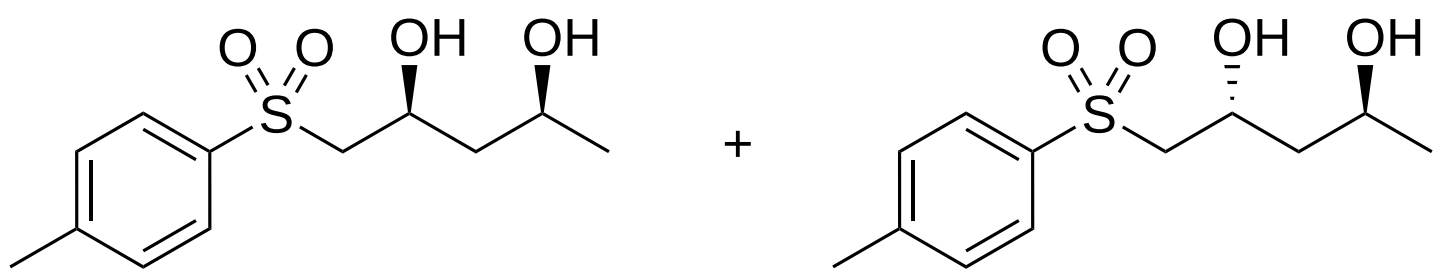

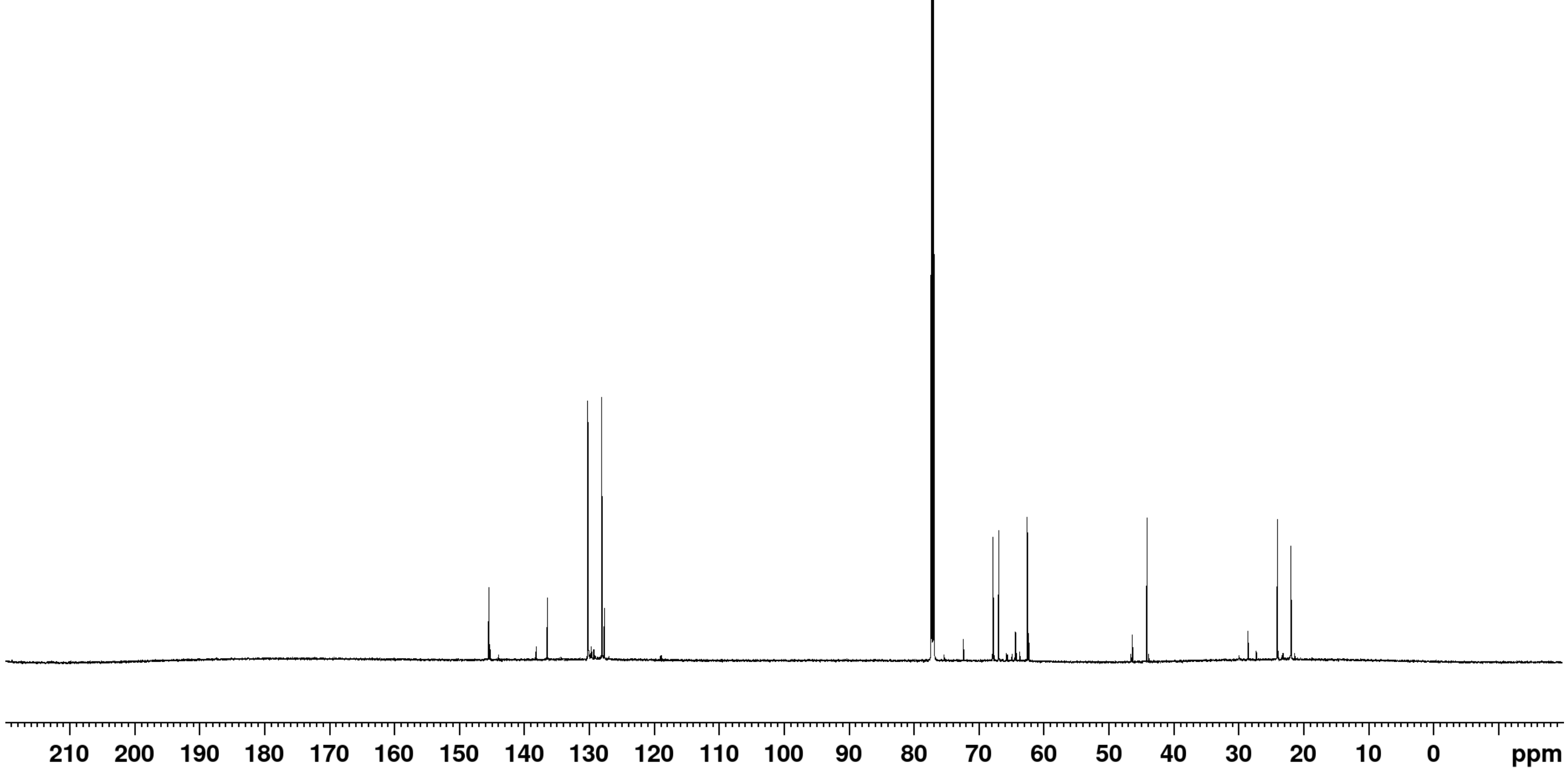

7-hydroxyundecan-5-one (*rac*-2c)

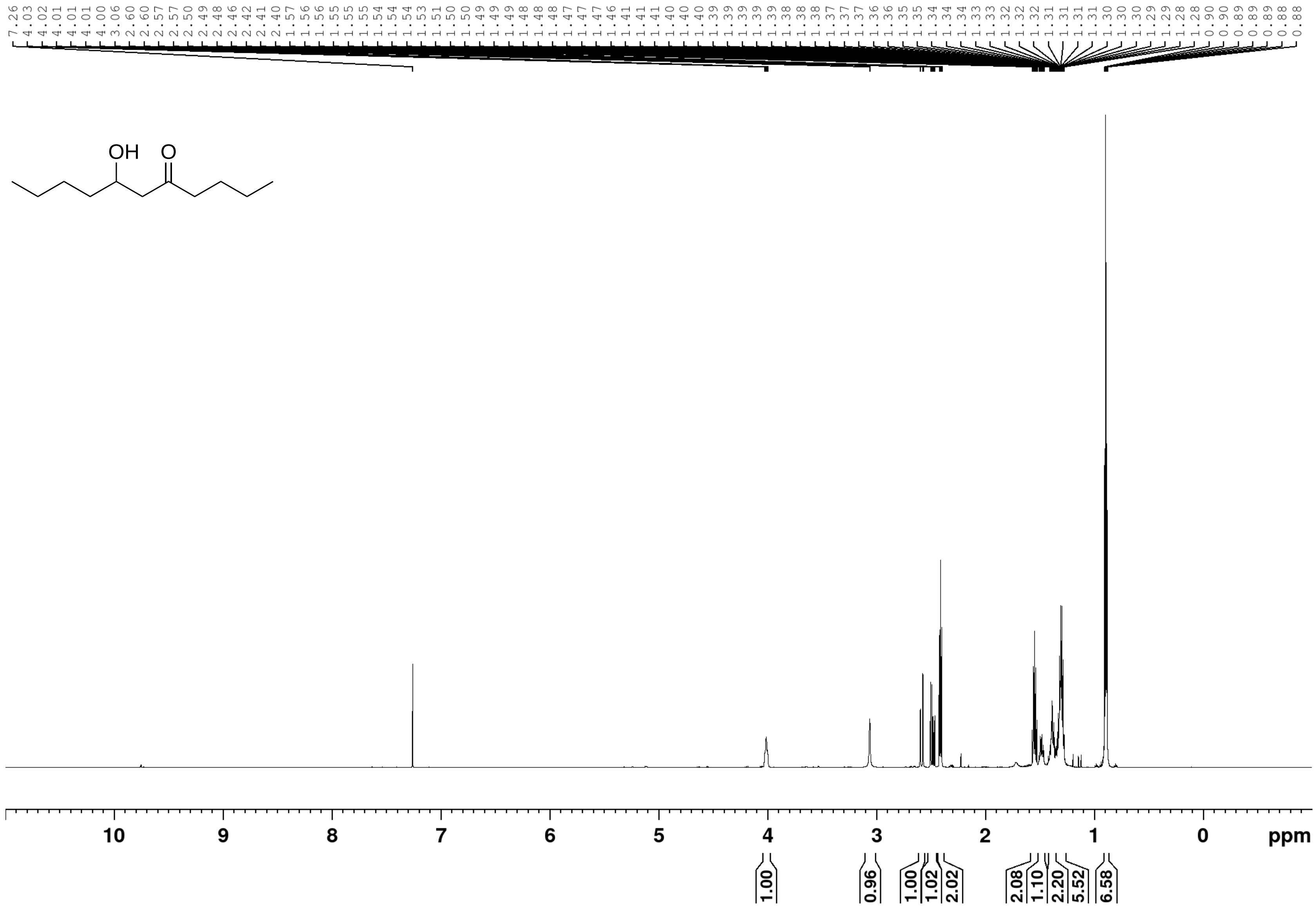

7-hydroxyundecan-5-one (*rac*-2c)

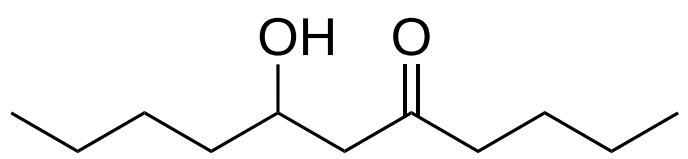

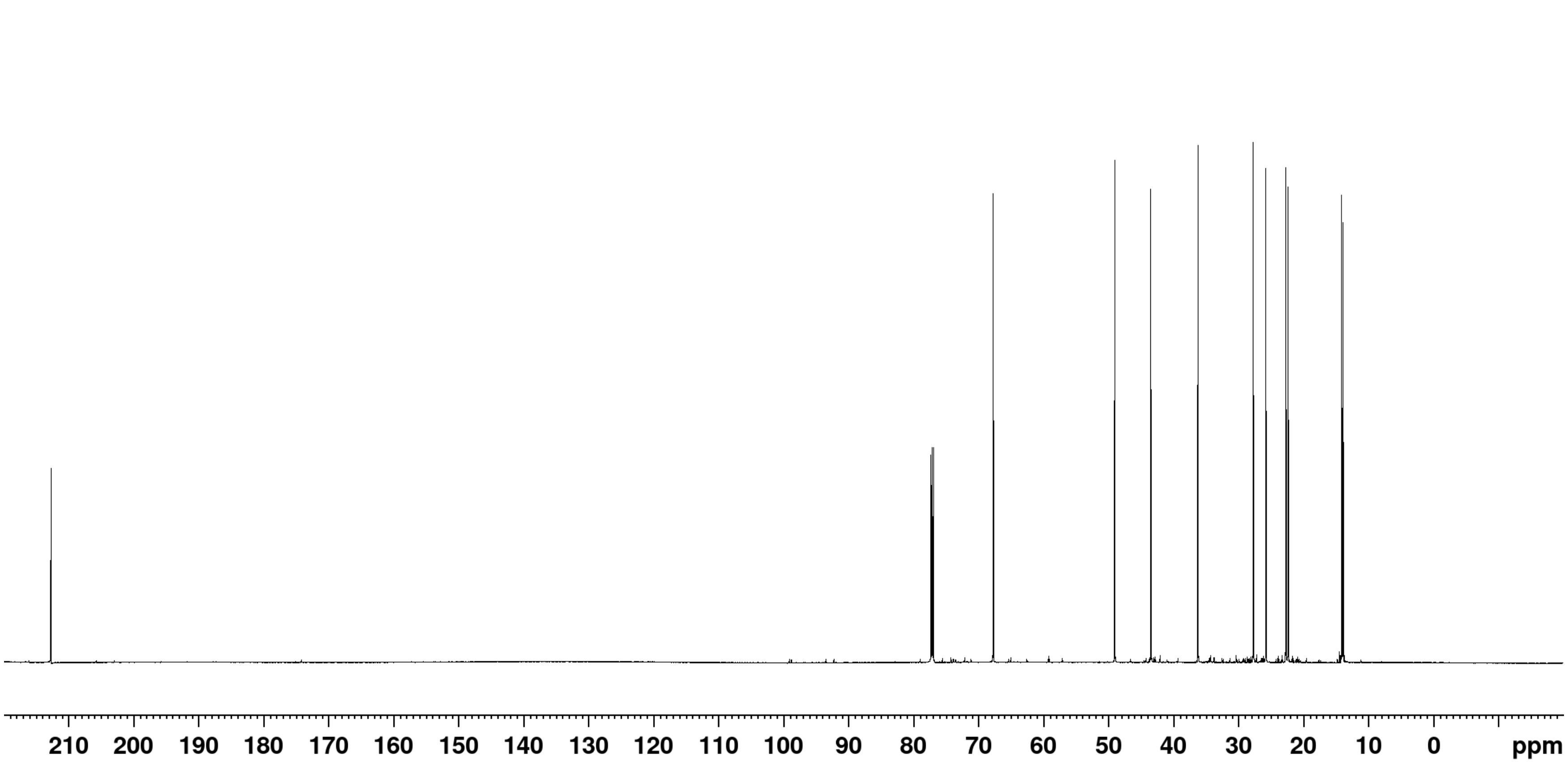

5-hydroxy-1,7-diphenylheptan-3-one (*rac*-2d)

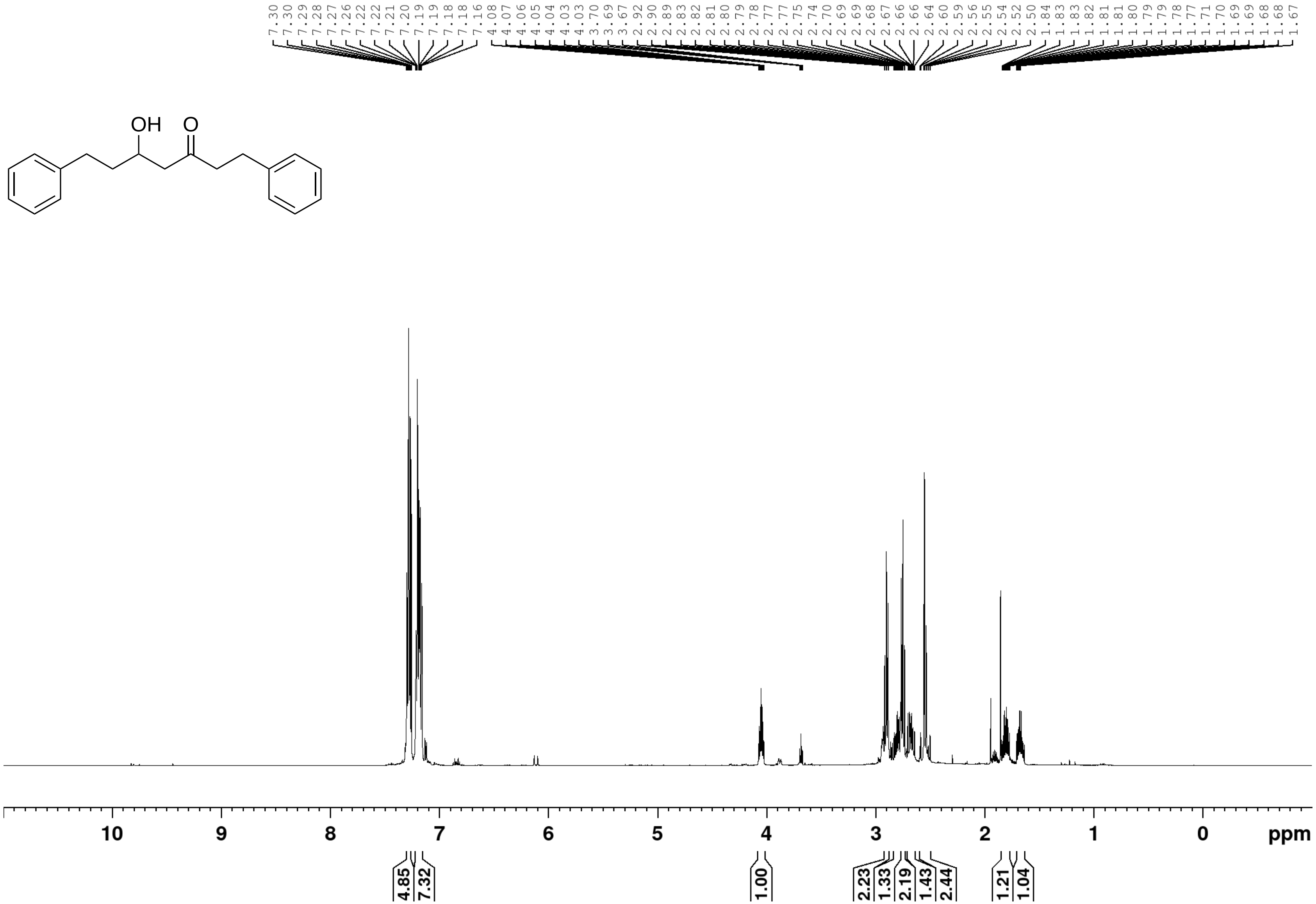

5-hydroxy-1,7-diphenylheptan-3-one (*rac*-2d)

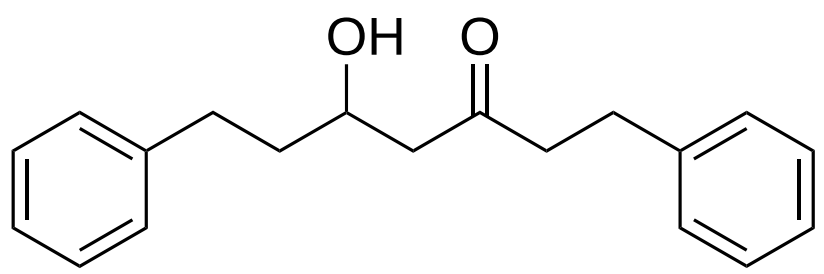

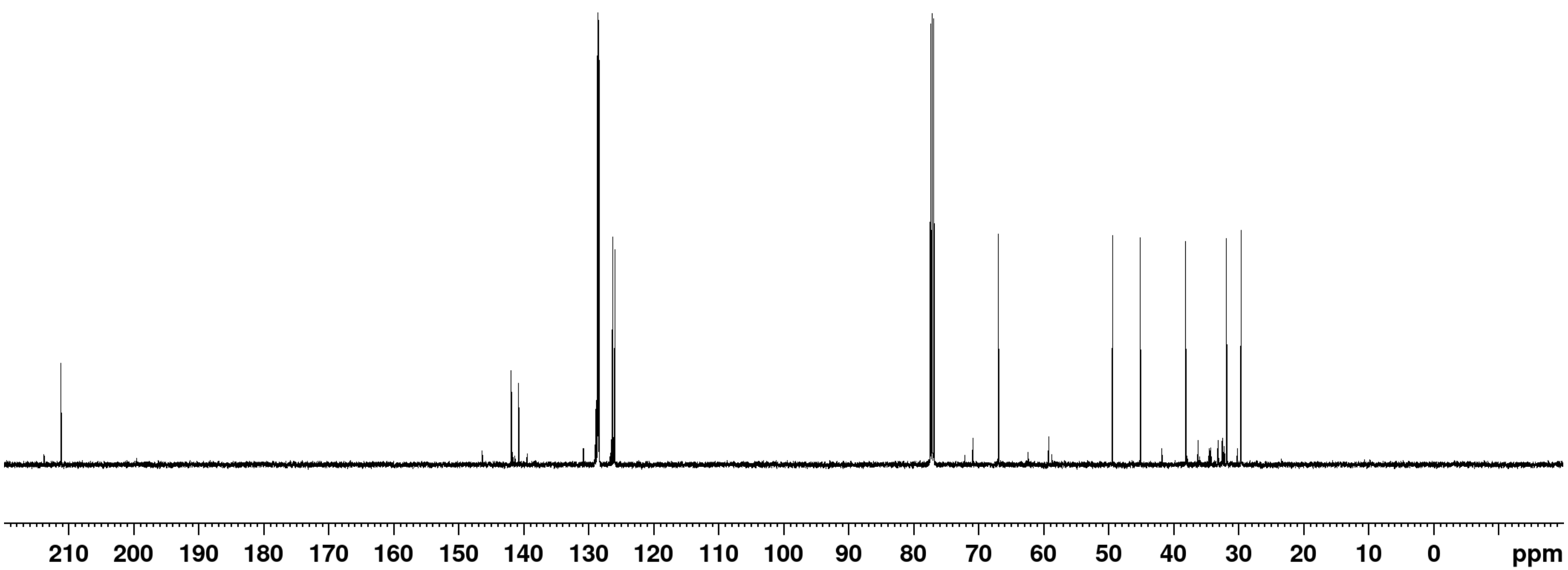

6-hydroxy-2,8-dimethylnonan-4-one (*rac*-2e)

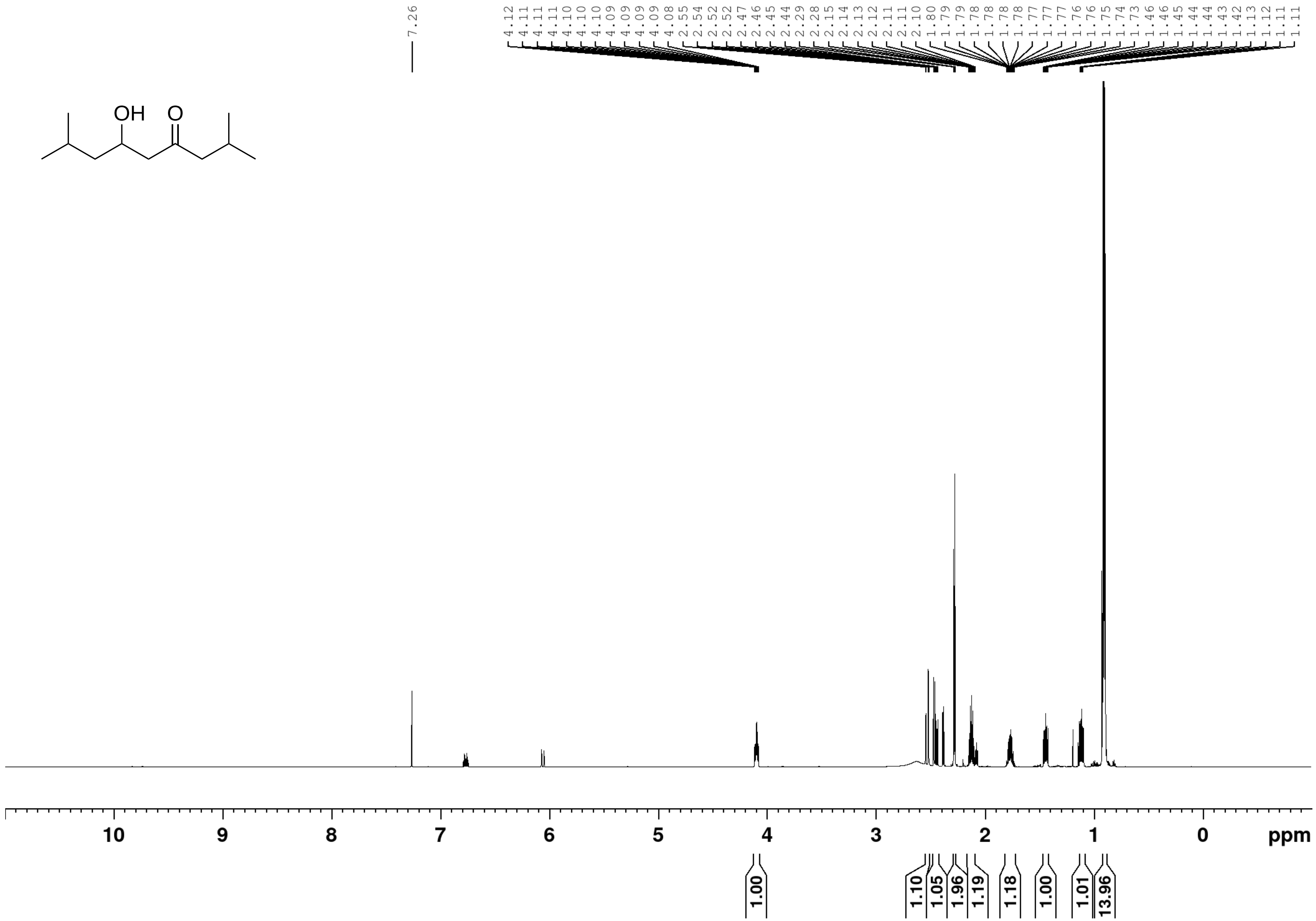

6-hydroxy-2,8-dimethylnonan-4-one (*rac*-2e)

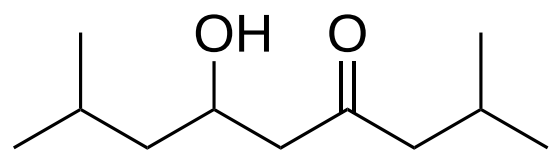

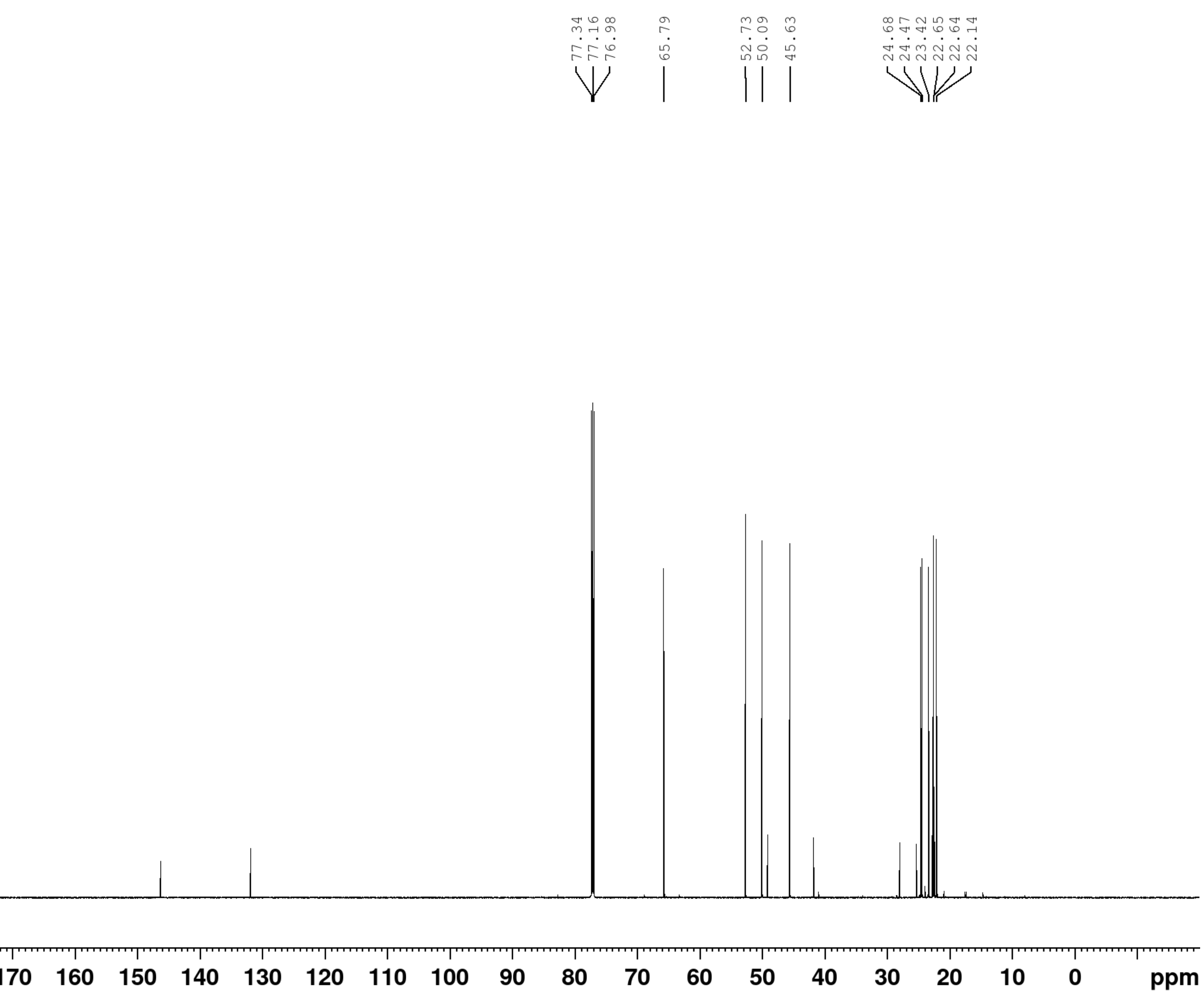

undecane-5,7-dione (*int*$_1$-1d-d4)

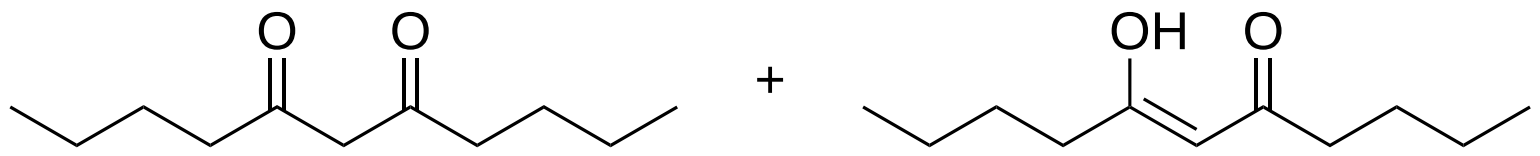

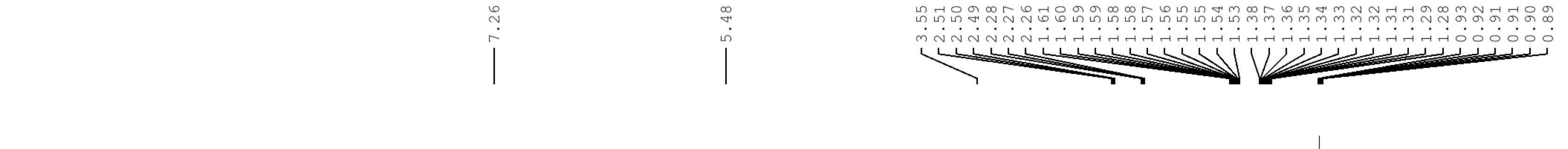

undecane-5,7-dione (*int₁*-1d-d4)

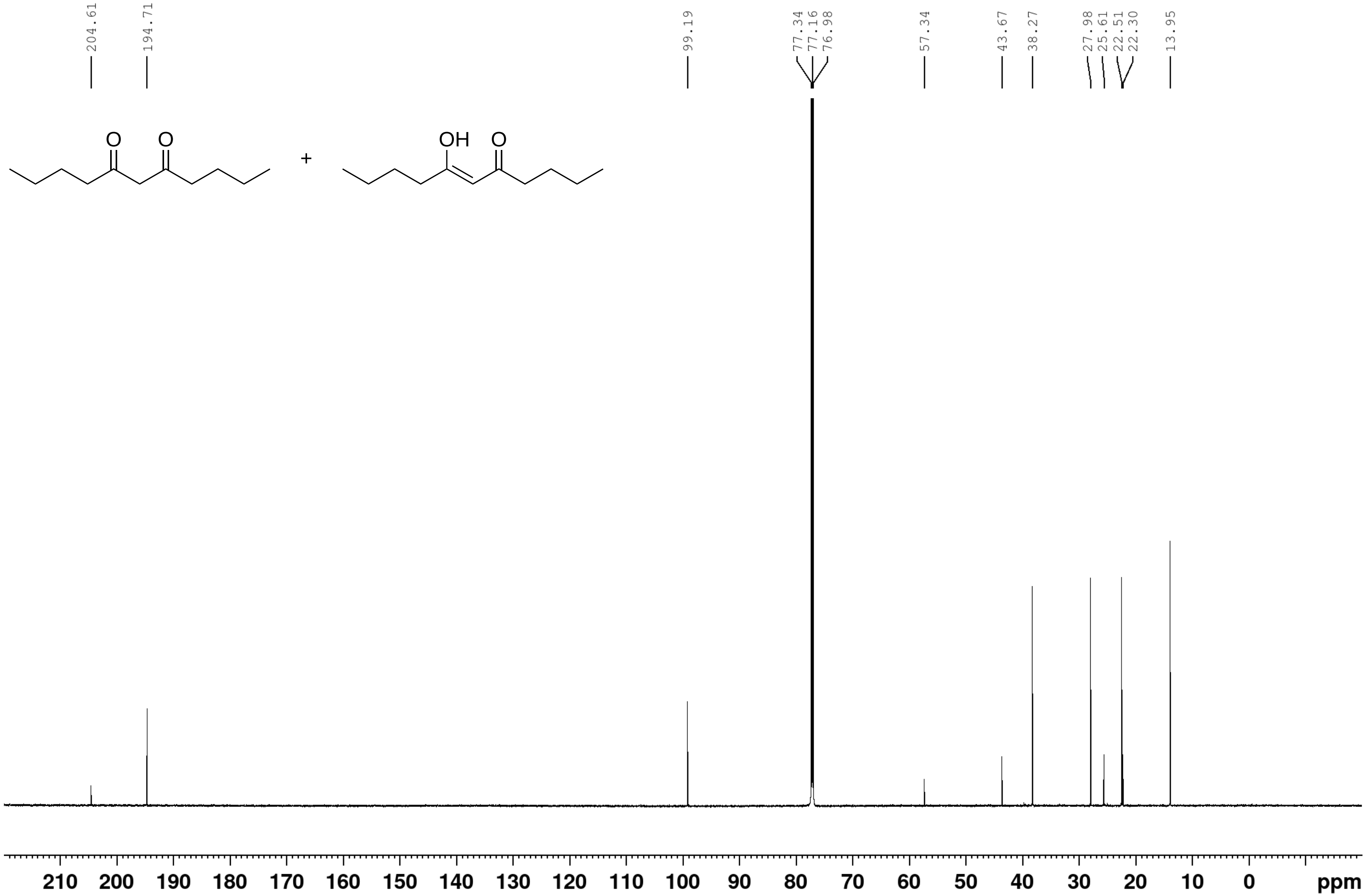

(*syn*)-undecane-5,6,6,7-d_4-5,7-diol (*meso*-1d)

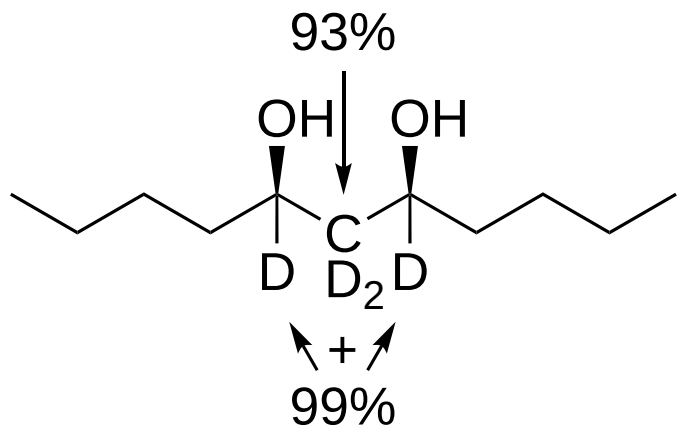

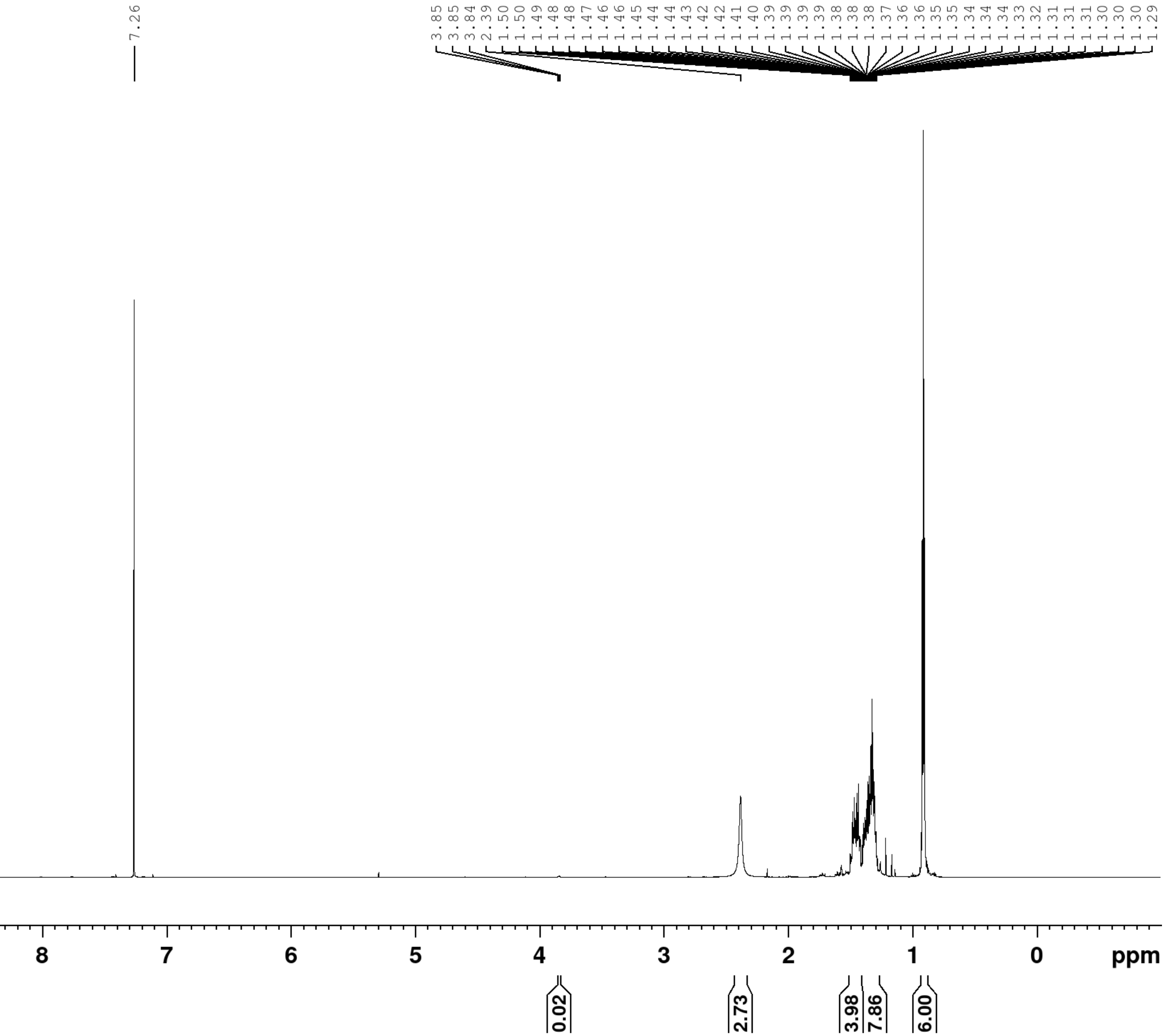

(*syn*)-undecane-5,6,6,7-d_4-5,7-diol (*meso*-1d)

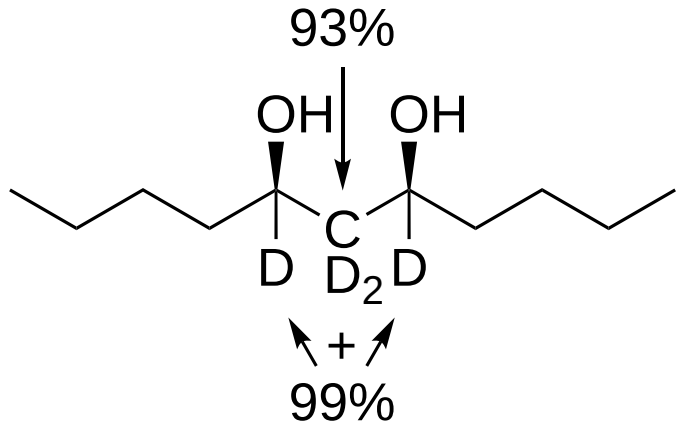

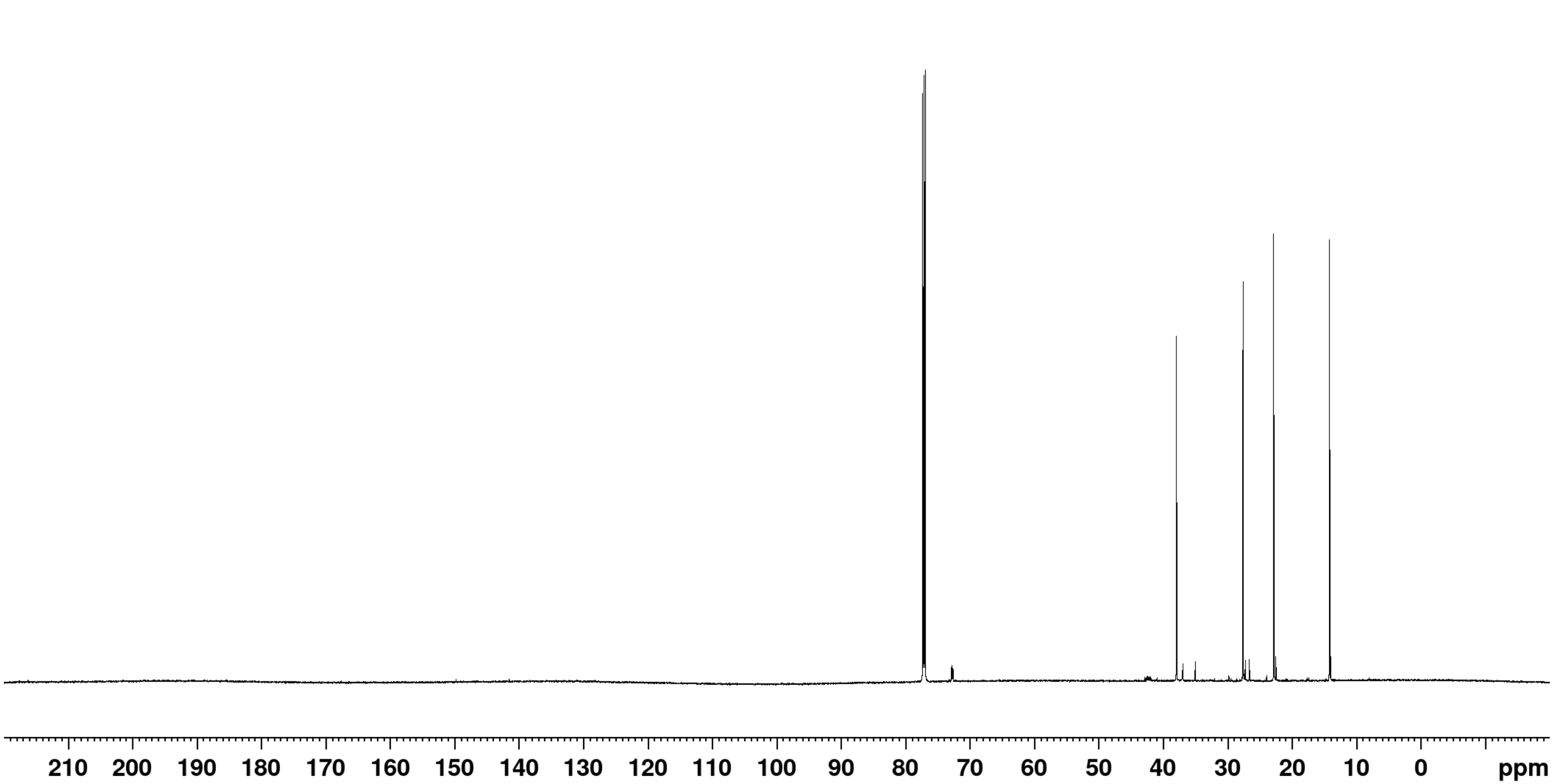

(*syn*)-undecane-5,6,6,7-d_4-5,7-diol (*meso*-1d-d4)

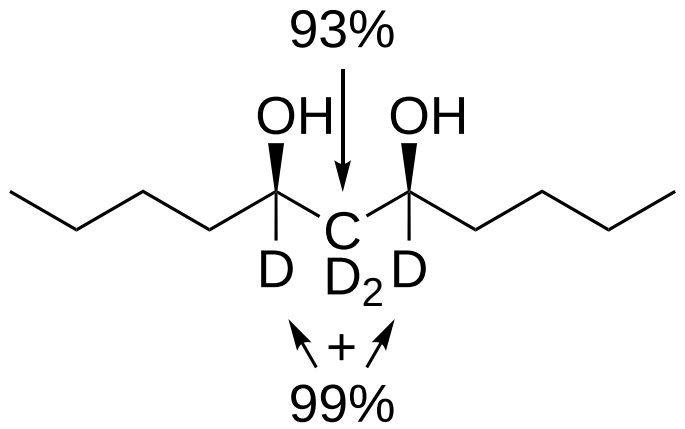

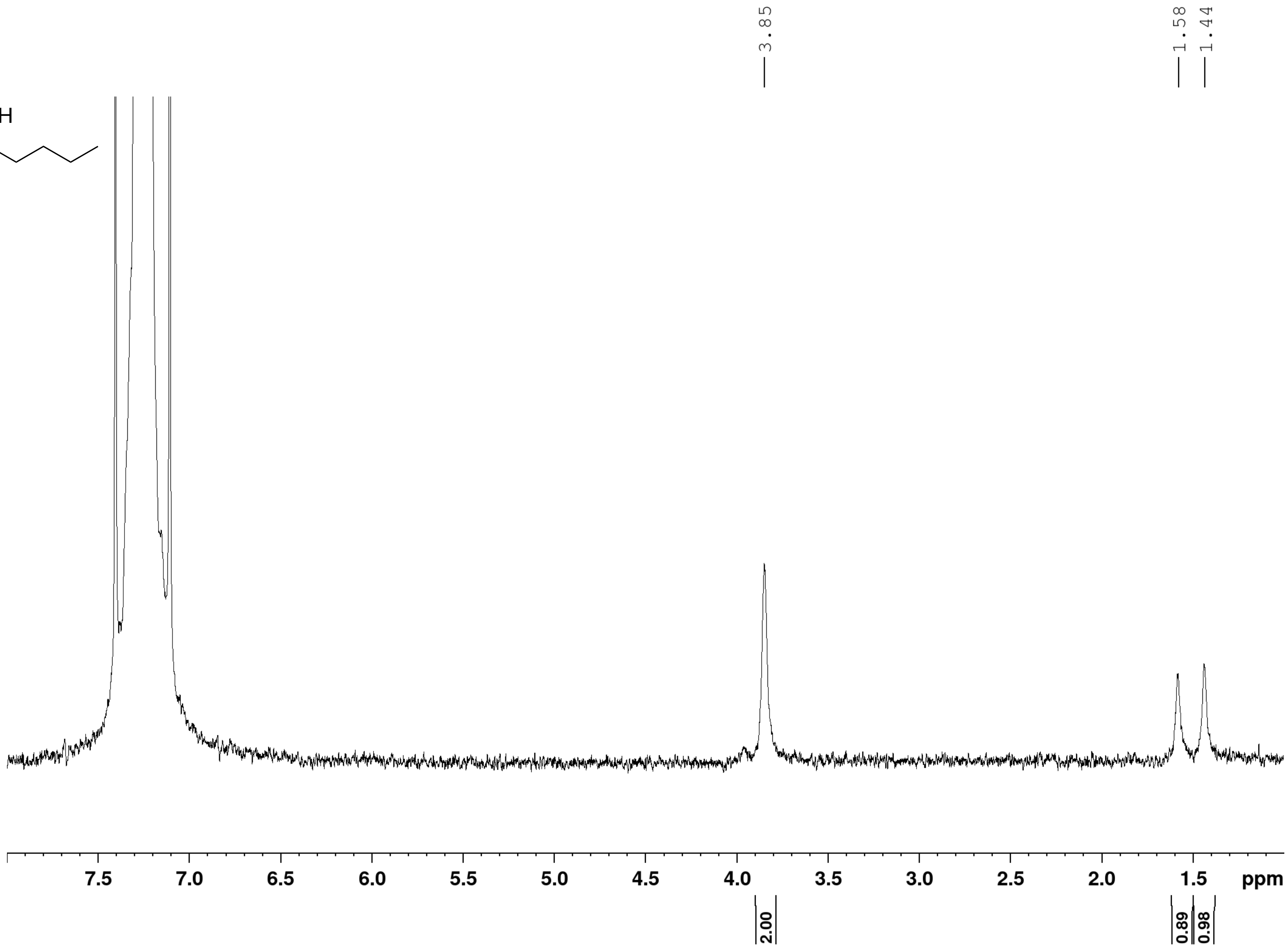

(*anti*)-undecane-5,6,6,7-d_4-5,7-diol (*rac*-1d)

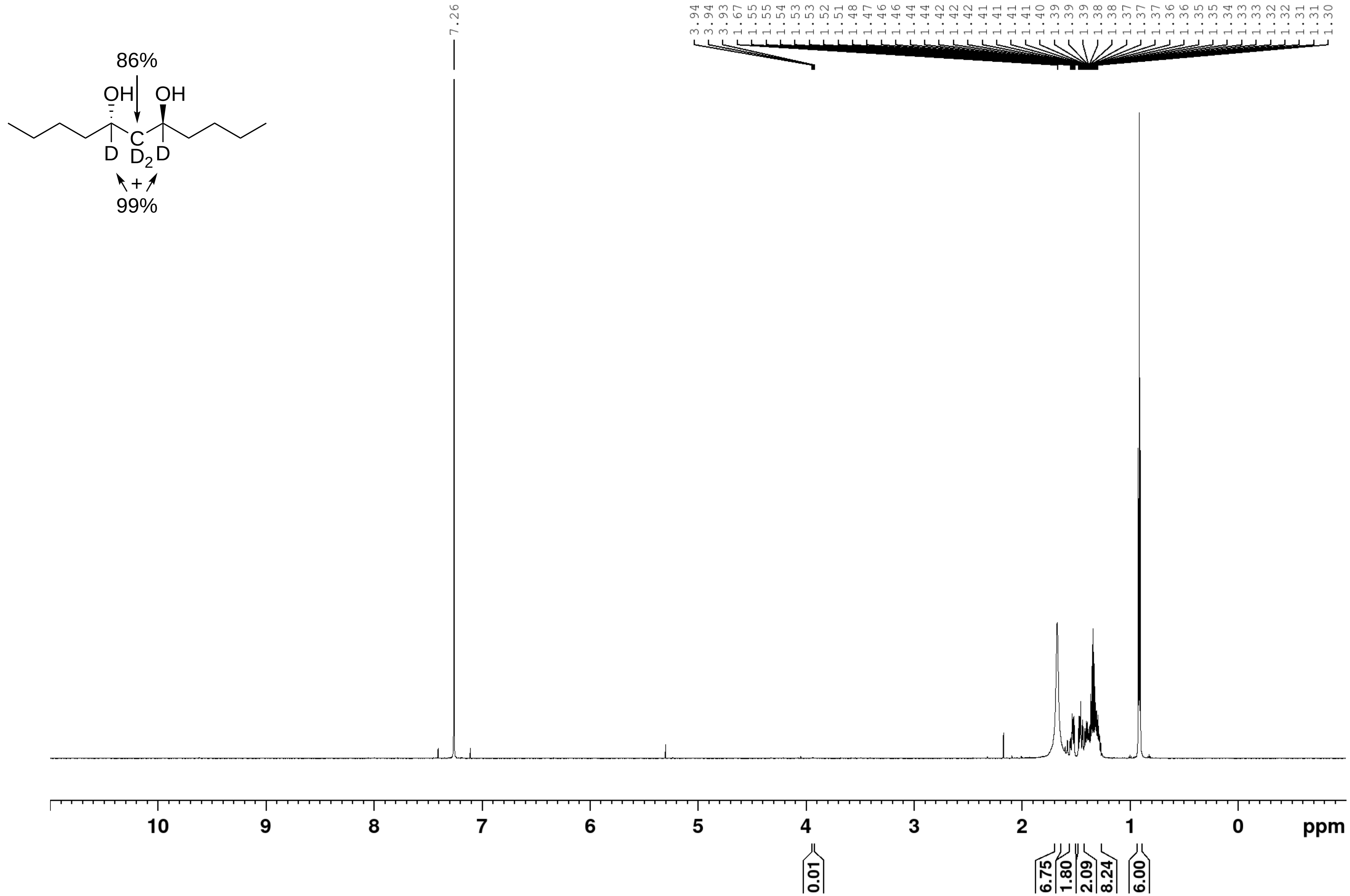

(*anti*)-undecane-5,6,6,7-d_4-5,7-diol (*rac*-1d)

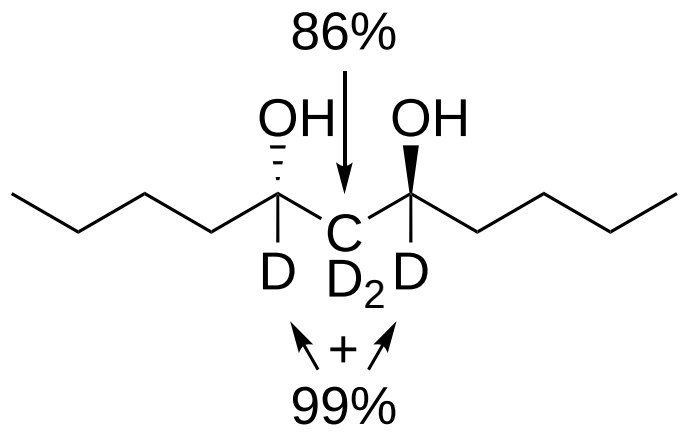

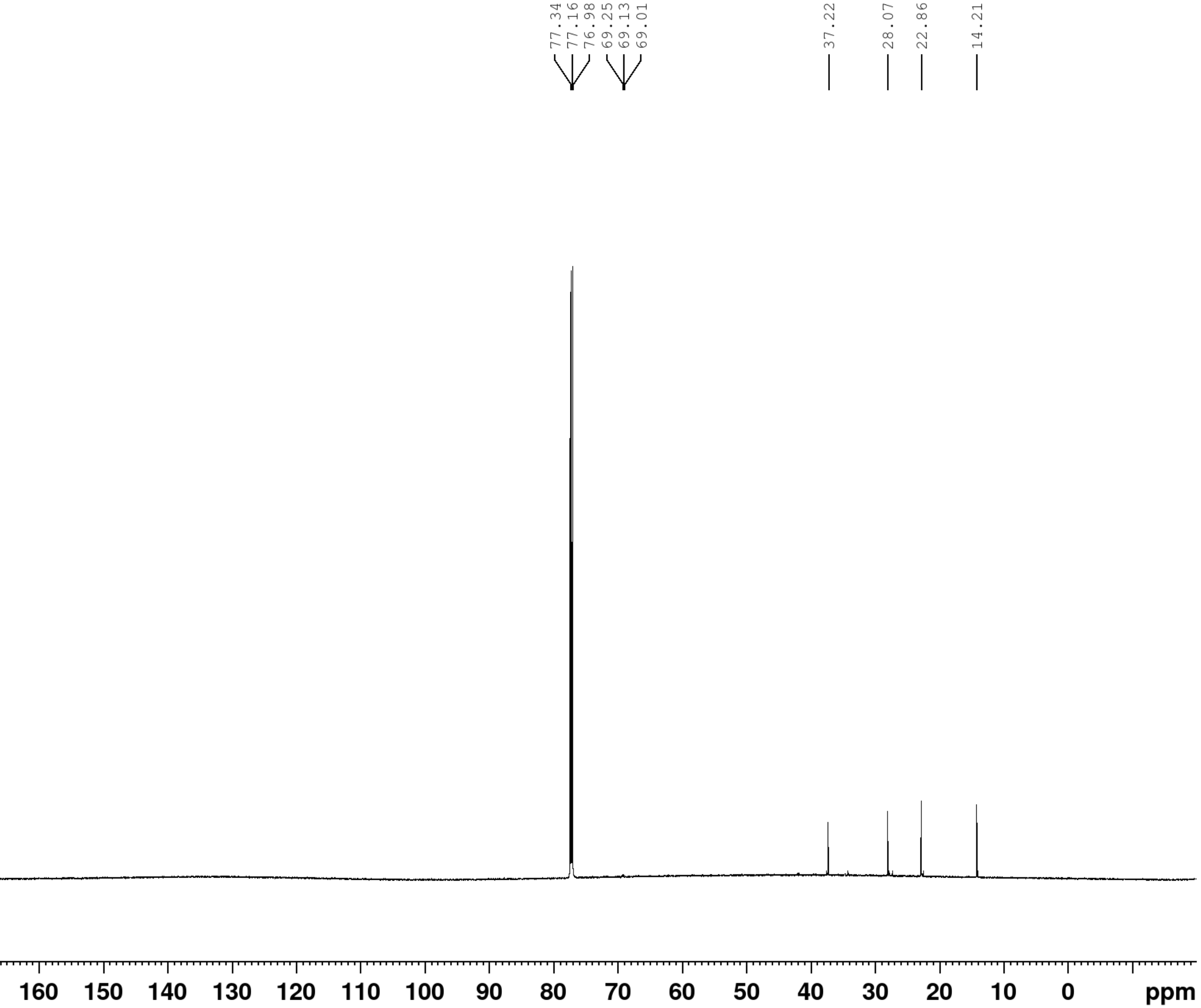

(*anti*)-undecane-5,6,6,7-d_4-5,7-diol (*rac*-1d)

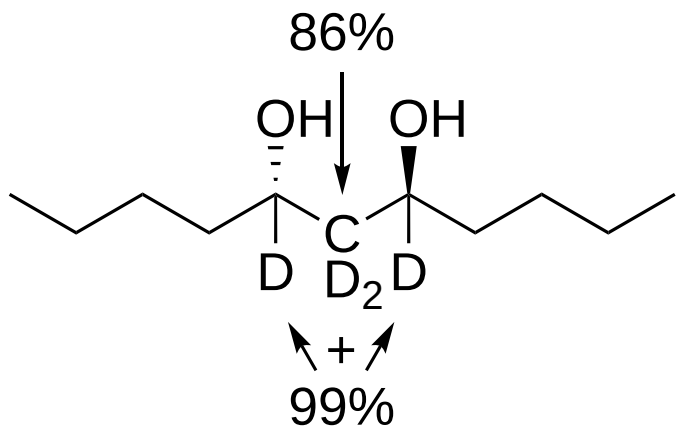

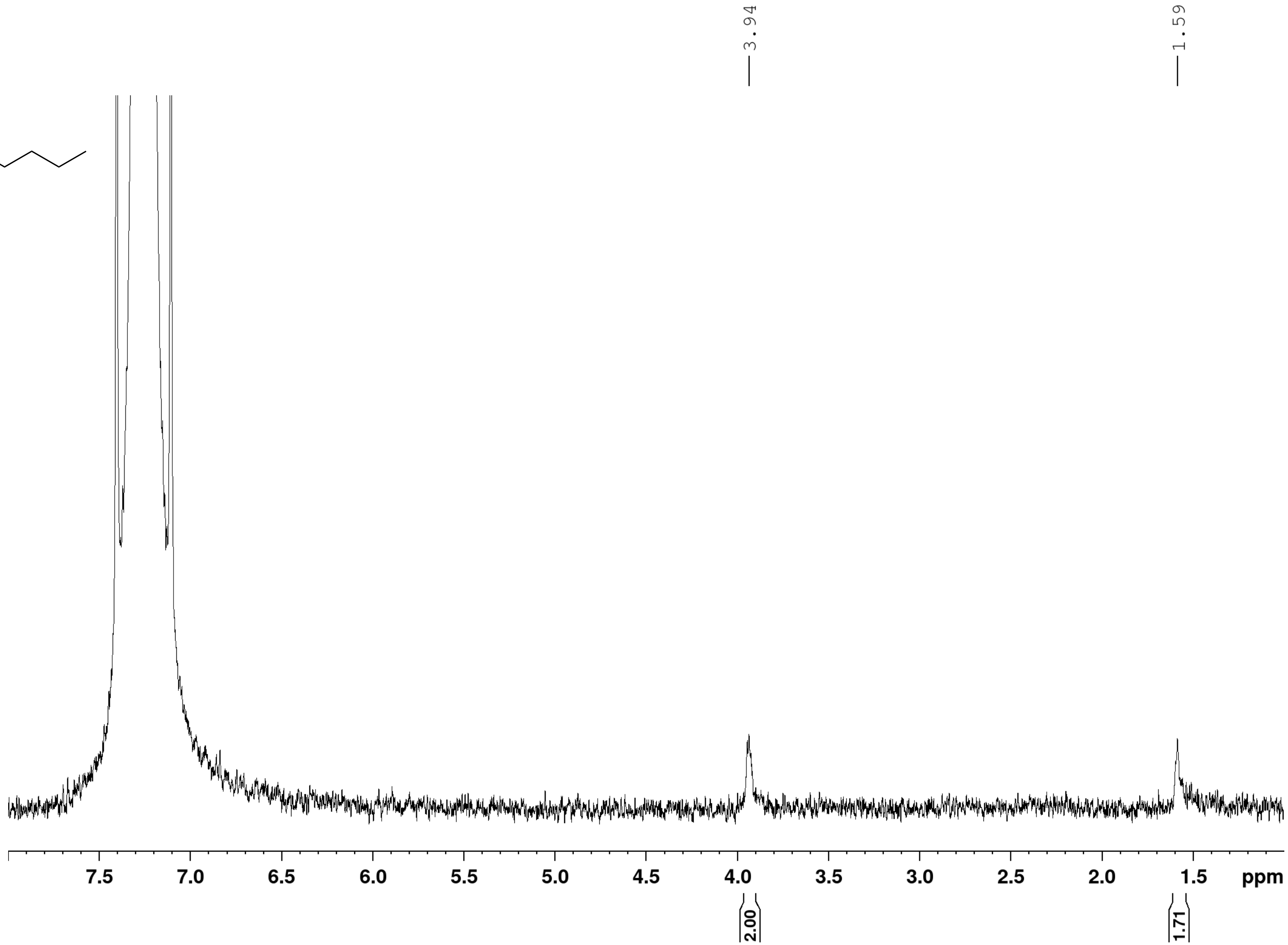

(*anti*)-1-Phenylpentane-2,4-diol (*rac*-*anti*-3a)

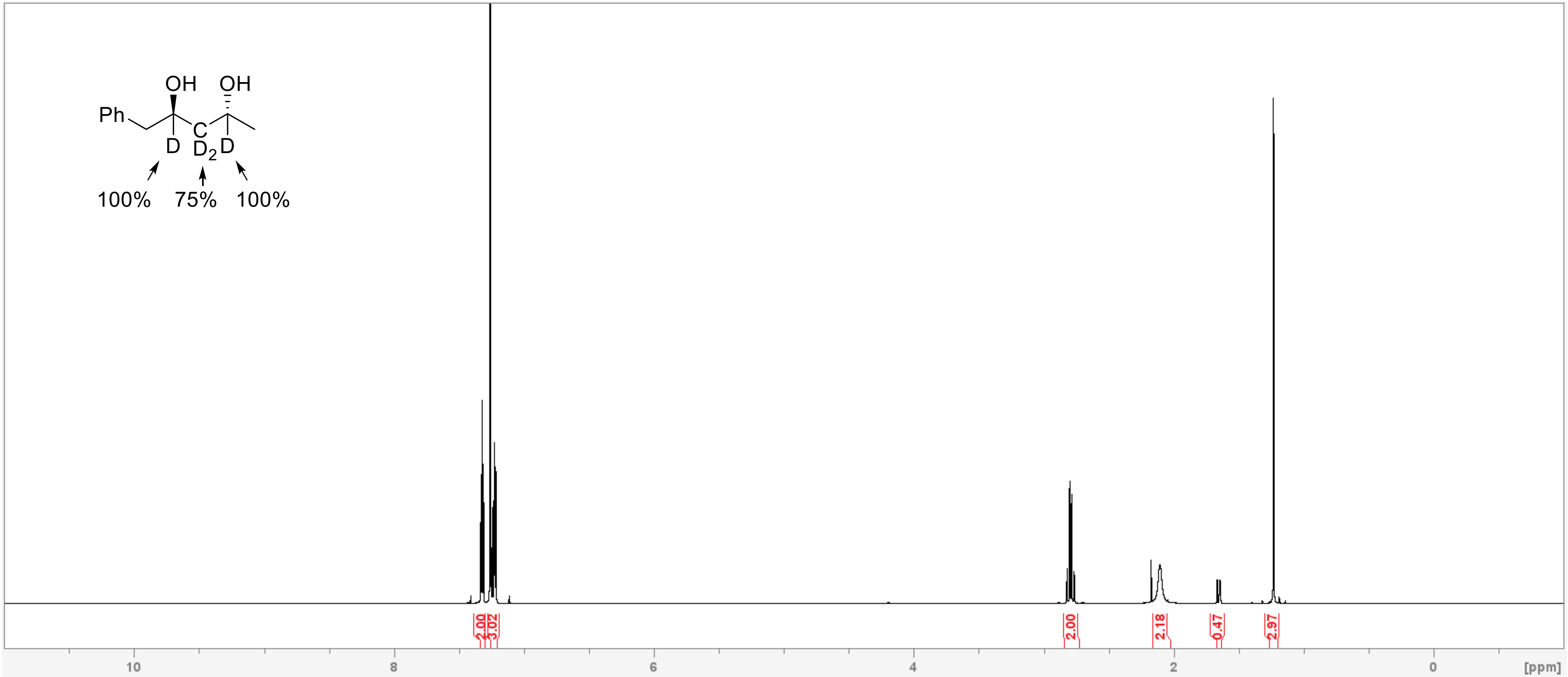

(*anti*)-1-Phenylpentane-2,4-diol (*rac-anti*-3a)

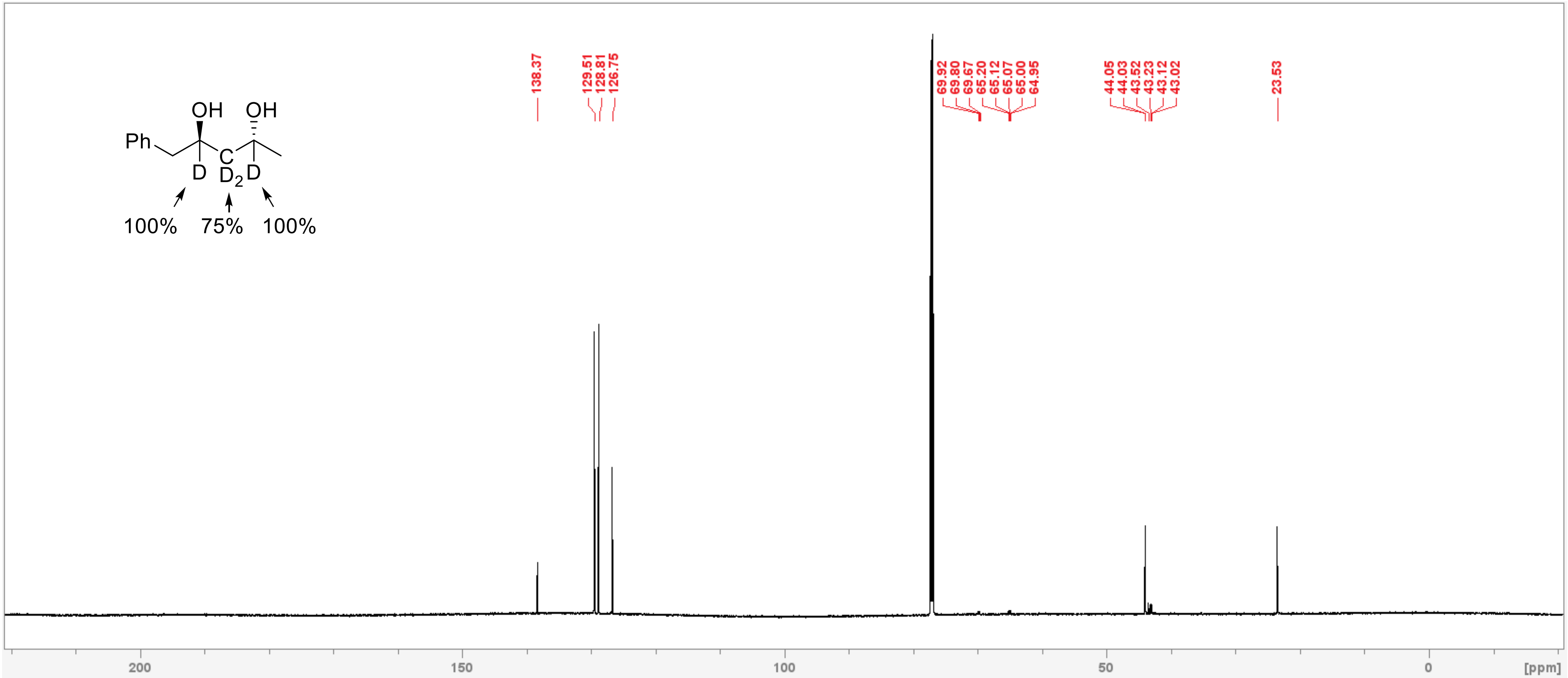

(*anti*)-1-Phenylpentane-2,4-diol (*rac*-*anti*-3a)

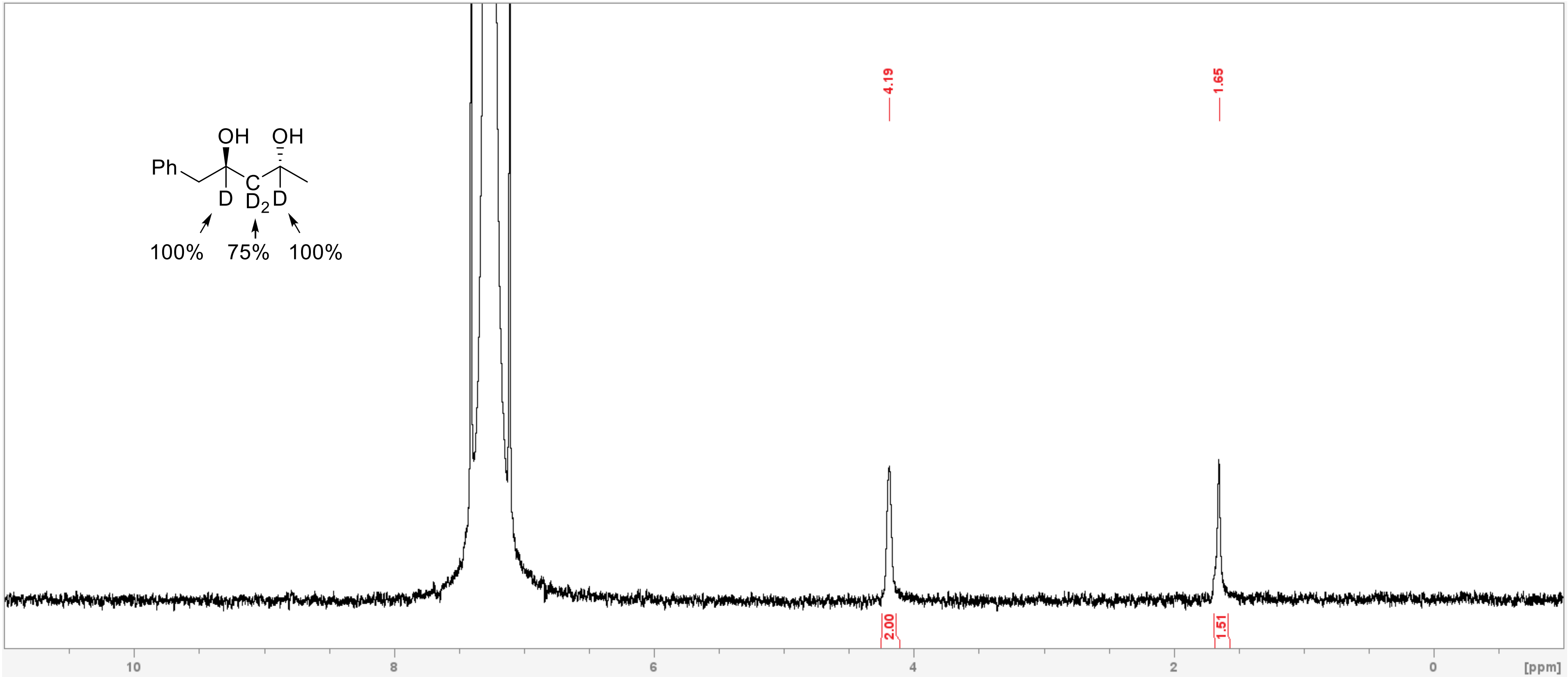

(*anti*)-1-Phenylpentane-2,4-diol (*rac*-*anti*-3a)

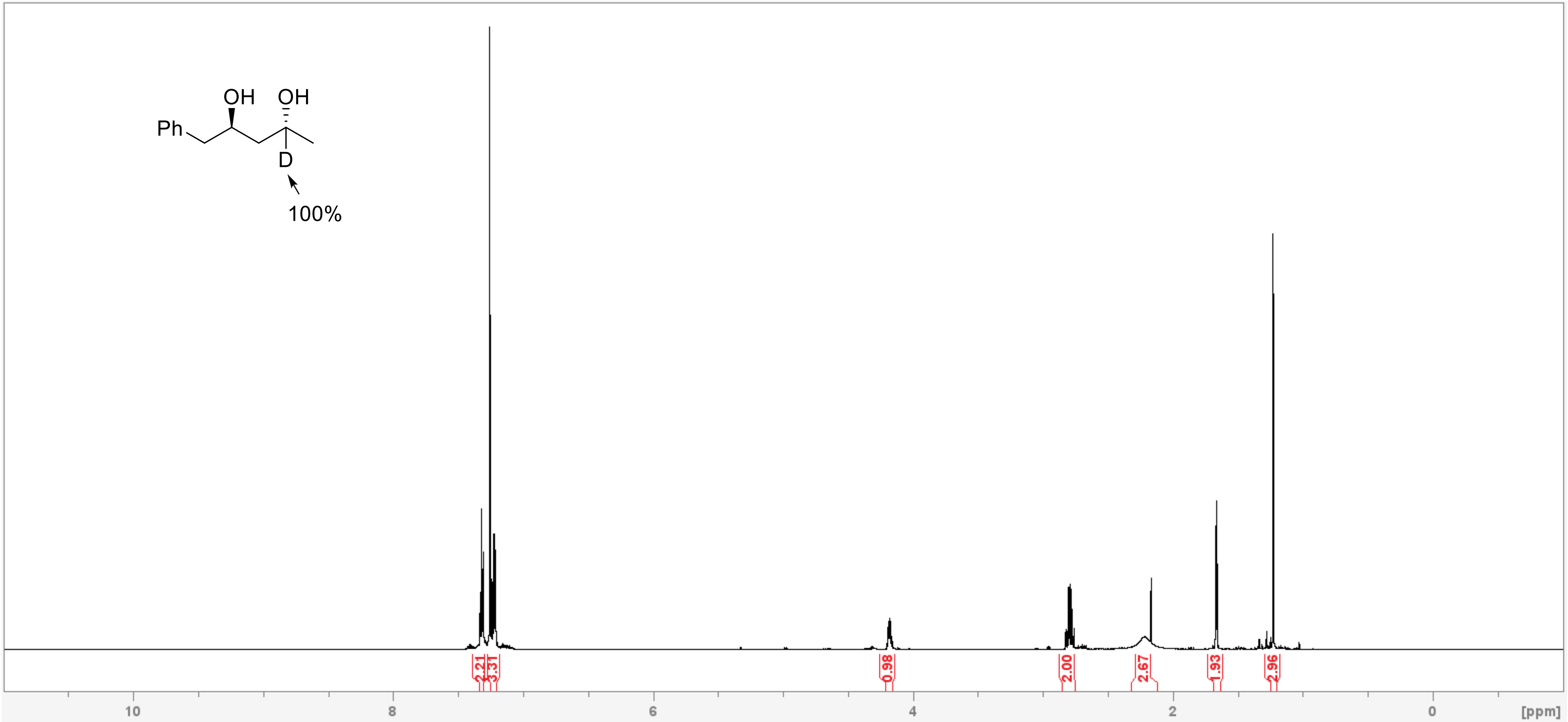

(*anti*)-1-Phenylpentane-2,4-diol (*rac-anti*-3a)

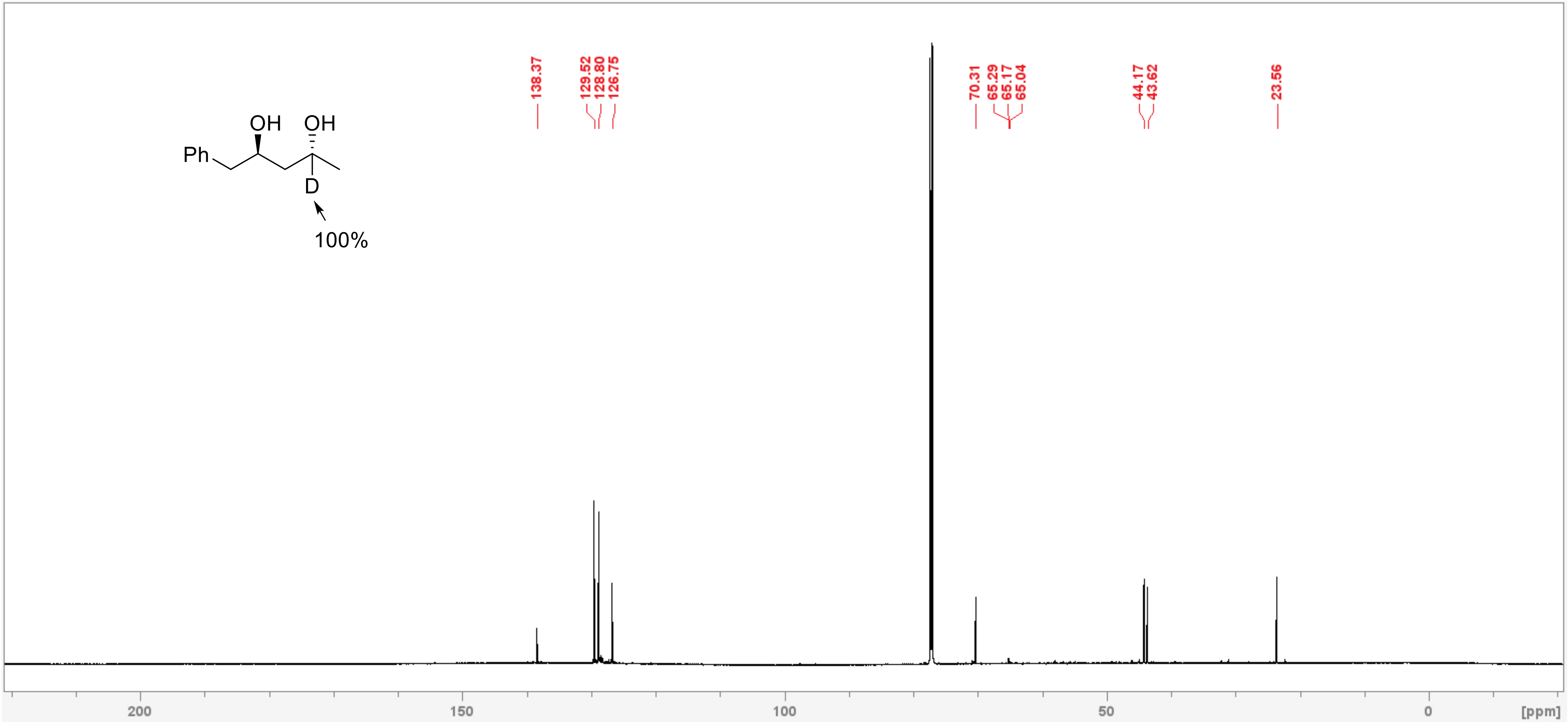

(*anti*)-1-Phenylpentane-2,4-diol (*rac*-*anti*-3a)

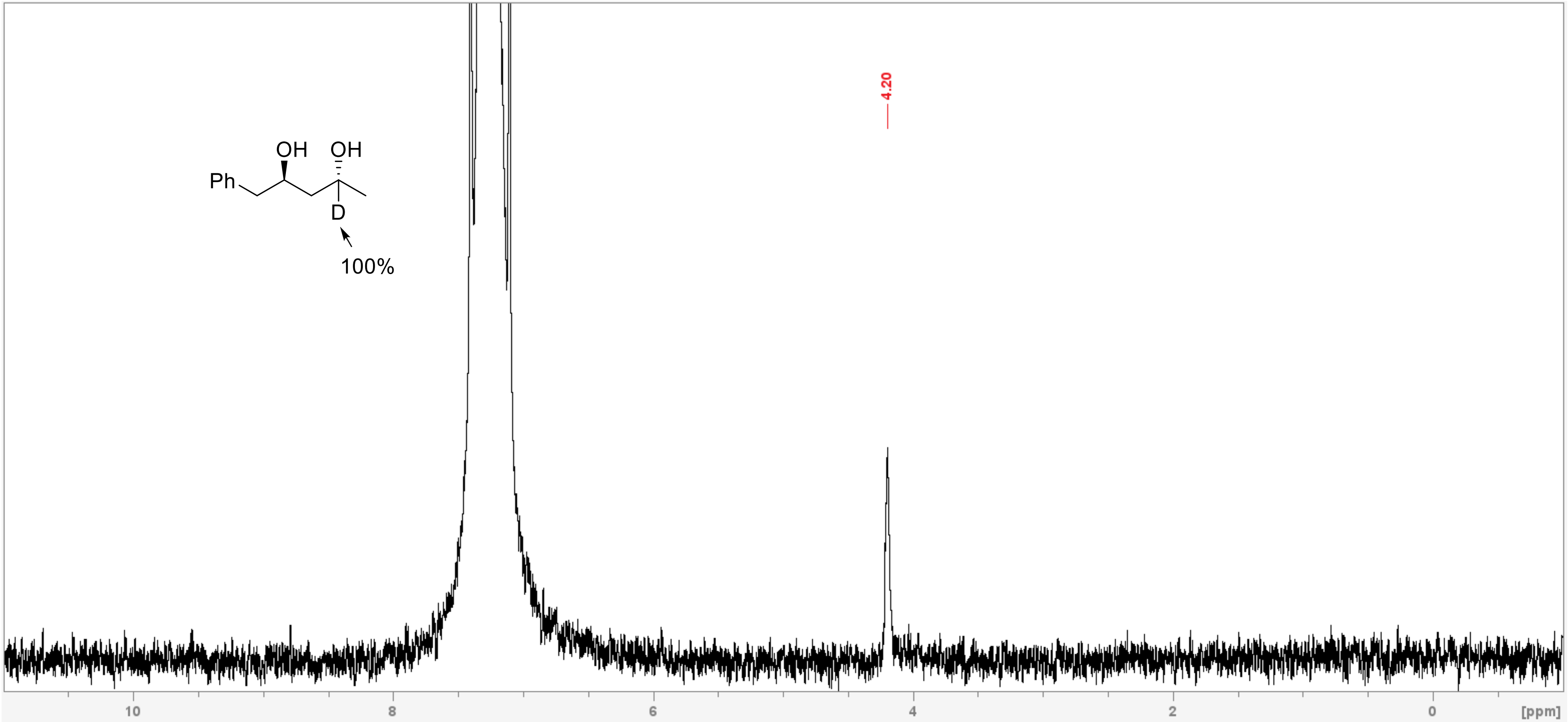

(*anti*)-6-Phenylhexane-2,4-diol (*rac*-*anti*-3d)

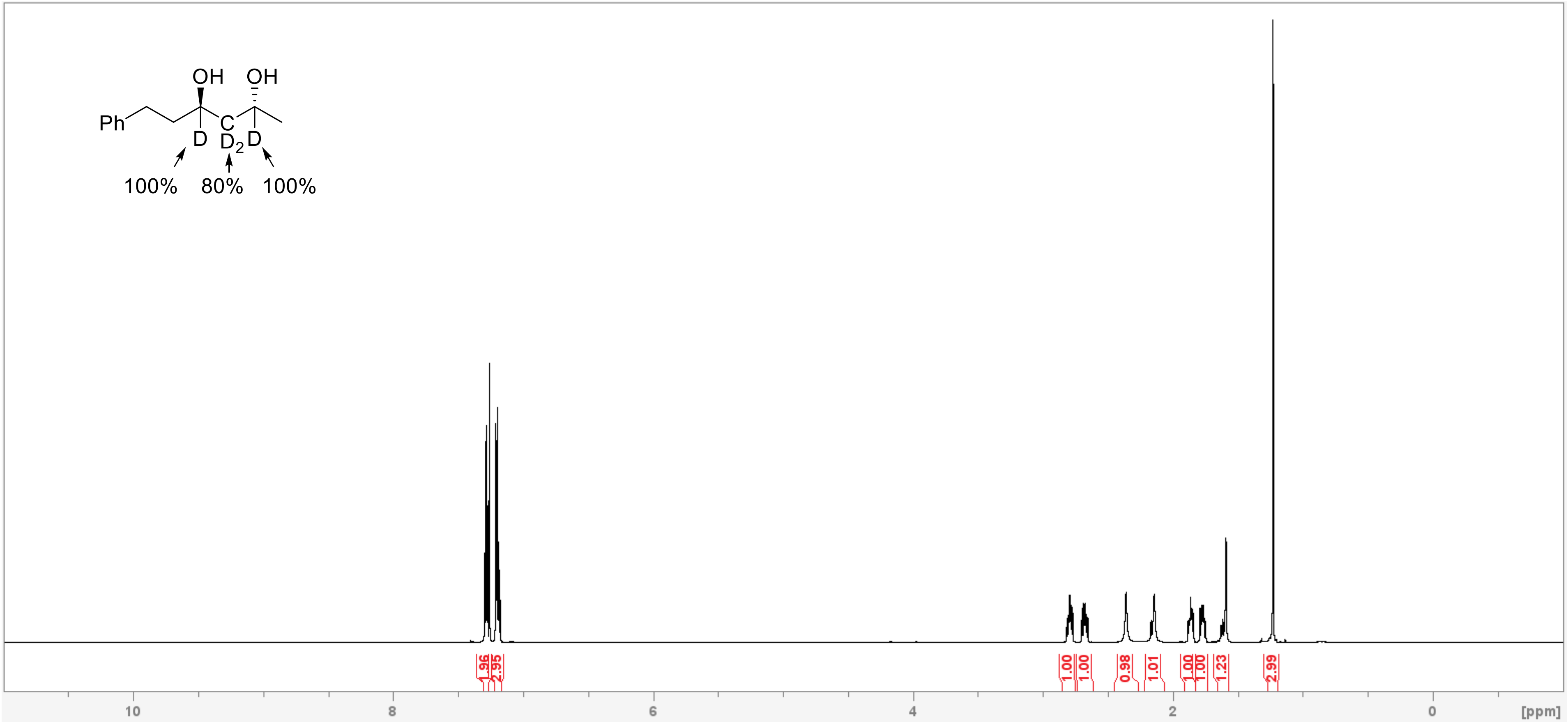

(*anti*)-6-Phenylhexane-2,4-diol (*rac*-*anti*-3d)

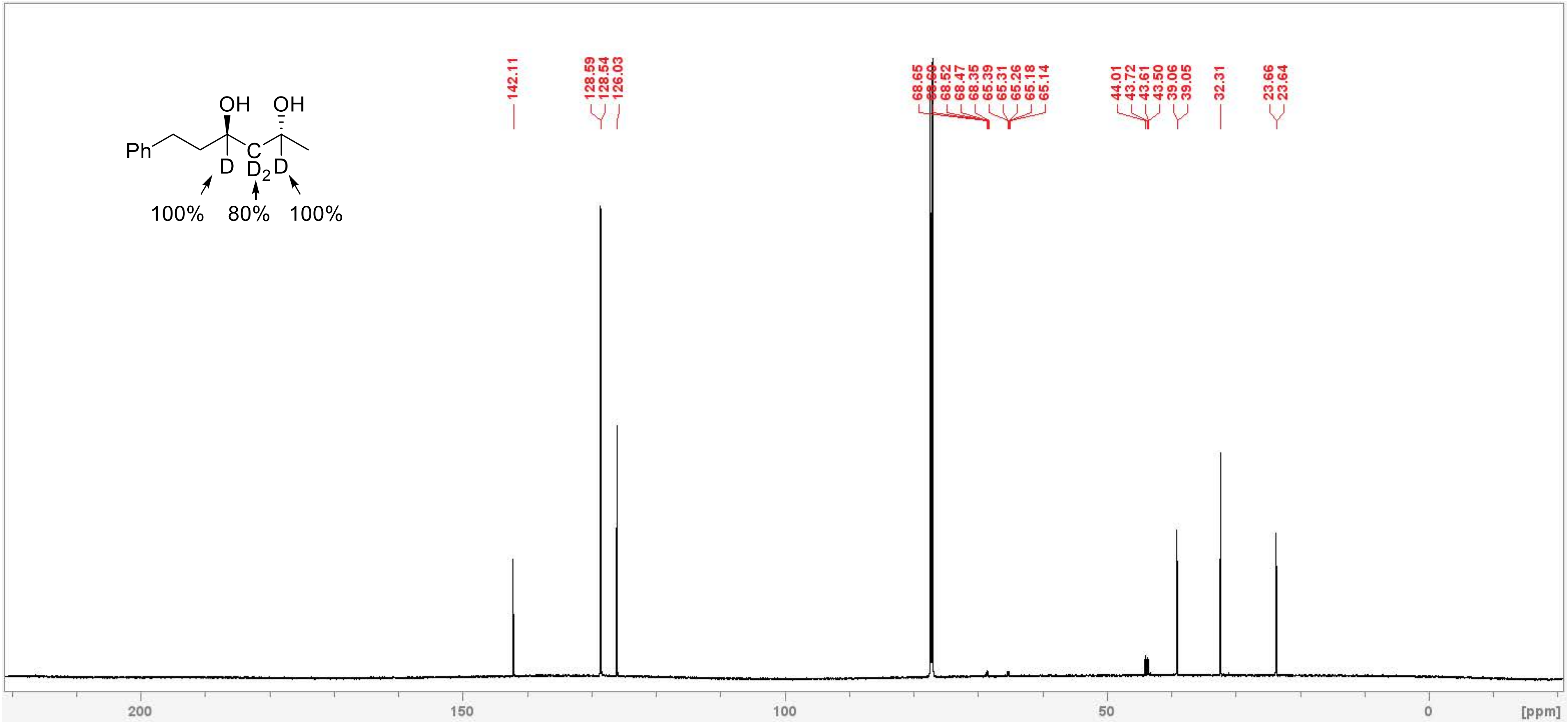

(*anti*)-6-Phenylhexane-2,4-diol (*rac*-*anti*-3d)

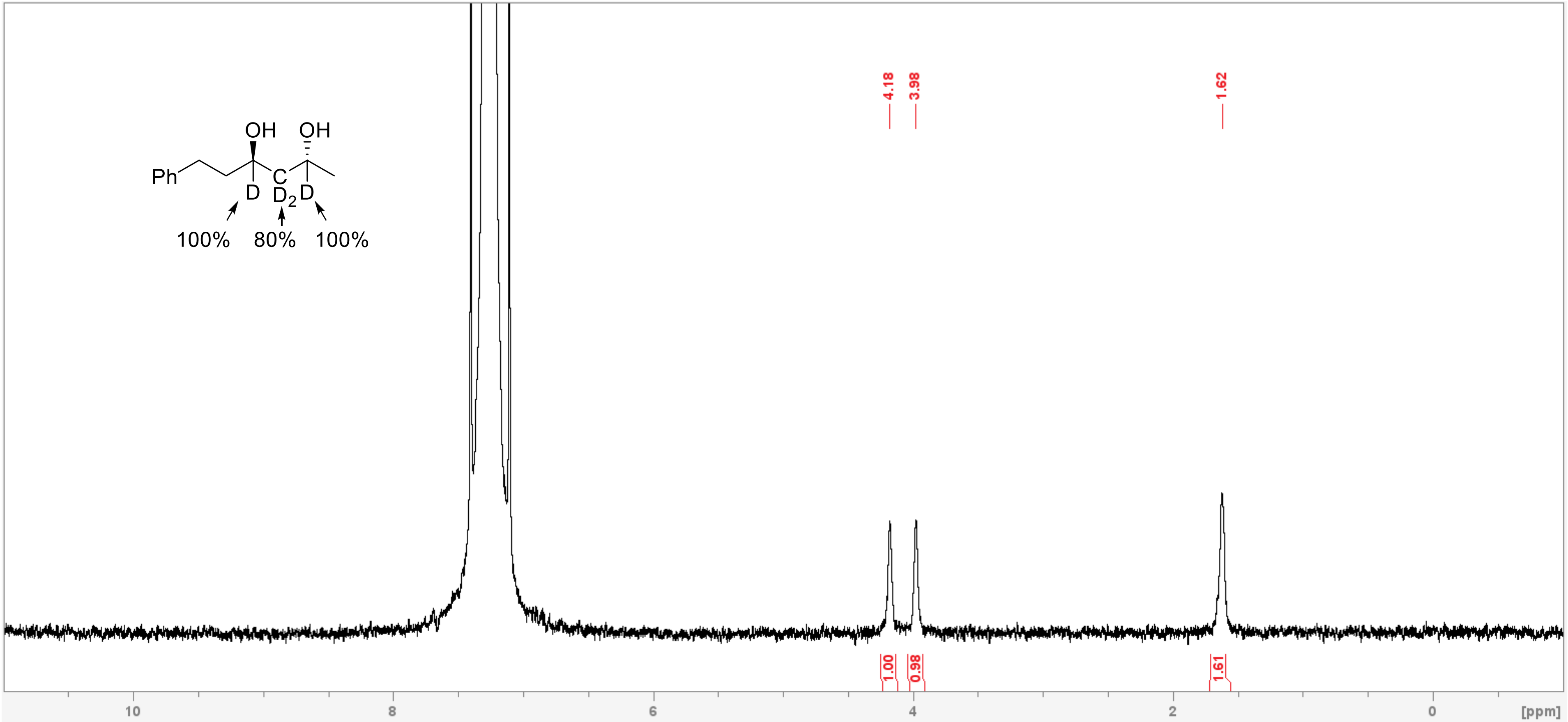

***syn*-butane-2,3-diol (4a-rac)**

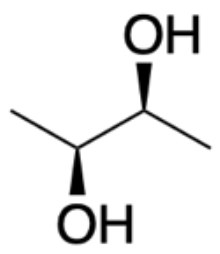

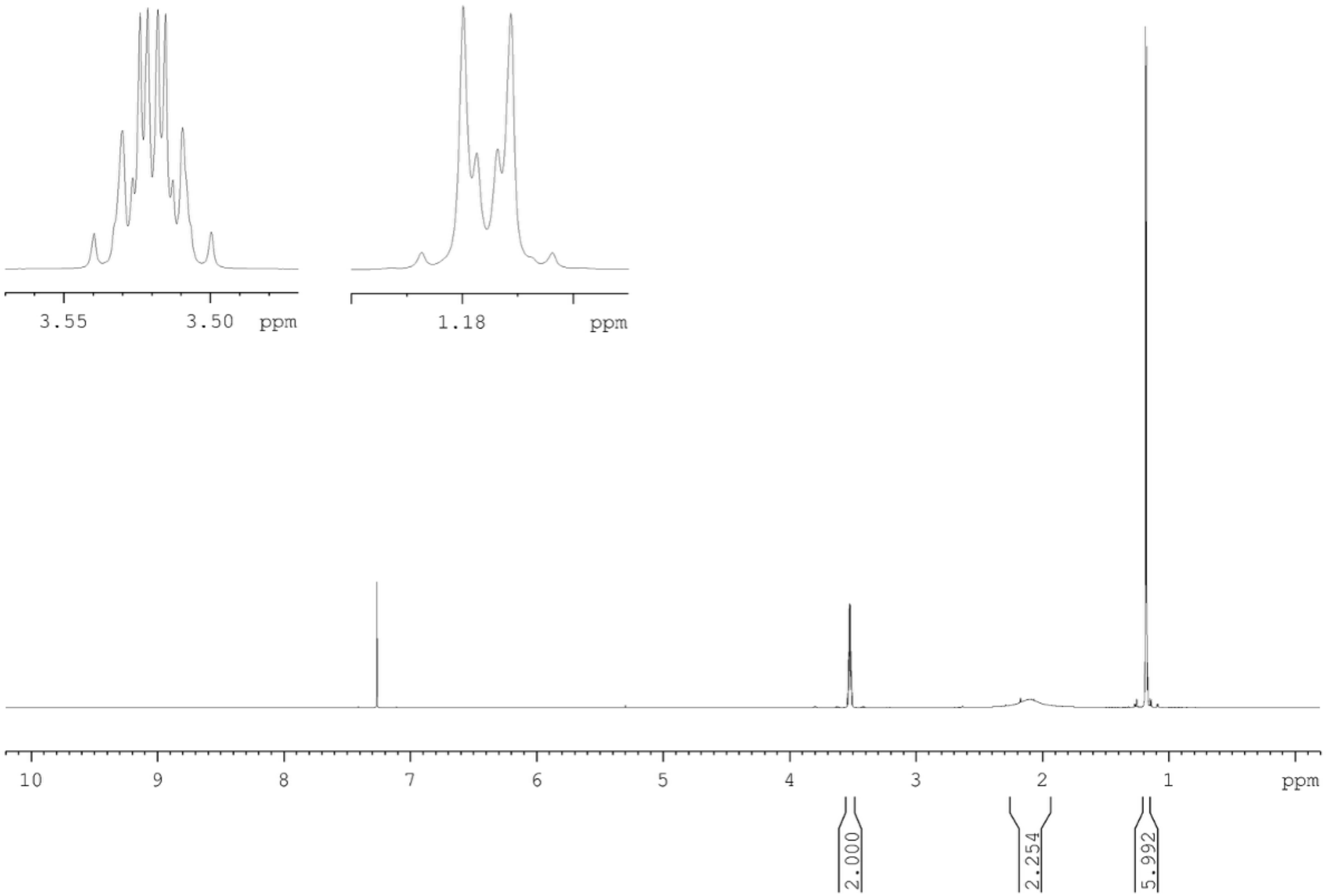

syn-butane-2,3-diol (4a-rac)

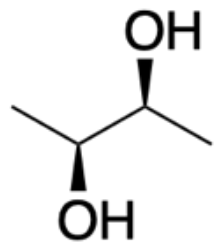

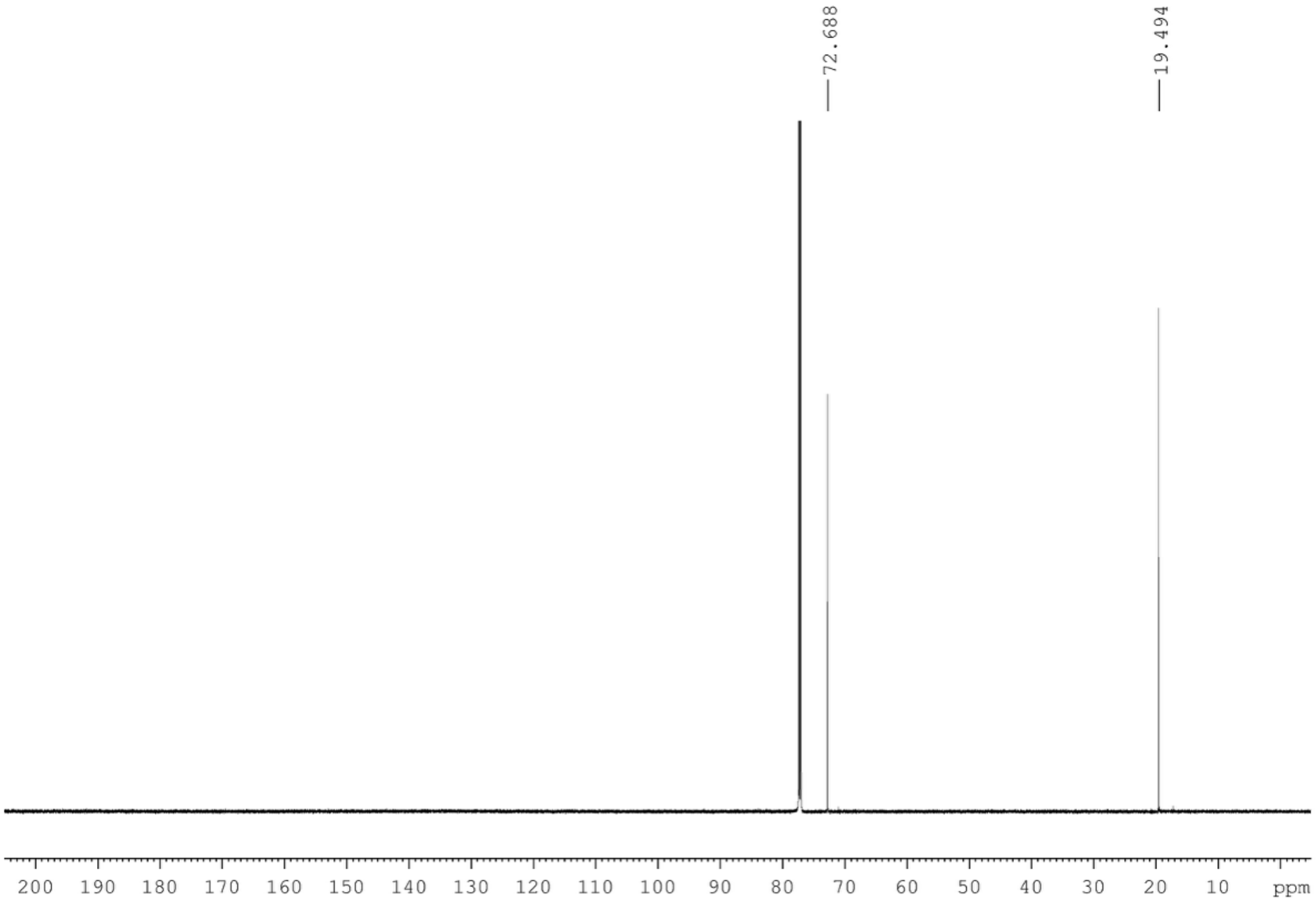

***syn*-octane-4,5-diol (4c-rac)**

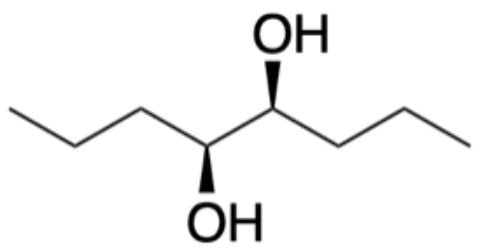

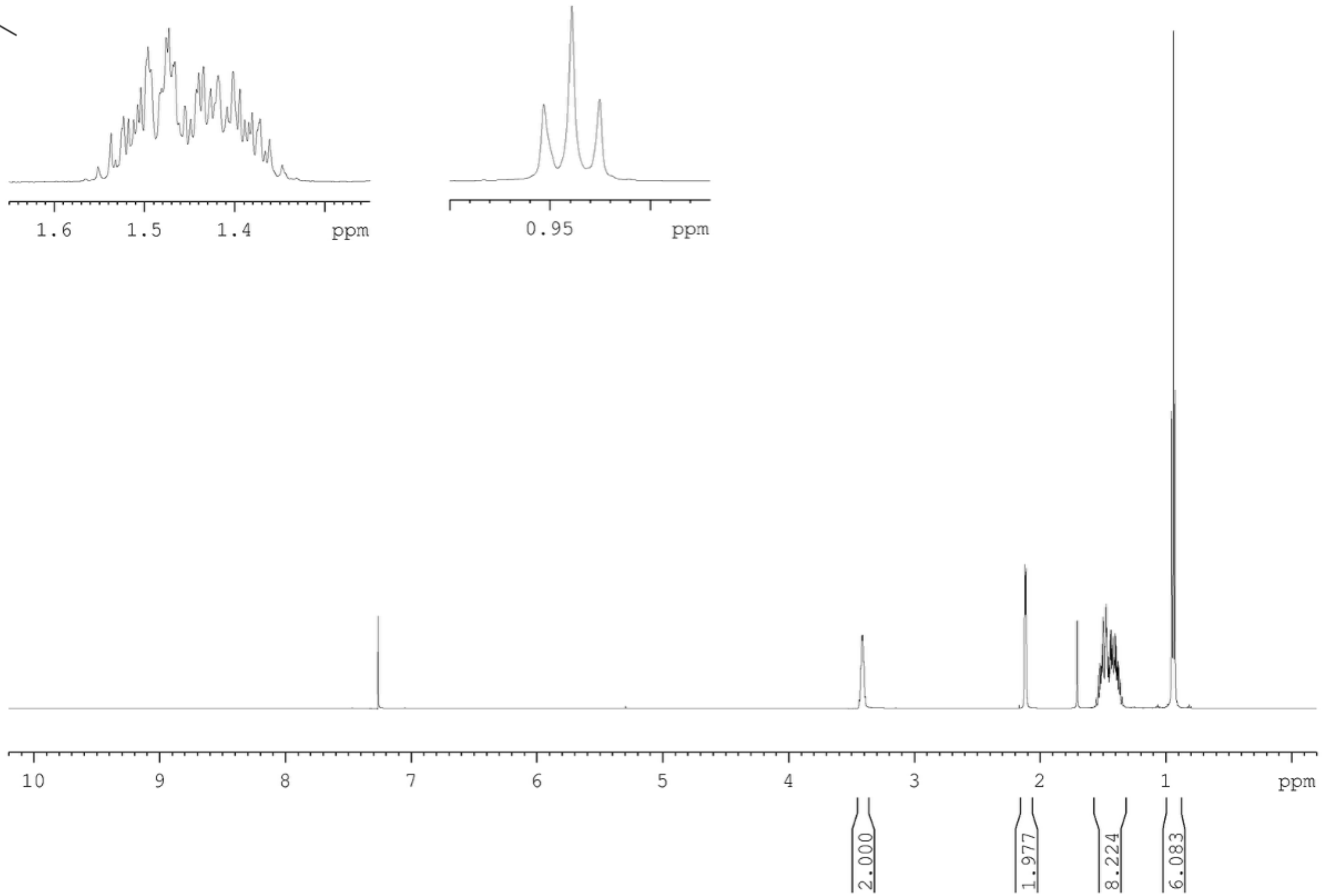

syn-octane-4,5-diol (4c-rac)

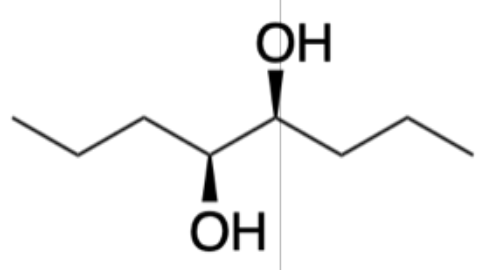

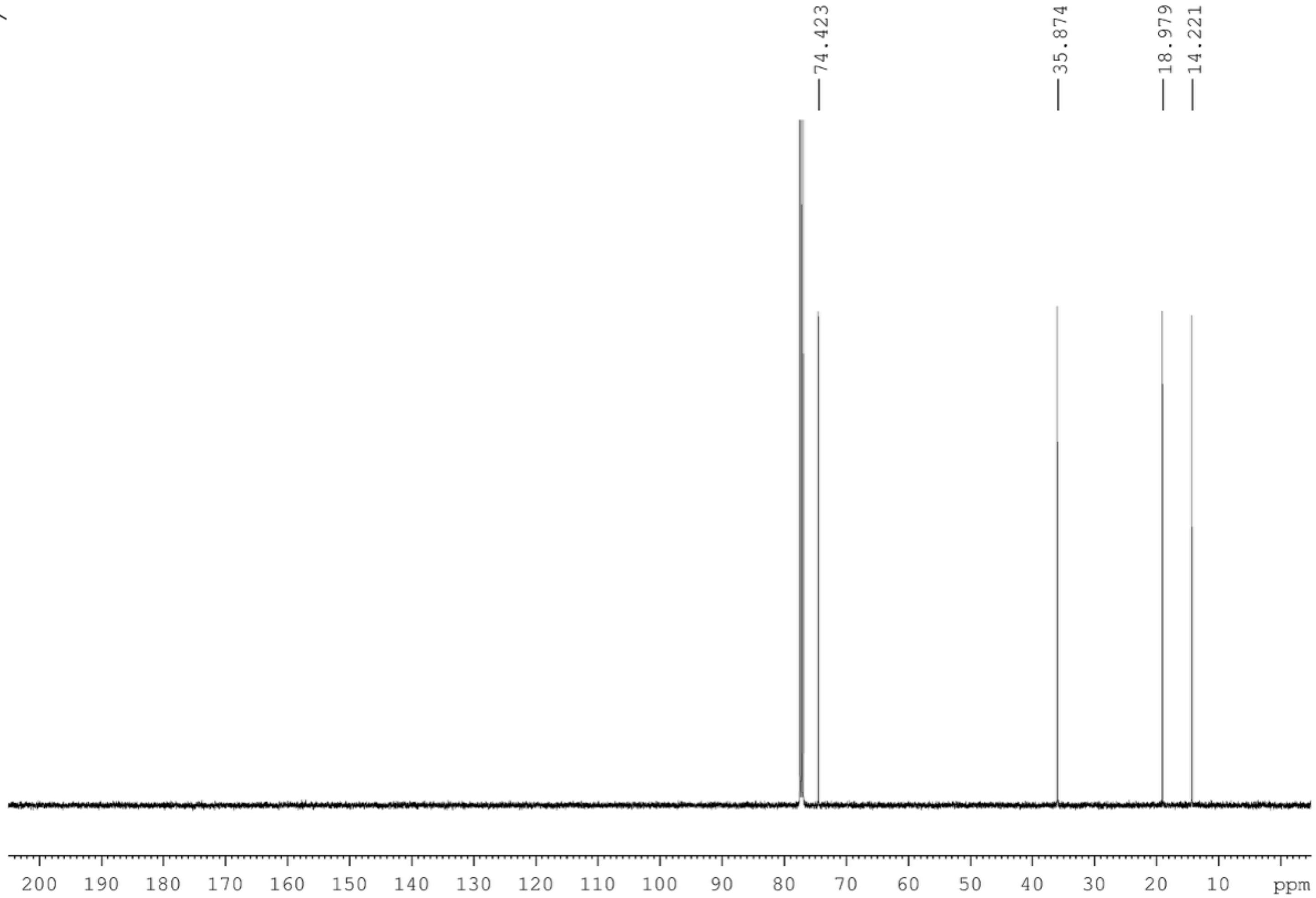

***syn*-decane-5,6-diol (4d-rac)**

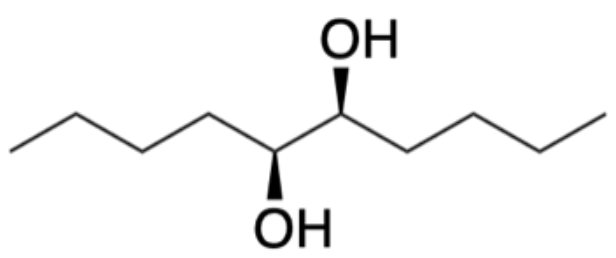

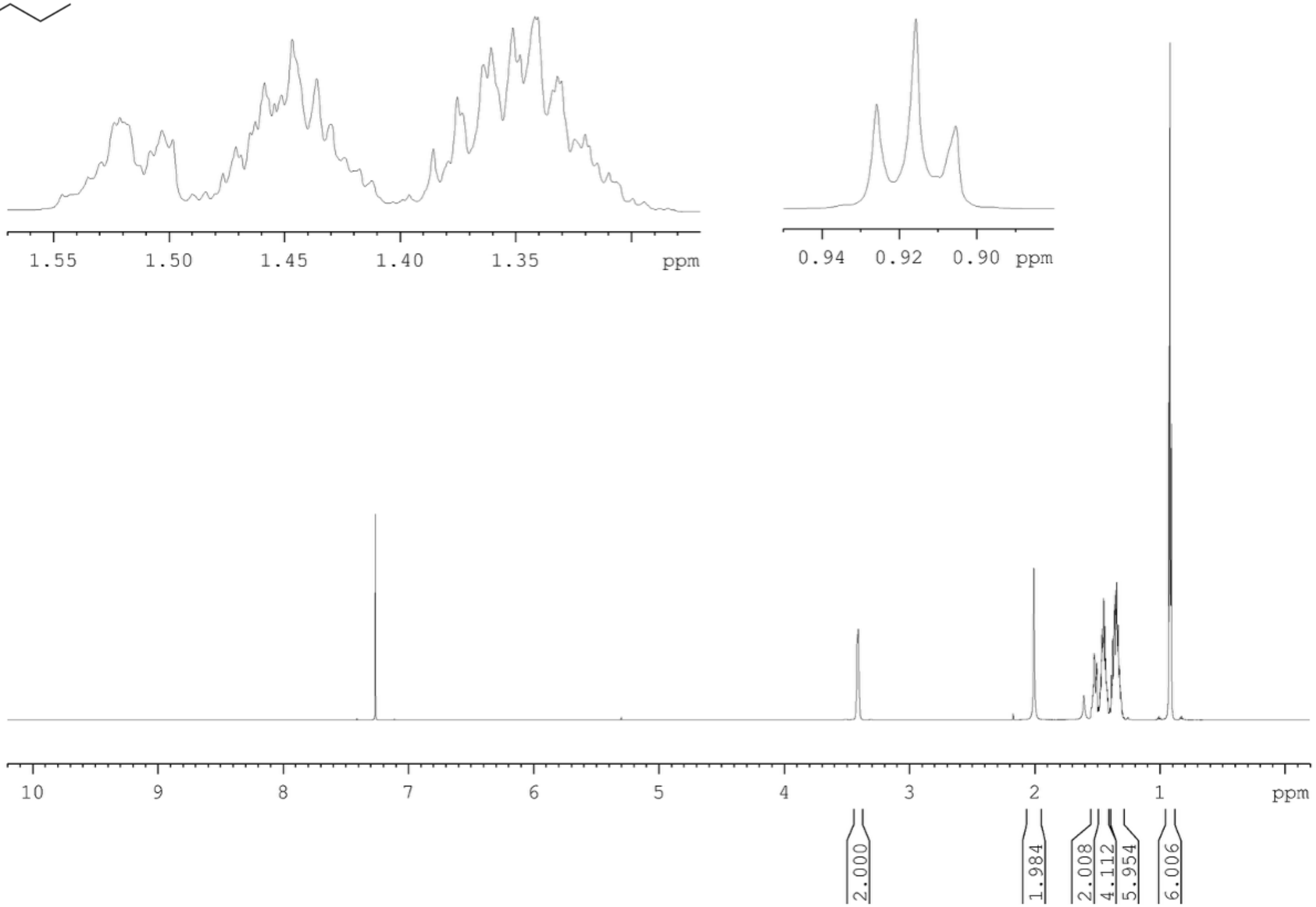

syn-decane-5,6-diol (4d-rac)

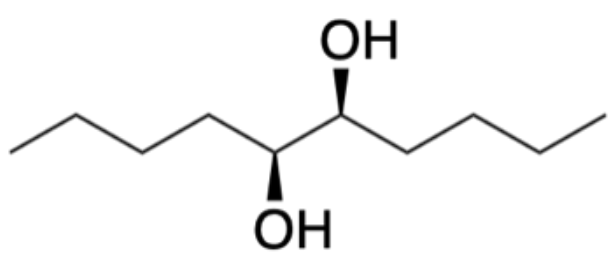

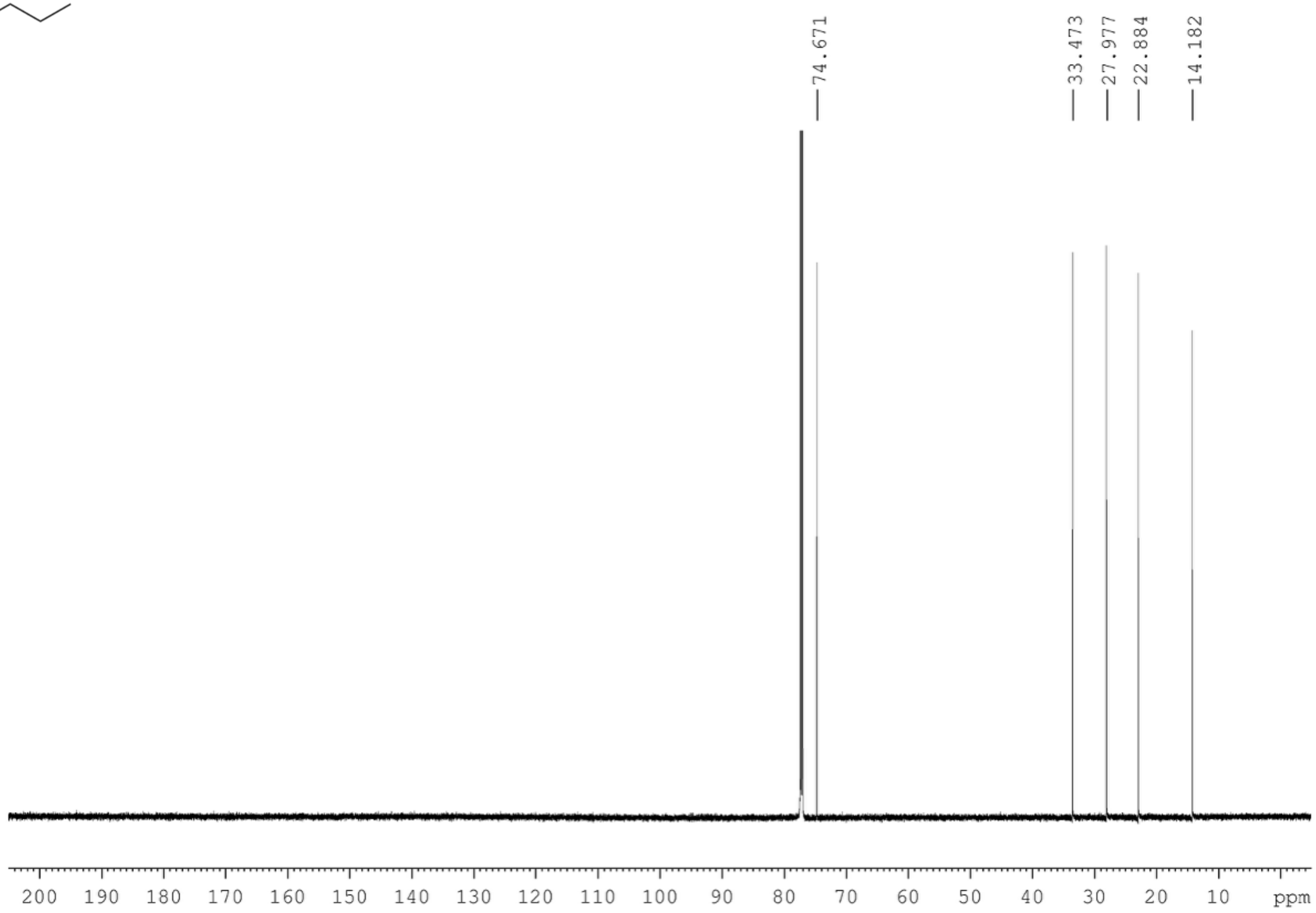

Di-*tert*-butyl *syn*-4,5-dihydroxyoctanedioate (4e-rac)

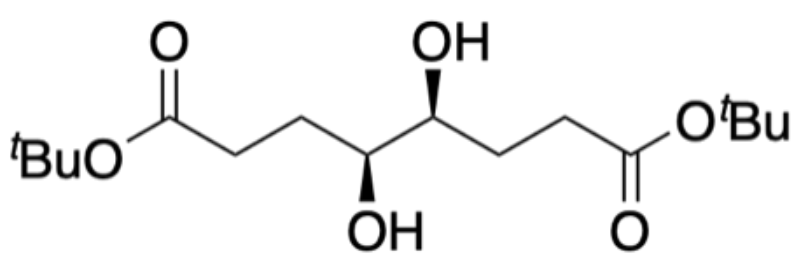

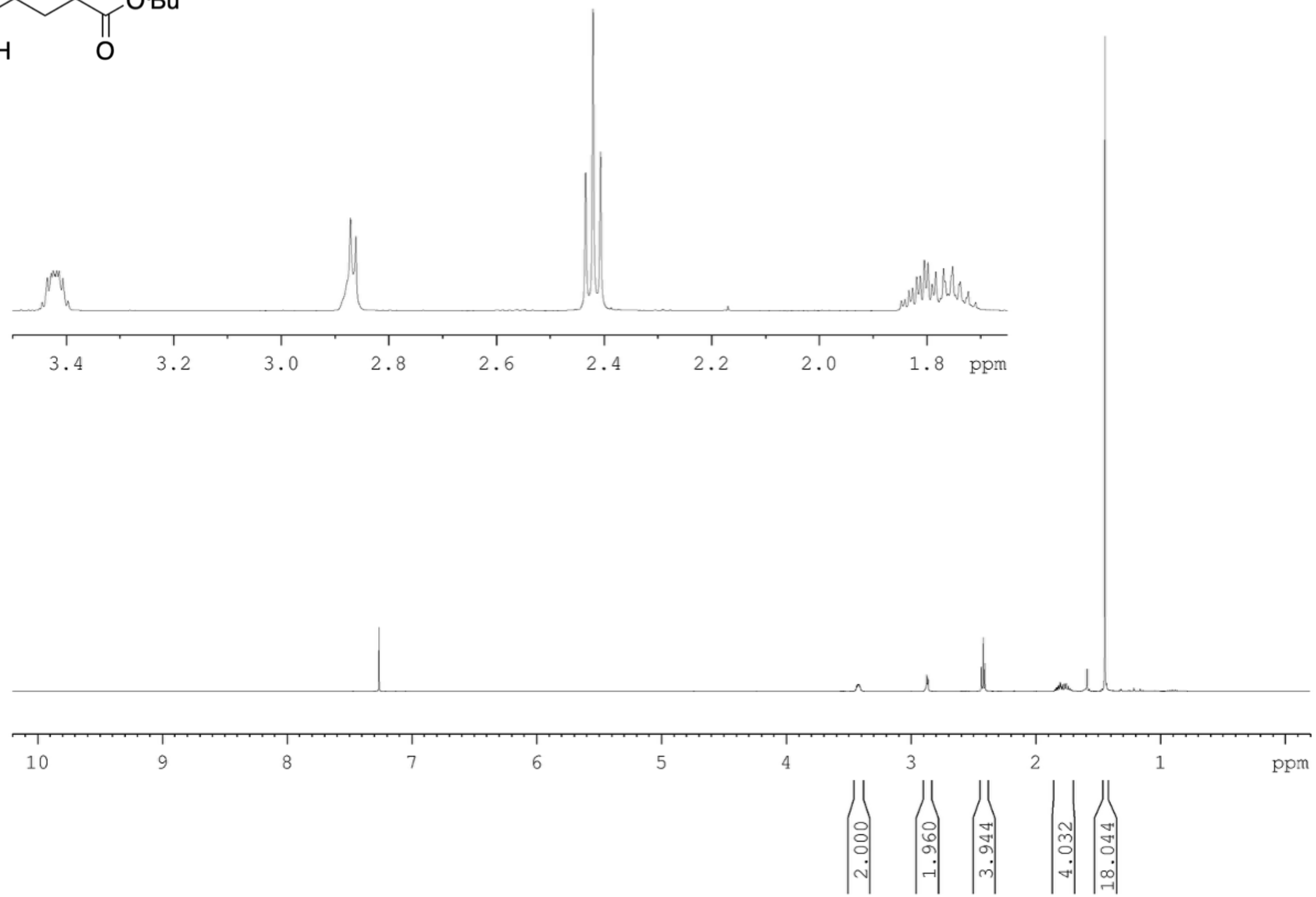

Di-*tert*-butyl *syn*-4,5-dihydroxyoctanedioate (4e-rac)

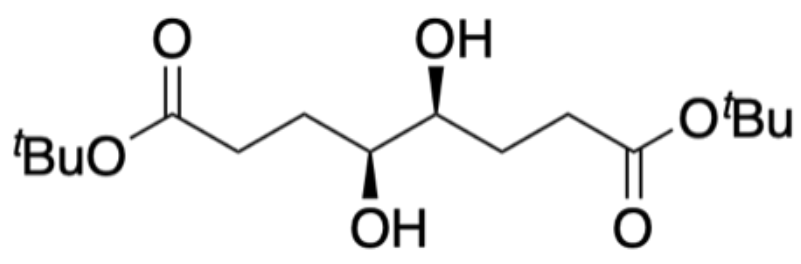

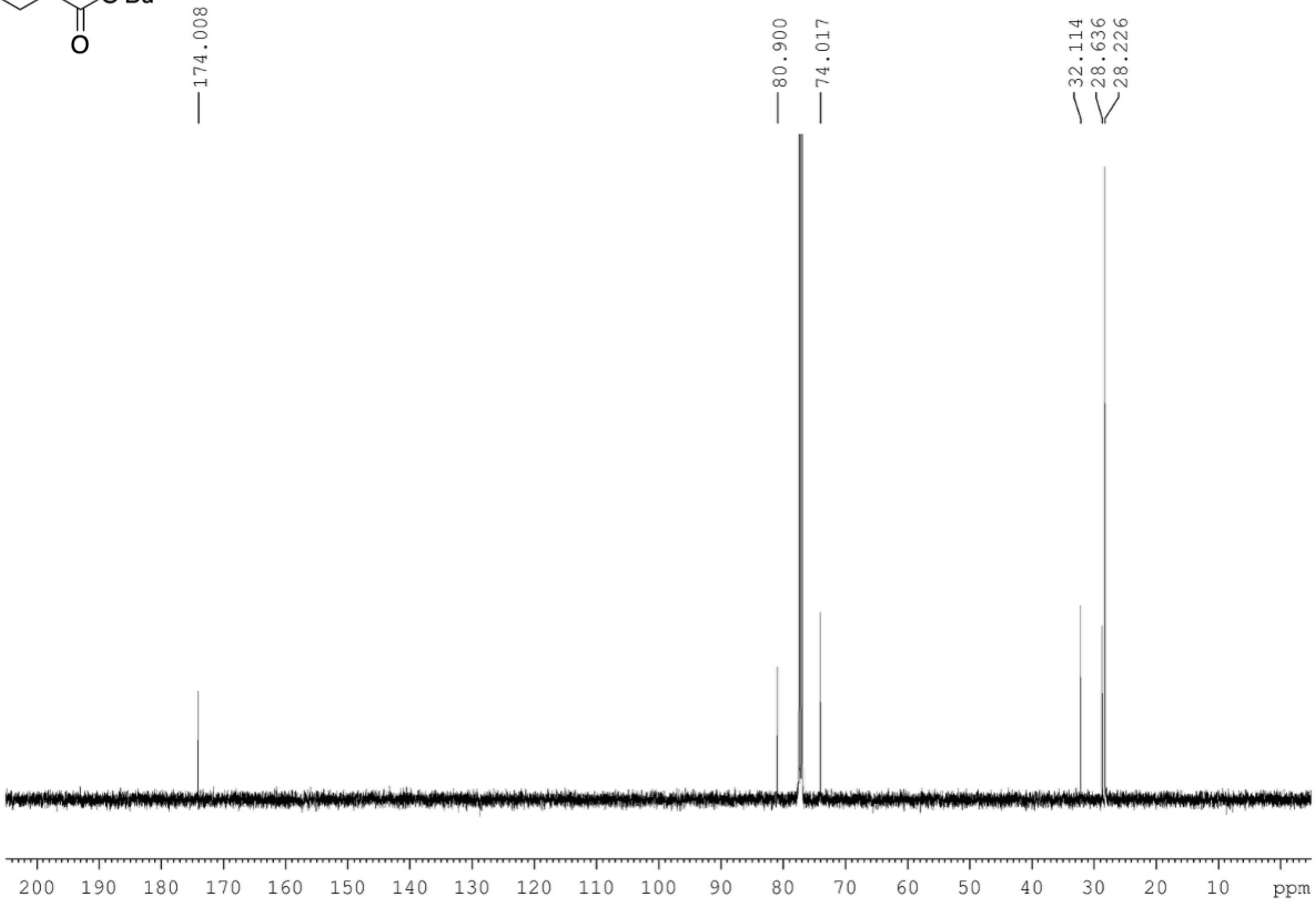

syn-1,4-diphenylbutane-2,3-diol (4f-rac)

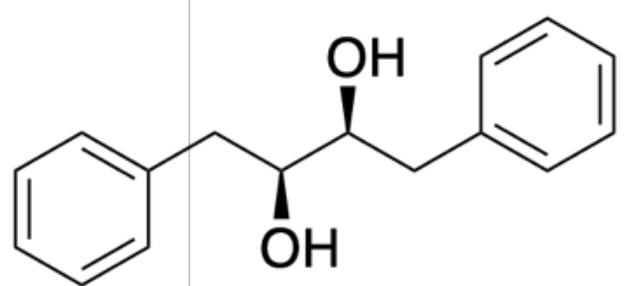

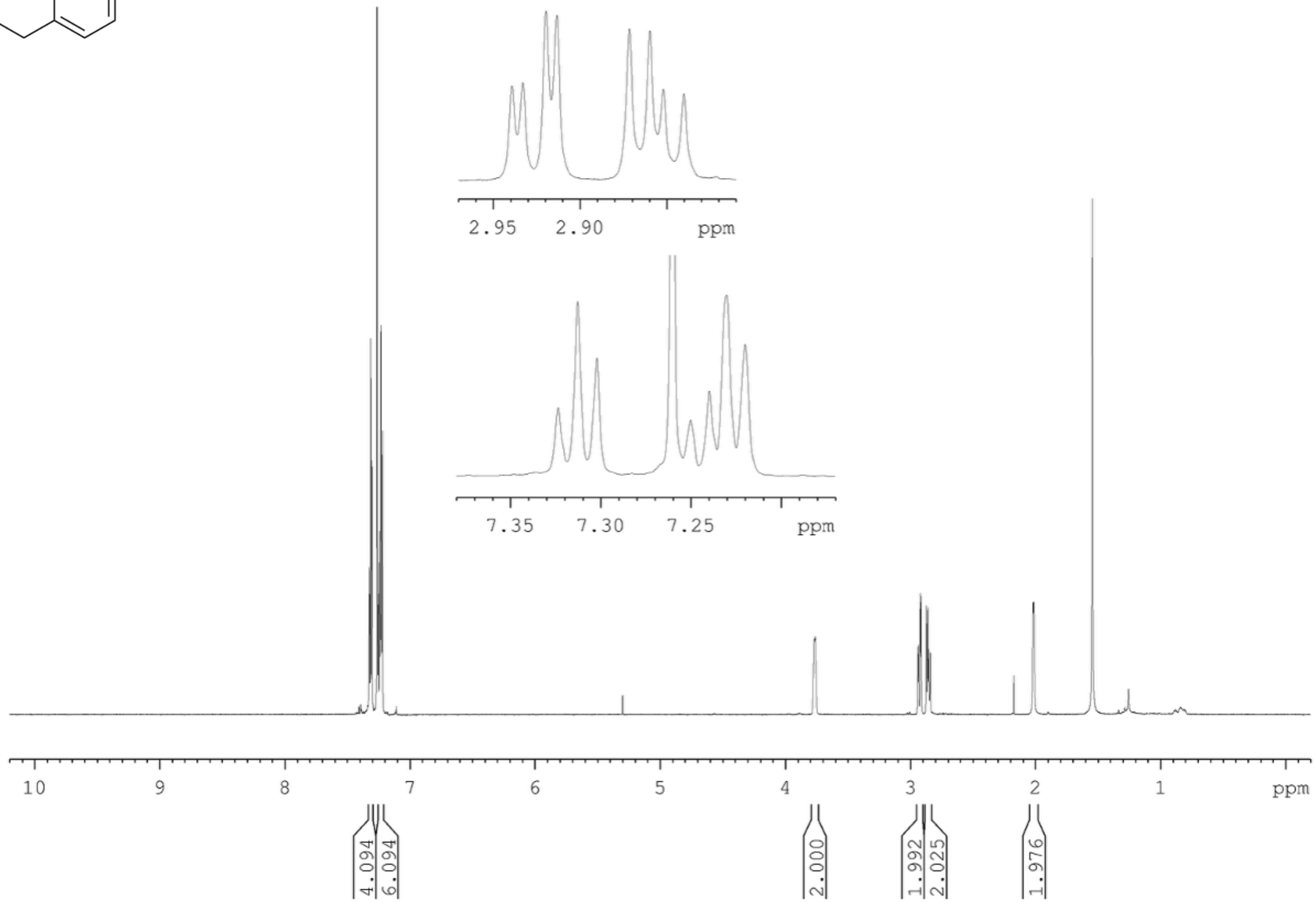

***syn*-1,4-diphenylbutane-2,3-diol (4f-rac)**

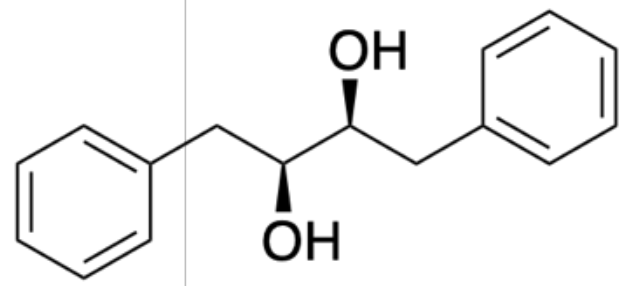

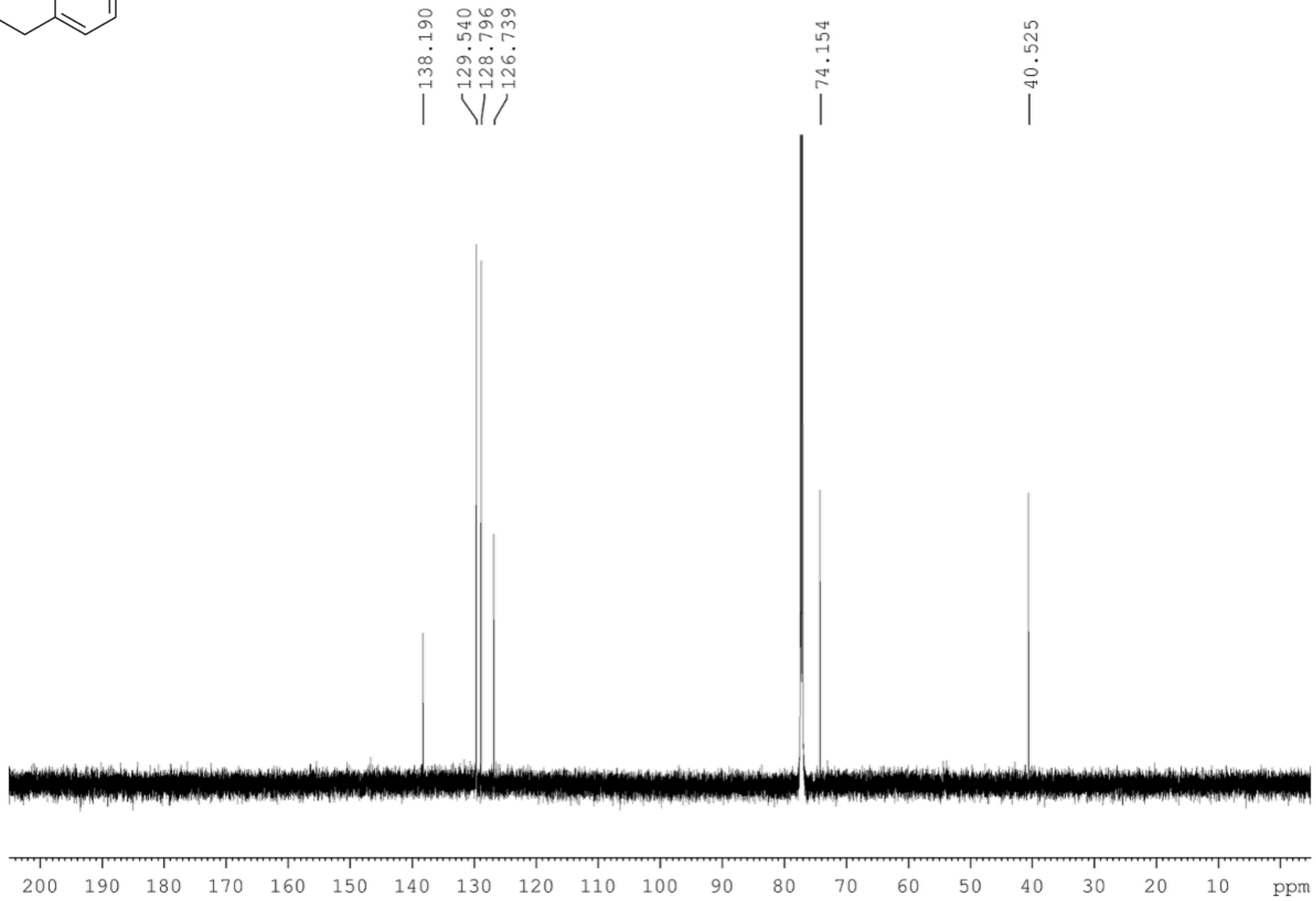

1,8-diphenyloct-4-ene (4g-I$_1$)

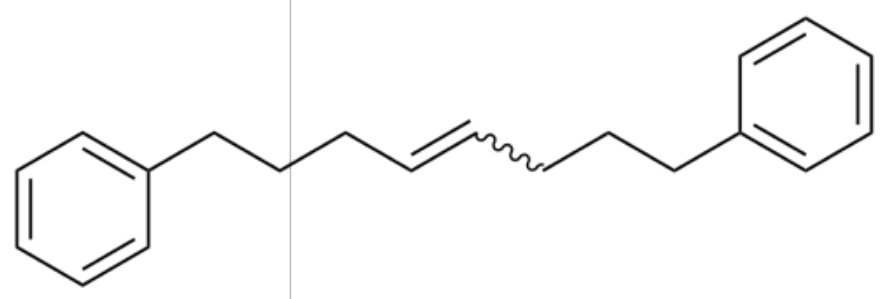

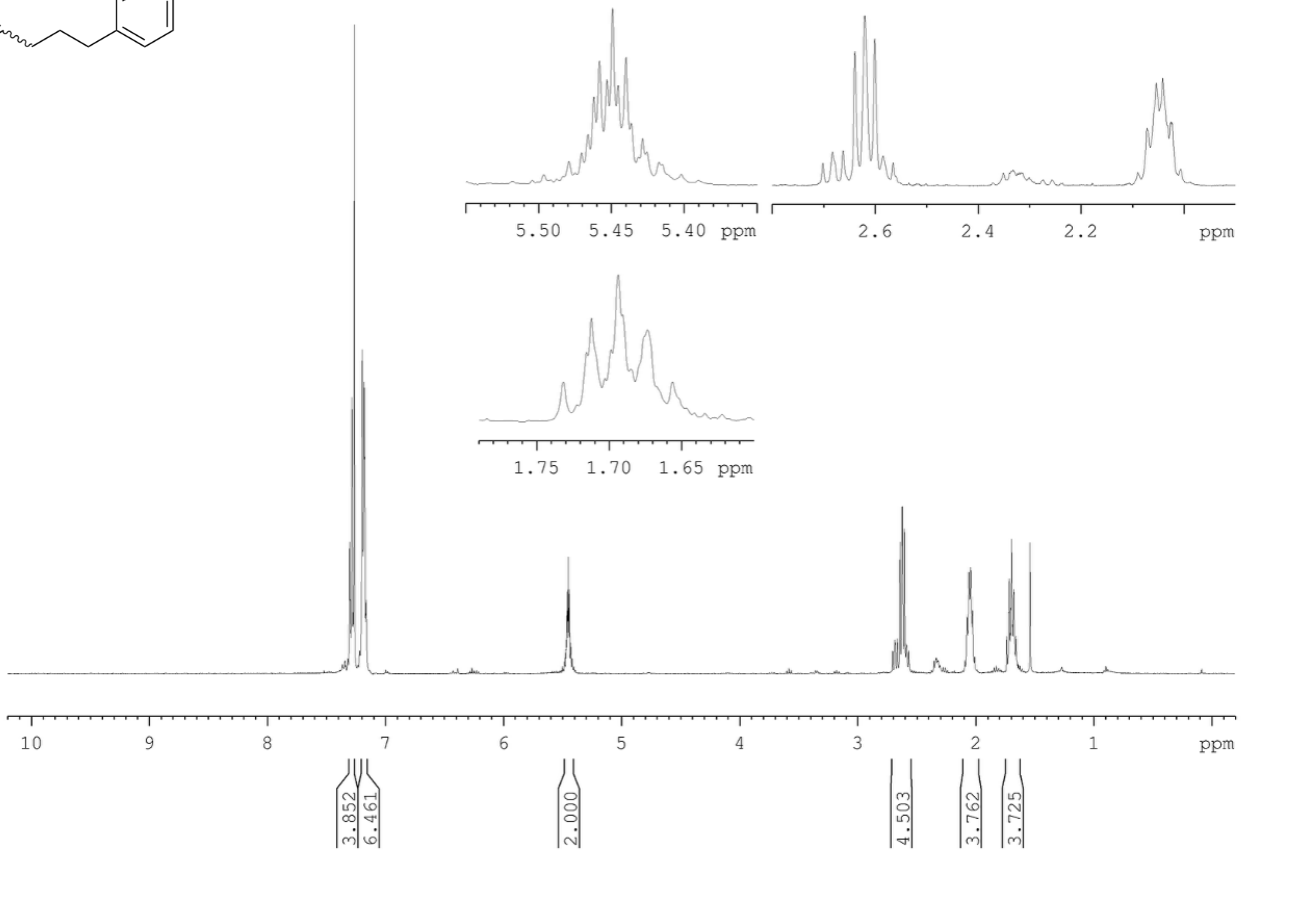

1,8-diphenyloct-4-ene (4g-I$_1$)

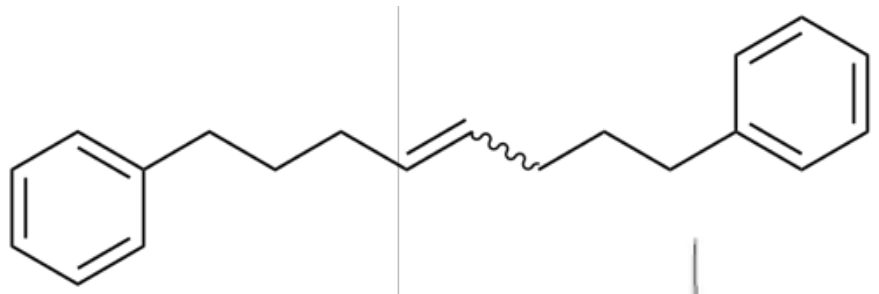

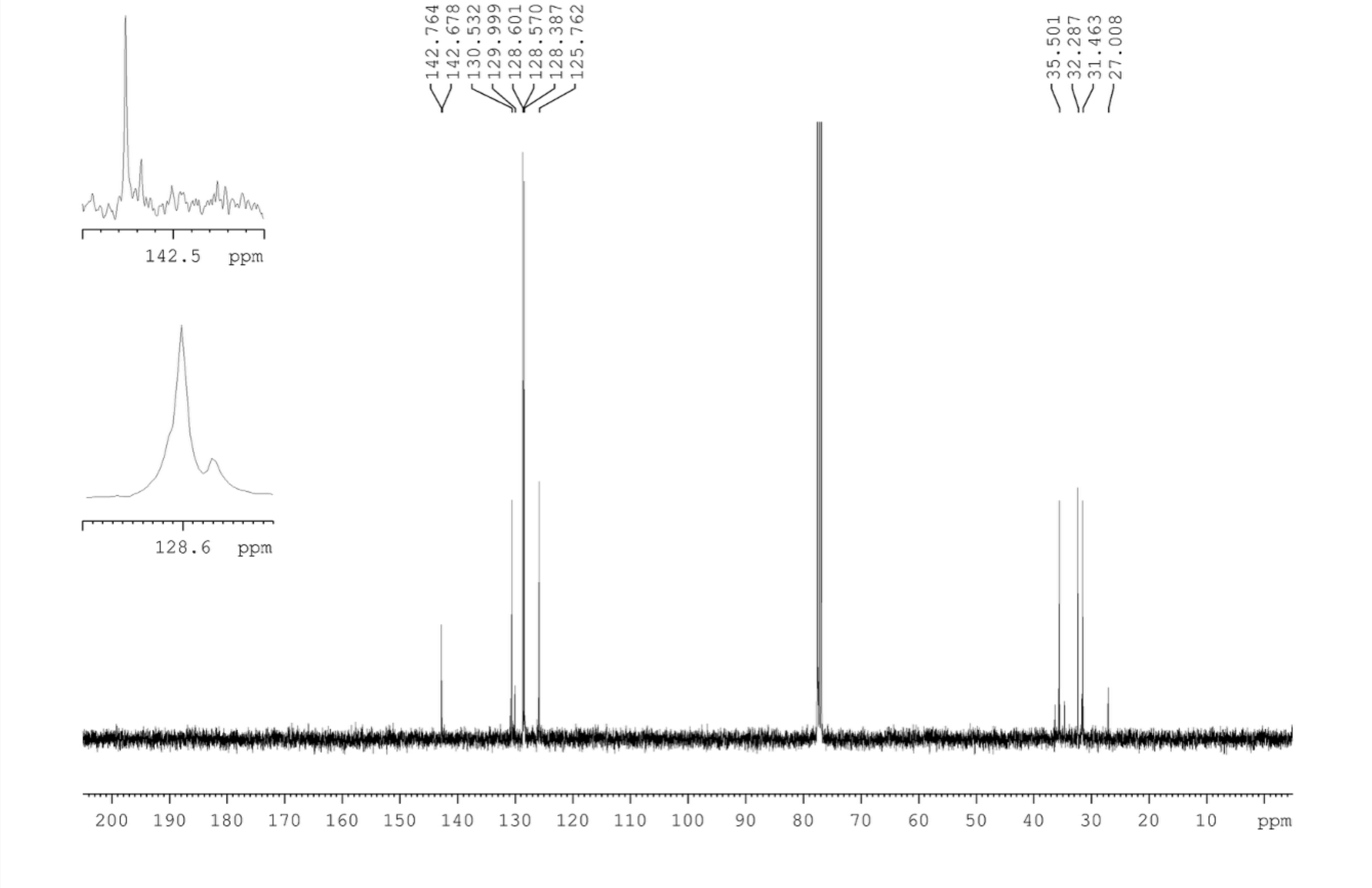

***syn*-1,8-diphenyloctane-4,5-diol (4g-rac)**

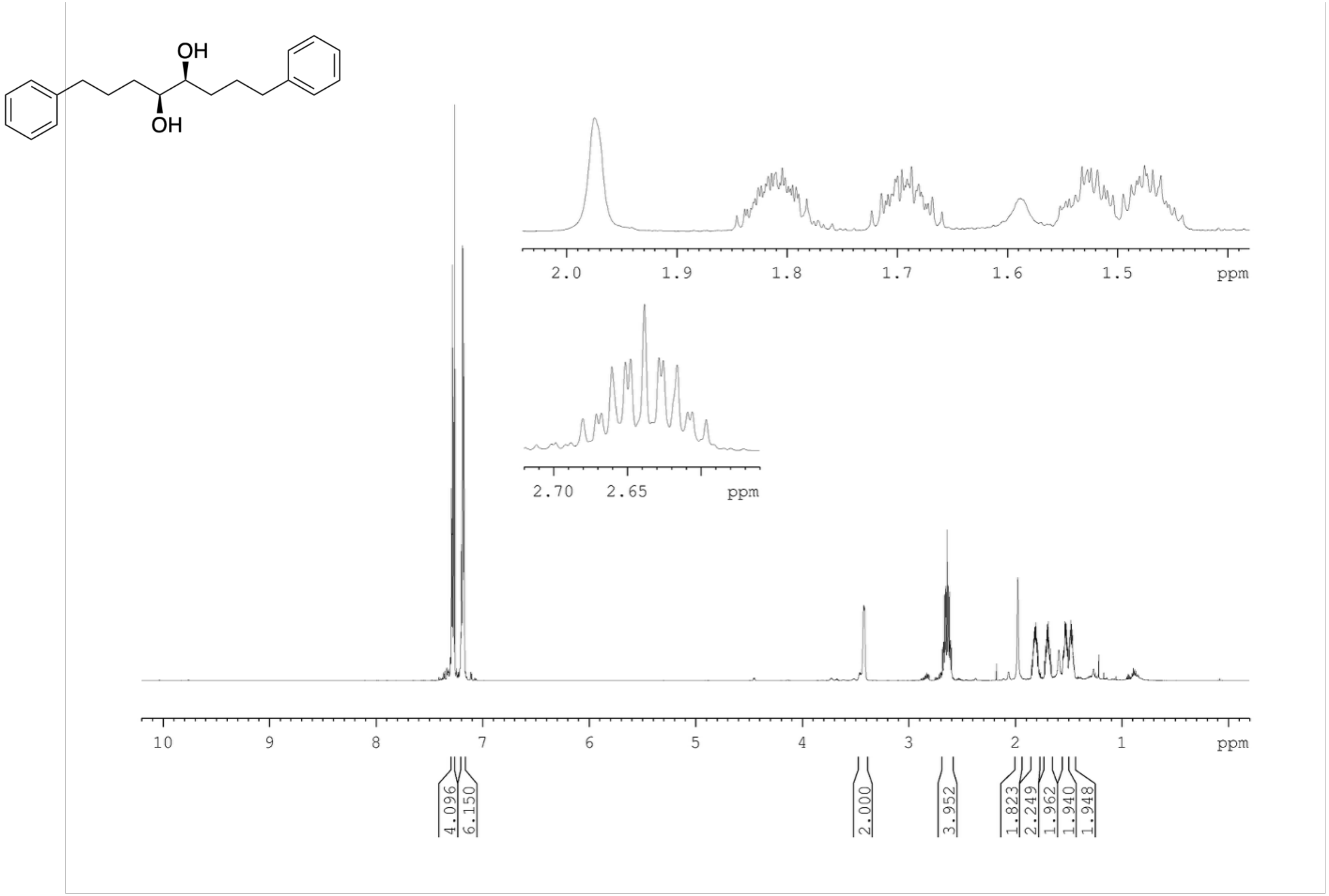

***syn*-1,8-diphenyloctane-4,5-diol (4g-rac)**

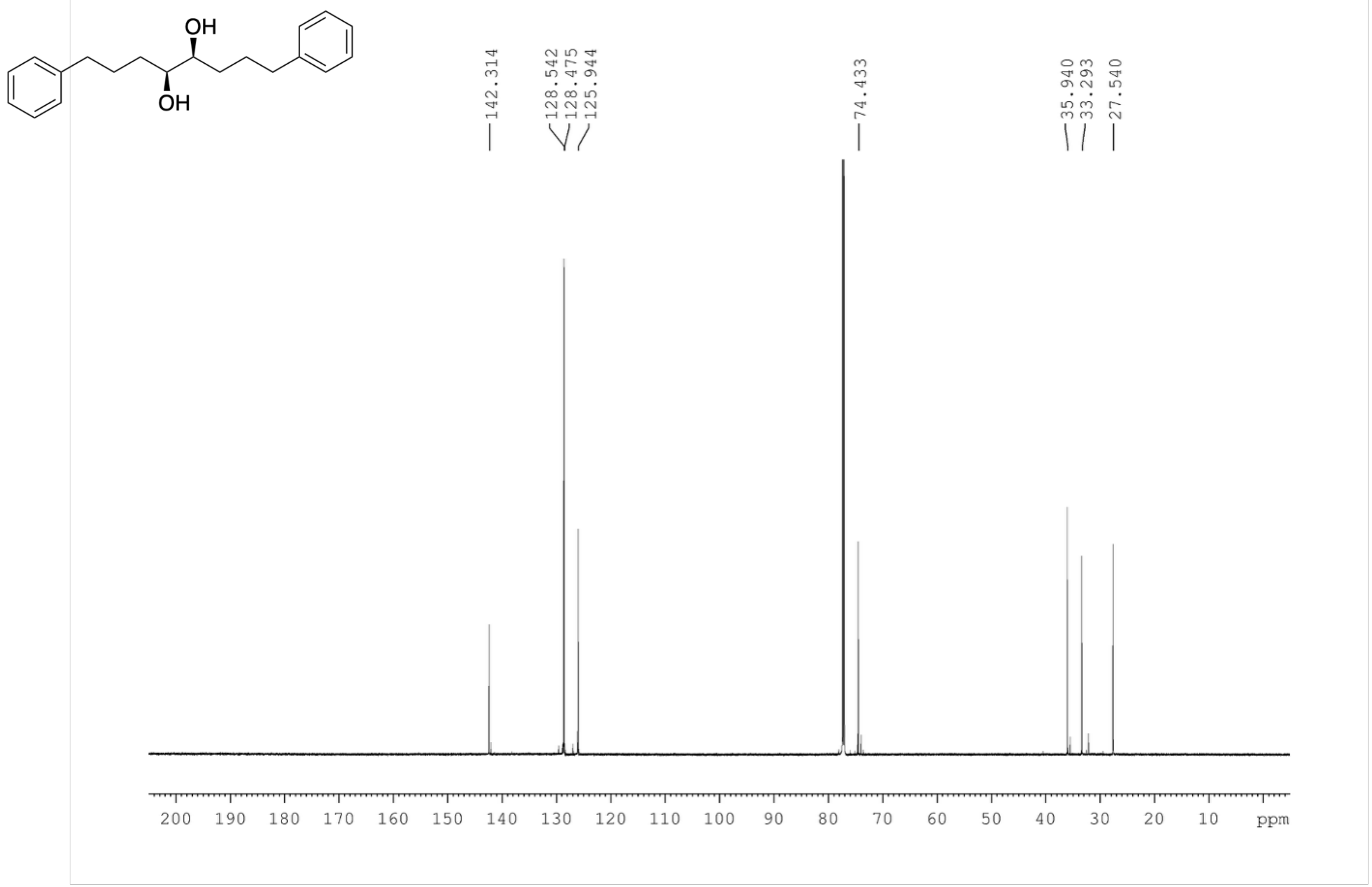

(*Z*)- + (*E*)-Pent-3-en-1-ylbenzene (*int$_1$*-4h)

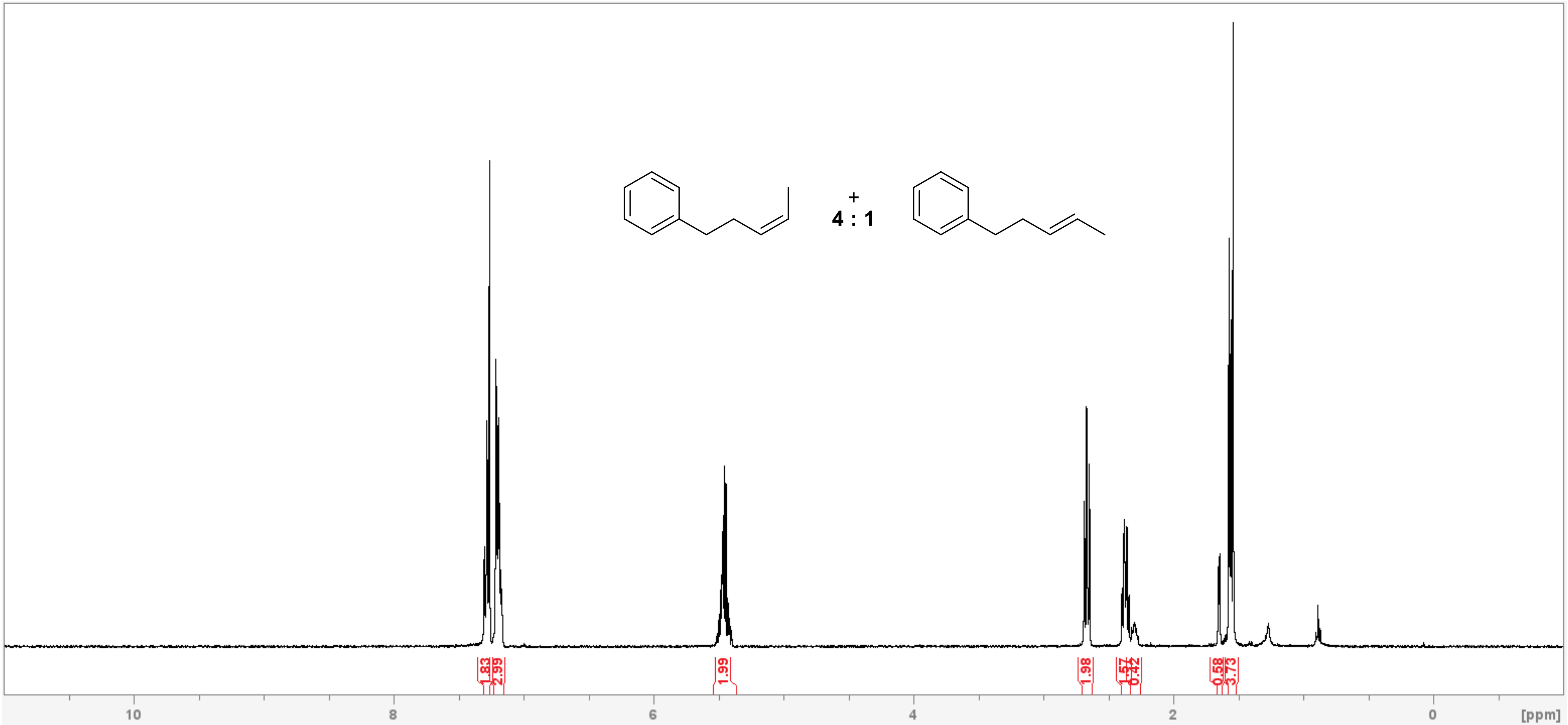

(*anti*)-5-Phenylpentane-2,3-diol (*rac*-*anti*-4h)

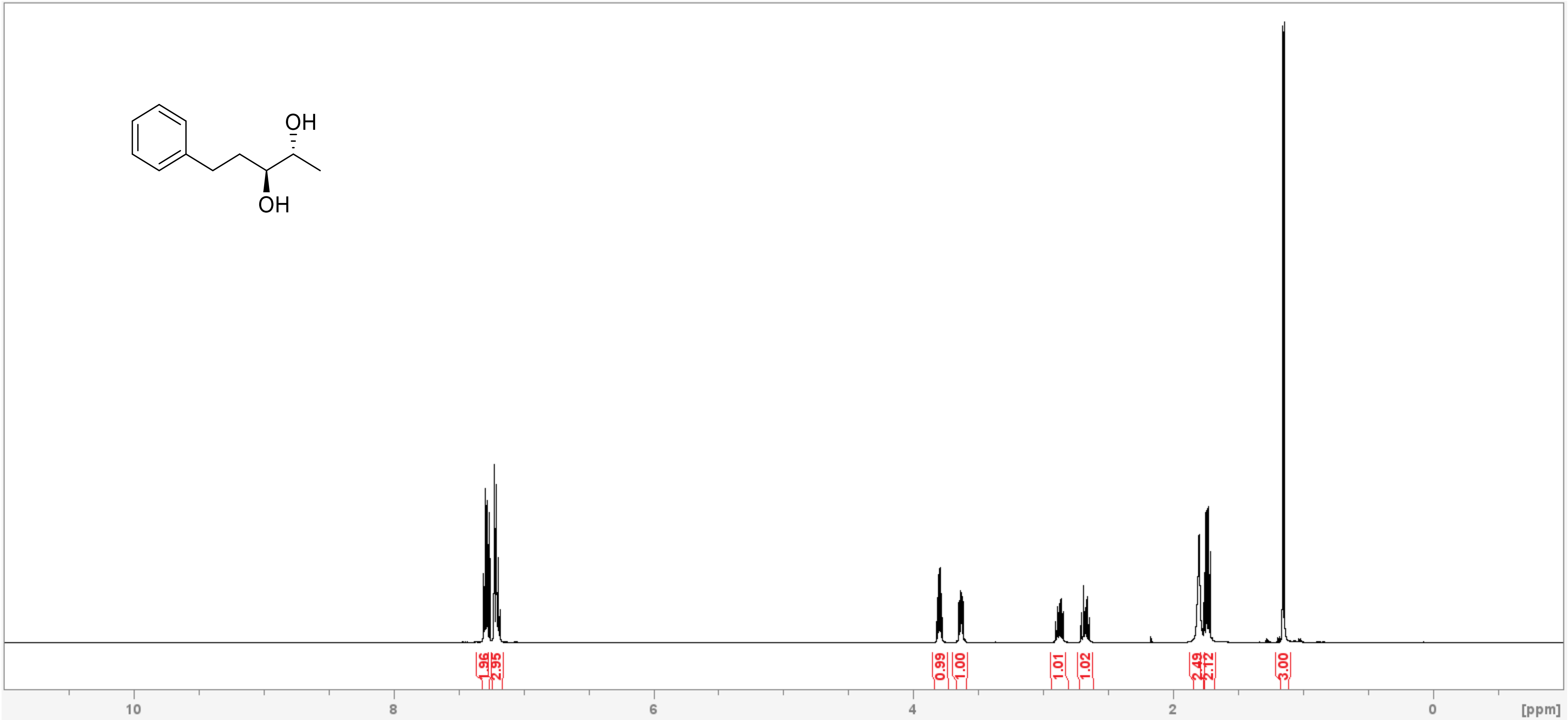

(*anti*)-5-Phenylpentane-2,3-diol (*rac*-*anti*-4h)

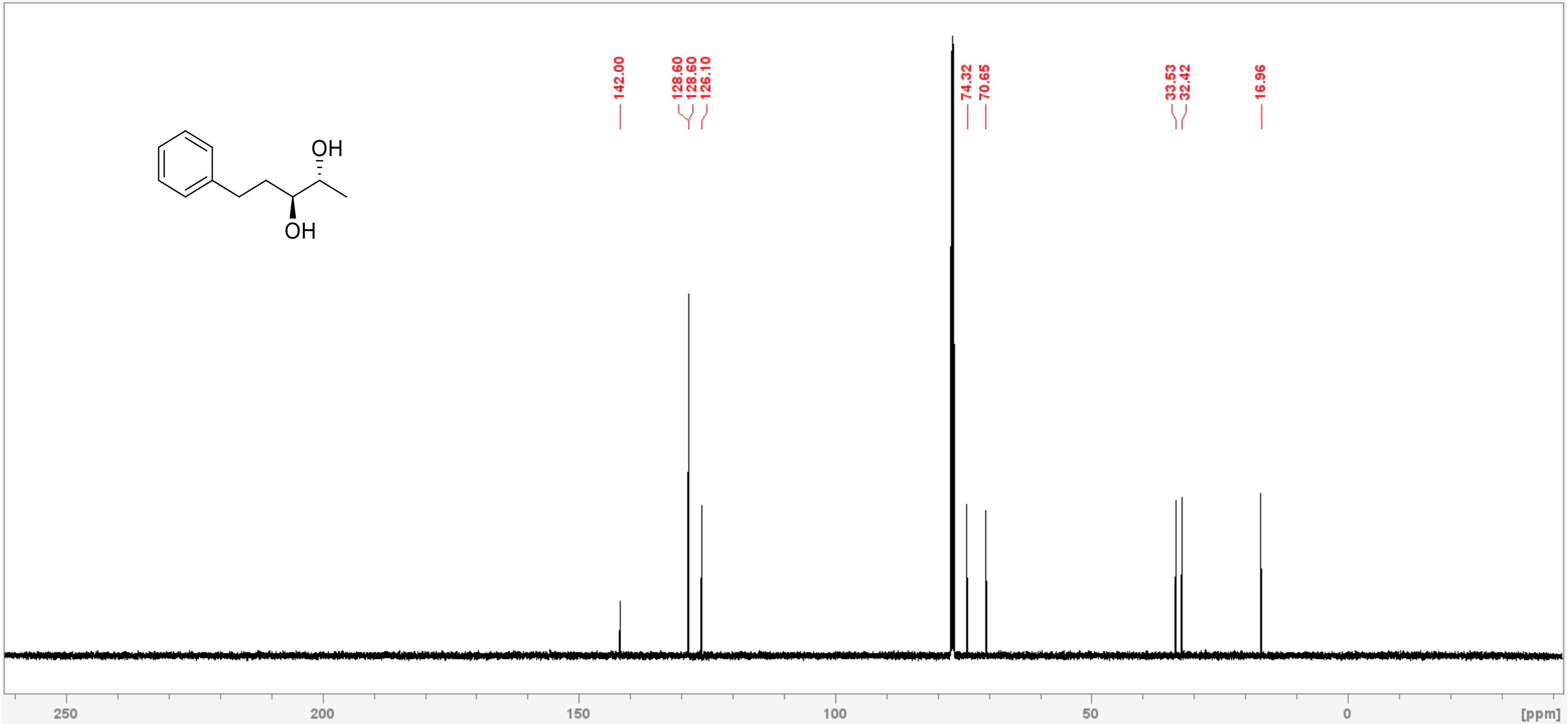

(*syn*)-5-Phenylpentane-2,3-diol (*rac-syn*-4h)

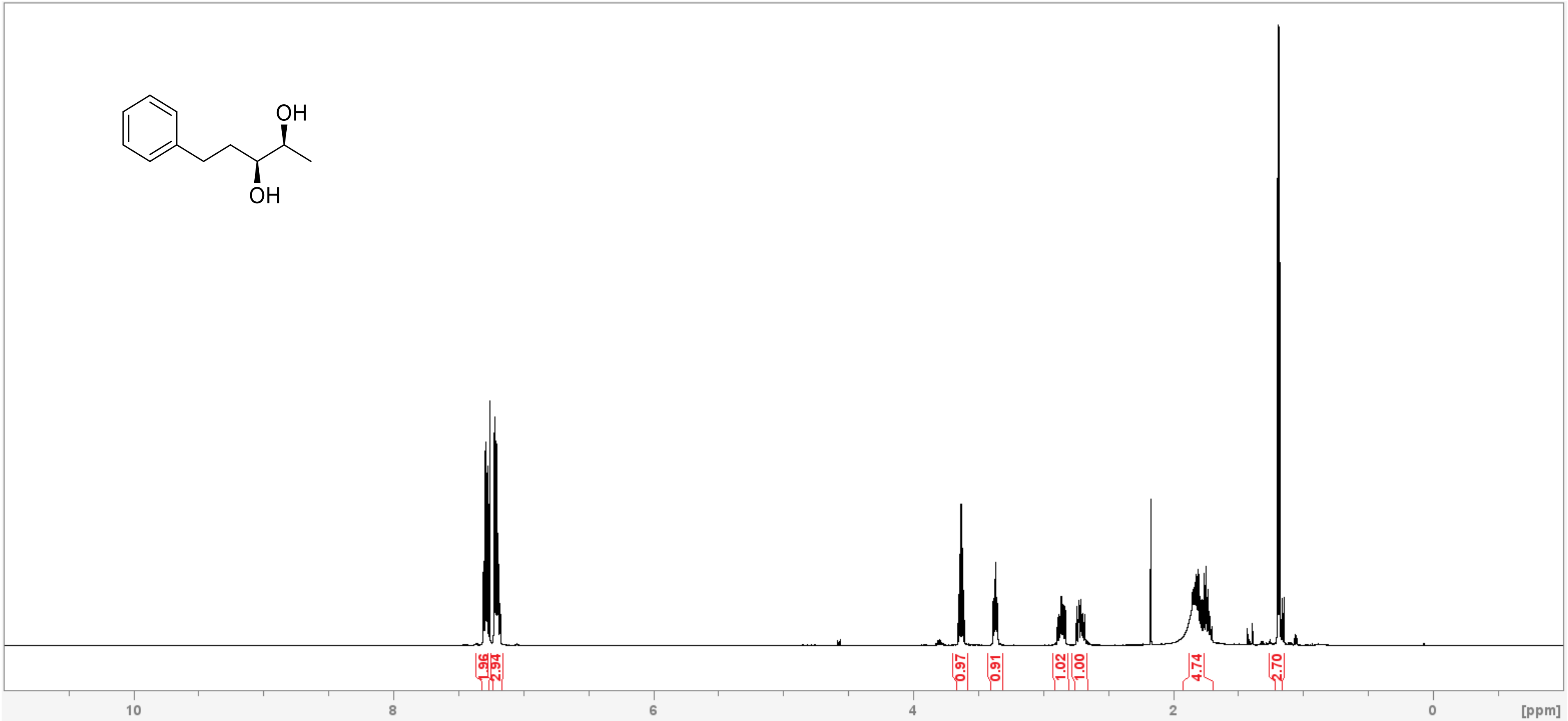

(*syn*)-5-Phenylpentane-2,3-diol (*rac*-*syn*-4h)

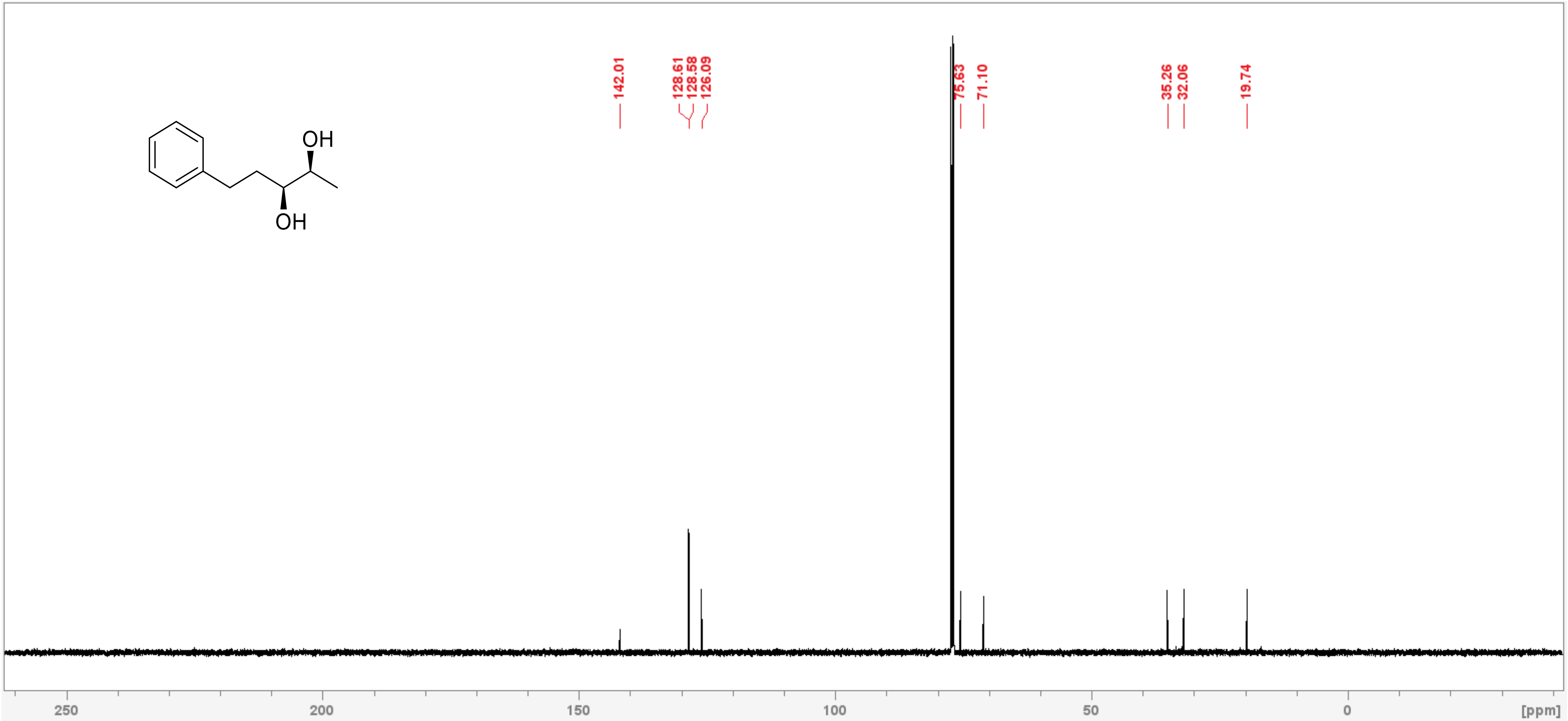

(Z)- + (E)-But-2-en-1-ylbenzene (*int$_1$*-4i)

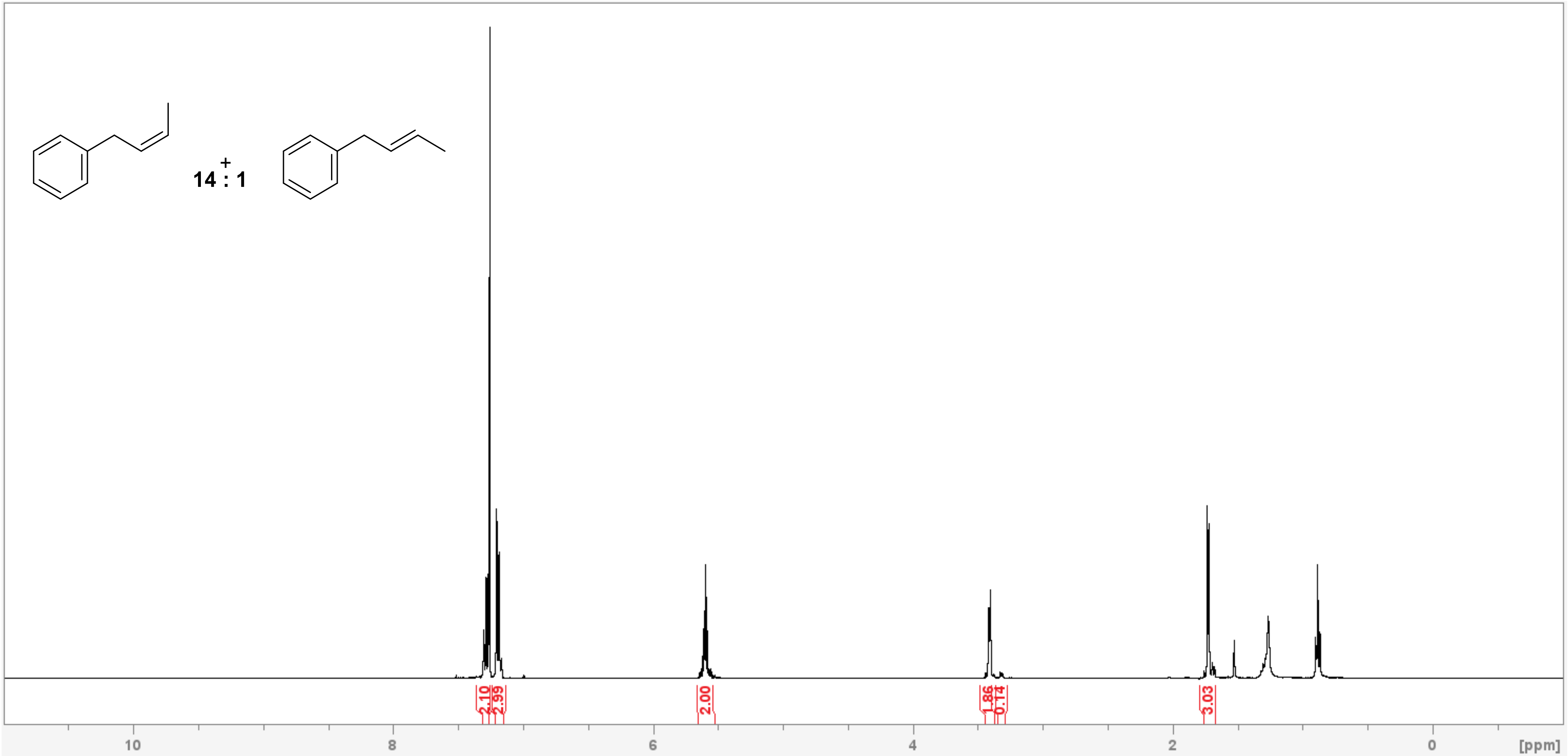

(*anti*)-1-Phenylbutane-2,3-diol (*rac*-*anti*-4i)

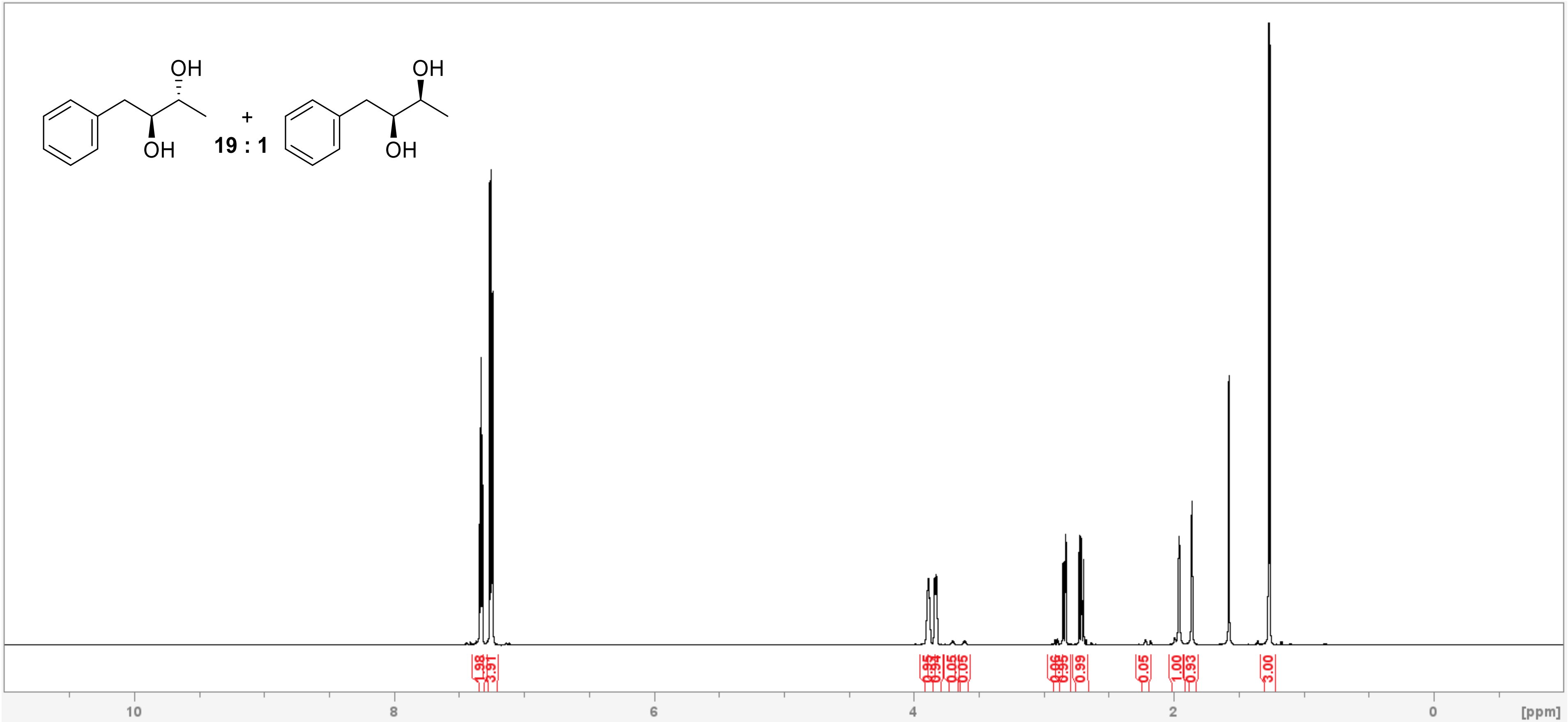

(*anti*)-1-Phenylbutane-2,3-diol (*rac*-*anti*-4i)

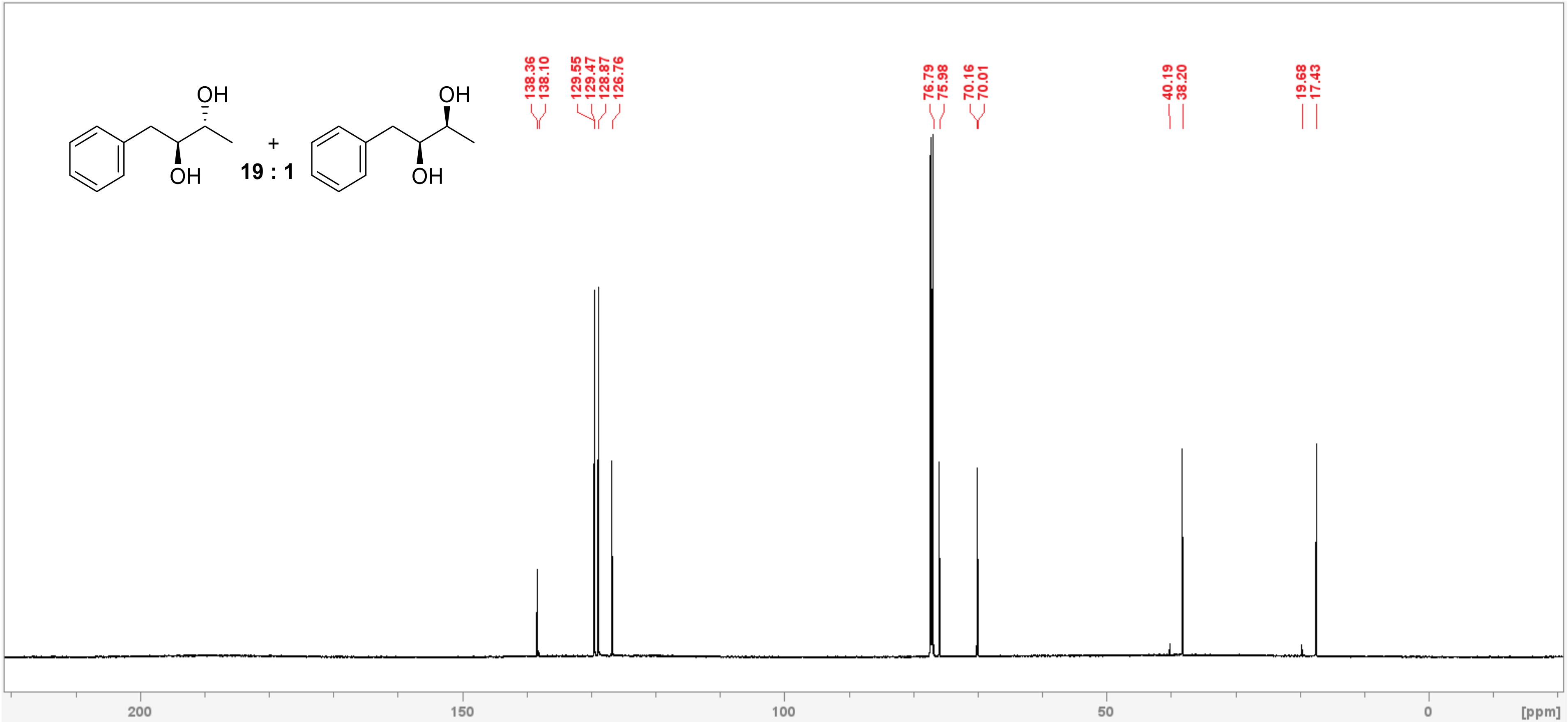

(*Z*)- + (*E*)-Hept-3-en-1-ylbenzene (4:1) (*int*$_1$-4j)

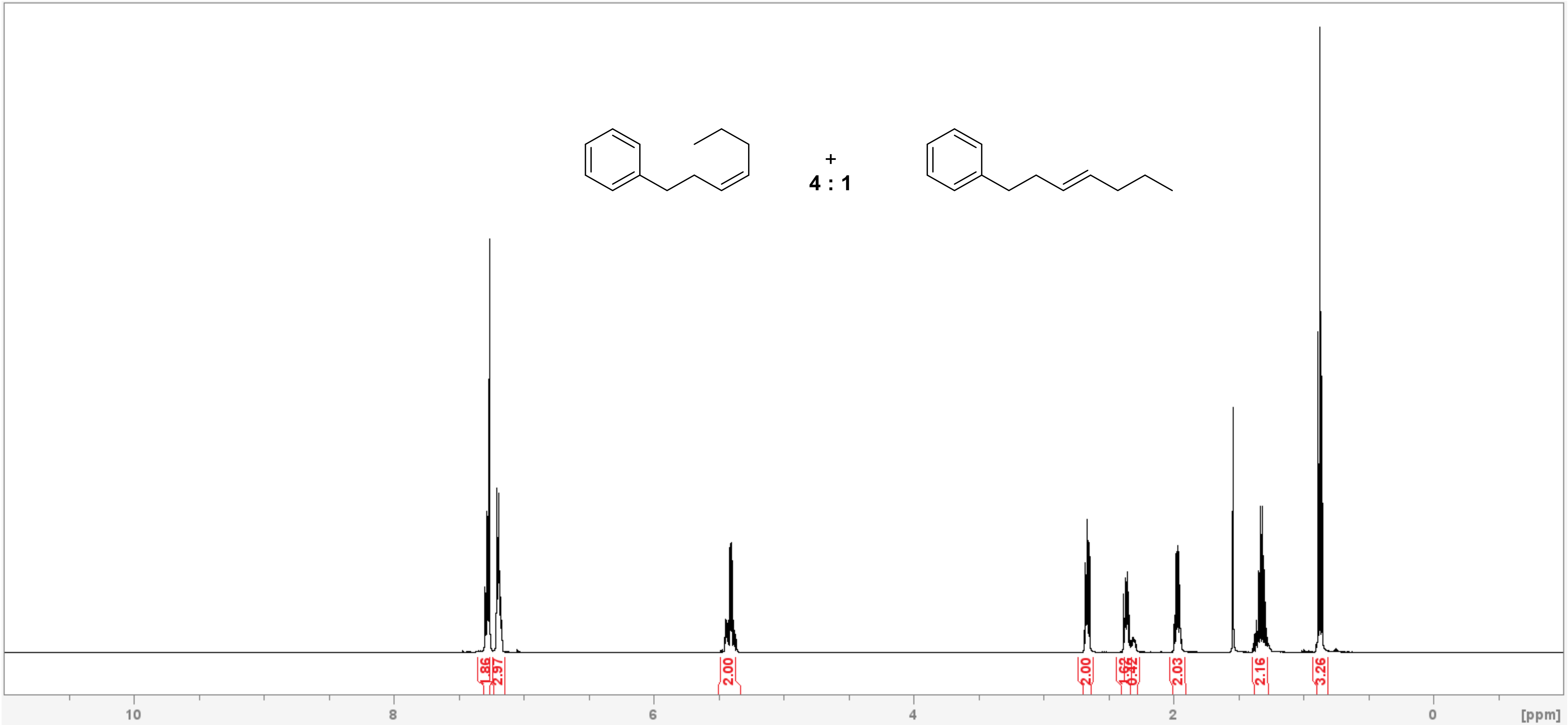

(*Z*)- + (*E*)-Hept-3-en-1-ylbenzene (4:1) (*int₁*-4j)

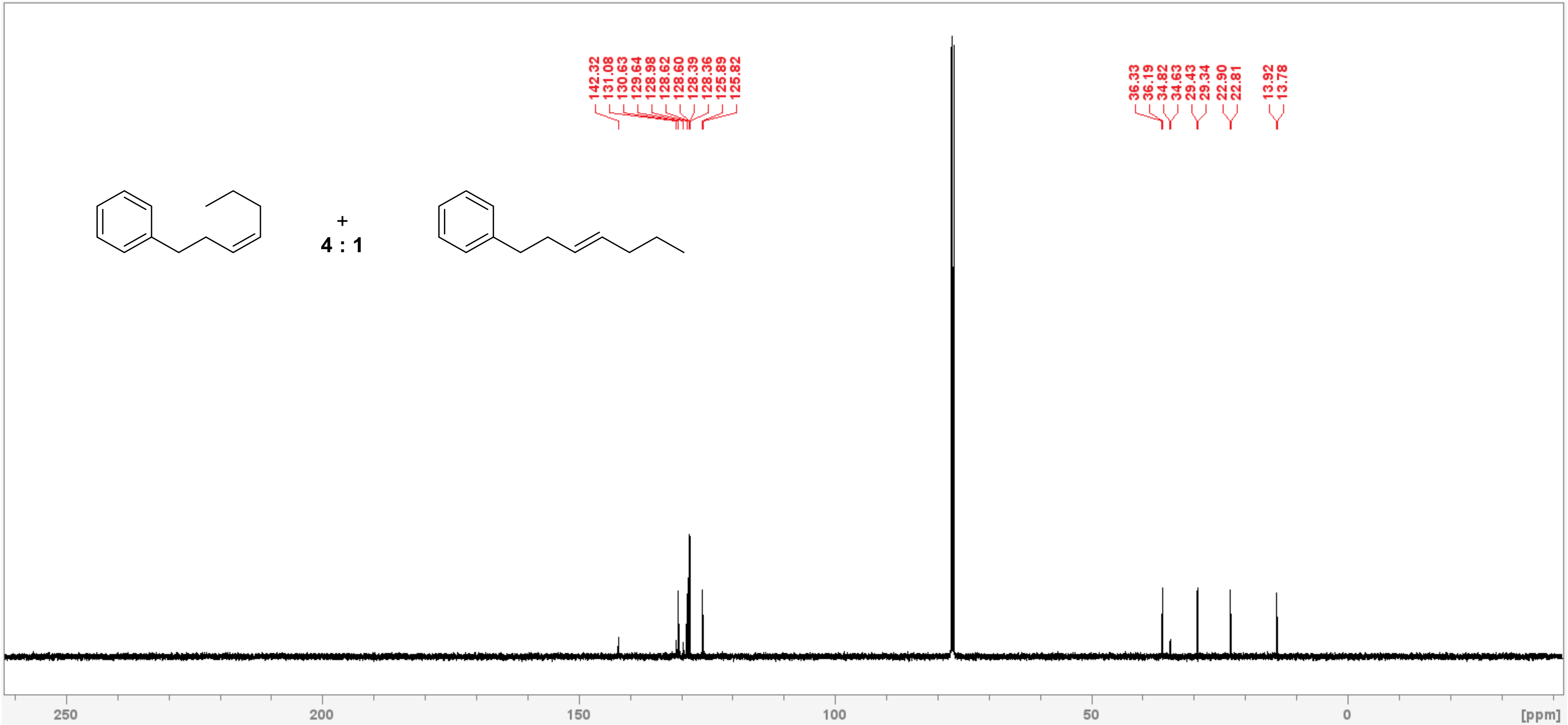

(*anti*)-1-Phenylheptane-3,4-diol (*rac*-*anti*-4j)

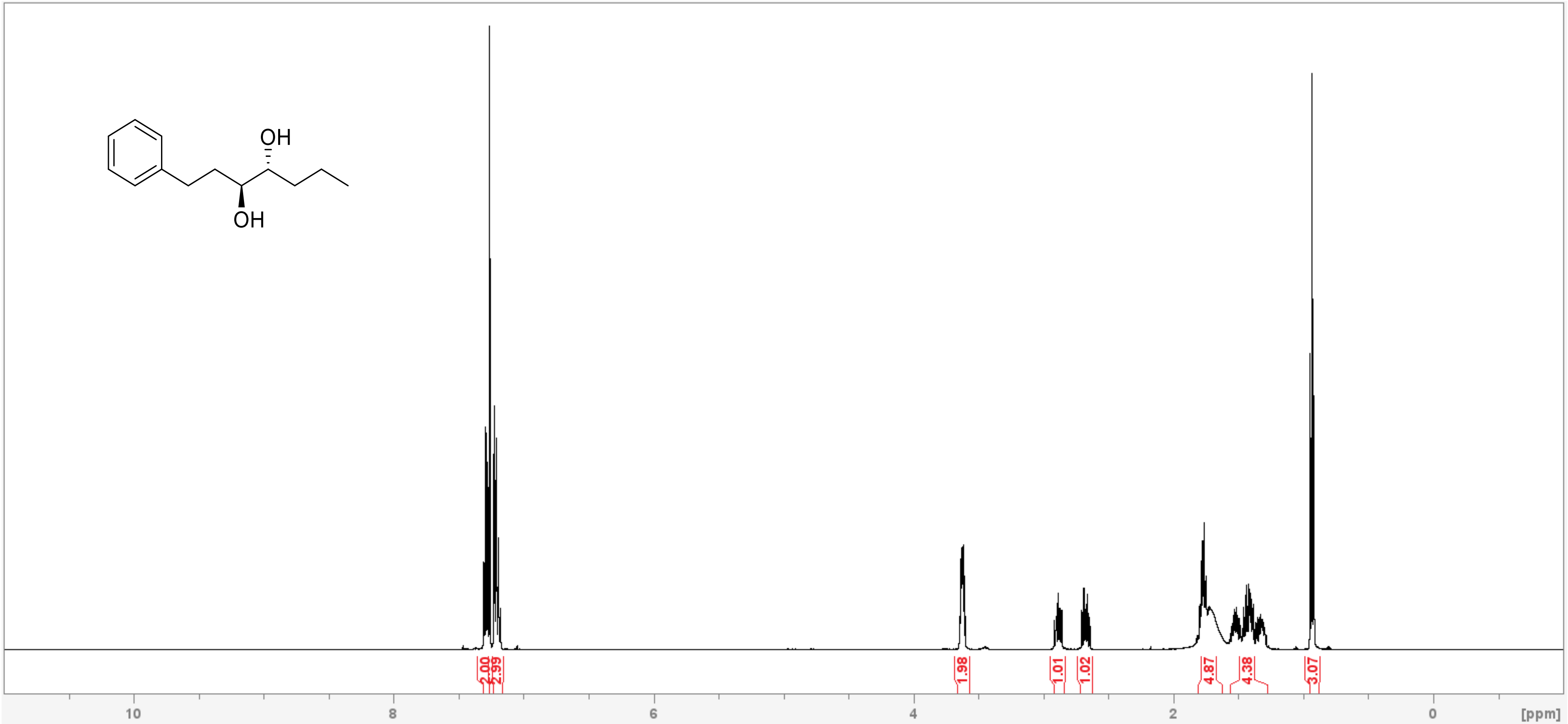

(*anti*)-1-Phenylheptane-3,4-diol (*rac*-*anti*-4j)

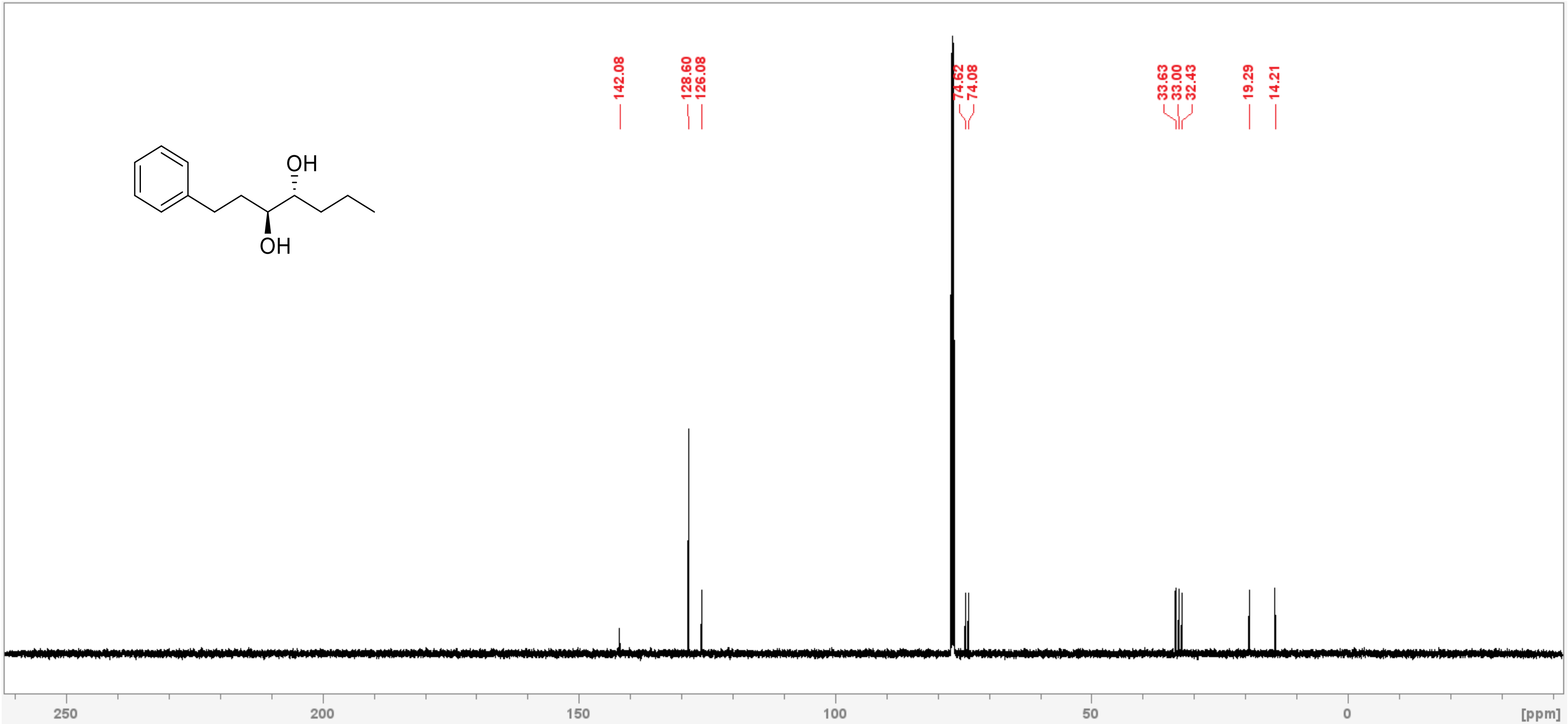

(*syn*)-1-Phenylheptane-3,4-diol (*rac*-*syn*-4j)

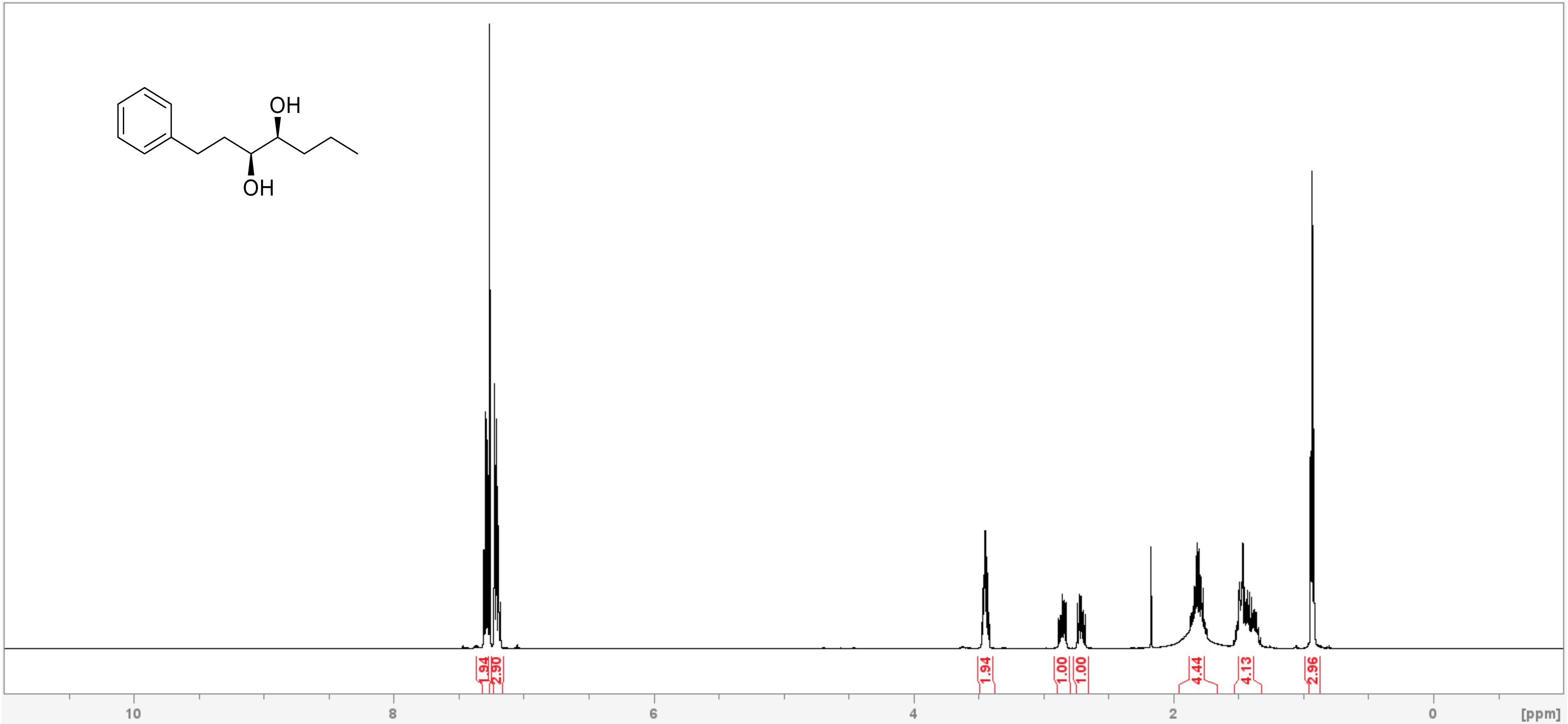

(*syn*)-1-Phenylheptane-3,4-diol (*rac*-*syn*-4j)

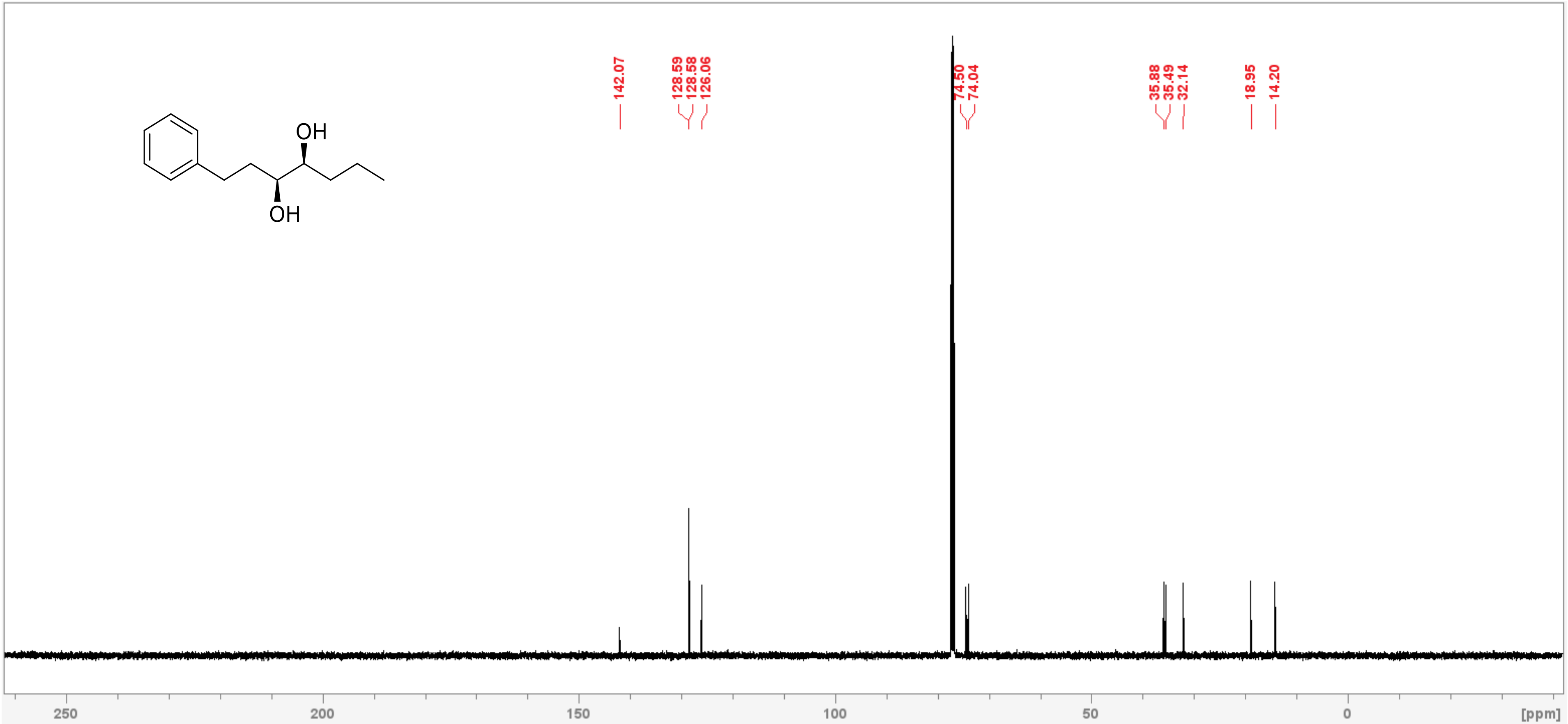

(Z)- + (E)-But-2-ene-1,1-diyldibenzene (int1-4k)

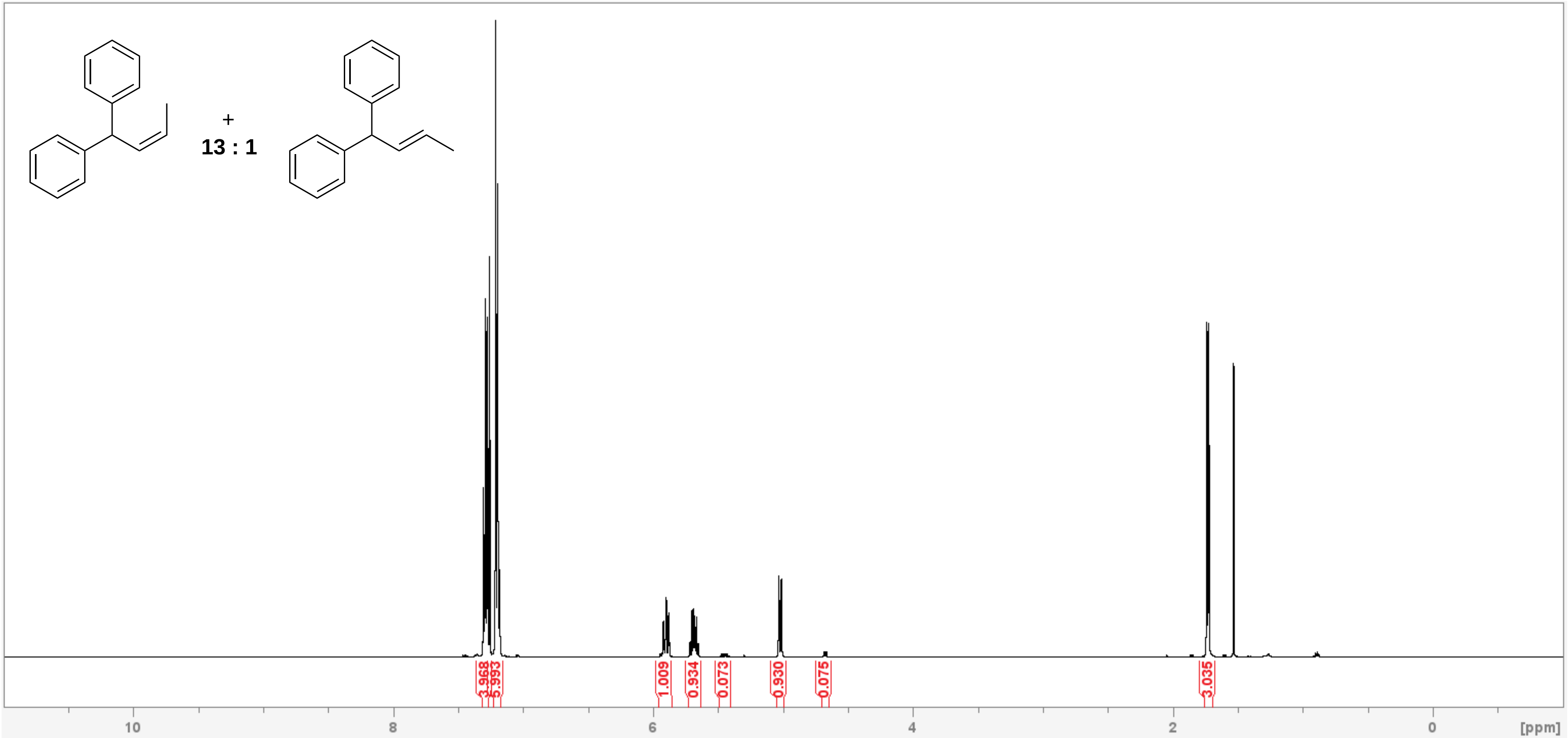

(Z)- + (E)-But-2-ene-1,1-diyldibenzene (int1-4k)

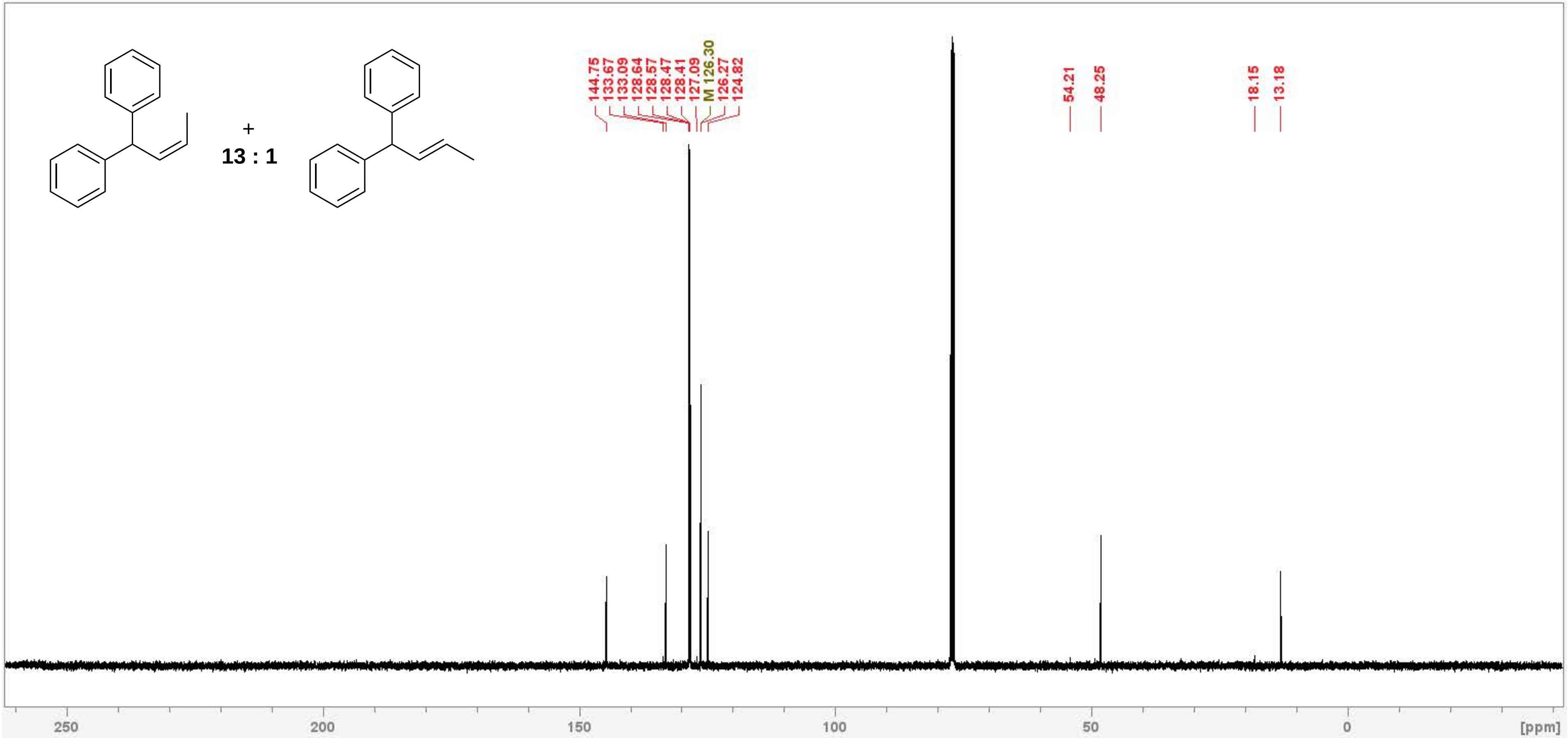

(*anti*)-1,1-Diphenylbutane-2,3-diol (*rac-anti*-4k)

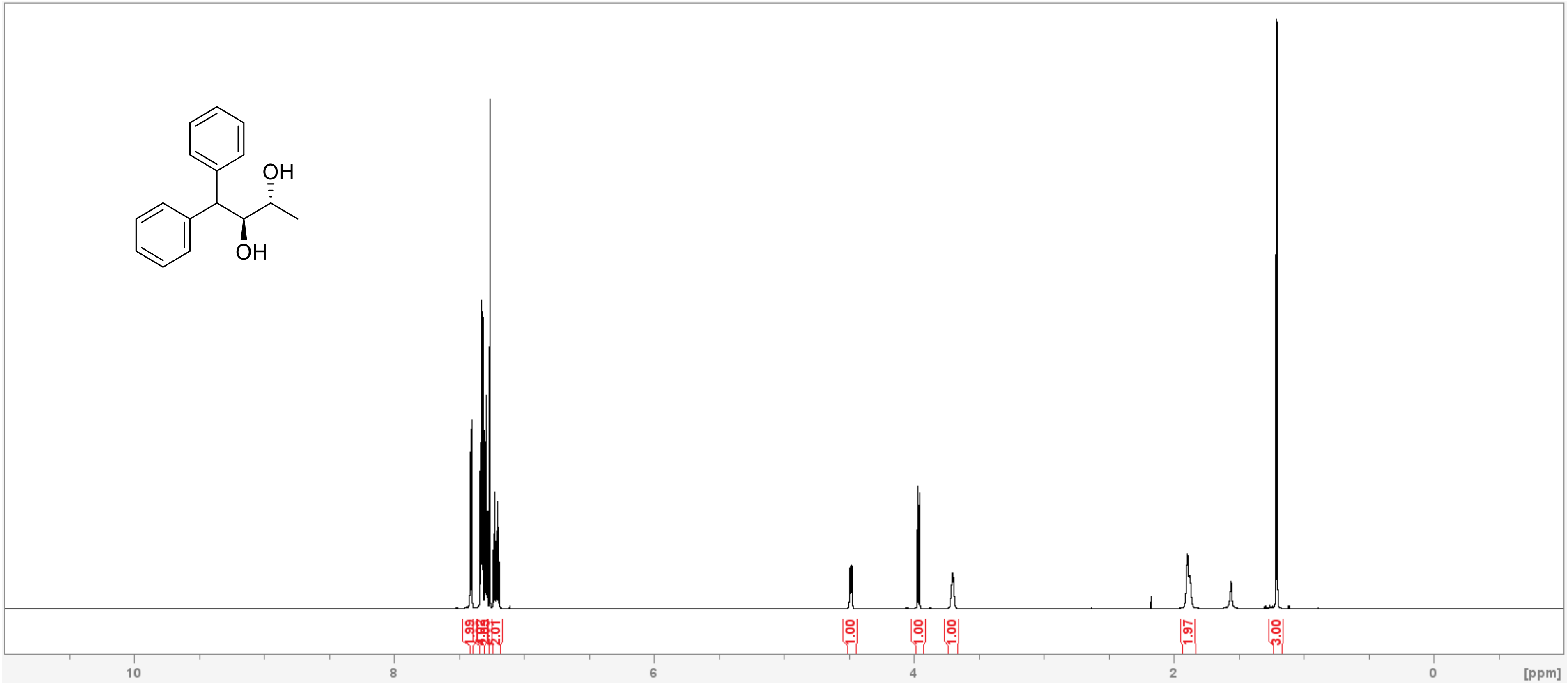

(*anti*)-1,1-Diphenylbutane-2,3-diol (*rac-anti*-4k)

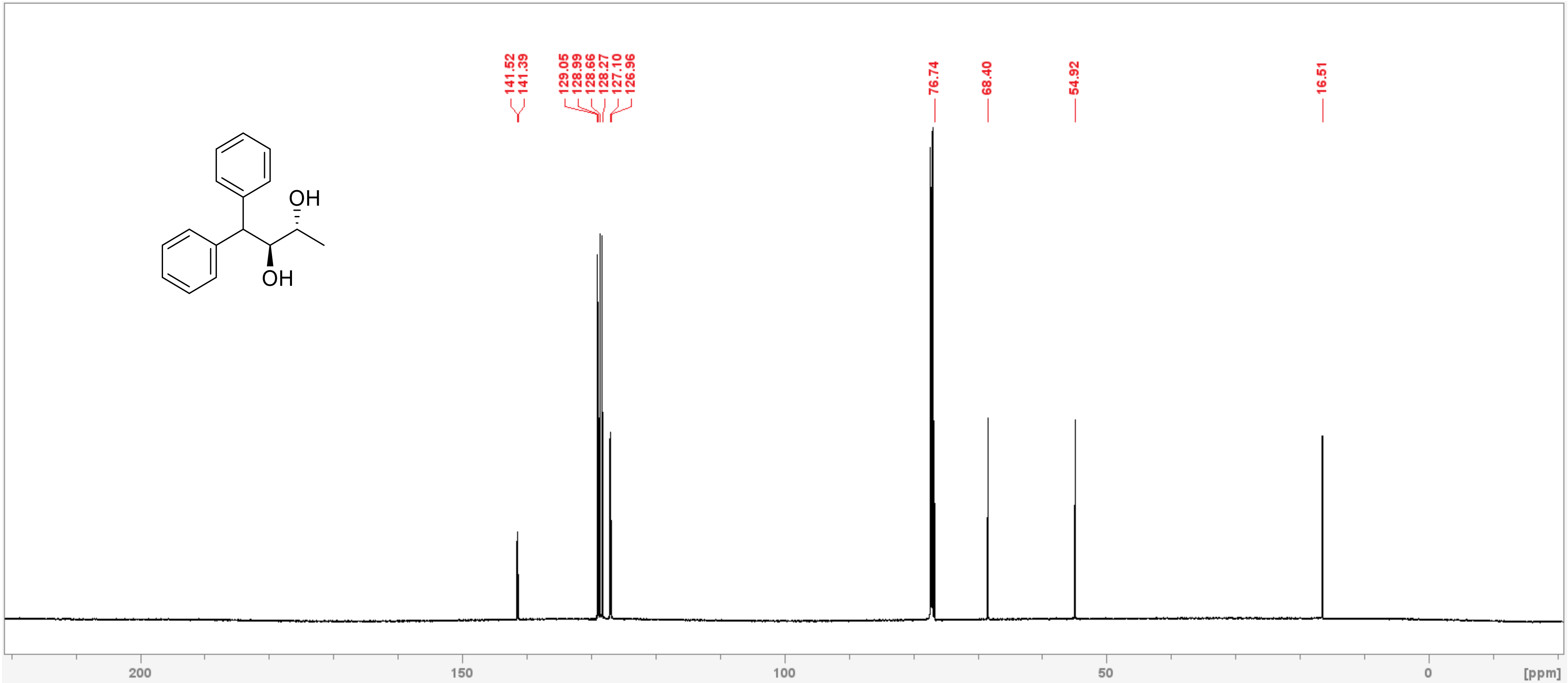

(*syn*)-1,1-Diphenylbutane-2,3-diol (*rac*-*syn*-4k)

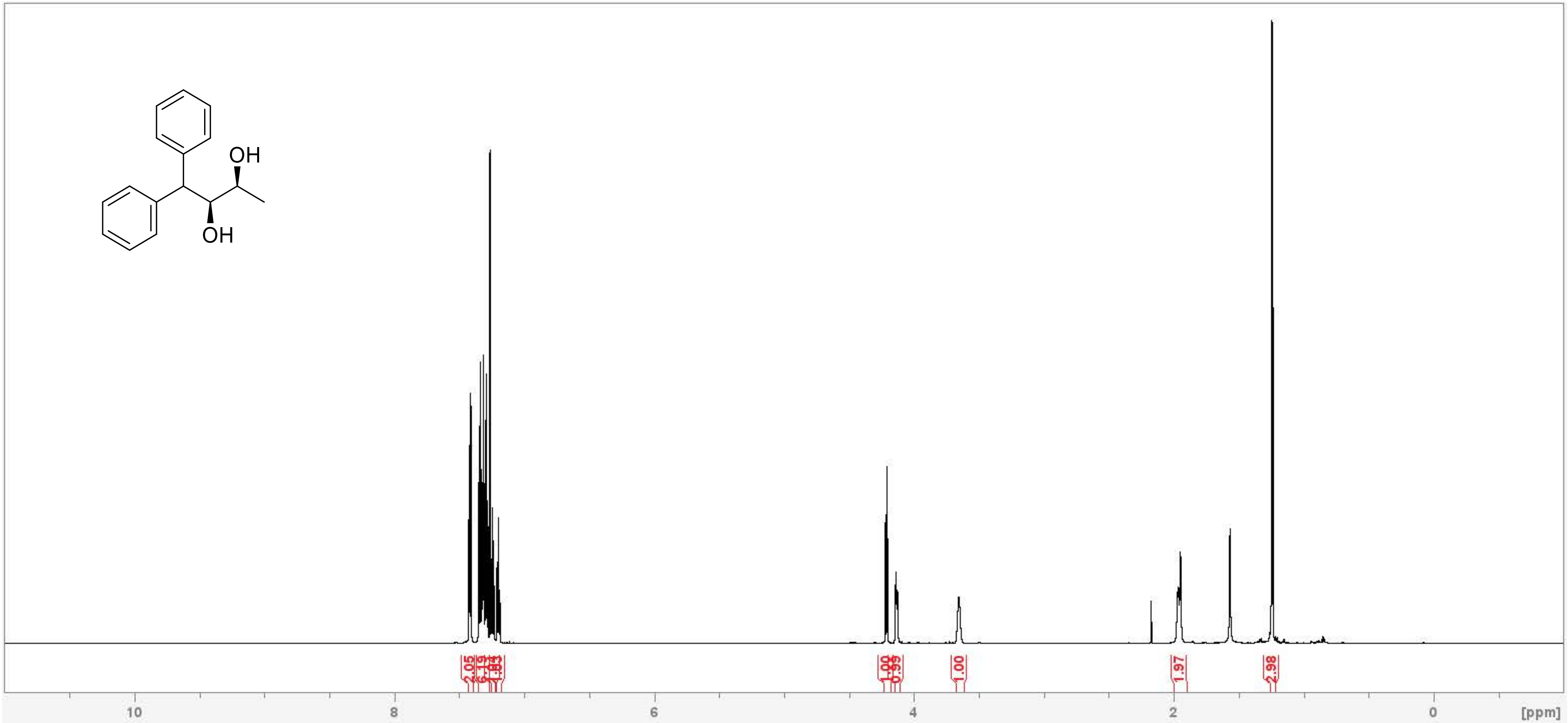

(*syn*)-1,1-Diphenylbutane-2,3-diol (*rac*-*syn*-4k)

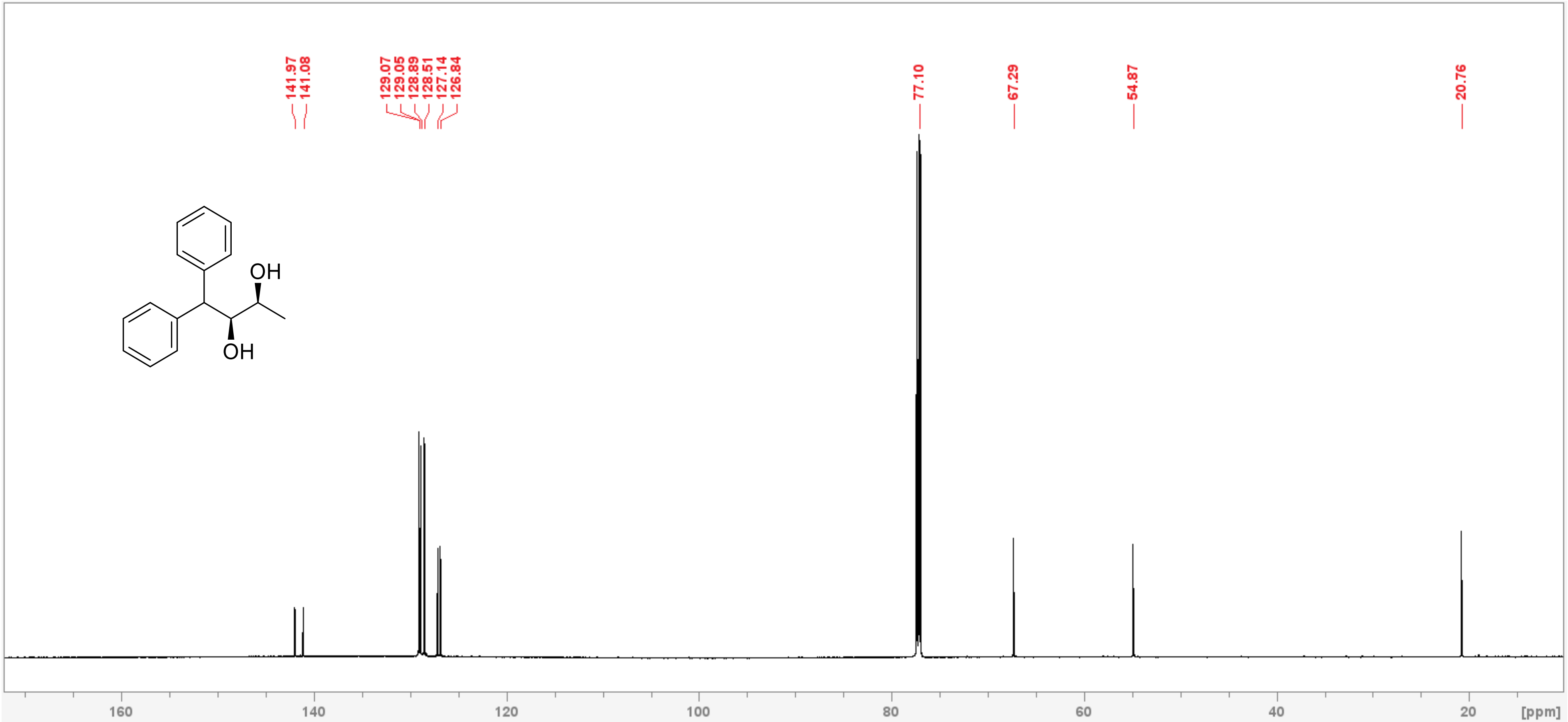

(*Z*)-Hex-4-en-1-yl benzoate ((*Z*)-*int*$_1$-*4l*)

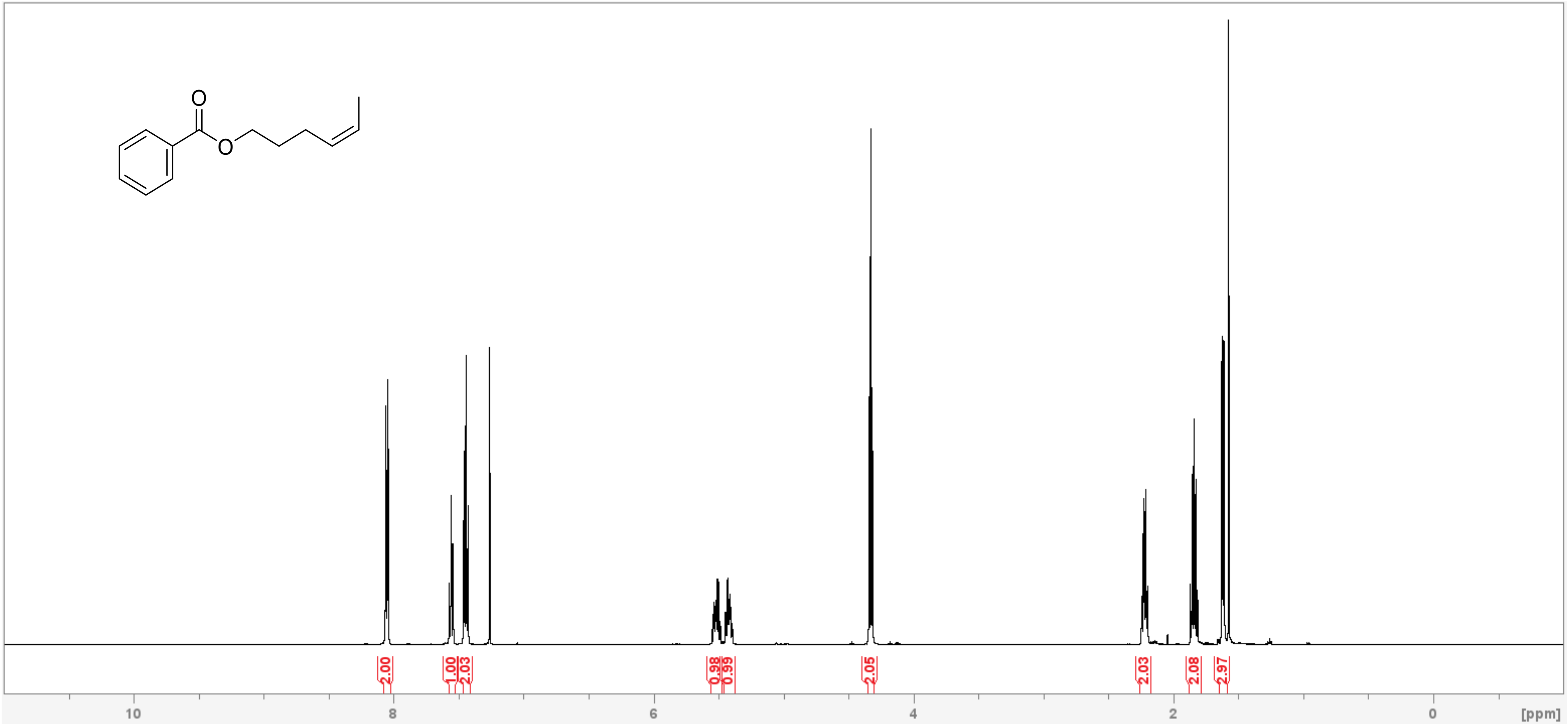

(*Z*)-Hex-4-en-1-yl benzoate ((*Z*)-*int*$_1$-4l)

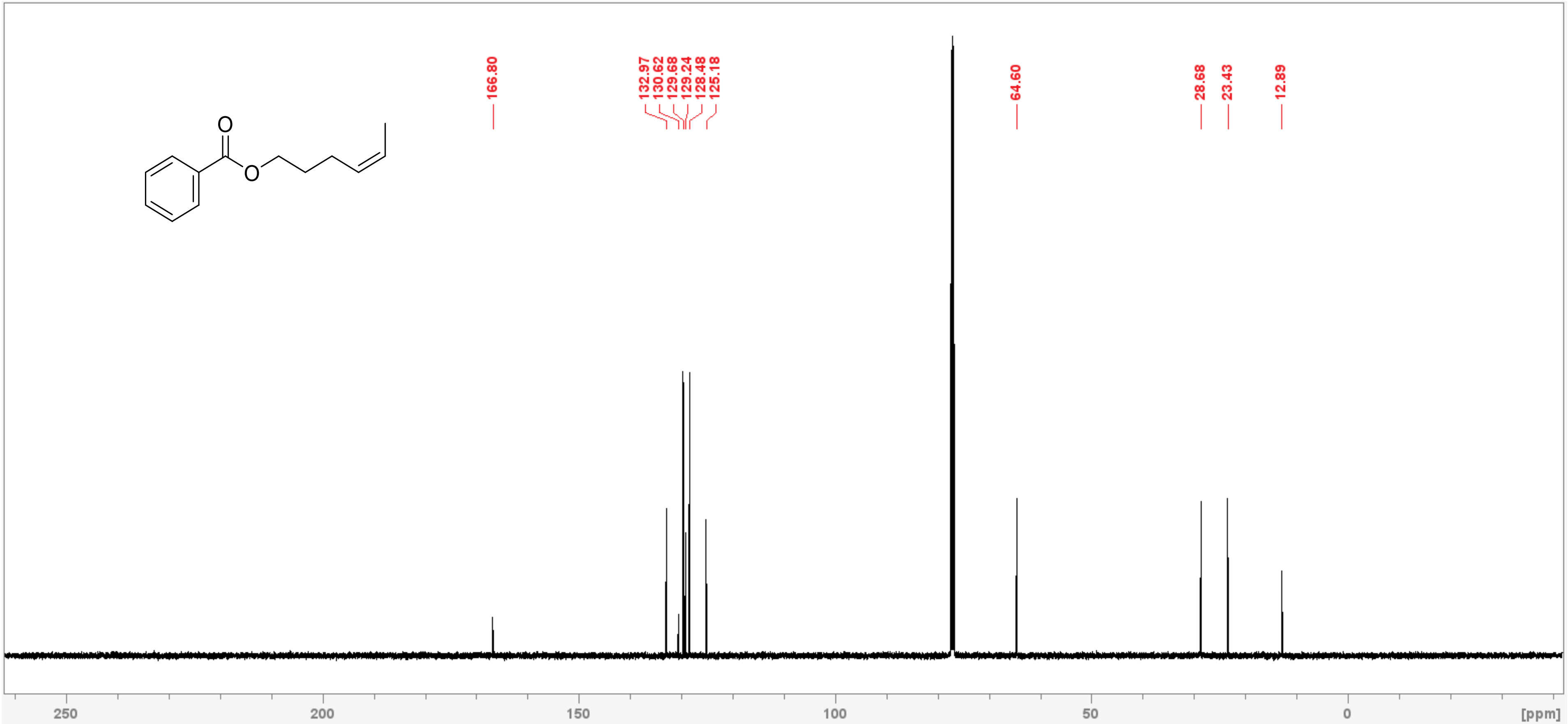

(*anti*)-4,5-Dihydroxyhexyl benzoate (*rac*-*anti*-4l)

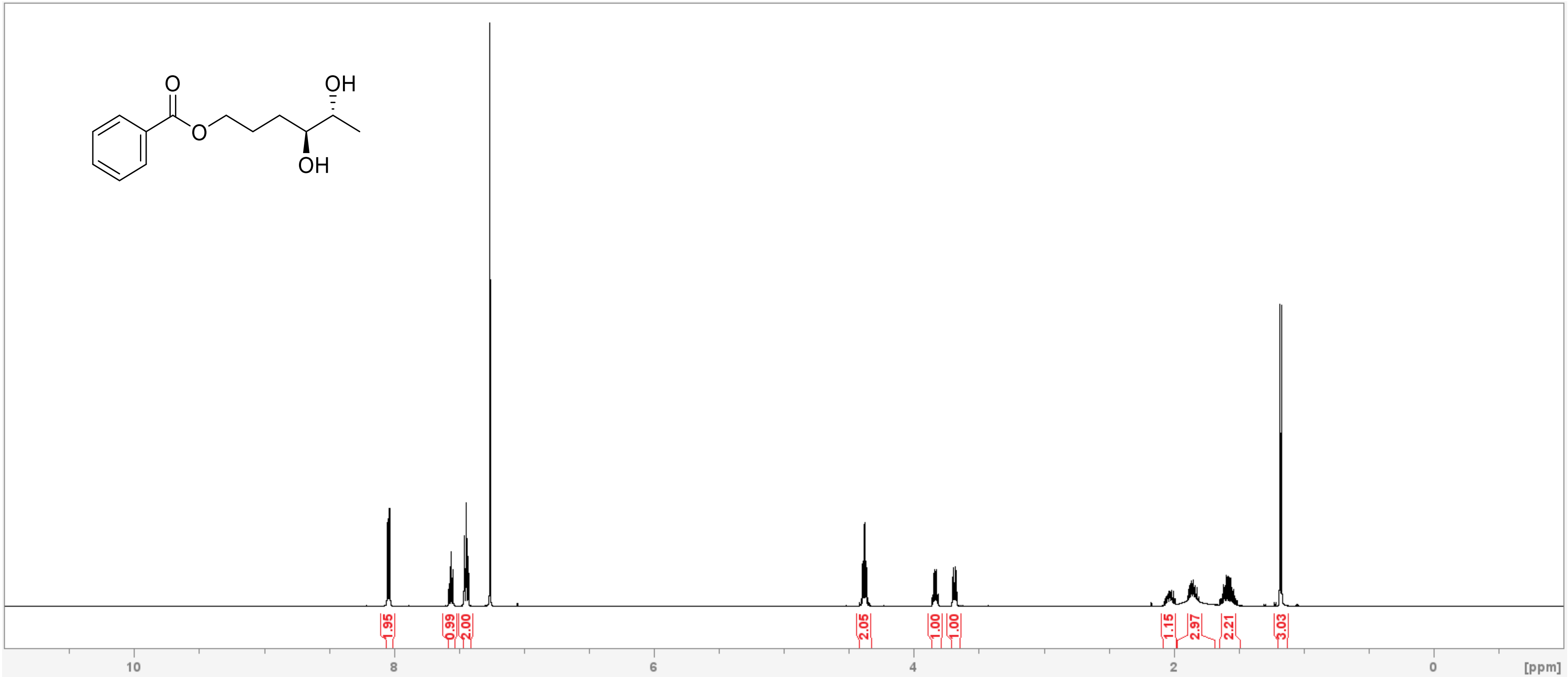

(*anti*)-4,5-Dihydroxyhexyl benzoate (*rac*-*anti*-4l)

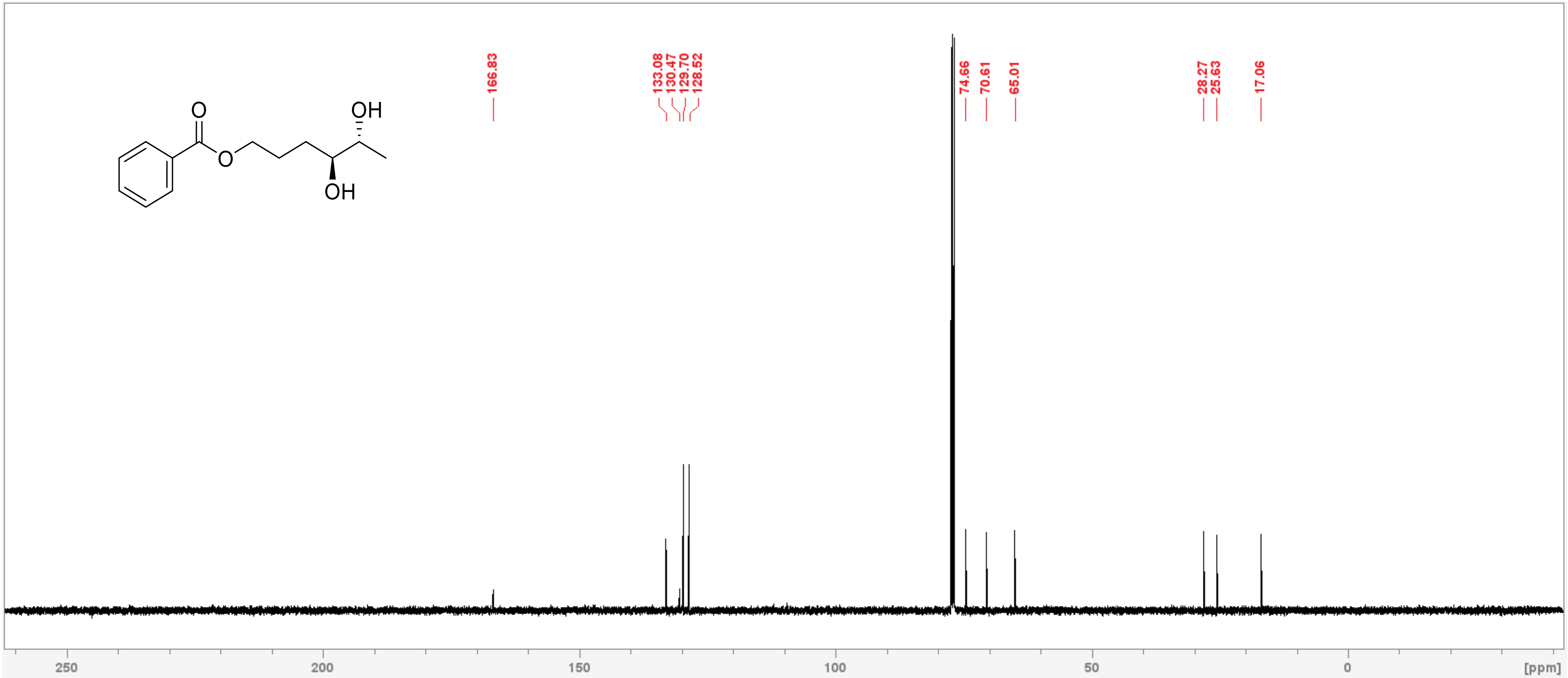

(*E*)-Hex-4-en-1-yl benzoate ((*E*)-*int₁*-4l)

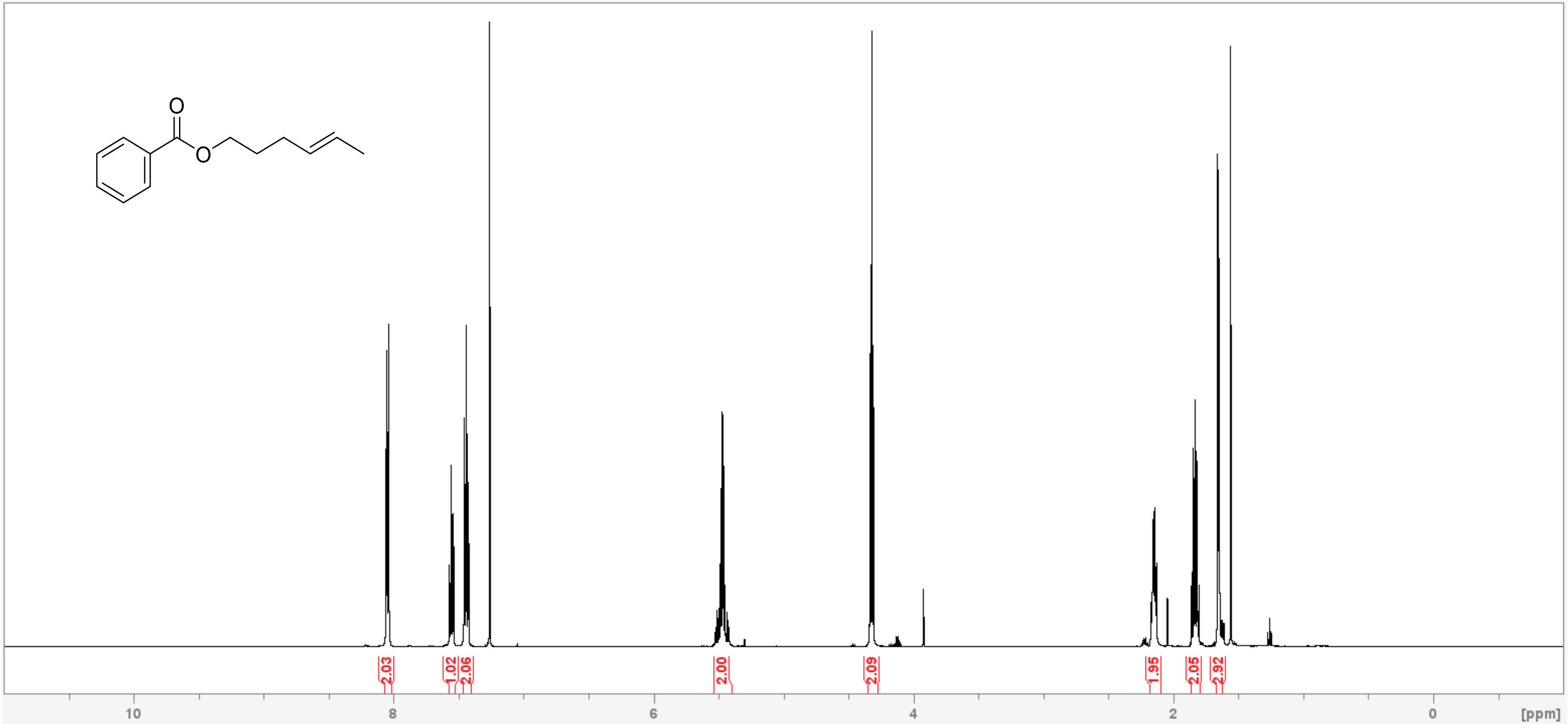

(*E*)-Hex-4-en-1-yl benzoate ((*E*)-*int₁*-4l)

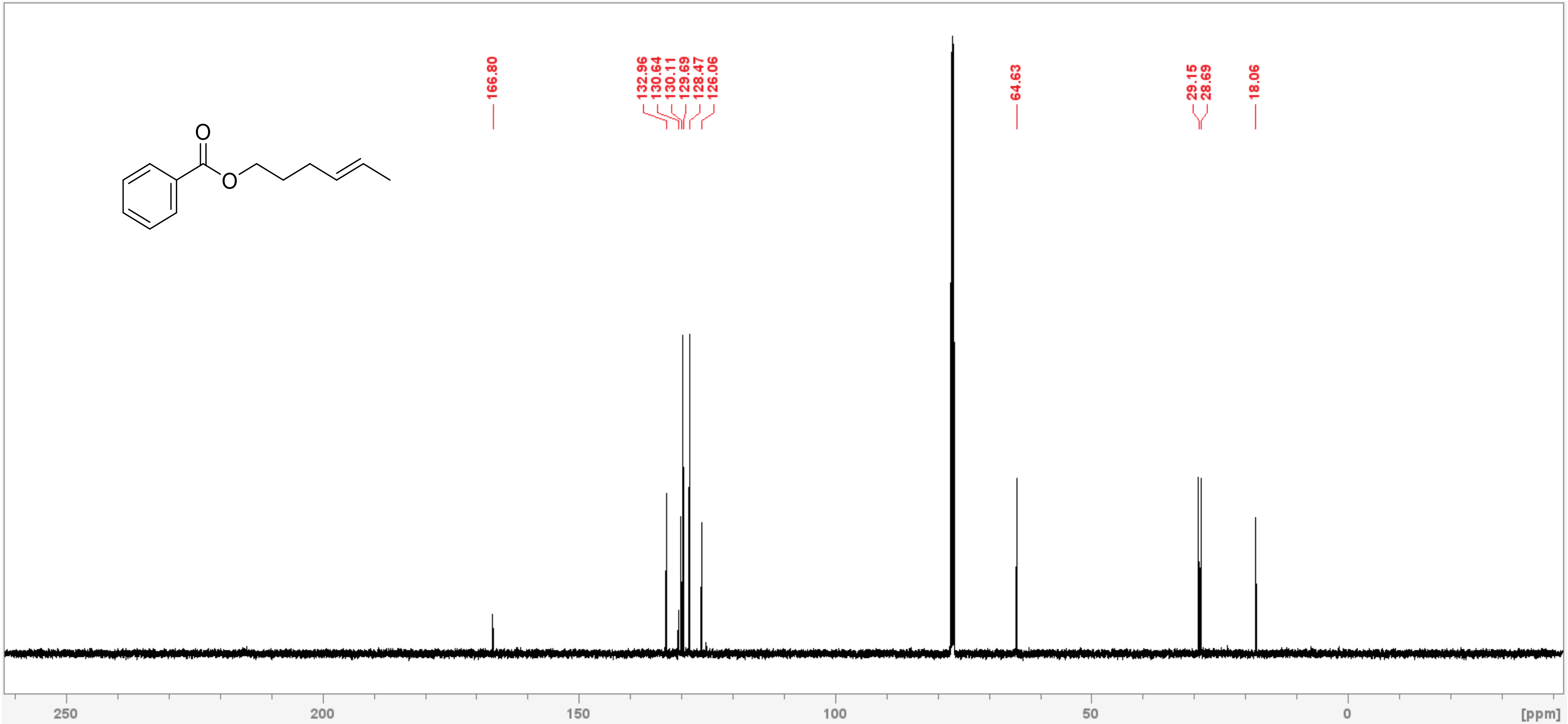

(*syn*)-4,5-Dihydroxyhexyl benzoate (*rac*-*syn*-4l)

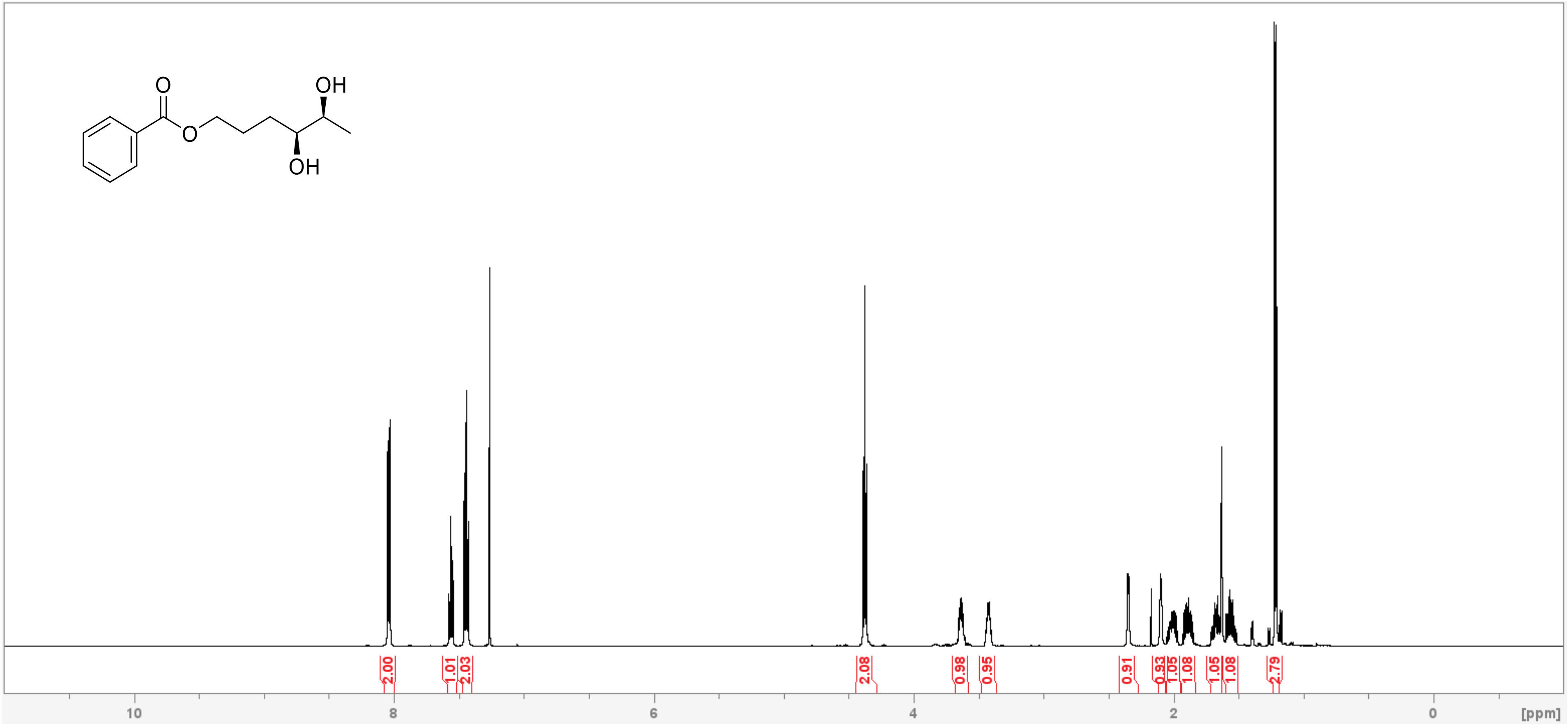

(*syn*)-4,5-Dihydroxyhexyl benzoate (*rac*-*syn*-4l)

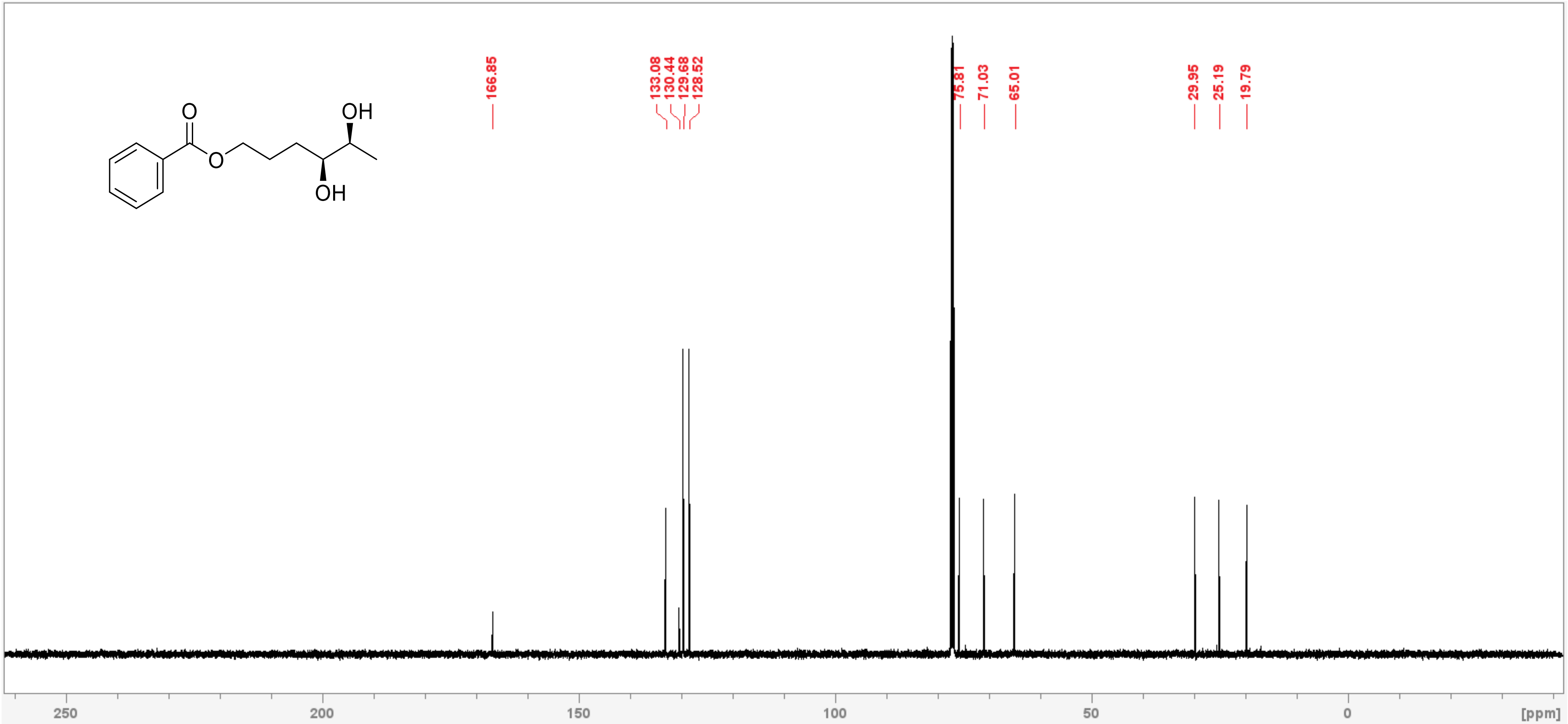

(*Z*)-Hex-4-en-1-yl diphenylphosphinate ((*Z*)-*int₁*-4m)

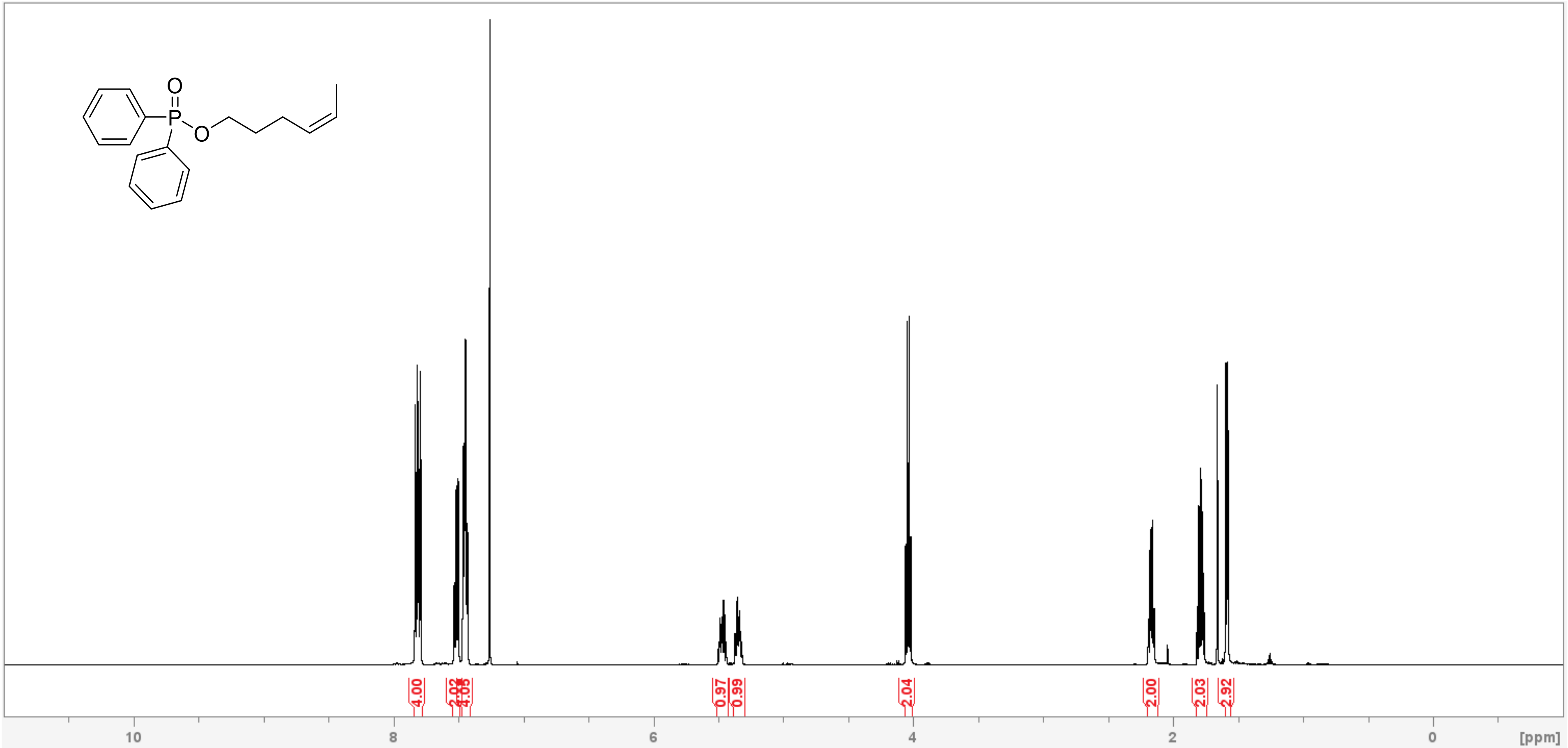

(*Z*)-Hex-4-en-1-yl diphenylphosphinate ((*Z*)-*int*$_1$-4m)

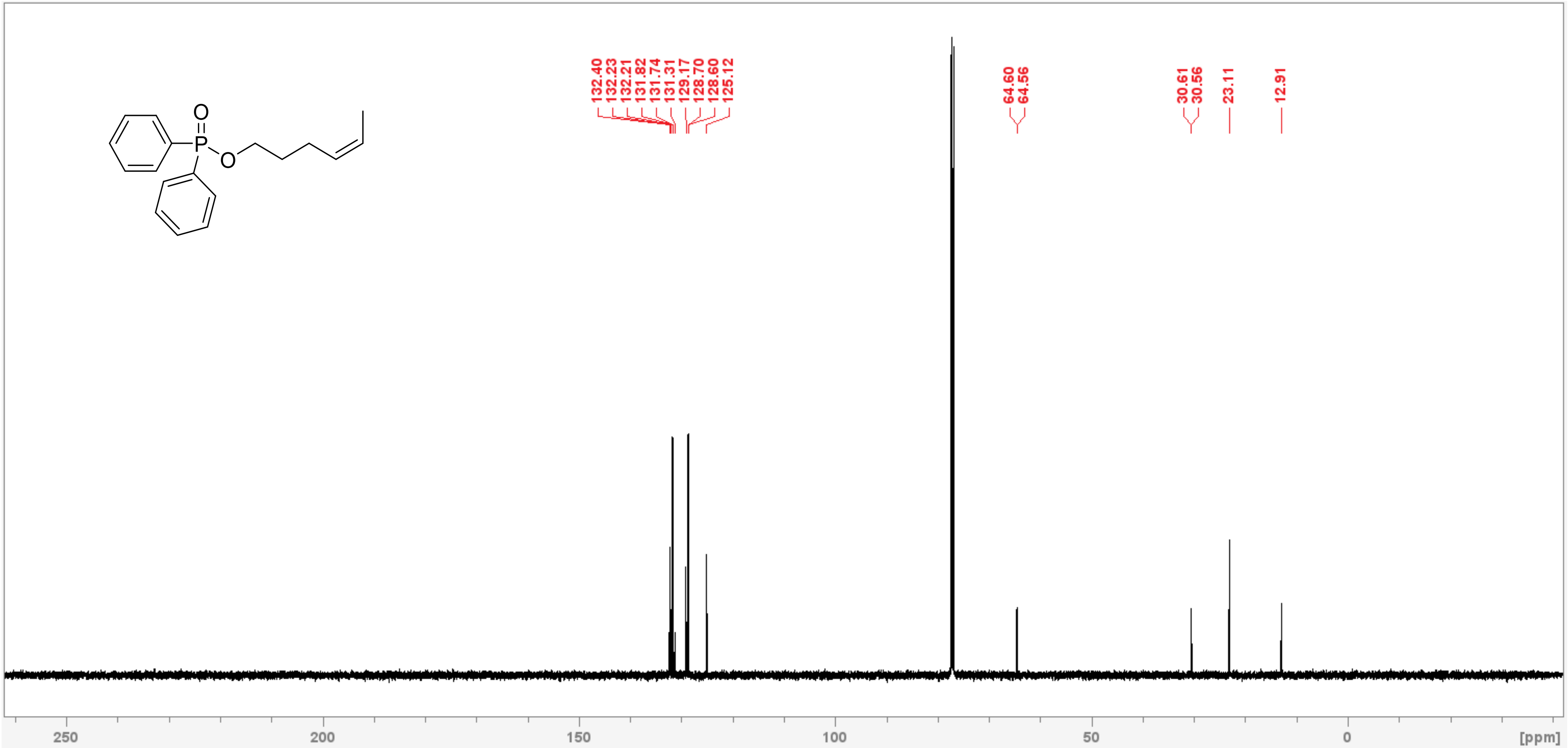

(*Z*)-Hex-4-en-1-yl diphenylphosphinate ((*Z*)-*int*$_1$-4m)

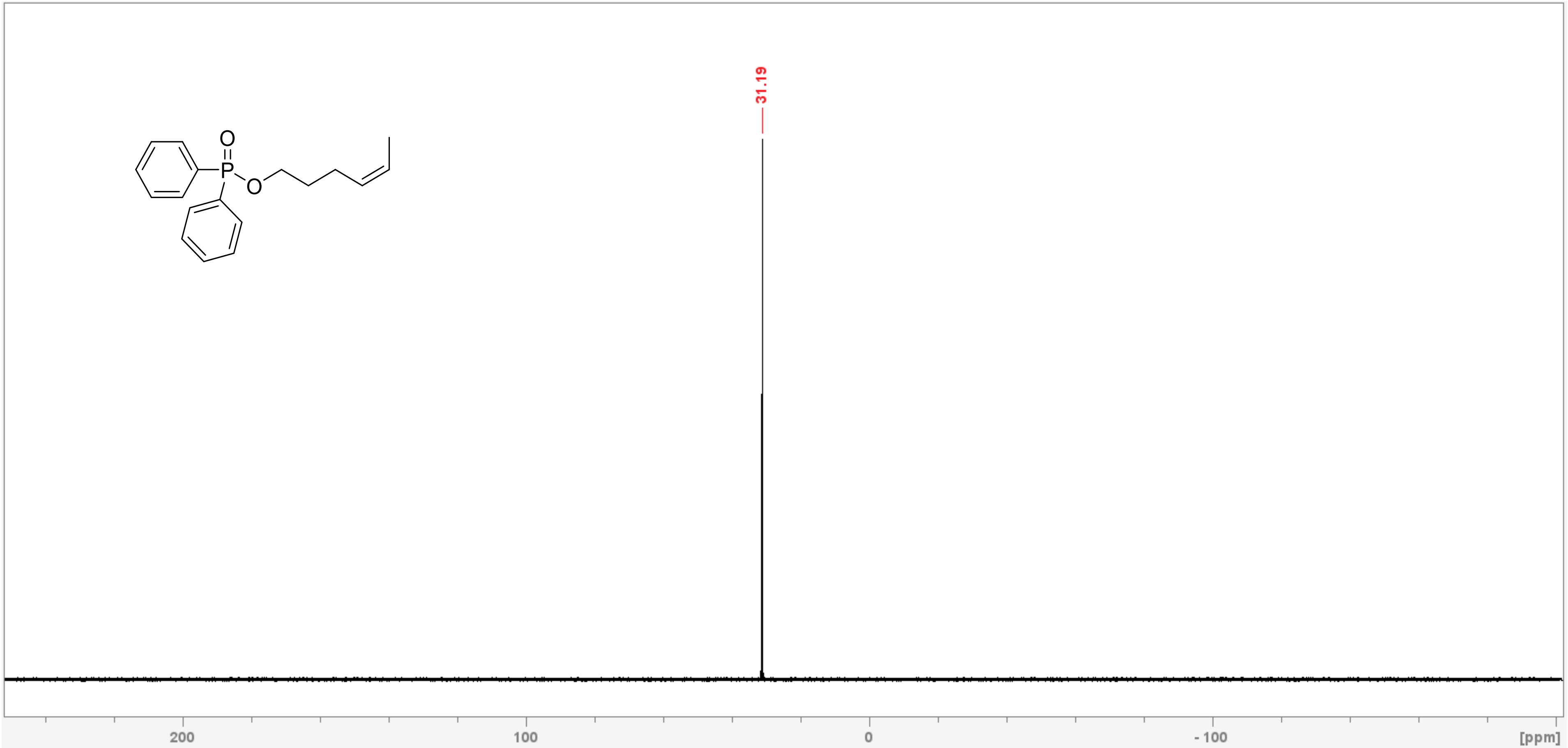

(*anti*)-4,5-Dihydroxyhexyl diphenylphosphinate (*rac*-*anti*-4m)

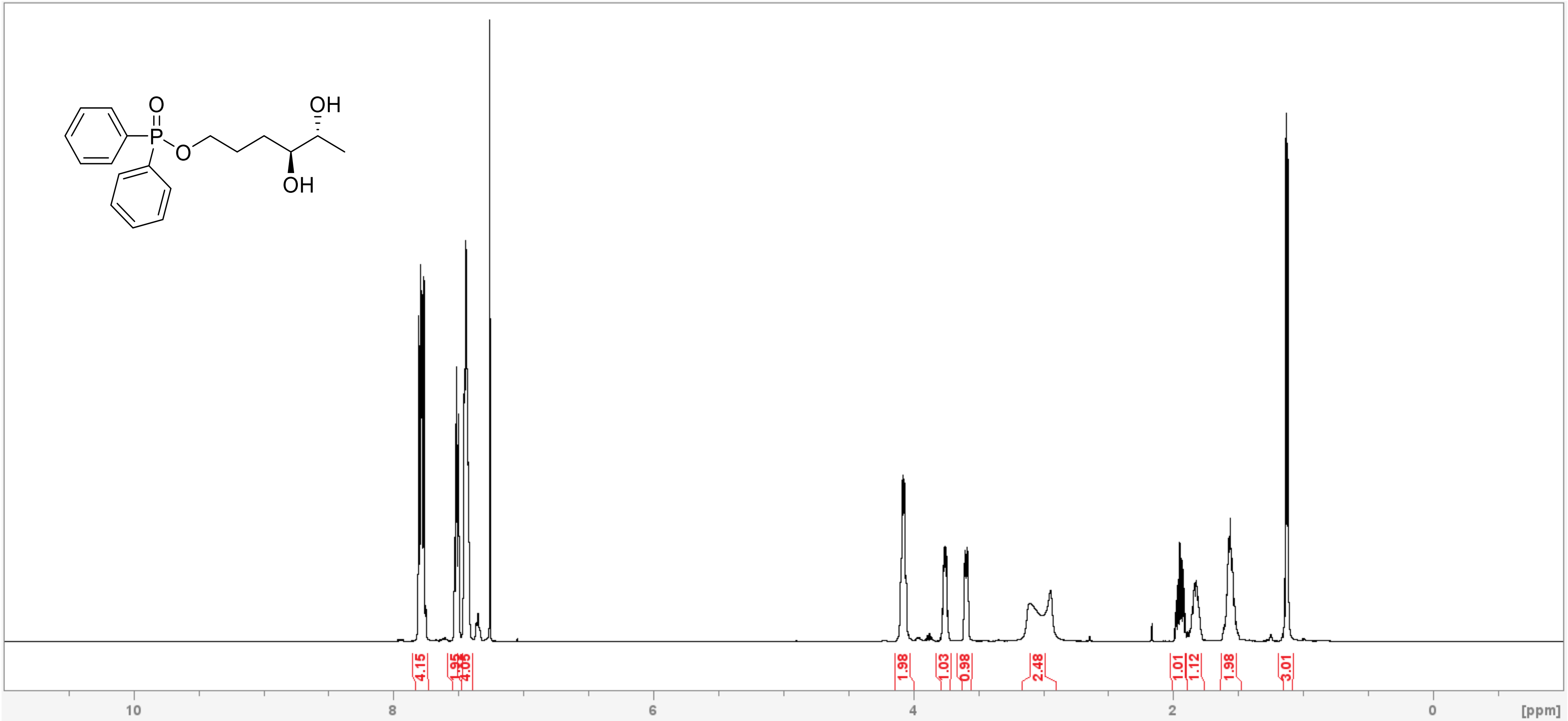

(*anti*)-4,5-Dihydroxyhexyl diphenylphosphinate (*rac*-*anti*-4m)

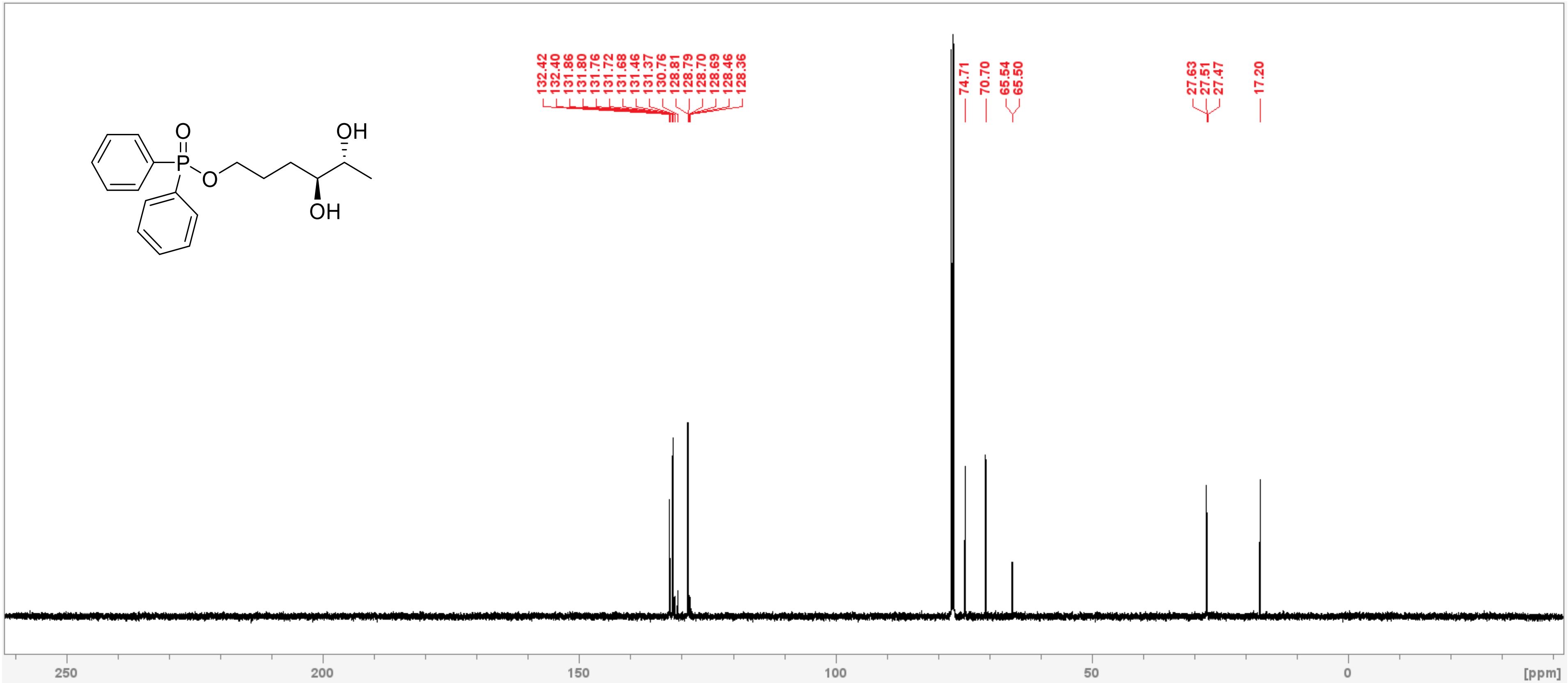

(*anti*)-4,5-Dihydroxyhexyl diphenylphosphinate (*rac*-*anti*-4m)

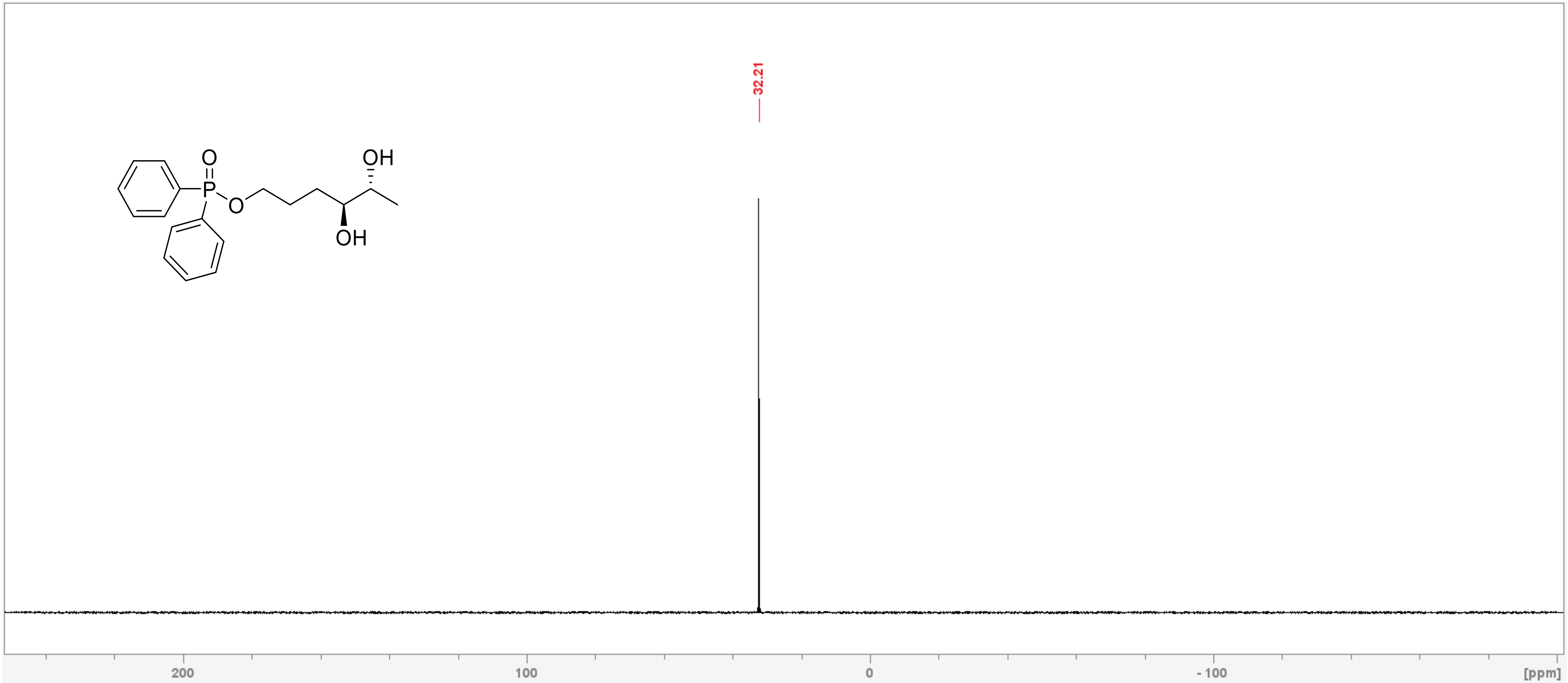

(*E*)-Hex-4-en-1-yl diphenylphosphinate ((*E*)-*int$_1$*-4m)

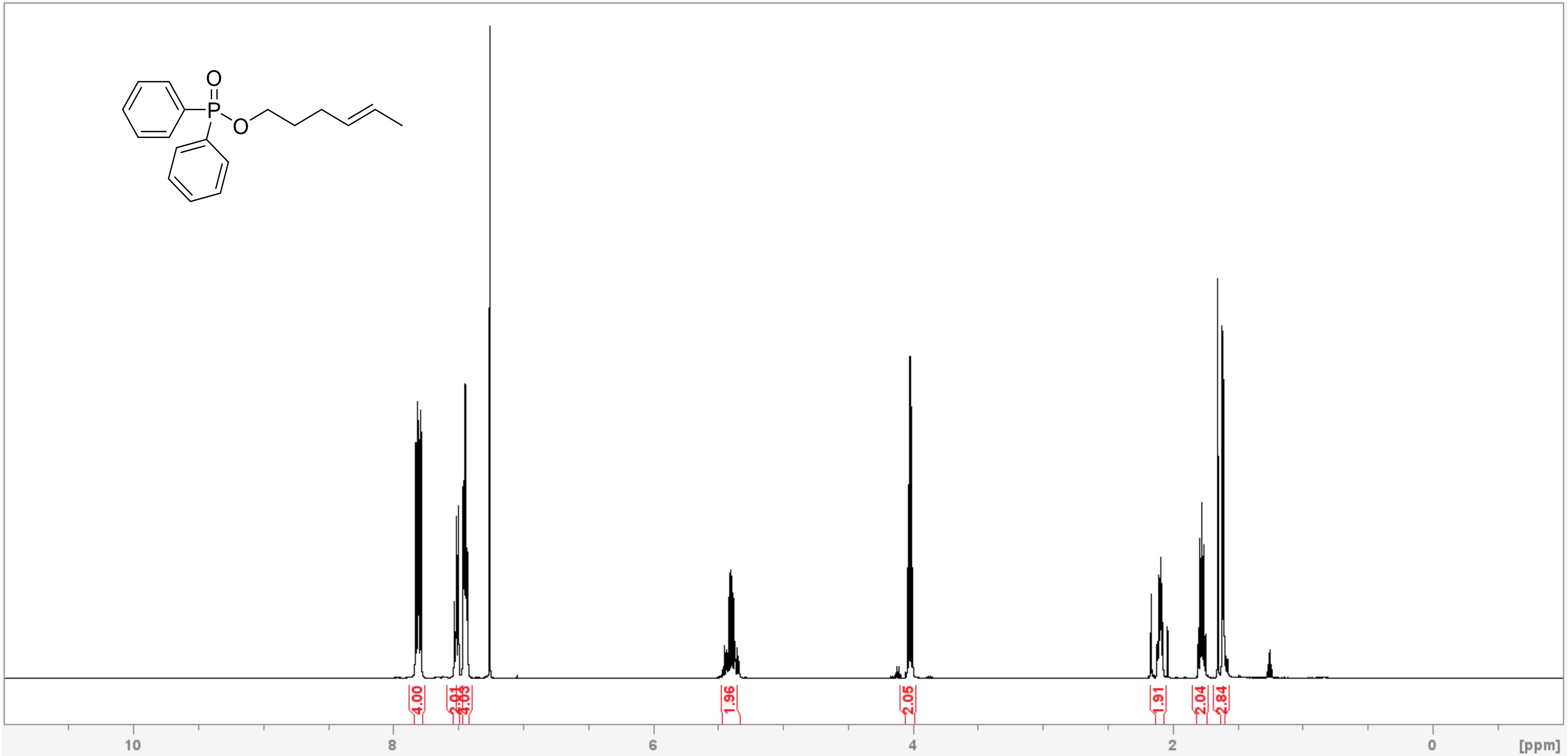

(*E*)-Hex-4-en-1-yl diphenylphosphinate ((*E*)-*int*$_1$-4m)

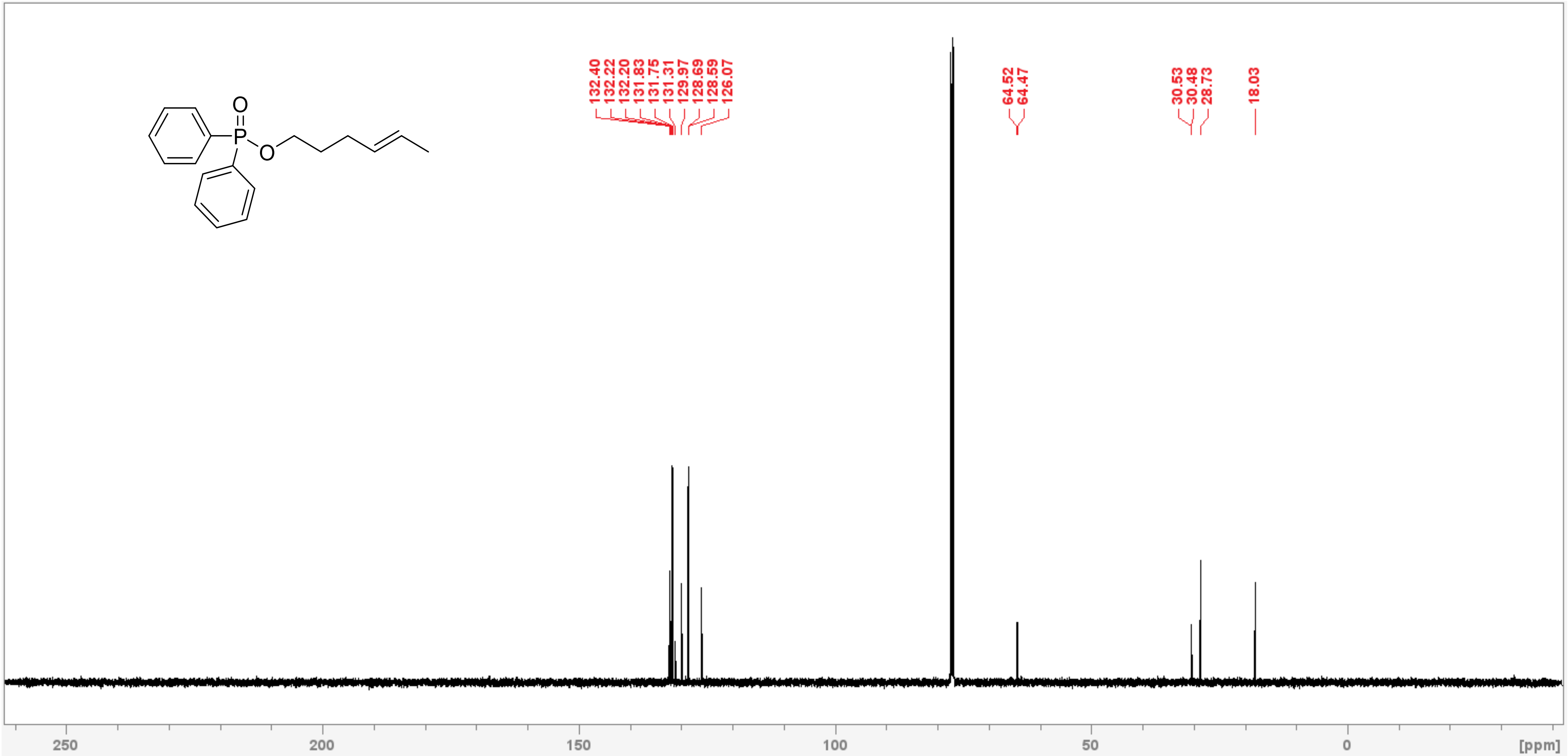

(*E*)-Hex-4-en-1-yl diphenylphosphinate ((*E*)-*int₁*-4m)

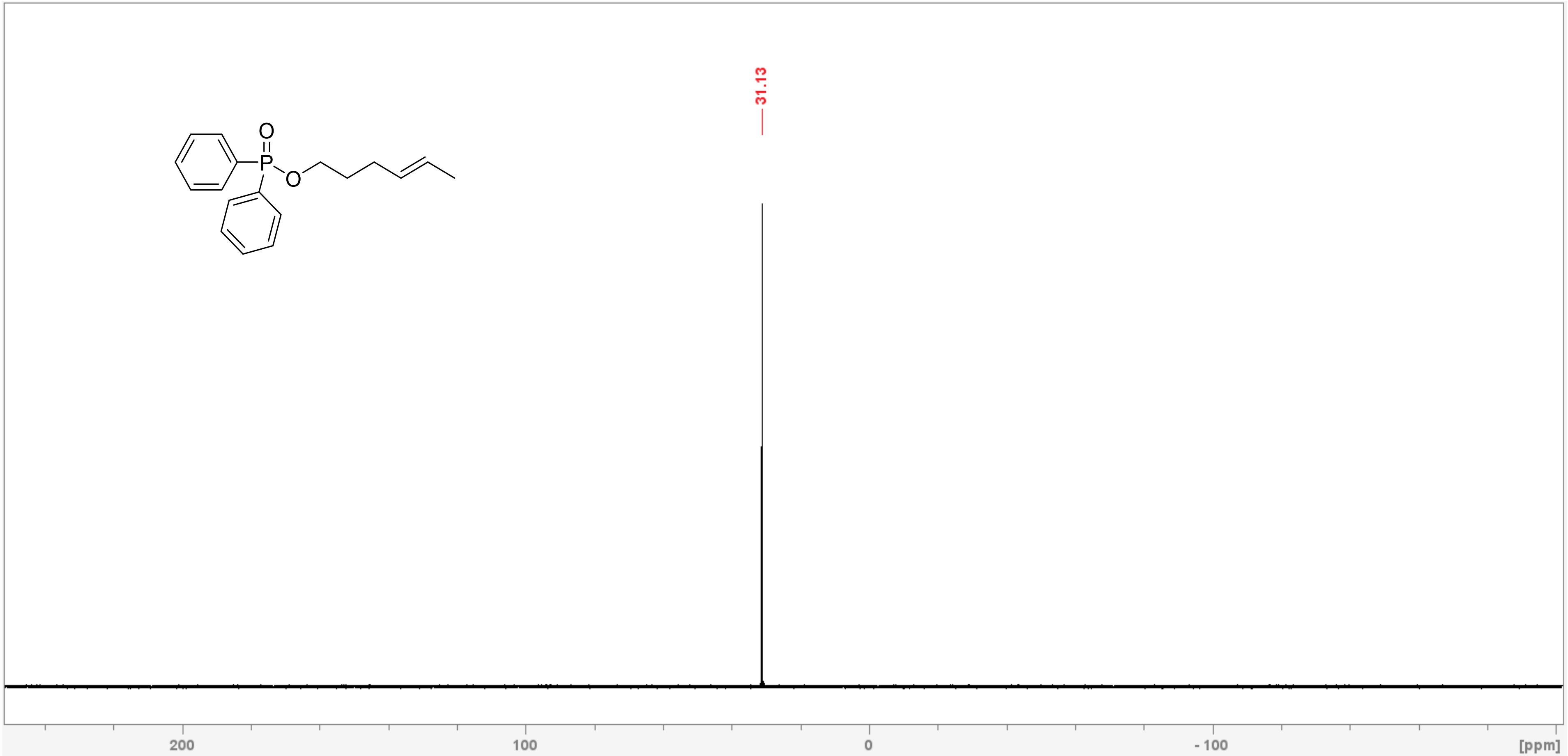

(*syn*)-4,5-Dihydroxyhexyl diphenylphosphinate (*rac*-*syn*-4m)

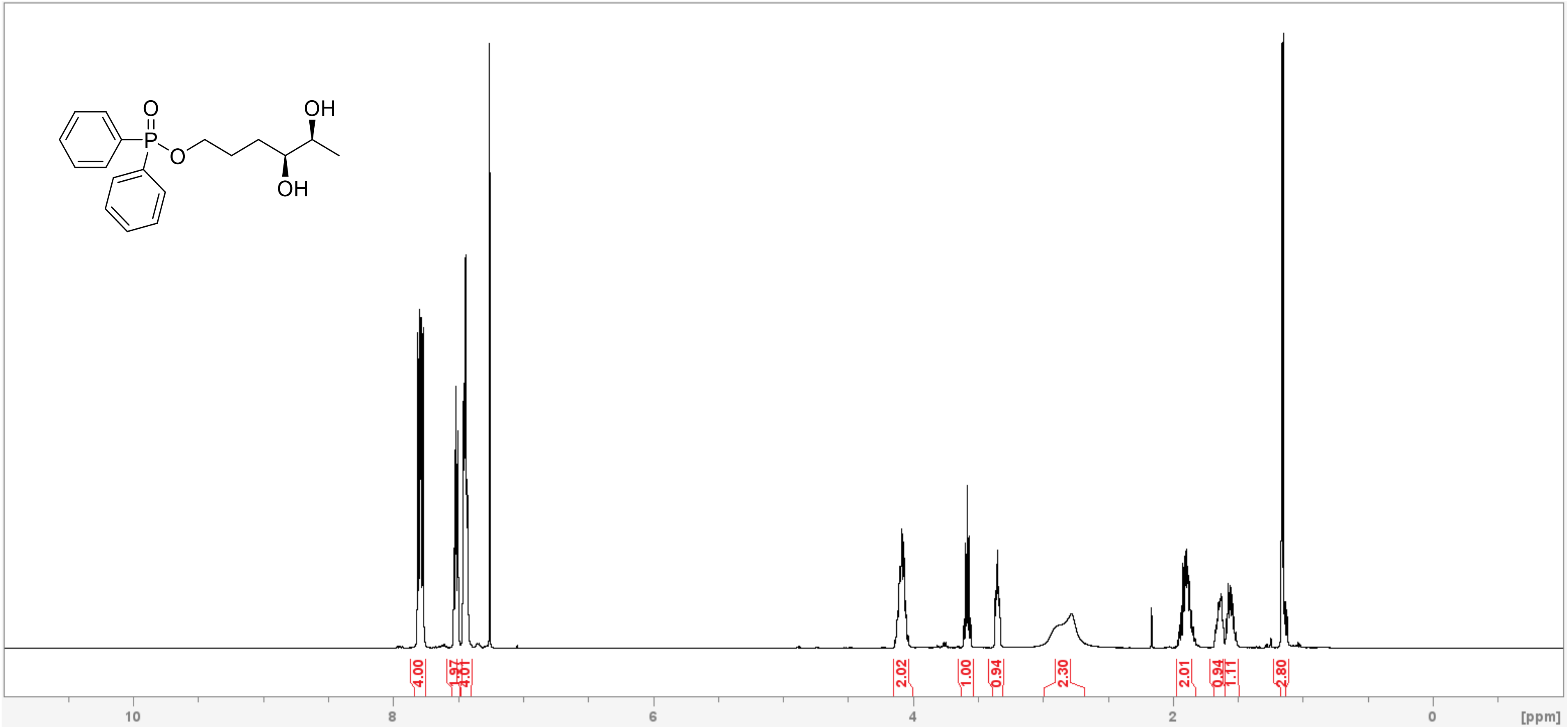

(*syn*)-4,5-Dihydroxyhexyl diphenylphosphinate (*rac*-*syn*-4m)

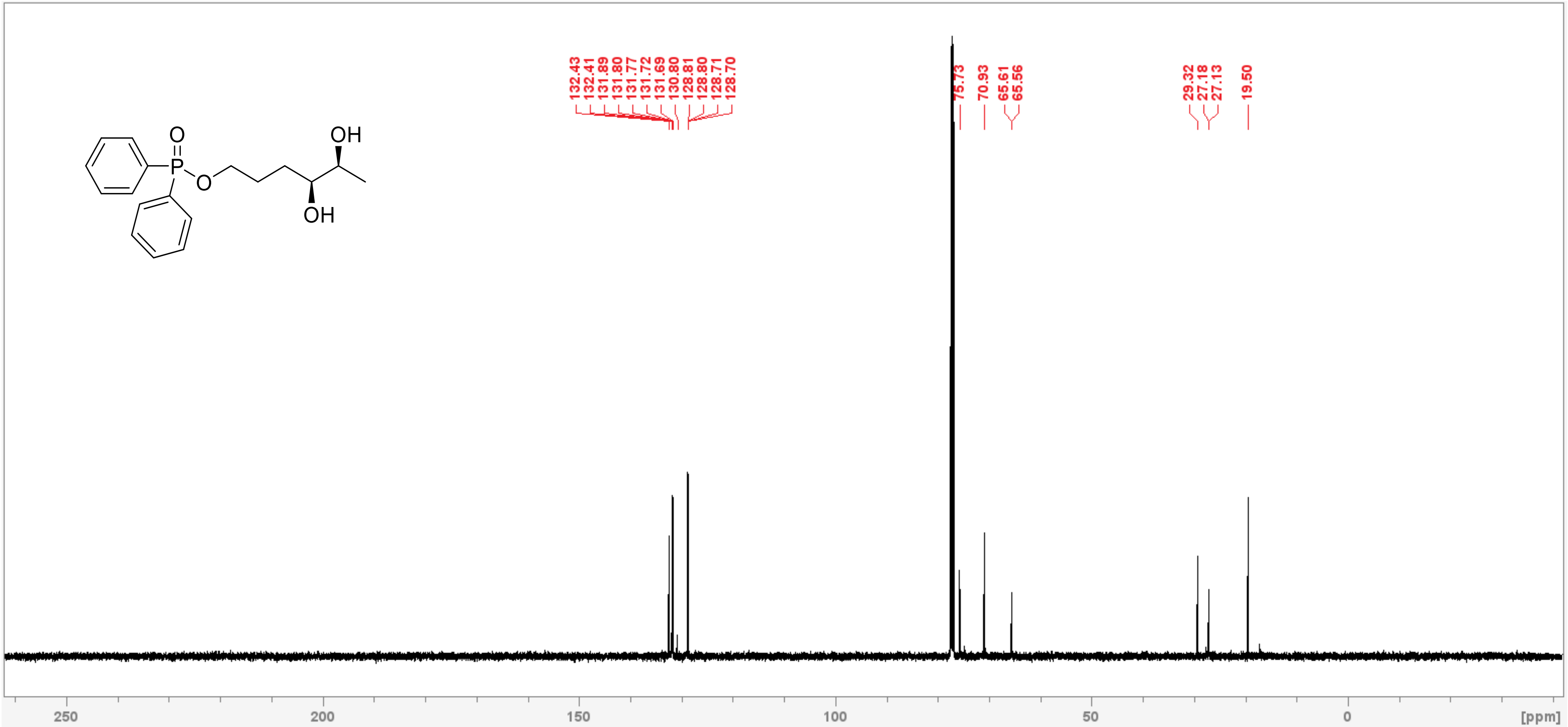

(*syn*)-4,5-Dihydroxyhexyl diphenylphosphinate (*rac*-*syn*-4m)

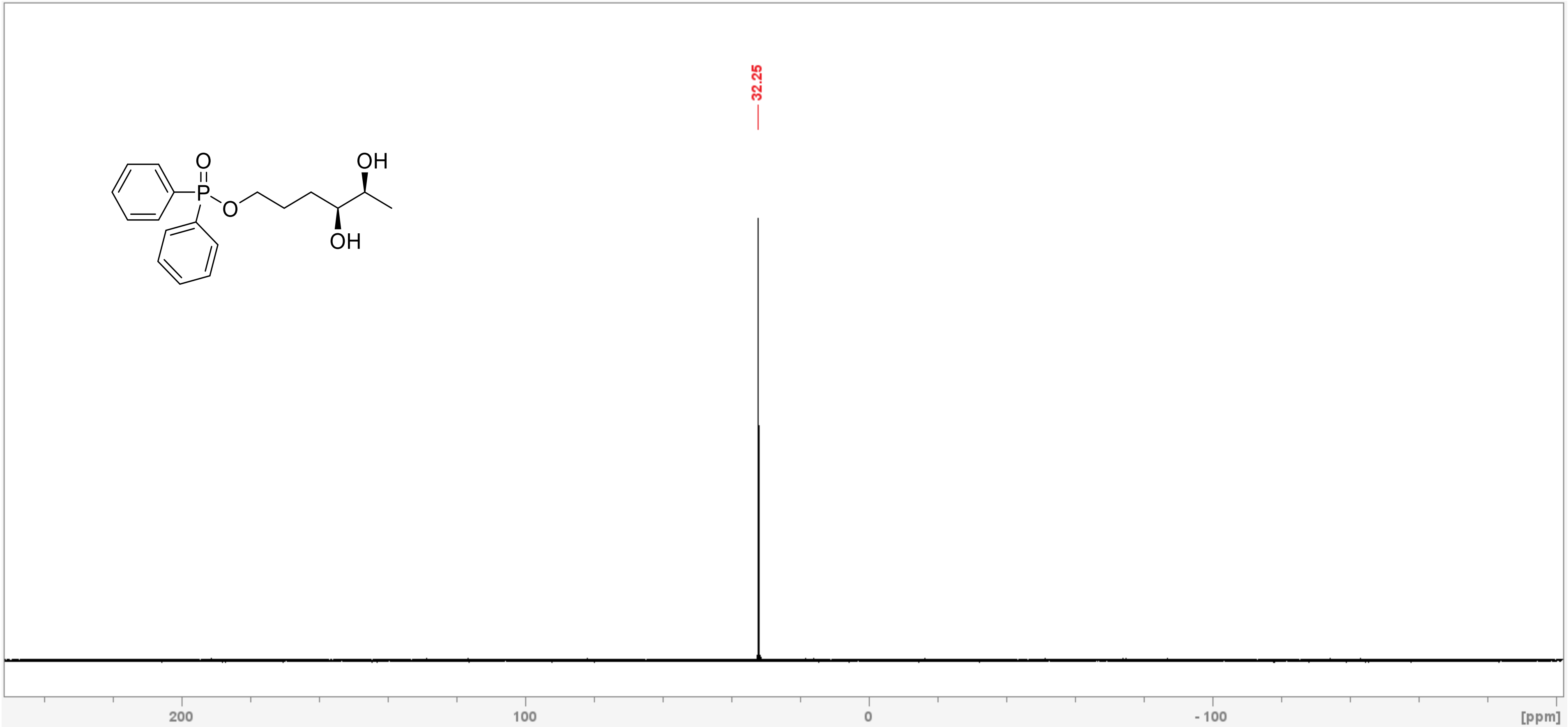

(2*S*,4*S*)-pentane-2,4-diol (1a)

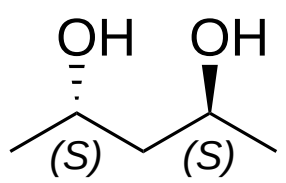

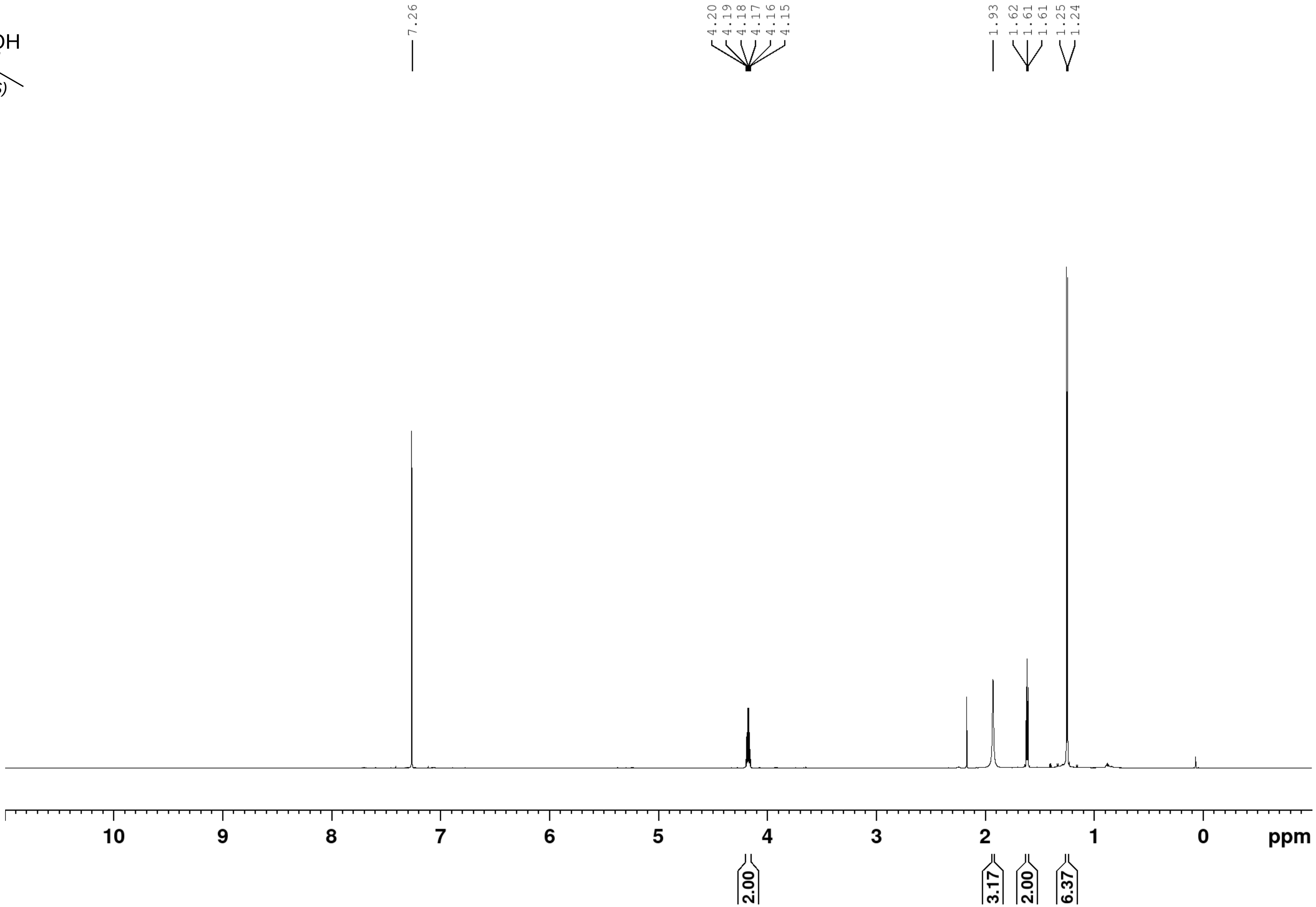

(2*S*,4*S*)-pentane-2,4-diol (1a)

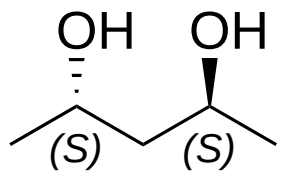

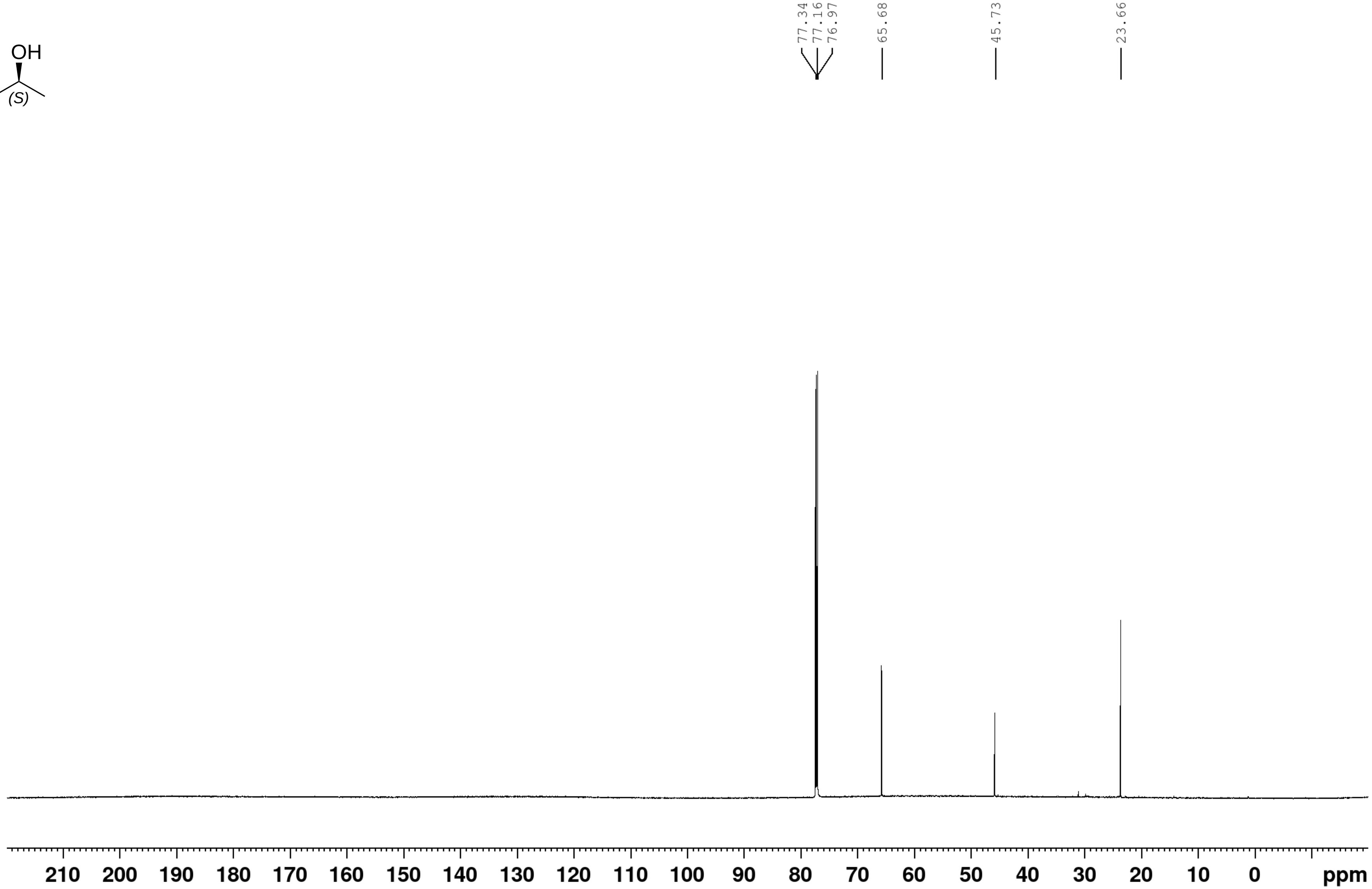

(2*S*,4*S*)-pentane-2,4-diyl dibenzoate (1a-Bz)

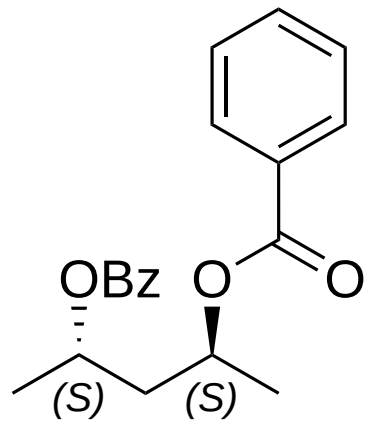

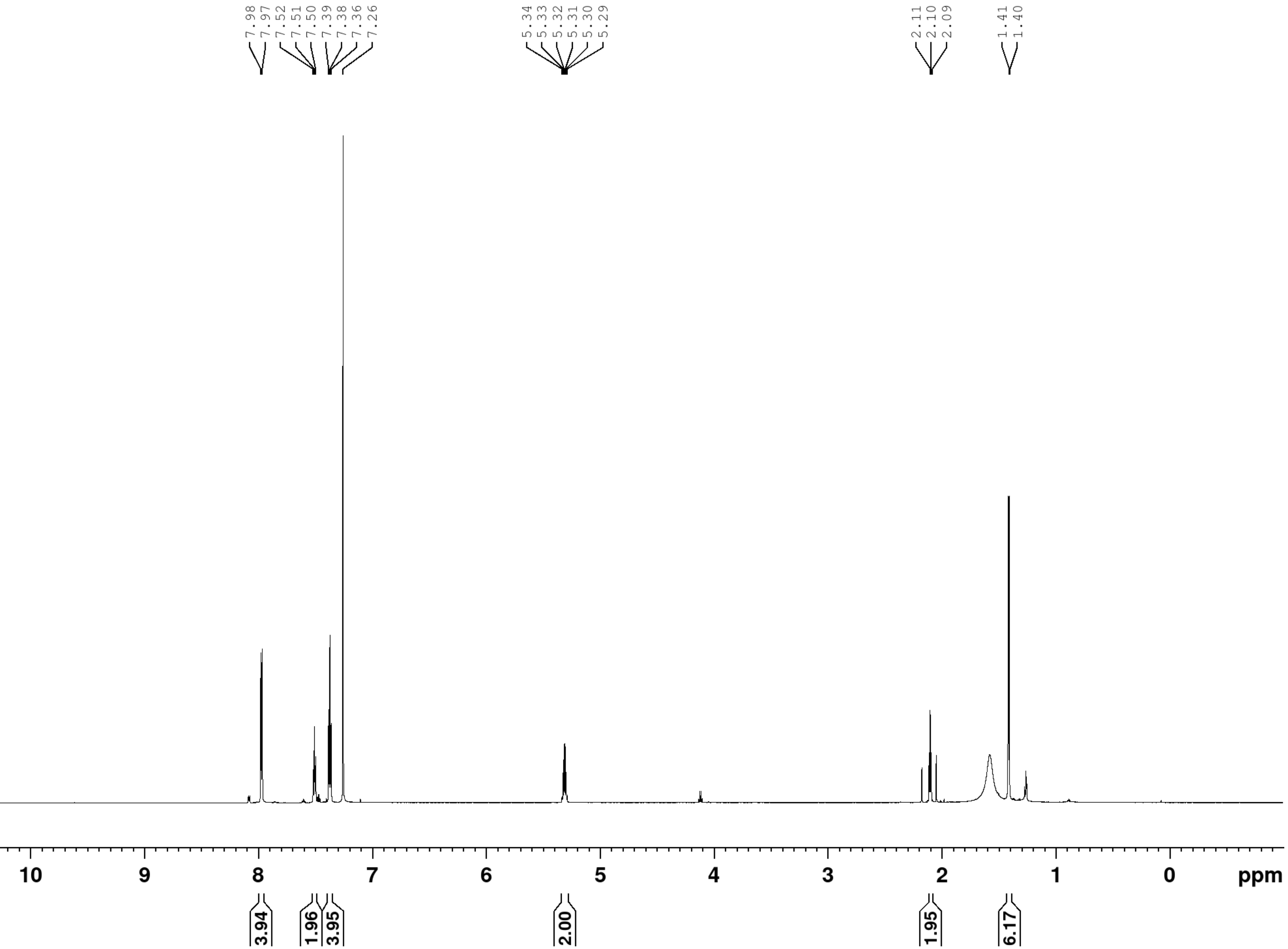

(2*S*,4*S*)-pentane-2,4-diyl dibenzoate (1a-Bz)

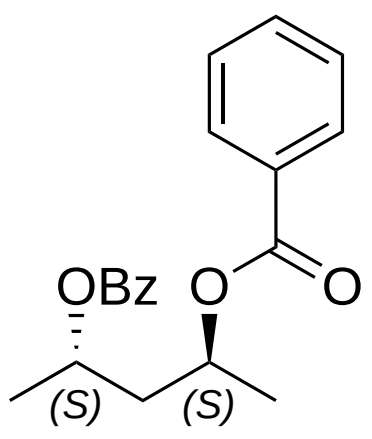

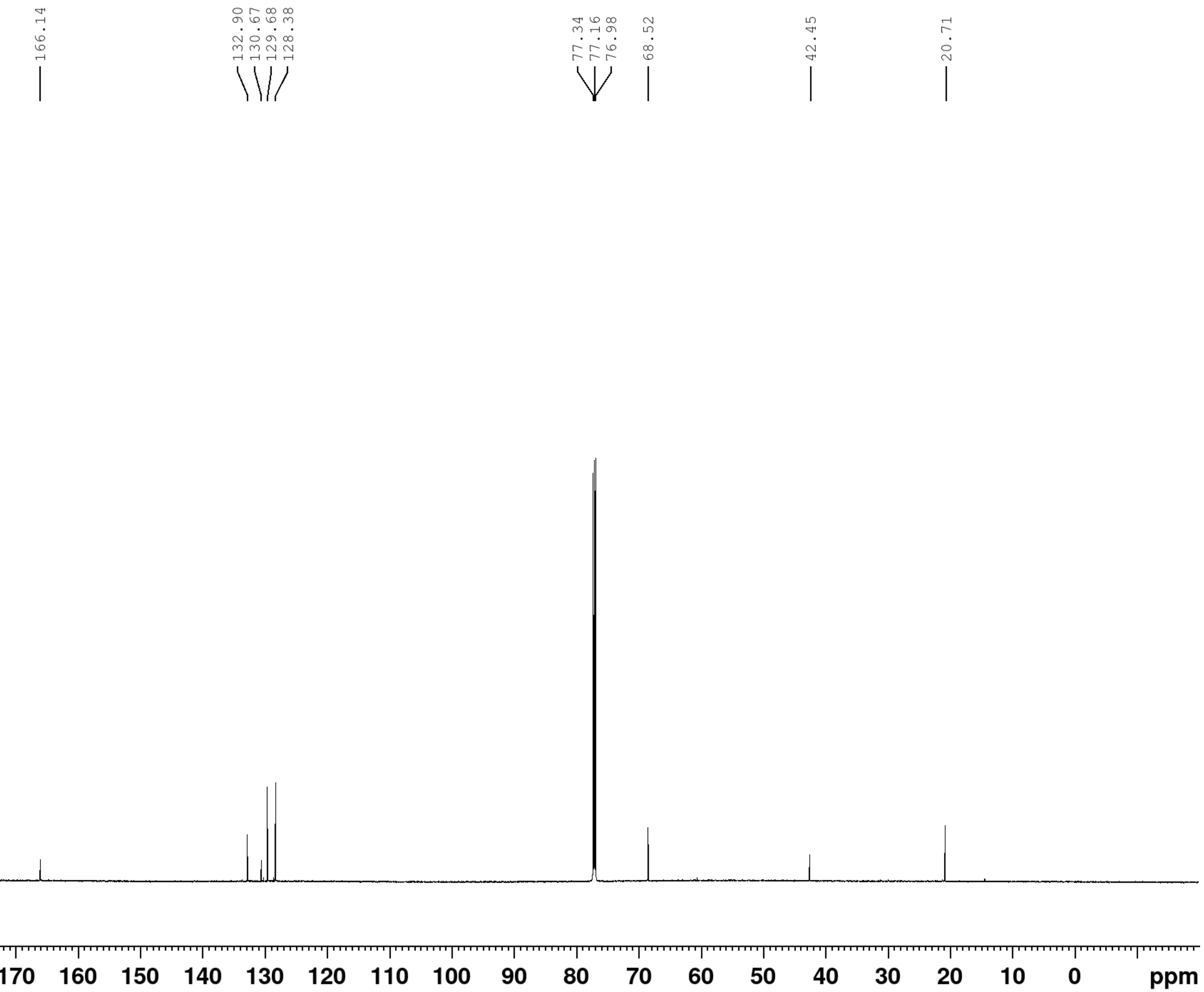

(3S,5S)-heptane-3,5-diol (1b)

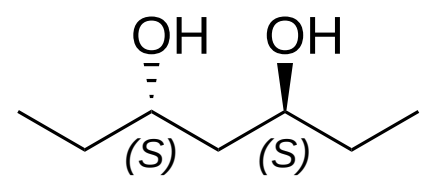

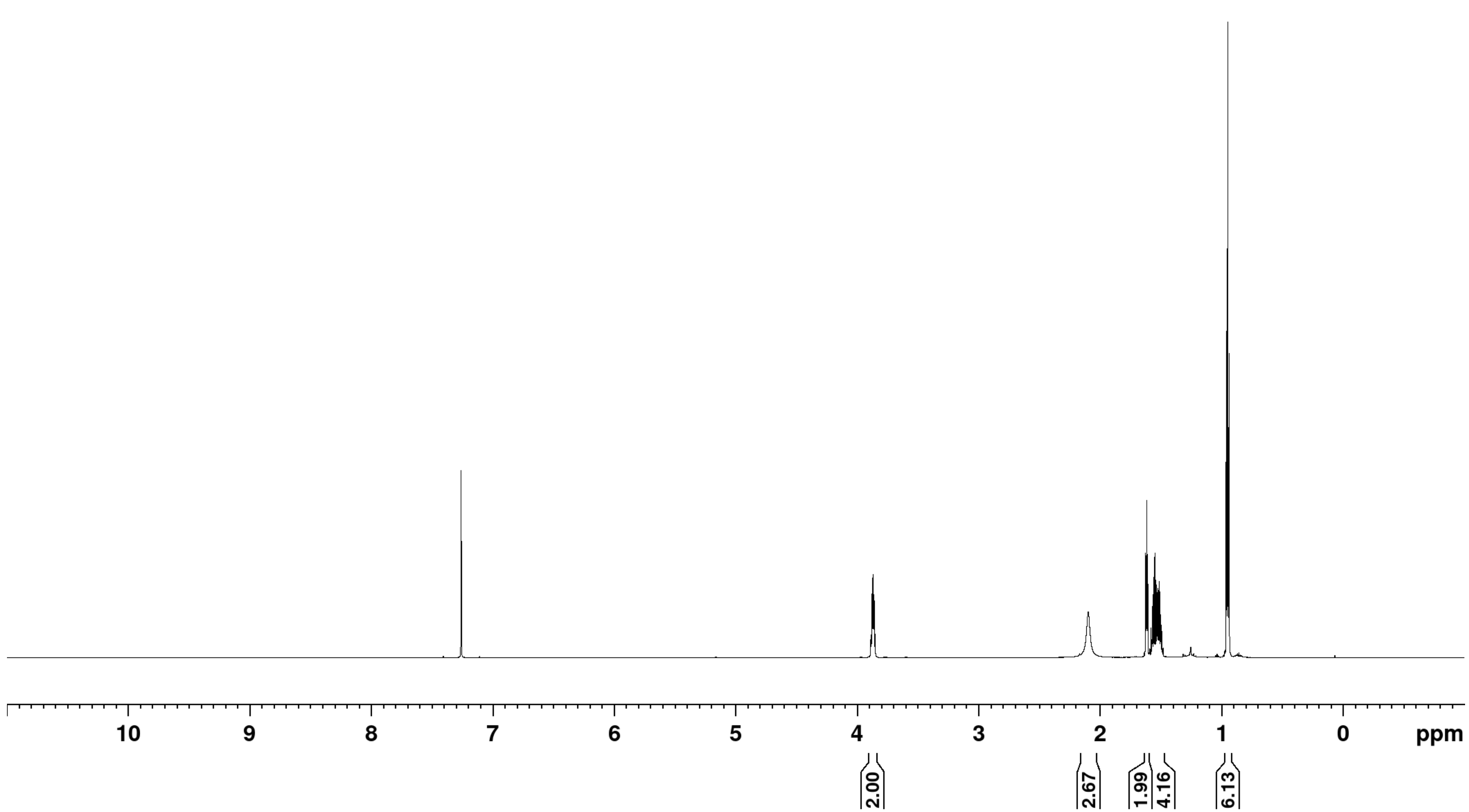

(3S,5S)-heptane-3,5-diol (1b)

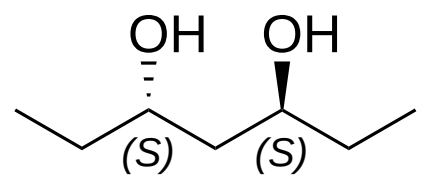

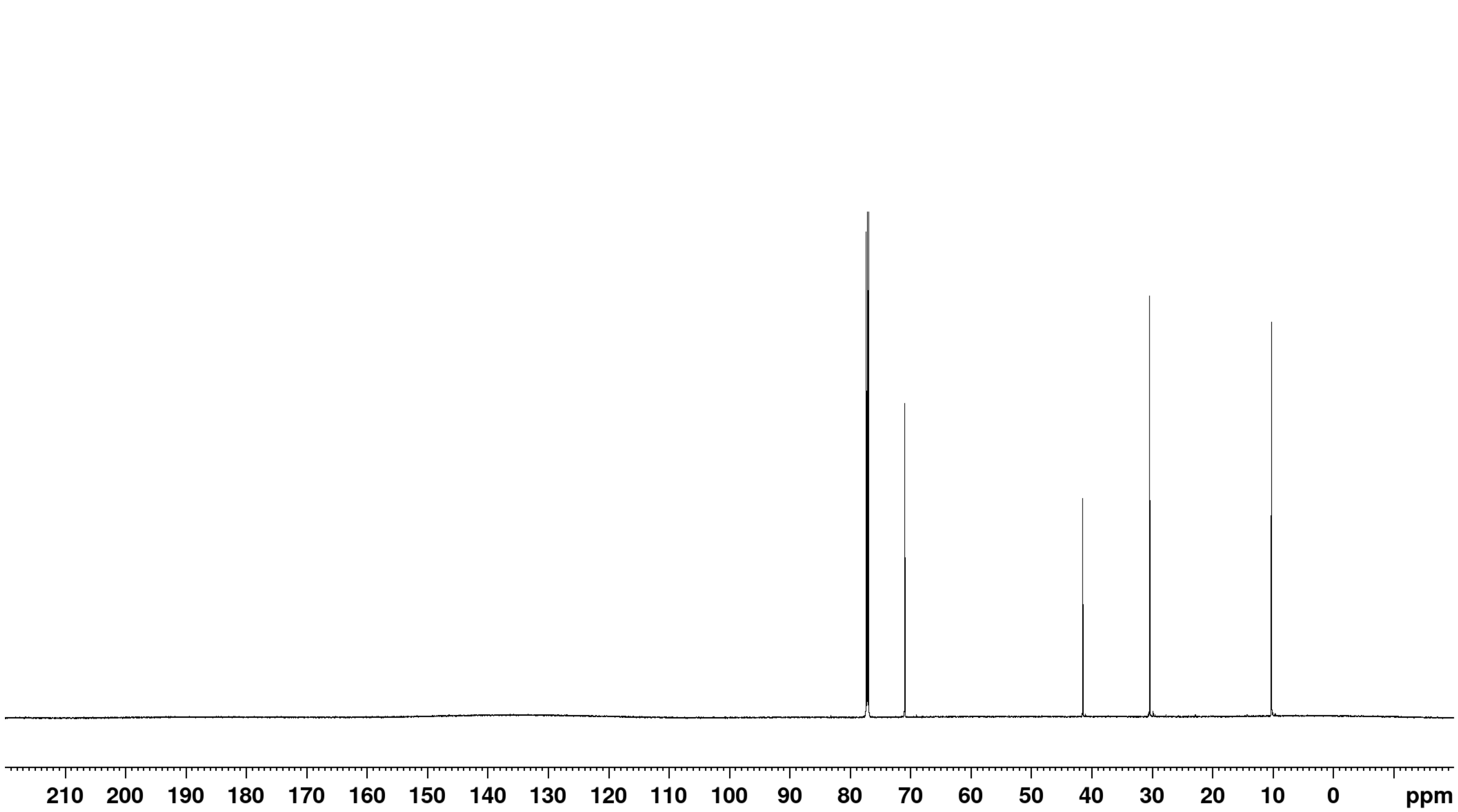

(3S,5S)-heptane-3,5-diyl dibenzoate (1b-Bz)

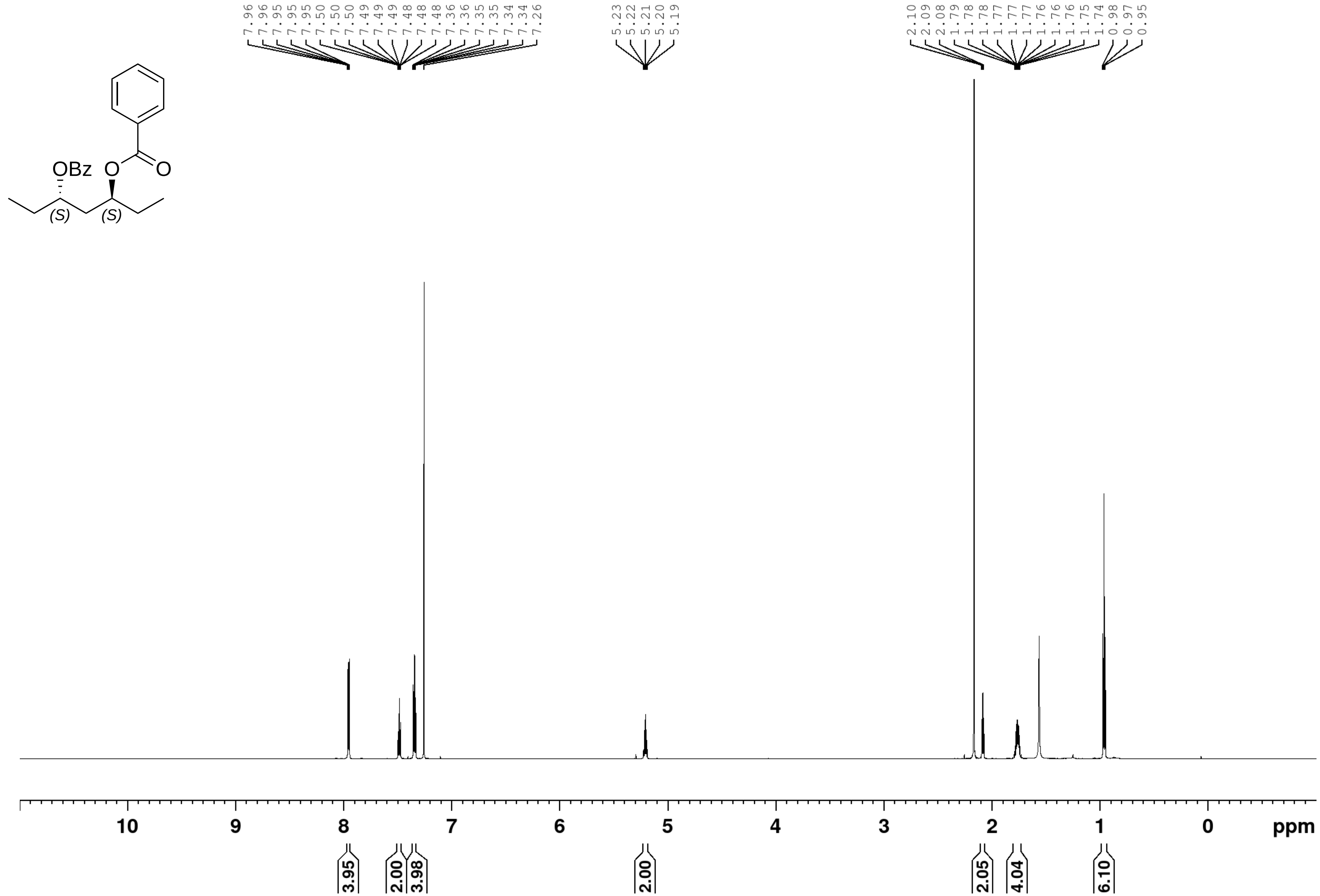

(3S,5S)-heptane-3,5-diyl dibenzoate (1b-Bz)

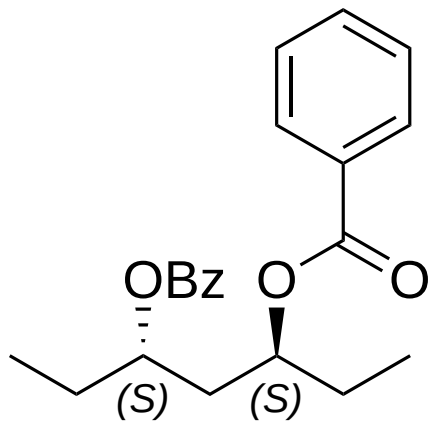

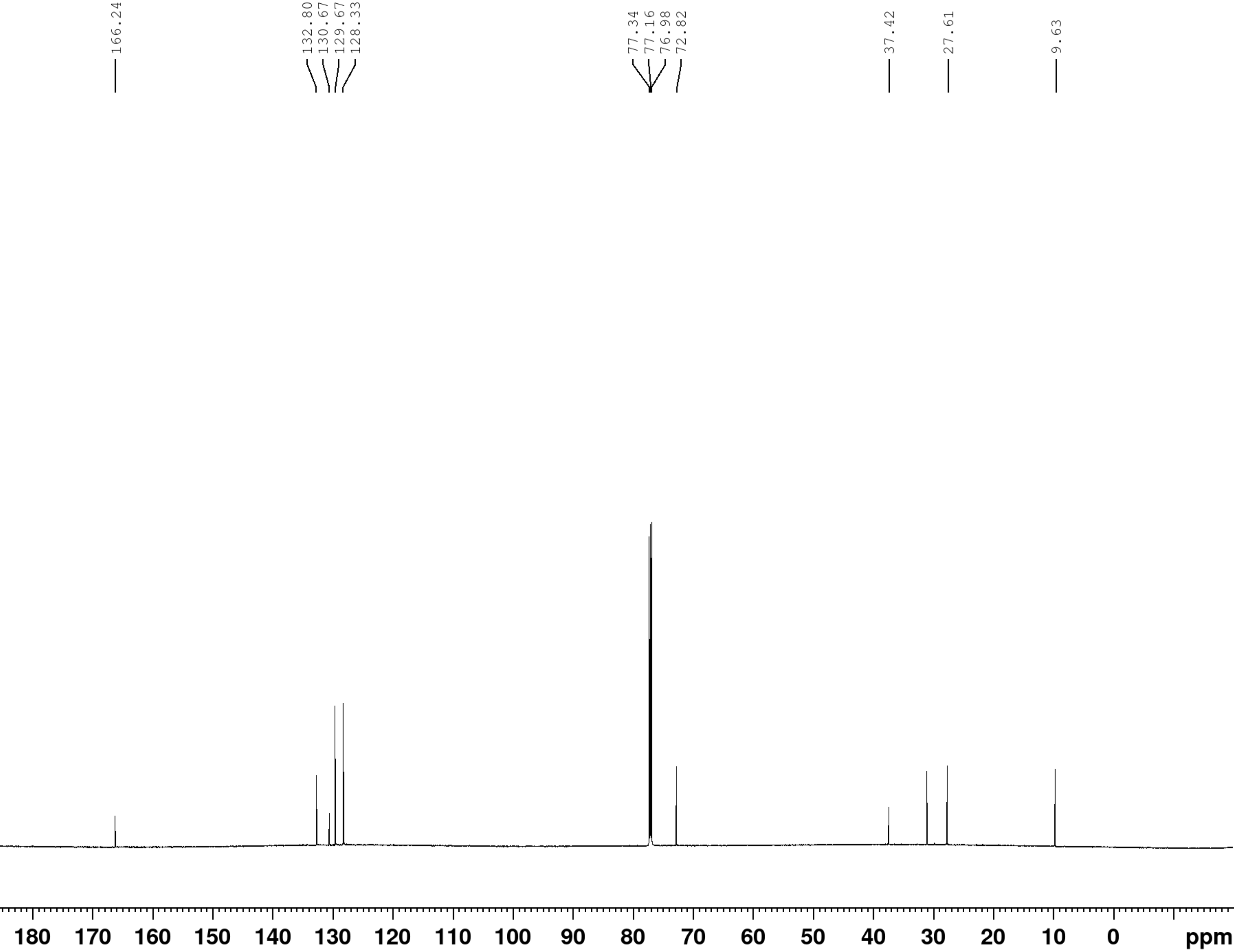

(4*S*,6*S*)-nonane-4,6-diol (1c)

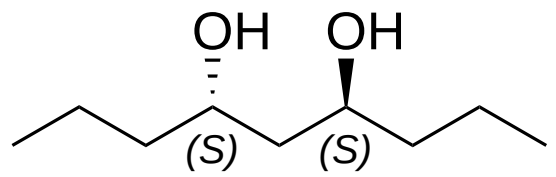

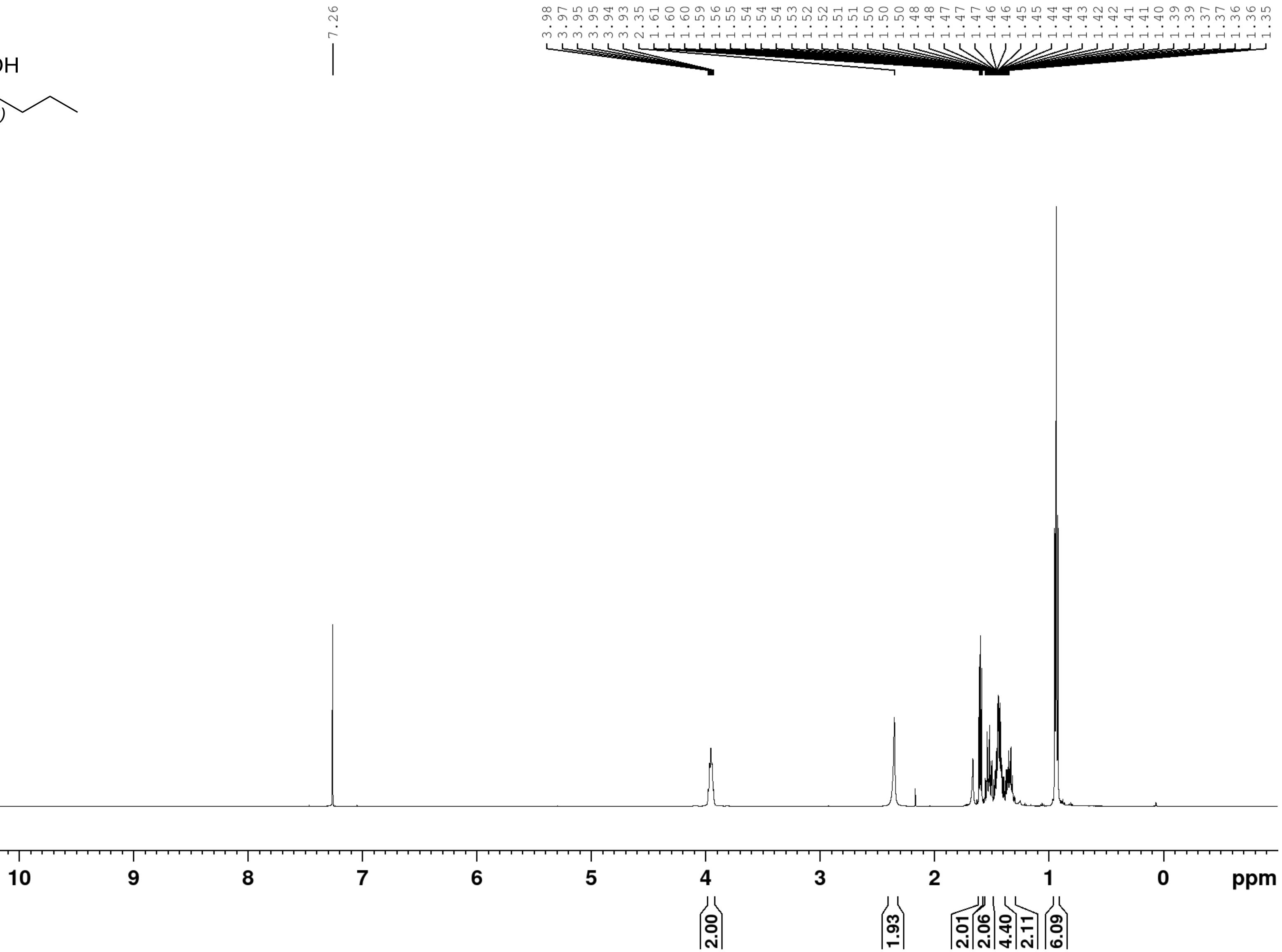

(4*S*,6*S*)-nonane-4,6-diol (1c)

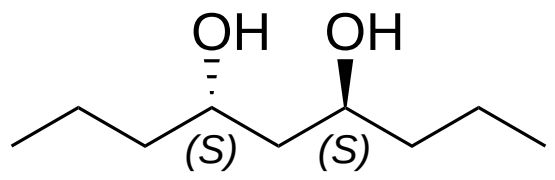

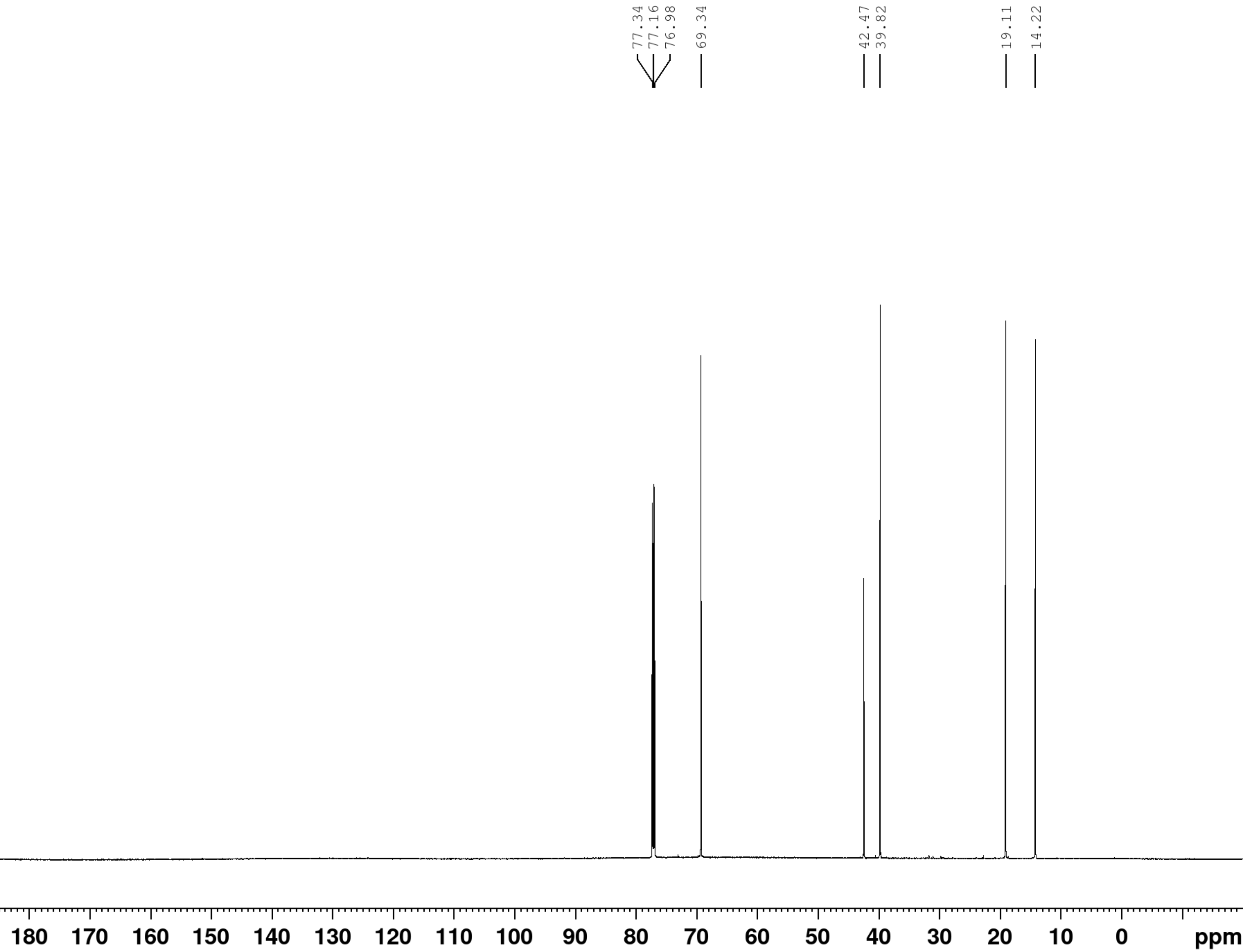

(4S,6S)-nonane-4,6-diyl dibenzoate (1c-Bz)

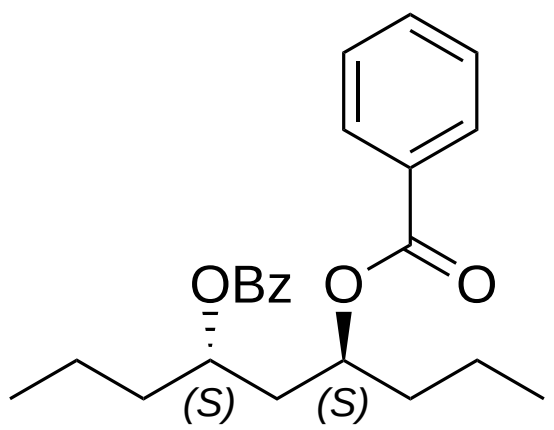

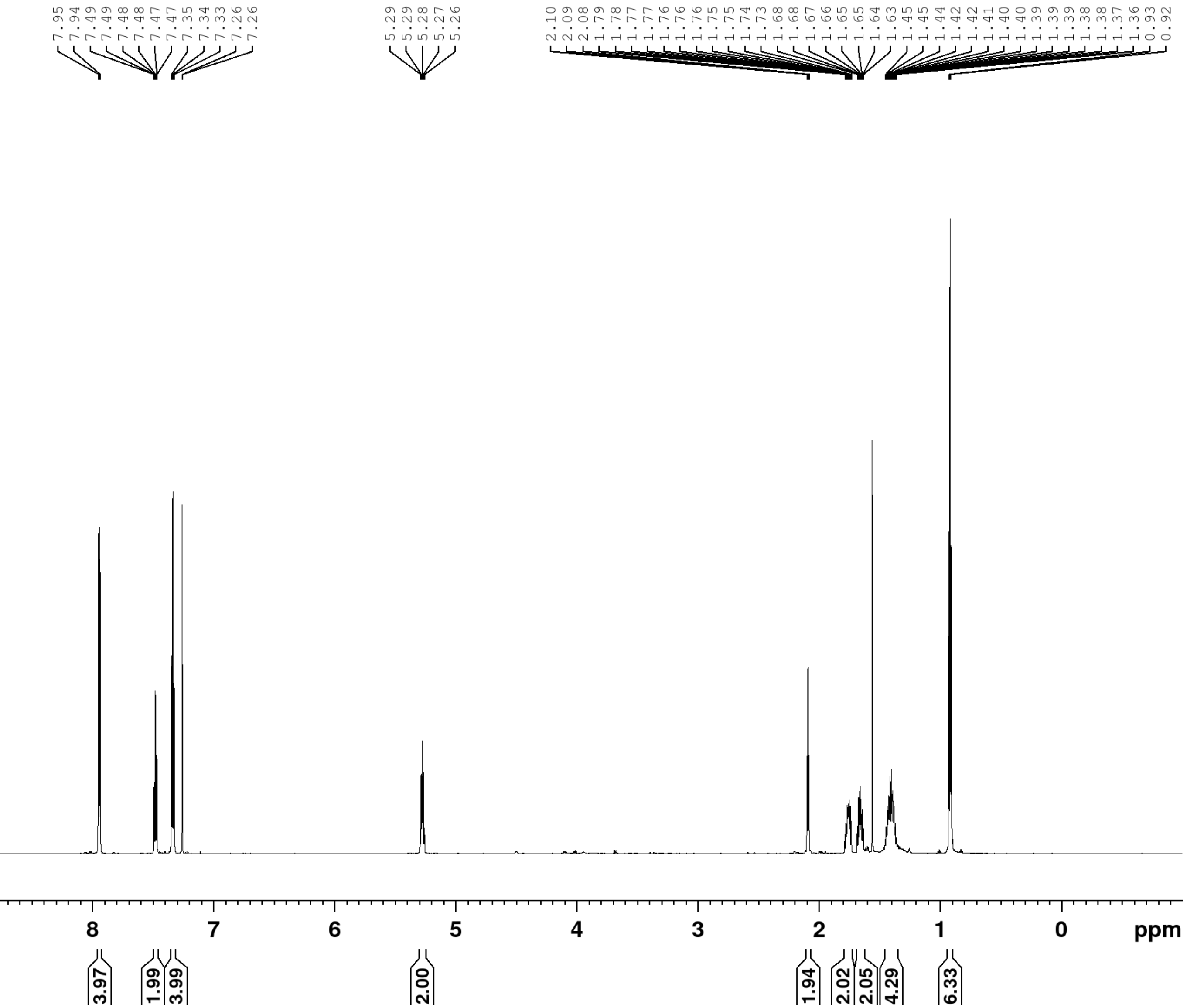

(4S,6S)-nonane-4,6-diyl dibenzoate (1c-Bz)

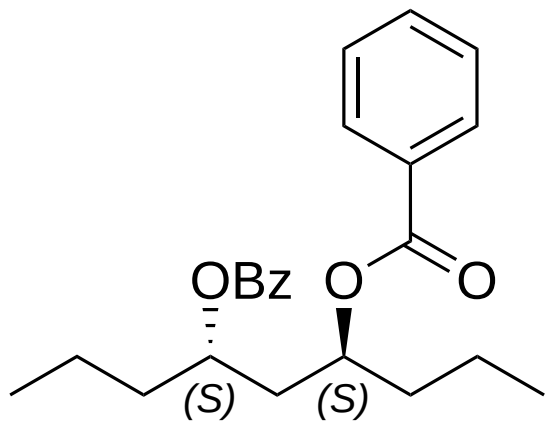

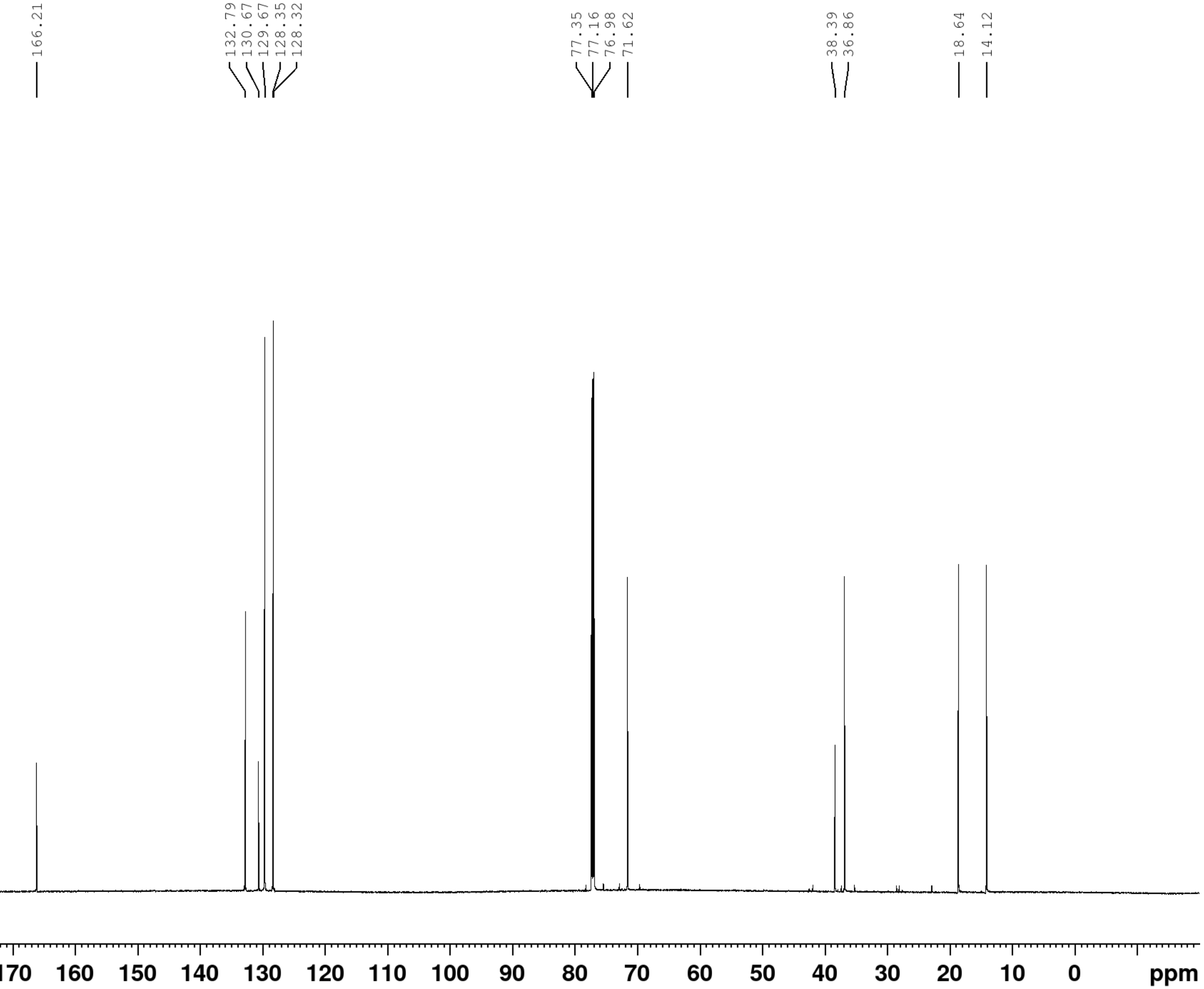

(5S,7S)-undecane-5,7-diol (1d)

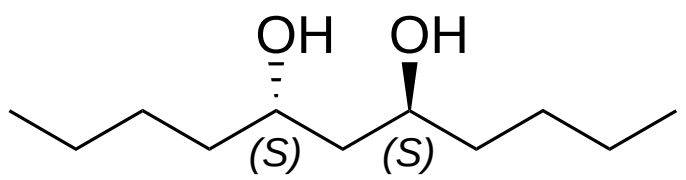

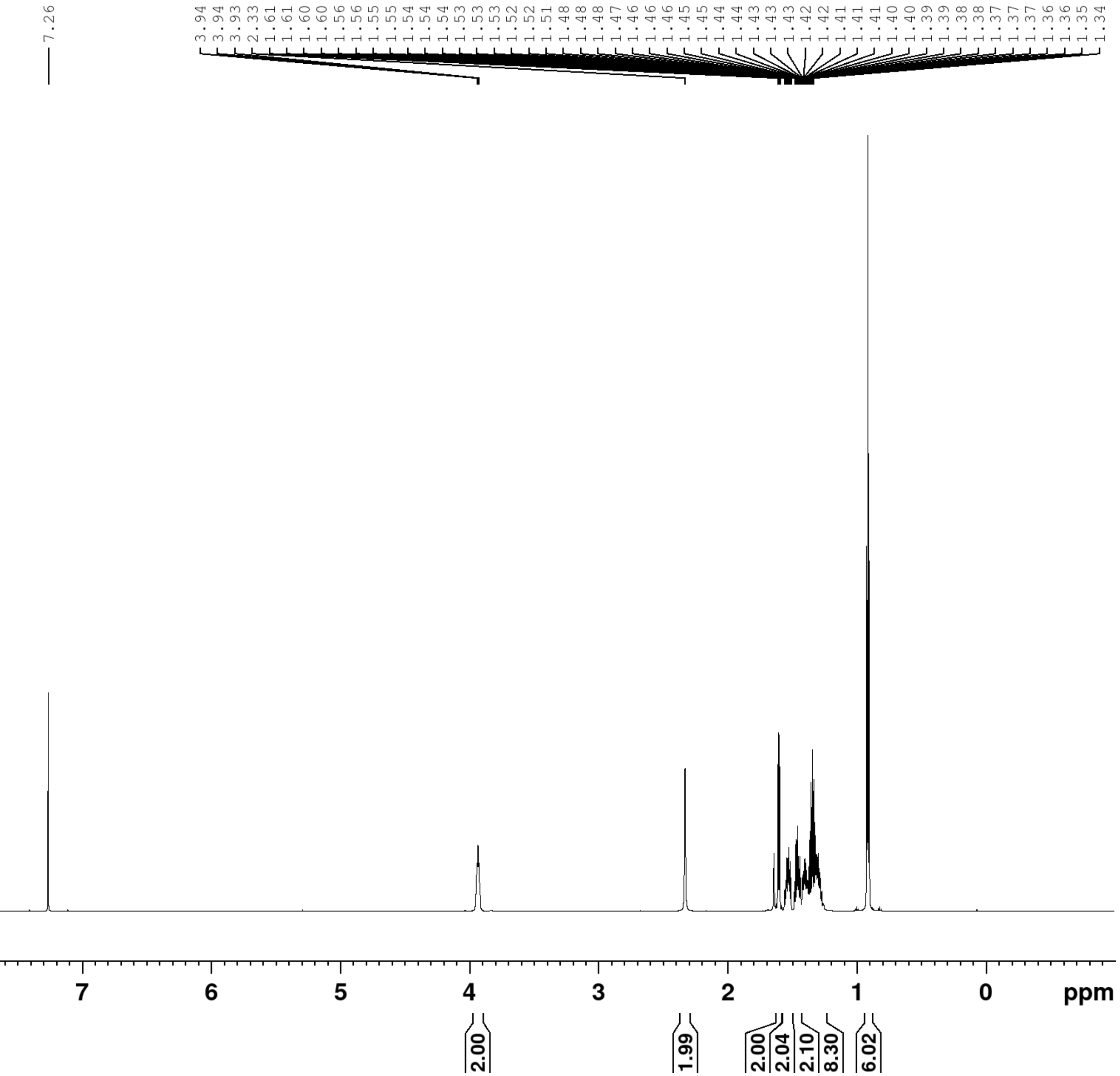

(5S,7S)-undecane-5,7-diol (1d)

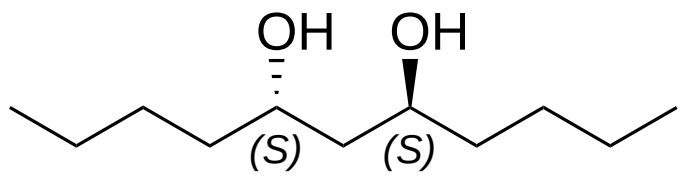

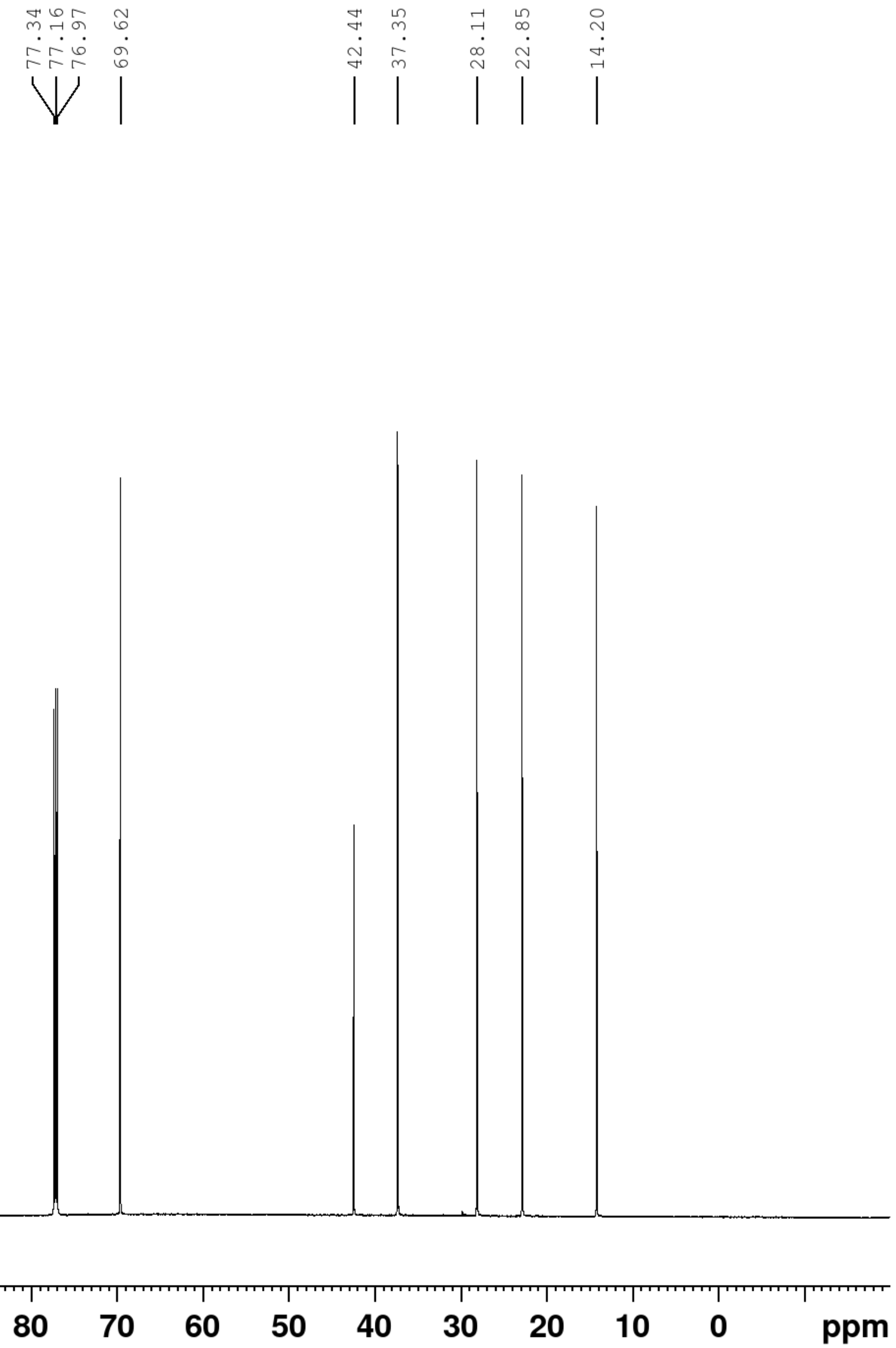

(5S,7S)-undecane-5,7-diyl dibenzoate (1d-Bz)

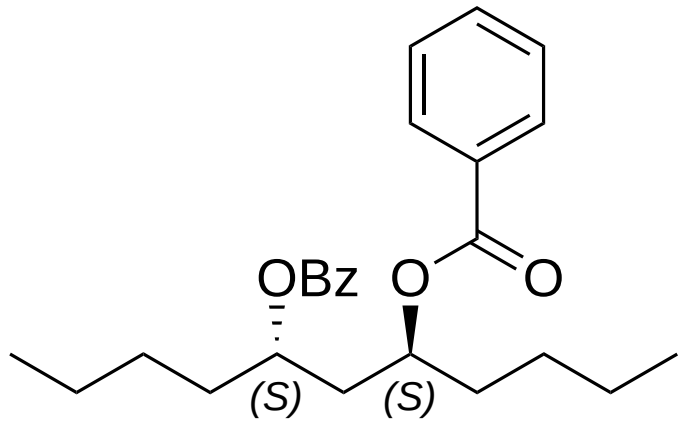

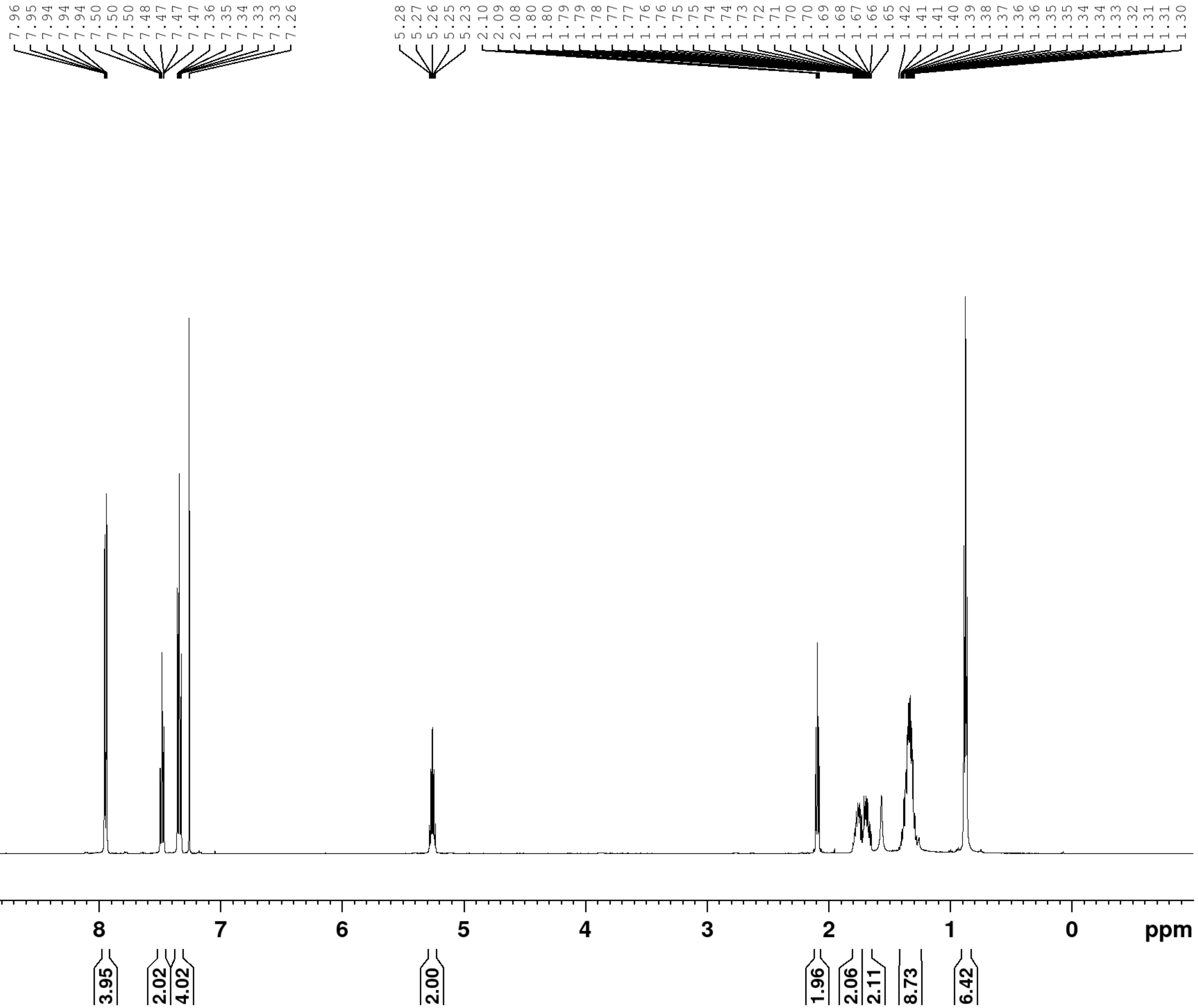

(5S,7S)-undecane-5,7-diyl dibenzoate (1d-Bz)

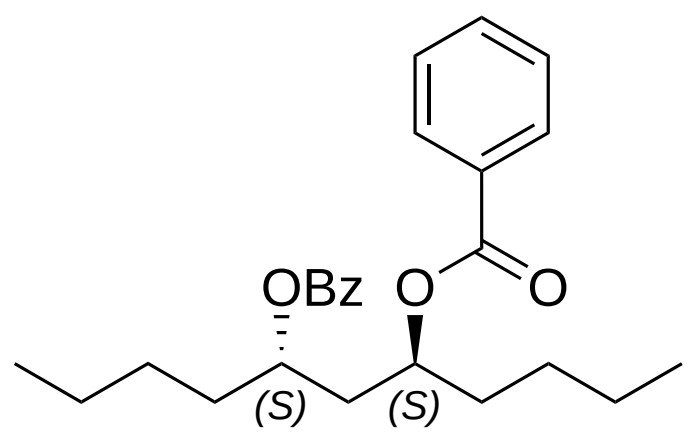

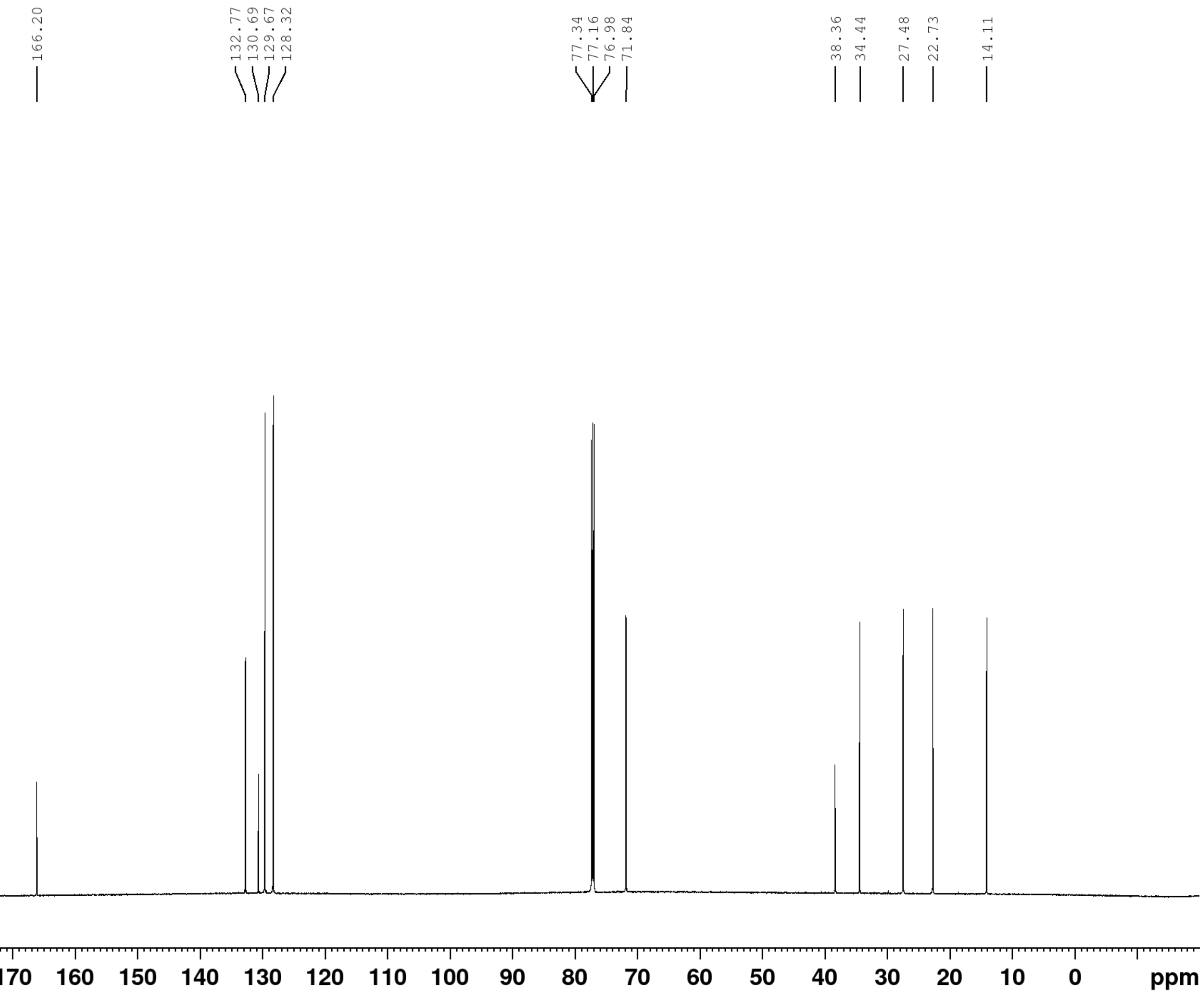

(4S,6S)-2,8-dimethylnonane-4,6-diol (1e)

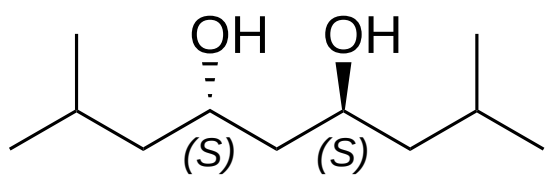

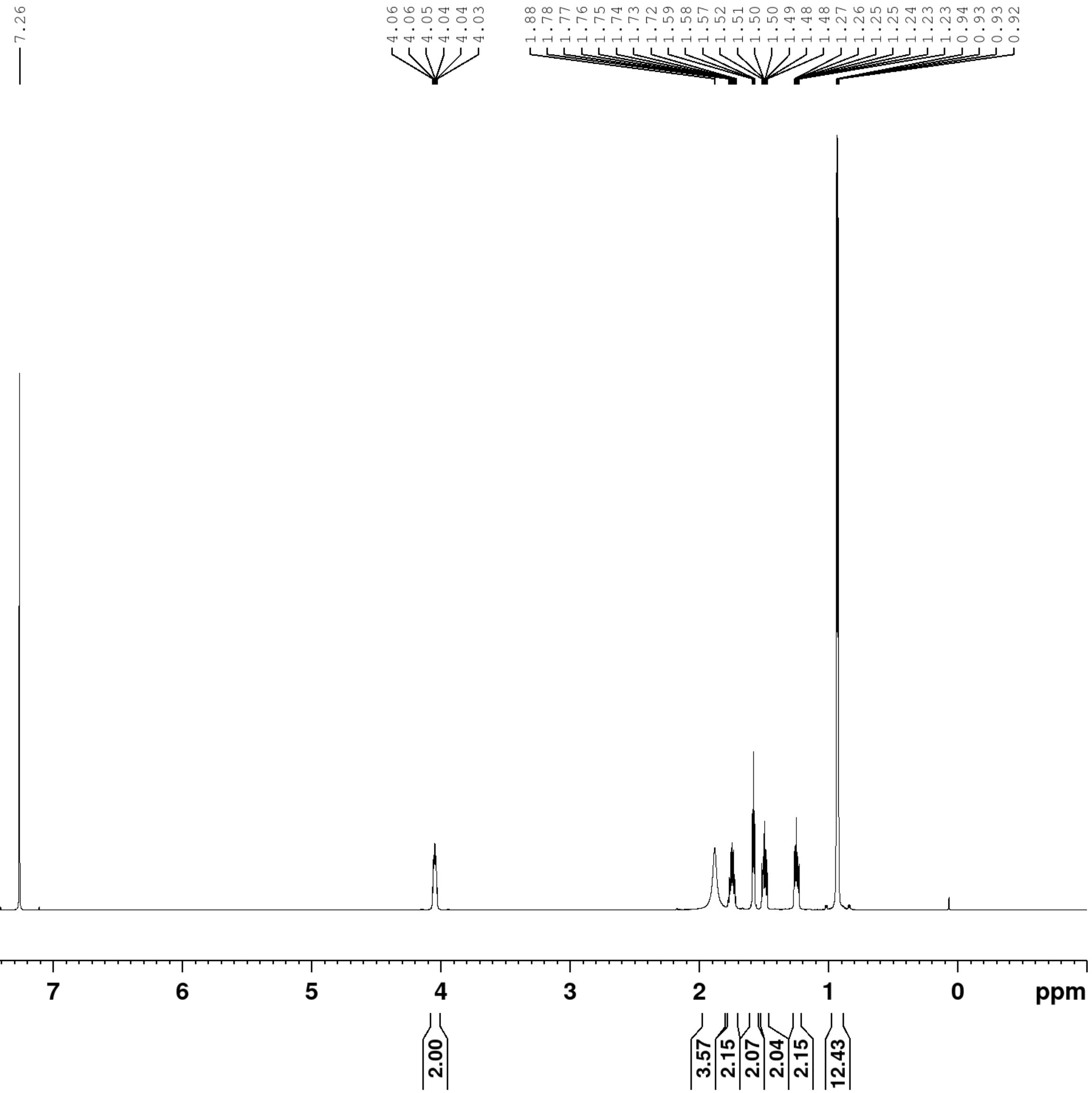

(4S,6S)-2,8-dimethylnonane-4,6-diol (1e)

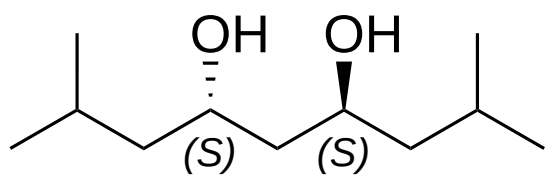

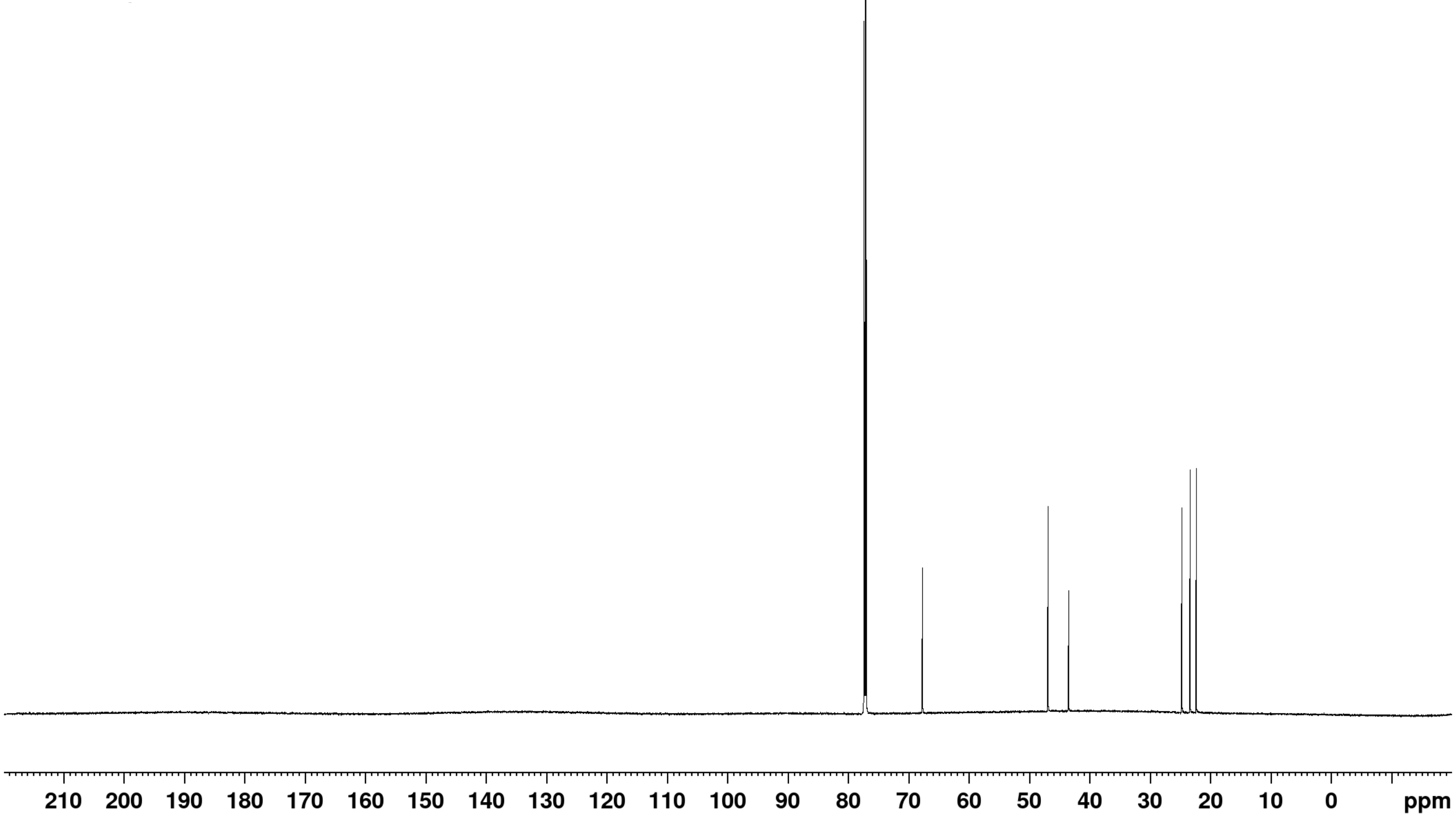

(4*S*,6*S*)-2,8-dimethylnonane-4,6-diyl dibenzoate (1e-Bz)

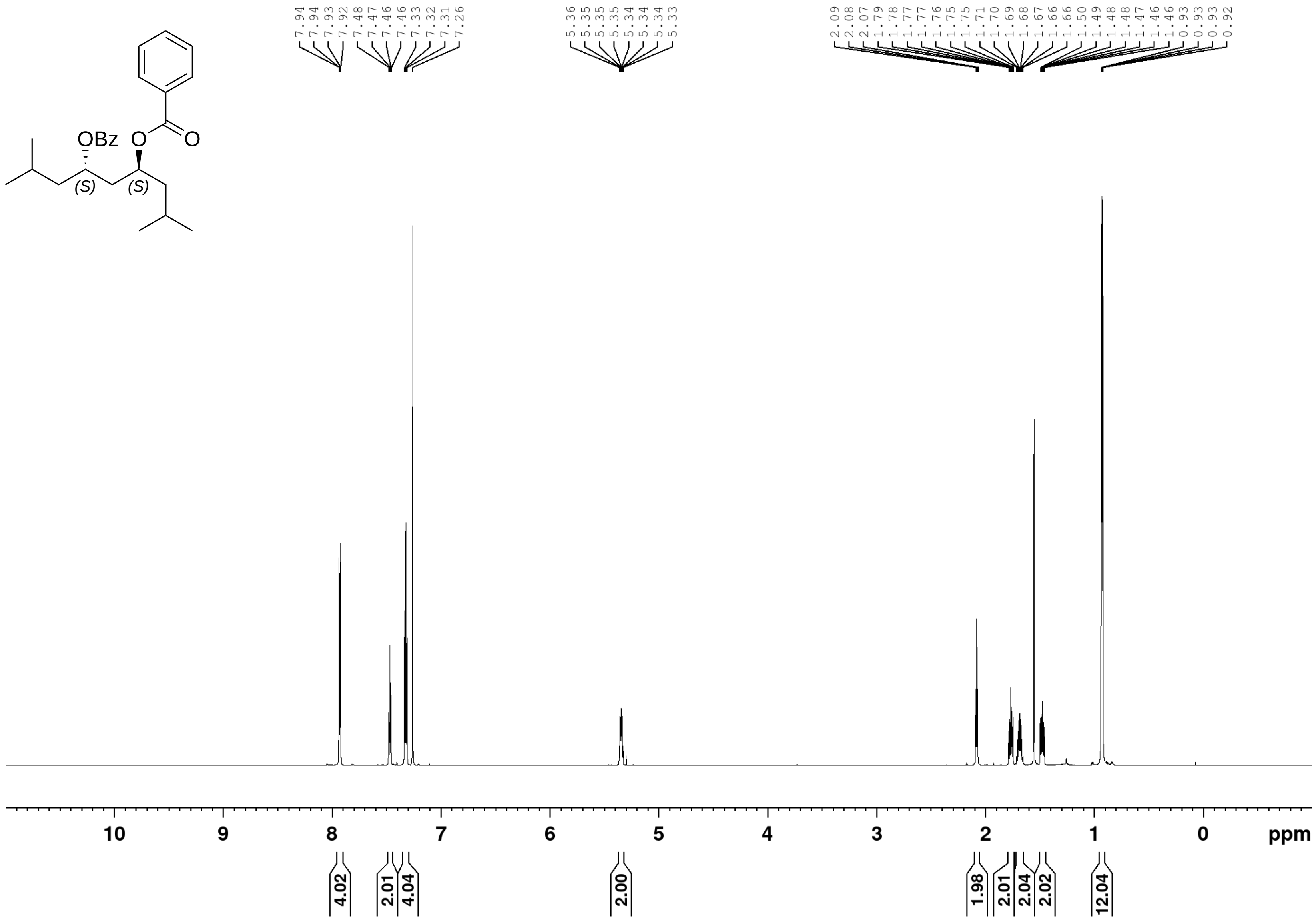

(4*S*,6*S*)-2,8-dimethylnonane-4,6-diyl dibenzoate (1e-Bz)

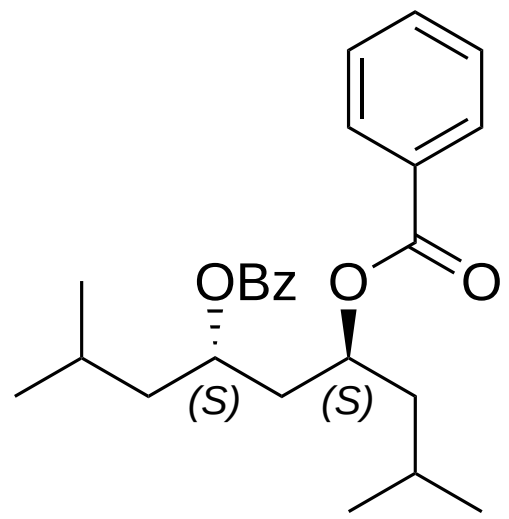

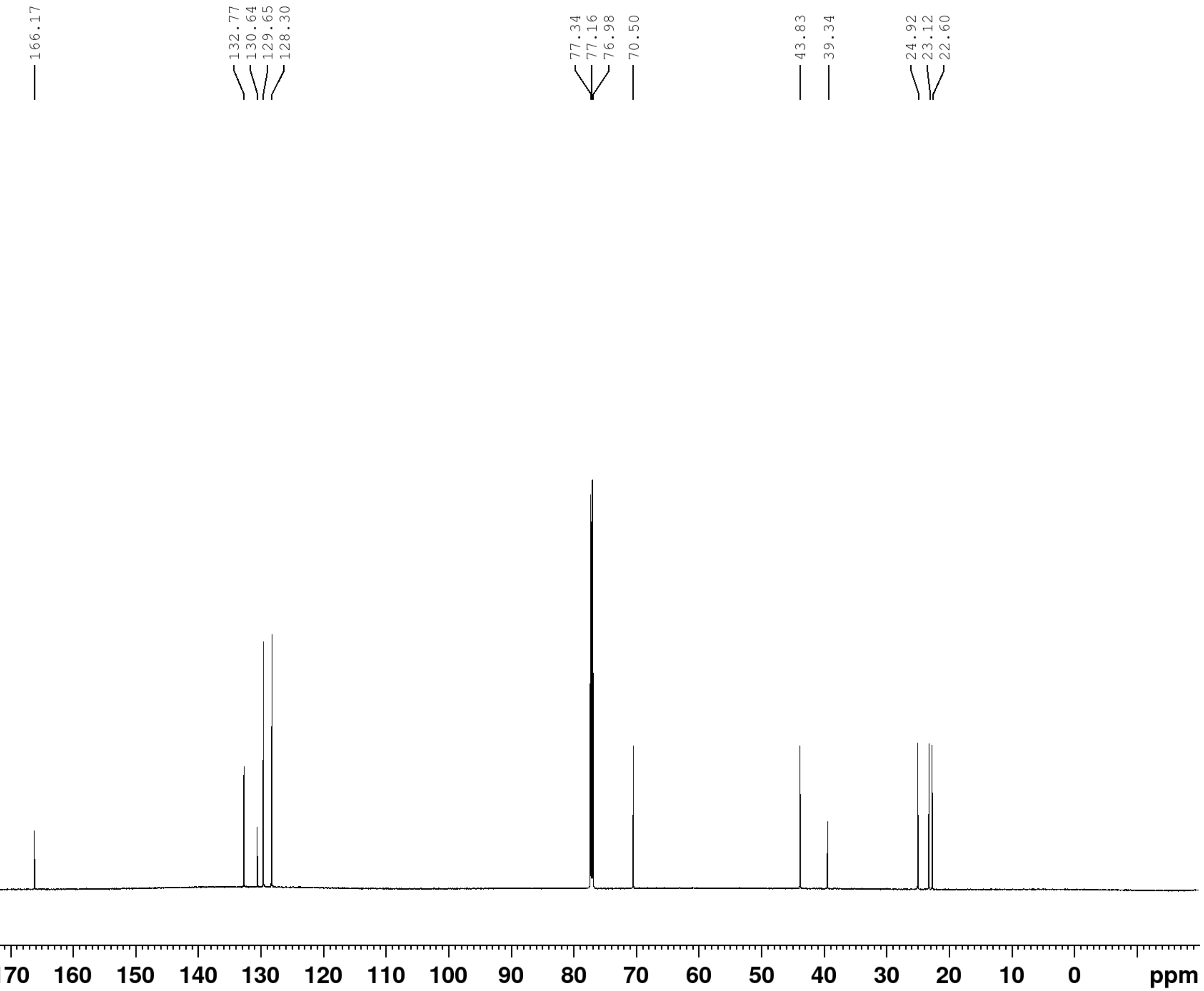

(5S,7S)-2,10-dimethylundecane-5,7-diol (1f)

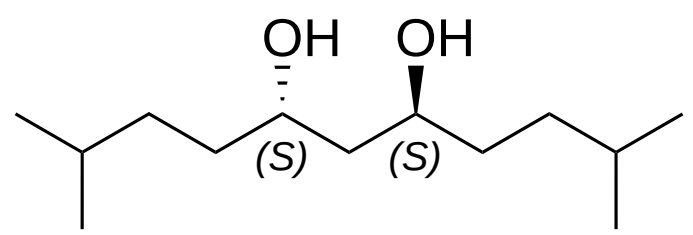

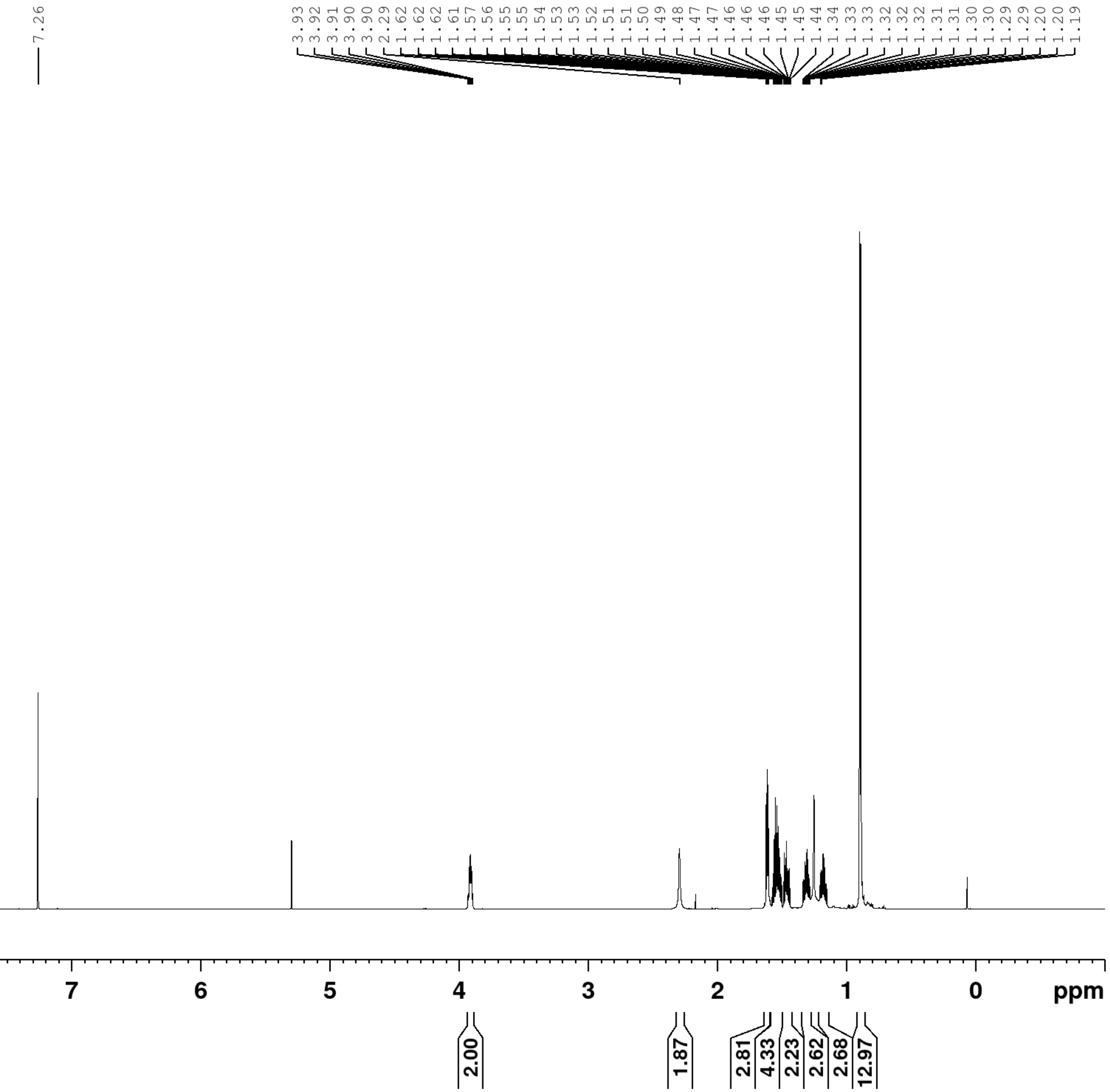

(5S,7S)-2,10-dimethylundecane-5,7-diol (1f)

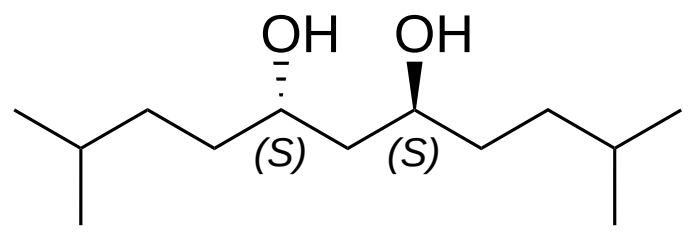

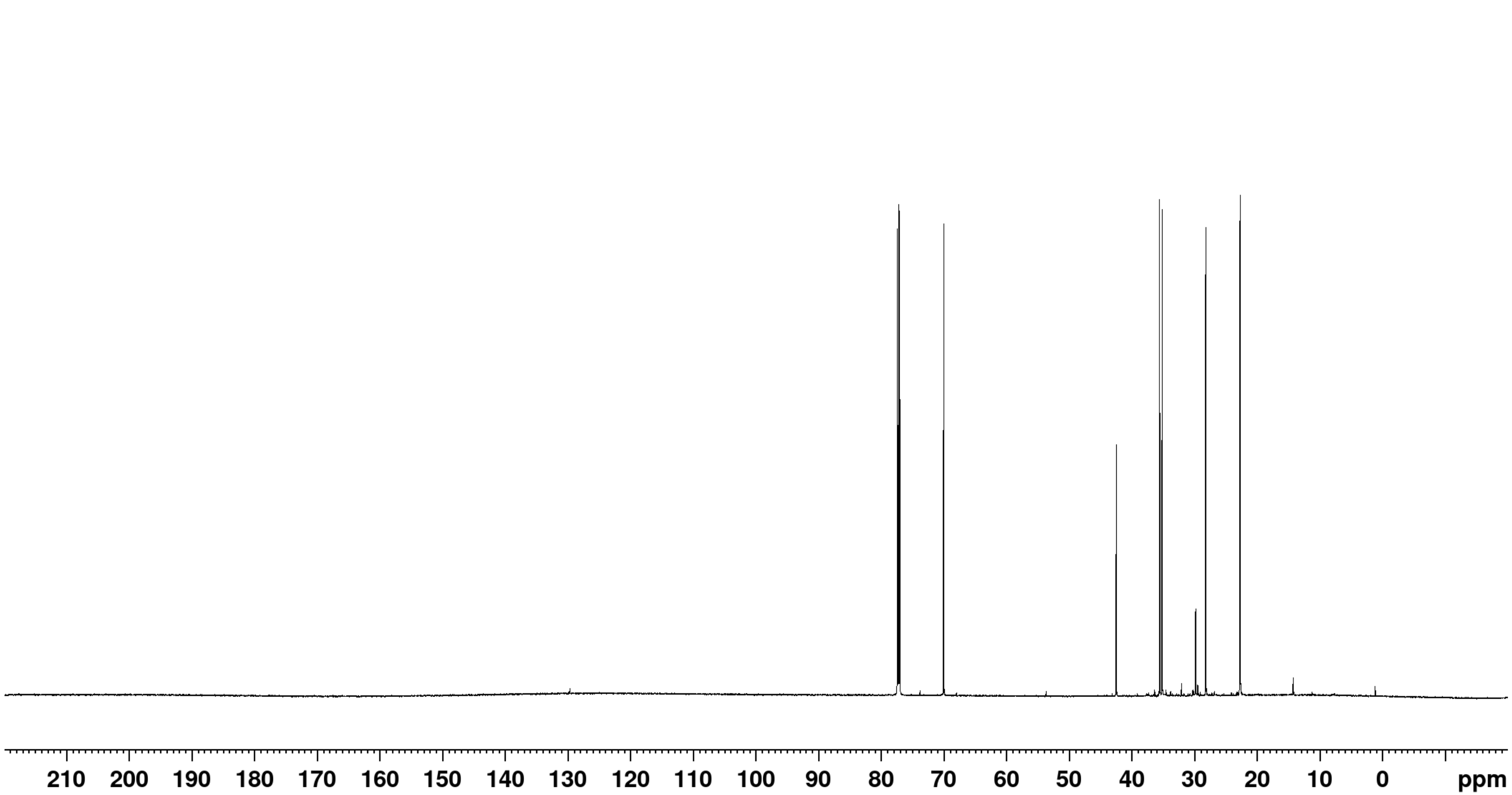

(5*S*,7*S*)-2,10-dimethylundecane-5,7-diyl dibenzoate (1f-Bz)

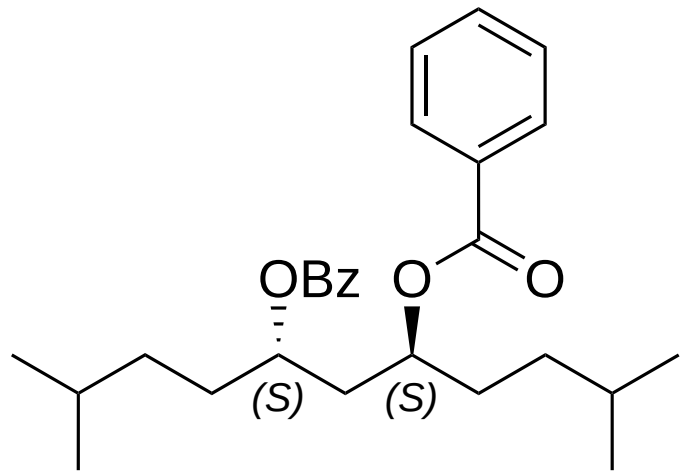

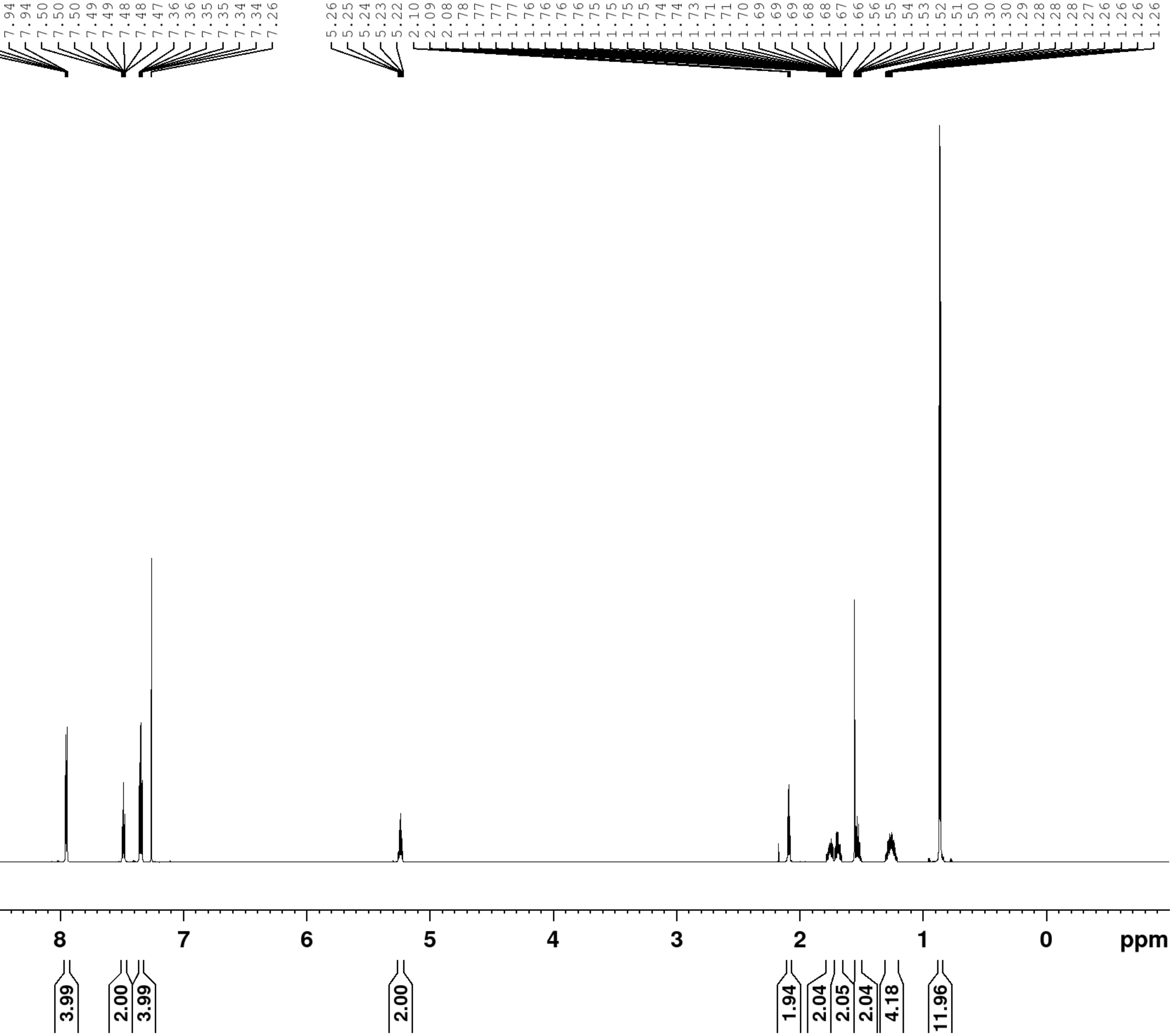

(5*S*,7*S*)-2,10-dimethylundecane-5,7-diyl dibenzoate (1f-Bz)

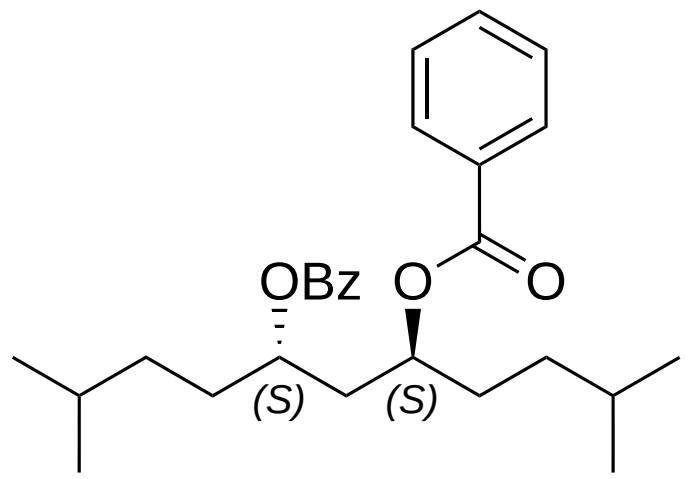

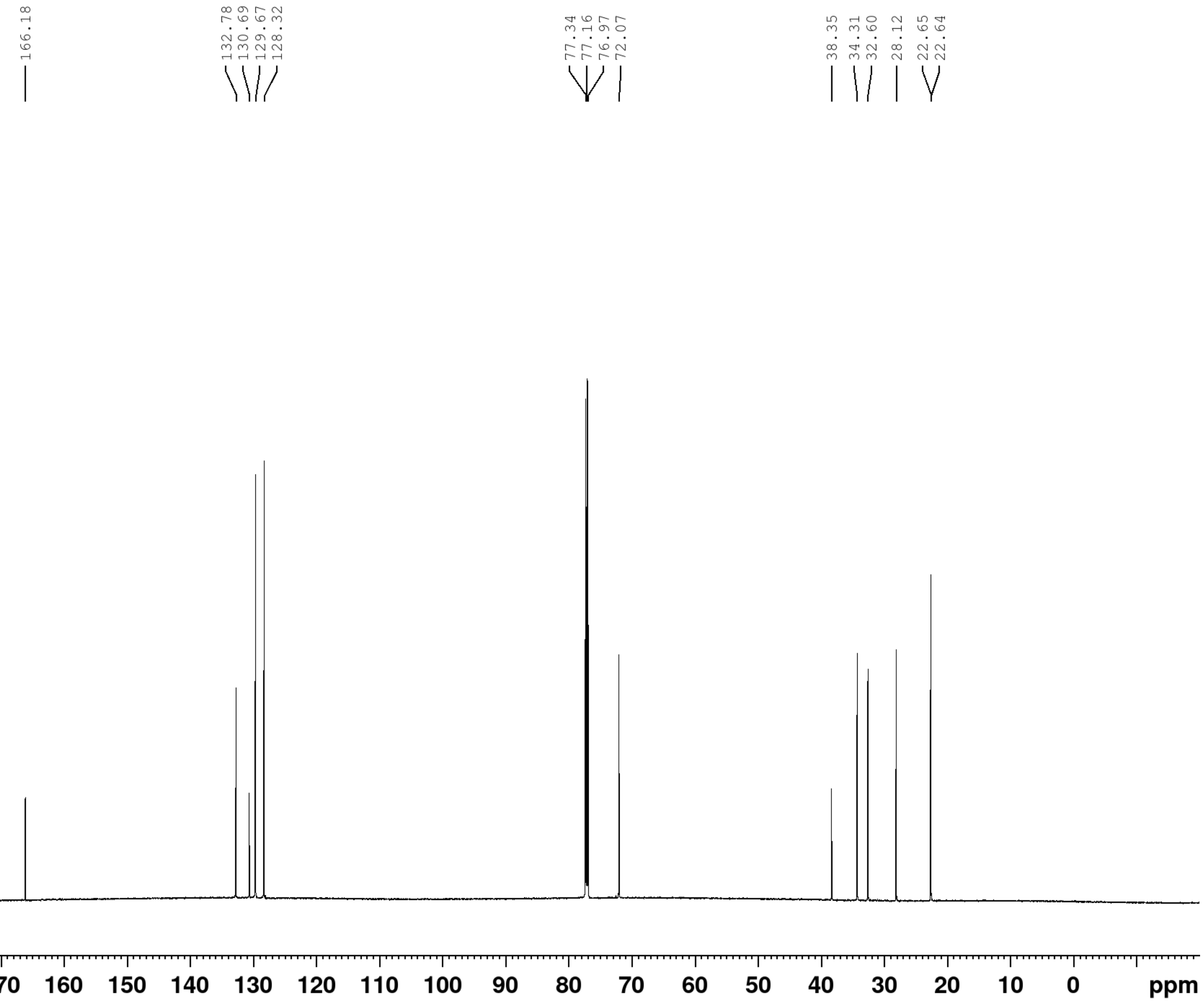

(3S,5S)-1,7-dicyclobutylheptane-3,5-diol (1g)

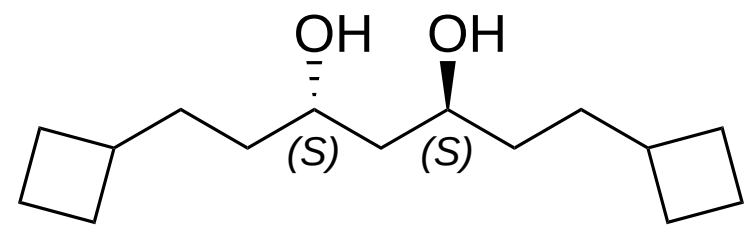

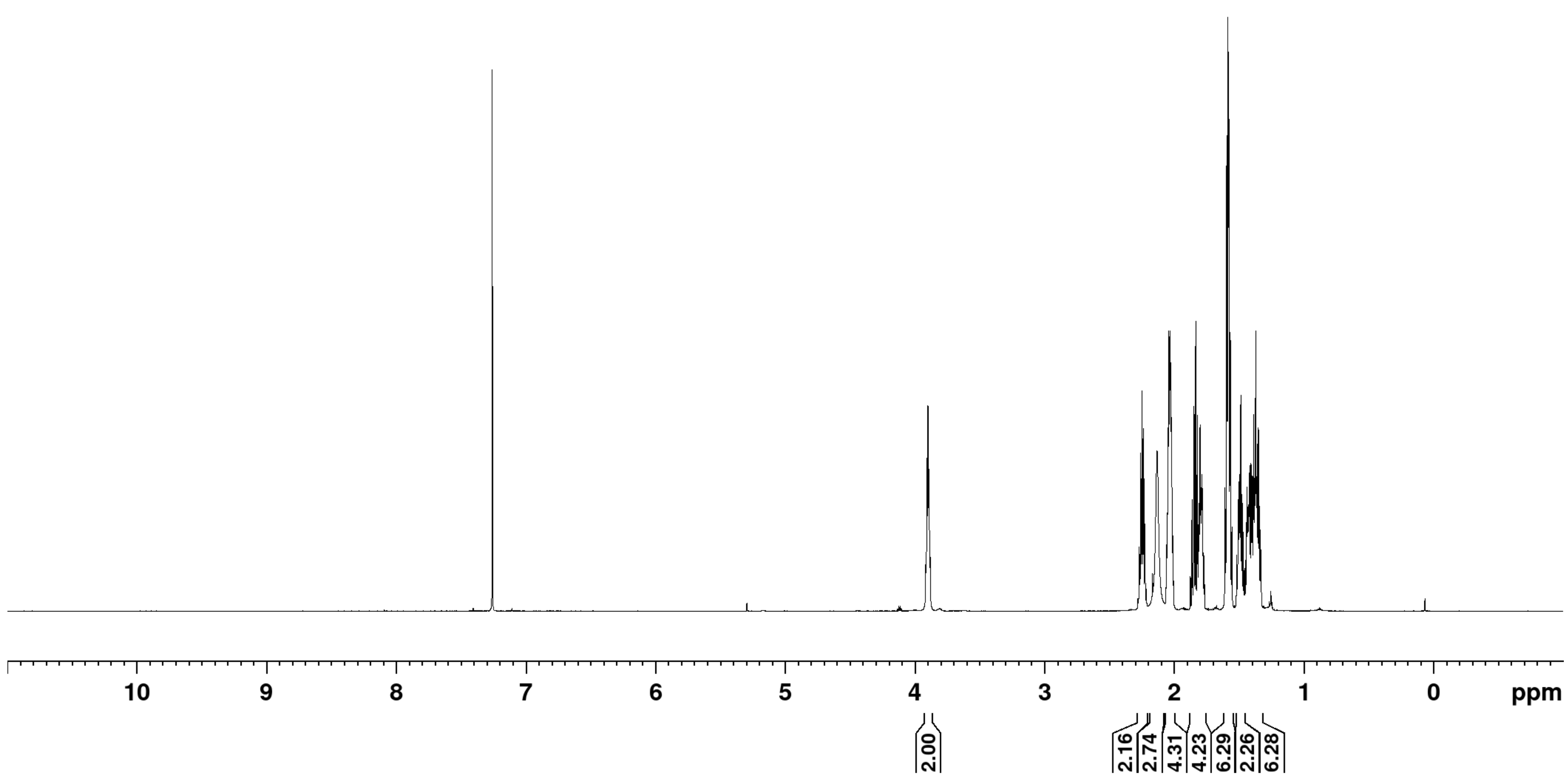

(3S,5S)-1,7-dicyclobutylheptane-3,5-diol (1g)

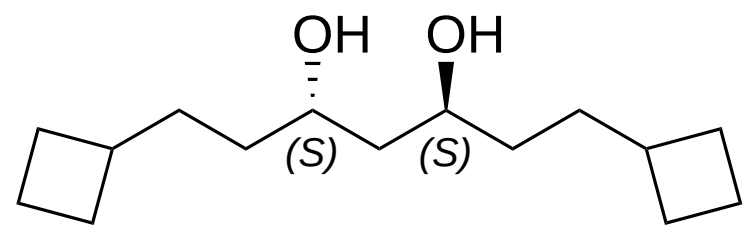

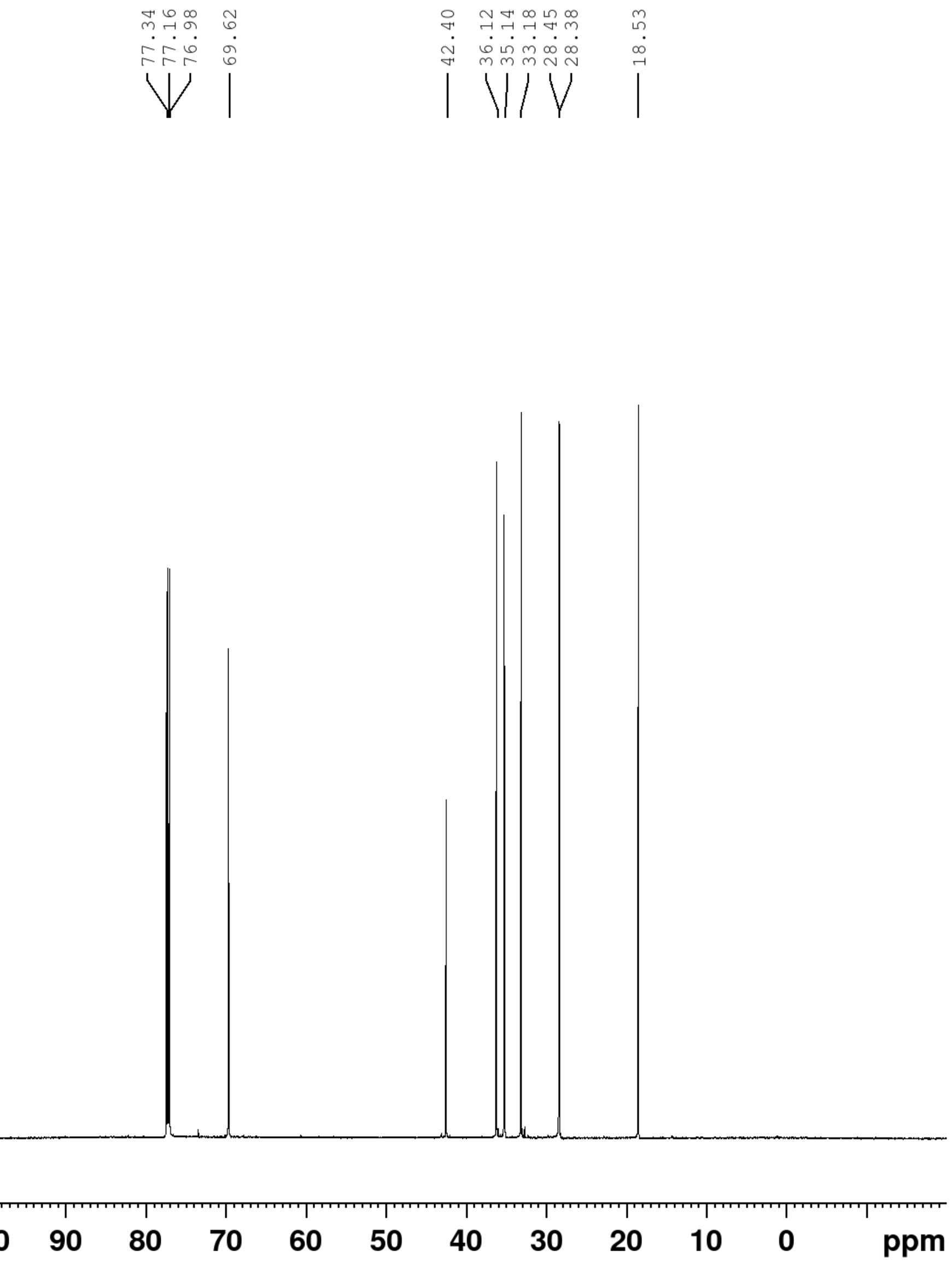

(3*S*,5*S*)-1,7-dicyclobutylheptane-3,5-diyl dibenzoate (1g-Bz)

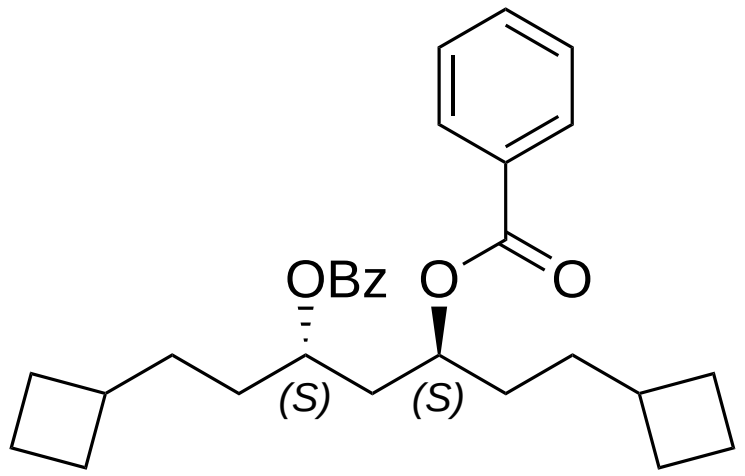

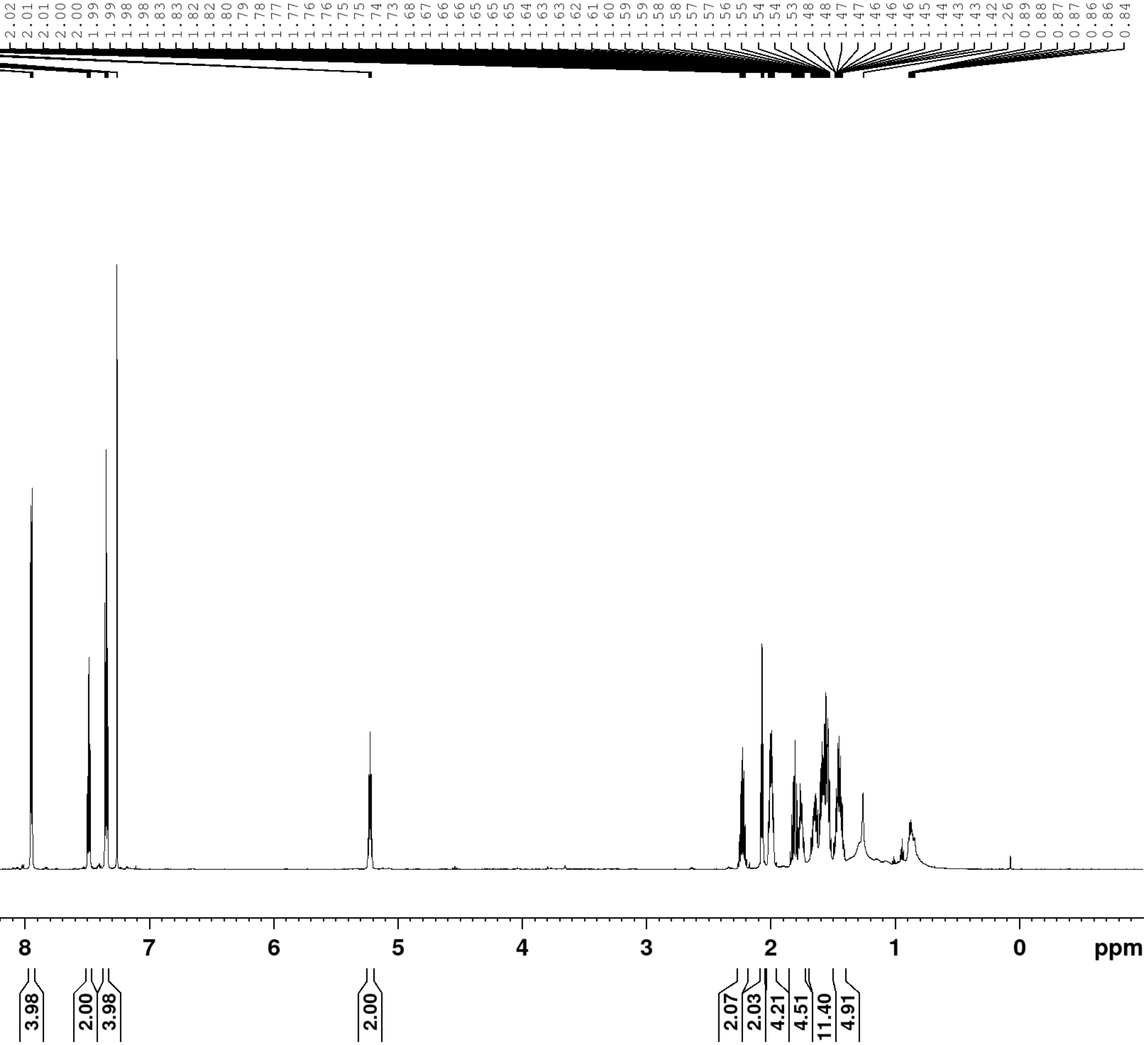

(3*S*,5*S*)-1,7-dicyclobutylheptane-3,5-diyl dibenzoate (1g-Bz)

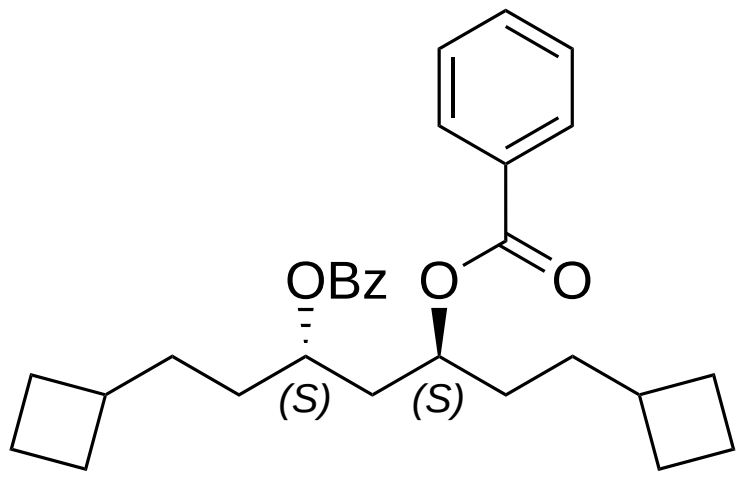

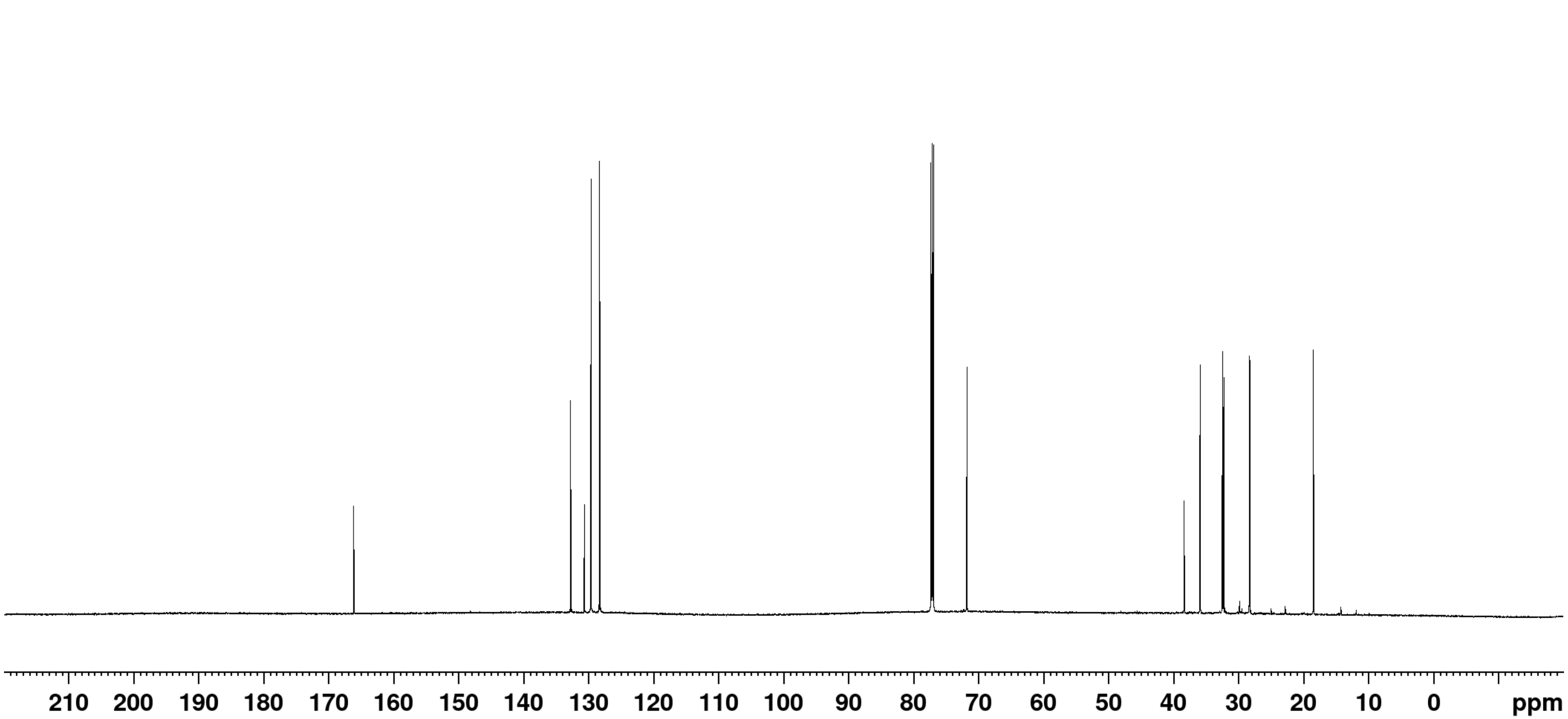

(2S,4S)-1,5-dicyclohexylpentane-2,4-diol (1h)

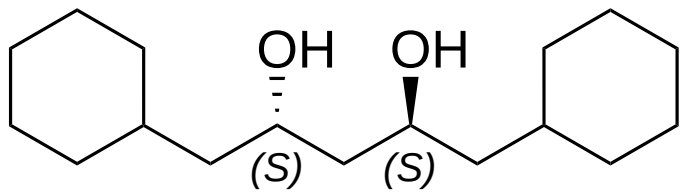

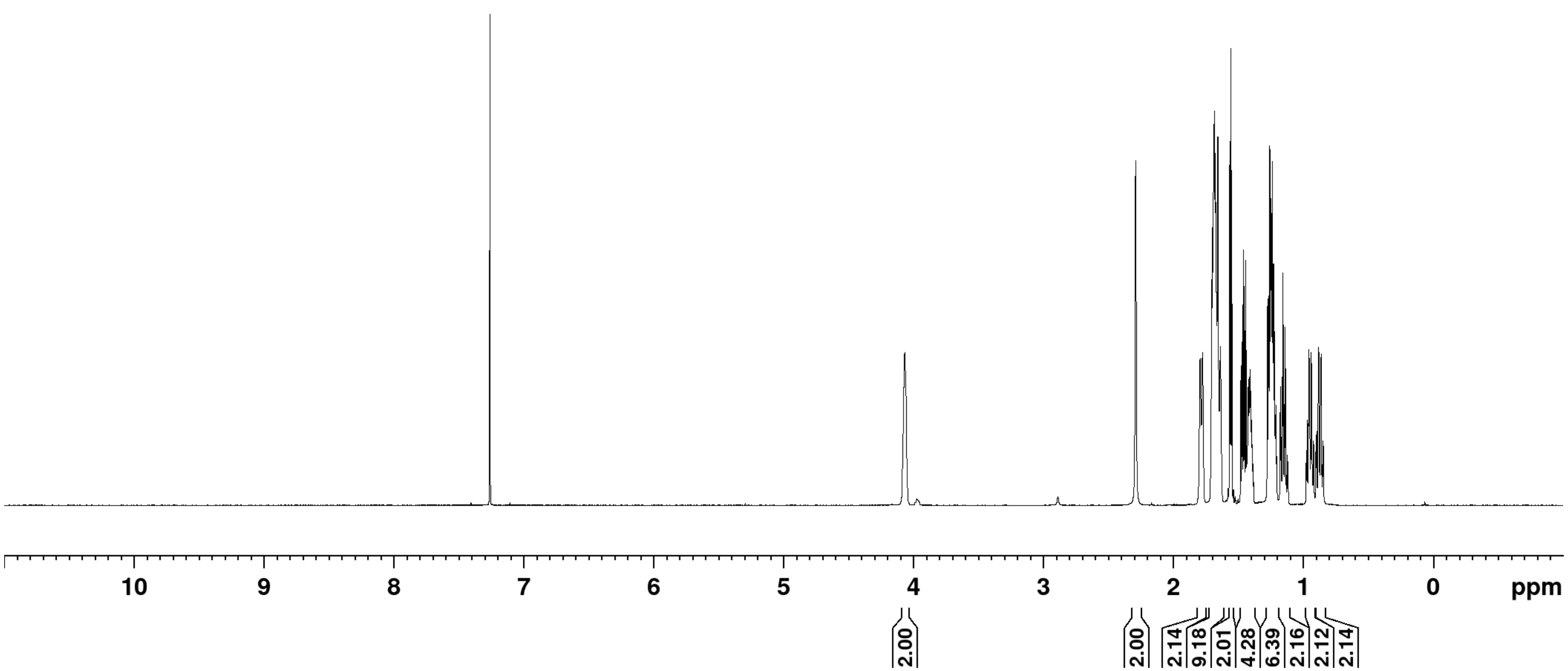

(2S,4S)-1,5-dicyclohexylpentane-2,4-diol (1h)

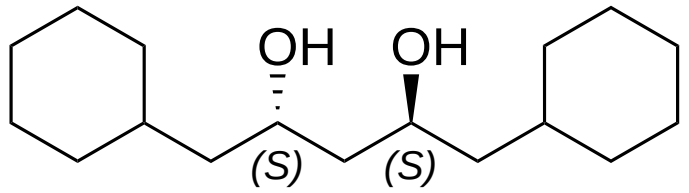

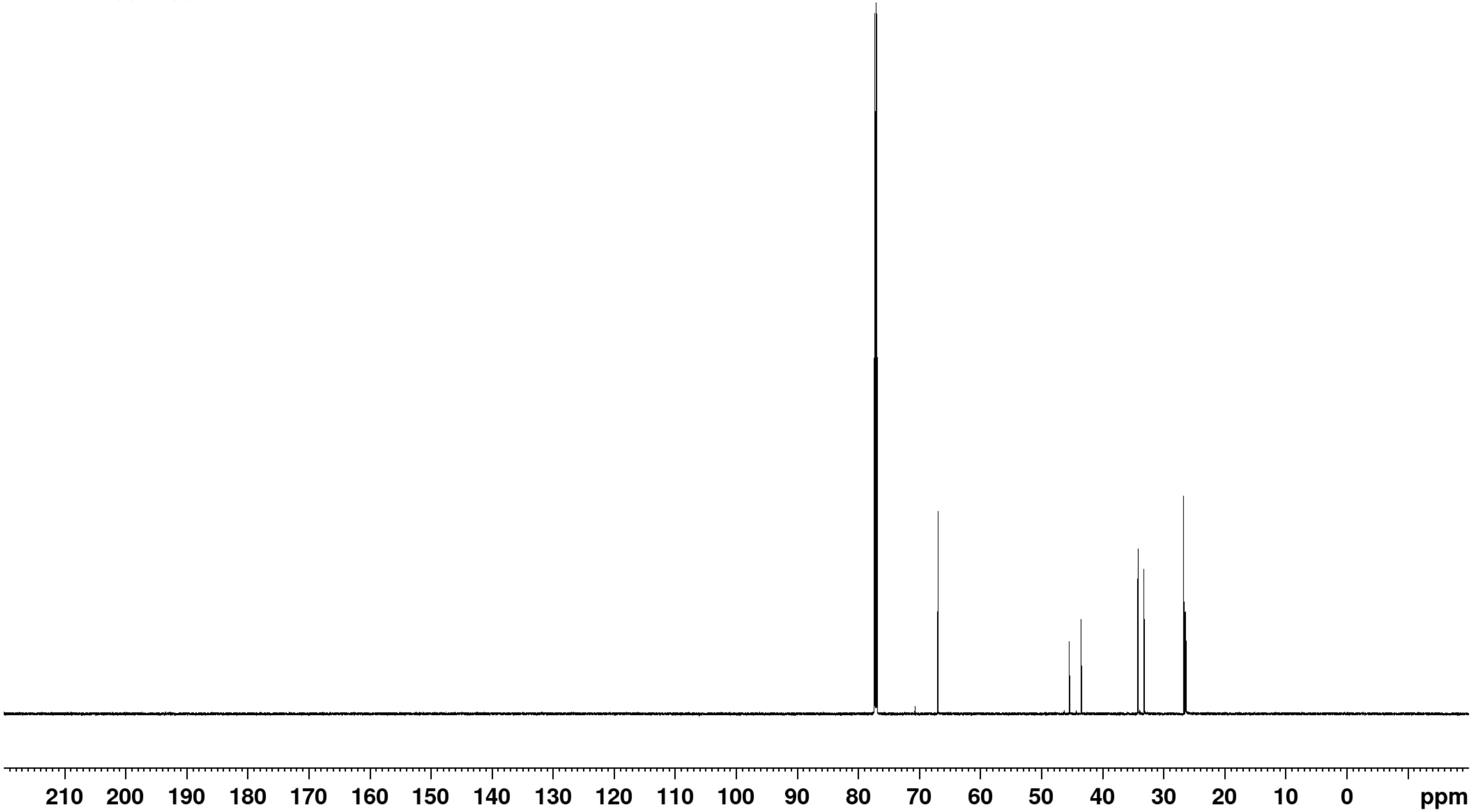

(2S,4S)-1,5-dicyclohexylpentane-2,4-diyl dibenzoate (1h-Bz)

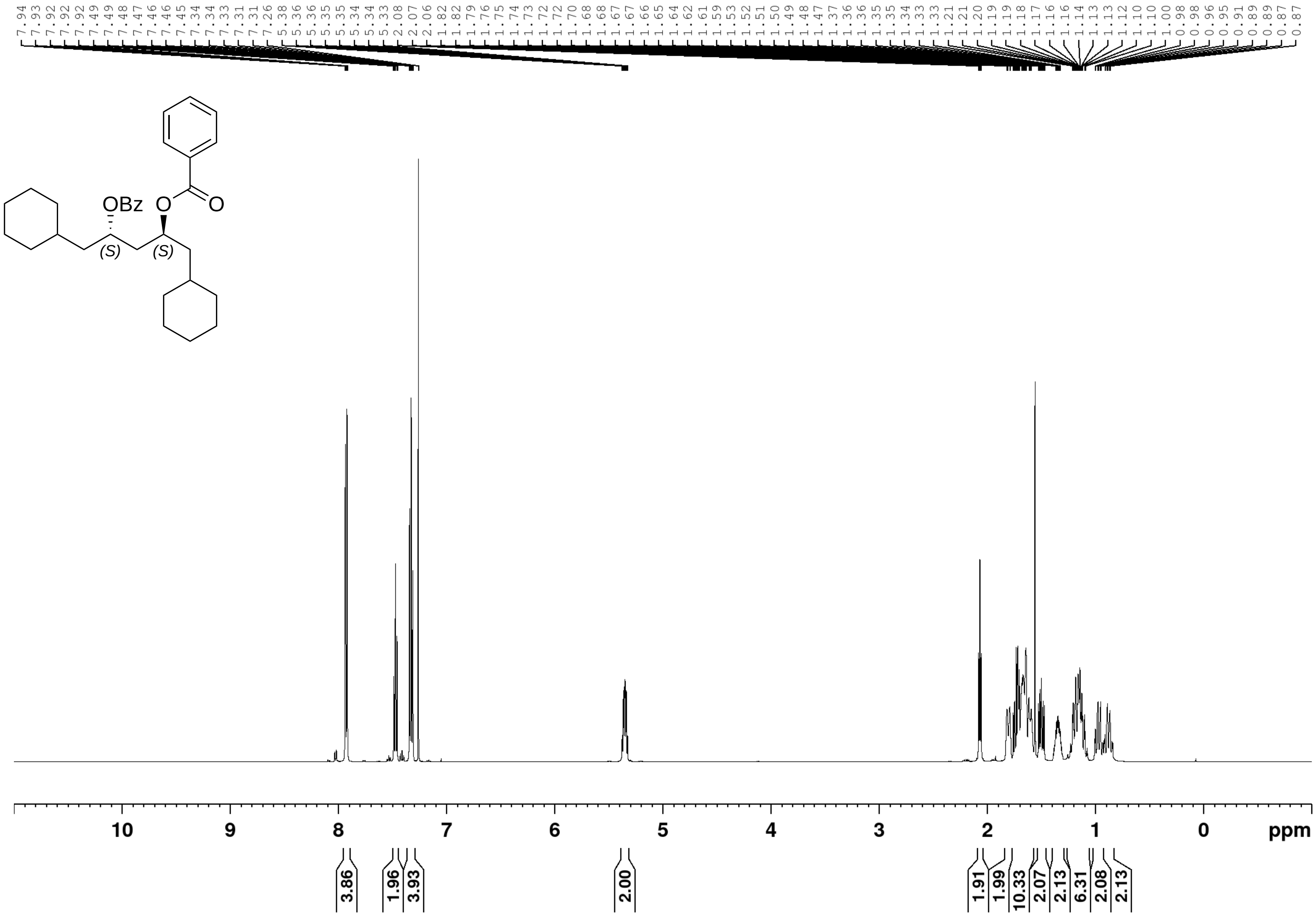

(2S,4S)-1,5-dicyclohexylpentane-2,4-diyl dibenzoate (1h-Bz)

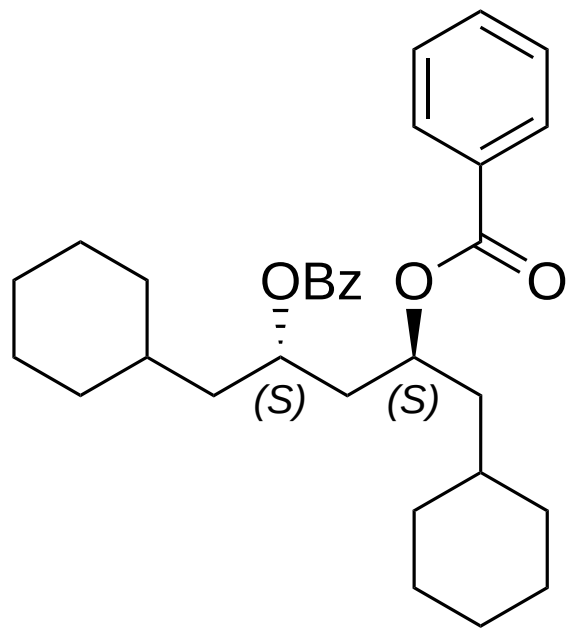

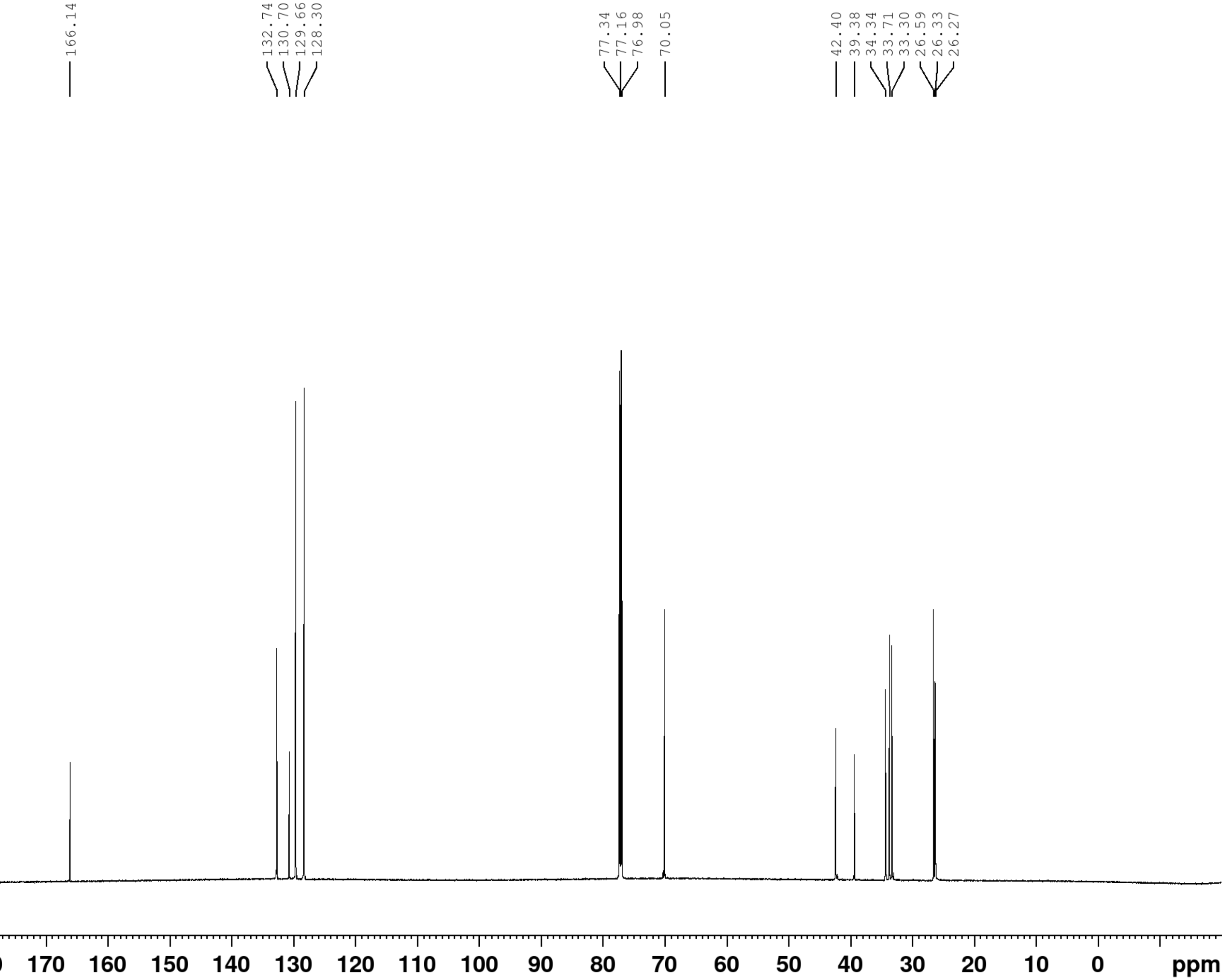

(3*S*,5*S*)-1,7-dicyclohexylheptane-3,5-diol (1i)

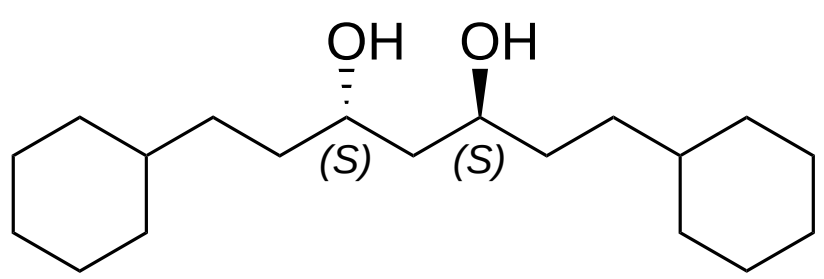

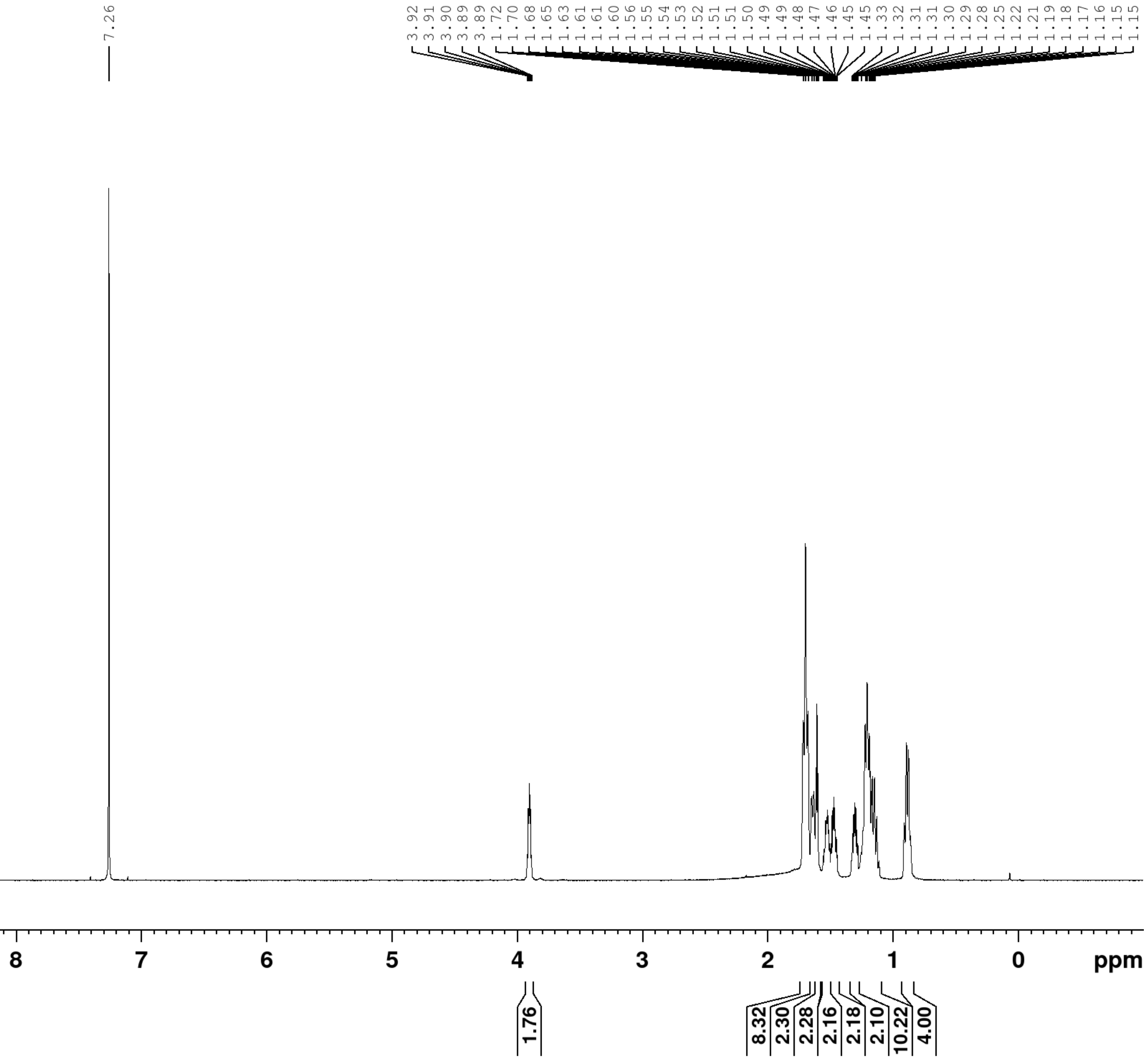

(3*S*,5*S*)-1,7-dicyclohexylheptane-3,5-diol (1i)

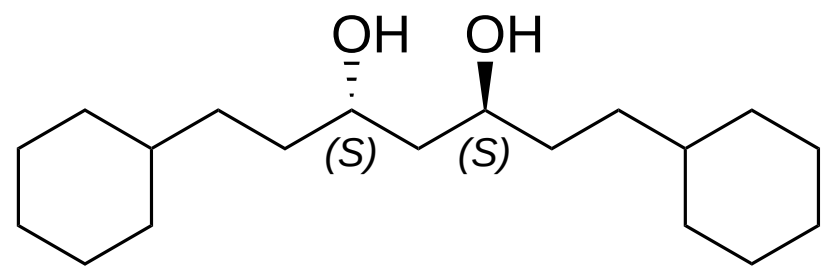

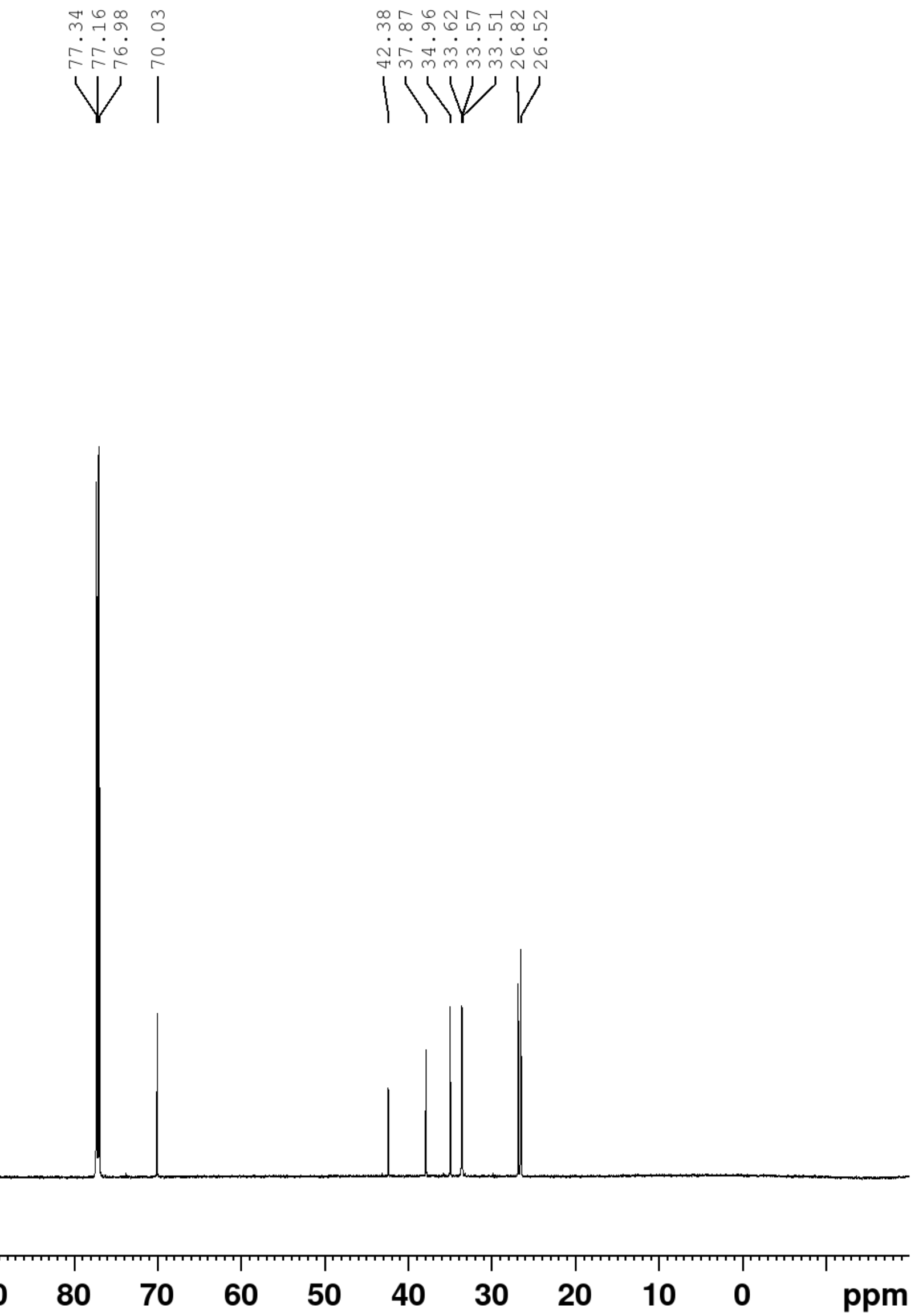

(3*S*,5*S*)-1,7-dicyclohexylheptane-3,5-diyl dibenzoate (1i-Bz)

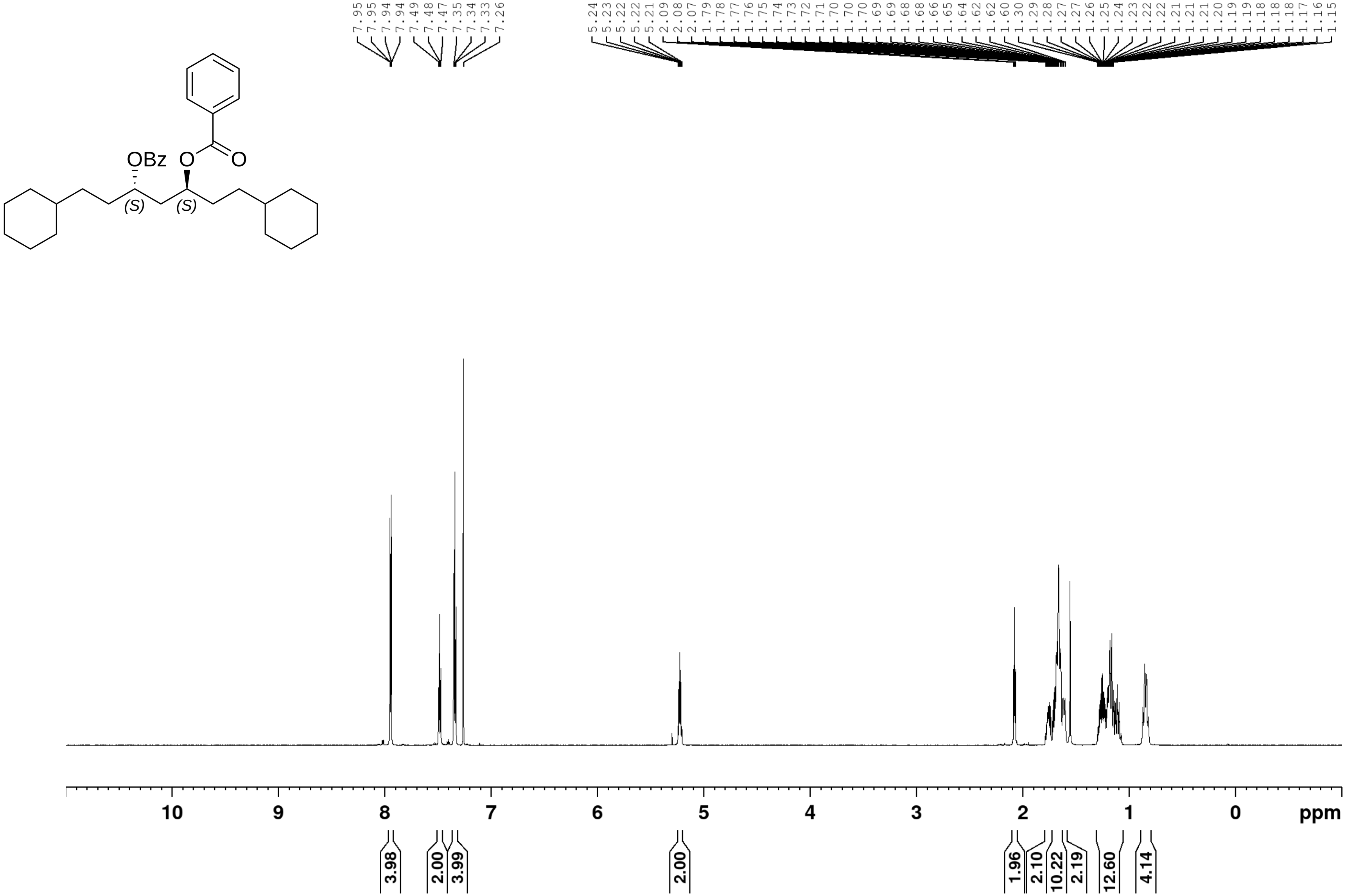

(3*S*,5*S*)-1,7-dicyclohexylheptane-3,5-diyl dibenzoate (1i-Bz)

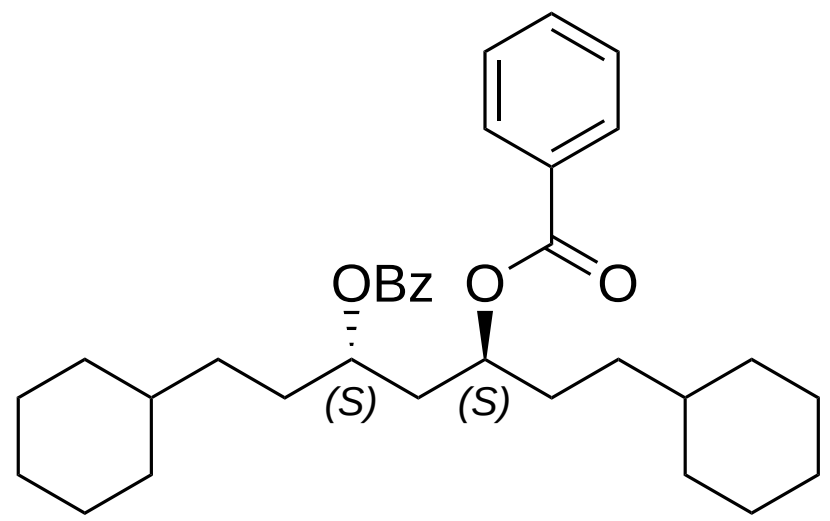

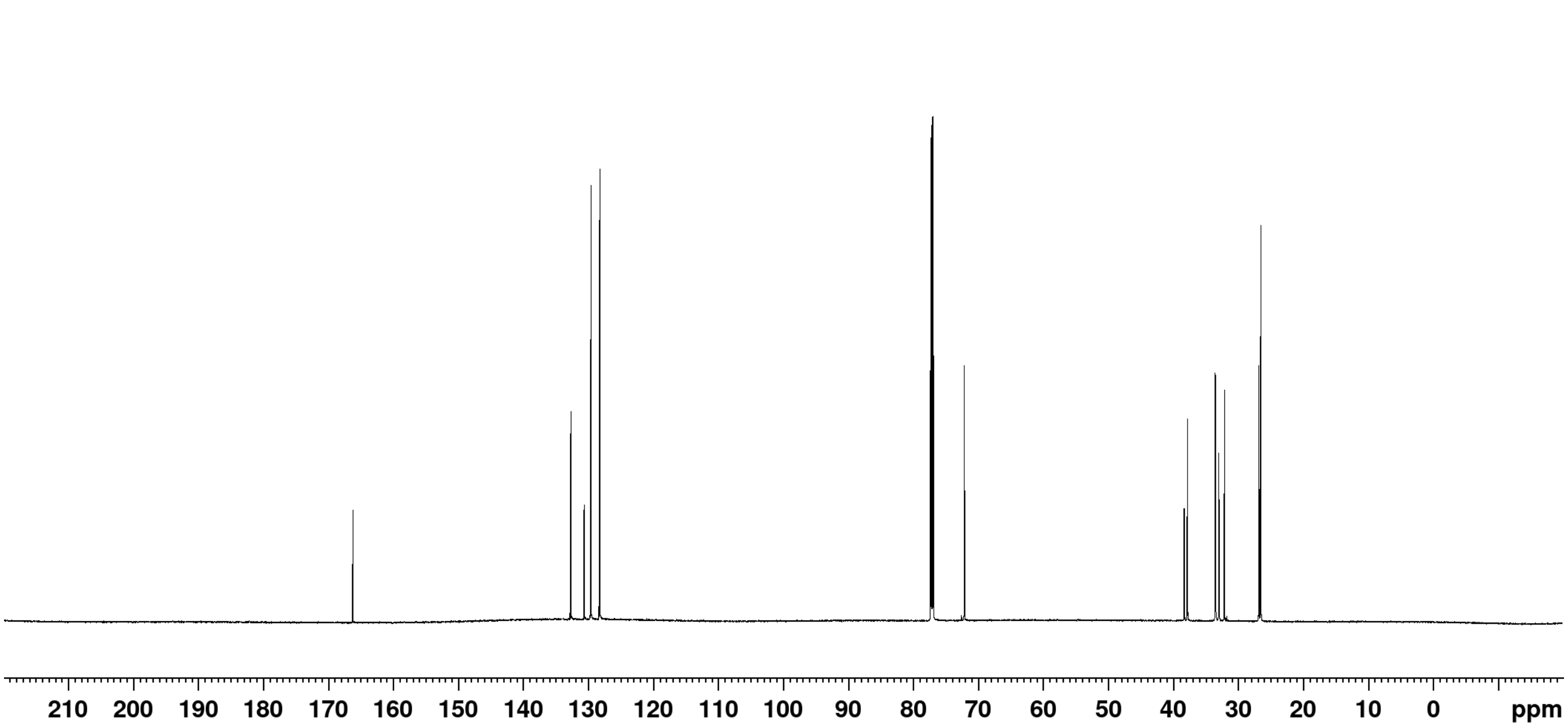

(4S,6S)-1,9-dicyclohexylnonane-4,6-diol (1j)

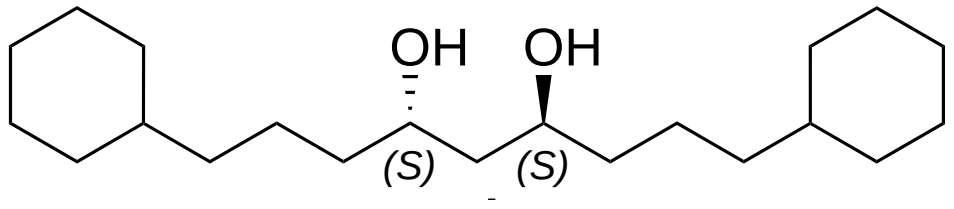

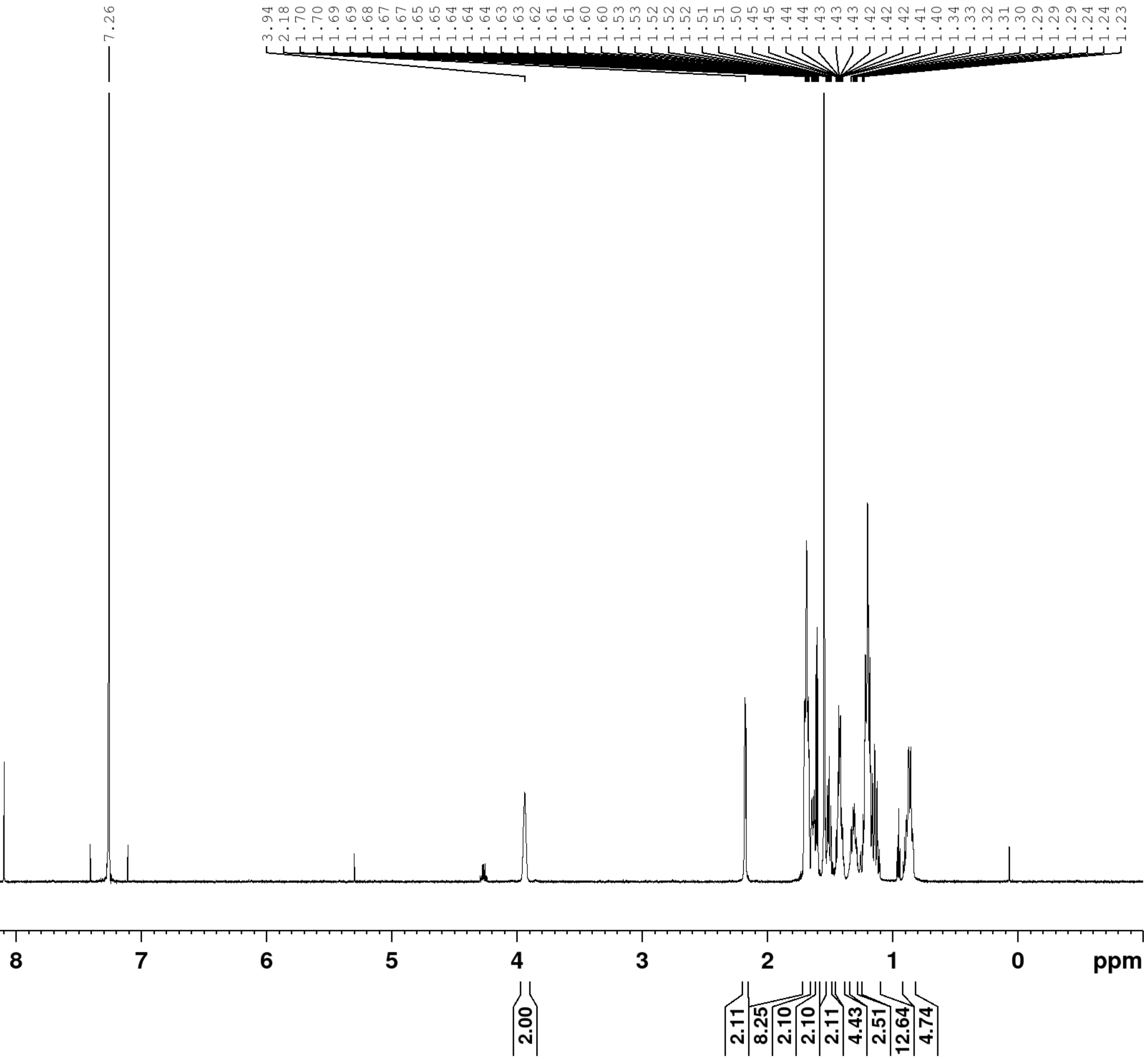

(4S,6S)-1,9-dicyclohexylnonane-4,6-diol (1j)

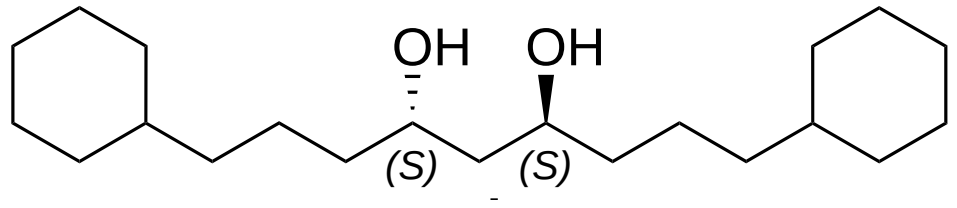

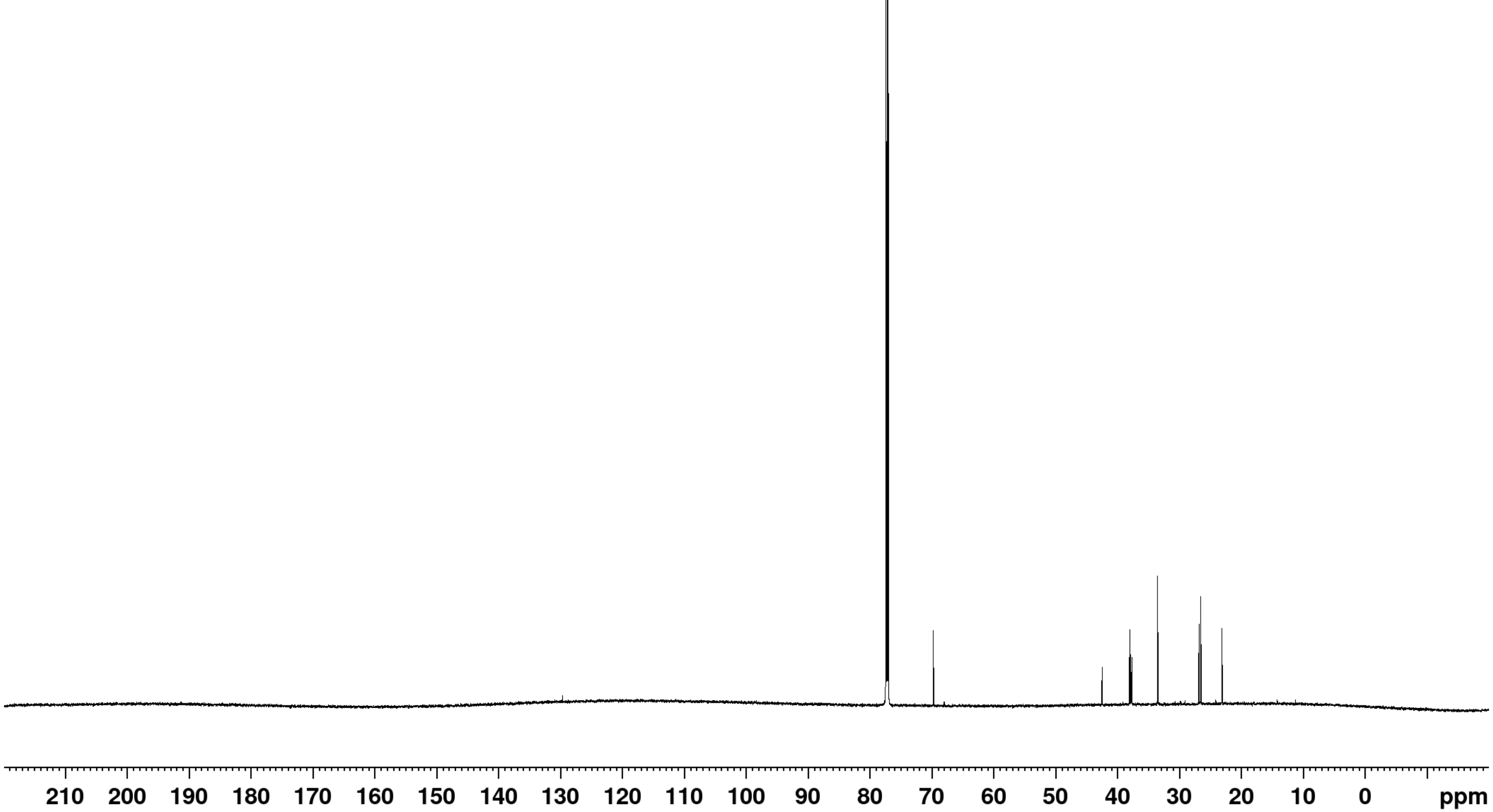

(4*S*,6*S*)-1,9-dicyclohexylnonane-4,6-diyl dibenzoate Number (1j-Bz)

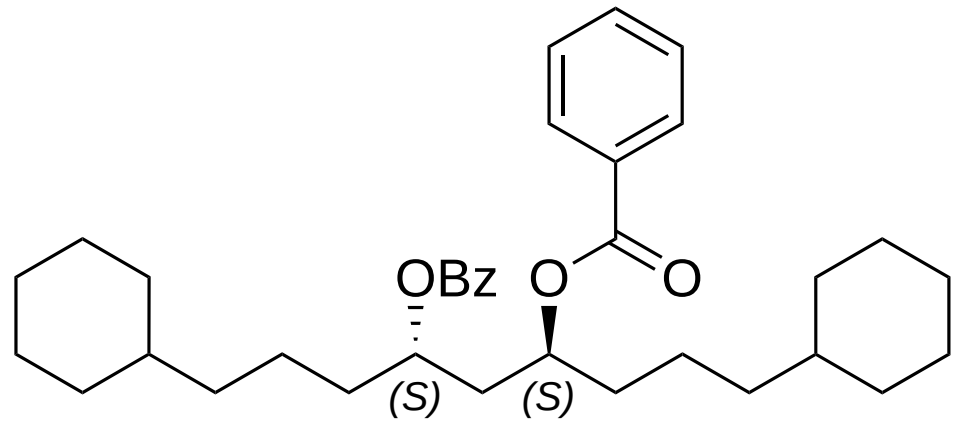

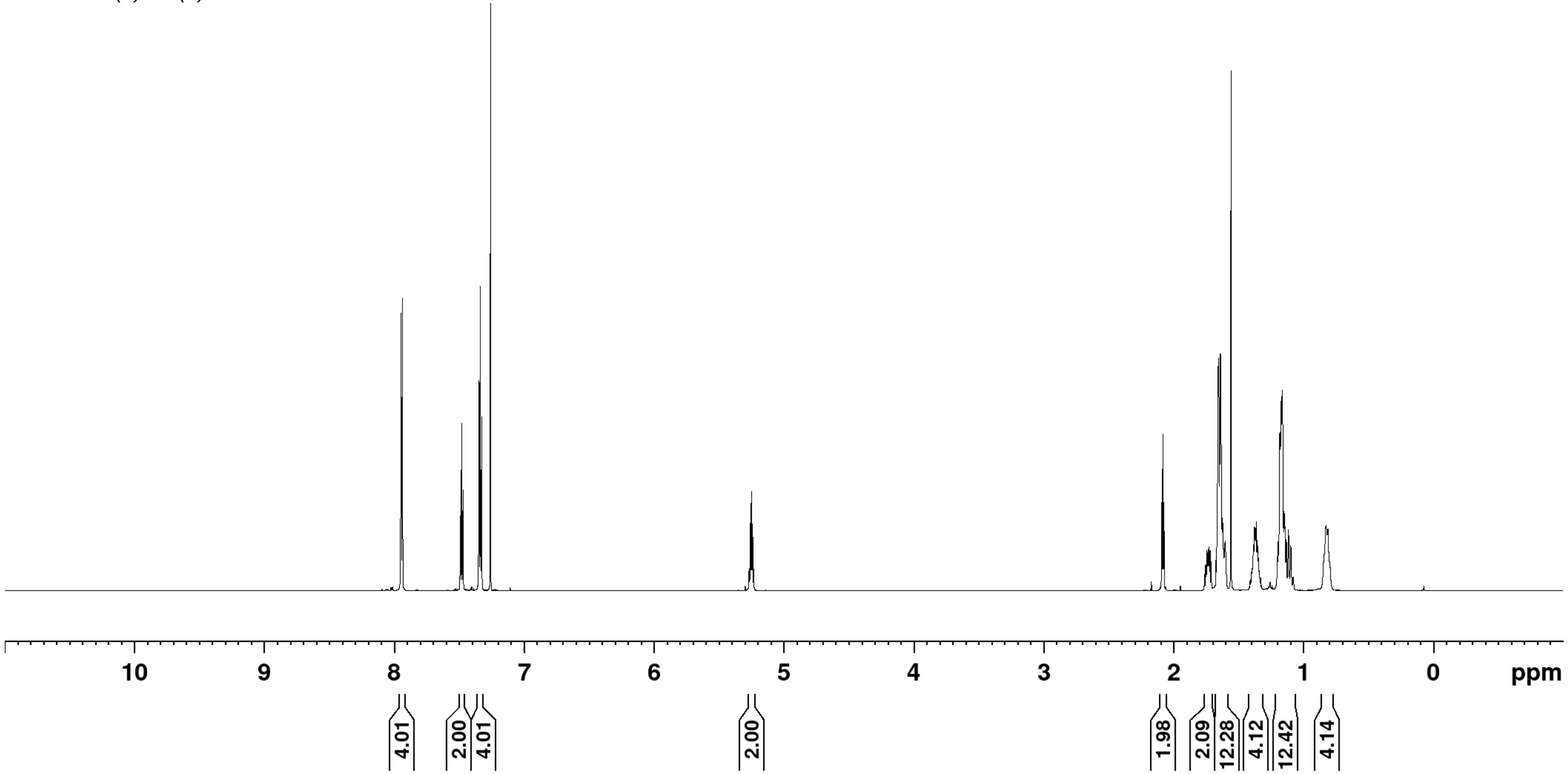

(4*S*,6*S*)-1,9-dicyclohexylnonane-4,6-diyl dibenzoate Number (1j-Bz)

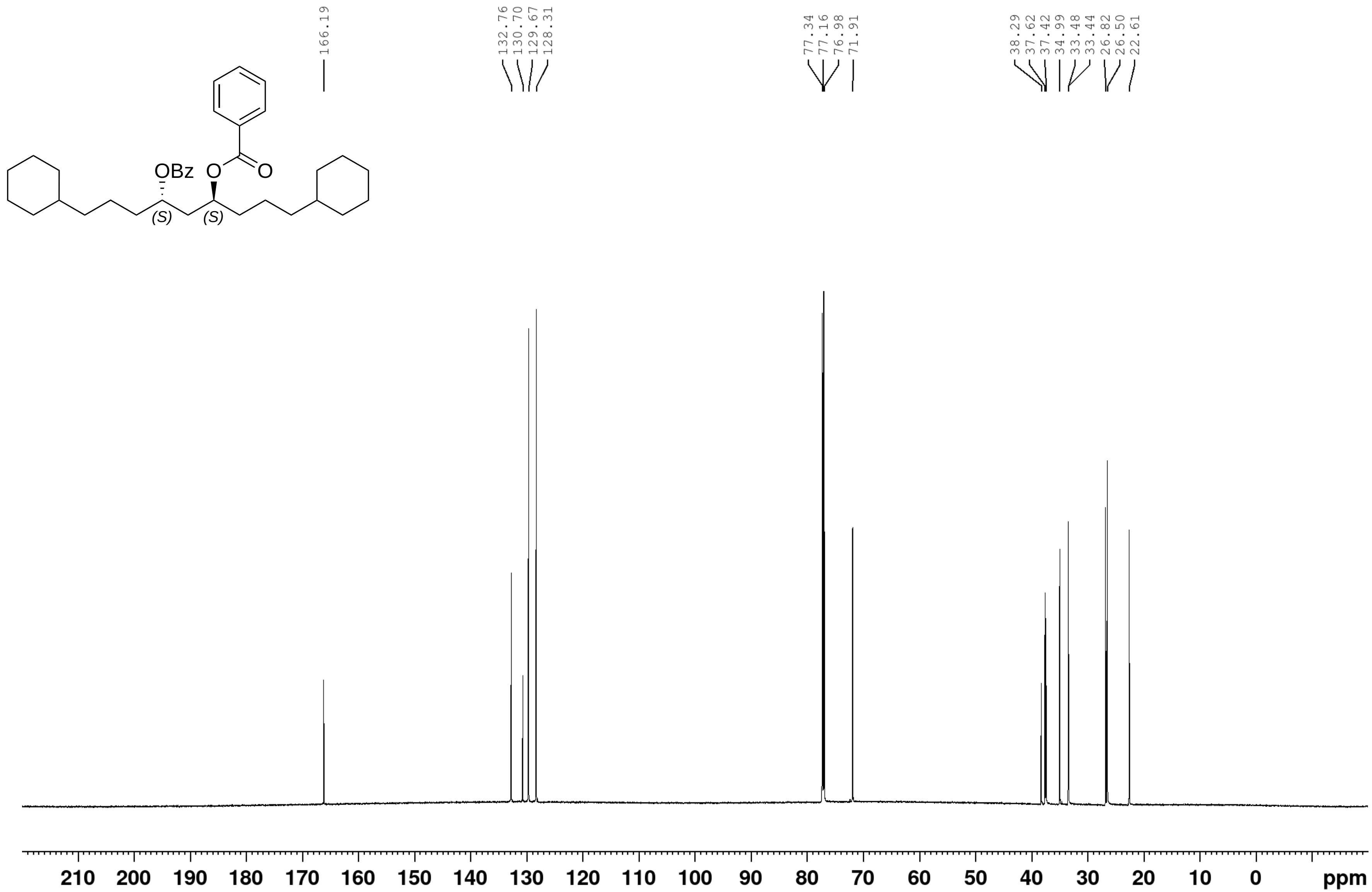

(3S,5S)-1,7-diphenylheptane-3,5-diol (1k)

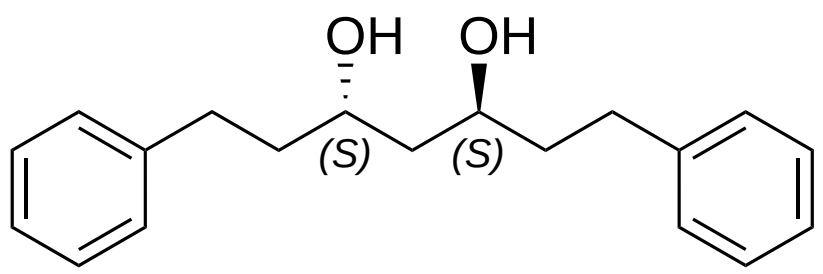

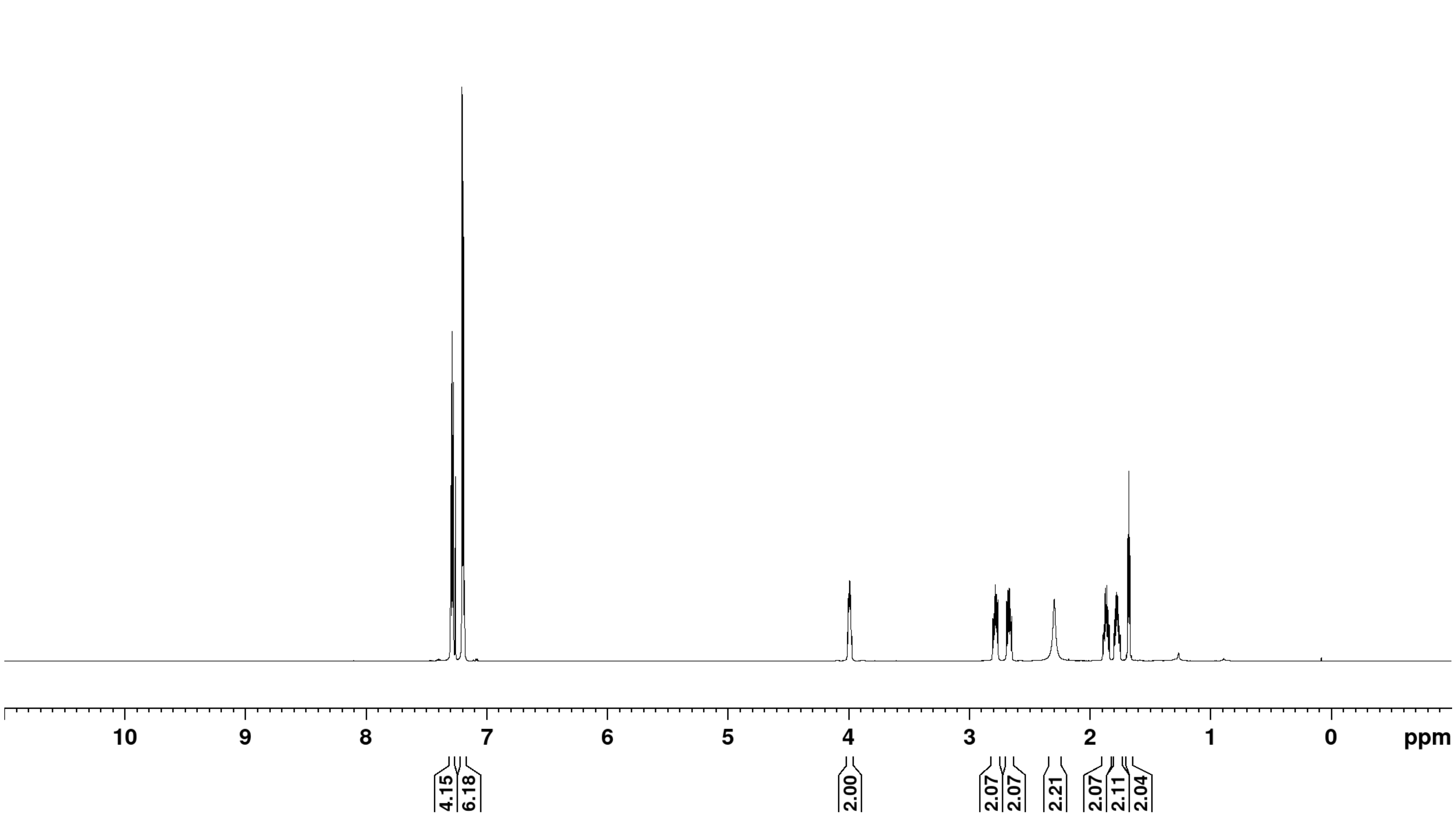

(3S,5S)-1,7-diphenylheptane-3,5-diol (1k)

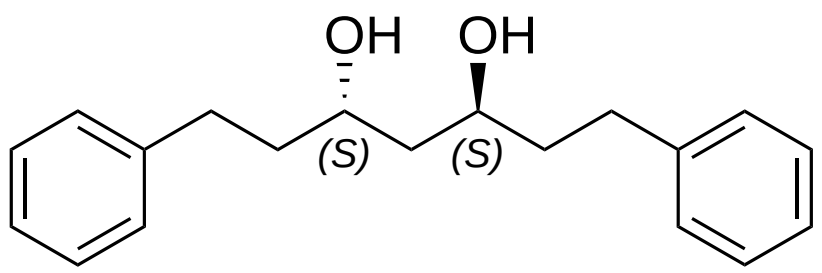

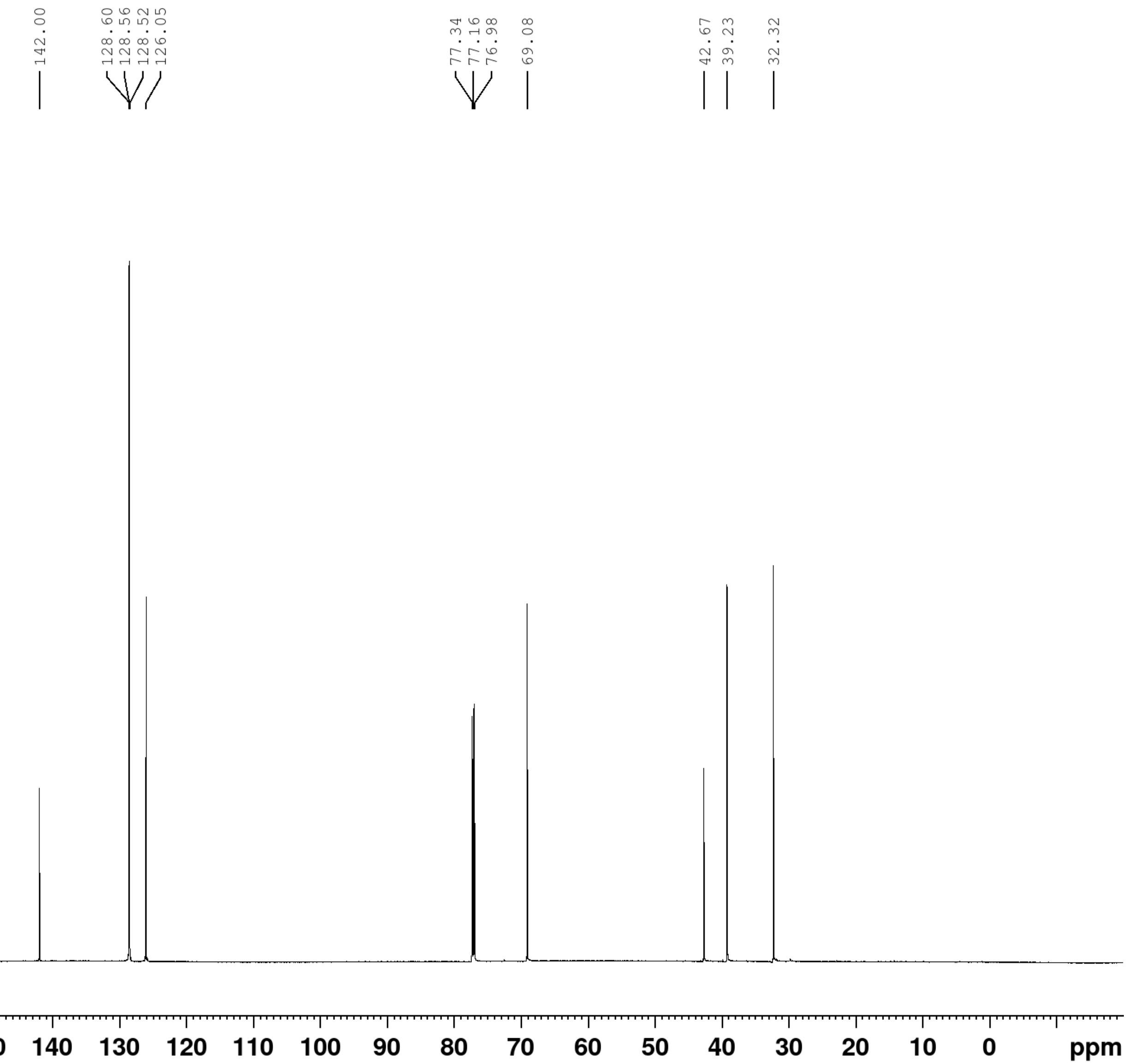

(4S,6S)-1,9-diphenylnonane-4,6-diol (1l)

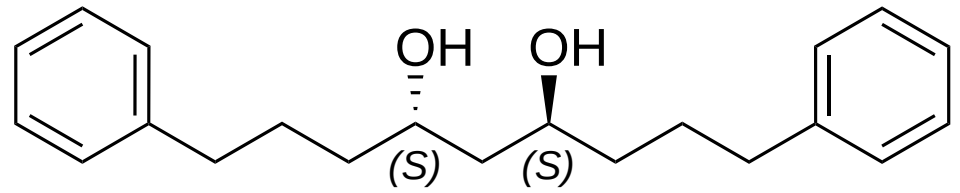

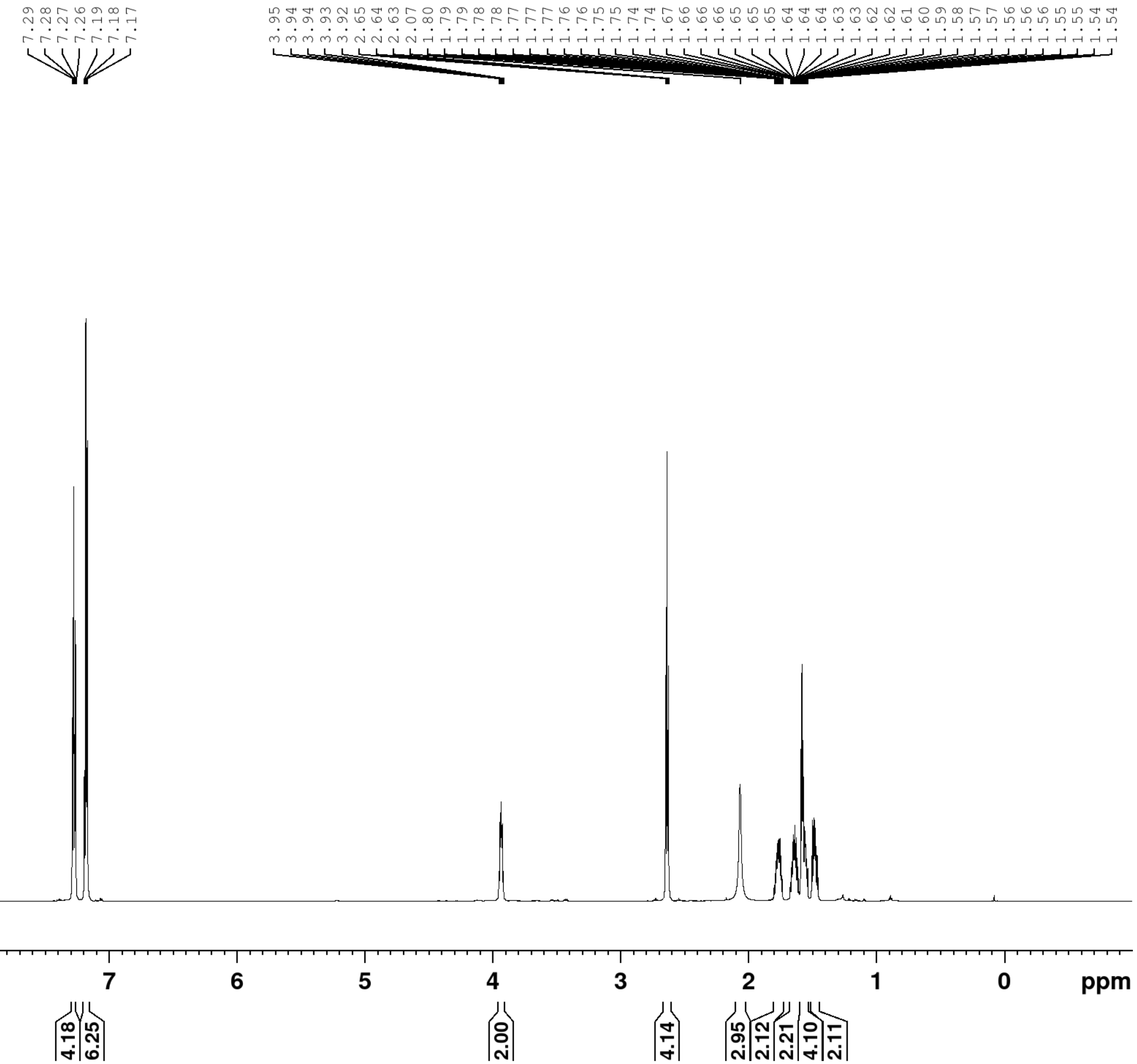

(4S,6S)-1,9-diphenylnonane-4,6-diol (1l)

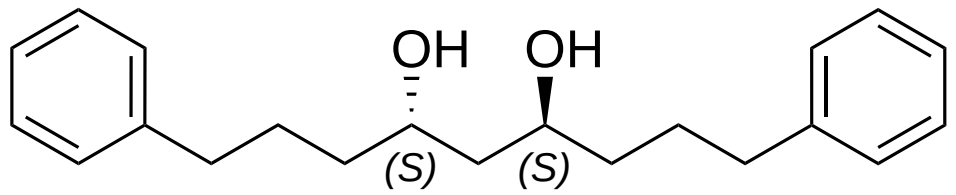

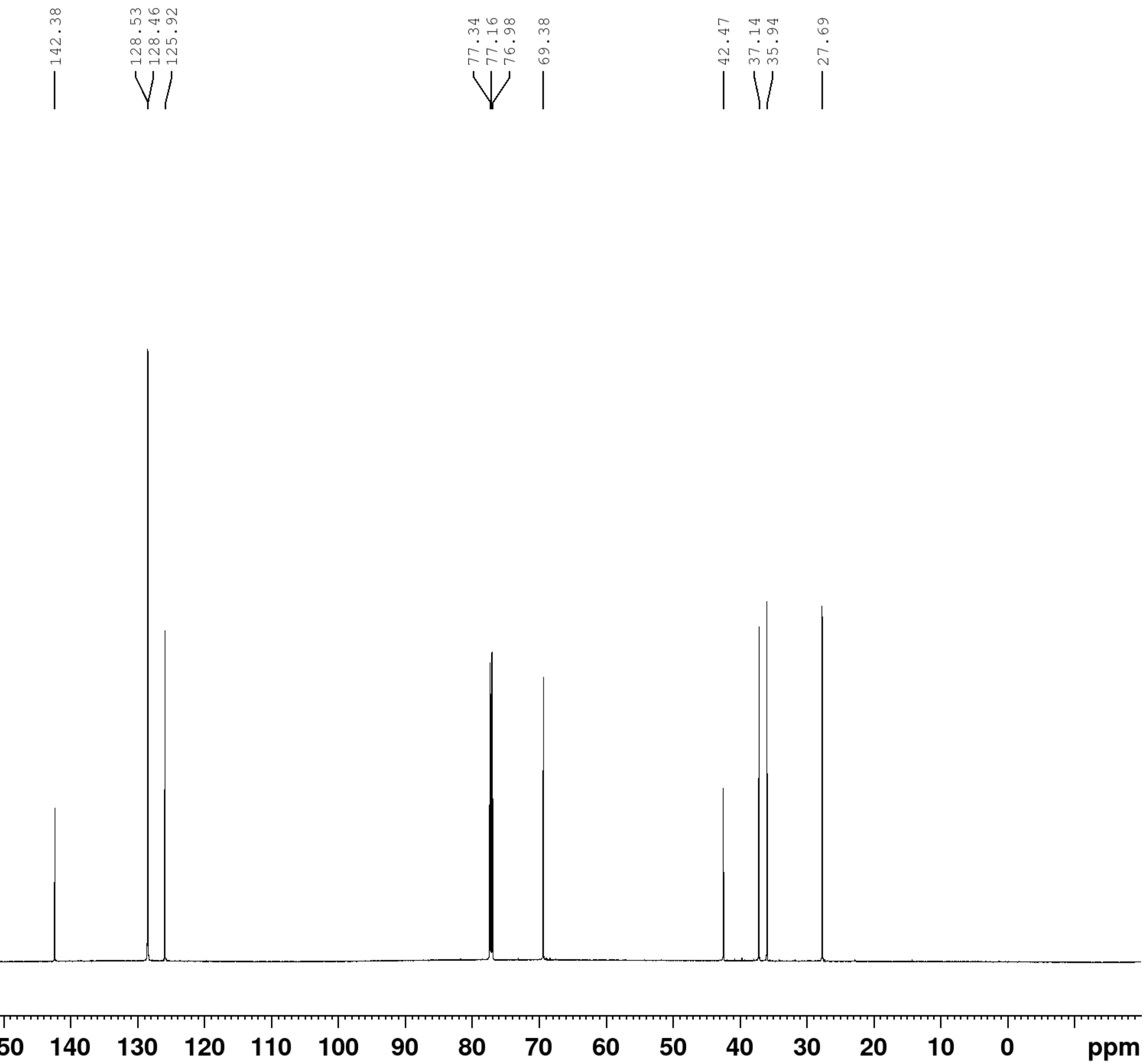

Reaction starting from syn-1,3-diol (1d)

(5S,7S)-undecane-5,7-diol (1d)

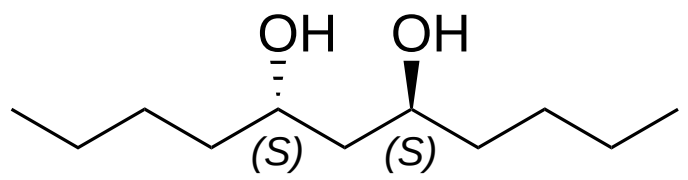

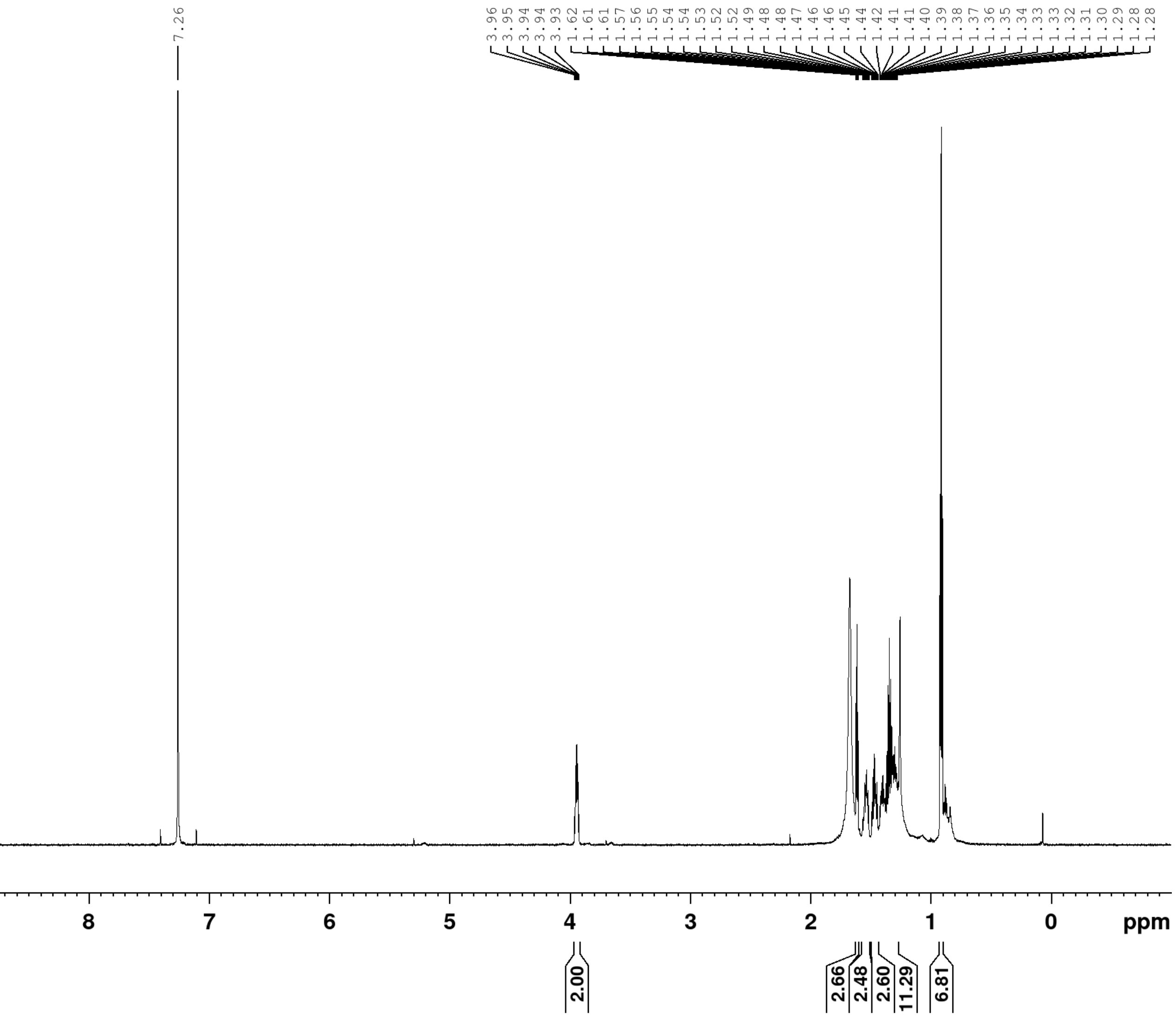

(5S,7S)-undecane-5,7-diol (1d)

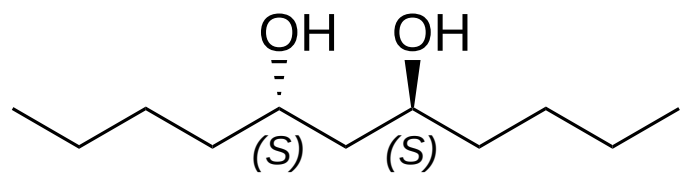

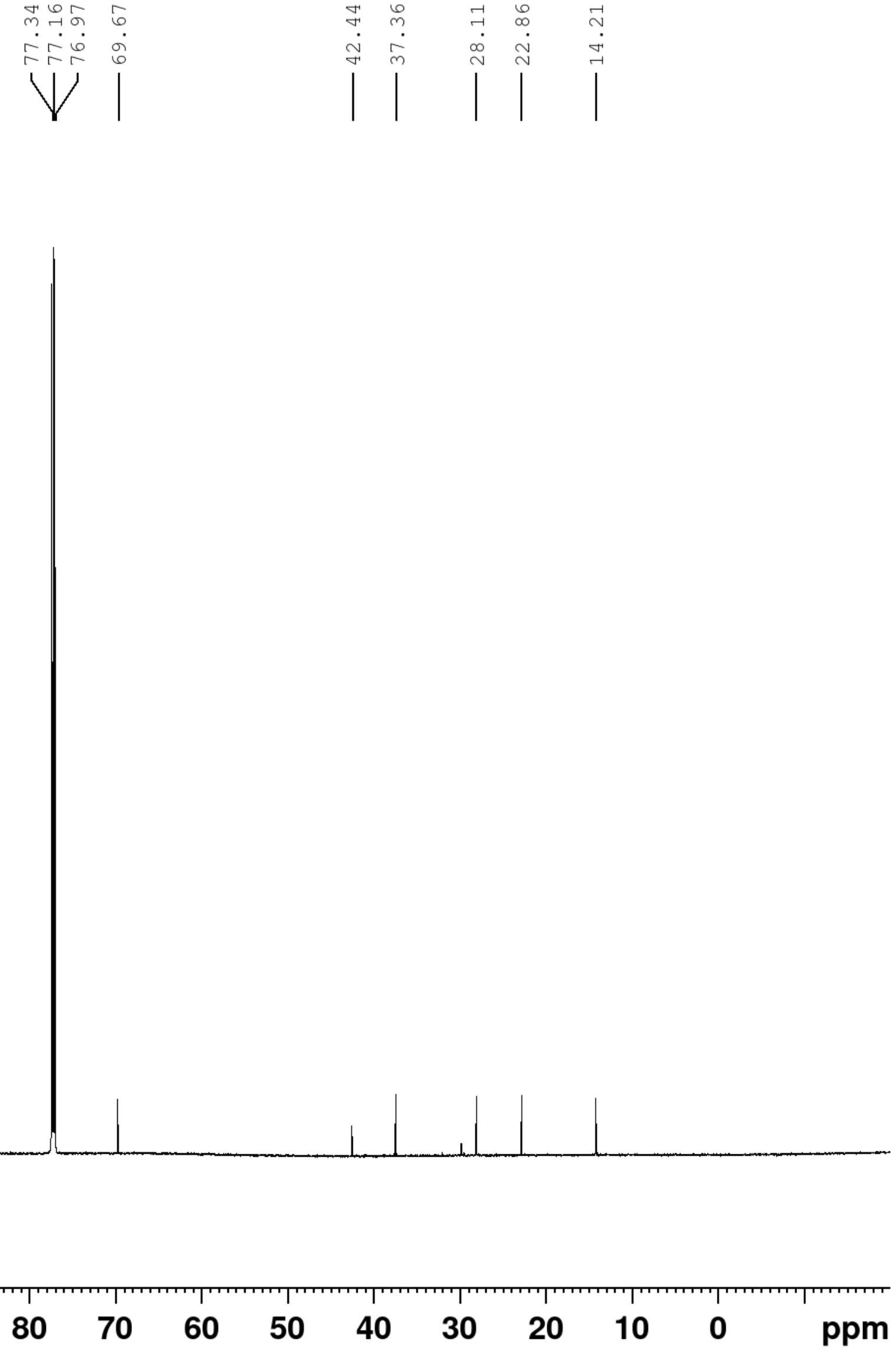

(5*S*,7*S*)-undecane-5,7-diyl dibenzoate (1d-Bz)

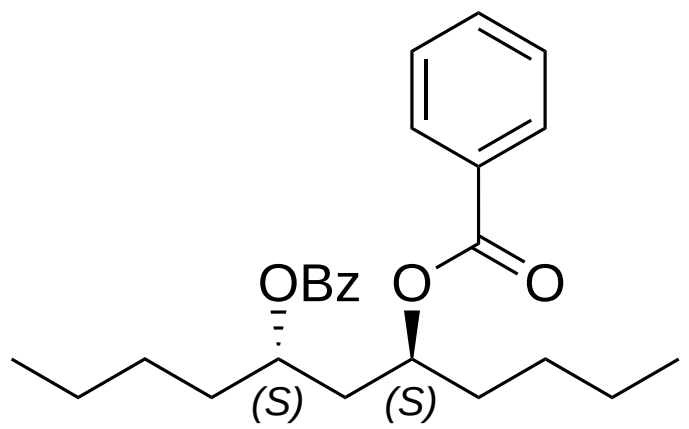

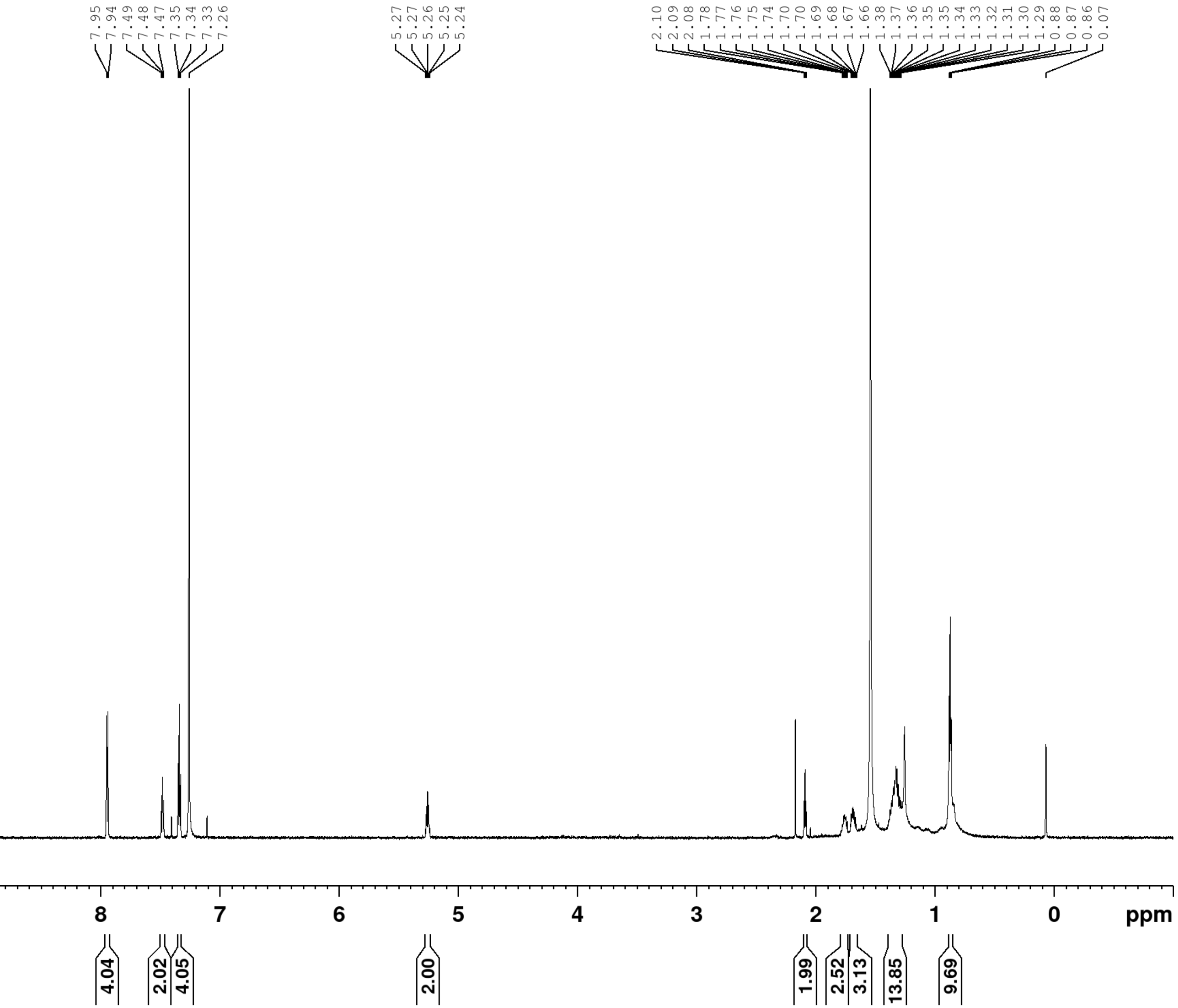

(5*S*,7*S*)-undecane-5,7-diyl dibenzoate (1d-Bz)

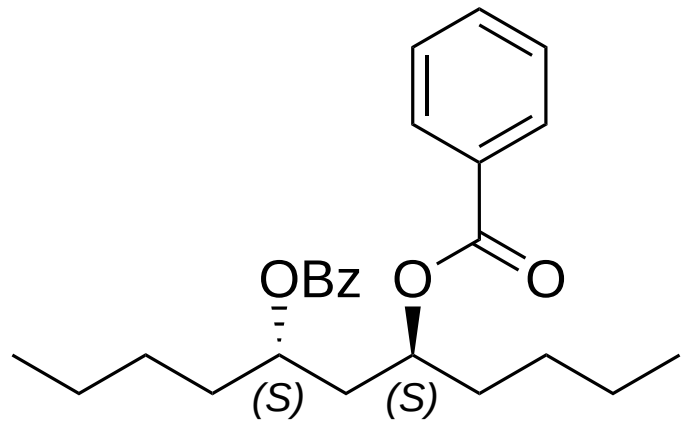

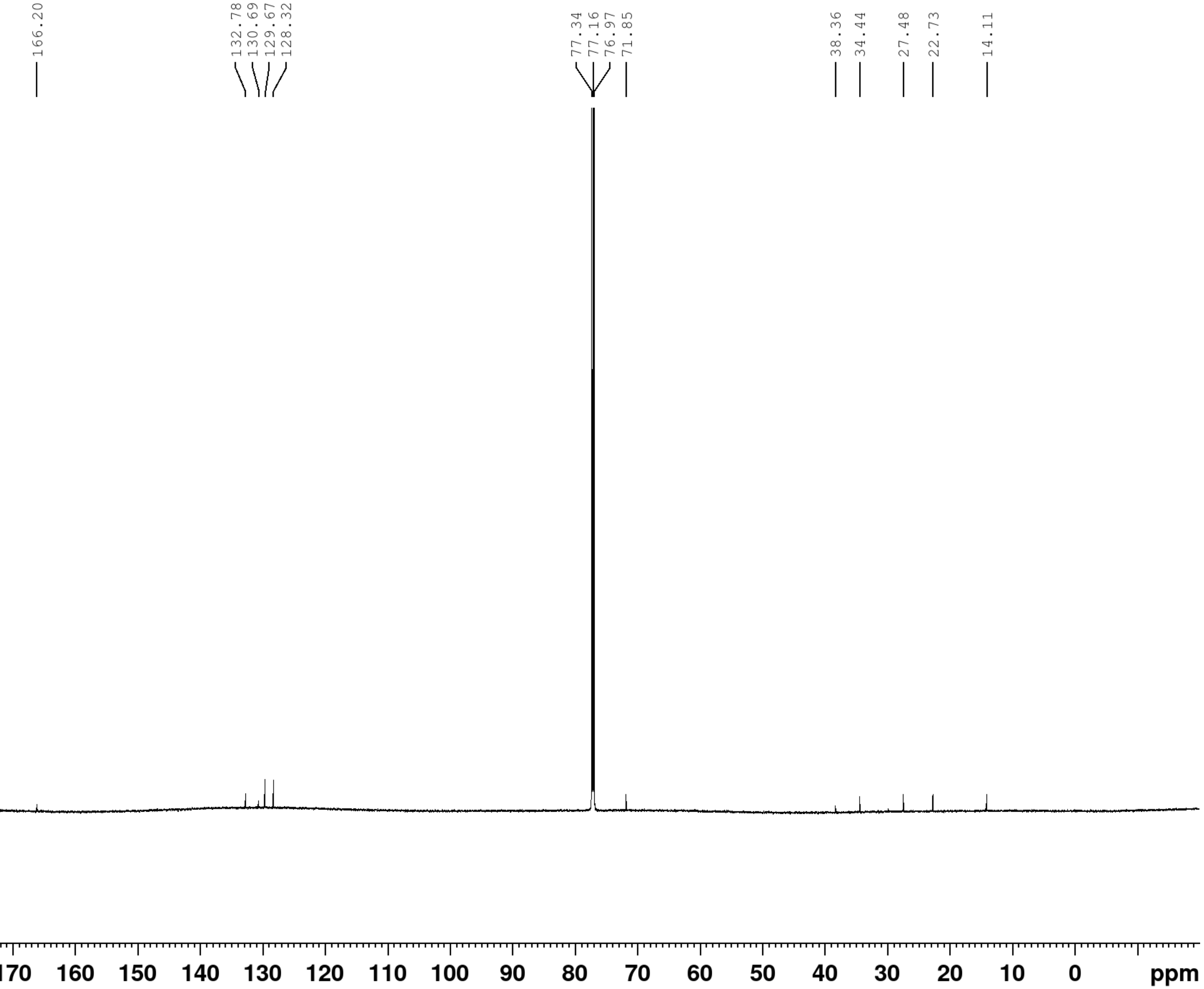

Deuterium study from *rac*-1d

(*syn*)-undecane-5,6,6,7-d_4-5,7-diol (*meso*-1d)

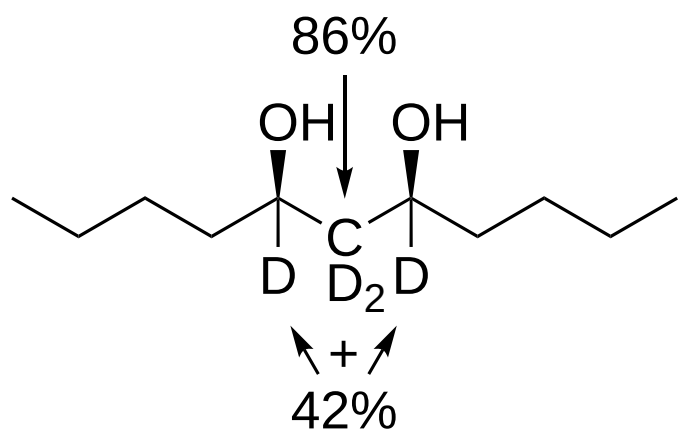

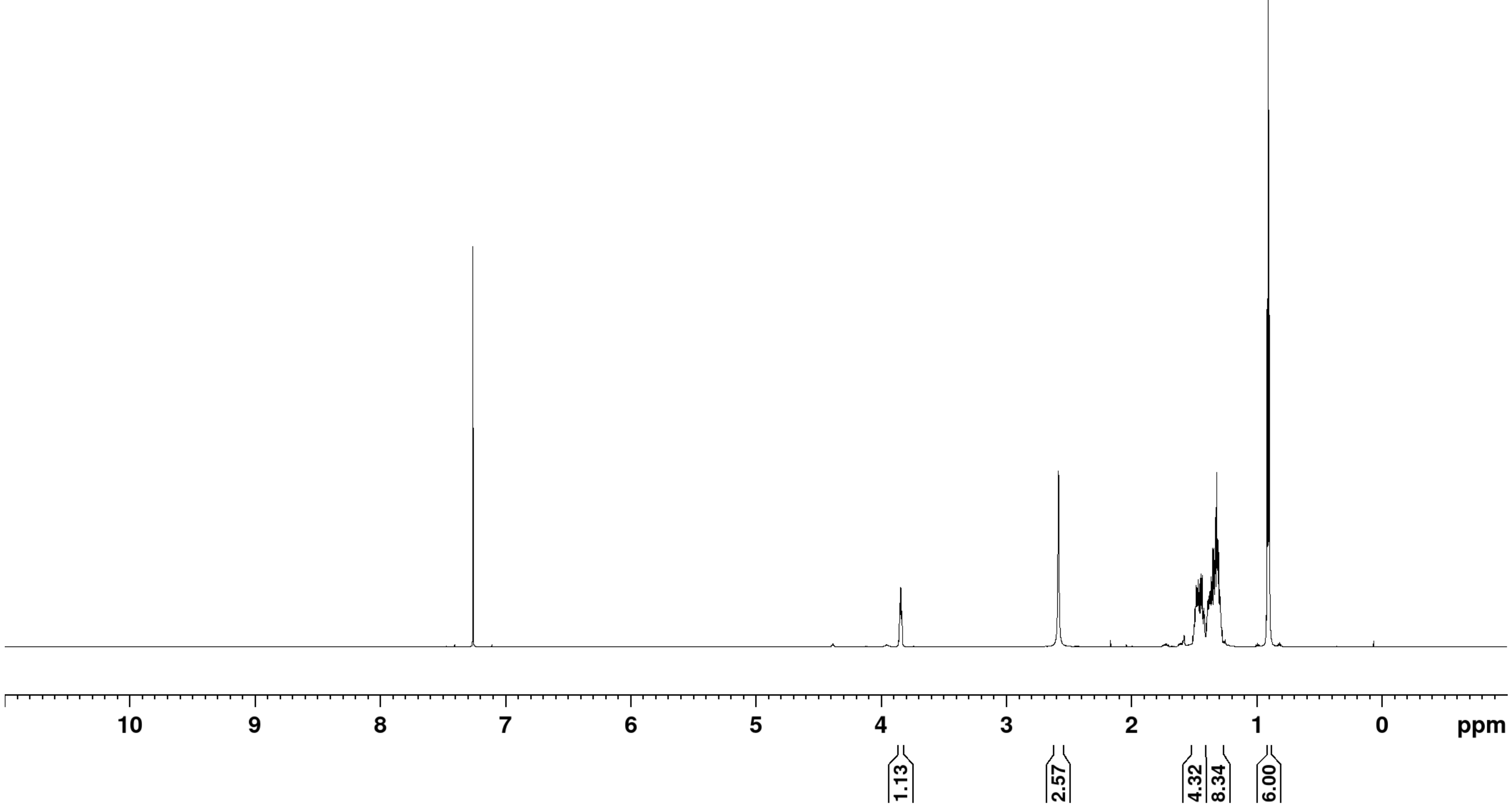

(*syn*)-undecane-5,6,6,7-d_4-5,7-diol (*meso*-1d)

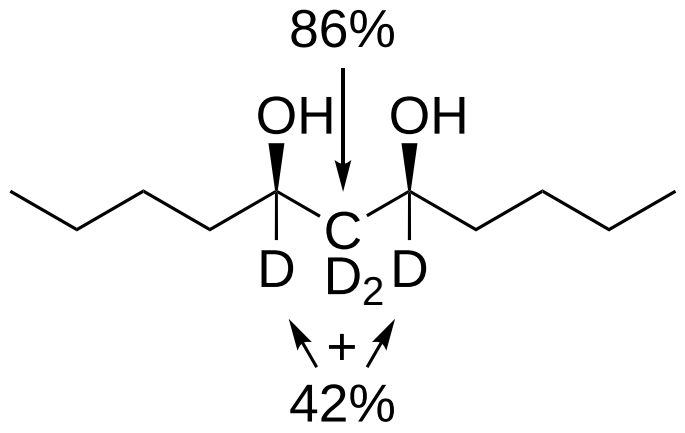

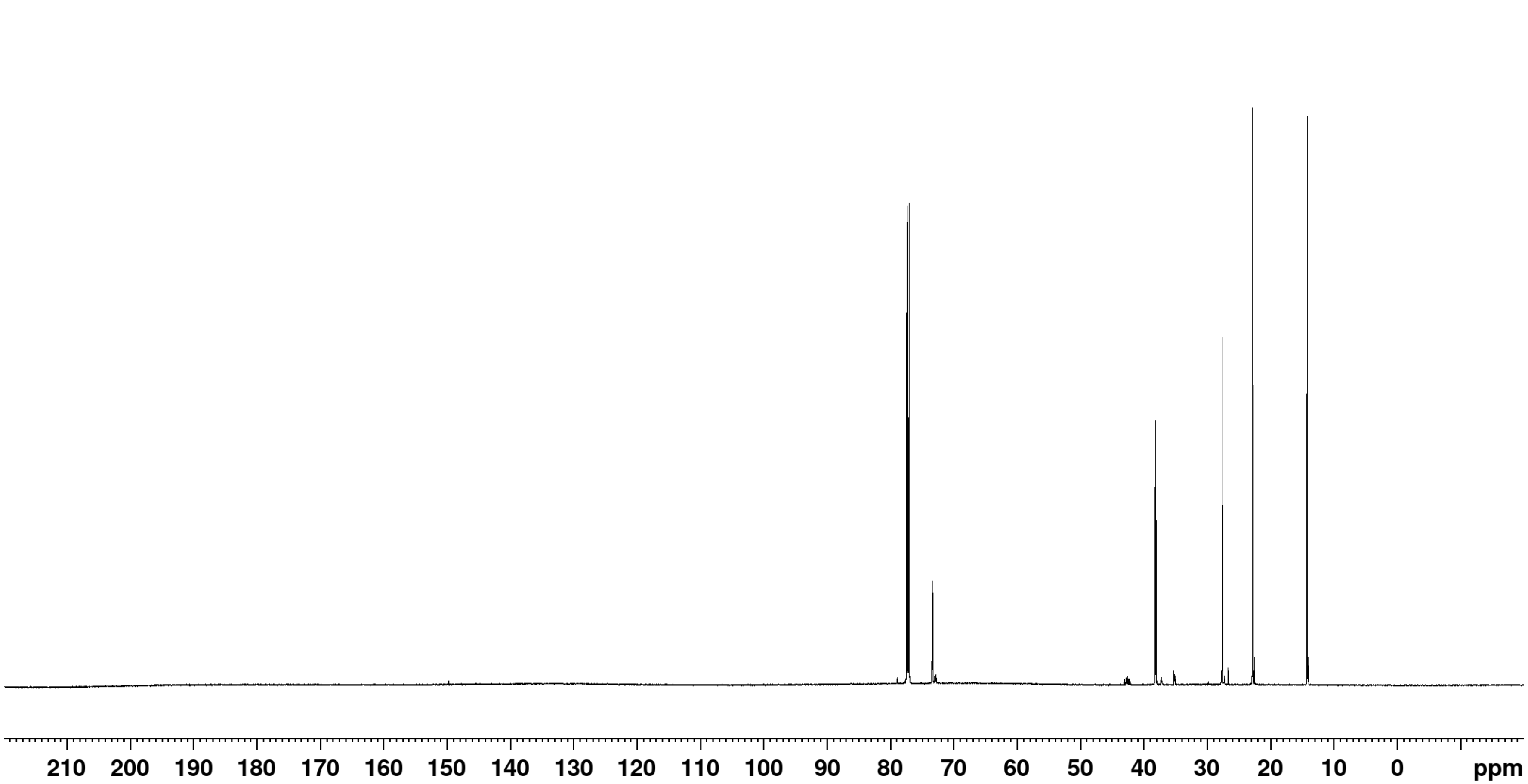

(*syn*)-undecane-5,6,6,7-d_4-5,7-diol (*meso*-1d)

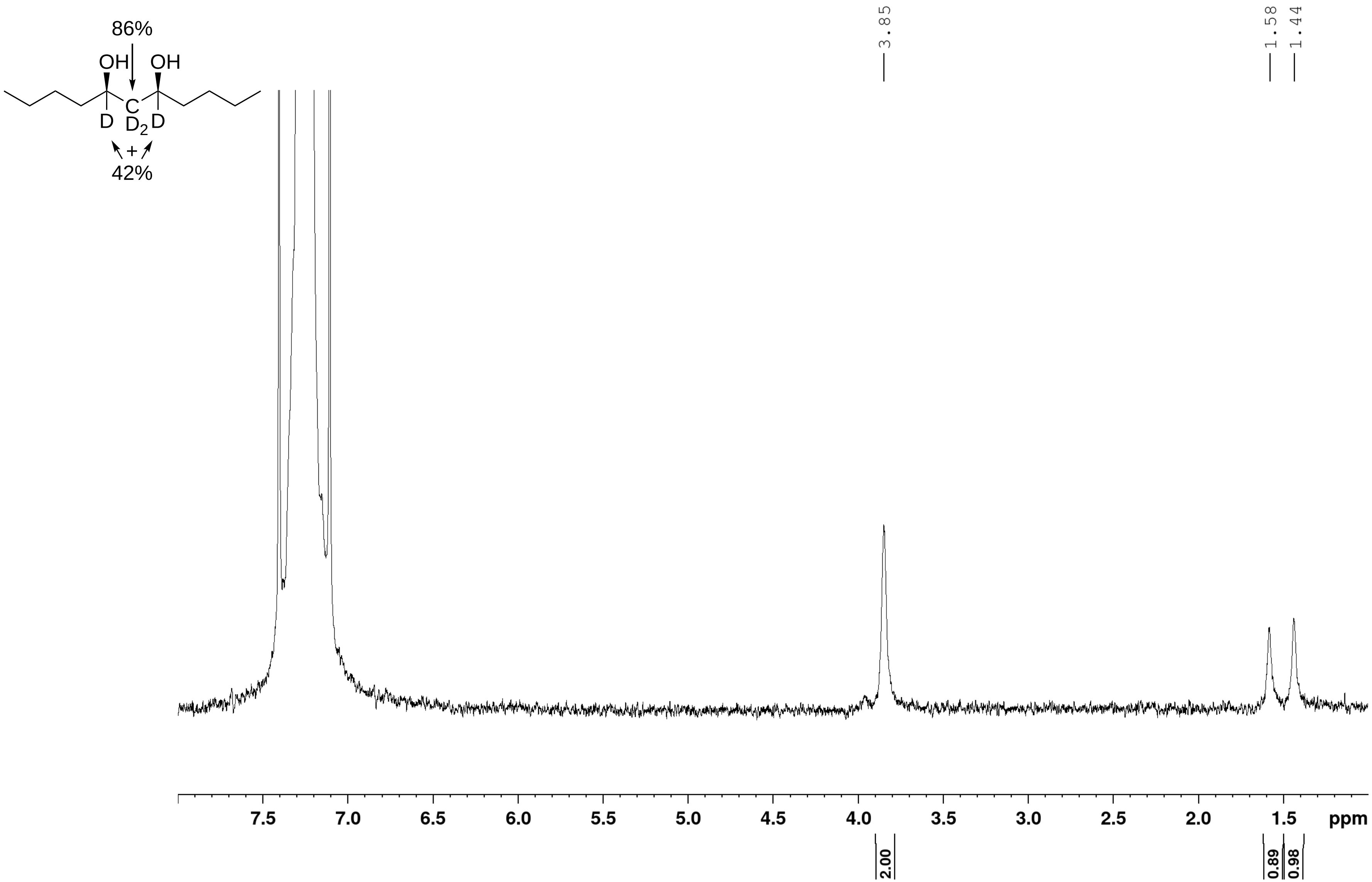

(5*S*,7*S*)-undecane-5,6,6,7-d_4-5,7-diol (1d)

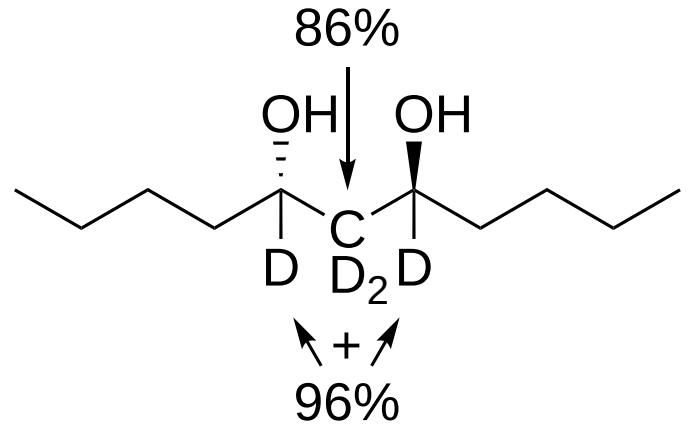

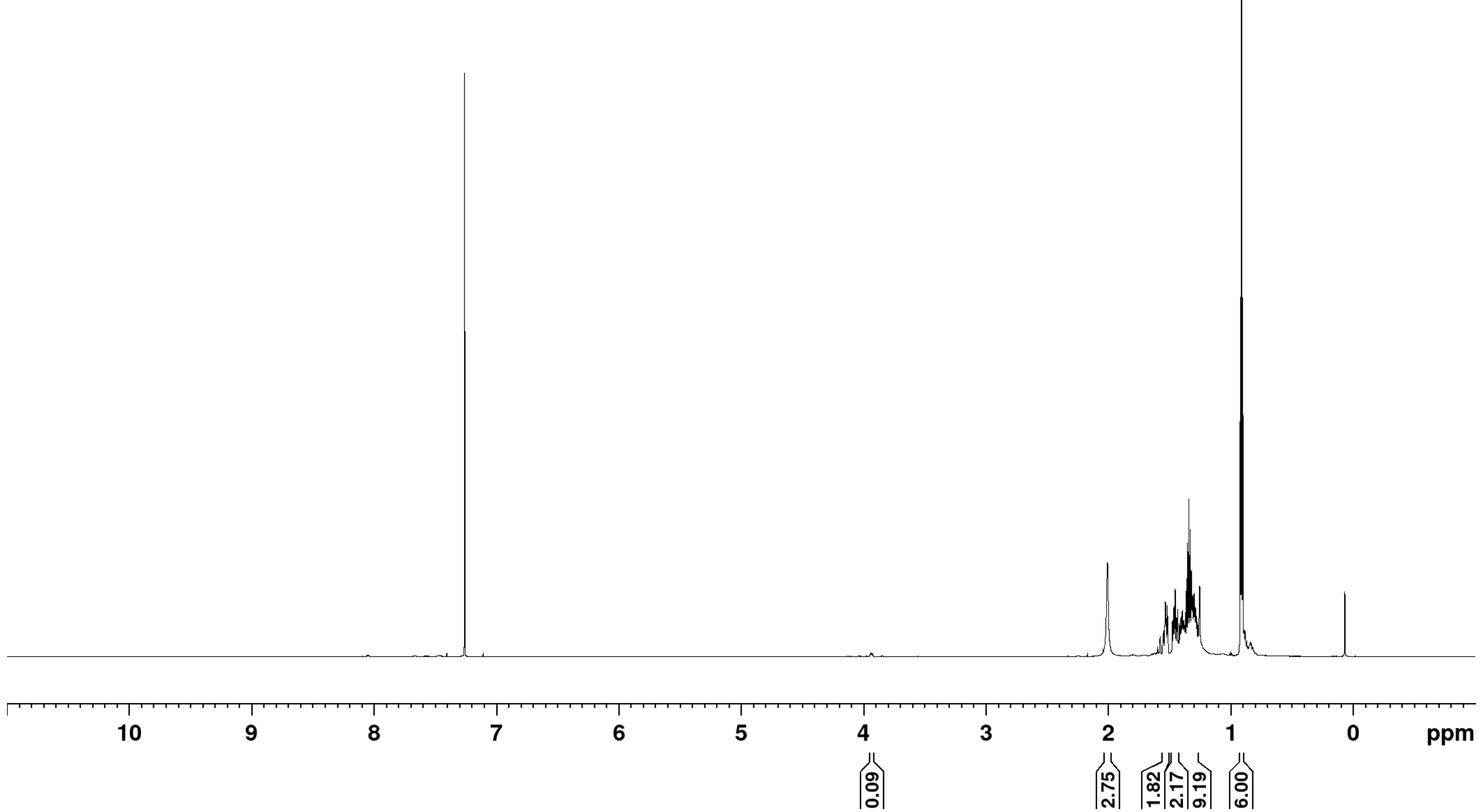

(5*S*,7*S*)-undecane-5,6,6,7-d_4-5,7-diol (1d)

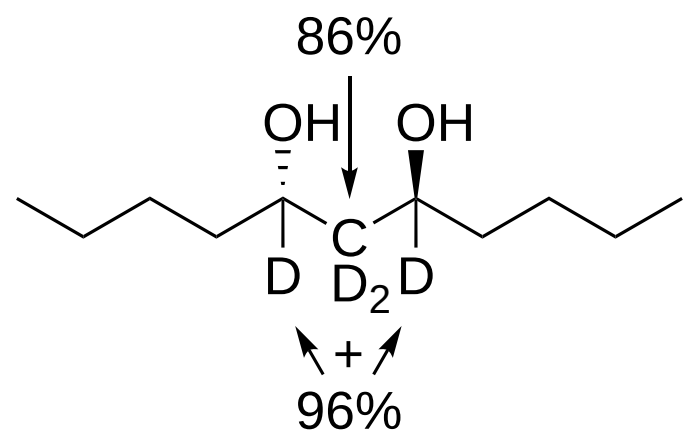

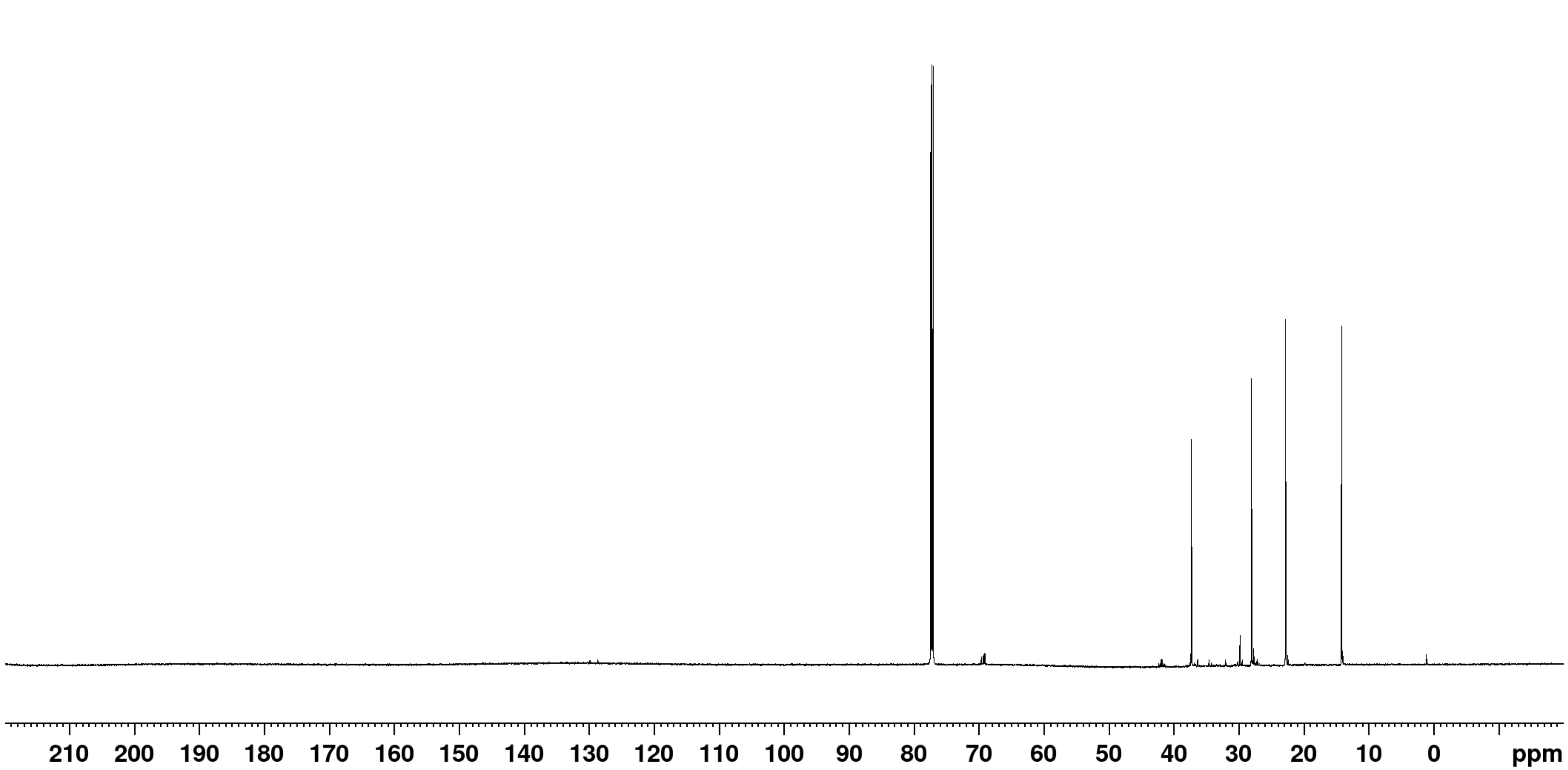

(5*S*,7*S*)-undecane-5,6,6,7-d_4-5,7-diol (1d)

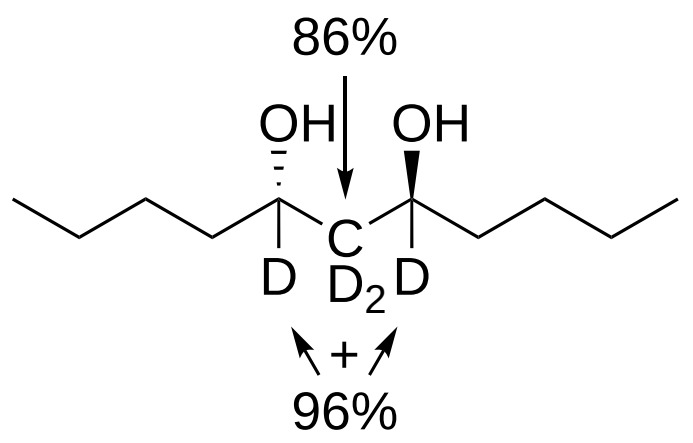

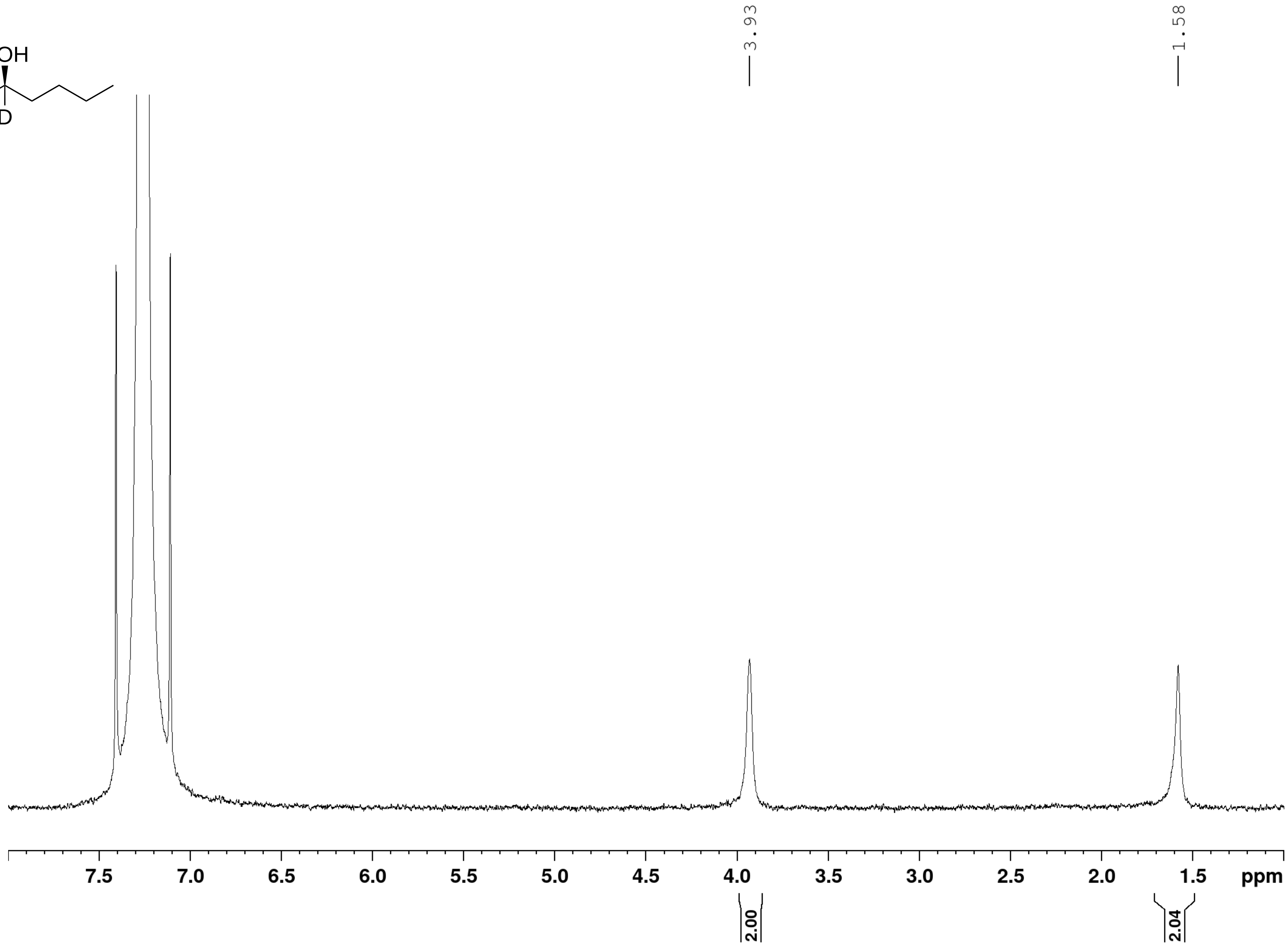

(5*S*,7*S*)-undecane-5,7-diyl-5,6,6,7-d_4 dibenzoate (1d-Bz)

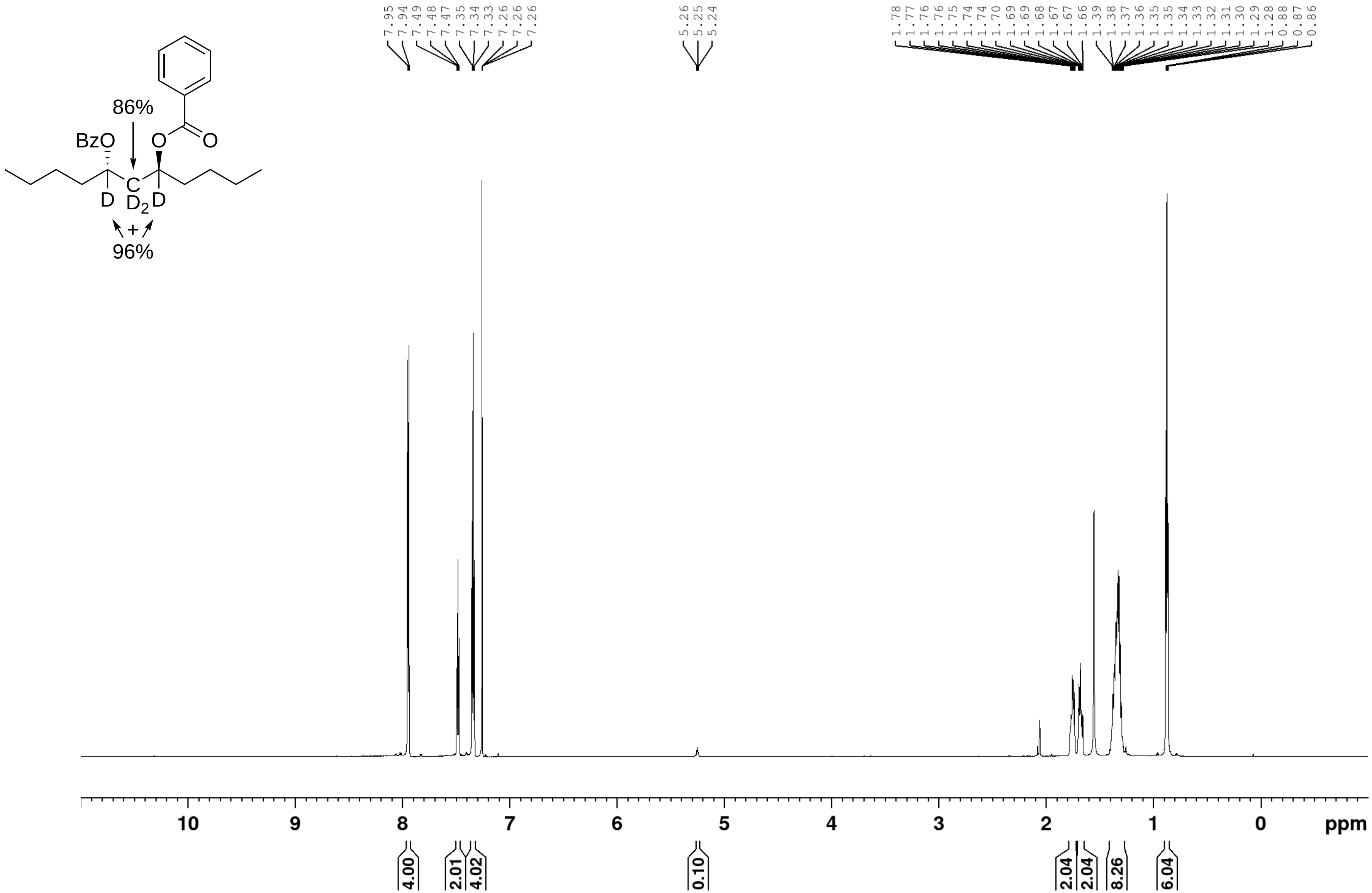

(5*S*,7*S*)-undecane-5,7-diyl-5,6,6,7-d_4 dibenzoate (1d-Bz)

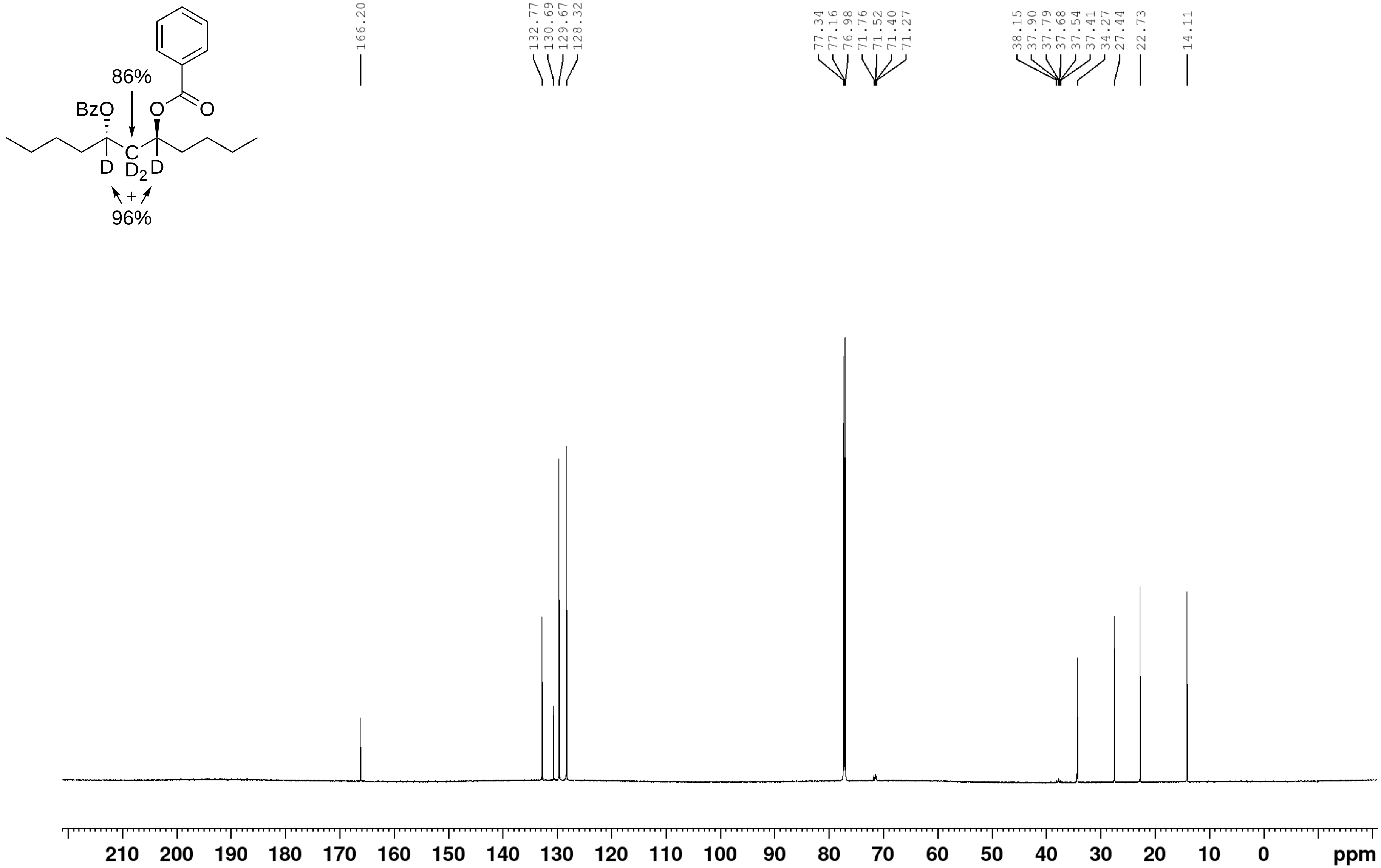

(5*S*,7*S*)-undecane-5,7-diyl-5,6,6,7-d_4 dibenzoate (1d-Bz)

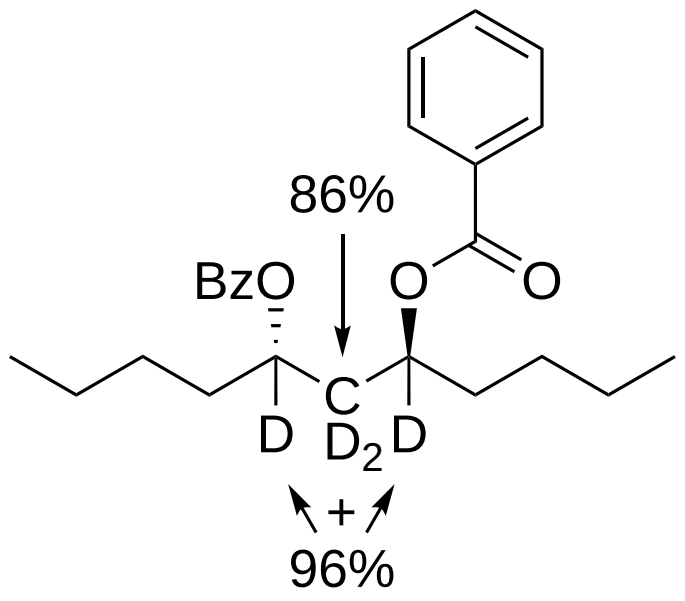

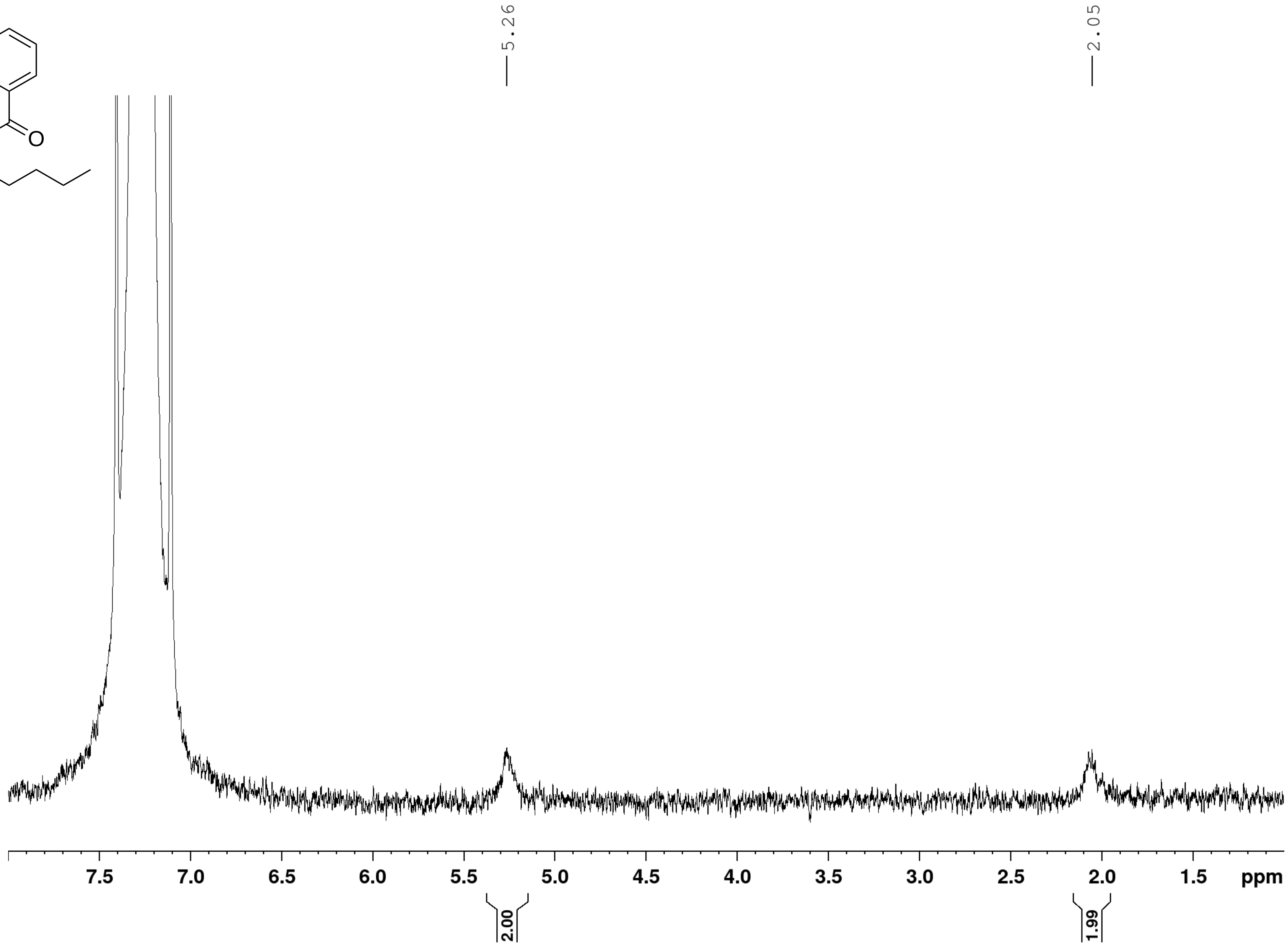

Deuterium study from *meso*-1d-d4

(*syn*)-undecane-5,6,6,7-d_4-5,7-diol (*meso*-1d)

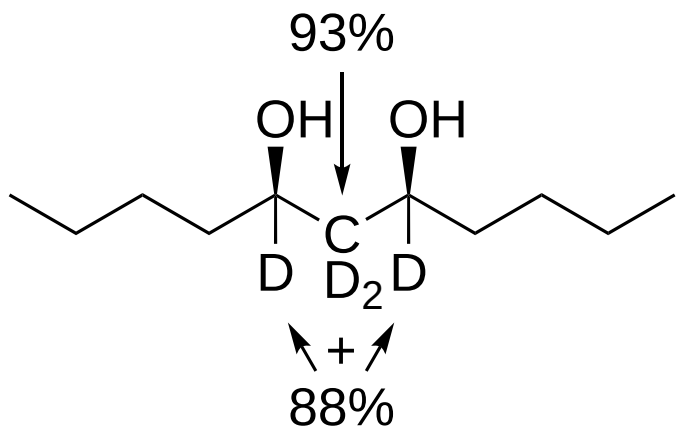

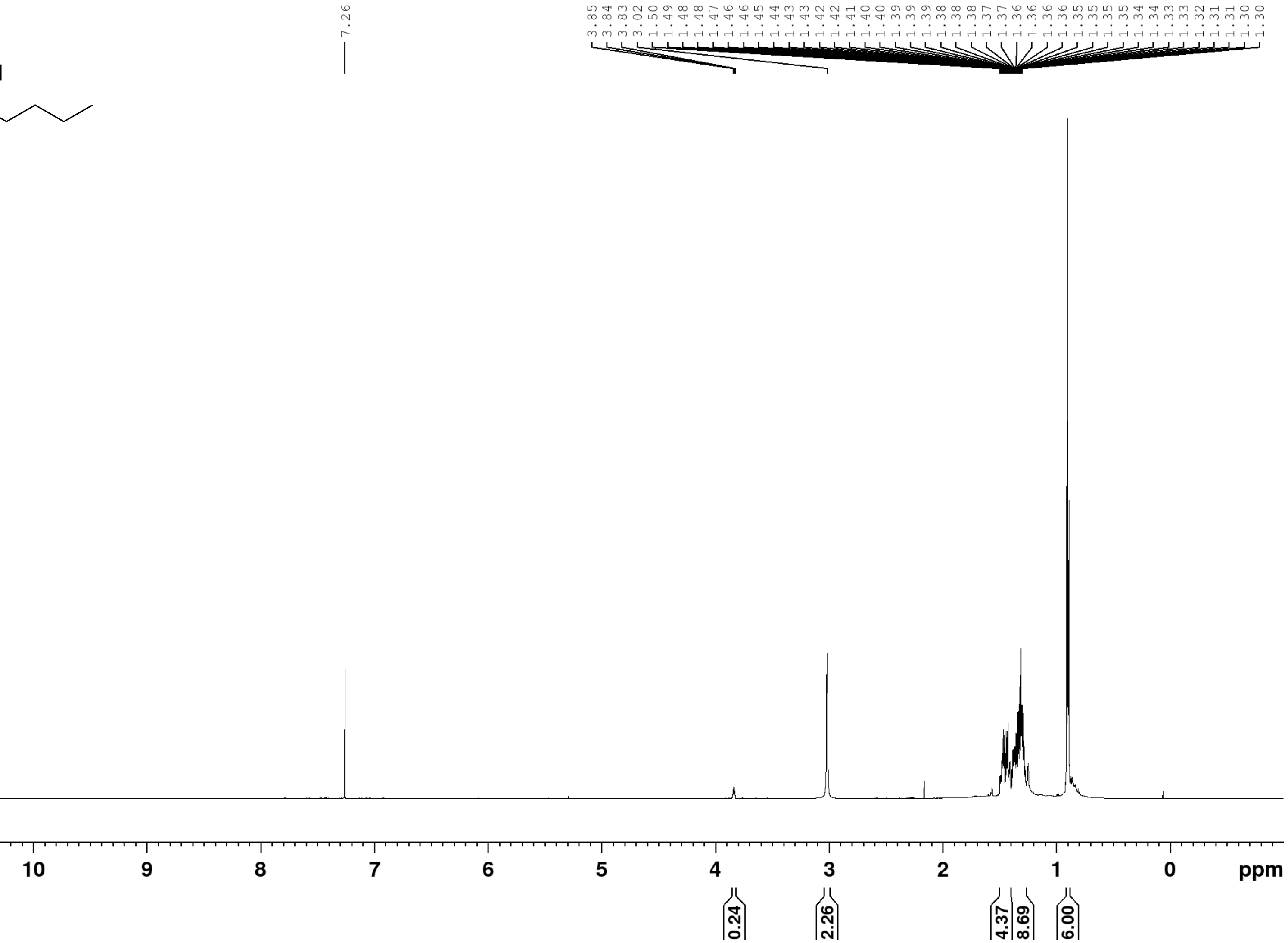

(*syn*)-undecane-5,6,6,7-d_4-5,7-diol (*meso*-1d)

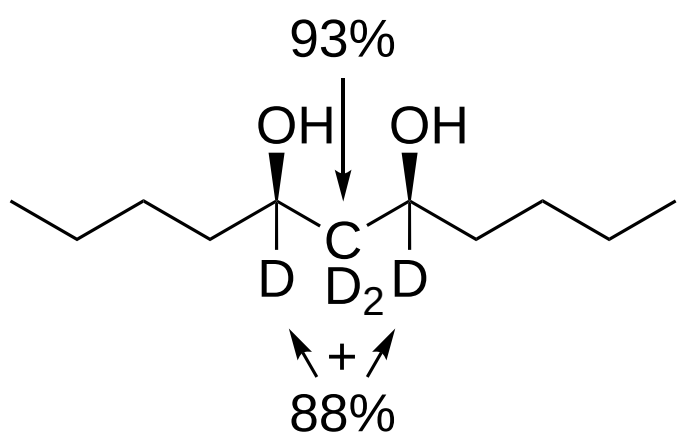

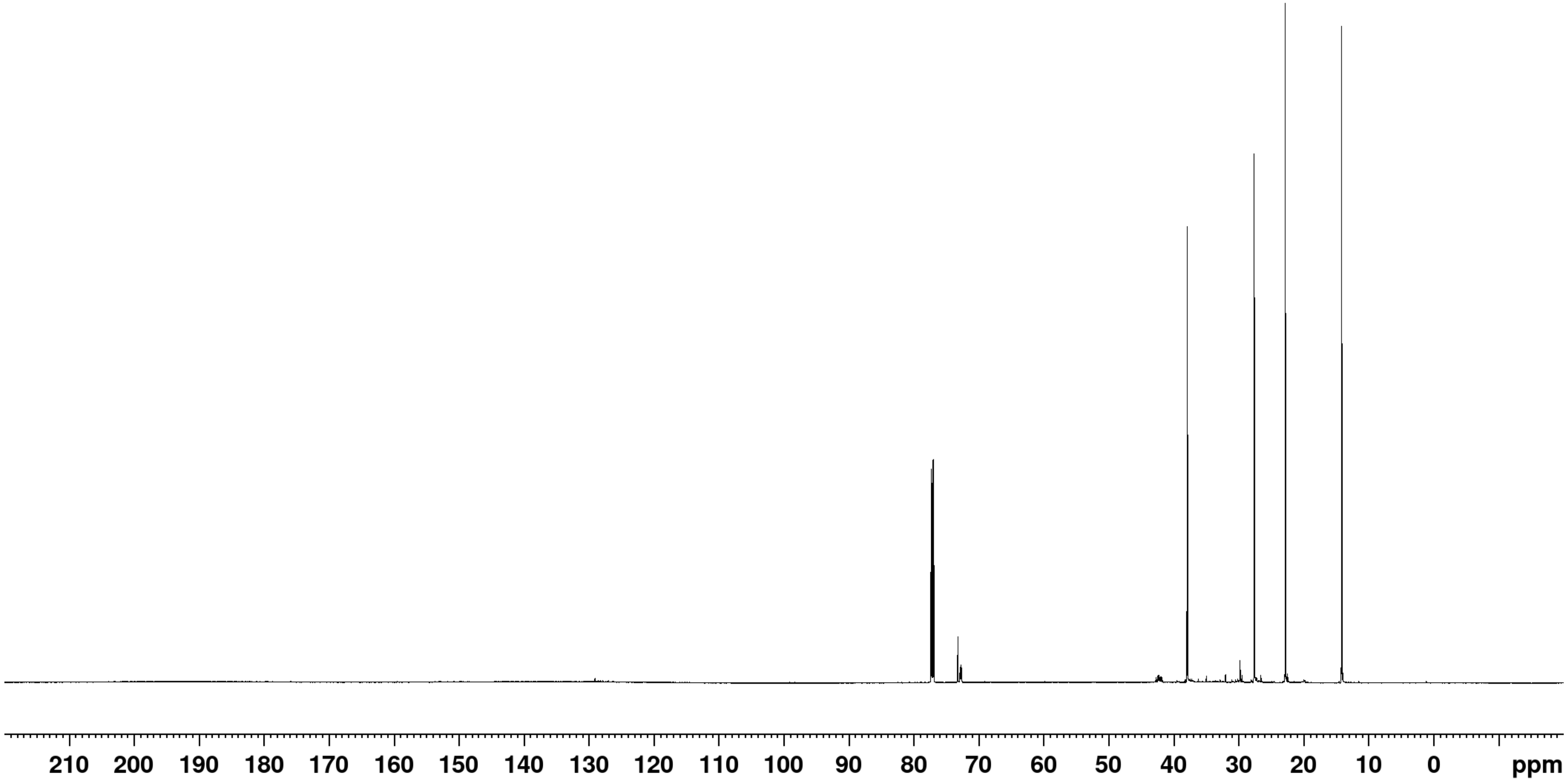

(*syn*)-undecane-5,6,6,7-d_4-5,7-diol (*meso*-1d)

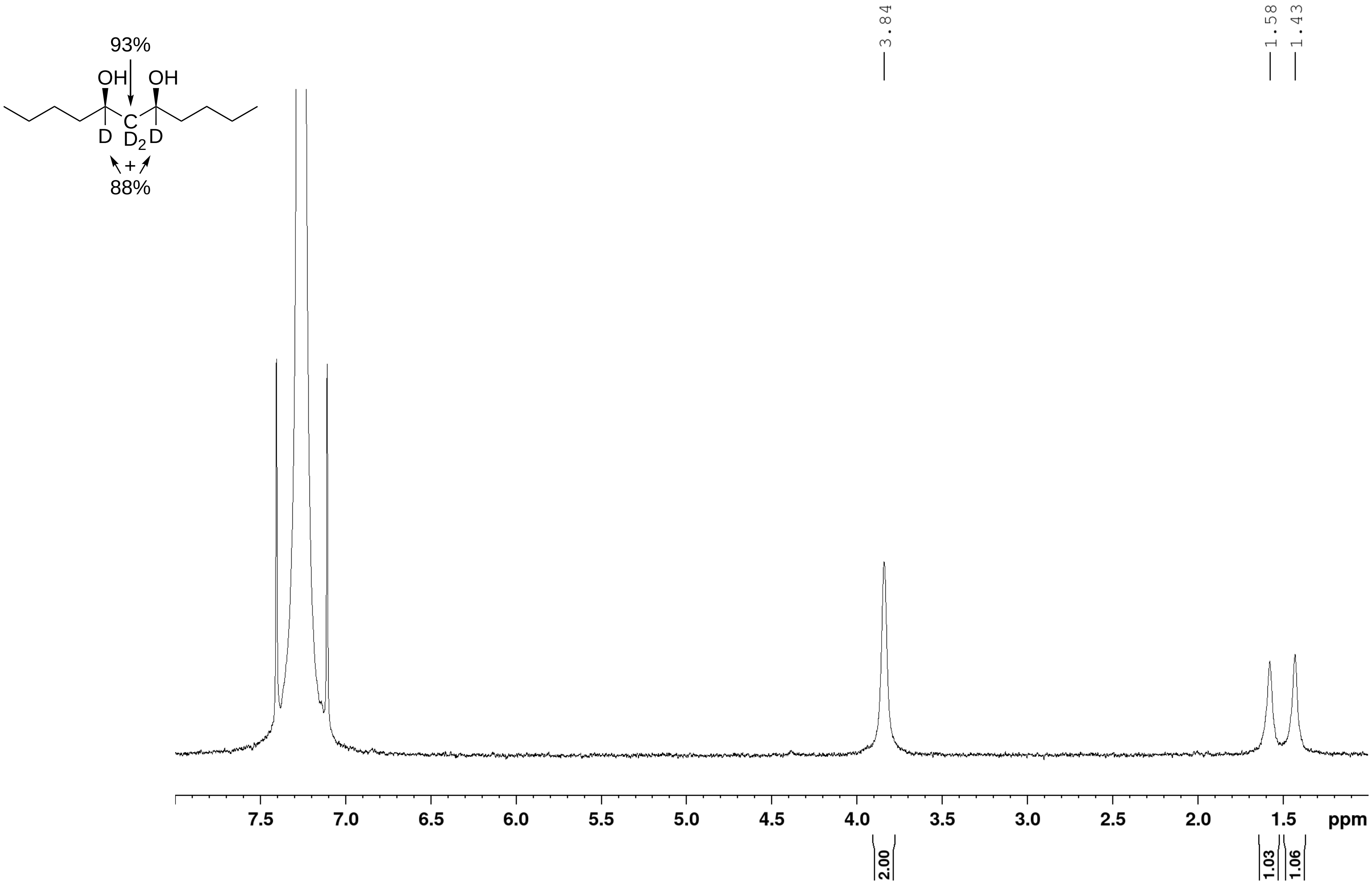

(5*S*,7*S*)-undecane-5,6,6,7-d_4-5,7-diol (1d)

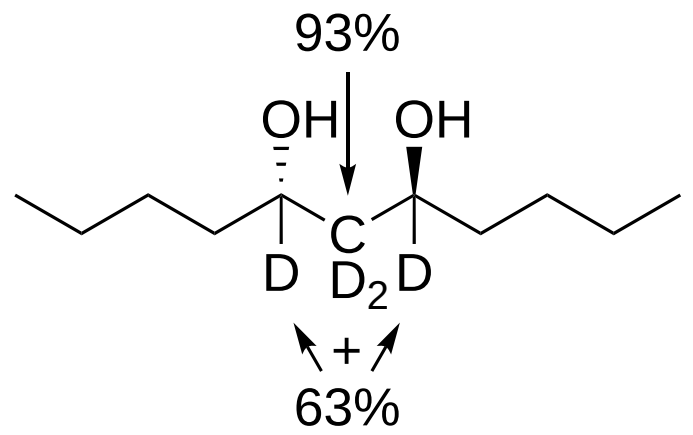

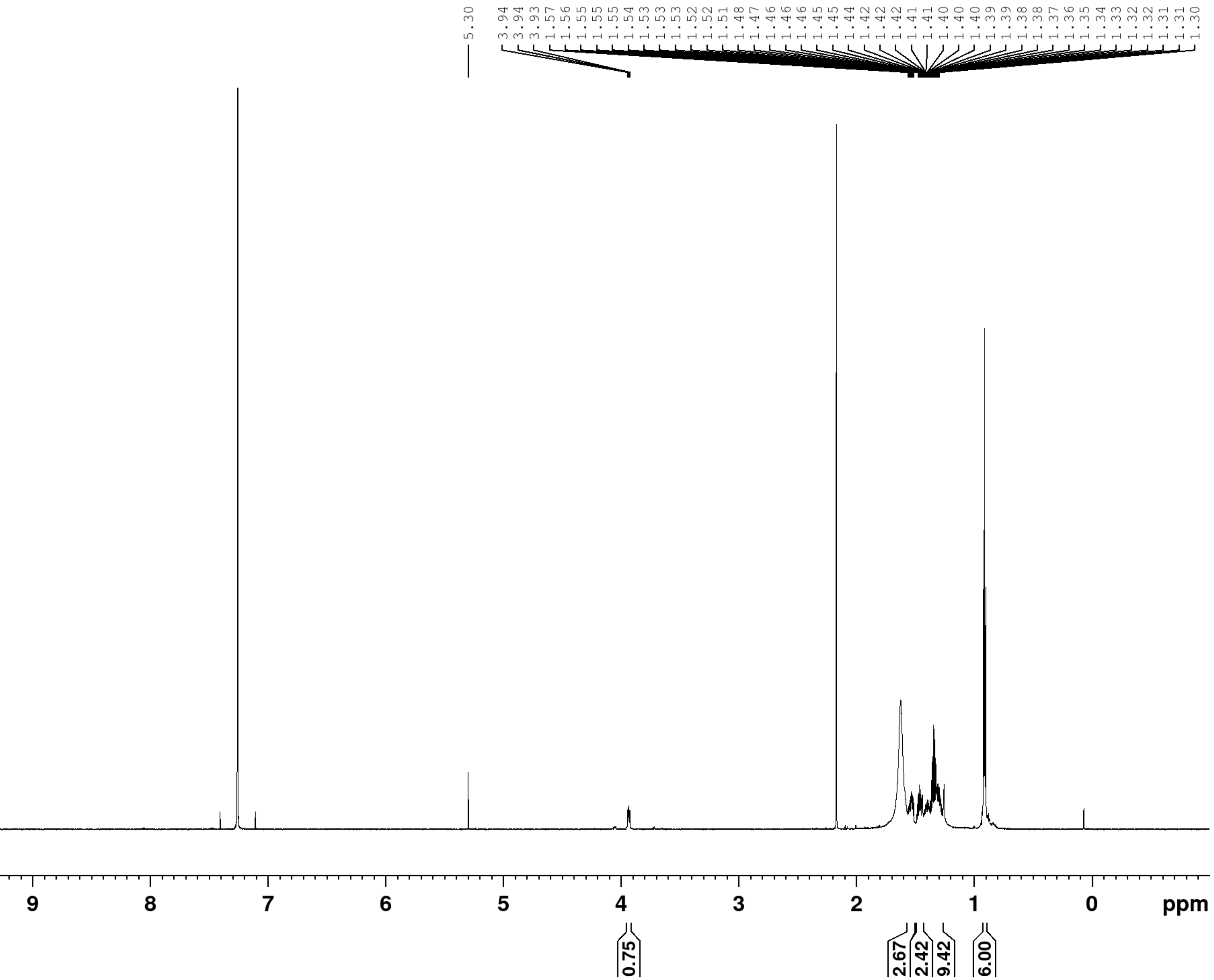

(5*S*,7*S*)-undecane-5,6,6,7-d_4-5,7-diol (1d)

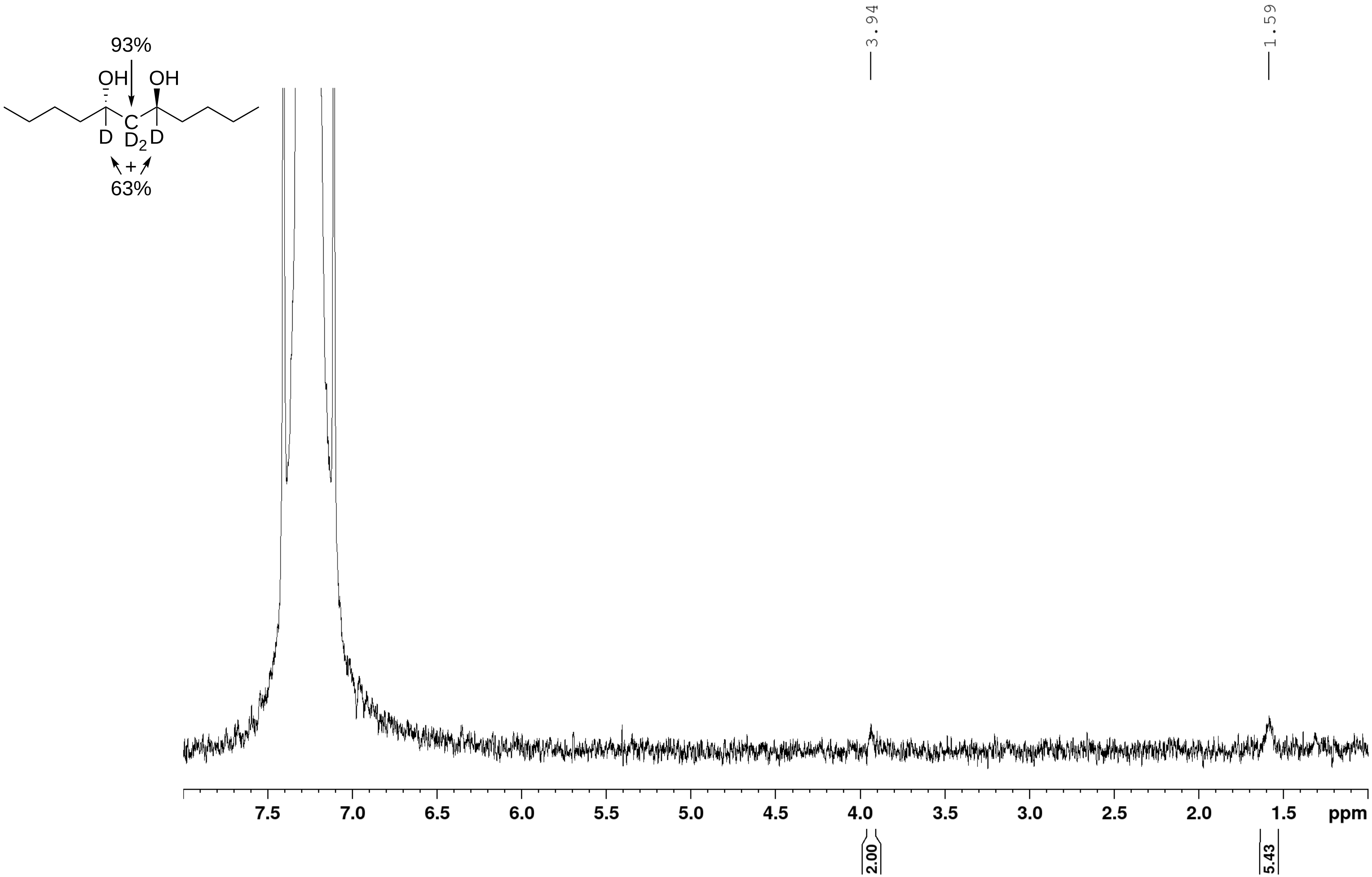

(5*S*,7*S*)-undecane-5,7-diyl-5,6,6,7-d_4 dibenzoate (1d-Bz)

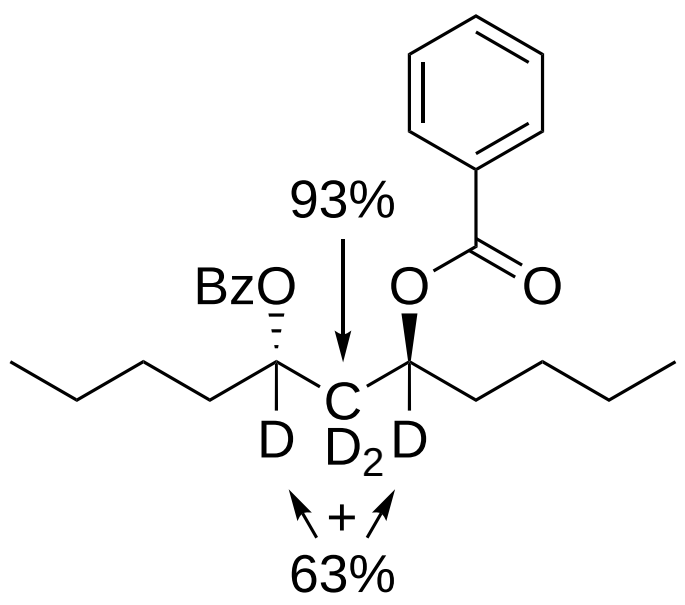

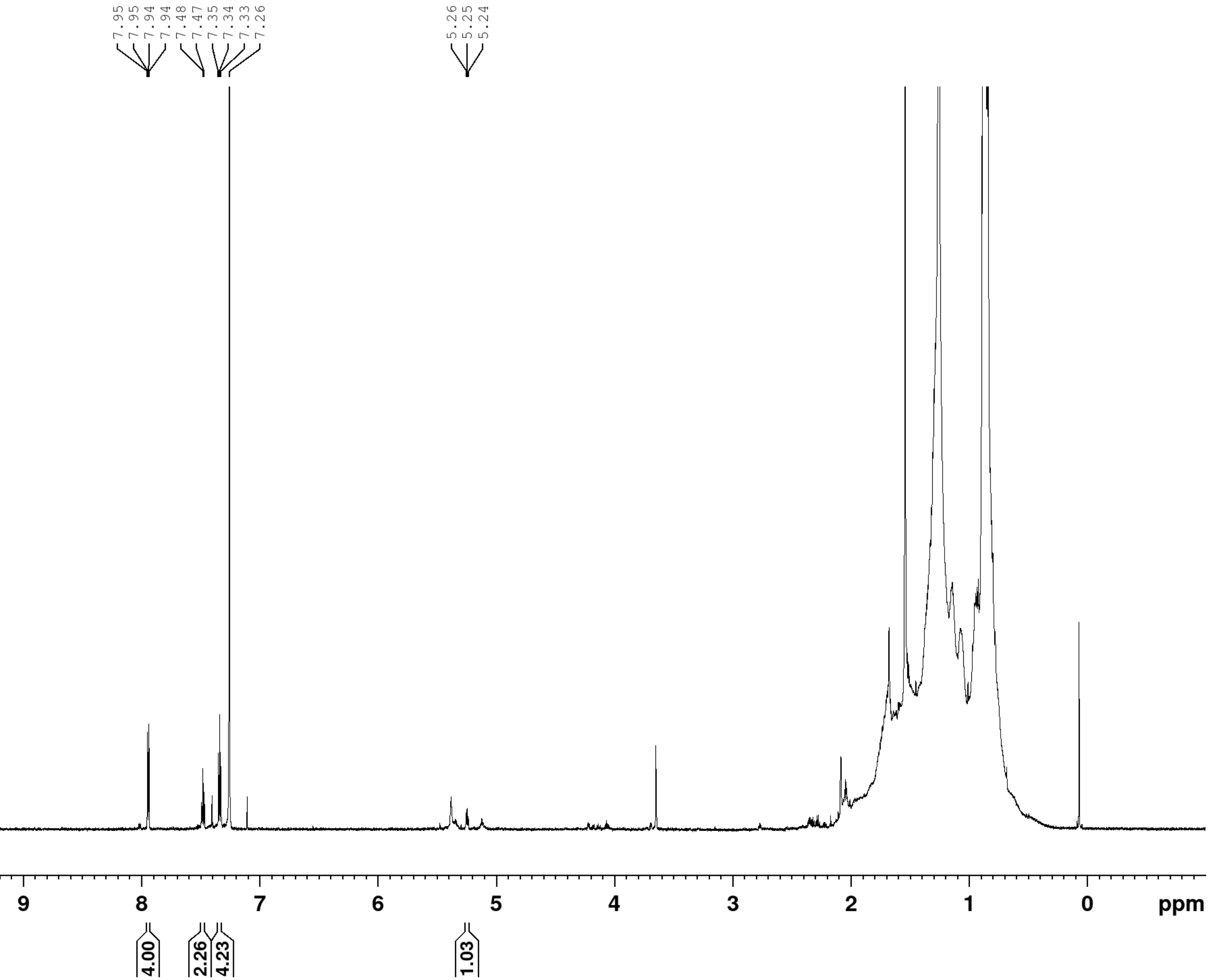

(5*S*,7*S*)-undecane-5,7-diyl-5,6,6,7-d_4 dibenzoate (1d-Bz)

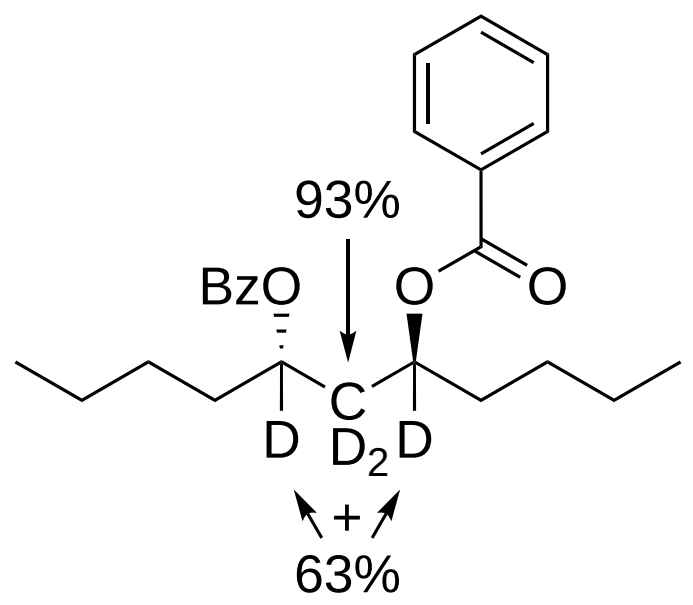

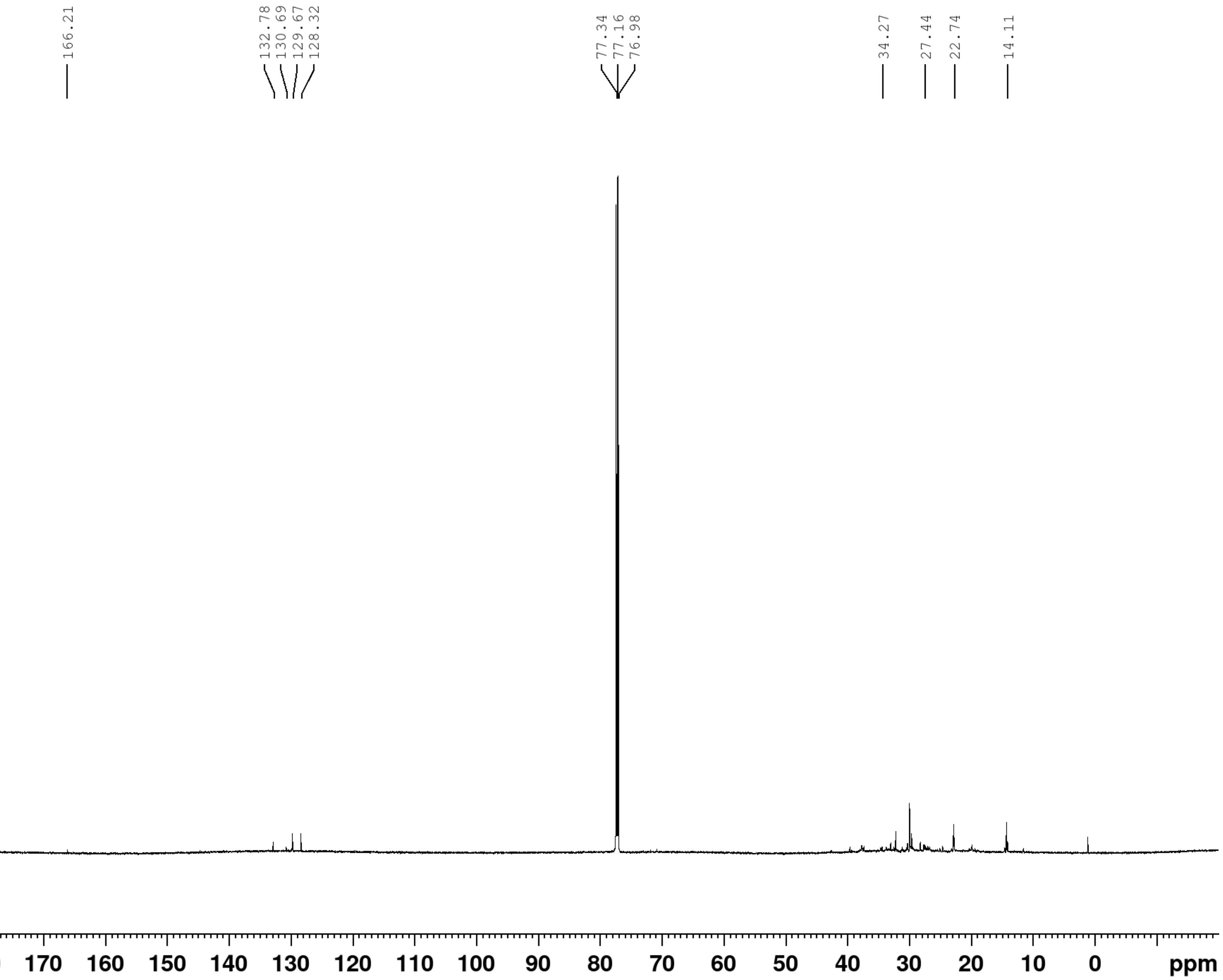

(5*S*,7*S*)-undecane-5,7-diyl-5,6,6,7-d_4 dibenzoate (1d-Bz)

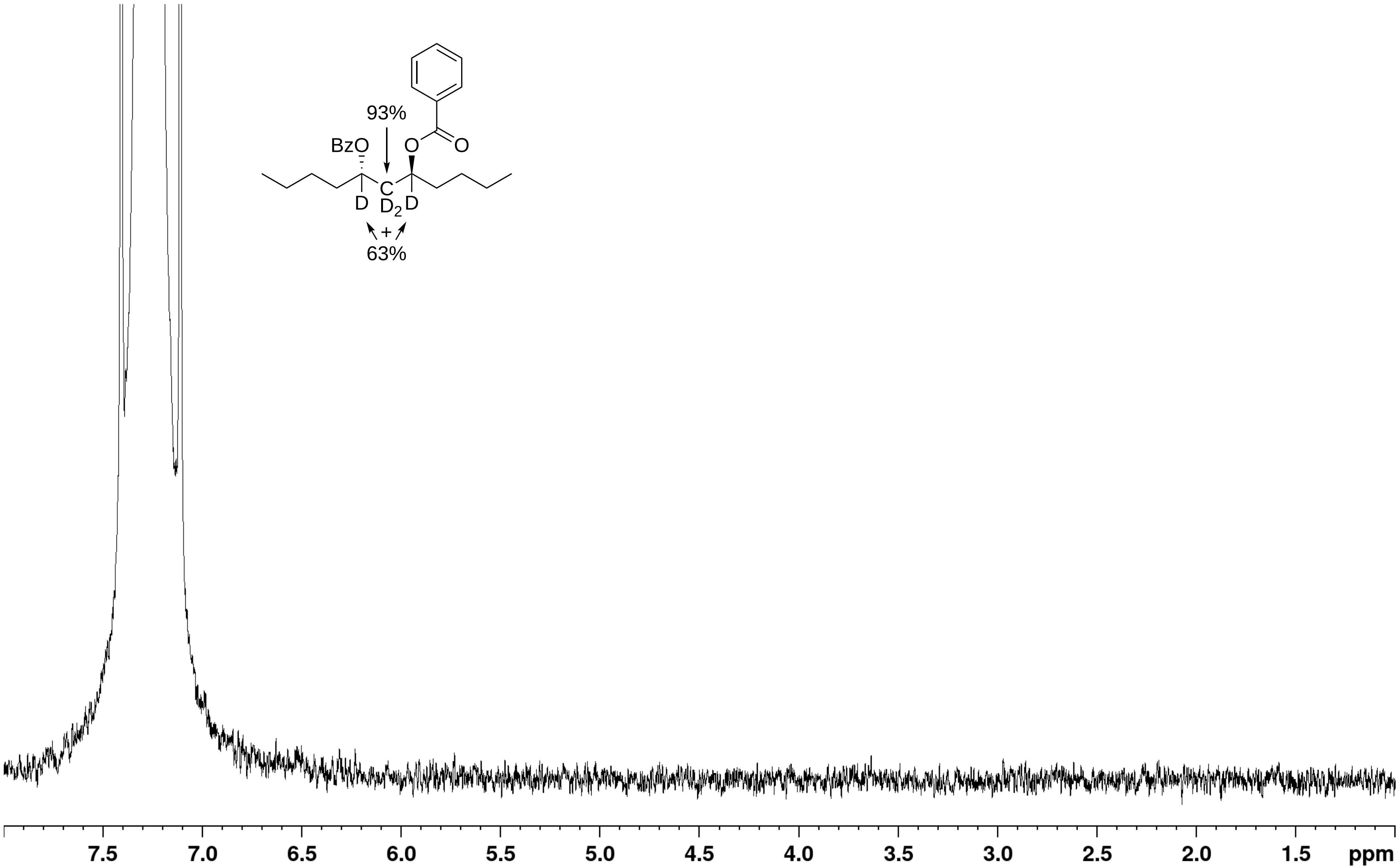

Giese reaction addition (Scheme 3D, upper) starting from *rac*-1d:

(5*S*,7*S*)-undecane-5,7-diol (1d) with *N*-phenylacrylamide impurity

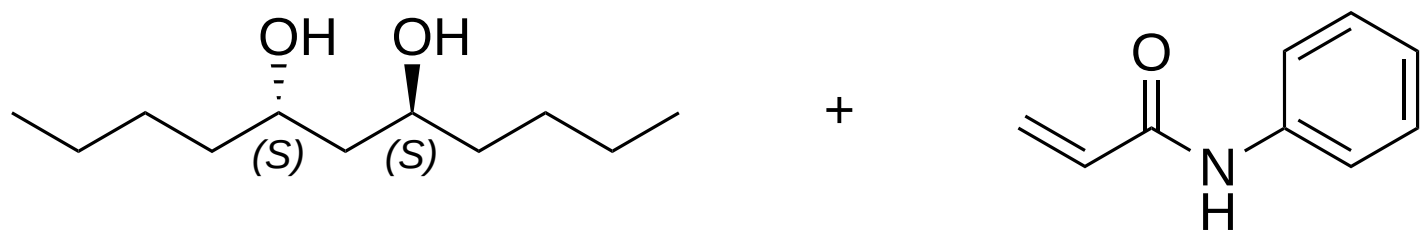

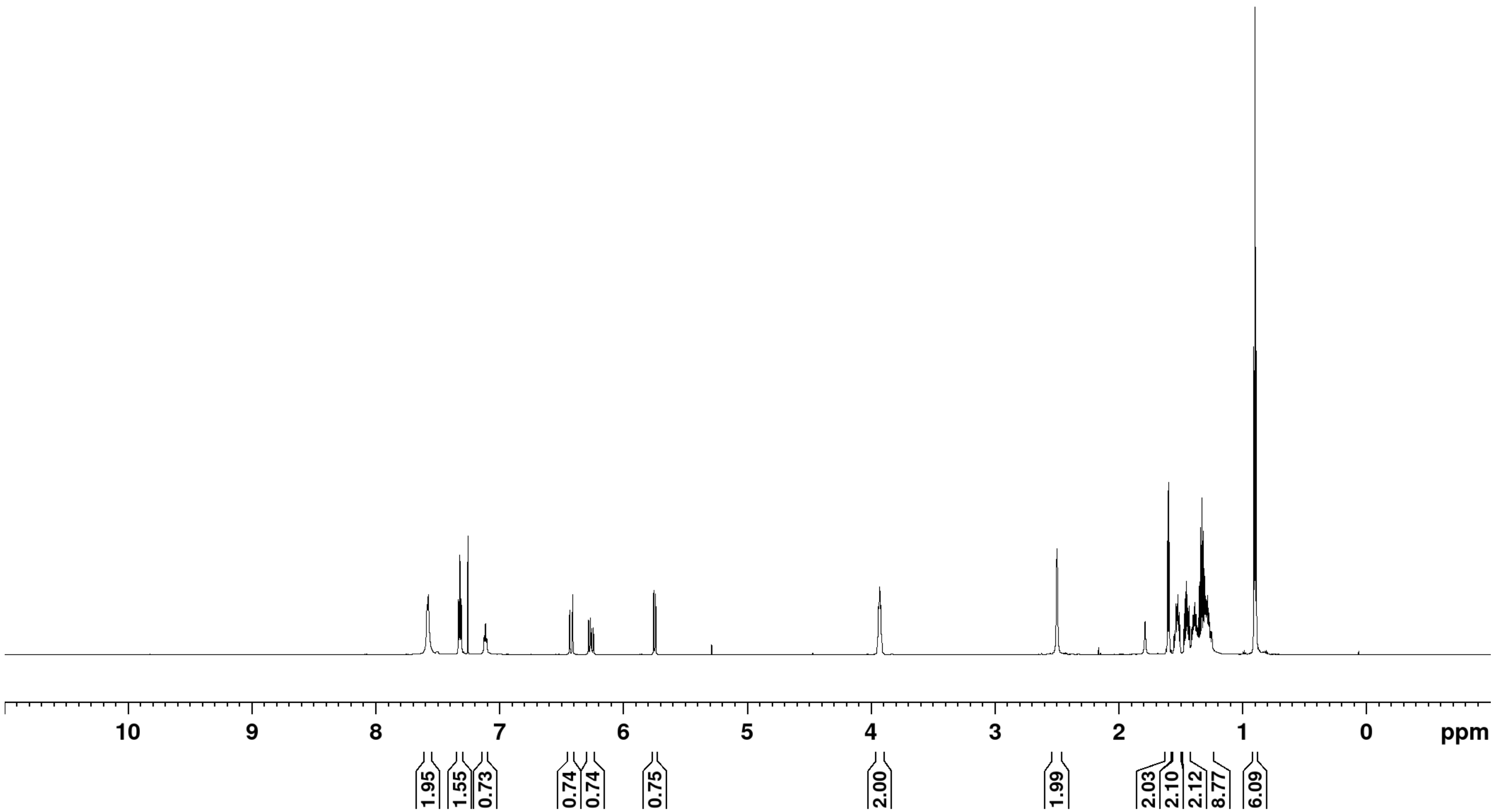

(5*S*,7*S*)-undecane-5,7-diol (1d) with *N*-phenylacrylamide impurity

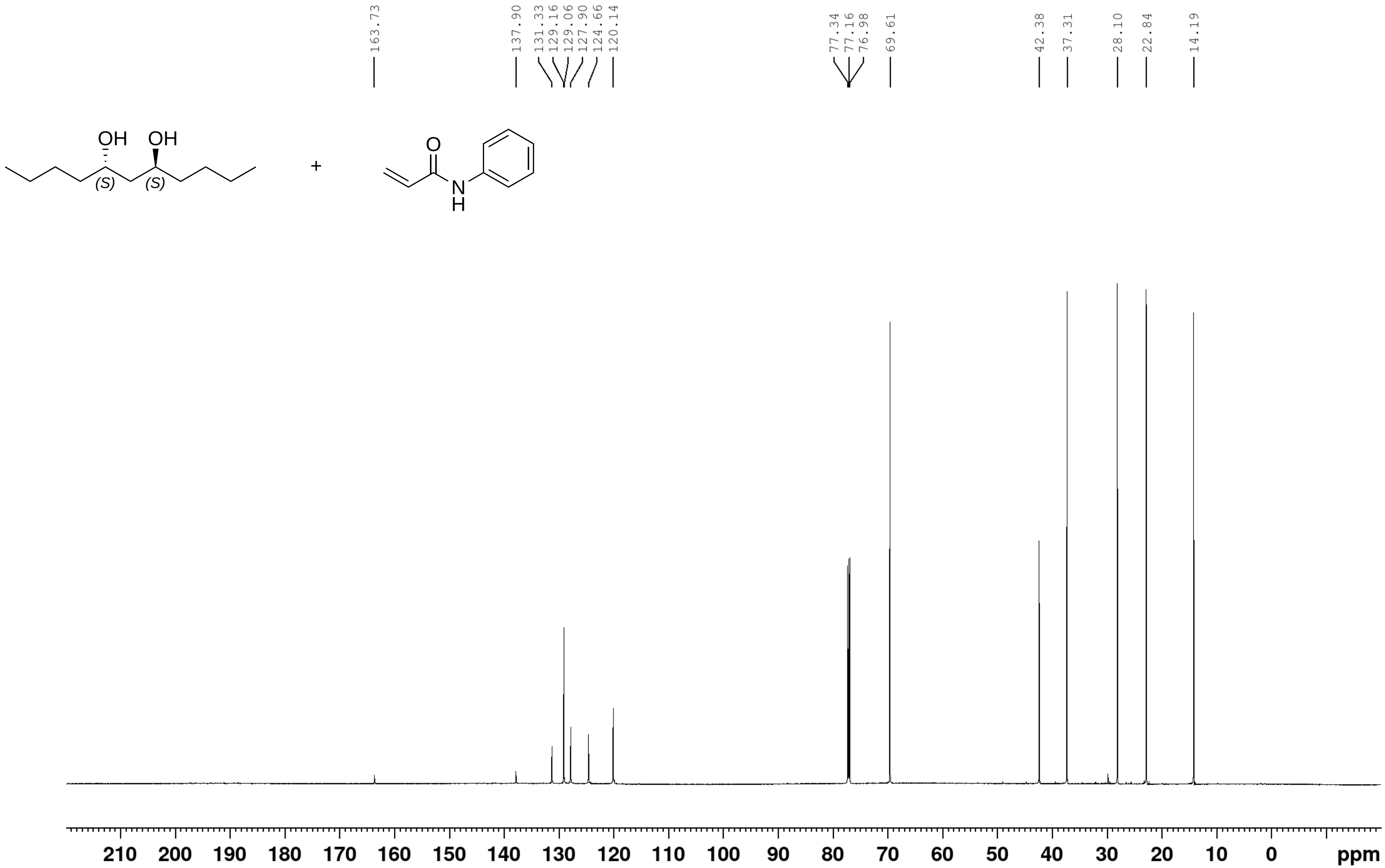

(5*S*,7*S*)-undecane-5,7-diyl dibenzoate (1d-Bz)

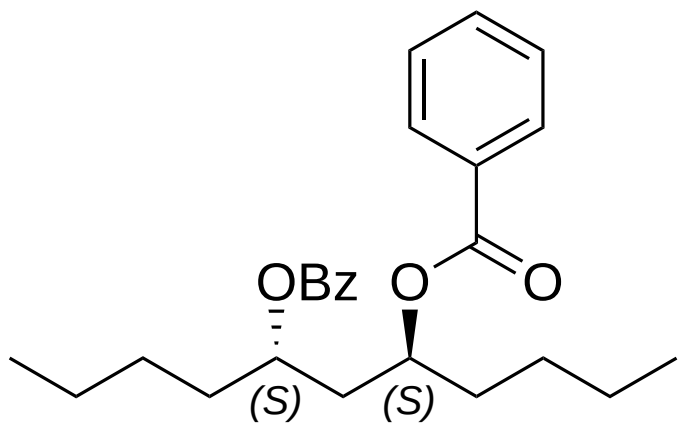

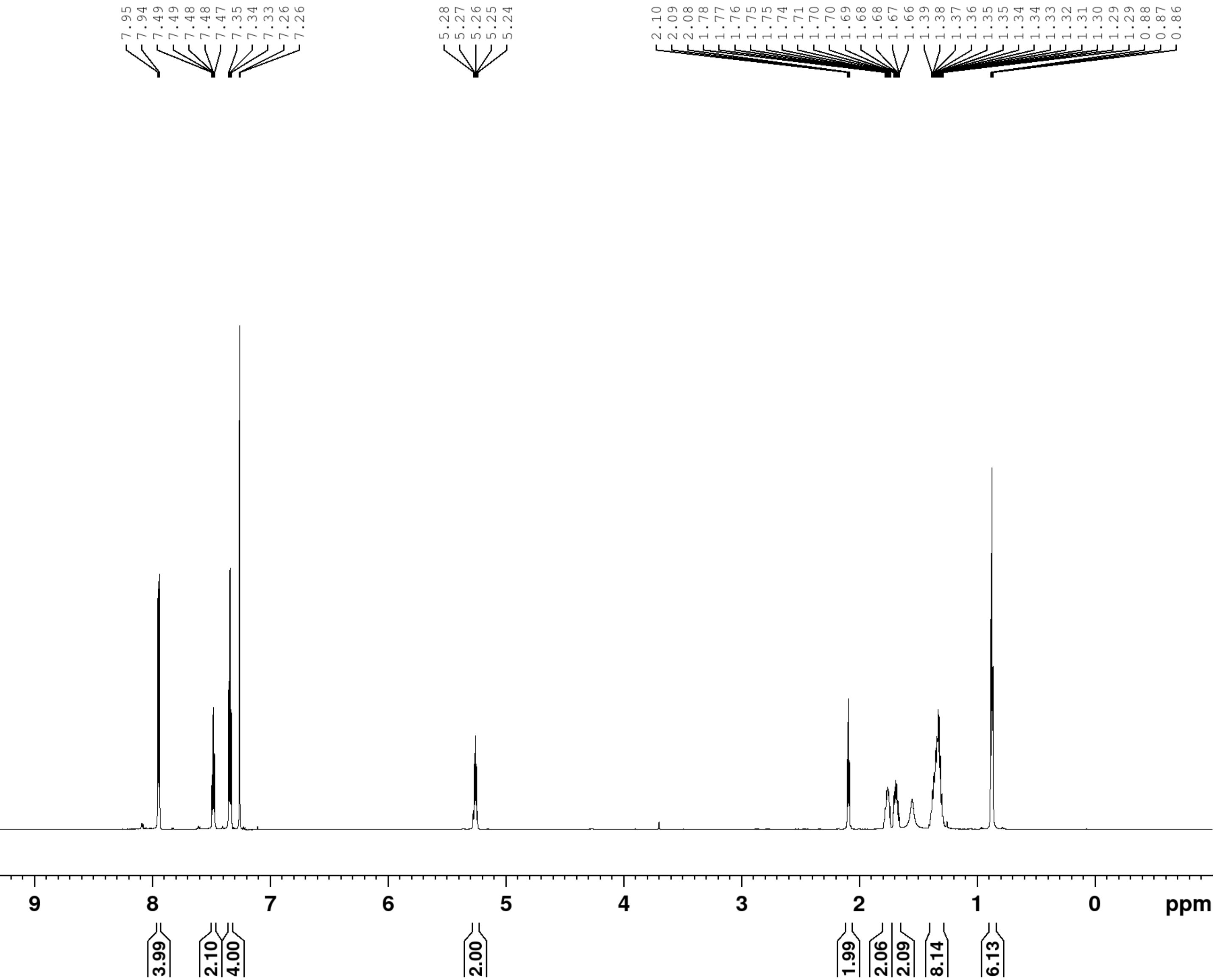

(5*S*,7*S*)-undecane-5,7-diyl dibenzoate (1d-Bz)

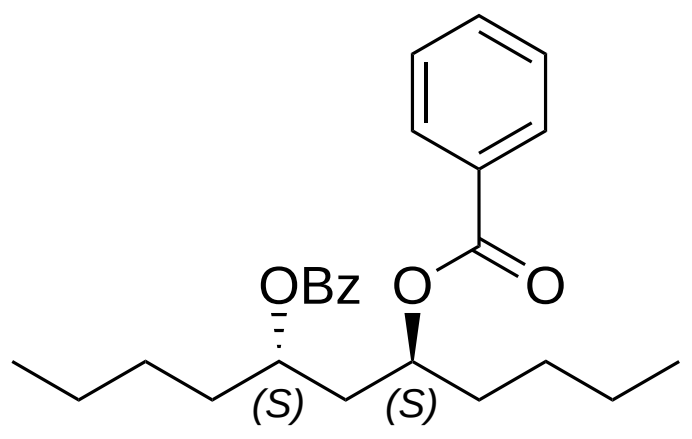

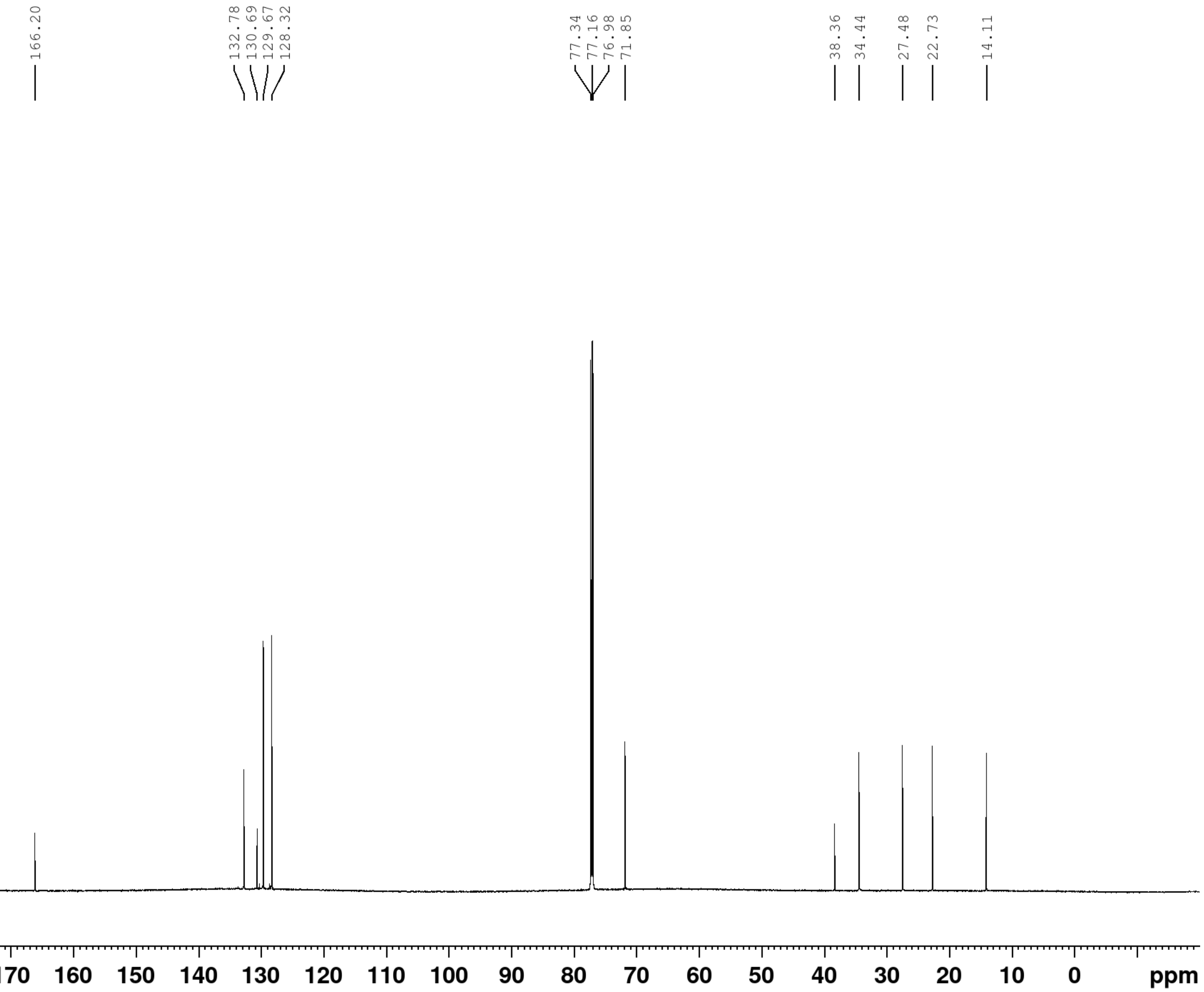

(6*R*)-4-butyl-4,6-dihydroxy-*N*-phenyldecanamide (2b)

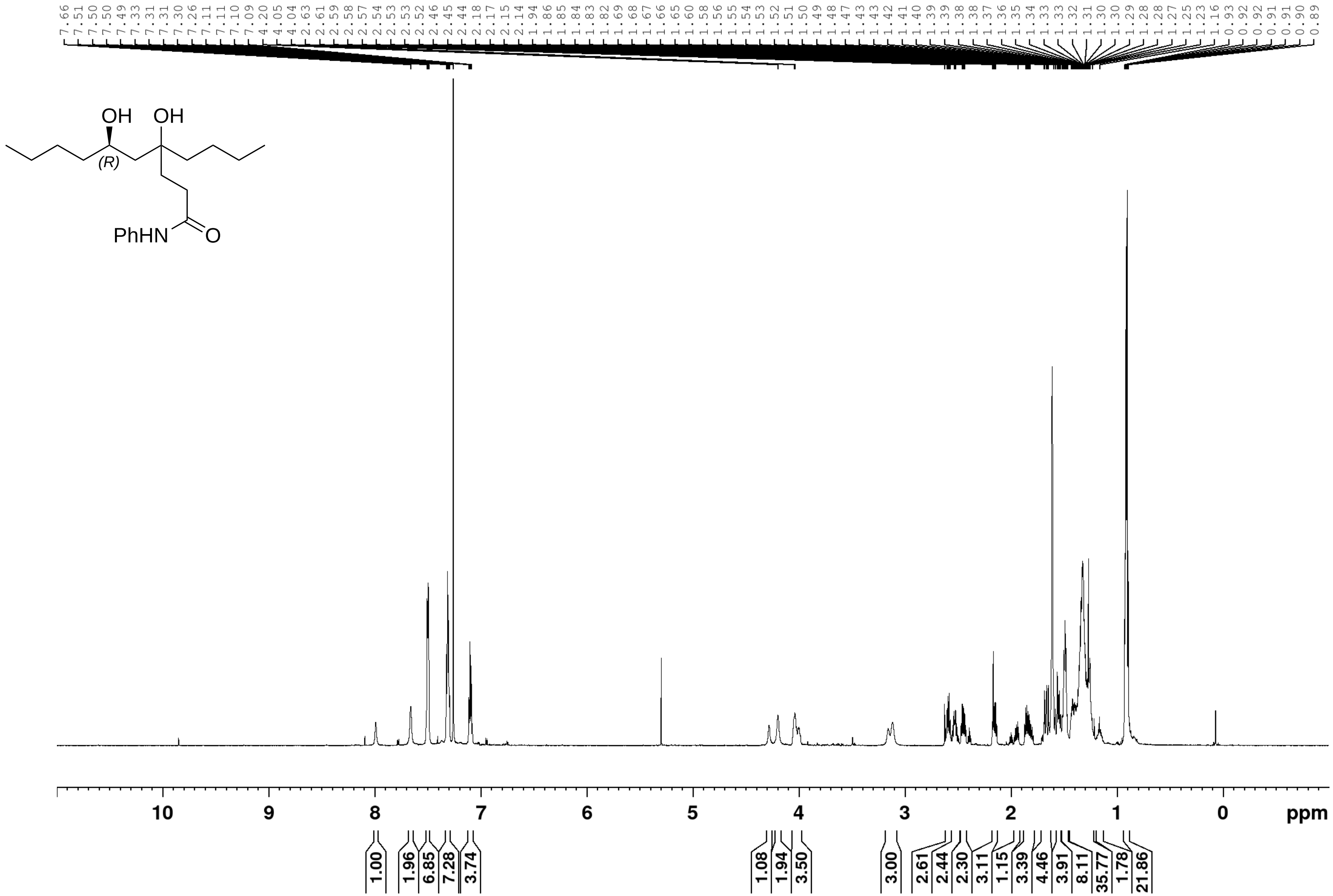

(6*R*)-4-butyl-4,6-dihydroxy-*N*-phenyldecanamide (2b)

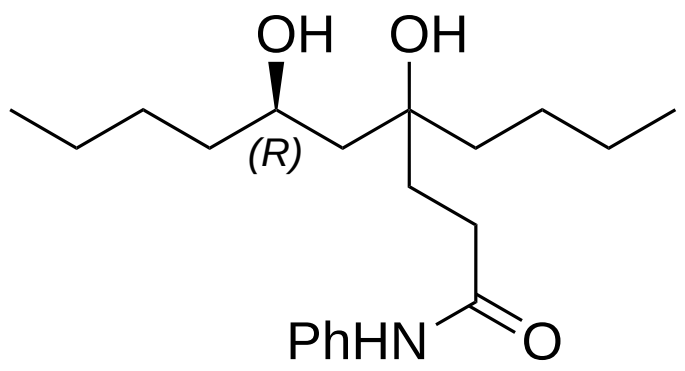

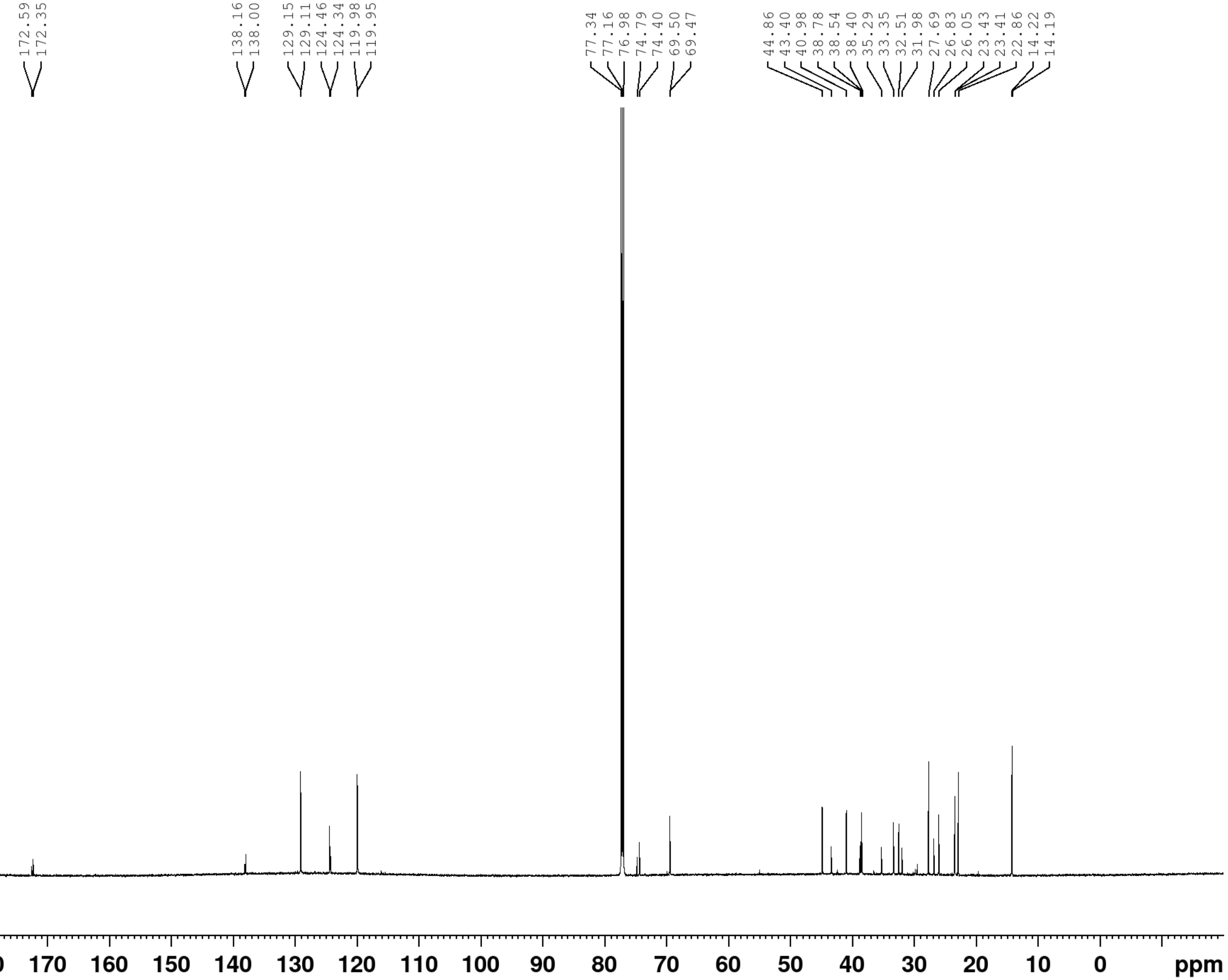

Giese reaction addition starting from *meso*-1d:

(6*S*)-4-butyl-4,6-dihydroxy-*N*-phenyldecanamide ((*S*)-2b)

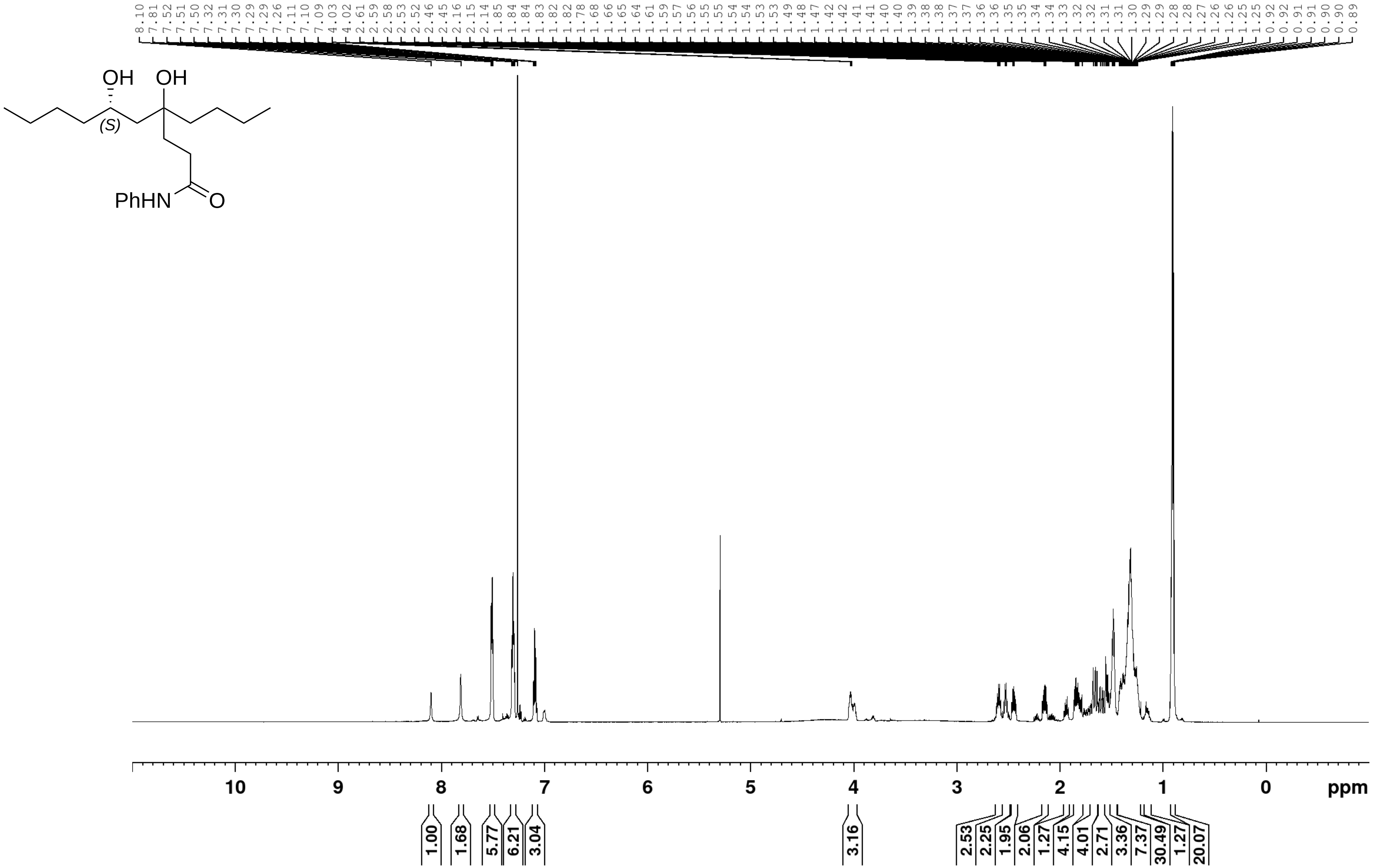

(6*S*)-4-butyl-4,6-dihydroxy-*N*-phenyldecanamide ((*S*)-2b)

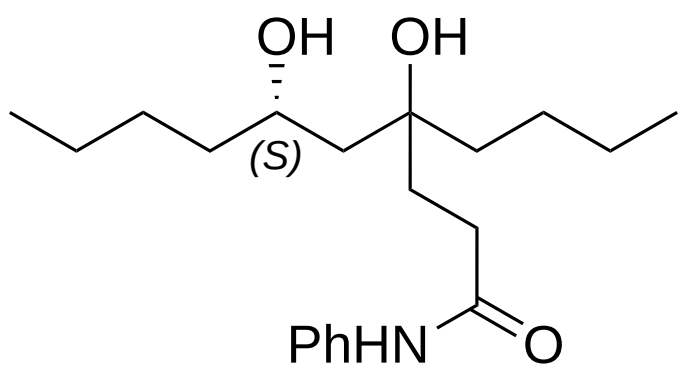

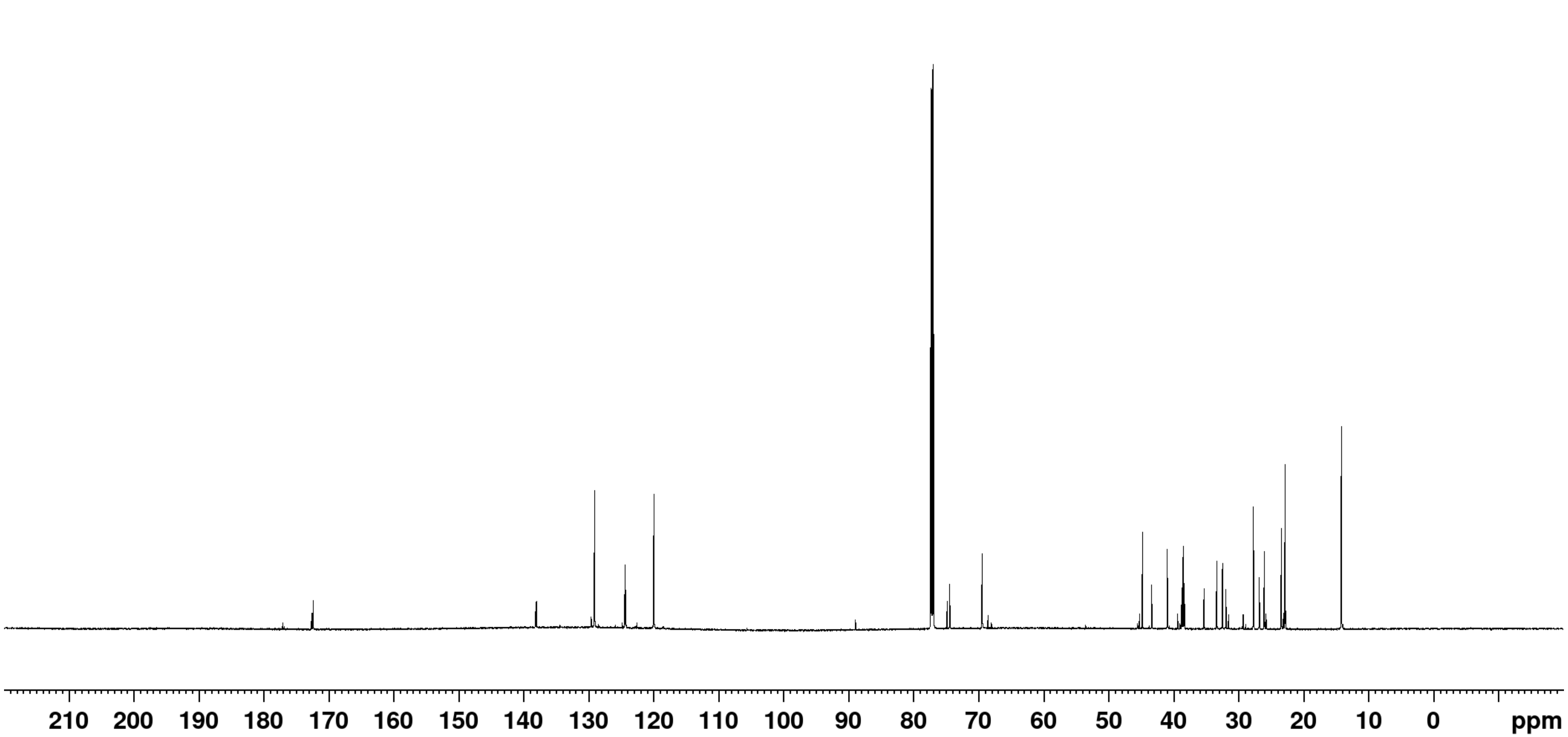

Oxidation reaction with DIAD starting from *rac*-1d:

(5*S*,7*S*)-undecane-5,7-diol (1d)

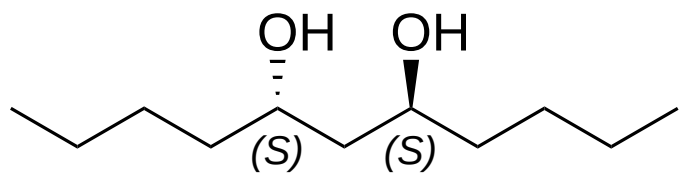

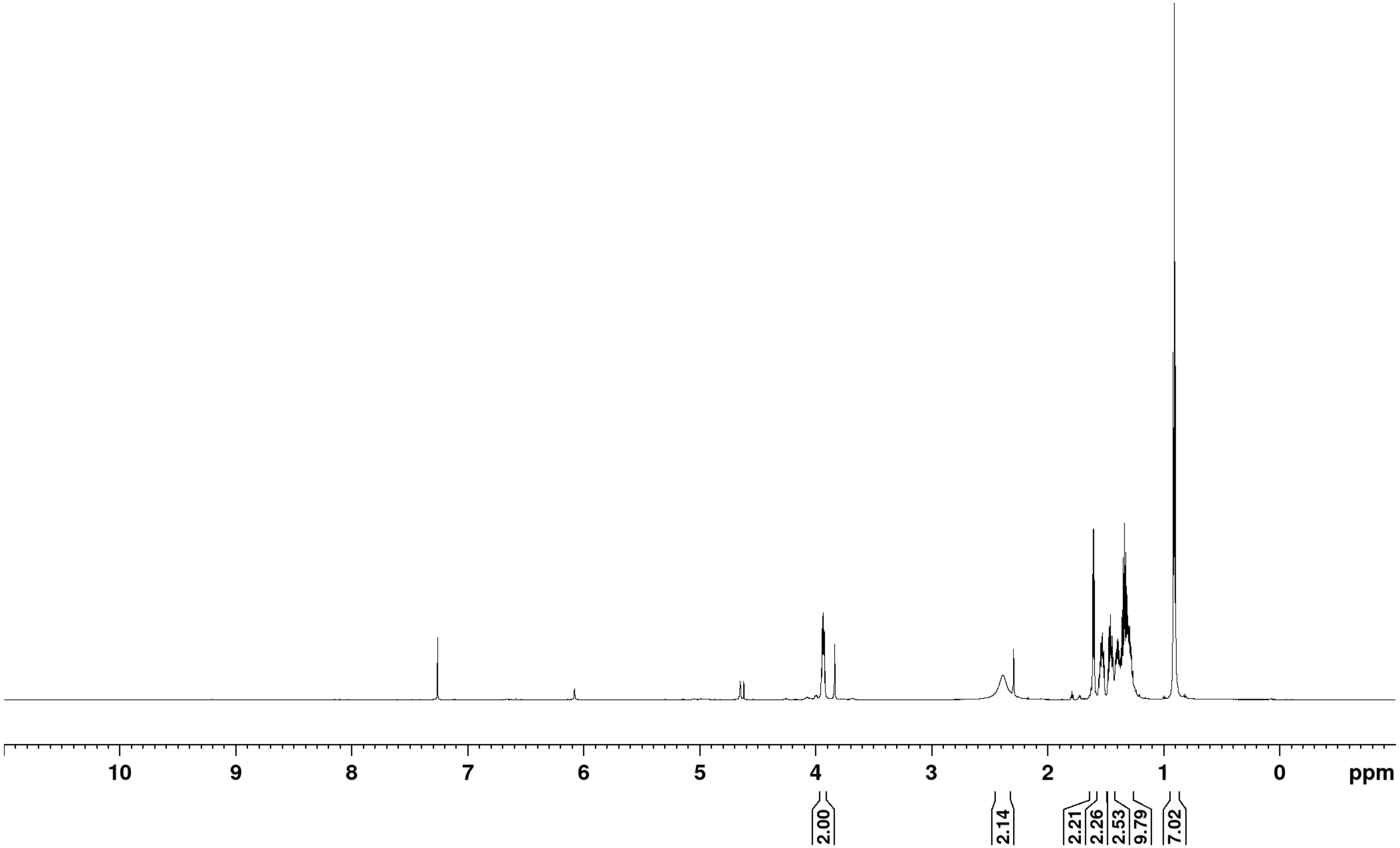

(5*S*,7*S*)-undecane-5,7-diol (1d)

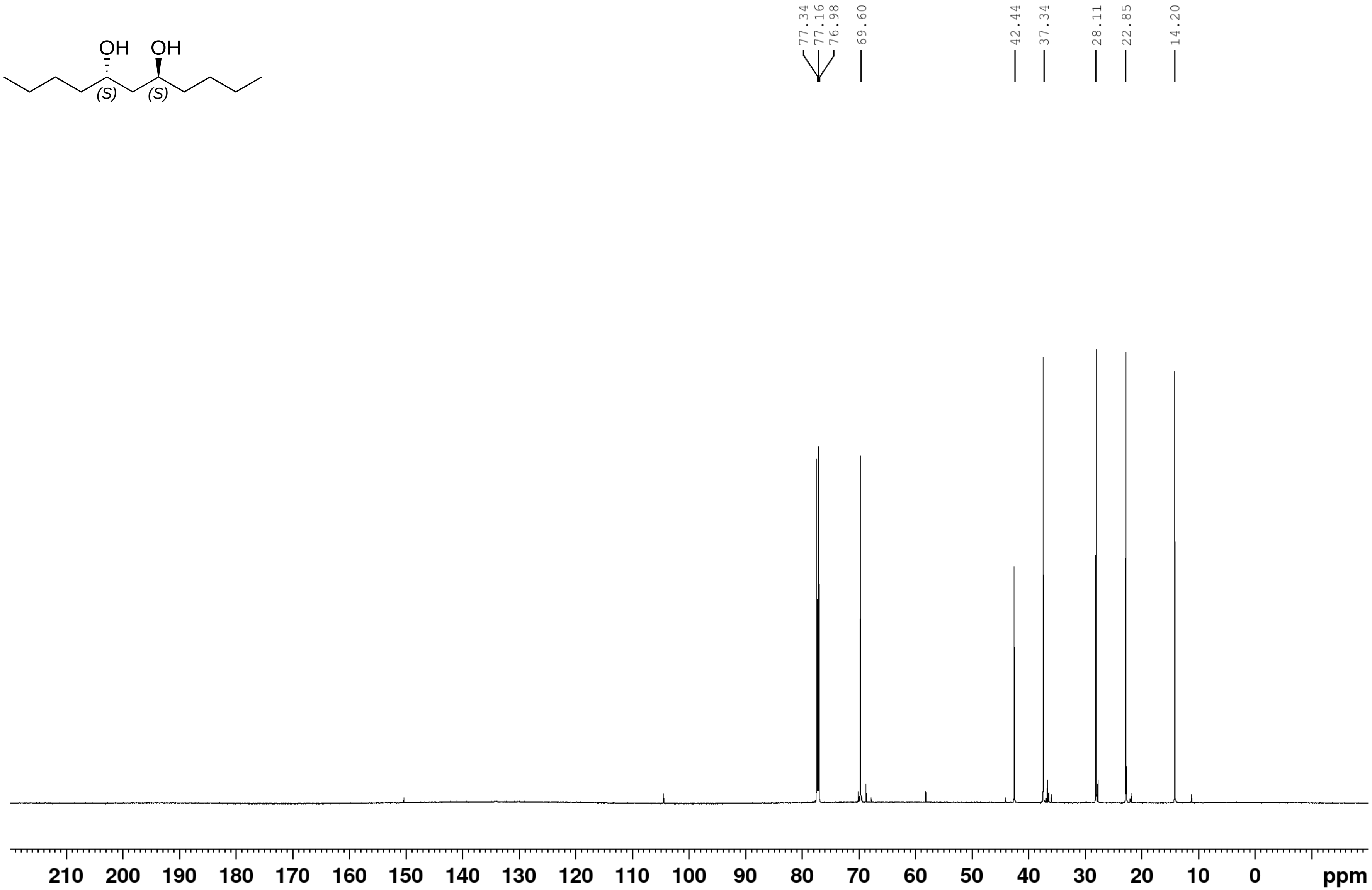

(5*S*,7*S*)-undecane-5,7-diyl dibenzoate (1d-Bz)

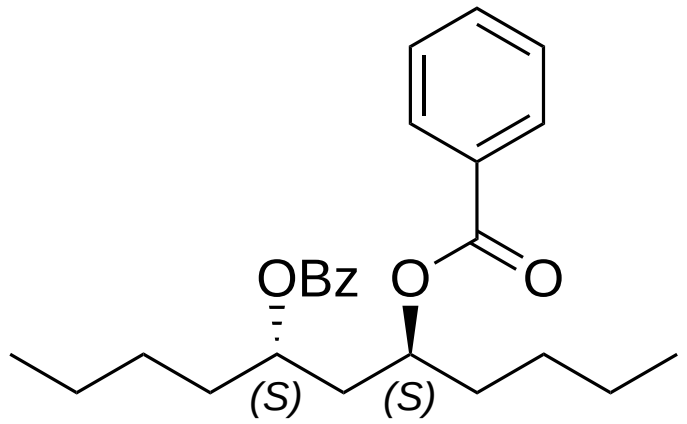

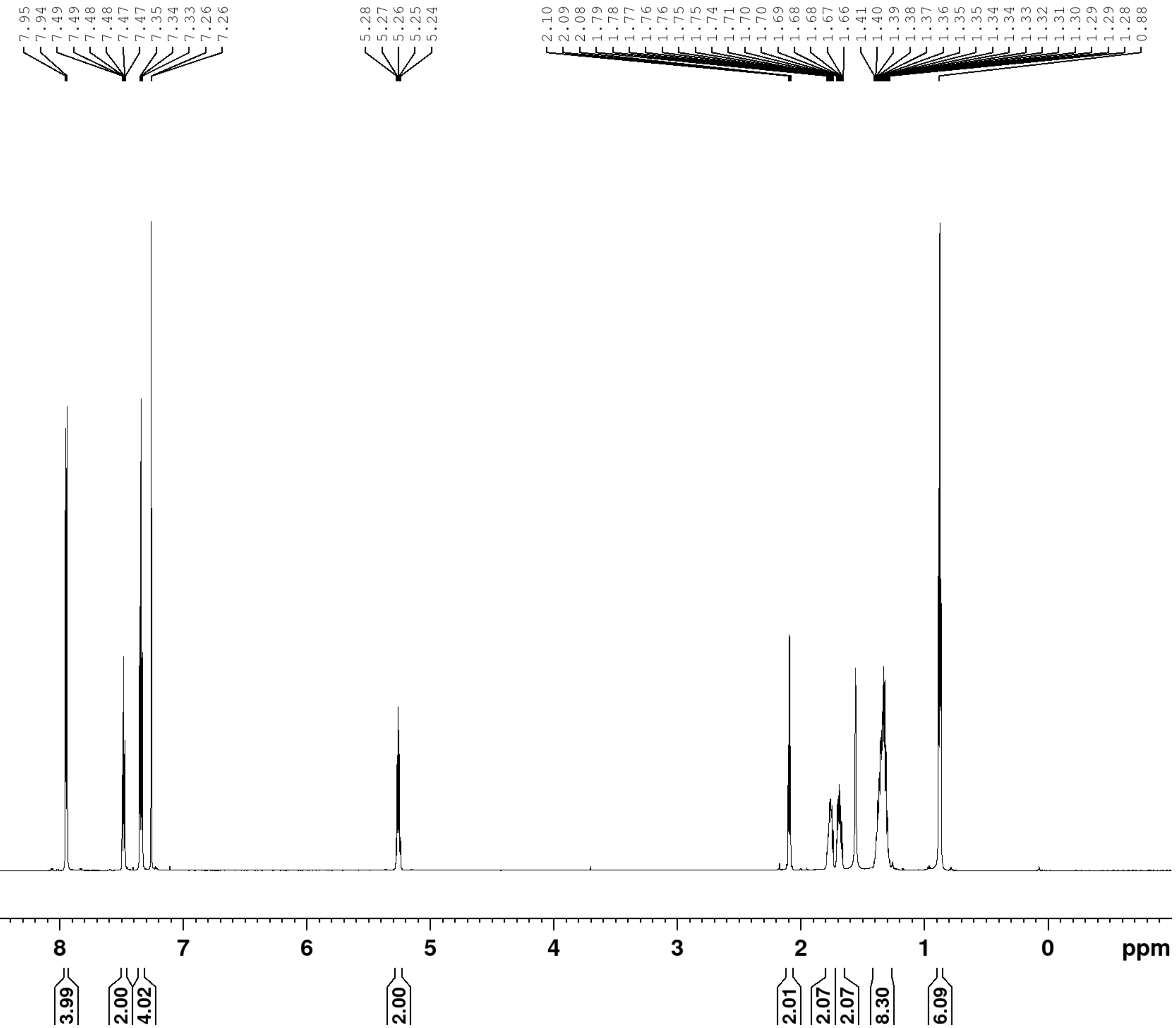

(5*S*,7*S*)-undecane-5,7-diyl dibenzoate (1d-Bz)

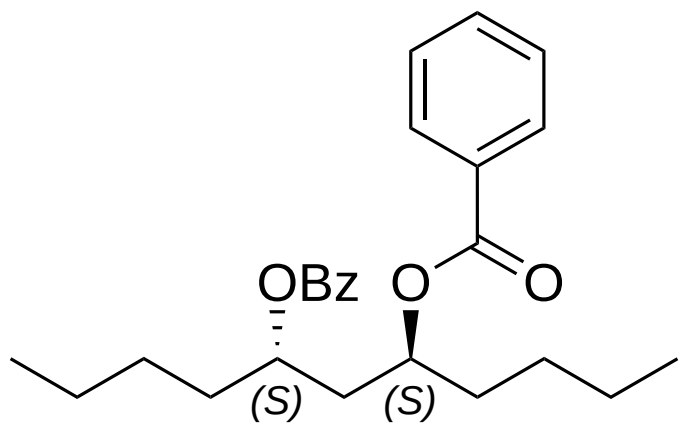

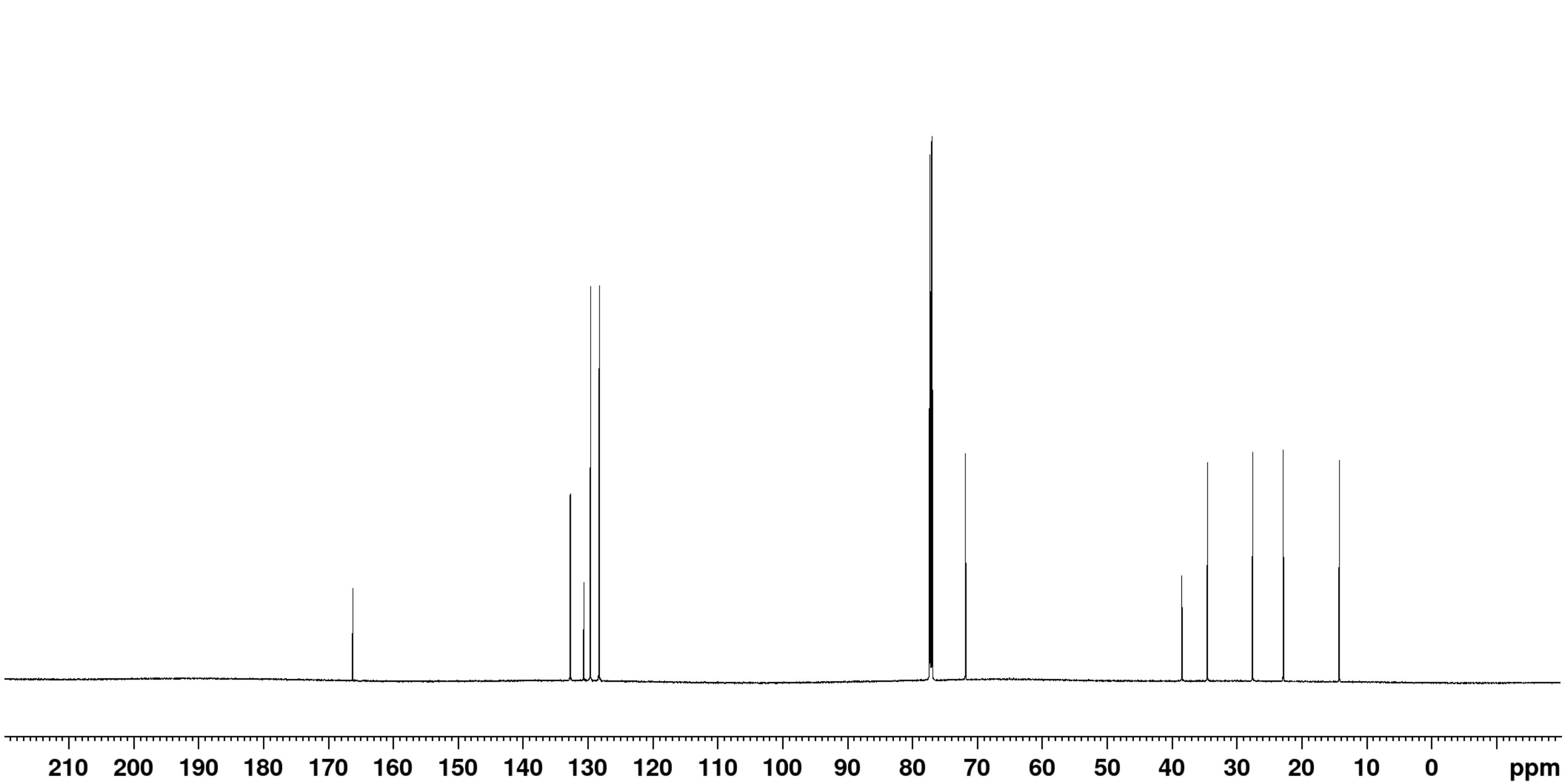

(*R*)-7-hydroxyundecan-5-one (2c)

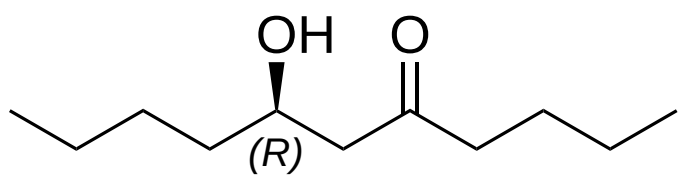

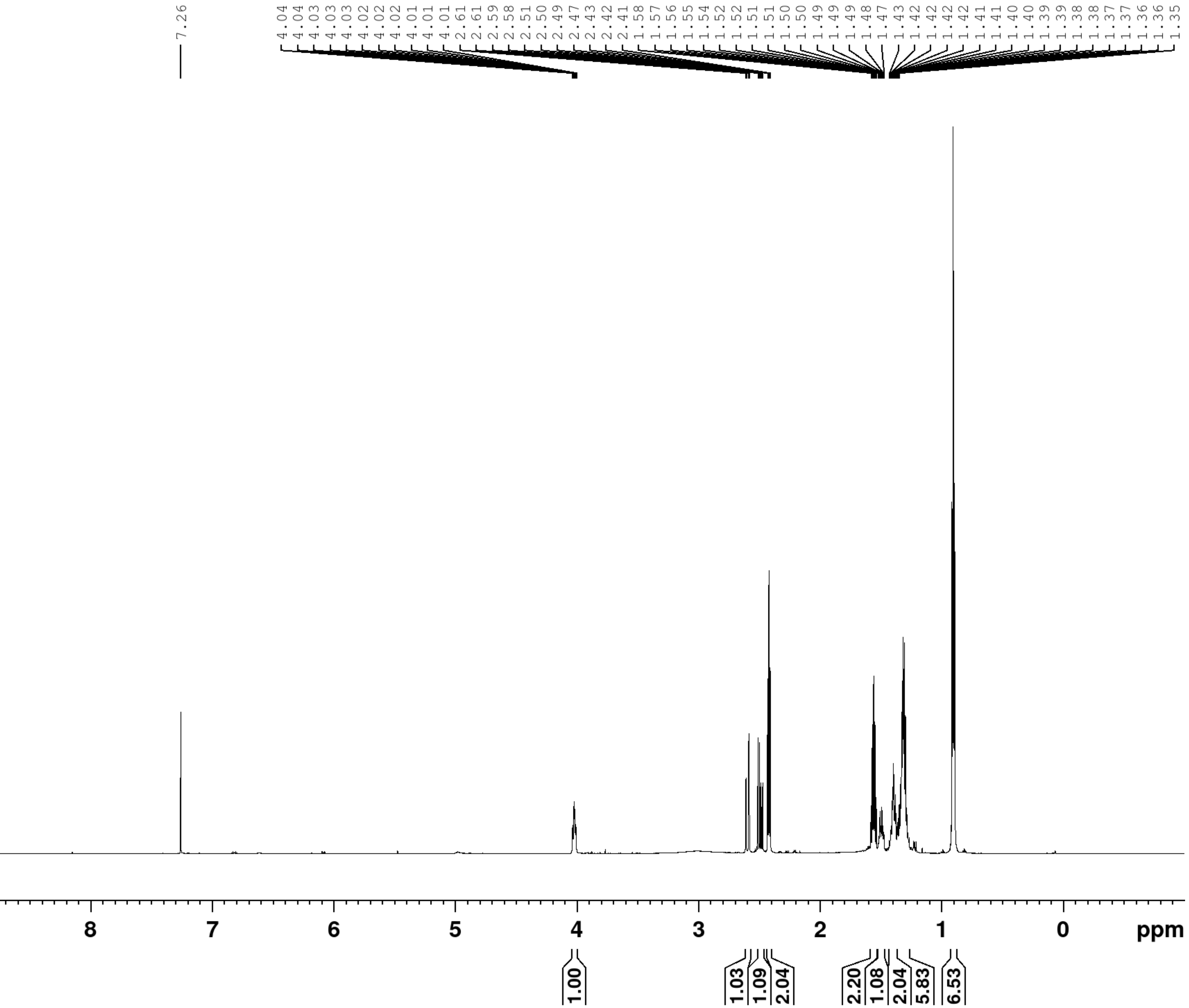

(*R*)-7-hydroxyundecan-5-one (2c)

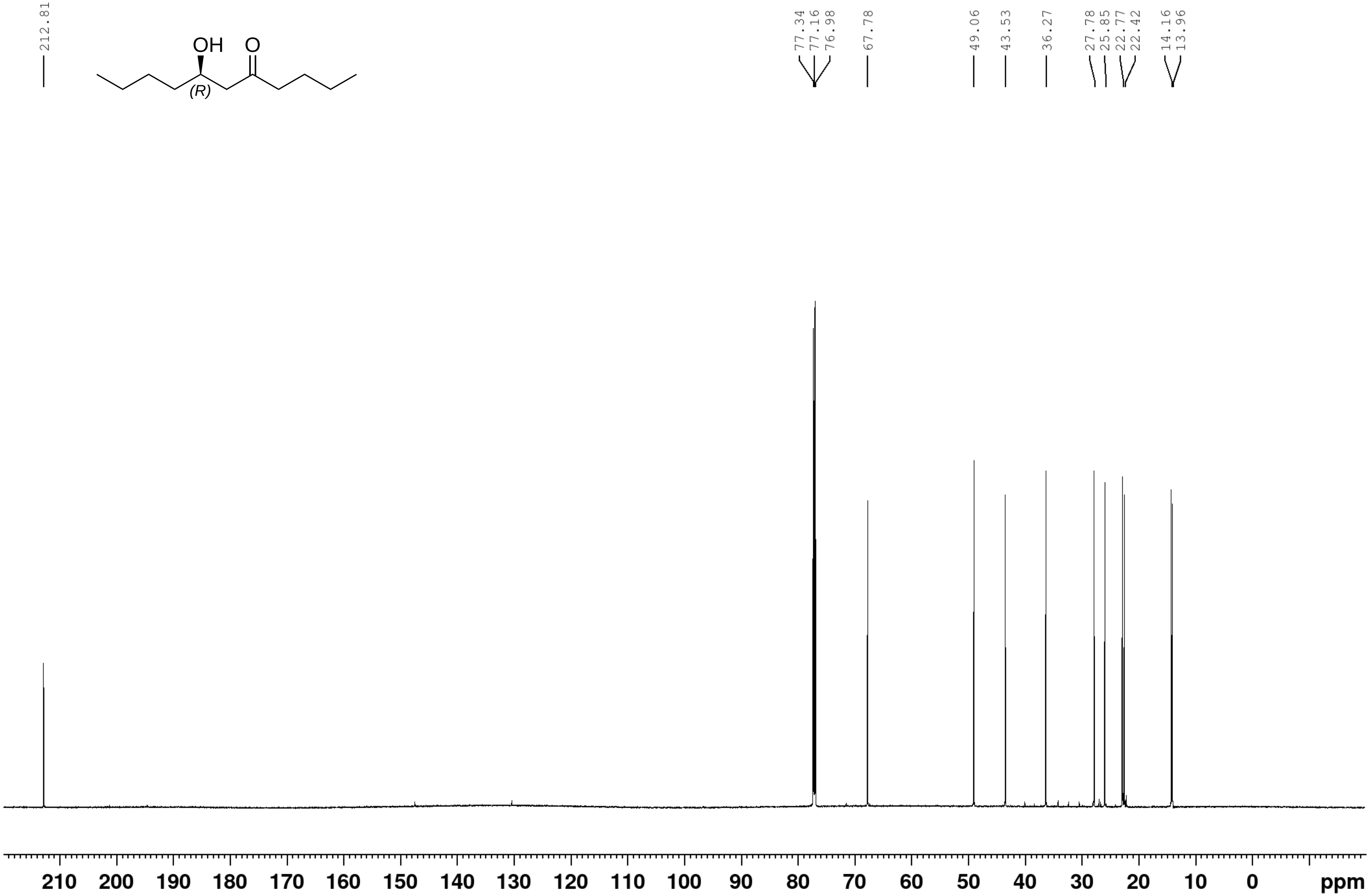

(*R*)-7-((*tert*-butyldiphenylsilyl)oxy)undecan-5-one (2c-TBDPS)

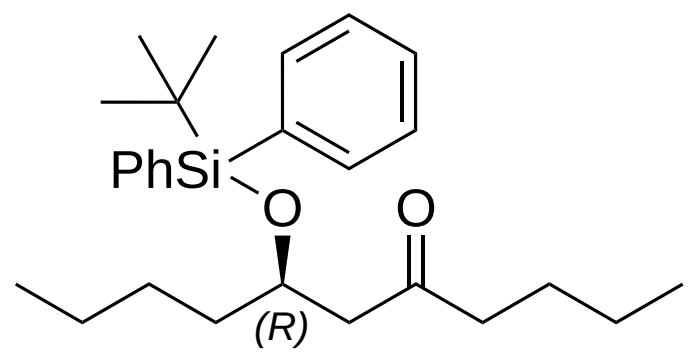

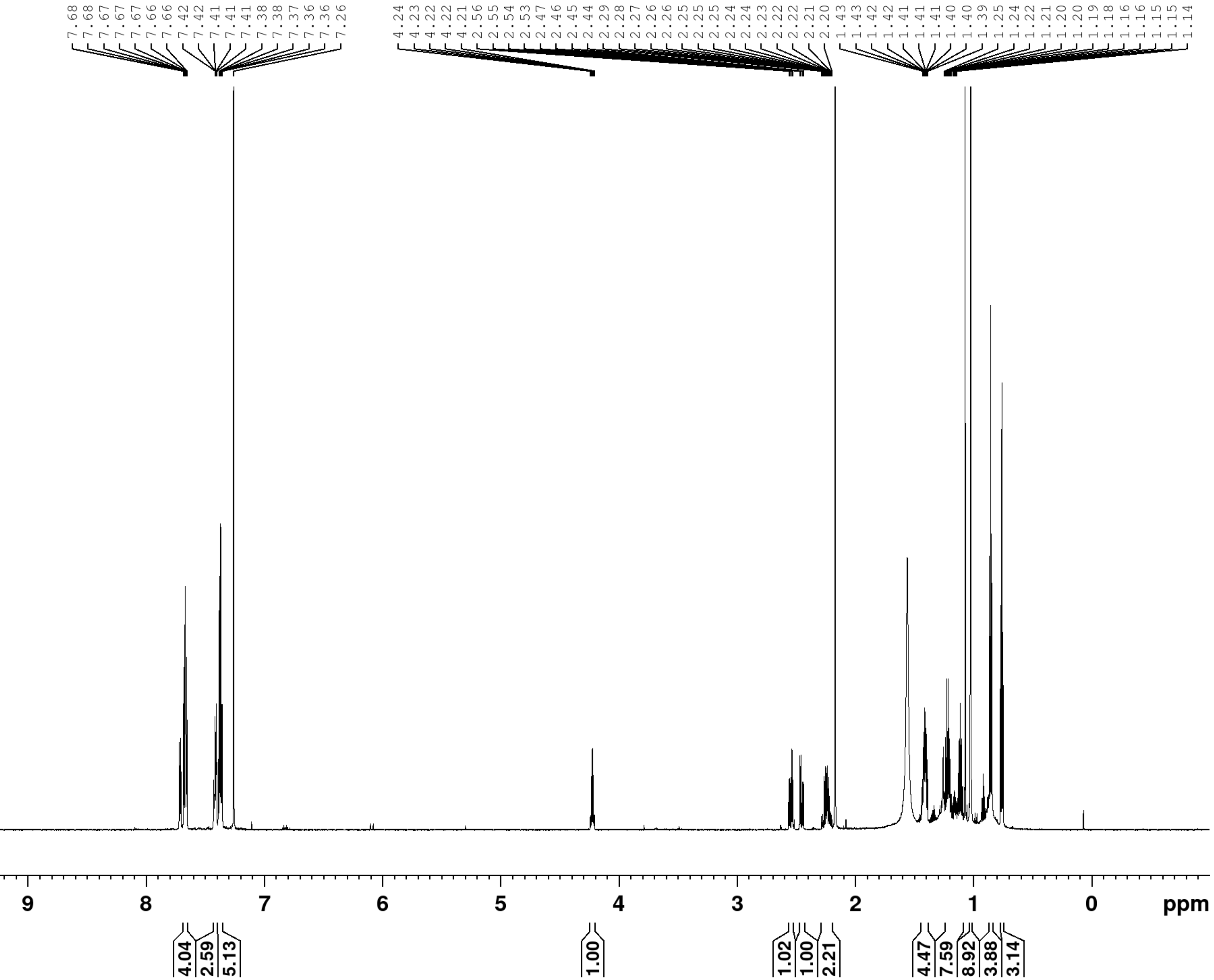

(*R*)-7-((*tert*-butyldiphenylsilyl)oxy)undecan-5-one (2c-TBDPS)

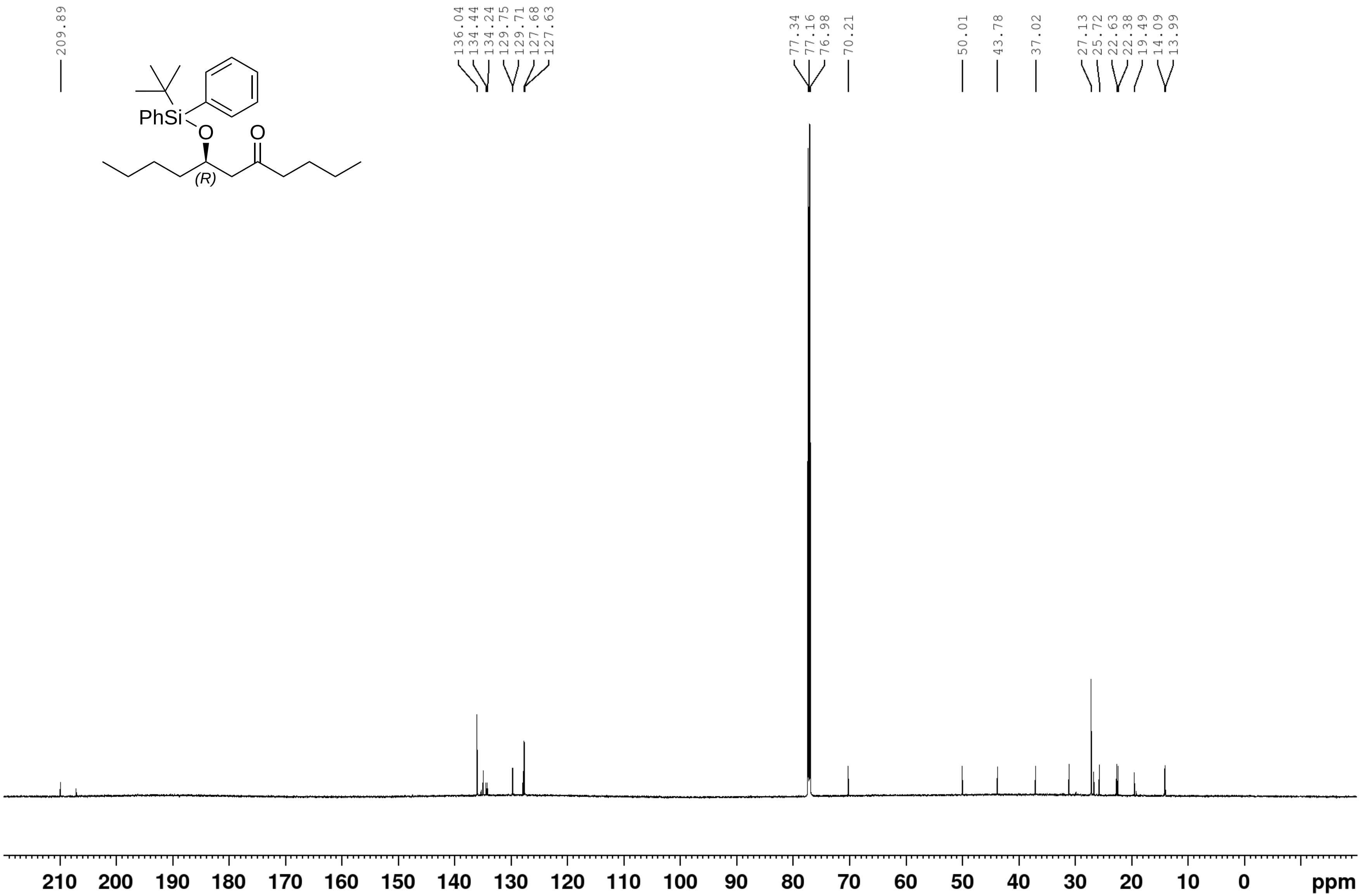

Oxidation reaction with DIAD starting from *rac*-1e

(4*S*,6*S*)-2,8-dimethylnonane-4,6-diol (1e)

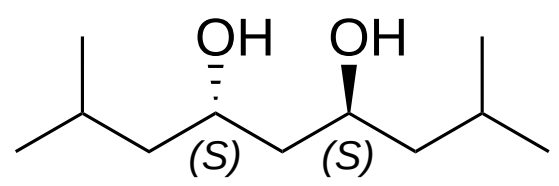

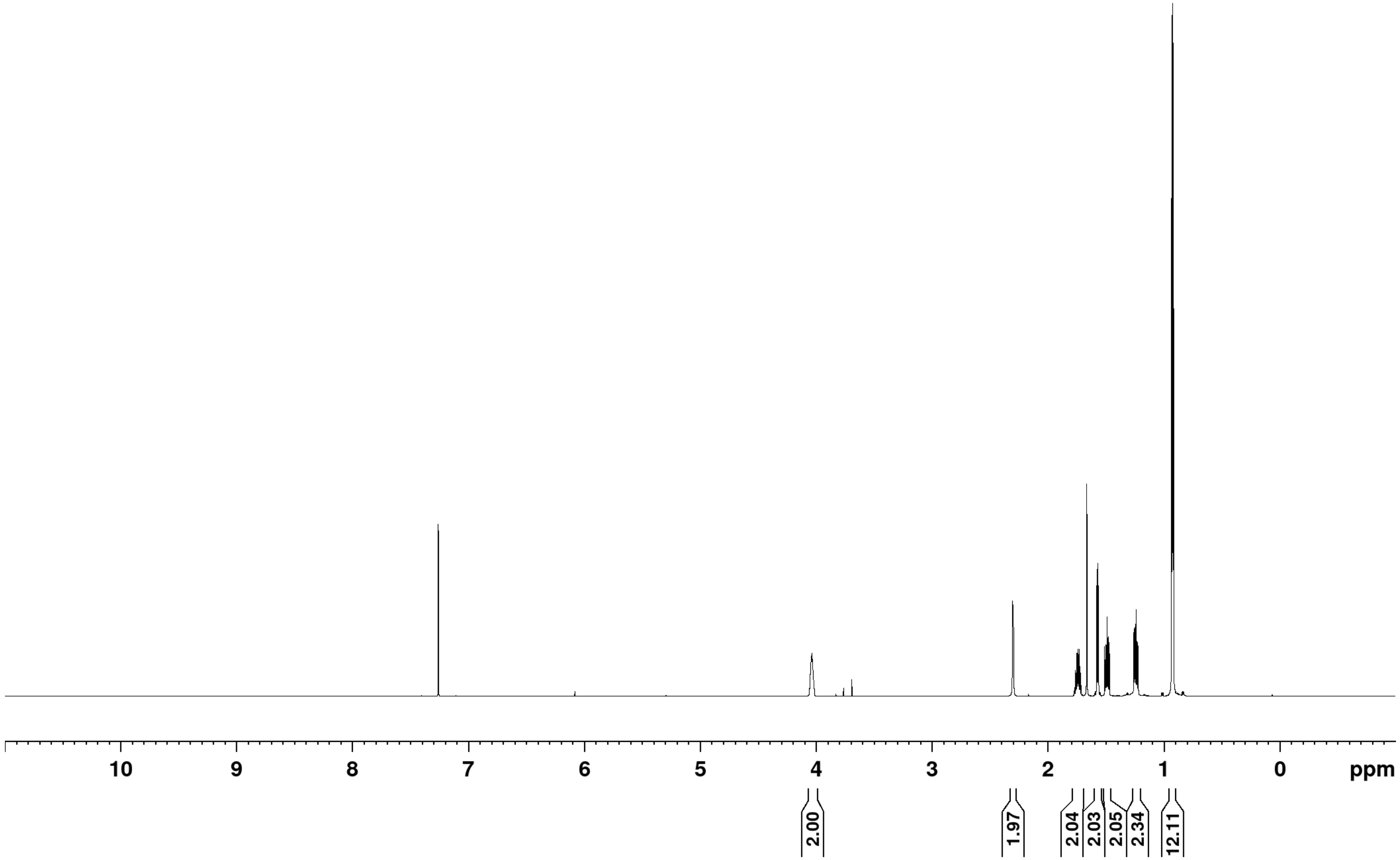

(4*S*,6*S*)-2,8-dimethylnonane-4,6-diol (1e)

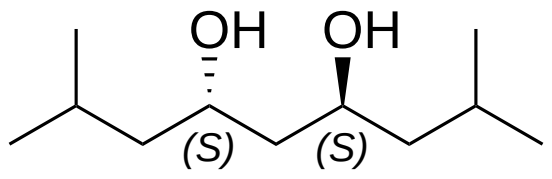

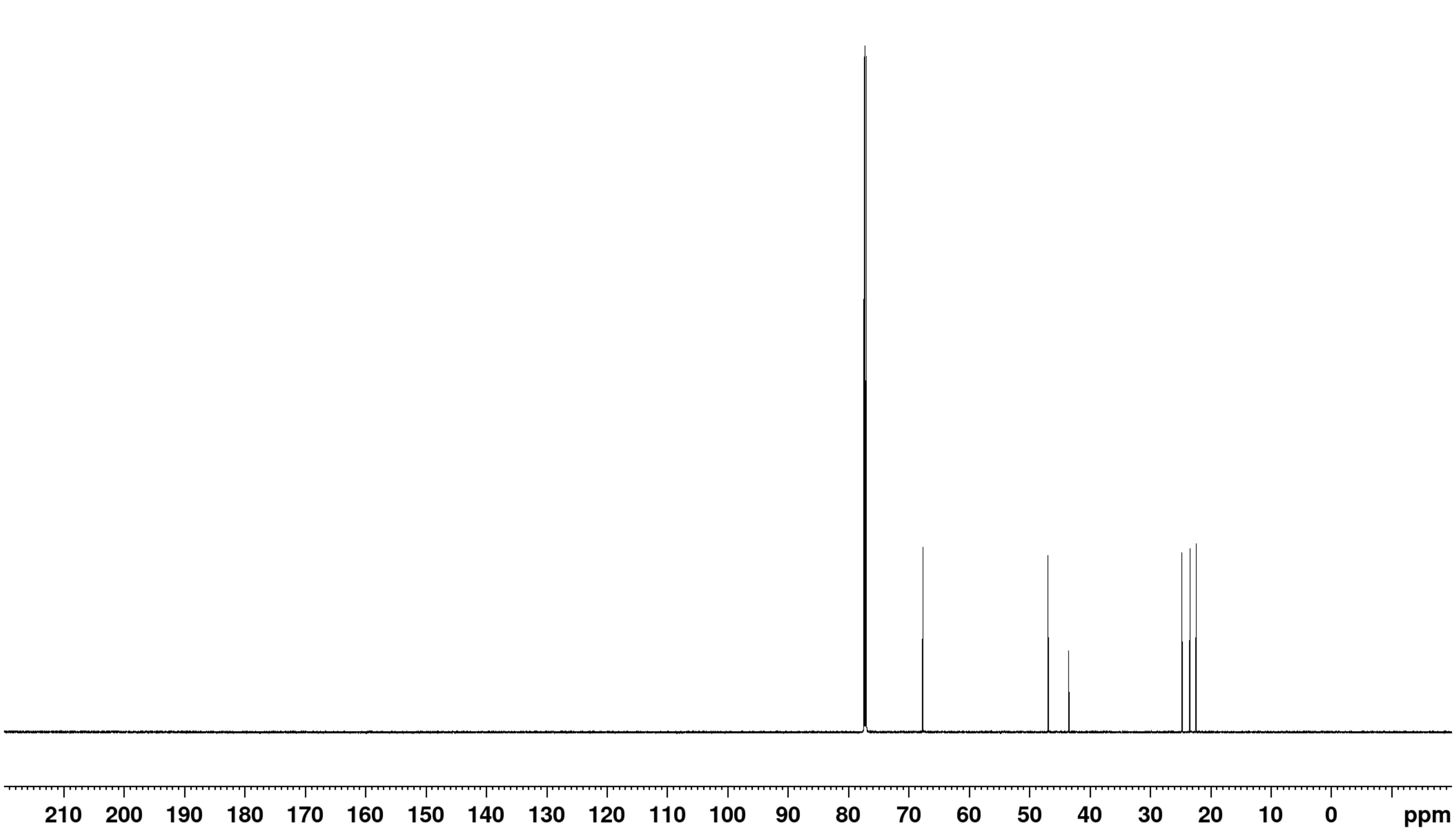

(4*S*,6*S*)-2,8-dimethylnonane-4,6-diyl dibenzoate (1e-Bz)

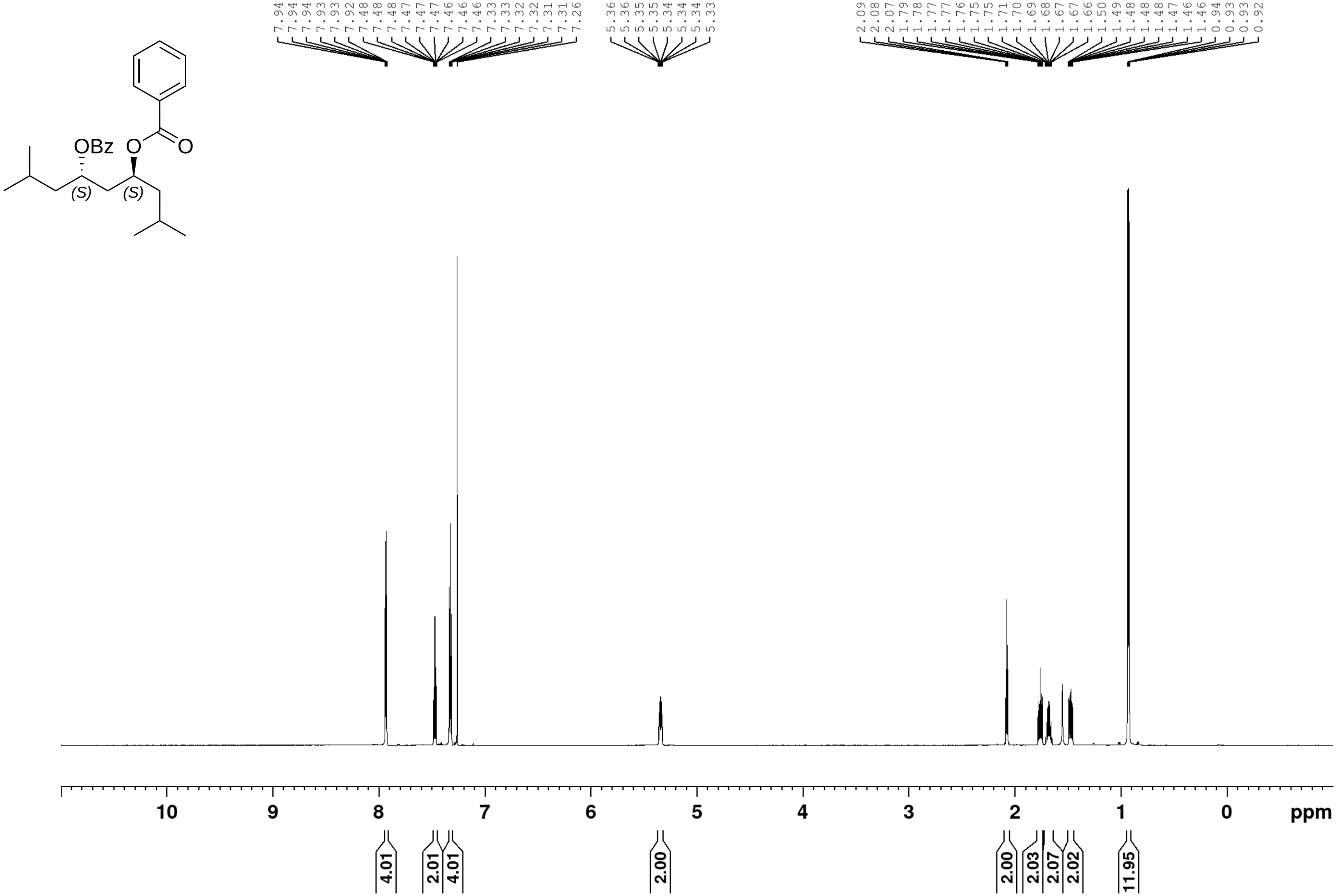

(4*S*,6*S*)-2,8-dimethylnonane-4,6-diyl dibenzoate (1e-Bz)

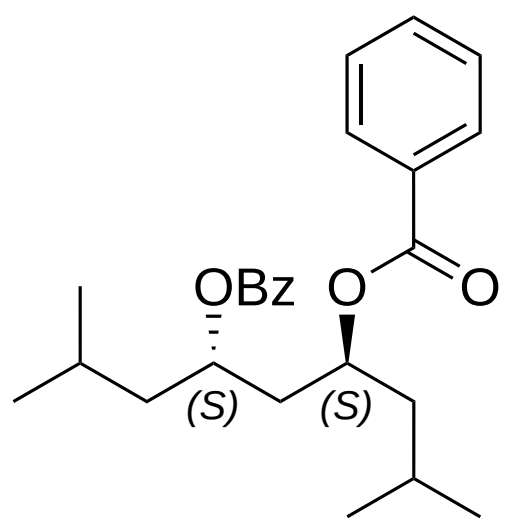

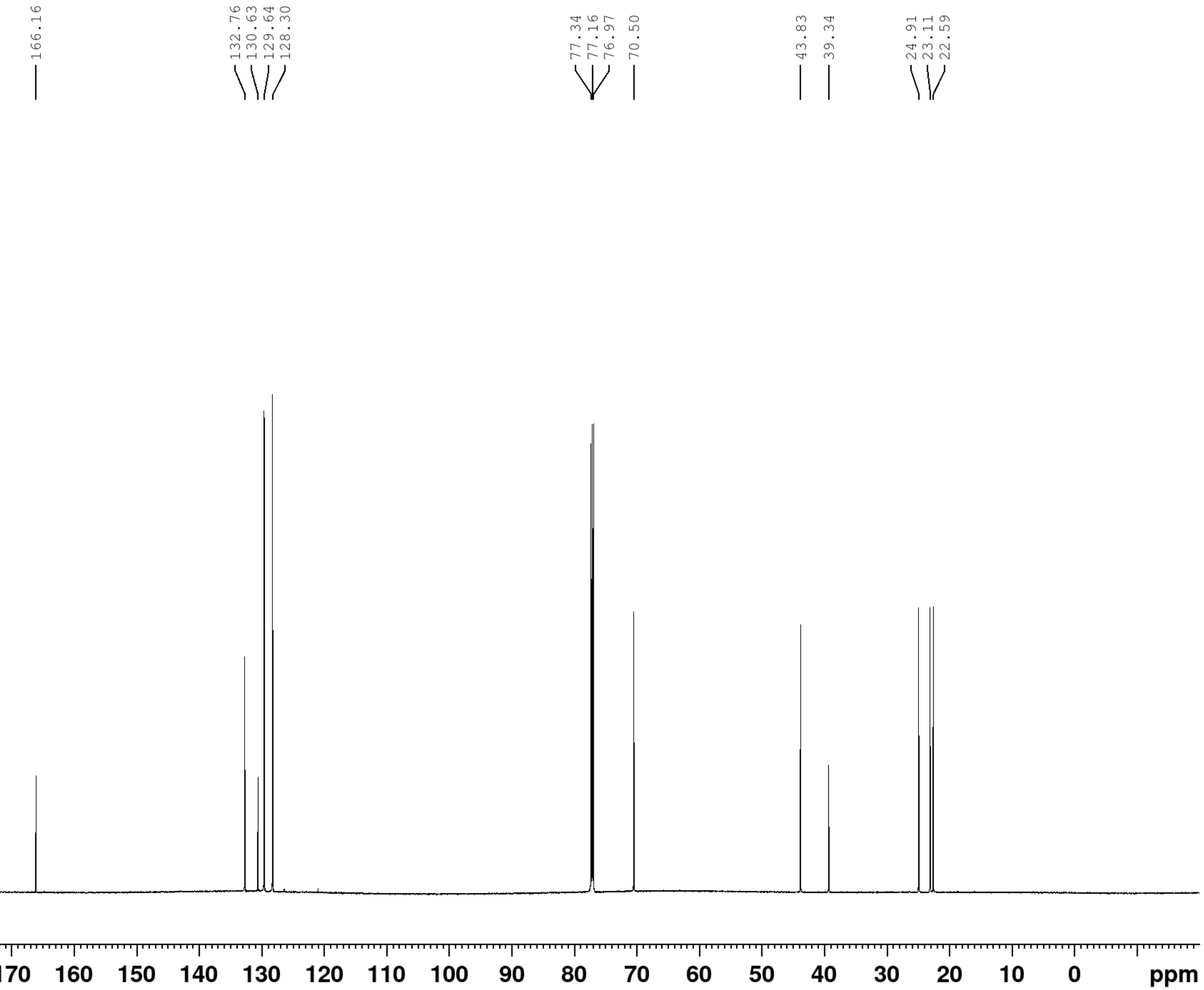

(*R*)-6-hydroxy-2,8-dimethylnonan-4-one (2d)

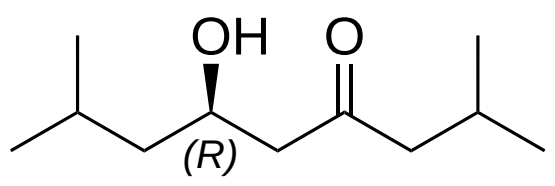

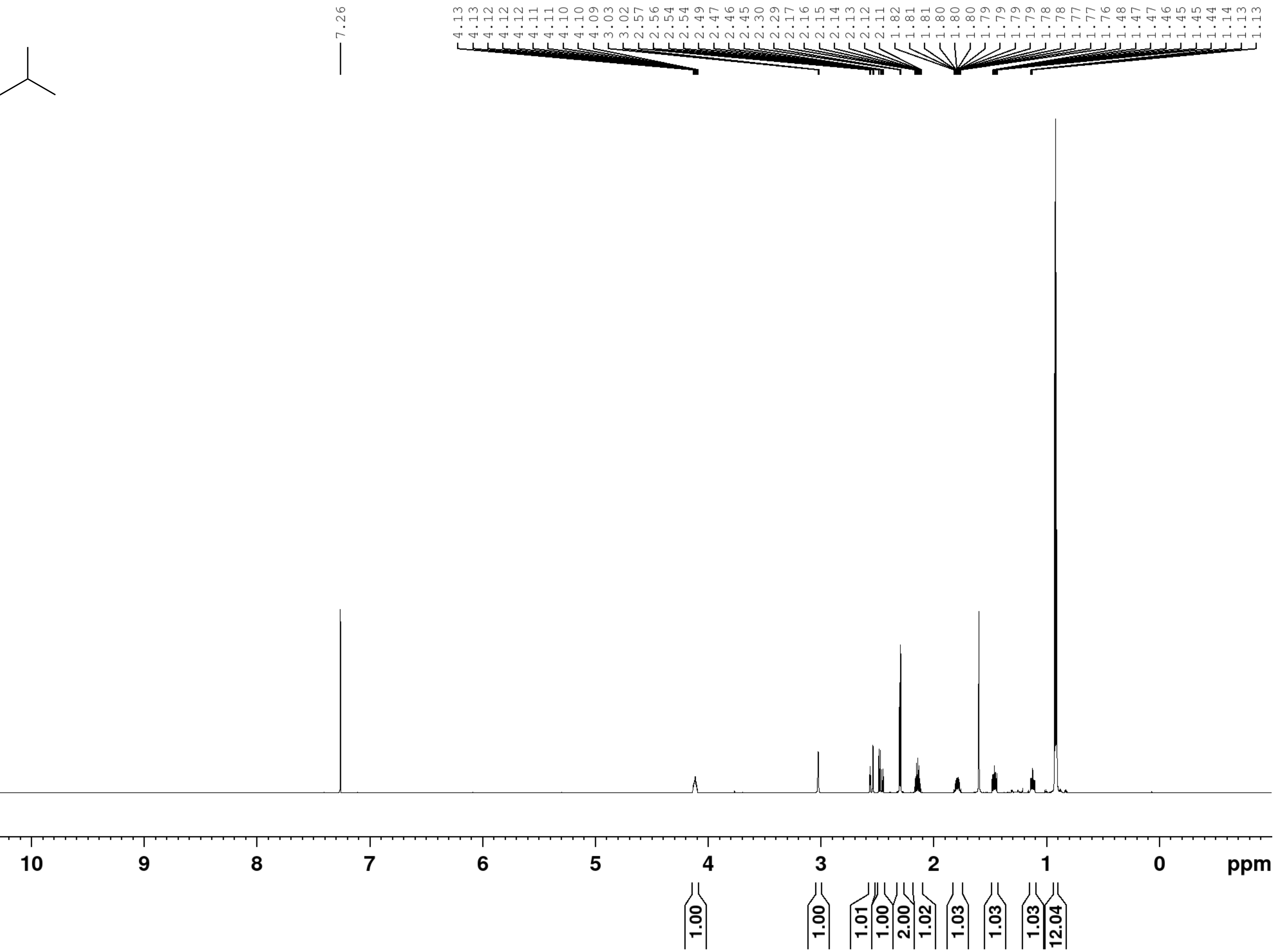

(*R*)-6-hydroxy-2,8-dimethylnonan-4-one (2d)

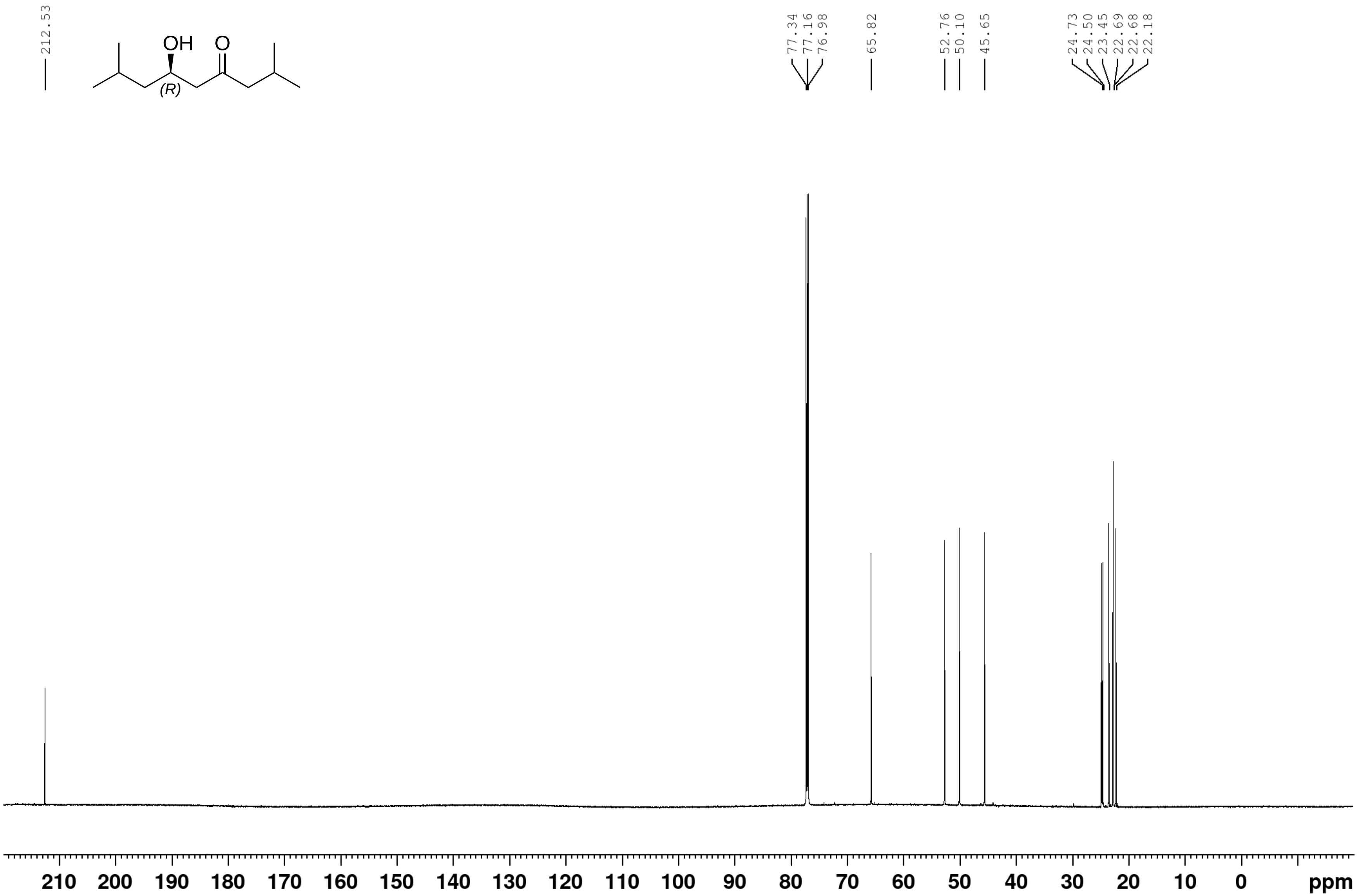

(*R*)-6-((*tert*-butyldiphenylsilyl)oxy)-2,8-dimethylnonan-4-one (2d-TBDPS)

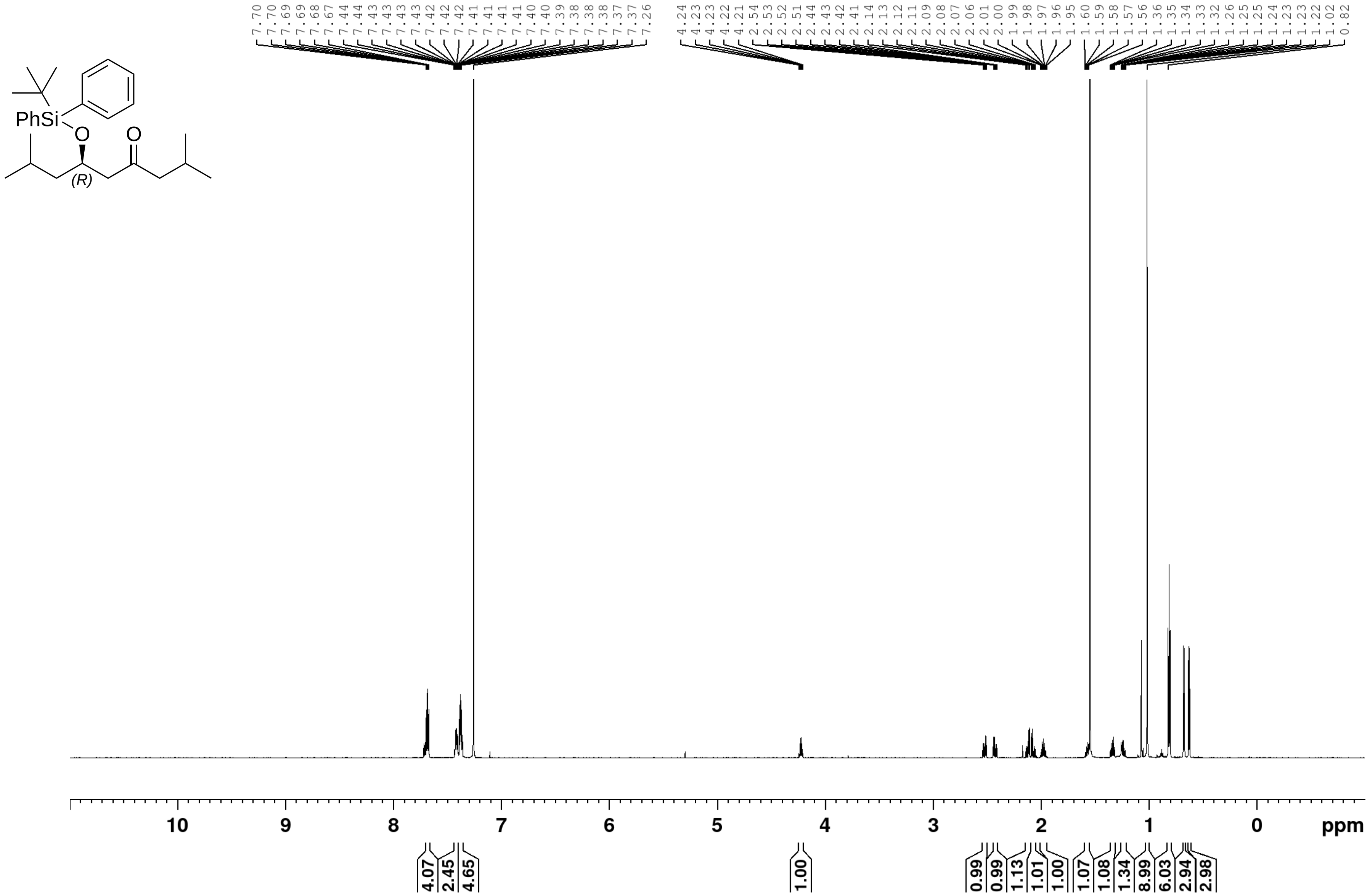

(*R*)-6-((*tert*-butyldiphenylsilyl)oxy)-2,8-dimethylnonan-4-one (2d-TBDPS)

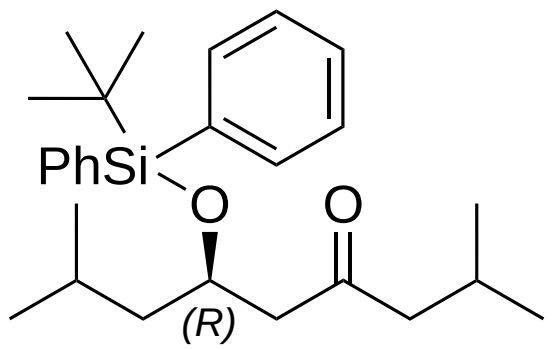

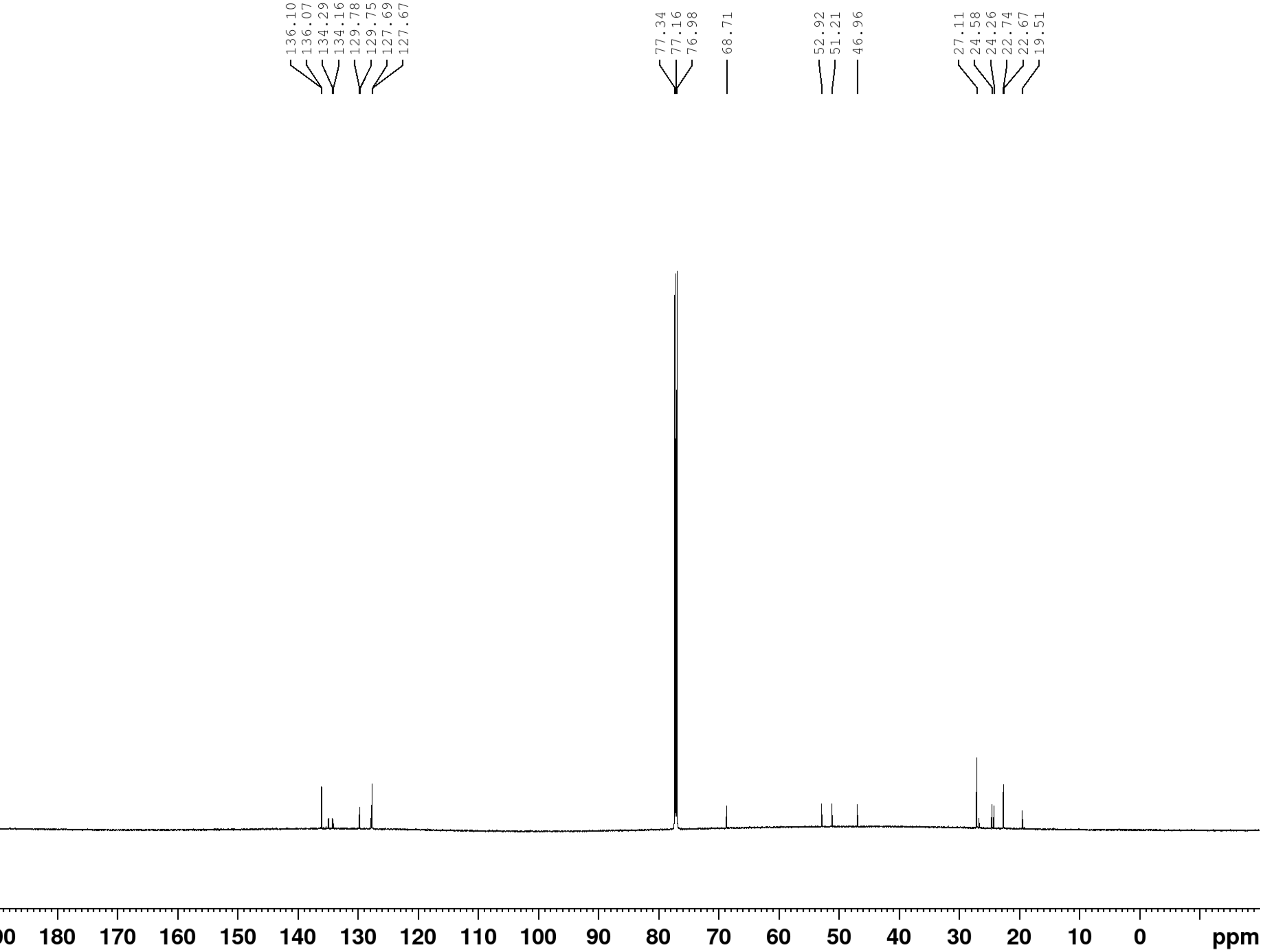

Oxidation reaction with DIAD starting from *rac*-1k

(3S,5S)-1,7-diphenylheptane-3,5-diol (1k) and Diisopropyl 1,2-hydrazinedicarboxylate

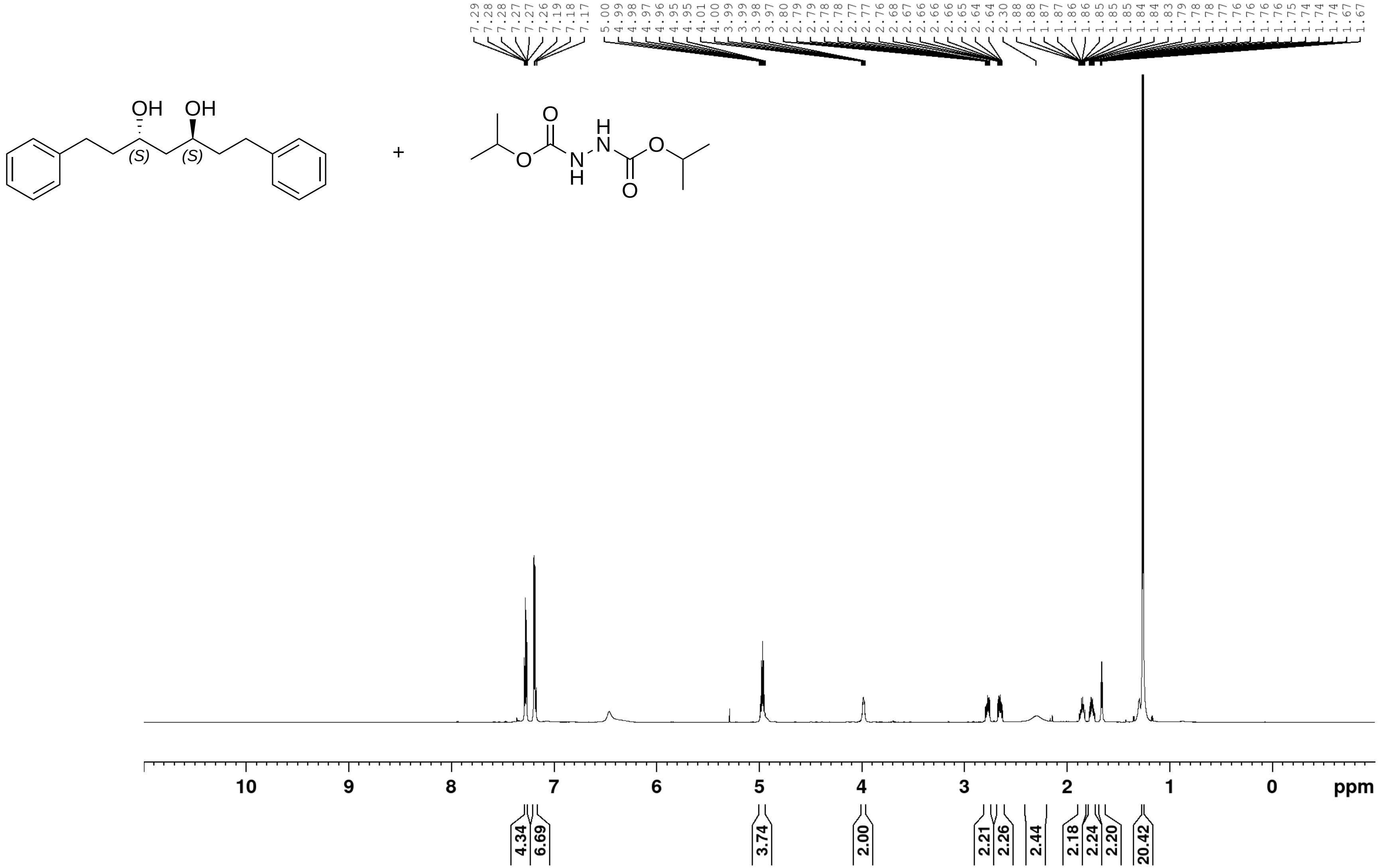

(3S,5S)-1,7-diphenylheptane-3,5-diol (1k) and Diisopropyl 1,2-hydrazinedicarboxylate

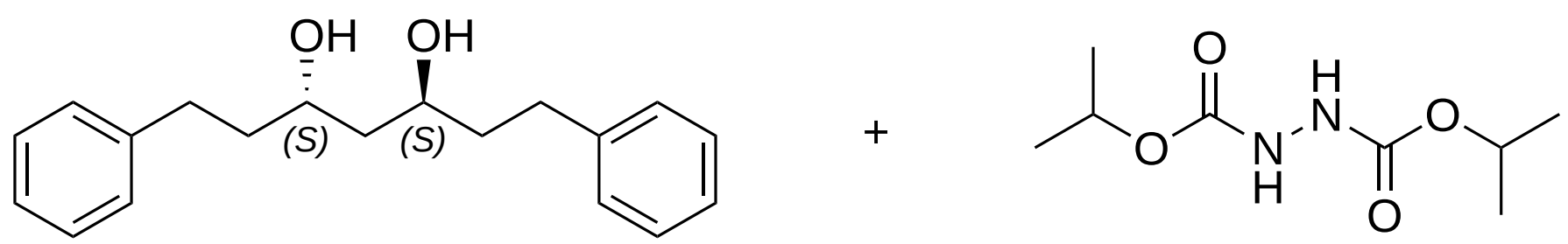

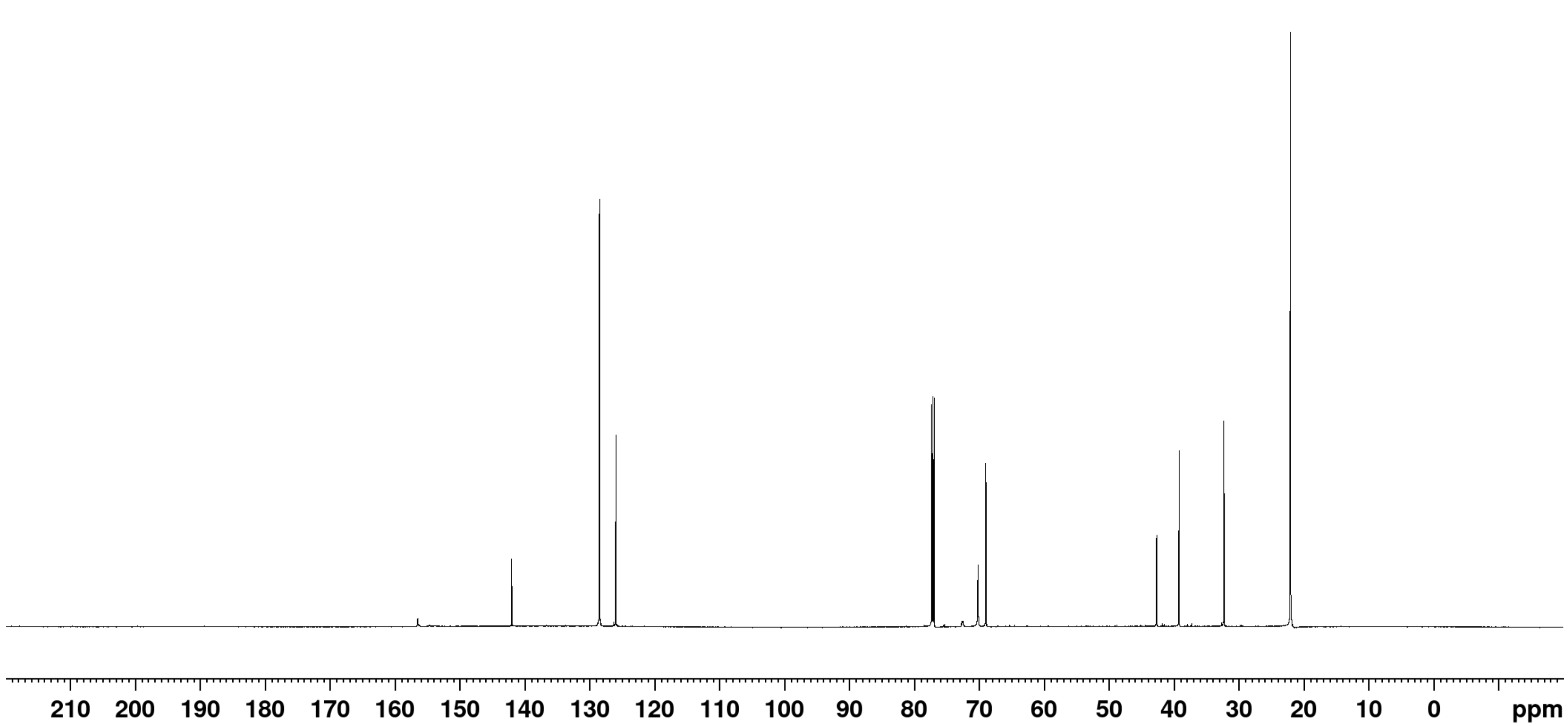

(*R*)-5-hydroxy-1,7-diphenylheptan-3-one (2e)

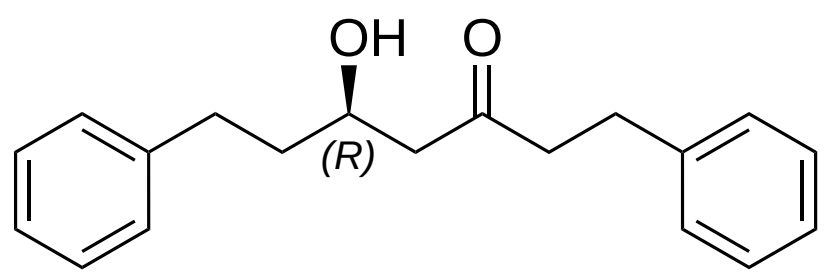

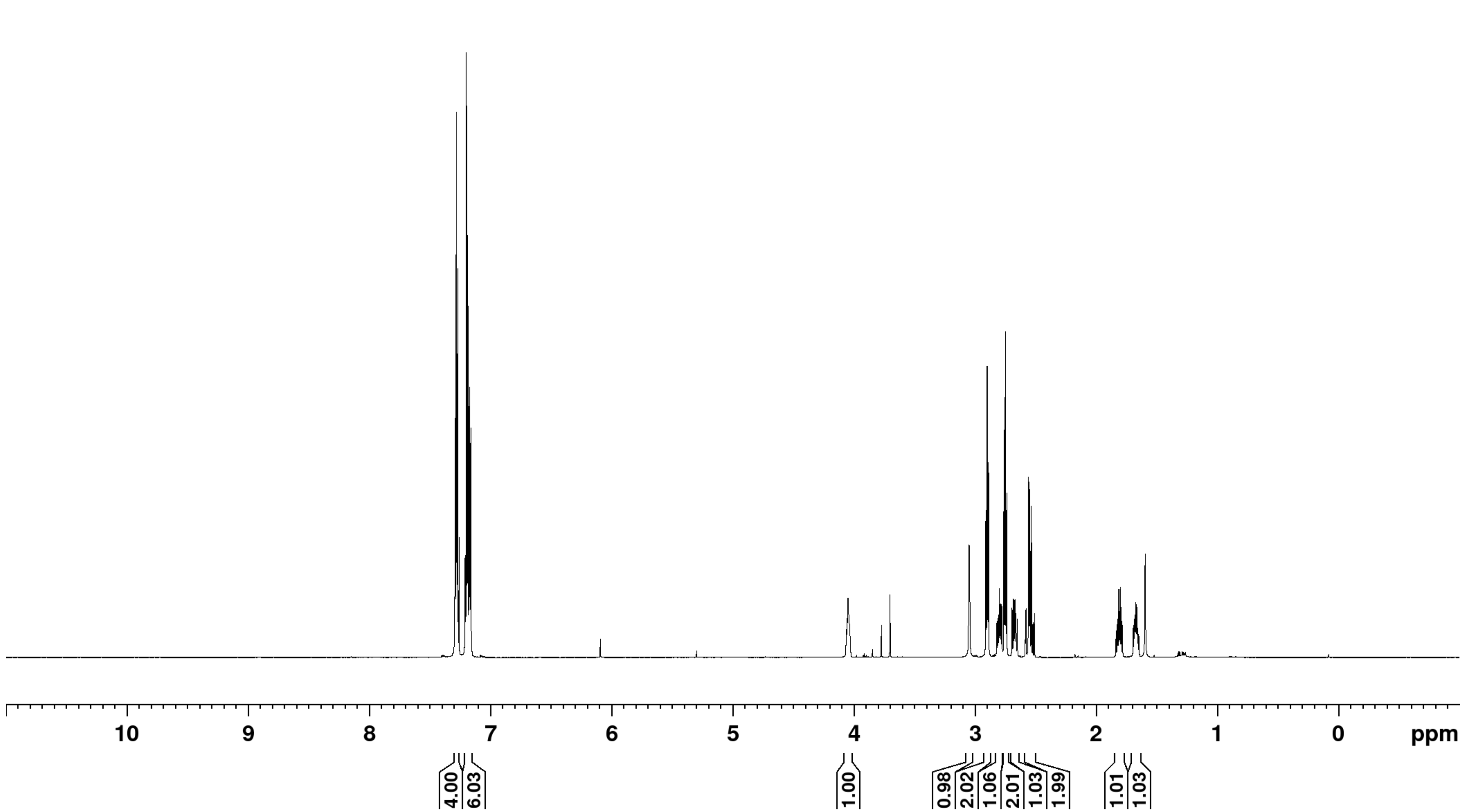

(*R*)-5-hydroxy-1,7-diphenylheptan-3-one (2e)

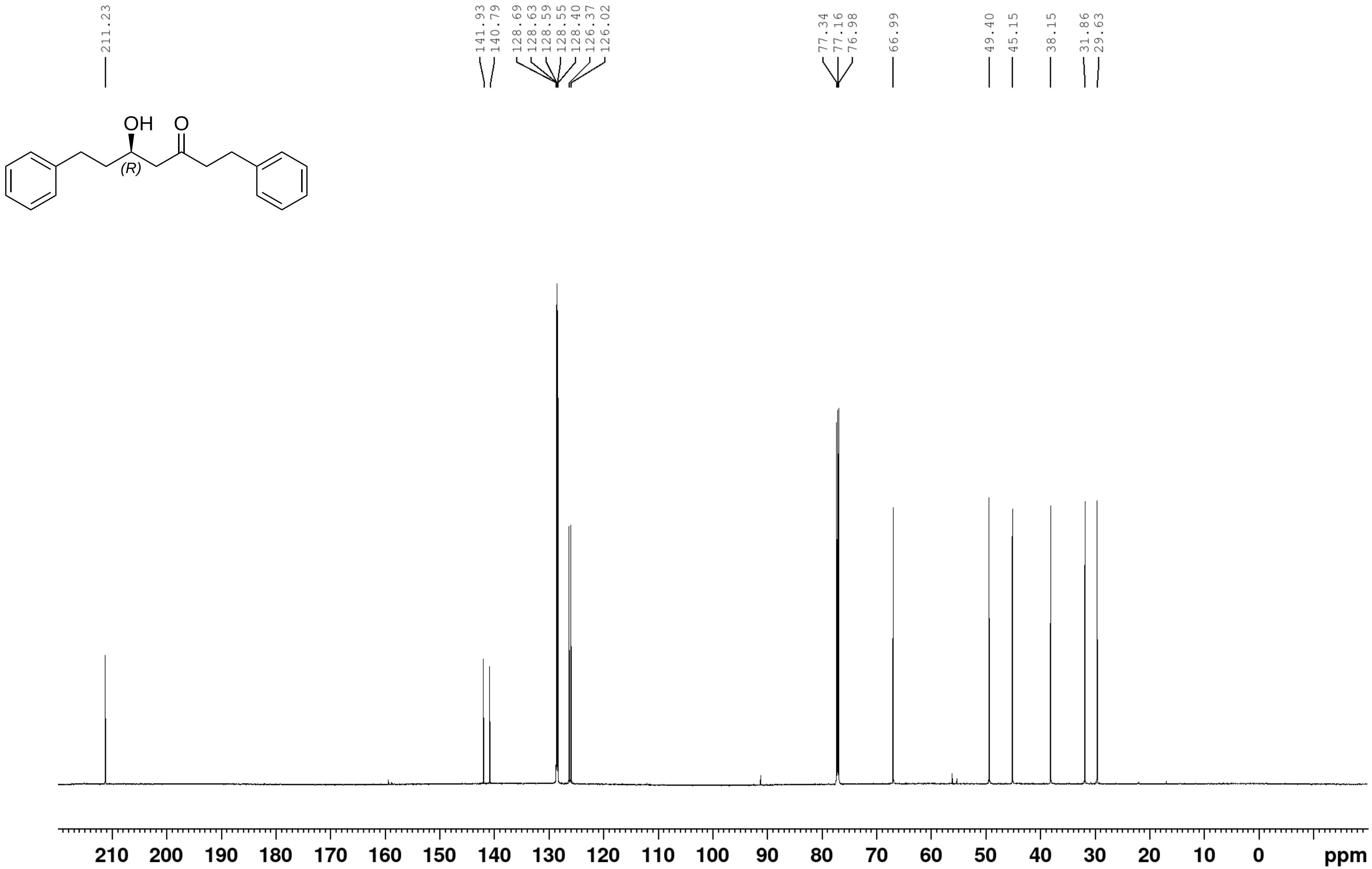

Access to Opposite Enantiomer

(5*R*,7*R*)-undecane-5,7-diol (*R*,*R*-1d)

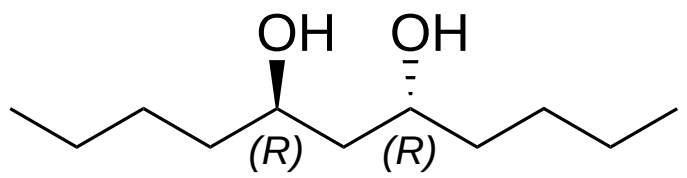

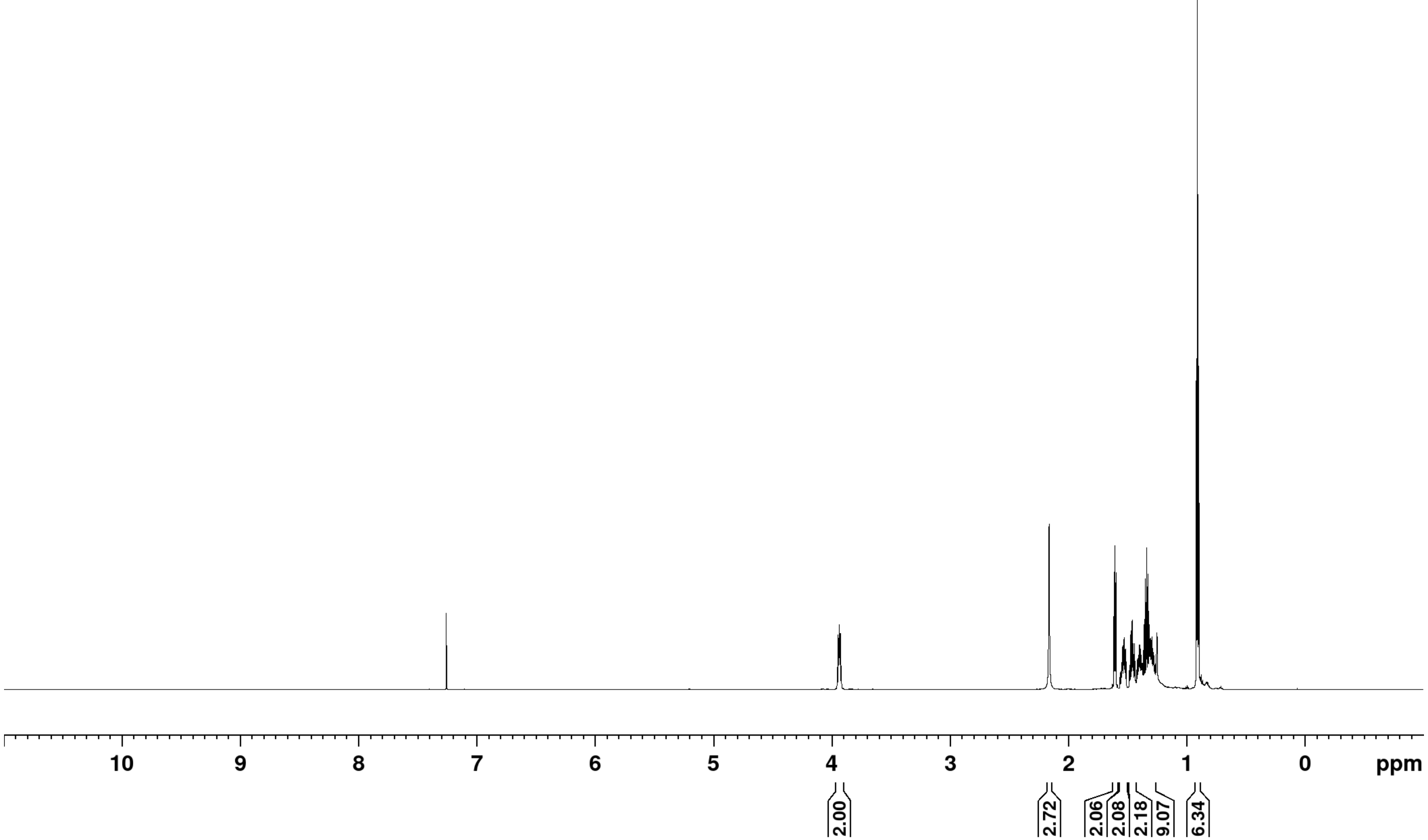

(5*R*,7*R*)-undecane-5,7-diol (*R*,*R*-1d)

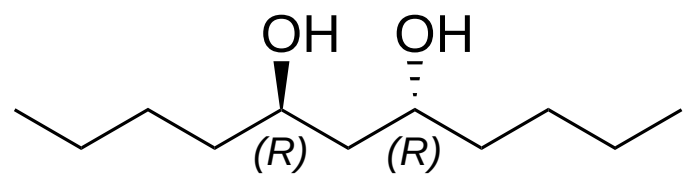

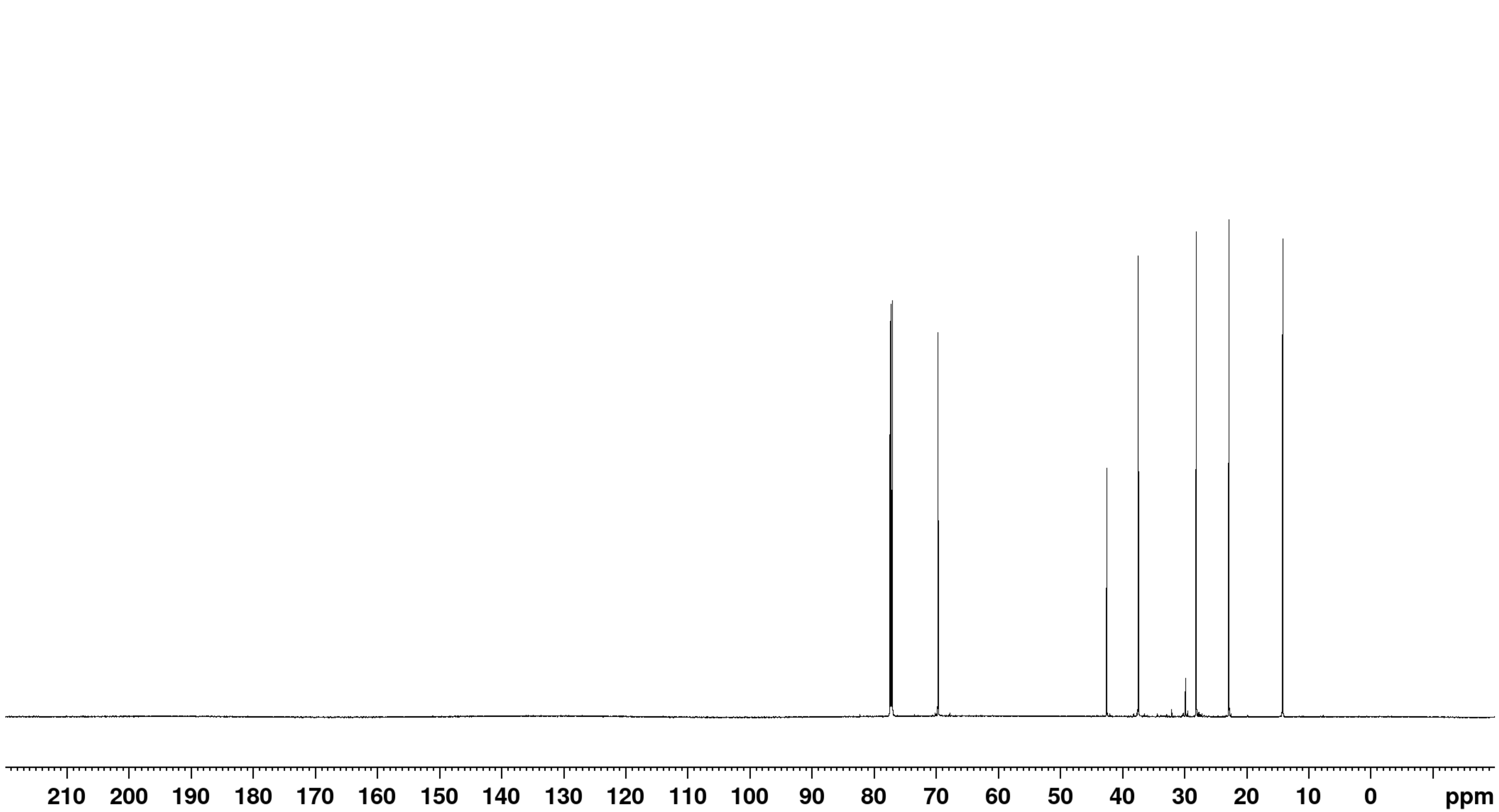

(4*R*,6*R*)-2,8-dimethylnonane-4,6-diyl dibenzoate ((*R*,*R*)-1d-Bz)

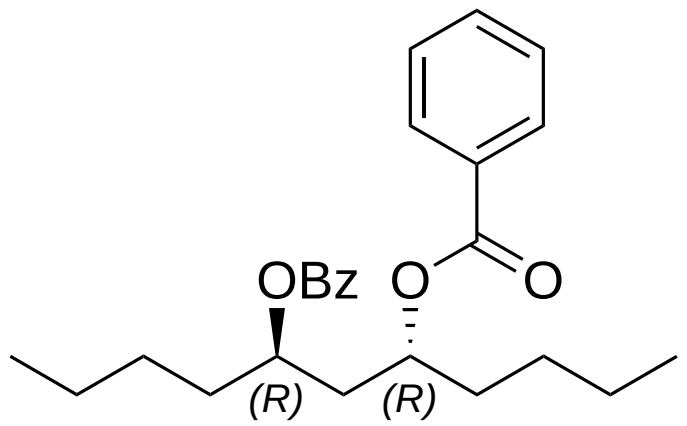

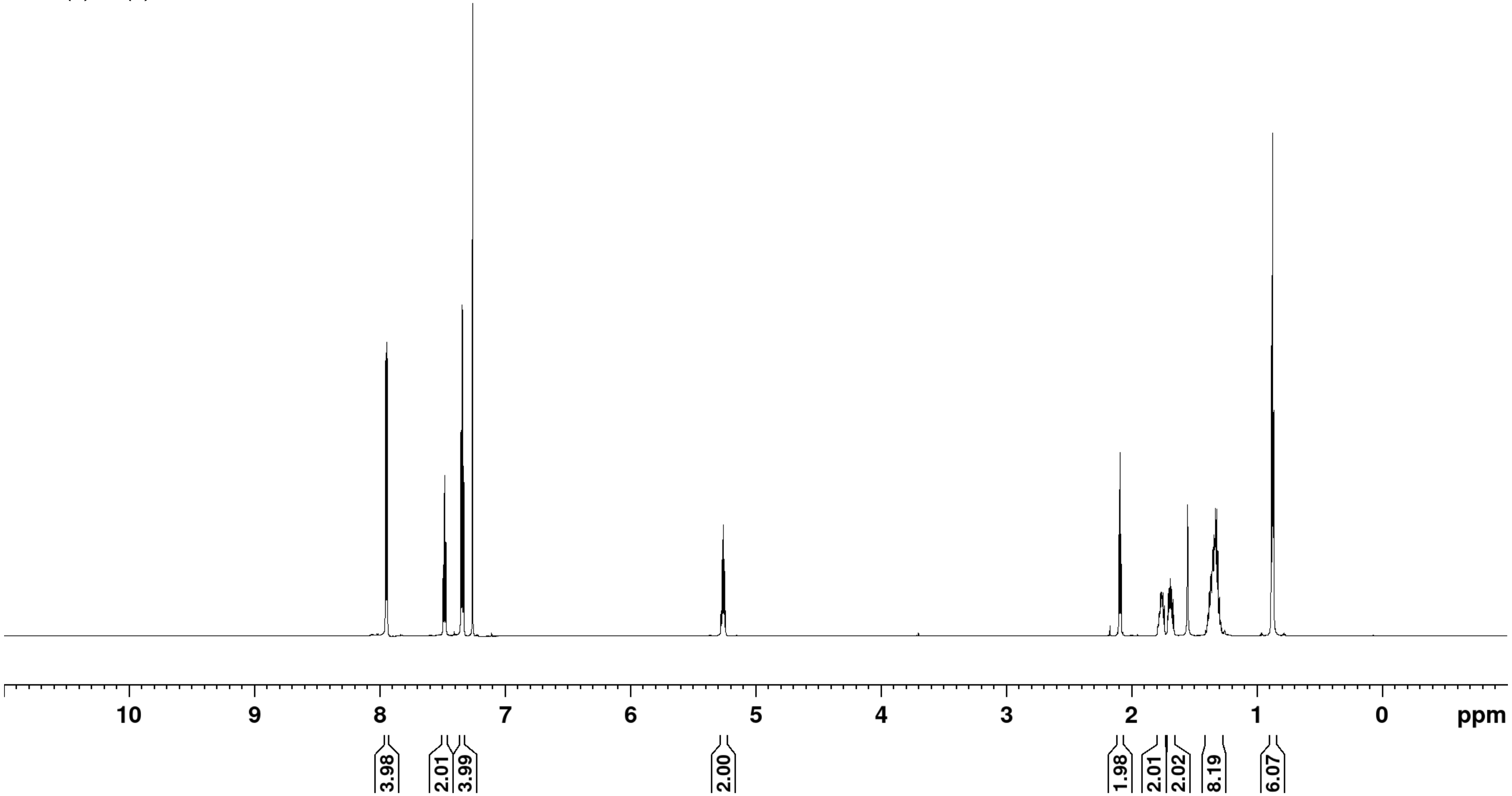

(4*R*,6*R*)-2,8-dimethylnonane-4,6-diyl dibenzoate ((*R*,*R*)-1d-Bz)

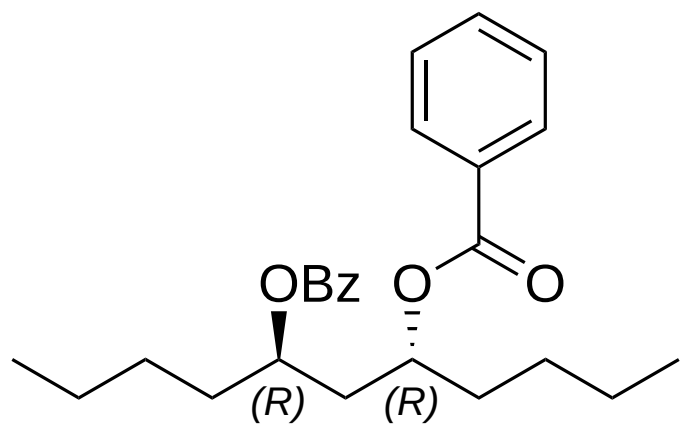

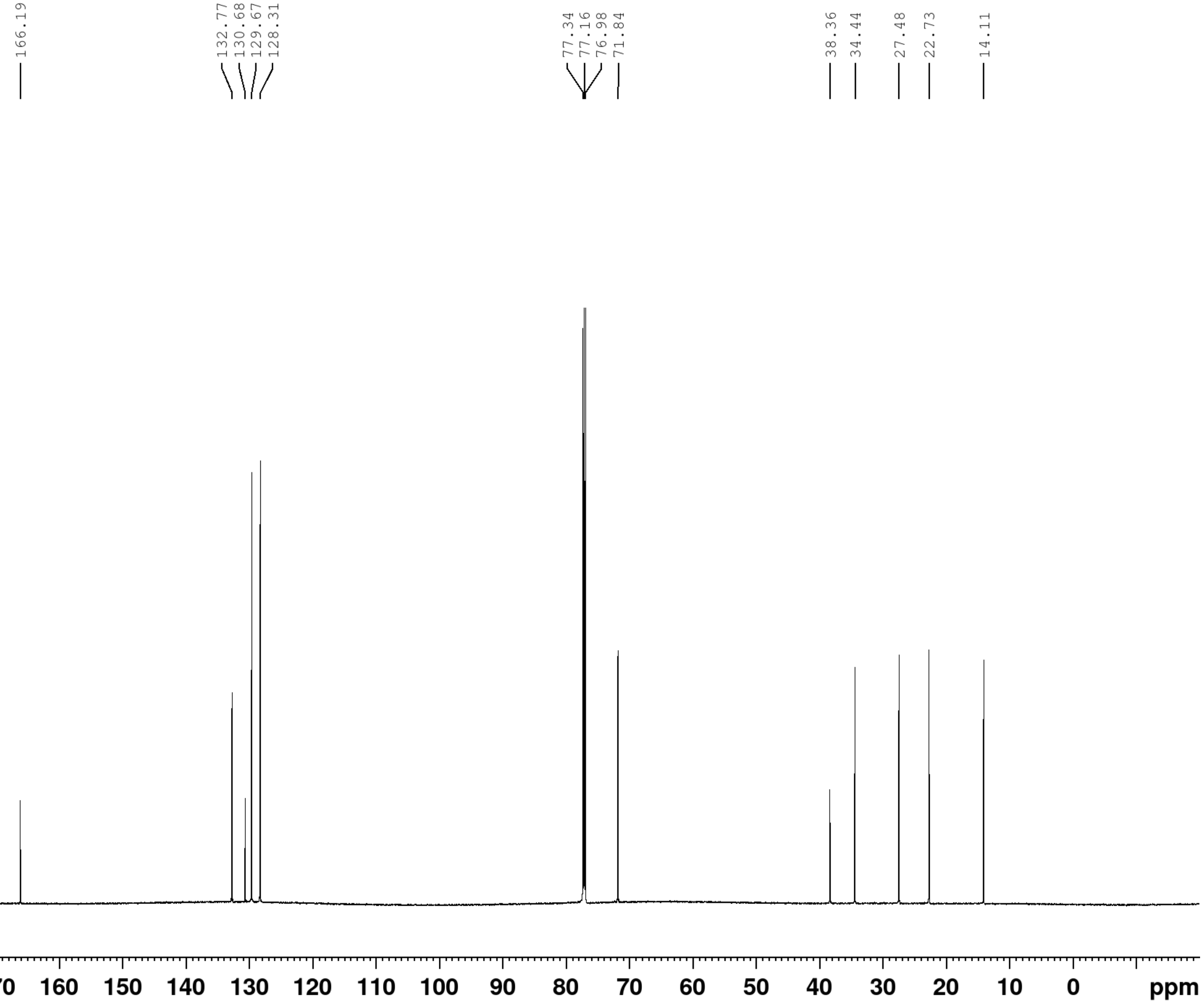

1 mmol scale up of symmetrical 1,3-diol (1d)

(*syn*)-undecane-5,7-diol (*meso*-1d)

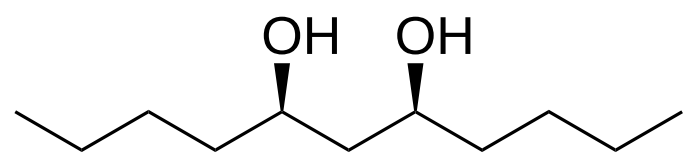

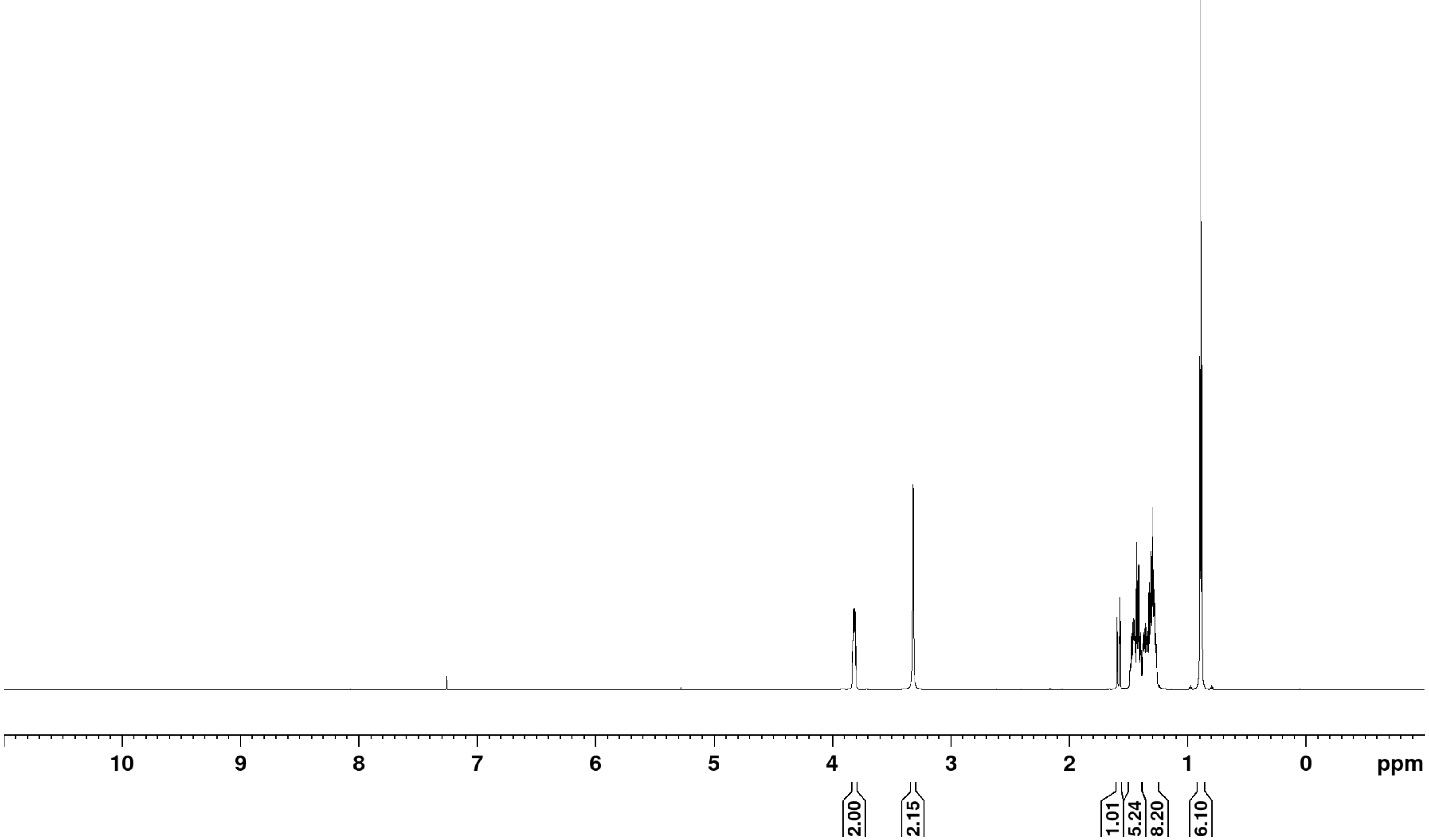

(*syn*)-undecane-5,7-diol (*meso*-1d)

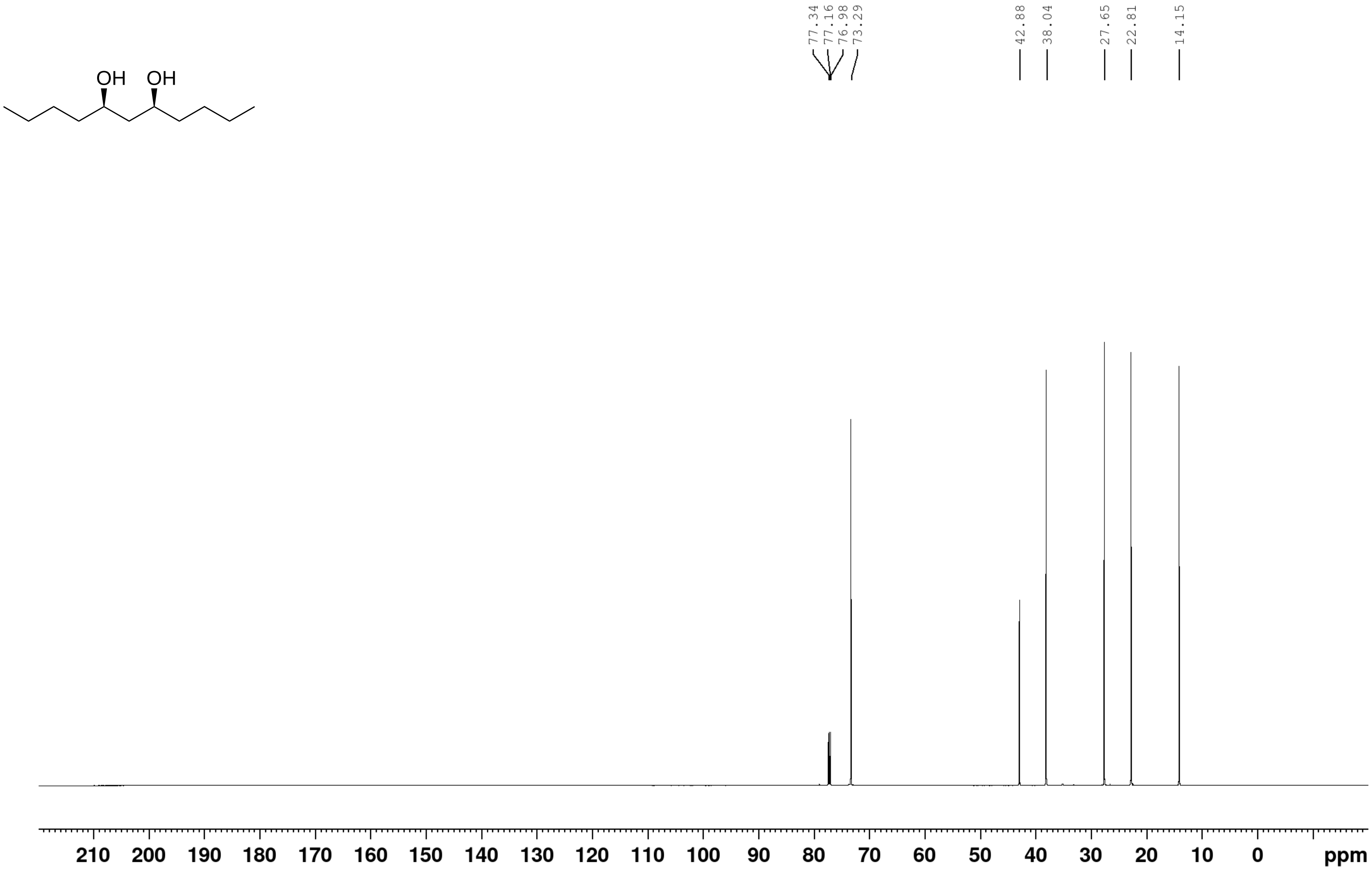

(5*S*,7*S*)-undecane-5,7-diol (1d)

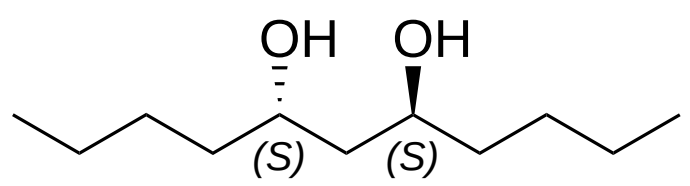

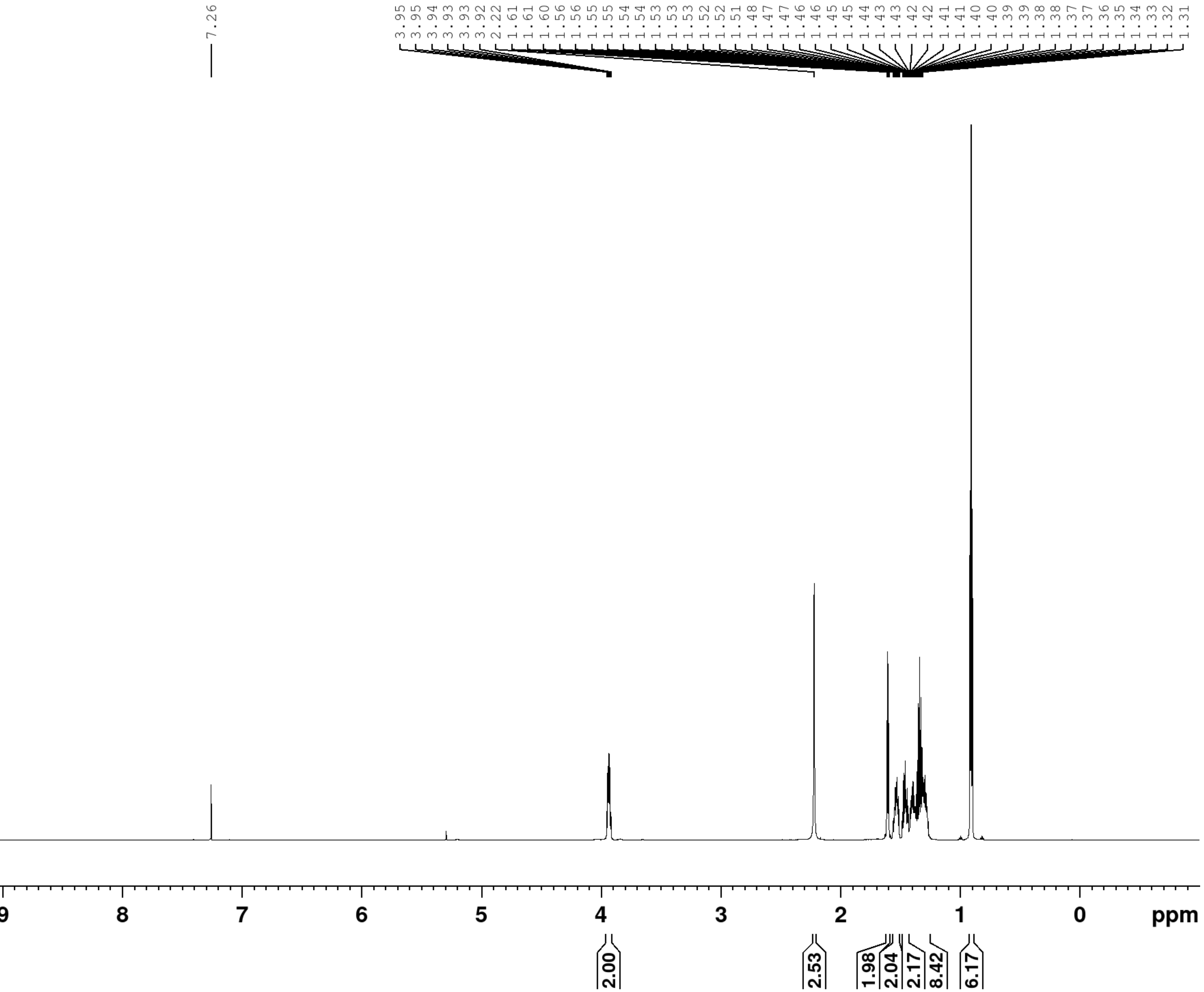

(5*S*,7*S*)-undecane-5,7-diol (1d)

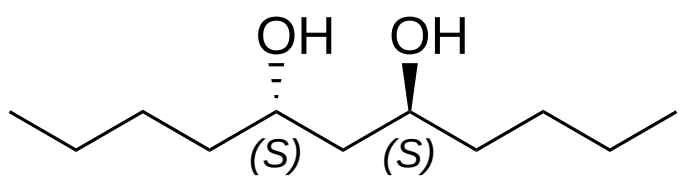

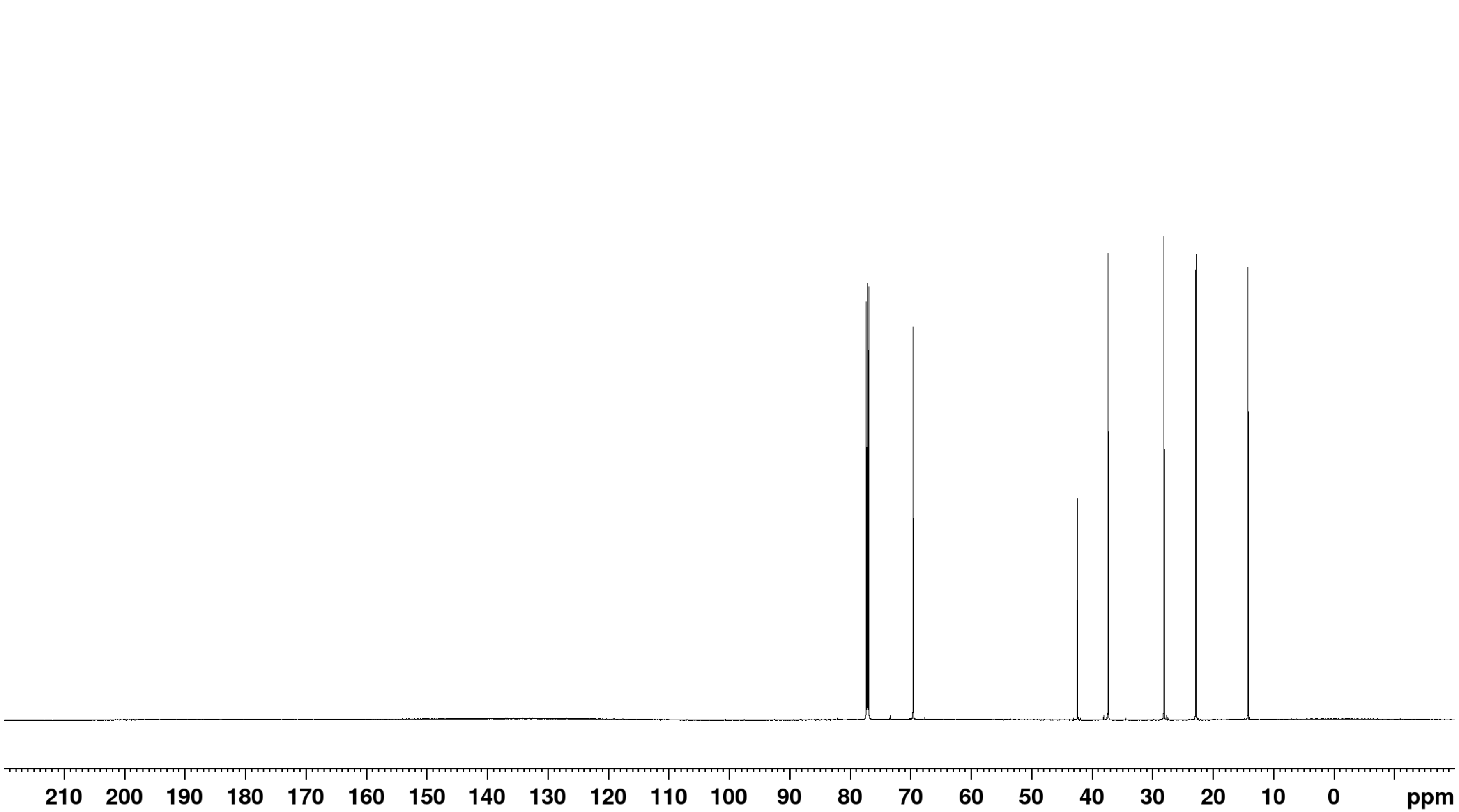

(5*S*,7*S*)-undecane-5,7-diyl dibenzoate (1d-Bz)

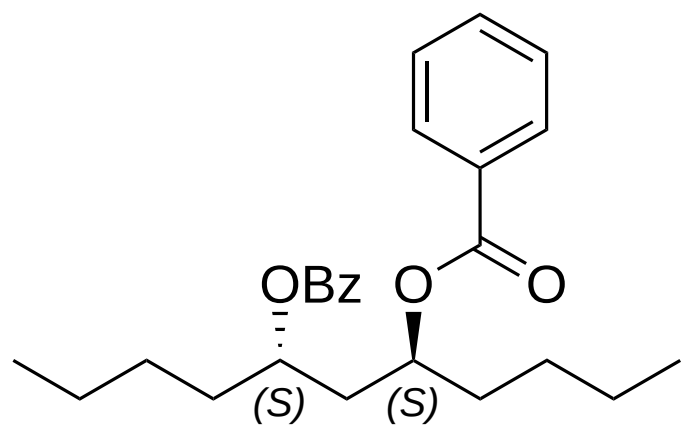

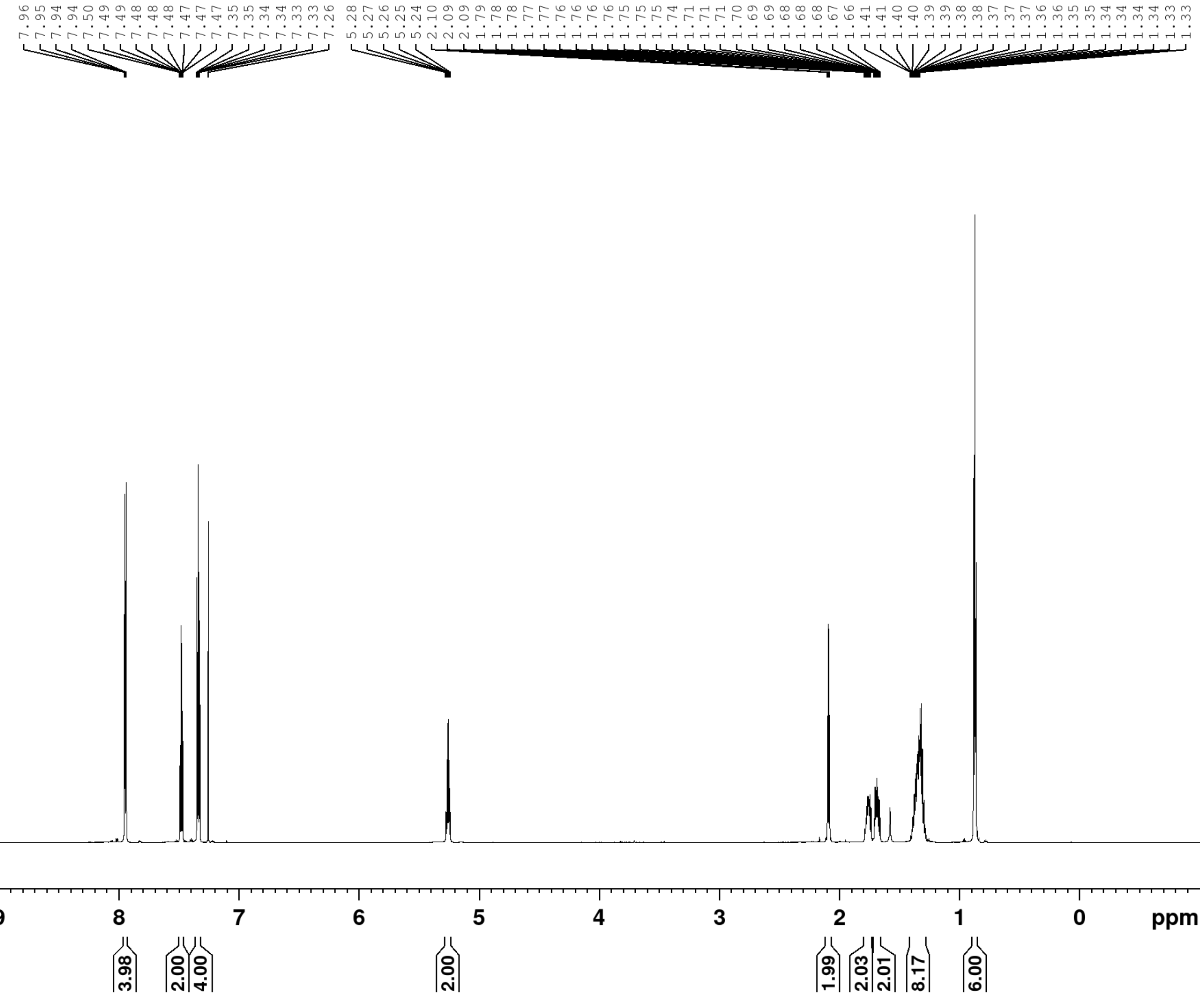

(5*S*,7*S*)-undecane-5,7-diyl dibenzoate (1d-Bz)

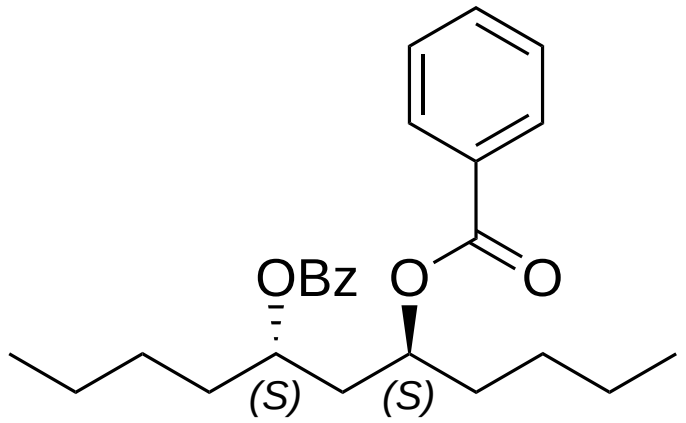

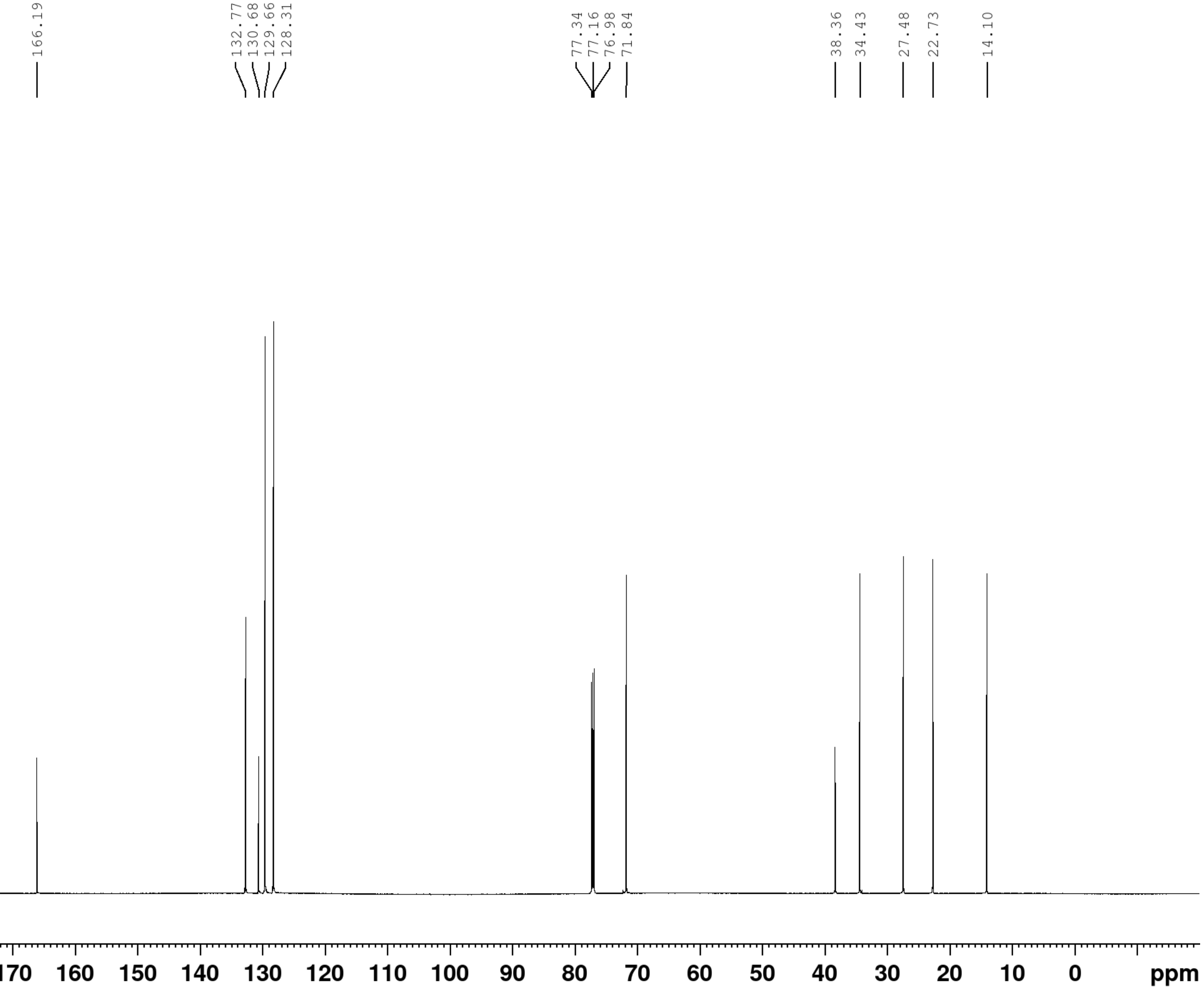

(*R*)-7-hydroxyundecan-5-one (2c)

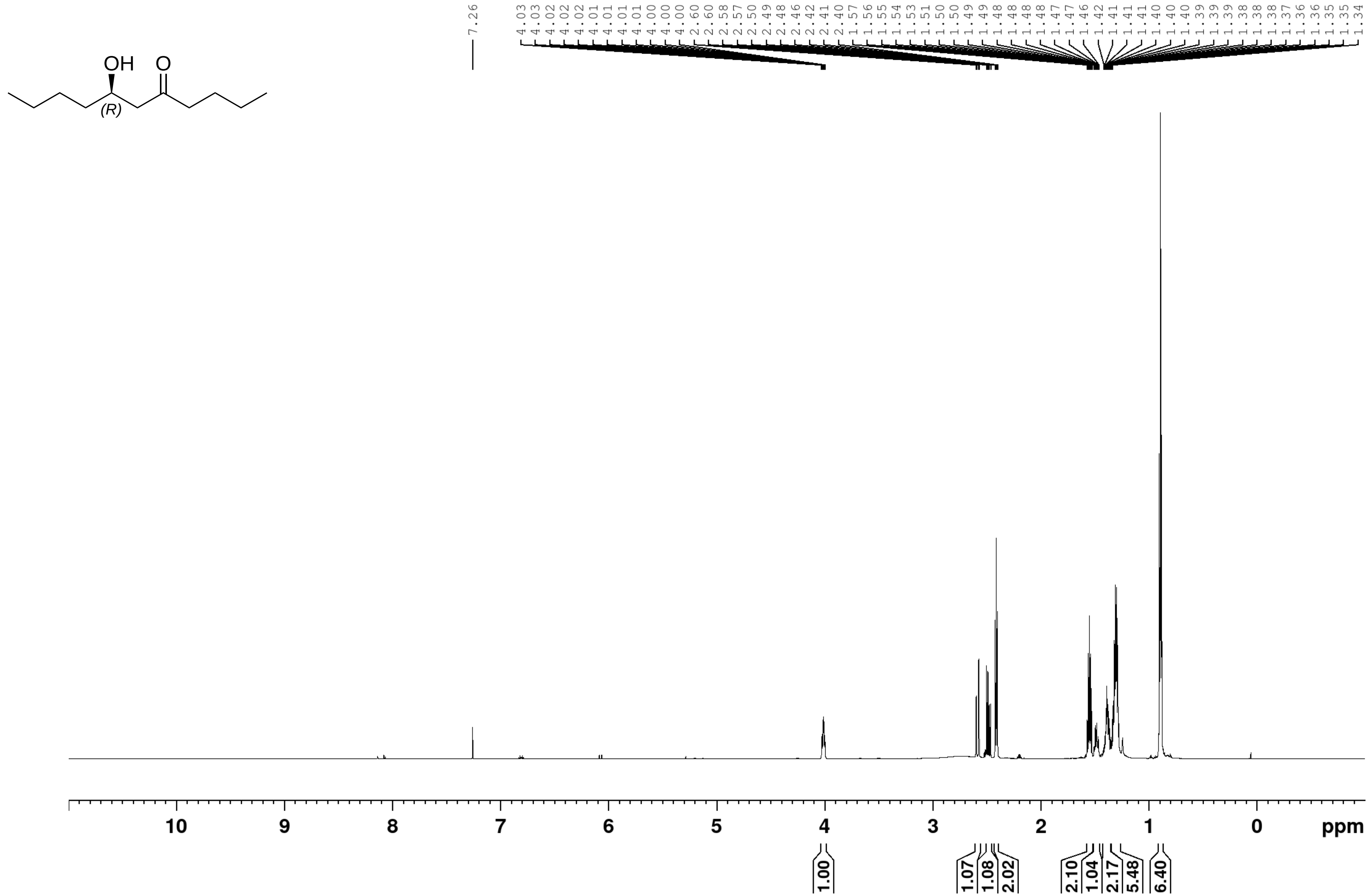

(*R*)-7-hydroxyundecan-5-one (2c)

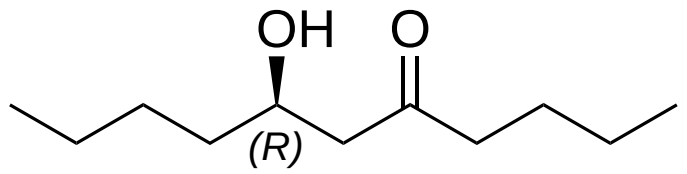

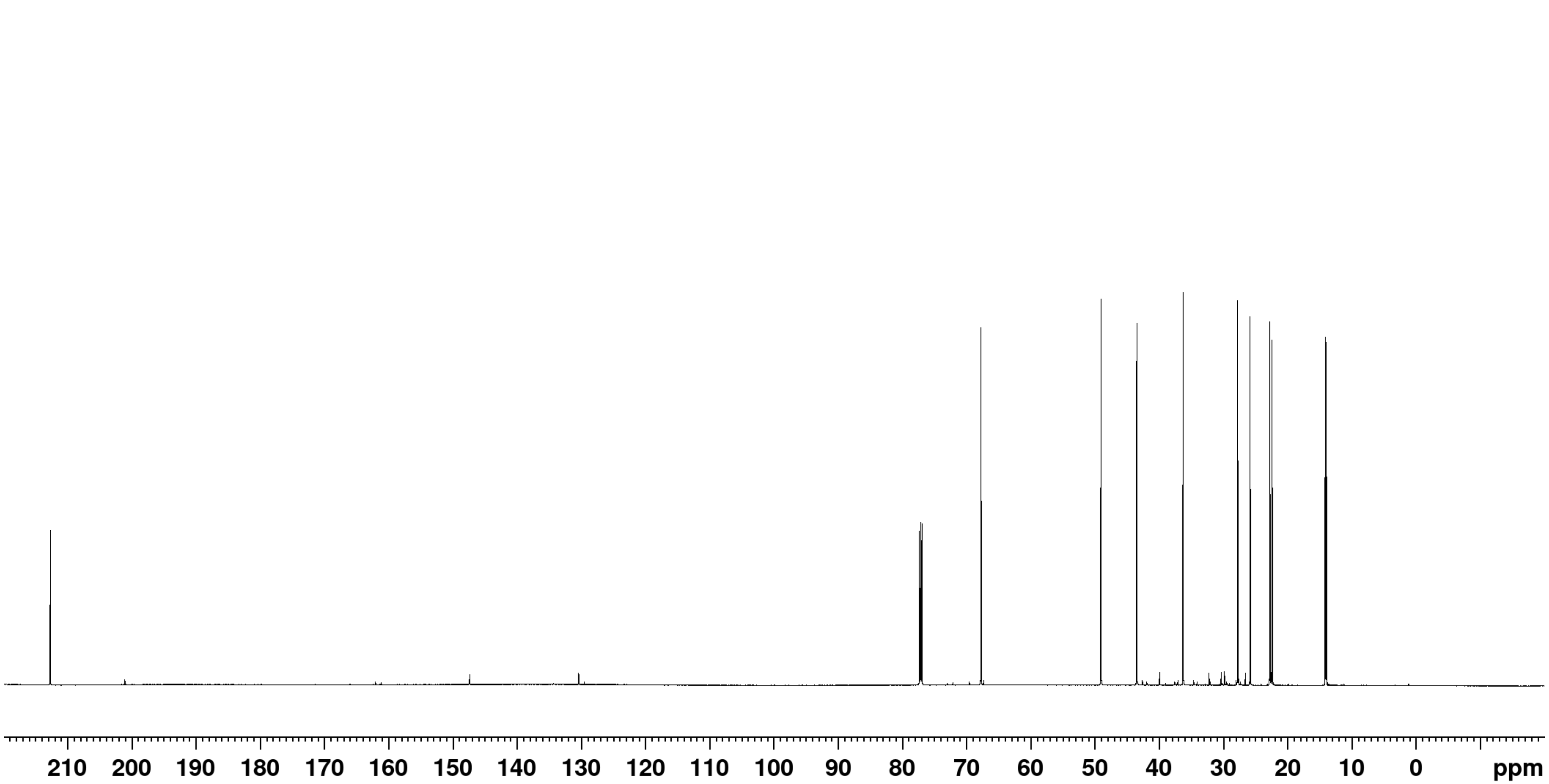

(*R*)-7-((*tert*-butyldiphenylsilyl)oxy)undecan-5-one (2c-TBDPS)

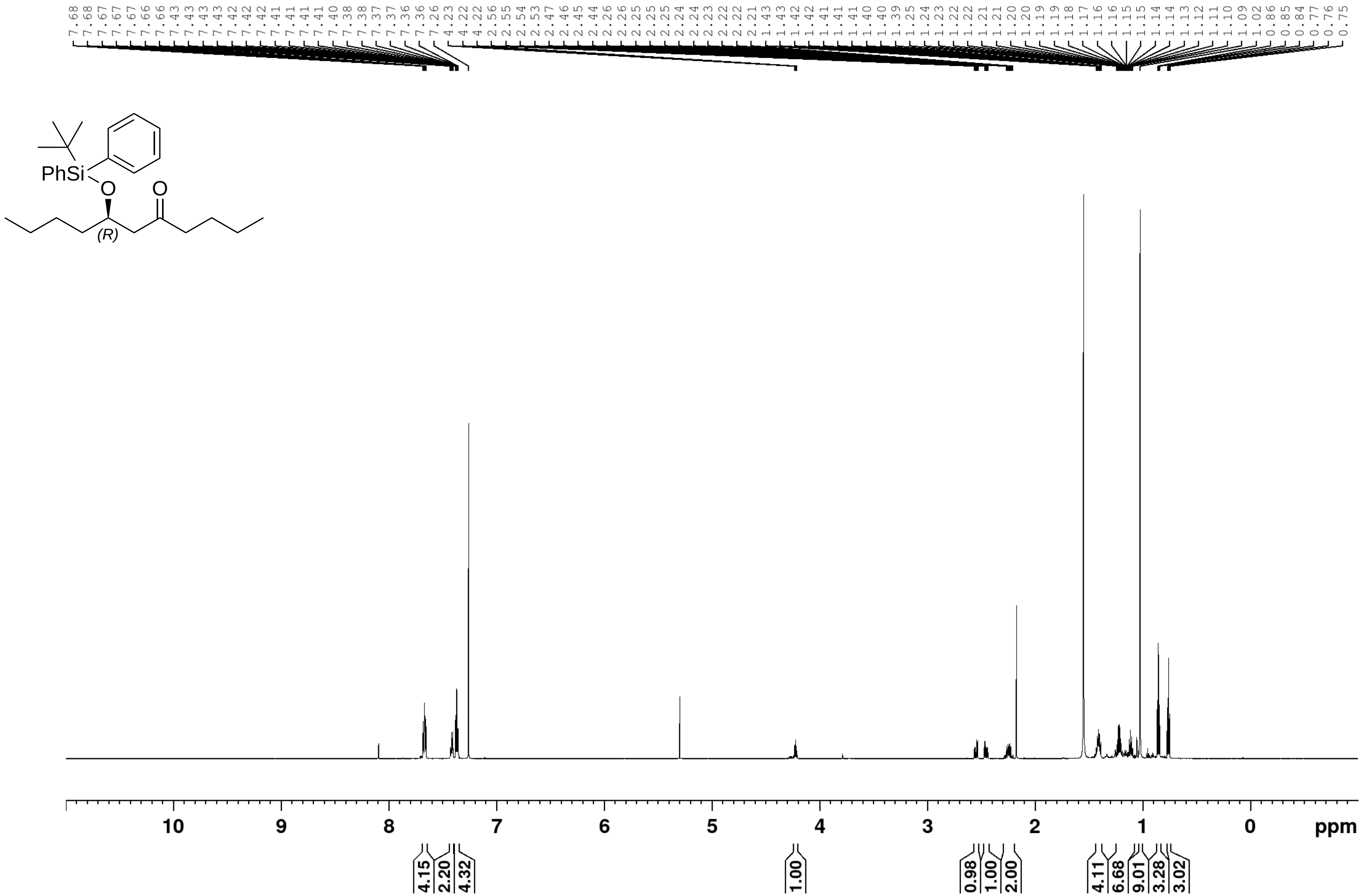

(*R*)-7-((*tert*-butyldiphenylsilyl)oxy)undecan-5-one (2c-TBDPS)

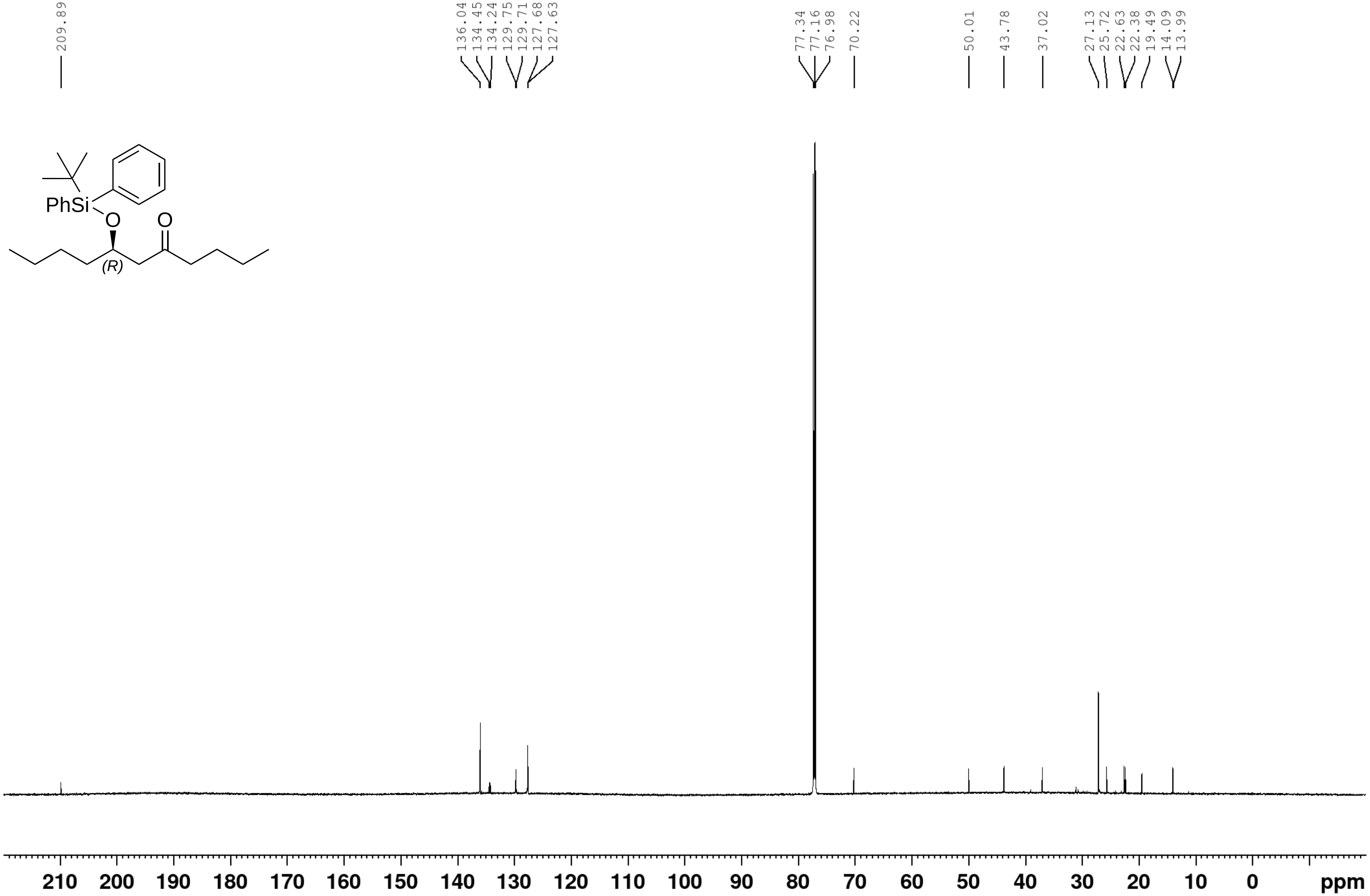

(*S*,*S*)-1-Phenylpentane-2,4-diol (*S*,*S*-3a)

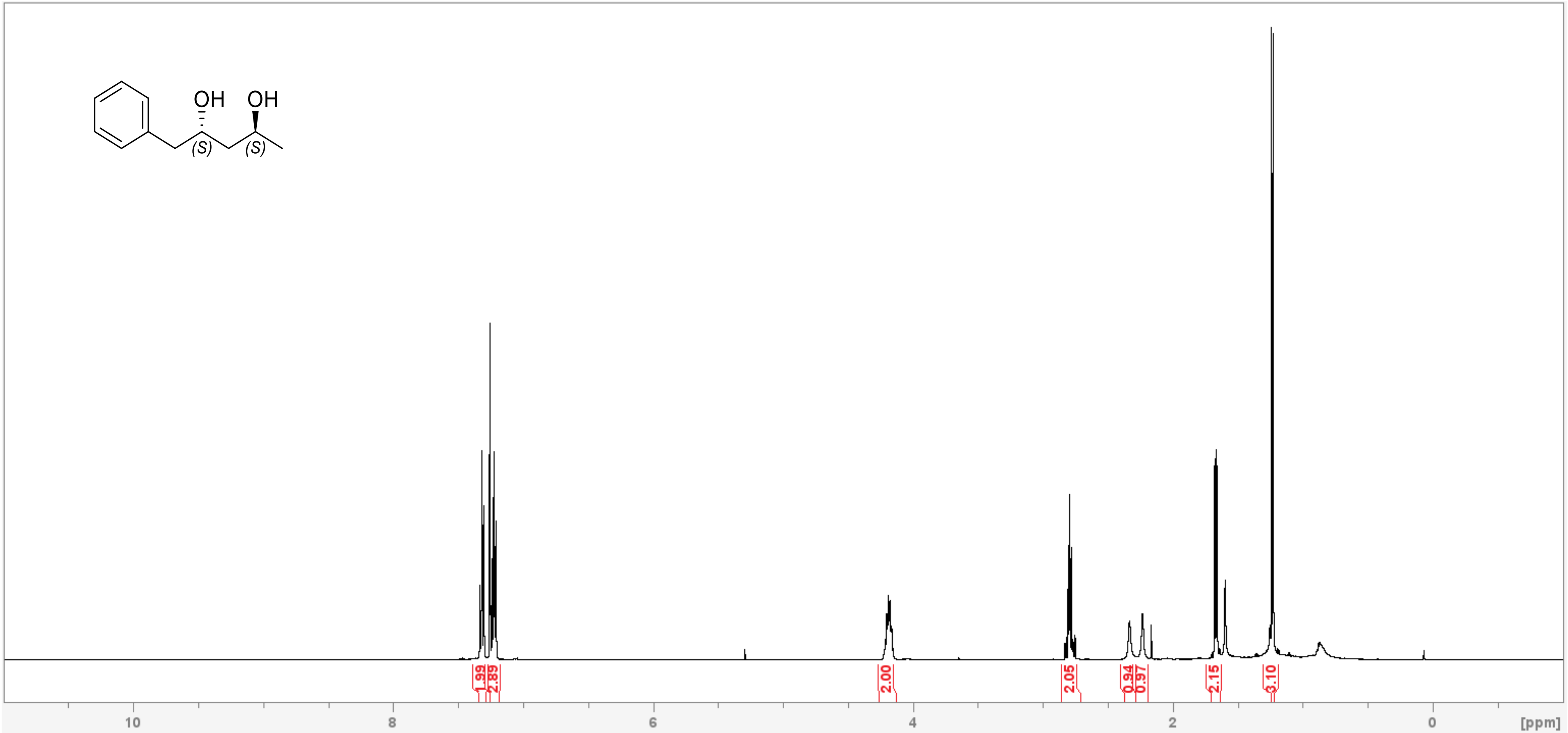

(*S*,*S*)-1-Phenylpentane-2,4-diol (*S*,*S*-3a)

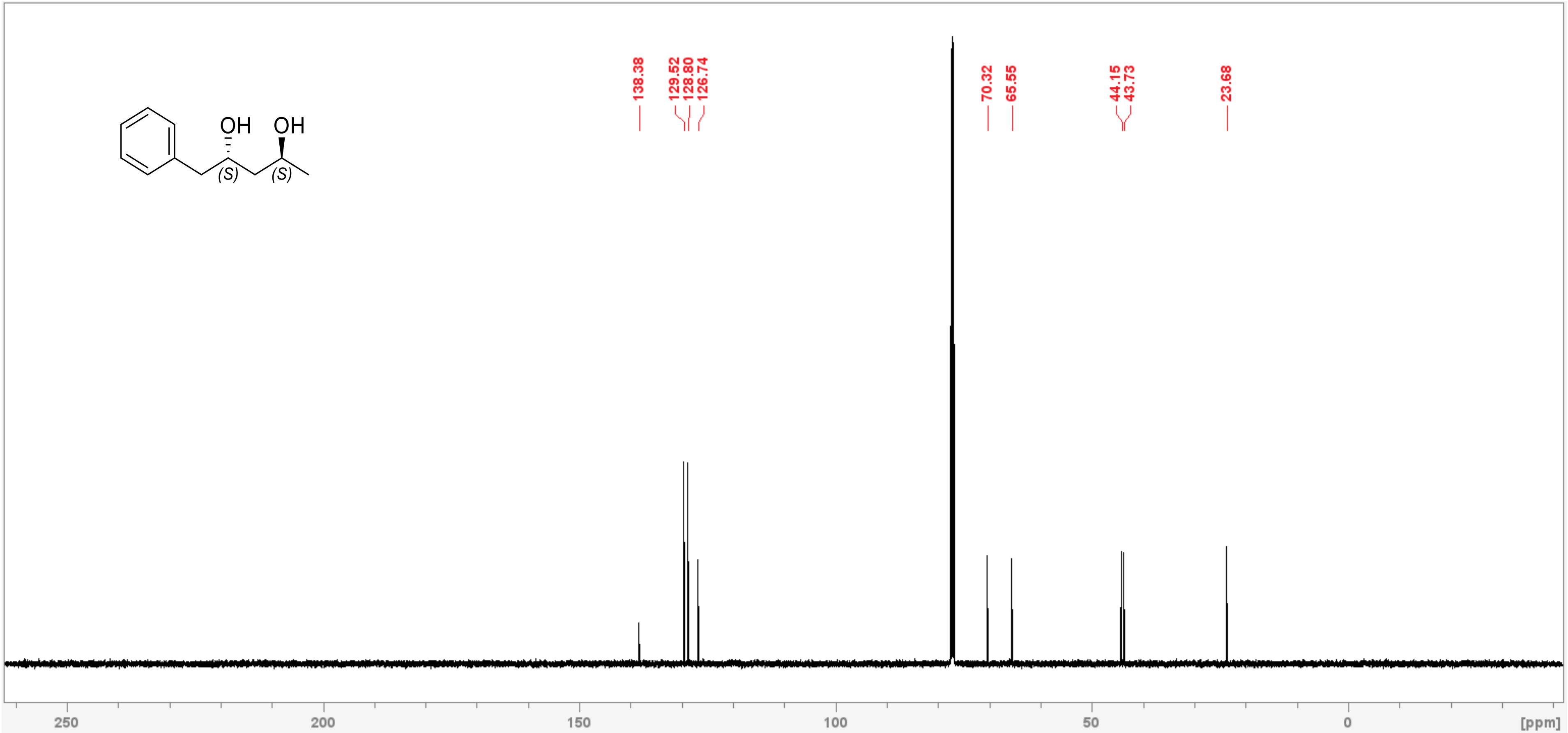

(2*R*,4*S*)-1-Phenylpentane-2,4-diol (2*R*,4*S*-3a)

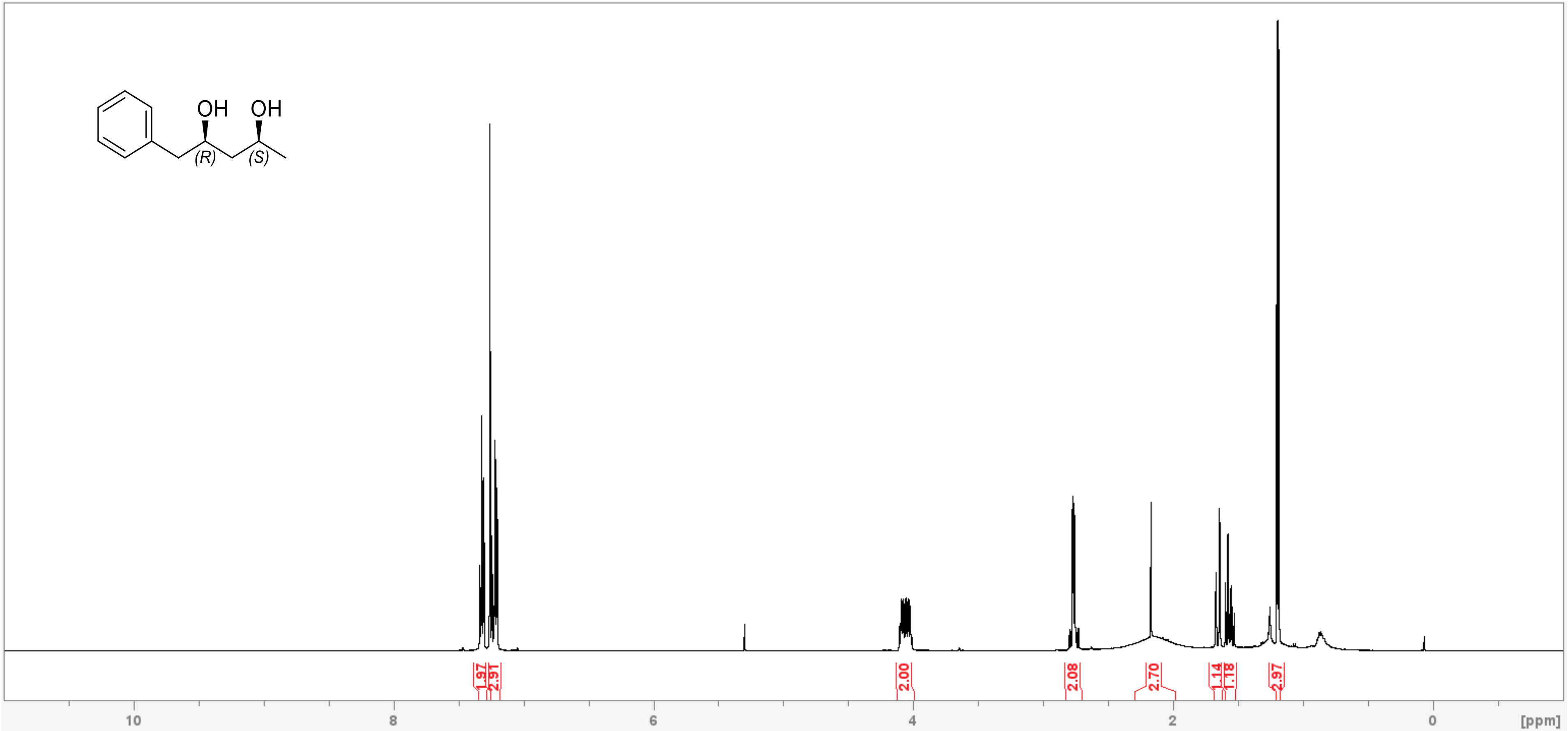

(2*R*,4*S*)-1-Phenylpentane-2,4-diol (2*R*,4*S*-3a)

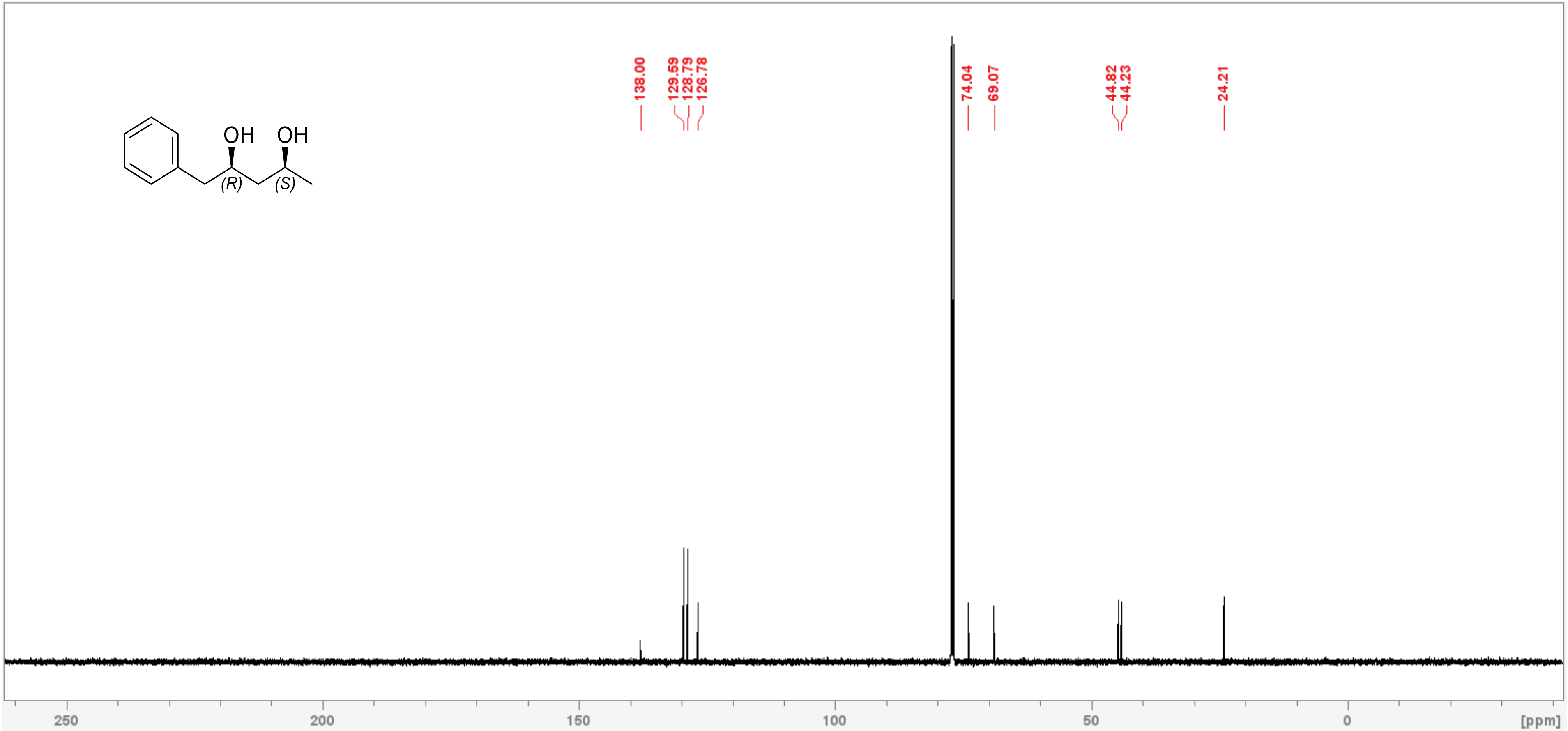

(*S*,*S*)-1-(4-Methoxyphenyl)pentane-2,4-diol (*S*,*S*-3b)

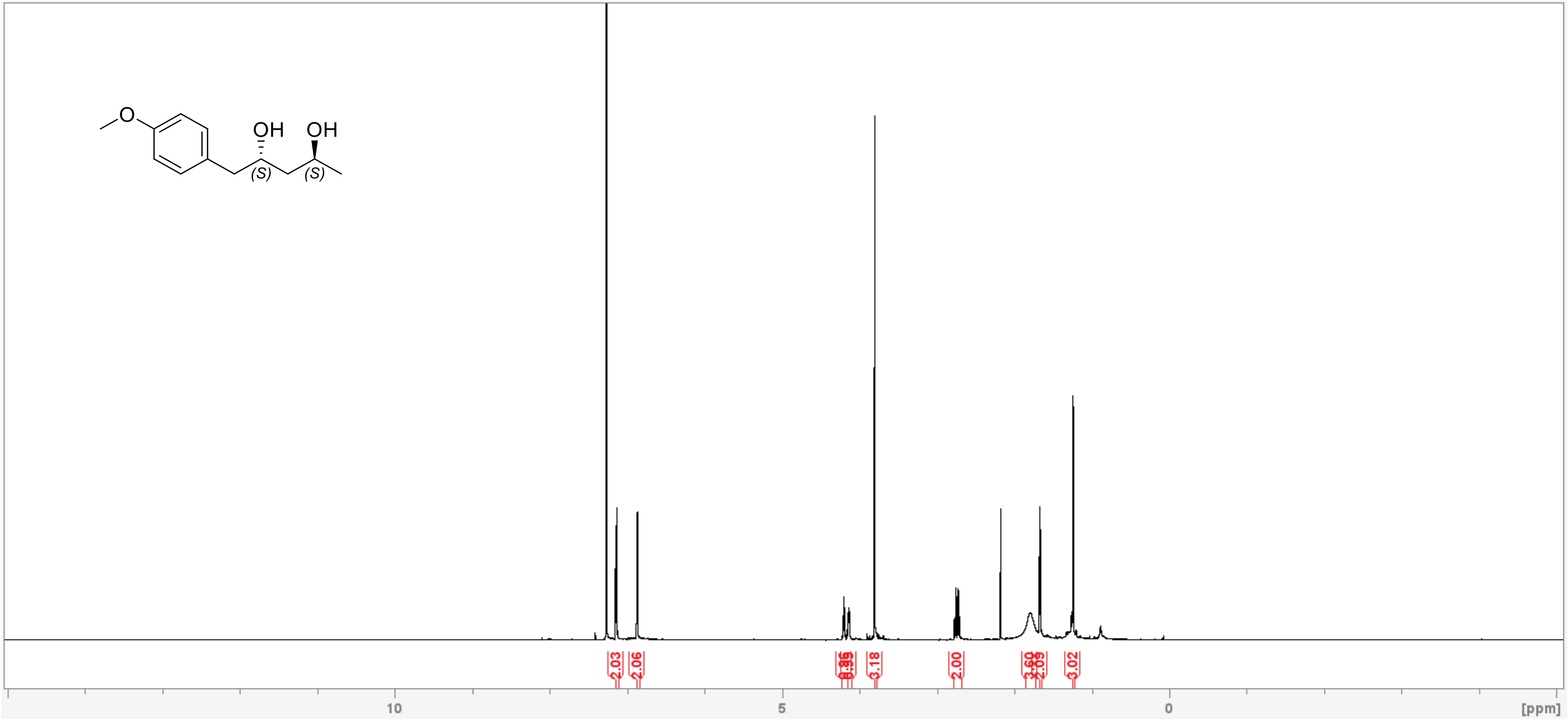

(*S*,*S*)-1-(4-Methoxyphenyl)pentane-2,4-diol (*S*,*S*-3b)

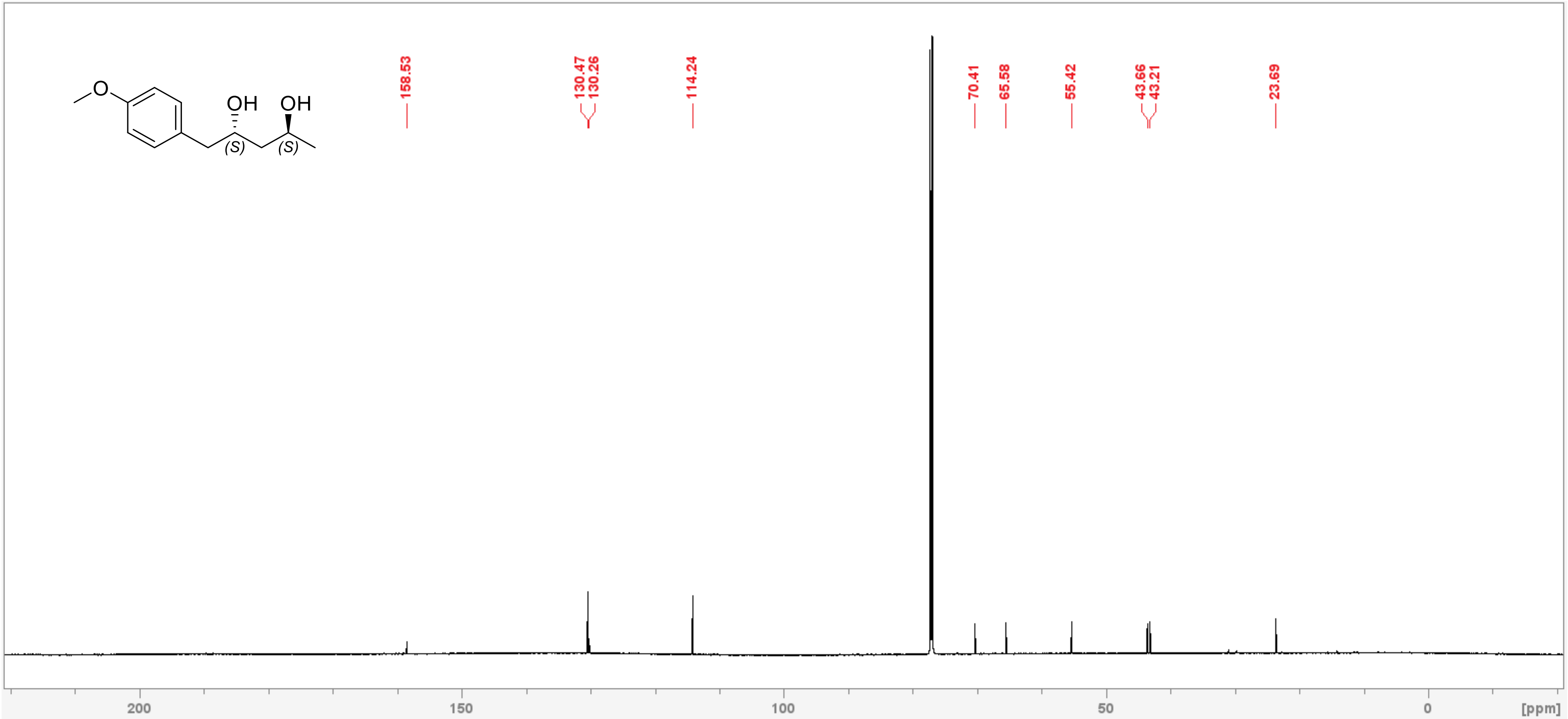

(2*R*,4*S*)-1-(4-Methoxyphenyl)pentane-2,4-diol (2*R*,4*S*-3b)

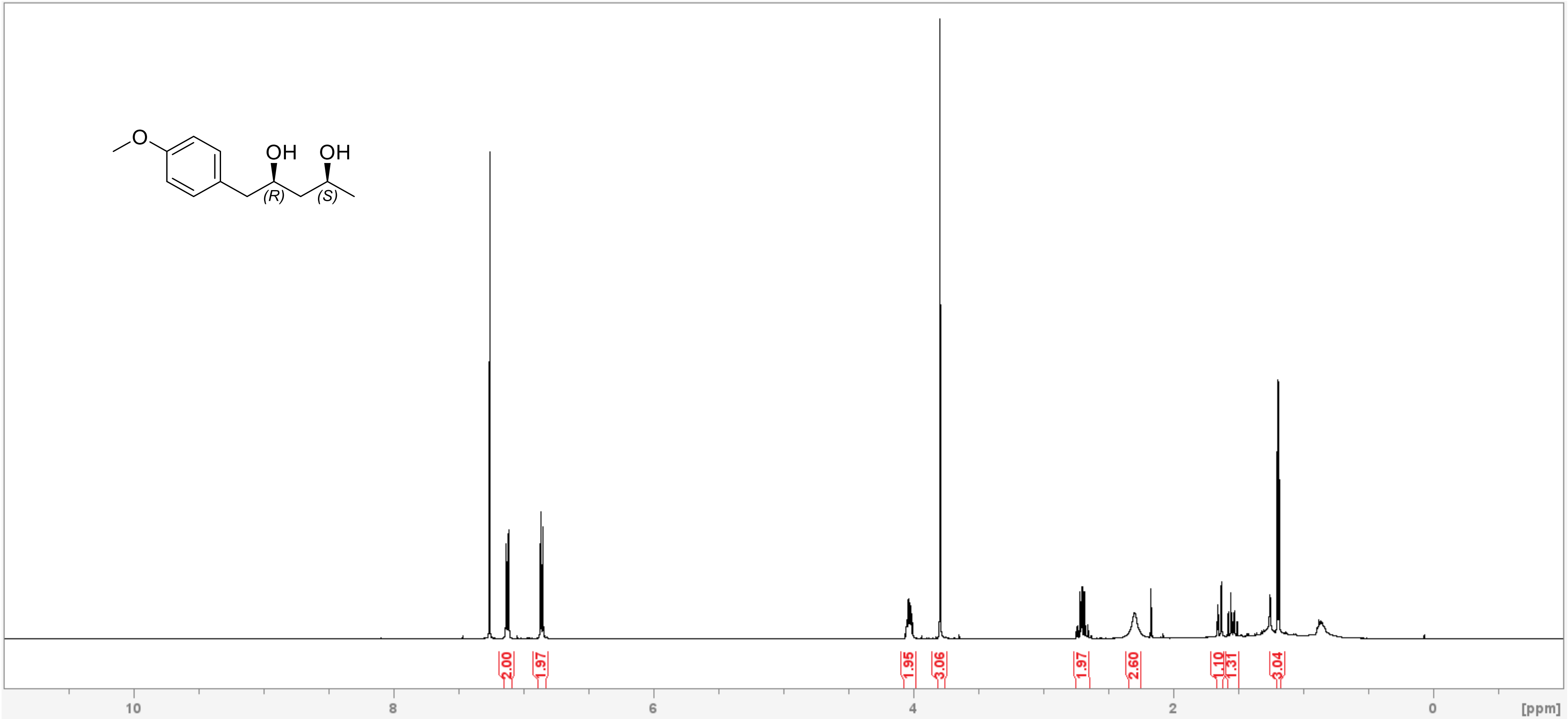

(2*R*,4*S*)-1-(4-Methoxyphenyl)pentane-2,4-diol (2*R*,4*S*-3b)

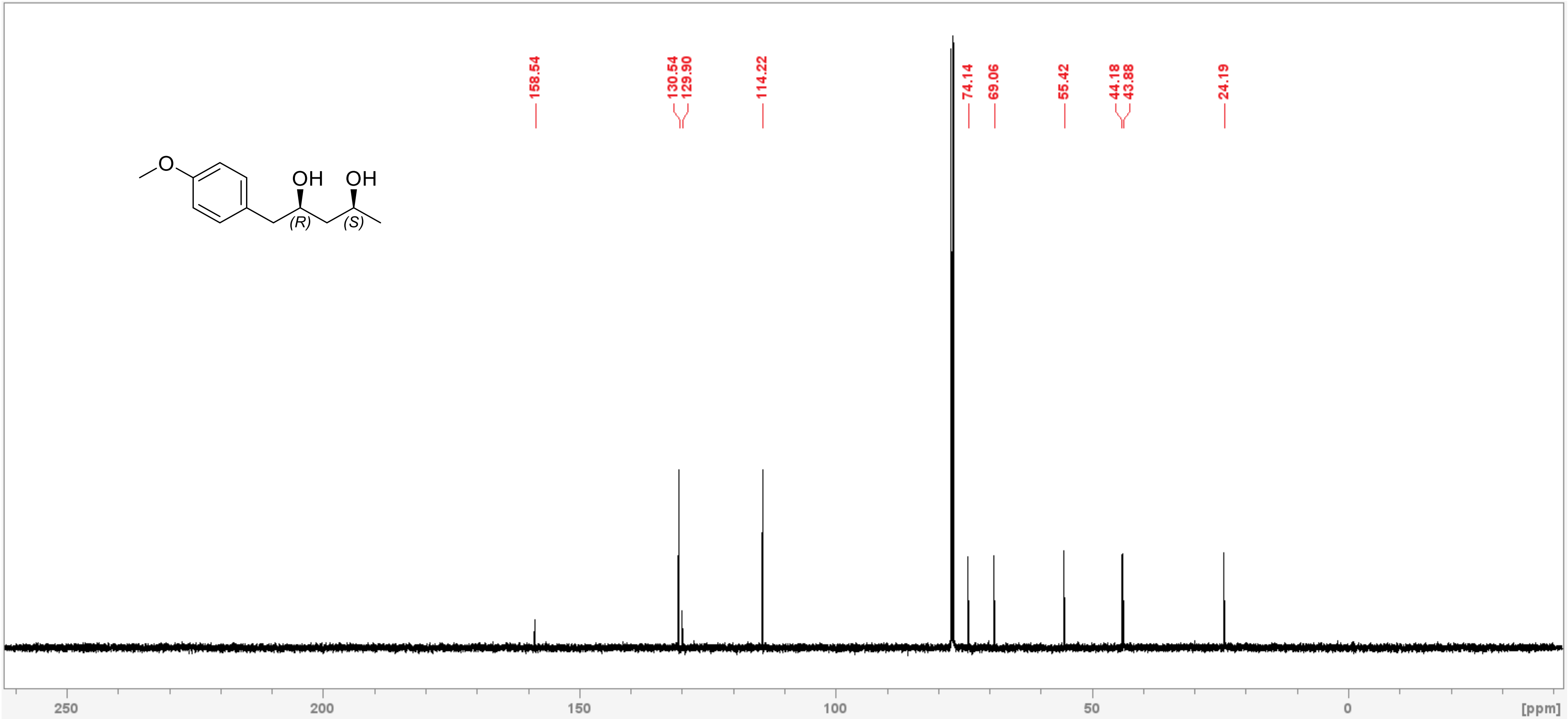

(*S*,*S*)-1-(Naphthalen-1-yl)pentane-2,4-diol (*S*,*S*-3c)

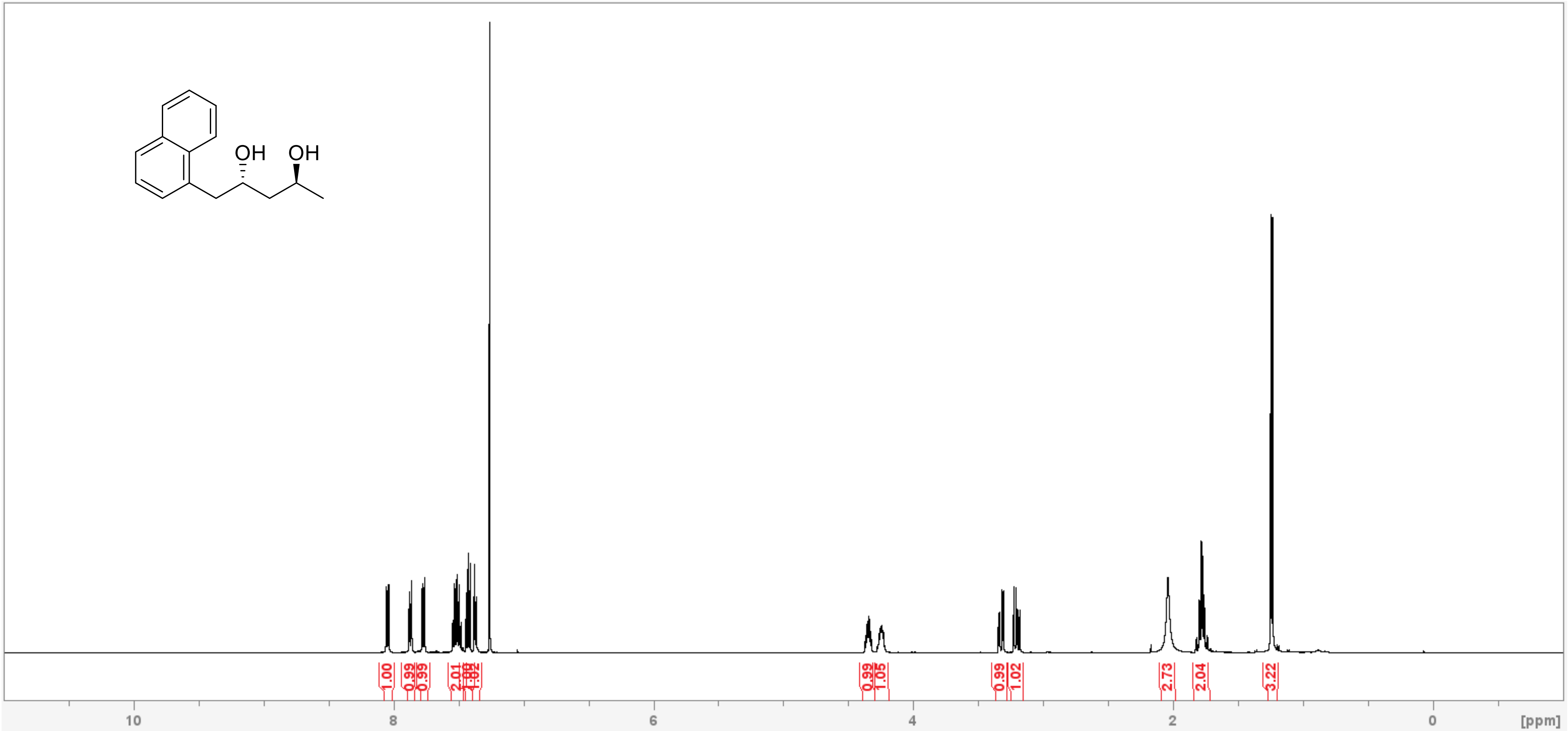

(*S*,*S*)-1-(Naphthalen-1-yl)pentane-2,4-diol (*S*,*S*-3c)

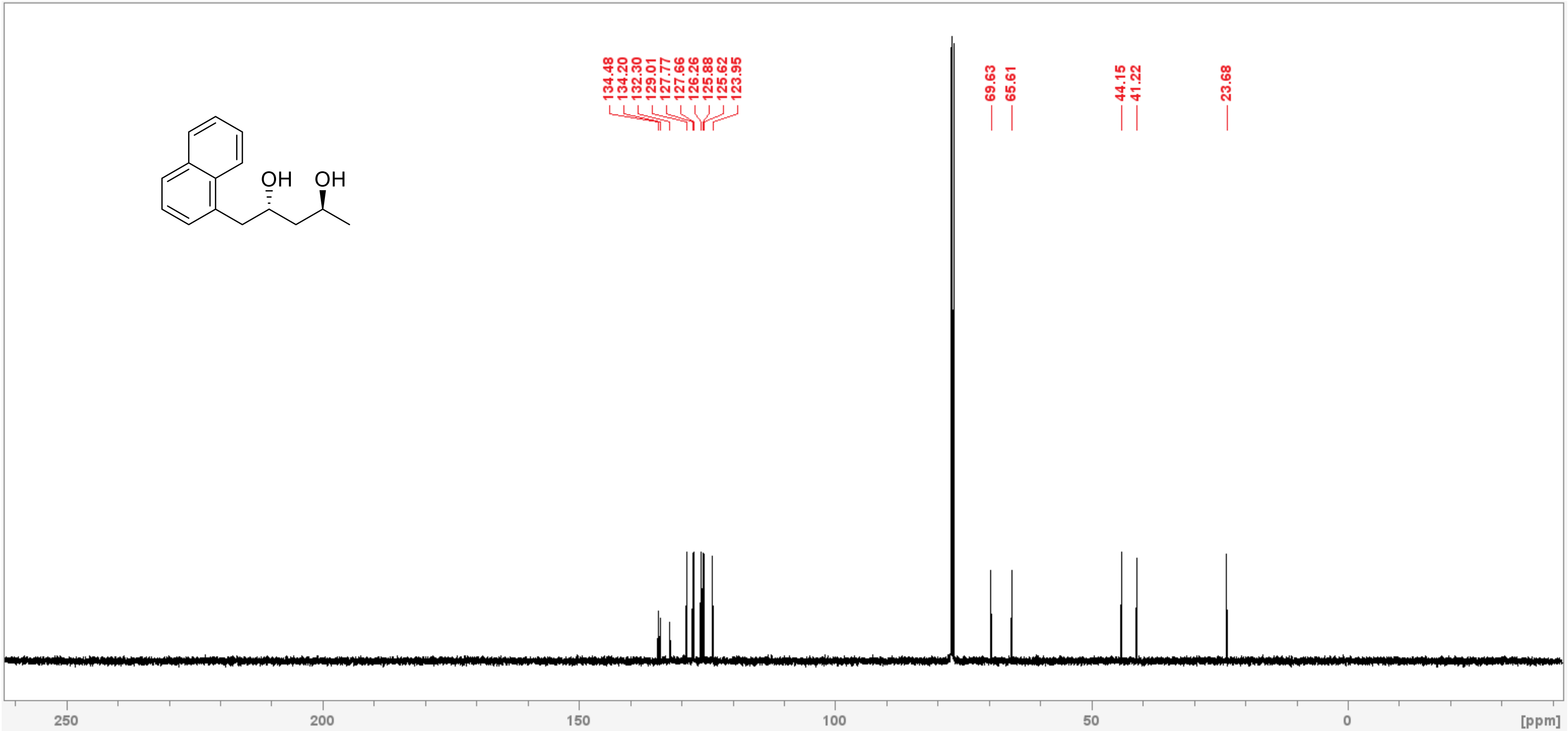

(2*R*,4*S*)-1-(Naphthalen-1-yl)pentane-2,4-diol (2*R*,4*S*-3c)

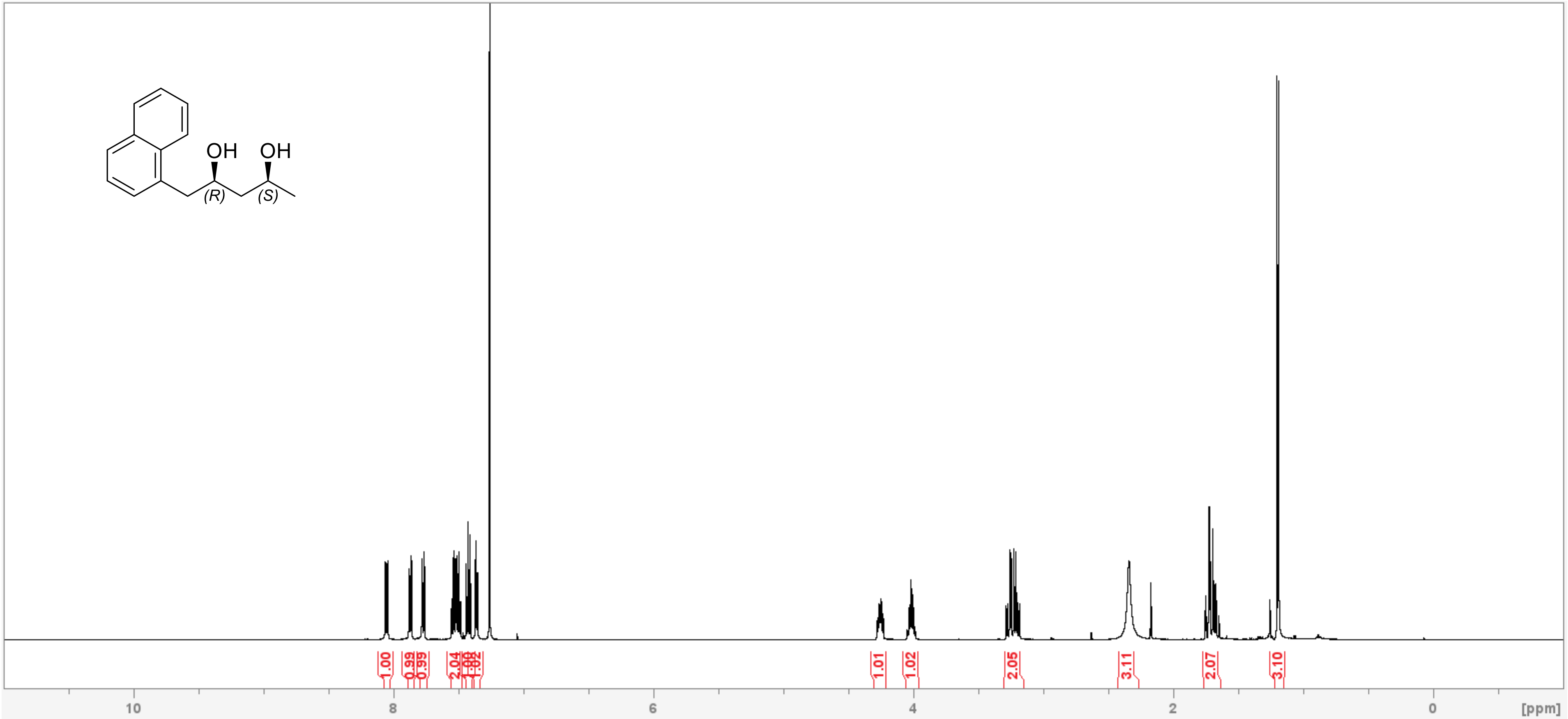

(2*R*,4*S*)-1-(Naphthalen-1-yl)pentane-2,4-diol (2*R*,4*S*-3c)

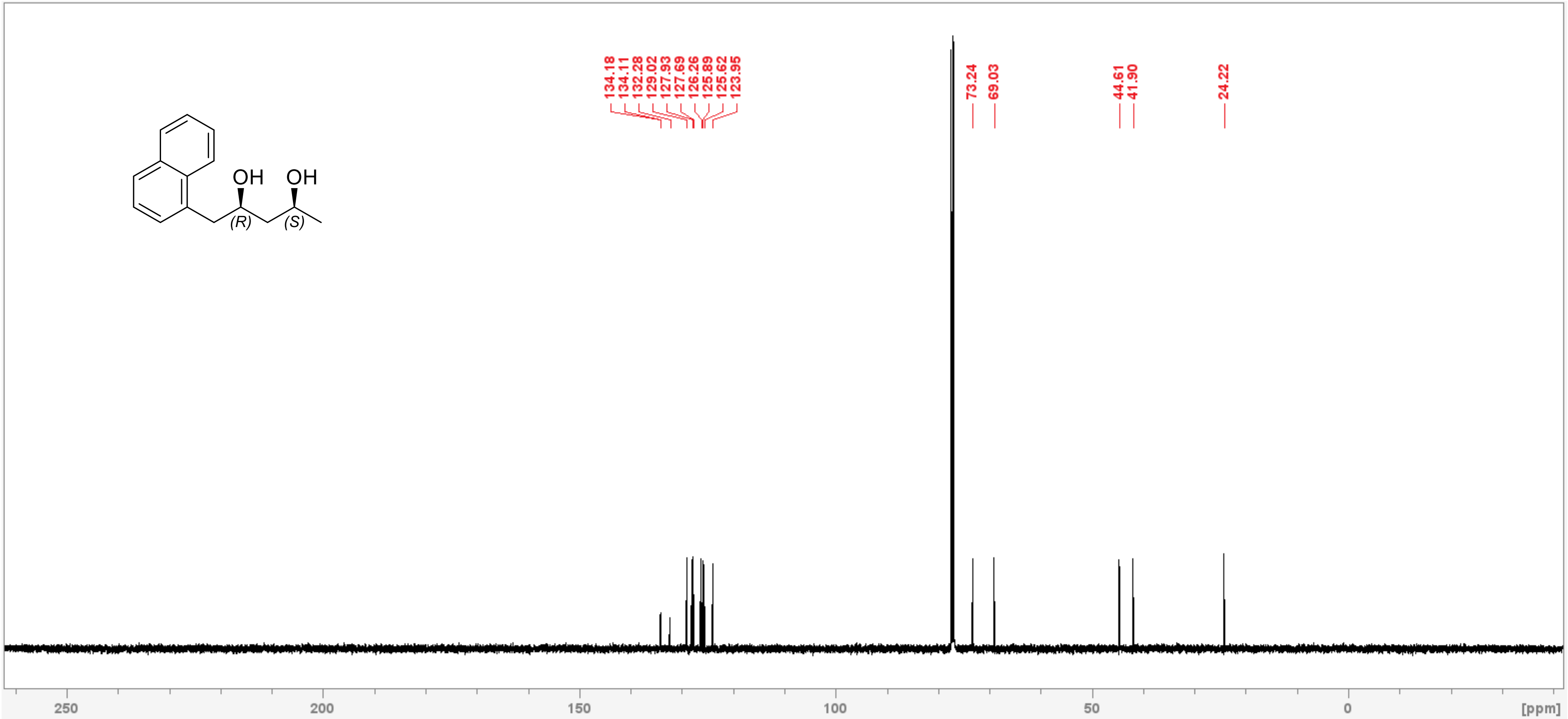

(*S*,*S*)-6-Phenylhexane-2,4-diol (*S*,*S*-3d)

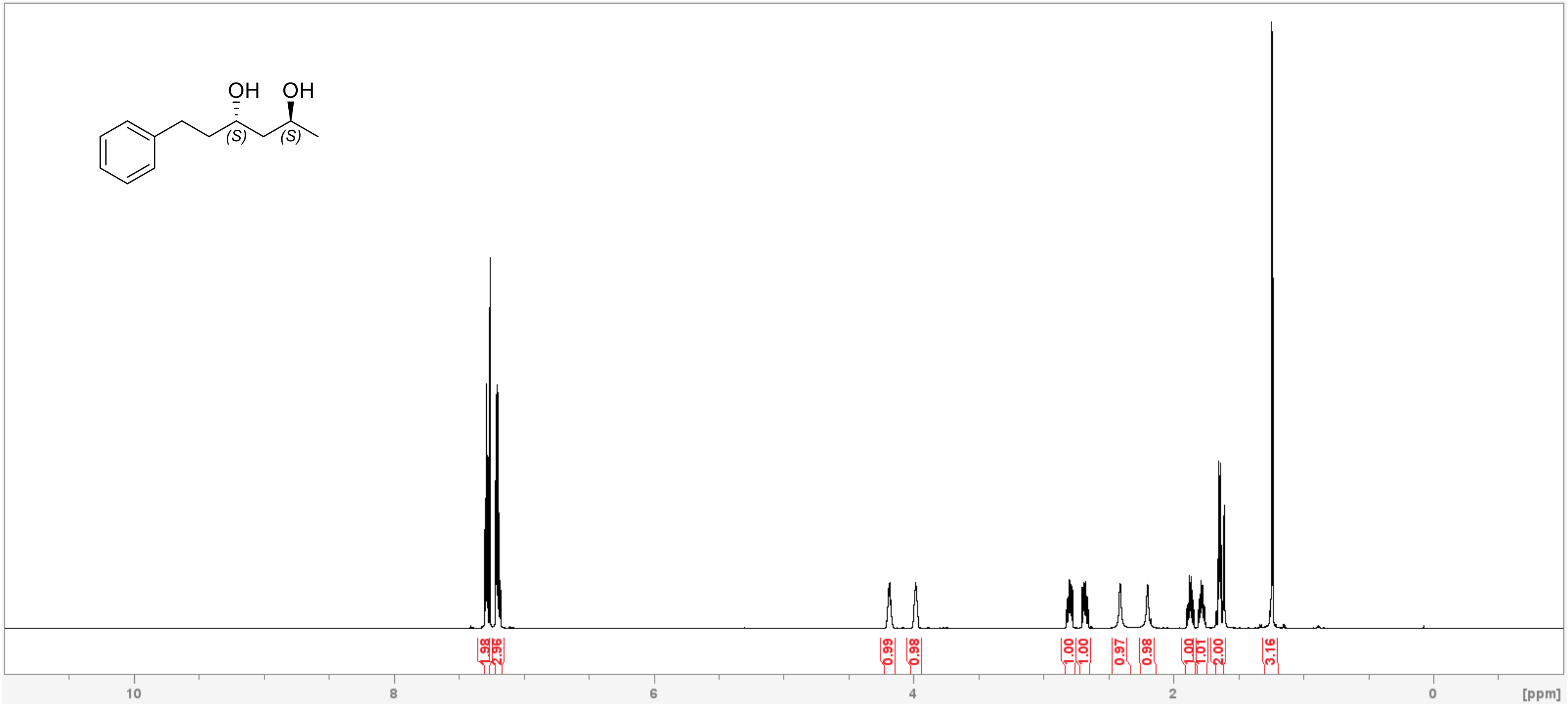

(*S*,*S*)-6-Phenylhexane-2,4-diol (*S*,*S*-3d)

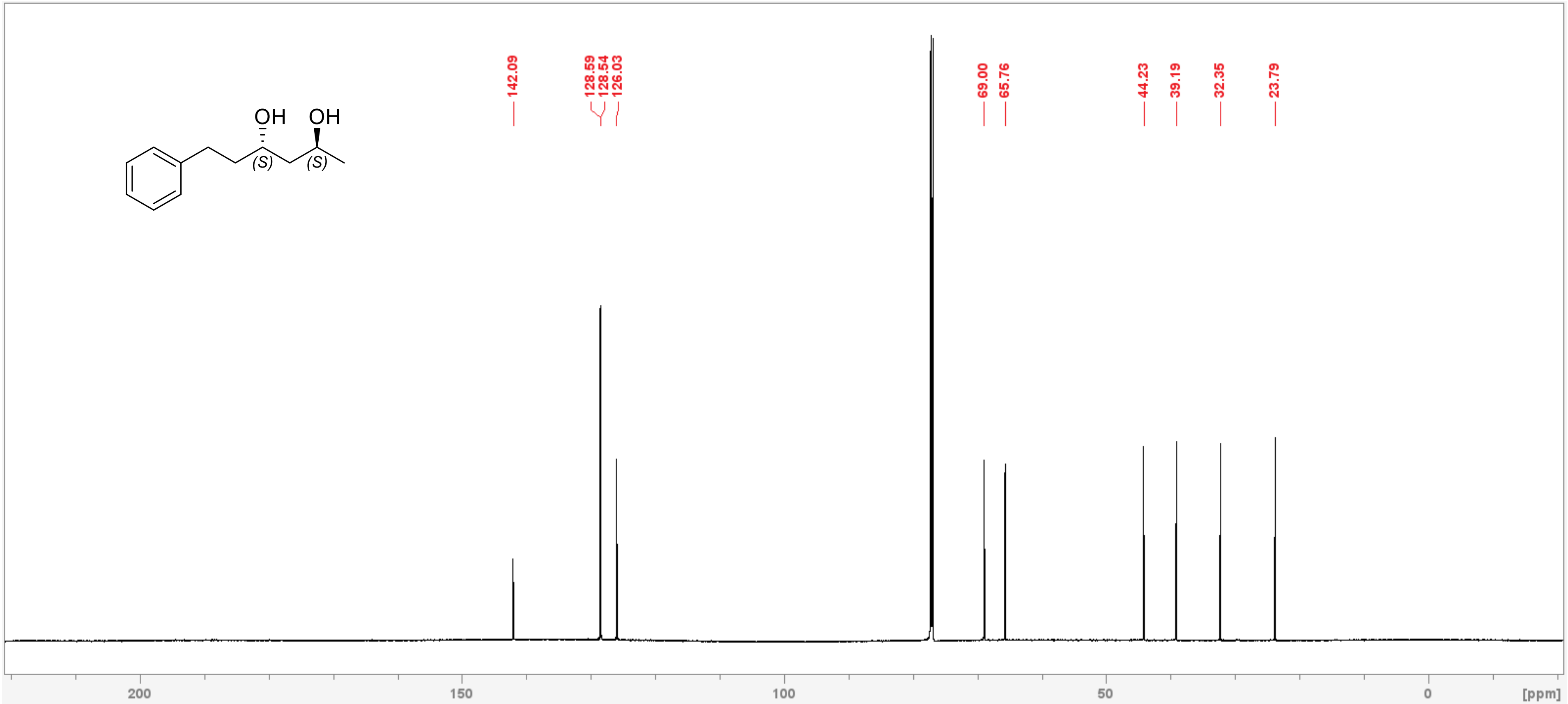

(2*S*,4*R*)-6-Phenylhexane-2,4-diol (2*S*,4*R*-3d)

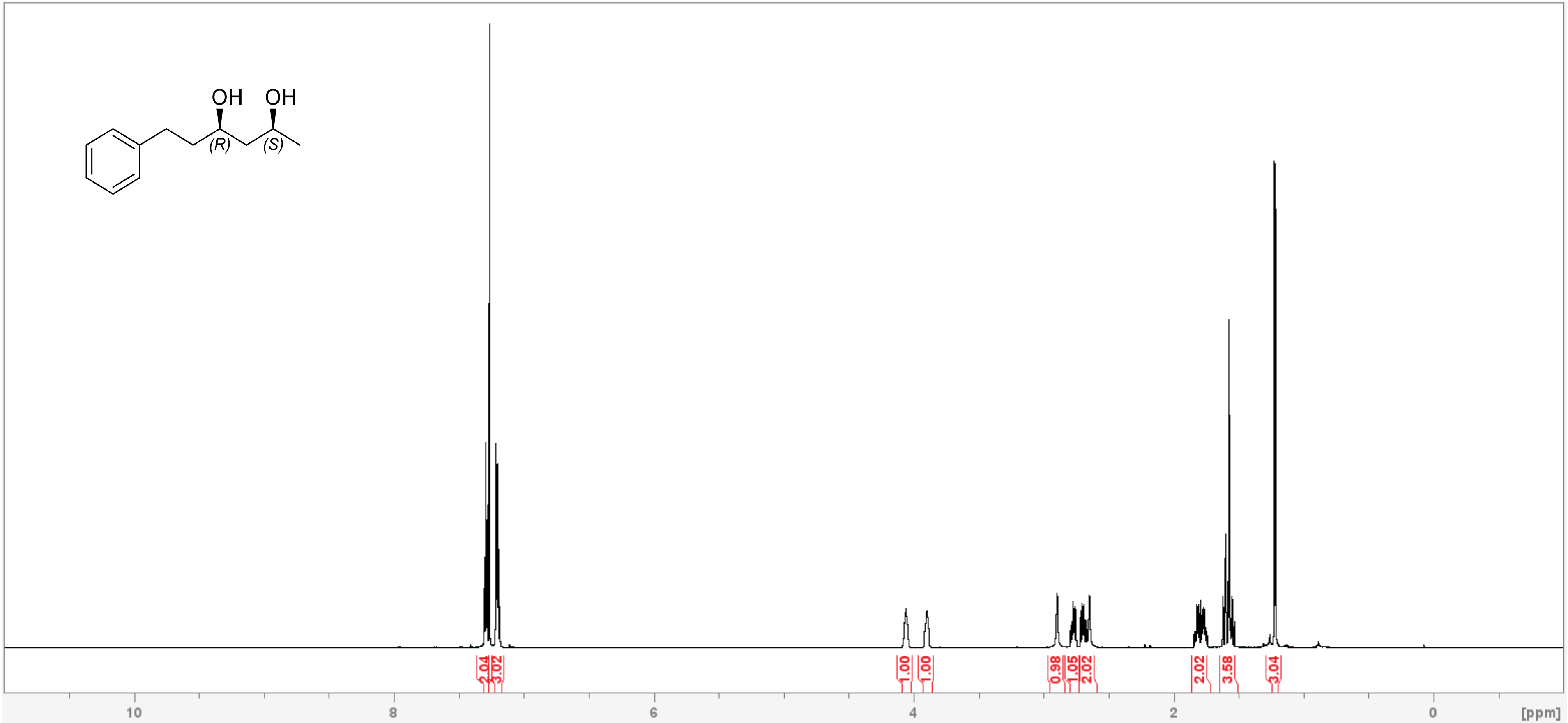

(2*S*,4*R*)-6-Phenylhexane-2,4-diol (2*S*,4*R*-3d)

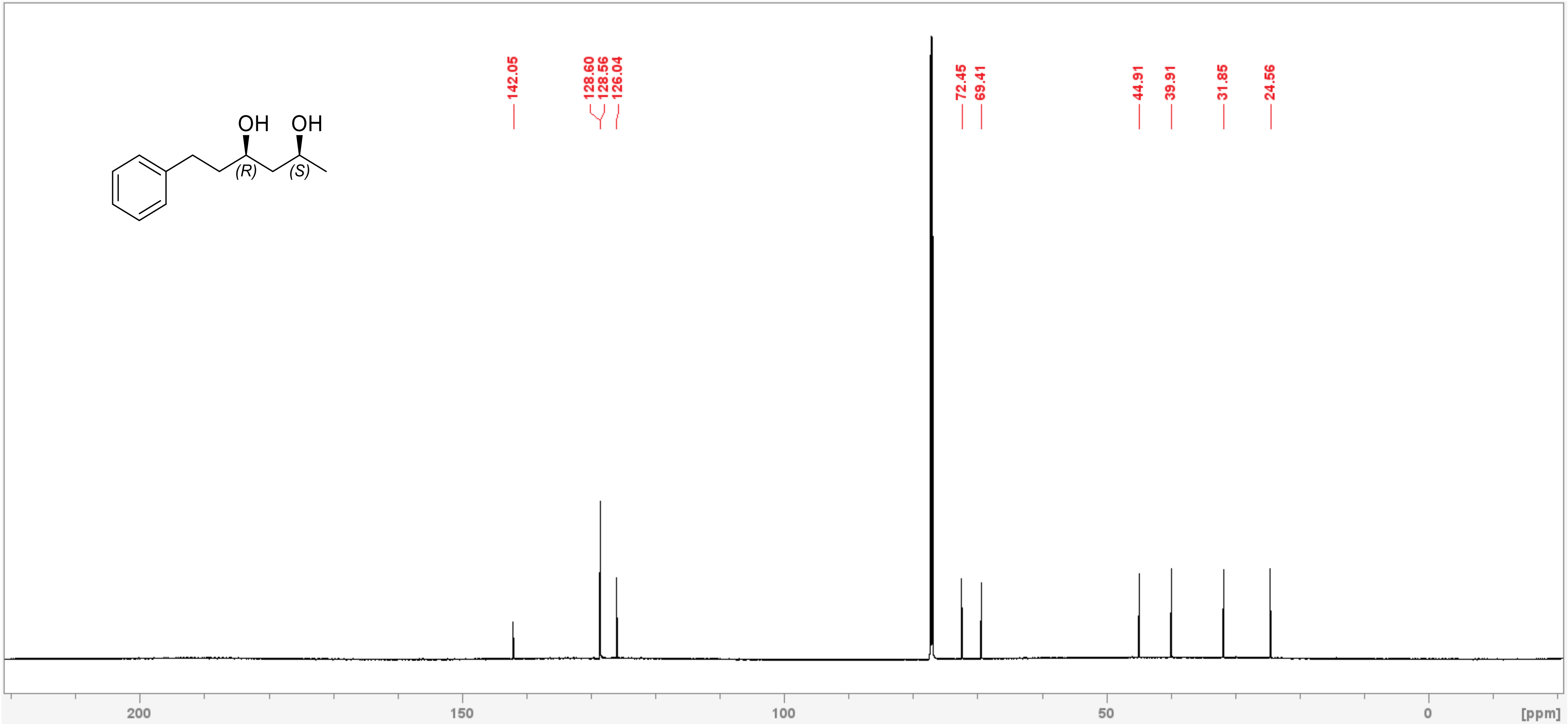

Ethyl 3-((*S*,*S*)-3,5-dihydroxyhexyl)benzoate (*S*,*S*-3e)

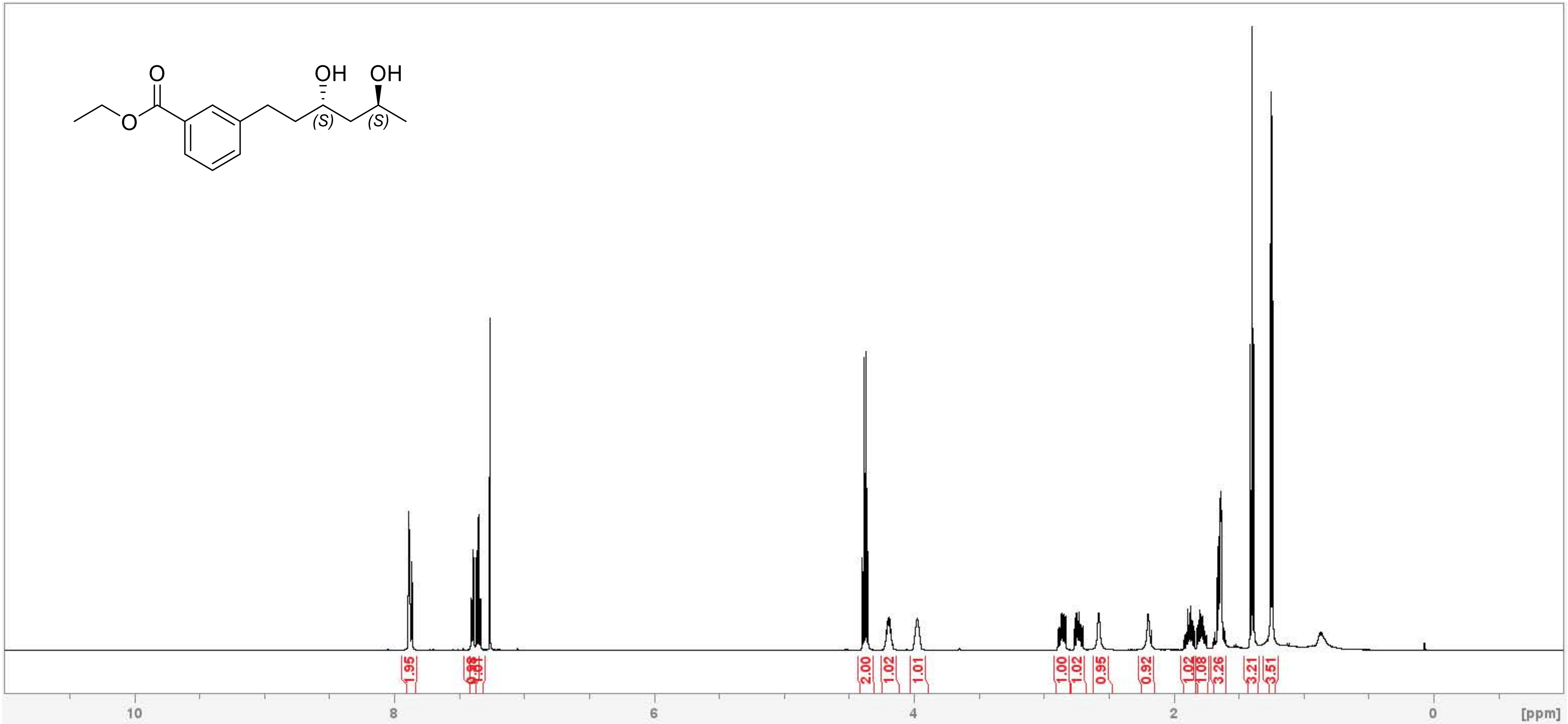

Ethyl 3-((*S*,*S*)-3,5-dihydroxyhexyl)benzoate (*S*,*S*-3e)

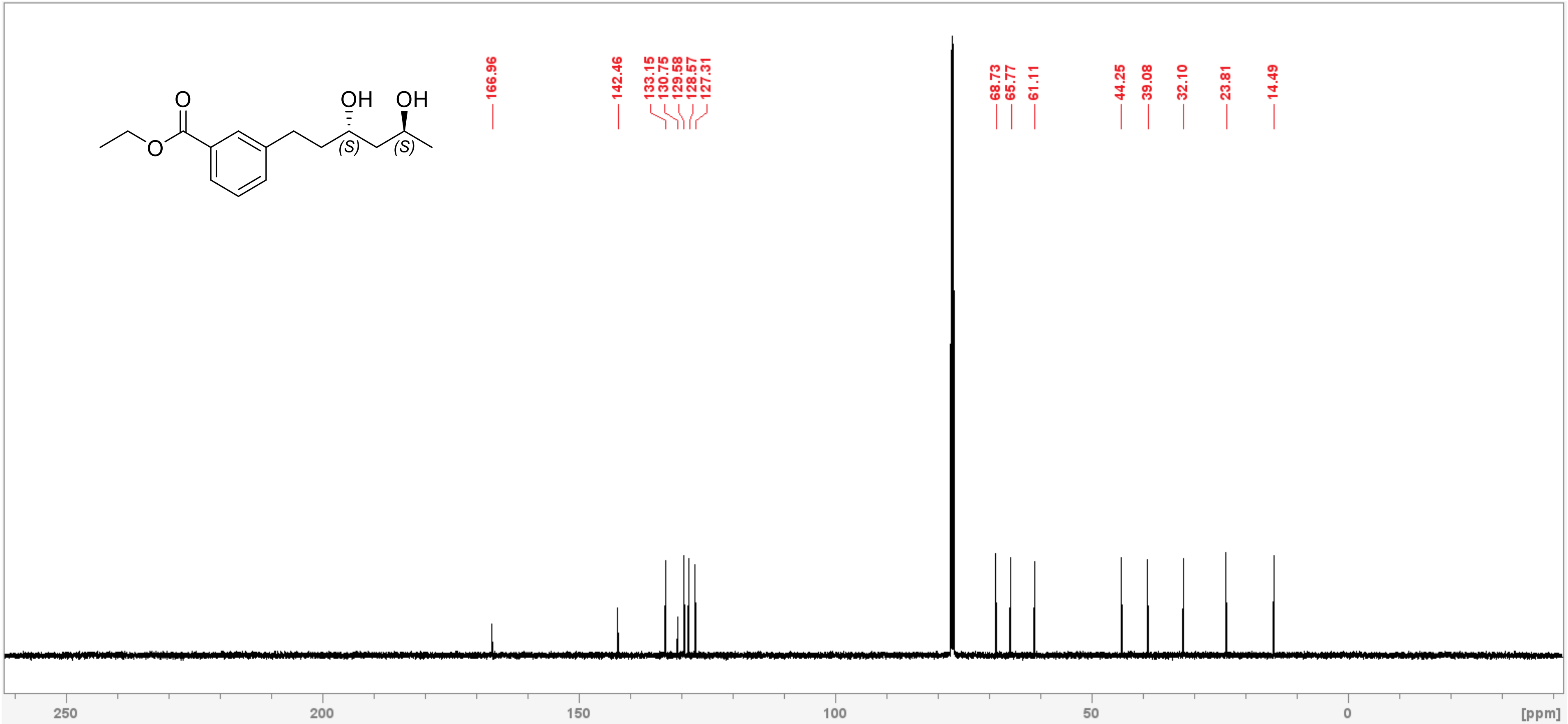

Ethyl 3-((3*R*,5*S*)-3,5-dihydroxyhexyl)benzoate (3*R*,5*S*-3e)

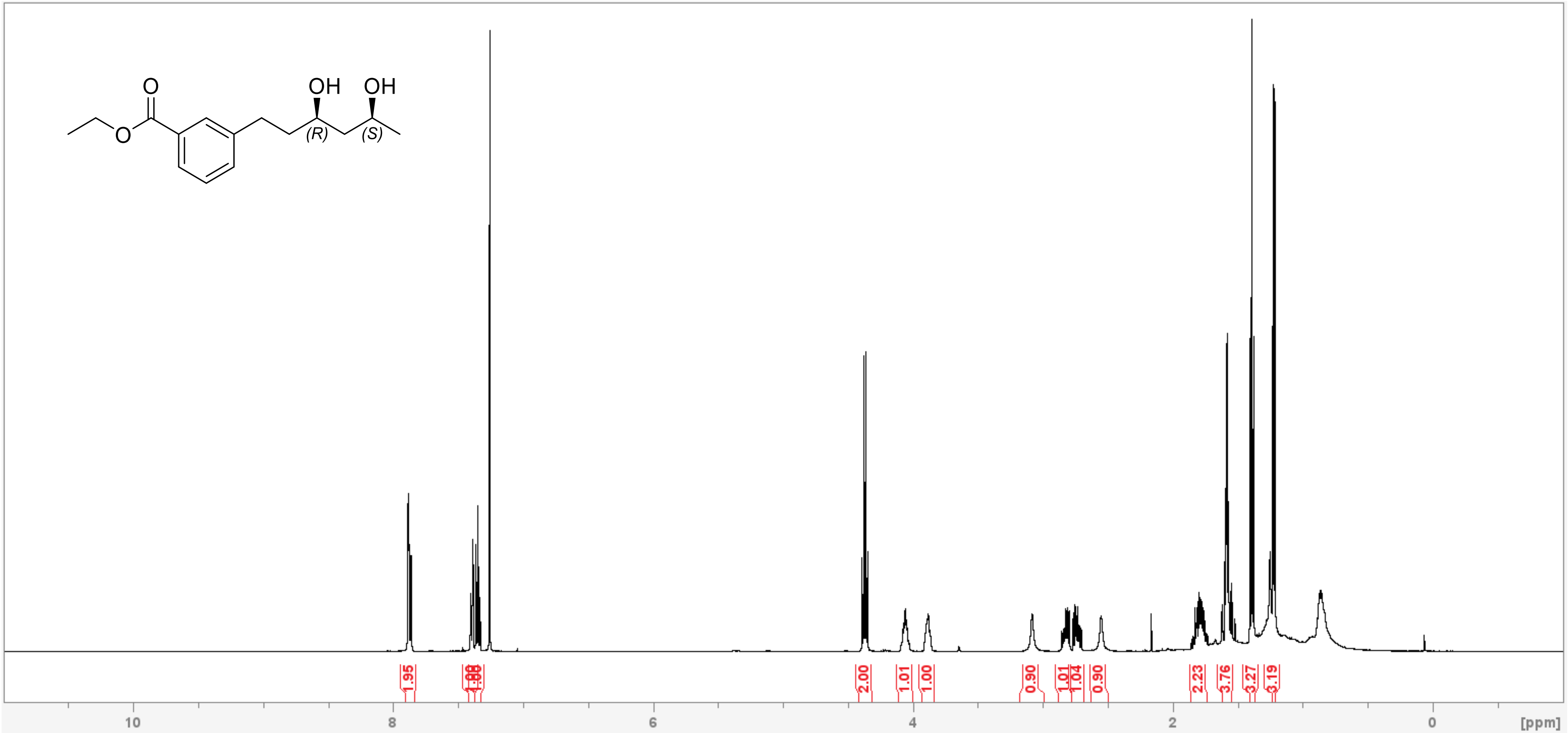

Ethyl 3-((3*R*,5*S*)-3,5-dihydroxyhexyl)benzoate (3*R*,5*S*-3e)

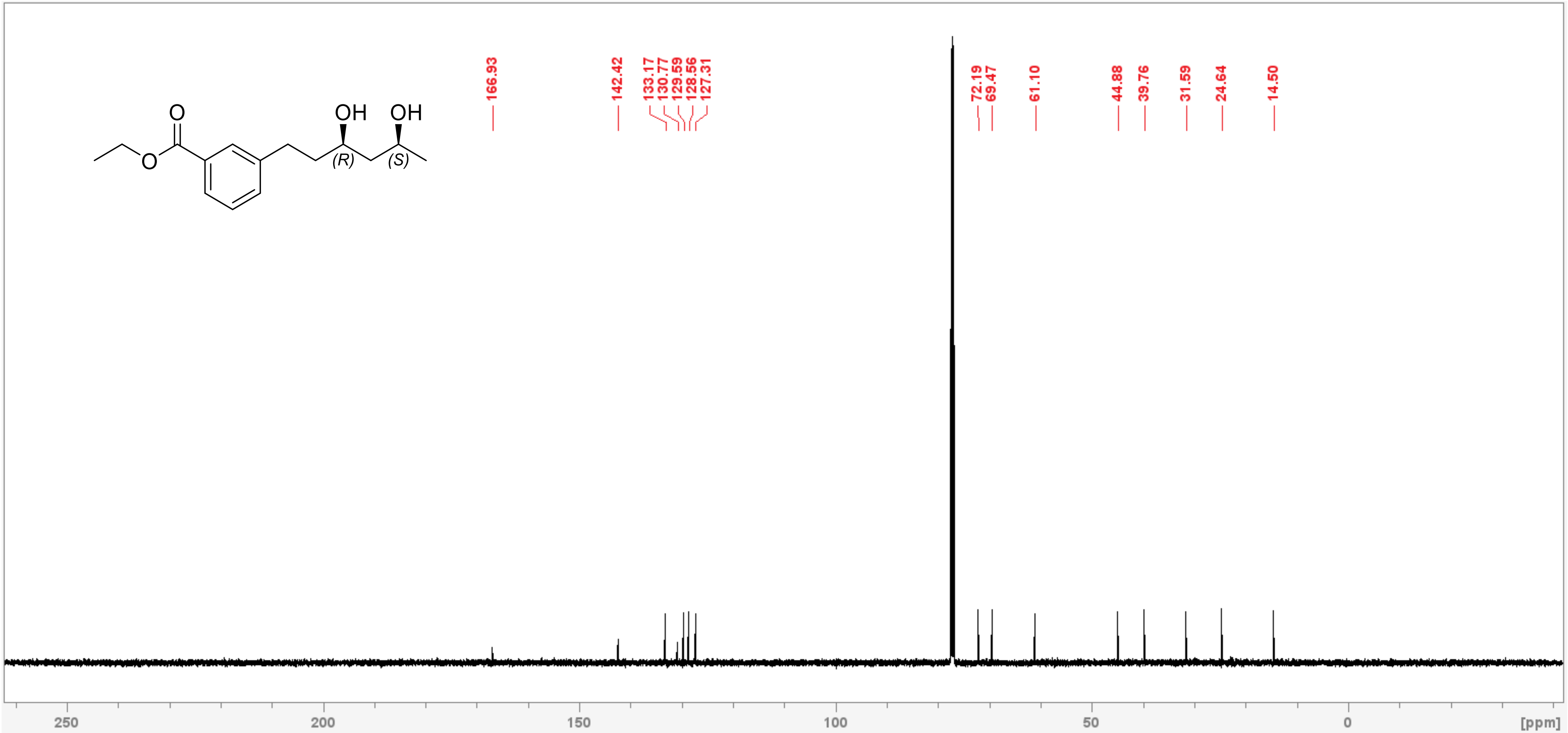

(*S*,*S*)-6-(2-(Trifluoromethyl)phenyl)hexane-2,4-diol (*S*,*S*-3f)

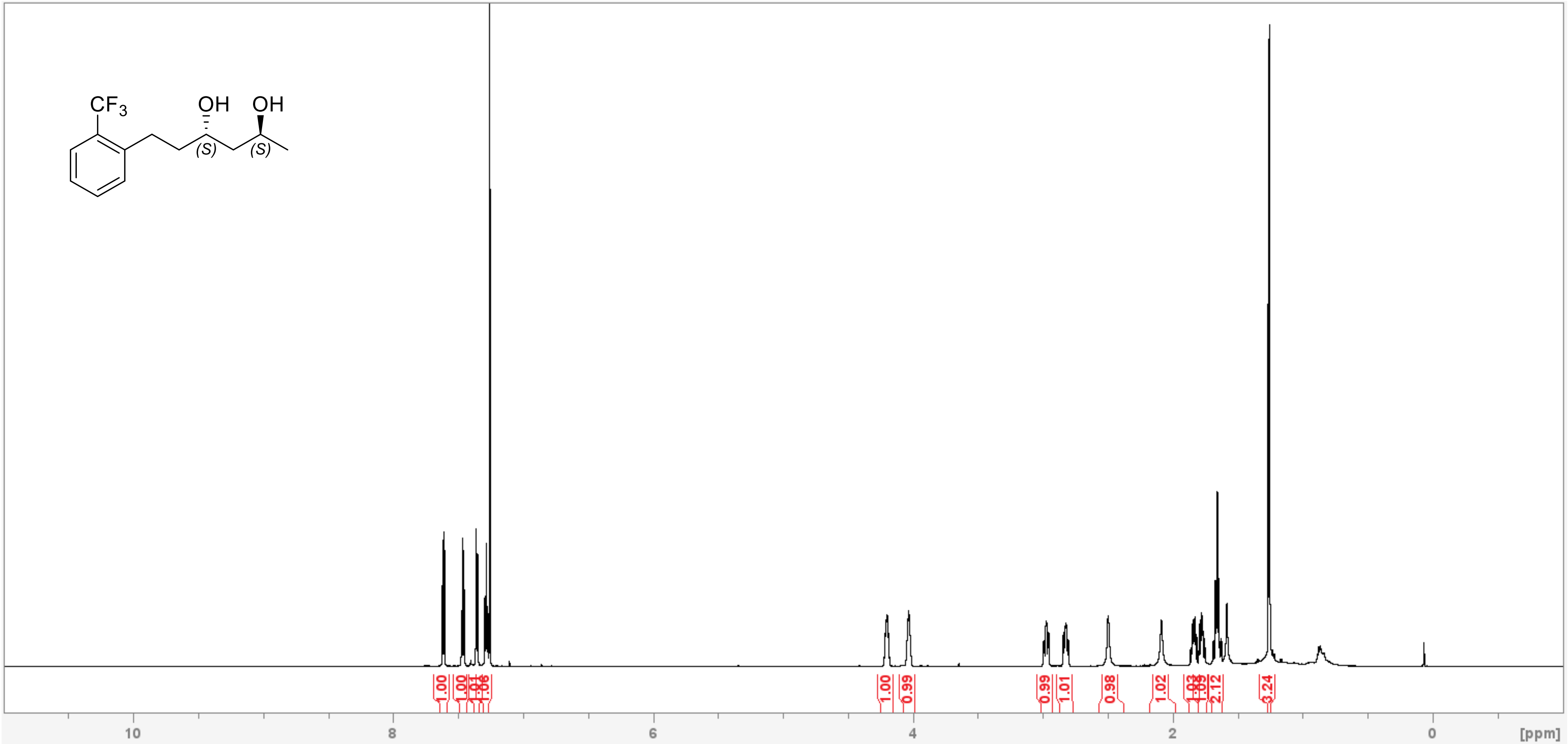

(*S*,*S*)-6-(2-(Trifluoromethyl)phenyl)hexane-2,4-diol (*S*,*S*-3f)

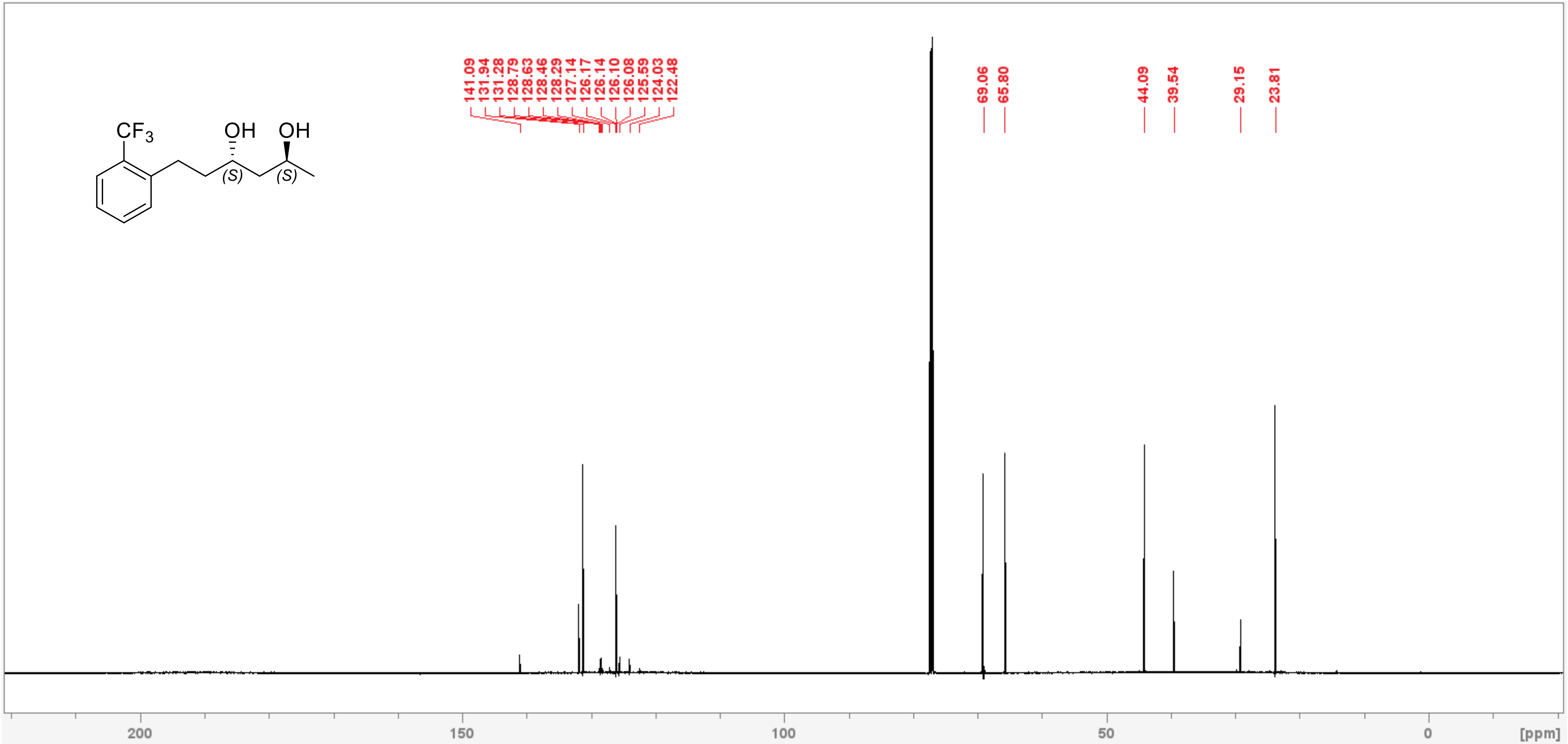

(*S*,*S*)-6-(2-(Trifluoromethyl)phenyl)hexane-2,4-diol (*S*,*S*-3f)

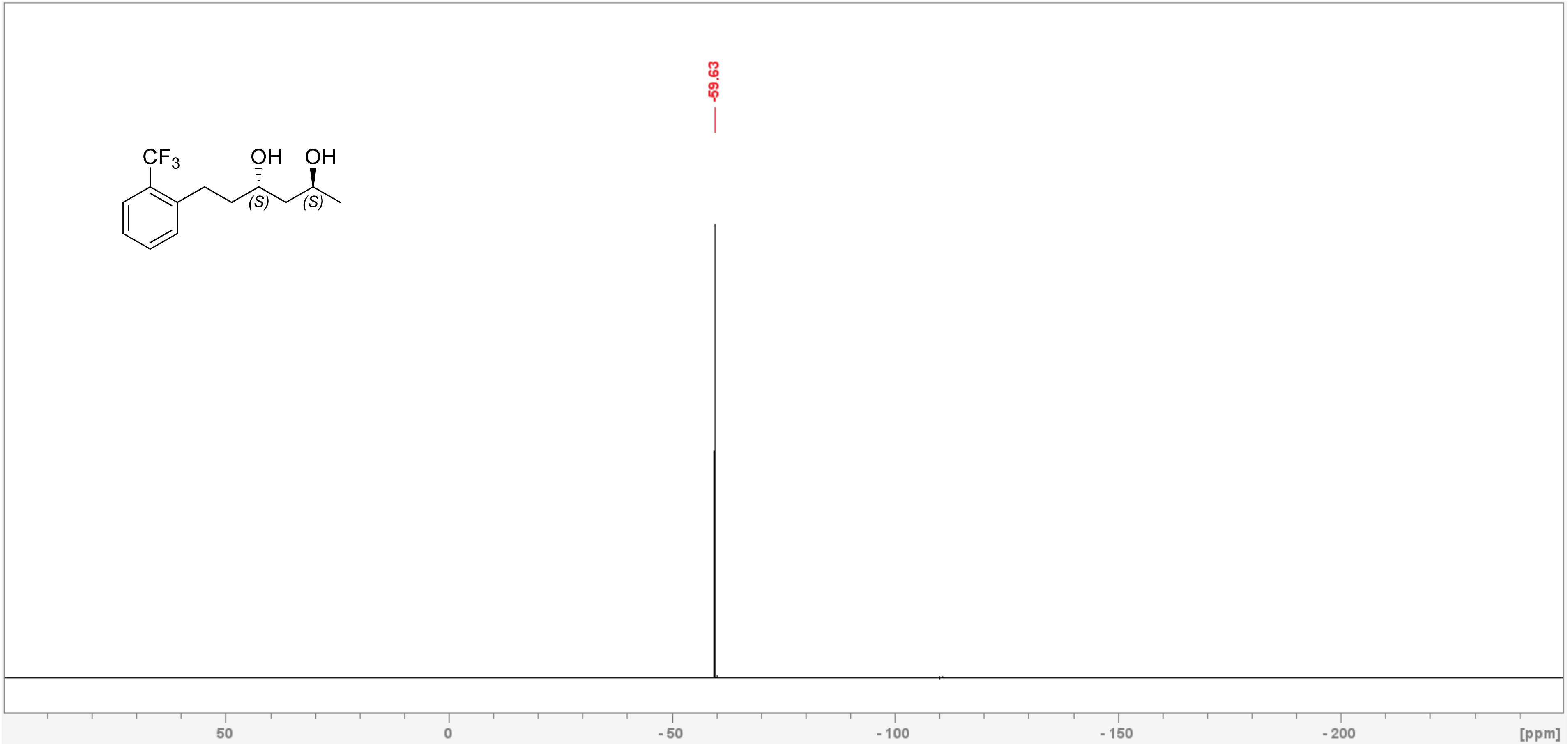

(2*S*,4*R*)-6-(2-(Trifluoromethyl)phenyl)hexane-2,4-diol (2*S*,4*R*-3f)

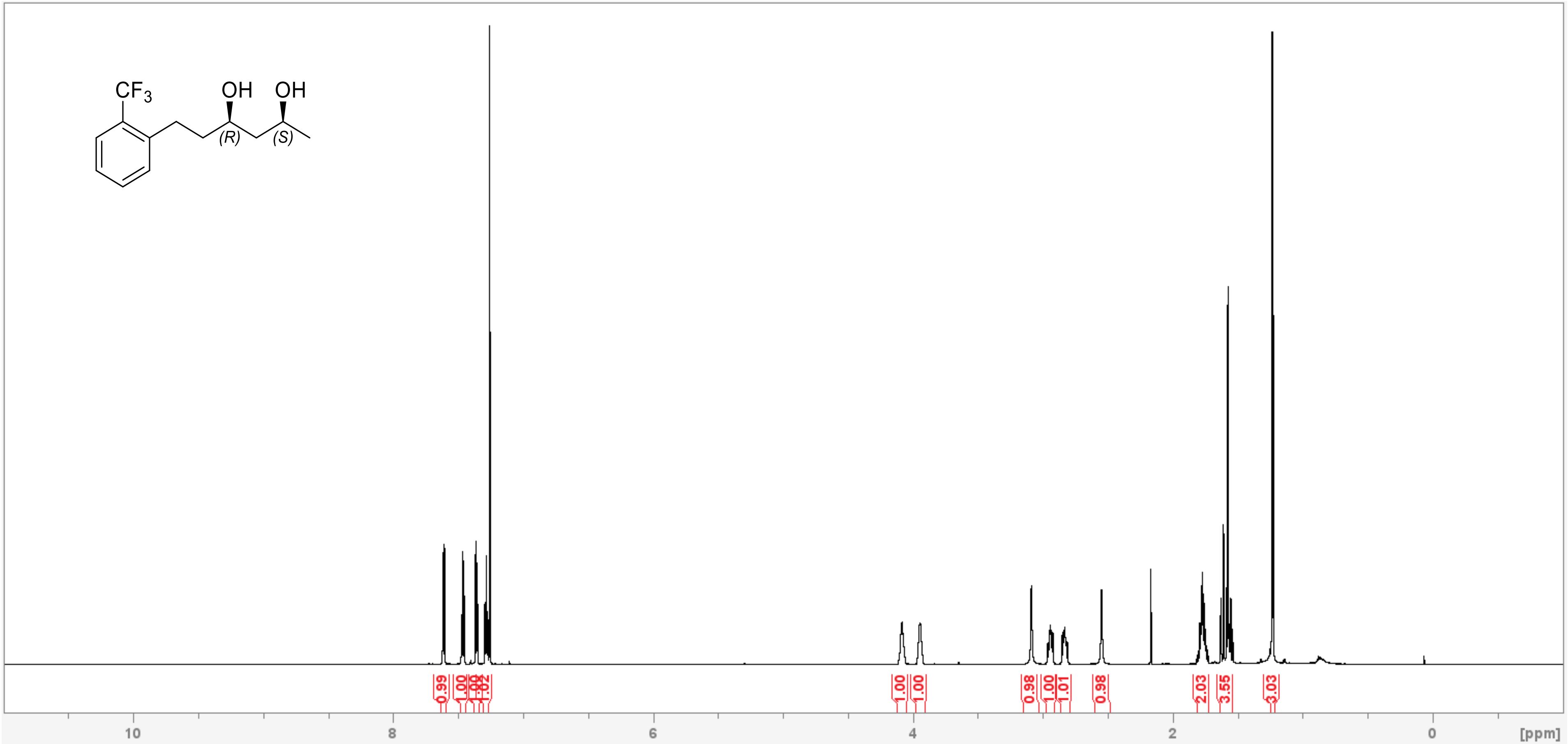

(2*S*,4*R*)-6-(2-(Trifluoromethyl)phenyl)hexane-2,4-diol (2*S*,4*R*-3f)

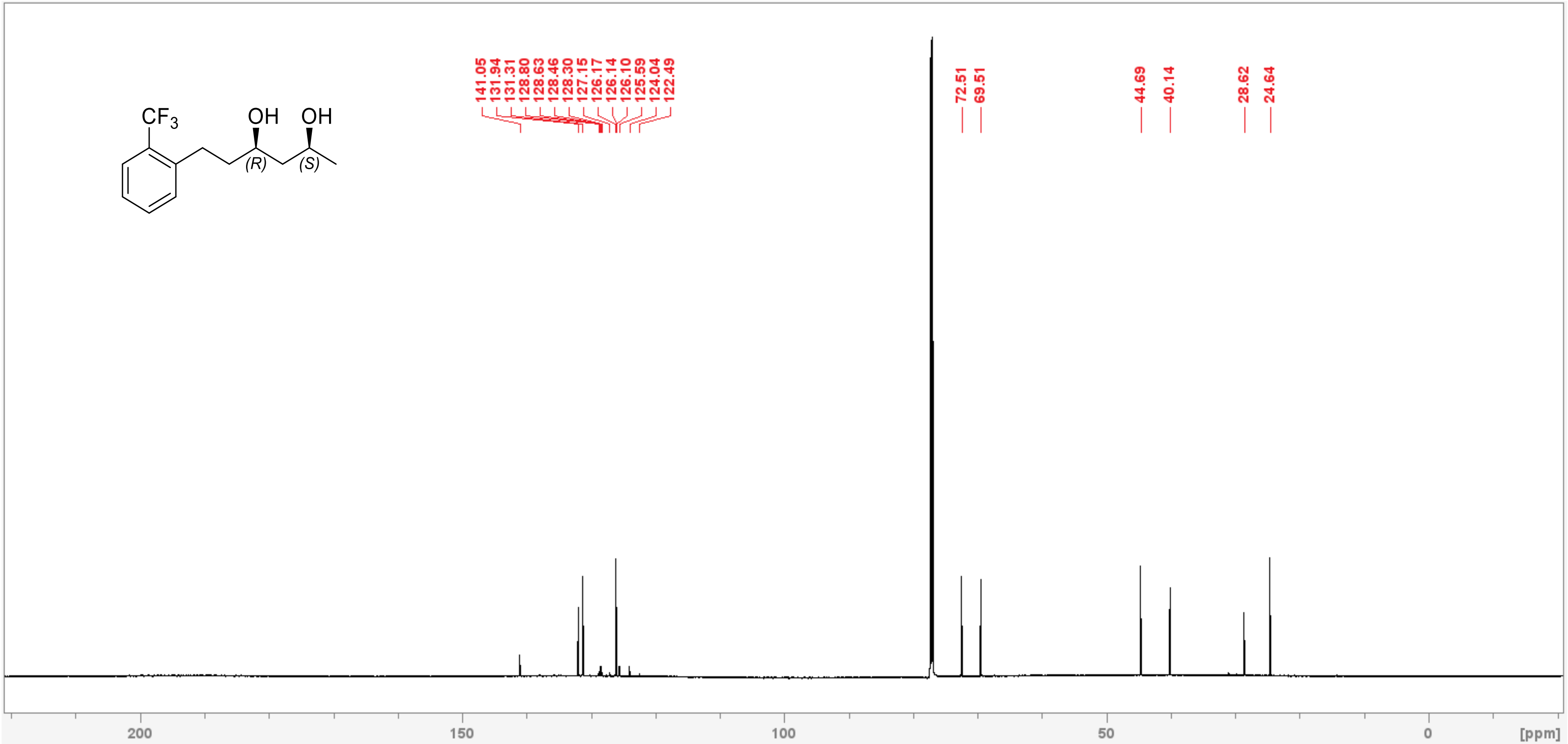

(2*S*,4*R*)-6-(2-(Trifluoromethyl)phenyl)hexane-2,4-diol (2*S*,4*R*-3f)

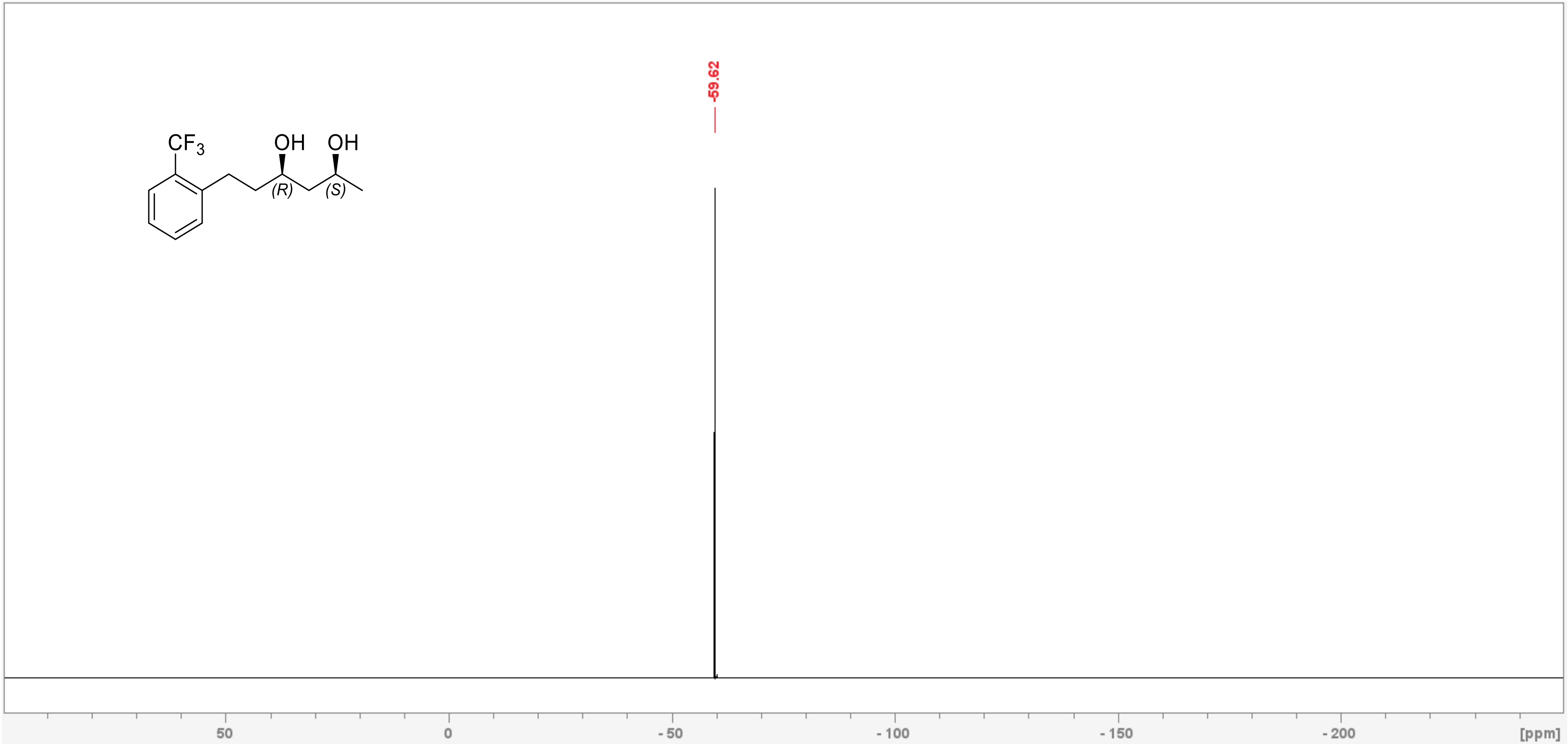

4-((*S*,*S*)-3,5-Dihydroxyhexyl)benzonitrile (*S*,*S*-3g)

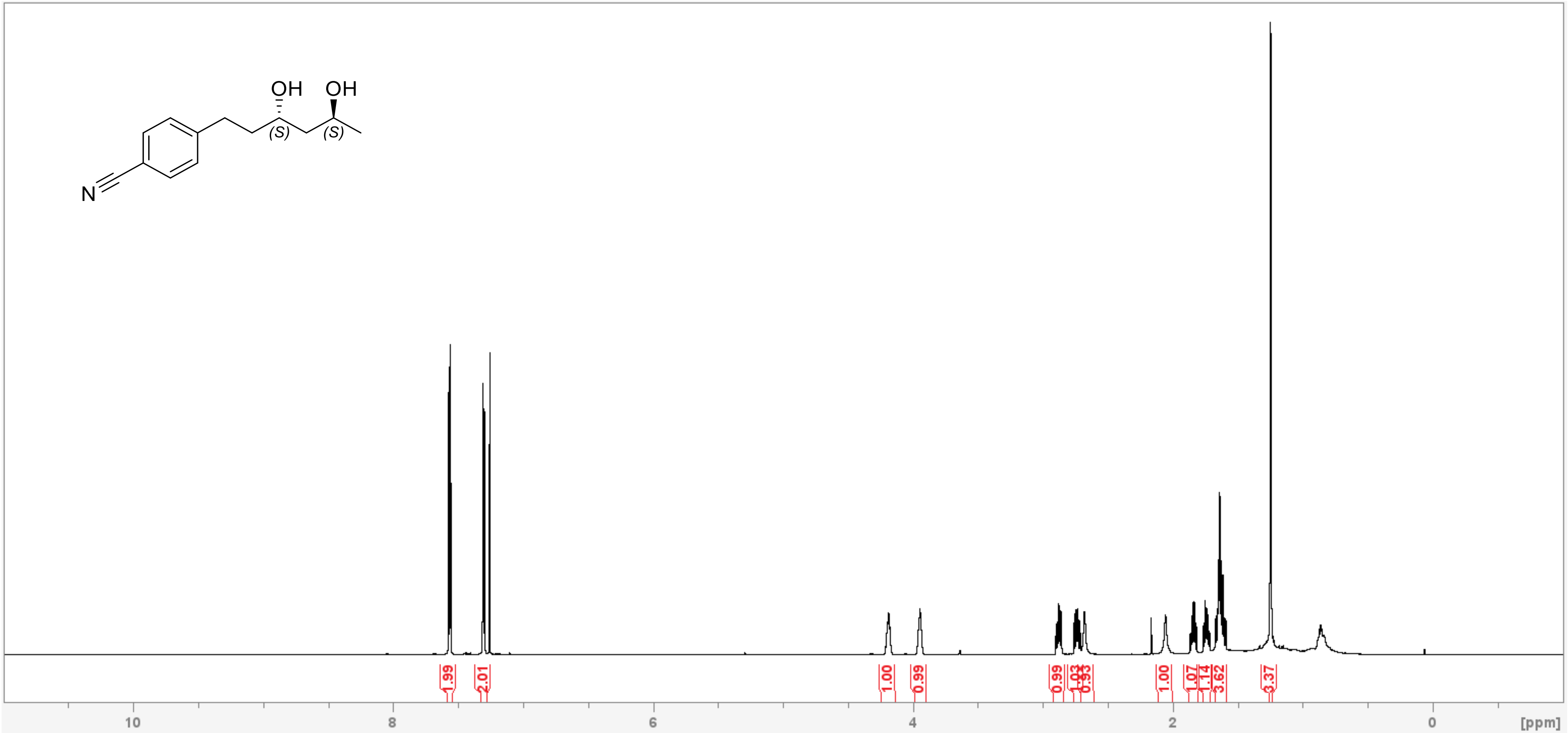

4-((*S*,*S*)-3,5-Dihydroxyhexyl)benzonitrile (*S*,*S*-3g)

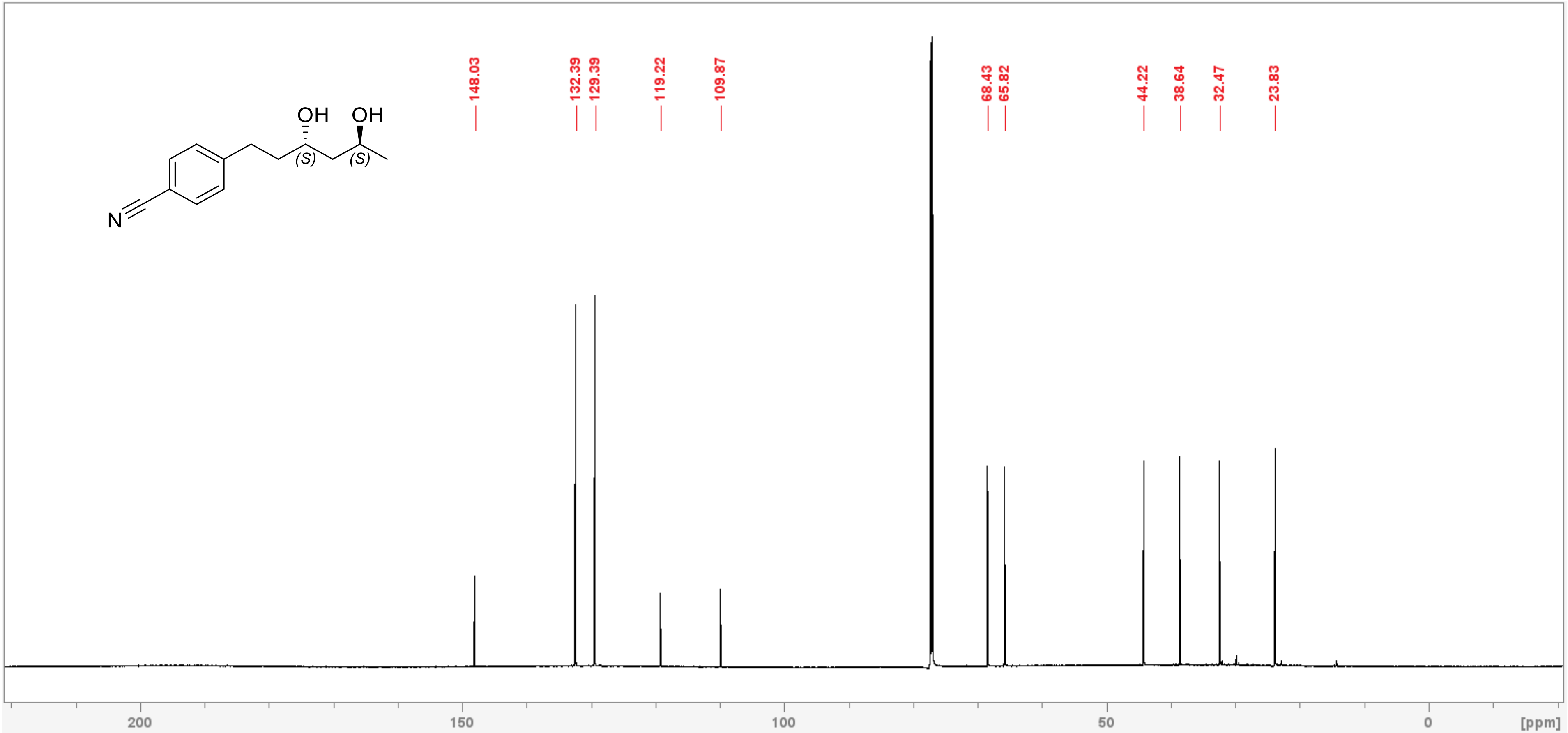

4-((3*R*,5*S*)-3,5-Dihydroxyhexyl)benzonitrile (3*R*,5*S*-3g)

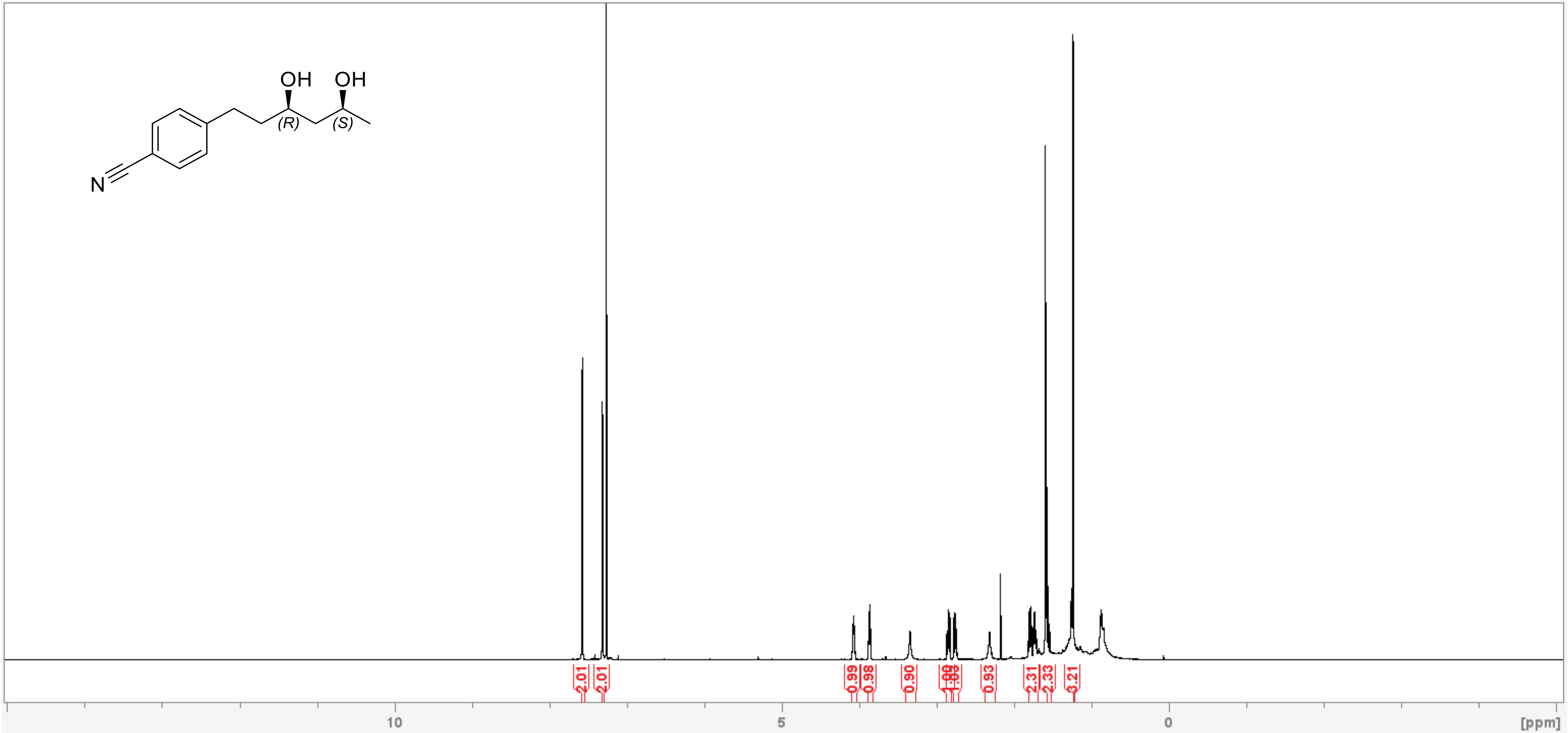

4-((3*R*,5*S*)-3,5-Dihydroxyhexyl)benzonitrile (3*R*,5*S*-3g)

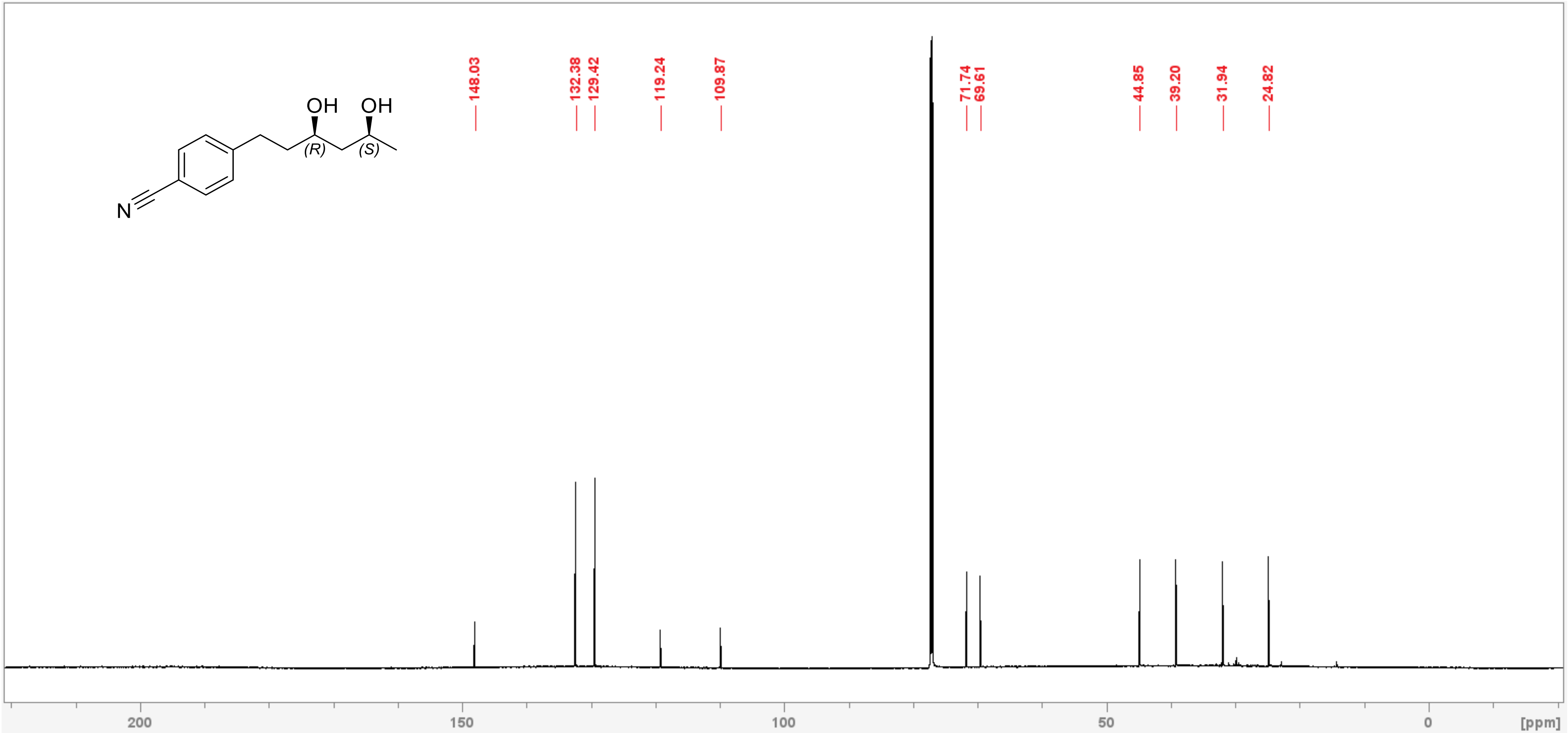

(*R*,*S*)-*N*-(4-(3,5-dihydroxyhexyl)phenyl)-2,2,2-trifluoroacetamide (*syn*-3h)

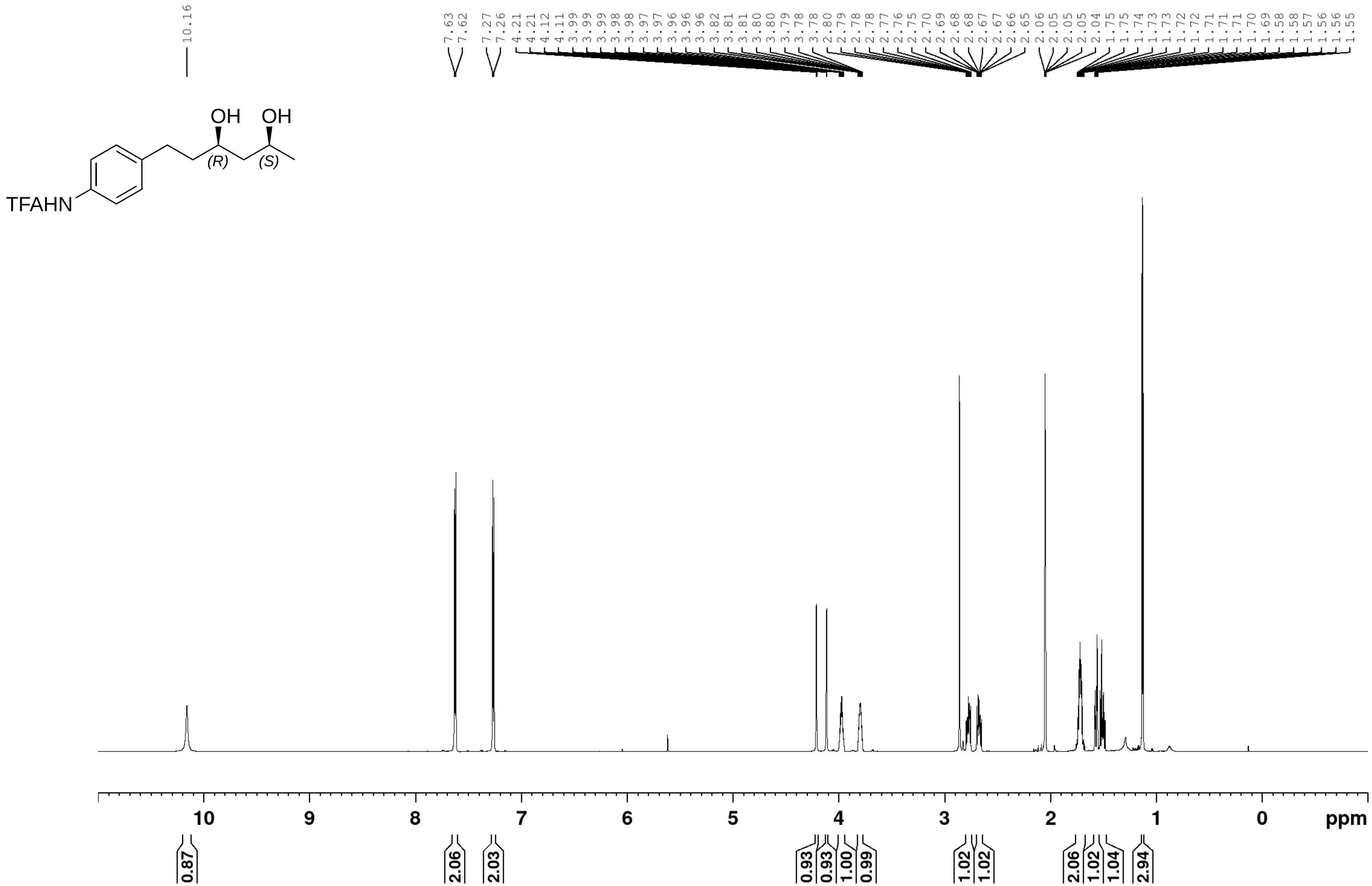

(*R*,*S*)-*N*-(4-(3,5-dihydroxyhexyl)phenyl)-2,2,2-trifluoroacetamide (*syn*-3h)

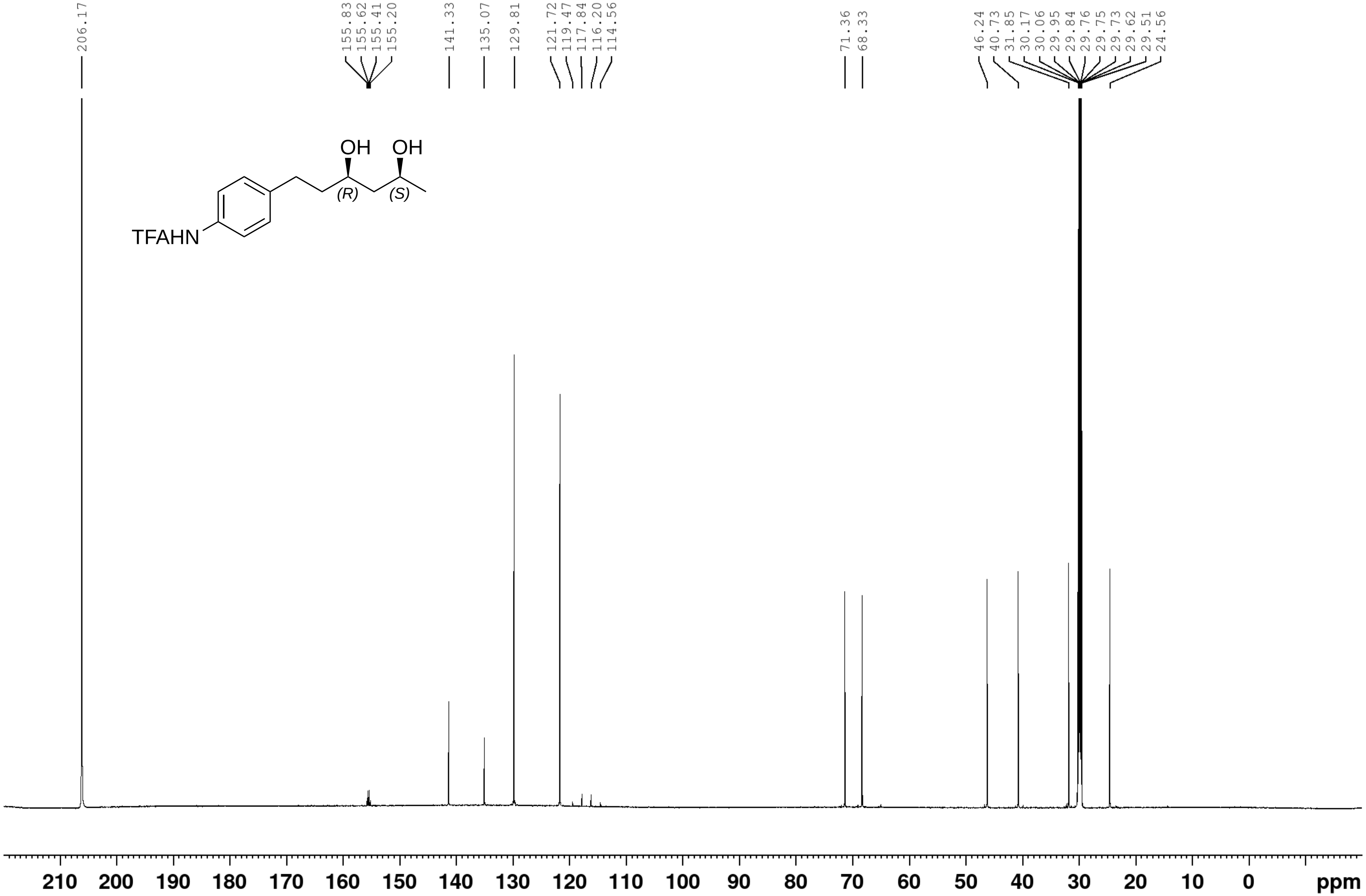

(*S*,*S*)-*N*-(4-(3,5-dihydroxyhexyl)phenyl)-2,2,2-trifluoroacetamide (*anti*-3h)

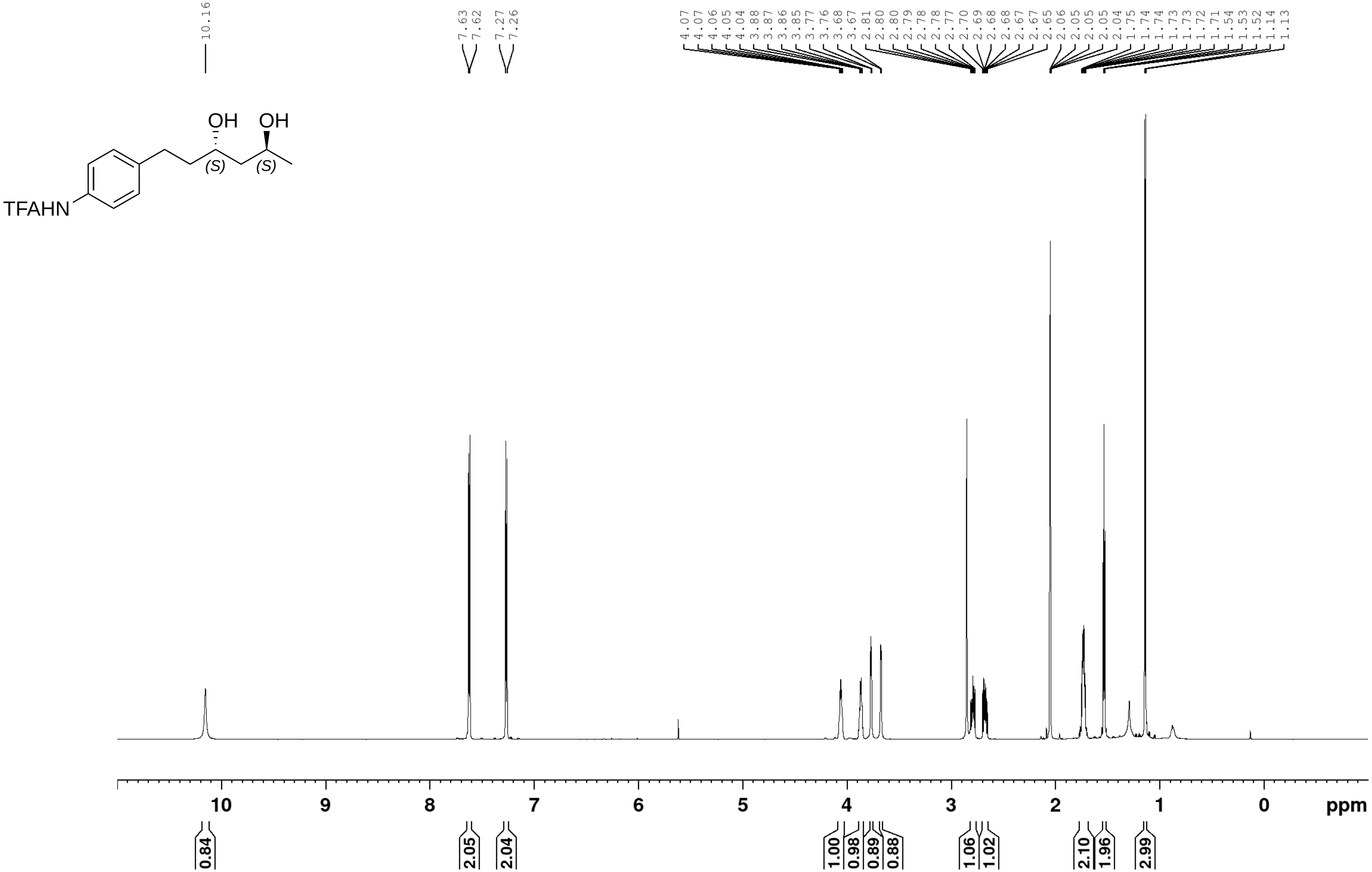

(*S*,*S*)-*N*-(4-(3,5-dihydroxyhexyl)phenyl)-2,2,2-trifluoroacetamide (*anti*-3h)

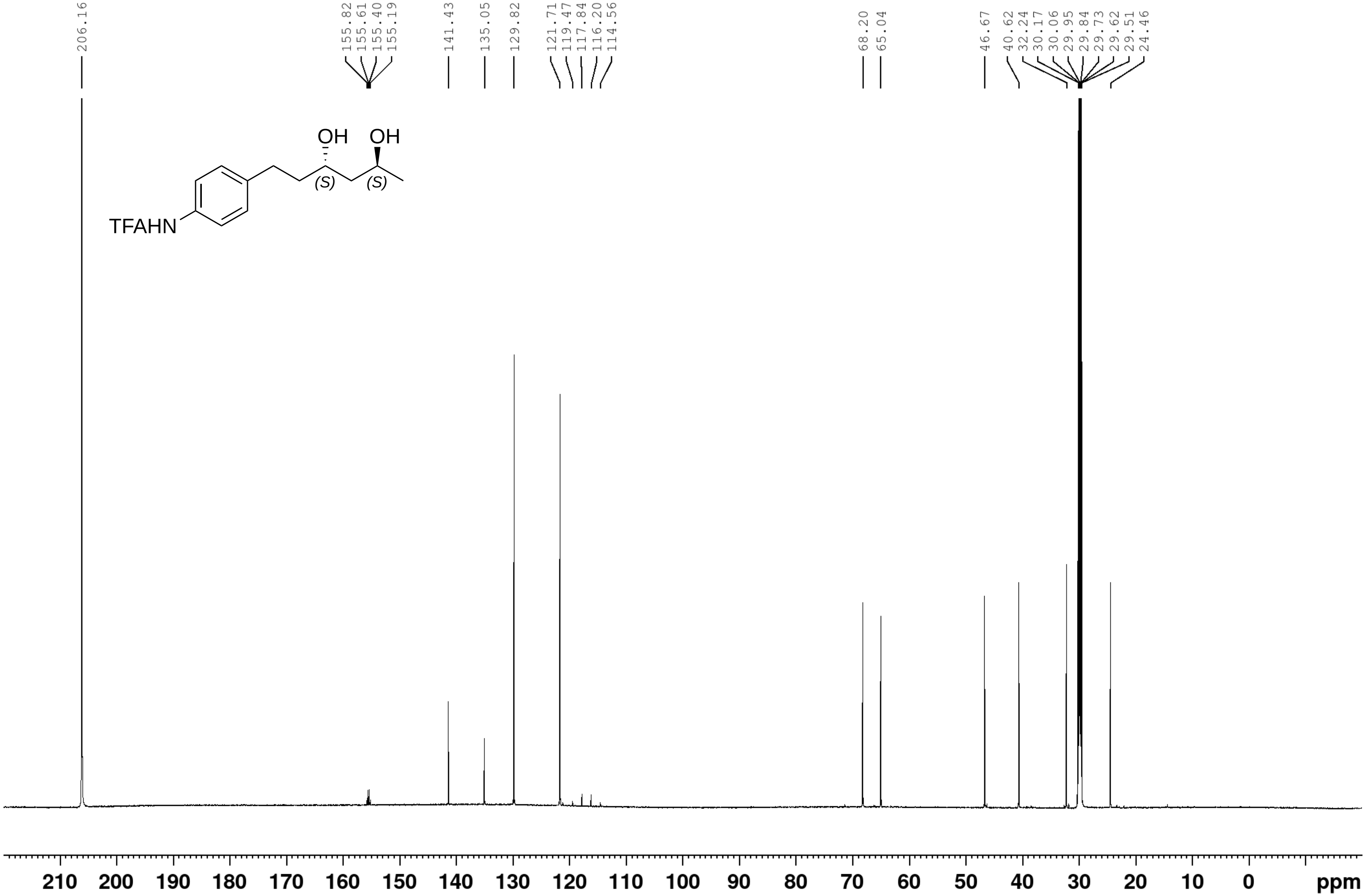

(3*S*,5*R*)-6-Methyl-1-phenylheptane-3,5-diol (*S*,*R*-3i)

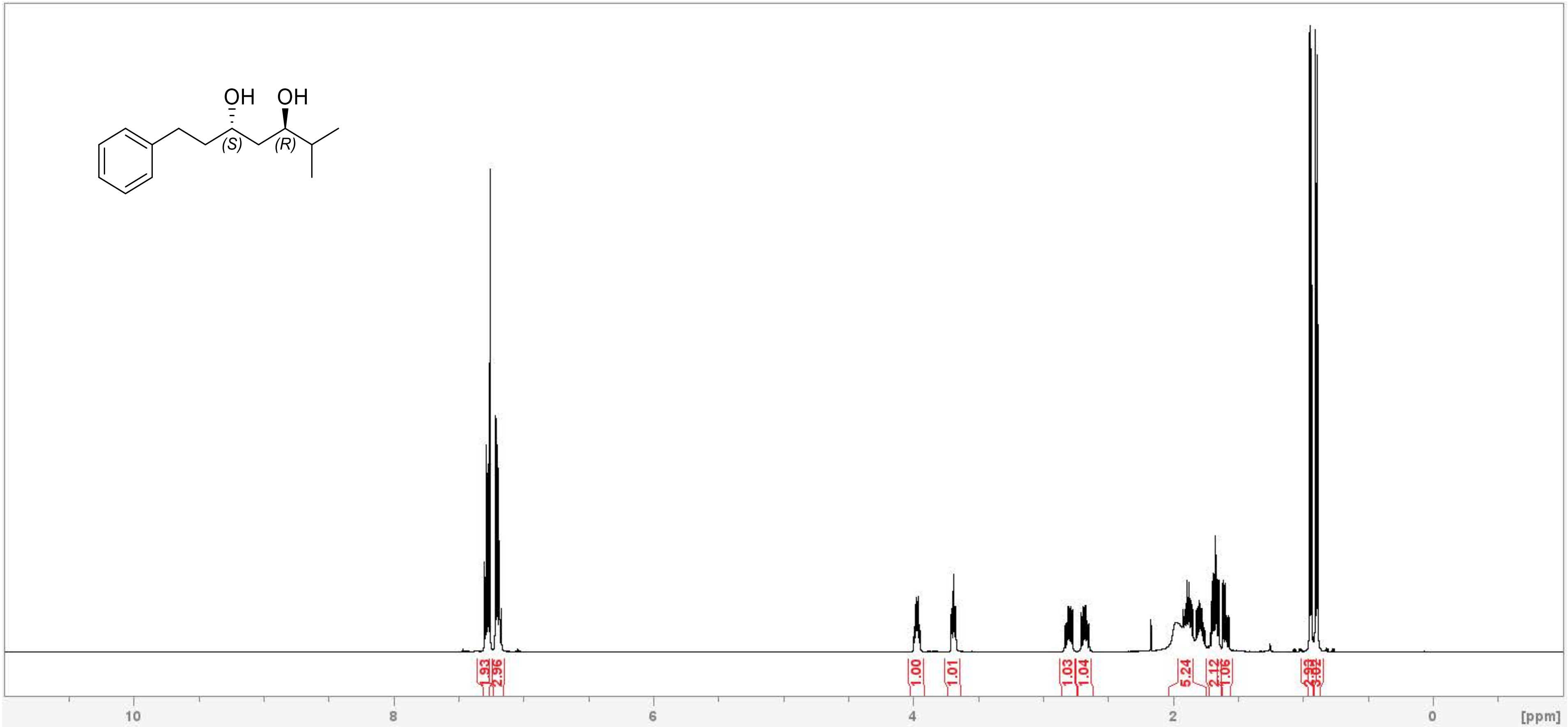

(3*S*,5*R*)-6-Methyl-1-phenylheptane-3,5-diol (*S*,*R*-3i)

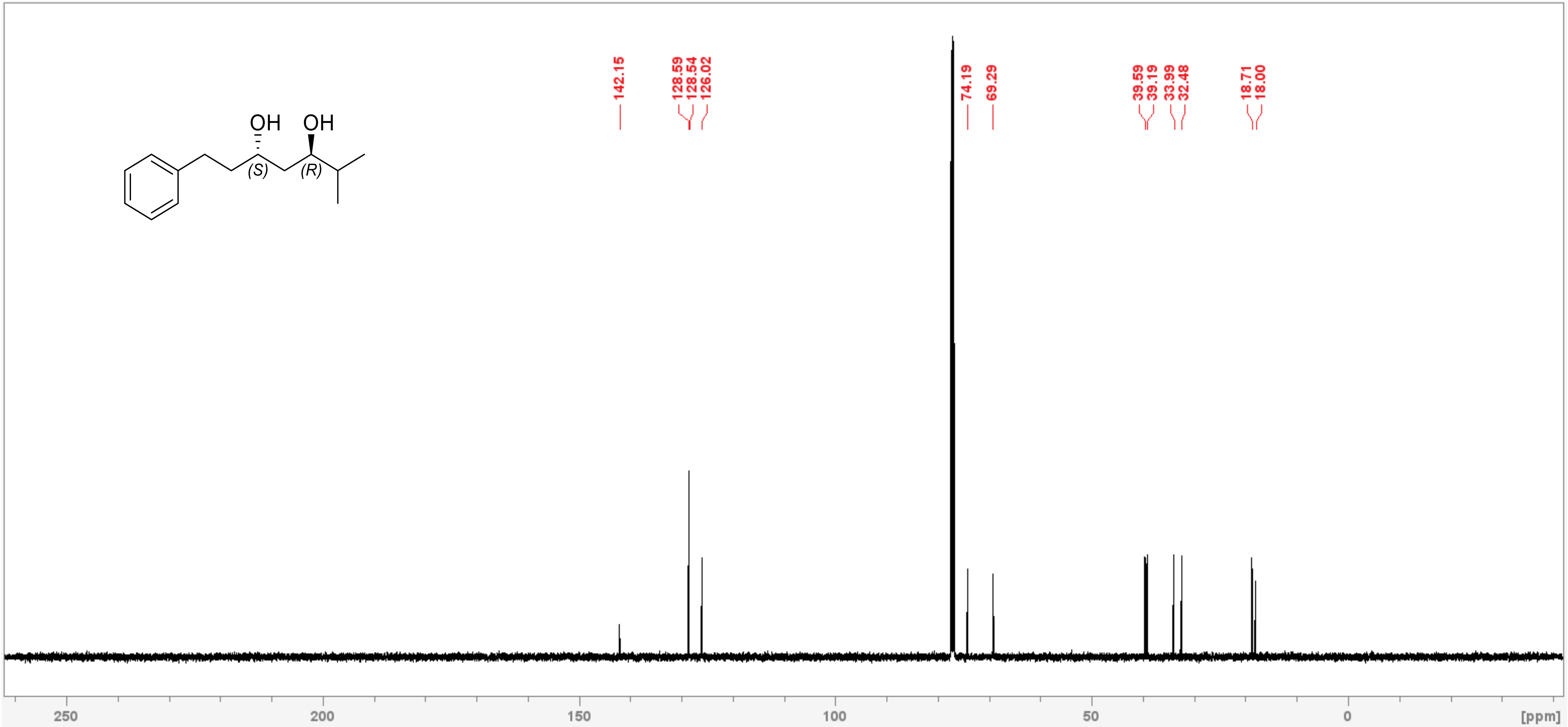

Enriched (*syn*)-6-Methyl-1-phenylheptane-3,5-diol (*enriched-syn*-3i)

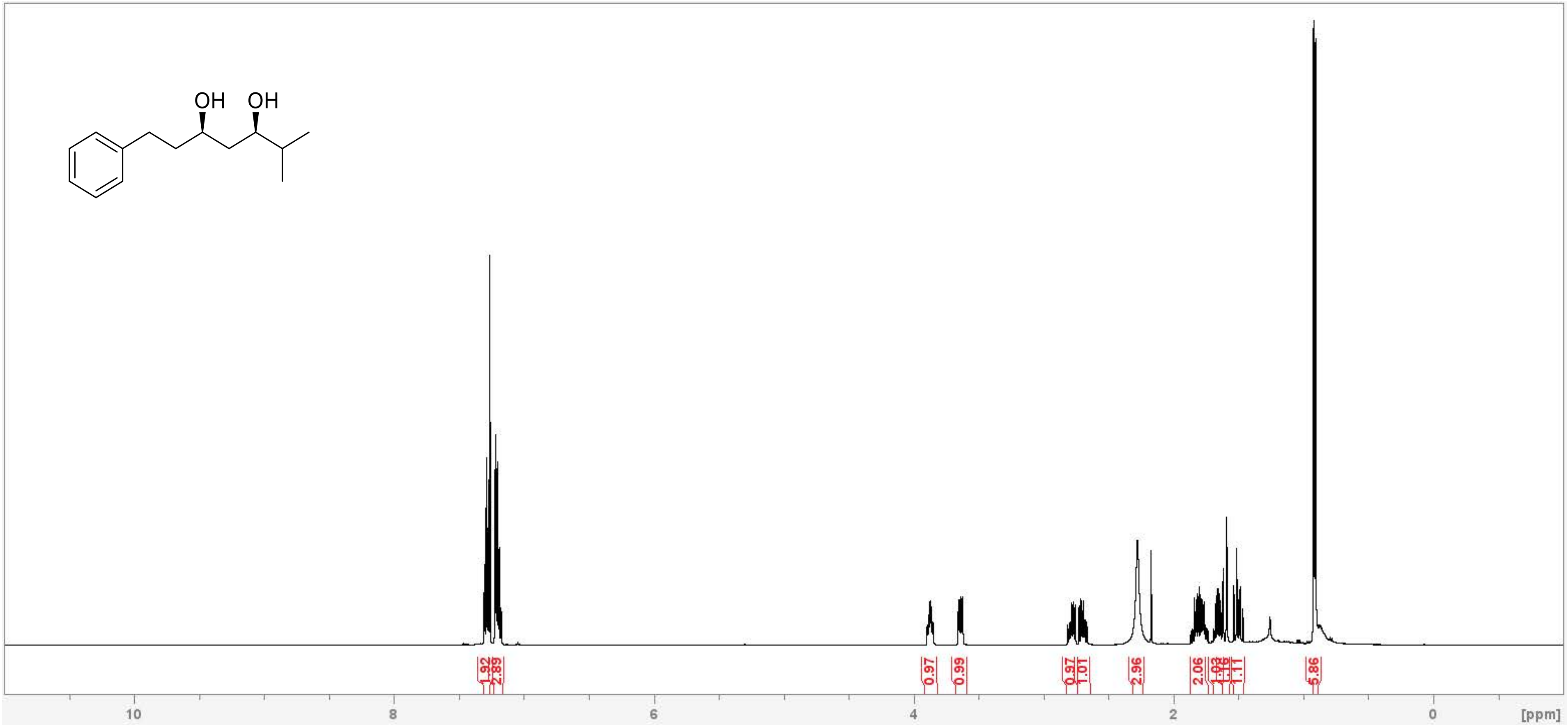

Enriched (*syn*)-6-Methyl-1-phenylheptane-3,5-diol (*enriched-syn*-3i)

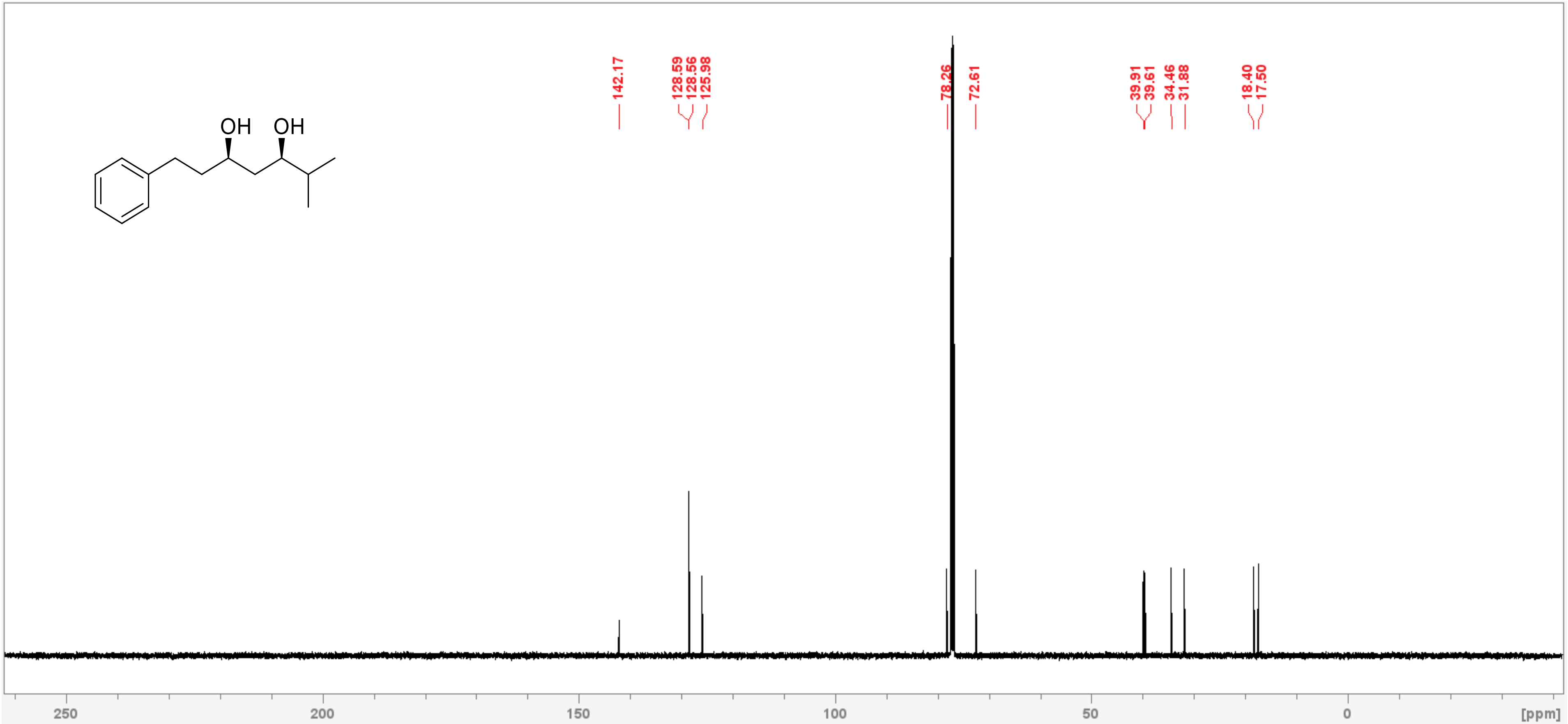

tert-butyl 4-((2_S_,4_R_)-2,4-dihydroxy-6-phenylhexyl)piperidine-1-carboxylate (_syn_-3j) and _tert_-butyl 4-((2_S_,4_S_)-2,4-dihydroxy-6-phenylhexyl)piperidine-1-carboxylate (_anti_-3j)

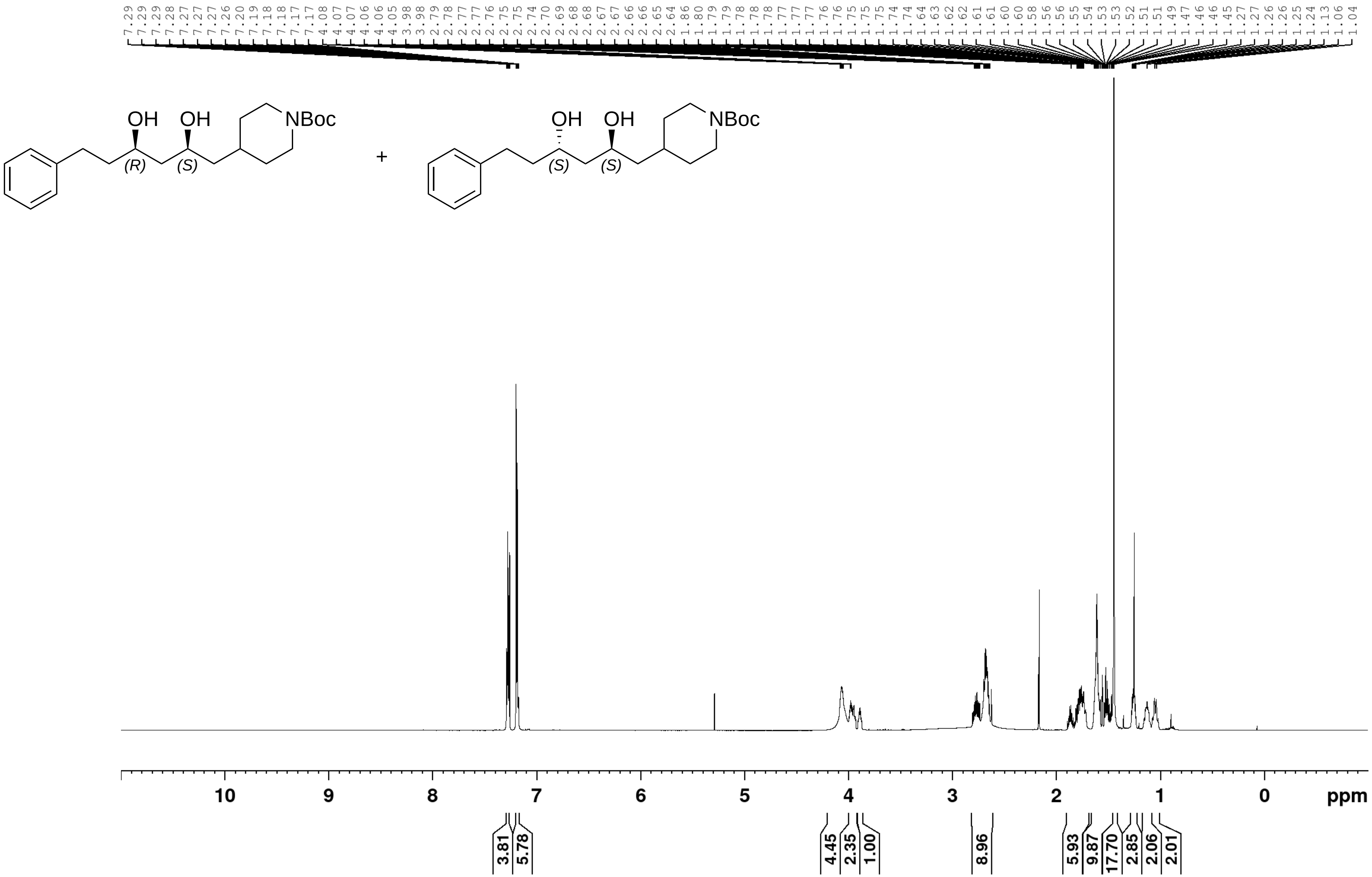

***tert*-butyl 4-((2*S*,4*R*)-2,4-dihydroxy-6-phenylhexyl)piperidine-1-carboxylate (*syn*-3j) and *tert*-butyl 4-((2*S*,4*S*)-2,4-dihydroxy-6-phenylhexyl)piperidine-1-carboxylate (*anti*-3j)**

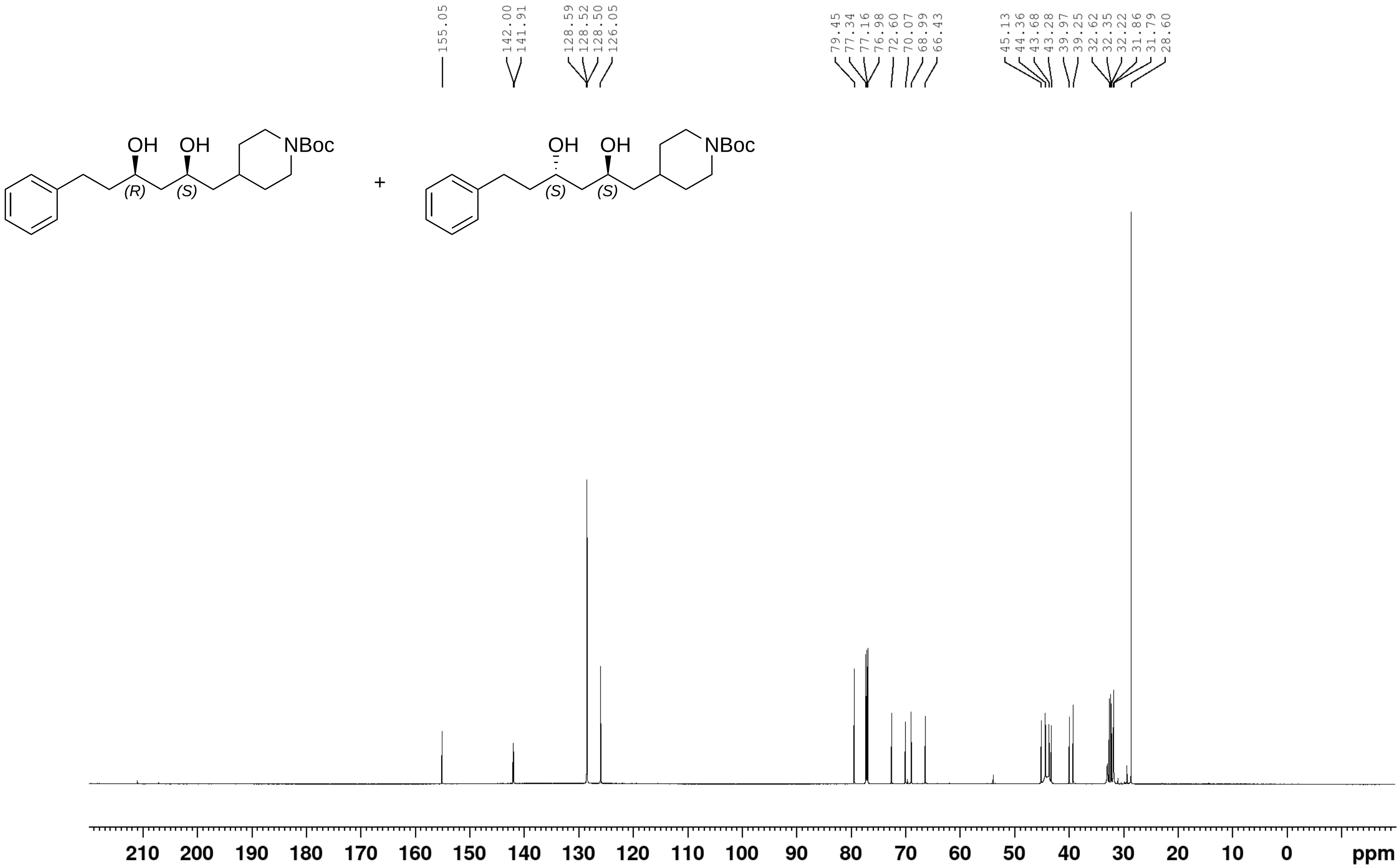

(*S*,*S*)-1-Phenylnonane-3,5-diol (*S*,*S*-3k)

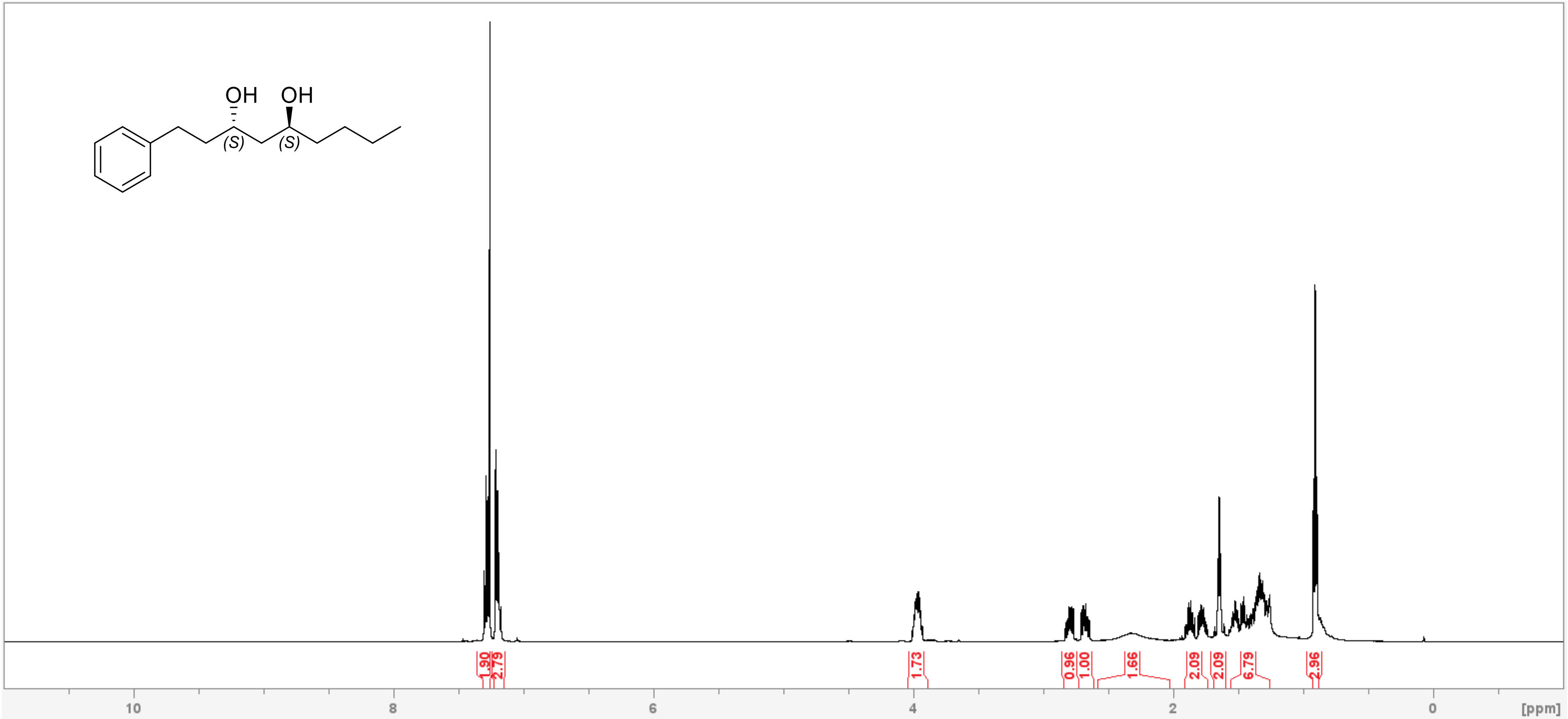

(*S*,*S*)-1-Phenylnonane-3,5-diol (*S*,*S*-3k)

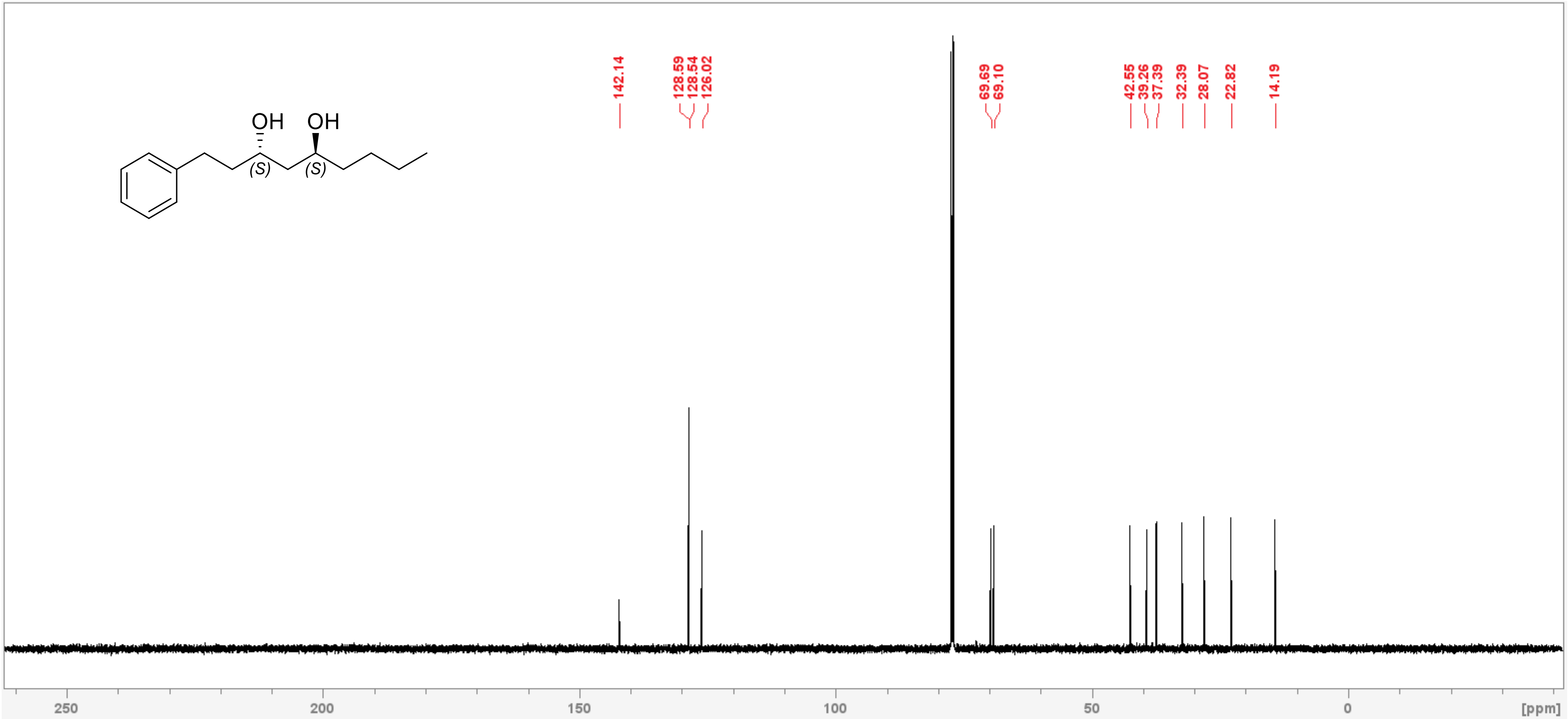

Enriched (*syn*)-1-Phenylnonane-3,5-diol (*enriched-syn*-3k)

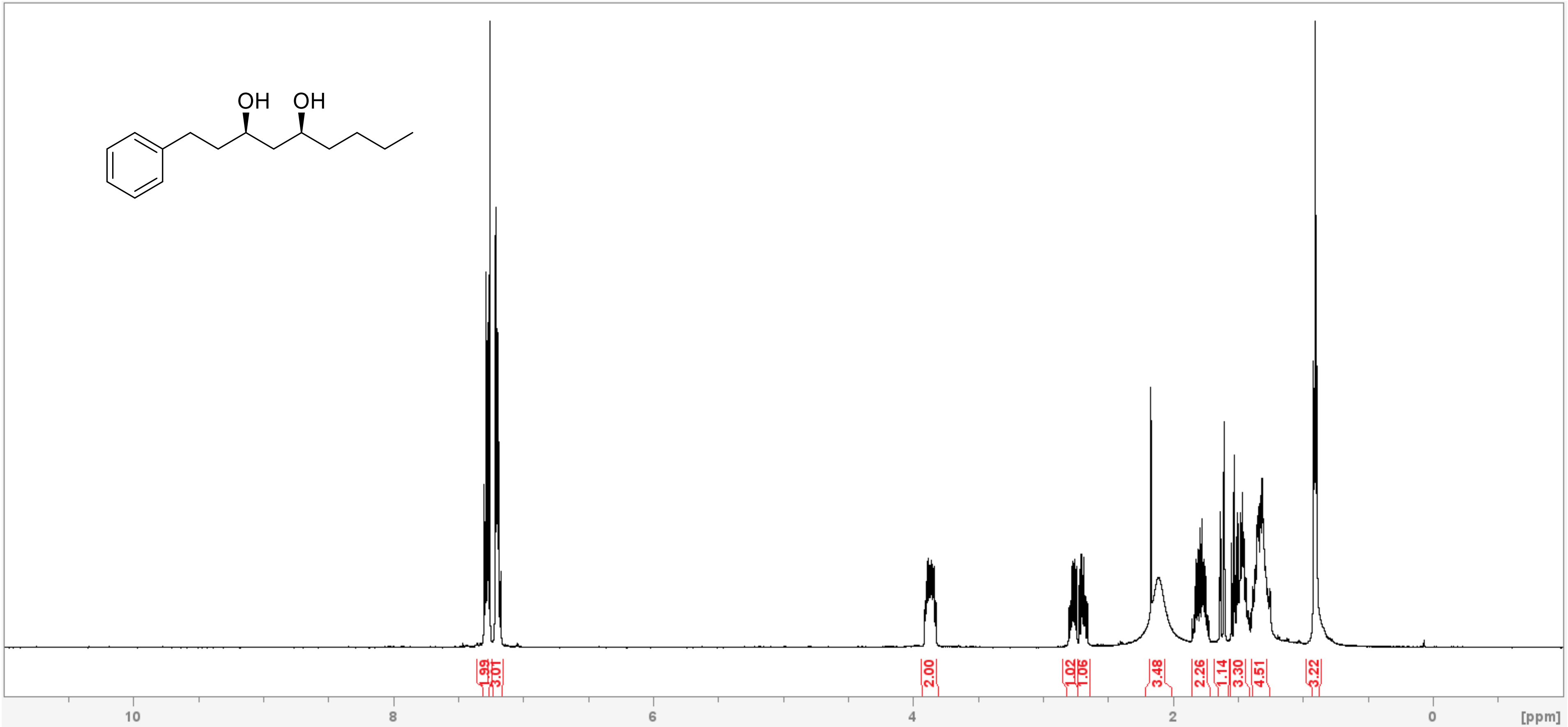

Enriched (*syn*)-1-Phenylnonane-3,5-diol (*enriched-syn*-3k)

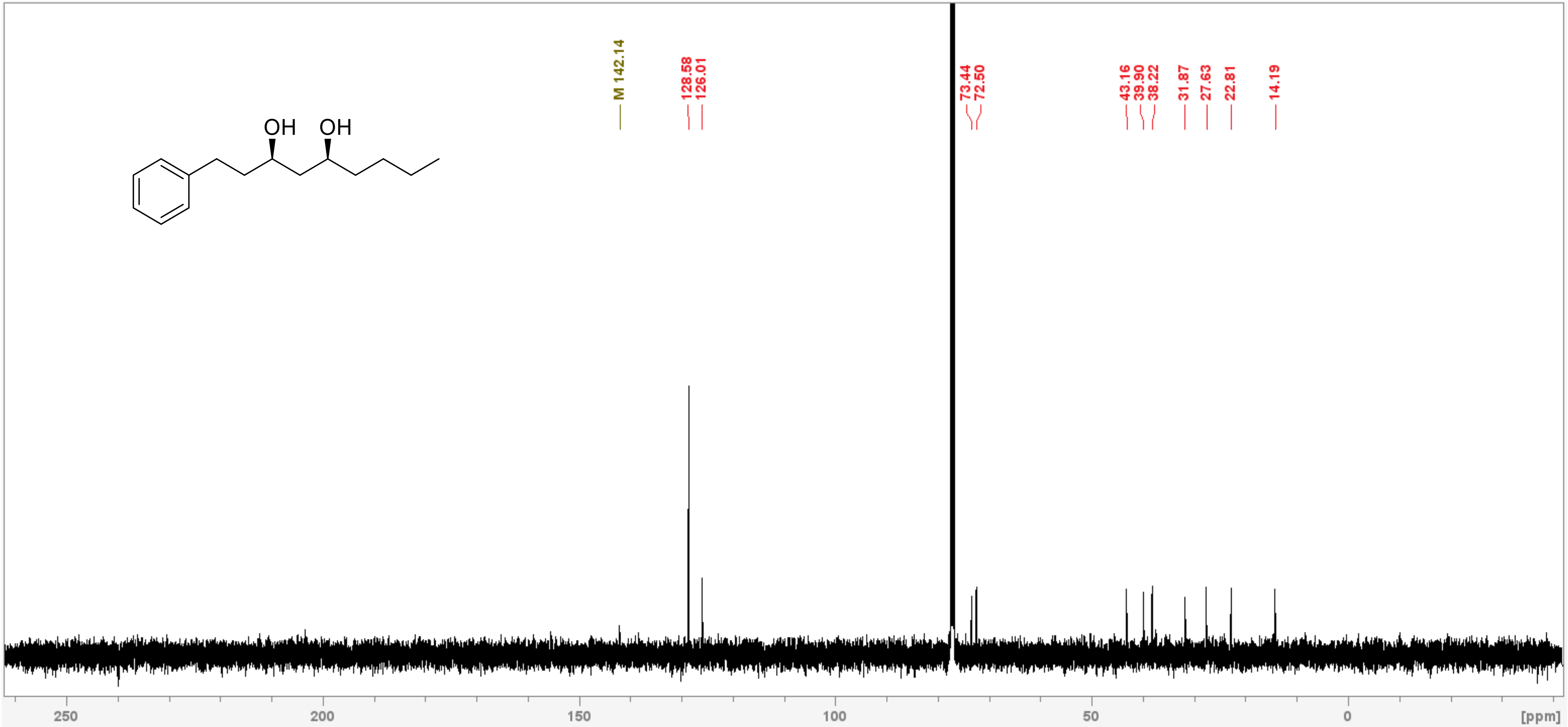

(3*R*,5*S*)-1-phenyl-7-((2,3,3-trimethylbutan-2-yl)oxy)heptane-3,5-diol (*syn*-3l) and (3*S*,5*S*)-1-phenyl-7-((2,3,3-trimethylbutan-2-yl)oxy)heptane-3,5-diol (*anti*-3l)

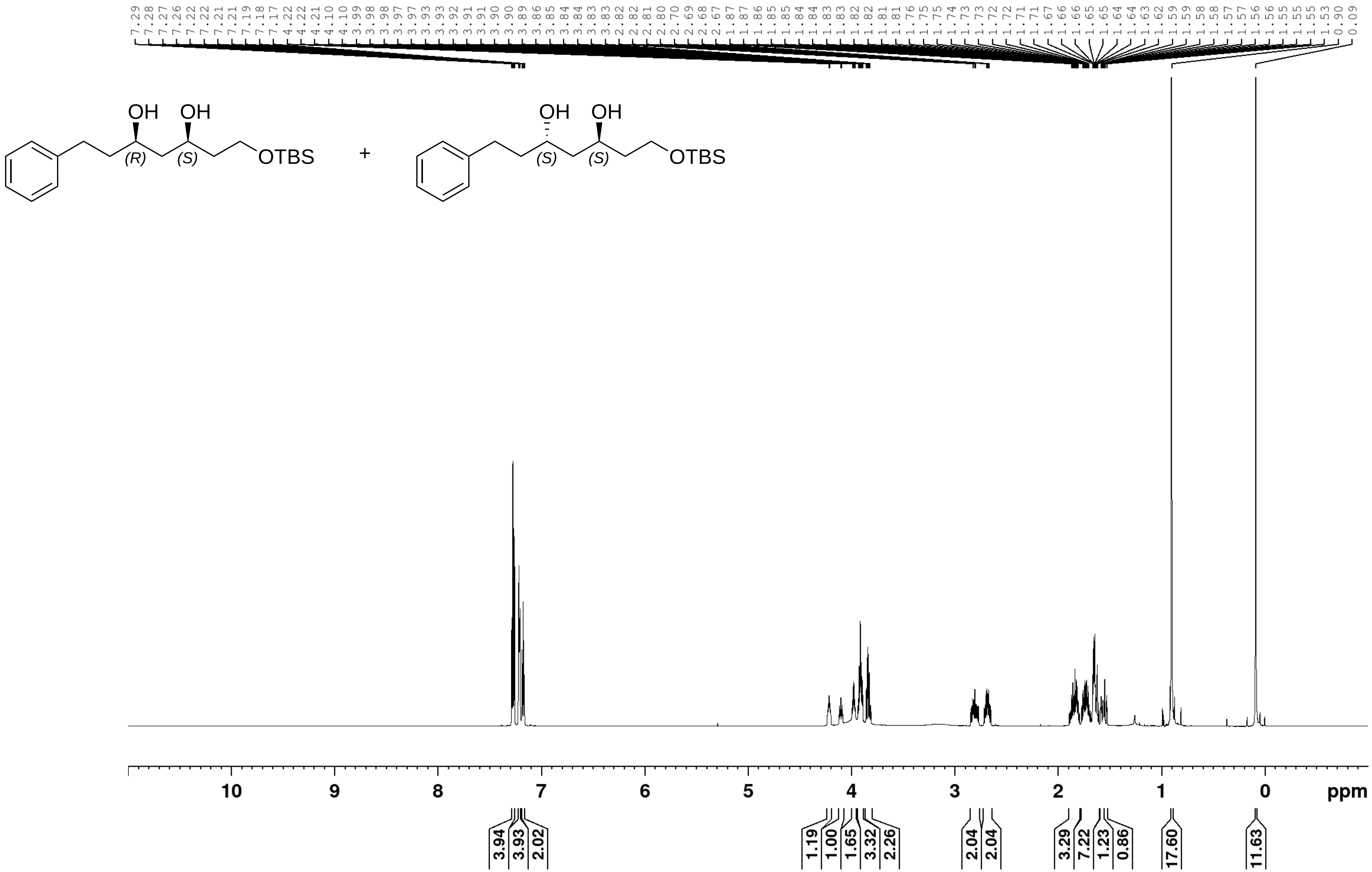

(3*R*,5*S*)-1-phenyl-7-((2,3,3-trimethylbutan-2-yl)oxy)heptane-3,5-diol (*syn*-3l) and (3*S*,5*S*)-1-phenyl-7-((2,3,3-trimethylbutan-2-yl)oxy)heptane-3,5-diol (*anti*-3l)

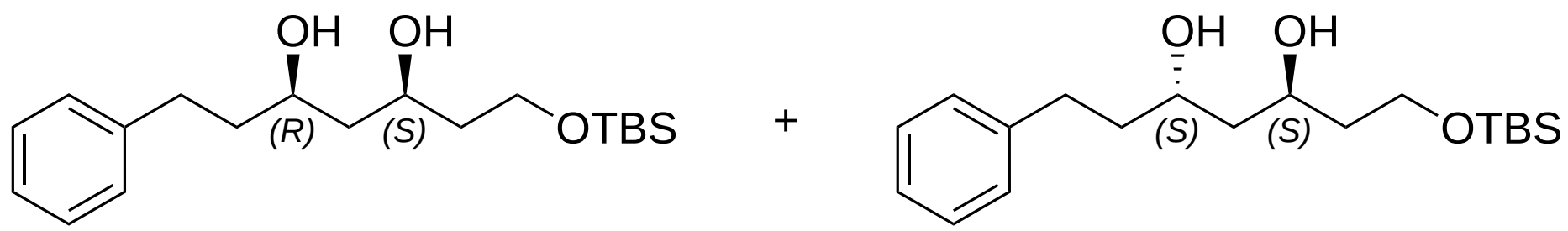

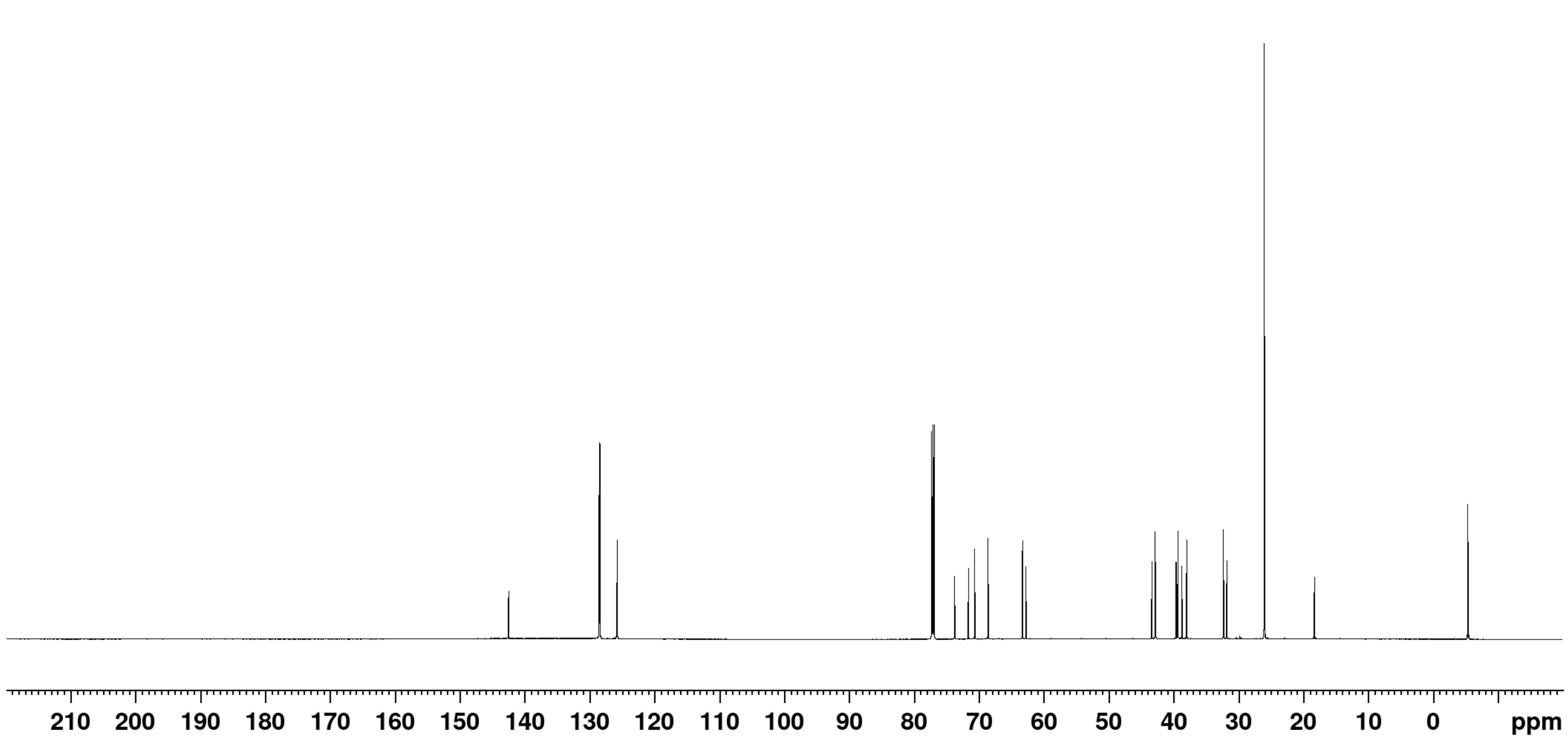

(2*R*,4*S*)-1-tosylpentane-2,4-diol (*syn*-3m) and (2*S*,4*S*)-1-tosylpentane-2,4-diol (*anti*-3m)

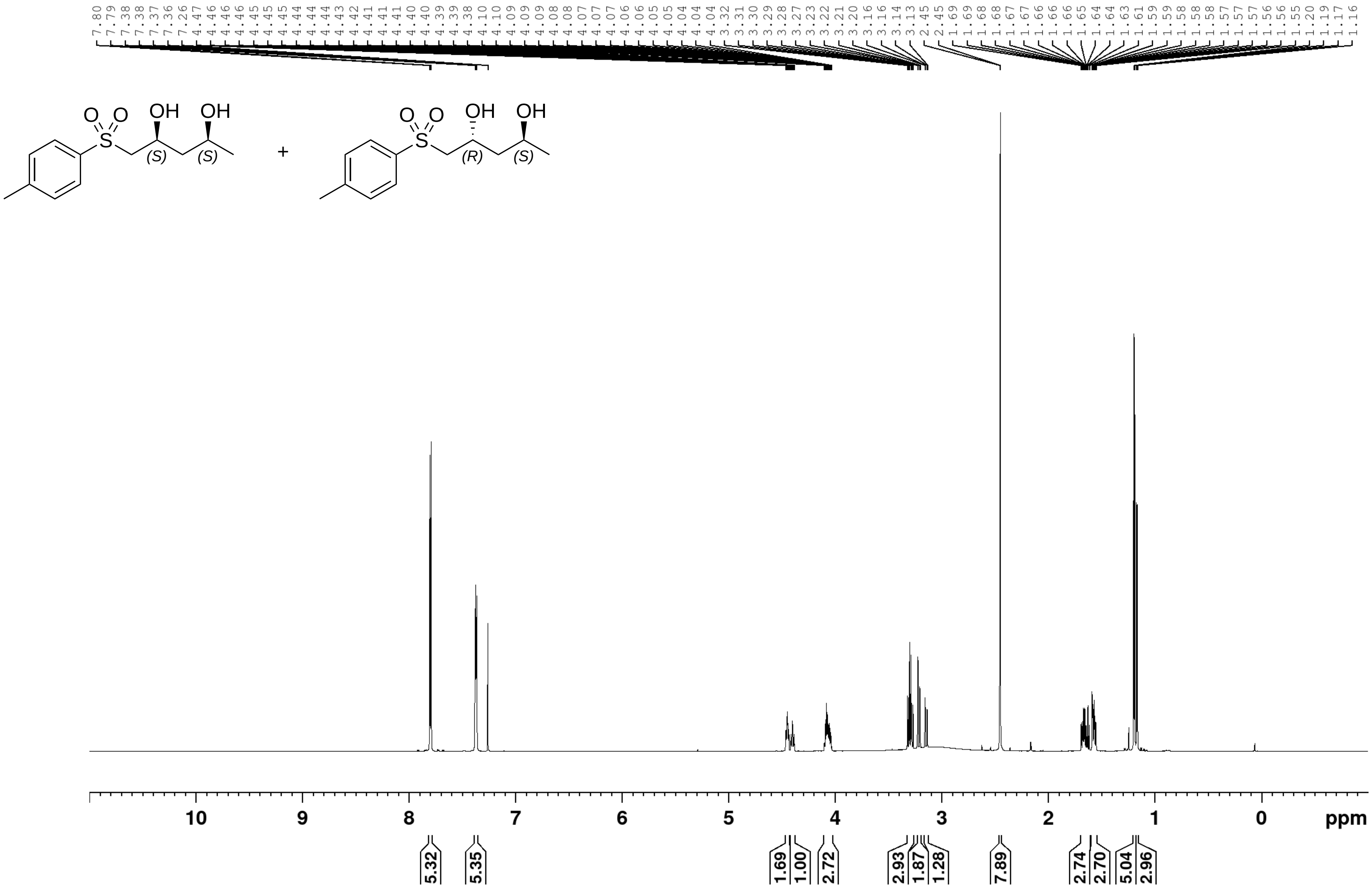

(2*R*,4*S*)-1-tosylpentane-2,4-diol (*syn*-3m) and (2*S*,4*S*)-1-tosylpentane-2,4-diol (*anti*-3m)

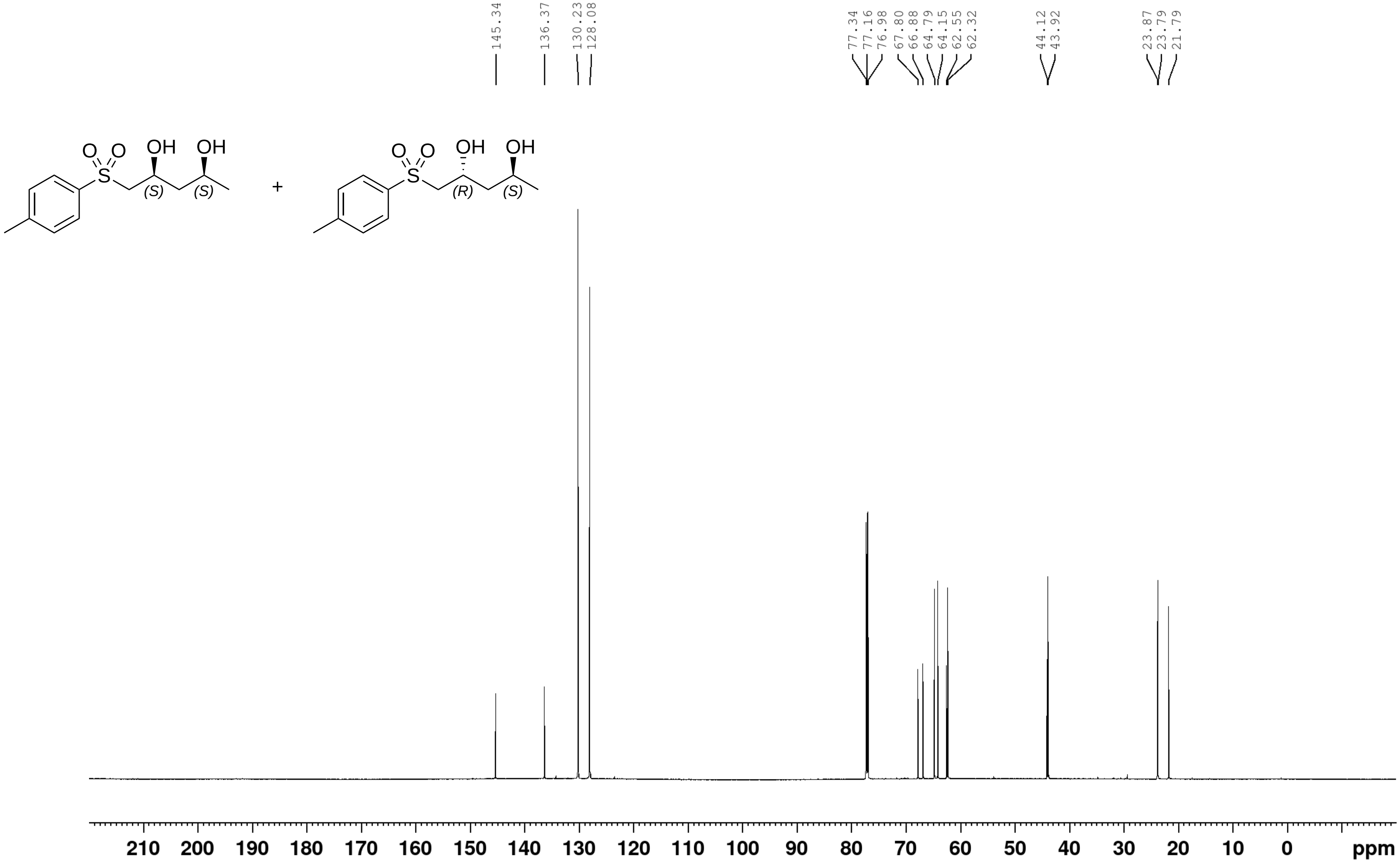

(*S*,*S*)-1-Phenylpentane-2,4-diol (*S*,*S*-3a)

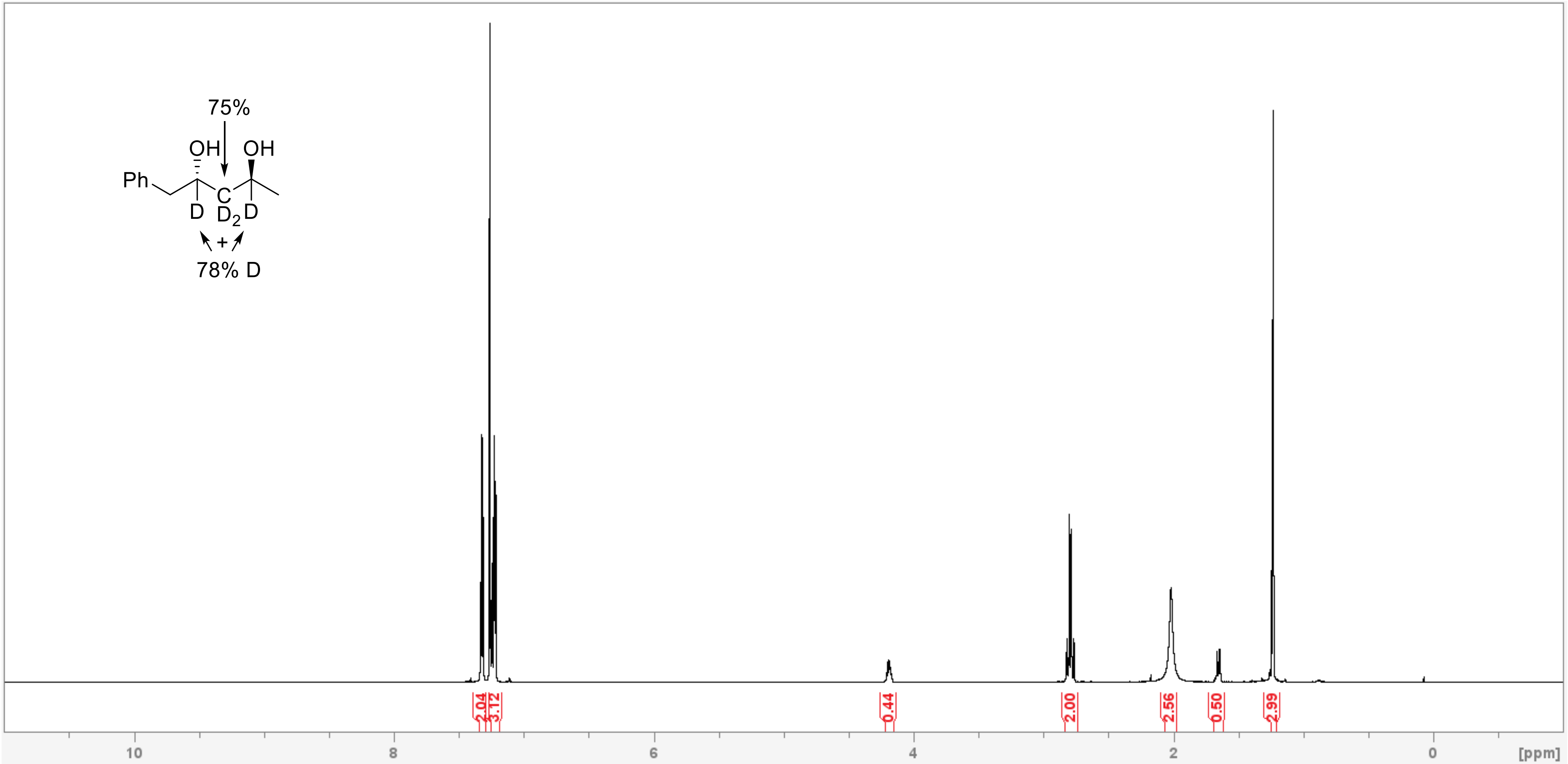

(*S*,*S*)-1-Phenylpentane-2,4-diol (*S*,*S*-3a)

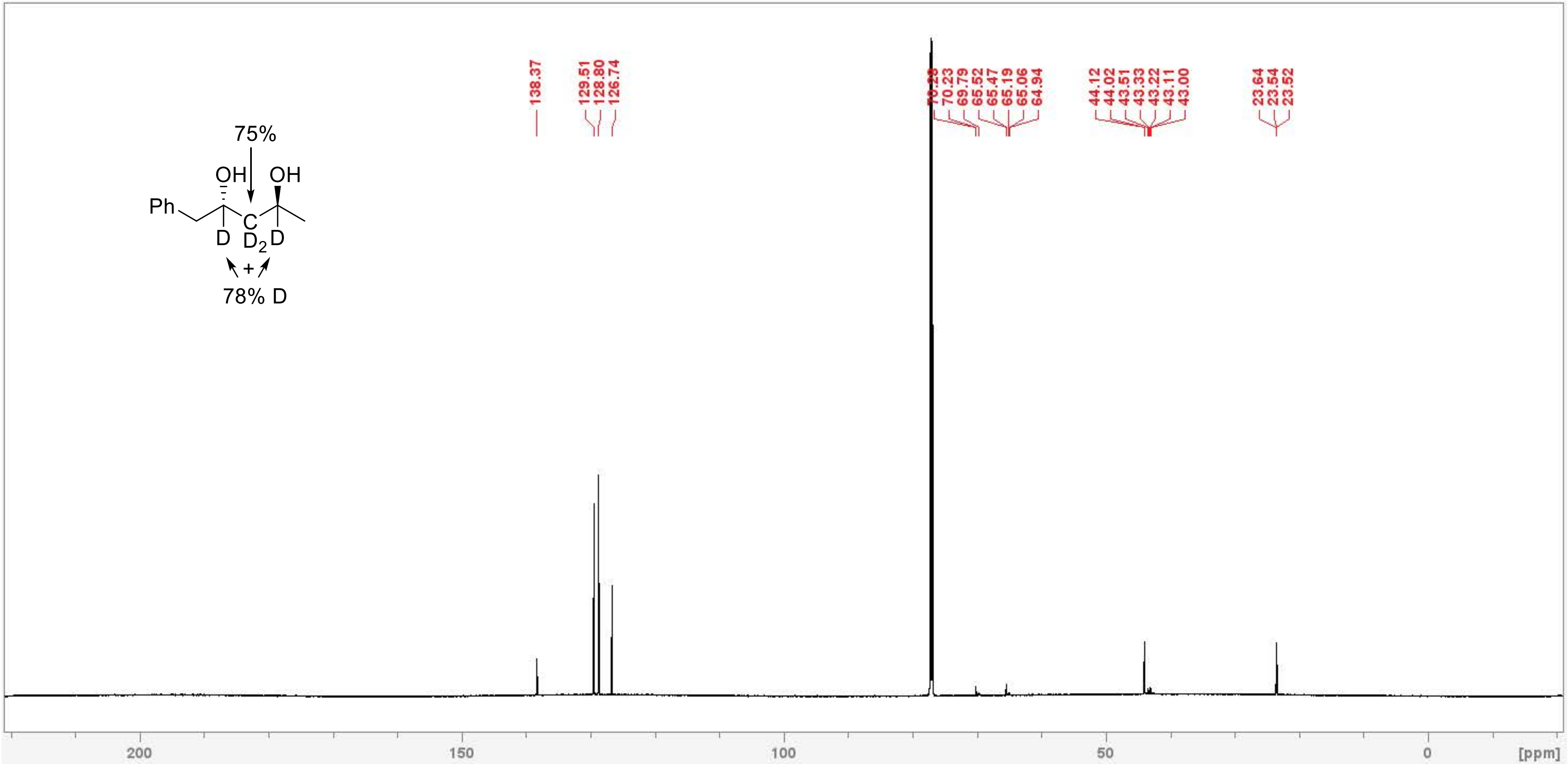

(*S*,*S*)-1-Phenylpentane-2,4-diol (*S*,*S*-3a)

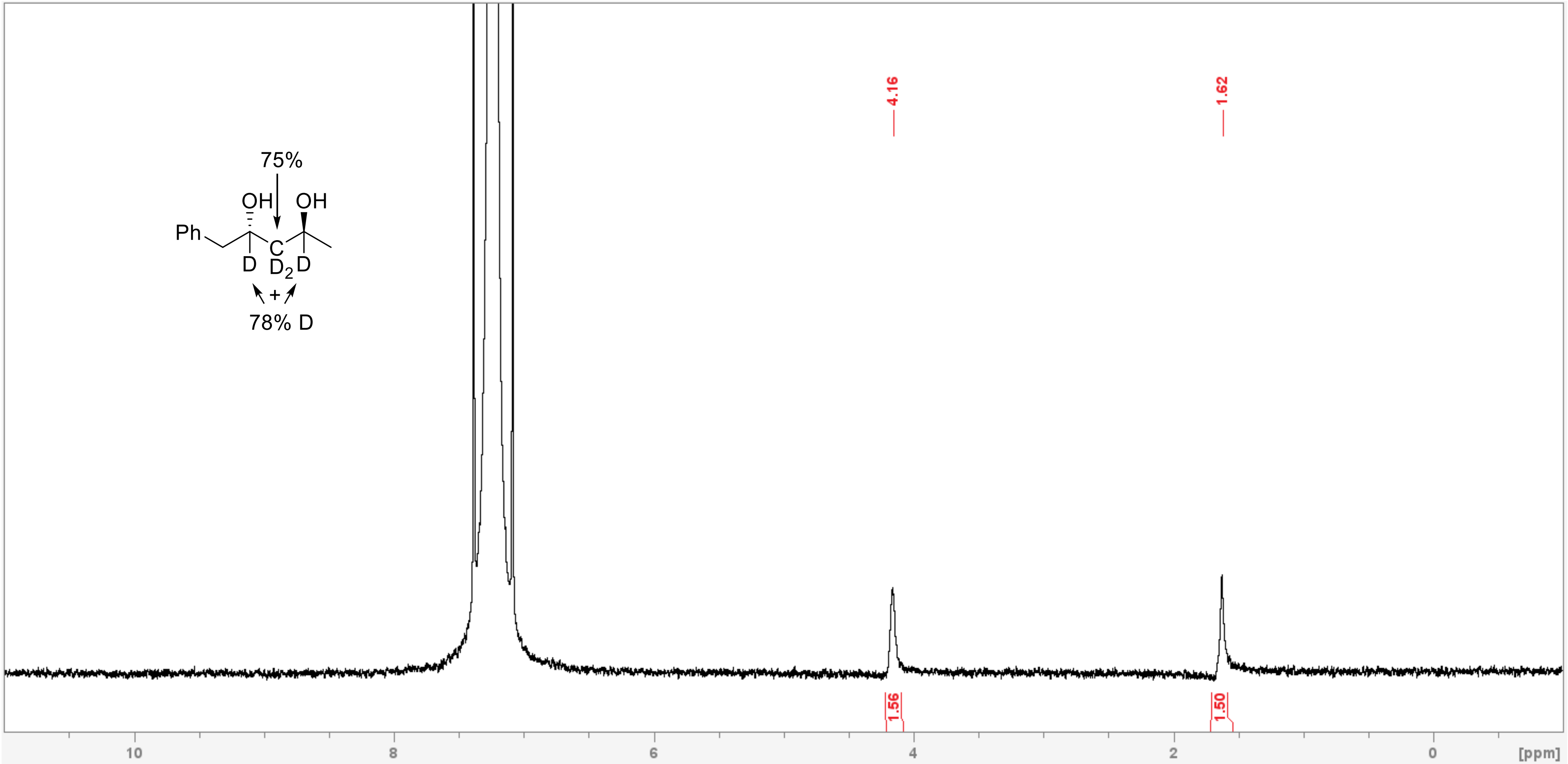

(2*R*,4*S*)-1-Phenylpentane-2,4-diol (2*R*,4*S*-3a)

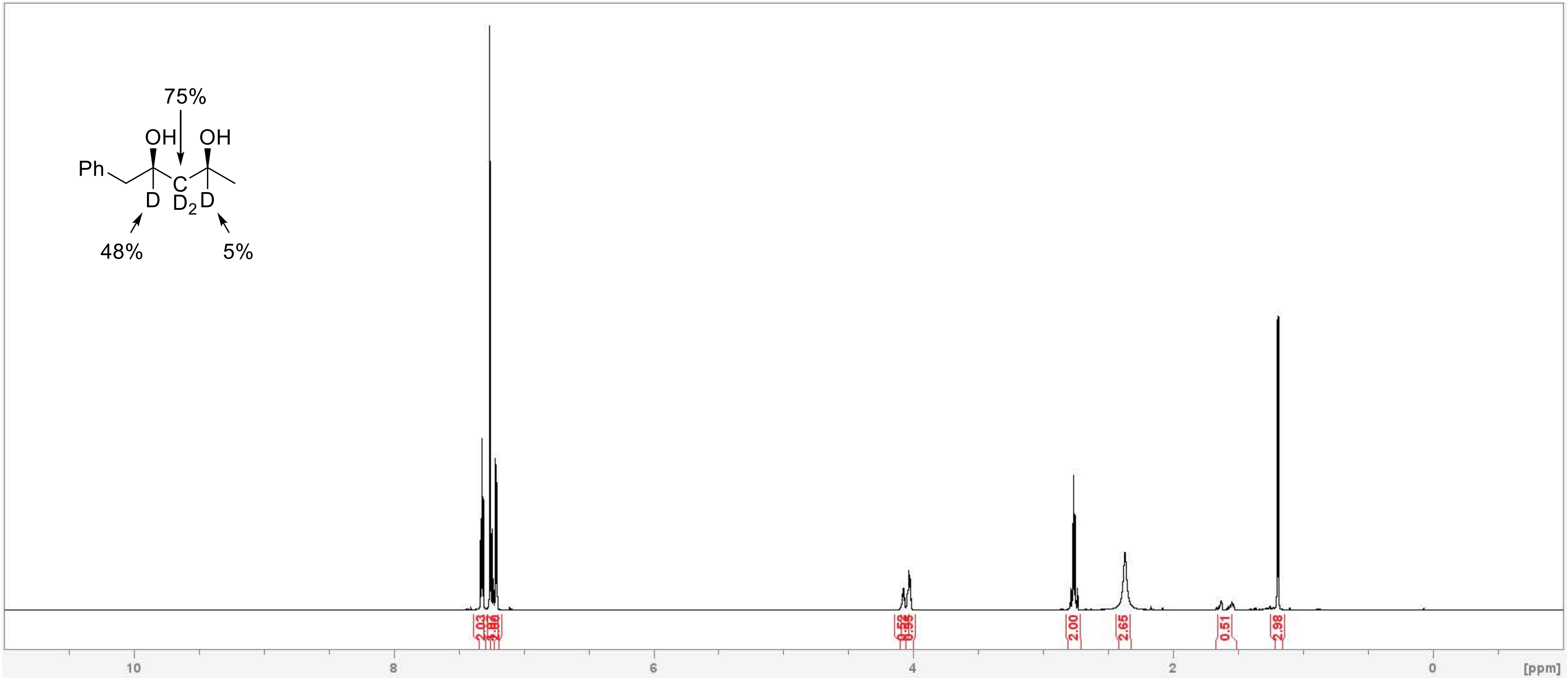

(2*R*,4*S*)-1-Phenylpentane-2,4-diol (2*R*,4*S*-3a)

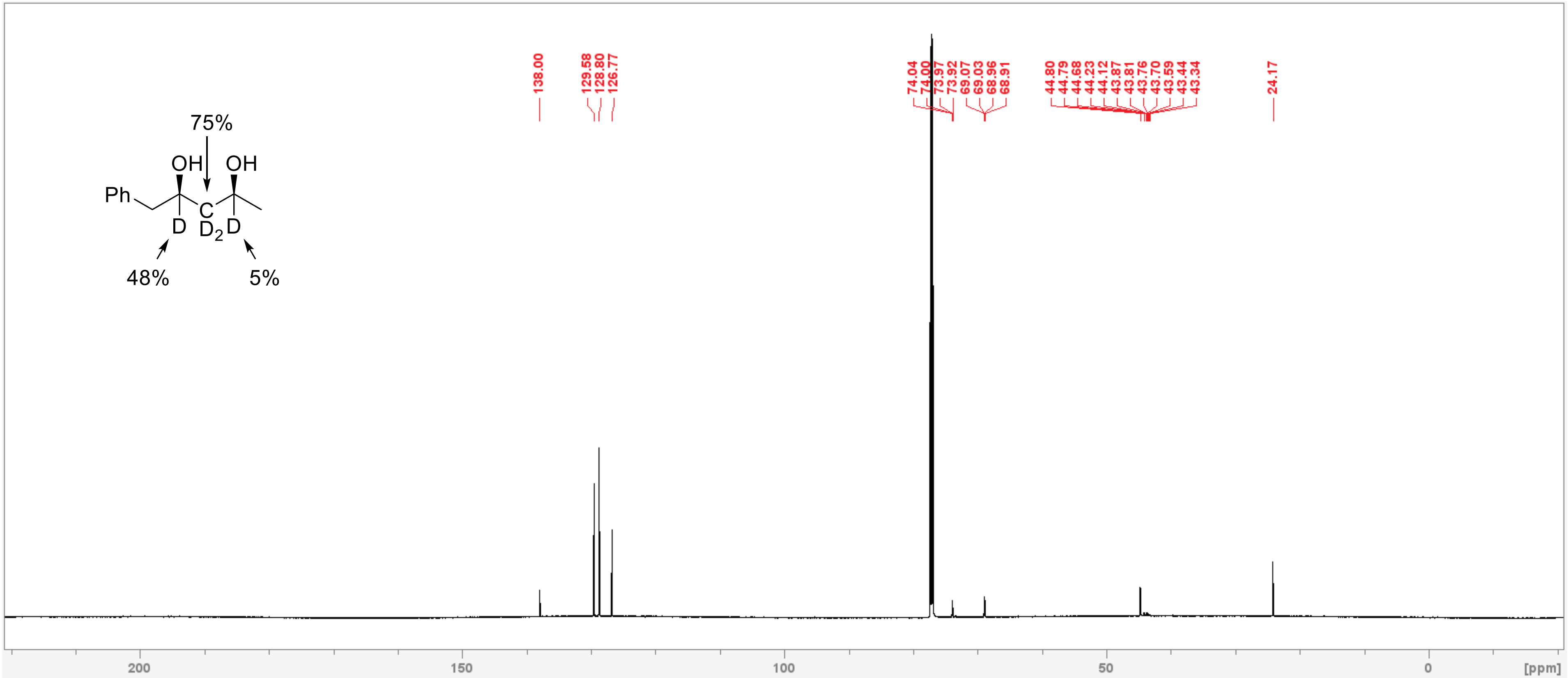

(2*R*,4*S*)-1-Phenylpentane-2,4-diol (2*R*,4*S*-3a)

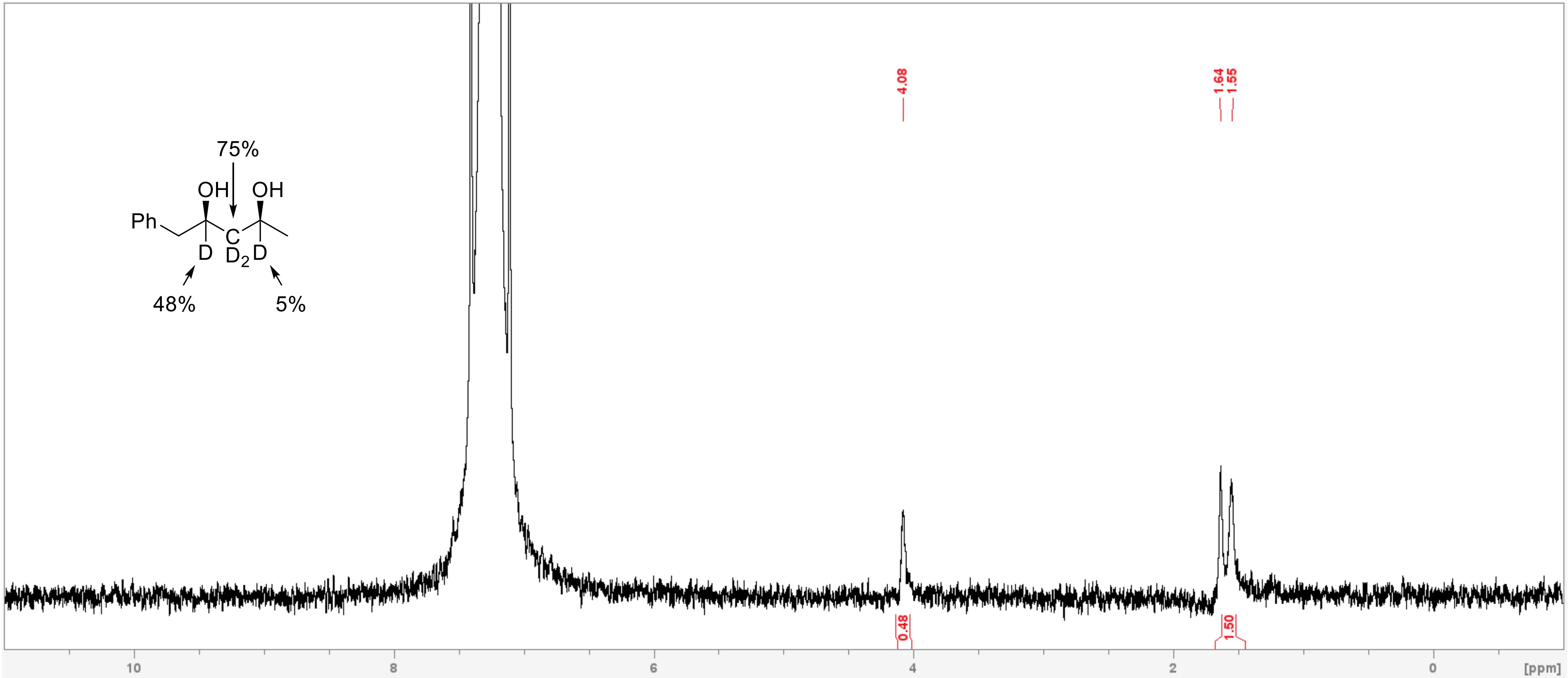

(*S*,*S*)-1-Phenylpentane-2,4-diol (*S*,*S*-3a)

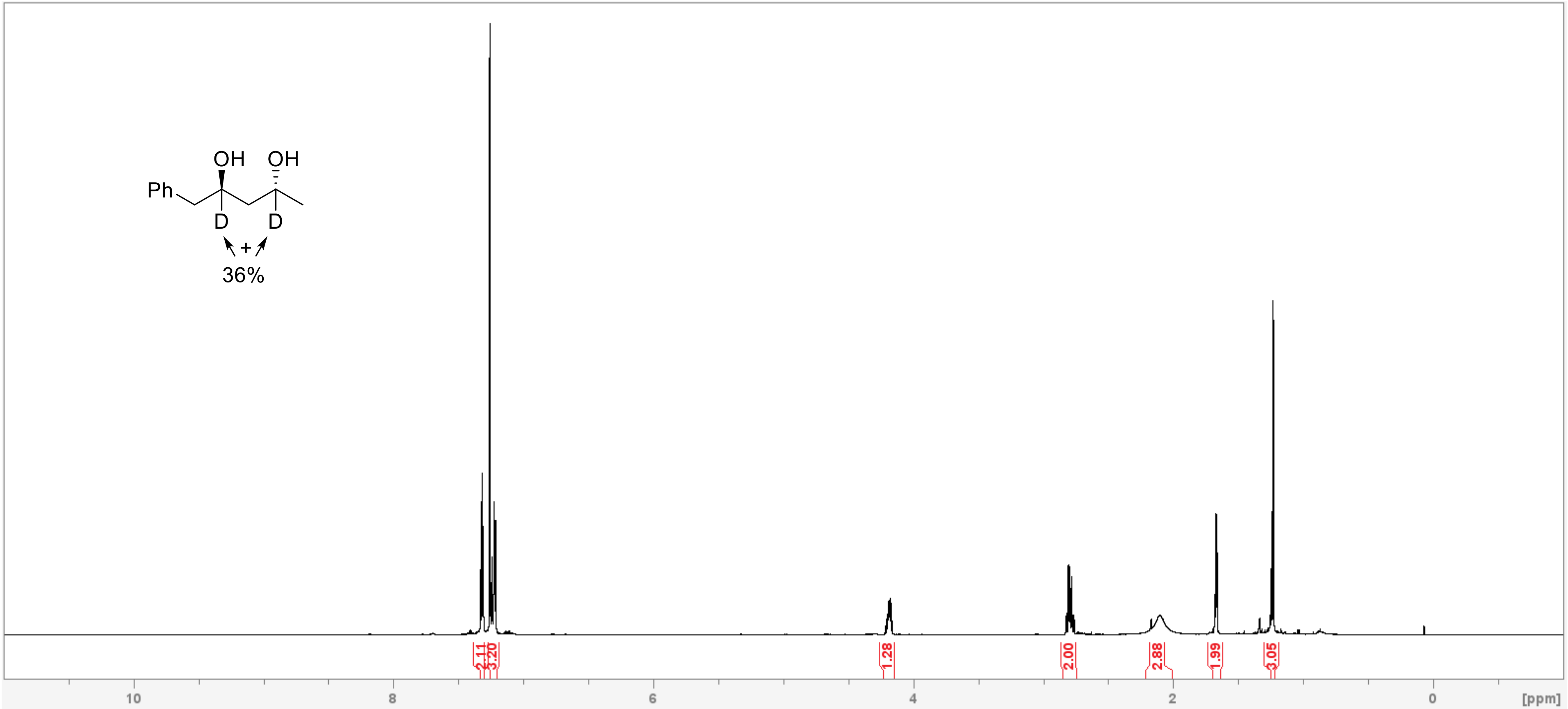

(*S*,*S*)-1-Phenylpentane-2,4-diol (*S*,*S*-3a)

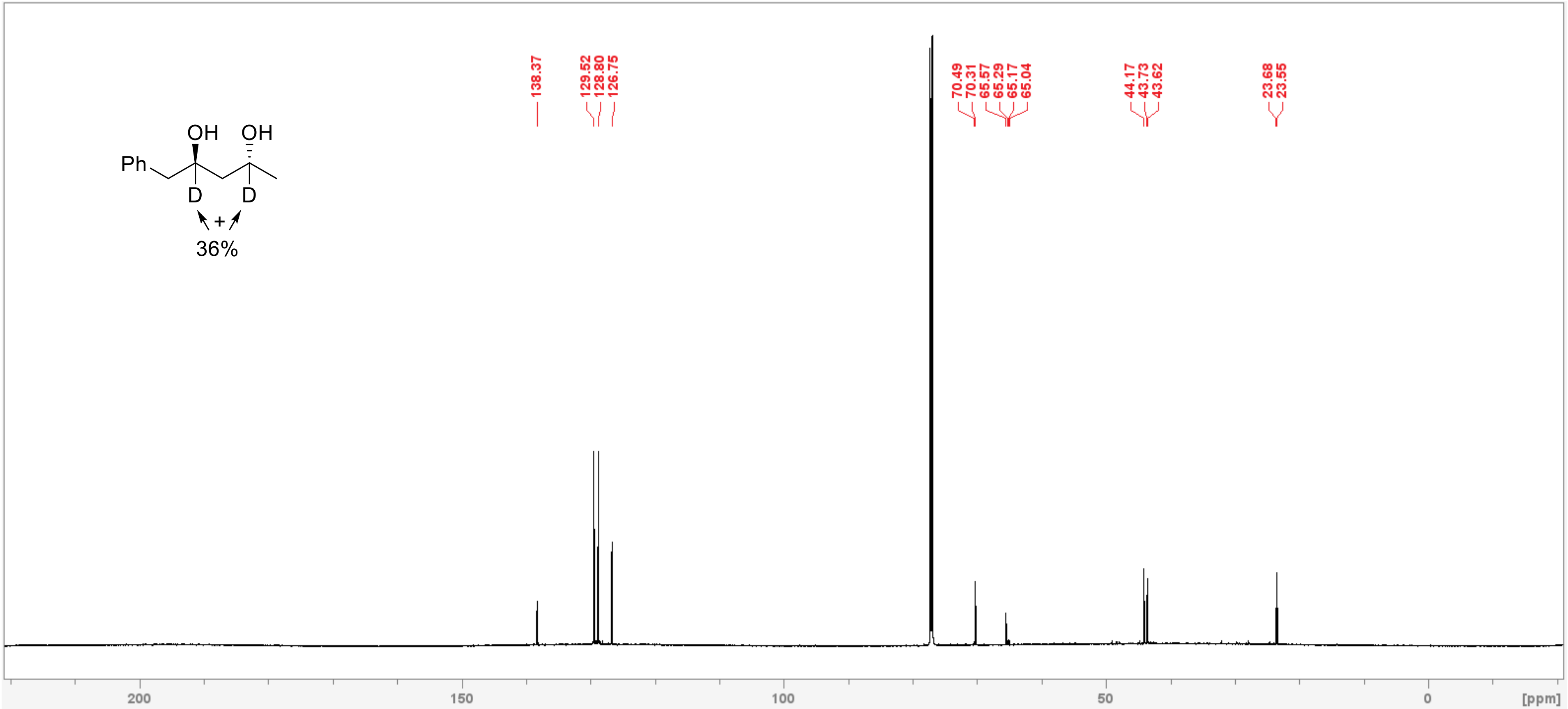

(*S*,*S*)-1-Phenylpentane-2,4-diol (*S*,*S*-3a)

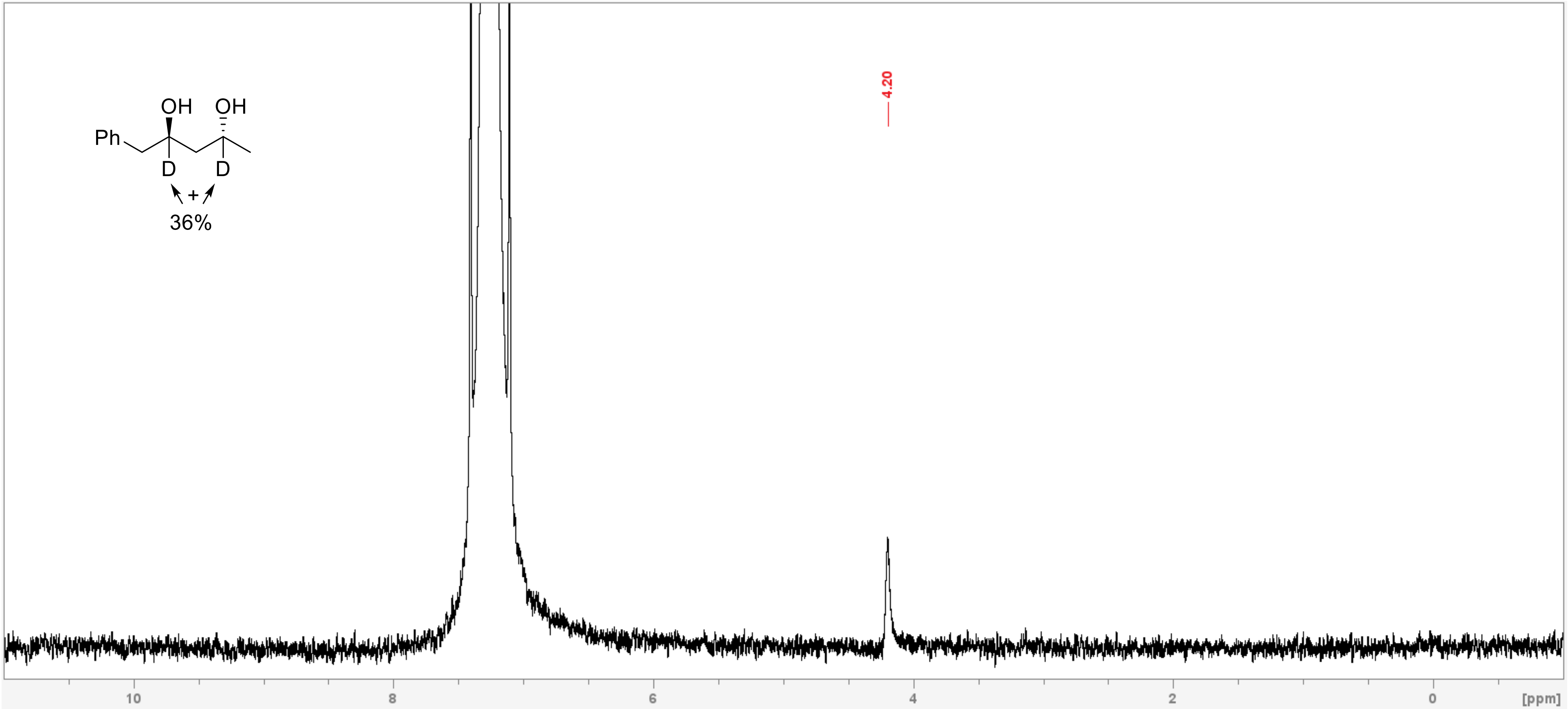

(2*R*,4*S*)-1-Phenylpentane-2,4-diol (2*R*,4*S*-3a)

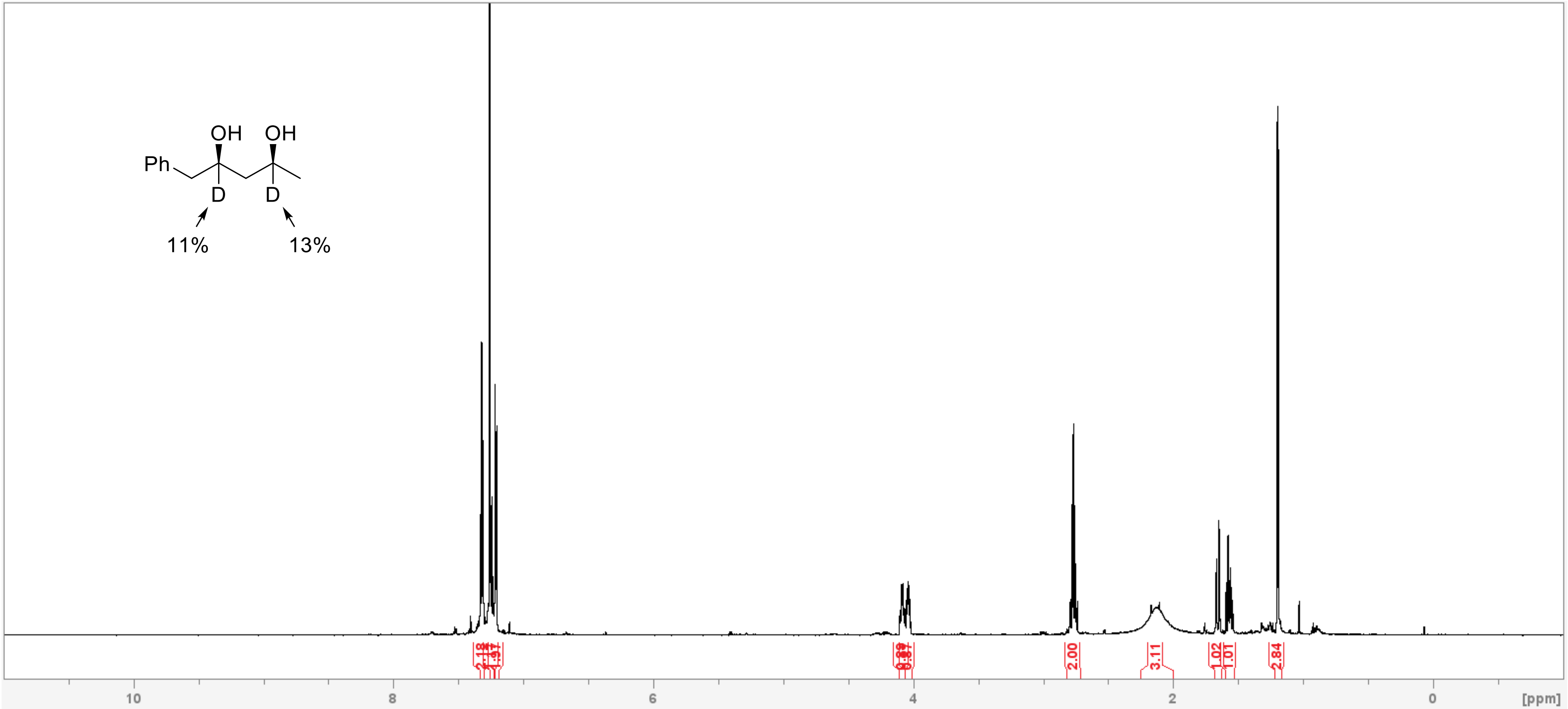

(2*R*,4*S*)-1-Phenylpentane-2,4-diol (2*R*,4*S*-3a)

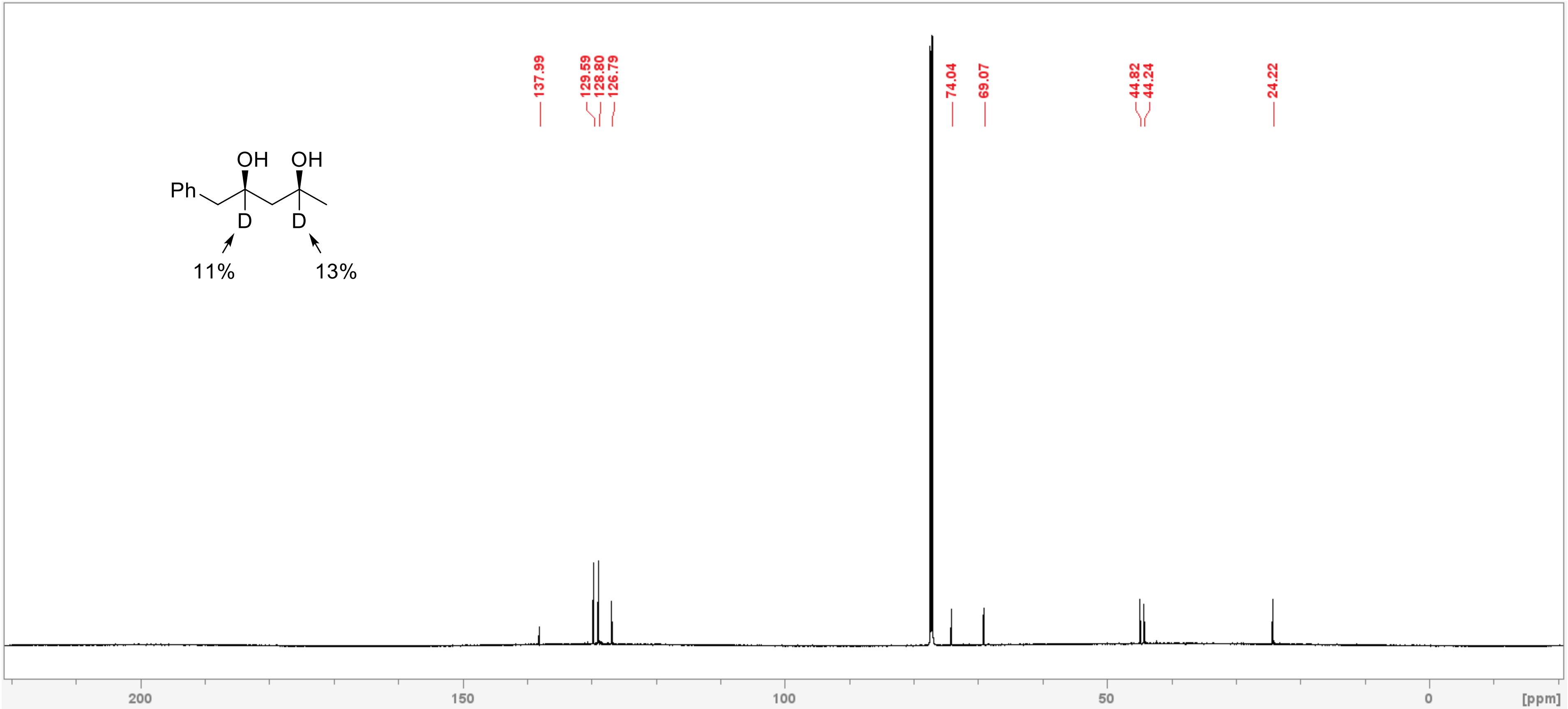

(*S*,*S*)-6-Phenylhexane-2,4-diol (*S*,*S*-3d)

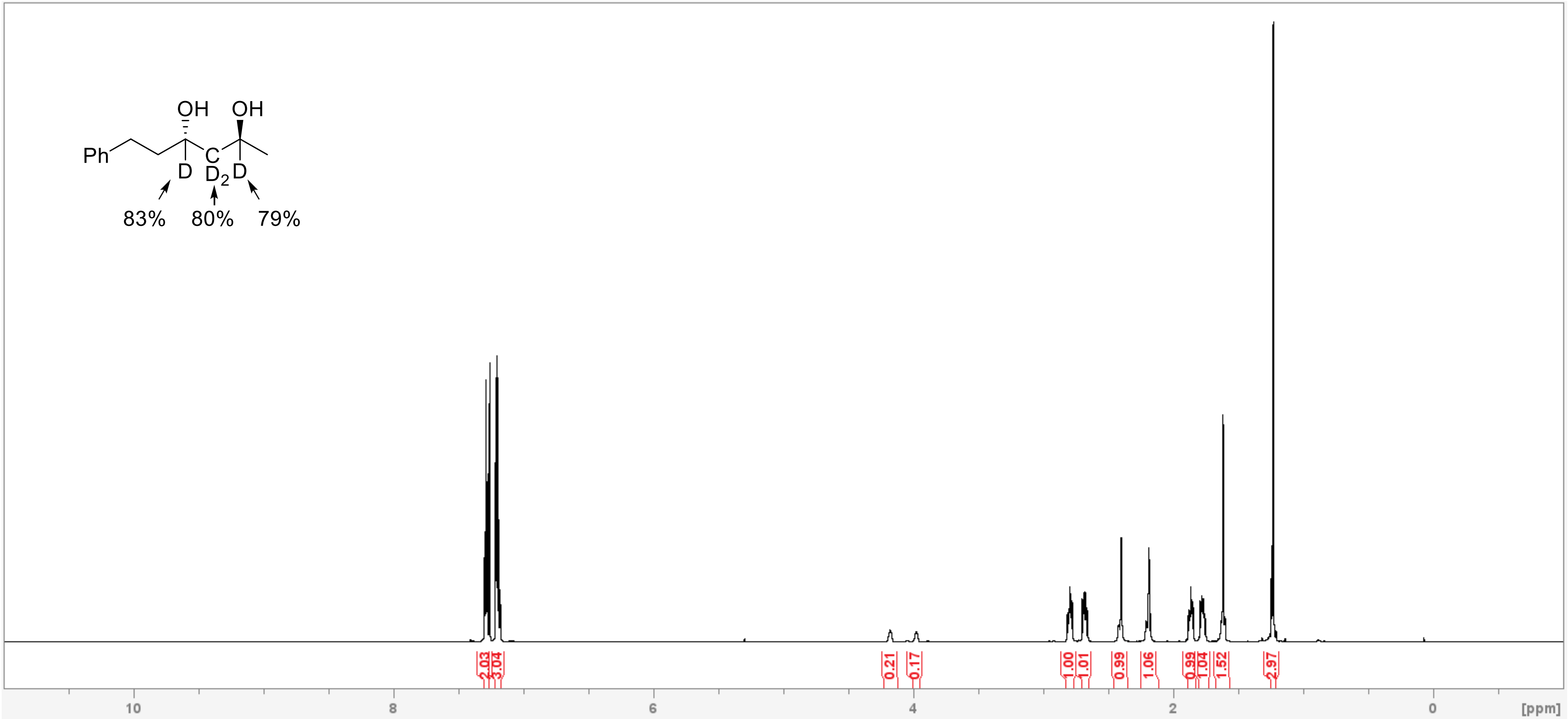

(*S*,*S*)-6-Phenylhexane-2,4-diol (*S*,*S*-3d)

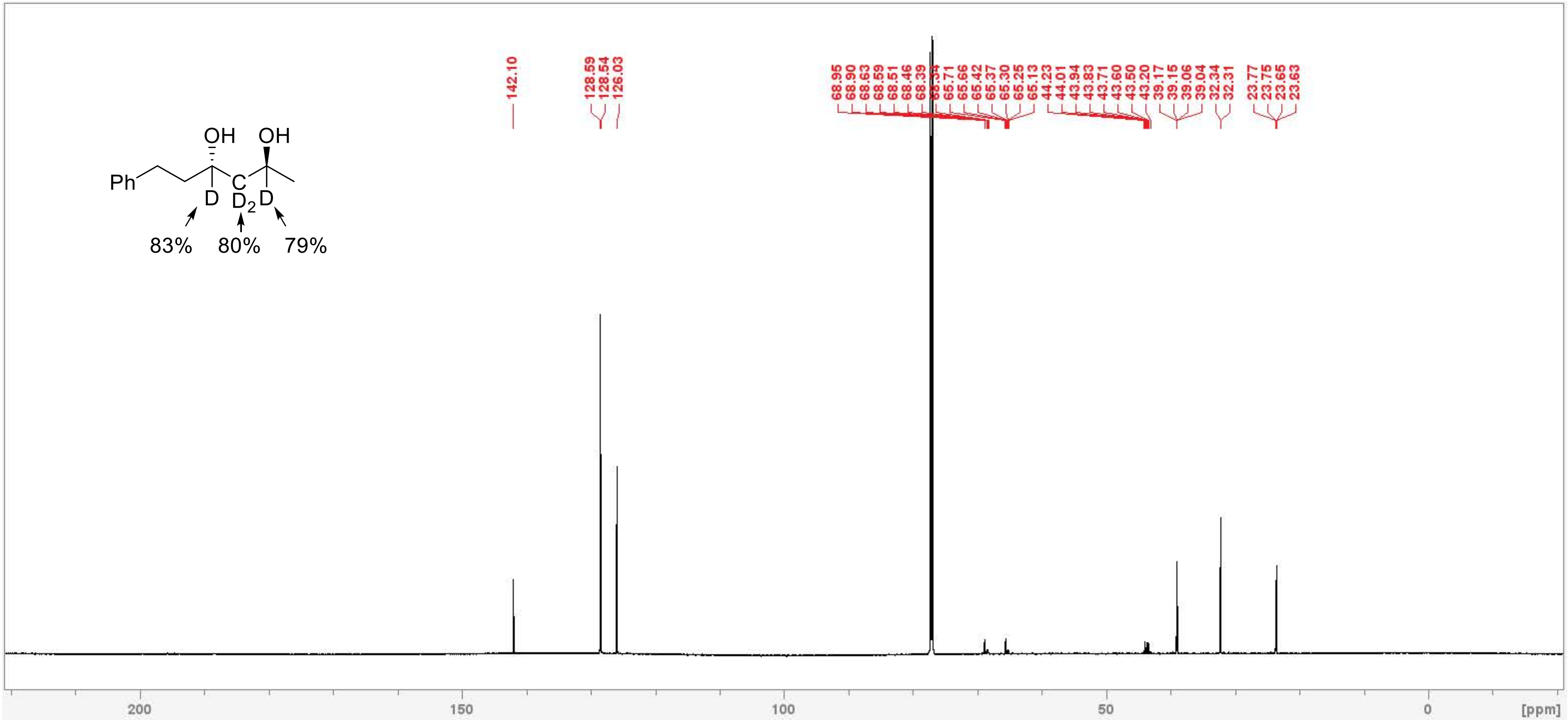

(*S*,*S*)-6-Phenylhexane-2,4-diol (*S*,*S*-3d)

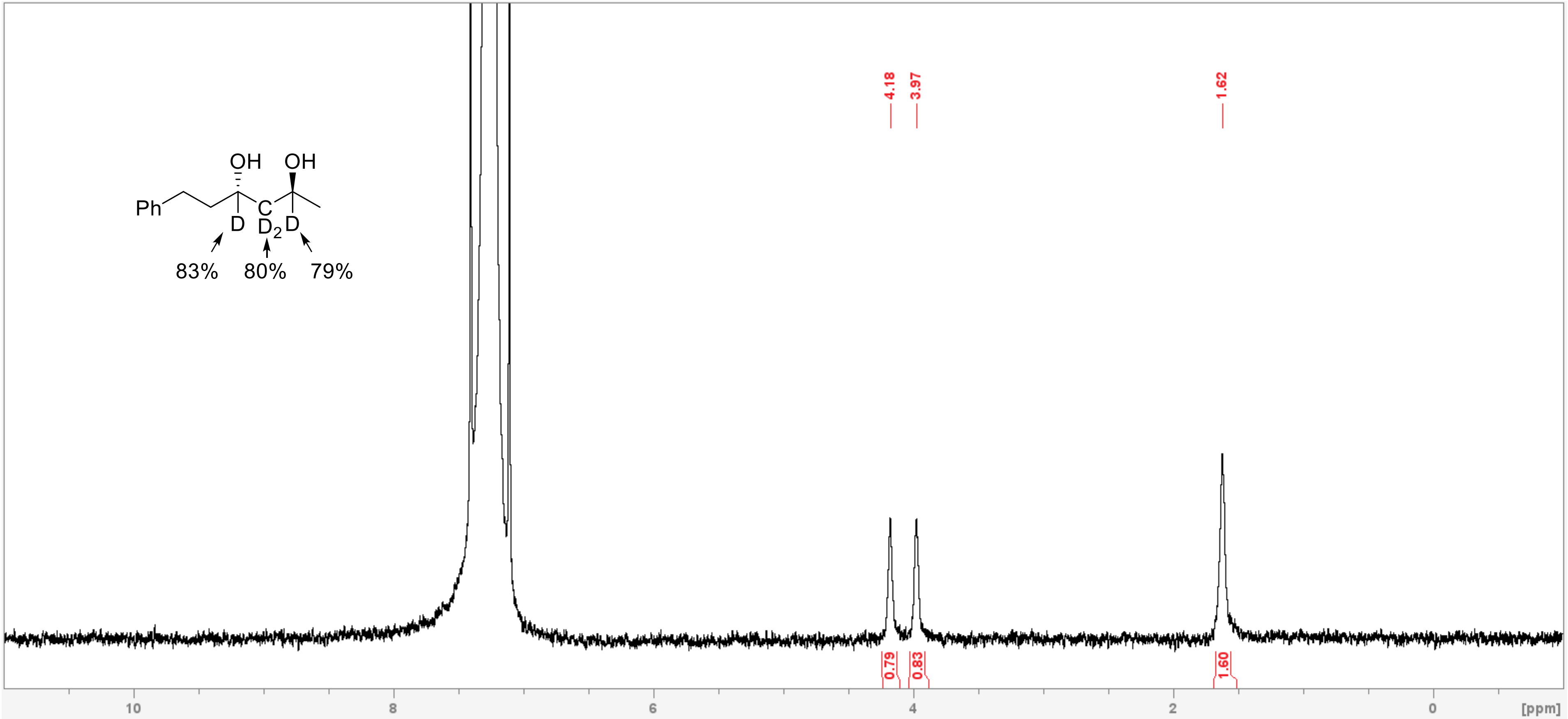

(2*S*,4*R*)-6-Phenylhexane-2,4-diol (2*S*,4*R*-3d)

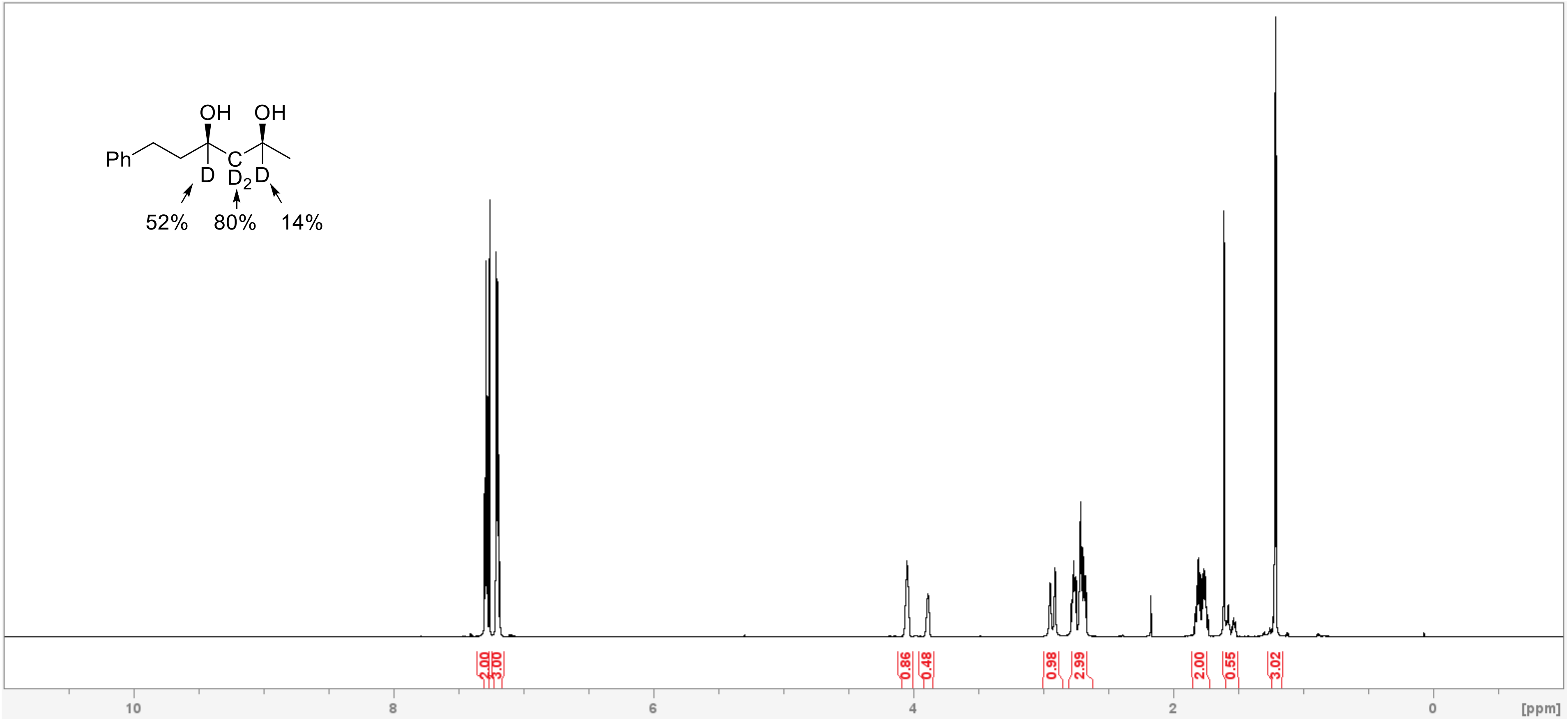

(2*S*,4*R*)-6-Phenylhexane-2,4-diol (2*S*,4*R*-3d)

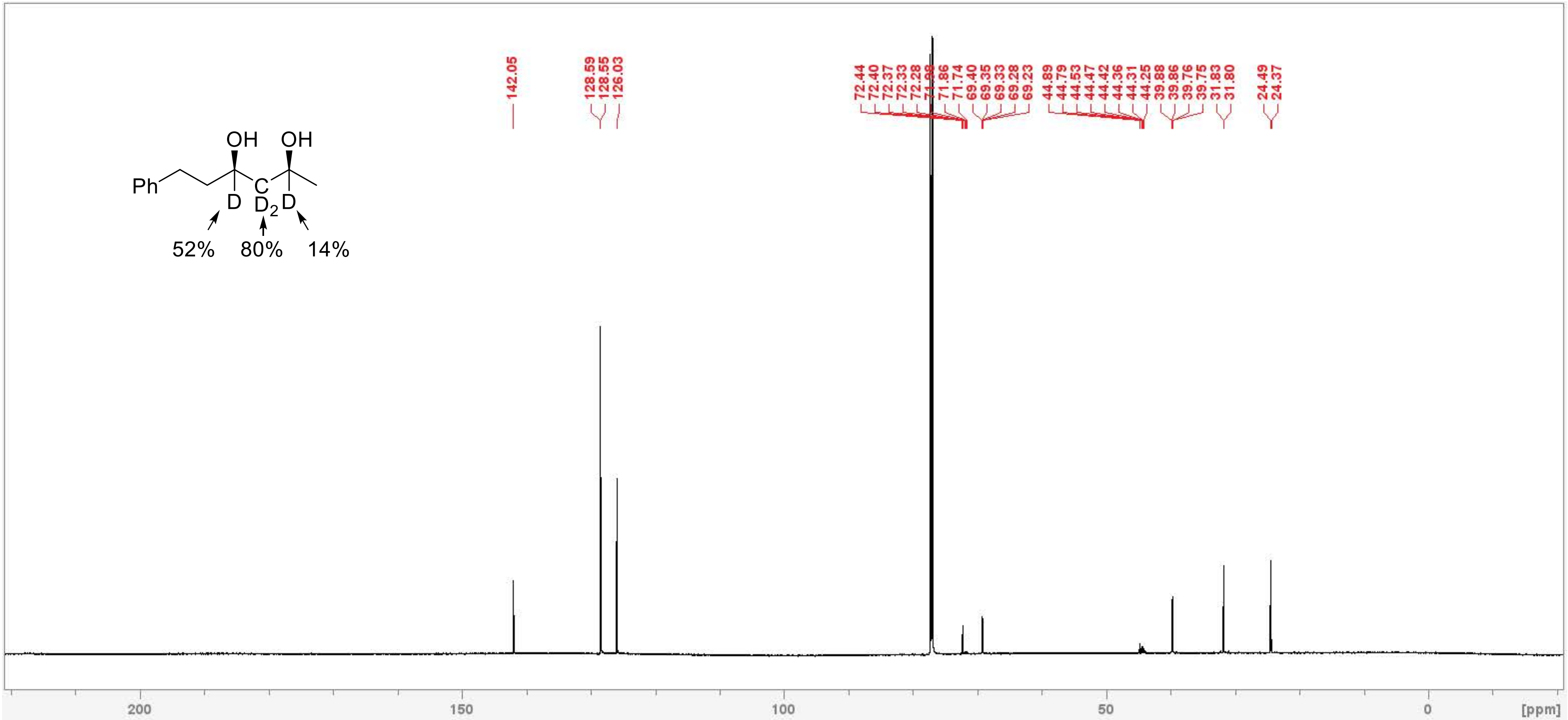

(2*S*,4*R*)-6-Phenylhexane-2,4-diol (2*S*,4*R*-3d)

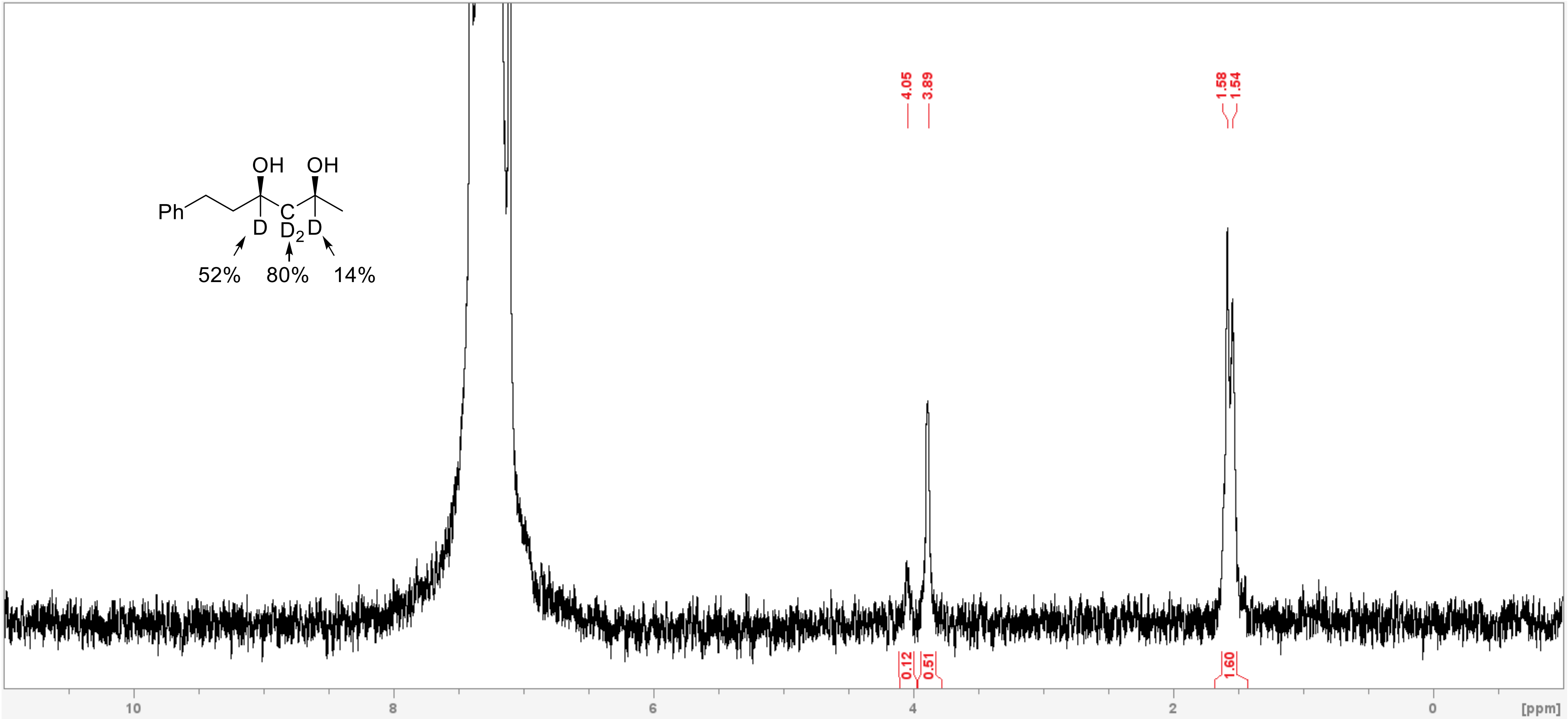

(2*S*,3*S*)-butane-2,3-diyl dibenzoate (4a-Bz)

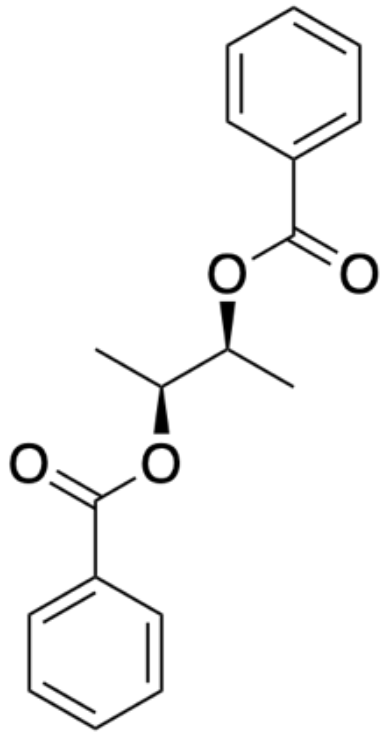

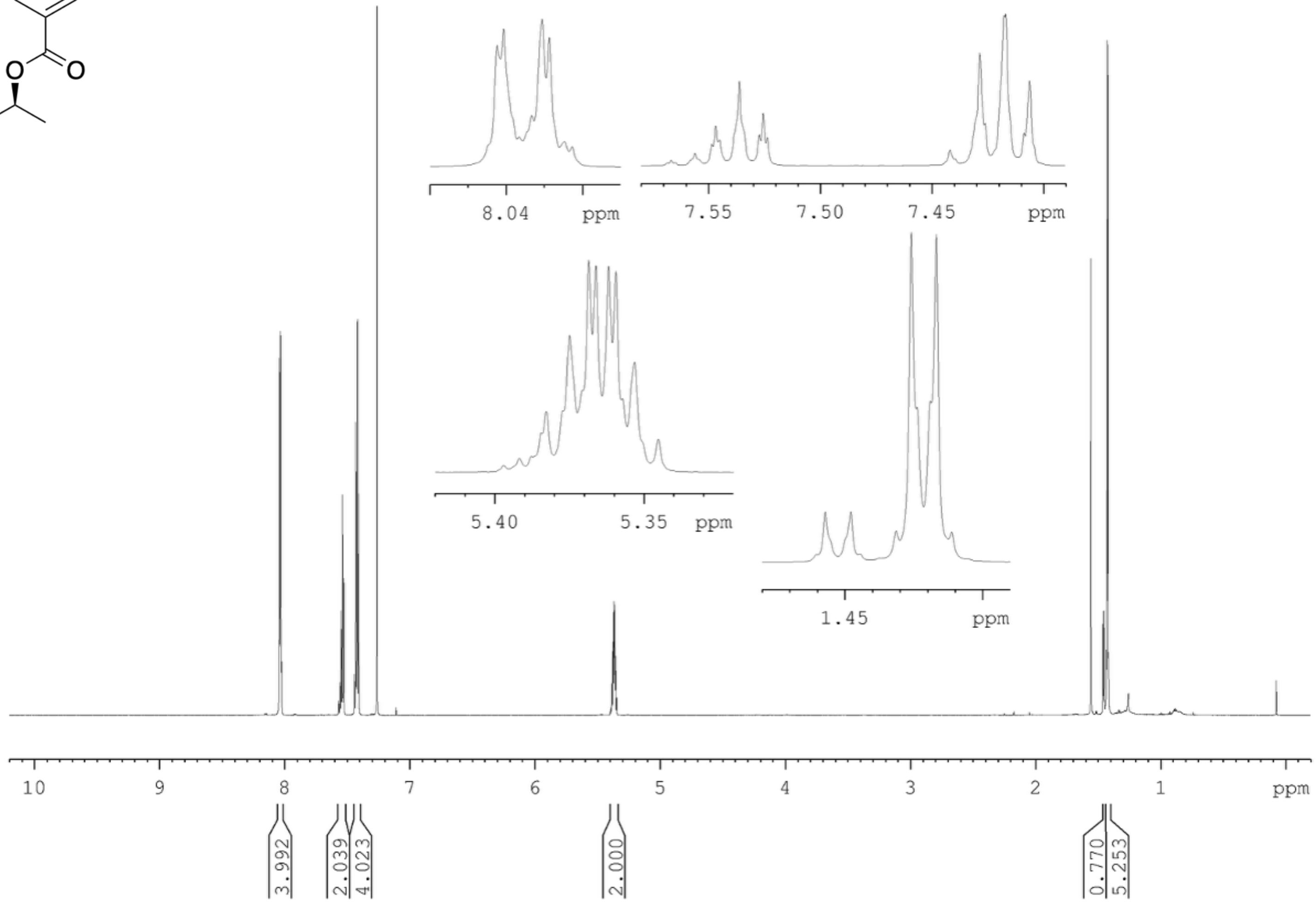

7:1 dr determined using the signals from the protons

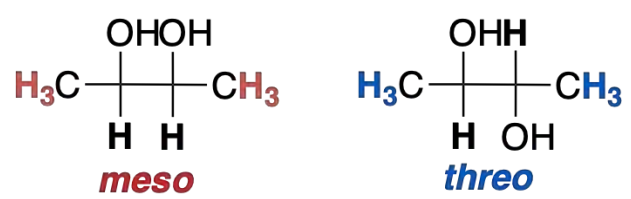

(2*S*,3*S*)-butane-2,3-diyl dibenzoate (4a-Bz)

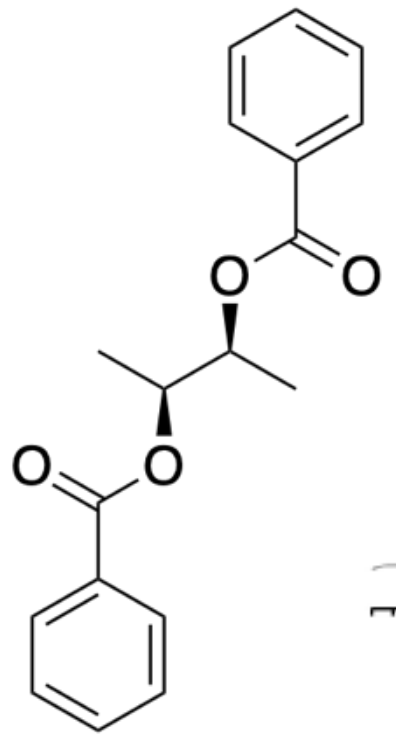

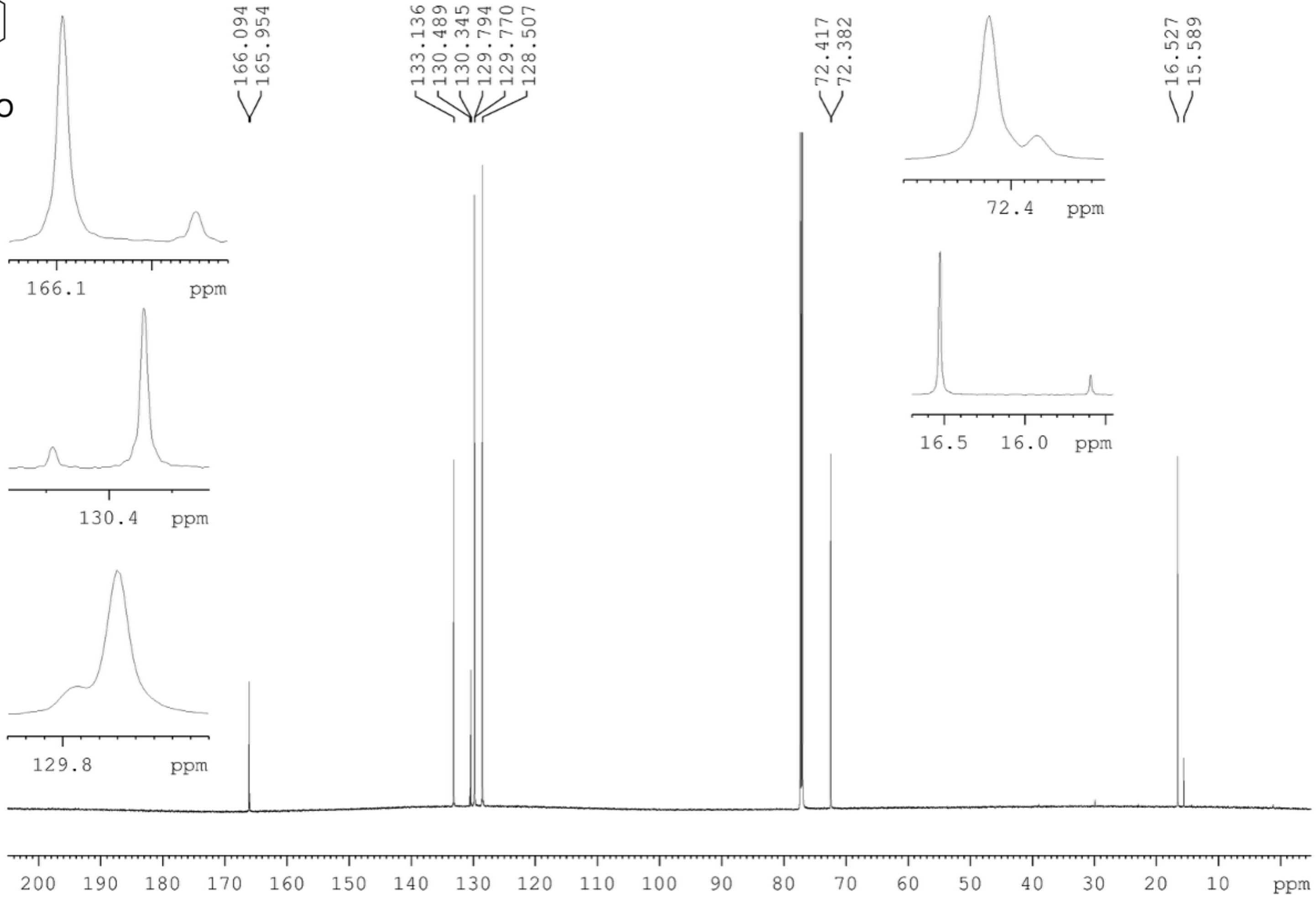

(3*S*,4*S*)-hexane-3,4-diyl dibenzoate (4b-Bz)

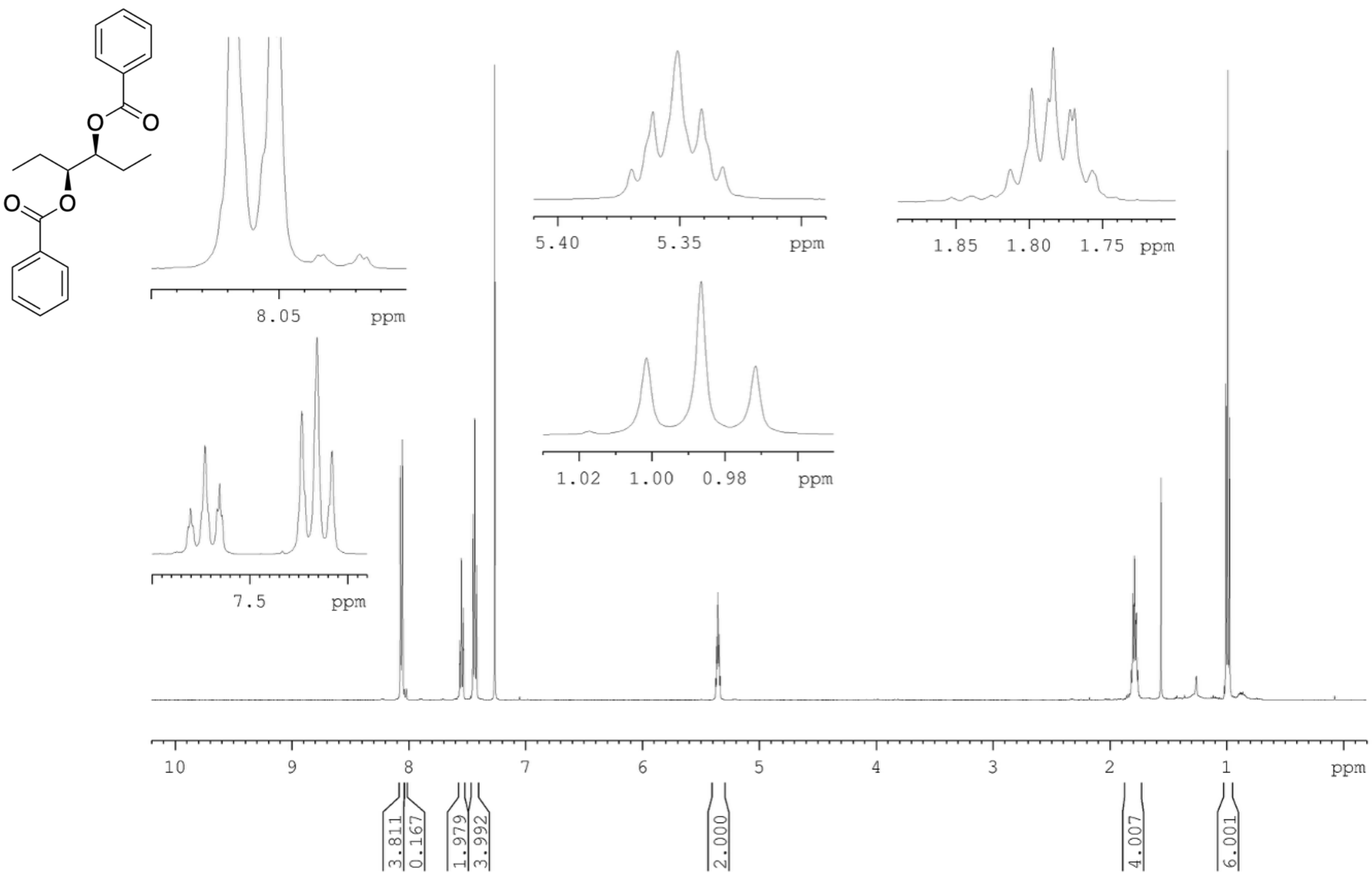

(3*S*,4*S*)-hexane-3,4-diyl dibenzoate (4b-Bz)

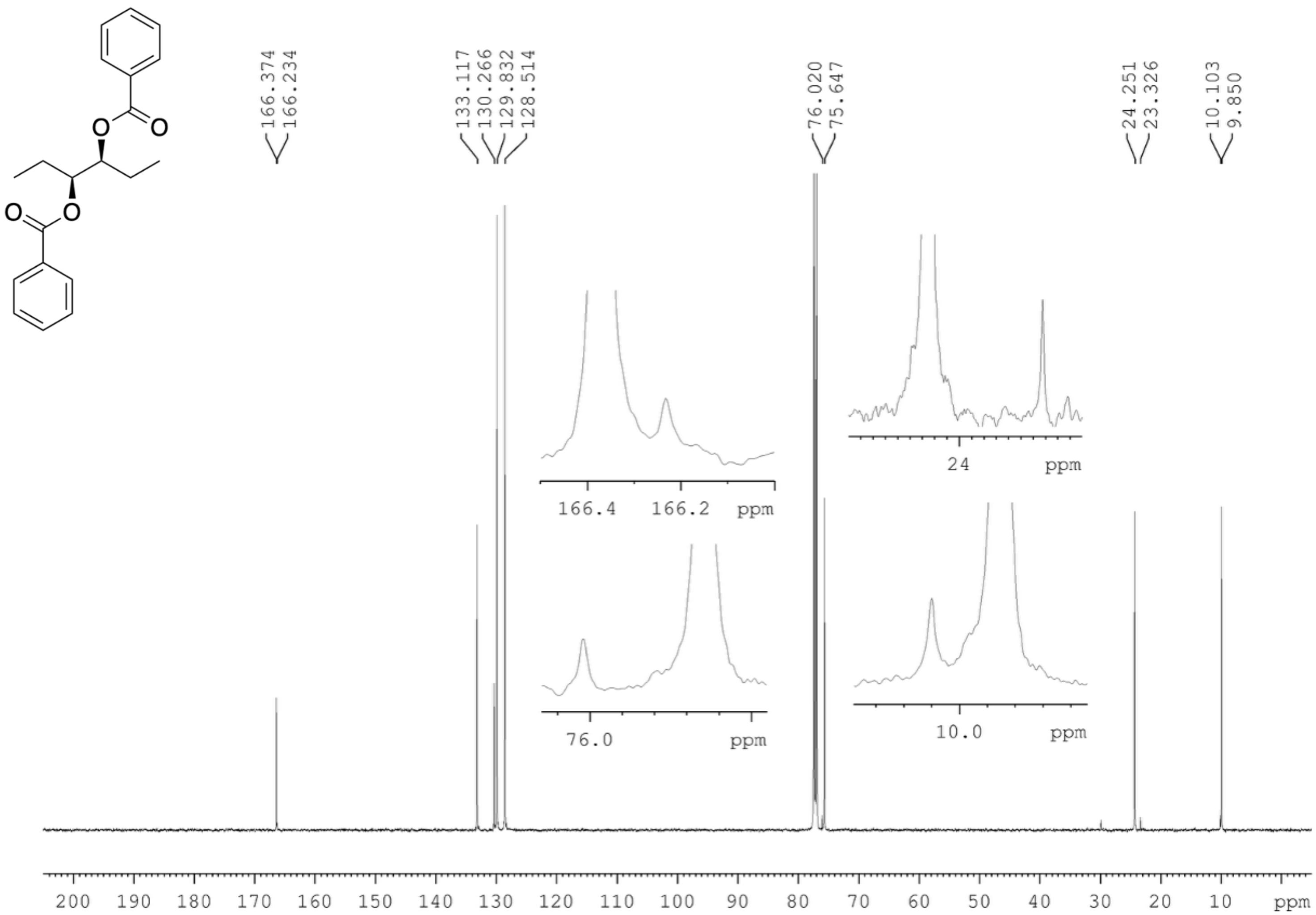

(4S,5S)-octane-4,5-diol (4c)

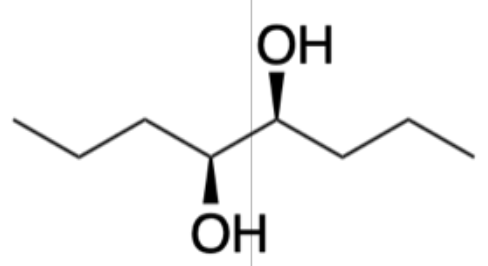

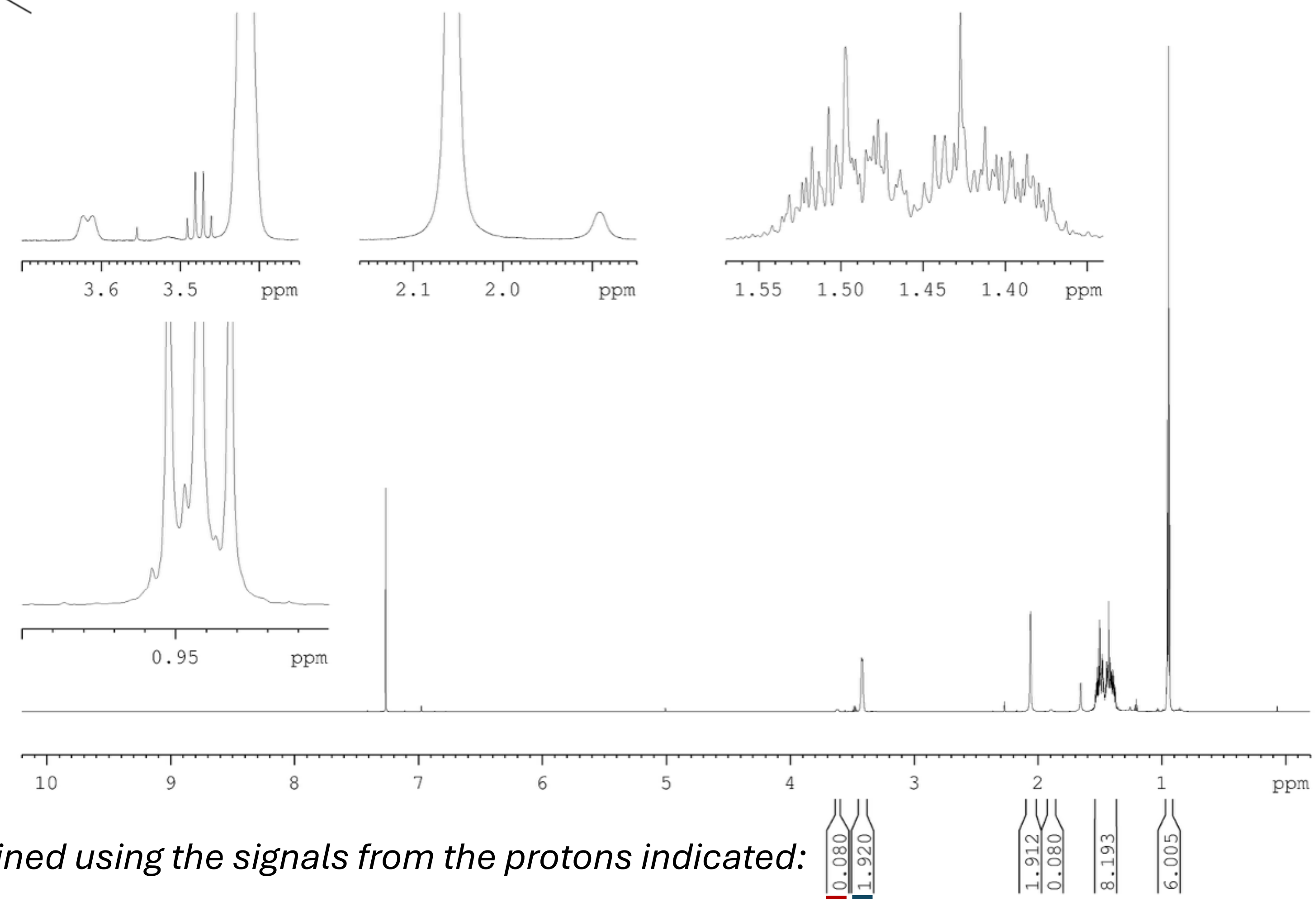

24:1 dr determined using the signals from the protons indicated:

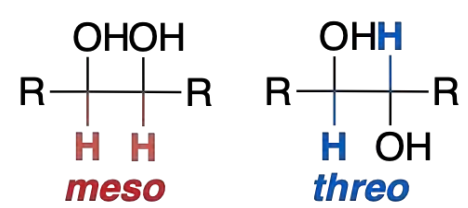

(4S,5S)-octane-4,5-diol (4c)

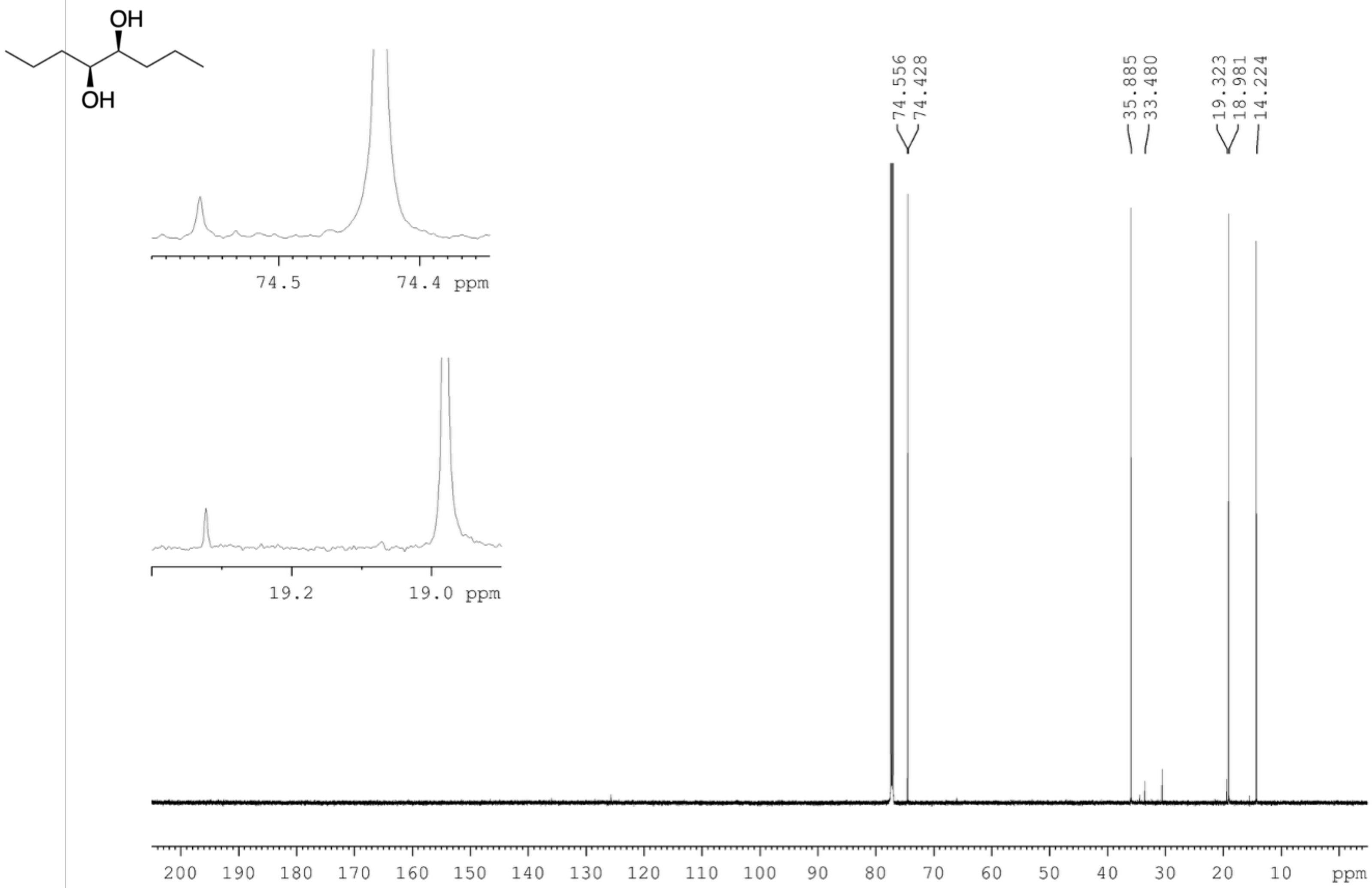

(4*S*,5*S*)-octane-4,5-diyl dibenzoate (4c-Bz)

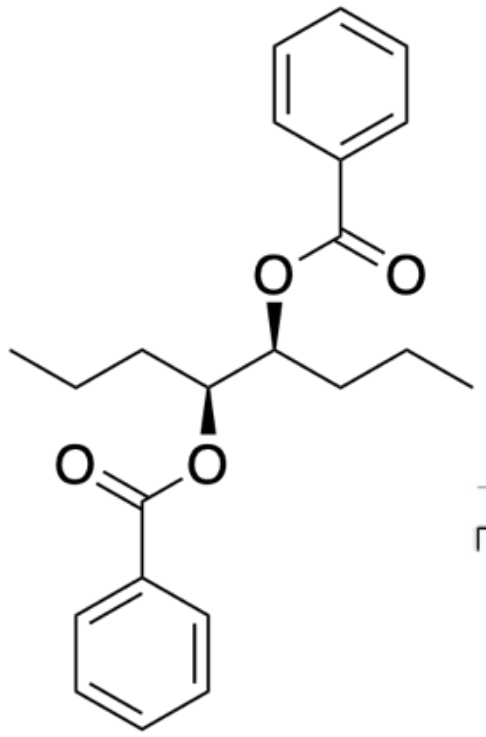

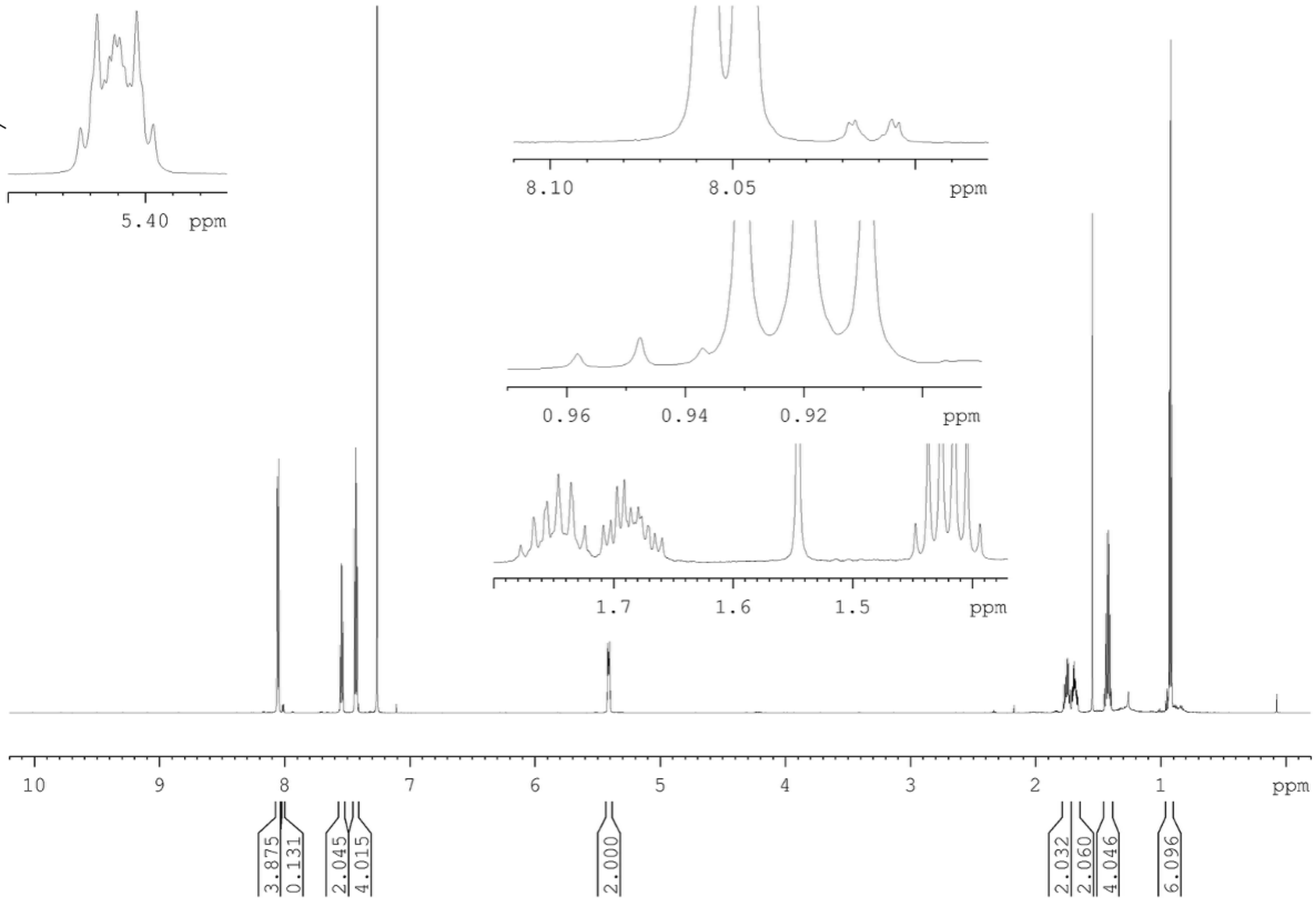

(4*S*,5*S*)-octane-4,5-diyl dibenzoate (4c-Bz)

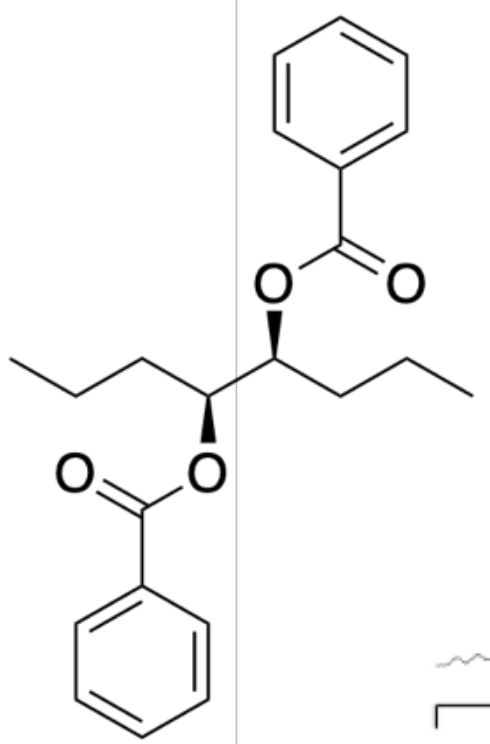

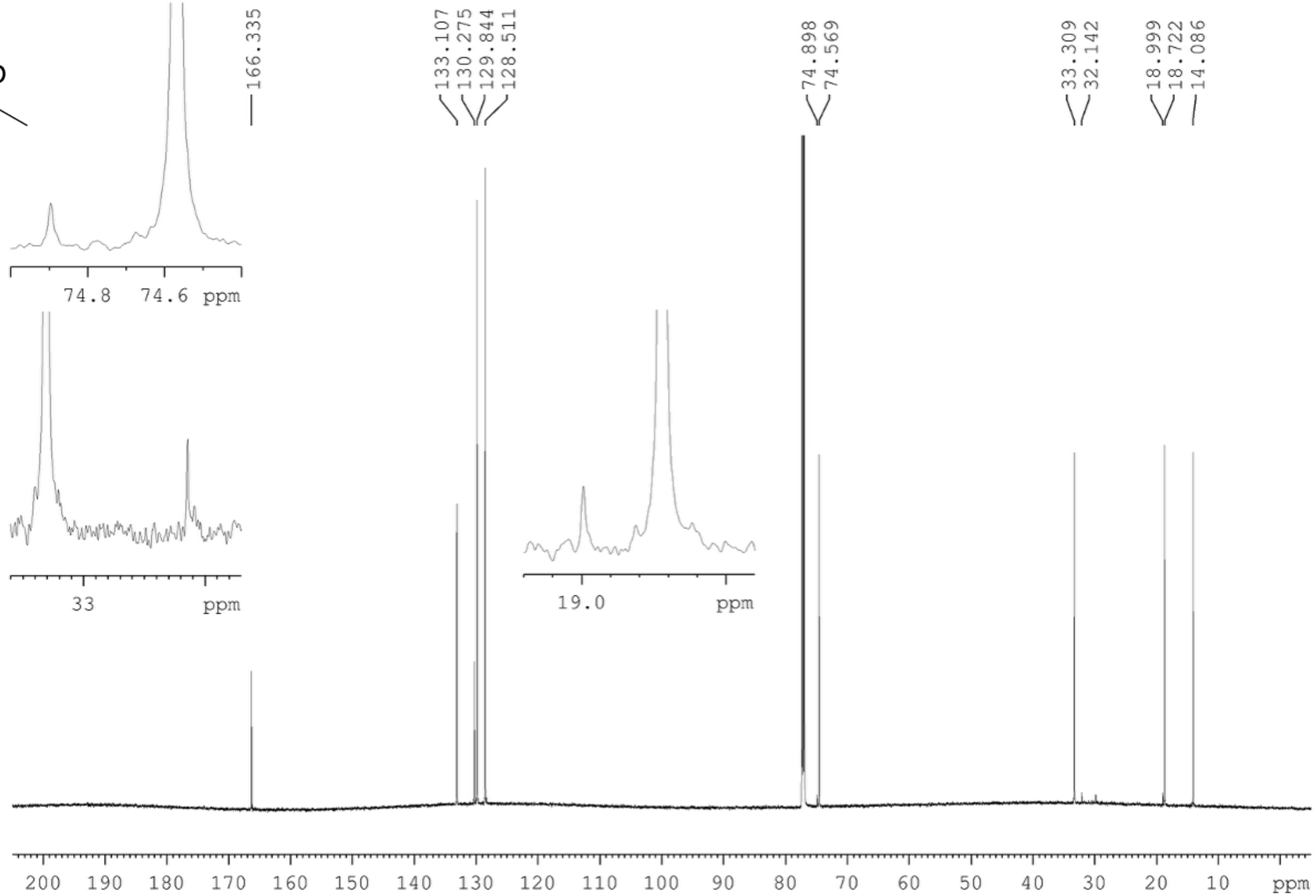

(5*S*,6*S*)-decane-5,6-diol (4d)

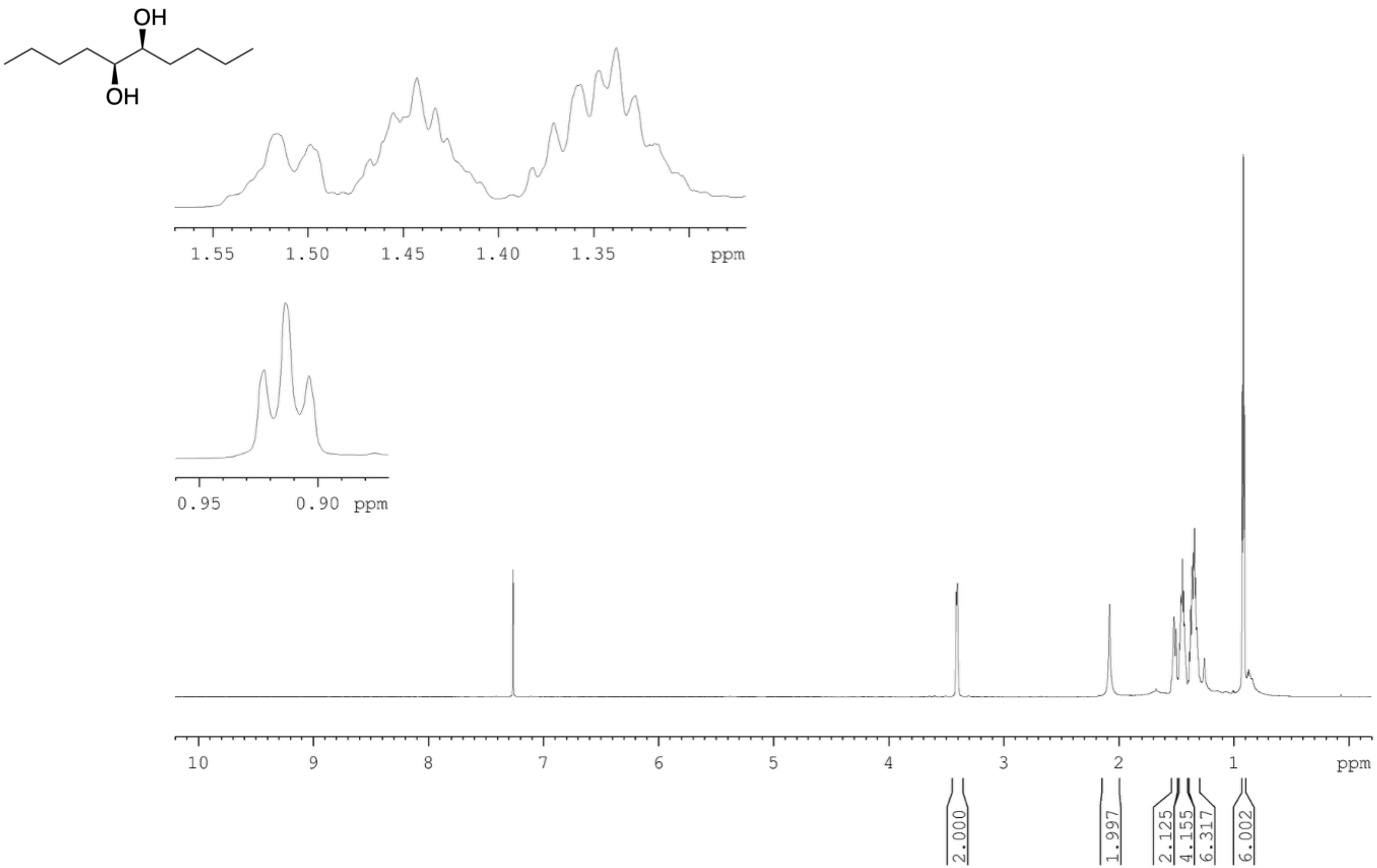

(5*S*,6*S*)-decane-5,6-diol (4d)

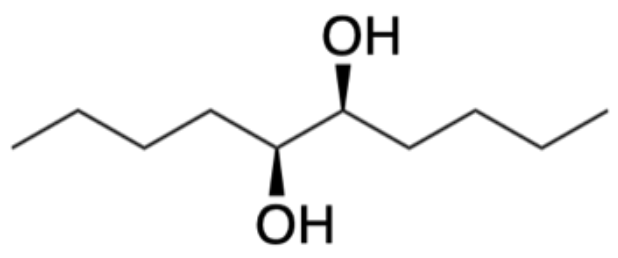

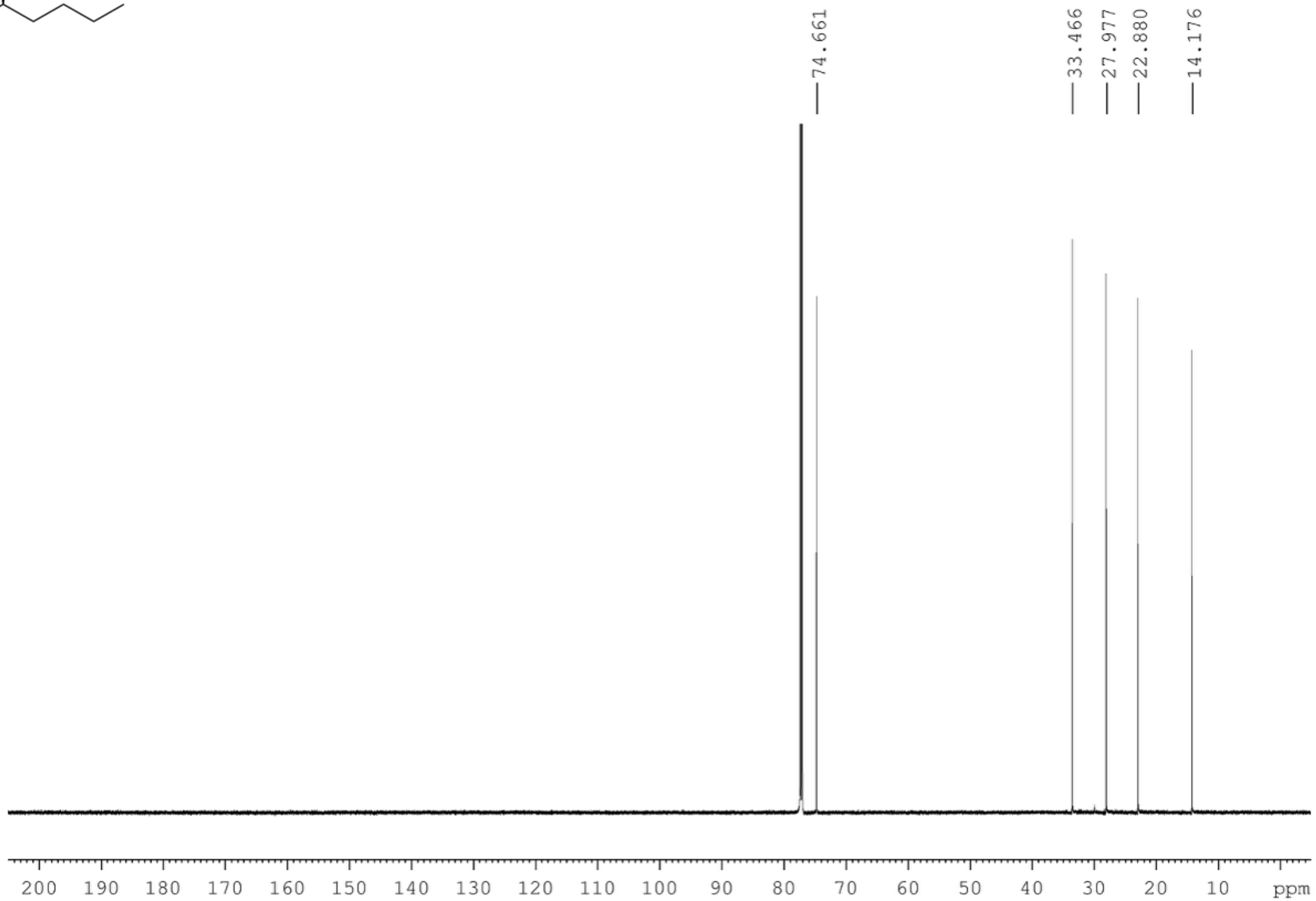

(5*S*,6*S*)-decane-5,6-diyl dibenzoate (4d-Bz)

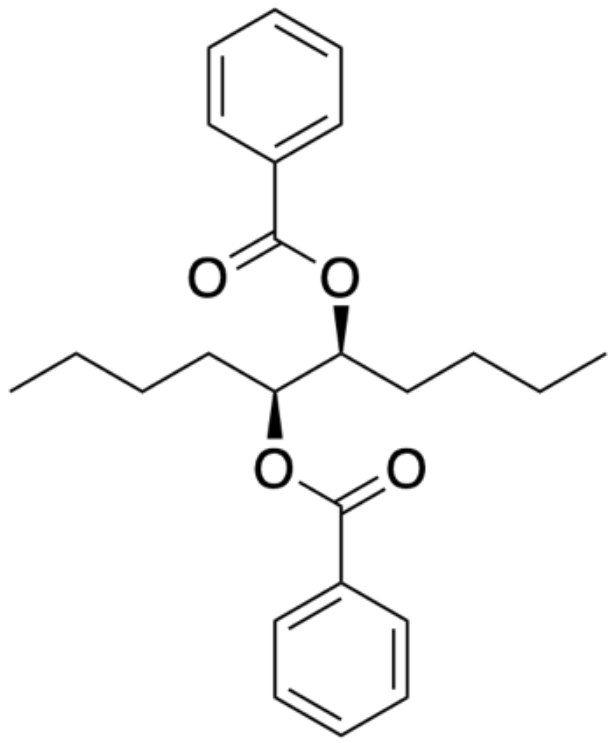

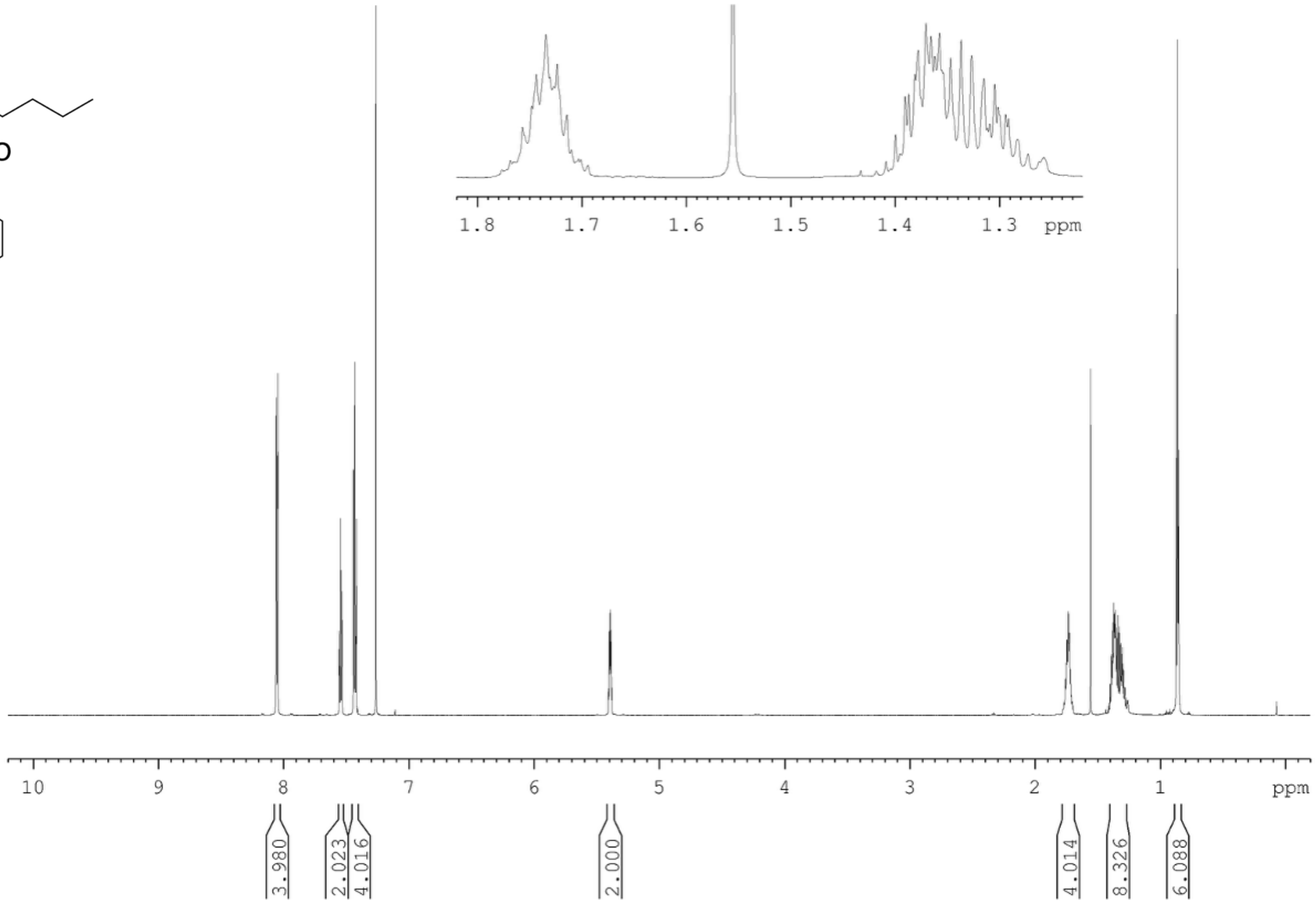

(5*S*,6*S*)-decane-5,6-diyl dibenzoate (4d-Bz)

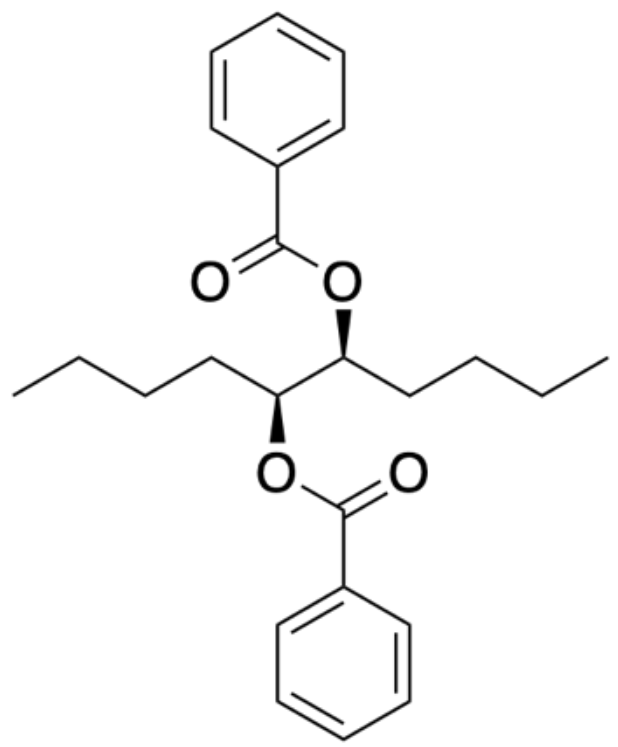

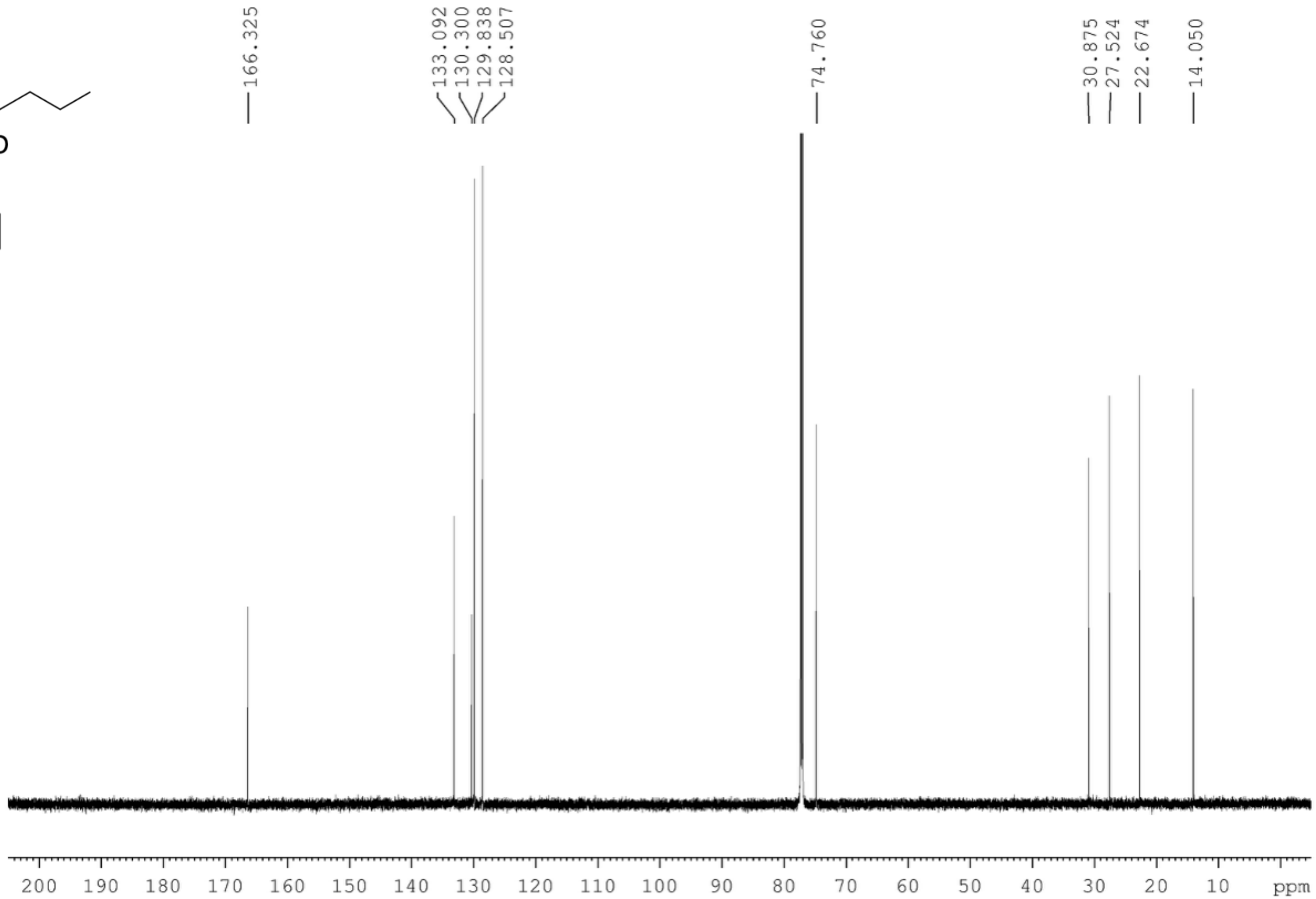

di-*tert*-butyl (4*S*, 5*S*)-4,5-dihydroxyoctanedioate (4e)

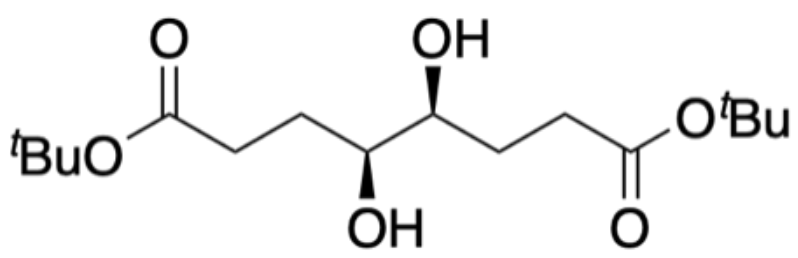

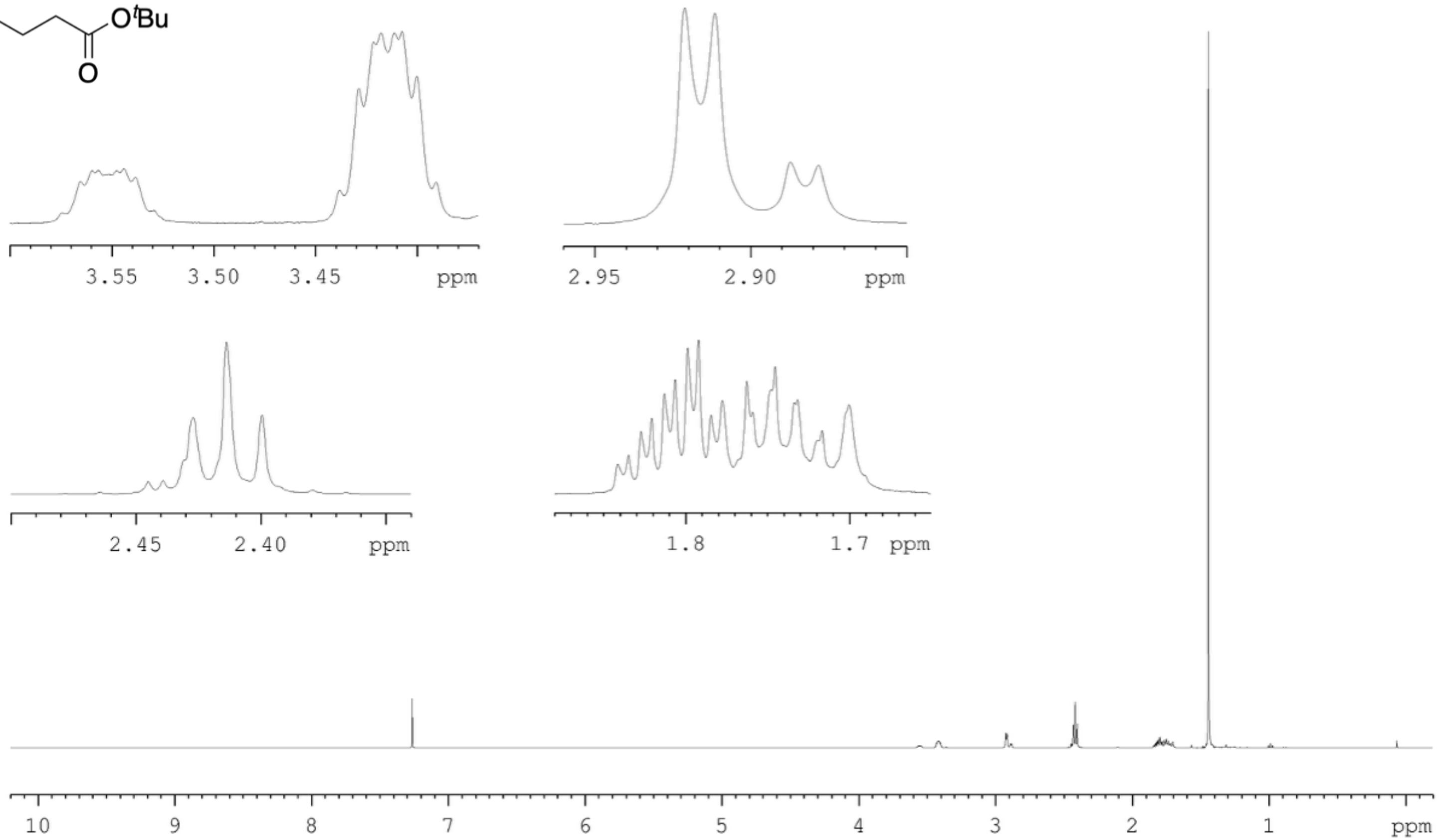

7:2 dr determined using the signals from the protons indicated:

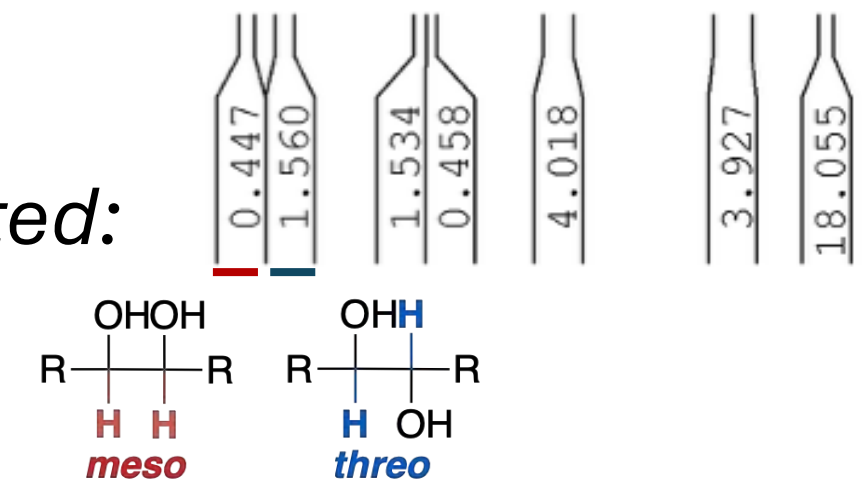

di-*tert*-butyl (4*S*, 5*S*)-4,5-dihydroxyoctanedioate (4e)

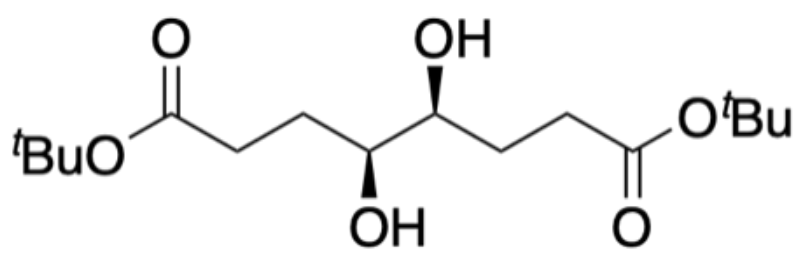

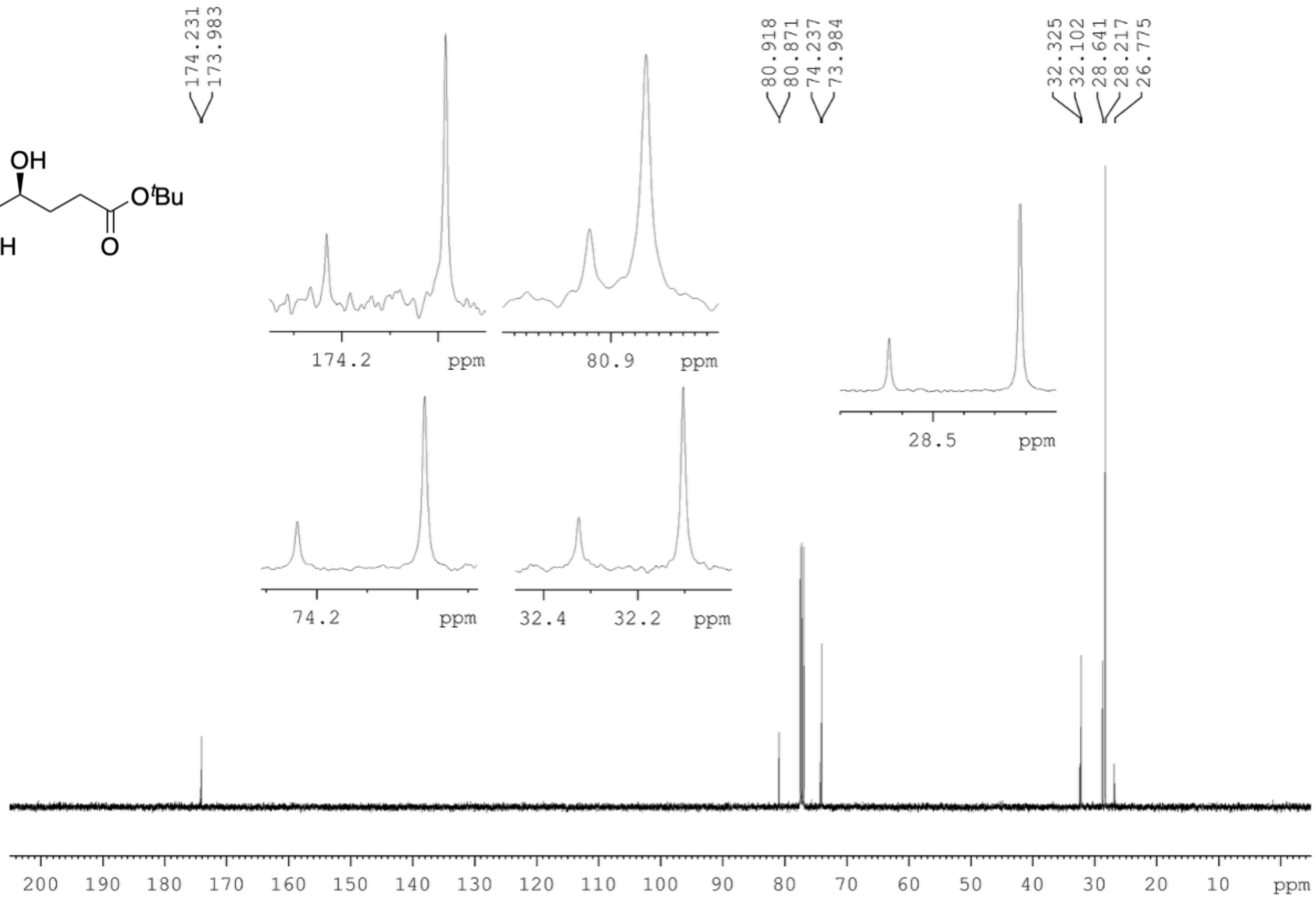

di-*tert*-butyl (4*S*,5*S*)-4,5-bis(benzoyloxy)octanedioate (4e-Bz)

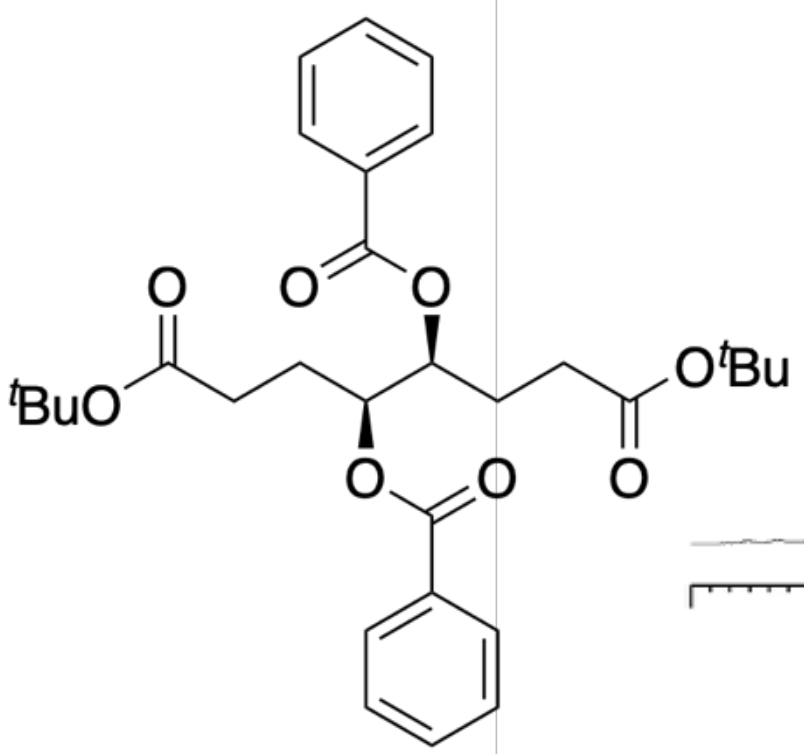

7:2 dr determined using the signals from the protons indicated:

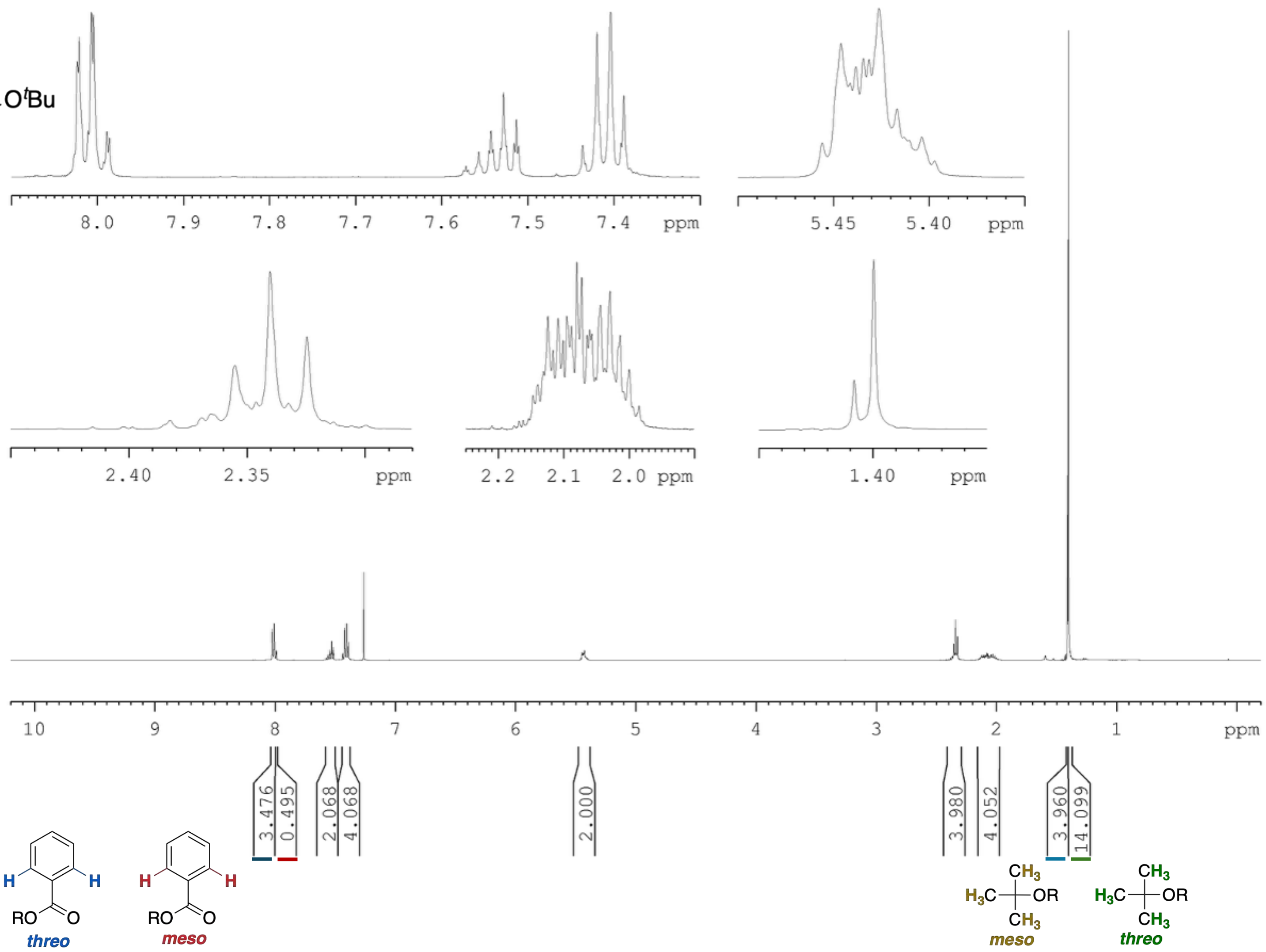

di-*tert*-butyl (4*S*,5*S*)-4,5-bis(benzoyloxy)octanedioate (4e-Bz)

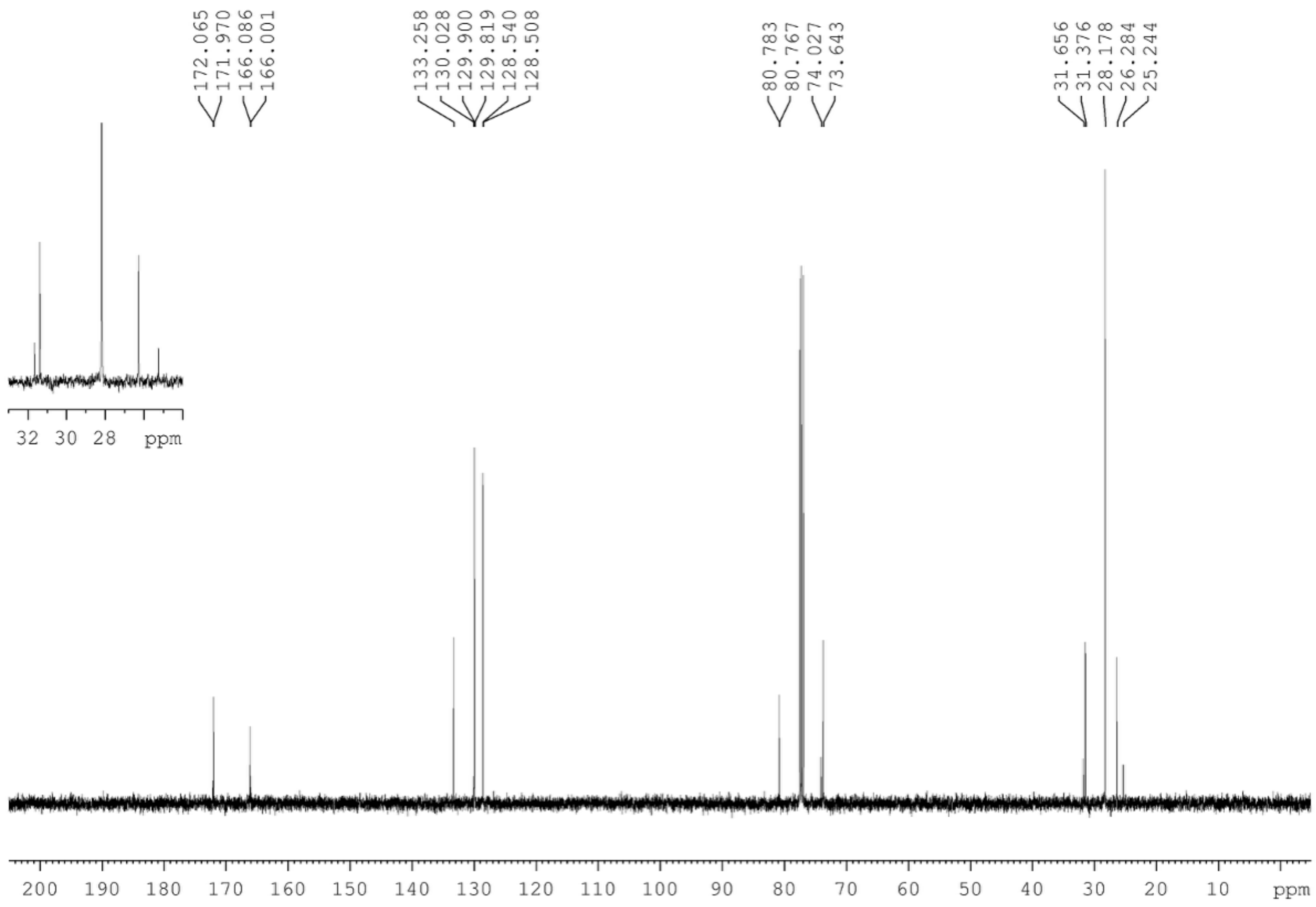

(2*S*,3*S*)-1,4-diphenylbutane-2,3-diol (4f)

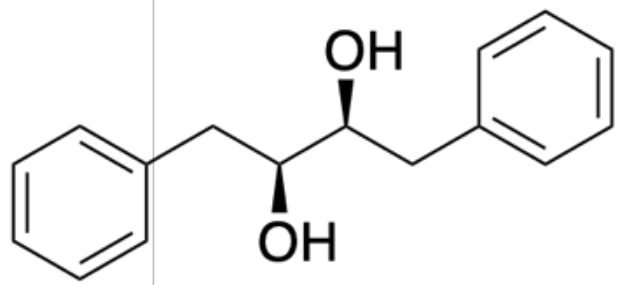

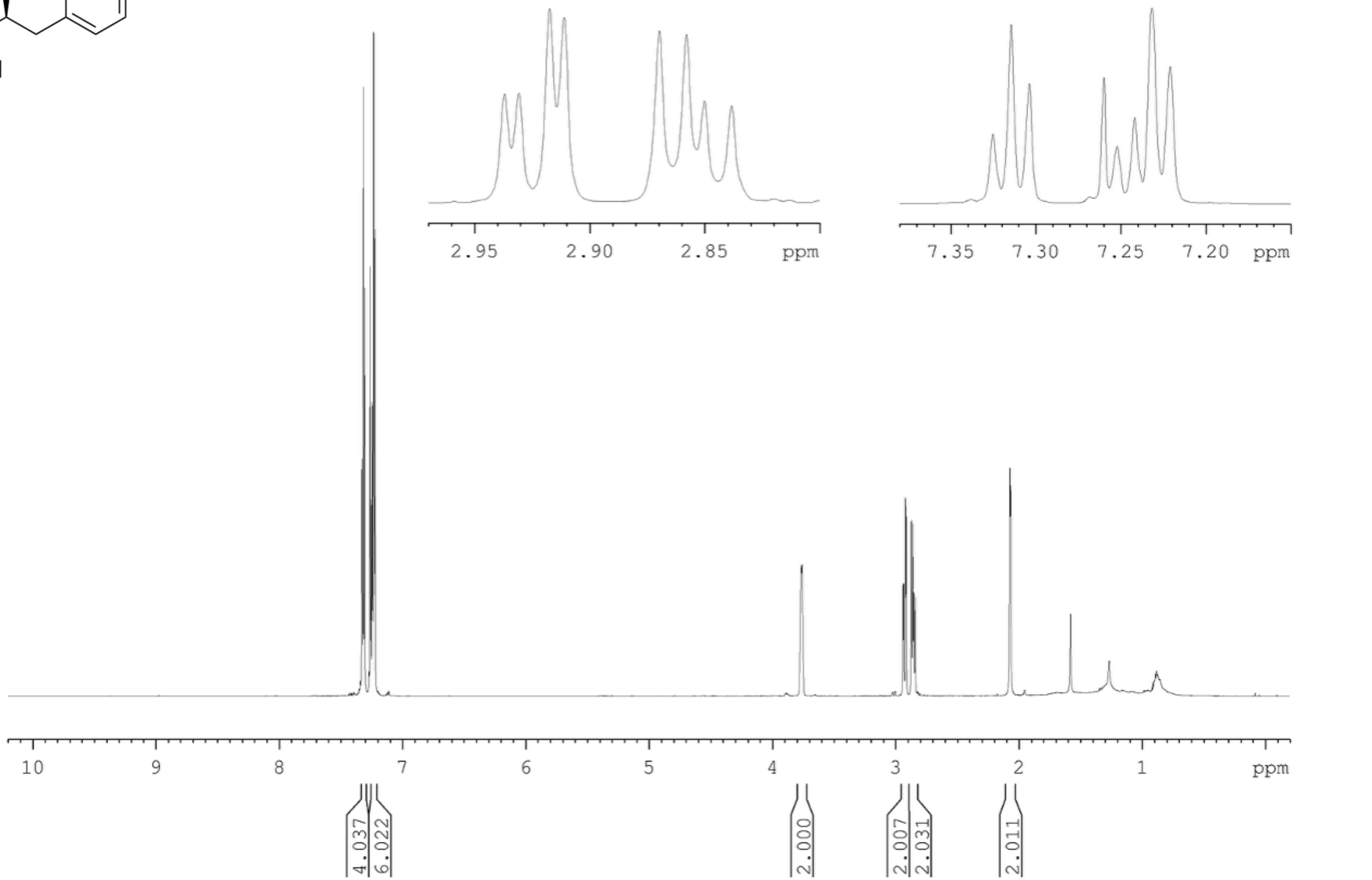

(2*S*,3*S*)-1,4-diphenylbutane-2,3-diol (4f)

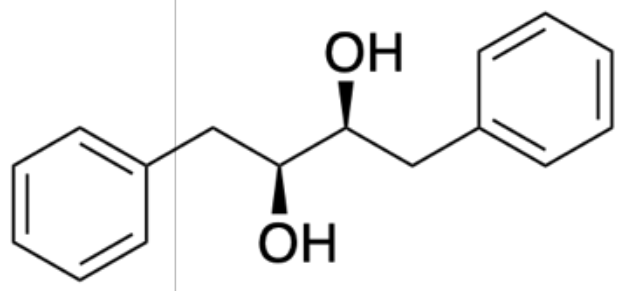

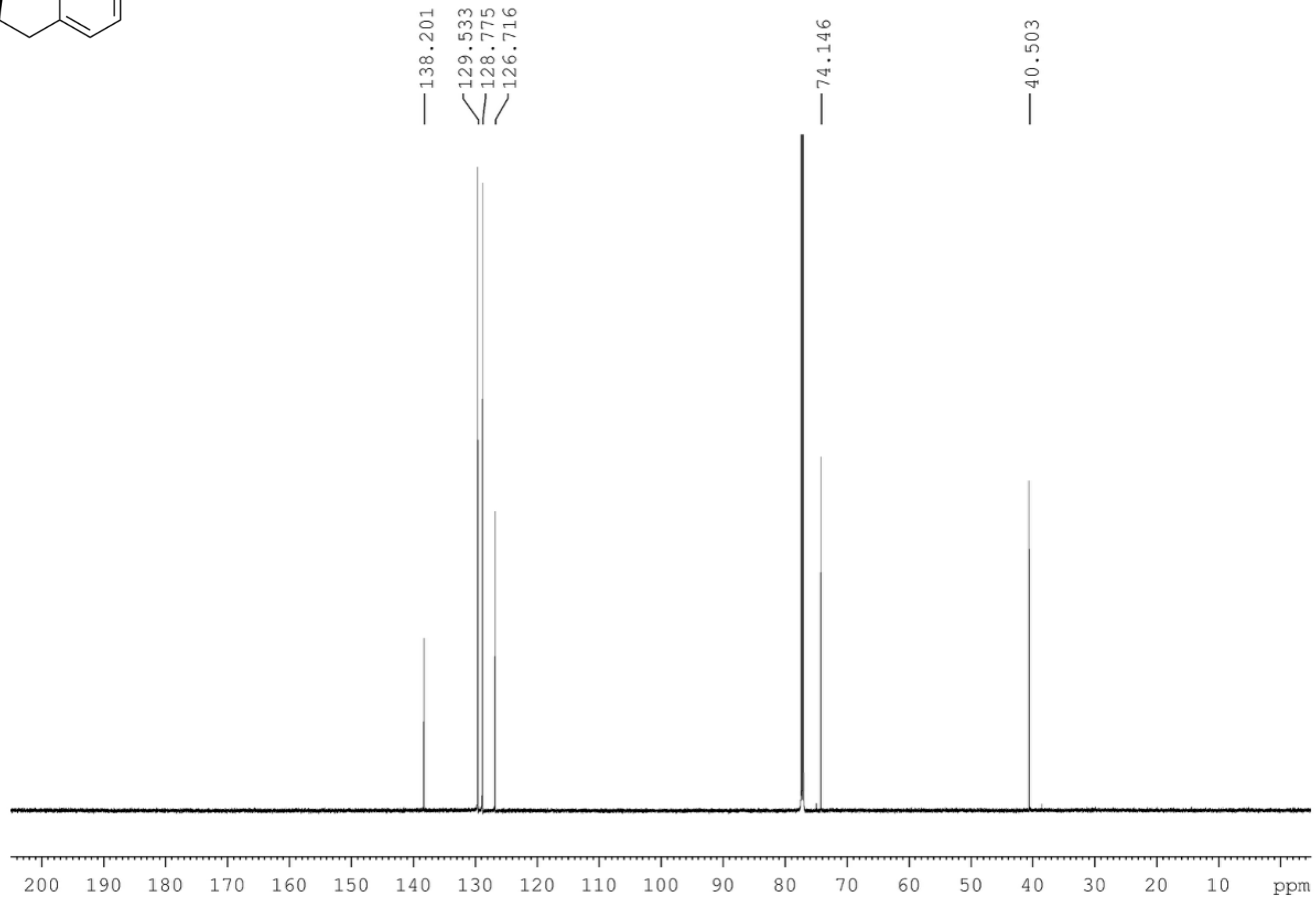

(4*S*,5*S*)-1,8-diphenyloctane-4,5-diol (4g)

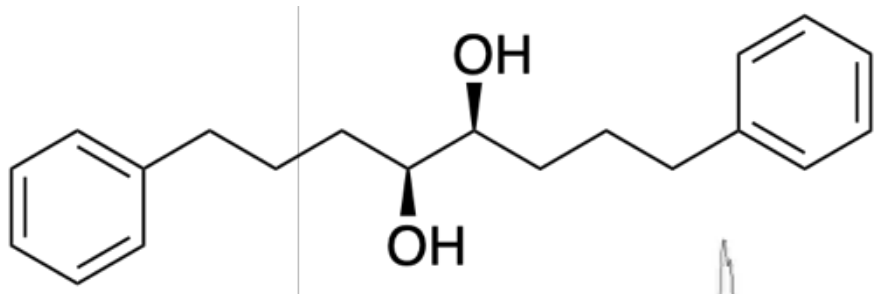

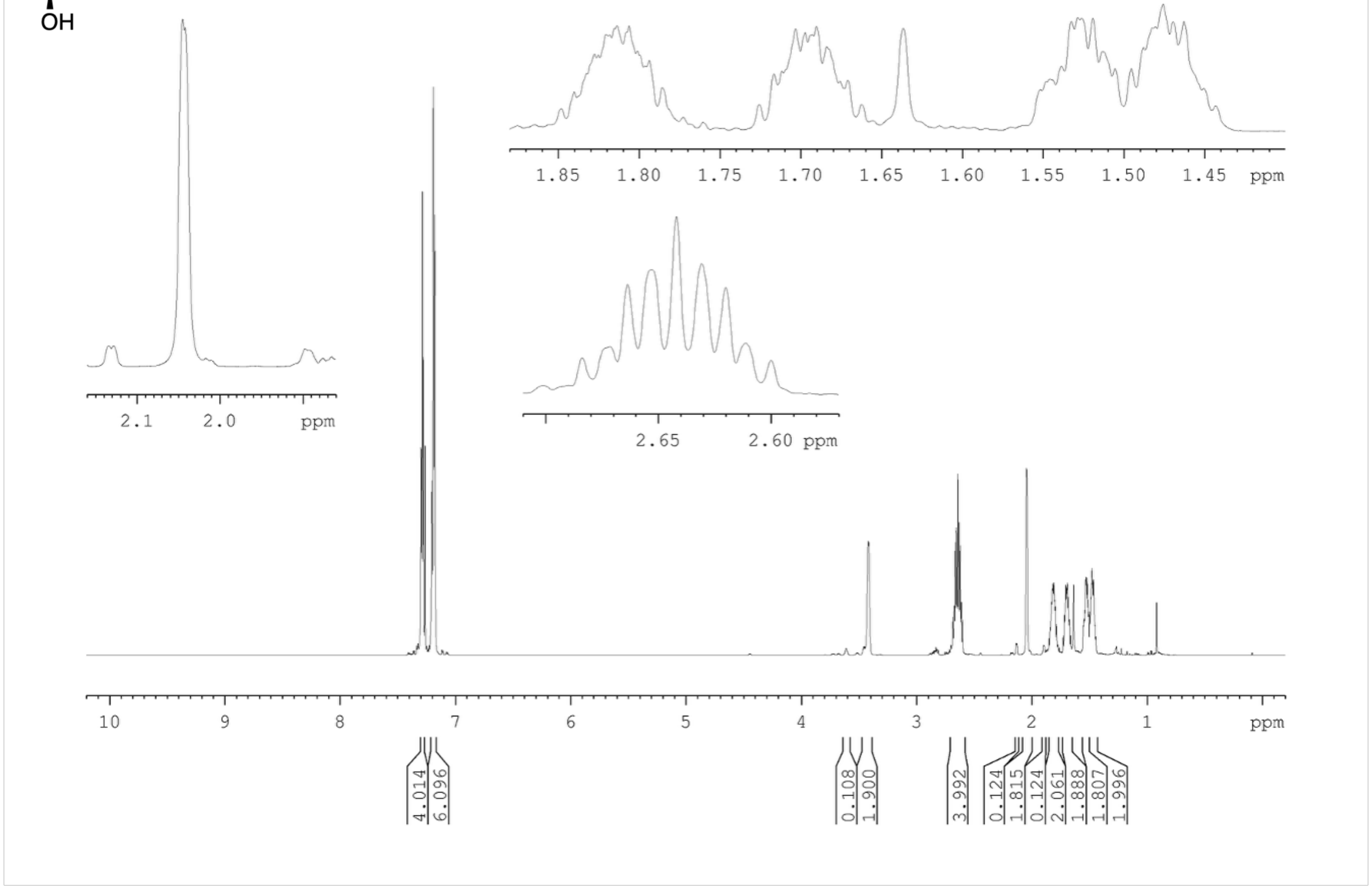

(4*S*,5*S*)-1,8-diphenyloctane-4,5-diol (4g)

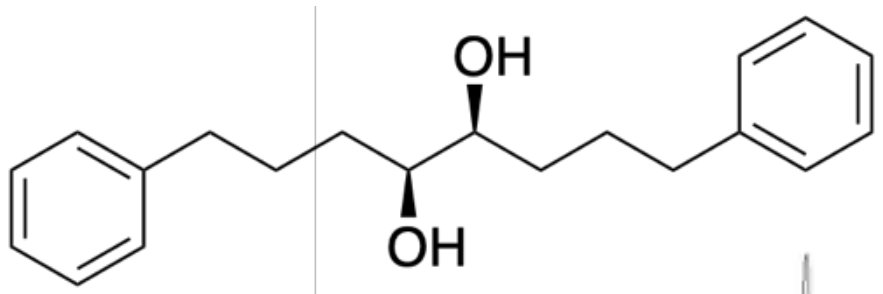

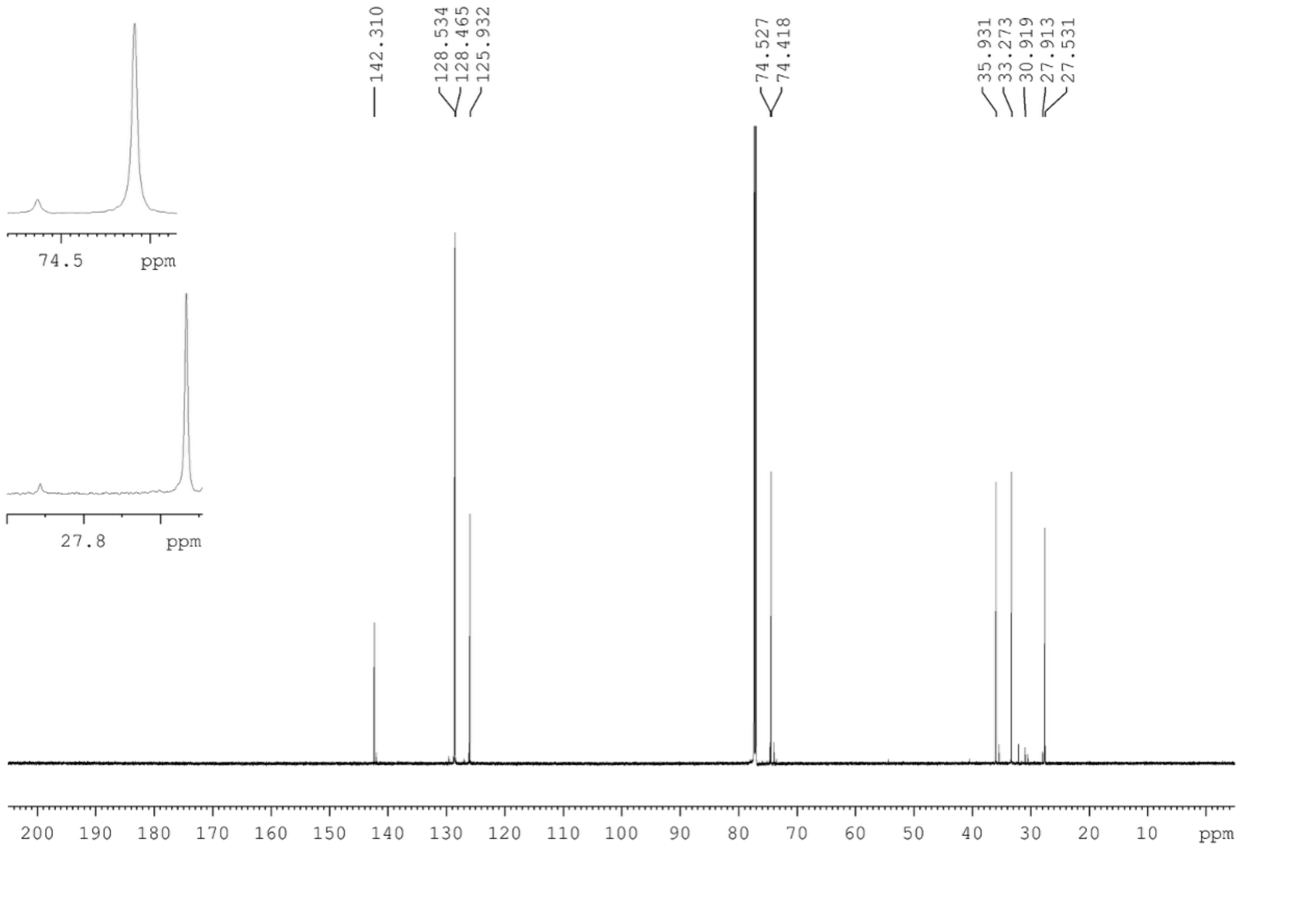

Enriched 5-Phenylpentane-2,3-diol (4h) starting from *syn*

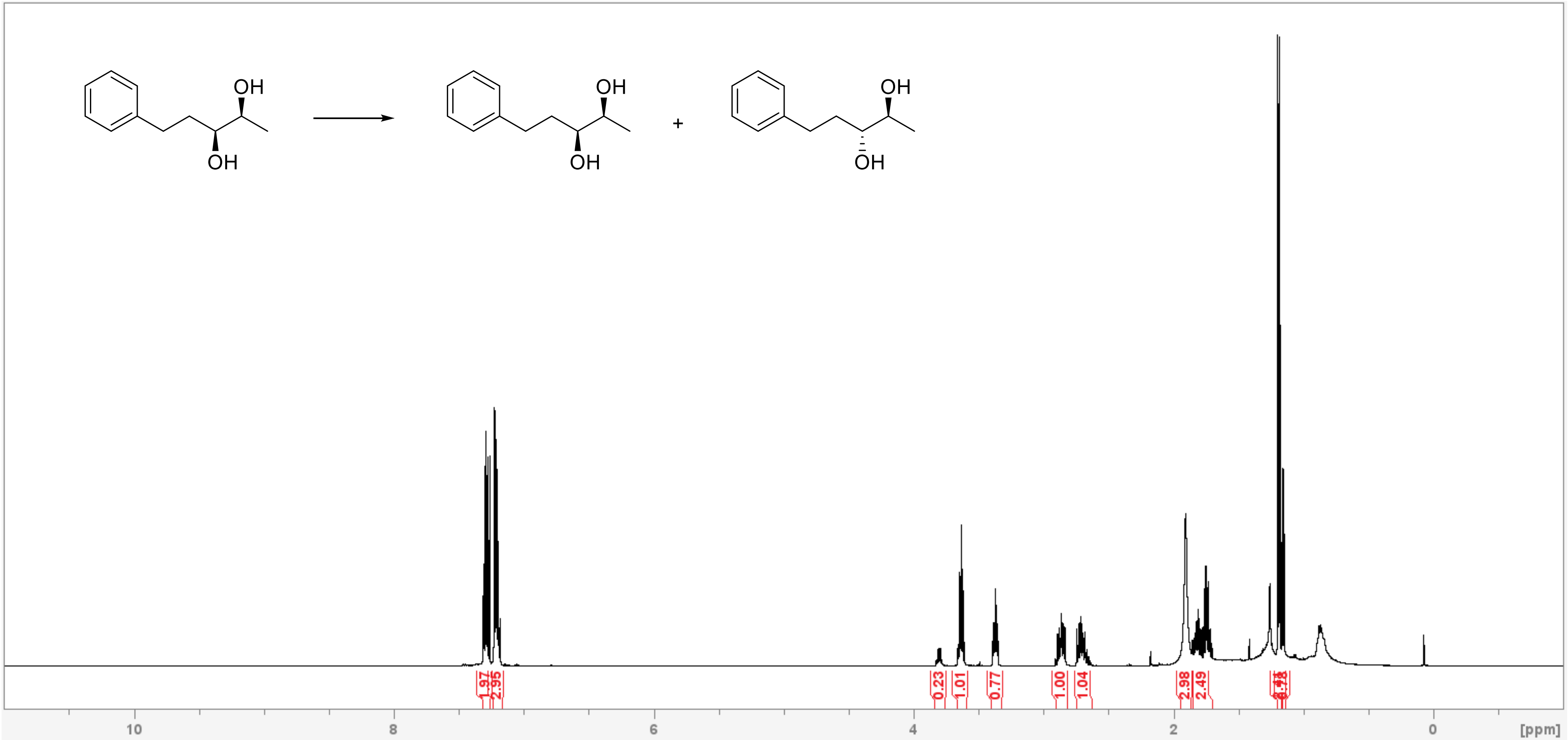

Enriched (*anti*)-5-Phenylpentane-2,3-diol (*anti*-4h)

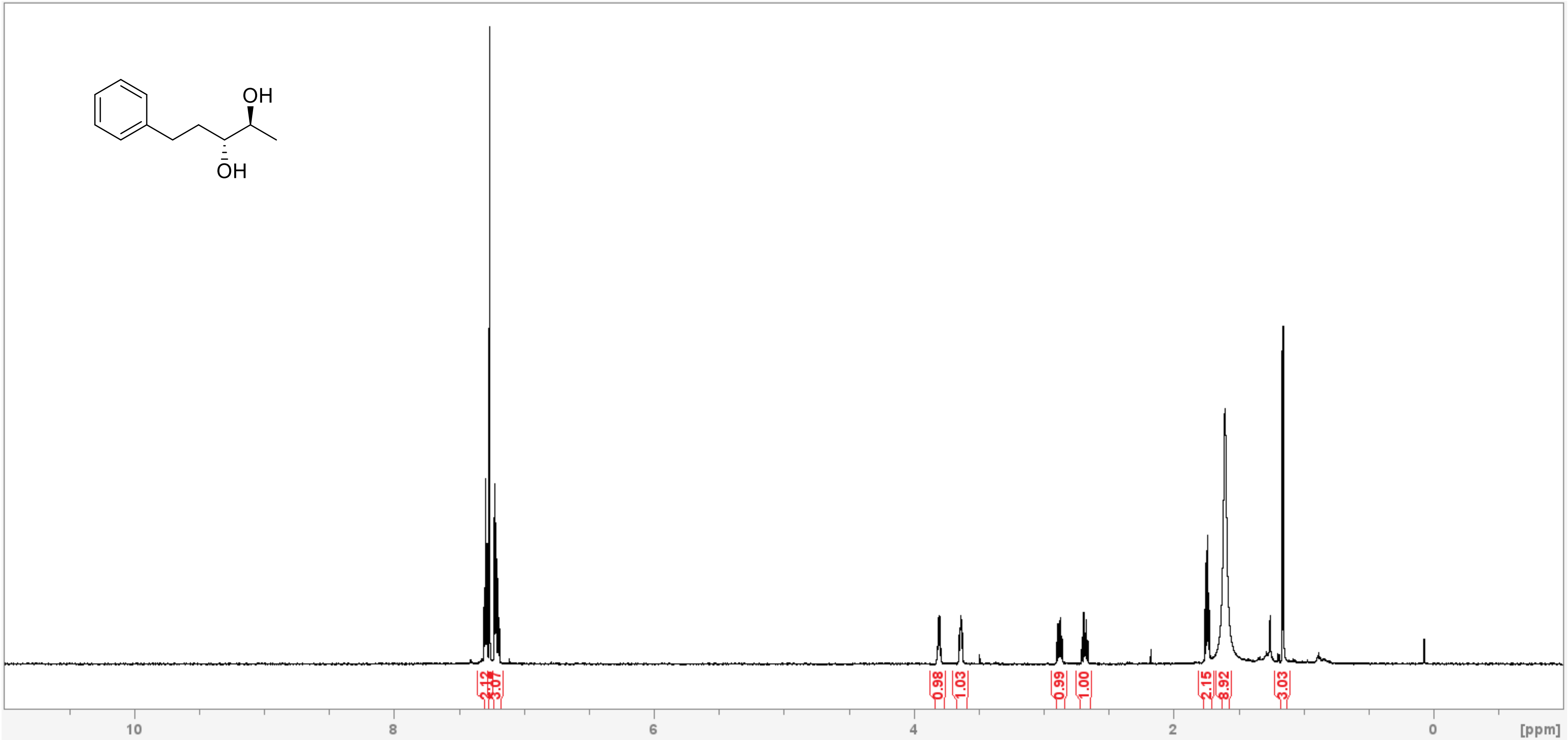

Enriched (*anti*)-5-Phenylpentane-2,3-diol (*anti*-4h)

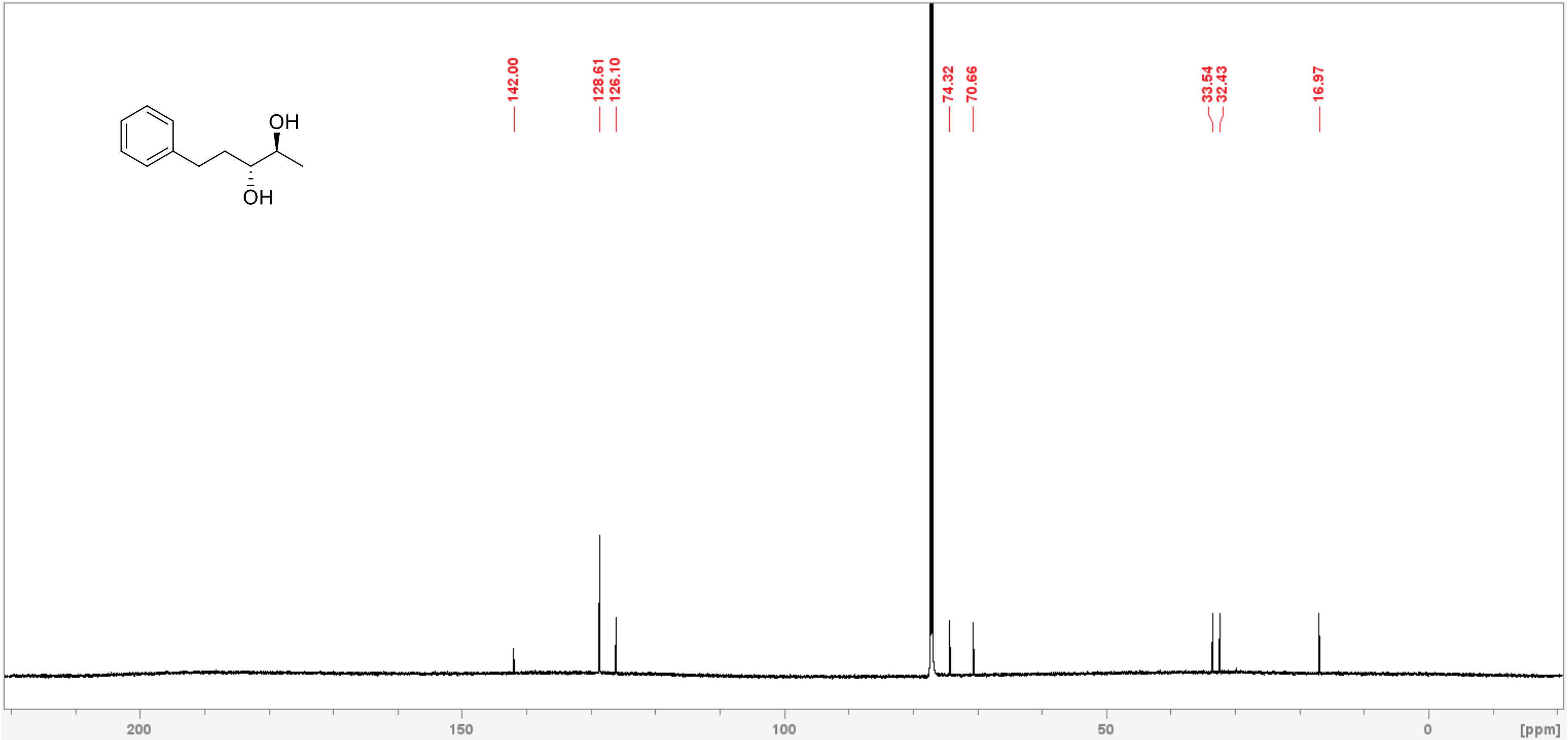

Enriched (*syn*)-5-Phenylpentane-2,3-diol (*syn*-4h)

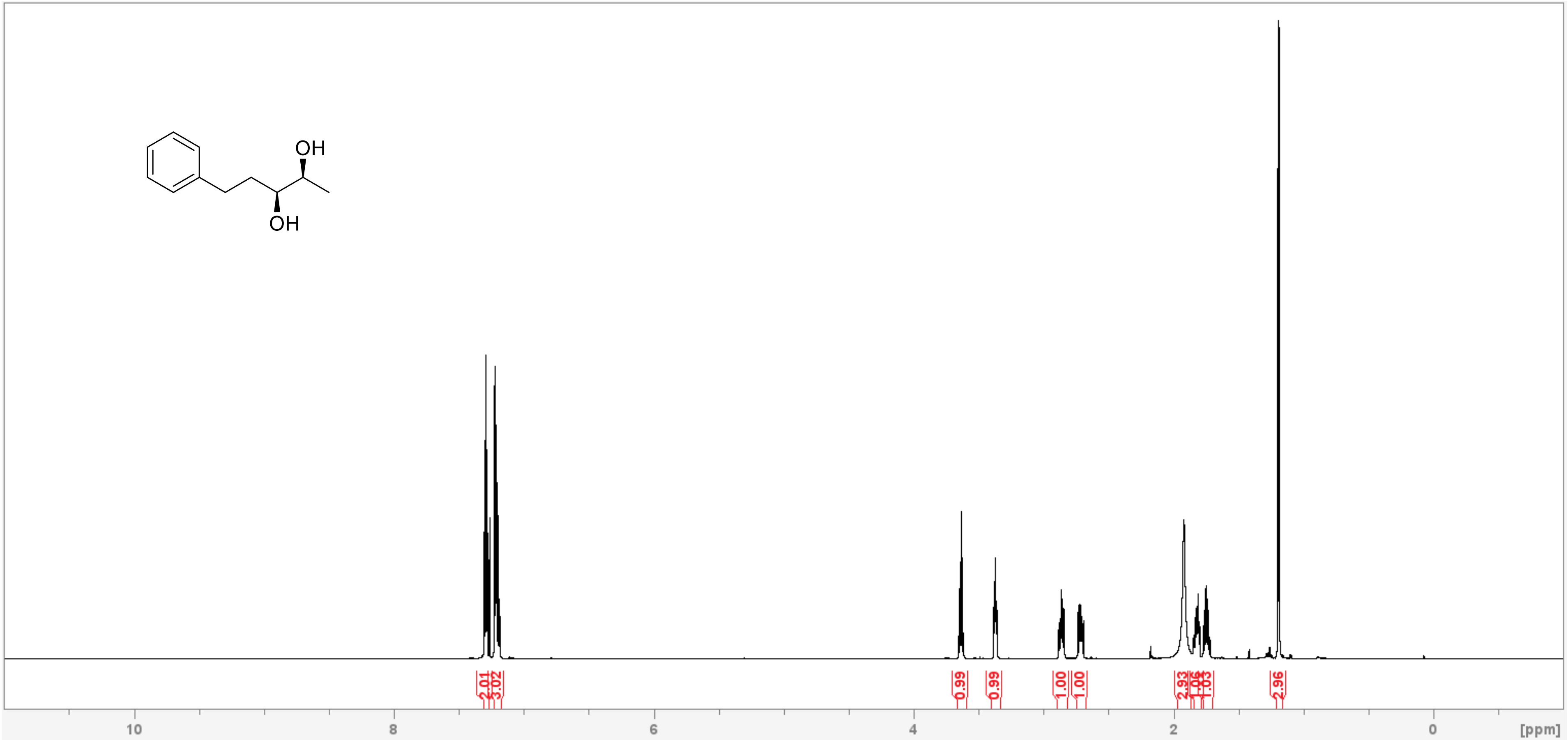

Enriched (*syn*)-5-Phenylpentane-2,3-diol (*syn*-4h)

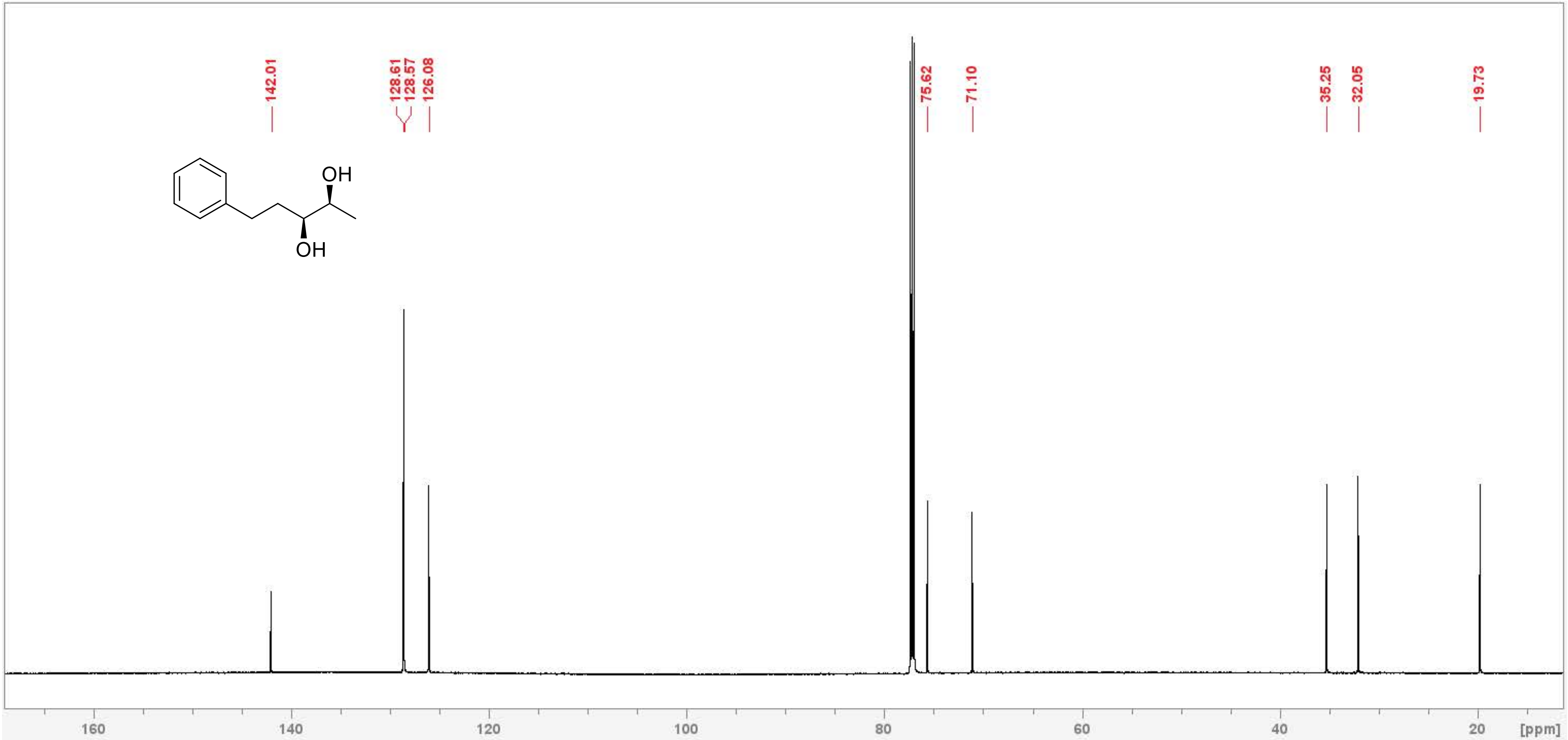

Enriched 1-Phenylbutane-2,3-diol (4i) starting from *anti*

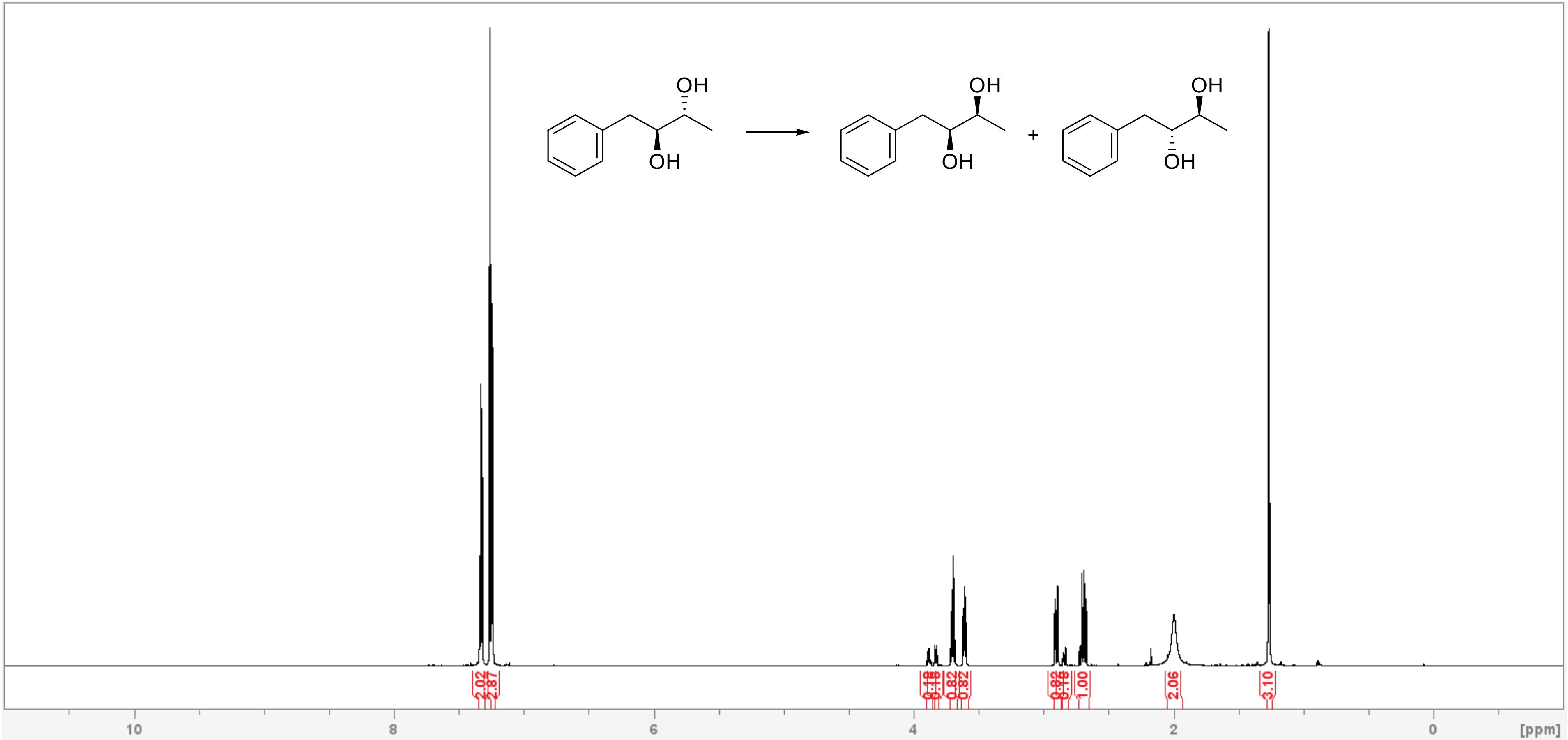

Enriched 1-Phenylheptane-3,4-diol (4j) starting from *anti*

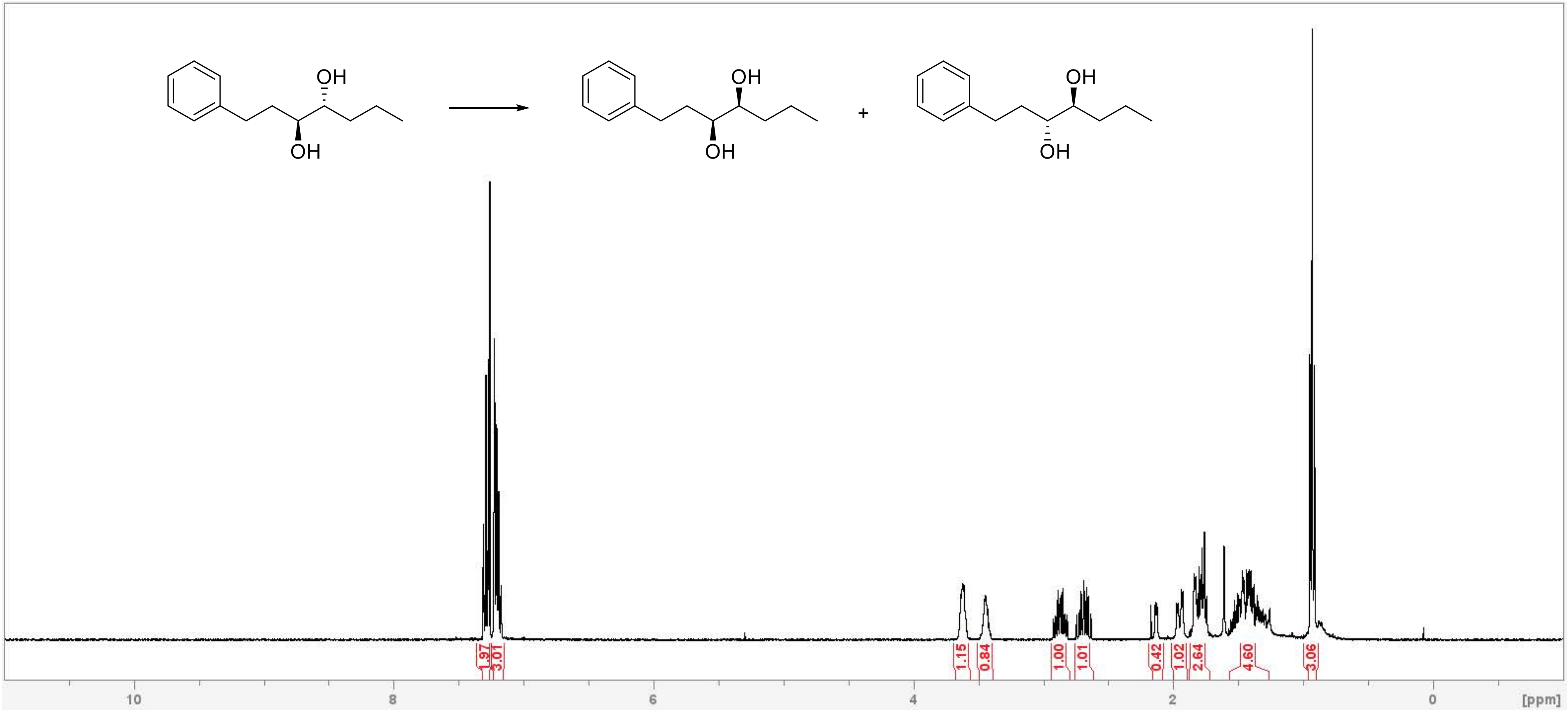

Enriched (*anti*)-1,1-Diphenylbutane-2,3-diol (*anti*-4k)

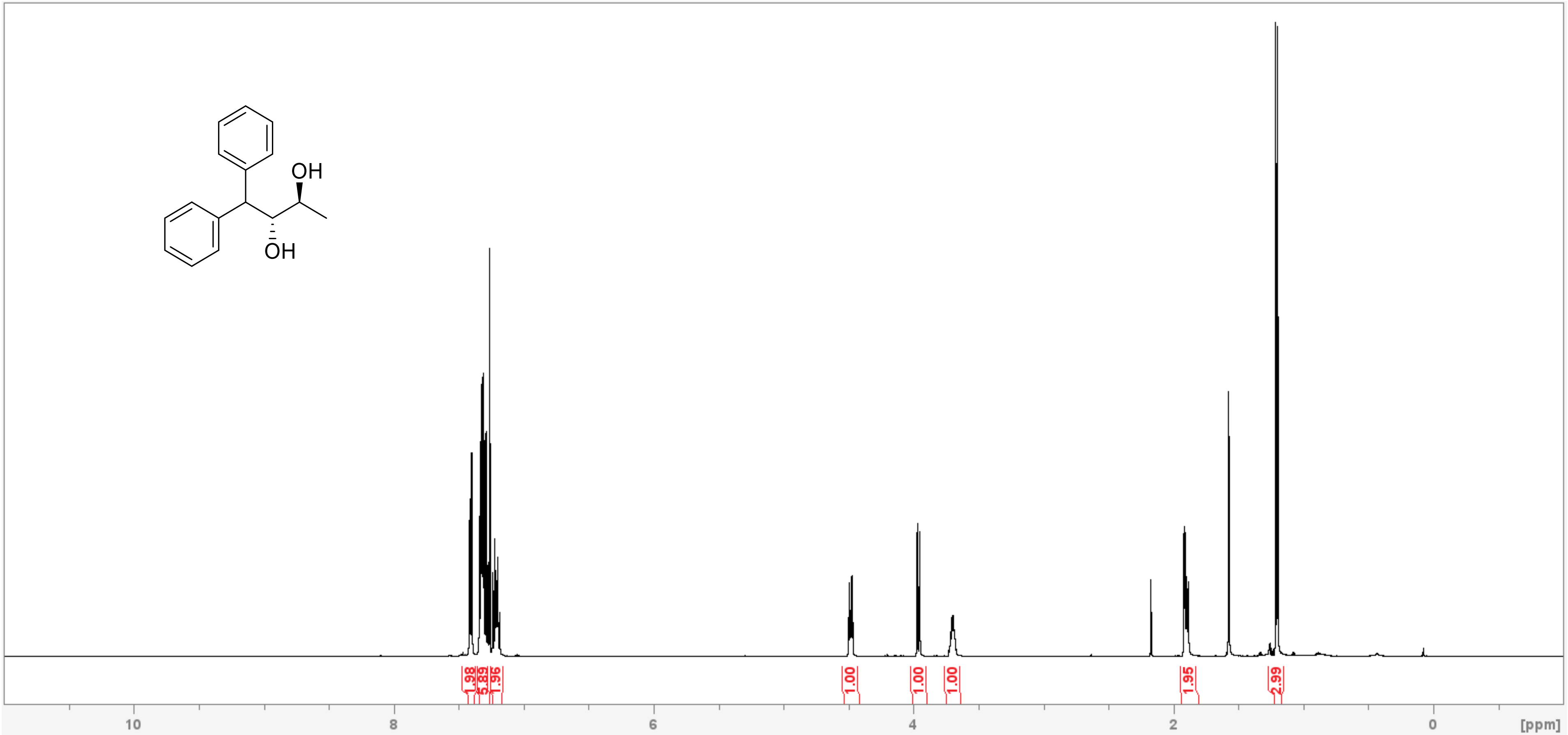

Enriched (*anti*)-1,1-Diphenylbutane-2,3-diol (*anti*-4k)

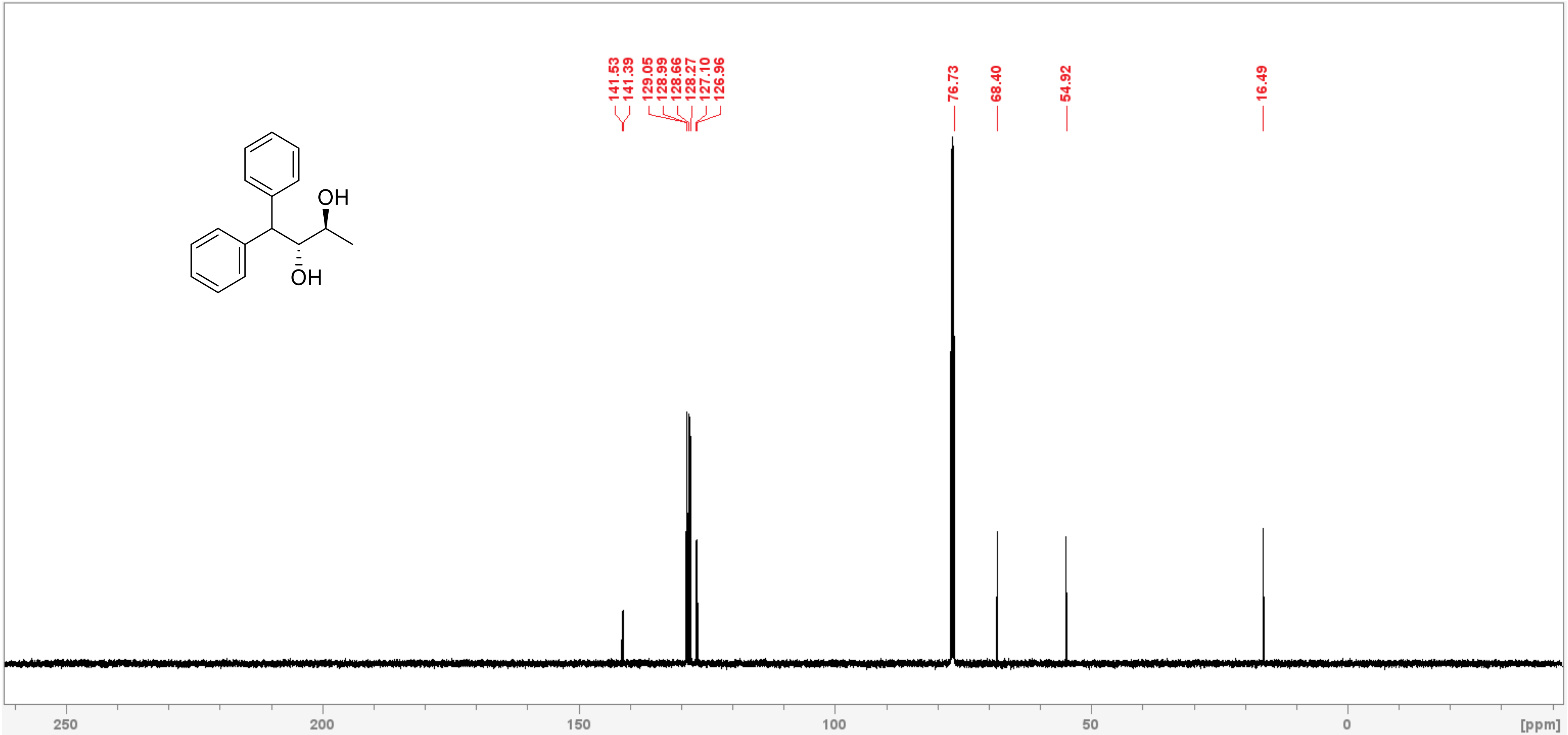

Enriched 4,5-Dihydroxyhexyl benzoate (4l) starting from *syn*

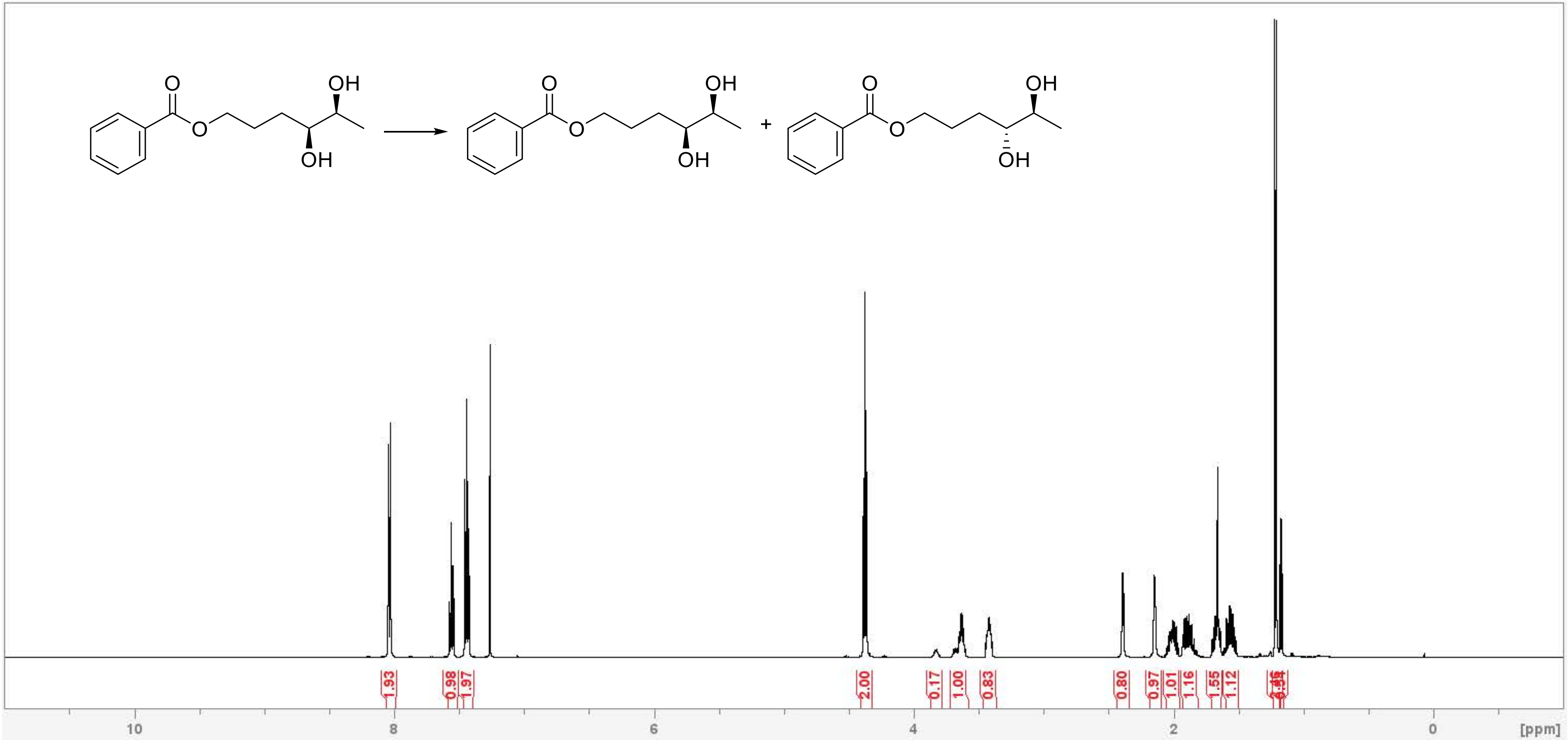

Enriched 4,5-Dihydroxyhexyl benzoate (4l) starting from *anti*

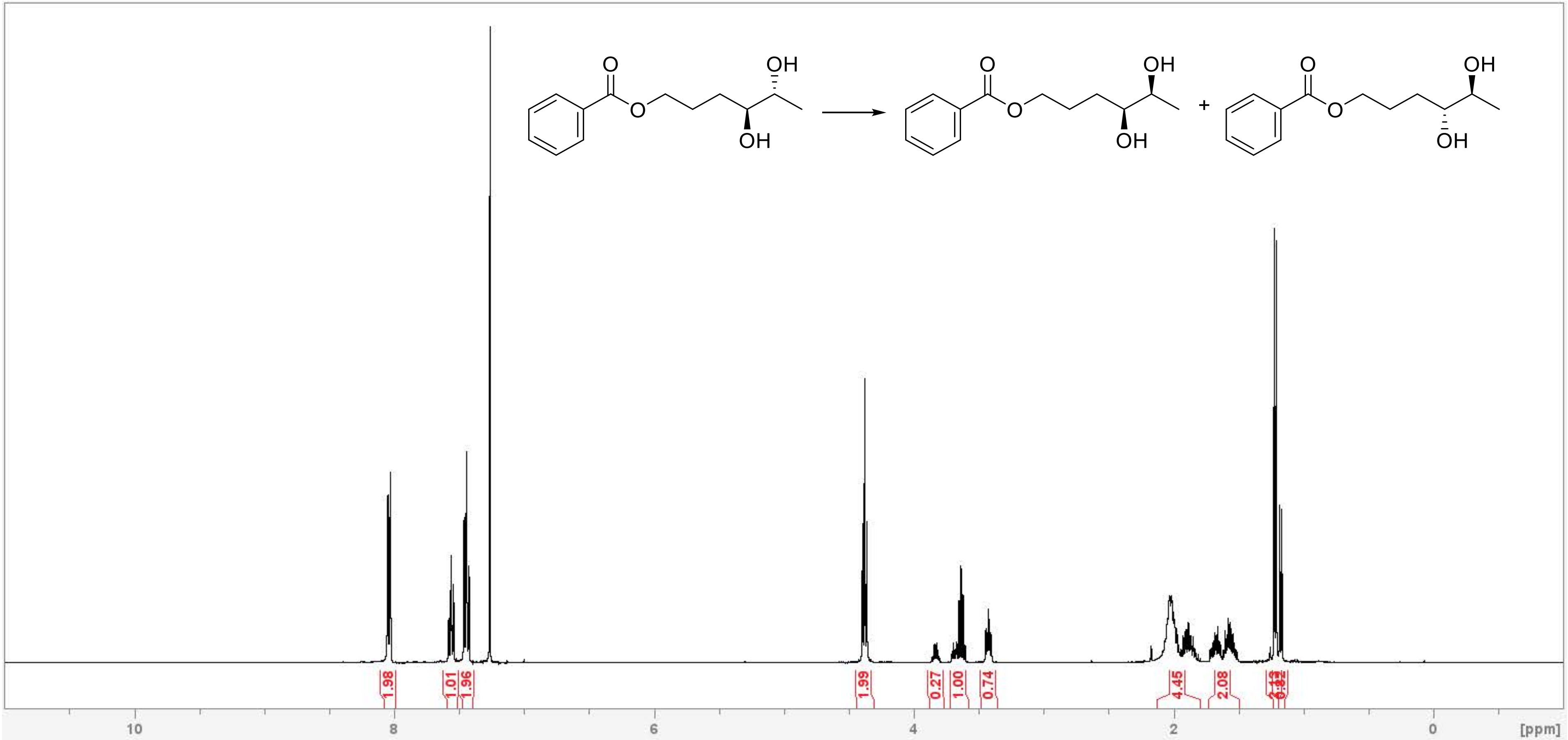

Enriched 4,5-Dihydroxyhexyl diphenylphosphinate (4m) starting from *syn*

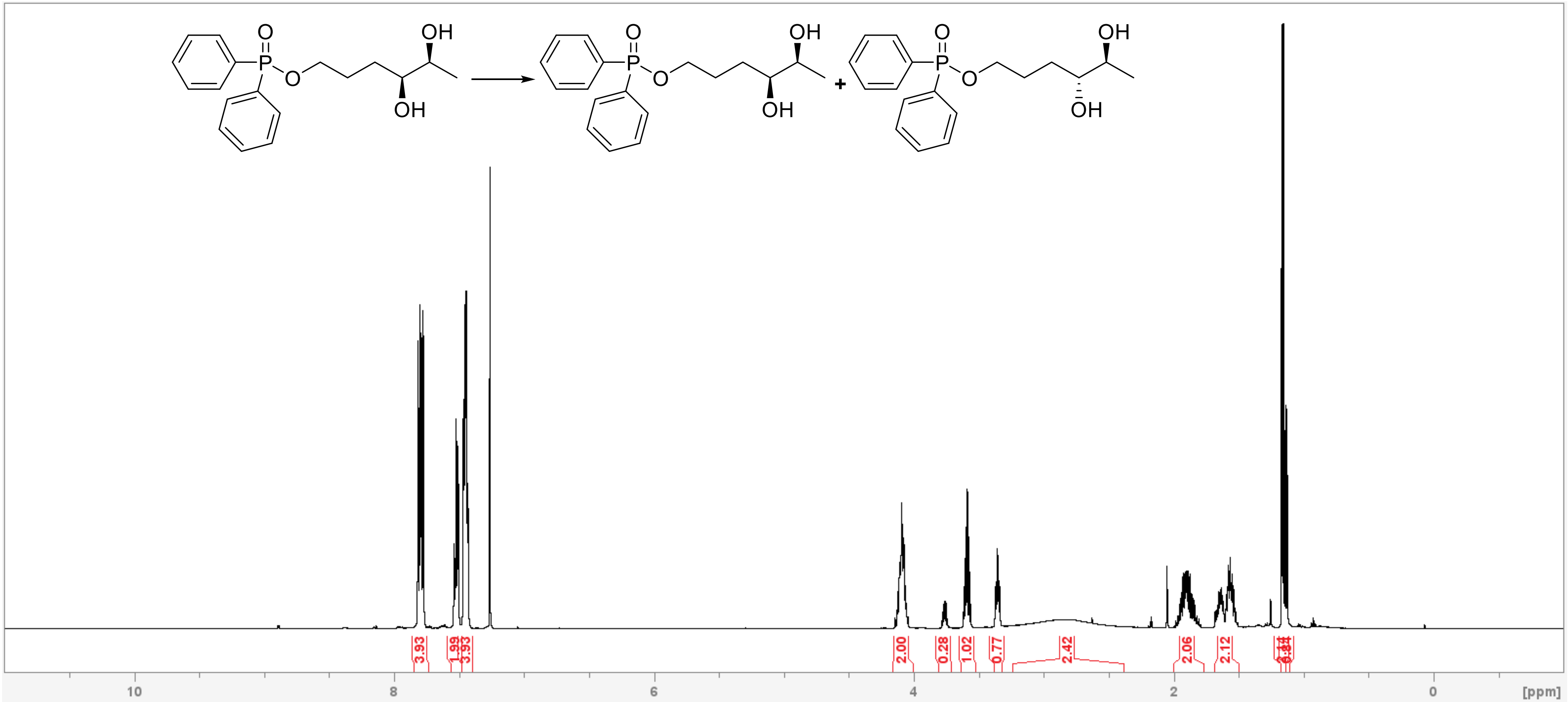

Enriched 4,5-Dihydroxyhexyl diphenylphosphinate (4m) starting from *anti*

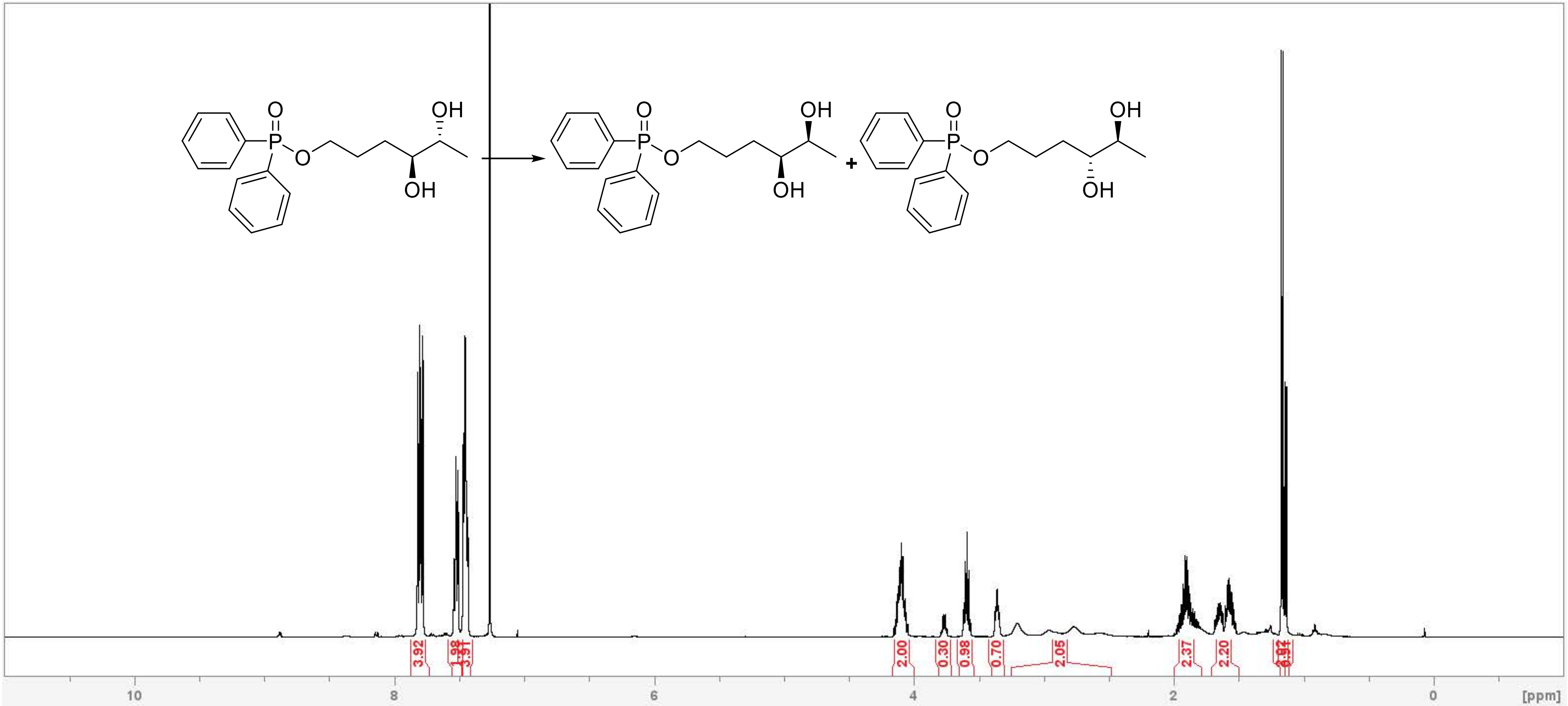

methyl 3-oxo-4-phenylbutanoate (*int*$_1$-3a)

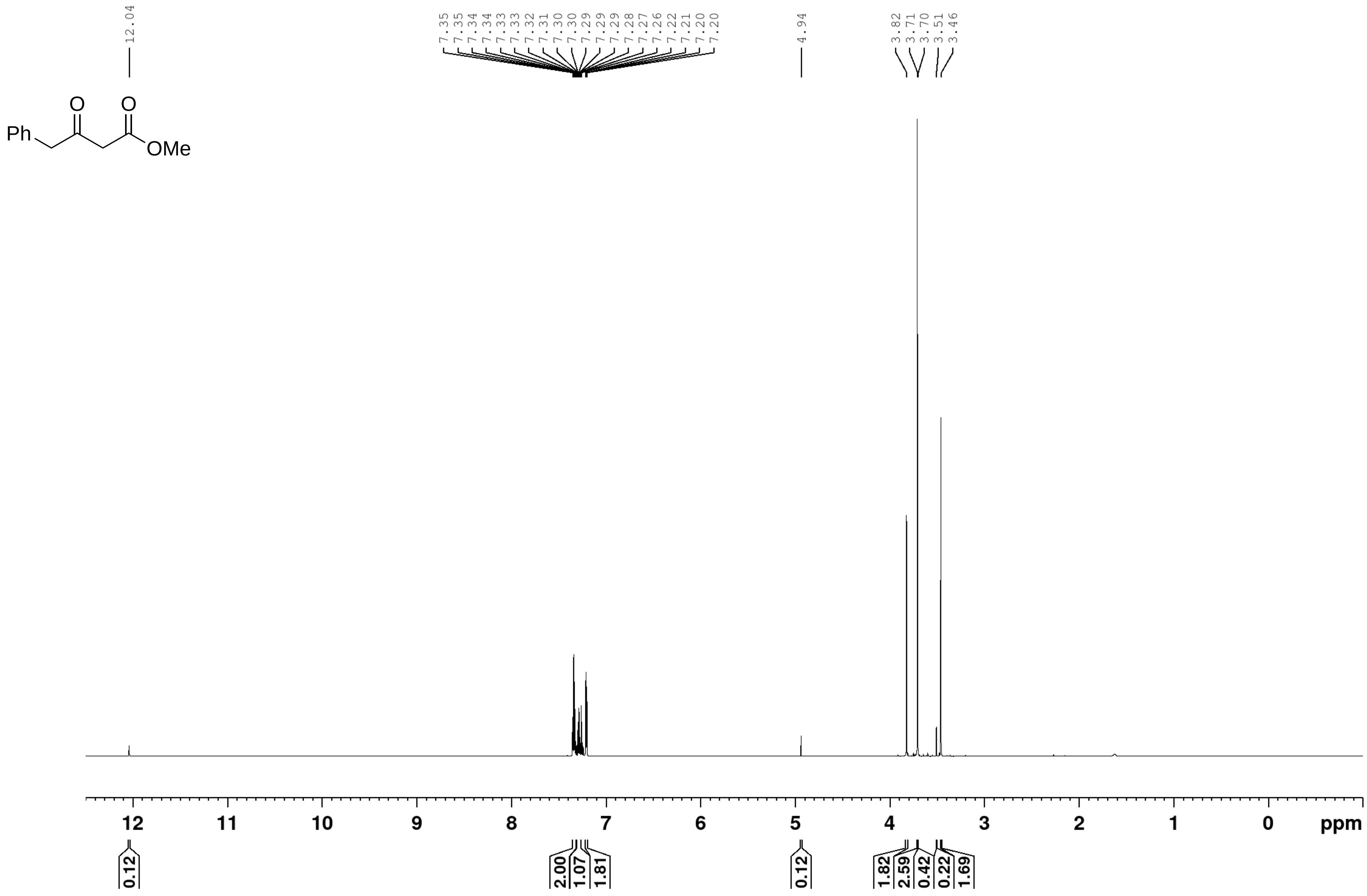

methyl 3-oxo-4-phenylbutanoate (*int₁*-3a)

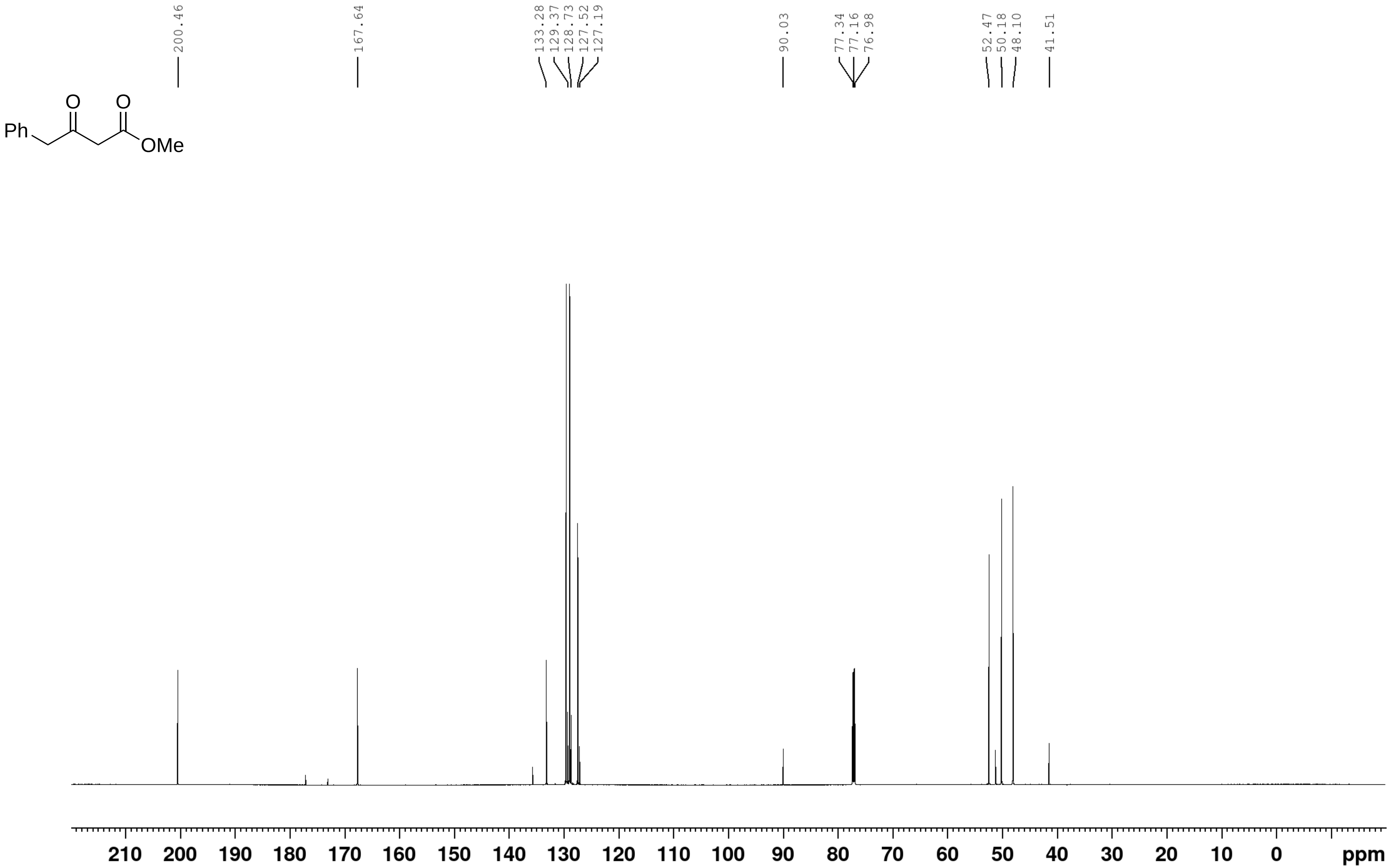

methyl (*R*)-3-hydroxy-4-phenylbutanoate (*int*$_2$-3a)

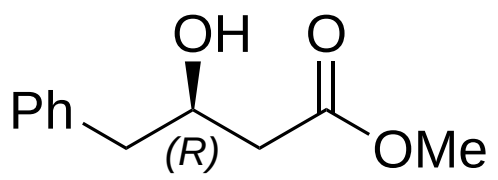

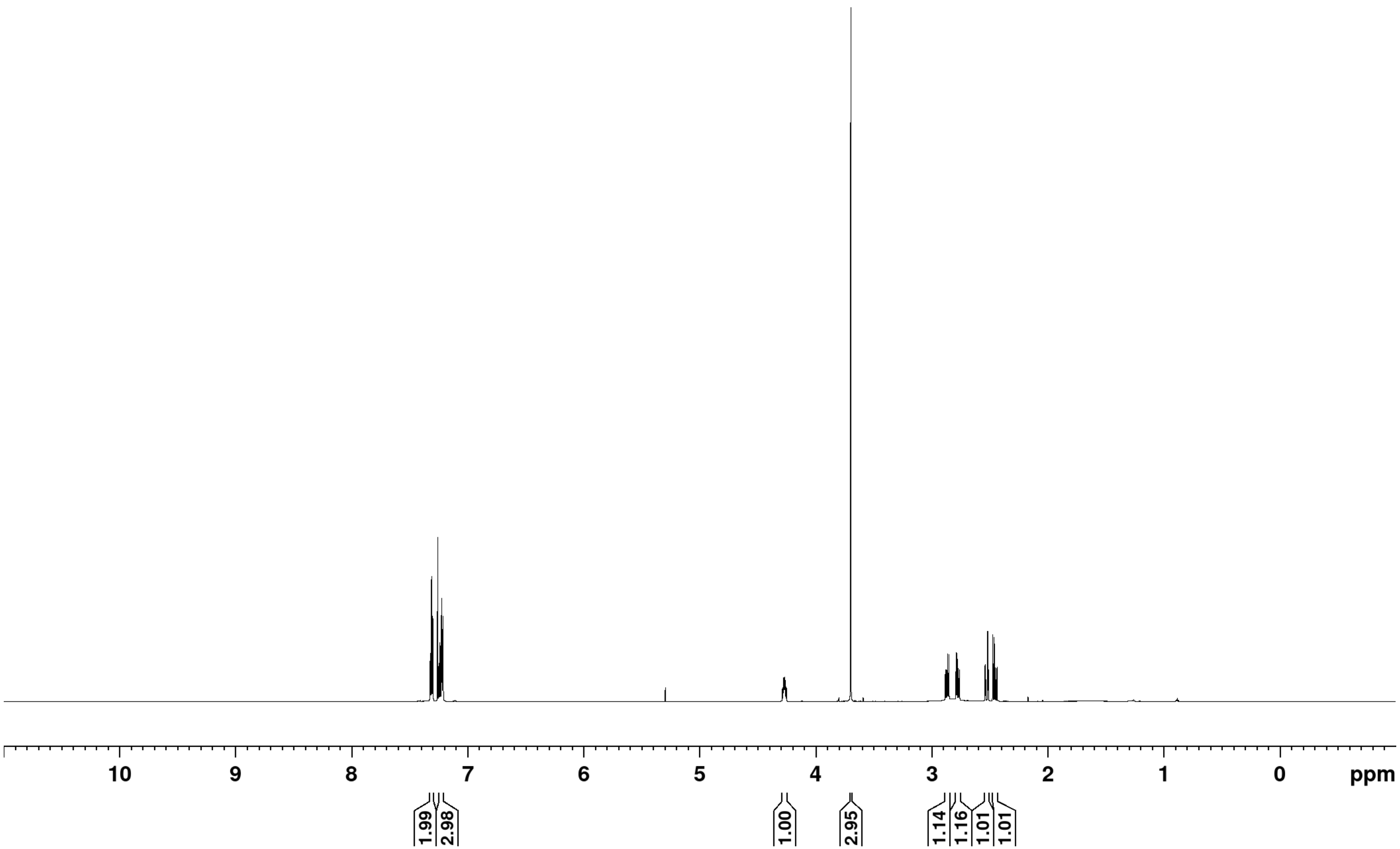

methyl (*R*)-3-hydroxy-4-phenylbutanoate (*int*$_2$-3a)

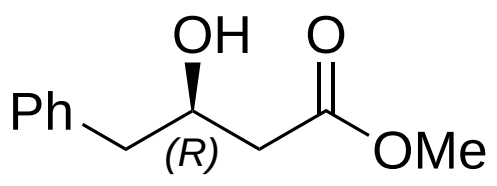

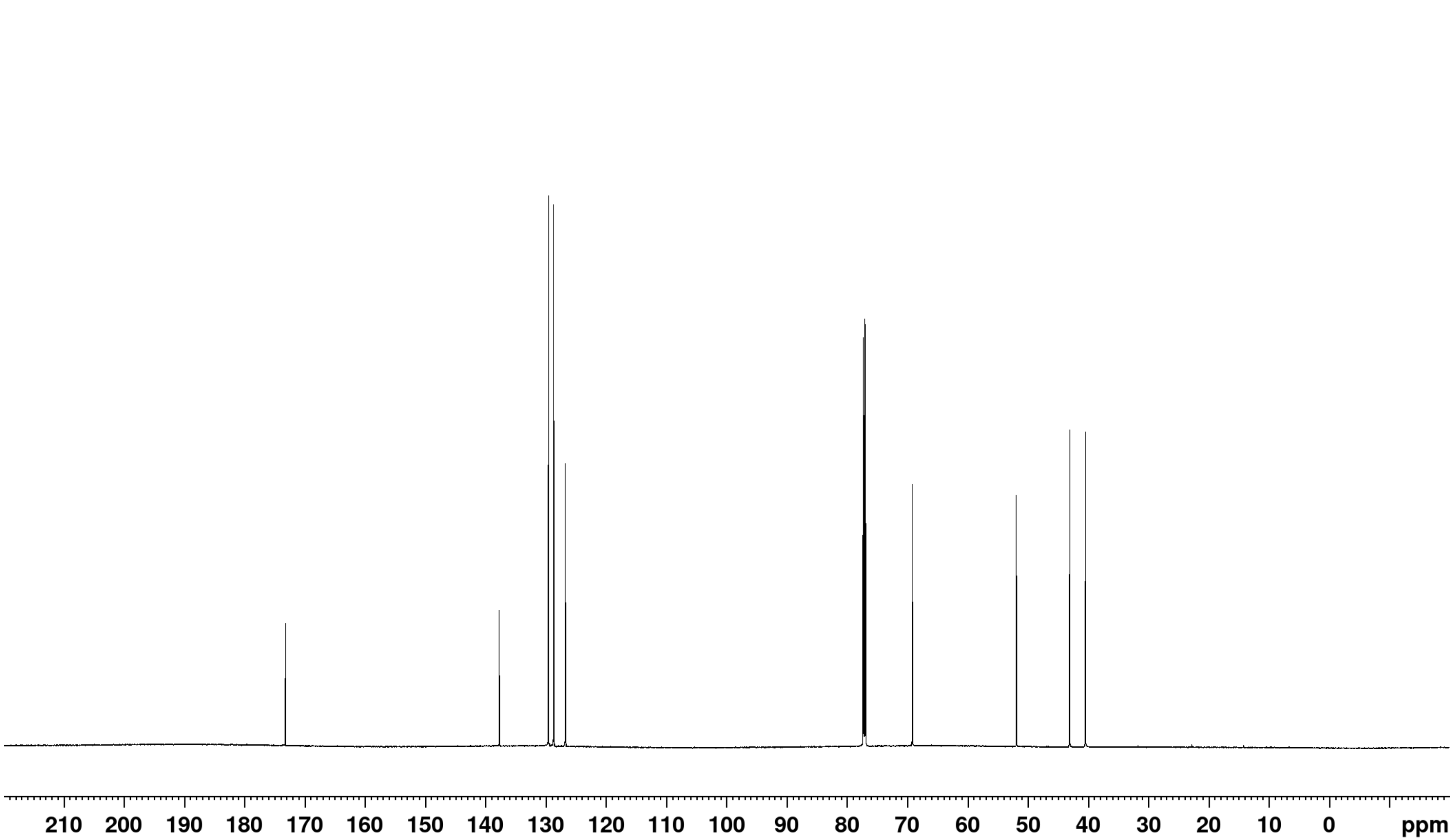

(*R*)-3-hydroxy-*N*-methoxy-*N*-methyl-4-phenylbutanamide (*int₃*-3a)

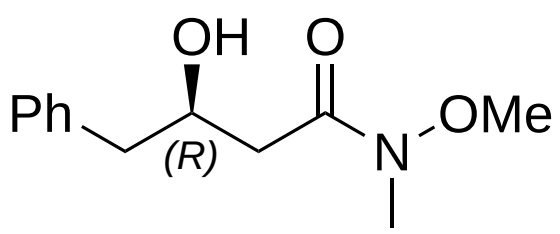

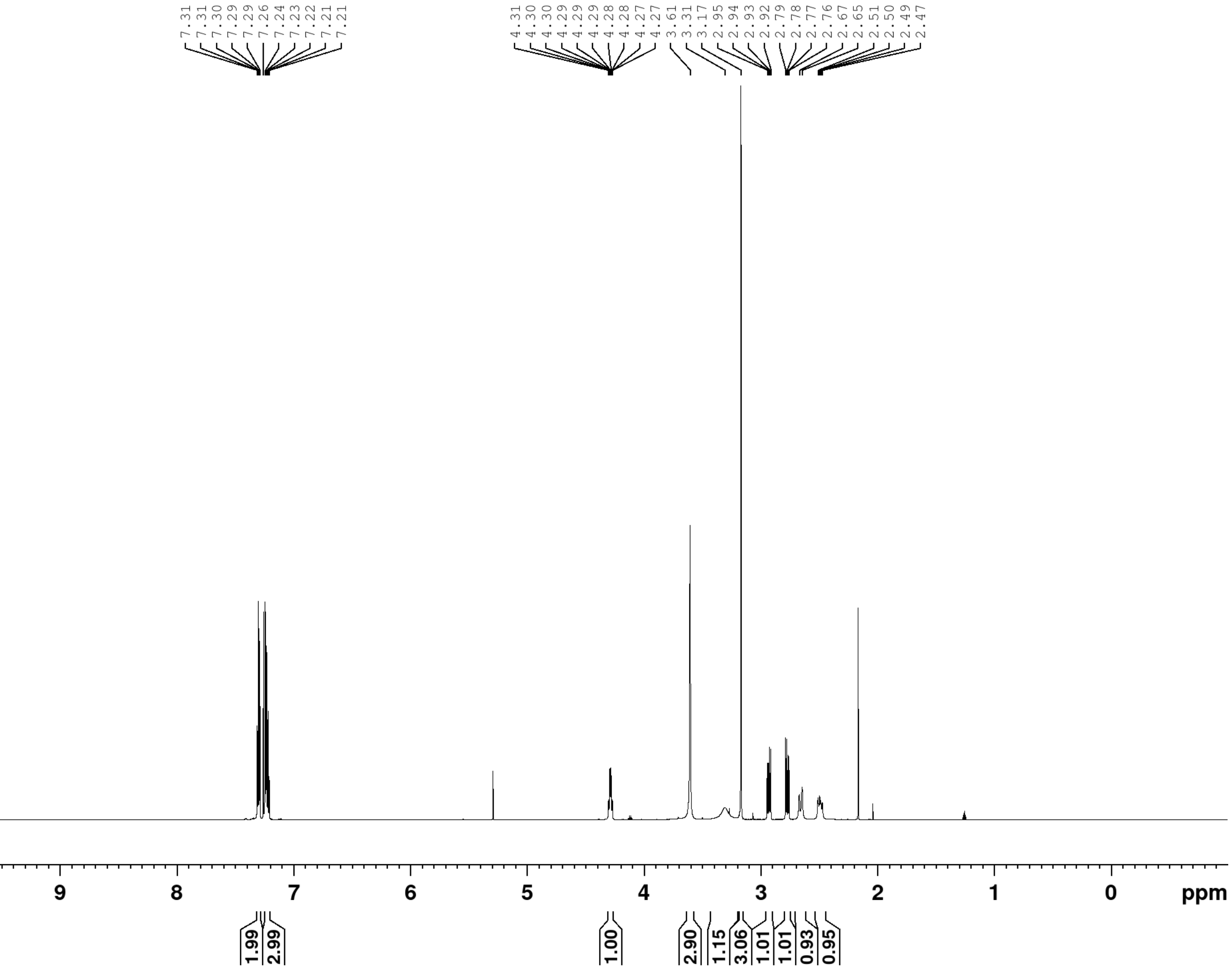

(*R*)-3-hydroxy-*N*-methoxy-*N*-methyl-4-phenylbutanamide (*int*$_3$-3a)

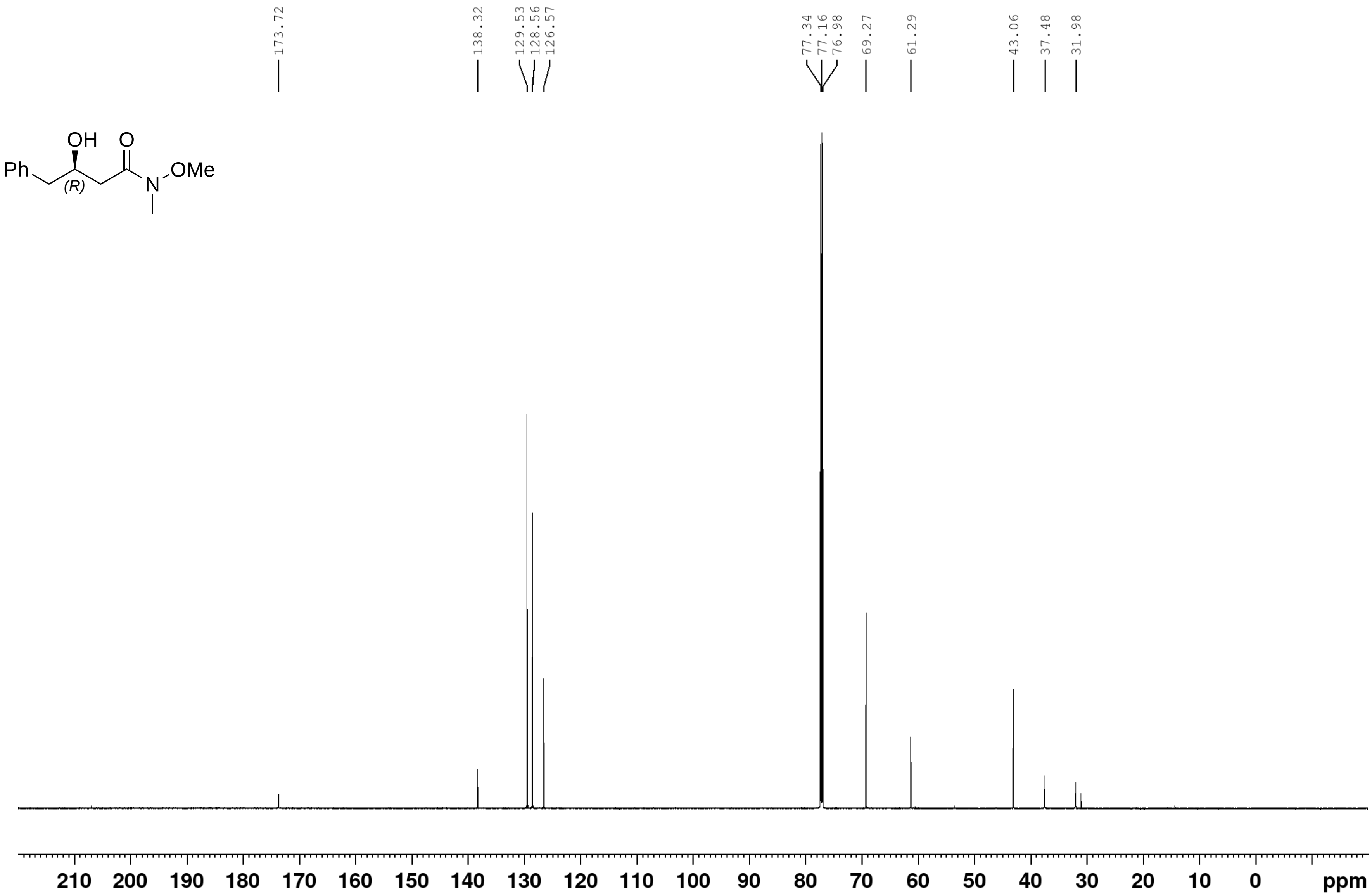

(R)-3-((tert-butyldimethylsilyl)oxy)-N-methoxy-N-methyl-4-phenylbutanamide (*int₄*-3a)

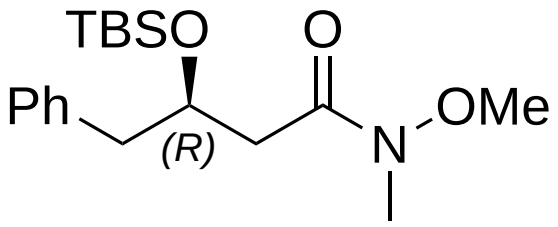

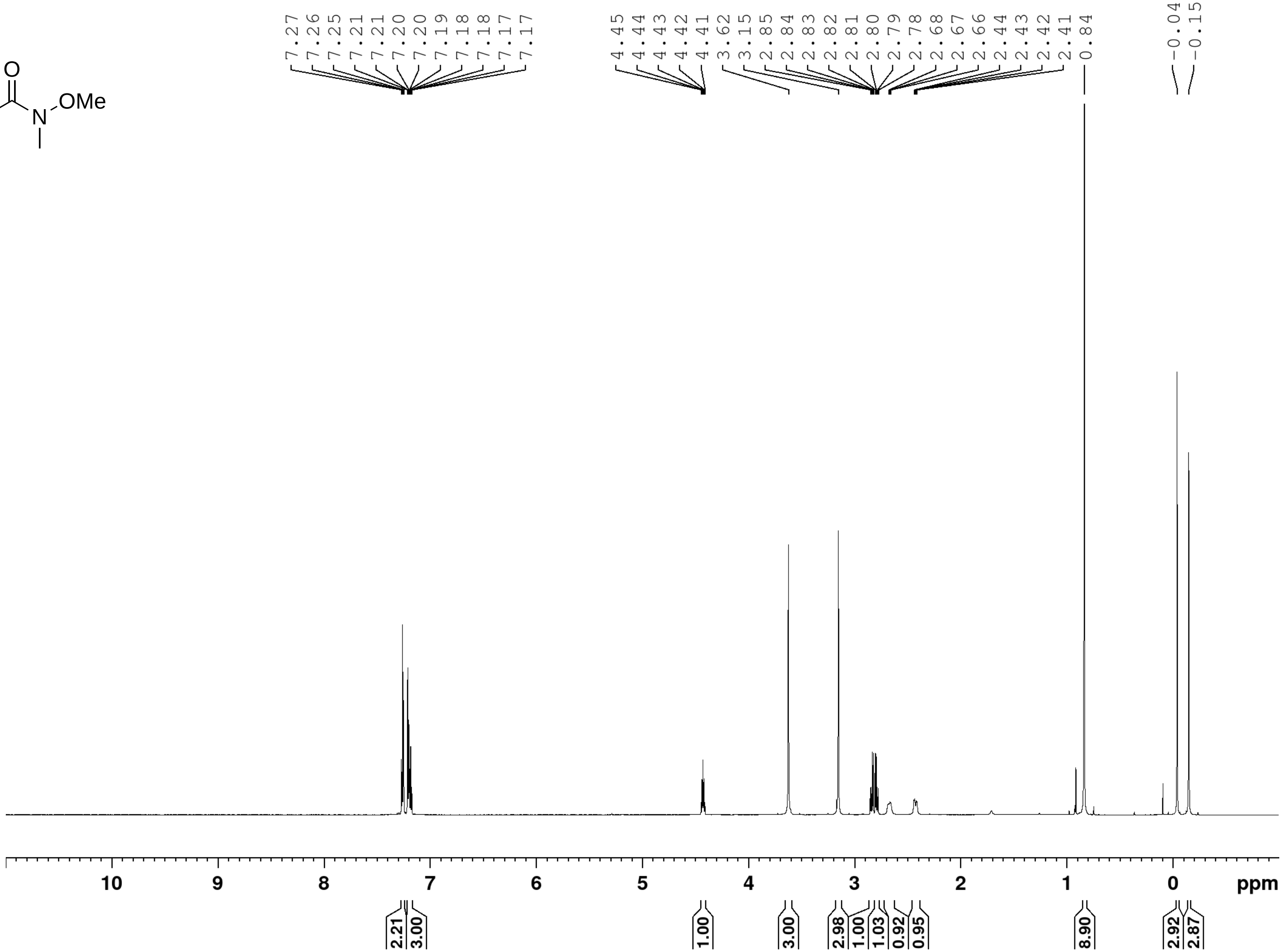

(R)-3-((tert-butyldimethylsilyl)oxy)-N-methoxy-N-methyl-4-phenylbutanamide (*int*$_4$-3a)

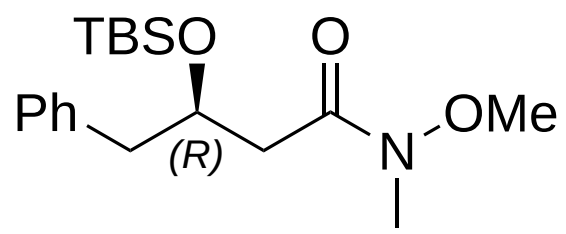

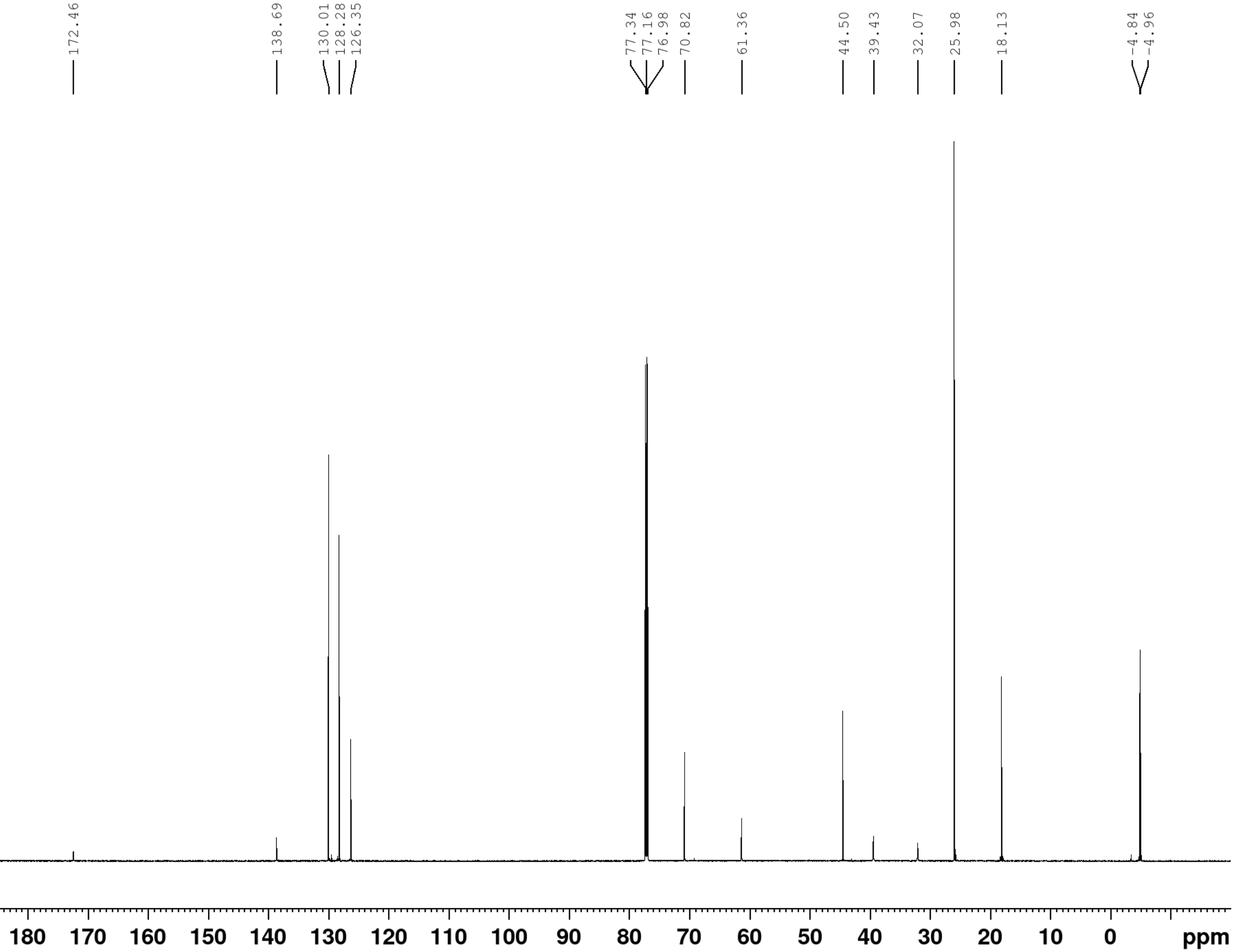

(2*S*,4*R*)-4-((*tert*-butyldimethylsilyl)oxy)-5-phenylpentan-2-ol (*syn-int*$_5$-3a) and (2*R*,4*R*)-4-((*tert*-butyldimethylsilyl)oxy)-5-phenylpentan-2-ol (*anti-int*$_5$-3a)

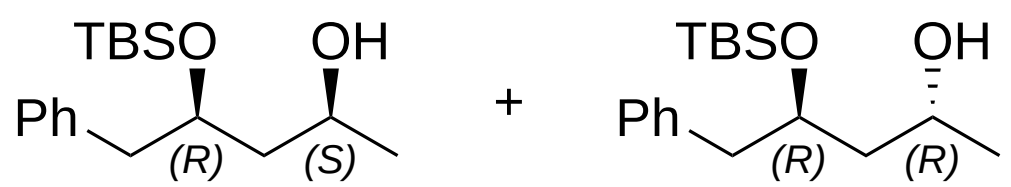

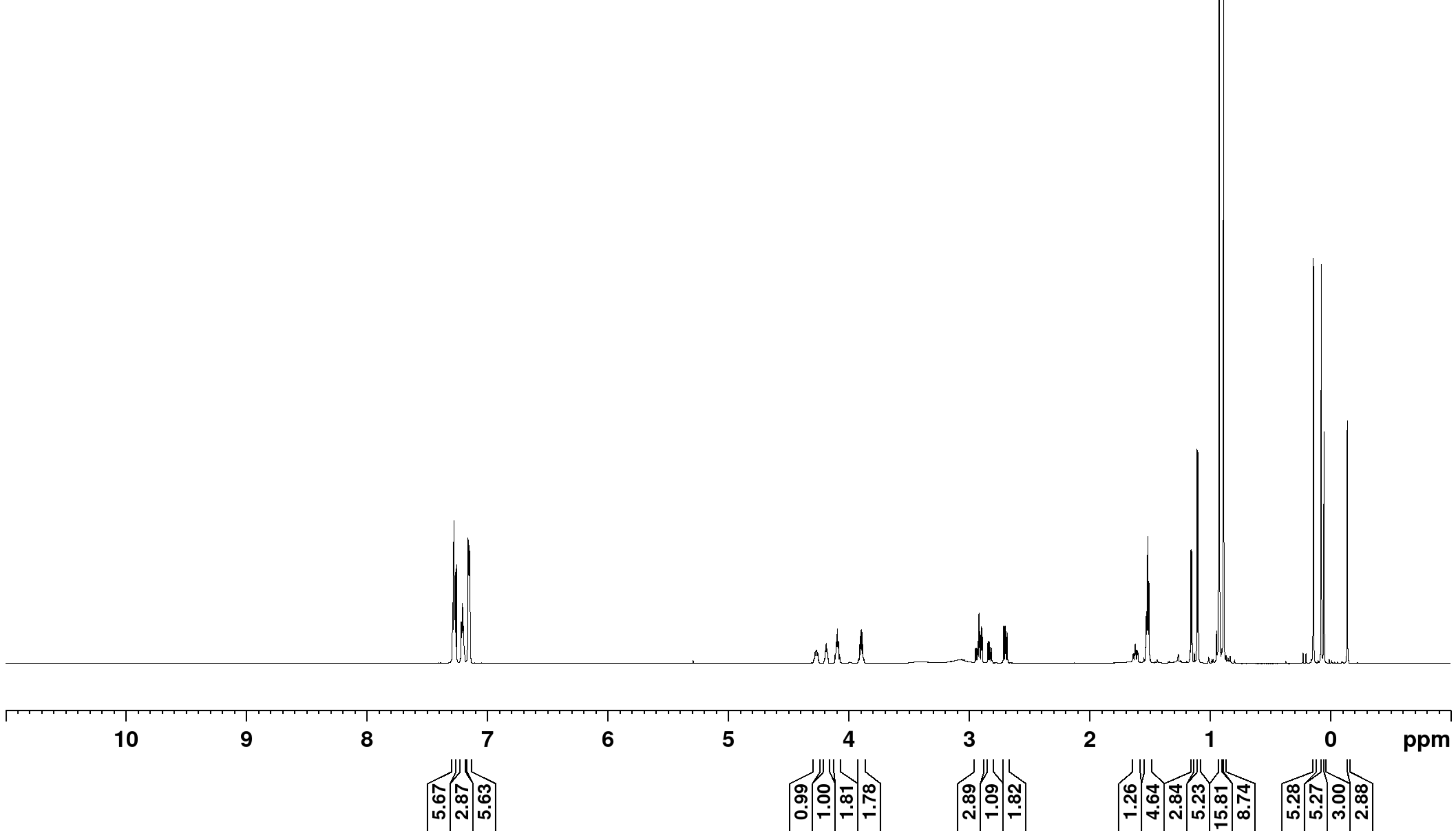

(2*S*,4*R*)-4-((*tert*-butyldimethylsilyl)oxy)-5-phenylpentan-2-ol (*syn-int*$_5$-3a) and (2*R*,4*R*)-4-((*tert*-butyldimethylsilyl)oxy)-5-phenylpentan-2-ol (*anti-int*$_5$-3a)

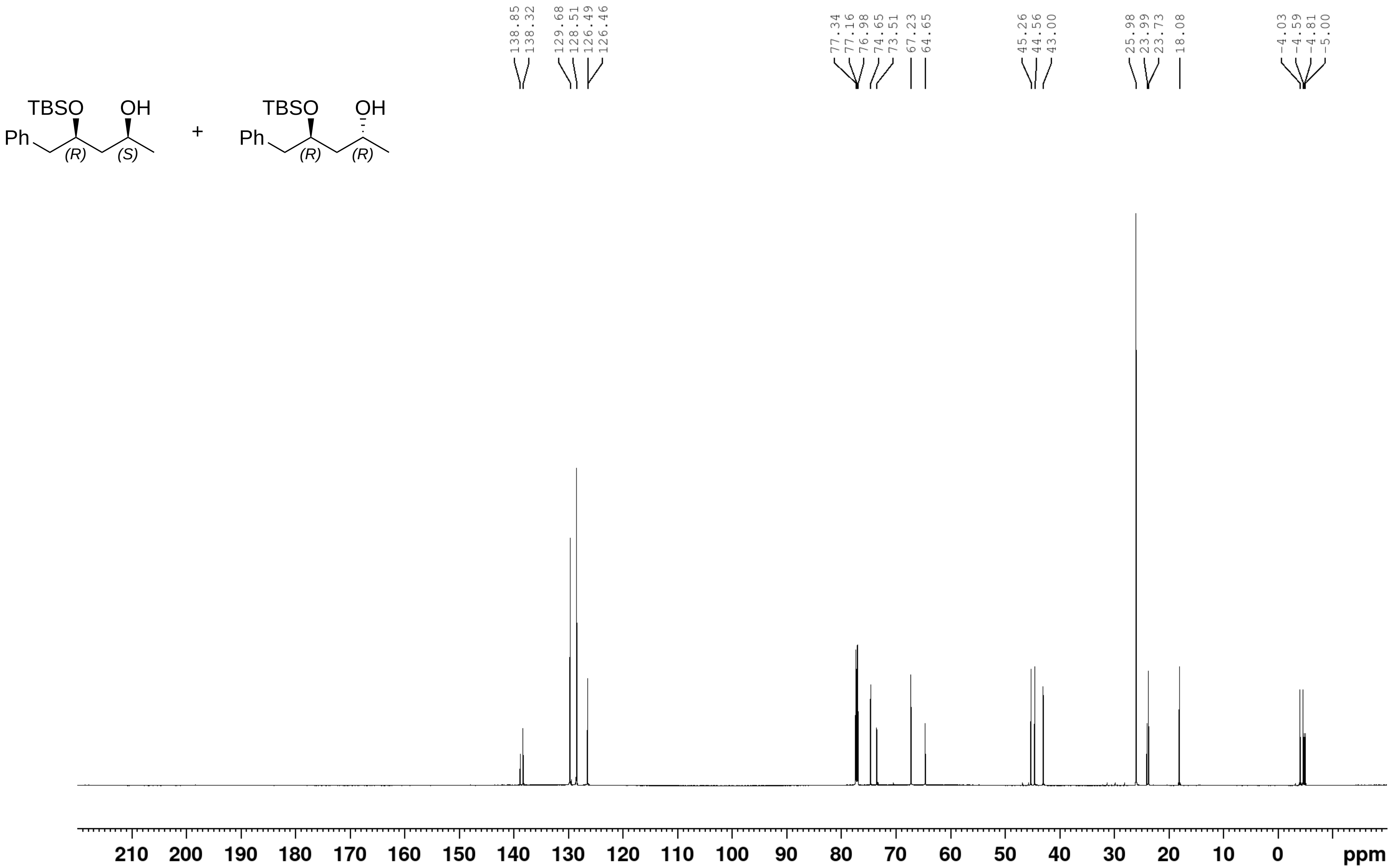

(2*R*,4*S*)-1-phenylpentane-2,4-diol ((*S*,*S*)-*syn*-3a)

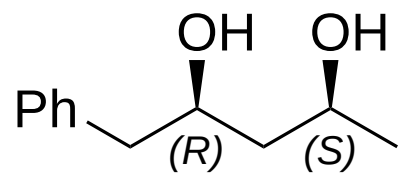

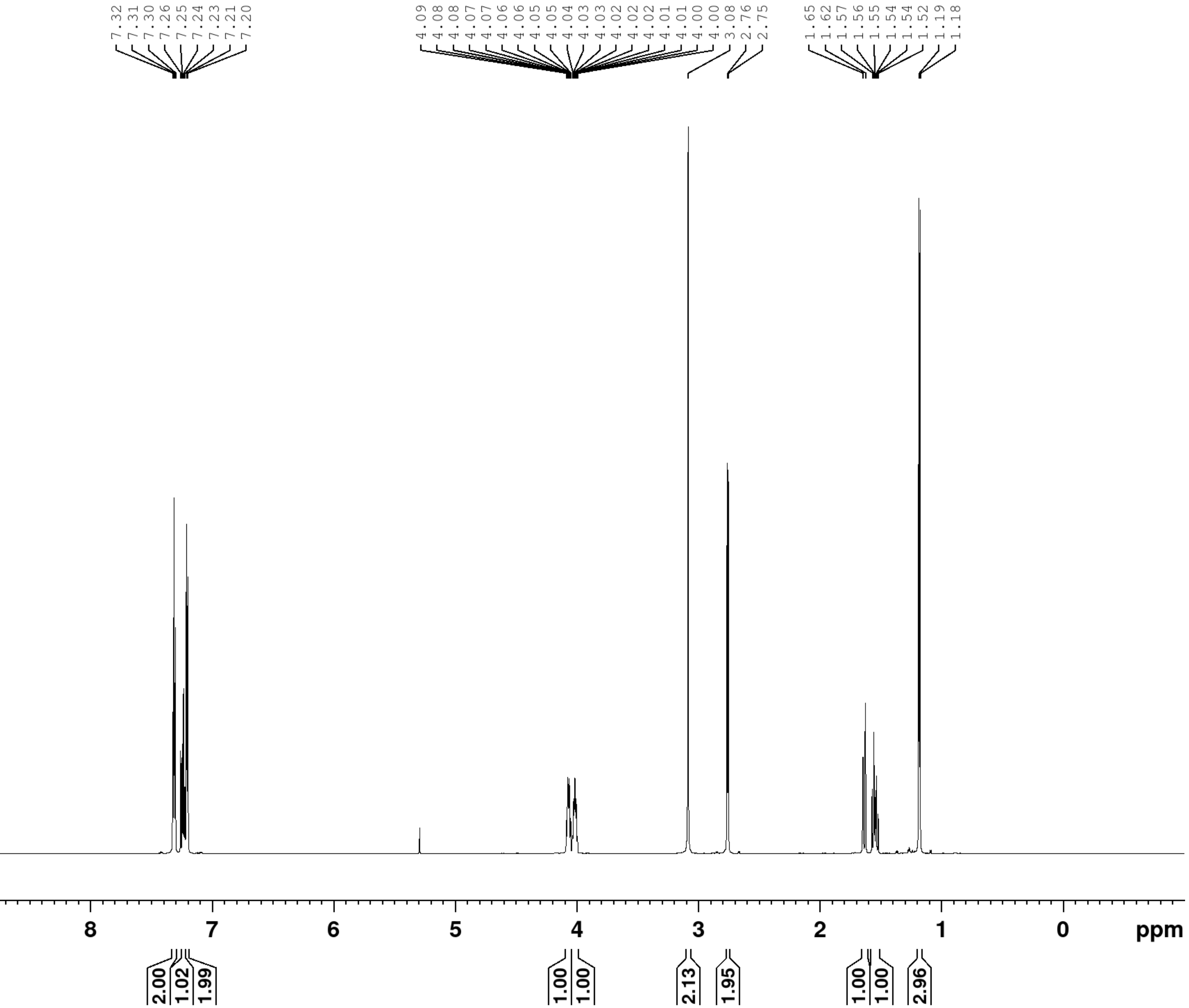

(2*R*,4*S*)-1-phenylpentane-2,4-diol ((*S*,*S*)-*syn*-3a)

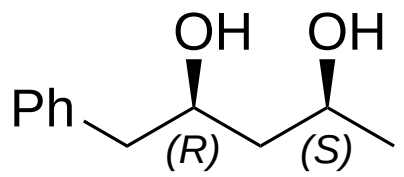

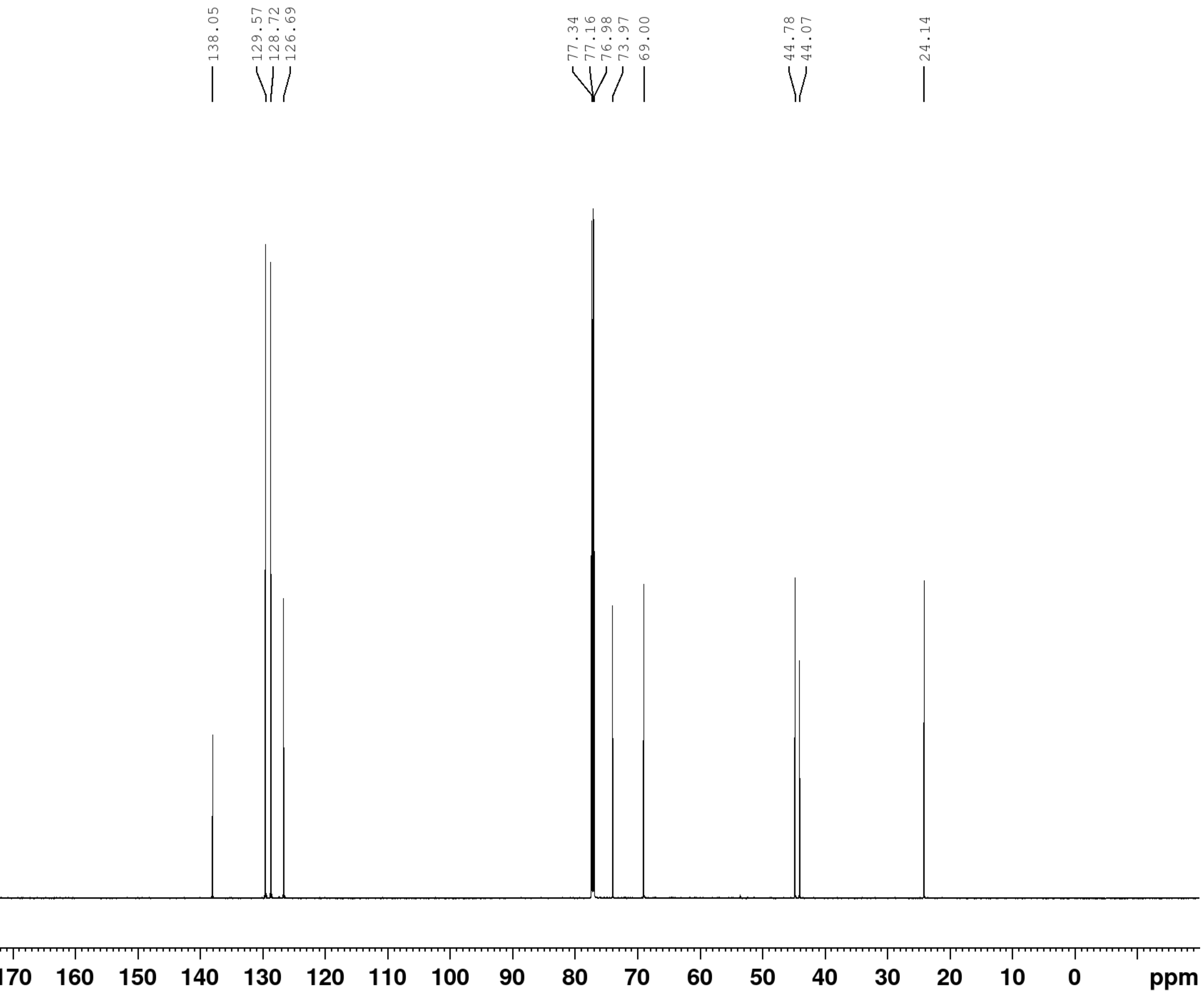

(2*R*,4*R*)-1-phenylpentane-2,4-diol ((*R*,*R*)-*anti*-3a)

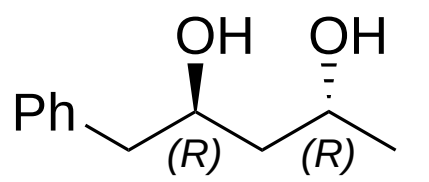

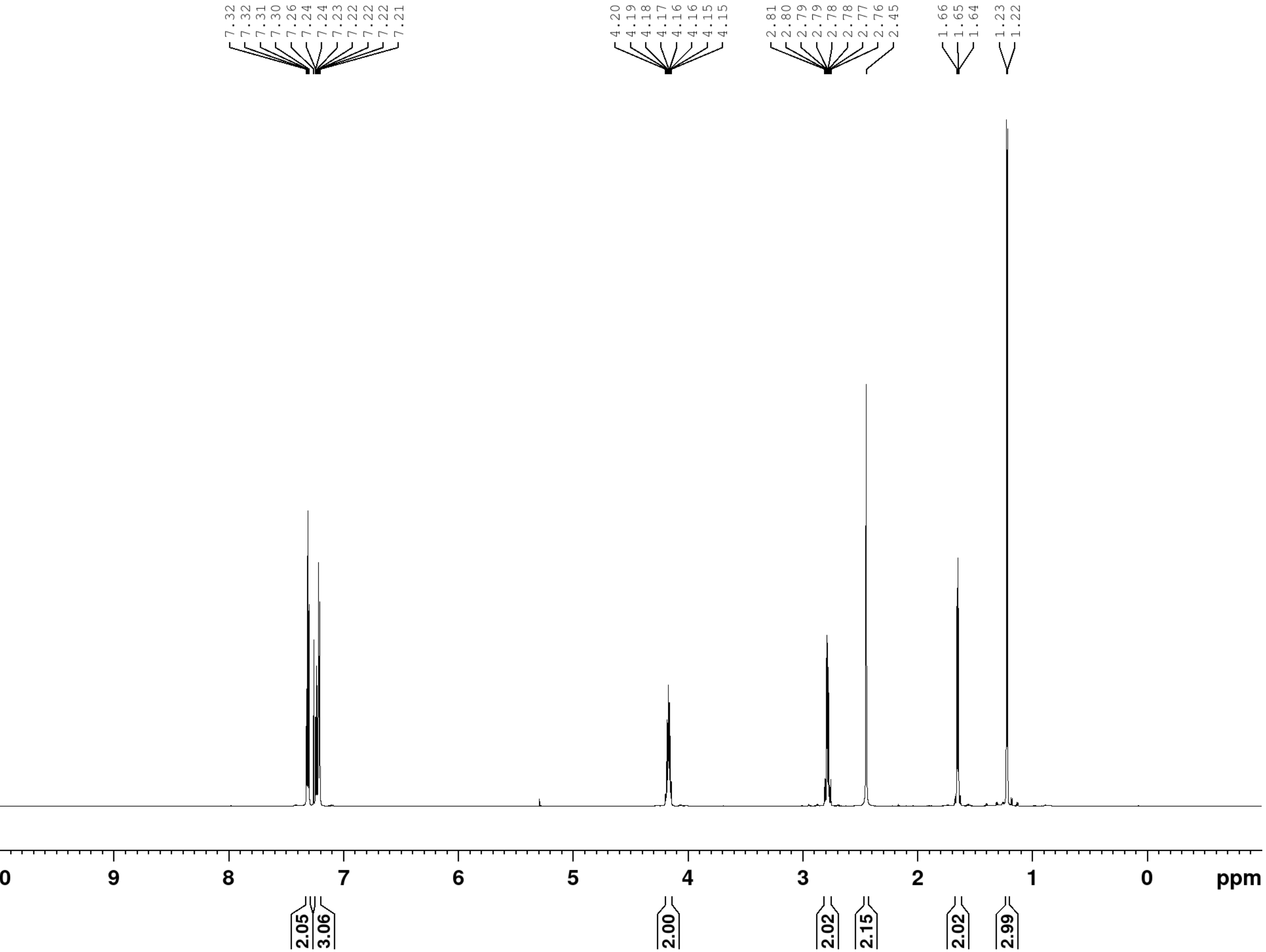

(2*R*,4*R*)-1-phenylpentane-2,4-diol ((*R*,*R*)-*anti*-3a)

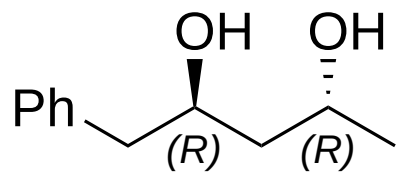

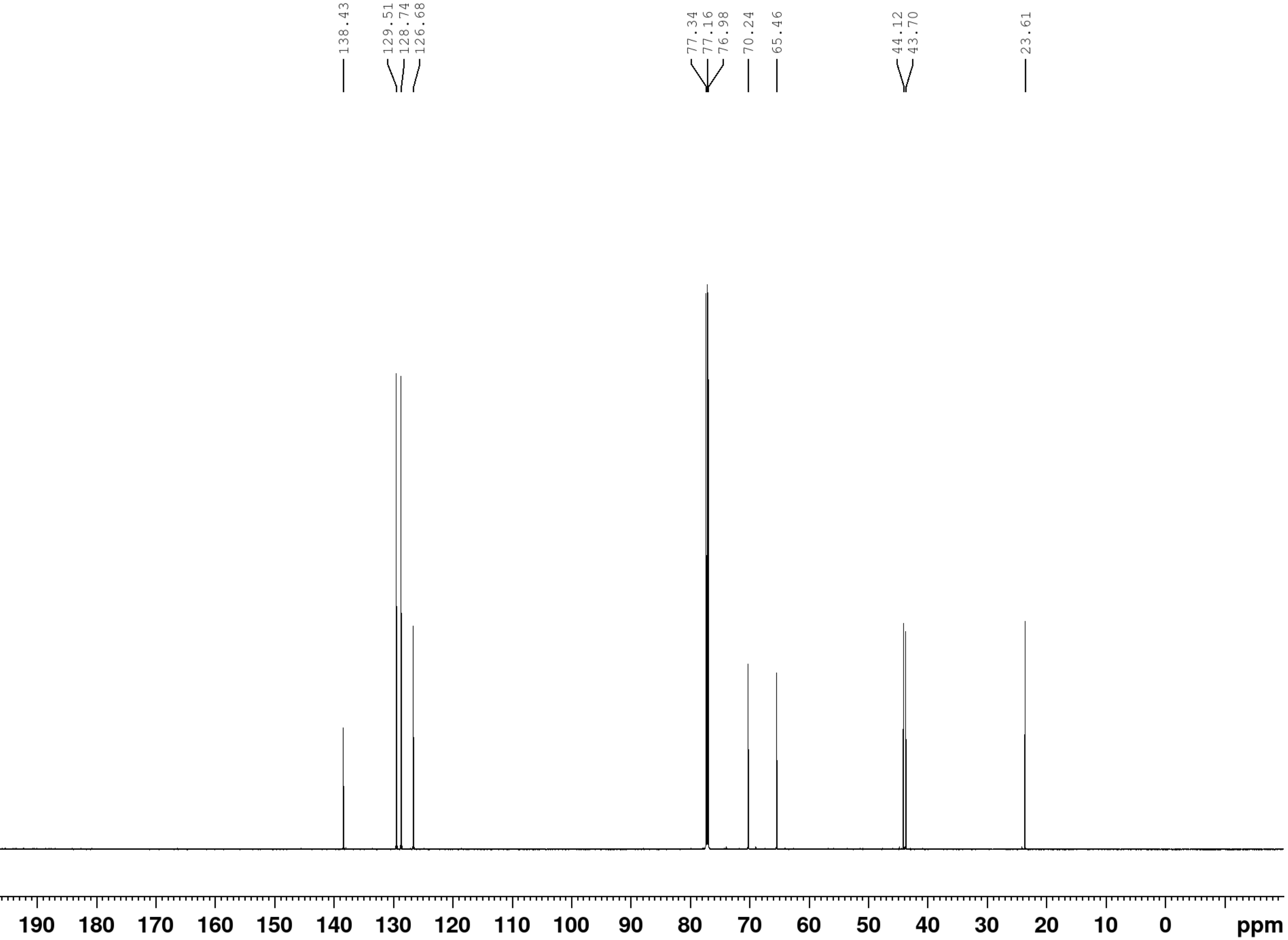

(2*R*)-4-methyl-6-(phenylsulfonyl)hexane-2,4-diol

Chiral SFC Analysis diastereomer 1: CHIRALPAK IK (CO_2:MeOH, 85:15, 2.5 ml min^{-1}, 40 °C, 214 nm) t_R = 4.9 (minor), 5.2 (major) min., 96% *ee*.

Chiral SFC Analysis diastereomer 2: CHIRALPAK IK (CO_2:MeOH, 85:15, 2.5 ml min^{-1}, 40 °C, 214 nm) t_R = 5.7 (major), 6.8 (minor) min., 96% *ee*.

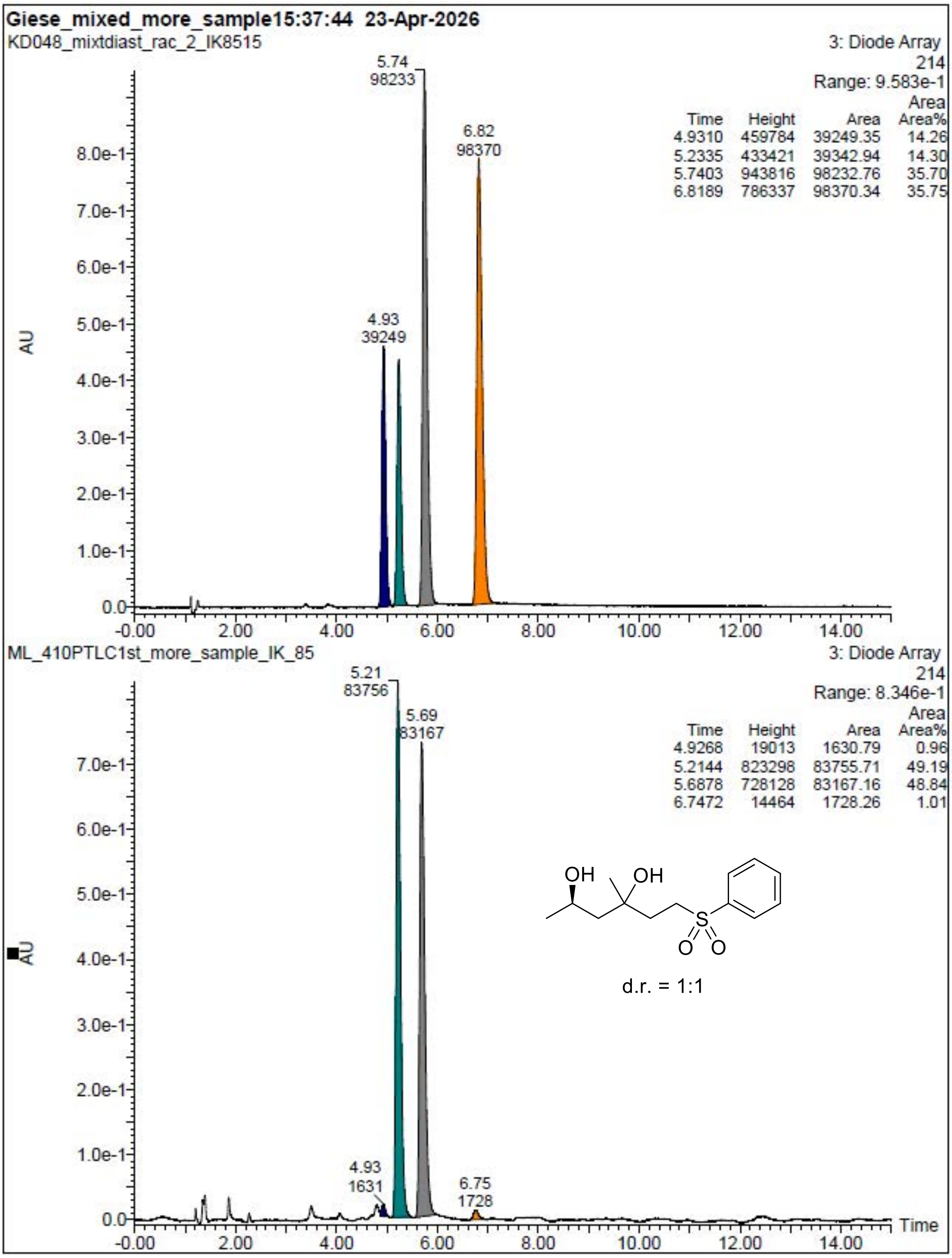

(*S*,*S*)-pentane-2,4-diyl dibenzoate (1a-Bz)

Chiral SFC Analysis CHIRALPAK IG (CO_2:MeOH, 95:05, 2.5 ml min^{-1}, 40 °C, 225 nm) t_R = 6.1 (minor), 9.6 (major) min, 23% *ee*.

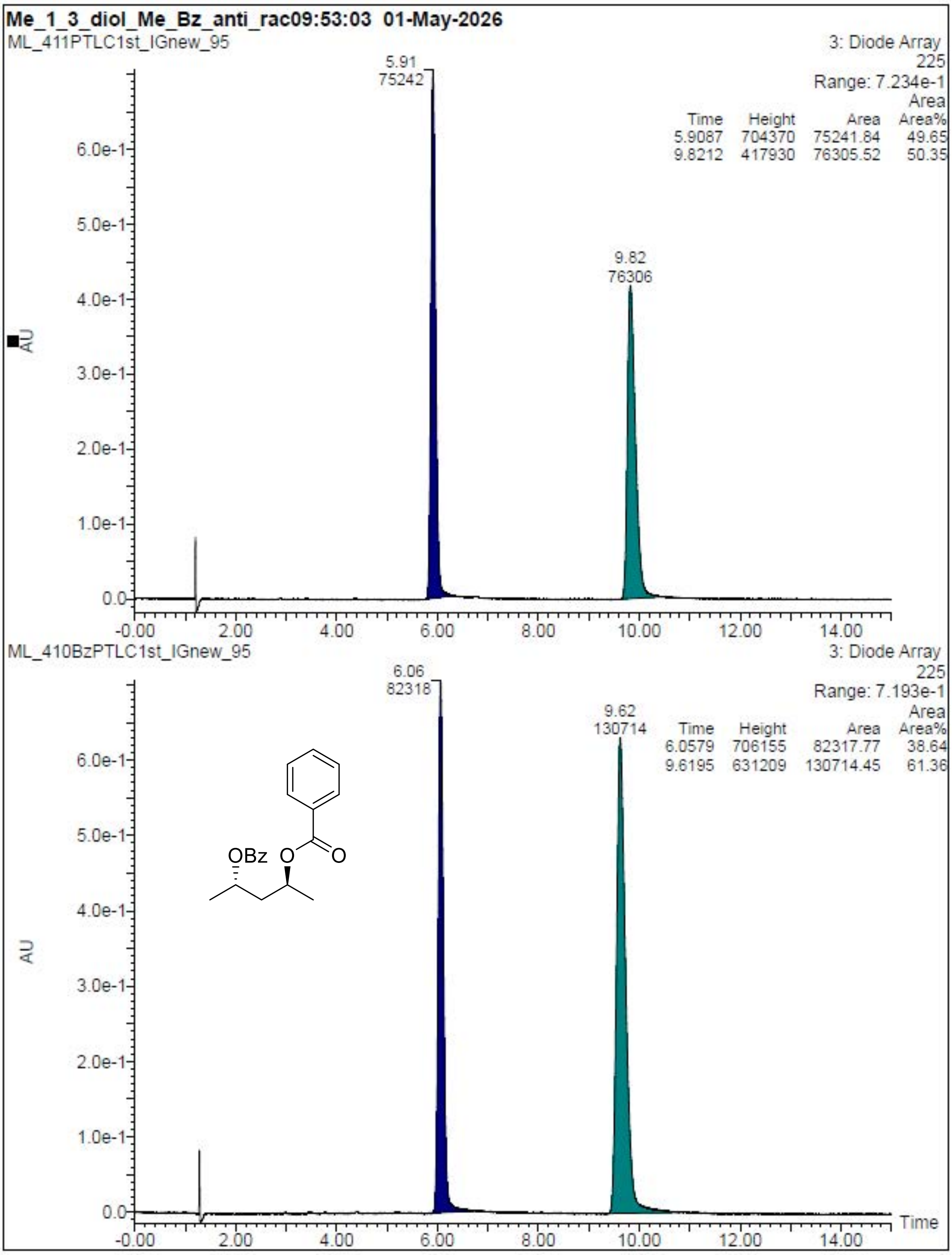

(2*S*,4*S*)-pentane-2,4-diyl dibenzoate (1a-Bz)

Chiral SFC Analysis CHIRALPAK IG (CO_2:MeOH, 95:05, 2.5 ml min^{-1}, 40 °C, 226 nm) t_R = 10.3 (major) minutes, 99% *ee*.

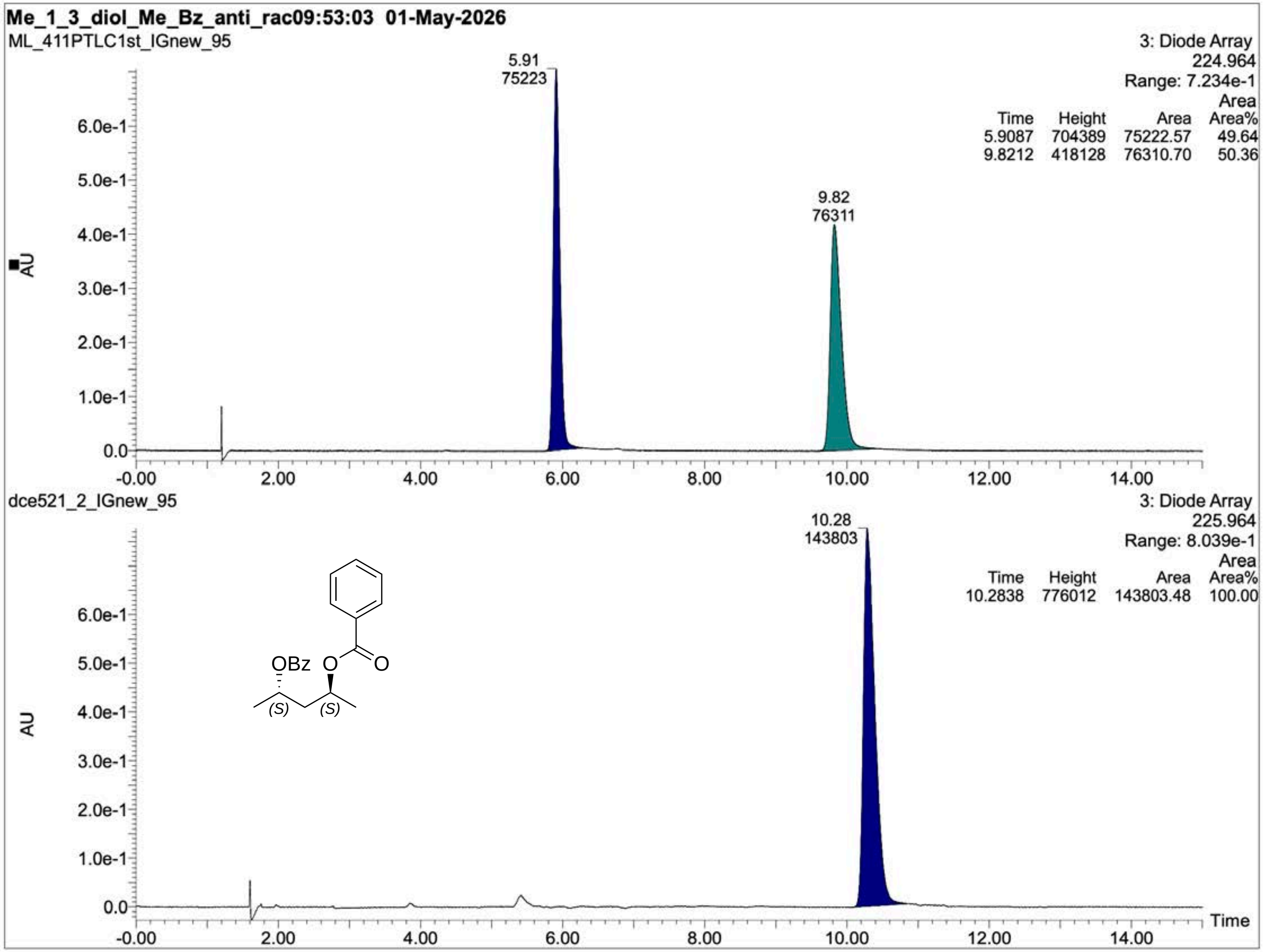

Time	Height	Area	Area Area%
5.9087	704389	75222.57	49.64
9.8212	418128	76310.70	50.36

Time	Height	Area	Area Area%
10.2838	776012	143803.48	100.00

(3S,5S)-heptane-3,5-diyl dibenzoate (1b-Bz)

Chiral SFC Analysis CHIRALPAK IG (CO_2:MeOH, 97:03, 2.5 ml min^{-1}, 40 °C, 225 nm) t_R = 7.8 (major) minutes, 99% *ee*.

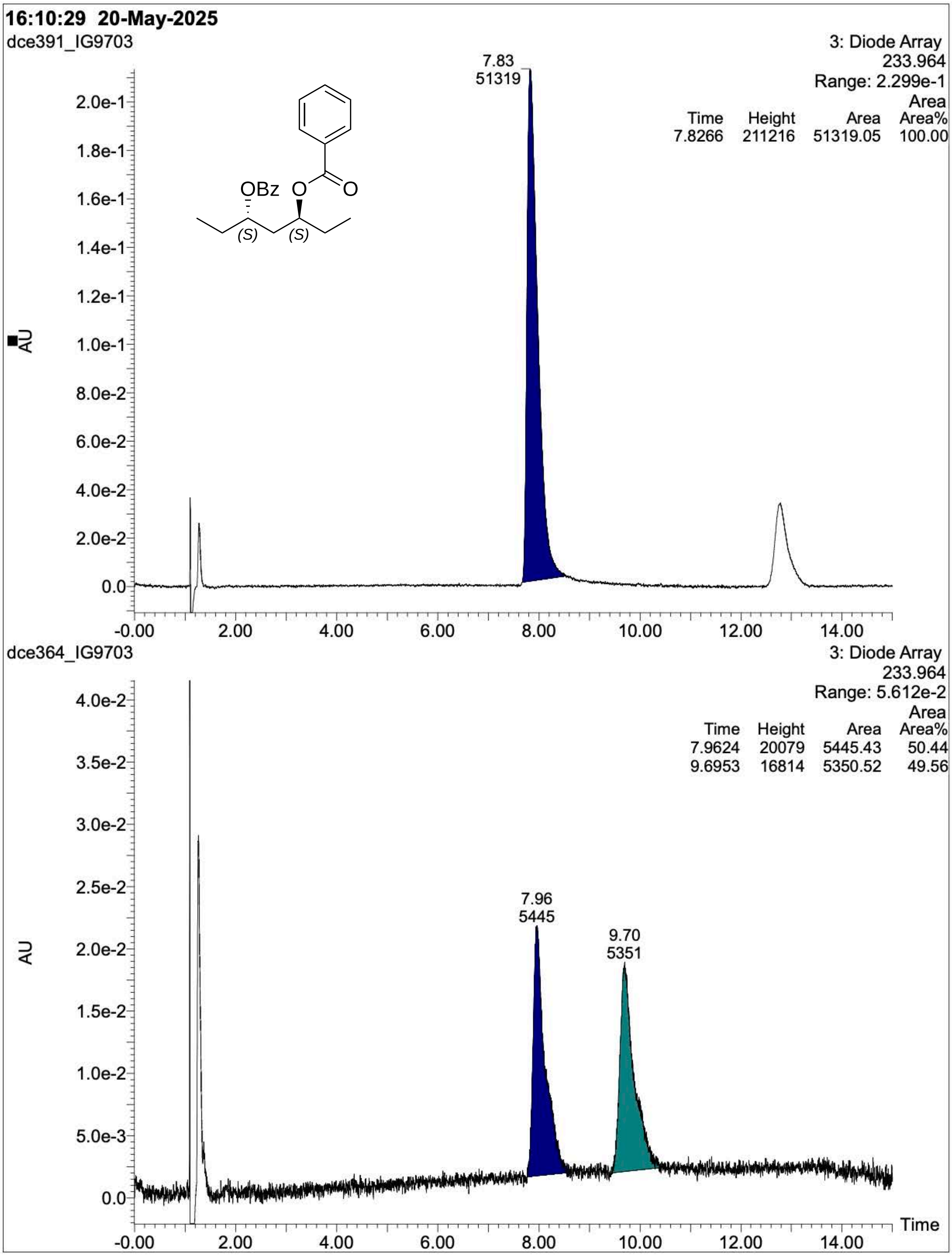

(4S,6S)-nonane-4,6-diyl dibenzoate (1c-Bz)

Chiral SFC Analysis CHIRALPAK IA (CO_2:MeOH, 99:01, 2.5 ml min^{-1}, 40 °C, 223 nm) t_R = 9.2 (major), 10.4 (minor) minutes, 97% *ee*.

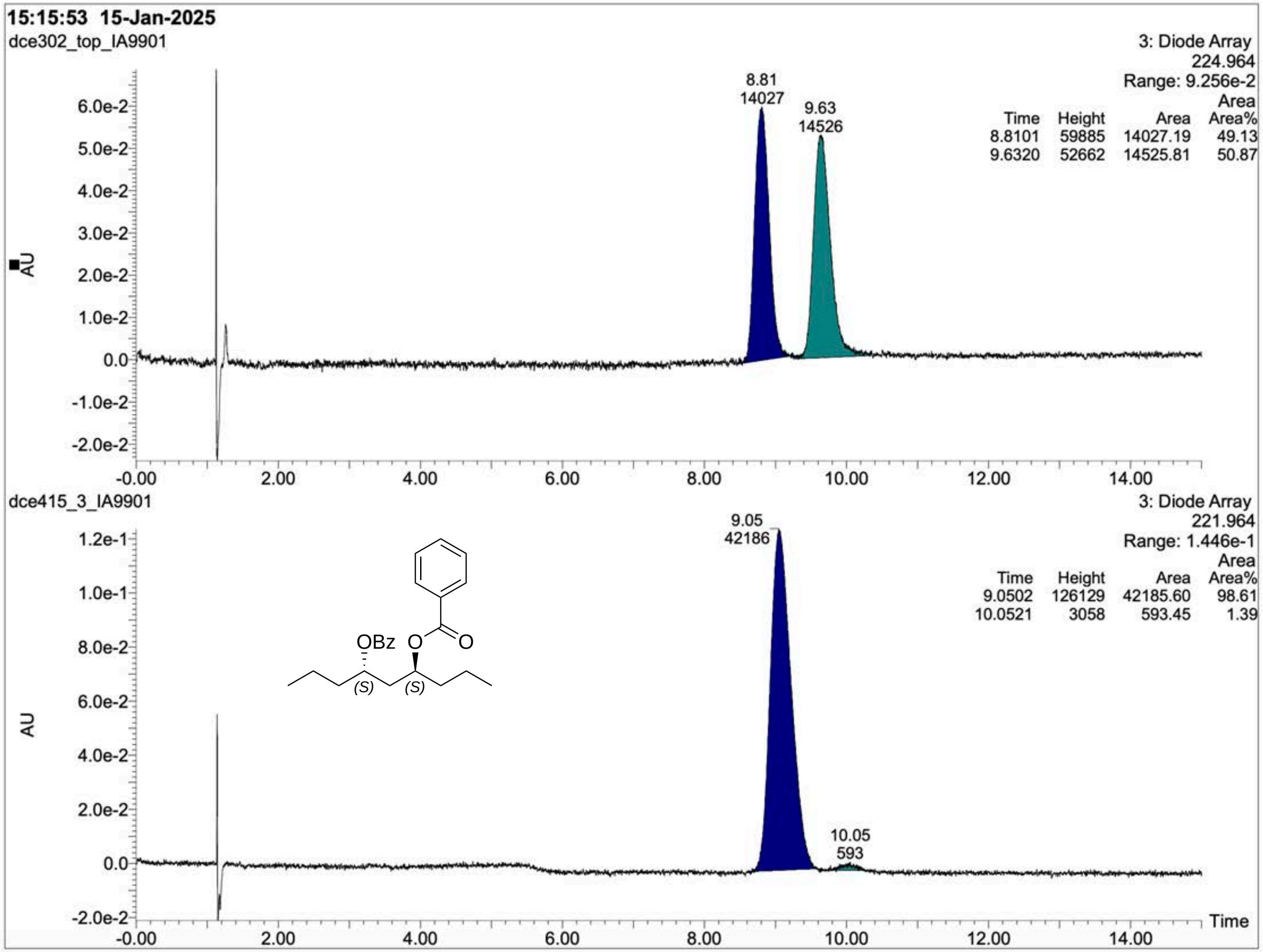

(5S,7S)-undecane-5,7-diyl dibenzoate (1d-Bz)

Chiral SFC Analysis CHIRALPAK IE (CO_2:MeOH, 97:03, 2.5 ml min^{-1}, 40 °C, 225 nm) t_R = 11.6 (major) minutes, 99% *ee*.

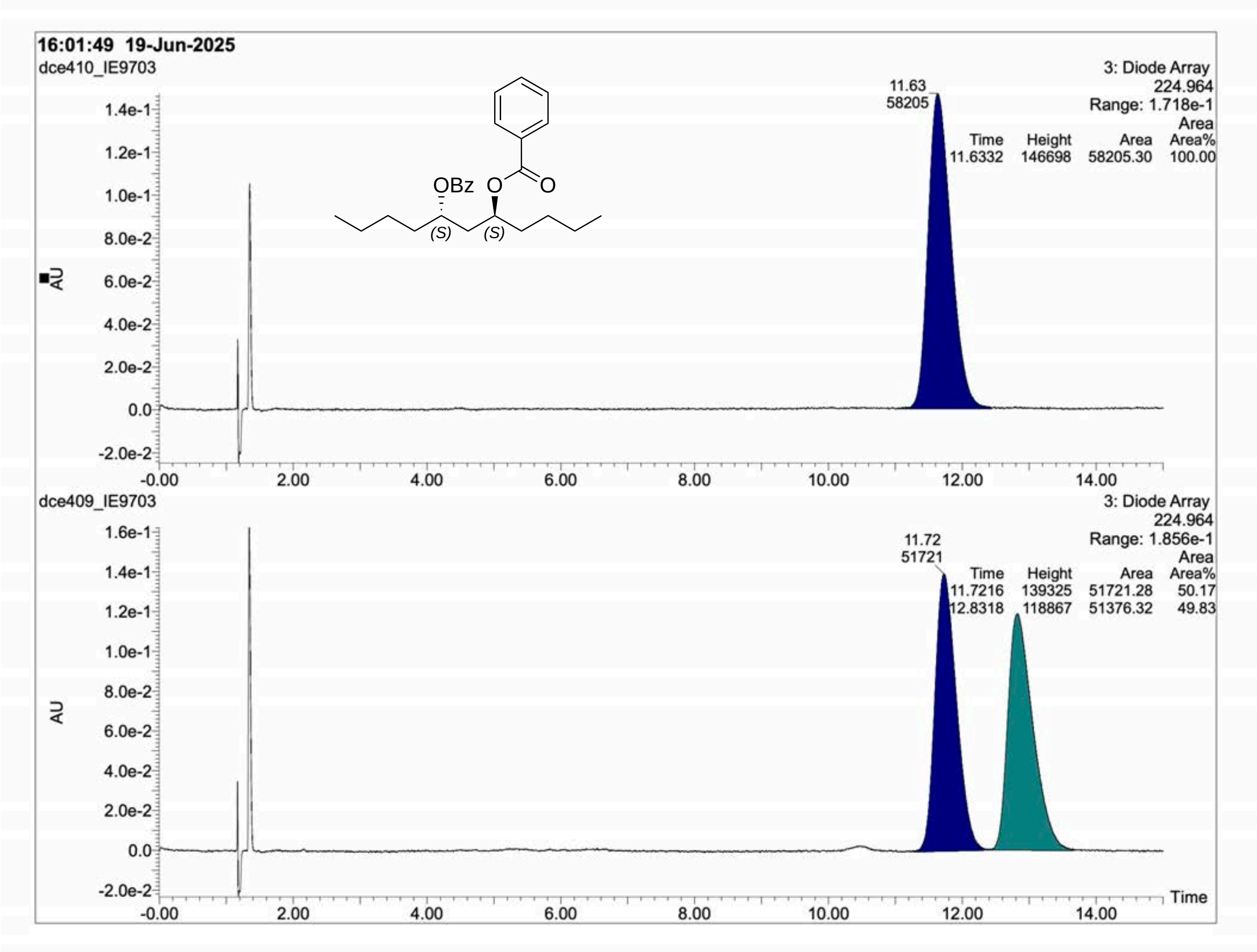

(4*S*,6*S*)-2,8-dimethylnonane-4,6-diyl dibenzoate (1e-Bz)

Chiral SFC Analysis CHIRALPAK IG (CO_2:MeOH, 97:03, 2.5 ml min^{-1}, 40 °C, 226 nm) t_R = 5.1 (major) minutes, 99% *ee*.

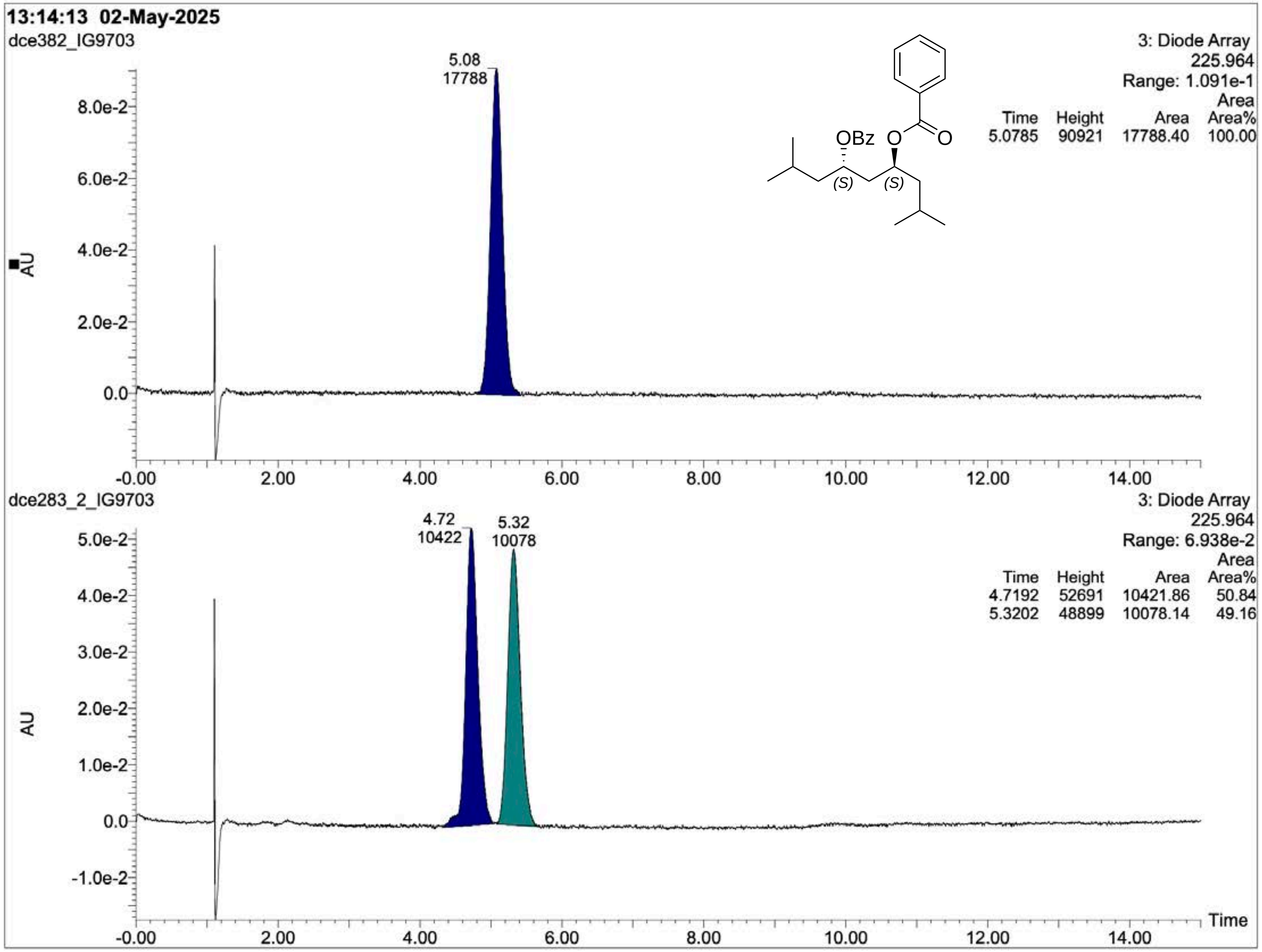

(5*S*,7*S*)-2,10-dimethylundecane-5,7-diyl dibenzoate (1f-Bz)

Chiral SFC Analysis CHIRALPAK IE (CO_2:MeOH, 97:03, 2.5 ml min^{-1}, 40 °C, 225 nm) t_R = 8.2 (major) minutes, 99% *ee*.

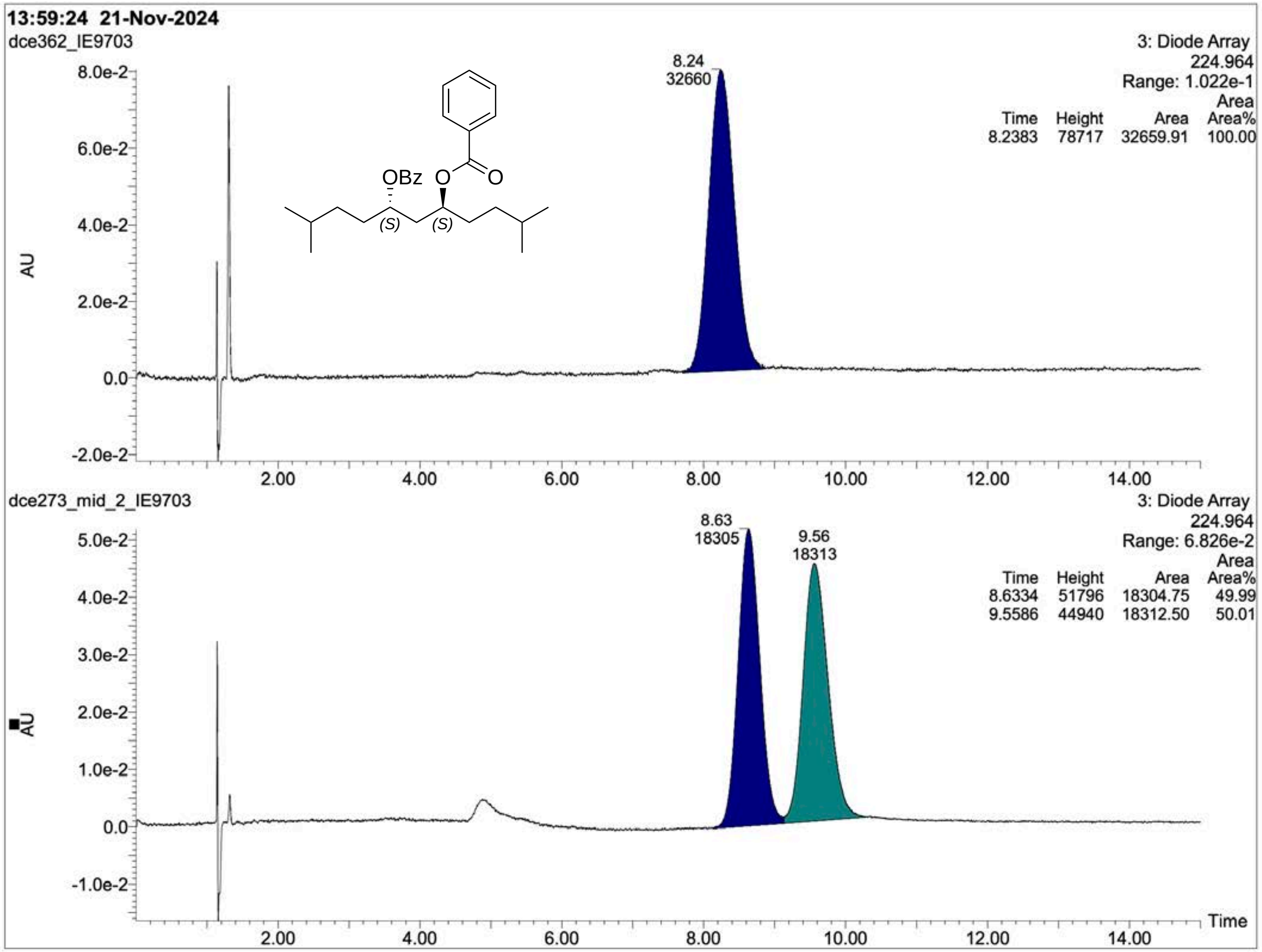

(3*S*,5*S*)-1,7-dicyclobutylheptane-3,5-diyl dibenzoate (1g-Bz)

Chiral SFC Analysis CHIRALPAK IJ (CO_2:MeOH, 97:03, 2.5 ml min^{-1}, 40 °C, 226 nm) t_R = 4.0 (major), 4.4 (minor) minutes, 88% *ee*.

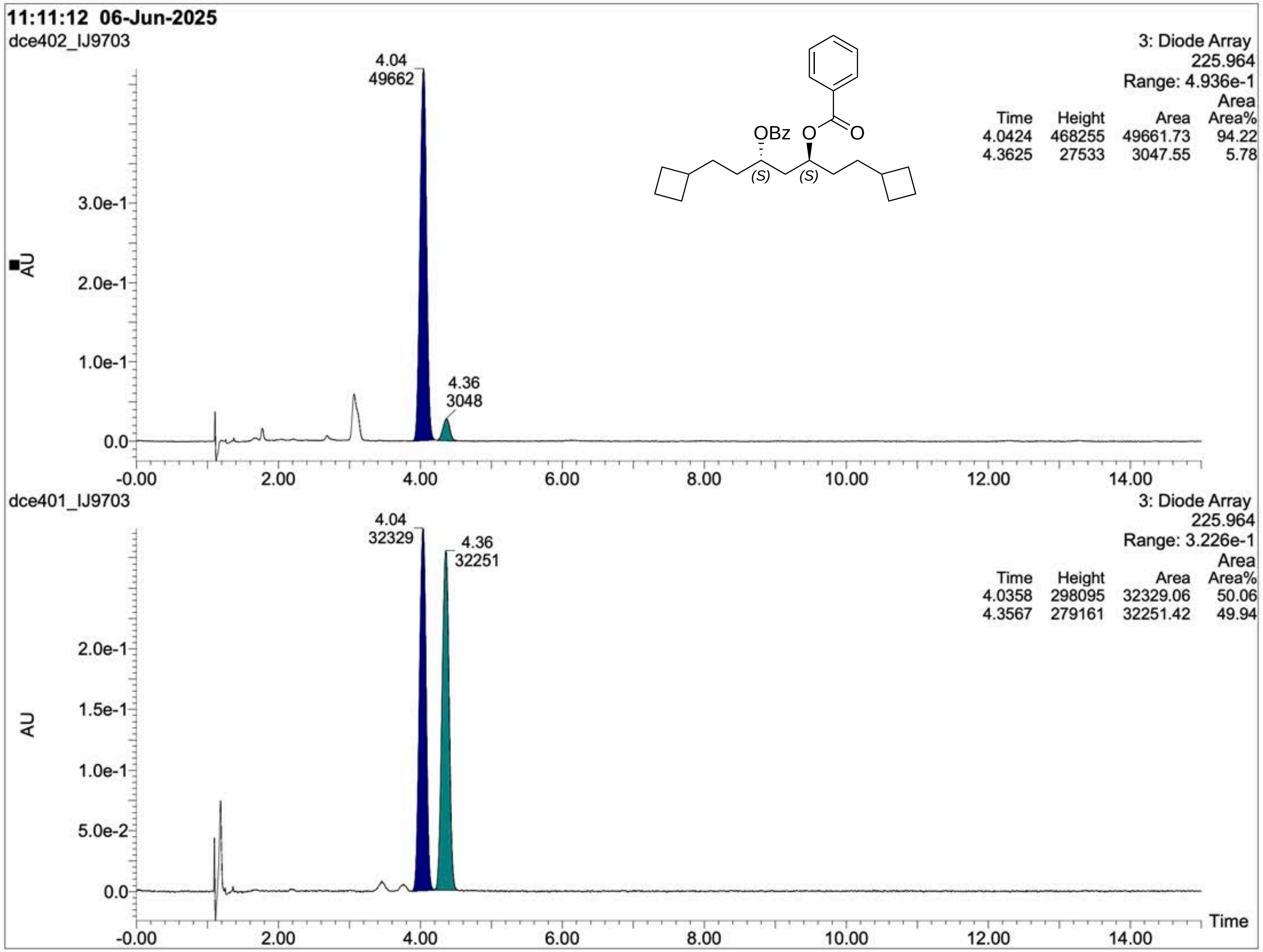

(2S,4S)-1,5-dicyclohexylpentane-2,4-diyl dibenzoate (1h-Bz)

Chiral SFC Analysis CHIRALPAK IE (CO_2:MeOH, 90:10, 2.5 ml min^{-1}, 40 °C, 226 nm) t_R = 6.7 (major), 7.3 (minor) minutes, 70% *ee*.

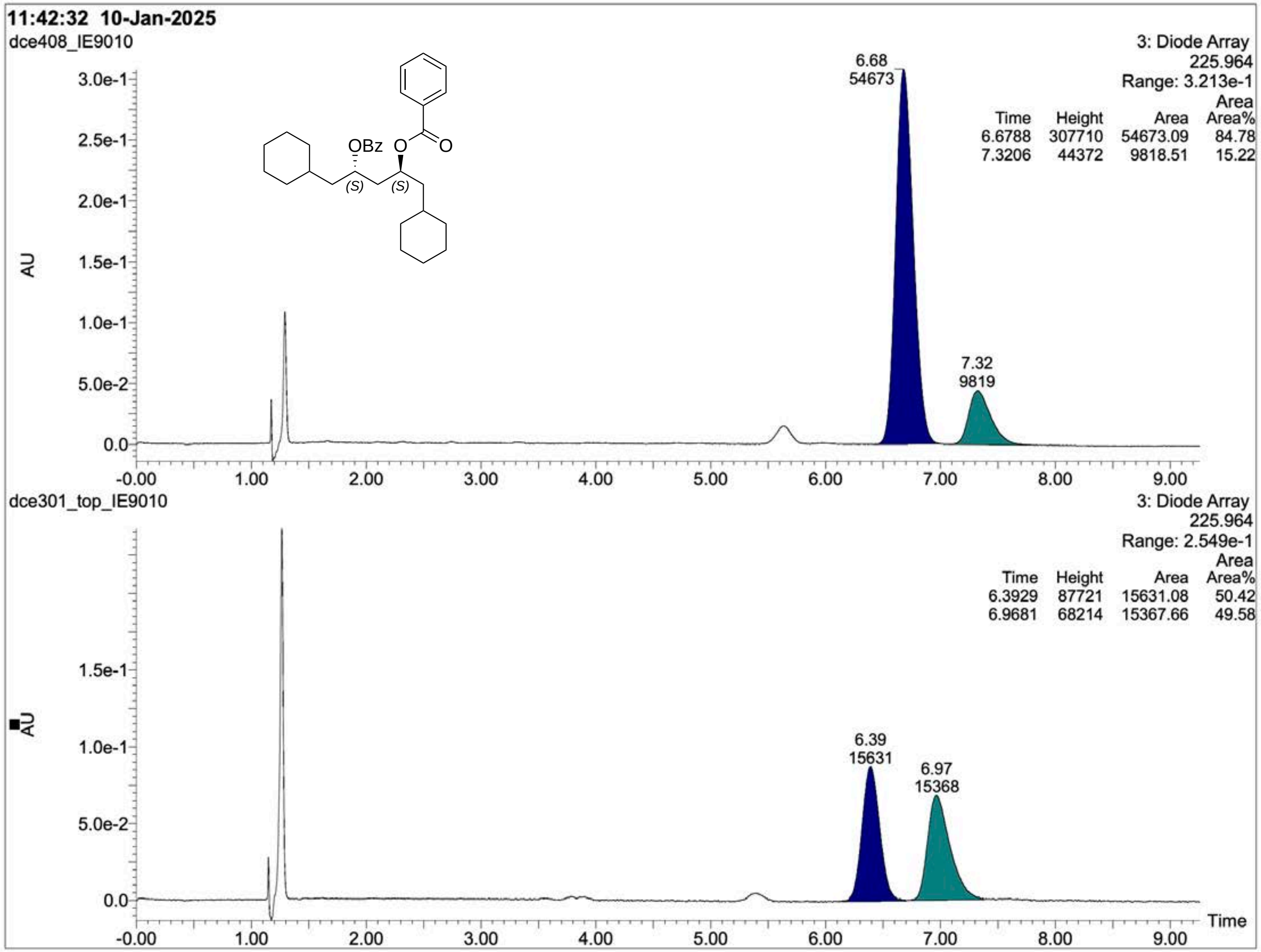

(3*S*,5*S*)-1,7-dicyclohexylheptane-3,5-diyl dibenzoate (1i-Bz)

Chiral SFC Analysis CHIRALPAK IG (CO_2:MeOH, 90:10, 2.5 ml min^{-1}, 40 °C, 226 nm) t_R = 8.7 (minor), 10.7 (major) minutes, 94% *ee*.

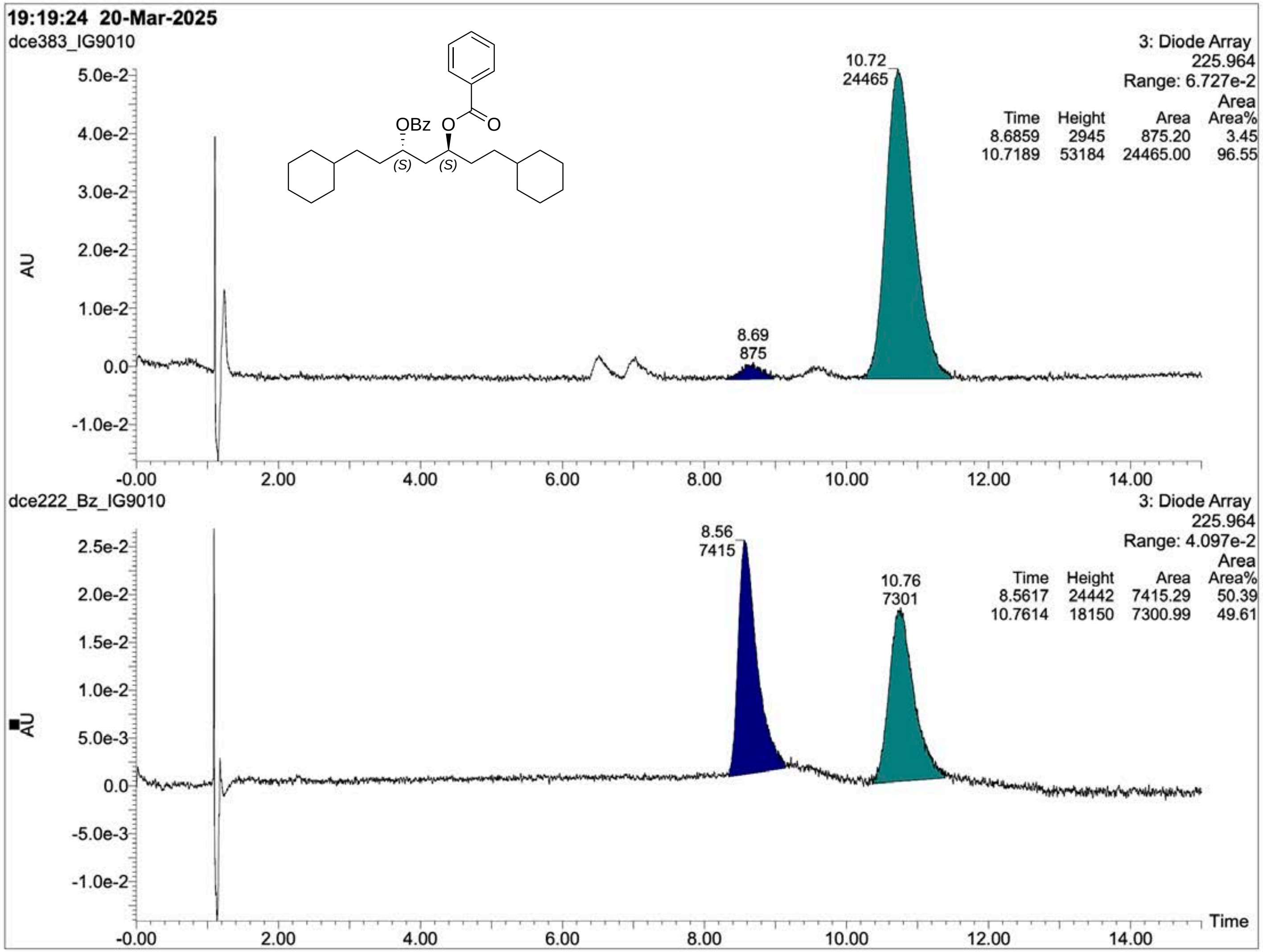

(4*S*,6*S*)-1,9-dicyclohexylnonane-4,6-diyl dibenzoate Number (1j-Bz)

Chiral SFC Analysis CHIRALPAK IK (CO_2:MeOH, 95:05, 2.5 ml min^{-1}, 40 °C, 225 nm) t_R = 6.5 (minor), 7.0 (major) minutes, 40% *ee*.

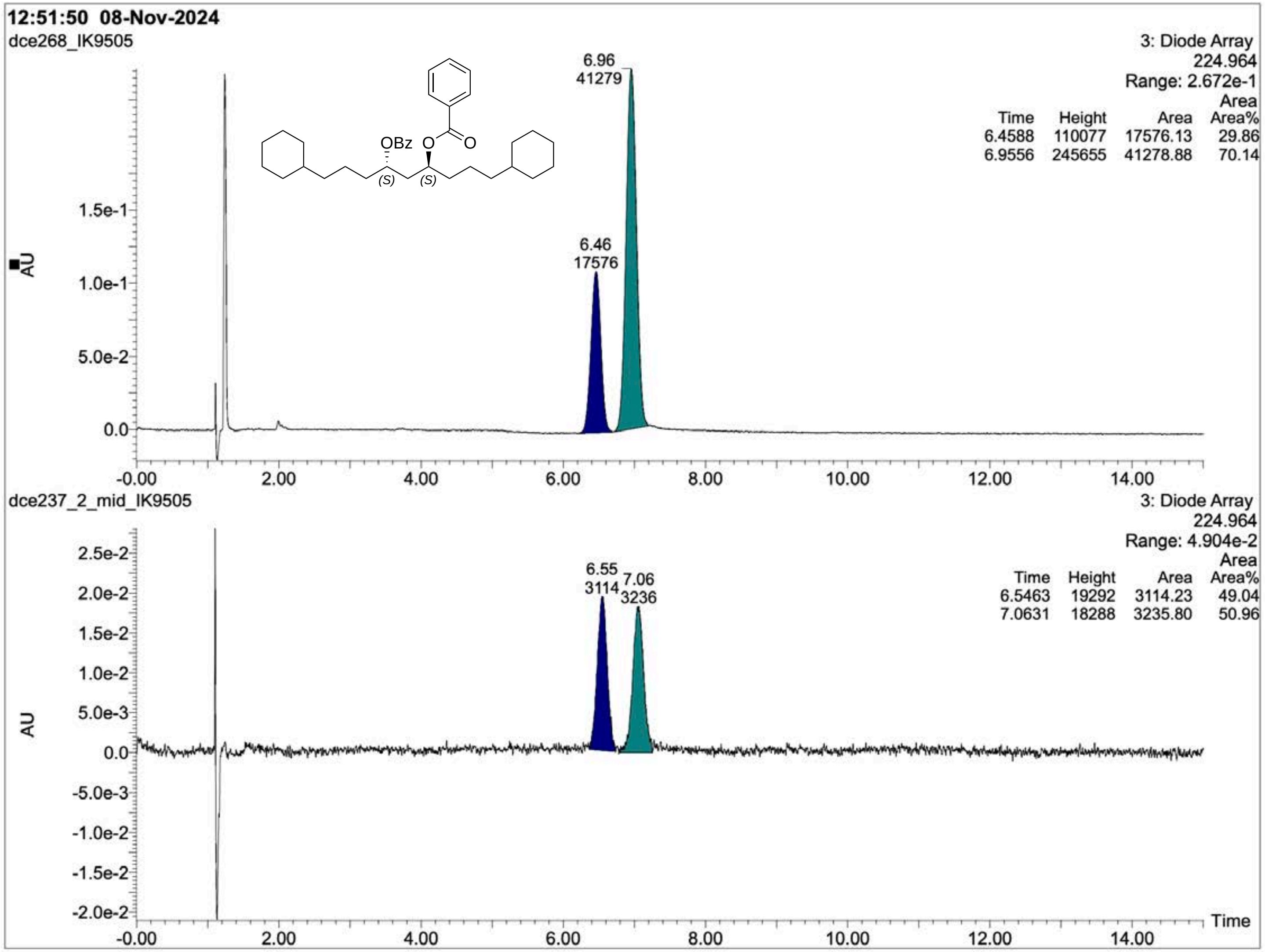

(3S,5S)-1,7-diphenylheptane-3,5-diol (1k)

Chiral SFC Analysis CHIRALPAK IA (CO_2:MeOH, 85:15, 2.5 ml min^{-1}, 40 °C, 204 nm) t_R = 5.0 (major), 5.6 (minor) minutes, 84% *ee*.

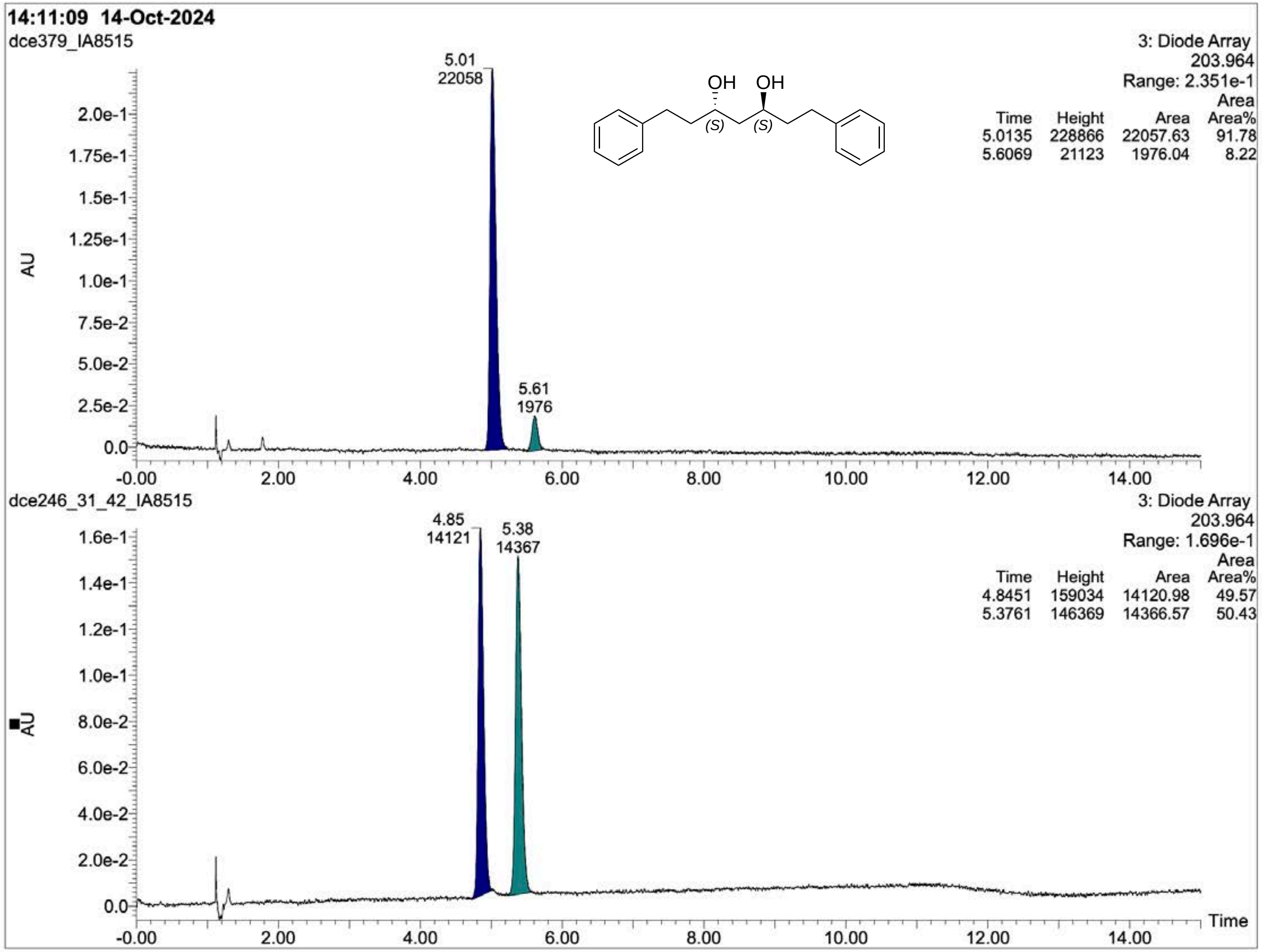

(4S,6S)-1,9-diphenylnonane-4,6-diol (1l)

Chiral HPLC Analysis CHIRALPAK IG (Hexane:iPrOH, 90:10, 1.25 mL min-1, 40 °C) t_R = 9.6 (major), 11.7 (minor) minutes, 85% *ee*

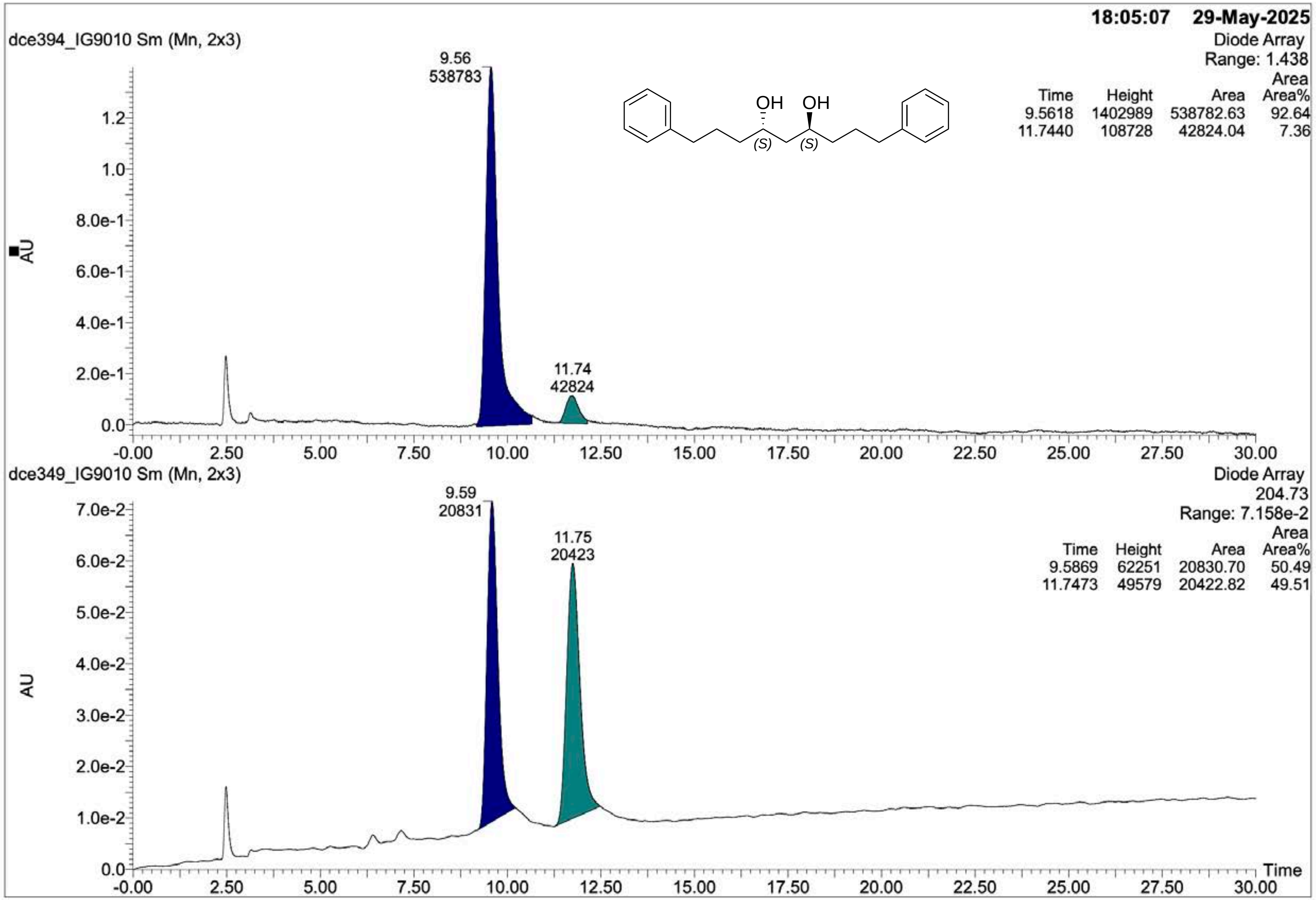

Reaction starting from syn-1,3-diol (1d)

(5*S*,7*S*)-undecane-5,7-diyl dibenzoate (1d-Bz)

Chiral SFC Analysis CHIRALPAK IE (CO_2:MeOH, 97:03, 2.5 ml min^{-1}, 40 °C, 237 nm) t_R = 11.1 (major) minutes, 99% *ee*.

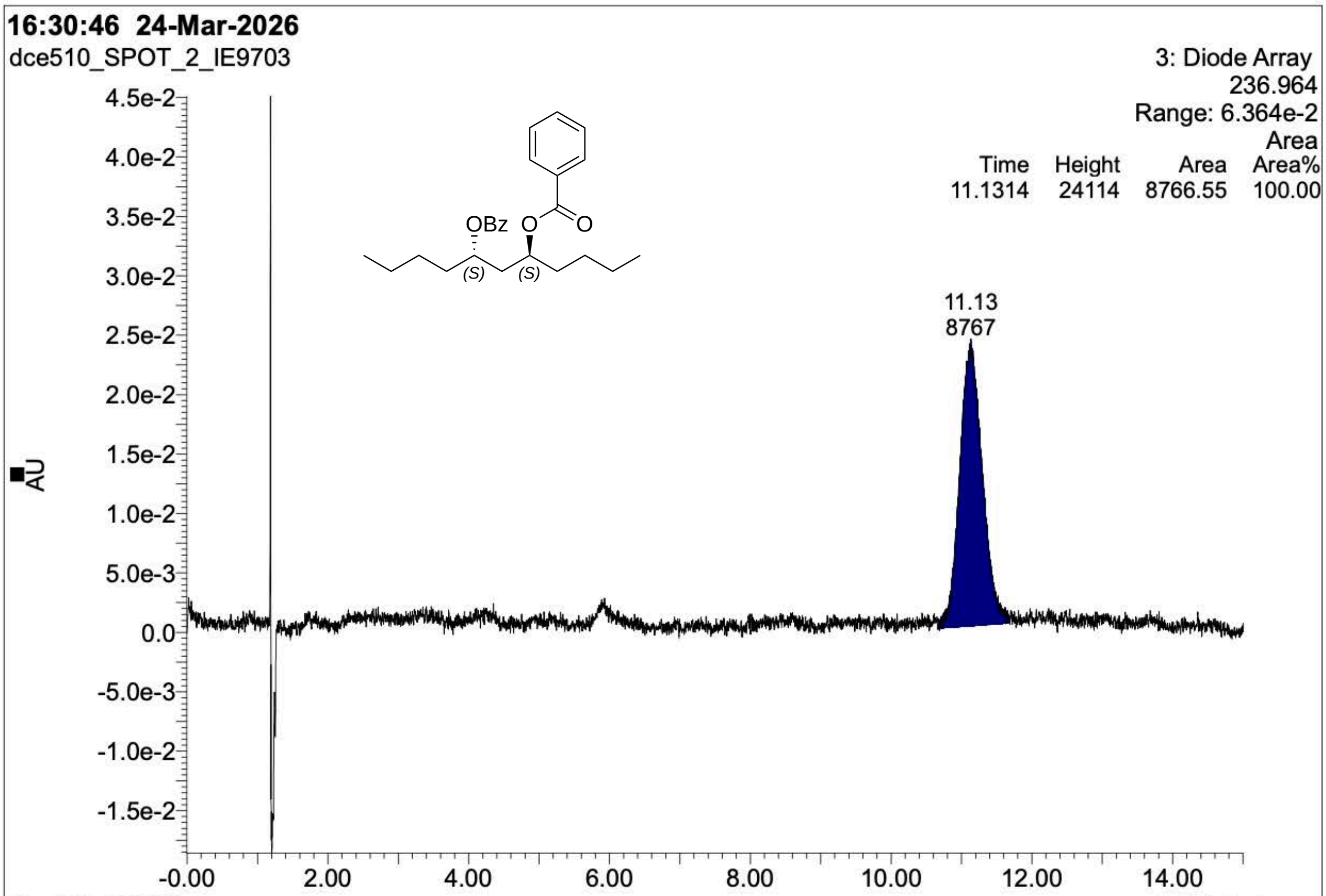

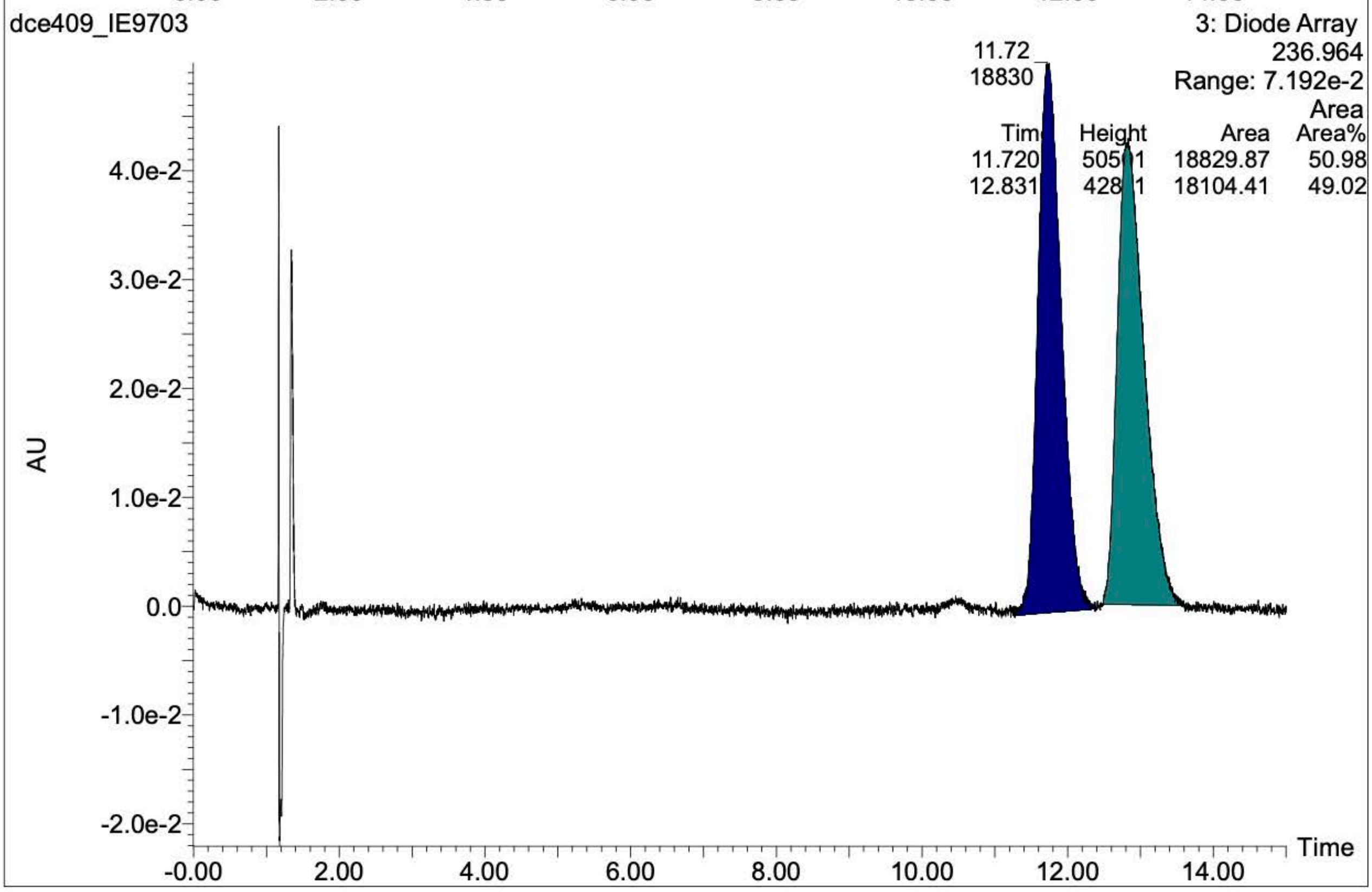

Deuterium Study from *rac*-1d

(5*S*,7*S*)-undecane-5,7-diyl-5,6,6,7-d_4 dibenzoate (1d-Bz)

Chiral SFC Analysis CHIRALPAK IE (CO_2:MeOH, 97:03, 2.5 ml min^{-1}, 40 °C, 231 nm) t_R = 10.8 (major) 12.0 (minor) minutes, 91% *ee*.

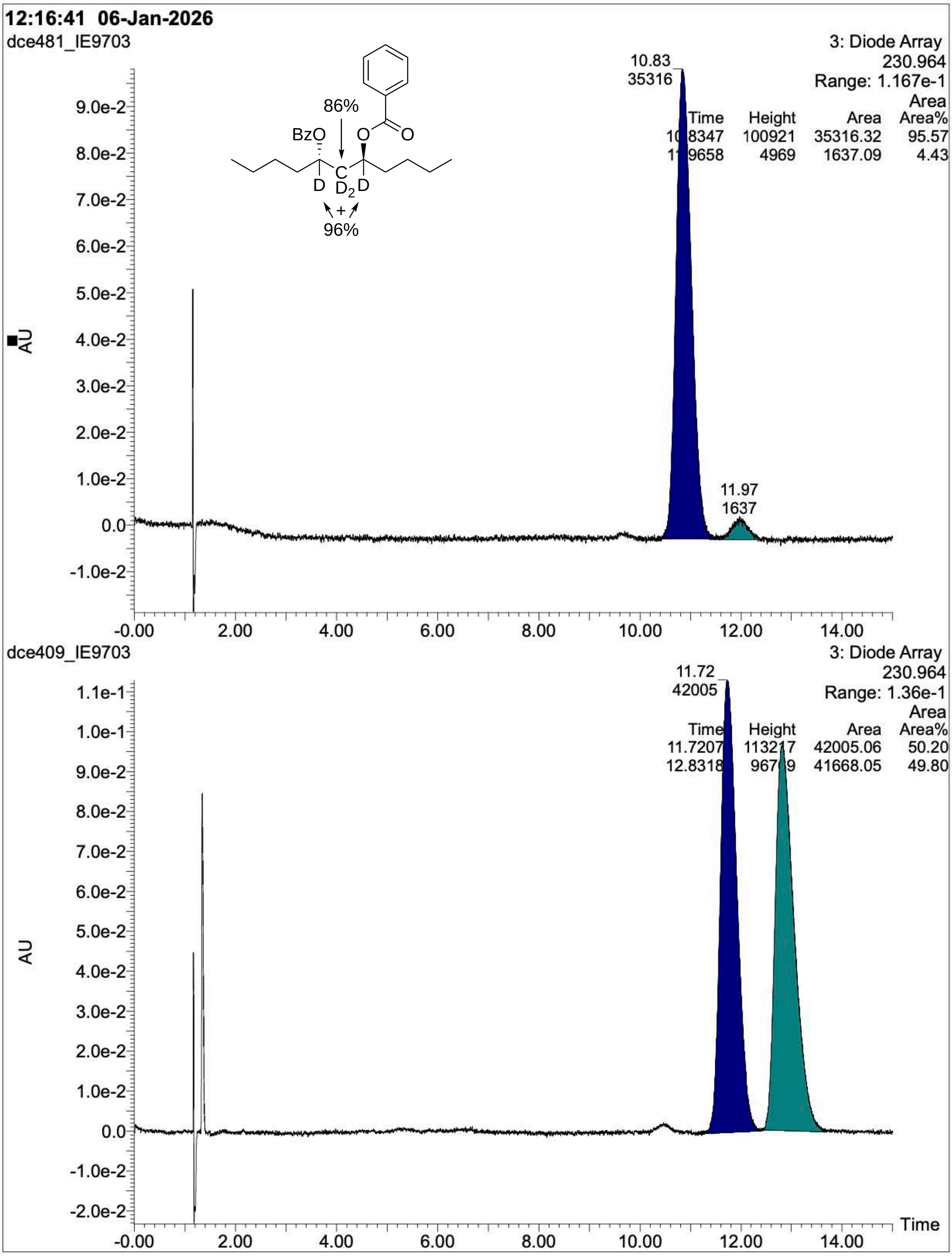

Time	Height	Area	Area%
10.8347	100921	35316.32	95.57
11.9658	4969	1637.09	4.43

Time	Height	Area	Area%
11.7207	113217	42005.06	50.20
12.8318	96769	41668.05	49.80

Deuterium Study from *meso*-1d

(5*S*,7*S*)-undecane-5,7-diyl-5,6,6,7-d_4 dibenzoate (1d-Bz)

Chiral SFC Analysis CHIRALPAK IE (CO_2:MeOH, 97:03, 2.5 ml min^{-1}, 40 °C, 231 nm) t_R = 11.3 (major) minutes, 99% *ee*.

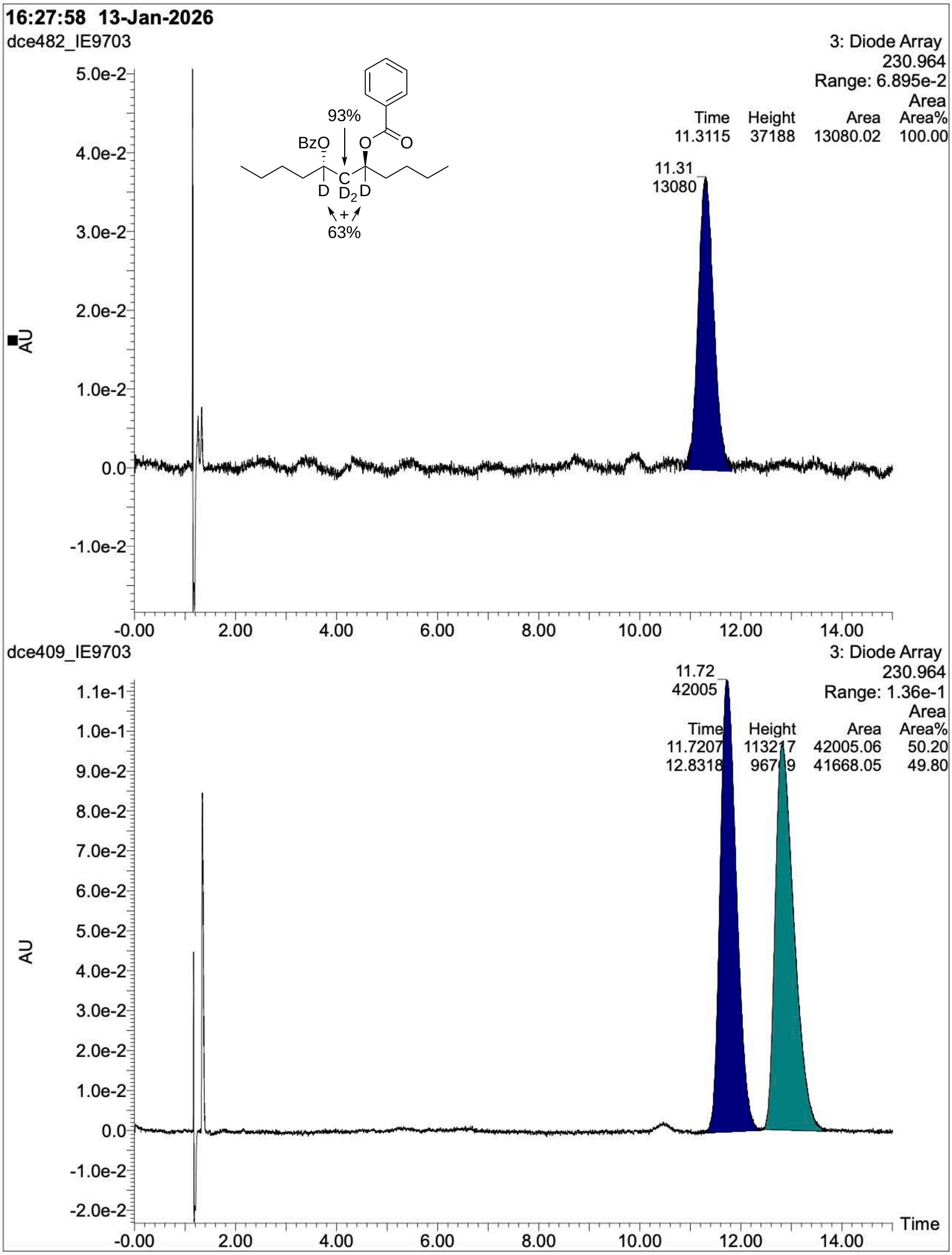

Giese reaction addition (Scheme 3D, upper) starting from *rac*-1d:

(5*S*,7*S*)-undecane-5,7-diyl dibenzoate (1d-Bz)

Chiral SFC Analysis CHIRALPAK IE (CO_2:MeOH, 97:03, 2.5 ml min^{-1}, 40 °C, 225 nm) t_R = 11.4 (major), 12.5 (minor) minutes, 57% *ee*.

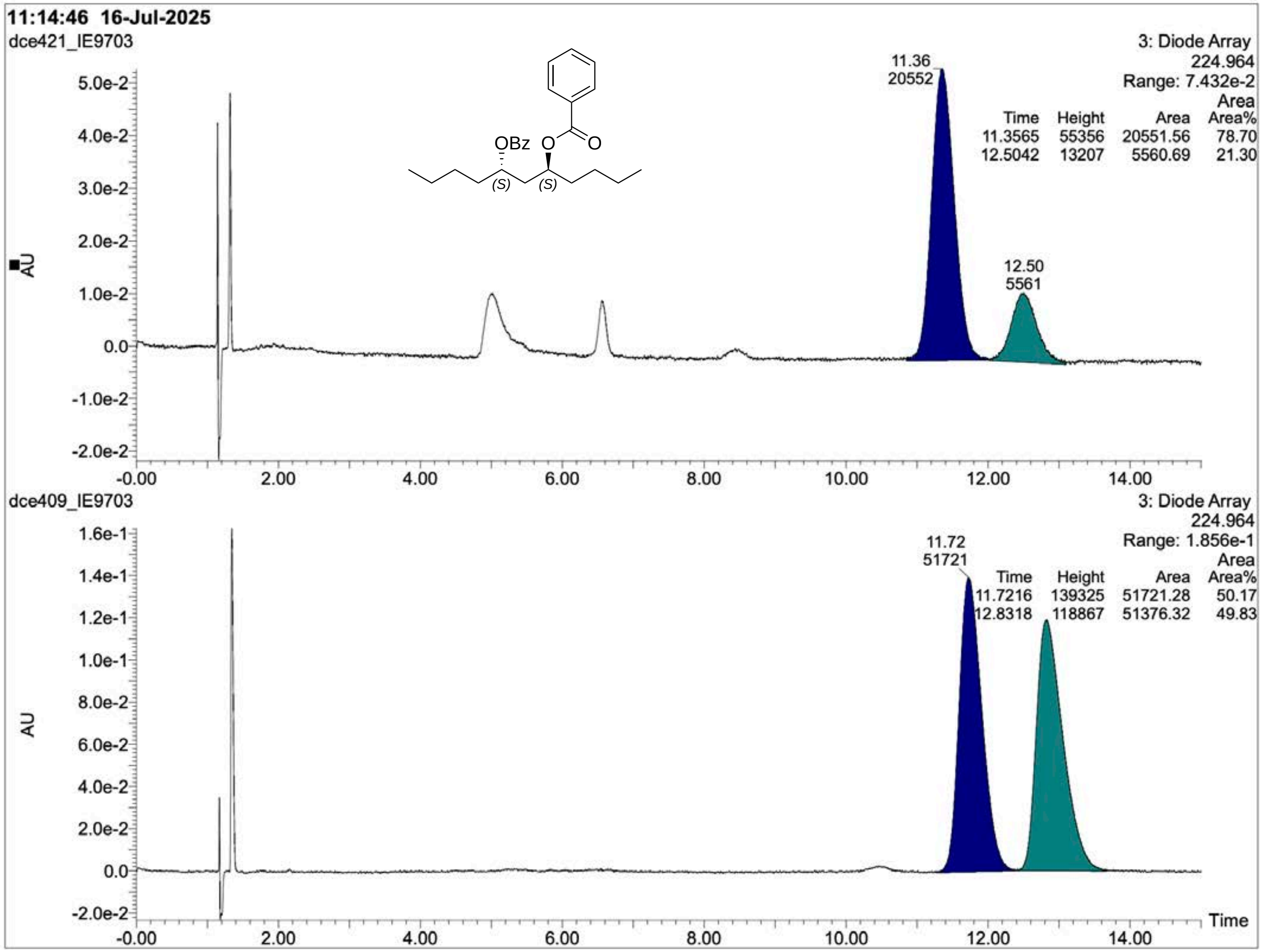

(6*R*)-4-butyl-4,6-dihydroxy-*N*-phenyldecanamide (2b, DR-1)

Chiral SFC Analysis CHIRALPAK IC (CO_2:MeOH, 90:10, 2.5 ml min^{-1}, 40 °C, 240 nm) t_R = 10.8 (major) minutes, 99% *ee*.

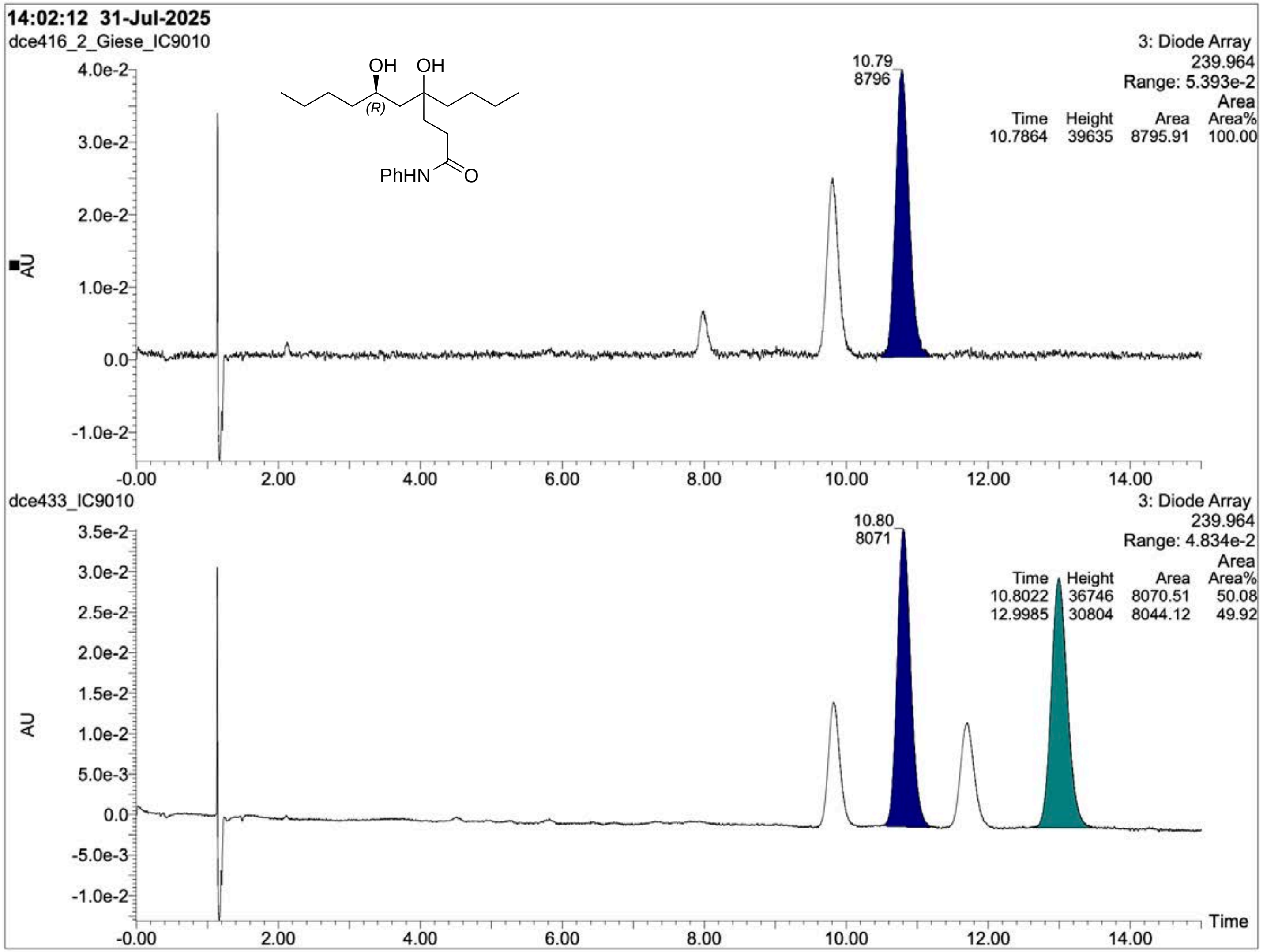

(6*R*)-4-butyl-4,6-dihydroxy-*N*-phenyldecanamide (2b, DR-2)

Chiral SFC Analysis CHIRALPAK IC (CO_2:MeOH, 90:10, 2.5 ml min^{-1}, 40 °C, 240 nm) t_R = 9.8 (major) minutes, 99% *ee*.

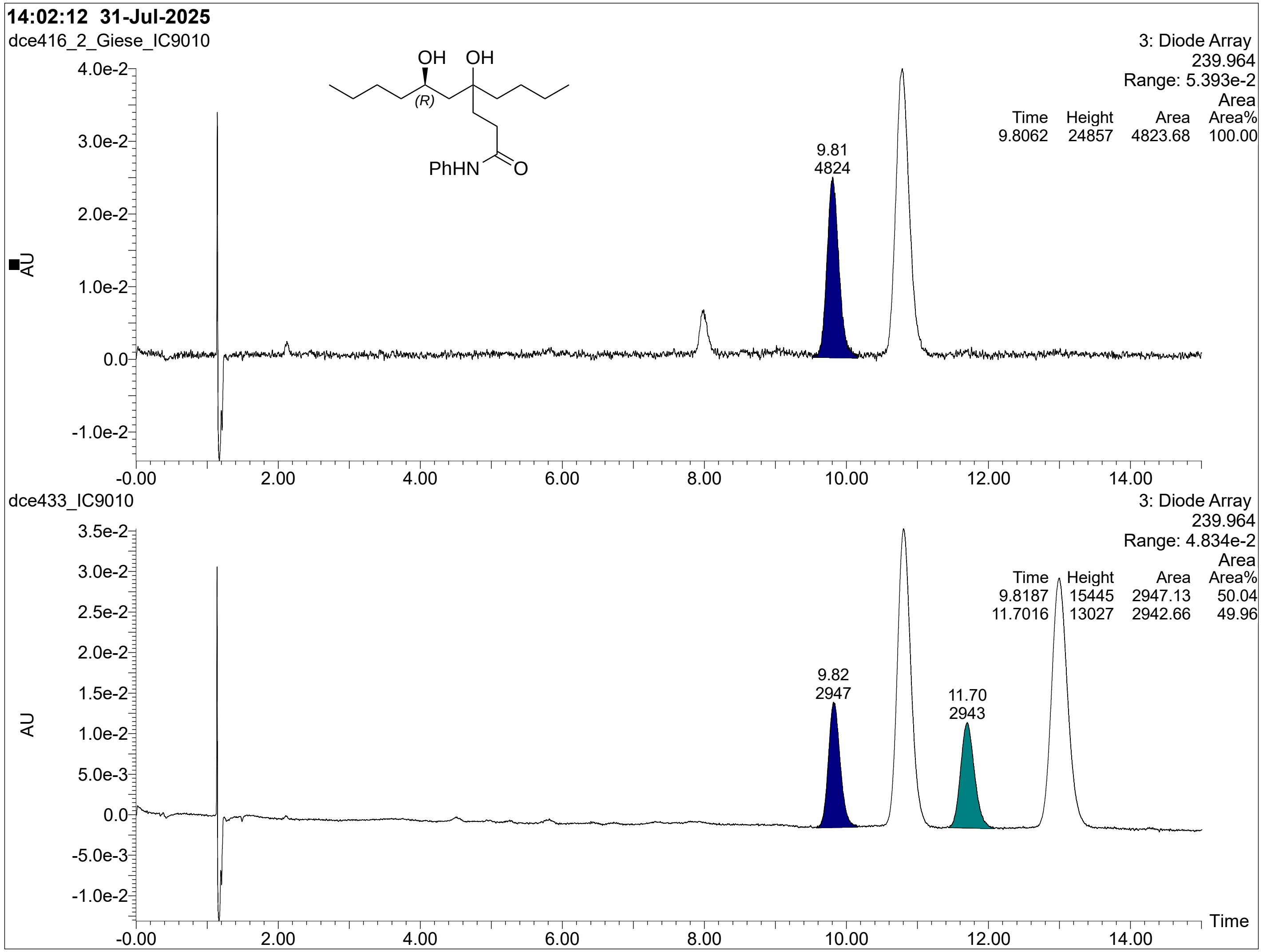

Oxidation reaction with DIAD starting from *rac*-1d

(5*S*,7*S*)-undecane-5,7-diyl dibenzoate (1d-Bz)

Chiral SFC Analysis CHIRALPAK IE (CO_2:MeOH, 97:03, 2.5 ml min^{-1}, 40 °C, 225 nm) t_R = 11.1 (major) minutes, 99% *ee*.

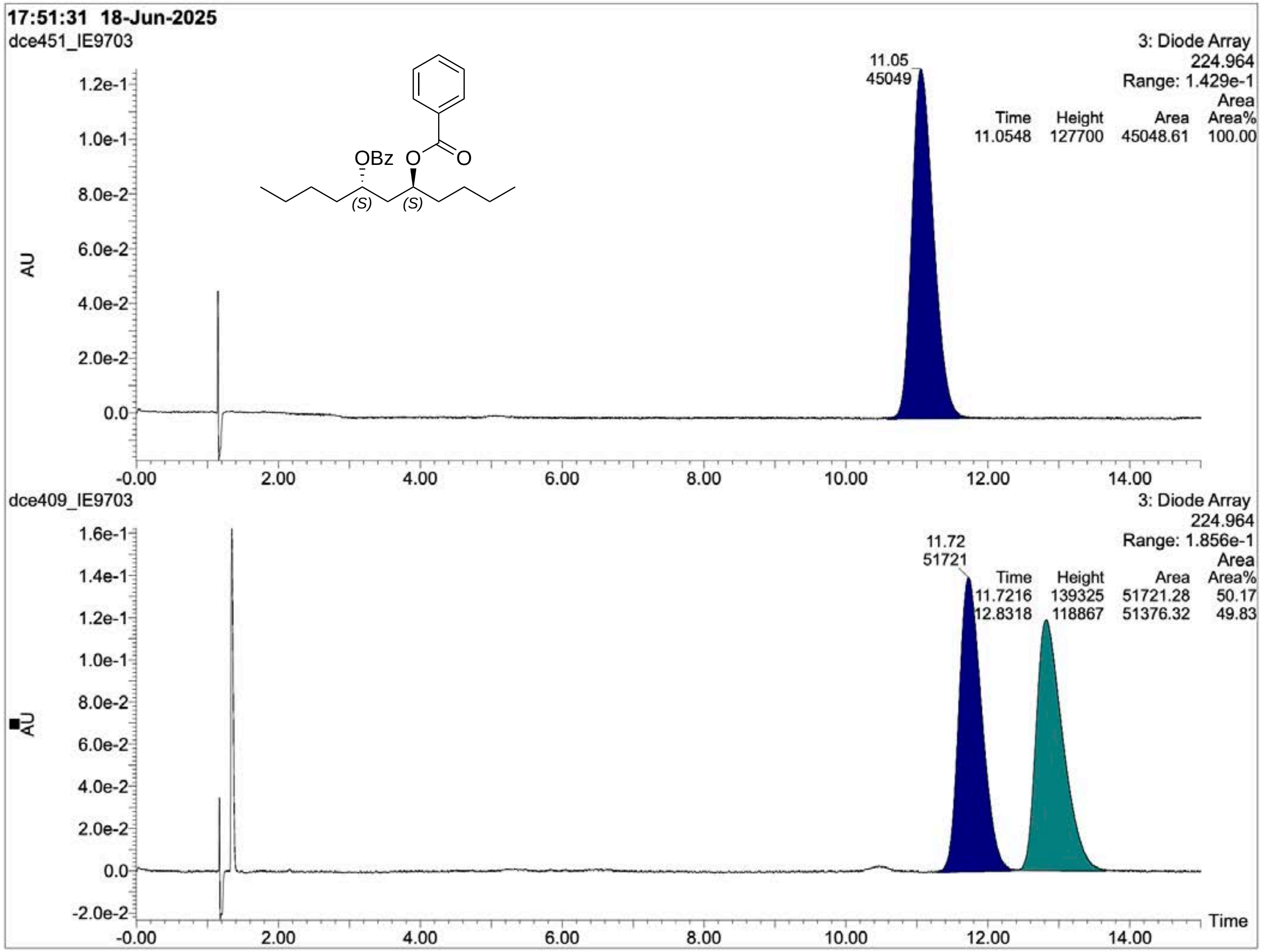

(*R*)-7-((*tert*-butyldiphenylsilyl)oxy)undecan-5-one (2c-TBDPS)

Chiral SFC Analysis CHIRALPAK IA (CO_2:MeOH, 99:01, 2.5 ml min^{-1}, 40 °C, 220 nm) t_R = 5.3 (major), 6.0 (minor) minutes, 80% *ee*.

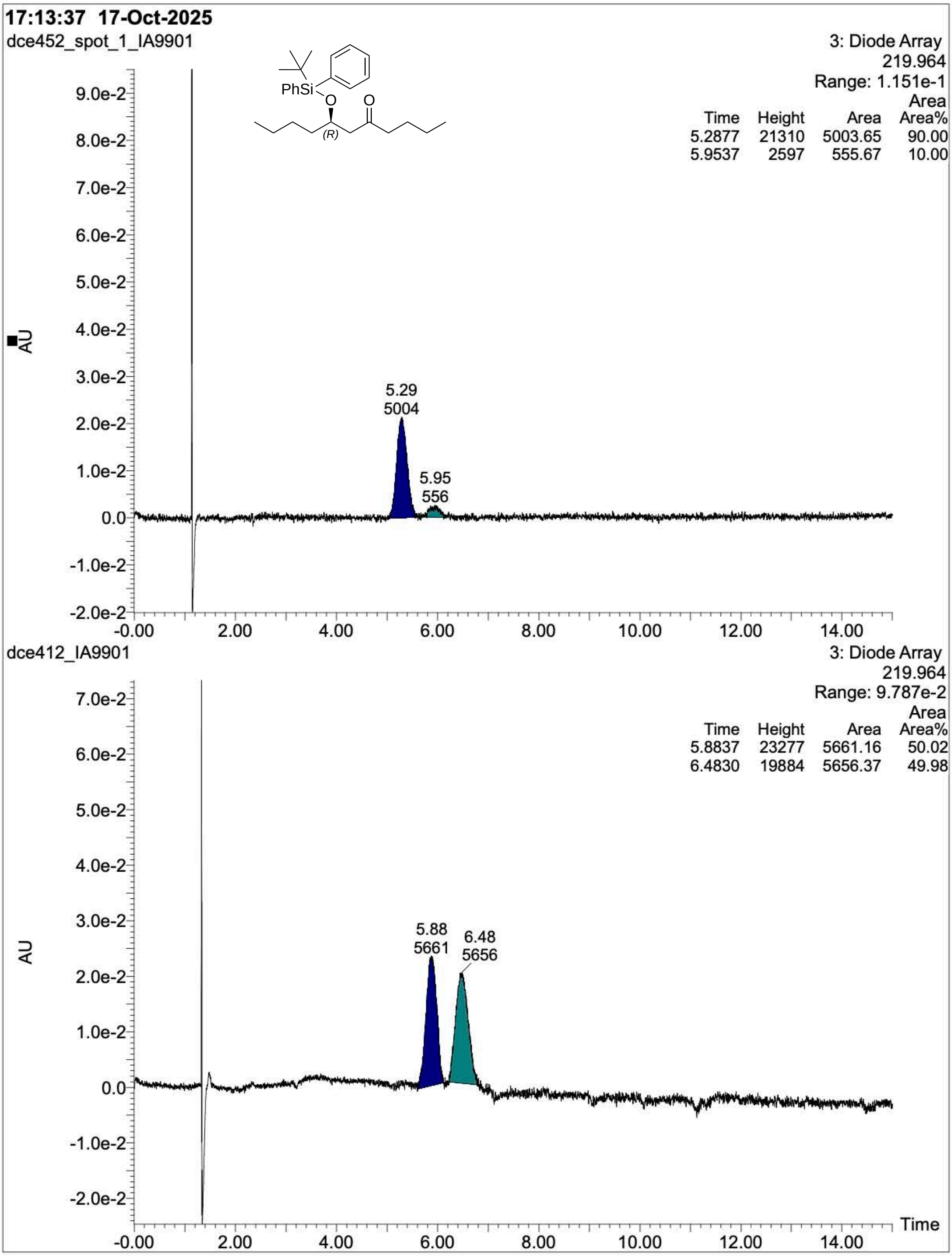

Oxidation reaction with DIAD starting from *rac*-1e

(4*S*,6*S*)-2,8-dimethylnonane-4,6-diyl dibenzoate (1e-Bz)

Chiral SFC Analysis CHIRALPAK IG (CO_2:MeOH, 97:03, 2.5 ml min^{-1}, 40 °C, 225 nm) t_R = 4.7 (minor), 5.2 (major) minutes, 97% *ee*.

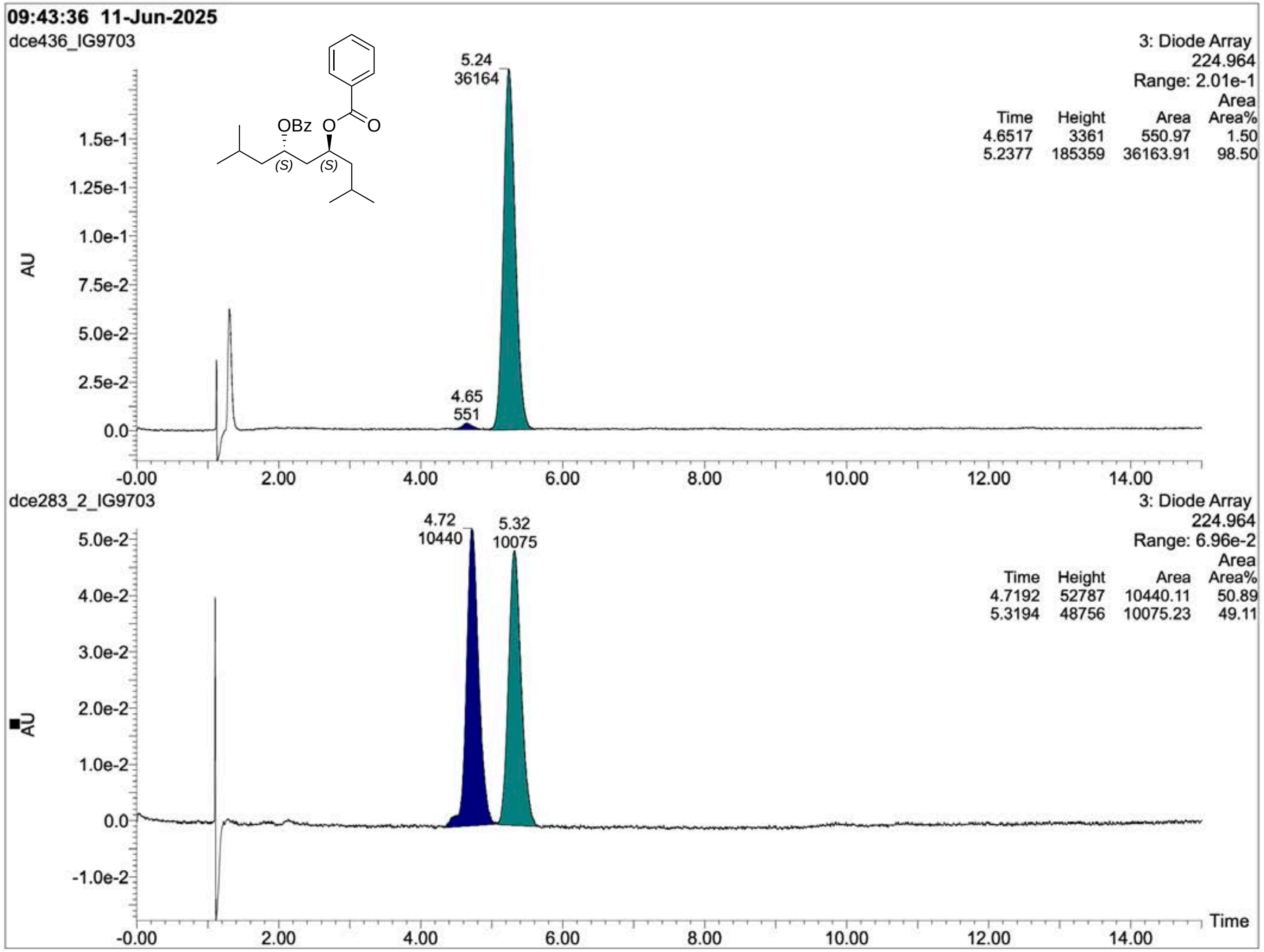

Time	Height	Area	Area%
4.6517	3361	550.97	1.50
5.2377	185359	36163.91	98.50

Time	Height	Area	Area%
4.7192	52787	10440.11	50.89
5.3194	48756	10075.23	49.11

(*R*)-6-((*tert*-butyldiphenylsilyl)oxy)-2,8-dimethylnonan-4-one (2d-TBDPS)

Chiral SFC Analysis CHIRALPAK IJ (CO_2:MeOH, 999:001, 2.5 ml min^{-1}, 40 °C, 214 nm) t_R = 2.0 (major), 2.2 (minor) minutes, 92% *ee*.

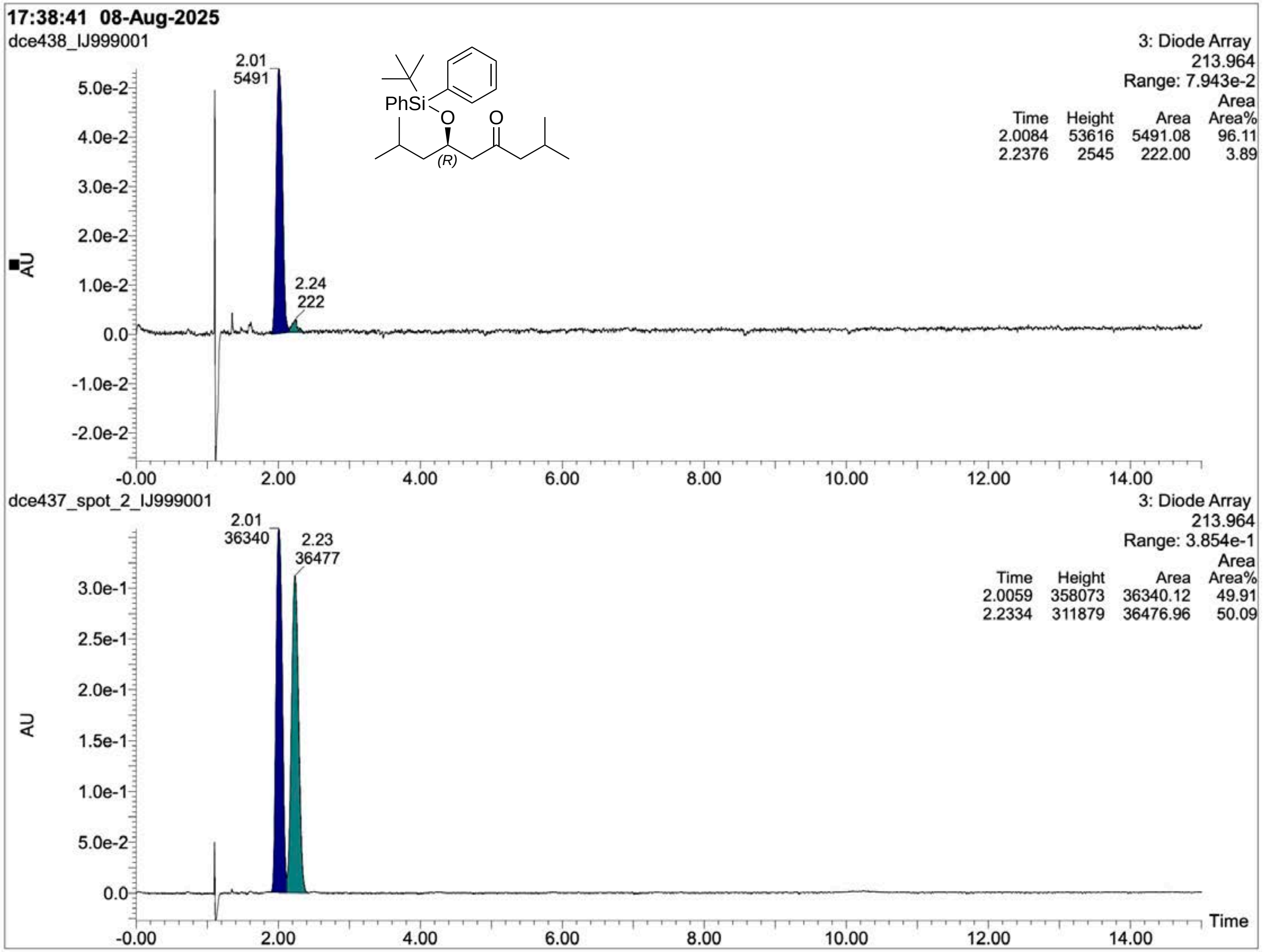

Oxidation reaction with DIAD starting from *rac*-1k

(3S,5S)-1,7-diphenylheptane-3,5-diol (1k)

Chiral SFC Analysis CHIRALPAK IA (CO_2:MeOH, 85:15, 2.5 ml min^{-1}, 40 °C, 204 nm) t_R = 4.9 (major), 5.5 (minor) minutes, 92% *ee*.

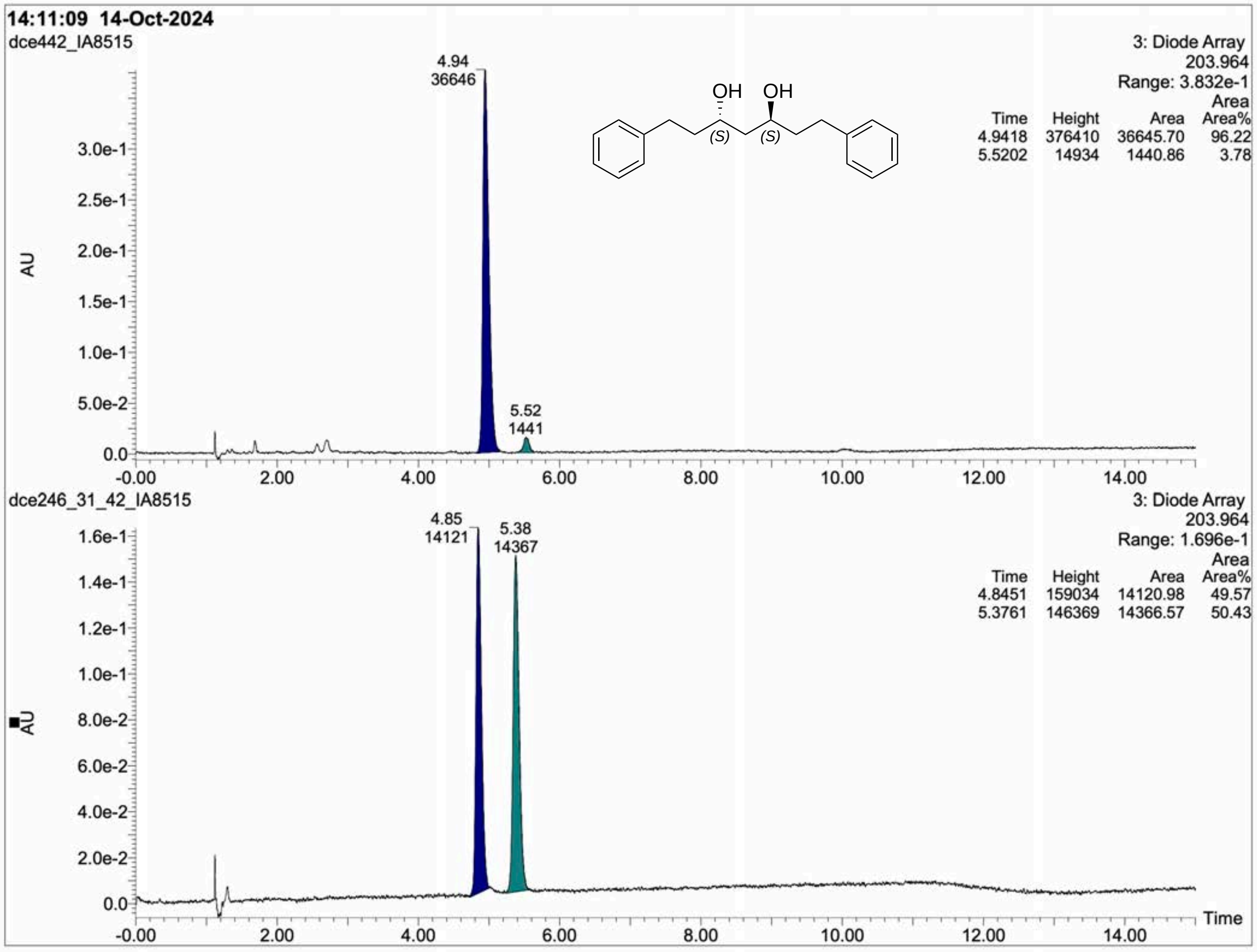

(*R*)-5-hydroxy-1,7-diphenylheptan-3-one (2e)

Chiral SFC Analysis CHIRALPAK IJ (CO_2:MeOH, 85:15, 2.5 ml min^{-1}, 40 °C, 206 nm) t_R = 6.5 (minor), 7.0 (major) minutes, 93% *ee*.

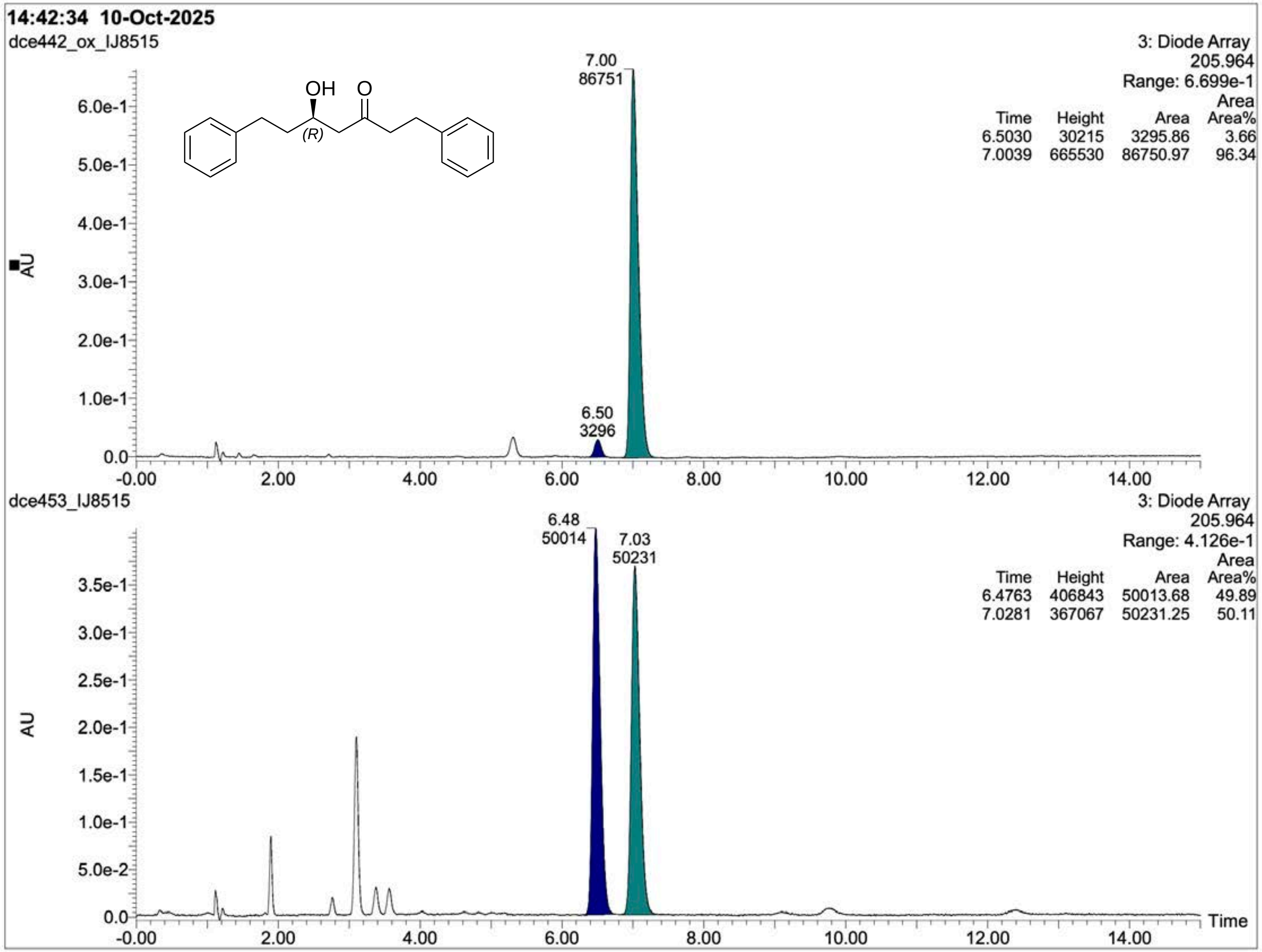

Access to Opposite Enantiomer

(4*R*,6*R*)-2,8-dimethylnonane-4,6-diyl dibenzoate ((*R*,*R*)-1d-Bz)

Chiral SFC Analysis CHIRALPAK IE (CO_2:MeOH, 97:03, 2.5 ml min^{-1}, 40 °C, 225 nm) t_R = 12.2 (major) minutes, -99% *ee*.

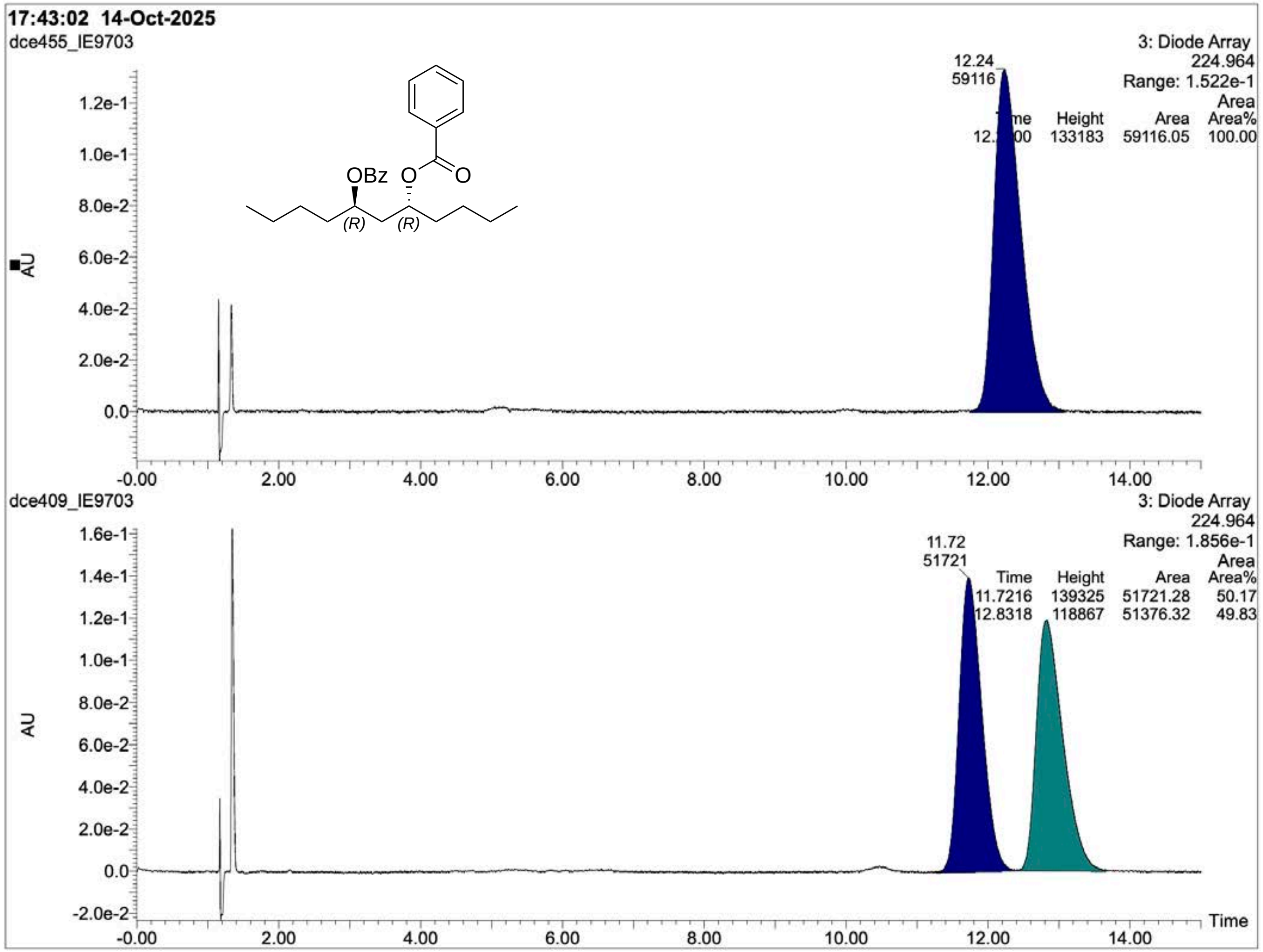

1 mmol scale up of symmetrical 1,3-diol (1d)

(5*S*,7*S*)-undecane-5,7-diyl dibenzoate (1d-Bz)

Chiral SFC Analysis CHIRALPAK IE (CO_2:MeOH, 97:03, 2.5 ml min^{-1}, 40 °C, 222 nm) t_R = 10.9 (major) minutes, 99% *ee*.

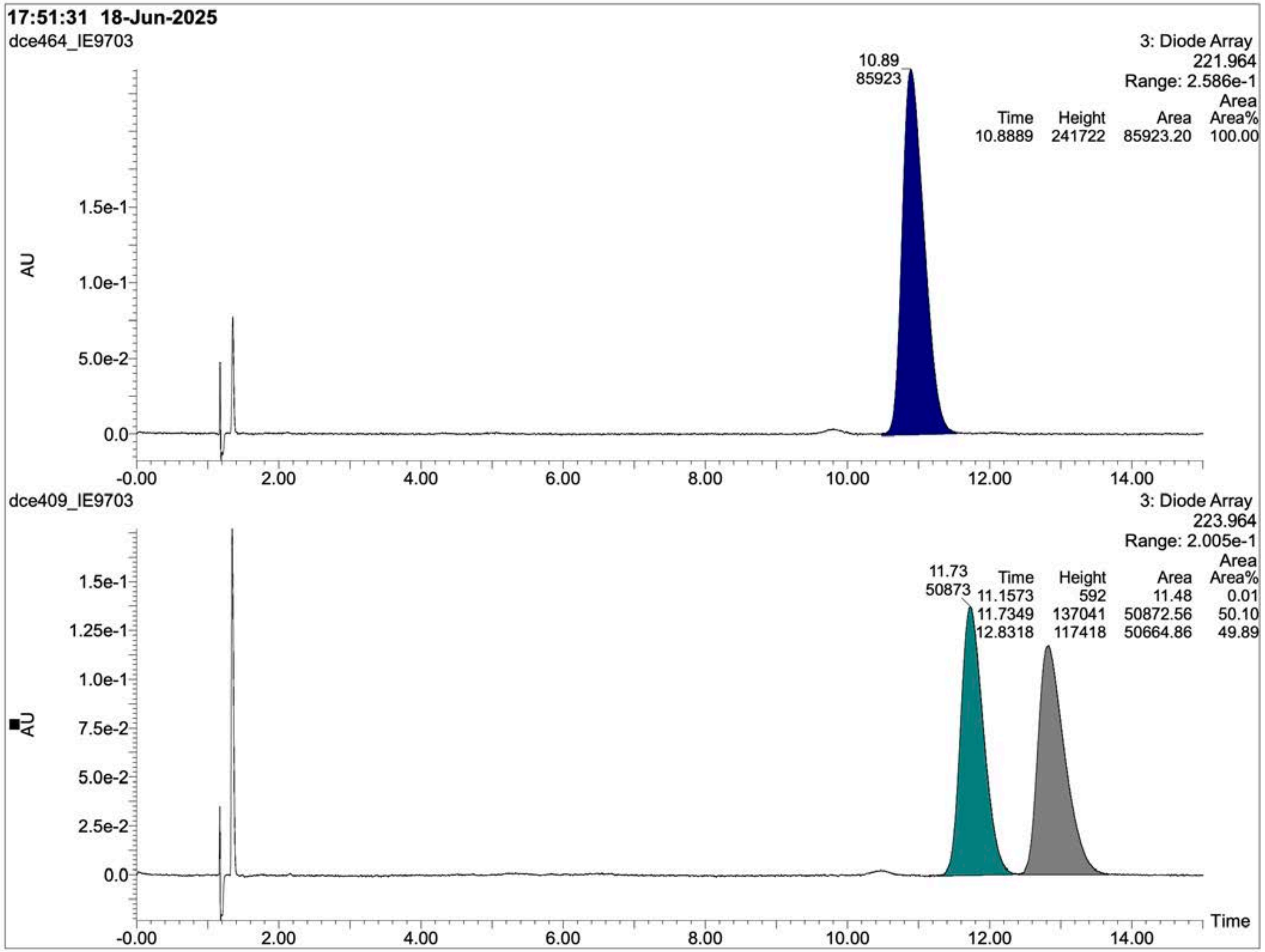

Time	Height	Area	Area Area%
10.8889	241722	85923.20	100.00

Time	Height	Area	Area Area%
11.1573	592	11.48	0.01
11.7349	137041	50872.56	50.10
12.8318	117418	50664.86	49.89

(*R*)-7-((*tert*-butyldiphenylsilyl)oxy)undecan-5-one (2c-TBDPS)

Chiral SFC Analysis CHIRALPAK IA (CO_2:MeOH, 99:01, 2.5 ml min^{-1}, 40 °C, 216 nm) t_R = 5.5 (minor), 6.3 (major) minutes, 57% *ee*.

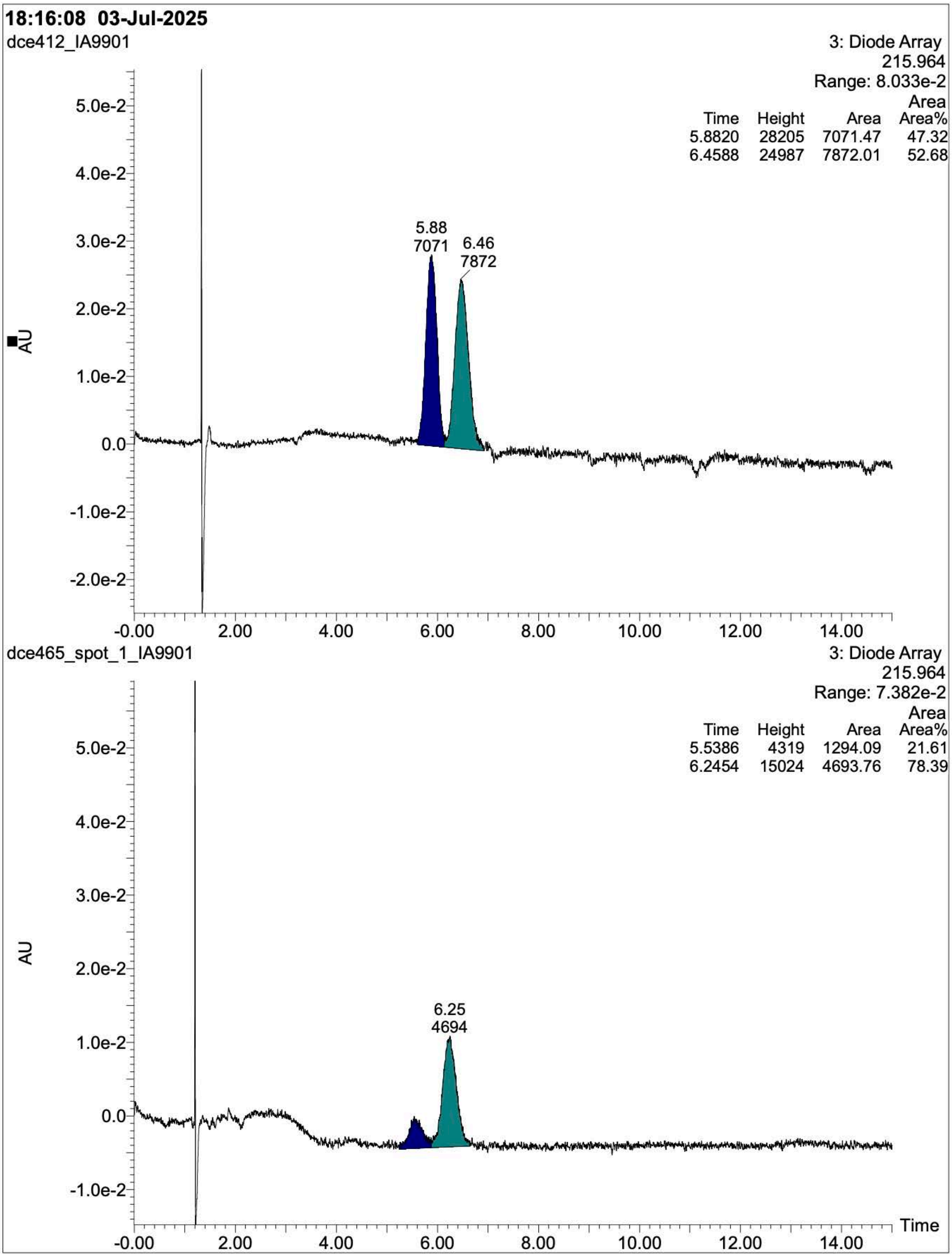

(*S*,*S*)-1-Phenylpentane-2,4-diol (*S*,*S*-3a)

Chiral SFC Analysis: CHIRALPAK IC (CO_2:MeOH, 96:4, 2.5 mL min^{-1}, 40 °C, 205 nm) t_R = 12.0 (major), 12.4 (minor) min, 99% *ee*.

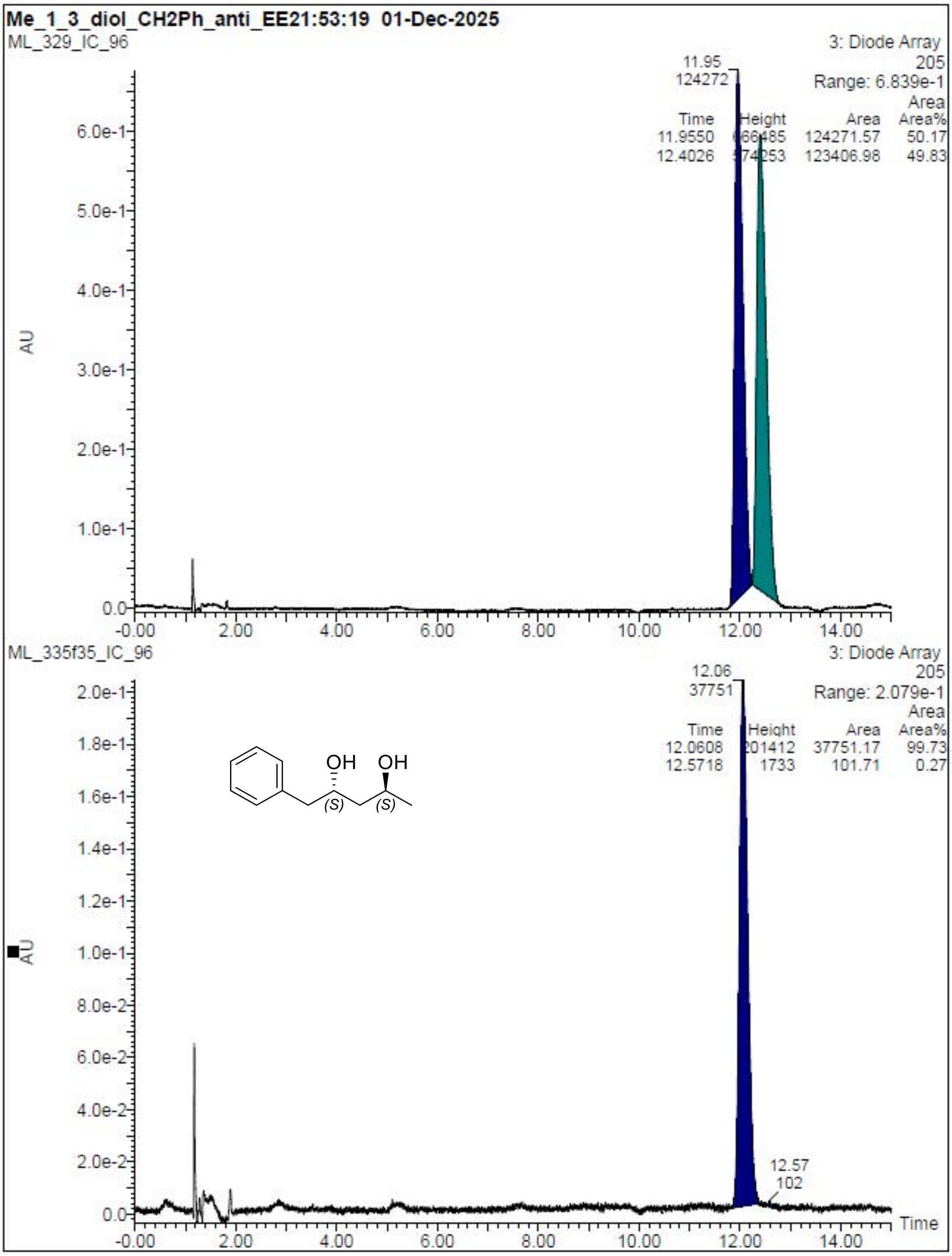

(2*R*,4*S*)-1-Phenylpentane-2,4-diol (2*R*,4*S*-3a)

Chiral SFC Analysis: CHIRALPAK IK (CO_2:MeOH, 93:7, 2.5 mL min^{-1}, 40 °C, 205 nm) t_R = 6.6 (minor), 8.0 (major) min, 65% *ee*.

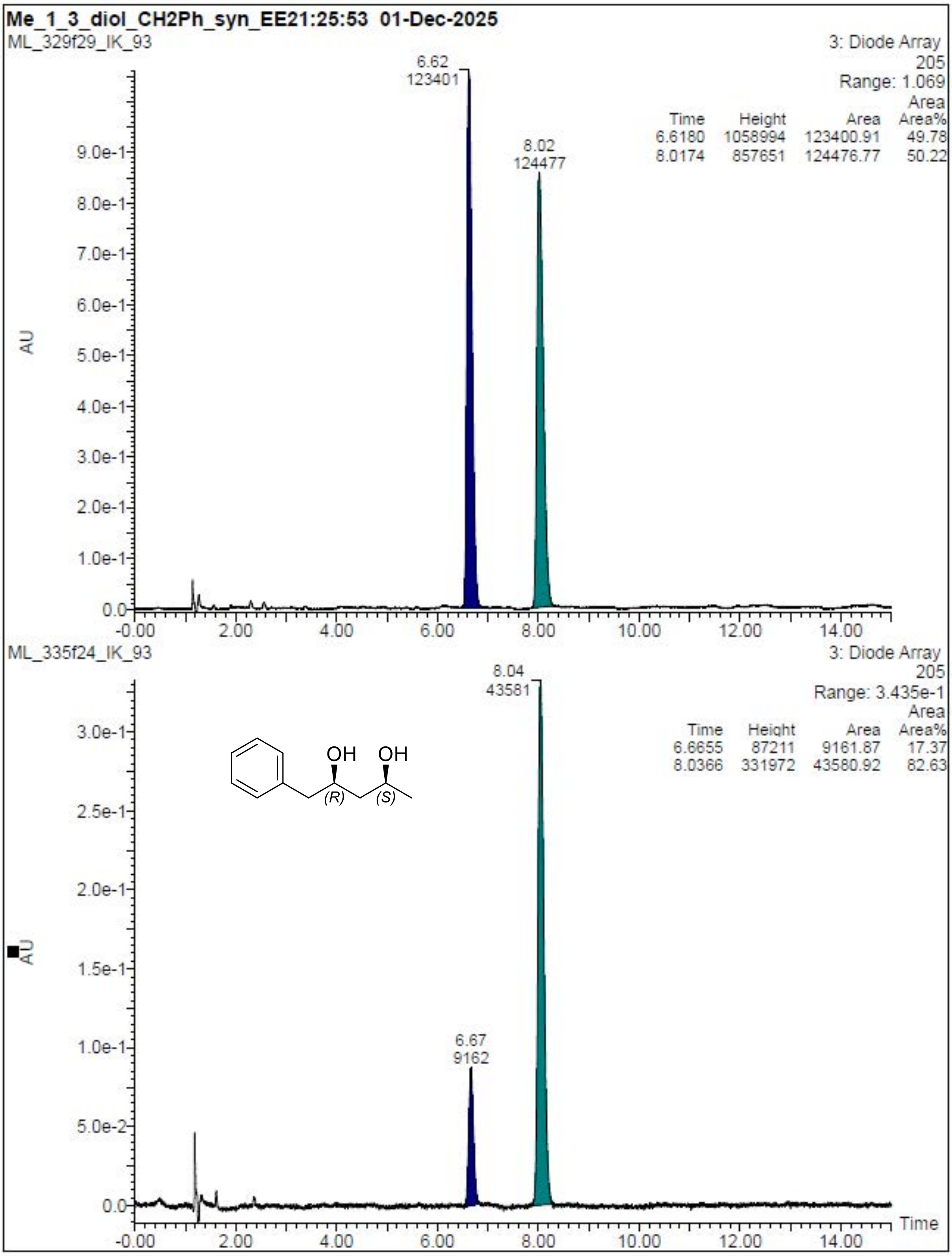

(*S*,*S*)-1-(4-Methoxyphenyl)pentane-2,4-diol (*S*,*S*-3b)

Chiral SFC Analysis: CHIRALPAK IC (CO_2:MeOH, 94:6, 2.5 mL min^{-1}, 40 °C, 223 nm) t_R = 11.6 (minor), 12.2 (major) min, 94% *ee*.

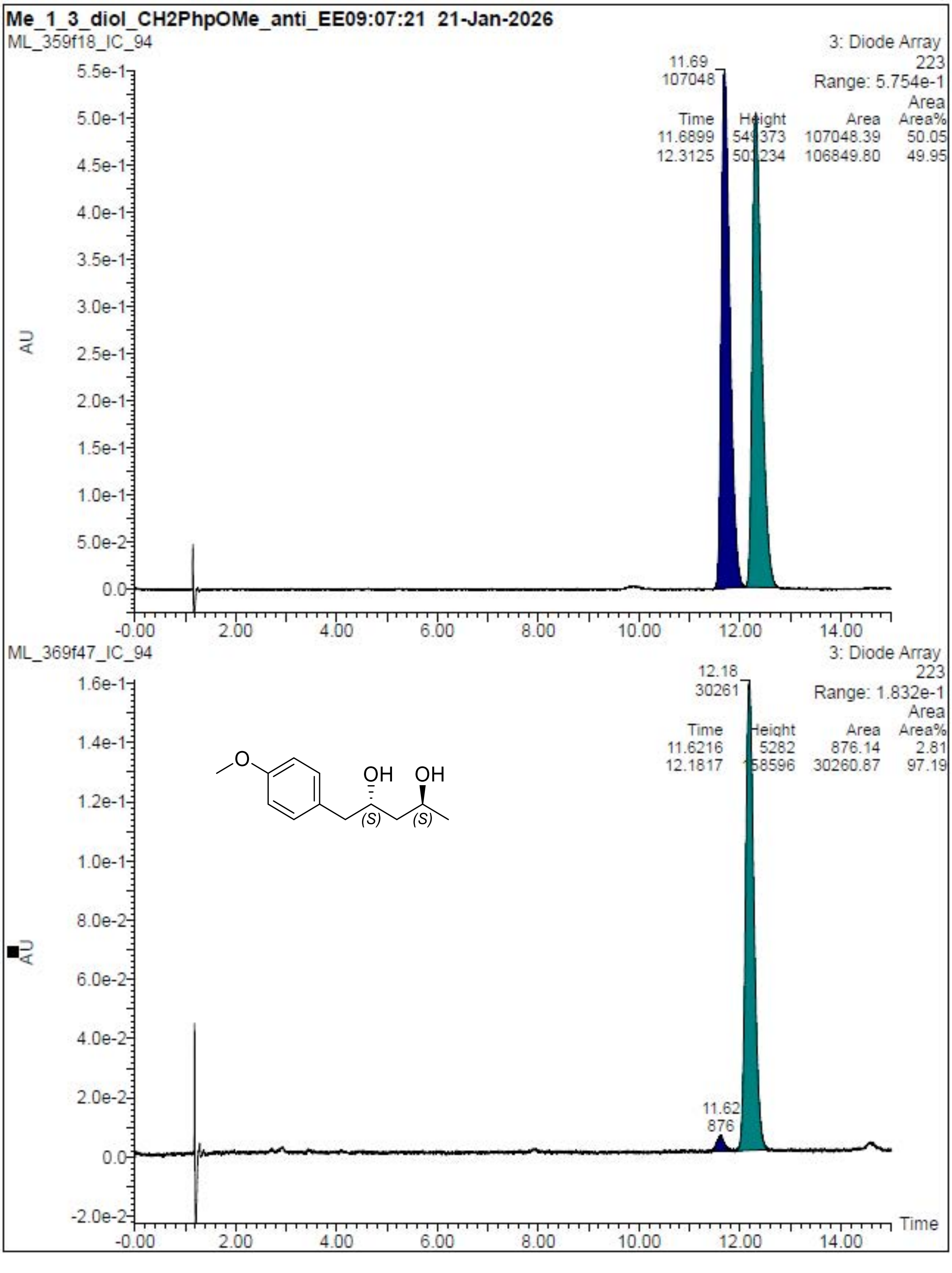

(2*R*,4*S*)-1-(4-Methoxyphenyl)pentane-2,4-diol (2*R*,4*S*-3b)

Chiral SFC Analysis: CHIRALPAK IK (CO_2:MeOH, 93:7, 2.5 mL min^{-1}, 40 °C, 223 nm) t_R = 10.8 (minor), 11.5 (major) min, 65% *ee*.

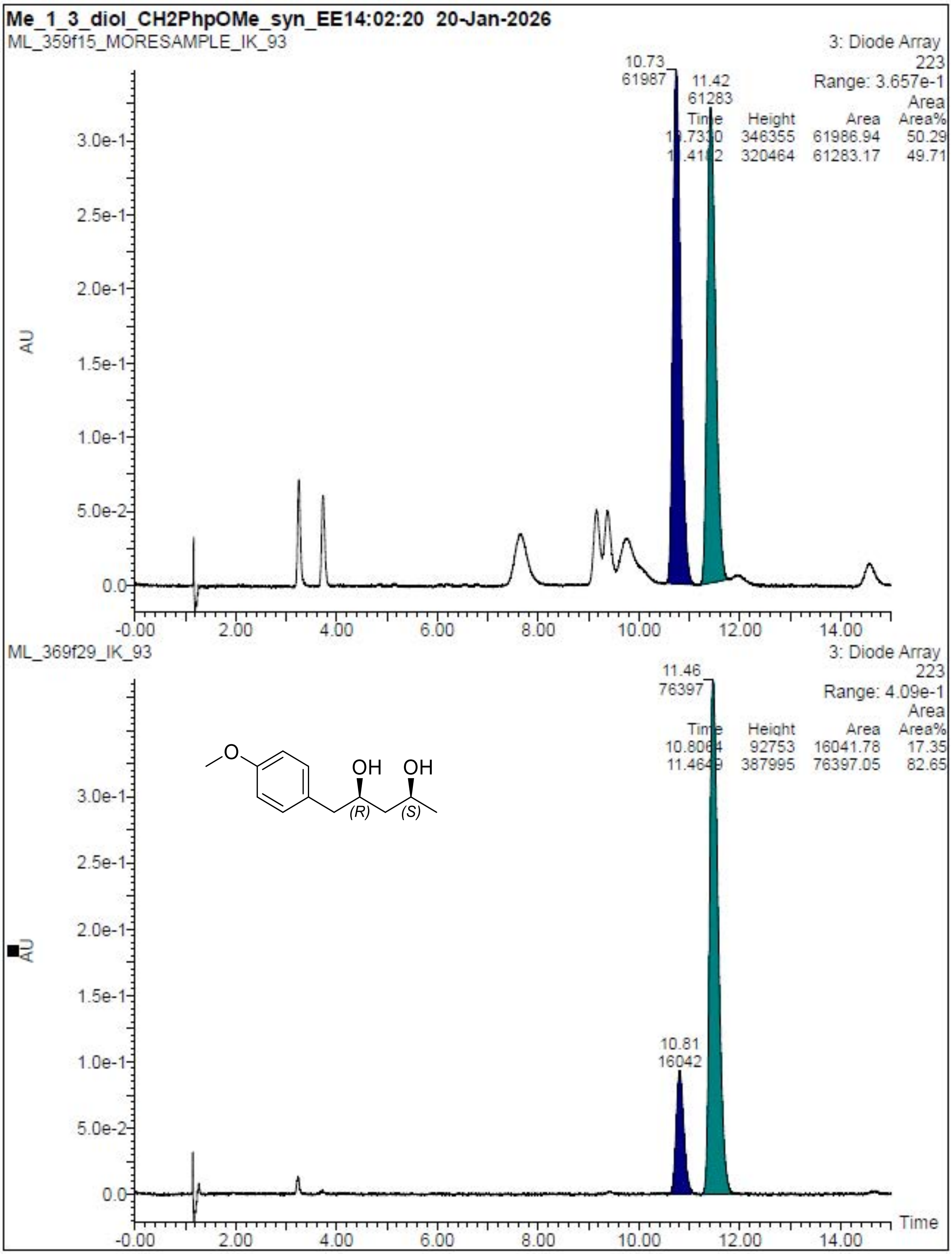

(*S*,*S*)-1-(Naphthalen-1-yl)pentane-2,4-diol (*S*,*S*-3c)

Chiral SFC Analysis: CHIRALPAK IK (CO_2:MeOH, 91:9, 2.5 mL min^{-1}, 40 °C, 280 nm) t_R = 9.9 (major), 11.2 (minor) min, 98% *ee*.

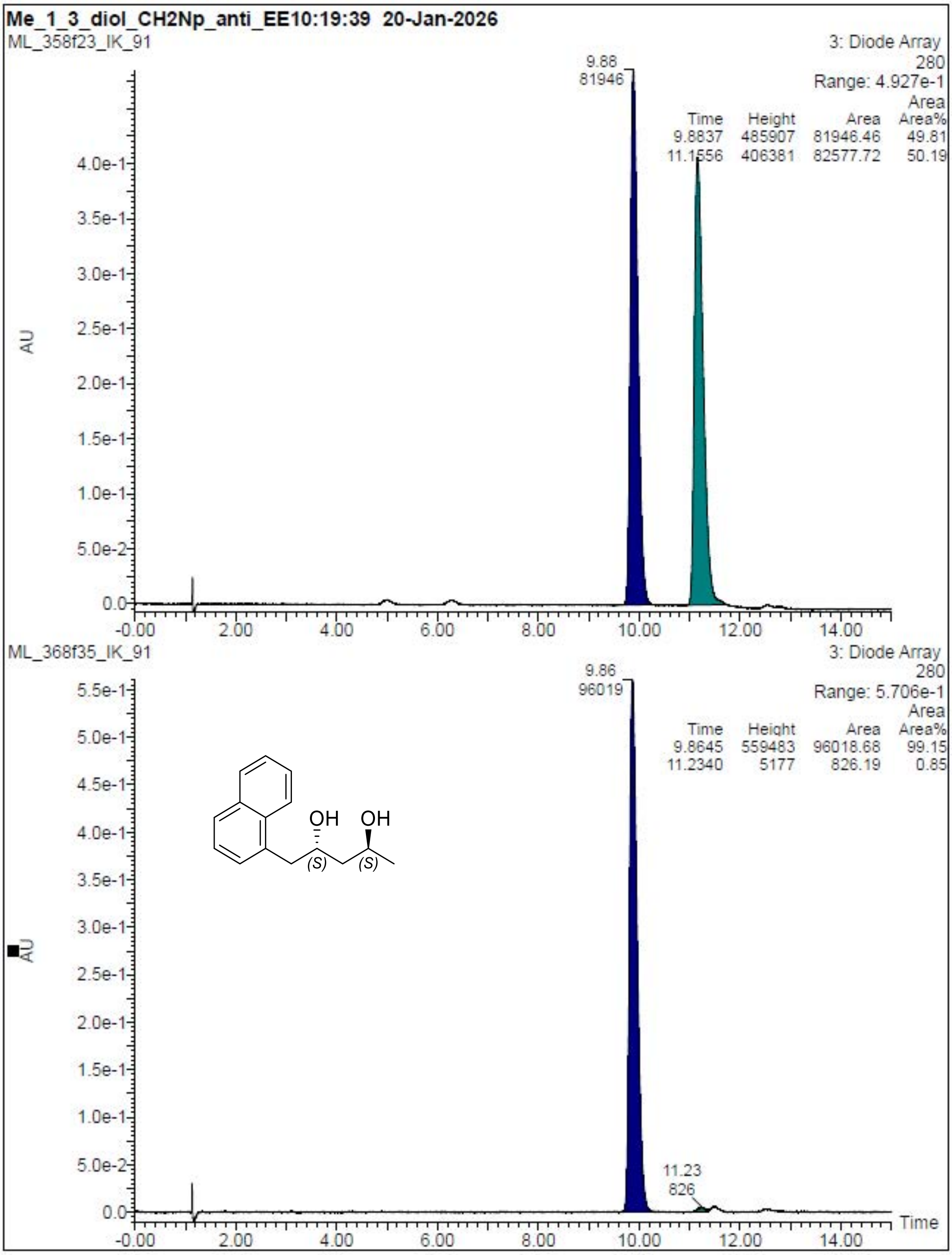

(2*R*,4*S*)-1-(Naphthalen-1-yl)pentane-2,4-diol (2*R*,4*S*-3c)

Chiral SFC Analysis: CHIRALPAK IK (CO_2:MeOH, 91:9, 2.5 mL min^{-1}, 40 °C, 280 nm) t_R = 11.5 (major), 12.8 (minor) min, 79% *ee*.

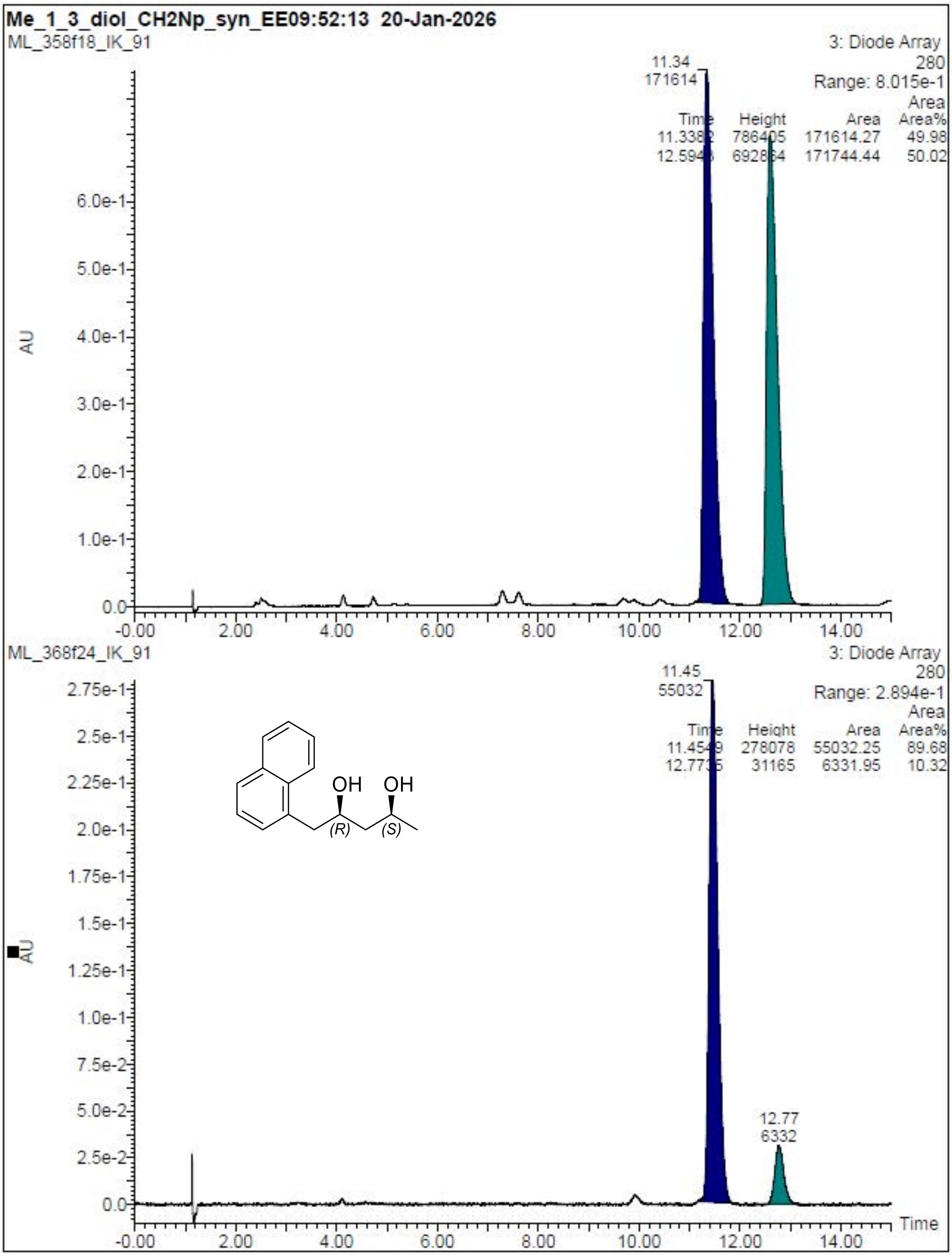

(*S*,*S*)-6-Phenylhexane-2,4-diol (*S*,*S*-3d)

Chiral SFC Analysis: CHIRALPAK IK (CO_2:MeOH, 95:5, 2.5 mL min^{-1}, 40 °C, 205 nm) t_R = 10.7 (major), 11.1 (minor) min, 99% *ee*.

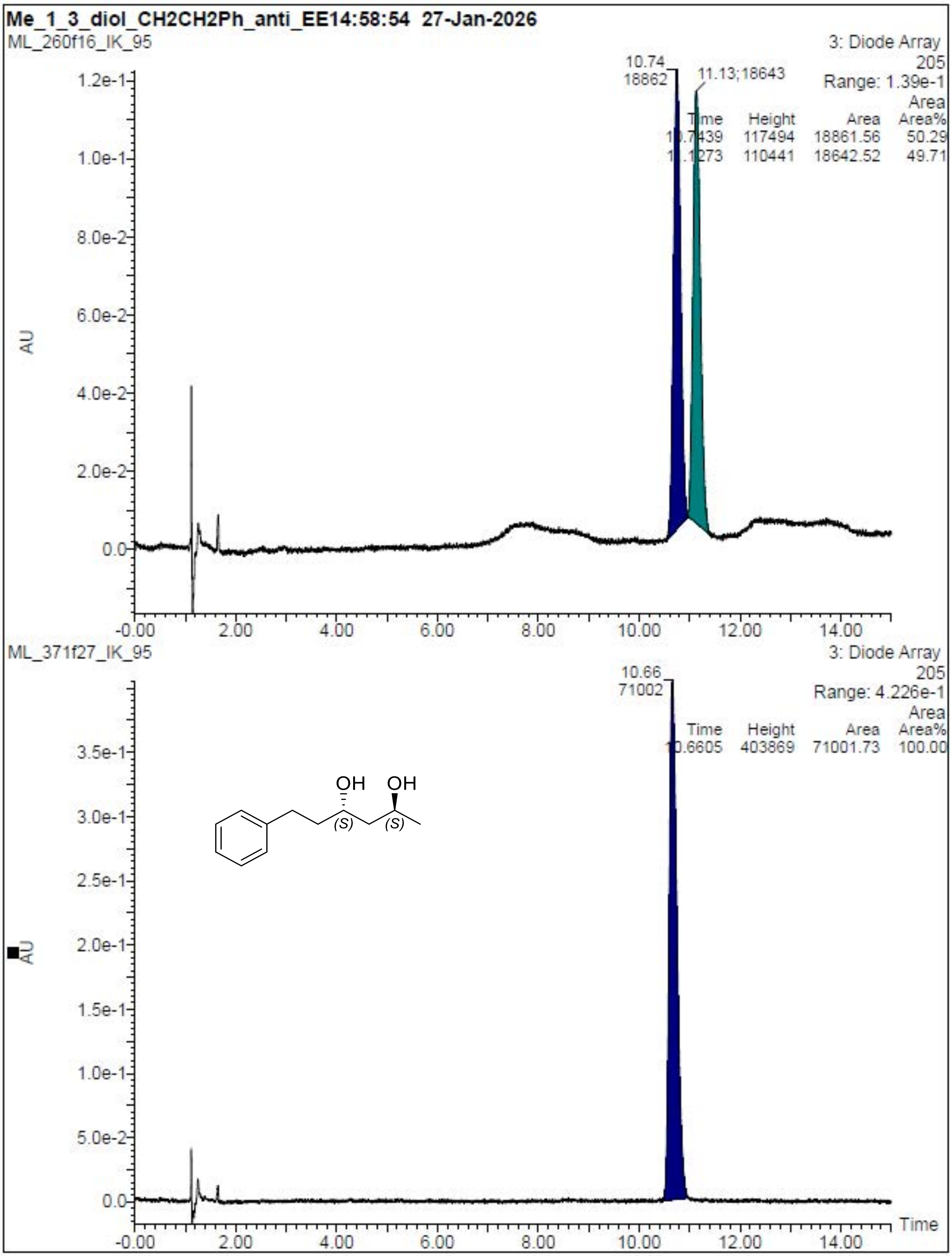

(2*S*,4*R*)-6-Phenylhexane-2,4-diol (2*S*,4*R*-3d)

Chiral SFC Analysis: CHIRALPAK IC (CO_2:MeOH, 95:5, 2.5 mL min^{-1}, 40 °C, 205 nm) t_R = 11.6 (minor), 14.0 (major) min, 32% *ee*.

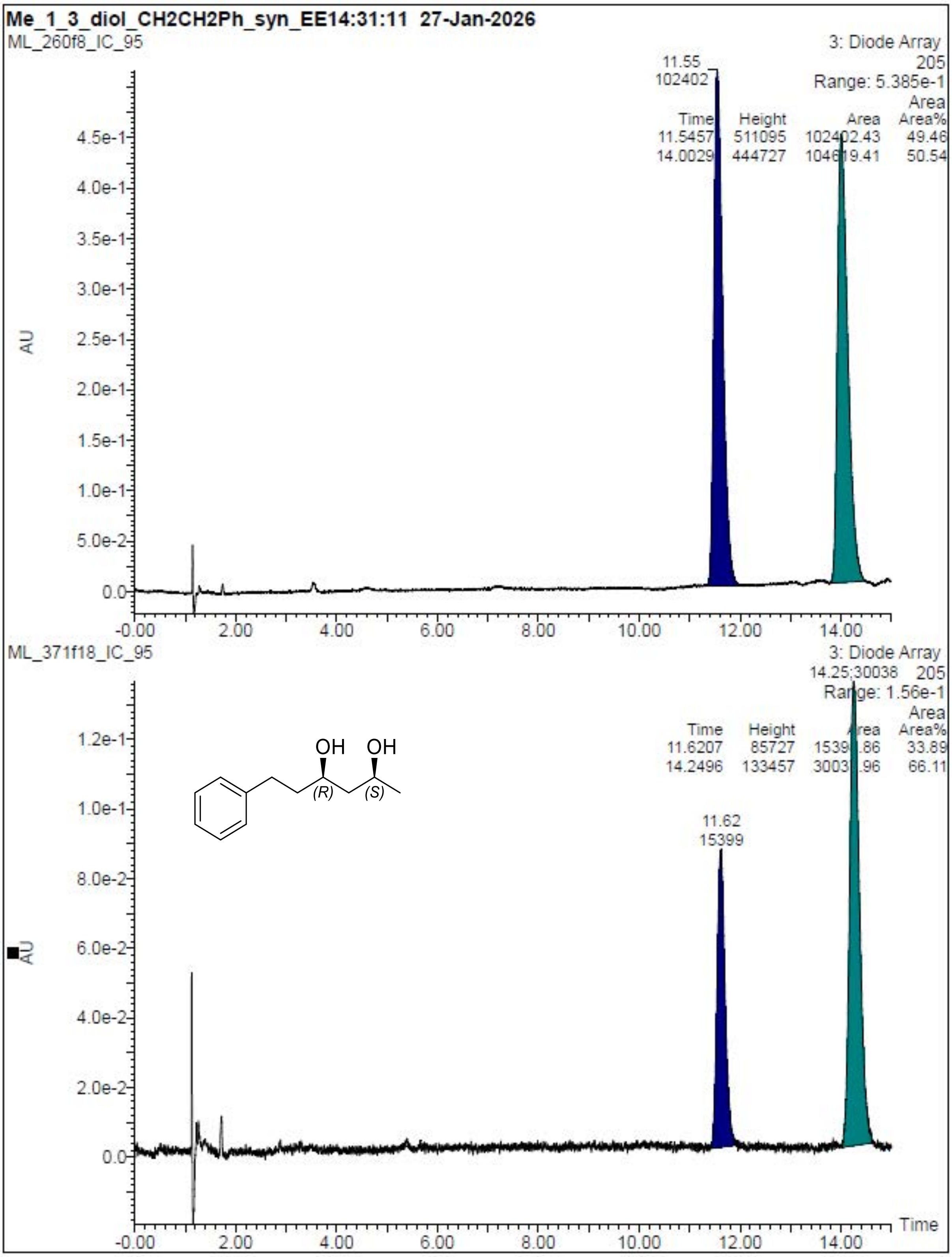

Ethyl 3-((*S*,*S*)-3,5-dihydroxyhexyl)benzoate (*S*,*S*-3e)

Chiral SFC Analysis: CHIRALPAK IK (CO_2:MeOH, 93:7, 2.5 mL min^{-1}, 40 °C, 230 nm) t_R = 11.5 (major), 12.4 (minor) min, 99% *ee*.

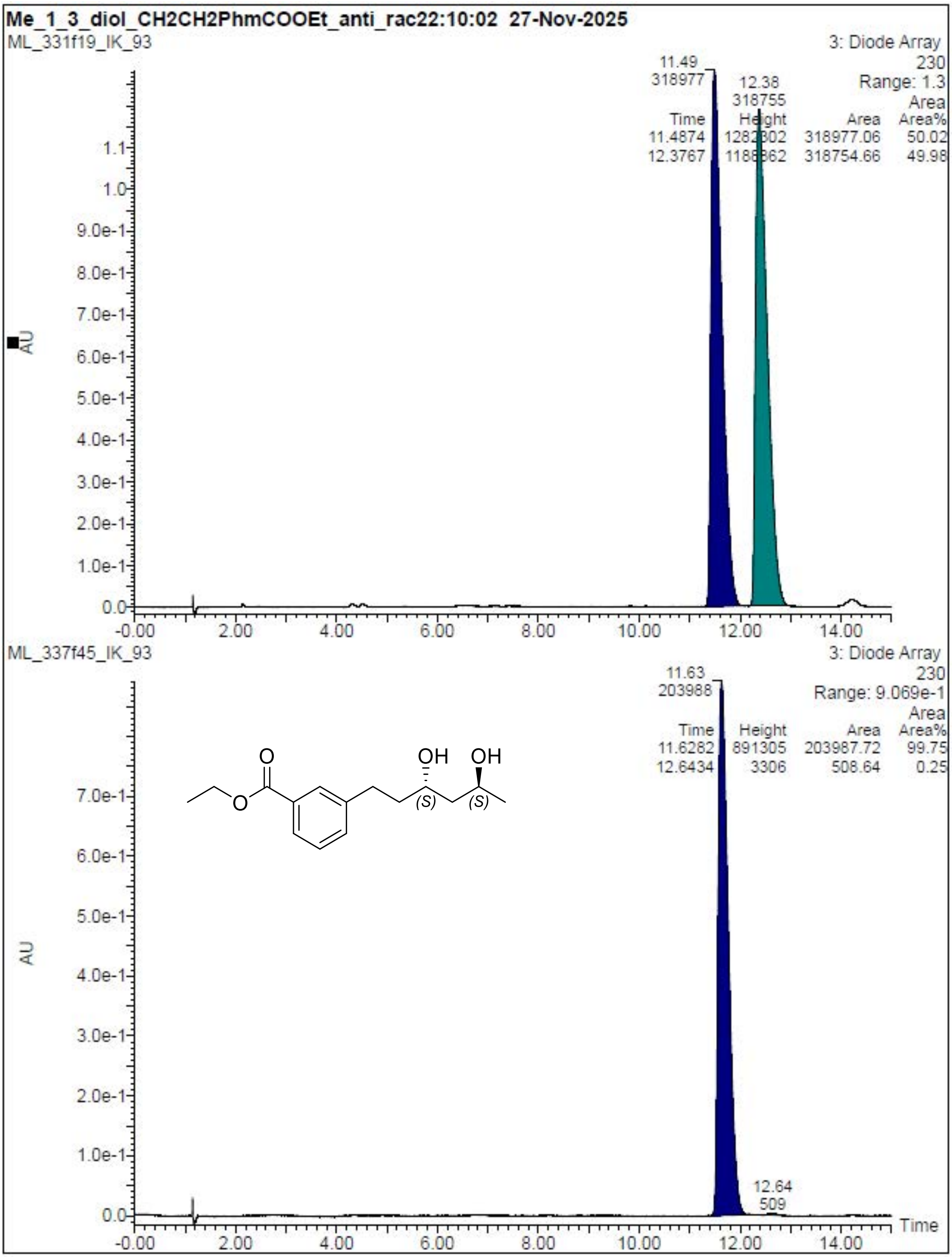

Ethyl 3-((3*R*,5*S*)-3,5-dihydroxyhexyl)benzoate (3*R*,5*S*-3e)

Chiral SFC Analysis: CHIRALPAK IK (CO_2:MeOH, 90:10, 2.5 mL min^{-1}, 40 °C, 230 nm) t_R = 7.56 (minor), 11.1 (major) min, 48% *ee*.

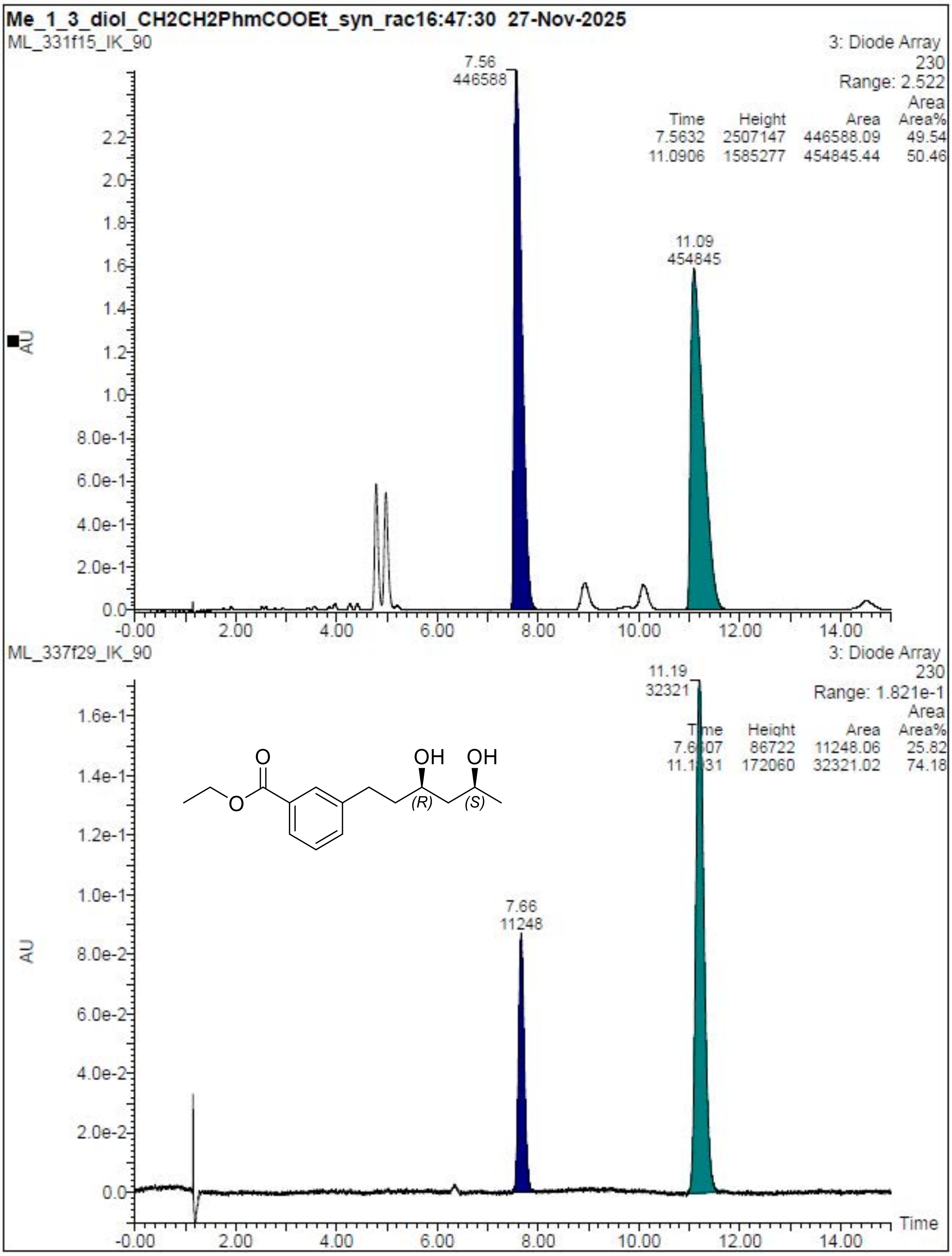

(*S*,*S*)-6-(2-(Trifluoromethyl)phenyl)hexane-2,4-diol (*S*,*S*-3f)

Chiral SFC Analysis: CHIRALPAK IK (CO_2:MeOH, 97:3, 2.5 mL min^{-1}, 40 °C, 207 nm) t_R = 8.5 (major), 9.0 (minor) min, 99% *ee*.

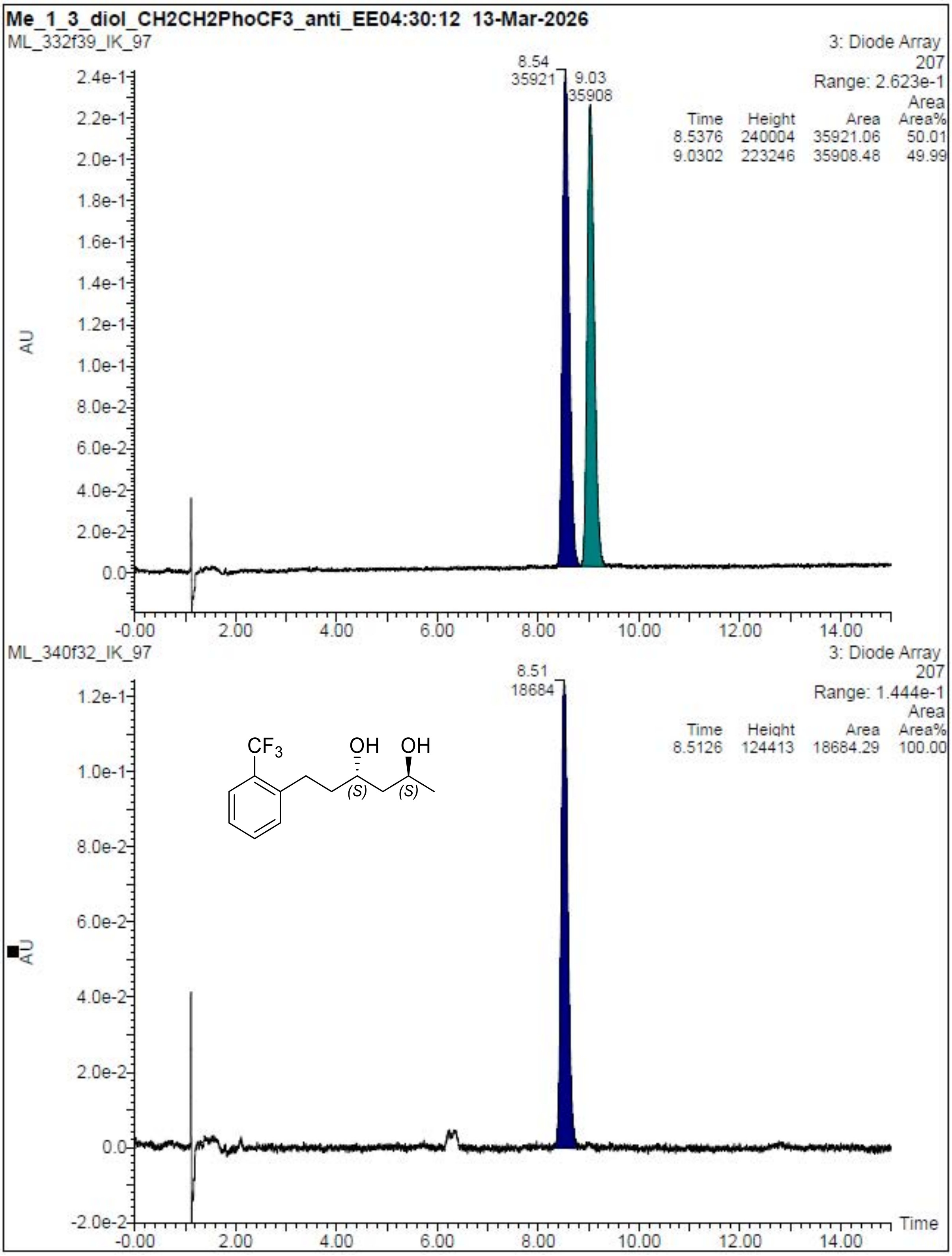

(2*S*,4*R*)-6-(2-(Trifluoromethyl)phenyl)hexane-2,4-diol (2*S*,4*R*-3f)

Chiral SFC Analysis: CHIRALPAK IK (CO_2:MeOH, 93:7, 2.5 mL min^{-1}, 40 °C, 207 nm) t_R = 3.8 (minor), 4.2 (major) min, 60% *ee*.

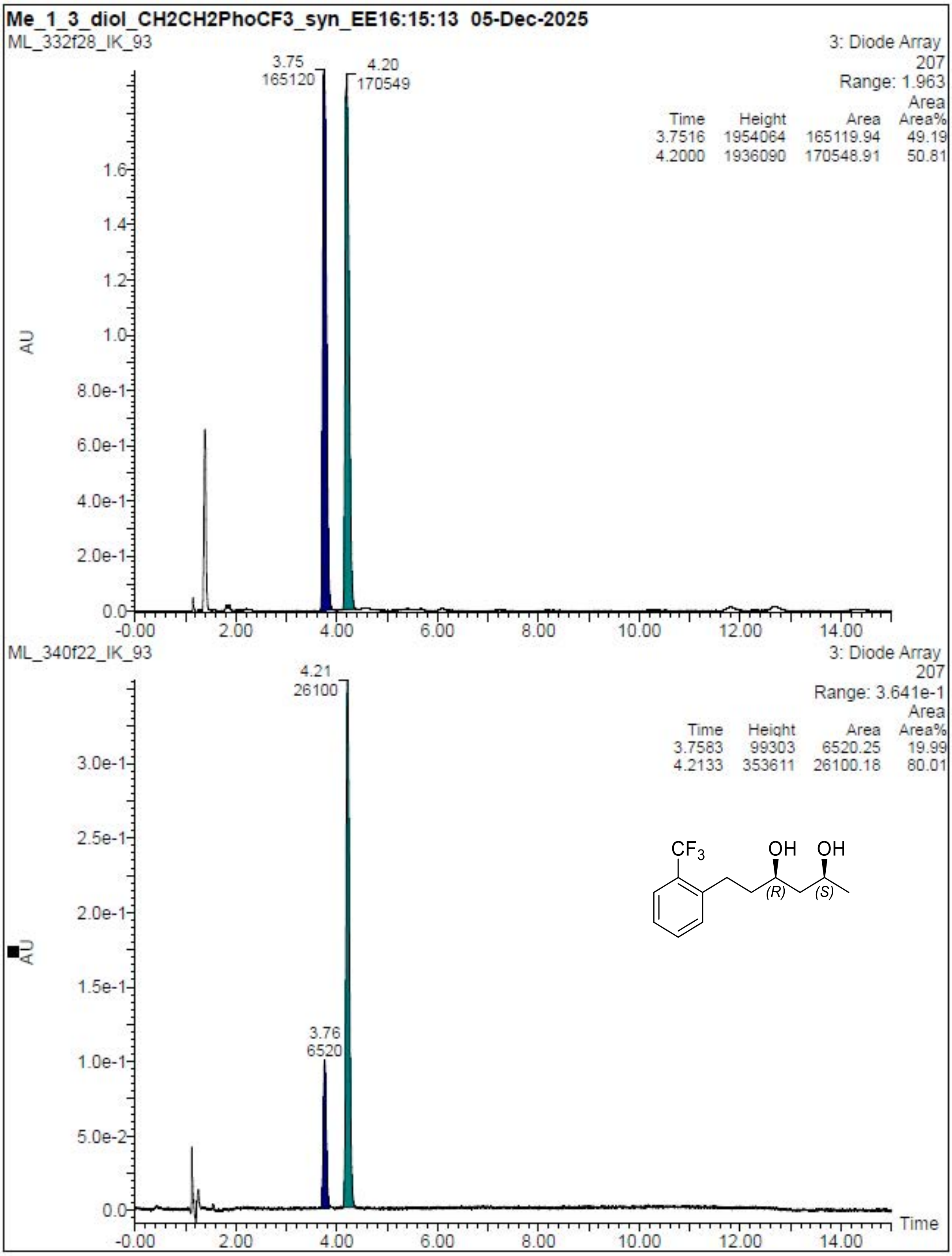

4-((*S*,*S*)-3,5-Dihydroxyhexyl)benzonitrile (*S*,*S*-3g)

Chiral SFC Analysis: CHIRALPAK IA (CO_2:MeOH, 92:8, 2.5 mL min^{-1}, 40 °C, 230 nm) t_R = 10.3 (major), 11.5 (minor) min, 99% *ee*.

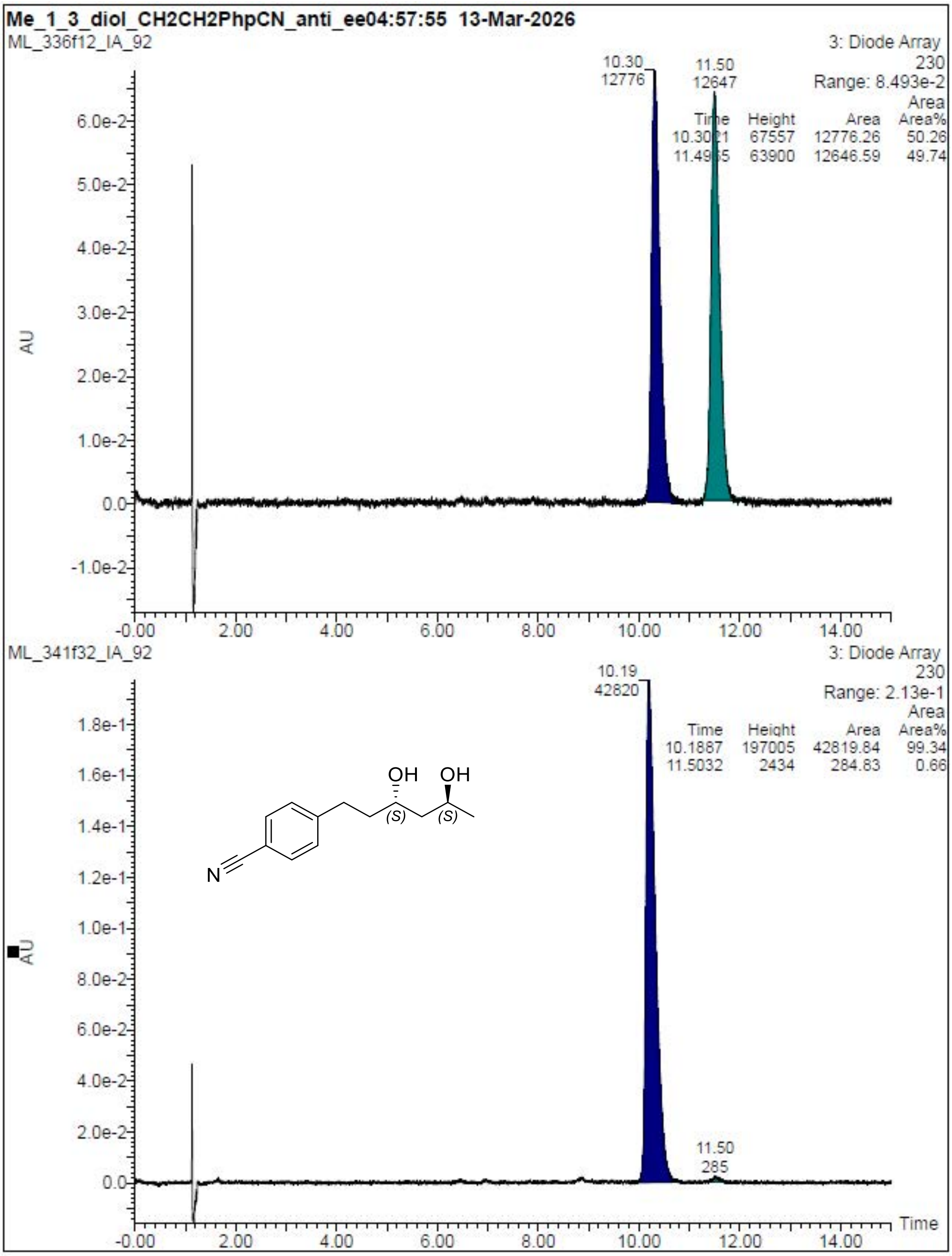

4-((3*R*,5*S*)-3,5-Dihydroxyhexyl)benzonitrile (3*R*,5*S*-3g)

Chiral SFC Analysis: CHIRALPAK IK (CO_2:MeOH, 90:10, 2.5 mL min^{-1}, 40 °C, 230 nm) t_R = 10.1 (minor), 11.5 (major) min, 71% *ee*.

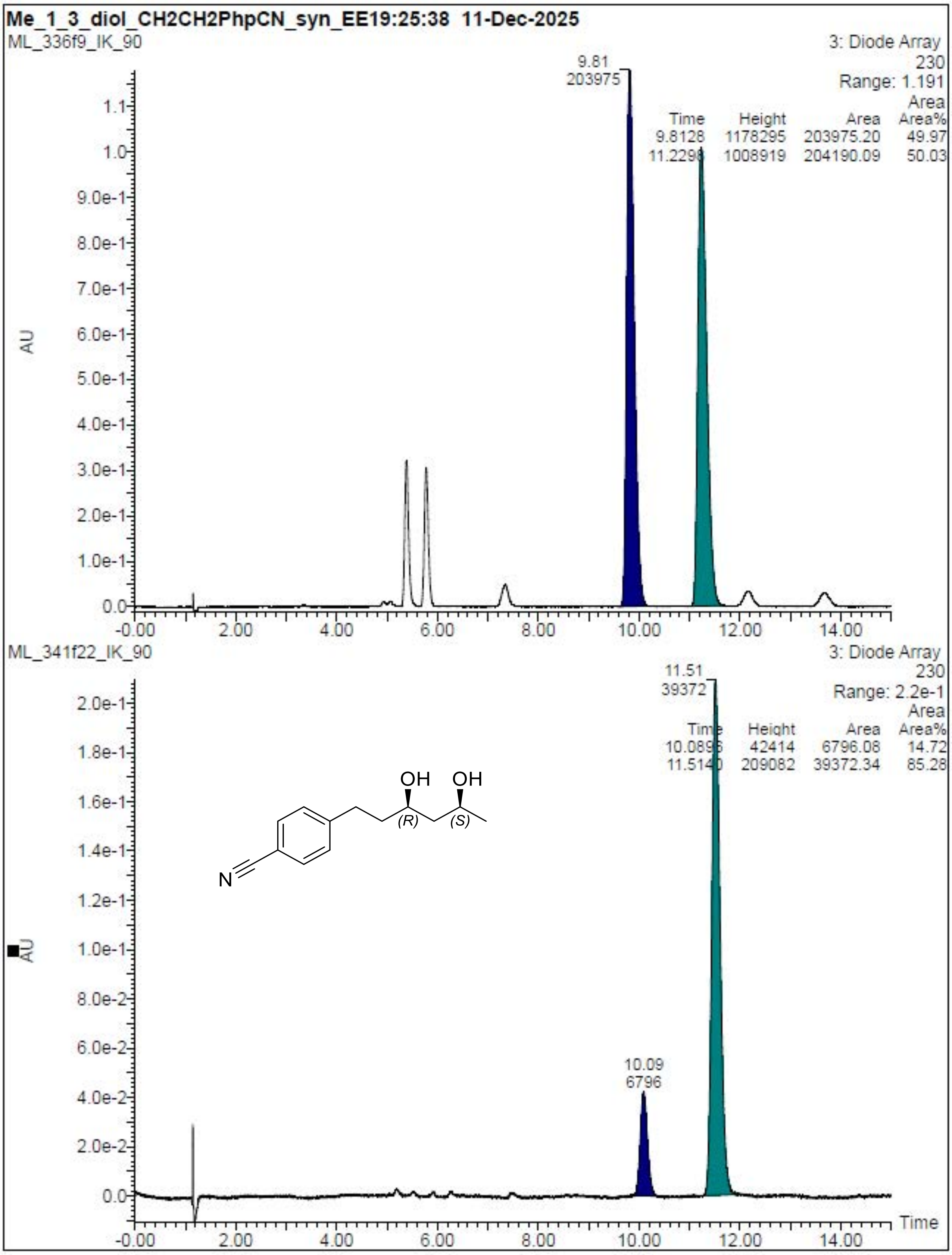

(*R*,*S*)-*N*-(4-(3,5-dihydroxyhexyl)phenyl)-2,2,2-trifluoroacetamide (*syn*-3h)

Chiral HPLC Analysis CHIRALPAK SC (Hexane:iPrOH, 90:10, 1.25 ml min^{-1}, 40 °C) t_R = 16.0 (minor), 19.1 (major) minutes, 52% *ee*.

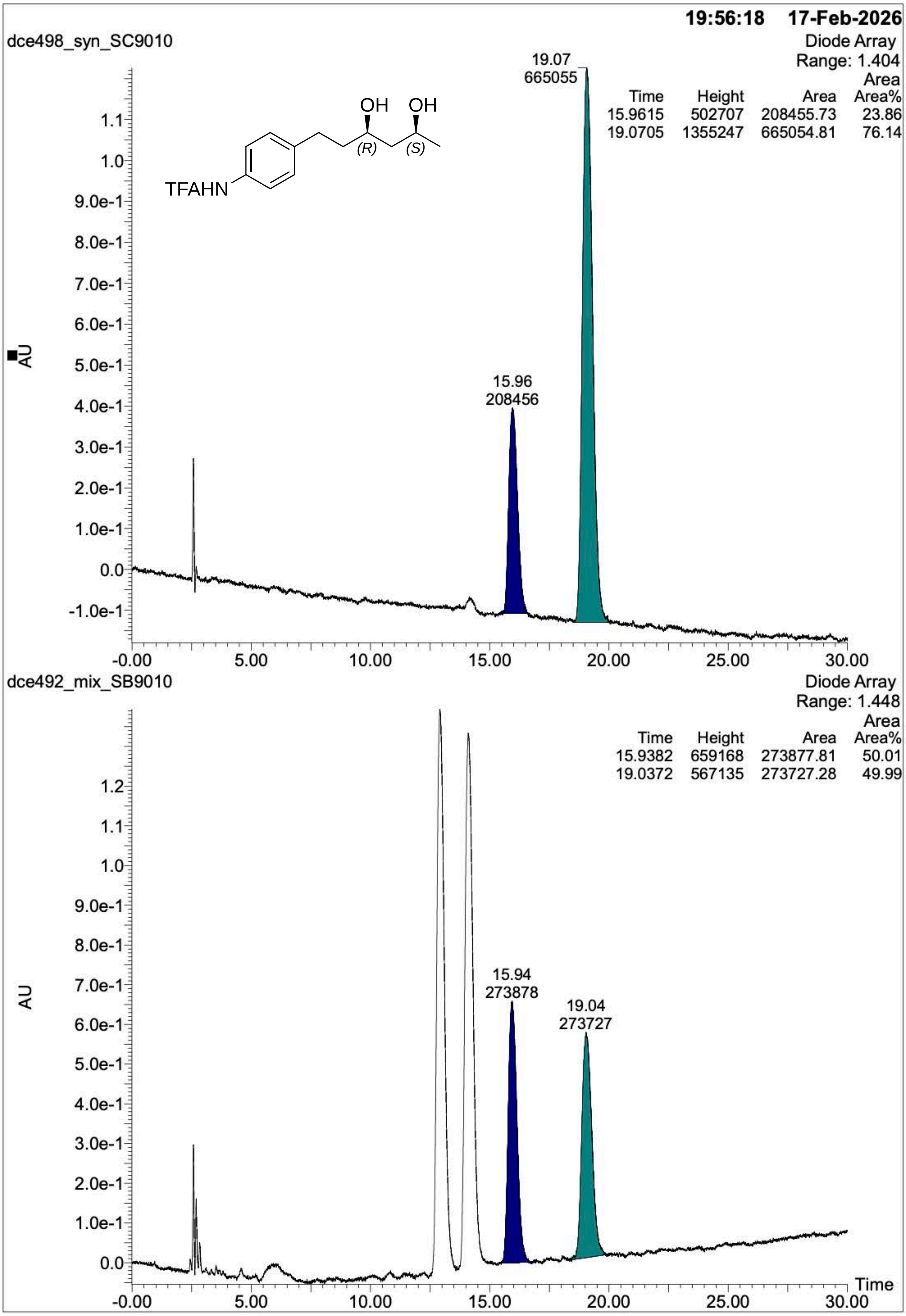

(*S*,*S*)-*N*-(4-(3,5-dihydroxyhexyl)phenyl)-2,2,2-trifluoroacetamide (*anti*-3h)

Chiral HPLC Analysis CHIRALPAK SC (Hexane:iPrOH, 90:10, 1.25 ml min^{-1}, 40 °C) t_R = 12.9 (minor), 14.1 (major) minutes, 97% *ee*.

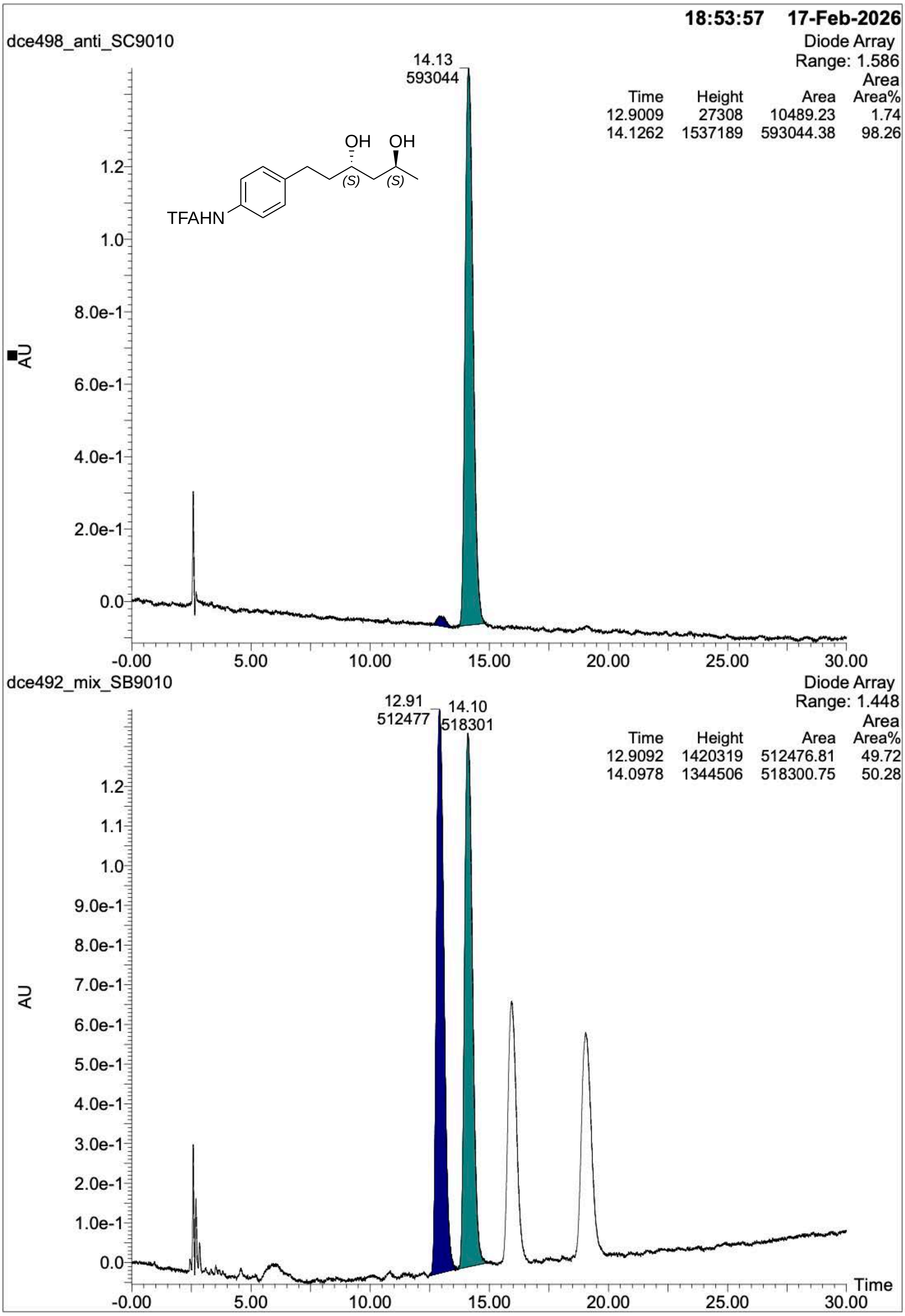

(3*S*,5*R*)-6-Methyl-1-phenylheptane-3,5-diol (*S*,*R*-3i)

Chiral SFC Analysis: CHIRALPAK IC (CO_2:MeOH, 95:5, 2.5 mL min^{-1}, 40 °C, 205 nm) t_R = 8.7 (minor), 9.2 (major) min, 99% *ee*.

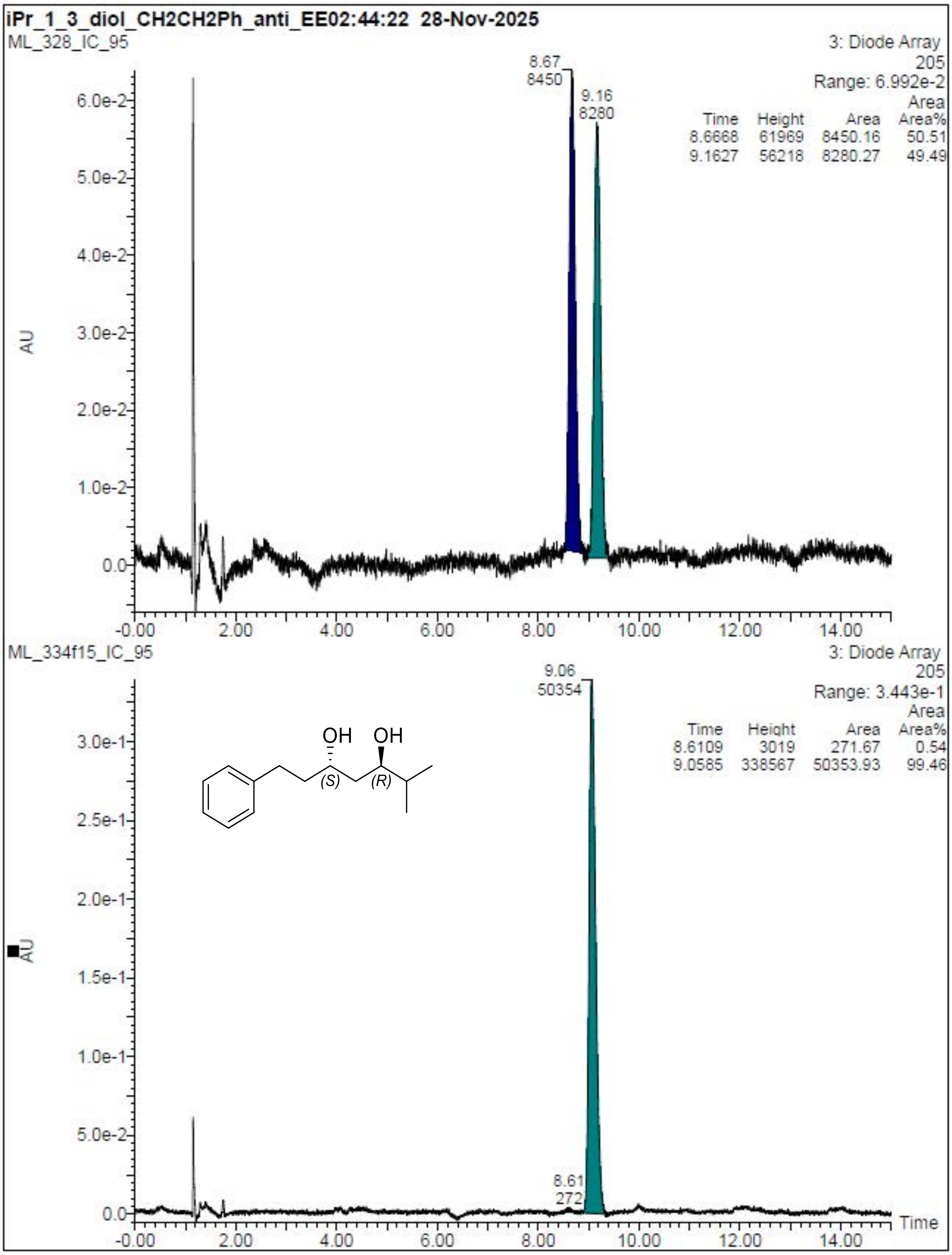

Enriched (*syn*)-6-Methyl-1-phenylheptane-3,5-diol (*enriched-syn*-3i)

Chiral SFC Analysis: CHIRALPAK IK (CO_2:MeOH, 93:7, 2.5 mL min^{-1}, 40 °C, 205 nm) t_R = 6.9 (major), 8.6 (minor) min, 38% *ee*.

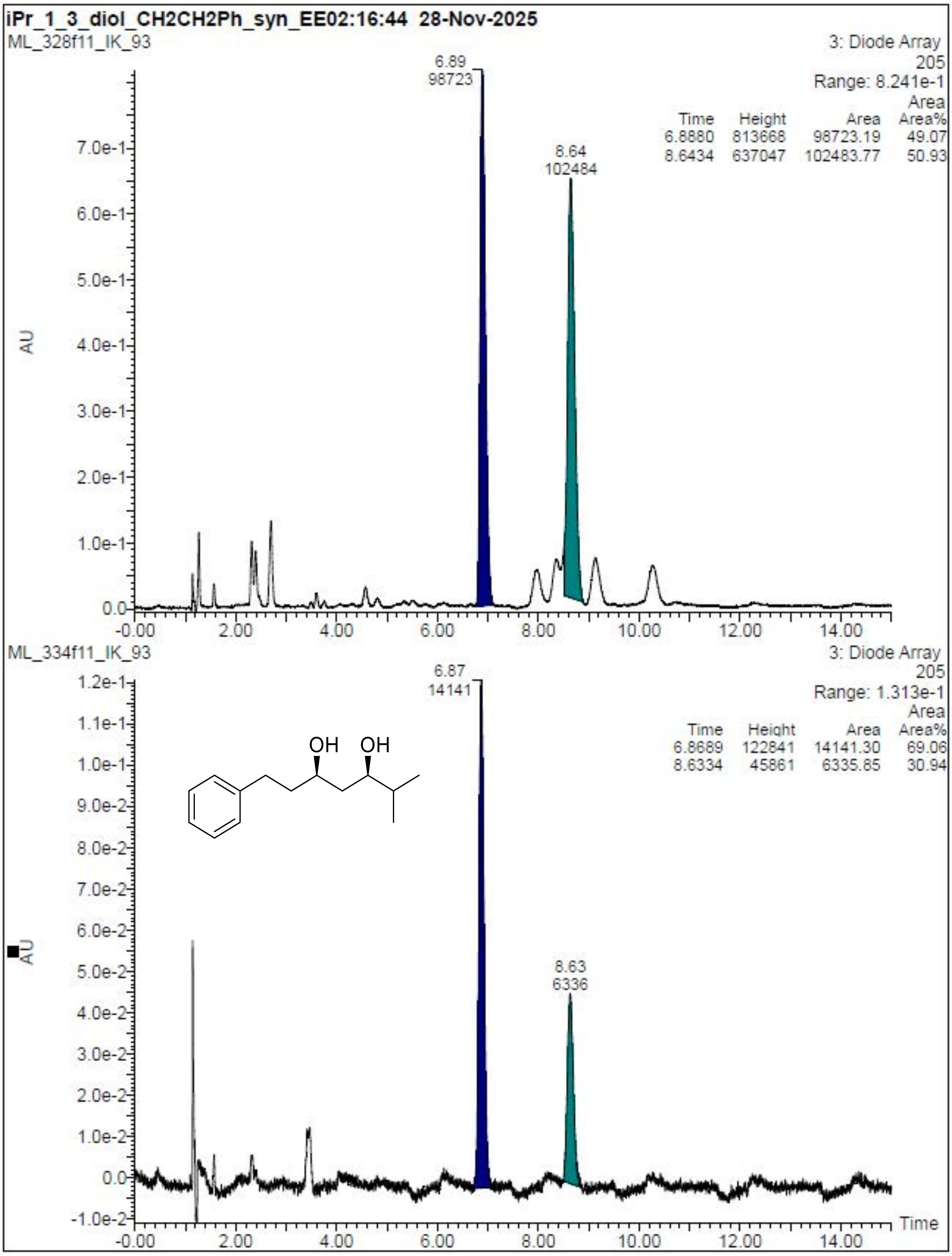

tert-butyl 4-((2_S_,4_R_)-2,4-dihydroxy-6-phenylhexyl)piperidine-1-carboxylate (_syn_-3j)

Chiral SFC Analysis CHIRALPAK IC (CO_2:MeOH, 80:20, 2.5 ml min^{-1}, 40 °C, 209 nm) t_R = 5.3 (minor), 7.3 (major) minutes, 76% _ee_.

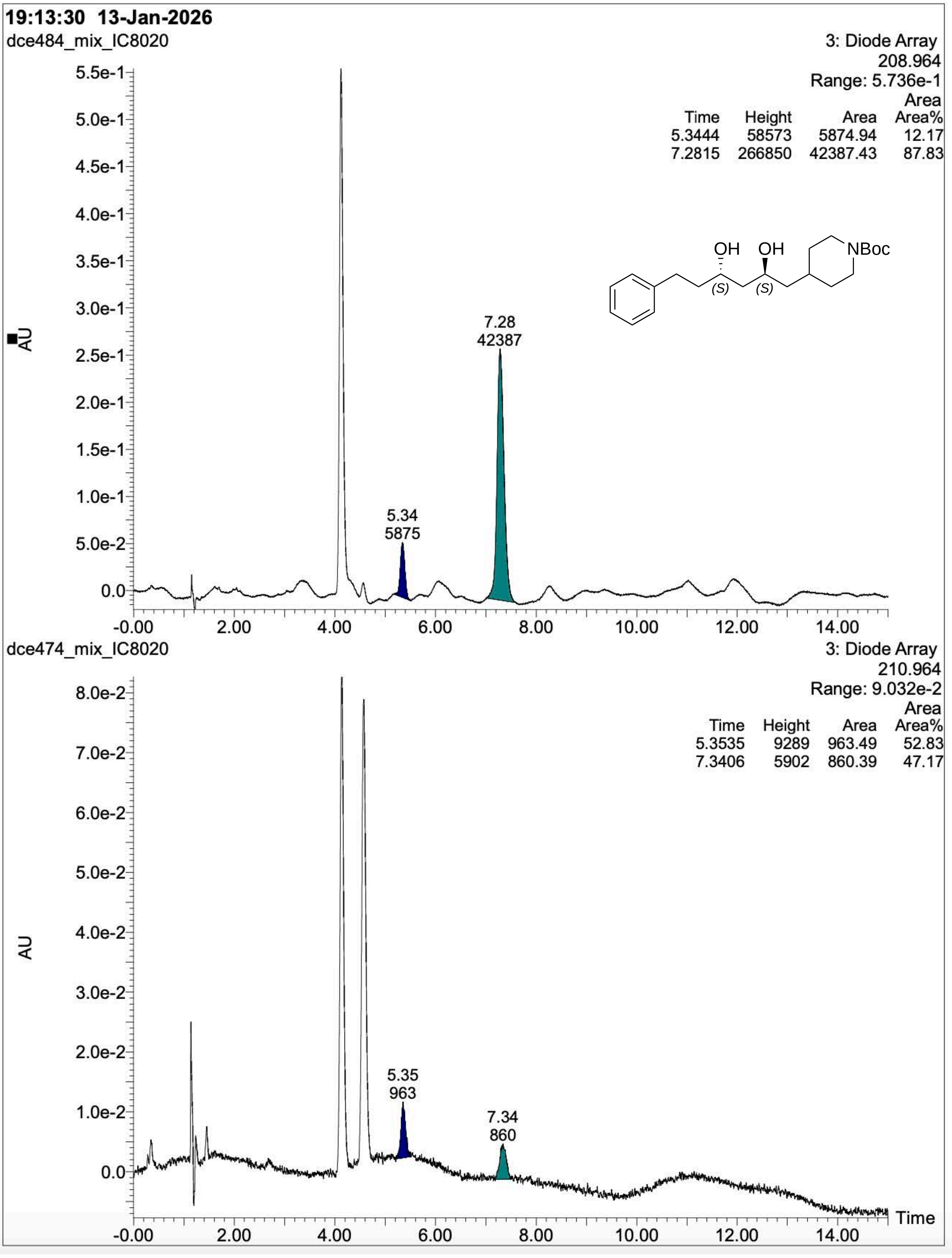

tert-butyl 4-((2_S_,4_S_)-2,4-dihydroxy-6-phenylhexyl)piperidine-1-carboxylate (_anti_-3j)

Chiral SFC Analysis CHIRALPAK IC (CO_2:MeOH, 80:20, 2.5 ml min^{-1}, 40 °C, 211 nm) t_R = 4.1 (major), 4.6 (minor) minutes, 94% *ee*.

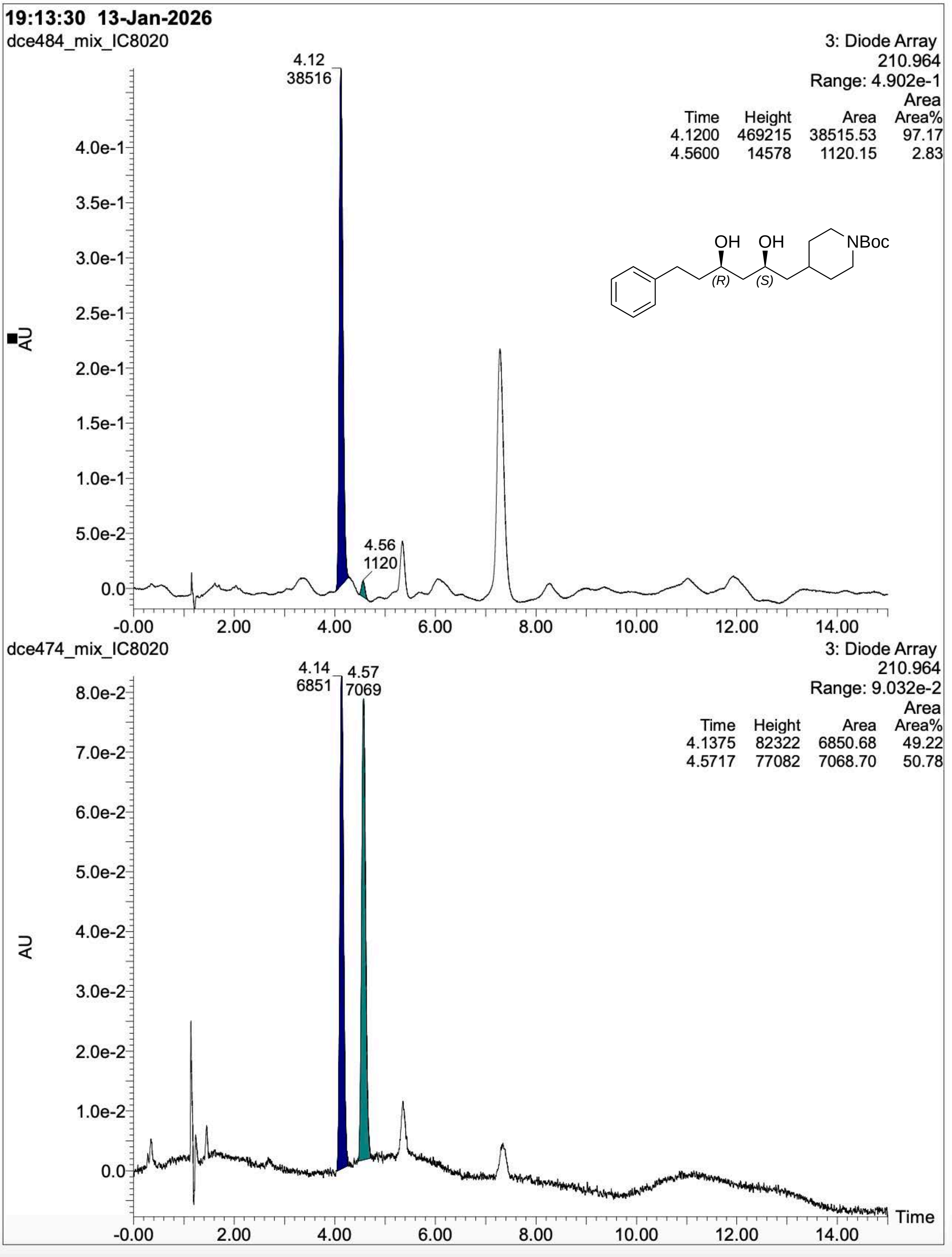

(*S*,*S*)-1-(4-Methoxyphenyl)pentane-2,4-diol (*S*,*S*-3k)

Chiral SFC Analysis: CHIRALPAK IC (CO_2:MeOH, 95:5, 2.5 mL min^{-1}, 40 °C, 205 nm) t_R = 10.7 (major), 11.3 (minor) min, 99% *ee*.

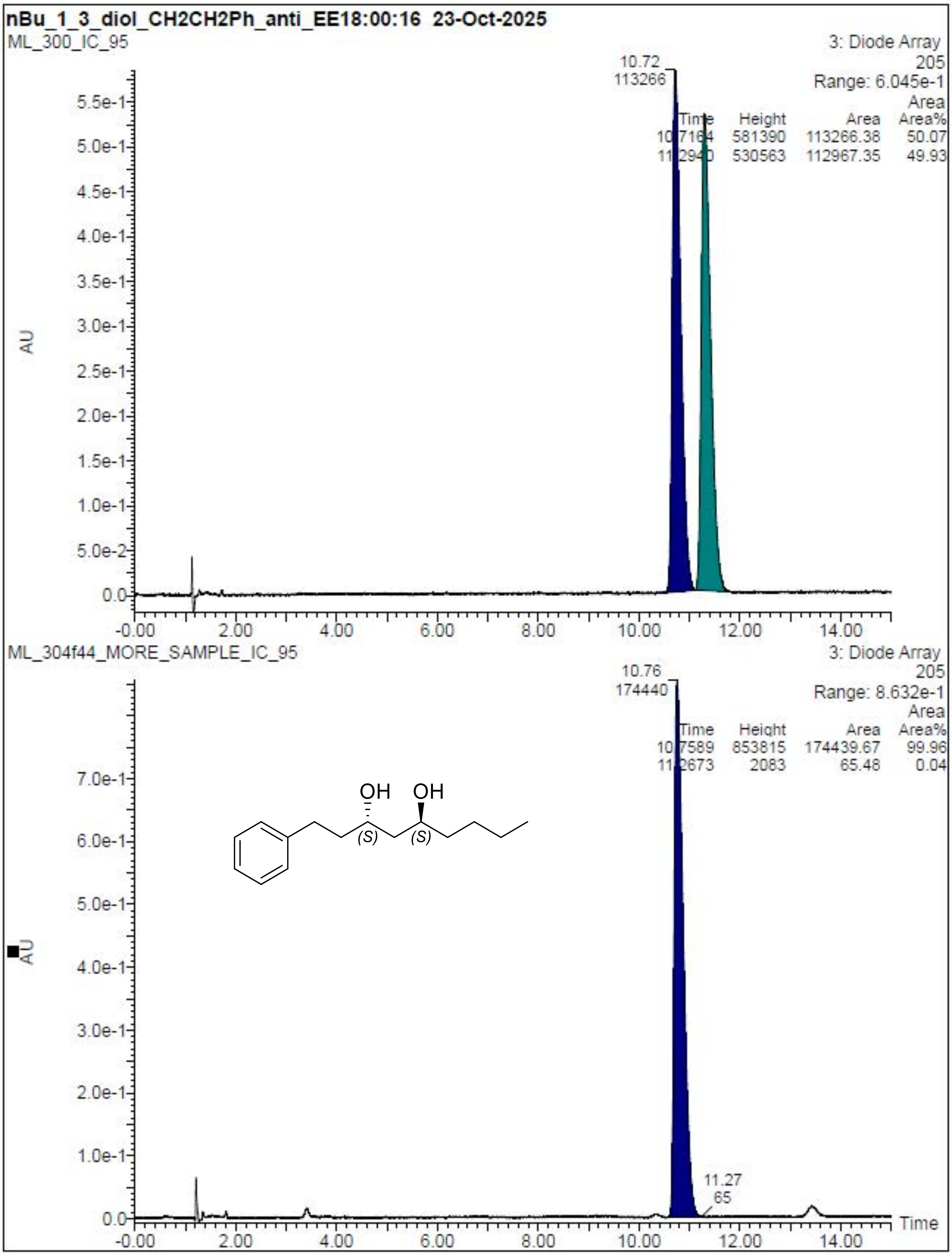

Enriched (*syn*)-1-Phenylnonane-3,5-diol (*enriched-syn*-3k)

Chiral SFC Analysis: CHIRALPAK IK (CO_2:MeOH, 93:7, 2.5 mL min^{-1}, 40 °C, 223 nm) t_R = 8.7 (minor), 10.5 (major) min, 9% *ee*.

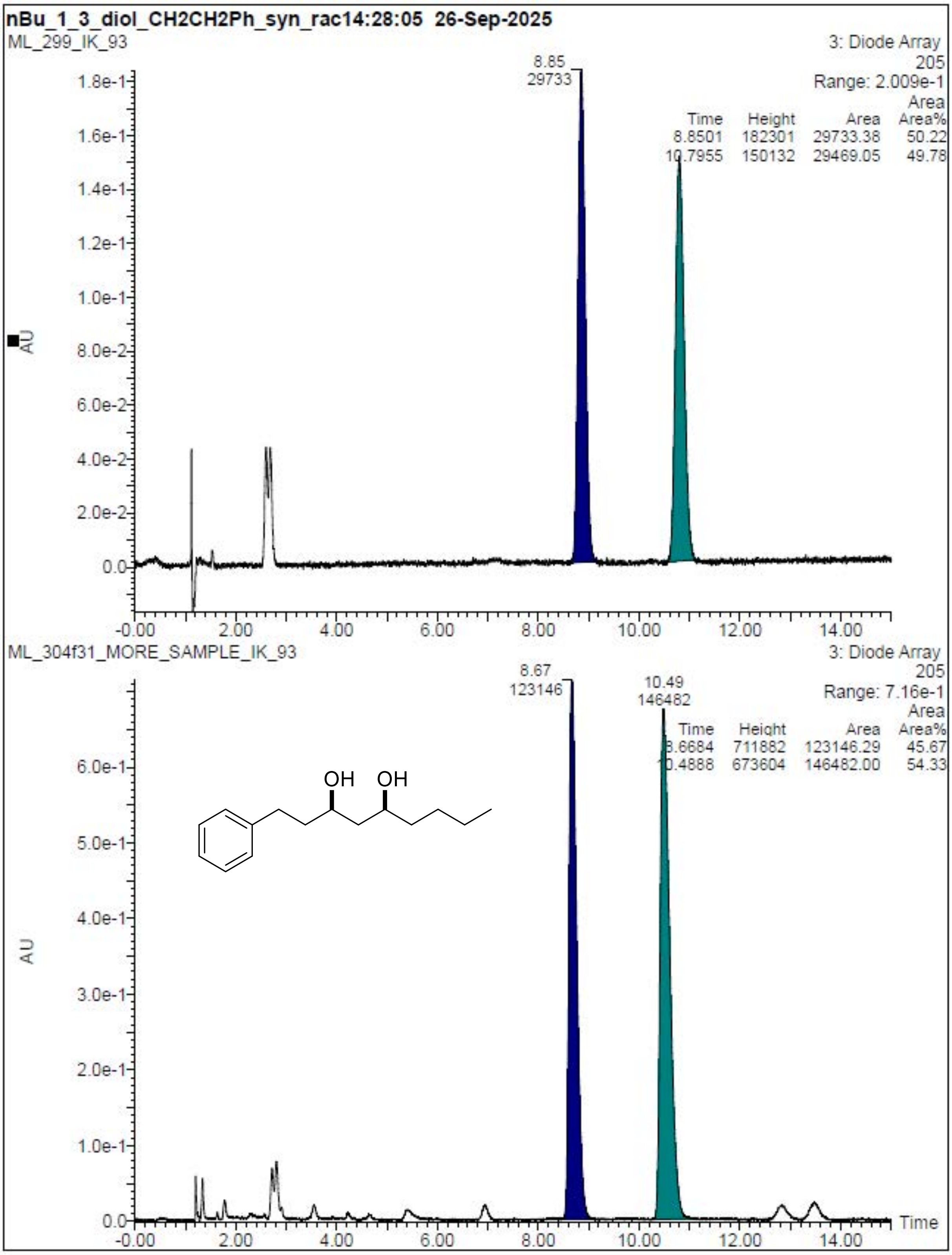

(3*R*,5*S*)-1-phenyl-7-((2,3,3-trimethylbutan-2-yl)oxy)heptane-3,5-diol (*syn*-3l)

Chiral SFC Analysis CHIRALPAK IC (CO_2:MeOH, 97:03, 2.5 ml min^{-1}, 40 °C, 210 nm) t_R = 12.8 (minor), 13.9 (major) minutes, 4% *ee*.

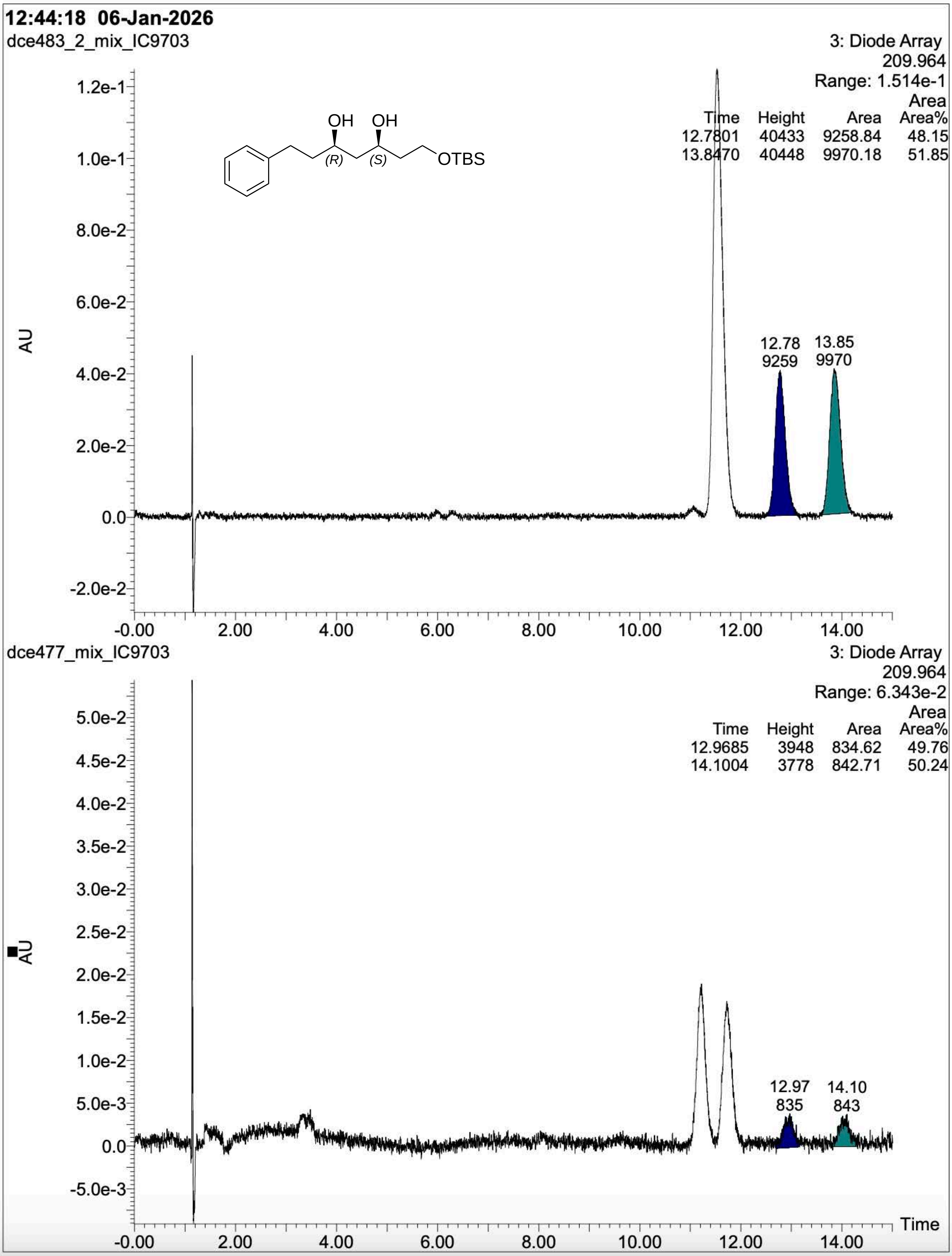

(3*S*,5*S*)-1-phenyl-7-((2,3,3-trimethylbutan-2-yl)oxy)heptane-3,5-diol (*anti*-3l)

Chiral SFC Analysis CHIRALPAK IC (CO_2:MeOH, 97:03, 2.5 ml min^{-1}, 40 °C, 210 nm) t_R = 11.1 (minor), 11.5 (major) minutes, 96% *ee*.

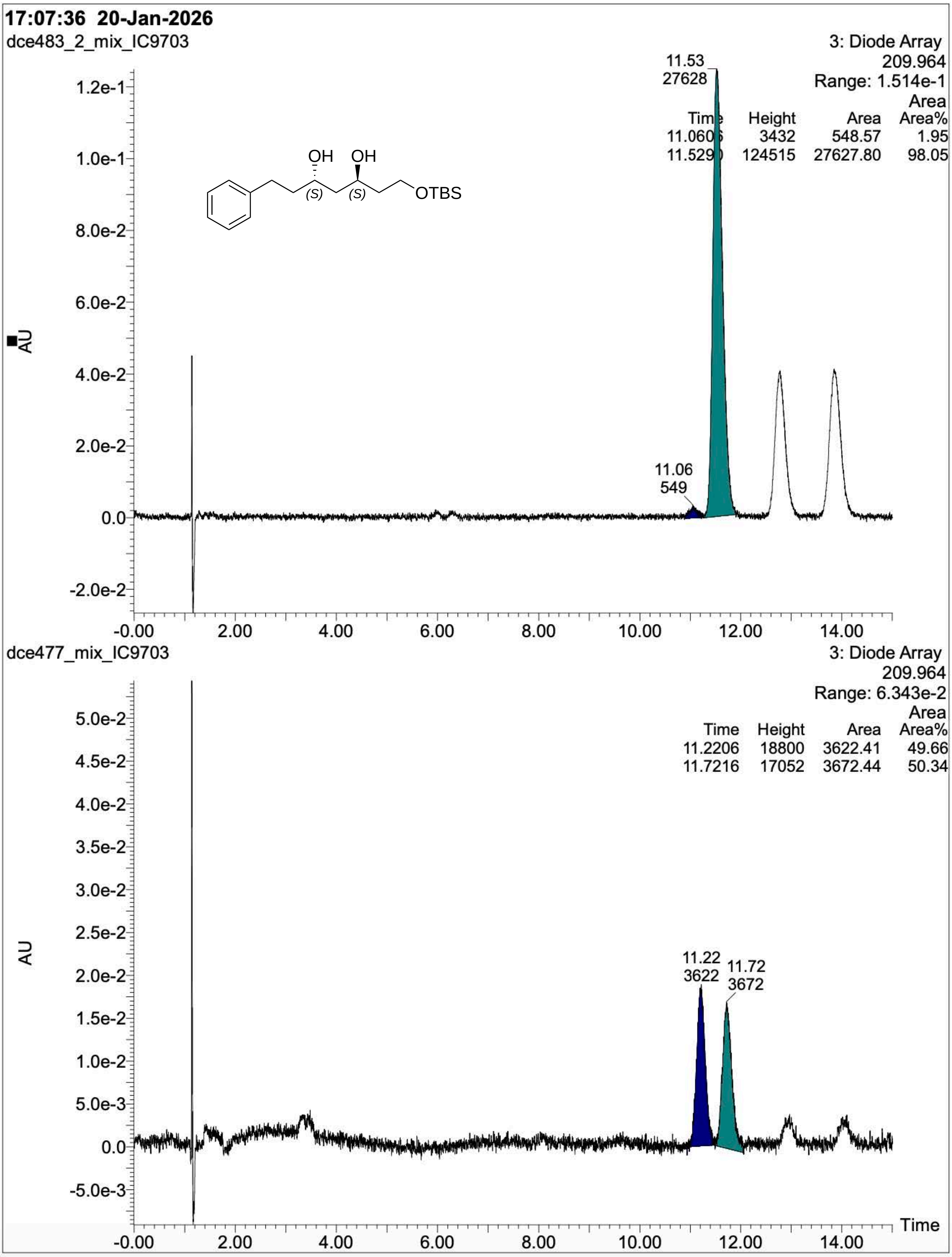

(2*R*,4*S*)-1-tosylpentane-2,4-diol (*syn*-3m)

Chiral SFC Analysis CHIRALPAK IA (CO_2:MeOH, 90:10, 2.5 ml min^{-1}, 40 °C, 228 nm) t_R = 7.2 (major), 7.5 (minor) minutes, 94% *ee*.

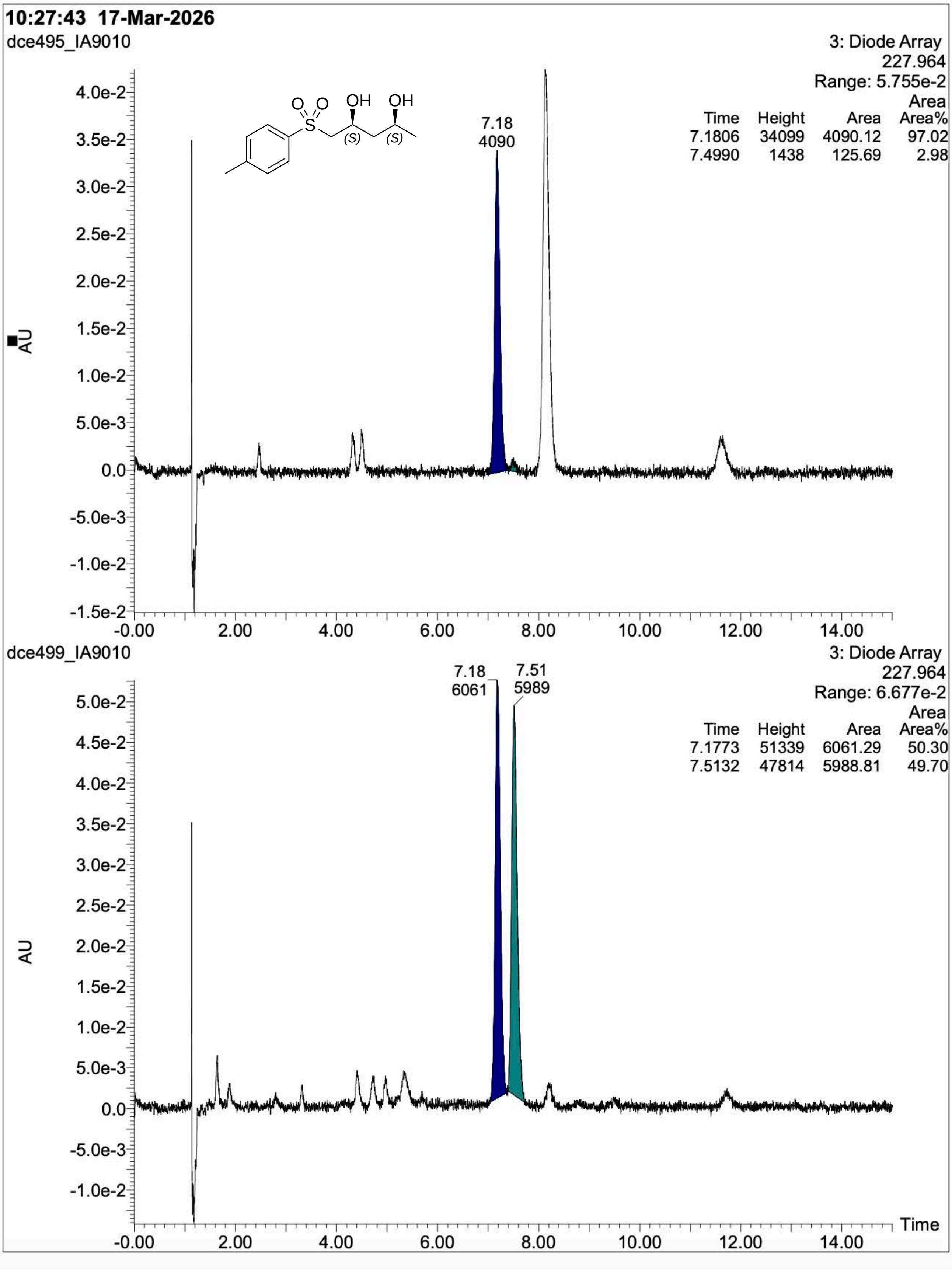

(2*S*,4*S*)-1-tosylpentane-2,4-diol (*anti*-3m)

Chiral SFC Analysis CHIRALPAK IA (CO_2:MeOH, 90:10, 2.5 ml min^{-1}, 40 °C, 228 nm) t_R = 8.1 (major), 11.7 (minor) minutes, 75% *ee*.

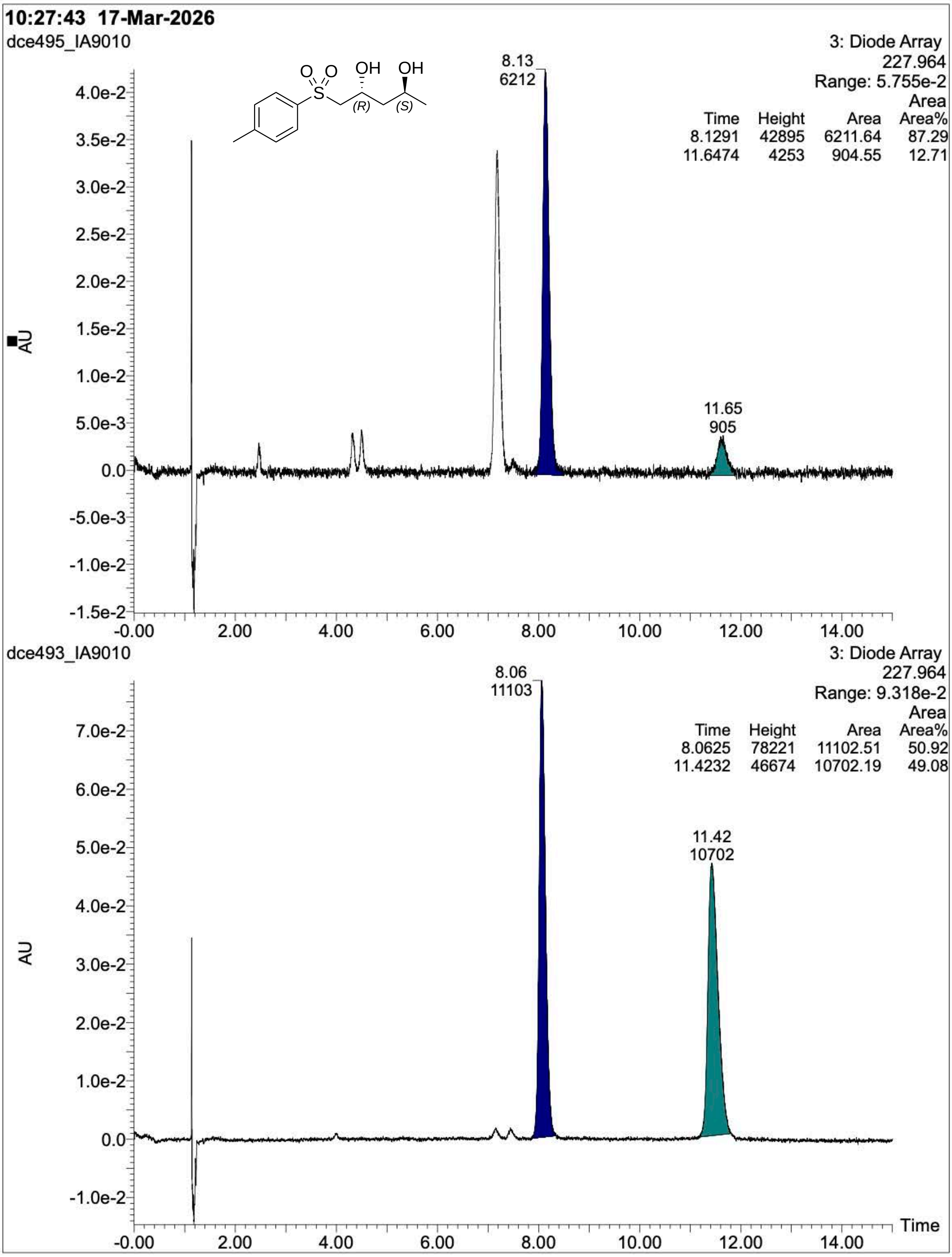

(*S*,*S*)-1-Phenylpentane-2,4-diol (*S*,*S*-3a) from d4

Chiral SFC Analysis: CHIRALPAK IC (CO_2:MeOH, 96:4, 2.5 mL min^{-1}, 40 °C, 205 nm) t_R = 12.0 (major), 12.4 (minor) min, 99% *ee*.

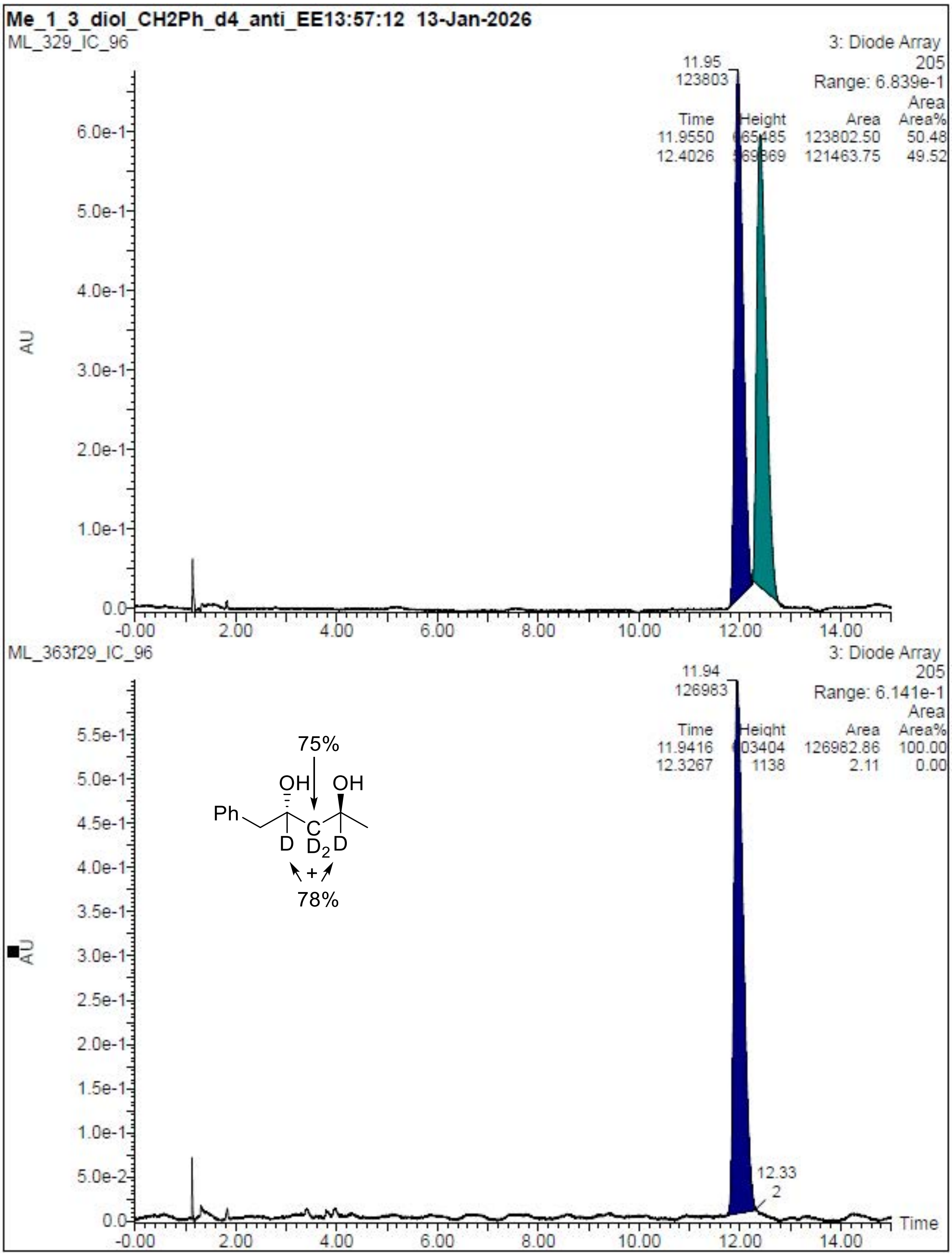

(2*R*,4*S*)-1-Phenylpentane-2,4-diol (2*R*,4*S*-3a) from d4

Chiral SFC Analysis: CHIRALPAK IK (CO_2:MeOH, 93:7, 2.5 mL min^{-1}, 40 °C, 205 nm) t_R = 6.6 (minor), 8.0 (major) min, 84% *ee*.

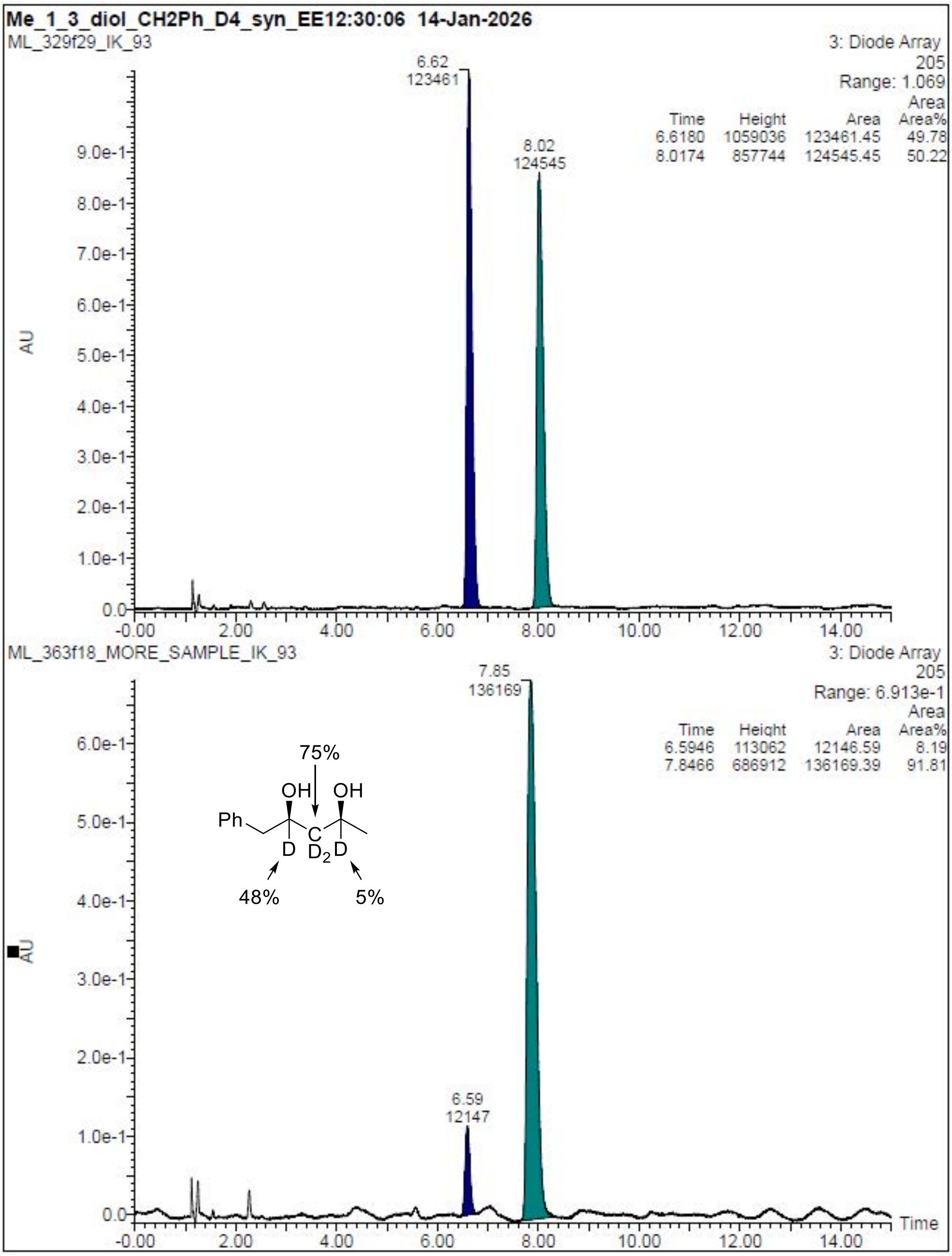

(*S*,*S*)-1-Phenylpentane-2,4-diol-d1 (*S*,*S*-3a-d1) from d1

Chiral SFC Analysis: CHIRALPAK IC (CO_2:MeOH, 96:4, 2.5 mL min^{-1}, 40 °C, 205 nm) t_R = 12.0 (major), 12.4 (minor) min, 99% *ee*.

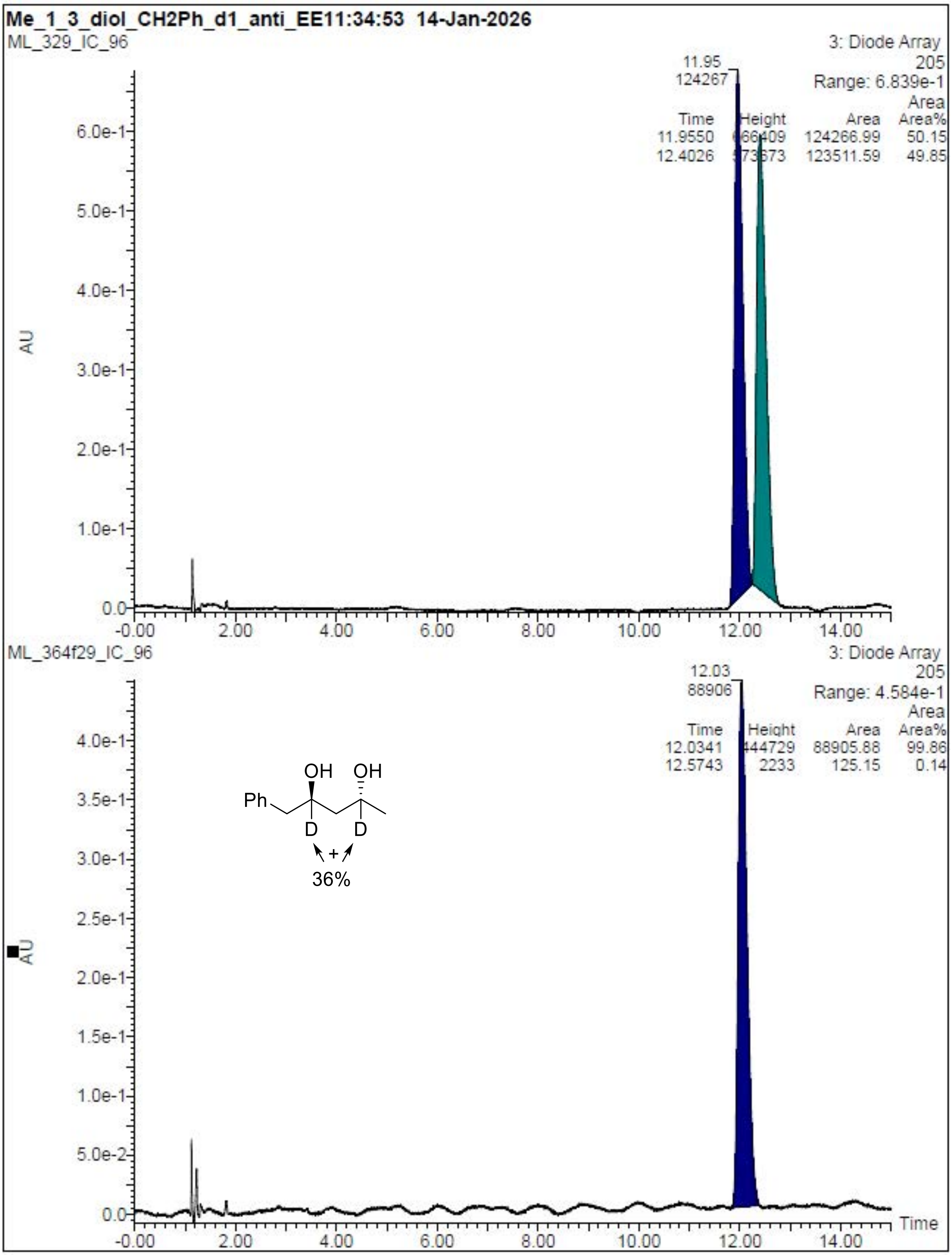

(2*R*,4*S*)-1-Phenylpentane-2,4-diol-d1 (2*R*,4*S*-3a-d1) from d4

Chiral SFC Analysis: CHIRALPAK IK (CO_2:MeOH, 93:7, 2.5 mL min^{-1}, 40 °C, 205 nm) t_R = 6.6 (minor), 8.0 (major) min, 65% *ee*.

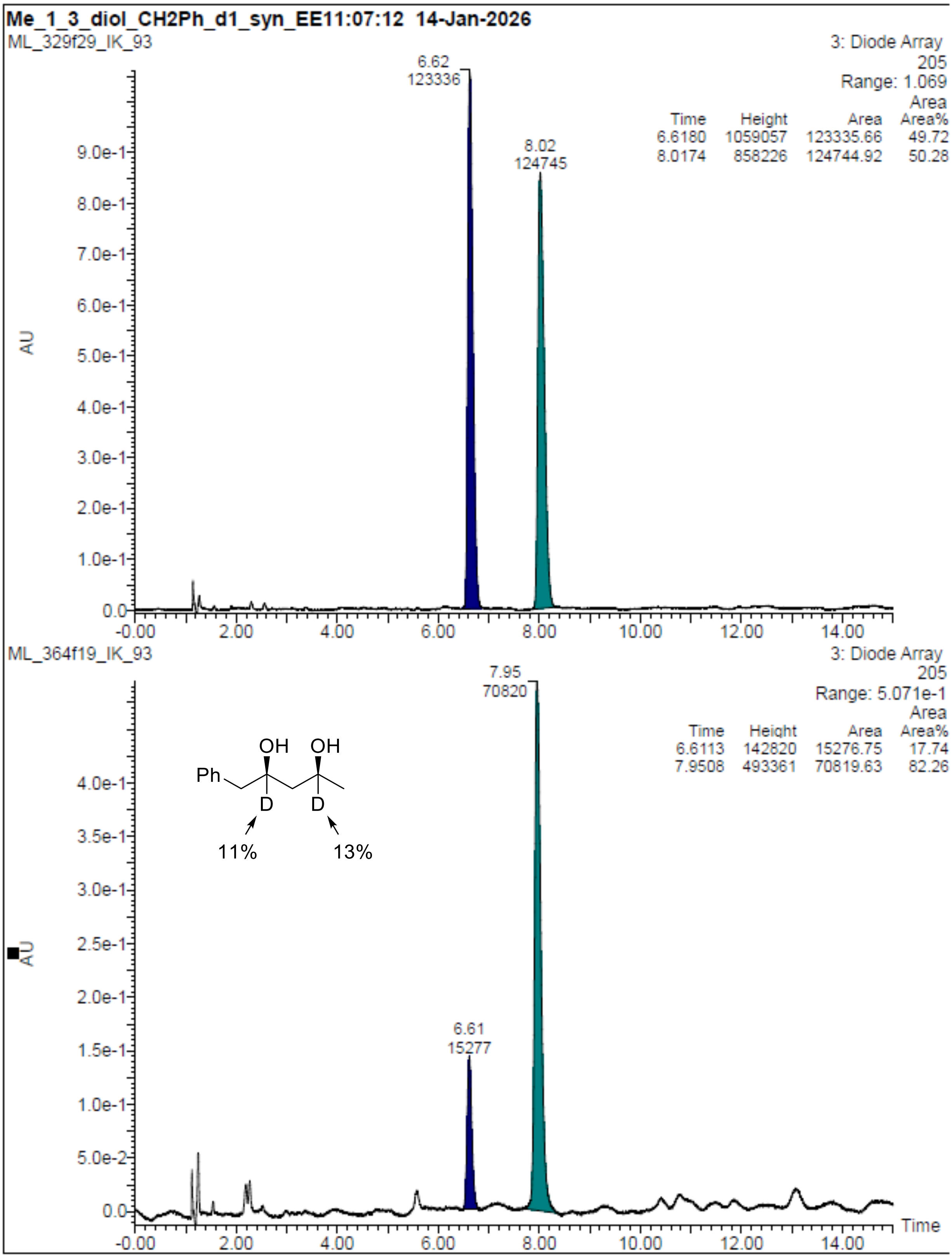

(*S*,*S*)-6-Phenylhexane-2,4-diol (*S*,*S*-3d)

Chiral SFC Analysis: CHIRALPAK IK (CO_2:MeOH, 95:5, 2.5 mL min^{-1}, 40 °C, 205 nm) t_R = 10.7 (major), 11.1 (minor) min, 99% *ee*.

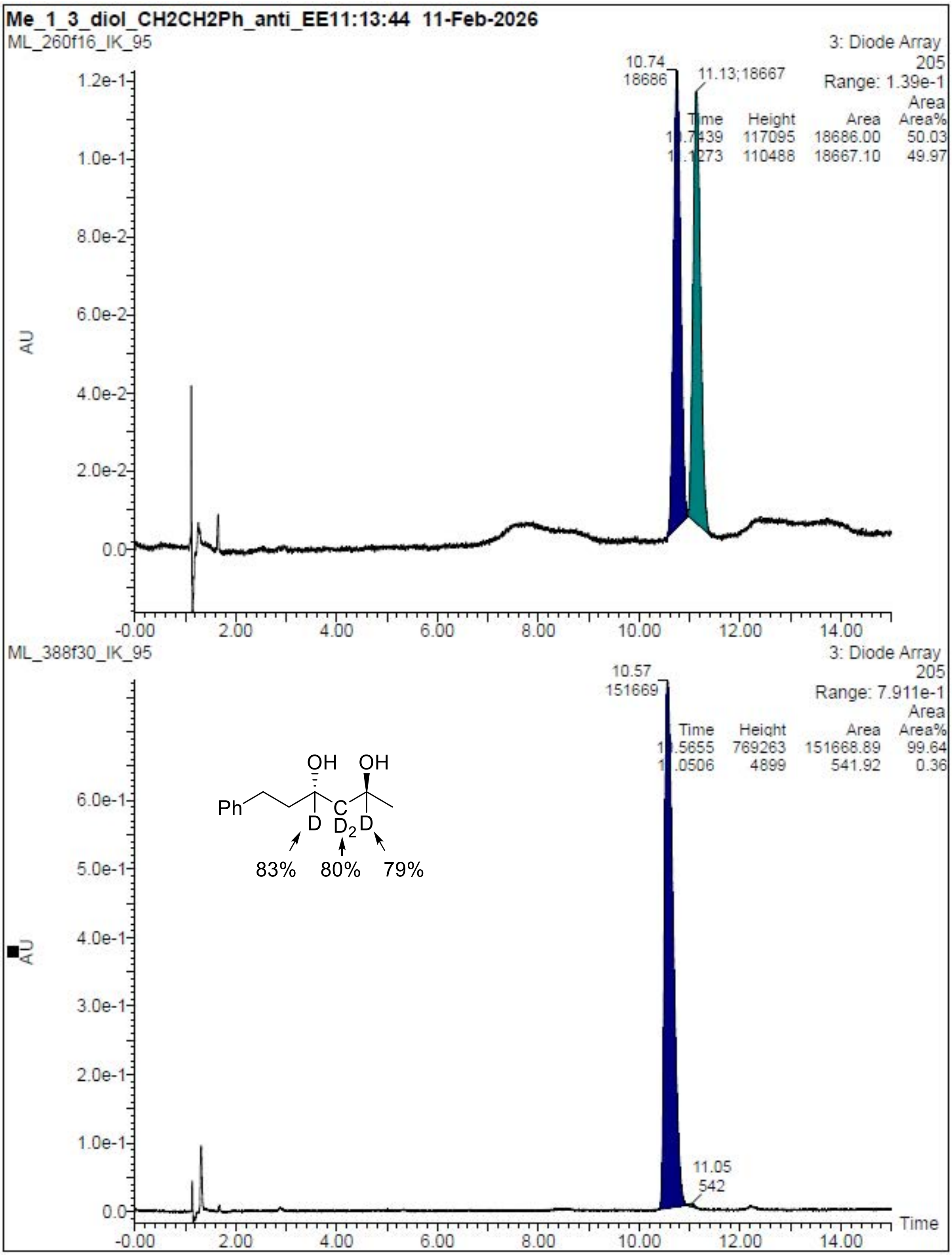

(2*S*,4*R*)-6-Phenylhexane-2,4-diol (2*S*,4*R*-3d)

Chiral SFC Analysis: CHIRALPAK IC (CO_2:MeOH, 95:5, 2.5 mL min^{-1}, 40 °C, 205 nm) t_R = 11.6 (minor), 14.0 (major) min, 47% *ee*.

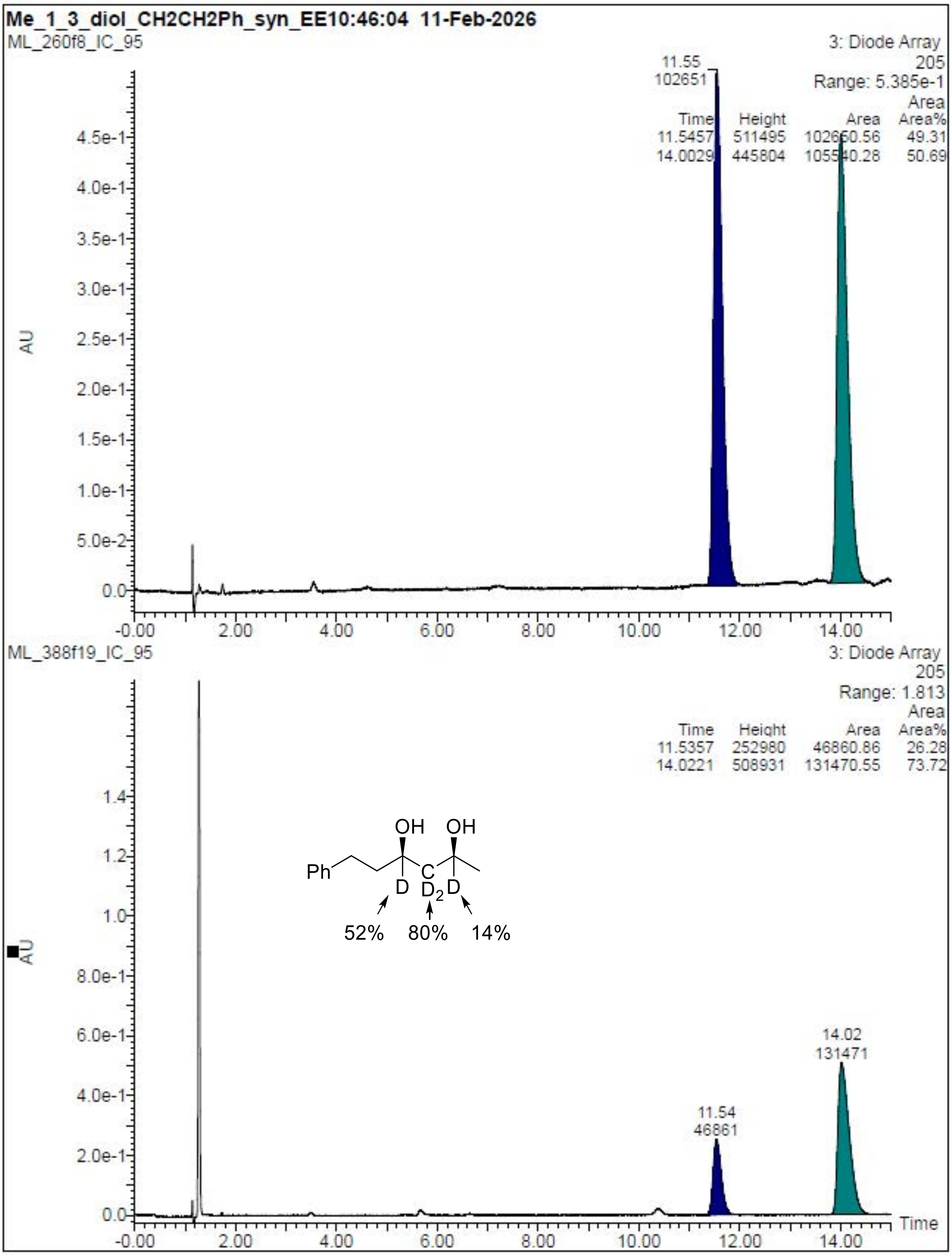

(2*S*,3*S*)-butane-2,3-diyl dibenzoate (4a-Bz)

Chiral SFC Analysis: CHIRALPAK IE (CO_2:MeOH, 98:02, 2.5 mL min^{-1}, 40 °C, 225 nm) t_R = 9.3 (minor), 10.7 (major) minutes, 97% *ee*.

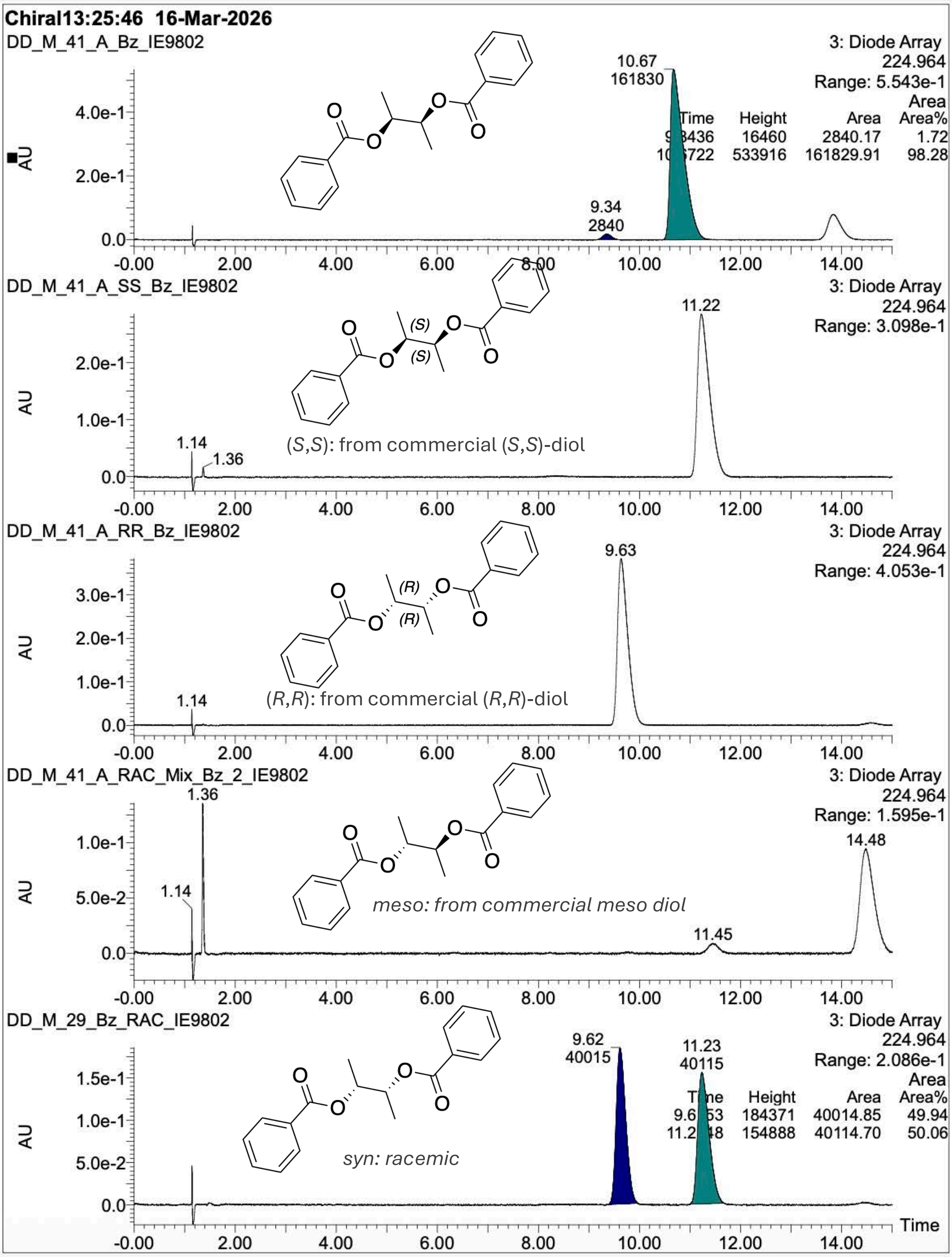

(3*S*,4*S*)-hexane-3,4-diyl dibenzoate (4b-Bz)

Chiral SFC Analysis: CHIRALPAK IK (CO_2:MeOH, 99.5:0.5, 1.25 mL min^{-1}, 40 °C, 225 nm) t_R = 22.6 (minor), 23.9 (major) minutes, 97% *ee*.

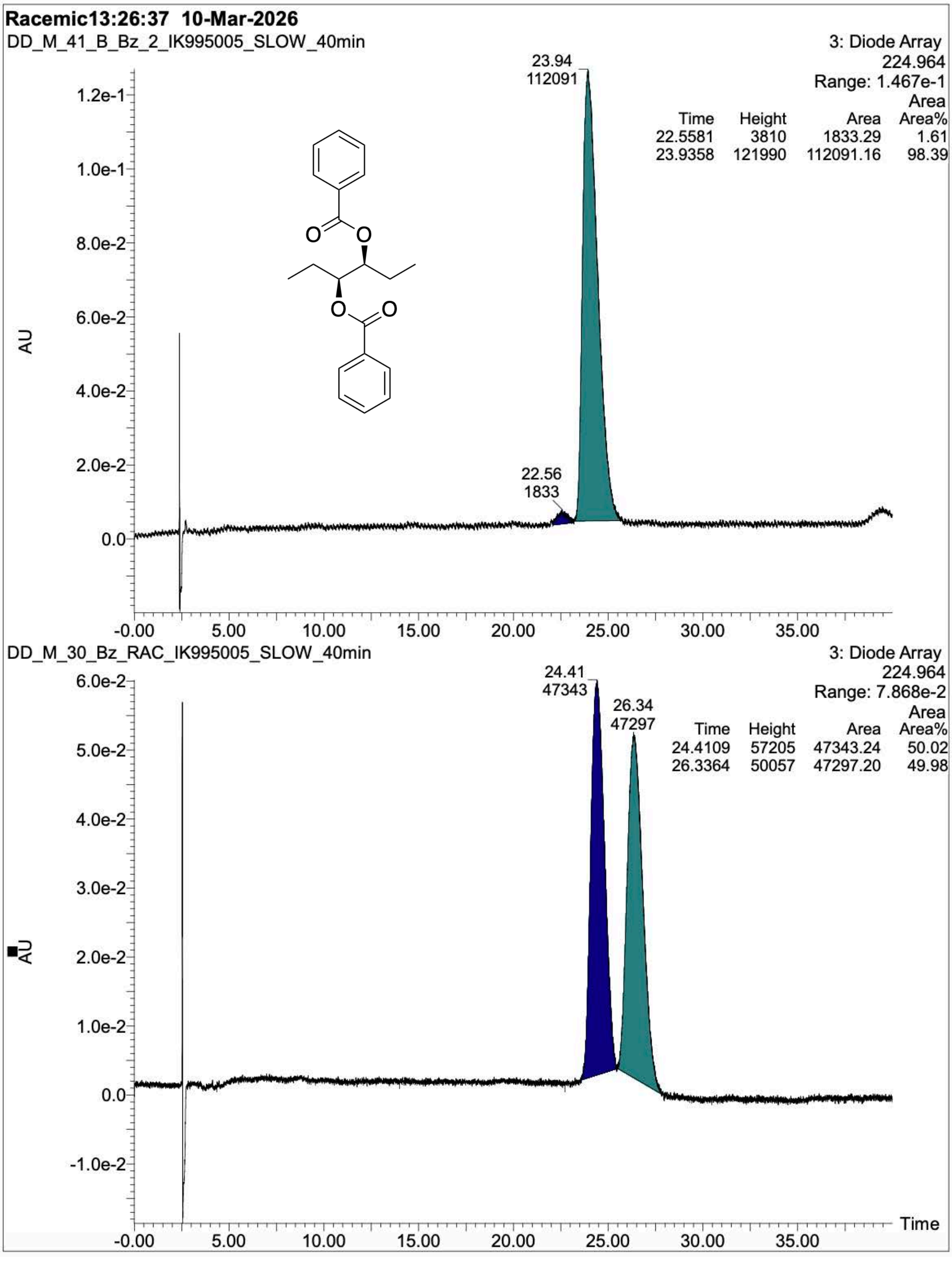

(4*S*,5*S*)-octane-4,5-diyl dibenzoate (4c-Bz)

Chiral SFC Analysis: CHIRALPAK IJ (CO_2:MeOH, 99.8:0.2, 1.25 mL min^{-1}, 40 °C, 225 nm) t_R = 7.9 (major), 9.4 (minor) minutes, 60% *ee*.

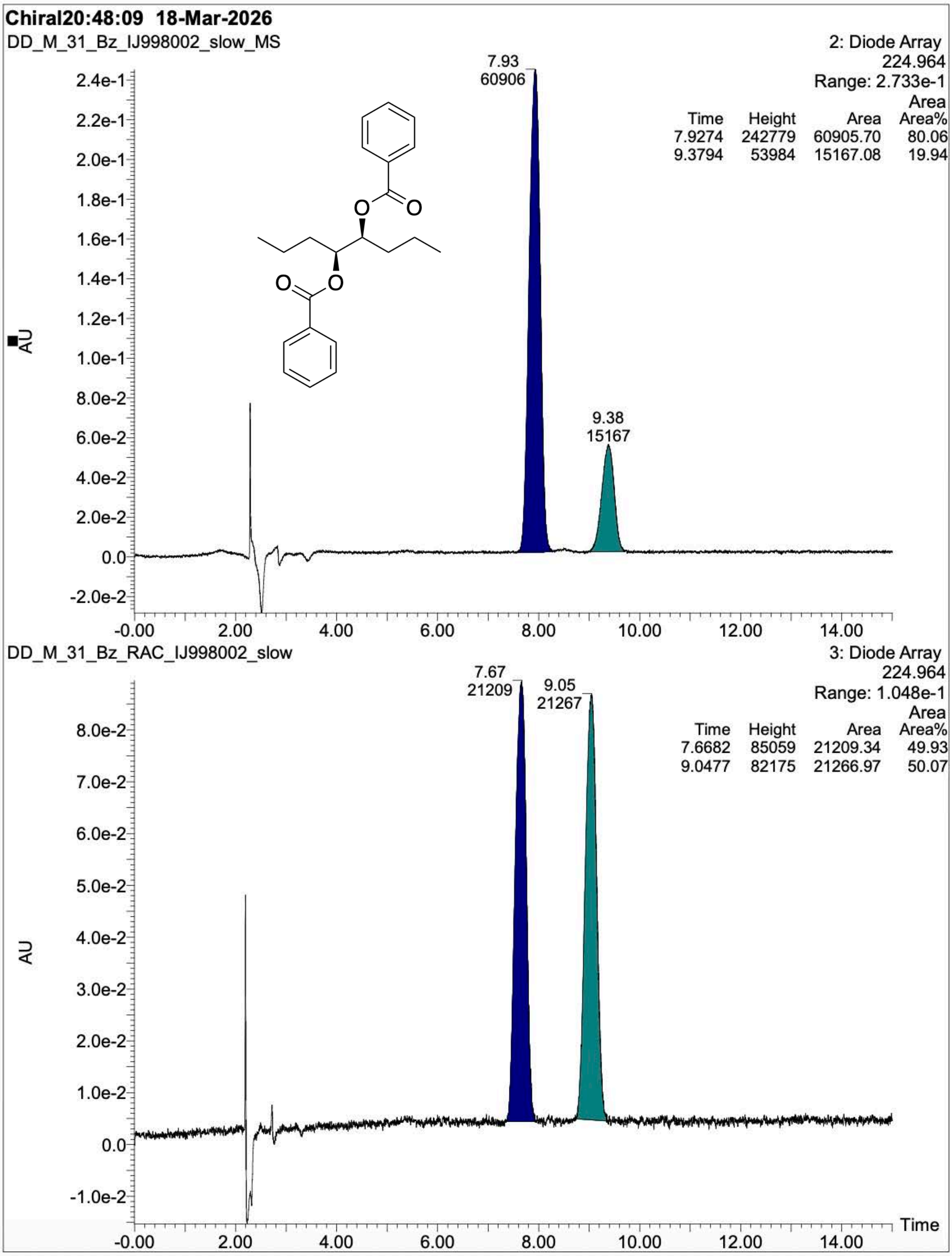

(5*S*,6*S*)-decane-5,6-diol (4d-Bz)

Chiral SFC Analysis: CHIRALPAK IG (CO_2:MeOH, 97:03, 1.25 mL min^{-1}, 40 °C, 225 nm) t_R = 9.0 (minor), 10.8 (major) minutes, 44% *ee*.

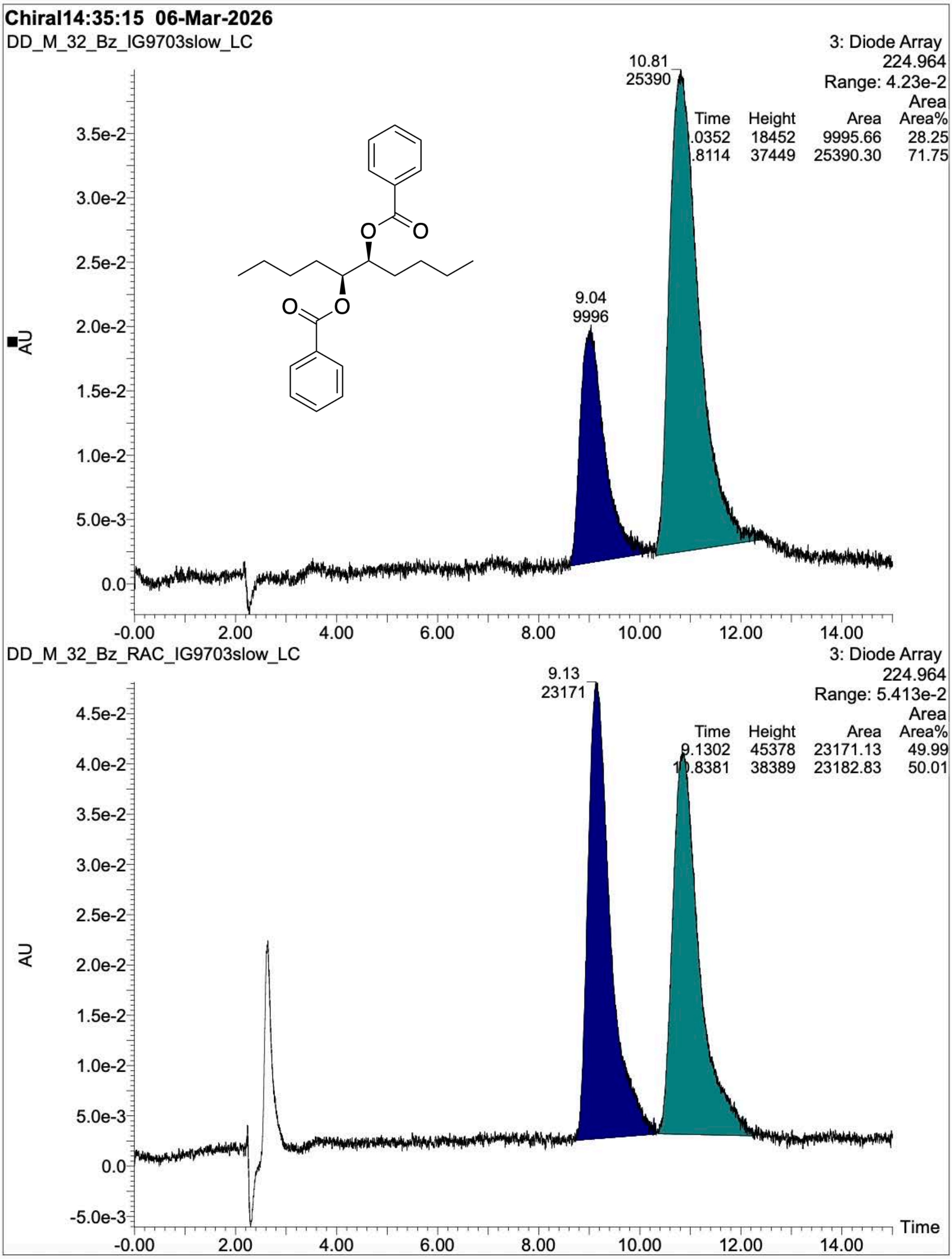

di-*tert*-butyl (4*S*,5*S*)-4,5-bis(benzoyloxy)octanedioate (4e-Bz)

Chiral SFC Analysis: CHIRALPAK IK (CO_2:MeOH, 98:02, 1.25 mL min^{-1}, 40 °C, 225 nm) t_R = 22.3 (minor), 23.9 (major) minutes, 52% *ee*.

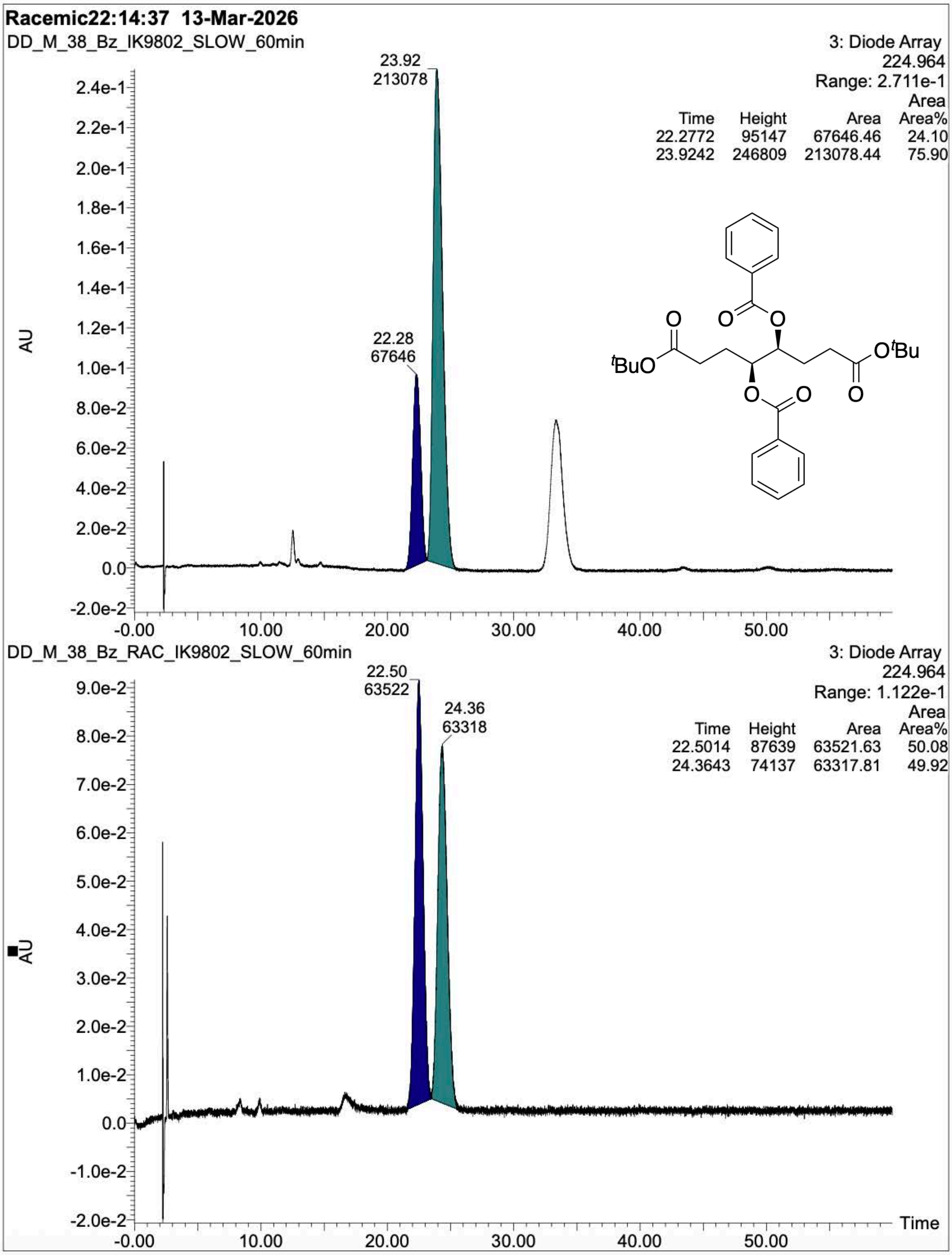

(2*S*,3*S*)-1,4-diphenylbutane-2,3-diol (4f)

Chiral HPLC Analysis: CHIRALPAK IH (Hexane:^{i}PrOH, 85:15, 1.25 mL min^{-1}, 40 °C, 209 nm) t_R = 9.7 (major), 13.8 (minor) minutes, 3% *ee*.

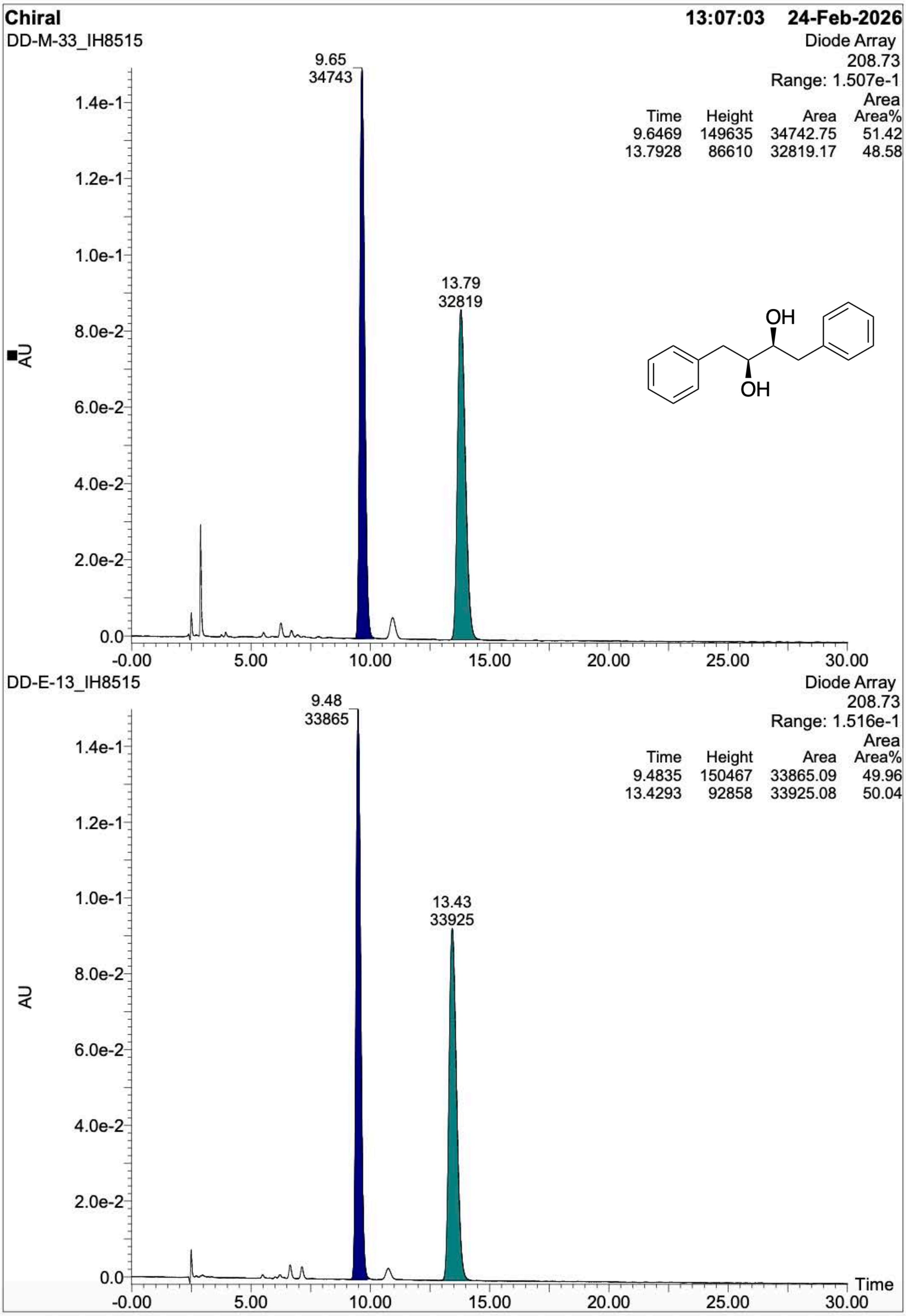

(4*S*,5*S*)-1,8-diphenyloctane-4,5-diol (4g)

Chiral SFC Analysis: CHIRALPAK IG (CO_2:MeOH, 75:25, 2.5 mL min^{-1}, 40 °C, 208 nm) t_R = 6.8 (major), 12.2 (minor) minutes, 27% *ee*.

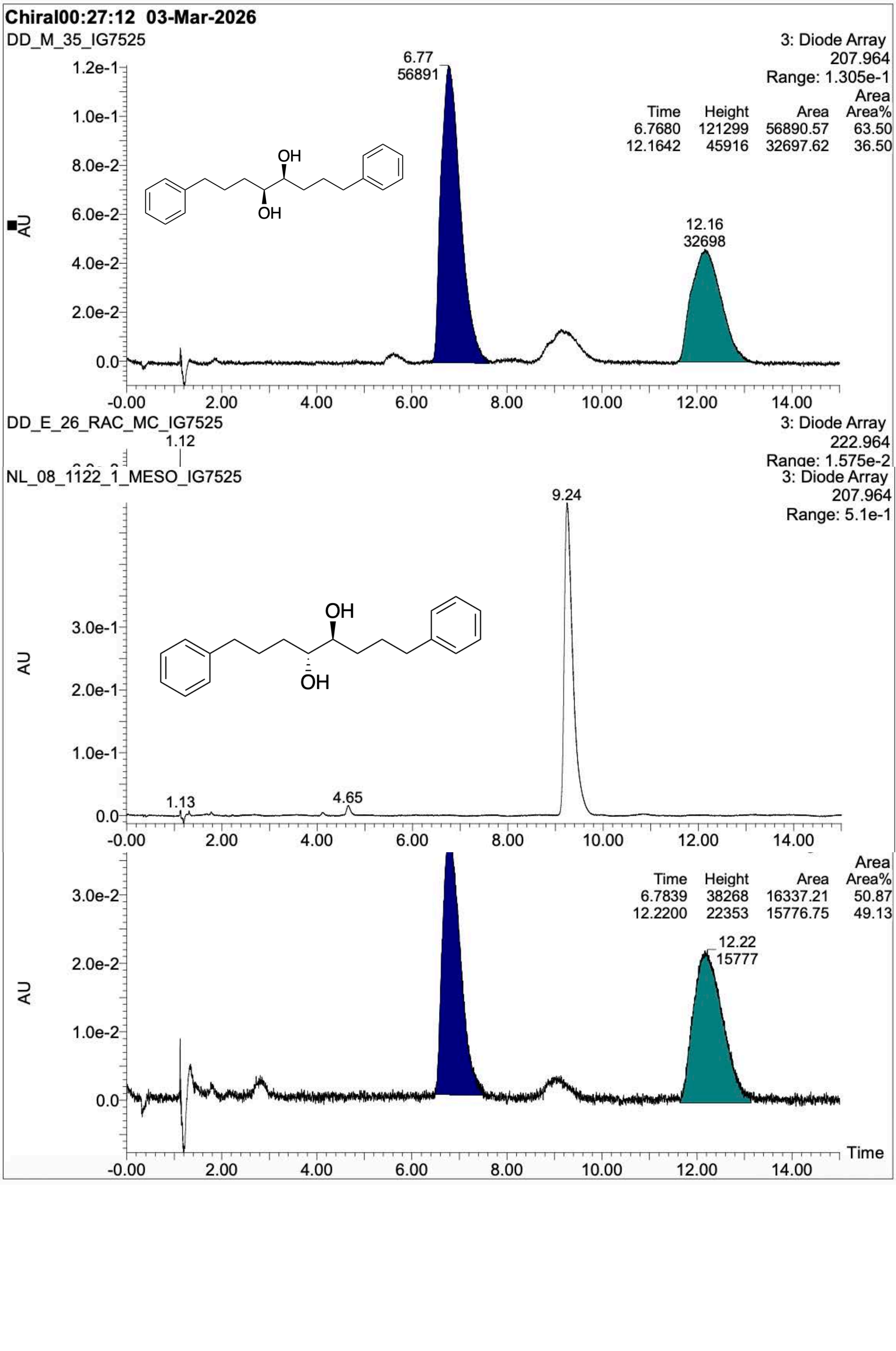

Enriched 5-Phenylpentane-2,3-diol (4h) starting from syn

Chiral SFC Analysis: *syn*: CHIRALPAK IA (CO_2:MeOH, 96:4, 2.5 mL min^{-1}, 40 °C, 204 nm) t_R = 9.3 (major), 9.72 (minor) min, 74% *ee*.

Chiral SFC Analysis: *anti* : CHIRALPAK IJ (CO_2:MeOH, 95:5, 2.5 mL min^{-1}, 40 °C, 204 nm) t_R = 5.1 (major), 5.3 (minor) min, 63% *ee*.

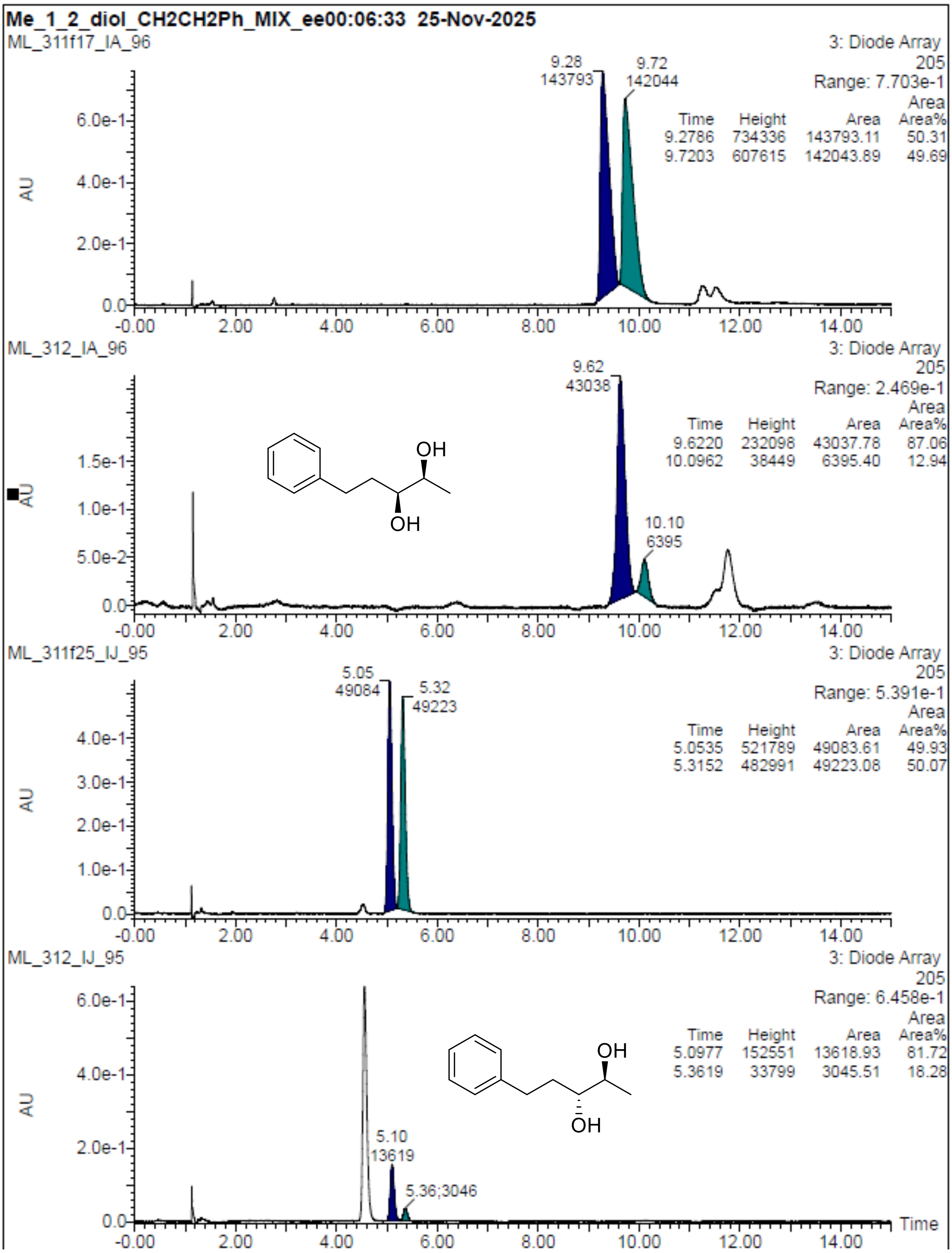

Enriched (*syn*)-5-Phenylpentane-2,3-diol (*syn*-4h)

Chiral SFC Analysis: CHIRALPAK IC (CO_2:MeOH, 96:4, 2.5 mL min^{-1}, 40 °C, 205 nm) t_R = 10.5 (minor), 11.3 (major) min, 89% *ee*.

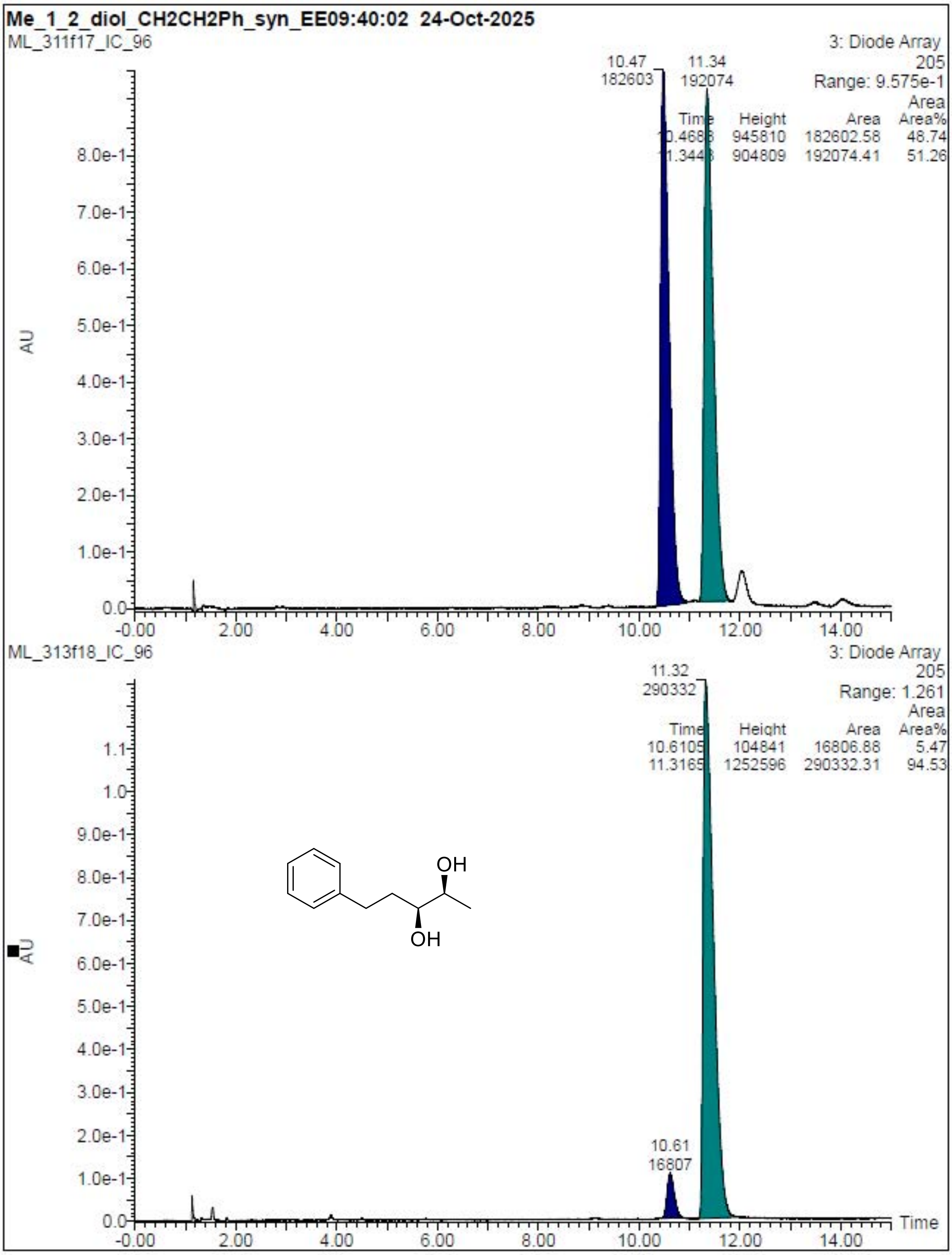

Enriched (*anti*)-5-Phenylpentane-2,3-diol (*anti*-4h)

Chiral SFC Analysis: CHIRALPAK IK (CO_2:MeOH, 93:7, 2.5 mL min^{-1}, 40 °C, 210 nm) t_R = 5.8 (minor), 6.1 (major) min, 87% *ee*.

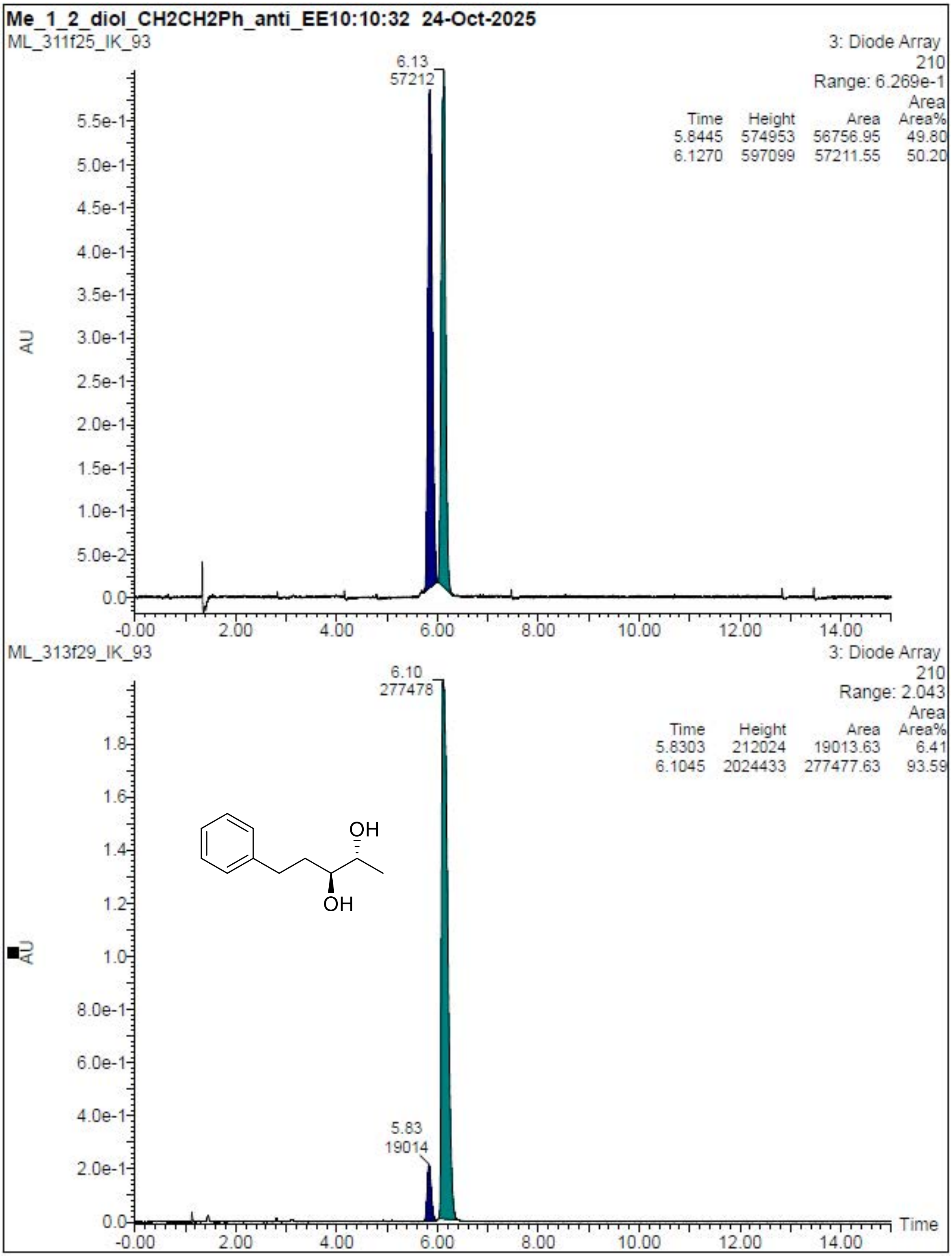

Enriched 1-Phenylbutane-2,3-diol (4i) starting from anti

Chiral SFC Analysis: *syn*: CHIRALPAK IE (CO_2:MeOH, 95:5, 2.5 mL min^{-1}, 40 °C, 225 nm) t_R = 8.9 (minor), 10.4 (major) min, 85% *ee*.

Chiral SFC Analysis: *anti* : CHIRALPAK IE (CO_2:MeOH, 95:5, 2.5 mL min^{-1}, 40 °C, 225 nm) t_R = 9.7 (major), 12.5 (minor) min, 87% *ee*.

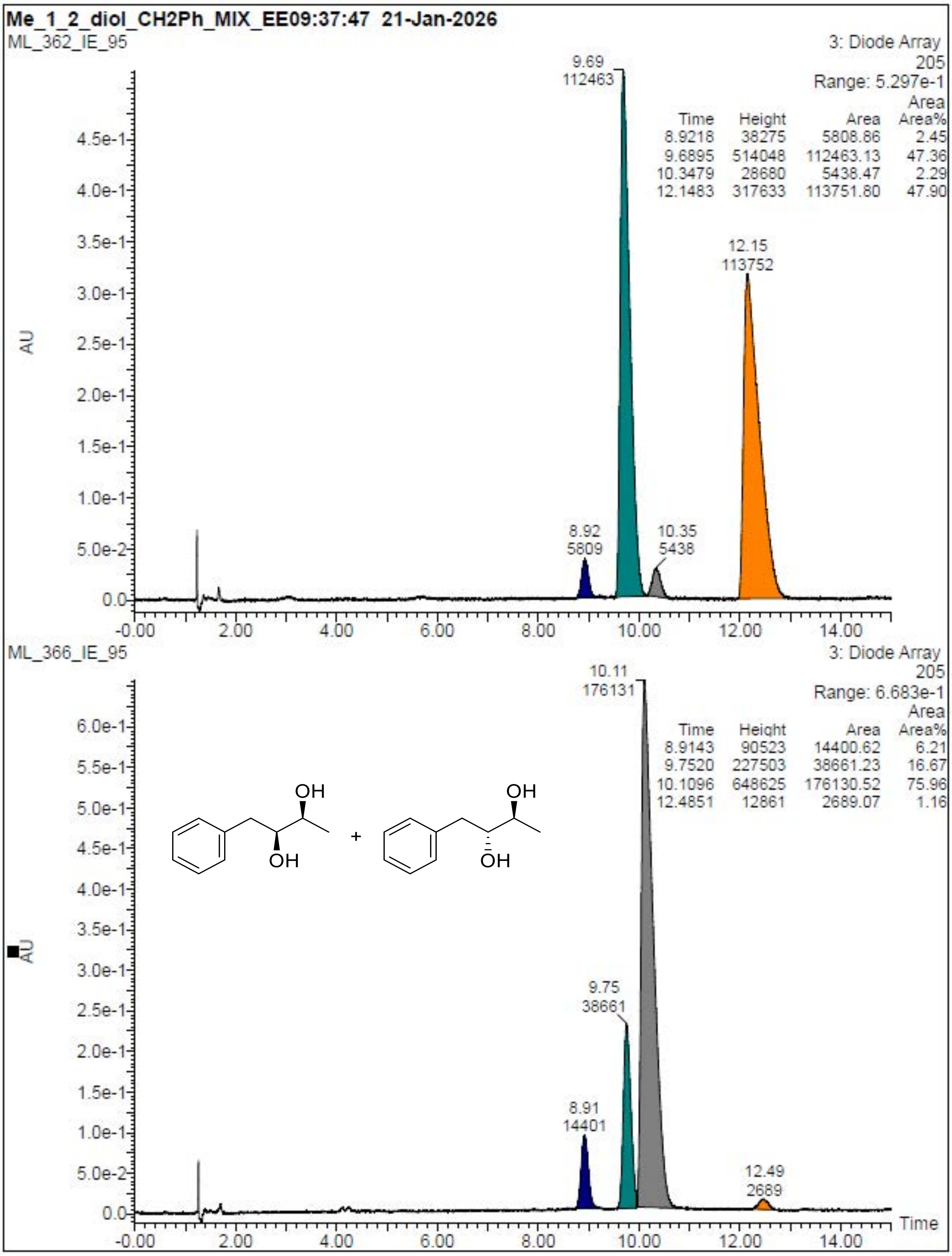

Enriched 1-Phenylheptane-3,4-diol (4j) starting from anti

Chiral SFC Analysis: *syn*: CHIRALPAK IA (CO_2:MeOH, 96:4, 2.5 mL min^{-1}, 40 °C, 204 nm) t_R = 9.5 (major), 10.0 (minor) min, 91% *ee*.

Chiral SFC Analysis: *anti* : CHIRALPAK IJ (CO_2:MeOH, 97:3, 2.5 mL min^{-1}, 40 °C, 204 nm) t_R = 8.6 (major), 9.2 (minor) min, 9% *ee*.

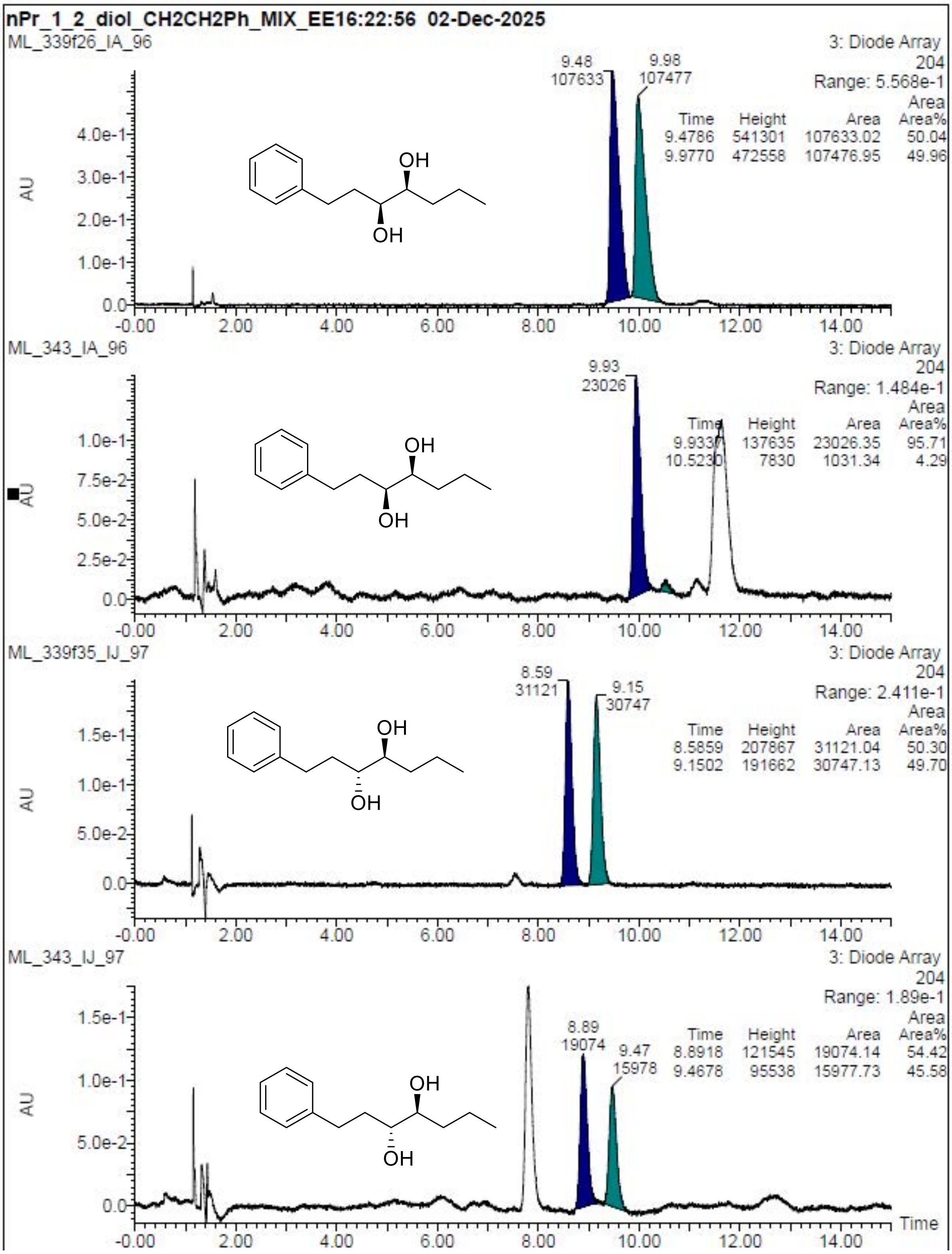

Enriched 1,1-Diphenylbutane-2,3-diol (4k) starting from anti

Chiral SFC Analysis: CHIRALPAK IJ (CO_2:MeOH, 90:10, 2.5 mL min^{-1}, 40 °C, 217 nm) t_R = 12.6 (major), 14.1 (minor) min, 66% *ee*.

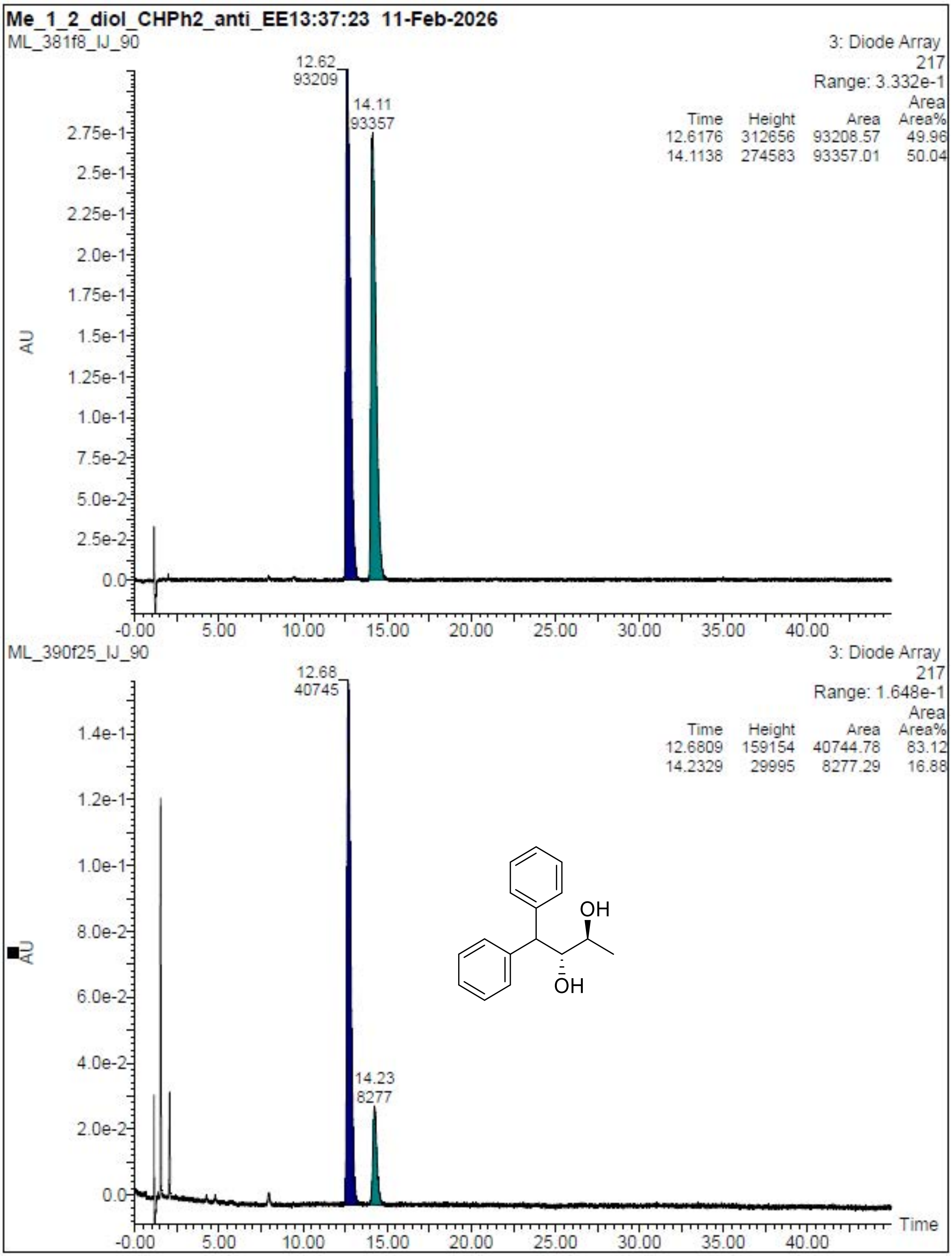

Enriched 4,5-Dihydroxyhexyl benzoate (4l) starting from syn

Chiral SFC Analysis: *syn*: CHIRALPAK IA (CO_2:MeOH, 94:6, 2.5 mL min^{-1}, 40 °C, 225 nm) t_R = 10.8 (minor), 11.3 (major) min, 23% *ee*.

Chiral SFC Analysis: *anti* : CHIRALPAK IA (CO_2:MeOH, 94:6, 2.5 mL min^{-1}, 40 °C, 225 nm) t_R = 12.5 (minor), 13.0 (major) min, 57% *ee*.

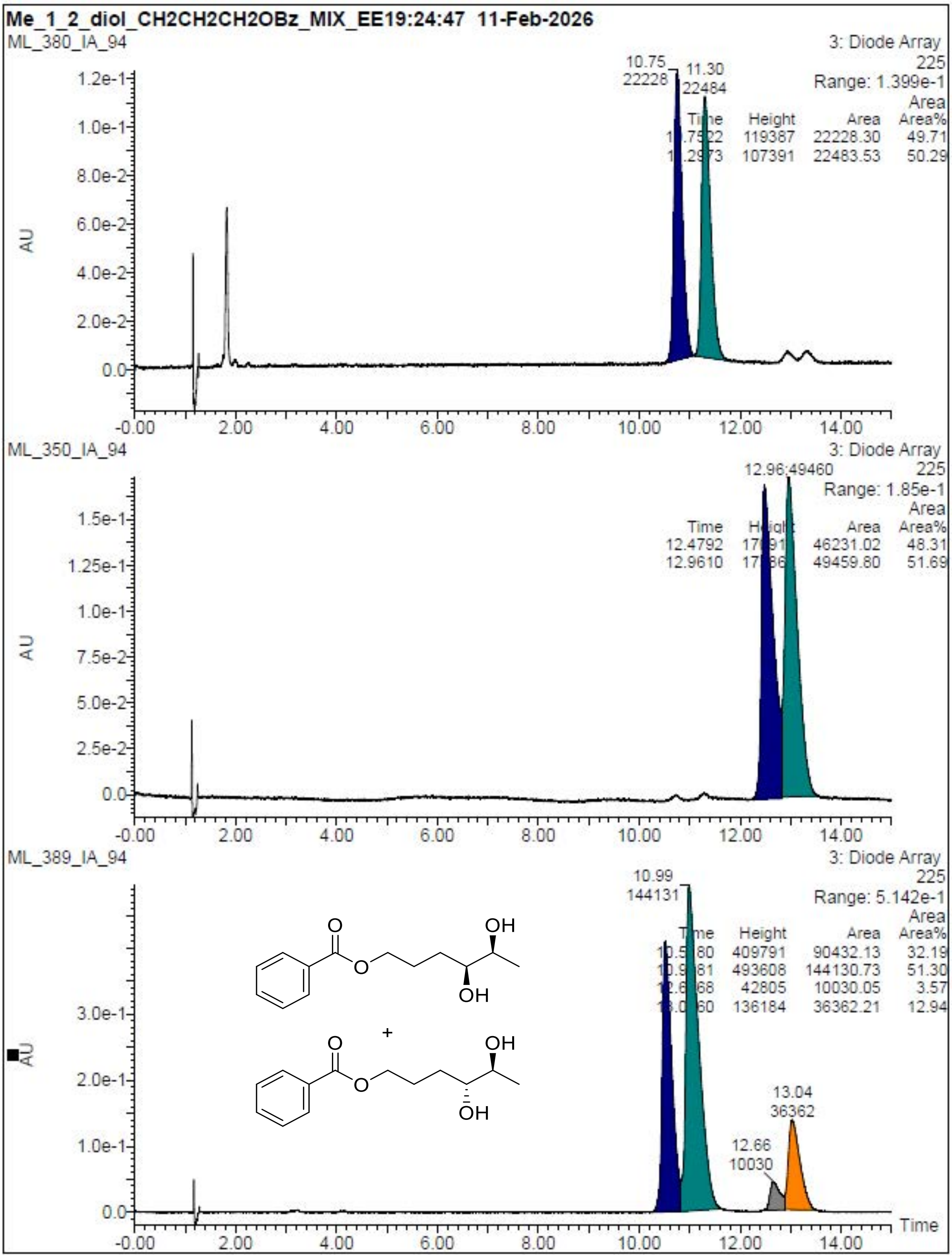

Enriched 4,5-Dihydroxyhexyl benzoate (4l) starting from anti

Chiral SFC Analysis: *syn*: CHIRALPAK IA (CO_2:MeOH, 94:6, 2.5 mL min^{-1}, 40 °C, 225 nm) t_R = 10.8 (minor), 11.3 (major) min, 92% *ee*.

Chiral SFC Analysis: *anti* : CHIRALPAK IA (CO_2:MeOH, 94:6, 2.5 mL min^{-1}, 40 °C, 225 nm) t_R = 12.5 (minor), 13.0 (major) min, 77% *ee*.

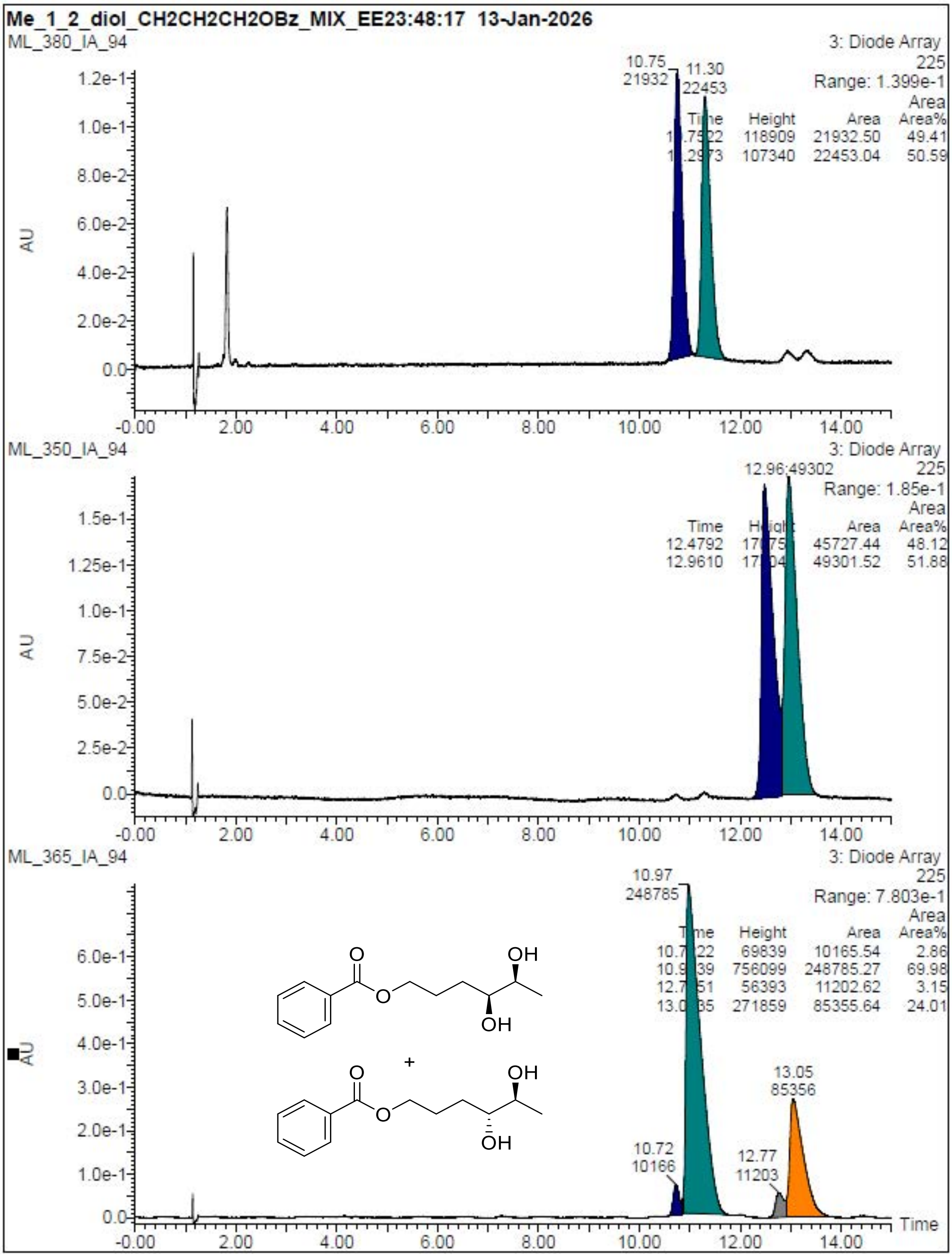

Enriched 4,5-Dihydroxyhexyl diphenylphosphinate (4m) starting from syn

Chiral HPLC Analysis: *syn*: CHIRALPAK SC (hexane:*i*PrOH, 85:15, 1.25 mL min^{-1}, 40 °C, 224 nm) t_R = 23.1 (major), 25.9 (minor) min, 82% *ee*.

Chiral HPLC Analysis: *anti* : CHIRALPAK SC (hexane:*i*PrOH, 85:15, 1.25 mL min^{-1}, 40 °C, 224 nm) t_R = 21.7 (minor), 24.6 (major) min, 72% *ee*.

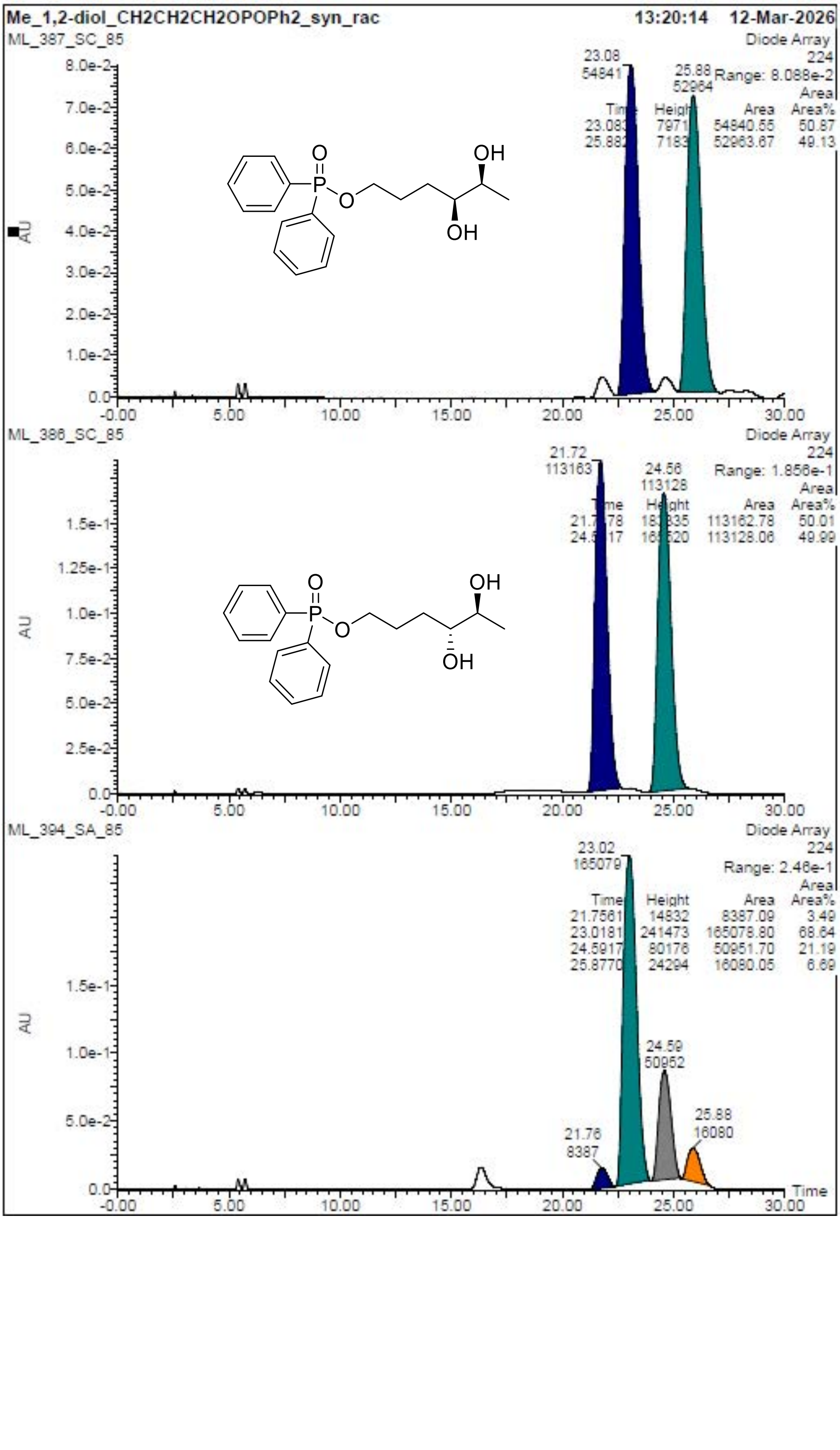

Enriched 4,5-Dihydroxyhexyl diphenylphosphinate (4m) starting from anti

Chiral HPLC Analysis: *syn*: CHIRALPAK SC (hexane:*i*PrOH, 85:15, 1.25 mL min^{-1}, 40 °C, 224 nm) t_R = 23.1 (major), 25.9 (minor) min, 93% *ee*.

Chiral HPLC Analysis: *anti* : CHIRALPAK SC (hexane:*i*PrOH, 85:15, 1.25 mL min^{-1}, 40 °C, 224 nm) t_R = 21.7 (minor), 24.6 (major) min, 77% *ee*.

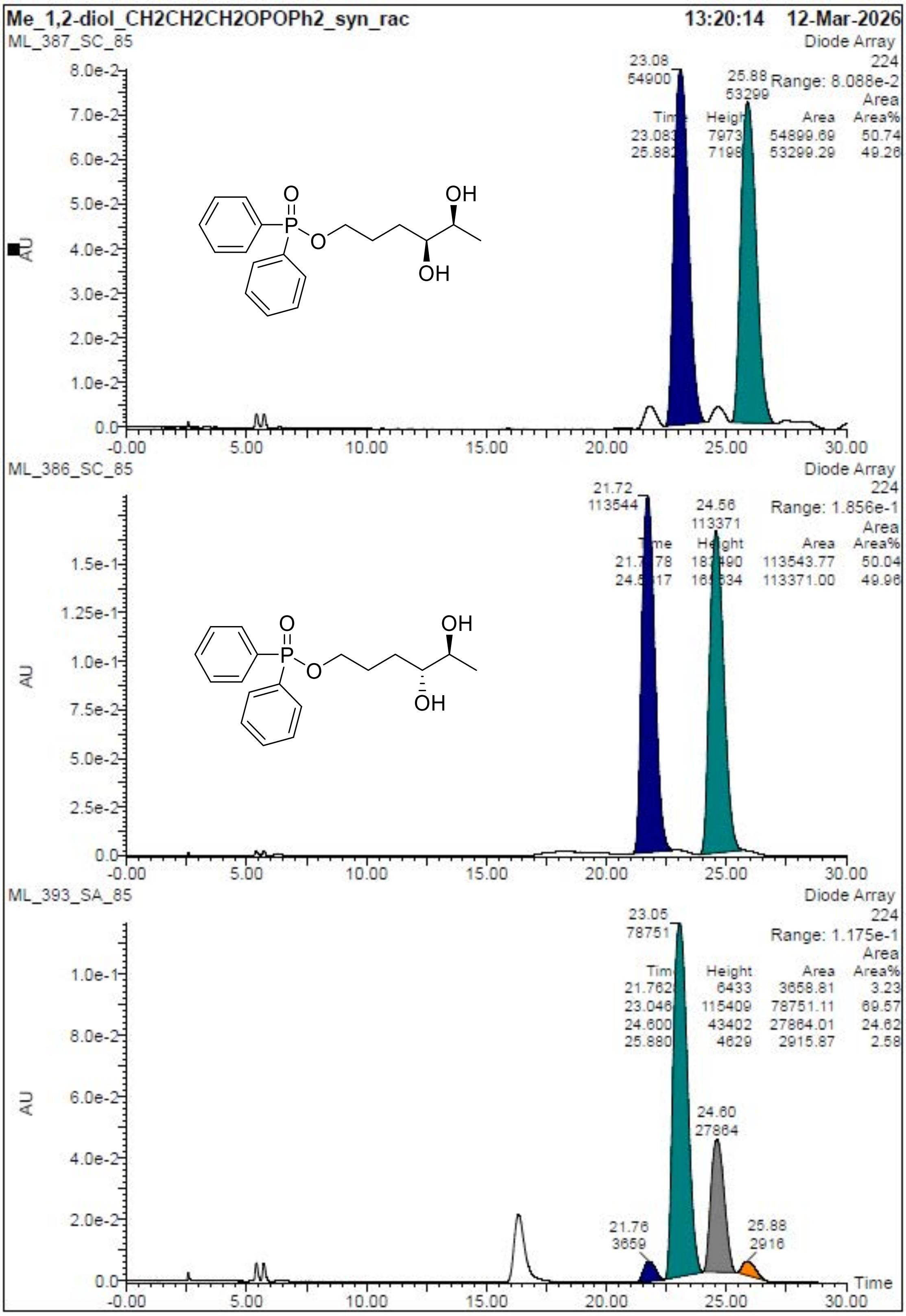

Sharpless Oxidation to Confirm Absolute Stereochemistry of 4l with Authentic Sample

(*S*,*S*)-4,5-Dihydroxyhexyl benzoate (*S*,*S*-4l)

Chiral SFC Analysis: CHIRALPAK IA (CO_2:MeOH, 94:6, 2.5 mL min^{-1}, 40 °C, 225 nm) t_R = 10.9 (minor), 11.3 (major) min, 85% *ee*.

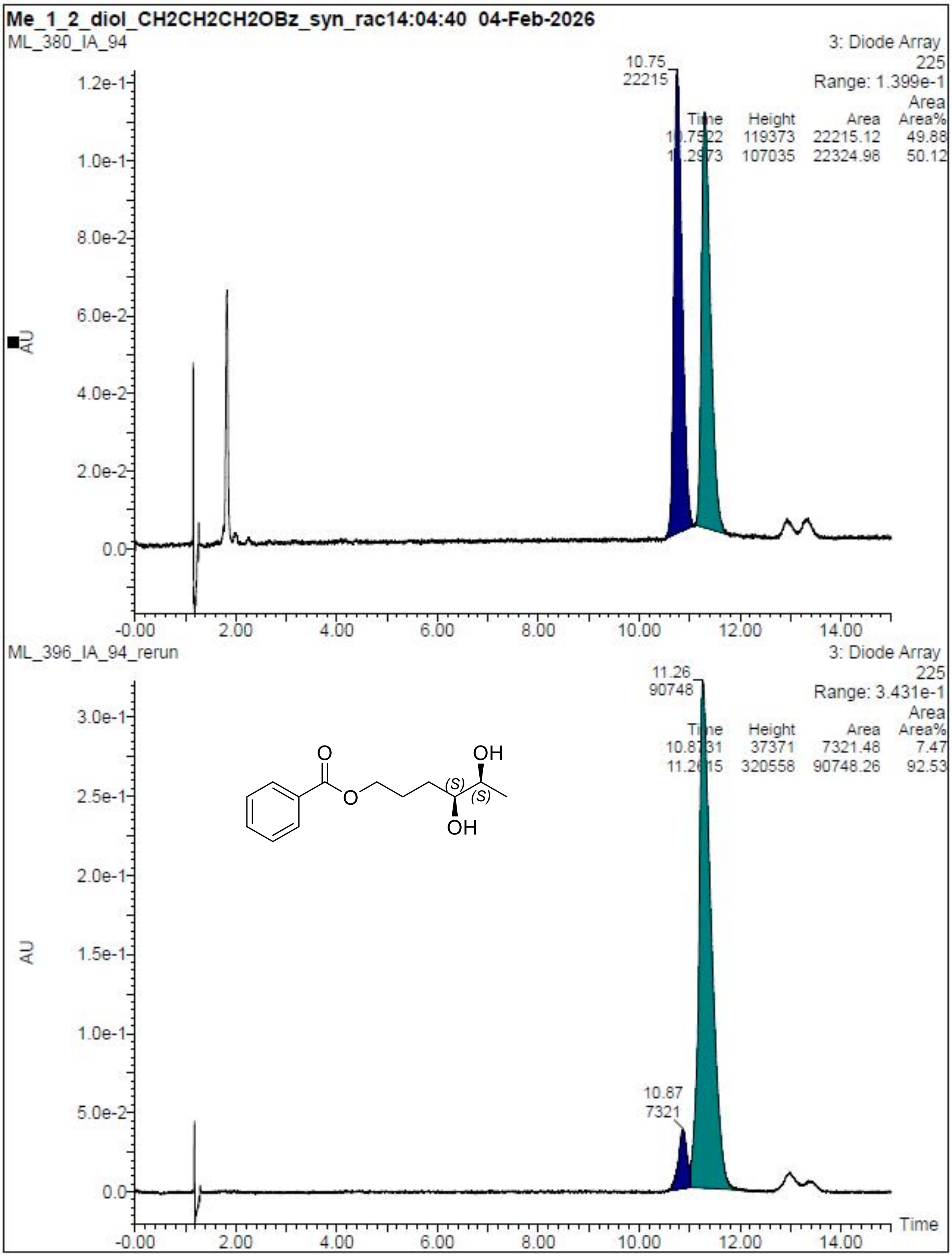

Sharpless Oxidation to Confirm Absolute Stereochemistry of 4l with Authentic Sample

(4*S*,5*R*)-4,5-Dihydroxyhexyl benzoate (4*S*,5*R*-4l) from Sharpless oxidation

Chiral SFC Analysis: CHIRALPAK IA (CO_2:MeOH, 94:6, 2.5 mL min^{-1}, 40 °C, 225 nm) t_R = 12.6 (major), 13.2 (minor) min, 23% *ee*.

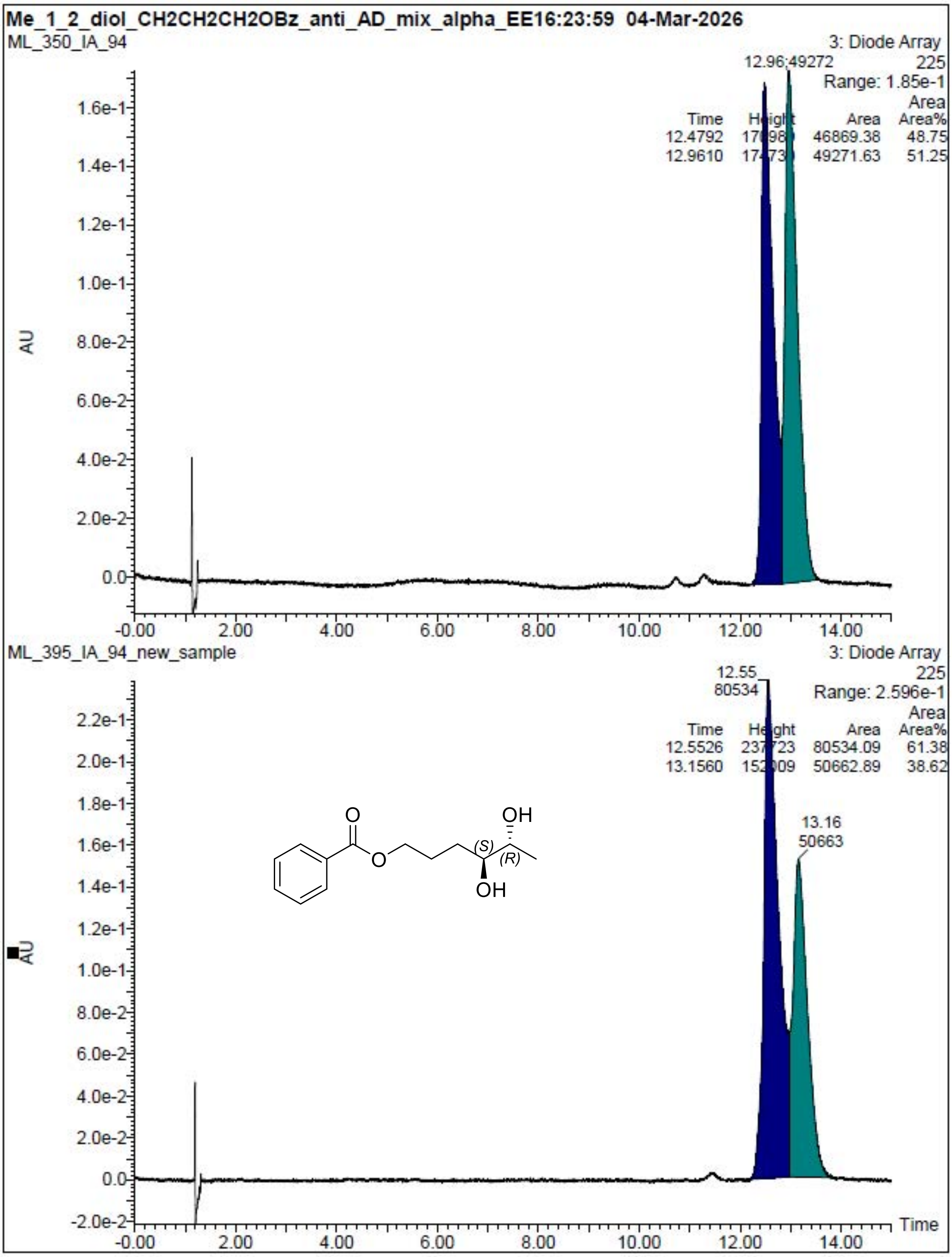

Noyori Hydrogenation to Confirm Absolute Stereochemistry of 3a with Authentic Sample

methyl (*R*)-3-hydroxy-4-phenylbutanoate (*int₂*-3a)

Chiral SFC Analysis CHIRALPAK IC (CO_2:MeOH, 95:05, 2.5 ml min^{-1}, 40 °C, 210 nm) t_R = 4.3 (minor), 4.9 (major) minutes, 90% *ee*.

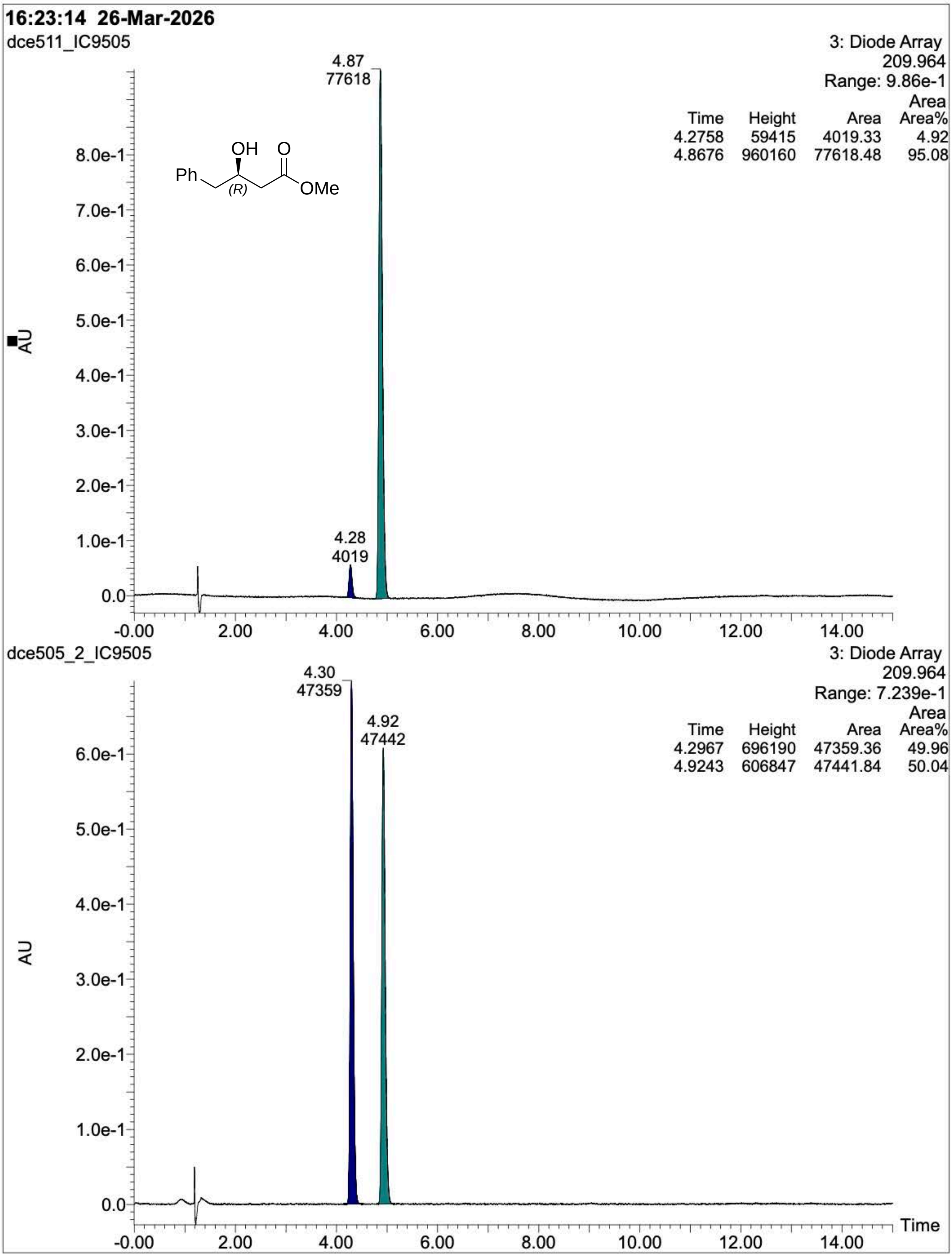

(2*R*,4*S*)-1-phenylpentane-2,4-diol ((*S*,*S*)-*syn*-3a) obtained from Noyori hydrogenation

Chiral SFC Analysis CHIRALPAK IK (CO_2:MeOH, 93:07, 2.5 ml min^{-1}, 40 °C, 207 nm) t_R = 6.6 (minor), 7.8 (major) minutes, 92% *ee*.

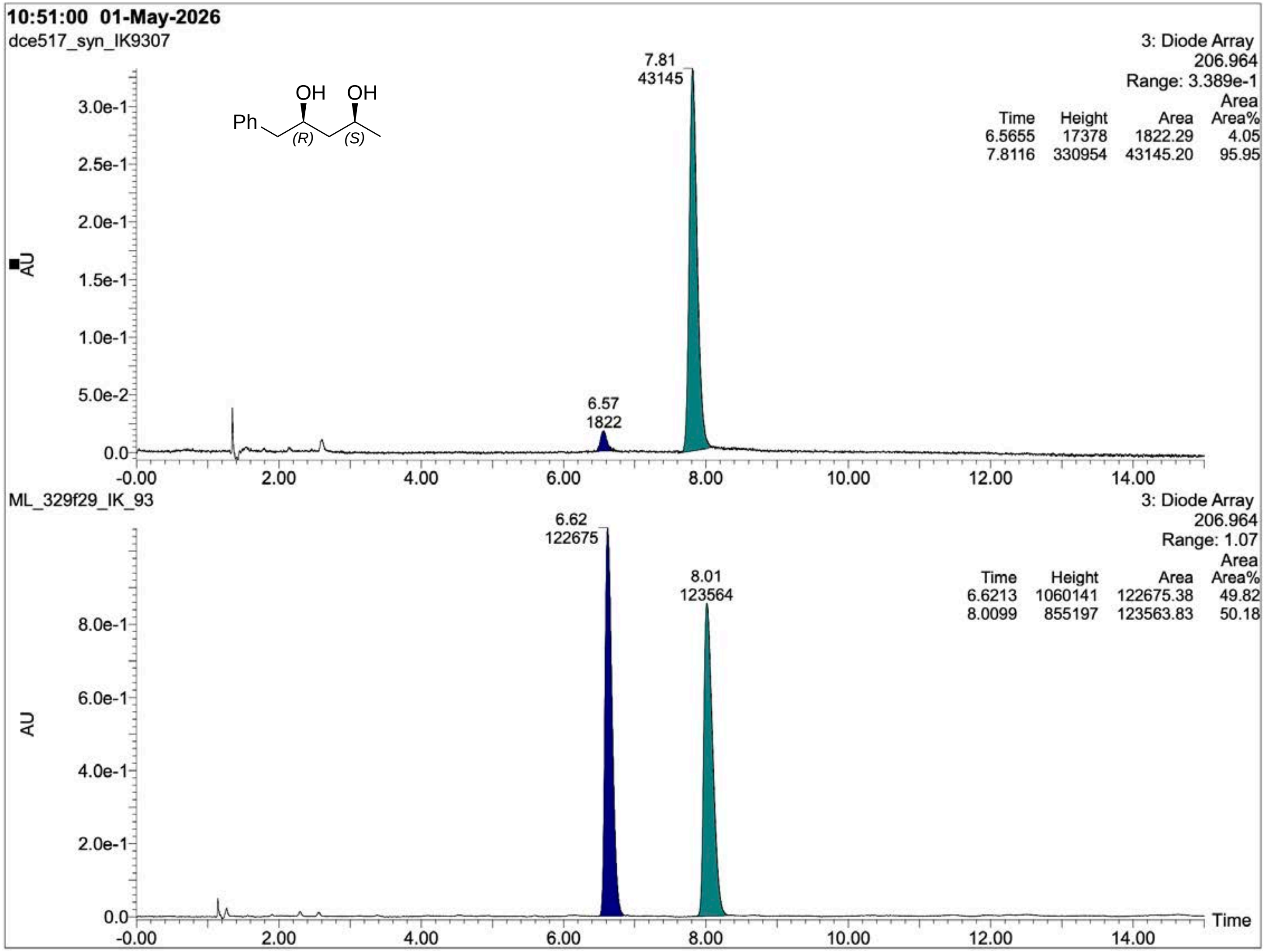

(2*R*,4*R*)-1-phenylpentane-2,4-diol ((*R*,*R*)-*anti*-3a)

(2*R*,4*R*)-1-phenylpentane-2,4-diol obtained from Noyori hydrogenation: Chiral SFC Analysis CHIRALPAK IK (CO_2:MeOH, 96:04, 2.5 ml min^{-1}, 40 °C, 205 nm) t_R = 11.0 (minor), 12.1 (major) minutes, 91% *ee*.

(2*S*,4*S*)-1-phenylpentane-2,4-diol obtained from stereoselective epimerization: Chiral SFC Analysis CHIRALPAK IK (CO_2:MeOH, 96:04, 2.5 ml min^{-1}, 40 °C, 205 nm) t_R = 11.1 (major) minutes, 99% *ee*.

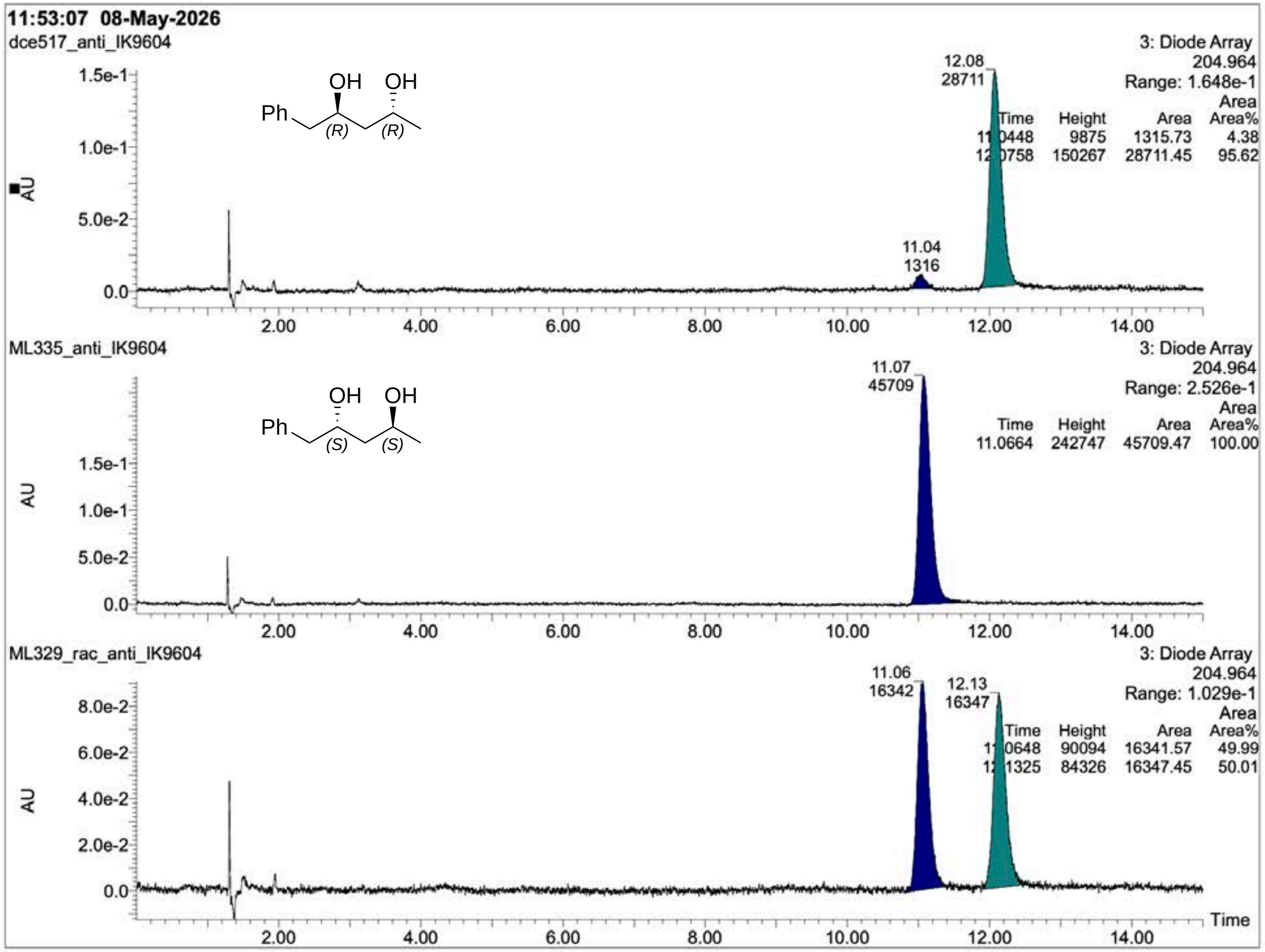

Supplement: Supplementary file 1 [file ja6c10166_si_002.pdf]
