# Supplementary material for: Stereoselective Epimerization of 1,3-Diols Using a Chiral Hydrogen Atom Abstraction Catalyst
Source: J Am Chem Soc. 2026 Jul 30;148(31):33641–51. doi: 10.1021/jacs.6c10166 (PMC13474961; doi:10.1021/jacs.6c10166)
Supplement: Supplementary file 2 [file ja6c10166_si_001.zip › derac_kinetics_simulator.html]

Symmetrical 1,3 diol epimerization - time course simulator


# Symmetrical 1,3 diol epimerization - time course simulator

Three stereoisomers with four HAA abstraction rate constants • RSH steps assumed fast and not rate‑limiting

Model

4 rate constants


8 HAA abstraction rate constants


k₁ (RR → meso)

k₇ (meso → RR)

k₆ (meso → SS)

k₄ (SS → meso)

[RR]₀ (M)

[meso]₀ (M)

[SS]₀ (M)

k₁ (RR → RS)

k₁₀ (RS → RR)

k₃ (RS → SS)

k₁₂ (SS → RS)

k₅ (SS → SR)

k₁₄ (SR → SS)

k₇ (SR → RR)

k₁₆ (RR → SR)

[RR]₀ (M)

[RS]₀ (M)

[SS]₀ (M)

[SR]₀ (M)
